# Comprehensive Analysis of Common Different Gene Expression Signatures in the Neutrophils of Sepsis

**DOI:** 10.1155/2021/6655425

**Published:** 2021-04-17

**Authors:** Zhaojun Liu, Yang Chen, Tingting Pan, Jialin Liu, Rui Tian, Shaoqiong Sun, Hongping Qu, Erzhen Chen

**Affiliations:** ^1^Department of Critical Care Medicine, Ruijin Hospital, Shanghai Jiao Tong University School of Medicine, 197 Ruijin Er Road, Shanghai 200025, China; ^2^Department of Emergency, Ruijin Hospital, Shanghai Jiao Tong University School of Medicine, 197 Ruijin Er Road, Shanghai 200025, China

## Abstract

The central component of sepsis pathogenesis is inflammatory disorder, which is related to dysfunction of the immune system. However, the specific molecular mechanism of sepsis has not yet been fully elucidated. The aim of our study was to identify genes that are significantly changed during sepsis development, for the identification of potential pathogenic factors. Differentially expressed genes (DEGs) were identified in 88 control and 214 septic patient samples. Gene ontology (GO) and pathway enrichment analyses were performed using David. A protein-protein interaction (PPI) network was established using STRING and Cytoscape. Further validation was performed using real-time polymerase chain reaction (RT-PCR). We identified 37 common DEGs. GO and pathway enrichment indicated that enzymes and transcription factors accounted for a large proportion of DEGs; immune system and inflammation signaling demonstrated the most significant changes. Furthermore, eight hub genes were identified via PPI analysis. Interestingly, four of the top five upregulated and all downregulated DEGs were involved in immune and inflammation signaling. In addition, the most intensive hub gene *AKT1* and the top DEGs in human clinical samples were validated using RT-PCR. This study explored the possible molecular mechanisms underpinning the inflammatory, immune, and PI3K/AKT pathways related to sepsis development.

## 1. Introduction

Sepsis is defined as life-threatening organ dysfunction triggered by a dysregulated host response to infection [1, 2]. Approximately 30 million people worldwide suffer from sepsis each year, which has a fatality rate of 20%-50%. Sepsis become one of the most frequent causes of mortality in intensive care units [3, 4]. Despite numerous advances in both fundamental and clinical research, the mortality rate for sepsis remains high [5]. Excessive inflammation leads to multiple organ failure, which is the main cause of mortality in early sepsis [6, 7]. Diagnosing the condition early may facilitate appropriate treatment, thereby improving patient outcomes. As such, urgent identification of novel sepsis-related biomarkers is vital for its early diagnosis and monitoring and to implement relevant therapeutic interventions.

Neutrophils are the first to arrive at sites of infection or injury, where they play a vital role in the acute phase of inflammation and the innate immune response [8]. Neutrophils can activate various signaling pathways and release inflammatory mediators that amplify the inflammatory response, eventually causing multiple system organ failure [9]. Moreover, studies have shown that neutrophils play important roles in infection control during sepsis and that their biological activity is impaired during this time, leading to dysregulated immune responses [10]. Neutrophil activation markers may be potential biomarkers for the diagnosis and prognosis of sepsis, with many studies showing that their regulation is a key part of treatment [11, 12]. Relevant mechanisms have been investigated, and clinical studies conducted, to find ways through which to block neutrophil dysfunction in patients with sepsis, which is a vital undertaking for the prevention and control of sepsis.

With recent advancements in genomics, transcriptional signature analysis has offered powerful insights into many disease [13, 14], including sepsis. Although hundreds of differentially expressed genes (DEGs) in sepsis were identified in several recently performed microarray profiling studies [15–17], few studies focus on neutrophils in sepsis. In addition, human genomic research on sepsis is limited by insufficient availability of clinical samples from a single cohort study.

In order to identify the key and promising genes or pathways associated with neutrophils in sepsis, we comprehensively reanalyzed a larger sample size (five previously collected microarrays) from the gene expression omnibus (GEO) database (https://www.ncbi.nlm.nih.gov/geo/). Common DEGs were identified and further analyzed using gene ontology (GO) annotation, pathway enrichment, and protein-protein interaction (PPI) analyses. We found that inflammation and immune response may be extremely important in the process of sepsis. These genes shed light on sepsis development, potentially providing future treatment targets.

## 2. Materials and Methods

### 2.1. Gene Expression Dataset Collection and Extraction

The National Center for Biotechnology Information (NCBI) GEO database is a public database supporting high-throughput gene expression data (http://www.ncbi.nlm.nih.gov/geo/). We searched GEO for relevant studies using the key words “sepsis,” “homo sapiens,” and “neutrophils.” A dataset was included in our study if it met the following selection criteria: (1) it comprised of gene expression profiles and (2) investigated neutrophils from human whole blood; five data profiles were included: GSE49755, GSE49756, GSE49757, GSE6535, and GSE5772 [18–20]. We excluded animal studies and samples with obvious age differences (ages < 18 and >80 years).

### 2.2. DEG Screening

DEGs were identified using GEO2R with default parameters (http://www.ncbi.nlm.nih.gov/geo/geo2r/). NCBI-generated annotations were used to display the DEG list. Only genes with a nominal *p* value < 0.05 were considered as DEGs. Common DEGs among the five GSE datasets were screened using R programming language. To identify overlapping genes between both groups, we used the VennDiagram function in R.

### 2.3. Functional and Pathway Enrichment Analysis

Common genes in any four datasets were collected to gain insight into their biological functions using GO and pathway enrichment analysis (Kyoto Encyclopedia of Genes and Genomes, KEGG). DAVID is a group of online tools that provide functional annotations for understanding biological meaning behind a large list of genes (https://david.ncifcrf.gov/) [21, 22]. We performed GO and pathway enrichment analysis using DAVID, with *p* < 0.05 considered statistically significant.

### 2.4. PPI Network Analysis

The online STRING 10.5 database [23] (https://string-db.org/) and Cytoscape [24] were used to establish a PPI network; the cut-off criterion was a combined score > 0.4 [23]. This PPI network, in which nodes and edges represented proteins and their interactions, respectively, was subsequently visualized in Cytoscape. Furthermore, the degree of a node was equal to the number of its linked edges. Additionally, CytohHubba in Cytoscape software was used to identify hub genes. Genes with edge degrees > 10 were defined as hub genes in this study.

### 2.5. Upstream Regulators of DEGs

Upstream regulatory networks from signatures of DEGs were inferred using the web application Expression2Kinases (X2K) [25] (http://X2K.cloud). We produced inferred networks of transcription factors (TF) and intermediate proteins predicted to control the expression of the inputted gene list, by combining TF enrichment analysis with PPI network expansion.

### 2.6. Sample Collection and Processing

In this study, we included patients who met the criteria for sepsis according to the definition outlined by the Surviving Sepsis campaign [2] in the intensive care unit of our hospital. Samples were acquired from peripheral vein blood of three healthy volunteers (control group) and three septic patients. Detailed clinical information was shown in Table S1. Heparinized blood was collected via venipuncture of one forearm vein under aseptic conditions, and all samples were processed within 1 h of collection. Following density centrifugation at 500 × g for 30 min, neutrophils were separated from whole blood samples using Polymorphprep (AXIS-SHIELD PoC AS, Oslo, Norway). Cell purity and viability were determined by Giemsa/Wright staining and trypan blue exclusion previously described in our published paper [26].

### 2.7. Ethics Statement

All patients and healthy volunteers provided informed consent before participation in the study. The study was conducted in accordance with the Declaration of Helsinki, and the protocol was approved by the Ruijin Hospital Ethics Committee, Shanghai Jiao Tong University School of Medicine, China (Reference Number: 2018179 released on 20 November 2018).

### 2.8. RNA Isolation and Real-Time Polymerase Chain Reaction (RT-PCR)

Total RNA was extracted from neutrophils using TRIzol reagent (Invitrogen, Grand Island, NY, USA), according to the manufacturer's instructions. For cDNA synthesis, RNA (1 *μ*g) was reverse transcribed using reverse transcriptase with random hexamers as primers (PrimeScript RT-PCR Kit; Takara, Kyoto, Japan). RT-PCR was performed using a SYBR Green PCR Master Mix (Takara) and the 7500 Fast RT-PCR System (Applied Biosystems, Foster City, CA, USA). Glyceraldehyde-3-phosphate dehydrogenase (GAPDH) was used as an internal control. All data were analyzed using the 2^−ΔΔCT^ (CT, cycle threshold) method, and expressed as fold changes relative to reference control samples. All primer sequences used in this study are listed in Table S2.

### 2.9. Statistical Analysis

RT-PCR data were displayed as the mean ± standard error of mean (SEM) and analyzed using an unpaired Student's *t*-test. All *p* values were two-sided, with a *p* < 0.05 considered statistically significant. All experiments were repeated at least three times. All statistical analyses and figures were prepared using GraphPad Prism version 6.0 (GraphPad Software, San Diego, CA, USA).

## 3. Results

### 3.1. Acquisition of Gene Expression Datasets

**Figure 1:**
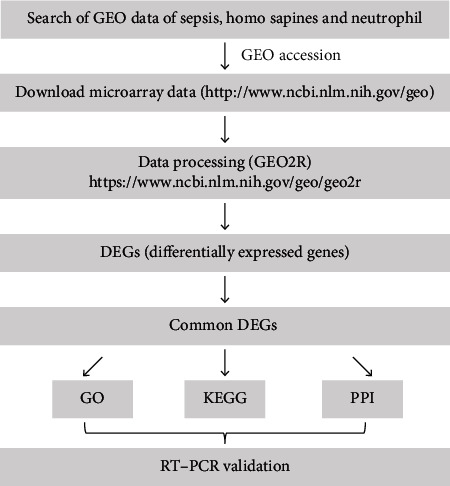
Overall study design.

The workflow of this study is illustrated in Figure 1. After using the GEO database to search for neutrophils in patients with sepsis, five datasets were selected. Altogether, microarrays of neutrophils from 88 and 214 control and septic samples, respectively, were collected. The basic information of these datasets, such as GEO accession number, sample source, number of cases and controls, platform, and published articles, is shown in Table 1.

### 3.2. Identification of DEGs

**Figure 2:**
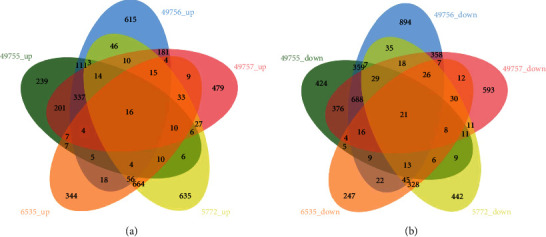
Summary of the differentially expressed genes in candidate datasets. (a) Up- and (b) downregulated genes were screened out between sepsis and controls, as shown in the Venn diagram.

Each acquired GSE dataset was analyzed using GEO2R with default parameters. There were 3437, 4755, 4285, 2036, and 2648 DEGs with a *p* < 0.05 in GSE49755, GSE49756, GSE49757, GSE6535, and GSE5772, respectively. R programming language screening identified 37 common DEGs, including 16 (43.2%) and 21 (56.8%) that were up- and downregulated, respectively (Figure 2); we provided full length DEGs tables of each dataset with gene symbol/name, probe id, logFC, *p* value, adj. *p*. val, and *t* value in Table S3–S7. The top five upregulated genes were *FKBP5*, *SORT1*, *VNN1*, *CST7*, and *GADD45A*, while the top five downregulated genes were *PRR5L*, *SH2B3*, *SULF2*, *PLEKHO1*, and *PTPN6*. These genes are listed in descending order according to the average fold change of their confirmed biological function (Table 2). Notably, the top five regulated DEGs were involved in inflammation, immune, and metabolism signaling. These results indicate that genes related to inflammation and immune signaling have a huge impact, potentially playing crucial roles in sepsis.

### 3.3. Functional and Pathway Enrichment Analysis

**Figure 3:**
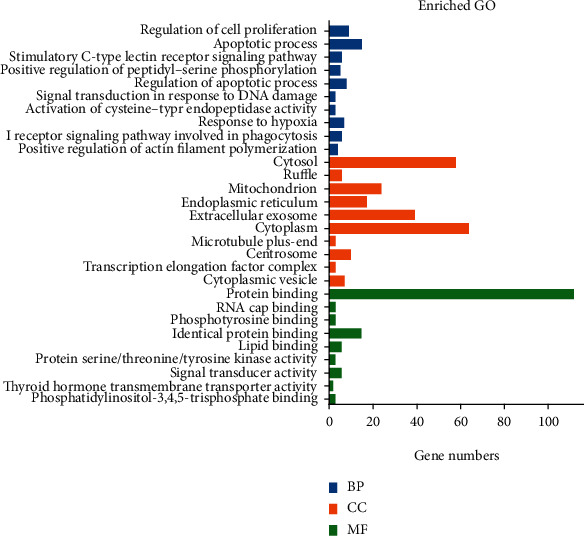
Gene Ontology (GO) analysis of differentially expressed genes (DEGs) in sepsis. The top 10 GO terms enriched by DEGs in septic and normal control neutrophil samples in 3 GO categories. MF: molecular function; CC: cellular component; BP: biological process.

**Figure 4:**
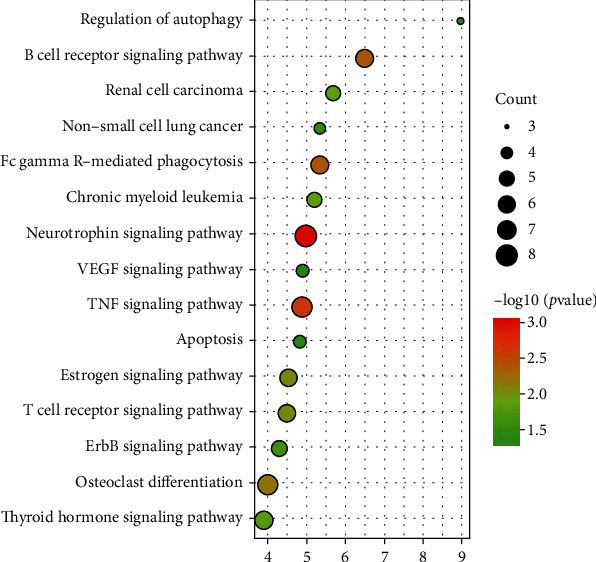
Kyoto Encyclopedia of Genes and Genomes (KEGG) pathway analysis of differentially expressed genes (DEGs) in sepsis. The top 15 KEGG pathways of DEGs in septic and normal control neutrophil samples. Colors closer to red represent higher significance.

Common DEGs identified in any four datasets were classified into the following functional categories: biological process (BP), cellular compartment (CC), and molecular function (MF), according to the GO hierarchy and a threshold significance of *p* < 0.05. DEGs were enriched in 43 GO-BP terms; the top 10 most significant terms are exhibited in Figure 3, including apoptotic process, intracellular signal transduction, response to hypoxia, and the Fc-gamma receptor signaling pathway involved in phagocytosis and the inflammatory response. Furthermore, these DEGs played essential roles in 16 GO-CC terms, primarily the cytosol, ruffle, and mitochondrion. These DEGs were also observed in 9 GO-MF terms, including protein binding, lipid binding, and signal transducer activity. We further used DAVID to analyze the total DEGs identified from any of the four datasets, with significantly enriched gene pathways then submitted to KEGG analysis; results are shown in Figure 4. As shown, these DEGs were enriched in 26 KEGG pathways, predominantly in autophagy regulation, Fc gamma R-mediated phagocytosis, the TNF signaling pathway, and apoptosis.

### 3.4. PPI Network Analysis

**Figure 5:**
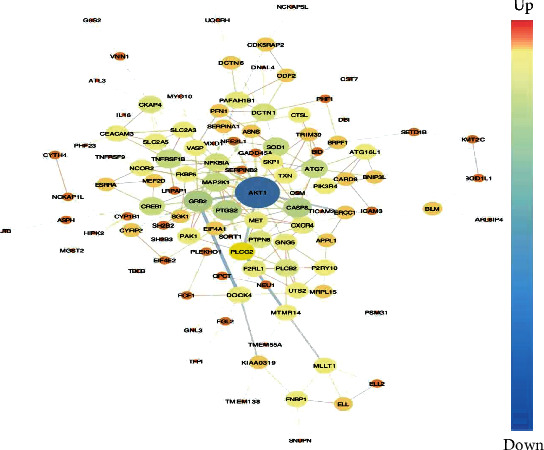
Protein-protein interaction **(**PPI) network complex. Functional network analysis of PPI networks based on neutrophil samples from sepsis patients. Total differentially expressed genes (DEGs) (up- and downregulated genes) were filtered into the PPI network. Red and blue nodes indicate up- and downregulated genes, respectively. Node size is proportional to edge degree. Edge color indicates significance according to the *p* value (the brighter the color, the smaller the *p* value).

**Figure 6:**
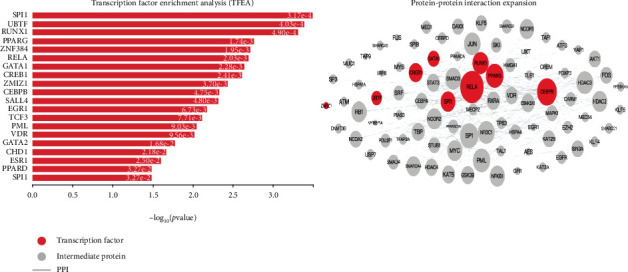
Upstream regulatory networks predicted to regulate the expression of the coregulated gene signatures in sepsis vs. the normal controls, as inferred from the Expression2Kinases (X2K) analysis. The inferred networks contain transcription factors (TFs, red nodes) and intermediate proteins (gray nodes). Gray edges indicate the interaction between two proteins (PPI). The size of nodes is relative to the level of expression degree.

Using STRING, we found 175 nodes with 215 PPI relationships (Figure 5). Among these 175 nodes, eight genes were identified as hub genes with an edge degree > 10; according to the edge degree rank, these eight genes were *AKT1*, *GRB2*, *CASP8*, *PTGS2*, *SOD1*, *ATG7*, *MAP2K1*, and *TNFRSF1B*. Excluding *ATG7*, *SOD1*, and *MAP2K1*, all genes were downregulated; *AKT1* was the most intensive hub gene, interacting with 37 genes in the network. Interestingly, some hub genes could interact with multiple other hub genes. For example, *AKT1* could interact directly with four other hub genes, namely, *ATG7*, *CASP8*, *GRB2*, and *MAP2K1*. Together, these results suggest that eight hub genes, especially *AKT1*, may play important roles in the development of sepsis. We further inferred networks of transcription factors (TF) and targeted differentially expressed genes using X2K (Figure 6). Transcription factors, such as RELA, CEBPB, CREB1, PPARG, RUNX1, SP11, and GATA1 have higher *k*-core values and are hubs. These are more centralized in the network and have a stronger capacity of modulating adjacent genes. Interestingly, we found that AKT1 interacts directly with other transcription factors, namely, CREB1 and GATA1. Together, these results suggest *AKT1* may play important roles in the development of sepsis.

### 3.5. RT-PCR Validation

**Figure 7:**
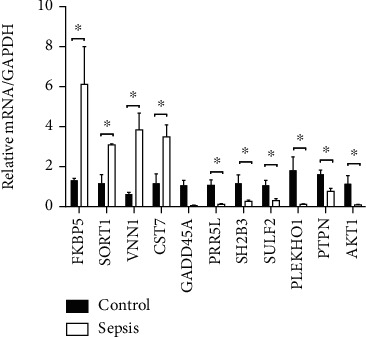
Validation of the mRNA expression level of selected genes. *FKBP5*, *SORT1*, *VNN1*, and *CST7* were significantly upregulated in septic patients, while *PRR5L*, *SH2B3*, *SULF2*, *PLEKHO1*, *PTPN6*, and *AKT1* were significantly downregulated in septic patients (*n* = 3), compared to healthy controls (*n* = 3). ^∗^*p* < 0.05.

*AKT1* and DEGs in Table 1 were randomly selected for validation. *AKT1*, top up- (*FKBP5*, *SORT1*, *VNN1*, and *CST7*) and downregulated (*PRR5L*, *SH2B3*, *SULF2*, *PLEKHO1*, and *PTPN6*) genes in sepsis were validated using RT-PCR (as shown in Figure 7).

## 4. Discussion

Sepsis is a common cause of death in intensive care units [27]. In the early stage of sepsis, activated innate immune cells, such as neutrophils, initiate a significant increase in both innate immune and inflammatory responses to clear invading pathogens from the host. If the initial response is not properly controlled, it may result in exaggerated innate immune and inflammatory responses, leading to organ damage and increased septic mortality [28, 29]. Consequently, a fundamental component of sepsis pathogenesis is inflammation, which is associated with immune system dysfunction [30]. High-throughput research may facilitate exploration of the critical mechanisms underpinning sepsis.

In this study, we performed a comprehensive bioinformatics analysis of neutrophil expression profiles collected from microarray studies of sepsis. Five gene expression profile datasets were analyzed, including 88 and 214 control and septic samples, respectively. Considering the different microarray platforms and analytical methods used in these five datasets, we reanalyzed the data using GEO2R with a uniform standard for DEGs. In total, 37 common DEGs were identified and further analyzed using functional enrichment analysis, to better understand their biological implications. Our results suggest that inflammatory response, autophagy regulation, Fc gamma R-mediated phagocytosis involved in immune responses, and the apoptotic process may be critical for the progression of sepsis. A total of 175 nodes and 215 edges were identified in the PPI network. According to PPI network analysis, *AKT1* (>30) had the highest degree of interaction; all other genes were <30. Using clinical sepsis samples and normal healthy controls, we identified the key genes associated with inflammation in sepsis, including remarkable increases in *FKBP5* and *SORT1* expression, as indicated through RT-PCR studies. In addition, we identified lower expression levels of *SH2B3*, *PLEKHO1*, and *PTPN6* (Figure 7).

The key finding of this study was that the inflammation signal and immune system play vital roles in the development of sepsis. The impact of hyperinflammation and immunosuppression on the development of sepsis has been studied for years [31, 32]. There are a number of factors that contribute to immunosuppression, including apoptosis of innate immune cells, resulting in a state of immune tolerance/paralysis in which many immune cells are reprogrammed via epigenetic alterations to an unresponsive phenotype [33–35]. In our study, apoptosis, B cell receptor signaling, Fc gamma R-mediated phagocytosis, TNF, and the VEGF signaling pathway were significantly enriched in pathway analysis (Figure 4). Many aspects of the immune and inflammatory pathways were also significantly changed in our study. First, four and five of the top five up- and downregulated genes, respectively, were involved in inflammation and immune response signaling (Table 2); these corresponded to several aspects, including NF-*κ*B signaling-driven inflammation and immunoregulation (*FKBP5*) [36–38], inflammation (*SORT1*, *SH2B3*, *PLEKHO1*, and *PTPN6*) [39–42], immune response to differentiation of monocytes to macrophages (*CST7*) [43], and apoptosis (*GADD45A*, *PRR5L*, and *SULF2*) [44–46]. Second, pathway analysis indicated that half of the top ten significantly changed sepsis pathways were related to immune and inflammatory signaling (Figure 4). With regard to immune response and inflammatory signaling in sepsis, the effects of identified DEGs and pathways may be of great interest for further study.

As the highest degree gene obtained through PPI network analysis, *AKT1* is a key regulator of the phosphoinositide 3 kinase (PI3K)/Akt signaling cascade, which controls cell growth and survival [47, 48]. Previous reports indicate that low molecular mass hyaluronan suppresses neutrophil apoptosis to trigger pulmonary inflammation via activating the PI3K/Akt pathway [49]. Apoptosis and phosphatidylinositol-3,4,5-trisphosphate (PIP3, a lipid second messenger formed by PI3K) binding and the TNF signaling pathway were significantly enriched in our pathway analysis. Moreover, *AKT1* has been reported functionally related with sepsis. Ling et al. showed that inhibition of VNN1 can alleviate lung injury through activation of the AKT signaling pathway in septic shock [50]. Polymicrobial sepsis causes cardiac dysfunction that is linked to activation of Akt signaling pathways [51]. What is more, expression of *AKT1* in septic patients was significantly decreased compared to healthy controls using RT-PCR validation (Figure 7). Therefore, the PI3K/Akt pathway in neutrophils may play a proinflammatory role in patients with sepsis.

Our study identified that *FKBP5* was the topmost upregulated gene in septic patients, compared to normal healthy controls. *FKBP5*, also as the regulatory gene of *AKT1*, encodes for a cochaperone protein that is acutely induced by stress and which can regulate immune and basic cellular processes involved in protein folding and trafficking [37, 38, 52]. Experiments in T immune cells showed that higher FKBP5 promotes inflammation by strengthening the interactions of NF-*κ*B regulatory kinases [36]. The upregulation of FKBP5 expression has been observed not only in stress exposure and glucocorticoid stimulation but also in melanoma, viral infection, depression, and some other diseases [53–55]. However, no studies have reported the expression level and role of FKBP5 in septic neutrophils. A dysregulated inflammatory response is one of the major characteristics of sepsis. Overexpression of *FKBP5*, as observed in our results, may play a role in promoting inflammation signaling in sepsis.

Although our analysis utilizes high-throughput and a large sample size, there are still limitations in the present work. First, molecular docking studies of hub genes would be needed to perform for modelling the interaction between a small molecule and a protein at the atomic level associated with sepsis. Second, more clinical samples and further relevant experimental assays, including animal models, should be conducted to confirm the underlying biological roles of key genes and pathways in our analysis of sepsis.

## 5. Conclusions

In conclusion, we offer a novel and comprehensive analysis of neutrophil gene expression profiles in clinical sepsis samples. Genes involved in inflammatory, immune, and PI3K/AKT pathways were significantly changed in sepsis. Our analysis provides valuable information for future research into the molecular mechanisms underpinning sepsis, thereby offering clues for the discovery of novel therapeutic strategies.

## Figures and Tables

**Table 1:** Gene expression datasets for sepsis in our study.

GEO accession	Cell type	Sepsis	Control	Range of ages (sepsis vs. control)	Platform	Published article
GSE 49755	Neutrophil samples	*n* = 24	*n* = 12	61.08 ± 3.37 vs. 56.67 ± 0.85	GPL10558	Damien Chaussabel et al. (2014) [16]
GSE 49756	Neutrophil samples	*n* = 29	*n* = 17	46.12 ± 1.76 vs. 65.28 ± 2.92	GPL10558	Damien Chaussabel et al. (2014) [16]
GSE 49757	Neutrophil samples	*n* = 35	*n* = 19	53.54 ± 2.46 vs. 50.63 ± 2.42	GPL10558	Damien Chaussabel et al. (2014) [16]
GSE 6535	Neutrophil samples	*n* = 55	*n* = 17	/	GPL4274	Ruby C. Y. Lin et al. (2008) [17]
GSE 5772	Neutrophil samples	*n* = 71	*n* = 23	/	GPL4274	Ruby C. Y. Lin et al. (2007) [18]

**Table 2:** The top five up- and downregulated genes in sepsis and their confirmed biological function.

Gene symbol	Log FC_A_^1^	Gene title (biological function)
*FKBP5*	1.74	FK506 binding protein 5 (inflammation and immune system)
*SORT1*	1.16	Sortilin 1 (metabolism and inflammation)
*VNN1*	0.84	Vanin 1 (metabolic pathway)
*CST7*	0.76	Cystatin F (immune system)
*GADD45A*	0.75	Growth arrest and DNA damage inducible alpha (apoptosis)
*PRR5L*	-0.95	Proline rich protein-5 like (apoptosis)
*SH2B3*	-0.84	SH2B adaptor protein 3 (inflammation and immune system)
*SULF2*	-0.75	Sulfatase 2 (metabolism and apoptosis)
*PLEKHO1*	-0.69	Pleckstrin homology domain containing 01 (inflammation)
*PTPN6*	-0.62	Protein tyrosine phosphatase nonreceptor type 6 (inflammation)

^1^FC_A_: average fold change of gene expression value.

## Data Availability

The datasets supporting the conclusions of this article are within the article and its additional files.
