# Supplementary material for: Comprehensive Analysis of Common Different Gene Expression Signatures in the Neutrophils of Sepsis
Source: Biomed Res Int. 2021 Apr 17;2021:6655425. doi: 10.1155/2021/6655425 (PMC8077712; doi:10.1155/2021/6655425)
Supplement: Supplementary 1 [file 6655425.f1.docx]

Table S1. Clinical parameters of sepsis and healthy controls

| **Sample** | **Age** | **Gender** | **Site of infection** | **ICU days** | **Ventilation days** | **APACHE II score** | **SOFA**  **score** | **Outcome** |
| --- | --- | --- | --- | --- | --- | --- | --- | --- |
| Sepsis-1 | 80 | Male | Lung | 17 | 24 | 10 | 3 | Survive |
| Sepsis-2 | 64 | Male | Abdomen | 10 | 0 | 12 | 3 | Survive |
| Sepsis-3 | 70 | Female | Lung | 6 | 0 | 14 | 4 | Survive |
| Control-1 | 27 | Female | NA | NA | NA | NA | NA | NA |
| Control-2 | 26 | Male | NA | NA | NA | NA | NA | NA |
| Control-3 | 28 | Male | NA | NA | NA | NA | NA | NA |

Abbreviations: NA, not applicable; APACHE II, Acute Physiology and Chronic Health Evaluation II. SOFA, Sequential Organ Failure Assessment.
