# Supplementary material for: Comprehensive Analysis of Common Different Gene Expression Signatures in the Neutrophils of Sepsis
Source: Biomed Res Int. 2021 Apr 17;2021:6655425. doi: 10.1155/2021/6655425 (PMC8077712; doi:10.1155/2021/6655425)
Supplement: Supplementary 2 [file 6655425.f2.docx]

Table S2. Primers list of hub gene and DEGs selected for RT-PCR.

| **Gene symbol** | **Forward primer** | **Reverse primer** |
| --- | --- | --- |
| AKT1 | TTGTGAAGGAGGGTTGGCTG | GCGCCACAGAGAAGTTGTTG |
| FKBP5 | CAAGAAGTTTGCAGAGCAGGAT | CACTGGGACTCTTCCCTCCTT |
| SORT1 | CCGGGTCCGGGACTTC | GCTATCTCCAACCCAGGACAAG |
| VNN1 | GGCACTTTCGGAACCCAGTA | CCGGATGTTGGCTTCAGACTA |
| CST7 | GTTCAGATAGTGAAAGGCCTGAA | CAGGTGCTGGTTTTTCTTGC |
| GADD45A | TGAGTGAGTGCAGAAAGCAG | TTTGCTGAGCACTTCCTCCA |
| PRR5L | AGGGCCAGGAGCTGACTATC | CAGCTTCACCTTCAGCAAGA |
| SH2B3 | CCGAAGAACTGGCCAACA | CCATTTCGTAGTCCGAGTCC |
| SULF2 | CCTTTGCCGTGTACCTCAAT | GCACGTAGGAGCCGTTGTAT |
| PLEKHO1 | ACCCGAGCCAAGAACCGTAT | TGGAAGCCACAGCCATTAGG |
| PTPN6 | AGGCCACGGTCAATGACTT | CACCTCTCGGGTGGTCAT |
