# Supplementary material for: Comprehensive Analysis of Common Different Gene Expression Signatures in the Neutrophils of Sepsis
Source: Biomed Res Int. 2021 Apr 17;2021:6655425. doi: 10.1155/2021/6655425 (PMC8077712; doi:10.1155/2021/6655425)
Supplement: Supplementary 3 [file 6655425.f3.docx]

Table S3. Detailed information of DEGs in GSE49755

| Gene symbol | probe ID | adj.P.Val | P.Value | t Value | B value | logFC |
| --- | --- | --- | --- | --- | --- | --- |
| ECHDC3 | ILMN_2072178 | 0.00981 | 2.00E-04 | 4.14 | 0.618146 | 2.88 |
| FKBP5 | ILMN_1778444 | 0.00281 | 2.94E-05 | 4.79 | 2.4103789 | 2.76 |
| IL18R1 | ILMN_1781700 | 9.43E-05 | 1.24E-07 | 6.58 | 7.533614 | 2.75 |
| KLF9 | ILMN_1778523 | 0.000407 | 1.42E-06 | 5.78 | 5.2533787 | 2.74 |
| TLR2 | ILMN_1772387 | 0.000189 | 3.47E-07 | 6.24 | 6.5735203 | 2.73 |
| IL18RAP | ILMN_1721762 | 0.000191 | 4.14E-07 | 6.18 | 6.4090618 | 2.55 |
| TPST1 | ILMN_1651950 | 0.000132 | 1.92E-07 | 6.43 | 7.1273439 | 2.17 |
| SLC16A10 | ILMN_1782938 | 0.00187 | 1.61E-05 | 4.98 | 2.9748541 | 2.14 |
| SLC2A11 | ILMN_1748090 | 0.000316 | 8.42E-07 | 5.95 | 5.7426321 | 2.05 |
| ABHD17C | ILMN_1788416 | 0.037 | 0.00159 | 3.42 | -1.2994413 | 1.95 |
| ZBTB16 | ILMN_2305407 | 0.0594 | 0.0035 | 3.13 | -2.0180254 | 1.94 |
| SLC25A51 | ILMN_2198499 | 0.0113 | 0.000249 | 4.07 | 0.4152576 | 1.9 |
| NLRP3 | ILMN_1696933 | 0.00544 | 7.66E-05 | 4.47 | 1.5145974 | 1.89 |
| IL1R2 | ILMN_1758371 | 1.80E-05 | 8.52E-09 | 7.47 | 10.0328139 | 1.83 |
| SLC26A8 | ILMN_1755843 | 0.000812 | 4.05E-06 | 5.44 | 4.2704253 | 1.83 |
| FGD4 | ILMN_1698728 | 0.00426 | 5.41E-05 | 4.58 | 1.8401 | 1.8 |
| ACSL4 | ILMN_2391458 | 0.000191 | 3.84E-07 | 6.21 | 6.4798732 | 1.75 |
| PRKDC | ILMN_1797499 | 0.000435 | 1.58E-06 | 5.74 | 5.153458 | 1.73 |
| CLEC4D | ILMN_1808979 | 0.00301 | 3.28E-05 | 4.75 | 2.3084429 | 1.72 |
| PRKDC | ILMN_2334121 | 0.00123 | 8.55E-06 | 5.19 | 3.568971 | 1.7 |
| ERLIN1 | ILMN_1730731 | 0.013 | 0.00032 | 3.98 | 0.181919 | 1.7 |
| GFOD1 | ILMN_1778240 | 0.00904 | 0.000173 | 4.19 | 0.7535541 | 1.69 |
| TBC1D14 | ILMN_1779886 | 0.000639 | 2.80E-06 | 5.56 | 4.6169142 | 1.64 |
| RAB3IL1 | ILMN_1741632 | 0.0122 | 0.000285 | 4.02 | 0.2893775 | 1.61 |
| ZNF281 | ILMN_1683127 | 0.000639 | 2.75E-06 | 5.56 | 4.632774 | 1.6 |
| NUDT16 | ILMN_1781996 | 0.0381 | 0.00168 | 3.4 | -1.3469704 | 1.6 |
| NLRP3 | ILMN_1712026 | 0.00603 | 9.23E-05 | 4.41 | 1.3413915 | 1.59 |
| CCND3 | ILMN_1668721 | 0.00247 | 2.43E-05 | 4.85 | 2.5881683 | 1.56 |
| SAMSN1 | ILMN_2171289 | 0.0151 | 0.000399 | 3.91 | -0.0204797 | 1.56 |
| BLM | ILMN_1709484 | 0.0325 | 0.0013 | 3.49 | -1.1124254 | 1.55 |
| SMA4 | ILMN_3238814 | 0.0409 | 0.0019 | 3.35 | -1.458474 | 1.55 |
| SIPA1L2 | ILMN_1732923 | 1.80E-05 | 1.02E-08 | 7.41 | 9.8661669 | 1.54 |
| NLRP3 | ILMN_2310896 | 0.011 | 0.000241 | 4.08 | 0.4461509 | 1.54 |
| IRAK3 | ILMN_1661695 | 0.00356 | 3.96E-05 | 4.69 | 2.1331189 | 1.53 |
| IRS2 | ILMN_2083469 | 8.15E-06 | 1.15E-09 | 8.15 | 11.8848083 | 1.52 |
| CD163 | ILMN_2379599 | 0.136 | 0.0148 | 2.56 | -3.3124987 | 1.52 |
| SLCO4A1 | ILMN_1727200 | 0.0119 | 0.000276 | 4.03 | 0.3209015 | 1.5 |
| GCLM | ILMN_1788547 | 0.0309 | 0.00119 | 3.52 | -1.030216 | 1.5 |
| SC5D | ILMN_1677607 | 0.000629 | 2.49E-06 | 5.6 | 4.7269551 | 1.49 |
| CCNJL | ILMN_3237926 | 0.00607 | 9.34E-05 | 4.4 | 1.3298559 | 1.46 |
| FAM65B | ILMN_1726597 | 0.000861 | 4.38E-06 | 5.41 | 4.1961157 | 1.45 |
| TMIGD3 | ILMN_1733259 | 0.057 | 0.00331 | 3.15 | -1.9659938 | 1.45 |
| PRKDC | ILMN_1769517 | 0.00495 | 6.60E-05 | 4.52 | 1.6542553 | 1.44 |
| SAP30 | ILMN_1700896 | 0.0332 | 0.00134 | 3.48 | -1.143166 | 1.42 |
| SLA | ILMN_2291954 | 0.0374 | 0.00164 | 3.41 | -1.3239919 | 1.42 |
| GNAQ | ILMN_1659923 | 0.0258 | 0.000895 | 3.62 | -0.7687575 | 1.41 |
| SORT1 | ILMN_1707077 | 0.0602 | 0.0036 | 3.12 | -2.0413719 | 1.41 |
| CEBPD | ILMN_1782050 | 2.39E-05 | 1.69E-08 | 7.24 | 9.3967482 | 1.36 |
| STK17B | ILMN_2166534 | 0.0125 | 0.000298 | 4.01 | 0.2490885 | 1.35 |
| SAMSN1 | ILMN_1684887 | 0.00986 | 0.000203 | 4.14 | 0.6084636 | 1.33 |
| TSC22D1 | ILMN_1692177 | 0.0113 | 0.00025 | 4.07 | 0.4119248 | 1.32 |
| SMAP2 | ILMN_1781468 | 0.00388 | 4.73E-05 | 4.63 | 1.9661254 | 1.31 |
| DAAM2 | ILMN_1752668 | 0.0844 | 0.00636 | 2.9 | -2.5567687 | 1.31 |
| TNFSF8 | ILMN_1761778 | 0.00864 | 0.000162 | 4.22 | 0.8165372 | 1.29 |
| CD163 | ILMN_1722622 | 0.174 | 0.023 | 2.38 | -3.6965146 | 1.29 |
| GCLM | ILMN_2225974 | 0.0135 | 0.000337 | 3.97 | 0.1358246 | 1.28 |
| CCNJL | ILMN_1763745 | 0.0205 | 0.00063 | 3.75 | -0.4437945 | 1.28 |
| TRIM25 | ILMN_1813625 | 0.144 | 0.0164 | 2.52 | -3.4008613 | 1.28 |
| SPATA13 | ILMN_1742824 | 0.00151 | 1.15E-05 | 5.09 | 3.2876449 | 1.27 |
| HLX | ILMN_2087646 | 0.119 | 0.0117 | 2.66 | -3.1034437 | 1.27 |
| MFSD13A | ILMN_1808566 | 0.153 | 0.0182 | 2.47 | -3.4924182 | 1.25 |
| LMNB1 | ILMN_2126706 | 0.00536 | 7.38E-05 | 4.48 | 1.5504665 | 1.24 |
| THBS1 | ILMN_1686116 | 0.0403 | 0.00185 | 3.36 | -1.4347943 | 1.24 |
| ZBTB16 | ILMN_2402817 | 0.155 | 0.0187 | 2.46 | -3.5147499 | 1.24 |
| PRKDC | ILMN_2253648 | 0.0121 | 0.000283 | 4.03 | 0.2967875 | 1.23 |
| PROK2 | ILMN_1775257 | 0.0304 | 0.00115 | 3.54 | -0.9972287 | 1.23 |
| NAIP | ILMN_2260082 | 0.112 | 0.0107 | 2.7 | -3.0186075 | 1.23 |
| AKAP13 | ILMN_2396956 | 0.0022 | 2.00E-05 | 4.91 | 2.7730863 | 1.22 |
| MAPK14 | ILMN_2388090 | 0.0077 | 0.000137 | 4.27 | 0.9726247 | 1.22 |
| TPK1 | ILMN_1804629 | 0.0848 | 0.00643 | 2.9 | -2.5664744 | 1.22 |
| MOB3C | ILMN_1798288 | 0.0953 | 0.00795 | 2.81 | -2.7571597 | 1.22 |
| TSC22D3 | ILMN_2276952 | 0.00113 | 7.25E-06 | 5.25 | 3.7234942 | 1.2 |
| HECW2 | ILMN_1775268 | 0.0157 | 0.000424 | 3.89 | -0.076809 | 1.2 |
| ACSL4 | ILMN_1683598 | 0.0117 | 0.000266 | 4.05 | 0.353595 | 1.19 |
| SIRT5 | ILMN_1683059 | 0.0925 | 0.0076 | 2.83 | -2.7164841 | 1.18 |
| CEACAM4 | ILMN_1657455 | 0.11 | 0.0104 | 2.71 | -2.9947267 | 1.18 |
| RGS1 | ILMN_1656011 | 0.00179 | 1.49E-05 | 5.01 | 3.0471353 | 1.17 |
| ELL2 | ILMN_1655930 | 0.00228 | 2.14E-05 | 4.89 | 2.7097877 | 1.17 |
| SPRY1 | ILMN_2329914 | 0.00037 | 1.15E-06 | 5.85 | 5.4483655 | 1.16 |
| MKNK1 | ILMN_1750429 | 0.00513 | 6.94E-05 | 4.5 | 1.6075863 | 1.16 |
| MEGF9 | ILMN_1658798 | 0.0279 | 0.00101 | 3.58 | -0.87547 | 1.16 |
| OLIG2 | ILMN_1727567 | 0.172 | 0.0226 | 2.38 | -3.6789428 | 1.16 |
| STK17B | ILMN_1798543 | 0.00037 | 1.18E-06 | 5.84 | 5.4231011 | 1.15 |
| KIAA1211L | ILMN_1776121 | 0.0398 | 0.00181 | 3.37 | -1.4175353 | 1.15 |
| RNF144B | ILMN_1752526 | 0.000158 | 2.60E-07 | 6.33 | 6.843281 | 1.14 |
| NLRP6 | ILMN_1702970 | 0.0246 | 0.000827 | 3.65 | -0.6961883 | 1.14 |
| TPK1 | ILMN_2367063 | 0.0437 | 0.00211 | 3.32 | -1.5564024 | 1.14 |
| TOB1 | ILMN_1672004 | 0.0618 | 0.00378 | 3.1 | -2.0873802 | 1.14 |
| ACSL1 | ILMN_1684585 | 0.000717 | 3.21E-06 | 5.51 | 4.4878069 | 1.13 |
| NSUN4 | ILMN_1716004 | 0.00551 | 7.86E-05 | 4.46 | 1.4907882 | 1.13 |
| NTPCR | ILMN_1657446 | 0.0342 | 0.00143 | 3.46 | -1.201808 | 1.13 |
| HIPK2 | ILMN_1687440 | 0.004 | 4.94E-05 | 4.61 | 1.9252073 | 1.12 |
| CDK5RAP2 | ILMN_2415529 | 0.0992 | 0.00846 | 2.79 | -2.8127278 | 1.12 |
| CDK5R1 | ILMN_1730928 | 0.103 | 0.00905 | 2.76 | -2.8725503 | 1.12 |
| NEDD9 | ILMN_1743619 | 0.152 | 0.018 | 2.48 | -3.4832203 | 1.12 |
| STEAP4 | ILMN_1772036 | 0.186 | 0.026 | 2.32 | -3.8034497 | 1.12 |
| MS4A6A | ILMN_1797731 | 0.191 | 0.0272 | 2.3 | -3.8403797 | 1.11 |
| ASPH | ILMN_2352934 | 0.00541 | 7.51E-05 | 4.47 | 1.5336952 | 1.1 |
| MAPK14 | ILMN_1788002 | 0.0151 | 4.00E-04 | 3.91 | -0.0228116 | 1.1 |
| CLEC4E | ILMN_1771664 | 0.069 | 0.00453 | 3.03 | -2.2510495 | 1.1 |
| MSL3 | ILMN_1713156 | 0.000316 | 8.23E-07 | 5.96 | 5.7638214 | 1.09 |
| KCNE5 | ILMN_1711650 | 0.0279 | 0.001 | 3.58 | -0.8727273 | 1.09 |
| AKAP13 | ILMN_1752247 | 0.0358 | 0.00152 | 3.43 | -1.2585999 | 1.09 |
| HIPK3 | ILMN_2337551 | 0.0398 | 0.00181 | 3.37 | -1.4162179 | 1.09 |
| ATF6 | ILMN_1703471 | 0.0411 | 0.00192 | 3.35 | -1.4704771 | 1.09 |
| TLR5 | ILMN_1722981 | 0.245 | 0.0428 | 2.1 | -4.2308066 | 1.09 |
| SYTL3 | ILMN_1720623 | 0.000943 | 5.31E-06 | 5.35 | 4.0154398 | 1.08 |
| NET1 | ILMN_1758311 | 0.00686 | 0.000115 | 4.33 | 1.1384864 | 1.08 |
| TSC22D3 | ILMN_1695382 | 0.0225 | 0.000714 | 3.7 | -0.5605499 | 1.08 |
| IL1R1 | ILMN_1810584 | 0.0297 | 0.00111 | 3.55 | -0.9650486 | 1.08 |
| TSC22D1 | ILMN_1787567 | 0.0338 | 0.0014 | 3.47 | -1.1792754 | 1.08 |
| KIF27 | ILMN_2366795 | 0.0809 | 0.00593 | 2.93 | -2.4937578 | 1.08 |
| RGS18 | ILMN_2101278 | 0.000187 | 3.35E-07 | 6.25 | 6.6055815 | 1.07 |
| SMIM3 | ILMN_1684368 | 0.0186 | 0.000547 | 3.8 | -0.3130221 | 1.07 |
| ABCG1 | ILMN_2329927 | 0.0227 | 0.000727 | 3.7 | -0.5761719 | 1.07 |
| NUDT5 | ILMN_1711314 | 0.0809 | 0.00593 | 2.93 | -2.4938124 | 1.07 |
| ARID5B | ILMN_1721626 | 0.107 | 0.00965 | 2.74 | -2.9301257 | 1.07 |
| PFKFB2 | ILMN_1723436 | 0.14 | 0.0157 | 2.54 | -3.3623923 | 1.07 |
| PXK | ILMN_1815063 | 0.000316 | 8.77E-07 | 5.94 | 5.7050219 | 1.06 |
| KCNH2 | ILMN_1739987 | 0.00222 | 2.05E-05 | 4.91 | 2.7502476 | 1.06 |
| PPP1R3B | ILMN_1712236 | 0.00576 | 8.52E-05 | 4.43 | 1.4162834 | 1.06 |
| TCAF2 | ILMN_1780798 | 0.0388 | 0.00173 | 3.39 | -1.3746861 | 1.06 |
| CYTIP | ILMN_3235928 | 0.000191 | 4.10E-07 | 6.18 | 6.4168414 | 1.05 |
| CPD | ILMN_1703074 | 0.00301 | 3.29E-05 | 4.75 | 2.3062314 | 1.05 |
| PAPSS2 | ILMN_2410929 | 0.00742 | 0.00013 | 4.29 | 1.0236039 | 1.05 |
| ETS2 | ILMN_1720158 | 0.0336 | 0.00138 | 3.47 | -1.1701411 | 1.05 |
| IRAK3 | ILMN_1913678 | 0.108 | 0.00982 | 2.73 | -2.9461327 | 1.05 |
| CD55 | ILMN_1800540 | 4.47E-05 | 4.24E-08 | 6.93 | 8.5386138 | 1.04 |
| SELPLG | ILMN_1734855 | 0.037 | 0.00159 | 3.42 | -1.2990263 | 1.04 |
| MAPK14 | ILMN_1737627 | 0.0747 | 0.00516 | 2.98 | -2.367876 | 1.04 |
| HLX | ILMN_1686862 | 0.154 | 0.0185 | 2.47 | -3.5041429 | 1.04 |
| ZC3HAV1 | ILMN_1724837 | 0.238 | 0.0407 | 2.12 | -4.1874548 | 1.04 |
| INHBB | ILMN_1685714 | 0.0966 | 0.0081 | 2.8 | -2.7742499 | 1.03 |
| CD177 | ILMN_3251610 | 0.161 | 0.0199 | 2.44 | -3.5701499 | 1.02 |
| GADD45A | ILMN_1694075 | 0.00426 | 5.40E-05 | 4.58 | 1.8419604 | 1.01 |
| ST6GALNAC3 | ILMN_1779061 | 0.00931 | 0.000184 | 4.17 | 0.6989146 | 1.01 |
| IQCC | ILMN_1774589 | 0.0181 | 0.000522 | 3.81 | -0.2695833 | 1.01 |
| PTRH2 | ILMN_2311548 | 0.0607 | 0.00365 | 3.11 | -2.0542866 | 1 |
| ANXA3 | ILMN_1694548 | 0.17 | 0.0219 | 2.4 | -3.6533687 | 1 |
| CYTIP | ILMN_2092041 | 0.00371 | 4.32E-05 | 4.66 | 2.0505946 | 0.998 |
| MAP2K1 | ILMN_1694240 | 0.00842 | 0.000156 | 4.23 | 0.8519015 | 0.996 |
| ST6GALNAC3 | ILMN_2127379 | 0.00391 | 4.79E-05 | 4.62 | 1.9534762 | 0.995 |
| RGMA | ILMN_1717636 | 0.0159 | 0.00043 | 3.88 | -0.0911469 | 0.994 |
| OSBPL9 | ILMN_2313856 | 0.000316 | 8.62E-07 | 5.94 | 5.7208101 | 0.992 |
| ABHD5 | ILMN_1655702 | 0.00037 | 1.17E-06 | 5.84 | 5.4311464 | 0.99 |
| SNORD12C | ILMN_3249286 | 0.00458 | 6.00E-05 | 4.55 | 1.7440379 | 0.99 |
| CEP68 | ILMN_1808500 | 0.061 | 0.00369 | 3.11 | -2.0638782 | 0.989 |
| HDAC4 | ILMN_1764396 | 0.0857 | 0.00655 | 2.89 | -2.5836665 | 0.989 |
| ZBTB16 | ILMN_1750496 | 0.182 | 0.0249 | 2.34 | -3.7653417 | 0.989 |
| ACSL3 | ILMN_2360705 | 0.00369 | 4.25E-05 | 4.66 | 2.0658008 | 0.988 |
| CDC123 | ILMN_1678605 | 0.0594 | 0.00351 | 3.13 | -2.0185458 | 0.985 |
| NUDT16P1 | ILMN_3239937 | 0.22 | 0.0351 | 2.19 | -4.060286 | 0.984 |
| ASPH | ILMN_1693771 | 0.0193 | 0.00058 | 3.78 | -0.3678625 | 0.983 |
| LOC154761 | ILMN_3237627 | 0.211 | 0.0326 | 2.22 | -3.997981 | 0.973 |
| B3GNT5 | ILMN_1702609 | 0.0873 | 0.0068 | 2.87 | -2.6163566 | 0.971 |
| FAM170B | ILMN_1738047 | 0.0322 | 0.00127 | 3.5 | -1.0882168 | 0.97 |
| HIPK3 | ILMN_1746941 | 0.0612 | 0.00371 | 3.11 | -2.0694365 | 0.969 |
| PER1 | ILMN_1653125 | 0.0801 | 0.00581 | 2.93 | -2.4752351 | 0.964 |
| MSL3 | ILMN_3226505 | 0.00081 | 3.85E-06 | 5.45 | 4.3166285 | 0.959 |
| SDHAF3 | ILMN_1771348 | 0.102 | 0.00901 | 2.76 | -2.8688737 | 0.956 |
| IL36G | ILMN_2158713 | 0.0674 | 0.00439 | 3.04 | -2.2214894 | 0.953 |
| THADA | ILMN_1811624 | 0.0118 | 0.00027 | 4.04 | 0.342206 | 0.944 |
| RNASE6 | ILMN_1780533 | 0.0366 | 0.00157 | 3.42 | -1.286491 | 0.944 |
| STK3 | ILMN_1666453 | 0.135 | 0.0147 | 2.56 | -3.3027908 | 0.944 |
| LIX1L | ILMN_1708098 | 0.0409 | 0.0019 | 3.35 | -1.4599217 | 0.941 |
| ABHD2 | ILMN_2403446 | 0.0142 | 0.00037 | 3.93 | 0.0477783 | 0.939 |
| GRB10 | ILMN_1669617 | 0.0366 | 0.00157 | 3.42 | -1.2846349 | 0.939 |
| SH3BP5 | ILMN_1814773 | 0.00822 | 0.000148 | 4.25 | 0.8981397 | 0.935 |
| OSBPL1A | ILMN_2405602 | 0.00281 | 2.91E-05 | 4.79 | 2.4206474 | 0.932 |
| ABCG1 | ILMN_1695968 | 0.12 | 0.0118 | 2.65 | -3.1110424 | 0.929 |
| STOM | ILMN_1696419 | 0.0805 | 0.00588 | 2.93 | -2.4868642 | 0.928 |
| CREB5 | ILMN_1728677 | 0.00369 | 4.19E-05 | 4.67 | 2.0802577 | 0.925 |
| ADORA3 | ILMN_1730710 | 0.0775 | 0.00542 | 2.96 | -2.4132927 | 0.925 |
| NEDD9 | ILMN_1726164 | 0.14 | 0.0157 | 2.54 | -3.3615656 | 0.924 |
| ARID5A | ILMN_1689700 | 0.0142 | 0.000369 | 3.93 | 0.0519512 | 0.923 |
| LOC494141 | ILMN_3292784 | 0.108 | 0.00991 | 2.72 | -2.9540554 | 0.923 |
| CABLES2 | ILMN_1762407 | 0.199 | 0.0294 | 2.27 | -3.9075319 | 0.92 |
| TSPAN2 | ILMN_1810093 | 0.0746 | 0.00513 | 2.98 | -2.3629262 | 0.919 |
| BLOC1S2 | ILMN_1679782 | 0.112 | 0.0106 | 2.7 | -3.0176028 | 0.918 |
| VNN1 | ILMN_1674574 | 0.13 | 0.0138 | 2.59 | -3.2466454 | 0.914 |
| ARID5A | ILMN_2298567 | 0.000812 | 4.05E-06 | 5.44 | 4.2685448 | 0.911 |
| ZNF679 | ILMN_1666420 | 0.0618 | 0.00378 | 3.1 | -2.0878435 | 0.909 |
| GIMAP8 | ILMN_1747305 | 0.0508 | 0.00274 | 3.22 | -1.7950218 | 0.908 |
| PSMG1 | ILMN_1659285 | 0.0478 | 0.0025 | 3.25 | -1.7103851 | 0.907 |
| MBNL1 | ILMN_2313158 | 0.00297 | 3.18E-05 | 4.76 | 2.3365492 | 0.906 |
| PMP22 | ILMN_1785646 | 0.14 | 0.0157 | 2.54 | -3.3642491 | 0.906 |
| CNIH4 | ILMN_1714759 | 0.0962 | 0.00806 | 2.81 | -2.76924 | 0.905 |
| COL9A2 | ILMN_1685122 | 0.222 | 0.0356 | 2.18 | -4.073743 | 0.902 |
| UBC | ILMN_2252160 | 0.106 | 0.00953 | 2.74 | -2.9188682 | 0.901 |
| DENND1A | ILMN_1727315 | 0.0505 | 0.00272 | 3.22 | -1.7861989 | 0.899 |
| ZDHHC20 | ILMN_1654141 | 0.033 | 0.00133 | 3.48 | -1.1352156 | 0.898 |
| MARC1 | ILMN_1799106 | 0.135 | 0.0147 | 2.56 | -3.3051741 | 0.898 |
| ZEB2 | ILMN_1688698 | 0.0538 | 0.00302 | 3.18 | -1.8815401 | 0.897 |
| SERINC3 | ILMN_1665065 | 0.00904 | 0.000174 | 4.19 | 0.7491127 | 0.896 |
| ZDHHC19 | ILMN_1766896 | 0.155 | 0.0187 | 2.46 | -3.5131381 | 0.896 |
| MS4A6A | ILMN_2359800 | 0.196 | 0.0287 | 2.28 | -3.8867696 | 0.896 |
| CASP4 | ILMN_1778059 | 0.151 | 0.0179 | 2.48 | -3.4773049 | 0.894 |
| IFRD1 | ILMN_1687390 | 0.0885 | 0.00695 | 2.87 | -2.6359029 | 0.891 |
| FPR2 | ILMN_2392569 | 0.00886 | 0.000168 | 4.2 | 0.7813416 | 0.889 |
| ERN1 | ILMN_1731231 | 0.032 | 0.00126 | 3.5 | -1.0804242 | 0.888 |
| TTPAL | ILMN_1744442 | 0.0613 | 0.00372 | 3.1 | -2.0730217 | 0.884 |
| ERRFI1 | ILMN_1665510 | 0.249 | 0.0446 | 2.08 | -4.2643416 | 0.884 |
| GADD45A | ILMN_2052208 | 0.0126 | 0.000307 | 4 | 0.2221377 | 0.883 |
| EVI2B | ILMN_2042651 | 0.098 | 0.00831 | 2.79 | -2.796961 | 0.883 |
| KLHL2 | ILMN_1701837 | 4.47E-05 | 4.63E-08 | 6.9 | 8.4573595 | 0.881 |
| KDSR | ILMN_2154053 | 0.168 | 0.0214 | 2.41 | -3.6323153 | 0.881 |
| ATL3 | ILMN_1751086 | 0.127 | 0.0131 | 2.61 | -3.204691 | 0.88 |
| CMSS1 | ILMN_2215545 | 0.000407 | 1.40E-06 | 5.78 | 5.2638034 | 0.878 |
| DCTN6 | ILMN_1744059 | 0.00972 | 0.000197 | 4.15 | 0.6364286 | 0.878 |
| SYAP1 | ILMN_1698470 | 0.00256 | 2.54E-05 | 4.83 | 2.5461591 | 0.873 |
| UQCRH | ILMN_1792138 | 0.0196 | 0.000594 | 3.77 | -0.3905279 | 0.873 |
| PAG1 | ILMN_1736806 | 0.0296 | 0.00109 | 3.55 | -0.951085 | 0.873 |
| SLA | ILMN_2345898 | 0.0637 | 0.00398 | 3.08 | -2.1332589 | 0.871 |
| DSC2 | ILMN_2381257 | 0.0395 | 0.00178 | 3.38 | -1.4009173 | 0.87 |
| STAU1 | ILMN_1730363 | 0.0531 | 0.00296 | 3.19 | -1.8638018 | 0.87 |
| SNHG7 | ILMN_3227023 | 0.0552 | 0.00314 | 3.17 | -1.9181092 | 0.87 |
| TCAF2 | ILMN_3184048 | 0.025 | 0.00085 | 3.64 | -0.7210516 | 0.869 |
| ZNF684 | ILMN_1751393 | 0.109 | 0.0102 | 2.71 | -2.976886 | 0.869 |
| RNF13 | ILMN_1719867 | 0.000786 | 3.63E-06 | 5.47 | 4.3726155 | 0.867 |
| SNORD12 | ILMN_3236049 | 0.00369 | 4.21E-05 | 4.67 | 2.0740191 | 0.867 |
| LRRC41 | ILMN_1712755 | 0.0308 | 0.00118 | 3.53 | -1.0223386 | 0.866 |
| CARD16 | ILMN_3252556 | 0.0949 | 0.00791 | 2.81 | -2.7526586 | 0.866 |
| MBOAT1 | ILMN_1764082 | 0.0972 | 0.0082 | 2.8 | -2.7844253 | 0.866 |
| NLRC4 | ILMN_1796976 | 0.028 | 0.00101 | 3.58 | -0.8798321 | 0.865 |
| GNG10 | ILMN_1652003 | 0.109 | 0.0102 | 2.71 | -2.97627 | 0.865 |
| SNTB2 | ILMN_1808374 | 0.00822 | 0.000149 | 4.24 | 0.8942327 | 0.864 |
| SPRY1 | ILMN_1691860 | 0.0142 | 0.000362 | 3.94 | 0.0675508 | 0.863 |
| ZNF252P | ILMN_3243961 | 0.125 | 0.0128 | 2.62 | -3.1808932 | 0.861 |
| RAB43 | ILMN_1781182 | 0.00521 | 7.11E-05 | 4.49 | 1.5842837 | 0.86 |
| ABHD2 | ILMN_1723662 | 0.0142 | 0.000362 | 3.94 | 0.068354 | 0.86 |
| GPX1 | ILMN_1787412 | 0.211 | 0.0326 | 2.22 | -3.9965589 | 0.86 |
| REPS2 | ILMN_1724668 | 0.00631 | 9.96E-05 | 4.38 | 1.2698975 | 0.856 |
| ABCG1 | ILMN_1794782 | 0.0719 | 0.00481 | 3.01 | -2.3044712 | 0.856 |
| F5 | ILMN_1709233 | 0.0103 | 0.000217 | 4.12 | 0.5458854 | 0.855 |
| XPO6 | ILMN_1755235 | 0.000499 | 1.88E-06 | 5.69 | 4.9897082 | 0.852 |
| SDHAF3 | ILMN_2134039 | 0.0483 | 0.00255 | 3.25 | -1.7277891 | 0.852 |
| HIPK2 | ILMN_1912737 | 0.0126 | 0.000308 | 4 | 0.2194865 | 0.851 |
| SAV1 | ILMN_2050654 | 0.163 | 0.0203 | 2.43 | -3.588694 | 0.851 |
| TSC22D1 | ILMN_2412380 | 0.129 | 0.0136 | 2.6 | -3.2340861 | 0.85 |
| ECM1 | ILMN_2329735 | 0.0175 | 0.000498 | 3.83 | -0.2274623 | 0.847 |
| SNORD95 | ILMN_3243452 | 0.132 | 0.0142 | 2.58 | -3.2720254 | 0.846 |
| SLC31A2 | ILMN_1758938 | 0.0123 | 0.00029 | 4.02 | 0.2744755 | 0.845 |
| TCAF2 | ILMN_1740818 | 0.0846 | 0.00639 | 2.9 | -2.5607116 | 0.843 |
| HDDC3 | ILMN_1781638 | 0.164 | 0.0206 | 2.42 | -3.5991857 | 0.843 |
| ABHD17B | ILMN_2374362 | 0.0479 | 0.00251 | 3.25 | -1.7151617 | 0.842 |
| ANKRD9 | ILMN_2048607 | 0.0978 | 0.00828 | 2.8 | -2.7936797 | 0.842 |
| ADGRE5 | ILMN_1676718 | 0.000985 | 5.93E-06 | 5.31 | 3.9123105 | 0.841 |
| SLA | ILMN_1667371 | 0.0621 | 0.00382 | 3.1 | -2.0956261 | 0.841 |
| PELI1 | ILMN_1679268 | 7.68E-05 | 9.04E-08 | 6.68 | 7.832039 | 0.84 |
| RPS6KA3 | ILMN_1770822 | 0.0708 | 0.00471 | 3.02 | -2.2864631 | 0.839 |
| CMSS1 | ILMN_1705753 | 0.0022 | 2.01E-05 | 4.91 | 2.7658521 | 0.838 |
| SLC38A1 | ILMN_1769911 | 0.0734 | 0.00497 | 2.99 | -2.3339338 | 0.838 |
| GJB6 | ILMN_2226223 | 0.157 | 0.019 | 2.46 | -3.528569 | 0.837 |
| NFIL3 | ILMN_1707312 | 0.000639 | 2.75E-06 | 5.56 | 4.6338418 | 0.835 |
| TMEM185B | ILMN_2231021 | 0.11 | 0.0103 | 2.71 | -2.9891592 | 0.835 |
| BMX | ILMN_1796138 | 0.00114 | 7.49E-06 | 5.24 | 3.6929428 | 0.829 |
| MCTP2 | ILMN_1792682 | 0.13 | 0.0137 | 2.59 | -3.2414119 | 0.828 |
| FAM110B | ILMN_1679217 | 0.0639 | 0.00401 | 3.08 | -2.1396232 | 0.827 |
| SERPINA1 | ILMN_2256050 | 0.00139 | 1.04E-05 | 5.13 | 3.3890012 | 0.826 |
| ZBED1 | ILMN_1694466 | 0.00383 | 4.64E-05 | 4.64 | 1.9838799 | 0.826 |
| SMAP2 | ILMN_2081682 | 0.0352 | 0.00148 | 3.44 | -1.2335251 | 0.826 |
| SERPINA1 | ILMN_1764980 | 0.0993 | 0.00847 | 2.79 | -2.8139921 | 0.826 |
| RBM47 | ILMN_1689046 | 0.109 | 0.0102 | 2.71 | -2.9768838 | 0.826 |
| SLF1 | ILMN_2214278 | 0.0193 | 0.000581 | 3.78 | -0.3696313 | 0.823 |
| ERI1 | ILMN_3245659 | 0.0819 | 0.00606 | 2.92 | -2.5128411 | 0.823 |
| PAOX | ILMN_2278265 | 0.138 | 0.0153 | 2.55 | -3.336001 | 0.823 |
| CARD16 | ILMN_1726591 | 0.139 | 0.0155 | 2.54 | -3.353014 | 0.822 |
| LGALSL | ILMN_1673548 | 0.0409 | 0.00191 | 3.35 | -1.4631262 | 0.821 |
| VNN3 | ILMN_2387680 | 0.00123 | 8.34E-06 | 5.2 | 3.5914583 | 0.816 |
| PHC2 | ILMN_1670147 | 0.0552 | 0.00314 | 3.17 | -1.9180842 | 0.815 |
| PDE4D | ILMN_1791483 | 0.0867 | 0.00671 | 2.88 | -2.6046253 | 0.815 |
| FOXC1 | ILMN_1738401 | 0.124 | 0.0127 | 2.62 | -3.1762131 | 0.815 |
| ATPAF1 | ILMN_1790603 | 0.228 | 0.0375 | 2.16 | -4.1183199 | 0.815 |
| GRASP | ILMN_1705210 | 0.135 | 0.0147 | 2.56 | -3.302787 | 0.812 |
| TMEM2 | ILMN_1784661 | 0.00369 | 4.25E-05 | 4.66 | 2.064951 | 0.809 |
| IDI1 | ILMN_1755075 | 0.078 | 0.00548 | 2.96 | -2.4221879 | 0.809 |
| UHRF2 | ILMN_1691444 | 0.0342 | 0.00143 | 3.46 | -1.196847 | 0.808 |
| ABHD17B | ILMN_1785661 | 0.0381 | 0.00169 | 3.4 | -1.3502442 | 0.808 |
| JADE2 | ILMN_1795285 | 0.0305 | 0.00115 | 3.53 | -1.0007591 | 0.805 |
| PICALM | ILMN_1778709 | 0.0311 | 0.0012 | 3.52 | -1.0407589 | 0.805 |
| TEK | ILMN_2066151 | 0.0534 | 0.00297 | 3.19 | -1.8685926 | 0.805 |
| IL1RL1 | ILMN_2313672 | 0.158 | 0.0193 | 2.45 | -3.5440489 | 0.802 |
| GSTM2 | ILMN_2201580 | 0.0333 | 0.00136 | 3.48 | -1.1523748 | 0.8 |
| MBNL1 | ILMN_1807304 | 0.00626 | 9.79E-05 | 4.39 | 1.2863053 | 0.796 |
| HEMK1 | ILMN_1731014 | 0.029 | 0.00106 | 3.56 | -0.9284684 | 0.795 |
| TNPO1 | ILMN_1763627 | 0.00297 | 3.19E-05 | 4.76 | 2.3341948 | 0.793 |
| TES | ILMN_1746243 | 0.00824 | 0.00015 | 4.24 | 0.887237 | 0.793 |
| OSBPL9 | ILMN_2313851 | 0.00383 | 4.61E-05 | 4.64 | 1.990159 | 0.792 |
| ZNF429 | ILMN_1695413 | 0.0314 | 0.00122 | 3.51 | -1.0534061 | 0.792 |
| GJB6 | ILMN_1718671 | 0.114 | 0.0111 | 2.68 | -3.0531012 | 0.792 |
| MAML2 | ILMN_1765729 | 0.00145 | 1.10E-05 | 5.11 | 3.3318885 | 0.791 |
| SNORD32A | ILMN_1743217 | 0.0155 | 0.000413 | 3.9 | -0.0545509 | 0.79 |
| PRDM8 | ILMN_1802082 | 0.00513 | 6.90E-05 | 4.5 | 1.6129955 | 0.788 |
| CARD16 | ILMN_1724474 | 0.0964 | 0.00809 | 2.81 | -2.7721738 | 0.787 |
| PLPP2 | ILMN_1675523 | 0.162 | 0.0201 | 2.43 | -3.580432 | 0.785 |
| IKBIP | ILMN_2324994 | 0.0998 | 0.00855 | 2.78 | -2.8217077 | 0.783 |
| NTN3 | ILMN_1656040 | 0.179 | 0.0243 | 2.35 | -3.7448473 | 0.783 |
| SYNJ2 | ILMN_2215119 | 0.227 | 0.0373 | 2.16 | -4.1137421 | 0.783 |
| PRPS1 | ILMN_1667050 | 0.00452 | 5.89E-05 | 4.56 | 1.7609277 | 0.782 |
| PFKFB2 | ILMN_1796560 | 0.195 | 0.0281 | 2.29 | -3.8703811 | 0.782 |
| KLHL6 | ILMN_1768814 | 0.00705 | 0.000121 | 4.32 | 1.0902241 | 0.779 |
| CLDN18 | ILMN_2284157 | 0.0138 | 0.000351 | 3.95 | 0.0966272 | 0.779 |
| EMILIN2 | ILMN_1697268 | 0.0994 | 0.00849 | 2.79 | -2.8159634 | 0.779 |
| FAM19A2 | ILMN_2172318 | 0.243 | 0.0424 | 2.11 | -4.2211318 | 0.777 |
| SNTB2 | ILMN_1786766 | 0.0756 | 0.00523 | 2.98 | -2.3807579 | 0.776 |
| AFF1 | ILMN_1673119 | 0.104 | 0.00928 | 2.75 | -2.8949234 | 0.775 |
| CRYL1 | ILMN_1714397 | 0.0865 | 0.00667 | 2.88 | -2.5996627 | 0.774 |
| LHFPL2 | ILMN_1747744 | 0.182 | 0.0251 | 2.34 | -3.7705322 | 0.774 |
| MMP23A | ILMN_2317701 | 0.0395 | 0.00179 | 3.38 | -1.4037855 | 0.773 |
| MGAT4A | ILMN_1755643 | 0.0197 | 0.000601 | 3.77 | -0.4006256 | 0.772 |
| CD7 | ILMN_1792538 | 0.145 | 0.0165 | 2.52 | -3.4044269 | 0.771 |
| SPIDR | ILMN_1887174 | 0.0599 | 0.00356 | 3.12 | -2.0334878 | 0.77 |
| H2AFY | ILMN_1746171 | 0.0278 | 0.000995 | 3.59 | -0.8657821 | 0.769 |
| ARHGEF40 | ILMN_1705743 | 0.0344 | 0.00144 | 3.45 | -1.207134 | 0.768 |
| GTDC1 | ILMN_1762167 | 0.0104 | 0.000222 | 4.11 | 0.5218175 | 0.765 |
| ACVR1B | ILMN_2243308 | 0.0197 | 0.000599 | 3.77 | -0.3970095 | 0.765 |
| TET2 | ILMN_1788818 | 0.0182 | 0.000528 | 3.81 | -0.2808841 | 0.764 |
| SNORD33 | ILMN_1682354 | 0.197 | 0.0288 | 2.28 | -3.8912682 | 0.763 |
| IKBIP | ILMN_2324989 | 0.0876 | 0.00683 | 2.87 | -2.6210574 | 0.762 |
| MEGF9 | ILMN_2290118 | 0.106 | 0.0096 | 2.74 | -2.9256346 | 0.761 |
| NDC80 | ILMN_1664511 | 0.185 | 0.0257 | 2.33 | -3.7906259 | 0.761 |
| CECR6 | ILMN_1702229 | 0.127 | 0.0133 | 2.61 | -3.2152732 | 0.76 |
| TLR4 | ILMN_1706217 | 0.000985 | 5.87E-06 | 5.32 | 3.921559 | 0.756 |
| GDPD1 | ILMN_2106265 | 0.0885 | 0.00695 | 2.87 | -2.6360446 | 0.756 |
| ZAK | ILMN_1768110 | 0.187 | 0.0262 | 2.32 | -3.8076432 | 0.756 |
| IL13RA1 | ILMN_1768505 | 0.000759 | 3.47E-06 | 5.49 | 4.4155399 | 0.755 |
| RFPL4A | ILMN_3227341 | 0.129 | 0.0135 | 2.6 | -3.231503 | 0.751 |
| CR1 | ILMN_1742601 | 0.203 | 0.0305 | 2.25 | -3.941588 | 0.75 |
| AGPAT2 | ILMN_1732176 | 0.0109 | 0.00024 | 4.08 | 0.4502186 | 0.747 |
| CCT6A | ILMN_1719611 | 0.0466 | 0.00239 | 3.27 | -1.6684752 | 0.747 |
| REPS2 | ILMN_2405797 | 0.00981 | 2.00E-04 | 4.15 | 0.6218528 | 0.746 |
| CBLB | ILMN_1685580 | 0.206 | 0.0312 | 2.24 | -3.9609761 | 0.746 |
| SNORD89 | ILMN_3238662 | 0.000158 | 2.56E-07 | 6.34 | 6.8566002 | 0.745 |
| ATP1B3 | ILMN_1654322 | 0.00888 | 0.000169 | 4.2 | 0.7773657 | 0.741 |
| RASA2 | ILMN_1703688 | 0.00057 | 2.23E-06 | 5.63 | 4.8310472 | 0.74 |
| SV2A | ILMN_1702009 | 0.0398 | 0.00181 | 3.37 | -1.4165254 | 0.737 |
| EXT1 | ILMN_2129927 | 0.186 | 0.0259 | 2.32 | -3.7986361 | 0.737 |
| BRAF | ILMN_1652472 | 0.108 | 0.00984 | 2.73 | -2.9477356 | 0.735 |
| FGR | ILMN_2272519 | 0.174 | 0.023 | 2.38 | -3.6941465 | 0.735 |
| IL17RA | ILMN_1728724 | 0.0186 | 0.000544 | 3.8 | -0.3083215 | 0.734 |
| FCGR3A | ILMN_2112580 | 0.243 | 0.0424 | 2.11 | -4.2216988 | 0.734 |
| KLHL8 | ILMN_1714083 | 0.0784 | 0.00553 | 2.95 | -2.4310515 | 0.729 |
| CST7 | ILMN_1679826 | 0.0933 | 0.0077 | 2.82 | -2.7288495 | 0.728 |
| UBALD2 | ILMN_1775498 | 0.000407 | 1.36E-06 | 5.79 | 5.2945913 | 0.727 |
| EIF4E3 | ILMN_1796146 | 0.0116 | 0.000263 | 4.05 | 0.3659584 | 0.727 |
| TMCC3 | ILMN_1685493 | 0.0534 | 0.00298 | 3.19 | -1.8705485 | 0.727 |
| BMX | ILMN_1672307 | 0.00938 | 0.000188 | 4.17 | 0.6799598 | 0.726 |
| REPS2 | ILMN_1766425 | 0.0103 | 0.000213 | 4.12 | 0.5614237 | 0.724 |
| MDM2 | ILMN_1736829 | 0.151 | 0.0179 | 2.48 | -3.475395 | 0.722 |
| NCK2 | ILMN_1724718 | 0.00659 | 0.000107 | 4.36 | 1.2036806 | 0.721 |
| MIR21 | ILMN_3310840 | 0.0663 | 0.00428 | 3.05 | -2.1984987 | 0.721 |
| SLF1 | ILMN_1805985 | 0.0188 | 0.000557 | 3.79 | -0.3297479 | 0.719 |
| KLHL8 | ILMN_2189222 | 0.101 | 0.00867 | 2.78 | -2.8342112 | 0.719 |
| GSTM1 | ILMN_2391861 | 0.0793 | 0.00566 | 2.94 | -2.4518673 | 0.718 |
| FAM160B1 | ILMN_3182275 | 0.000943 | 5.30E-06 | 5.35 | 4.0168739 | 0.717 |
| SNORD48 | ILMN_2157020 | 0.248 | 0.0443 | 2.09 | -4.2585765 | 0.717 |
| TMEM185B | ILMN_2231020 | 0.258 | 0.0477 | 2.05 | -4.3207357 | 0.715 |
| SEC61A2 | ILMN_1779381 | 0.258 | 0.0478 | 2.05 | -4.3230657 | 0.715 |
| TLR8 | ILMN_1705047 | 0.0194 | 0.000586 | 3.77 | -0.3781736 | 0.713 |
| TMLHE | ILMN_1683575 | 0.0581 | 0.0034 | 3.14 | -1.991689 | 0.711 |
| RBM47 | ILMN_3306730 | 0.0677 | 0.00441 | 3.04 | -2.2273486 | 0.711 |
| SLC22A15 | ILMN_1730639 | 0.117 | 0.0115 | 2.67 | -3.0840536 | 0.711 |
| LOC105372881 | ILMN_1867439 | 0.00371 | 4.33E-05 | 4.66 | 2.0490171 | 0.71 |
| PIK3C2B | ILMN_2117323 | 0.246 | 0.0435 | 2.09 | -4.2441641 | 0.71 |
| MCL1 | ILMN_1756806 | 0.000191 | 3.87E-07 | 6.2 | 6.4719164 | 0.709 |
| OSBPL5 | ILMN_2307032 | 0.0322 | 0.00127 | 3.5 | -1.0923625 | 0.709 |
| TMX4 | ILMN_1702759 | 0.0735 | 0.00498 | 2.99 | -2.3360131 | 0.708 |
| FES | ILMN_1693650 | 0.149 | 0.0174 | 2.49 | -3.4524417 | 0.707 |
| KLHL15 | ILMN_1701648 | 0.229 | 0.0381 | 2.15 | -4.1316581 | 0.707 |
| FURIN | ILMN_1790228 | 0.00742 | 0.000129 | 4.29 | 1.0269284 | 0.706 |
| FPR2 | ILMN_1740875 | 0.0197 | 0.000598 | 3.77 | -0.3958175 | 0.706 |
| LOC102724190 | ILMN_1855346 | 0.0693 | 0.00456 | 3.03 | -2.2562267 | 0.705 |
| CELF2 | ILMN_2400947 | 0.101 | 0.00873 | 2.78 | -2.8406518 | 0.705 |
| ELF2 | ILMN_2252295 | 0.000265 | 6.23E-07 | 6.05 | 6.0246396 | 0.703 |
| ATP2B4 | ILMN_2367753 | 0.24 | 0.0411 | 2.12 | -4.1960077 | 0.703 |
| TTPAL | ILMN_2364131 | 0.135 | 0.0147 | 2.56 | -3.3036544 | 0.702 |
| GSR | ILMN_1775182 | 0.0422 | 0.002 | 3.34 | -1.505817 | 0.701 |
| DDX21 | ILMN_1735461 | 0.0606 | 0.00364 | 3.11 | -2.0514791 | 0.701 |
| TALDO1 | ILMN_1670998 | 0.0777 | 0.00544 | 2.96 | -2.4164118 | 0.701 |
| FOS | ILMN_1669523 | 0.00872 | 0.000165 | 4.21 | 0.800771 | 0.698 |
| CREG1 | ILMN_1680624 | 0.196 | 0.0286 | 2.28 | -3.8833093 | 0.698 |
| SLC5A9 | ILMN_1748366 | 0.0208 | 0.000641 | 3.74 | -0.4600367 | 0.696 |
| IFI16 | ILMN_1710937 | 0.177 | 0.0239 | 2.36 | -3.7293921 | 0.696 |
| ZNF250 | ILMN_1757230 | 0.0498 | 0.00267 | 3.23 | -1.7724063 | 0.695 |
| MSL3 | ILMN_1670723 | 0.11 | 0.0103 | 2.71 | -2.9920772 | 0.695 |
| HIST2H3D | ILMN_1664706 | 0.181 | 0.0246 | 2.35 | -3.7551027 | 0.694 |
| LCOR | ILMN_1689817 | 0.0127 | 0.000312 | 3.99 | 0.2065947 | 0.693 |
| RNF149 | ILMN_2112524 | 0.000877 | 4.50E-06 | 5.4 | 4.1703688 | 0.69 |
| KRTAP19-6 | ILMN_1784216 | 0.0596 | 0.00354 | 3.12 | -2.0273086 | 0.69 |
| RPS6KA3 | ILMN_1806294 | 0.0119 | 0.000275 | 4.04 | 0.3233979 | 0.689 |
| TGIF1 | ILMN_1702211 | 0.11 | 0.0103 | 2.71 | -2.989585 | 0.689 |
| TSC22D3 | ILMN_2376403 | 0.00301 | 3.28E-05 | 4.75 | 2.3081169 | 0.688 |
| ATP2B4 | ILMN_1680579 | 0.179 | 0.0244 | 2.35 | -3.7463283 | 0.688 |
| LCOR | ILMN_1872122 | 0.0619 | 0.00381 | 3.1 | -2.0931647 | 0.687 |
| CCNH | ILMN_2187830 | 0.0103 | 0.000215 | 4.12 | 0.5540098 | 0.686 |
| TUBA4A | ILMN_1784300 | 0.051 | 0.00277 | 3.21 | -1.805253 | 0.685 |
| ROCK1P1 | ILMN_3248379 | 0.0142 | 0.000366 | 3.94 | 0.058565 | 0.684 |
| ATP13A3 | ILMN_1663684 | 0.00927 | 0.000181 | 4.18 | 0.7126779 | 0.68 |
| RALB | ILMN_1676358 | 0.0155 | 0.000418 | 3.89 | -0.0643991 | 0.68 |
| RIMKLB | ILMN_3245707 | 0.0568 | 0.00329 | 3.15 | -1.9607301 | 0.68 |
| SH3BP5 | ILMN_1675898 | 0.0458 | 0.00229 | 3.29 | -1.6302226 | 0.679 |
| SNORD13 | ILMN_1892403 | 0.025 | 0.00085 | 3.64 | -0.7212306 | 0.678 |
| SHB | ILMN_1732612 | 0.136 | 0.015 | 2.56 | -3.3194196 | 0.678 |
| SECTM1 | ILMN_1652277 | 0.226 | 0.0371 | 2.17 | -4.1091628 | 0.678 |
| CR1 | ILMN_2388112 | 0.247 | 0.0438 | 2.09 | -4.2494276 | 0.678 |
| TREML2 | ILMN_1740864 | 0.133 | 0.0142 | 2.58 | -3.2746777 | 0.676 |
| ENTPD1 | ILMN_1773125 | 0.177 | 0.024 | 2.36 | -3.7314728 | 0.675 |
| GAS7 | ILMN_1745994 | 0.192 | 0.0274 | 2.3 | -3.8476291 | 0.675 |
| EXOC6 | ILMN_1651628 | 0.0602 | 0.00359 | 3.12 | -2.0403677 | 0.674 |
| GTDC1 | ILMN_1762741 | 0.0613 | 0.00372 | 3.1 | -2.0724744 | 0.674 |
| AFF1 | ILMN_2129015 | 0.0844 | 0.00636 | 2.9 | -2.5570036 | 0.674 |
| FNIP2 | ILMN_1819783 | 0.164 | 0.0205 | 2.42 | -3.5969243 | 0.673 |
| RNF122 | ILMN_1691119 | 0.0247 | 0.000833 | 3.65 | -0.702219 | 0.671 |
| VNN3 | ILMN_1804935 | 0.0135 | 0.00034 | 3.96 | 0.1278887 | 0.669 |
| SDC2 | ILMN_1784553 | 0.0921 | 0.00745 | 2.84 | -2.6993208 | 0.669 |
| RNF144A | ILMN_1777660 | 0.133 | 0.0143 | 2.58 | -3.2763141 | 0.669 |
| RALGAPA2 | ILMN_1726333 | 0.0138 | 0.00035 | 3.95 | 0.0995498 | 0.668 |
| RHOT1 | ILMN_1785762 | 0.0388 | 0.00172 | 3.39 | -1.3715521 | 0.667 |
| KCNJ4 | ILMN_1697620 | 0.21 | 0.0322 | 2.23 | -3.9876717 | 0.667 |
| MPZL3 | ILMN_1795739 | 0.104 | 0.0093 | 2.75 | -2.8967676 | 0.666 |
| LINC00152 | ILMN_3230508 | 0.169 | 0.0218 | 2.4 | -3.6501633 | 0.666 |
| KIF21A | ILMN_1681249 | 0.045 | 0.00221 | 3.3 | -1.5988773 | 0.663 |
| GJB2 | ILMN_1769388 | 0.187 | 0.0263 | 2.32 | -3.8132545 | 0.663 |
| SYN2 | ILMN_1781060 | 0.21 | 0.0322 | 2.23 | -3.9877707 | 0.66 |
| CCNA1 | ILMN_2157099 | 0.244 | 0.0426 | 2.1 | -4.2252 | 0.656 |
| PCNX1 | ILMN_1740010 | 0.000985 | 6.04E-06 | 5.31 | 3.8947362 | 0.655 |
| RBM47 | ILMN_3224926 | 0.072 | 0.00483 | 3.01 | -2.3083482 | 0.654 |
| HSCB | ILMN_2184789 | 0.0308 | 0.00118 | 3.53 | -1.0242307 | 0.653 |
| SNHG7 | ILMN_3233229 | 0.0454 | 0.00224 | 3.29 | -1.6113855 | 0.652 |
| WIPF1 | ILMN_2262957 | 0.0854 | 0.00651 | 2.89 | -2.5783066 | 0.652 |
| LOC102724002 | ILMN_1839719 | 0.0104 | 0.000217 | 4.12 | 0.542331 | 0.651 |
| CLN8 | ILMN_1701094 | 0.0238 | 0.000779 | 3.67 | -0.640913 | 0.651 |
| UBE2F | ILMN_2164242 | 0.0428 | 0.00205 | 3.33 | -1.5274492 | 0.65 |
| RAB43 | ILMN_1696230 | 0.0322 | 0.00127 | 3.5 | -1.0903509 | 0.649 |
| SNORD110 | ILMN_3235397 | 0.177 | 0.0237 | 2.36 | -3.7234449 | 0.649 |
| SLC36A4 | ILMN_2082324 | 0.0352 | 0.00149 | 3.44 | -1.236051 | 0.648 |
| PSMG1 | ILMN_1779264 | 0.0392 | 0.00176 | 3.38 | -1.3921986 | 0.648 |
| TLE3 | ILMN_2234412 | 0.0851 | 0.00646 | 2.89 | -2.5708955 | 0.647 |
| SRPK1 | ILMN_1798804 | 0.000825 | 4.16E-06 | 5.43 | 4.2448005 | 0.646 |
| MYO10 | ILMN_2232712 | 0.108 | 0.00995 | 2.72 | -2.9570964 | 0.646 |
| FAR2 | ILMN_2202915 | 0.171 | 0.0222 | 2.39 | -3.6666834 | 0.645 |
| FAM86B1 | ILMN_1658861 | 0.0802 | 0.00582 | 2.93 | -2.4773719 | 0.644 |
| KIAA1211L | ILMN_3243366 | 0.189 | 0.0267 | 2.31 | -3.8267318 | 0.644 |
| ARL4A | ILMN_1775405 | 0.206 | 0.0312 | 2.24 | -3.9602688 | 0.642 |
| VMP1 | ILMN_1692754 | 0.00742 | 0.000129 | 4.29 | 1.0294877 | 0.64 |
| RP9P | ILMN_3244444 | 0.112 | 0.0108 | 2.69 | -3.026588 | 0.64 |
| PPP1R3B | ILMN_2131103 | 0.173 | 0.0228 | 2.38 | -3.688525 | 0.64 |
| CSNK1D | ILMN_2286870 | 0.018 | 0.000513 | 3.82 | -0.2543402 | 0.639 |
| CSGALNACT2 | ILMN_1799208 | 0.123 | 0.0124 | 2.63 | -3.1536904 | 0.639 |
| MGAM | ILMN_1714643 | 0.0422 | 0.002 | 3.34 | -1.5050736 | 0.638 |
| PAG1 | ILMN_2055156 | 0.0524 | 0.00289 | 3.2 | -1.8433937 | 0.637 |
| ADGRE5 | ILMN_2413508 | 0.151 | 0.018 | 2.48 | -3.4809443 | 0.635 |
| LIPN | ILMN_3243466 | 0.00758 | 0.000135 | 4.28 | 0.9894127 | 0.634 |
| RFESD | ILMN_2100458 | 0.213 | 0.0332 | 2.22 | -4.0141793 | 0.634 |
| PYGL | ILMN_1696187 | 0.00828 | 0.000152 | 4.24 | 0.8754662 | 0.633 |
| FAR2 | ILMN_1694596 | 0.0567 | 0.00328 | 3.15 | -1.9581616 | 0.631 |
| GSTTP2 | ILMN_2246548 | 0.0809 | 0.00593 | 2.93 | -2.4931447 | 0.631 |
| C5orf30 | ILMN_1677292 | 0.106 | 0.00952 | 2.74 | -2.9179471 | 0.631 |
| ST3GAL1 | ILMN_1683313 | 0.00539 | 7.46E-05 | 4.48 | 1.5401666 | 0.628 |
| BMP6 | ILMN_1747650 | 0.0972 | 0.0082 | 2.8 | -2.7848831 | 0.628 |
| CLEC1A | ILMN_1691339 | 0.201 | 0.0301 | 2.26 | -3.9287095 | 0.628 |
| VAPA | ILMN_1690822 | 0.0649 | 0.00412 | 3.07 | -2.1655694 | 0.625 |
| ELF2 | ILMN_1691559 | 0.000925 | 4.96E-06 | 5.37 | 4.0802609 | 0.623 |
| PCDHB9 | ILMN_2047885 | 0.154 | 0.0185 | 2.47 | -3.5033927 | 0.623 |
| PRDM10 | ILMN_2352295 | 0.194 | 0.0281 | 2.29 | -3.8688956 | 0.623 |
| SNORD55 | ILMN_1680393 | 0.196 | 0.0284 | 2.28 | -3.8794388 | 0.622 |
| USP10 | ILMN_1721116 | 0.0695 | 0.00459 | 3.03 | -2.2628522 | 0.619 |
| MGAT5 | ILMN_1816244 | 0.101 | 0.00872 | 2.78 | -2.8394885 | 0.619 |
| EVI2A | ILMN_1662747 | 0.111 | 0.0105 | 2.7 | -3.0060901 | 0.618 |
| ACSL3 | ILMN_1666096 | 0.105 | 0.00933 | 2.75 | -2.9001726 | 0.617 |
| SYAP1 | ILMN_2089175 | 0.00185 | 1.55E-05 | 5 | 3.0099146 | 0.616 |
| YRDC | ILMN_1736008 | 0.00405 | 5.07E-05 | 4.61 | 1.900286 | 0.616 |
| KIAA1551 | ILMN_1726289 | 0.00031 | 7.74E-07 | 5.98 | 5.8216593 | 0.613 |
| EIF4E3 | ILMN_2225144 | 0.0113 | 0.000252 | 4.07 | 0.4057566 | 0.613 |
| ADAM19 | ILMN_1713751 | 0.047 | 0.00243 | 3.26 | -1.685829 | 0.613 |
| RABGEF1 | ILMN_2230577 | 0.0652 | 0.00416 | 3.06 | -2.1733498 | 0.612 |
| MANSC1 | ILMN_2142752 | 0.11 | 0.0104 | 2.71 | -2.9932326 | 0.612 |
| CHPT1 | ILMN_1729112 | 0.0549 | 0.0031 | 3.17 | -1.9080266 | 0.61 |
| SLC38A2 | ILMN_1651799 | 0.145 | 0.0166 | 2.51 | -3.4130803 | 0.61 |
| DSC2 | ILMN_1663119 | 0.181 | 0.0246 | 2.35 | -3.7556449 | 0.61 |
| RAB27A | ILMN_1665859 | 0.0535 | 0.00299 | 3.19 | -1.8737235 | 0.609 |
| GAB2 | ILMN_1815758 | 0.147 | 0.017 | 2.5 | -3.4305991 | 0.609 |
| ZFAS1 | ILMN_3188984 | 0.000407 | 1.40E-06 | 5.78 | 5.2691742 | 0.608 |
| BROX | ILMN_1654864 | 0.0398 | 0.00181 | 3.37 | -1.4173134 | 0.608 |
| ADAM9 | ILMN_1727524 | 0.0854 | 0.00652 | 2.89 | -2.5785433 | 0.608 |
| CHPT1 | ILMN_2202940 | 0.105 | 0.00945 | 2.74 | -2.9119585 | 0.607 |
| C10orf12 | ILMN_1665508 | 0.0465 | 0.00238 | 3.27 | -1.6641608 | 0.605 |
| FCMR | ILMN_1775542 | 0.127 | 0.0132 | 2.61 | -3.2062563 | 0.605 |
| FAM172A | ILMN_1654542 | 0.0306 | 0.00116 | 3.53 | -1.006056 | 0.604 |
| JMJD1C | ILMN_2410742 | 0.0128 | 0.000314 | 3.99 | 0.2005171 | 0.602 |
| SNORA18 | ILMN_3244348 | 0.0613 | 0.00373 | 3.1 | -2.0757852 | 0.602 |
| HAUS4 | ILMN_1771003 | 0.181 | 0.0248 | 2.34 | -3.7612068 | 0.602 |
| SPC24 | ILMN_2181432 | 0.15 | 0.0177 | 2.49 | -3.4650723 | 0.601 |
| GNG10 | ILMN_1757074 | 0.247 | 0.044 | 2.09 | -4.2525583 | 0.601 |
| REPS2 | ILMN_1656934 | 0.0256 | 0.00088 | 3.63 | -0.7527666 | 0.6 |
| YOD1 | ILMN_1678919 | 0.078 | 0.00549 | 2.96 | -2.424423 | 0.6 |
| LINC00999 | ILMN_1660227 | 0.132 | 0.0141 | 2.58 | -3.2670761 | 0.6 |
| ACTA2 | ILMN_1671703 | 0.253 | 0.0458 | 2.07 | -4.2863989 | 0.6 |
| ZCCHC6 | ILMN_1779677 | 0.000158 | 2.49E-07 | 6.35 | 6.8833209 | 0.599 |
| UTP18 | ILMN_1779584 | 0.174 | 0.0232 | 2.37 | -3.7039176 | 0.599 |
| PLXDC2 | ILMN_1753312 | 0.0467 | 0.0024 | 3.27 | -1.6740395 | 0.598 |
| SMARCD3 | ILMN_2309180 | 0.0921 | 0.00747 | 2.84 | -2.7009305 | 0.598 |
| SH3GLB1 | ILMN_1766045 | 0.00367 | 4.14E-05 | 4.67 | 2.0900055 | 0.597 |
| TEK | ILMN_1751576 | 0.177 | 0.0238 | 2.36 | -3.7267378 | 0.597 |
| PPM1K | ILMN_2070043 | 0.145 | 0.0166 | 2.52 | -3.4080699 | 0.596 |
| MAN1A1 | ILMN_1742187 | 0.2 | 0.0297 | 2.26 | -3.9180685 | 0.596 |
| ST3GAL1 | ILMN_2267135 | 0.071 | 0.00473 | 3.01 | -2.2899029 | 0.595 |
| CLEC7A | ILMN_1700610 | 0.00388 | 4.73E-05 | 4.63 | 1.9657944 | 0.594 |
| PCTP | ILMN_1802257 | 0.113 | 0.0109 | 2.69 | -3.0412239 | 0.594 |
| SKIL | ILMN_2098119 | 0.254 | 0.0459 | 2.07 | -4.2888562 | 0.594 |
| UGCG | ILMN_1736939 | 0.26 | 0.0484 | 2.04 | -4.3343533 | 0.594 |
| GYPC | ILMN_1682332 | 0.0896 | 0.00709 | 2.86 | -2.6544001 | 0.591 |
| PHC2 | ILMN_1808047 | 0.13 | 0.0138 | 2.59 | -3.2496874 | 0.591 |
| PDCL3 | ILMN_1667925 | 0.0333 | 0.00135 | 3.48 | -1.1491577 | 0.59 |
| GPX1 | ILMN_1749662 | 0.179 | 0.0242 | 2.35 | -3.7396806 | 0.59 |
| USP3 | ILMN_1725862 | 0.0245 | 0.000819 | 3.66 | -0.686237 | 0.588 |
| CCNH | ILMN_1742250 | 0.0434 | 0.00209 | 3.32 | -1.5457749 | 0.588 |
| GSTK1 | ILMN_1725241 | 0.0295 | 0.00109 | 3.56 | -0.9480432 | 0.587 |
| WNT2B | ILMN_1740269 | 0.036 | 0.00154 | 3.43 | -1.2648039 | 0.586 |
| SLC36A1 | ILMN_2124471 | 0.0922 | 0.00749 | 2.84 | -2.7035392 | 0.584 |
| H2AFY | ILMN_1674034 | 0.113 | 0.0109 | 2.69 | -3.0405292 | 0.584 |
| MGST2 | ILMN_1802027 | 0.243 | 0.042 | 2.11 | -4.2128685 | 0.584 |
| MANSC1 | ILMN_1652490 | 0.108 | 0.00996 | 2.72 | -2.9588045 | 0.583 |
| LIN7A | ILMN_1806293 | 0.202 | 0.0302 | 2.26 | -3.9311651 | 0.583 |
| PTEN | ILMN_1701134 | 0.0422 | 0.002 | 3.34 | -1.5053713 | 0.582 |
| SLCO4C1 | ILMN_1686464 | 0.0925 | 0.00754 | 2.83 | -2.709652 | 0.582 |
| C9orf72 | ILMN_1666742 | 0.187 | 0.0262 | 2.32 | -3.809614 | 0.582 |
| TCAF2 | ILMN_1778202 | 0.0162 | 0.000444 | 3.87 | -0.1202822 | 0.58 |
| BCL6 | ILMN_1737314 | 0.0479 | 0.00251 | 3.25 | -1.713777 | 0.58 |
| RAD23B | ILMN_1722662 | 0.0423 | 0.002 | 3.34 | -1.5068311 | 0.579 |
| TMLHE | ILMN_1707002 | 0.0579 | 0.00339 | 3.14 | -1.9881939 | 0.579 |
| LCOR | ILMN_2062381 | 0.0374 | 0.00163 | 3.41 | -1.3207264 | 0.578 |
| PDCD4-AS1 | ILMN_3199489 | 0.00705 | 0.000121 | 4.32 | 1.09132 | 0.577 |
| CUL4B | ILMN_1691535 | 0.0745 | 0.00511 | 2.98 | -2.3591336 | 0.576 |
| GRB10 | ILMN_1652662 | 0.127 | 0.0131 | 2.61 | -3.2045569 | 0.576 |
| ITPR1 | ILMN_1789505 | 0.255 | 0.0465 | 2.06 | -4.2992054 | 0.576 |
| KIAA0319L | ILMN_1683277 | 0.0497 | 0.00266 | 3.23 | -1.768729 | 0.574 |
| PRPS2 | ILMN_1758104 | 0.102 | 0.009 | 2.76 | -2.8683944 | 0.573 |
| SERINC3 | ILMN_1713752 | 0.0902 | 0.00721 | 2.85 | -2.6693834 | 0.572 |
| DNMBP | ILMN_1702443 | 0.078 | 0.00549 | 2.96 | -2.4250634 | 0.571 |
| ARMCX6 | ILMN_1716026 | 0.0261 | 0.000912 | 3.62 | -0.7855589 | 0.57 |
| ANKRD23 | ILMN_1747151 | 0.0458 | 0.00229 | 3.29 | -1.630893 | 0.57 |
| MGARP | ILMN_2072101 | 0.19 | 0.027 | 2.31 | -3.8345811 | 0.57 |
| PRPSAP2 | ILMN_1812445 | 0.0135 | 0.000338 | 3.97 | 0.1331175 | 0.569 |
| DLX1 | ILMN_2388445 | 0.135 | 0.0147 | 2.56 | -3.3022111 | 0.569 |
| 8-Mar | ILMN_2336335 | 0.224 | 0.0362 | 2.18 | -4.0867364 | 0.569 |
| CXCR1 | ILMN_1662524 | 0.000747 | 3.38E-06 | 5.5 | 4.4402094 | 0.568 |
| TMEM181 | ILMN_1710078 | 0.181 | 0.0246 | 2.35 | -3.755577 | 0.568 |
| CSF2RA | ILMN_1661196 | 0.199 | 0.0293 | 2.27 | -3.9070255 | 0.566 |
| PITPNC1 | ILMN_1670638 | 0.00513 | 6.98E-05 | 4.5 | 1.6024887 | 0.565 |
| TLE1 | ILMN_1751572 | 0.0983 | 0.00834 | 2.79 | -2.7998993 | 0.565 |
| PRR21 | ILMN_1704043 | 0.126 | 0.013 | 2.62 | -3.1924104 | 0.565 |
| SP140 | ILMN_1703263 | 0.112 | 0.0106 | 2.7 | -3.0169281 | 0.562 |
| REPS2 | ILMN_1759633 | 0.184 | 0.0253 | 2.34 | -3.7783413 | 0.562 |
| TSPAN14 | ILMN_1785060 | 0.084 | 0.0063 | 2.9 | -2.5477137 | 0.561 |
| UBR3 | ILMN_3242176 | 0.0911 | 0.00734 | 2.84 | -2.6858666 | 0.56 |
| PHACTR4 | ILMN_1736548 | 0.193 | 0.0279 | 2.29 | -3.8620812 | 0.56 |
| TMCO3 | ILMN_2220739 | 0.013 | 0.00032 | 3.98 | 0.1828875 | 0.559 |
| SLC7A8 | ILMN_1807894 | 0.165 | 0.0209 | 2.42 | -3.6139387 | 0.559 |
| CPEB4 | ILMN_1722025 | 0.0322 | 0.00127 | 3.5 | -1.0910837 | 0.558 |
| FLVCR2 | ILMN_2204876 | 0.0325 | 0.0013 | 3.49 | -1.1110608 | 0.558 |
| MAT2A | ILMN_1737298 | 0.131 | 0.0139 | 2.59 | -3.2573117 | 0.558 |
| 2-Mar | ILMN_1669592 | 0.258 | 0.048 | 2.05 | -4.3262198 | 0.558 |
| H6PD | ILMN_1721136 | 0.0894 | 0.00708 | 2.86 | -2.6526631 | 0.557 |
| LRRK1 | ILMN_1696004 | 0.0607 | 0.00365 | 3.11 | -2.0548557 | 0.556 |
| NSUN7 | ILMN_1767960 | 0.16 | 0.0196 | 2.44 | -3.5566205 | 0.556 |
| CLK1 | ILMN_1652790 | 0.105 | 0.00946 | 2.74 | -2.9124752 | 0.555 |
| ANGPTL4 | ILMN_2386444 | 0.0459 | 0.0023 | 3.28 | -1.6362427 | 0.554 |
| FCGR2A | ILMN_1706523 | 0.124 | 0.0126 | 2.63 | -3.1681991 | 0.554 |
| SLC12A6 | ILMN_1767992 | 0.136 | 0.015 | 2.56 | -3.3192959 | 0.554 |
| C5orf34 | ILMN_1662184 | 0.0538 | 0.00302 | 3.18 | -1.8835341 | 0.553 |
| RNF144A | ILMN_3238326 | 0.193 | 0.0278 | 2.29 | -3.8606236 | 0.553 |
| RABGEF1 | ILMN_2230579 | 0.105 | 0.00946 | 2.74 | -2.91274 | 0.551 |
| YWHAZ | ILMN_1669286 | 0.101 | 0.00868 | 2.78 | -2.8355831 | 0.55 |
| PSMA1 | ILMN_1760542 | 0.154 | 0.0185 | 2.47 | -3.5046852 | 0.55 |
| SNTB1 | ILMN_1793410 | 0.219 | 0.0347 | 2.2 | -4.0517311 | 0.549 |
| ADAM15 | ILMN_1799728 | 0.0695 | 0.00461 | 3.02 | -2.2662307 | 0.545 |
| VRK2 | ILMN_1750088 | 0.111 | 0.0105 | 2.7 | -3.0065939 | 0.545 |
| MGAT5 | ILMN_3238058 | 0.13 | 0.0137 | 2.59 | -3.2413319 | 0.545 |
| PALD1 | ILMN_1796751 | 0.108 | 0.00986 | 2.73 | -2.9492894 | 0.544 |
| AKIRIN2 | ILMN_2148847 | 0.246 | 0.0435 | 2.09 | -4.2429984 | 0.544 |
| ARID5A | ILMN_2415157 | 0.0183 | 0.000535 | 3.81 | -0.292552 | 0.543 |
| CLEC7A | ILMN_2323992 | 0.0465 | 0.00237 | 3.27 | -1.6612384 | 0.543 |
| MAPKAPK2 | ILMN_1774844 | 0.0702 | 0.00466 | 3.02 | -2.2766784 | 0.543 |
| C16orf72 | ILMN_1773407 | 0.12 | 0.0119 | 2.65 | -3.1197587 | 0.543 |
| FAM45A | ILMN_1691760 | 0.177 | 0.0239 | 2.36 | -3.7299273 | 0.542 |
| ANG | ILMN_1760727 | 0.237 | 0.0401 | 2.13 | -4.175265 | 0.541 |
| TNFAIP3 | ILMN_1702691 | 0.179 | 0.0243 | 2.35 | -3.7450716 | 0.539 |
| FAM134B | ILMN_2283597 | 0.256 | 0.0471 | 2.06 | -4.3105163 | 0.539 |
| ENSA | ILMN_1760779 | 0.00155 | 1.20E-05 | 5.08 | 3.2479511 | 0.538 |
| DYSF | ILMN_1810420 | 0.0921 | 0.00746 | 2.84 | -2.6997543 | 0.538 |
| SESTD1 | ILMN_1724495 | 0.103 | 0.00907 | 2.76 | -2.8746401 | 0.538 |
| ADD3 | ILMN_2311278 | 0.0979 | 0.0083 | 2.8 | -2.79517 | 0.537 |
| ANTXR2 | ILMN_1812926 | 0.186 | 0.0259 | 2.32 | -3.7996262 | 0.536 |
| SLC46A3 | ILMN_1658639 | 0.237 | 0.0402 | 2.13 | -4.1766202 | 0.536 |
| EXOC4 | ILMN_1742827 | 0.0549 | 0.0031 | 3.17 | -1.9075535 | 0.535 |
| HN1 | ILMN_2384591 | 0.172 | 0.0226 | 2.38 | -3.6791883 | 0.535 |
| CMAHP | ILMN_1704084 | 0.248 | 0.0443 | 2.08 | -4.2590208 | 0.535 |
| POLR2J4 | ILMN_1699383 | 0.0618 | 0.00378 | 3.1 | -2.0868685 | 0.534 |
| NDEL1 | ILMN_2397199 | 0.00639 | 0.000103 | 4.37 | 1.2394289 | 0.533 |
| ST3GAL1 | ILMN_1860638 | 0.0847 | 0.00641 | 2.9 | -2.5639426 | 0.532 |
| TUBA1C | ILMN_1742167 | 0.134 | 0.0145 | 2.57 | -3.2885016 | 0.532 |
| AATK | ILMN_1699334 | 0.0821 | 0.00609 | 2.92 | -2.5171529 | 0.531 |
| ARAP1 | ILMN_1786357 | 0.112 | 0.0107 | 2.69 | -3.0218812 | 0.531 |
| DGCR11 | ILMN_3236211 | 0.0465 | 0.00236 | 3.27 | -1.6586274 | 0.53 |
| ACSL3 | ILMN_1654414 | 0.0739 | 0.00502 | 2.99 | -2.3440747 | 0.53 |
| H2AFY | ILMN_2373495 | 0.105 | 0.00935 | 2.75 | -2.9021532 | 0.53 |
| PIGK | ILMN_1772743 | 0.119 | 0.0117 | 2.66 | -3.0985159 | 0.53 |
| SNORD104 | ILMN_3248218 | 0.151 | 0.0178 | 2.48 | -3.4736779 | 0.53 |
| PHF12 | ILMN_1808781 | 0.155 | 0.0186 | 2.47 | -3.5105451 | 0.53 |
| GK | ILMN_1725471 | 0.0653 | 0.00417 | 3.06 | -2.1758091 | 0.529 |
| DKFZP586I1420 | ILMN_1803856 | 0.1 | 0.0086 | 2.78 | -2.8276764 | 0.529 |
| CYTIP | ILMN_1746864 | 0.00705 | 0.000121 | 4.32 | 1.0917896 | 0.528 |
| MEX3A | ILMN_3239177 | 0.106 | 0.00962 | 2.74 | -2.9274391 | 0.528 |
| FOXP1 | ILMN_1682495 | 0.122 | 0.0122 | 2.64 | -3.1362937 | 0.528 |
| UQCRHL | ILMN_1718136 | 0.0473 | 0.00246 | 3.26 | -1.6955184 | 0.527 |
| PITPNC1 | ILMN_1738796 | 0.129 | 0.0136 | 2.6 | -3.2333619 | 0.527 |
| ETS1 | ILMN_1687538 | 0.141 | 0.0159 | 2.53 | -3.3733051 | 0.527 |
| ERI1 | ILMN_1652716 | 0.219 | 0.0349 | 2.19 | -4.0554476 | 0.527 |
| WLS | ILMN_1753913 | 0.222 | 0.0358 | 2.18 | -4.0785615 | 0.527 |
| JDP2 | ILMN_1747205 | 0.256 | 0.0468 | 2.06 | -4.3053792 | 0.527 |
| CRISPLD2 | ILMN_1790689 | 0.178 | 0.0241 | 2.36 | -3.7373605 | 0.526 |
| ABHD17B | ILMN_1660440 | 0.196 | 0.0284 | 2.28 | -3.8801127 | 0.526 |
| MYLIP | ILMN_1656111 | 0.185 | 0.0255 | 2.33 | -3.7854971 | 0.525 |
| TNPO1 | ILMN_1837935 | 0.00828 | 0.000153 | 4.24 | 0.8709535 | 0.524 |
| GYPC | ILMN_1668039 | 0.129 | 0.0135 | 2.6 | -3.2312025 | 0.524 |
| ZC3H18 | ILMN_1658834 | 0.0868 | 0.00672 | 2.88 | -2.6067353 | 0.523 |
| PRDM1 | ILMN_1655077 | 0.161 | 0.0197 | 2.44 | -3.5624591 | 0.523 |
| OGFOD3 | ILMN_1811991 | 0.228 | 0.0377 | 2.16 | -4.1213324 | 0.523 |
| GYG1 | ILMN_2230862 | 0.198 | 0.0291 | 2.27 | -3.8983776 | 0.522 |
| STRN | ILMN_1749882 | 0.0811 | 0.00596 | 2.92 | -2.4978354 | 0.52 |
| STRIP2 | ILMN_2161286 | 0.121 | 0.012 | 2.65 | -3.1275248 | 0.52 |
| KLF6 | ILMN_1735014 | 0.000414 | 1.48E-06 | 5.76 | 5.2133287 | 0.519 |
| ENTPD3 | ILMN_2087941 | 0.0911 | 0.00734 | 2.84 | -2.6860824 | 0.519 |
| ANXA11 | ILMN_1807003 | 0.162 | 0.0202 | 2.43 | -3.5834847 | 0.519 |
| RNF24 | ILMN_1717809 | 0.195 | 0.0282 | 2.29 | -3.8725515 | 0.519 |
| NCK2 | ILMN_2356632 | 0.102 | 0.00893 | 2.77 | -2.8611448 | 0.518 |
| ERV3-1 | ILMN_2118663 | 0.0859 | 0.00659 | 2.89 | -2.5884067 | 0.517 |
| CRK | ILMN_1666494 | 0.175 | 0.0233 | 2.37 | -3.705539 | 0.517 |
| SKP1 | ILMN_3229770 | 0.0873 | 0.00679 | 2.87 | -2.6162347 | 0.515 |
| DNAJB5 | ILMN_1794056 | 0.169 | 0.0217 | 2.4 | -3.6468683 | 0.515 |
| GLCCI1 | ILMN_1781431 | 0.222 | 0.0357 | 2.18 | -4.0748334 | 0.515 |
| RNF10 | ILMN_1697529 | 0.174 | 0.023 | 2.38 | -3.6965123 | 0.514 |
| JADE1 | ILMN_1655194 | 0.0169 | 0.000468 | 3.85 | -0.1694696 | 0.513 |
| GPR1 | ILMN_2107004 | 0.127 | 0.0131 | 2.61 | -3.2045399 | 0.513 |
| NSUN7 | ILMN_2205882 | 0.154 | 0.0185 | 2.47 | -3.5052411 | 0.513 |
| SLC5A10 | ILMN_2412849 | 0.157 | 0.019 | 2.46 | -3.5281144 | 0.513 |
| TBC1D15 | ILMN_1803941 | 0.188 | 0.0266 | 2.31 | -3.8212565 | 0.513 |
| TMEM207 | ILMN_1704022 | 0.198 | 0.0291 | 2.27 | -3.8991337 | 0.512 |
| PDK4 | ILMN_1684982 | 0.0679 | 0.00443 | 3.04 | -2.2300364 | 0.51 |
| CCDC6 | ILMN_1745904 | 0.141 | 0.0158 | 2.53 | -3.3683109 | 0.51 |
| PACSIN2 | ILMN_3307772 | 0.0297 | 0.00111 | 3.55 | -0.9655698 | 0.508 |
| LMO2 | ILMN_1800078 | 0.0857 | 0.00656 | 2.89 | -2.5844768 | 0.508 |
| IGFBPL1 | ILMN_1677158 | 0.218 | 0.0347 | 2.2 | -4.0505133 | 0.508 |
| MSANTD3 | ILMN_1714278 | 0.15 | 0.0177 | 2.49 | -3.4653073 | 0.507 |
| EPB41L4A | ILMN_1791867 | 0.0944 | 0.00784 | 2.82 | -2.744624 | 0.506 |
| SIRT5 | ILMN_1799598 | 0.108 | 0.00985 | 2.73 | -2.9480508 | 0.506 |
| NUFIP2 | ILMN_2102693 | 0.0219 | 0.00069 | 3.72 | -0.5286618 | 0.505 |
| OGFRL1 | ILMN_2095759 | 0.201 | 0.0301 | 2.26 | -3.92802 | 0.505 |
| OSBPL1A | ILMN_1773063 | 0.0575 | 0.00335 | 3.14 | -1.9768622 | 0.504 |
| SNORD1A | ILMN_3244281 | 0.109 | 0.0101 | 2.72 | -2.9710259 | 0.504 |
| ZFP36L2 | ILMN_2150258 | 0.176 | 0.0236 | 2.36 | -3.7190682 | 0.504 |
| CELF2 | ILMN_1800638 | 0.228 | 0.0376 | 2.16 | -4.119499 | 0.504 |
| UQCRH | ILMN_2232936 | 0.0296 | 0.0011 | 3.55 | -0.9561405 | 0.503 |
| DEK | ILMN_1747630 | 0.0306 | 0.00117 | 3.53 | -1.0144627 | 0.503 |
| PRMT5 | ILMN_1811955 | 0.133 | 0.0144 | 2.57 | -3.2846176 | 0.503 |
| C11orf96 | ILMN_1677402 | 0.159 | 0.0194 | 2.45 | -3.5469723 | 0.503 |
| TBC1D32 | ILMN_2228196 | 0.162 | 0.0202 | 2.43 | -3.5806128 | 0.503 |
| FOXP1 | ILMN_2250923 | 0.176 | 0.0236 | 2.36 | -3.7190808 | 0.503 |
| DOCK3 | ILMN_1712352 | 0.247 | 0.0439 | 2.09 | -4.2521145 | 0.503 |
| NFE2L2 | ILMN_1790909 | 0.0186 | 0.000546 | 3.8 | -0.3117168 | 0.502 |
| LGALS8 | ILMN_2266214 | 0.13 | 0.0138 | 2.59 | -3.2465032 | 0.502 |
| MLX | ILMN_1792207 | 0.0998 | 0.00857 | 2.78 | -2.823901 | 0.501 |
| HBA2 | ILMN_2127842 | 0.148 | 0.0172 | 2.5 | -3.4434558 | 0.501 |
| LONP2 | ILMN_2216265 | 0.246 | 0.0434 | 2.09 | -4.2408626 | 0.501 |
| ENSA | ILMN_2364700 | 0.00142 | 1.07E-05 | 5.12 | 3.3601322 | 0.5 |
| ZNF493 | ILMN_1667858 | 0.103 | 0.00913 | 2.76 | -2.8809318 | 0.5 |
| BCL2L11 | ILMN_1766541 | 0.232 | 0.039 | 2.14 | -4.1503109 | 0.5 |
| ZNF473 | ILMN_2346649 | 0.0926 | 0.00761 | 2.83 | -2.7181347 | 0.499 |
| ZDHHC3 | ILMN_2201347 | 0.0104 | 0.000218 | 4.11 | 0.5384042 | 0.498 |
| PLP1 | ILMN_1790106 | 0.105 | 0.00936 | 2.75 | -2.9028003 | 0.498 |
| TRIM73 | ILMN_1790253 | 0.124 | 0.0127 | 2.62 | -3.1756874 | 0.498 |
| NUP153 | ILMN_1705907 | 0.0373 | 0.00162 | 3.41 | -1.3150394 | 0.497 |
| MME | ILMN_1678170 | 0.196 | 0.0286 | 2.28 | -3.8860968 | 0.497 |
| JPH2 | ILMN_1802434 | 0.0801 | 0.0058 | 2.94 | -2.4737713 | 0.495 |
| SIGLEC6 | ILMN_1685630 | 0.142 | 0.0161 | 2.53 | -3.3829725 | 0.495 |
| EXO5 | ILMN_2117569 | 0.0472 | 0.00245 | 3.26 | -1.6928884 | 0.494 |
| TCAIM | ILMN_1671387 | 0.0798 | 0.00575 | 2.94 | -2.4664605 | 0.494 |
| ZNF407 | ILMN_1741300 | 0.238 | 0.0407 | 2.12 | -4.1862425 | 0.494 |
| PTPRC | ILMN_1804279 | 0.236 | 0.0398 | 2.13 | -4.1682886 | 0.493 |
| UBXN2B | ILMN_3236556 | 0.00475 | 6.26E-05 | 4.54 | 1.7040206 | 0.492 |
| APP | ILMN_2404065 | 0.0126 | 0.000302 | 4 | 0.2366832 | 0.492 |
| FAM153A | ILMN_1745669 | 0.0992 | 0.00844 | 2.79 | -2.8109486 | 0.492 |
| CTH | ILMN_1777060 | 0.0876 | 0.00684 | 2.87 | -2.6223801 | 0.491 |
| SUMO1P1 | ILMN_1785615 | 0.119 | 0.0117 | 2.66 | -3.1022919 | 0.491 |
| KCNJ15 | ILMN_1675756 | 0.176 | 0.0236 | 2.36 | -3.7190963 | 0.491 |
| ARMCX6 | ILMN_2414533 | 0.0317 | 0.00124 | 3.51 | -1.0654426 | 0.49 |
| MPRIP | ILMN_1774547 | 0.145 | 0.0166 | 2.51 | -3.4132523 | 0.49 |
| LMO4 | ILMN_1703487 | 0.0171 | 0.00048 | 3.84 | -0.1936798 | 0.489 |
| RAX2 | ILMN_1653412 | 0.0465 | 0.00236 | 3.27 | -1.6584128 | 0.488 |
| KCNJ15 | ILMN_2396903 | 0.257 | 0.0472 | 2.06 | -4.3120148 | 0.487 |
| SWAP70 | ILMN_1785175 | 0.0246 | 0.000828 | 3.65 | -0.6968356 | 0.486 |
| IFT88 | ILMN_1669366 | 0.031 | 0.0012 | 3.52 | -1.0355939 | 0.486 |
| ATPIF1 | ILMN_1685978 | 0.0627 | 0.00389 | 3.09 | -2.1134769 | 0.486 |
| TMEM191A | ILMN_3228529 | 0.12 | 0.0119 | 2.65 | -3.1184635 | 0.486 |
| ZNF331 | ILMN_1711199 | 0.163 | 0.0204 | 2.43 | -3.5933072 | 0.486 |
| PFKFB2 | ILMN_2385298 | 0.246 | 0.0434 | 2.09 | -4.2423094 | 0.485 |
| RNF13 | ILMN_2339748 | 0.00301 | 3.28E-05 | 4.75 | 2.3084804 | 0.484 |
| ZNF493 | ILMN_2278653 | 0.0244 | 0.000805 | 3.66 | -0.6707939 | 0.484 |
| BRSK1 | ILMN_2185845 | 0.228 | 0.0377 | 2.16 | -4.1215703 | 0.483 |
| OLAH | ILMN_1674696 | 0.248 | 0.0445 | 2.08 | -4.2622346 | 0.483 |
| CXCR2 | ILMN_1680397 | 0.0142 | 0.000369 | 3.93 | 0.0518597 | 0.482 |
| HSDL2 | ILMN_1787843 | 0.0663 | 0.00428 | 3.05 | -2.1995965 | 0.481 |
| DUSP22 | ILMN_1730765 | 0.255 | 0.0462 | 2.07 | -4.2953066 | 0.481 |
| PDE4B | ILMN_1782922 | 0.00369 | 4.27E-05 | 4.66 | 2.0622767 | 0.48 |
| NTSR1 | ILMN_1778831 | 0.173 | 0.0227 | 2.38 | -3.6861095 | 0.479 |
| TSC22D3 | ILMN_1748124 | 0.00855 | 0.000159 | 4.22 | 0.8340538 | 0.478 |
| TLR8 | ILMN_1682251 | 0.0925 | 0.00755 | 2.83 | -2.711104 | 0.478 |
| CLDN14 | ILMN_2328575 | 0.209 | 0.032 | 2.23 | -3.9818274 | 0.477 |
| LHFP | ILMN_1767448 | 0.151 | 0.0178 | 2.48 | -3.4733037 | 0.476 |
| KDSR | ILMN_3241164 | 0.262 | 0.0491 | 2.04 | -4.3460108 | 0.476 |
| GAB2 | ILMN_1665964 | 0.0306 | 0.00117 | 3.53 | -1.0121805 | 0.475 |
| CASP2 | ILMN_2410540 | 0.0944 | 0.00783 | 2.82 | -2.7435299 | 0.475 |
| PPP2R2B | ILMN_2298365 | 0.00352 | 3.86E-05 | 4.7 | 2.155474 | 0.474 |
| SIPA1L1 | ILMN_2128162 | 0.105 | 0.00944 | 2.74 | -2.9109016 | 0.474 |
| TXN | ILMN_1680314 | 0.165 | 0.0209 | 2.42 | -3.6104857 | 0.474 |
| NEK7 | ILMN_1758548 | 0.247 | 0.044 | 2.09 | -4.2526232 | 0.474 |
| PABPC5 | ILMN_1796926 | 0.131 | 0.0139 | 2.59 | -3.2516315 | 0.473 |
| UGT1A3 | ILMN_2206420 | 0.172 | 0.0225 | 2.39 | -3.6749732 | 0.473 |
| ALX1 | ILMN_3236443 | 0.196 | 0.0286 | 2.28 | -3.8839755 | 0.473 |
| ACER3 | ILMN_1812552 | 0.199 | 0.0294 | 2.27 | -3.9099125 | 0.473 |
| IL1RAP | ILMN_2357062 | 0.0627 | 0.00386 | 3.09 | -2.1069844 | 0.472 |
| SPTLC2 | ILMN_1704290 | 0.212 | 0.033 | 2.22 | -4.0068338 | 0.472 |
| MMGT1 | ILMN_3237986 | 0.101 | 0.0088 | 2.77 | -2.8480354 | 0.471 |
| TMX4 | ILMN_2204754 | 0.134 | 0.0146 | 2.57 | -3.2956932 | 0.471 |
| PTP4A1 | ILMN_2154654 | 0.0306 | 0.00117 | 3.53 | -1.0125155 | 0.47 |
| CHSY1 | ILMN_1791576 | 0.0801 | 0.00579 | 2.94 | -2.4719529 | 0.47 |
| ARHGAP24 | ILMN_1801833 | 0.122 | 0.0123 | 2.64 | -3.1425996 | 0.47 |
| CECR1 | ILMN_1666174 | 0.139 | 0.0155 | 2.54 | -3.3528535 | 0.468 |
| DHRS12 | ILMN_1669177 | 0.257 | 0.0474 | 2.05 | -4.3157183 | 0.468 |
| S100A1 | ILMN_1653494 | 0.199 | 0.0294 | 2.27 | -3.9078141 | 0.466 |
| CSGALNACT1 | ILMN_2055523 | 0.0786 | 0.00557 | 2.95 | -2.4366195 | 0.465 |
| MRPL57 | ILMN_2203807 | 0.179 | 0.0243 | 2.35 | -3.7450857 | 0.465 |
| MIR345 | ILMN_3310386 | 0.205 | 0.031 | 2.25 | -3.9548448 | 0.464 |
| CPNE1 | ILMN_2276000 | 0.0747 | 0.00515 | 2.98 | -2.3670851 | 0.463 |
| SNX22 | ILMN_2080760 | 0.122 | 0.0122 | 2.64 | -3.1367056 | 0.462 |
| TLR8 | ILMN_1657892 | 0.165 | 0.0207 | 2.42 | -3.6061302 | 0.461 |
| ZBTB20 | ILMN_1878029 | 0.194 | 0.0279 | 2.29 | -3.8626421 | 0.461 |
| AVPR2 | ILMN_1698324 | 0.231 | 0.0387 | 2.15 | -4.1434902 | 0.461 |
| SLC37A3 | ILMN_1779979 | 0.199 | 0.0293 | 2.27 | -3.907073 | 0.46 |
| FNDC3A | ILMN_2362581 | 0.199 | 0.0295 | 2.27 | -3.9121244 | 0.46 |
| LOC100130476 | ILMN_3257004 | 0.243 | 0.0423 | 2.11 | -4.2197627 | 0.46 |
| CDK5R1 | ILMN_2062271 | 0.252 | 0.0453 | 2.07 | -4.2775154 | 0.46 |
| STK16 | ILMN_1742520 | 0.26 | 0.0485 | 2.04 | -4.3363538 | 0.46 |
| MKNK1 | ILMN_1725738 | 0.143 | 0.0161 | 2.53 | -3.3861649 | 0.458 |
| CSNK1D | ILMN_1720708 | 0.0342 | 0.00143 | 3.46 | -1.2000866 | 0.455 |
| GNA13 | ILMN_2176037 | 0.109 | 0.0101 | 2.72 | -2.9673972 | 0.455 |
| IFT20 | ILMN_3235216 | 0.112 | 0.0107 | 2.69 | -3.0224663 | 0.455 |
| PFDN2 | ILMN_1678754 | 0.222 | 0.0356 | 2.18 | -4.0734941 | 0.455 |
| RNF146 | ILMN_1685679 | 0.0131 | 0.000323 | 3.98 | 0.1733241 | 0.454 |
| SNORA73B | ILMN_3243677 | 0.177 | 0.0239 | 2.36 | -3.7294518 | 0.454 |
| RPS6KA3 | ILMN_1652736 | 0.0561 | 0.00323 | 3.16 | -1.9428818 | 0.453 |
| RHBDL2 | ILMN_2053538 | 0.147 | 0.0169 | 2.51 | -3.4278008 | 0.453 |
| SP3 | ILMN_1662168 | 0.117 | 0.0115 | 2.66 | -3.089376 | 0.452 |
| IGF2R | ILMN_1807662 | 0.00954 | 0.000192 | 4.16 | 0.6585574 | 0.451 |
| HPS1 | ILMN_2267787 | 0.0335 | 0.00137 | 3.47 | -1.1603758 | 0.451 |
| NDRG3 | ILMN_2385097 | 0.145 | 0.0167 | 2.51 | -3.4147562 | 0.451 |
| RNF213 | ILMN_1731203 | 0.199 | 0.0293 | 2.27 | -3.9049138 | 0.451 |
| NUP98 | ILMN_1684074 | 0.0317 | 0.00124 | 3.51 | -1.0678414 | 0.45 |
| STXBP5 | ILMN_1684402 | 0.121 | 0.0121 | 2.64 | -3.1319058 | 0.45 |
| FAM172A | ILMN_3241099 | 0.2 | 0.0297 | 2.26 | -3.9173204 | 0.45 |
| CPQ | ILMN_1713031 | 0.0103 | 0.000216 | 4.12 | 0.5484282 | 0.449 |
| MET | ILMN_1715175 | 0.14 | 0.0156 | 2.54 | -3.356333 | 0.449 |
| SPRR1A | ILMN_1716591 | 0.14 | 0.0157 | 2.54 | -3.3635863 | 0.449 |
| MIR877 | ILMN_3309544 | 0.258 | 0.0476 | 2.05 | -4.3205497 | 0.449 |
| RNLS | ILMN_1783873 | 0.258 | 0.0478 | 2.05 | -4.3234978 | 0.449 |
| HNRNPA3 | ILMN_1761083 | 0.0169 | 0.000467 | 3.85 | -0.1668655 | 0.448 |
| LMO4 | ILMN_2117171 | 0.0554 | 0.00317 | 3.16 | -1.9282223 | 0.448 |
| ASS1 | ILMN_2395451 | 0.109 | 0.0102 | 2.71 | -2.9753445 | 0.448 |
| LILRB2 | ILMN_1695744 | 0.219 | 0.0348 | 2.19 | -4.0533312 | 0.448 |
| GK | ILMN_2393296 | 0.11 | 0.0104 | 2.71 | -2.9965819 | 0.447 |
| ADD3 | ILMN_1787378 | 0.124 | 0.0127 | 2.63 | -3.1721543 | 0.447 |
| MMGT1 | ILMN_1776216 | 0.0468 | 0.00241 | 3.27 | -1.6781653 | 0.446 |
| SETDB2 | ILMN_1731644 | 0.222 | 0.0358 | 2.18 | -4.0771369 | 0.445 |
| DUSP16 | ILMN_1764361 | 0.134 | 0.0144 | 2.57 | -3.2868548 | 0.444 |
| CFI | ILMN_1727815 | 0.212 | 0.033 | 2.22 | -4.0074773 | 0.444 |
| HBA2 | ILMN_1667796 | 0.226 | 0.0372 | 2.16 | -4.1108782 | 0.444 |
| LY6G5C | ILMN_1763467 | 0.231 | 0.0387 | 2.15 | -4.1453021 | 0.444 |
| MIR1185-1 | ILMN_3309164 | 0.0767 | 0.00534 | 2.97 | -2.3997481 | 0.442 |
| MSL3 | ILMN_3232573 | 0.243 | 0.0423 | 2.11 | -4.2198131 | 0.442 |
| ZC3H11A | ILMN_1715574 | 0.122 | 0.0122 | 2.64 | -3.1356516 | 0.441 |
| MIR129-2 | ILMN_3308505 | 0.161 | 0.0198 | 2.44 | -3.5647937 | 0.441 |
| SNORA73B | ILMN_3240155 | 0.187 | 0.0262 | 2.32 | -3.8082526 | 0.441 |
| NUFIP2 | ILMN_1765829 | 0.0284 | 0.00103 | 3.57 | -0.8990992 | 0.44 |
| LOC101928714 | ILMN_1891324 | 0.124 | 0.0125 | 2.63 | -3.163101 | 0.44 |
| CNTNAP3 | ILMN_1717541 | 0.177 | 0.0239 | 2.36 | -3.7301984 | 0.44 |
| ASNS | ILMN_1796417 | 0.252 | 0.0454 | 2.07 | -4.2792694 | 0.44 |
| KIR3DL1 | ILMN_2131828 | 0.204 | 0.0307 | 2.25 | -3.9464828 | 0.438 |
| DLX4 | ILMN_1771113 | 0.0839 | 0.00629 | 2.9 | -2.5466801 | 0.437 |
| SLC19A2 | ILMN_2201668 | 0.191 | 0.0274 | 2.3 | -3.8464912 | 0.437 |
| LMAN1 | ILMN_1715814 | 0.139 | 0.0155 | 2.54 | -3.3516803 | 0.436 |
| PLXDC2 | ILMN_1861376 | 0.155 | 0.0186 | 2.47 | -3.5114905 | 0.436 |
| FRRS1 | ILMN_2214734 | 0.176 | 0.0235 | 2.37 | -3.7144018 | 0.436 |
| SNORA1 | ILMN_3245893 | 0.187 | 0.0263 | 2.32 | -3.8110616 | 0.436 |
| TNPO1 | ILMN_1786347 | 0.0414 | 0.00195 | 3.34 | -1.4826607 | 0.435 |
| NCEH1 | ILMN_2061446 | 0.0809 | 0.00592 | 2.93 | -2.4923931 | 0.435 |
| SOD1 | ILMN_1662438 | 0.056 | 0.00322 | 3.16 | -1.9399813 | 0.434 |
| TPTE2P6 | ILMN_2183424 | 0.238 | 0.0404 | 2.13 | -4.1808047 | 0.434 |
| JAGN1 | ILMN_3238036 | 0.12 | 0.0119 | 2.65 | -3.1202399 | 0.433 |
| DOCK5 | ILMN_1752455 | 0.129 | 0.0136 | 2.6 | -3.2319685 | 0.433 |
| PCSK5 | ILMN_1767934 | 0.211 | 0.0326 | 2.22 | -3.9967608 | 0.433 |
| TMEM267 | ILMN_2150465 | 0.0922 | 0.00748 | 2.84 | -2.7021108 | 0.432 |
| OR9A4 | ILMN_1739155 | 0.177 | 0.024 | 2.36 | -3.7319303 | 0.432 |
| FOXN3 | ILMN_1665304 | 0.209 | 0.0321 | 2.23 | -3.9840979 | 0.432 |
| LRBA | ILMN_1652160 | 0.137 | 0.015 | 2.55 | -3.3239213 | 0.43 |
| FAM49A | ILMN_1794038 | 0.054 | 0.00303 | 3.18 | -1.8872546 | 0.429 |
| ZFAND5 | ILMN_1795228 | 0.0809 | 0.00593 | 2.93 | -2.4945828 | 0.429 |
| KRT17 | ILMN_1666845 | 0.149 | 0.0173 | 2.5 | -3.4467062 | 0.429 |
| USP6 | ILMN_1721727 | 0.243 | 0.042 | 2.11 | -4.213542 | 0.429 |
| TCERG1 | ILMN_1706839 | 0.248 | 0.0445 | 2.08 | -4.2620737 | 0.429 |
| ST8SIA4 | ILMN_2394161 | 0.078 | 0.00549 | 2.96 | -2.4238486 | 0.428 |
| ANKRD40 | ILMN_1758846 | 0.153 | 0.0182 | 2.47 | -3.4937979 | 0.428 |
| ROM1 | ILMN_1723743 | 0.204 | 0.0308 | 2.25 | -3.9490072 | 0.428 |
| SNORA8 | ILMN_3238707 | 0.212 | 0.033 | 2.22 | -4.007536 | 0.428 |
| RFESD | ILMN_1802162 | 0.248 | 0.0443 | 2.09 | -4.258277 | 0.428 |
| SNORA10 | ILMN_2189842 | 0.225 | 0.0368 | 2.17 | -4.1009285 | 0.427 |
| TSPYL6 | ILMN_1701264 | 0.247 | 0.0438 | 2.09 | -4.2487145 | 0.427 |
| RPRD1A | ILMN_1764207 | 0.0788 | 0.00561 | 2.95 | -2.4438807 | 0.426 |
| PNPT1 | ILMN_3251723 | 0.126 | 0.013 | 2.61 | -3.1953137 | 0.426 |
| BLZF1 | ILMN_2106656 | 0.262 | 0.0492 | 2.04 | -4.3480785 | 0.426 |
| SPTLC1 | ILMN_1665094 | 0.101 | 0.00873 | 2.77 | -2.8410857 | 0.425 |
| ZNF786 | ILMN_1713706 | 0.0911 | 0.00733 | 2.84 | -2.6837777 | 0.424 |
| ROCK1 | ILMN_1808768 | 0.124 | 0.0127 | 2.62 | -3.1749836 | 0.424 |
| RIMKLB | ILMN_1681757 | 0.218 | 0.0344 | 2.2 | -4.0445186 | 0.422 |
| CDH26 | ILMN_1765255 | 0.252 | 0.0456 | 2.07 | -4.2827528 | 0.422 |
| RPS4Y2 | ILMN_2191331 | 0.0798 | 0.00575 | 2.94 | -2.4656241 | 0.421 |
| HACE1 | ILMN_1740217 | 0.135 | 0.0148 | 2.56 | -3.309833 | 0.421 |
| ACCS | ILMN_3187680 | 0.243 | 0.0421 | 2.11 | -4.2150908 | 0.421 |
| TIMM9 | ILMN_1653709 | 0.246 | 0.0434 | 2.09 | -4.241928 | 0.421 |
| MBP | ILMN_2331544 | 0.106 | 0.0096 | 2.74 | -2.9256641 | 0.42 |
| FAM126B | ILMN_1779486 | 0.139 | 0.0154 | 2.55 | -3.3441319 | 0.42 |
| PTGER4 | ILMN_1795930 | 0.104 | 0.00926 | 2.75 | -2.8932162 | 0.419 |
| SSTR2 | ILMN_2152257 | 0.139 | 0.0155 | 2.54 | -3.3478419 | 0.419 |
| ADGRE5 | ILMN_1673363 | 0.157 | 0.0191 | 2.46 | -3.5316992 | 0.419 |
| PKIG | ILMN_2406169 | 0.172 | 0.0225 | 2.38 | -3.677701 | 0.419 |
| IL21R | ILMN_1661687 | 0.254 | 0.0459 | 2.07 | -4.2895807 | 0.419 |
| FNDC3A | ILMN_1705111 | 0.26 | 0.0483 | 2.04 | -4.3323664 | 0.419 |
| CASP10 | ILMN_1797578 | 0.0996 | 0.00852 | 2.78 | -2.8187348 | 0.418 |
| FGL1 | ILMN_1803597 | 0.135 | 0.0147 | 2.56 | -3.302169 | 0.418 |
| SUV39H2 | ILMN_1789351 | 0.151 | 0.0179 | 2.48 | -3.4752196 | 0.418 |
| EIF2AK4 | ILMN_1755114 | 0.144 | 0.0163 | 2.52 | -3.3945893 | 0.417 |
| IDS | ILMN_1798448 | 0.165 | 0.0208 | 2.42 | -3.606627 | 0.417 |
| CCNL1 | ILMN_1683129 | 0.247 | 0.0438 | 2.09 | -4.2493544 | 0.417 |
| ROCK1 | ILMN_1739583 | 0.257 | 0.0473 | 2.05 | -4.3139102 | 0.417 |
| RAP1GDS1 | ILMN_1806266 | 0.231 | 0.0385 | 2.15 | -4.1392817 | 0.415 |
| PPT1 | ILMN_1669273 | 0.0462 | 0.00232 | 3.28 | -1.6440032 | 0.414 |
| LOC441455 | ILMN_3278170 | 0.111 | 0.0106 | 2.7 | -3.0107337 | 0.412 |
| IL17RD | ILMN_2407851 | 0.123 | 0.0125 | 2.63 | -3.159823 | 0.412 |
| ZNF695 | ILMN_1747943 | 0.202 | 0.0303 | 2.26 | -3.934868 | 0.412 |
| PIAS2 | ILMN_1694799 | 0.166 | 0.0211 | 2.41 | -3.6226905 | 0.411 |
| TRPM6 | ILMN_1814296 | 0.208 | 0.0319 | 2.23 | -3.9775246 | 0.411 |
| USP32 | ILMN_1804743 | 0.134 | 0.0145 | 2.57 | -3.2920182 | 0.41 |
| FAM83F | ILMN_1683231 | 0.225 | 0.0367 | 2.17 | -4.0999035 | 0.41 |
| DIAPH2 | ILMN_1744046 | 0.254 | 0.0461 | 2.07 | -4.2932911 | 0.41 |
| PPP2R5C | ILMN_1780913 | 0.162 | 0.0201 | 2.43 | -3.5782505 | 0.409 |
| GRAMD1B | ILMN_3237376 | 0.165 | 0.0208 | 2.42 | -3.6067428 | 0.409 |
| PPP1R12B | ILMN_2379375 | 0.172 | 0.0223 | 2.39 | -3.6689806 | 0.409 |
| EXTL3 | ILMN_1778226 | 0.0854 | 0.00653 | 2.89 | -2.5799744 | 0.408 |
| APP | ILMN_2404063 | 0.137 | 0.0151 | 2.55 | -3.3255389 | 0.408 |
| CSF2RA | ILMN_2376458 | 0.237 | 0.0402 | 2.13 | -4.1762603 | 0.408 |
| IQSEC1 | ILMN_1808299 | 0.108 | 0.0098 | 2.73 | -2.9436993 | 0.407 |
| LRRC37A | ILMN_1783673 | 0.108 | 0.00981 | 2.73 | -2.944448 | 0.407 |
| TOMM5 | ILMN_1808661 | 0.216 | 0.0341 | 2.2 | -4.0365599 | 0.407 |
| NSMAF | ILMN_1716596 | 0.229 | 0.0379 | 2.16 | -4.1271324 | 0.407 |
| TMEM38B | ILMN_1669940 | 0.0821 | 0.00609 | 2.92 | -2.5177231 | 0.406 |
| SNX6 | ILMN_1696294 | 0.102 | 0.00901 | 2.76 | -2.8685089 | 0.406 |
| OTOGL | ILMN_1866255 | 0.17 | 0.0219 | 2.4 | -3.6546612 | 0.405 |
| SIRPB1 | ILMN_1733997 | 0.0388 | 0.00173 | 3.39 | -1.3724392 | 0.404 |
| H3F3AP4 | ILMN_2402936 | 0.133 | 0.0142 | 2.58 | -3.2743215 | 0.404 |
| EMX2OS | ILMN_3241729 | 0.206 | 0.0313 | 2.24 | -3.961255 | 0.404 |
| ZNF200 | ILMN_1761965 | 0.216 | 0.0339 | 2.21 | -4.0319433 | 0.404 |
| C3orf58 | ILMN_1797372 | 0.037 | 0.0016 | 3.42 | -1.3017119 | 0.403 |
| CCT6P1 | ILMN_2050617 | 0.167 | 0.0212 | 2.41 | -3.6266018 | 0.403 |
| PCBP2 | ILMN_3249963 | 0.243 | 0.0421 | 2.11 | -4.2162071 | 0.402 |
| HNRNPU | ILMN_2370135 | 0.199 | 0.0295 | 2.27 | -3.9101927 | 0.401 |
| CD302 | ILMN_2070940 | 0.248 | 0.0445 | 2.08 | -4.261954 | 0.401 |
| PRR13 | ILMN_1795944 | 0.0925 | 0.0076 | 2.83 | -2.7161472 | 0.4 |
| RPL32 | ILMN_1782167 | 0.174 | 0.023 | 2.38 | -3.6965538 | 0.4 |
| CLRN1 | ILMN_1698745 | 0.244 | 0.0424 | 2.1 | -4.2228068 | 0.4 |
| ERCC1 | ILMN_2277676 | 0.149 | 0.0176 | 2.49 | -3.4600129 | 0.399 |
| CASP3 | ILMN_1731639 | 0.173 | 0.0228 | 2.38 | -3.6891943 | 0.396 |
| LCP2 | ILMN_1658962 | 0.0801 | 0.00579 | 2.94 | -2.4722956 | 0.395 |
| ARL6IP4 | ILMN_1791149 | 0.191 | 0.0273 | 2.3 | -3.8460865 | 0.394 |
| CHST1 | ILMN_1677719 | 0.0924 | 0.00751 | 2.83 | -2.7059095 | 0.393 |
| RPRD1A | ILMN_2209180 | 0.129 | 0.0135 | 2.6 | -3.2258375 | 0.393 |
| DUSP15 | ILMN_2382471 | 0.204 | 0.0307 | 2.25 | -3.947249 | 0.393 |
| TXN | ILMN_2038776 | 0.234 | 0.0395 | 2.14 | -4.161174 | 0.393 |
| SNORA25 | ILMN_1682038 | 0.187 | 0.0263 | 2.32 | -3.8131127 | 0.392 |
| SAMD15 | ILMN_3243606 | 0.228 | 0.0376 | 2.16 | -4.1188602 | 0.392 |
| APH1B | ILMN_1767816 | 0.0627 | 0.00387 | 3.09 | -2.1084237 | 0.391 |
| DNAH6 | ILMN_1884041 | 0.182 | 0.025 | 2.34 | -3.7700766 | 0.391 |
| AUH | ILMN_2231911 | 0.231 | 0.0385 | 2.15 | -4.1389585 | 0.391 |
| ATXN1 | ILMN_2153332 | 0.0675 | 0.00439 | 3.04 | -2.2230877 | 0.39 |
| DENR | ILMN_2168952 | 0.209 | 0.0319 | 2.23 | -3.9791825 | 0.39 |
| SRGAP2 | ILMN_1759549 | 0.24 | 0.0412 | 2.12 | -4.1967271 | 0.39 |
| YBX3 | ILMN_1782788 | 0.146 | 0.0167 | 2.51 | -3.4174833 | 0.389 |
| ZCCHC11 | ILMN_2373099 | 0.158 | 0.0191 | 2.45 | -3.5353631 | 0.389 |
| LSMEM1 | ILMN_1804895 | 0.182 | 0.0251 | 2.34 | -3.7717206 | 0.389 |
| PHYH | ILMN_2282352 | 0.0818 | 0.00603 | 2.92 | -2.5095084 | 0.388 |
| EXT2 | ILMN_2328029 | 0.135 | 0.0148 | 2.56 | -3.3069582 | 0.386 |
| PARVB | ILMN_1787919 | 0.147 | 0.0169 | 2.51 | -3.4282731 | 0.386 |
| CPQ | ILMN_2058795 | 0.00668 | 0.000109 | 4.35 | 1.1831313 | 0.385 |
| XIST | ILMN_1764573 | 0.129 | 0.0135 | 2.6 | -3.2280114 | 0.385 |
| TFPI | ILMN_1707124 | 0.21 | 0.0323 | 2.23 | -3.9892444 | 0.385 |
| RRN3P2 | ILMN_3246274 | 0.22 | 0.035 | 2.19 | -4.0591365 | 0.385 |
| HACD4 | ILMN_1690114 | 0.2 | 0.0297 | 2.27 | -3.9167056 | 0.384 |
| MED1 | ILMN_3248122 | 0.127 | 0.0131 | 2.61 | -3.2000872 | 0.383 |
| APPL1 | ILMN_1763730 | 0.135 | 0.0147 | 2.56 | -3.304727 | 0.383 |
| NTAN1 | ILMN_1815552 | 0.2 | 0.0296 | 2.27 | -3.9153548 | 0.383 |
| MSMO1 | ILMN_2402499 | 0.212 | 0.0329 | 2.22 | -4.0058682 | 0.383 |
| ELMO2 | ILMN_1778168 | 0.223 | 0.036 | 2.18 | -4.0827895 | 0.383 |
| GJA5 | ILMN_1722753 | 0.145 | 0.0167 | 2.51 | -3.4152343 | 0.382 |
| COL4A1 | ILMN_1653028 | 0.246 | 0.0435 | 2.09 | -4.2427836 | 0.381 |
| TLR6 | ILMN_1749287 | 0.0407 | 0.00188 | 3.36 | -1.4513923 | 0.38 |
| MMD | ILMN_1733937 | 0.109 | 0.0102 | 2.71 | -2.9753166 | 0.38 |
| VCL | ILMN_1795429 | 0.147 | 0.0169 | 2.51 | -3.4285718 | 0.38 |
| HNRNPA2B1 | ILMN_2369682 | 0.00928 | 0.000182 | 4.18 | 0.7080652 | 0.379 |
| ZMAT3 | ILMN_1654262 | 0.00544 | 7.67E-05 | 4.47 | 1.5137435 | 0.378 |
| TMEM234 | ILMN_1673752 | 0.225 | 0.0366 | 2.17 | -4.0968182 | 0.378 |
| DUSP1 | ILMN_1781285 | 0.0113 | 0.000251 | 4.07 | 0.4092509 | 0.377 |
| CALN1 | ILMN_1778121 | 0.245 | 0.043 | 2.1 | -4.2334269 | 0.377 |
| ARL6IP1 | ILMN_1708416 | 0.0847 | 0.00641 | 2.9 | -2.5633979 | 0.376 |
| SLC4A5 | ILMN_2273224 | 0.168 | 0.0214 | 2.41 | -3.6347184 | 0.376 |
| GNA13 | ILMN_1758906 | 0.0446 | 0.00217 | 3.3 | -1.5822336 | 0.375 |
| ERGIC1 | ILMN_1664068 | 0.0719 | 0.00482 | 3.01 | -2.3066816 | 0.375 |
| WBP1L | ILMN_1658830 | 0.0925 | 0.00758 | 2.83 | -2.7145324 | 0.375 |
| ANO10 | ILMN_1767111 | 0.124 | 0.0127 | 2.62 | -3.1748257 | 0.375 |
| AGFG1 | ILMN_1792497 | 0.15 | 0.0177 | 2.49 | -3.465498 | 0.375 |
| RIN3 | ILMN_1731736 | 0.165 | 0.0207 | 2.42 | -3.6047446 | 0.374 |
| HNRNPK | ILMN_3260017 | 0.173 | 0.0229 | 2.38 | -3.6911345 | 0.374 |
| ELF1 | ILMN_1664010 | 0.00428 | 5.46E-05 | 4.58 | 1.8314272 | 0.372 |
| RNY1 | ILMN_3237623 | 0.149 | 0.0174 | 2.49 | -3.453871 | 0.372 |
| MPP1 | ILMN_1733675 | 0.00938 | 0.000188 | 4.17 | 0.6796172 | 0.371 |
| GSPT1 | ILMN_1750130 | 0.0136 | 0.000341 | 3.96 | 0.1233607 | 0.37 |
| TGIF1 | ILMN_2318643 | 0.227 | 0.0375 | 2.16 | -4.1166365 | 0.37 |
| FPR1 | ILMN_2092118 | 0.0358 | 0.00153 | 3.43 | -1.2596292 | 0.369 |
| PHLDB3 | ILMN_3307944 | 0.232 | 0.0389 | 2.14 | -4.1478757 | 0.369 |
| ACOT9 | ILMN_2367070 | 0.243 | 0.0421 | 2.11 | -4.2157889 | 0.369 |
| ADAT3 | ILMN_1806275 | 0.258 | 0.0478 | 2.05 | -4.3228132 | 0.369 |
| HIST1H3H | ILMN_1749368 | 0.03 | 0.00113 | 3.54 | -0.9807031 | 0.368 |
| ALPP | ILMN_1693789 | 0.158 | 0.0192 | 2.45 | -3.5398709 | 0.368 |
| SMCHD1 | ILMN_1808148 | 0.255 | 0.0464 | 2.06 | -4.2984379 | 0.368 |
| YIPF4 | ILMN_1782444 | 0.123 | 0.0123 | 2.64 | -3.1491997 | 0.367 |
| CMTM3 | ILMN_1667840 | 0.241 | 0.0416 | 2.11 | -4.2059363 | 0.366 |
| CLDN14 | ILMN_1661194 | 0.0437 | 0.00211 | 3.32 | -1.5544859 | 0.365 |
| TSPAN9 | ILMN_1729453 | 0.0617 | 0.00377 | 3.1 | -2.0843648 | 0.364 |
| TXNDC5 | ILMN_1788108 | 0.237 | 0.0402 | 2.13 | -4.1766427 | 0.363 |
| PDE4B | ILMN_2340259 | 0.0407 | 0.00188 | 3.36 | -1.451183 | 0.362 |
| ITGAM | ILMN_1685009 | 0.174 | 0.0231 | 2.37 | -3.698806 | 0.362 |
| OIP5-AS1 | ILMN_3297455 | 0.235 | 0.0397 | 2.14 | -4.1658142 | 0.362 |
| MNDA | ILMN_1738992 | 0.0528 | 0.00293 | 3.19 | -1.8550595 | 0.361 |
| NFAT5 | ILMN_1780291 | 0.192 | 0.0275 | 2.3 | -3.8504829 | 0.361 |
| TRIM9 | ILMN_1763433 | 0.262 | 0.0492 | 2.04 | -4.3471615 | 0.361 |
| TWF1 | ILMN_1681203 | 0.263 | 0.0495 | 2.03 | -4.3519486 | 0.36 |
| RAB27A | ILMN_1699878 | 0.0742 | 0.00507 | 2.99 | -2.3532993 | 0.359 |
| ZNF773 | ILMN_1685365 | 0.235 | 0.0397 | 2.14 | -4.1660103 | 0.359 |
| SSH2 | ILMN_1672834 | 0.0983 | 0.00835 | 2.79 | -2.8005854 | 0.358 |
| TMED10P1 | ILMN_2213558 | 0.174 | 0.0232 | 2.37 | -3.701686 | 0.358 |
| HPS1 | ILMN_1741483 | 0.181 | 0.0247 | 2.35 | -3.757015 | 0.358 |
| MBP | ILMN_2398939 | 0.18 | 0.0244 | 2.35 | -3.7478704 | 0.357 |
| MCHR2 | ILMN_2383150 | 0.256 | 0.047 | 2.06 | -4.308811 | 0.357 |
| MTRF1L | ILMN_1786684 | 0.0762 | 0.00527 | 2.97 | -2.3883277 | 0.356 |
| SOS2 | ILMN_1764414 | 0.0251 | 0.000858 | 3.64 | -0.7297861 | 0.355 |
| UBE2H | ILMN_1674633 | 0.107 | 0.00976 | 2.73 | -2.9406839 | 0.355 |
| TCP1 | ILMN_1660661 | 0.16 | 0.0196 | 2.44 | -3.5579407 | 0.355 |
| TFPI | ILMN_1662619 | 0.188 | 0.0265 | 2.31 | -3.818456 | 0.355 |
| ARSA | ILMN_2047240 | 0.24 | 0.0411 | 2.12 | -4.1958539 | 0.355 |
| NAA15 | ILMN_1669142 | 0.144 | 0.0164 | 2.52 | -3.4020182 | 0.354 |
| SUMF1 | ILMN_1753472 | 0.169 | 0.0217 | 2.4 | -3.6449451 | 0.354 |
| PPP1CB | ILMN_2405023 | 0.175 | 0.0234 | 2.37 | -3.7093425 | 0.353 |
| SH2D2A | ILMN_1733874 | 0.226 | 0.0371 | 2.17 | -4.1092896 | 0.353 |
| TCEAL9 | ILMN_2340935 | 0.184 | 0.0254 | 2.33 | -3.7835045 | 0.352 |
| NEO1 | ILMN_1696702 | 0.203 | 0.0306 | 2.25 | -3.9419294 | 0.351 |
| JMJD1C | ILMN_1764970 | 0.146 | 0.0167 | 2.51 | -3.417364 | 0.349 |
| IFT88 | ILMN_2373755 | 0.151 | 0.0178 | 2.48 | -3.4728305 | 0.349 |
| SNCA | ILMN_1701933 | 0.161 | 0.0199 | 2.44 | -3.5681983 | 0.349 |
| HEBP2 | ILMN_1755077 | 0.109 | 0.0101 | 2.72 | -2.9670283 | 0.348 |
| DDX19B | ILMN_2330584 | 0.208 | 0.0317 | 2.24 | -3.9738359 | 0.347 |
| MAN2A2 | ILMN_1815148 | 0.264 | 0.0497 | 2.03 | -4.3562669 | 0.347 |
| AGTPBP1 | ILMN_1718071 | 0.165 | 0.0209 | 2.42 | -3.6131086 | 0.346 |
| ODF3B | ILMN_2209614 | 0.258 | 0.0477 | 2.05 | -4.3217909 | 0.344 |
| LATS2 | ILMN_1703412 | 0.124 | 0.0125 | 2.63 | -3.163194 | 0.341 |
| CLEC2B | ILMN_1784608 | 0.207 | 0.0314 | 2.24 | -3.96662 | 0.341 |
| ADD3 | ILMN_1814526 | 0.237 | 0.0403 | 2.13 | -4.1796038 | 0.341 |
| SLC6A6 | ILMN_1673586 | 0.172 | 0.0226 | 2.38 | -3.6792103 | 0.34 |
| FIBCD1 | ILMN_1727135 | 0.0862 | 0.00662 | 2.88 | -2.5930255 | 0.339 |
| N4BP2 | ILMN_2222101 | 0.11 | 0.0103 | 2.71 | -2.9925611 | 0.338 |
| MCL1 | ILMN_1803988 | 0.196 | 0.0285 | 2.28 | -3.882881 | 0.338 |
| TRIM66 | ILMN_3235584 | 0.231 | 0.0386 | 2.15 | -4.1421647 | 0.338 |
| ZNF138 | ILMN_1676240 | 0.158 | 0.0192 | 2.45 | -3.5381102 | 0.337 |
| UTS2 | ILMN_2236625 | 0.213 | 0.0332 | 2.22 | -4.0138842 | 0.337 |
| ITGAX | ILMN_1726175 | 0.185 | 0.0256 | 2.33 | -3.7893744 | 0.336 |
| 5-Mar | ILMN_1702764 | 0.243 | 0.0424 | 2.11 | -4.2216173 | 0.336 |
| ACOT9 | ILMN_1658995 | 0.0605 | 0.00362 | 3.11 | -2.0484578 | 0.335 |
| KIAA0922 | ILMN_1668469 | 0.254 | 0.0459 | 2.07 | -4.2894567 | 0.335 |
| INTS6 | ILMN_1655557 | 0.131 | 0.0139 | 2.59 | -3.2525305 | 0.334 |
| DCTN4 | ILMN_1741564 | 0.0925 | 0.00759 | 2.83 | -2.7149613 | 0.333 |
| PHC3 | ILMN_2179873 | 0.169 | 0.0218 | 2.4 | -3.6491888 | 0.333 |
| NFAT5 | ILMN_1797594 | 0.213 | 0.0332 | 2.22 | -4.0141757 | 0.333 |
| RPS2 | ILMN_2218277 | 0.231 | 0.0386 | 2.15 | -4.1420456 | 0.333 |
| MUC4 | ILMN_1792039 | 0.26 | 0.0483 | 2.05 | -4.331533 | 0.333 |
| IL1RAP | ILMN_1686884 | 0.0747 | 0.00514 | 2.98 | -2.3642313 | 0.332 |
| PARP8 | ILMN_1806651 | 0.19 | 0.027 | 2.31 | -3.8337691 | 0.332 |
| ING1 | ILMN_1655537 | 0.239 | 0.0408 | 2.12 | -4.1895233 | 0.332 |
| THADA | ILMN_1706818 | 0.112 | 0.0106 | 2.7 | -3.0143291 | 0.331 |
| SNORA33 | ILMN_2096747 | 0.256 | 0.047 | 2.06 | -4.3091312 | 0.331 |
| GOLGA6L6 | ILMN_3243175 | 0.226 | 0.0371 | 2.17 | -4.1088585 | 0.33 |
| ATP11B | ILMN_1658884 | 0.205 | 0.0311 | 2.24 | -3.9570042 | 0.329 |
| FBXL5 | ILMN_2378100 | 0.201 | 0.03 | 2.26 | -3.9249949 | 0.327 |
| ADIPOR1 | ILMN_2096322 | 0.00904 | 0.000174 | 4.19 | 0.7508615 | 0.326 |
| ARID4B | ILMN_1761334 | 0.198 | 0.0291 | 2.27 | -3.8992125 | 0.326 |
| AGO2 | ILMN_1695719 | 0.0638 | 0.00399 | 3.08 | -2.134778 | 0.325 |
| CSNK1A1 | ILMN_2364174 | 0.0739 | 0.00501 | 2.99 | -2.3424412 | 0.325 |
| SEMA4D | ILMN_1687533 | 0.0807 | 0.0059 | 2.93 | -2.4898481 | 0.324 |
| KIF5B | ILMN_1788160 | 0.12 | 0.0119 | 2.65 | -3.1129038 | 0.323 |
| MRPS18B | ILMN_1721337 | 0.2 | 0.0296 | 2.27 | -3.9136135 | 0.323 |
| LNPEP | ILMN_1758661 | 0.0663 | 0.00428 | 3.05 | -2.2004614 | 0.322 |
| CD37 | ILMN_2375825 | 0.2 | 0.0295 | 2.27 | -3.9128184 | 0.322 |
| PCM1 | ILMN_1690487 | 0.224 | 0.0363 | 2.18 | -4.0890564 | 0.322 |
| NDRG1 | ILMN_1809931 | 0.213 | 0.0332 | 2.22 | -4.0126251 | 0.321 |
| MEFV | ILMN_1804738 | 0.225 | 0.0368 | 2.17 | -4.1015415 | 0.321 |
| CBL | ILMN_1716080 | 0.2 | 0.0297 | 2.27 | -3.9163779 | 0.32 |
| PTCHD4 | ILMN_1742467 | 0.223 | 0.036 | 2.18 | -4.081457 | 0.319 |
| FOXN2 | ILMN_1736510 | 0.0465 | 0.00238 | 3.27 | -1.664808 | 0.317 |
| PHF3 | ILMN_2057826 | 0.24 | 0.0412 | 2.12 | -4.1978021 | 0.317 |
| TSTD1 | ILMN_3197097 | 0.248 | 0.0443 | 2.08 | -4.2598215 | 0.316 |
| ICMT | ILMN_1723021 | 0.123 | 0.0125 | 2.63 | -3.1573576 | 0.315 |
| EGLN1 | ILMN_1749892 | 0.219 | 0.0348 | 2.19 | -4.0545027 | 0.314 |
| CCDC121 | ILMN_2183772 | 0.158 | 0.0193 | 2.45 | -3.5415155 | 0.313 |
| TDP2 | ILMN_1785821 | 0.196 | 0.0286 | 2.28 | -3.8837902 | 0.313 |
| KIAA1551 | ILMN_2229922 | 0.0229 | 0.000739 | 3.69 | -0.5922561 | 0.312 |
| EDARADD | ILMN_1761820 | 0.14 | 0.0158 | 2.54 | -3.3652535 | 0.311 |
| VAPA | ILMN_1779410 | 0.177 | 0.0239 | 2.36 | -3.7291103 | 0.311 |
| PTP4A1 | ILMN_1760575 | 0.0702 | 0.00466 | 3.02 | -2.276074 | 0.309 |
| SYPL1 | ILMN_1691458 | 0.123 | 0.0125 | 2.63 | -3.1576939 | 0.309 |
| RIT1 | ILMN_1656335 | 0.165 | 0.0209 | 2.42 | -3.6128385 | 0.309 |
| NFYA | ILMN_1690325 | 0.139 | 0.0154 | 2.55 | -3.3420531 | 0.308 |
| RNF219 | ILMN_2070210 | 0.196 | 0.0287 | 2.28 | -3.8871875 | 0.308 |
| CYSTM1 | ILMN_1761566 | 0.242 | 0.0418 | 2.11 | -4.2089369 | 0.307 |
| MTA1 | ILMN_1671911 | 0.127 | 0.0131 | 2.61 | -3.2032186 | 0.306 |
| GDE1 | ILMN_1728698 | 0.0423 | 0.00201 | 3.33 | -1.5098001 | 0.305 |
| RAD51C | ILMN_1760635 | 0.253 | 0.0457 | 2.07 | -4.2861338 | 0.302 |
| ZNF738 | ILMN_3225102 | 0.195 | 0.0281 | 2.29 | -3.8702759 | 0.301 |
| FBXO7 | ILMN_1667112 | 0.202 | 0.0304 | 2.25 | -3.9370587 | 0.3 |
| ZNF320 | ILMN_2103480 | 0.237 | 0.04 | 2.13 | -4.1728061 | 0.3 |
| TXLNG | ILMN_1882000 | 0.245 | 0.0431 | 2.1 | -4.2367728 | 0.3 |
| CTBP2 | ILMN_1691294 | 0.255 | 0.0465 | 2.06 | -4.2992474 | 0.3 |
| SON | ILMN_2323302 | 0.151 | 0.0179 | 2.48 | -3.4776919 | 0.297 |
| DTNBP1 | ILMN_1682960 | 0.239 | 0.0409 | 2.12 | -4.1914168 | 0.297 |
| CASC4 | ILMN_2325574 | 0.242 | 0.0418 | 2.11 | -4.2104488 | 0.297 |
| RPL26L1 | ILMN_2110532 | 0.0409 | 0.0019 | 3.35 | -1.46197 | 0.295 |
| ZDBF2 | ILMN_1740711 | 0.139 | 0.0156 | 2.54 | -3.3536313 | 0.295 |
| SVIL | ILMN_1690754 | 0.229 | 0.0379 | 2.16 | -4.1269882 | 0.295 |
| DCUN1D1 | ILMN_1810719 | 0.211 | 0.0326 | 2.22 | -3.998095 | 0.294 |
| S100PBP | ILMN_2294274 | 0.213 | 0.0334 | 2.21 | -4.017827 | 0.294 |
| PSEN1 | ILMN_1796669 | 0.107 | 0.00973 | 2.73 | -2.9373471 | 0.292 |
| GRK5 | ILMN_1728830 | 0.162 | 0.0202 | 2.43 | -3.5807593 | 0.292 |
| UBR5 | ILMN_1741253 | 0.17 | 0.0219 | 2.4 | -3.6524255 | 0.292 |
| ENO1-AS1 | ILMN_1896149 | 0.244 | 0.0427 | 2.1 | -4.2279901 | 0.292 |
| MAGEL2 | ILMN_1657478 | 0.247 | 0.0438 | 2.09 | -4.2487912 | 0.291 |
| CRK | ILMN_1803302 | 0.262 | 0.0492 | 2.04 | -4.3483686 | 0.291 |
| PREX1 | ILMN_1777342 | 0.0834 | 0.00623 | 2.91 | -2.5384781 | 0.29 |
| MAGT1 | ILMN_1721349 | 0.203 | 0.0305 | 2.25 | -3.9389565 | 0.29 |
| SLC5A12 | ILMN_3307836 | 0.248 | 0.0444 | 2.08 | -4.2618373 | 0.29 |
| SNORD36A | ILMN_2135175 | 0.255 | 0.0464 | 2.06 | -4.2989483 | 0.29 |
| PIK3CA | ILMN_1705468 | 0.263 | 0.0495 | 2.03 | -4.3525832 | 0.29 |
| IKBIP | ILMN_1701402 | 0.251 | 0.0451 | 2.08 | -4.2747398 | 0.289 |
| MBD2 | ILMN_1750395 | 0.202 | 0.0303 | 2.26 | -3.9334527 | 0.288 |
| CAPS2 | ILMN_3307742 | 0.225 | 0.0368 | 2.17 | -4.1007475 | 0.288 |
| SKAP2 | ILMN_2125010 | 0.244 | 0.0424 | 2.1 | -4.2224449 | 0.288 |
| UBE2B | ILMN_1663099 | 0.124 | 0.0127 | 2.62 | -3.1729308 | 0.287 |
| STX3 | ILMN_1659544 | 0.252 | 0.0454 | 2.07 | -4.2802301 | 0.287 |
| RB1CC1 | ILMN_1736796 | 0.0508 | 0.00274 | 3.22 | -1.795701 | 0.285 |
| KDM6A | ILMN_1654488 | 0.0724 | 0.00488 | 3 | -2.3172937 | 0.285 |
| IQGAP1 | ILMN_1803819 | 0.0916 | 0.00741 | 2.84 | -2.6936717 | 0.284 |
| ZFP36 | ILMN_1720829 | 0.0768 | 0.00535 | 2.97 | -2.4018911 | 0.282 |
| PTPN12 | ILMN_1695509 | 0.0834 | 0.00622 | 2.91 | -2.5361408 | 0.28 |
| RBM3 | ILMN_1698213 | 0.171 | 0.0222 | 2.39 | -3.6636786 | 0.28 |
| HCFC1R1 | ILMN_1757877 | 0.21 | 0.0322 | 2.23 | -3.9863191 | 0.28 |
| CD37 | ILMN_1786176 | 0.226 | 0.037 | 2.17 | -4.1068661 | 0.279 |
| SLC22A4 | ILMN_1685057 | 0.121 | 0.012 | 2.65 | -3.1243683 | 0.278 |
| PDCD4 | ILMN_1768004 | 0.186 | 0.0261 | 2.32 | -3.8057786 | 0.277 |
| ZNF217 | ILMN_1755303 | 0.226 | 0.0371 | 2.17 | -4.1075833 | 0.276 |
| ASAH1 | ILMN_1684054 | 0.25 | 0.0449 | 2.08 | -4.2713191 | 0.276 |
| IFNGR1 | ILMN_1675939 | 0.108 | 0.00997 | 2.72 | -2.9589748 | 0.274 |
| SYPL1 | ILMN_1764087 | 0.0439 | 0.00213 | 3.31 | -1.5632401 | 0.273 |
| ANXA11 | ILMN_2380494 | 0.117 | 0.0115 | 2.66 | -3.0862514 | 0.271 |
| CSMD1 | ILMN_1746945 | 0.135 | 0.0147 | 2.56 | -3.305131 | 0.271 |
| ACSL4 | ILMN_2285506 | 0.159 | 0.0194 | 2.45 | -3.5483743 | 0.271 |
| TFDP1 | ILMN_2117987 | 0.188 | 0.0266 | 2.31 | -3.822049 | 0.271 |
| GNS | ILMN_1744517 | 0.0615 | 0.00375 | 3.1 | -2.079927 | 0.27 |
| HM13 | ILMN_1807833 | 0.245 | 0.0428 | 2.1 | -4.2307966 | 0.269 |
| ITPRIP | ILMN_1805192 | 0.257 | 0.0474 | 2.05 | -4.3158022 | 0.269 |
| GNG5 | ILMN_1701854 | 0.259 | 0.048 | 2.05 | -4.3273598 | 0.269 |
| LAPTM4A | ILMN_1745110 | 0.176 | 0.0235 | 2.37 | -3.7143794 | 0.268 |
| LRRC37B | ILMN_1777895 | 0.185 | 0.0255 | 2.33 | -3.7859827 | 0.268 |
| NRBF2 | ILMN_3237385 | 0.205 | 0.0312 | 2.24 | -3.9590282 | 0.268 |
| MARK2 | ILMN_1736747 | 0.215 | 0.0337 | 2.21 | -4.0255939 | 0.268 |
| PLAA | ILMN_1810387 | 0.15 | 0.0176 | 2.49 | -3.4626167 | 0.267 |
| STAT5B | ILMN_1684034 | 0.209 | 0.0319 | 2.23 | -3.9791702 | 0.266 |
| IL10 | ILMN_2073307 | 0.152 | 0.018 | 2.48 | -3.4828014 | 0.265 |
| PPA2 | ILMN_1687785 | 0.181 | 0.0248 | 2.34 | -3.7598056 | 0.264 |
| LOC107133515 | ILMN_3245600 | 0.246 | 0.0436 | 2.09 | -4.2456152 | 0.264 |
| FOXP1 | ILMN_1738179 | 0.26 | 0.0486 | 2.04 | -4.3374474 | 0.262 |
| METTL22 | ILMN_1658290 | 0.186 | 0.0259 | 2.33 | -3.7982511 | 0.261 |
| GNG11 | ILMN_1782419 | 0.103 | 0.0091 | 2.76 | -2.8780085 | 0.26 |
| NDEL1 | ILMN_1705064 | 0.0145 | 0.000377 | 3.93 | 0.0309592 | 0.259 |
| RERG-AS1 | ILMN_1897279 | 0.198 | 0.0292 | 2.27 | -3.9023176 | 0.258 |
| CHD7 | ILMN_1677376 | 0.126 | 0.013 | 2.61 | -3.1959512 | 0.257 |
| MAX | ILMN_1802457 | 0.243 | 0.042 | 2.11 | -4.2145564 | 0.257 |
| DIP2B | ILMN_1755589 | 0.247 | 0.044 | 2.09 | -4.2538155 | 0.257 |
| CCDC96 | ILMN_1799710 | 0.26 | 0.0485 | 2.04 | -4.3357498 | 0.257 |
| PIP4K2A | ILMN_3236637 | 0.162 | 0.0201 | 2.43 | -3.5765209 | 0.252 |
| EVI2B | ILMN_1763452 | 0.223 | 0.0359 | 2.18 | -4.0812901 | 0.251 |
| SBF2 | ILMN_2123665 | 0.172 | 0.0224 | 2.39 | -3.6731607 | 0.249 |
| ZNF586 | ILMN_1666727 | 0.213 | 0.0335 | 2.21 | -4.0199825 | 0.249 |
| MAP3K5 | ILMN_1726547 | 0.151 | 0.0178 | 2.48 | -3.4725377 | 0.248 |
| CHD1 | ILMN_1654583 | 0.127 | 0.0131 | 2.61 | -3.2036073 | 0.246 |
| NCS1 | ILMN_1756784 | 0.248 | 0.0443 | 2.09 | -4.2589143 | 0.244 |
| NFKBIA | ILMN_1773154 | 0.16 | 0.0197 | 2.44 | -3.5598056 | 0.243 |
| GSTT2B | ILMN_1784267 | 0.21 | 0.0323 | 2.23 | -3.9882731 | 0.243 |
| ANKRD9 | ILMN_1675937 | 0.174 | 0.0232 | 2.37 | -3.7032457 | 0.242 |
| PLD4 | ILMN_1695490 | 0.195 | 0.0282 | 2.29 | -3.8724599 | 0.241 |
| DHRSX | ILMN_1704139 | 0.261 | 0.0487 | 2.04 | -4.3398496 | 0.24 |
| SUMO1P3 | ILMN_1732080 | 0.103 | 0.00911 | 2.76 | -2.8788237 | 0.236 |
| PACSIN2 | ILMN_1702396 | 0.186 | 0.0261 | 2.32 | -3.8061913 | 0.236 |
| WDFY2 | ILMN_1802292 | 0.26 | 0.0485 | 2.04 | -4.3359967 | 0.236 |
| PHIP | ILMN_1898682 | 0.24 | 0.0412 | 2.12 | -4.1978552 | 0.233 |
| CASP10 | ILMN_2321720 | 0.211 | 0.0325 | 2.23 | -3.9946817 | 0.231 |
| SCIMP | ILMN_3307757 | 0.181 | 0.0247 | 2.35 | -3.7574028 | 0.229 |
| CARMIL3 | ILMN_3244860 | 0.264 | 0.0498 | 2.03 | -4.3580634 | 0.228 |
| BCAP31 | ILMN_1812403 | 0.196 | 0.0287 | 2.28 | -3.8864144 | 0.224 |
| BASP1 | ILMN_1651826 | 0.0716 | 0.00479 | 3.01 | -2.3003707 | 0.223 |
| LNPEP | ILMN_1814737 | 0.195 | 0.0283 | 2.29 | -3.8745908 | 0.222 |
| EEF1D | ILMN_1782543 | 0.242 | 0.0419 | 2.11 | -4.2110192 | 0.22 |
| FAAP20 | ILMN_2097790 | 0.194 | 0.0281 | 2.29 | -3.8680536 | 0.218 |
| ADIPOR1 | ILMN_1688322 | 0.0925 | 0.00754 | 2.83 | -2.7100858 | 0.217 |
| RABL2B | ILMN_1810058 | 0.105 | 0.00942 | 2.74 | -2.9088701 | 0.216 |
| AKAP12 | ILMN_1684836 | 0.246 | 0.0435 | 2.09 | -4.2445895 | 0.215 |
| PHIP | ILMN_1788689 | 0.123 | 0.0124 | 2.63 | -3.1548594 | 0.213 |
| CSNK1A1 | ILMN_1785988 | 0.185 | 0.0256 | 2.33 | -3.7875359 | 0.213 |
| LEMD3 | ILMN_1727361 | 0.199 | 0.0295 | 2.27 | -3.9103672 | 0.212 |
| ESD | ILMN_1720285 | 0.218 | 0.0344 | 2.2 | -4.0441901 | 0.211 |
| WWP2 | ILMN_1668847 | 0.239 | 0.0408 | 2.12 | -4.1891802 | 0.21 |
| RPS9 | ILMN_2038772 | 0.247 | 0.0439 | 2.09 | -4.2515276 | 0.208 |
| ABI1 | ILMN_1739081 | 0.198 | 0.0291 | 2.27 | -3.9002122 | 0.207 |
| FBXO34 | ILMN_1765060 | 0.232 | 0.0388 | 2.15 | -4.146355 | 0.207 |
| TREM1 | ILMN_1688231 | 0.246 | 0.0435 | 2.09 | -4.2438752 | 0.205 |
| DCP1B | ILMN_1743992 | 0.241 | 0.0415 | 2.11 | -4.2042618 | 0.202 |
| NAAA | ILMN_2285568 | 0.255 | 0.0464 | 2.06 | -4.2988716 | 0.202 |
| TFDP2 | ILMN_1793671 | 0.256 | 0.0467 | 2.06 | -4.3039838 | 0.196 |
| LRRFIP2 | ILMN_1772329 | 0.232 | 0.0389 | 2.14 | -4.1485292 | 0.195 |
| UBQLN2 | ILMN_1680770 | 0.258 | 0.0475 | 2.05 | -4.3174286 | 0.195 |
| CBX3 | ILMN_1790625 | 0.188 | 0.0265 | 2.32 | -3.8176336 | 0.194 |
| DYRK1A | ILMN_1664560 | 0.247 | 0.0439 | 2.09 | -4.2508451 | 0.194 |
| WDR37 | ILMN_1796464 | 0.258 | 0.0475 | 2.05 | -4.3177456 | 0.184 |
| GOLGB1 | ILMN_1747935 | 0.255 | 0.0462 | 2.07 | -4.2948109 | 0.183 |
| RBM4 | ILMN_1757439 | 0.211 | 0.0325 | 2.23 | -3.9944498 | 0.178 |
| ANKRD17 | ILMN_1787064 | 0.243 | 0.0421 | 2.11 | -4.2161326 | 0.176 |
| PPP3R1 | ILMN_1796962 | 0.108 | 0.0099 | 2.72 | -2.953233 | 0.173 |
| EREG | ILMN_1657248 | 0.225 | 0.0366 | 2.17 | -4.0977907 | 0.168 |
| NIPAL2 | ILMN_1774373 | 0.24 | 0.0414 | 2.12 | -4.2016264 | 0.167 |
| PAPD4 | ILMN_1681845 | 0.114 | 0.0111 | 2.68 | -3.0554027 | 0.157 |
| RHOA | ILMN_1781290 | 0.224 | 0.0363 | 2.18 | -4.08881 | 0.153 |
| ZNF234 | ILMN_2103397 | 0.248 | 0.0444 | 2.08 | -4.2612441 | 0.153 |
| KCTD3 | ILMN_2134555 | 0.258 | 0.0476 | 2.05 | -4.3199251 | 0.144 |
| PTGES | ILMN_1713829 | 9.87E-06 | 2.79E-09 | -7.85 | 11.0677708 | -2.12 |
| TGM2 | ILMN_1705750 | 0.000338 | 1.02E-06 | -5.89 | 5.5640815 | -1.99 |
| AKR1A1 | ILMN_1728047 | 0.00188 | 1.64E-05 | -4.98 | 2.9605894 | -1.93 |
| AKR1A1 | ILMN_2380771 | 0.00383 | 4.63E-05 | -4.64 | 1.9854459 | -1.88 |
| KCNH4 | ILMN_1800396 | 9.87E-06 | 2.38E-09 | -7.9 | 11.2148815 | -1.82 |
| FAM213B | ILMN_3243682 | 0.0103 | 0.000212 | -4.12 | 0.566909 | -1.78 |
| UPB1 | ILMN_1678690 | 4.47E-05 | 4.59E-08 | -6.91 | 8.4645394 | -1.77 |
| SNRNP25 | ILMN_1801118 | 0.00639 | 0.000102 | -4.37 | 1.2454858 | -1.71 |
| CYB561A3 | ILMN_2129505 | 0.00103 | 6.52E-06 | -5.28 | 3.8226215 | -1.7 |
| C15orf48 | ILMN_1654696 | 0.00261 | 2.64E-05 | -4.82 | 2.5134289 | -1.68 |
| SRC | ILMN_1729987 | 0.00673 | 0.000112 | -4.34 | 1.1617318 | -1.58 |
| VWF | ILMN_1752755 | 0.0155 | 0.000415 | -3.89 | -0.0577628 | -1.55 |
| TNFAIP8L1 | ILMN_1684346 | 0.00122 | 8.11E-06 | -5.21 | 3.6183396 | -1.54 |
| GPR162 | ILMN_1730816 | 0.064 | 0.00402 | -3.08 | -2.1432335 | -1.49 |
| C15orf48 | ILMN_1805410 | 0.00187 | 1.60E-05 | -4.99 | 2.9822523 | -1.48 |
| EML2 | ILMN_3240541 | 9.64E-06 | 1.82E-09 | -8 | 11.4649016 | -1.45 |
| PRR5L | ILMN_1697491 | 0.0162 | 0.000442 | -3.87 | -0.1162726 | -1.45 |
| LFNG | ILMN_1663080 | 0.000639 | 2.70E-06 | -5.57 | 4.6515757 | -1.44 |
| MMP9 | ILMN_1796316 | 0.0502 | 0.0027 | -3.22 | -1.781527 | -1.42 |
| MARCKSL1 | ILMN_1714433 | 0.000348 | 1.07E-06 | -5.87 | 5.5221957 | -1.4 |
| GATSL3 | ILMN_2098418 | 0.000909 | 4.71E-06 | -5.39 | 4.1276767 | -1.4 |
| AMPD3 | ILMN_1774447 | 0.0026 | 2.61E-05 | -4.83 | 2.5217235 | -1.37 |
| ABCF1 | ILMN_2392635 | 0.00229 | 2.19E-05 | -4.88 | 2.6859202 | -1.35 |
| LRRC75A | ILMN_2221784 | 0.0268 | 0.000947 | -3.6 | -0.8202197 | -1.35 |
| SLC35B2 | ILMN_1789001 | 0.000985 | 6.05E-06 | -5.31 | 3.8923733 | -1.33 |
| ZBTB46 | ILMN_1710092 | 7.52E-07 | 3.54E-11 | -9.39 | 15.0719212 | -1.32 |
| KCNK6 | ILMN_1701173 | 0.00617 | 9.59E-05 | -4.39 | 1.305061 | -1.32 |
| ARRB1 | ILMN_2325168 | 0.00672 | 0.000111 | -4.34 | 1.1687906 | -1.32 |
| PNPLA1 | ILMN_1808241 | 0.000331 | 9.52E-07 | -5.91 | 5.6278994 | -1.31 |
| SLC25A19 | ILMN_1666553 | 0.000925 | 5.01E-06 | -5.37 | 4.0697214 | -1.31 |
| SIK1 | ILMN_3235647 | 0.0122 | 0.000288 | -4.02 | 0.2827098 | -1.3 |
| BCKDHA | ILMN_1735979 | 0.0266 | 0.000937 | -3.61 | -0.8106364 | -1.29 |
| RFX5 | ILMN_1741200 | 0.0627 | 0.00388 | -3.09 | -2.1110922 | -1.29 |
| C11orf21 | ILMN_3235922 | 0.000965 | 5.54E-06 | -5.33 | 3.9749977 | -1.27 |
| TNF | ILMN_1728106 | 0.162 | 0.0201 | -2.43 | -3.577552 | -1.27 |
| LSM4 | ILMN_1788099 | 0.00513 | 6.91E-05 | -4.5 | 1.6114284 | -1.25 |
| ZNF770 | ILMN_1734254 | 0.011 | 0.000242 | -4.08 | 0.4430683 | -1.24 |
| KIF3B | ILMN_2081398 | 0.000337 | 1.00E-06 | -5.89 | 5.5816505 | -1.22 |
| IDH2 | ILMN_1751753 | 0.000646 | 2.86E-06 | -5.55 | 4.5963455 | -1.22 |
| APBA3 | ILMN_1686610 | 0.00828 | 0.000153 | -4.24 | 0.8716241 | -1.21 |
| POLR3K | ILMN_1801664 | 0.0458 | 0.00229 | -3.29 | -1.6293097 | -1.21 |
| PRPSAP1 | ILMN_1768449 | 0.00124 | 8.67E-06 | -5.19 | 3.5555816 | -1.19 |
| PDE6D | ILMN_1790680 | 0.00596 | 9.07E-05 | -4.41 | 1.3576585 | -1.18 |
| SIK1 | ILMN_1717639 | 0.0224 | 0.000708 | -3.71 | -0.5520132 | -1.18 |
| IL1A | ILMN_1658483 | 0.159 | 0.0194 | -2.45 | -3.5477607 | -1.18 |
| COMMD3 | ILMN_1690392 | 0.0902 | 0.00722 | -2.85 | -2.6710897 | -1.17 |
| RTN2 | ILMN_1749115 | 0.000414 | 1.47E-06 | -5.77 | 5.2222063 | -1.16 |
| CLCF1 | ILMN_1661197 | 0.00935 | 0.000185 | -4.17 | 0.6933874 | -1.16 |
| C15orf48 | ILMN_2389064 | 0.0395 | 0.00179 | -3.38 | -1.4046136 | -1.16 |
| MED19 | ILMN_1754553 | 0.00562 | 8.18E-05 | -4.45 | 1.4535131 | -1.15 |
| ZNF324 | ILMN_1745784 | 0.00589 | 8.85E-05 | -4.42 | 1.3807729 | -1.15 |
| HOMER3 | ILMN_1811579 | 0.0449 | 0.0022 | -3.3 | -1.5944084 | -1.14 |
| HMGCS1 | ILMN_1797728 | 0.0409 | 0.00189 | -3.35 | -1.4576189 | -1.13 |
| ABCF1 | ILMN_1763875 | 0.00229 | 2.19E-05 | -4.88 | 2.6866474 | -1.12 |
| SGTA | ILMN_1677800 | 1.80E-05 | 8.79E-09 | -7.46 | 10.0037433 | -1.11 |
| LYRM7 | ILMN_3238623 | 0.0263 | 0.000921 | -3.61 | -0.7944195 | -1.11 |
| NKIRAS1 | ILMN_1664216 | 0.0465 | 0.00237 | -3.27 | -1.6624956 | -1.11 |
| CADM4 | ILMN_1812096 | 0.00236 | 2.31E-05 | -4.87 | 2.6368891 | -1.1 |
| ETFB | ILMN_2300970 | 0.00822 | 0.000149 | -4.24 | 0.8952319 | -1.1 |
| TJAP1 | ILMN_1743763 | 0.0152 | 0.000405 | -3.9 | -0.0353846 | -1.1 |
| FKBP2 | ILMN_1674337 | 1.10E-05 | 3.61E-09 | -7.76 | 10.8278615 | -1.09 |
| NAT9 | ILMN_1776088 | 0.00824 | 0.00015 | -4.24 | 0.8857596 | -1.09 |
| BBS4 | ILMN_1762466 | 0.0438 | 0.00212 | -3.31 | -1.5596604 | -1.08 |
| GSE1 | ILMN_1807767 | 0.000985 | 6.12E-06 | -5.3 | 3.8816632 | -1.07 |
| TBC1D9B | ILMN_2390227 | 0.00552 | 7.93E-05 | -4.46 | 1.483082 | -1.07 |
| LIF | ILMN_1738725 | 3.17E-05 | 2.39E-08 | -7.12 | 9.0750347 | -1.06 |
| ADAP1 | ILMN_3247424 | 0.00154 | 1.19E-05 | -5.08 | 3.2597536 | -1.06 |
| EDC4 | ILMN_1665212 | 0.00216 | 1.95E-05 | -4.92 | 2.7934535 | -1.05 |
| PDXP | ILMN_1736441 | 0.00711 | 0.000123 | -4.31 | 1.0766344 | -1.05 |
| CD300LF | ILMN_2112357 | 0.0148 | 0.000386 | -3.92 | 0.0083878 | -1.05 |
| ADORA2A | ILMN_1807372 | 0.0185 | 0.000539 | -3.8 | -0.2995803 | -1.05 |
| ALDOC | ILMN_1755974 | 0.134 | 0.0145 | -2.57 | -3.2911143 | -1.05 |
| ASB6 | ILMN_2397776 | 0.00129 | 9.26E-06 | -5.17 | 3.4940554 | -1.04 |
| TMX2 | ILMN_1799367 | 0.0168 | 0.000465 | -3.85 | -0.1633446 | -1.04 |
| NUDT16L1 | ILMN_1735415 | 0.0208 | 0.000643 | -3.74 | -0.4639108 | -1.04 |
| TMEM268 | ILMN_1803652 | 0.0381 | 0.00168 | -3.4 | -1.347957 | -1.04 |
| TRAF3 | ILMN_2383774 | 0.0023 | 2.21E-05 | -4.88 | 2.6779922 | -1.03 |
| EXOSC1 | ILMN_2396648 | 0.024 | 0.00079 | -3.67 | -0.6538246 | -1.03 |
| SLC25A39 | ILMN_1743911 | 0.00131 | 9.41E-06 | -5.16 | 3.4791516 | -1.02 |
| SIGLEC10 | ILMN_1655549 | 0.0104 | 0.000225 | -4.1 | 0.5101218 | -1.02 |
| MYPOP | ILMN_1704793 | 0.0373 | 0.00162 | -3.41 | -1.3124172 | -1.02 |
| PRDX1 | ILMN_2366391 | 0.128 | 0.0133 | -2.6 | -3.2180952 | -1.02 |
| ASB6 | ILMN_1806705 | 0.000944 | 5.38E-06 | -5.34 | 4.0034807 | -1.01 |
| PREB | ILMN_1733930 | 0.00228 | 2.14E-05 | -4.89 | 2.7069562 | -1.01 |
| TRIOBP | ILMN_1735788 | 0.00354 | 3.91E-05 | -4.69 | 2.1440434 | -1.01 |
| NSUN5 | ILMN_1751958 | 0.0165 | 0.000454 | -3.86 | -0.1418782 | -1.01 |
| CCDC189 | ILMN_2179726 | 0.0266 | 0.000935 | -3.61 | -0.8088839 | -1.01 |
| CCL3 | ILMN_1671509 | 0.138 | 0.0153 | -2.55 | -3.3395698 | -1.01 |
| NFKBID | ILMN_1763560 | 0.000639 | 2.77E-06 | -5.56 | 4.6268322 | -1 |
| ETFB | ILMN_1729374 | 0.00123 | 8.50E-06 | -5.19 | 3.5735114 | -1 |
| OSM | ILMN_1780546 | 0.00935 | 0.000185 | -4.17 | 0.69068 | -1 |
| TNFAIP2 | ILMN_1727689 | 0.00168 | 1.35E-05 | -5.04 | 3.1374704 | -0.999 |
| IL1B | ILMN_1775501 | 0.158 | 0.0193 | -2.45 | -3.544322 | -0.999 |
| ARRB1 | ILMN_1819608 | 0.13 | 0.0138 | -2.59 | -3.2451742 | -0.998 |
| NSUN5 | ILMN_2408400 | 0.0244 | 0.00081 | -3.66 | -0.6760167 | -0.997 |
| ARRB1 | ILMN_1730620 | 0.00359 | 4.03E-05 | -4.68 | 2.1165405 | -0.995 |
| HVCN1 | ILMN_2365248 | 0.123 | 0.0124 | -2.63 | -3.1520446 | -0.994 |
| RAB37 | ILMN_2243912 | 0.00579 | 8.61E-05 | -4.43 | 1.4061751 | -0.993 |
| AGAP3 | ILMN_1795918 | 0.00972 | 0.000197 | -4.15 | 0.633039 | -0.985 |
| RUNX3 | ILMN_1787461 | 1.21E-05 | 4.55E-09 | -7.68 | 10.6138006 | -0.982 |
| PTPN22 | ILMN_2246328 | 0.0116 | 0.000263 | -4.05 | 0.366754 | -0.98 |
| GJC2 | ILMN_1723048 | 0.0822 | 0.0061 | -2.92 | -2.5196677 | -0.979 |
| FAM162A | ILMN_1803647 | 0.0602 | 0.0036 | -3.12 | -2.0430702 | -0.975 |
| AP3S2 | ILMN_1731596 | 0.00261 | 2.65E-05 | -4.82 | 2.5091009 | -0.971 |
| P2RY2 | ILMN_2372915 | 0.0667 | 0.00433 | -3.05 | -2.2095462 | -0.96 |
| SULF2 | ILMN_2345142 | 0.0183 | 0.000531 | -3.81 | -0.2866386 | -0.958 |
| SH3BP1 | ILMN_1692539 | 0.00452 | 5.90E-05 | -4.56 | 1.7590975 | -0.957 |
| TCFL5 | ILMN_1814247 | 0.00981 | 2.00E-04 | -4.14 | 0.6184858 | -0.955 |
| PGLYRP2 | ILMN_3307921 | 0.000985 | 6.07E-06 | -5.3 | 3.8891104 | -0.95 |
| ESYT1 | ILMN_1761159 | 0.0254 | 0.000871 | -3.63 | -0.74339 | -0.95 |
| TMEM218 | ILMN_2395856 | 0.00429 | 5.49E-05 | -4.58 | 1.8262471 | -0.948 |
| ADARB2 | ILMN_1749493 | 0.0397 | 0.0018 | -3.37 | -1.4100132 | -0.948 |
| CD33 | ILMN_1747622 | 0.031 | 0.00119 | -3.52 | -1.0329982 | -0.939 |
| SEC24C | ILMN_1676600 | 0.168 | 0.0214 | -2.41 | -3.6326907 | -0.938 |
| HINFP | ILMN_1667453 | 0.000812 | 3.99E-06 | -5.44 | 4.2824628 | -0.937 |
| DUSP2 | ILMN_1712959 | 0.0121 | 0.00028 | -4.03 | 0.3078434 | -0.935 |
| ARL5A | ILMN_2332558 | 0.0627 | 0.00388 | -3.09 | -2.1110665 | -0.935 |
| ASCL2 | ILMN_1723412 | 0.0104 | 0.000222 | -4.11 | 0.5236959 | -0.934 |
| PLCB2 | ILMN_1724066 | 0.00513 | 6.96E-05 | -4.5 | 1.6052021 | -0.933 |
| SDHAF1 | ILMN_2070355 | 0.0594 | 0.00351 | -3.13 | -2.0208103 | -0.933 |
| PMPCA | ILMN_1764239 | 8.55E-05 | 1.05E-07 | -6.63 | 7.6953891 | -0.932 |
| TNNT3 | ILMN_2334080 | 0.0022 | 2.02E-05 | -4.91 | 2.7624461 | -0.93 |
| BID | ILMN_1763386 | 0.000168 | 2.85E-07 | -6.3 | 6.7569706 | -0.927 |
| MAN2B1 | ILMN_1759341 | 0.000283 | 6.80E-07 | -6.02 | 5.9427197 | -0.925 |
| KIF3B | ILMN_1702279 | 0.00169 | 1.38E-05 | -5.03 | 3.1178094 | -0.925 |
| PLAGL2 | ILMN_1786601 | 0.00983 | 0.000201 | -4.14 | 0.6140357 | -0.924 |
| BMF | ILMN_2308338 | 0.0225 | 0.000711 | -3.71 | -0.5567883 | -0.923 |
| IER5 | ILMN_1721833 | 0.0159 | 0.000433 | -3.88 | -0.0979253 | -0.921 |
| FAM43A | ILMN_1706015 | 0.0266 | 0.000933 | -3.61 | -0.8065052 | -0.92 |
| PHKG2 | ILMN_1669607 | 0.0525 | 0.00291 | -3.2 | -1.8484276 | -0.92 |
| TGM3 | ILMN_1786847 | 0.0885 | 0.00697 | -2.86 | -2.6396511 | -0.915 |
| IKBKE | ILMN_1755024 | 0.0113 | 0.000251 | -4.07 | 0.4088212 | -0.914 |
| VGLL4 | ILMN_1768480 | 0.0852 | 0.00648 | -2.89 | -2.5731186 | -0.912 |
| ABHD8 | ILMN_1712707 | 0.0017 | 1.40E-05 | -5.03 | 3.1028748 | -0.911 |
| NOL12 | ILMN_1759991 | 0.000179 | 3.12E-07 | -6.27 | 6.6739761 | -0.91 |
| LAGE3 | ILMN_1708151 | 0.0125 | 0.000301 | -4 | 0.2404991 | -0.909 |
| LRG1 | ILMN_1805228 | 0.256 | 0.0468 | -2.06 | -4.3053493 | -0.907 |
| TRMT1 | ILMN_1812940 | 0.00263 | 2.68E-05 | -4.82 | 2.4977989 | -0.904 |
| SNAPC1 | ILMN_1725346 | 0.0737 | 0.005 | -2.99 | -2.3393475 | -0.904 |
| RNF121 | ILMN_2356031 | 0.00229 | 2.18E-05 | -4.88 | 2.6889629 | -0.899 |
| ZNF845 | ILMN_3237579 | 0.0464 | 0.00235 | -3.28 | -1.6530962 | -0.899 |
| PRDX1 | ILMN_2366388 | 0.144 | 0.0164 | -2.52 | -3.3989093 | -0.899 |
| BID | ILMN_2259495 | 0.0155 | 0.000418 | -3.89 | -0.0636178 | -0.897 |
| RAB37 | ILMN_2255579 | 0.00872 | 0.000165 | -4.21 | 0.8012804 | -0.896 |
| VEGFB | ILMN_1726981 | 0.00593 | 8.99E-05 | -4.41 | 1.3652859 | -0.895 |
| MEF2C | ILMN_1742544 | 0.0482 | 0.00254 | -3.25 | -1.7250375 | -0.892 |
| FAM234A | ILMN_1810055 | 9.18E-05 | 1.17E-07 | -6.6 | 7.5932971 | -0.889 |
| AP4B1 | ILMN_1669377 | 0.00158 | 1.25E-05 | -5.07 | 3.2106482 | -0.887 |
| RELT | ILMN_1748614 | 0.0271 | 0.000966 | -3.6 | -0.8391888 | -0.885 |
| ATP7A | ILMN_1808115 | 0.0395 | 0.00179 | -3.38 | -1.4051263 | -0.884 |
| COA5 | ILMN_1683065 | 0.224 | 0.0363 | -2.18 | -4.0892108 | -0.884 |
| RINL | ILMN_1790962 | 0.00176 | 1.45E-05 | -5.02 | 3.0700197 | -0.882 |
| PSMD4 | ILMN_1728355 | 2.03E-05 | 1.24E-08 | -7.34 | 9.6835215 | -0.881 |
| XXYLT1 | ILMN_1671116 | 0.0426 | 0.00203 | -3.33 | -1.5203423 | -0.881 |
| ORAI3 | ILMN_1736628 | 0.0484 | 0.00257 | -3.24 | -1.7362192 | -0.88 |
| CPSF2 | ILMN_1673185 | 0.0364 | 0.00156 | -3.43 | -1.27878 | -0.879 |
| PSME3 | ILMN_2346573 | 0.0997 | 0.00853 | -2.78 | -2.8203121 | -0.879 |
| PTP4A3 | ILMN_1769779 | 0.000639 | 2.69E-06 | -5.57 | 4.6521773 | -0.877 |
| TECPR1 | ILMN_1767651 | 0.019 | 0.000566 | -3.79 | -0.3453849 | -0.877 |
| RASSF1 | ILMN_1734205 | 0.171 | 0.0221 | -2.39 | -3.6619006 | -0.877 |
| RHOQ | ILMN_1810559 | 0.00589 | 8.83E-05 | -4.42 | 1.3826401 | -0.876 |
| PPP2R5D | ILMN_1780940 | 0.00136 | 1.01E-05 | -5.14 | 3.4133106 | -0.873 |
| CCL3L1 | ILMN_1747355 | 0.179 | 0.0242 | -2.35 | -3.7414697 | -0.871 |
| SNAPC4 | ILMN_1677484 | 0.0683 | 0.00446 | -3.04 | -2.2375784 | -0.87 |
| BID | ILMN_2372413 | 0.00157 | 1.23E-05 | -5.07 | 3.2288176 | -0.869 |
| DNAL4 | ILMN_1801845 | 0.0854 | 0.00649 | -2.89 | -2.5756299 | -0.869 |
| NFKBIE | ILMN_1717313 | 0.0132 | 0.000327 | -3.98 | 0.1625999 | -0.868 |
| DAPK3 | ILMN_1792710 | 0.0104 | 0.000218 | -4.11 | 0.5380956 | -0.866 |
| NFKB2 | ILMN_1799062 | 0.0116 | 0.000261 | -4.05 | 0.3730306 | -0.866 |
| ATP6V0A1 | ILMN_1752579 | 0.00928 | 0.000183 | -4.18 | 0.7047933 | -0.865 |
| SAR1B | ILMN_2394296 | 0.065 | 0.00413 | -3.07 | -2.1669653 | -0.865 |
| RHOF | ILMN_1652918 | 0.22 | 0.0352 | -2.19 | -4.0641062 | -0.865 |
| AMACR | ILMN_2367172 | 0.115 | 0.0112 | -2.67 | -3.063652 | -0.864 |
| MCRIP2 | ILMN_1730523 | 0.000158 | 2.44E-07 | -6.35 | 6.9025018 | -0.86 |
| ZBTB45 | ILMN_1661484 | 0.00123 | 8.52E-06 | -5.19 | 3.5717571 | -0.859 |
| CYP27A1 | ILMN_1704985 | 0.000639 | 2.65E-06 | -5.57 | 4.6669287 | -0.858 |
| ZNF12 | ILMN_1784577 | 0.0619 | 0.00381 | -3.1 | -2.0931834 | -0.858 |
| SAR1B | ILMN_1736888 | 0.0152 | 0.000405 | -3.9 | -0.035538 | -0.857 |
| CD59 | ILMN_2333687 | 0.0629 | 0.00392 | -3.09 | -2.119288 | -0.857 |
| LMAN2L | ILMN_1755221 | 0.00252 | 2.49E-05 | -4.84 | 2.5662619 | -0.855 |
| STK19 | ILMN_1659782 | 0.243 | 0.0422 | -2.11 | -4.2176824 | -0.855 |
| GON7 | ILMN_1685781 | 0.0542 | 0.00305 | -3.18 | -1.8927324 | -0.854 |
| AP3M1 | ILMN_1667086 | 0.0786 | 0.00557 | -2.95 | -2.4378498 | -0.854 |
| EIF4ENIF1 | ILMN_1794967 | 0.1 | 0.0086 | -2.78 | -2.8274318 | -0.854 |
| TESC | ILMN_1750181 | 0.000243 | 5.61E-07 | -6.08 | 6.1241871 | -0.853 |
| SH2B3 | ILMN_1752046 | 0.0379 | 0.00167 | -3.4 | -1.3423423 | -0.853 |
| C7orf50 | ILMN_1718336 | 0.102 | 0.00888 | -2.77 | -2.8556953 | -0.853 |
| CYBB | ILMN_1682312 | 0.258 | 0.0479 | -2.05 | -4.3245206 | -0.853 |
| SHPK | ILMN_2216918 | 0.00751 | 0.000132 | -4.28 | 1.0058228 | -0.852 |
| GHRL | ILMN_1696380 | 0.0888 | 0.00701 | -2.86 | -2.6443876 | -0.851 |
| AGPAT1 | ILMN_1679520 | 0.00606 | 9.30E-05 | -4.4 | 1.3341635 | -0.85 |
| CLN5 | ILMN_1778203 | 0.0599 | 0.00357 | -3.12 | -2.0341306 | -0.85 |
| TKTL1 | ILMN_1674009 | 0.147 | 0.017 | -2.51 | -3.4293365 | -0.849 |
| ZNF140 | ILMN_1727923 | 0.00772 | 0.000138 | -4.27 | 0.9676726 | -0.848 |
| TUSC2 | ILMN_1804329 | 0.0142 | 0.000367 | -3.94 | 0.0567866 | -0.848 |
| EEPD1 | ILMN_1811616 | 0.0373 | 0.00162 | -3.41 | -1.3160705 | -0.848 |
| MED22 | ILMN_1697218 | 0.0879 | 0.00689 | -2.87 | -2.6287686 | -0.848 |
| SMUG1 | ILMN_1804642 | 0.0978 | 0.00828 | -2.8 | -2.7931248 | -0.846 |
| UROD | ILMN_1740742 | 0.0502 | 0.0027 | -3.23 | -1.7797524 | -0.845 |
| SLC16A6 | ILMN_1729691 | 0.188 | 0.0265 | -2.32 | -3.8182797 | -0.845 |
| TRAF1 | ILMN_1698218 | 0.0113 | 0.00025 | -4.07 | 0.4122605 | -0.844 |
| CARD9 | ILMN_1712532 | 0.0992 | 0.00844 | -2.79 | -2.810827 | -0.844 |
| UBAC1 | ILMN_1807044 | 0.165 | 0.0207 | -2.42 | -3.6054107 | -0.844 |
| DPEP3 | ILMN_1731275 | 0.207 | 0.0317 | -2.24 | -3.9723088 | -0.844 |
| SPAG7 | ILMN_1684446 | 0.0104 | 0.000221 | -4.11 | 0.5270808 | -0.843 |
| ARL5A | ILMN_1688526 | 0.0419 | 0.00197 | -3.34 | -1.4953033 | -0.843 |
| FIBP | ILMN_1657797 | 0.0648 | 0.00409 | -3.07 | -2.1590072 | -0.84 |
| PSMB10 | ILMN_1683026 | 0.117 | 0.0114 | -2.67 | -3.077442 | -0.84 |
| C1orf50 | ILMN_1801941 | 0.143 | 0.0163 | -2.52 | -3.3931561 | -0.839 |
| IER5L | ILMN_1755620 | 0.23 | 0.0382 | -2.15 | -4.1328441 | -0.839 |
| PRDX5 | ILMN_1815024 | 3.51E-06 | 3.31E-10 | -8.59 | 13.0329126 | -0.838 |
| PTP4A3 | ILMN_1662427 | 0.002 | 1.77E-05 | -4.95 | 2.8847872 | -0.838 |
| AMACR | ILMN_1759670 | 0.149 | 0.0176 | -2.49 | -3.4600876 | -0.837 |
| ESRRA | ILMN_1774272 | 0.00024 | 5.43E-07 | -6.09 | 6.1536716 | -0.836 |
| FMNL3 | ILMN_2395214 | 0.00187 | 1.61E-05 | -4.98 | 2.9737521 | -0.836 |
| TESK2 | ILMN_1654370 | 0.0486 | 0.00259 | -3.24 | -1.7438106 | -0.835 |
| TRAPPC4 | ILMN_1814650 | 0.177 | 0.024 | -2.36 | -3.7312667 | -0.835 |
| ARFGAP1 | ILMN_1675709 | 0.0314 | 0.00122 | -3.51 | -1.0517478 | -0.833 |
| CCL3L1 | ILMN_1773245 | 0.134 | 0.0146 | -2.57 | -3.2960769 | -0.833 |
| PSMD4 | ILMN_2344130 | 7.22E-05 | 7.93E-08 | -6.72 | 7.9546747 | -0.832 |
| FAM118A | ILMN_1809147 | 0.0244 | 0.00081 | -3.66 | -0.6760302 | -0.831 |
| E4F1 | ILMN_1720287 | 0.0465 | 0.00238 | -3.27 | -1.665578 | -0.831 |
| SMPD2 | ILMN_1672176 | 0.0315 | 0.00122 | -3.51 | -1.0567909 | -0.83 |
| RASSF1 | ILMN_2393573 | 0.149 | 0.0174 | -2.49 | -3.4525566 | -0.829 |
| RPP25L | ILMN_1683175 | 0.218 | 0.0345 | -2.2 | -4.0463827 | -0.829 |
| VEGFB | ILMN_1722855 | 0.000812 | 3.99E-06 | -5.44 | 4.2825852 | -0.827 |
| BAD | ILMN_1738652 | 0.00552 | 7.93E-05 | -4.46 | 1.483167 | -0.827 |
| PLAU | ILMN_1656057 | 0.117 | 0.0114 | -2.67 | -3.0817206 | -0.827 |
| AMACR | ILMN_1792741 | 0.0429 | 0.00206 | -3.32 | -1.5338072 | -0.826 |
| TBC1D2B | ILMN_2064606 | 0.0648 | 0.00409 | -3.07 | -2.1593356 | -0.825 |
| PSMD4 | ILMN_3307799 | 0.000191 | 4.12E-07 | -6.18 | 6.4128506 | -0.822 |
| COPS6 | ILMN_1764431 | 0.00842 | 0.000156 | -4.23 | 0.8503774 | -0.822 |
| STXBP6 | ILMN_1750912 | 0.143 | 0.0162 | -2.52 | -3.3874727 | -0.822 |
| HIC1 | ILMN_1738825 | 0.000519 | 1.98E-06 | -5.67 | 4.9406526 | -0.82 |
| TNFAIP8L2 | ILMN_1744113 | 0.0664 | 0.0043 | -3.05 | -2.2033932 | -0.818 |
| PIP5K1C | ILMN_1668514 | 0.0579 | 0.00339 | -3.14 | -1.987915 | -0.817 |
| CHD9 | ILMN_1762972 | 0.00113 | 7.33E-06 | -5.24 | 3.7127475 | -0.813 |
| RAB37 | ILMN_1796136 | 0.0509 | 0.00277 | -3.22 | -1.8028427 | -0.812 |
| ZNF627 | ILMN_2197519 | 0.177 | 0.0237 | -2.36 | -3.722518 | -0.81 |
| AAGAB | ILMN_3237396 | 0.0315 | 0.00123 | -3.51 | -1.0586778 | -0.807 |
| MTFP1 | ILMN_2355665 | 0.0898 | 0.00712 | -2.86 | -2.6580151 | -0.807 |
| NQO2 | ILMN_1712918 | 0.048 | 0.00252 | -3.25 | -1.7167864 | -0.806 |
| HPS6 | ILMN_1718537 | 0.226 | 0.0371 | -2.17 | -4.1093539 | -0.806 |
| TNIP1 | ILMN_1703650 | 0.000334 | 9.75E-07 | -5.9 | 5.6056629 | -0.802 |
| NDUFA6 | ILMN_3238269 | 0.0595 | 0.00353 | -3.12 | -2.0237531 | -0.8 |
| PDRG1 | ILMN_1731720 | 0.00553 | 7.97E-05 | -4.45 | 1.4781455 | -0.798 |
| MIF4GD | ILMN_1743806 | 0.00872 | 0.000164 | -4.21 | 0.805243 | -0.797 |
| PSME3 | ILMN_1800975 | 0.173 | 0.0227 | -2.38 | -3.6849793 | -0.797 |
| ZNF213 | ILMN_1758337 | 0.174 | 0.0231 | -2.37 | -3.7003244 | -0.796 |
| MKRN2 | ILMN_2056760 | 0.155 | 0.0187 | -2.46 | -3.5149547 | -0.792 |
| B3GNT8 | ILMN_1741389 | 0.000925 | 5.00E-06 | -5.37 | 4.0719898 | -0.789 |
| LBHD1 | ILMN_1739345 | 0.0694 | 0.00457 | -3.03 | -2.2596233 | -0.789 |
| BMF | ILMN_1695354 | 0.0588 | 0.00346 | -3.13 | -2.006901 | -0.788 |
| SS18L1 | ILMN_1676625 | 0.0772 | 0.0054 | -2.96 | -2.4095385 | -0.787 |
| MRPS11 | ILMN_1722905 | 0.148 | 0.0172 | -2.5 | -3.4409686 | -0.787 |
| PAM16 | ILMN_1763884 | 0.0169 | 0.000471 | -3.85 | -0.1760564 | -0.786 |
| ZW10 | ILMN_1712556 | 0.0125 | 0.000301 | -4.01 | 0.2412179 | -0.784 |
| NSUN5P1 | ILMN_2352448 | 0.0404 | 0.00185 | -3.36 | -1.4367902 | -0.784 |
| ZNF395 | ILMN_1772876 | 0.135 | 0.0146 | -2.57 | -3.2983263 | -0.783 |
| NDUFS3 | ILMN_1756355 | 0.0297 | 0.00111 | -3.55 | -0.9658404 | -0.782 |
| SLC16A5 | ILMN_1755649 | 0.0528 | 0.00293 | -3.19 | -1.8552613 | -0.782 |
| TMEM79 | ILMN_2090802 | 0.0531 | 0.00295 | -3.19 | -1.8628824 | -0.781 |
| PPP2R5B | ILMN_2124082 | 0.158 | 0.0193 | -2.45 | -3.5435263 | -0.78 |
| SUN2 | ILMN_2099301 | 0.00426 | 5.39E-05 | -4.59 | 1.8432839 | -0.779 |
| NOL12 | ILMN_2151368 | 0.0134 | 0.000335 | -3.97 | 0.1408454 | -0.778 |
| NBN | ILMN_2358041 | 0.203 | 0.0306 | -2.25 | -3.9421594 | -0.778 |
| HDGF | ILMN_1765621 | 0.0323 | 0.00129 | -3.49 | -1.1030592 | -0.777 |
| GBA2 | ILMN_1674560 | 0.00168 | 1.37E-05 | -5.04 | 3.1288986 | -0.773 |
| PITPNM1 | ILMN_1653220 | 0.00187 | 1.61E-05 | -4.98 | 2.9729672 | -0.772 |
| LYSMD2 | ILMN_1724493 | 0.027 | 0.000957 | -3.6 | -0.8306927 | -0.772 |
| FAM136A | ILMN_1655307 | 0.0655 | 0.0042 | -3.06 | -2.1817541 | -0.772 |
| ANKRD49 | ILMN_1669259 | 0.243 | 0.0422 | -2.11 | -4.2172587 | -0.772 |
| CD300LB | ILMN_1782741 | 0.0627 | 0.00389 | -3.09 | -2.1124922 | -0.77 |
| PIGS | ILMN_1691291 | 0.132 | 0.0141 | -2.58 | -3.2658386 | -0.77 |
| ELK1 | ILMN_1654289 | 0.106 | 0.00952 | -2.74 | -2.9176256 | -0.769 |
| NFKBIB | ILMN_1690473 | 0.0322 | 0.00127 | -3.5 | -1.0941468 | -0.768 |
| RANBP3 | ILMN_1723689 | 0.000984 | 5.70E-06 | -5.33 | 3.9493402 | -0.766 |
| NFKB2 | ILMN_2390859 | 0.0204 | 0.000627 | -3.75 | -0.4397373 | -0.766 |
| CDKN1A | ILMN_1784602 | 0.0381 | 0.00169 | -3.4 | -1.3522545 | -0.765 |
| ZNRD1 | ILMN_1722894 | 0.00153 | 1.18E-05 | -5.09 | 3.2695933 | -0.764 |
| RASSF7 | ILMN_1733110 | 0.158 | 0.0193 | -2.45 | -3.5428426 | -0.763 |
| TFRC | ILMN_1674243 | 0.256 | 0.0468 | -2.06 | -4.3056755 | -0.762 |
| TBC1D22A | ILMN_2096743 | 0.0126 | 0.000305 | -4 | 0.2268178 | -0.761 |
| CES2 | ILMN_1696675 | 0.0551 | 0.00312 | -3.17 | -1.9126652 | -0.761 |
| TMEM203 | ILMN_2073010 | 0.0478 | 0.00249 | -3.25 | -1.7081333 | -0.76 |
| ZDHHC13 | ILMN_1684663 | 0.077 | 0.00538 | -2.96 | -2.4053061 | -0.76 |
| MPC1 | ILMN_1666967 | 0.00613 | 9.50E-05 | -4.4 | 1.3140999 | -0.759 |
| P4HA1 | ILMN_1693334 | 0.183 | 0.0252 | -2.34 | -3.7769025 | -0.759 |
| HIGD1A | ILMN_1674522 | 0.00136 | 1.00E-05 | -5.14 | 3.4214208 | -0.758 |
| BRPF1 | ILMN_1669308 | 0.00631 | 9.99E-05 | -4.38 | 1.2670393 | -0.757 |
| ANKDD1A | ILMN_1813139 | 0.00495 | 6.58E-05 | -4.52 | 1.6574518 | -0.756 |
| INPPL1 | ILMN_1728426 | 0.00822 | 0.000148 | -4.25 | 0.8998826 | -0.756 |
| TXNDC17 | ILMN_1659437 | 0.131 | 0.0139 | -2.59 | -3.2565496 | -0.755 |
| SYTL1 | ILMN_1750785 | 0.0287 | 0.00105 | -3.57 | -0.9131104 | -0.754 |
| PRPSAP2 | ILMN_2116661 | 0.0803 | 0.00584 | -2.93 | -2.4804384 | -0.753 |
| SLC2A6 | ILMN_1778321 | 0.149 | 0.0173 | -2.5 | -3.446684 | -0.753 |
| MPC1 | ILMN_2226324 | 0.000388 | 1.26E-06 | -5.82 | 5.3645256 | -0.752 |
| TGIF2 | ILMN_1709044 | 0.0125 | 0.000297 | -4.01 | 0.2512542 | -0.752 |
| B3GALT4 | ILMN_1690682 | 0.177 | 0.024 | -2.36 | -3.7320496 | -0.751 |
| POLR2D | ILMN_1792672 | 0.00576 | 8.49E-05 | -4.43 | 1.419603 | -0.749 |
| NOMO1 | ILMN_2126957 | 0.0233 | 0.000755 | -3.68 | -0.6111139 | -0.748 |
| CD83 | ILMN_2328666 | 0.175 | 0.0234 | -2.37 | -3.7121701 | -0.748 |
| ARL5B | ILMN_2120022 | 0.198 | 0.0292 | -2.27 | -3.90225 | -0.748 |
| LINC01451 | ILMN_1838767 | 0.124 | 0.0127 | -2.62 | -3.1746665 | -0.747 |
| FAM220A | ILMN_1717219 | 0.0219 | 0.000689 | -3.72 | -0.5274876 | -0.746 |
| ZNF615 | ILMN_1672135 | 0.0336 | 0.00139 | -3.47 | -1.1718658 | -0.746 |
| PEA15 | ILMN_1771376 | 0.165 | 0.0209 | -2.42 | -3.6145061 | -0.746 |
| CCDC88B | ILMN_1772208 | 0.000316 | 8.63E-07 | -5.94 | 5.7193621 | -0.745 |
| BCL2L12 | ILMN_1752953 | 0.0232 | 0.000749 | -3.69 | -0.6045978 | -0.745 |
| AK2 | ILMN_1670542 | 0.0105 | 0.000227 | -4.1 | 0.5012007 | -0.744 |
| ARIH2 | ILMN_1792825 | 0.0663 | 0.00427 | -3.05 | -2.1981036 | -0.744 |
| WRAP73 | ILMN_1711166 | 0.0925 | 0.00758 | -2.83 | -2.7142619 | -0.744 |
| STRIP1 | ILMN_1791217 | 0.0138 | 0.000349 | -3.95 | 0.1018631 | -0.743 |
| NELFCD | ILMN_1663954 | 0.00267 | 2.72E-05 | -4.81 | 2.4821824 | -0.742 |
| AK2 | ILMN_1716053 | 0.00639 | 0.000102 | -4.37 | 1.2472919 | -0.742 |
| PRPF38A | ILMN_1675626 | 0.0609 | 0.00368 | -3.11 | -2.0614445 | -0.742 |
| NANOS3 | ILMN_1804007 | 0.113 | 0.0109 | -2.69 | -3.0377642 | -0.741 |
| FUNDC1 | ILMN_1728540 | 0.0055 | 7.83E-05 | -4.46 | 1.4950141 | -0.74 |
| CYB561D2 | ILMN_1738718 | 0.0848 | 0.00643 | -2.9 | -2.566532 | -0.74 |
| CD47 | ILMN_1771333 | 0.218 | 0.0344 | -2.2 | -4.0442531 | -0.74 |
| MZF1 | ILMN_1749838 | 0.129 | 0.0136 | -2.6 | -3.2366229 | -0.738 |
| FBXO18 | ILMN_1729430 | 0.145 | 0.0165 | -2.52 | -3.4059695 | -0.738 |
| PTP4A3 | ILMN_2359710 | 0.00168 | 1.35E-05 | -5.04 | 3.1367747 | -0.735 |
| ADAP1 | ILMN_2047511 | 0.0155 | 0.000415 | -3.89 | -0.0586452 | -0.735 |
| FAM89B | ILMN_1804117 | 0.0338 | 0.0014 | -3.46 | -1.1806206 | -0.735 |
| PDE9A | ILMN_2306540 | 0.243 | 0.0421 | -2.11 | -4.2161591 | -0.735 |
| PDE12 | ILMN_1660305 | 0.0038 | 4.52E-05 | -4.64 | 2.0093617 | -0.734 |
| TRIM52 | ILMN_1754130 | 0.0739 | 0.00504 | -2.99 | -2.3464556 | -0.734 |
| TBL2 | ILMN_1764489 | 0.00209 | 1.88E-05 | -4.93 | 2.828159 | -0.733 |
| KLHL22 | ILMN_1705390 | 0.0225 | 0.000714 | -3.7 | -0.5595228 | -0.733 |
| DNAJC14 | ILMN_1785177 | 0.139 | 0.0156 | -2.54 | -3.3532941 | -0.733 |
| DENND4A | ILMN_2041161 | 0.16 | 0.0197 | -2.44 | -3.5606759 | -0.733 |
| AGO3 | ILMN_1761049 | 0.0118 | 0.000269 | -4.04 | 0.344428 | -0.732 |
| NINJ2 | ILMN_1731745 | 2.39E-05 | 1.61E-08 | -7.25 | 9.439101 | -0.731 |
| PECAM1 | ILMN_1689518 | 0.102 | 0.00889 | -2.77 | -2.8566568 | -0.731 |
| LONP1 | ILMN_1766125 | 0.185 | 0.0257 | -2.33 | -3.7929079 | -0.731 |
| TNNI2 | ILMN_2169261 | 0.19 | 0.0269 | -2.31 | -3.8324307 | -0.731 |
| LRR1 | ILMN_1715616 | 0.186 | 0.0261 | -2.32 | -3.8051504 | -0.73 |
| ERO1A | ILMN_1744963 | 0.212 | 0.0329 | -2.22 | -4.0044954 | -0.73 |
| FCHO1 | ILMN_1654571 | 0.121 | 0.0121 | -2.64 | -3.1323938 | -0.729 |
| CPSF4 | ILMN_1660426 | 0.0686 | 0.00449 | -3.03 | -2.2436418 | -0.726 |
| CCDC47 | ILMN_1804522 | 0.149 | 0.0175 | -2.49 | -3.454574 | -0.726 |
| PGAM1 | ILMN_2112417 | 0.0419 | 0.00197 | -3.34 | -1.4953029 | -0.724 |
| CD83 | ILMN_1780582 | 0.17 | 0.022 | -2.4 | -3.6552321 | -0.724 |
| JUN | ILMN_1806023 | 0.00713 | 0.000123 | -4.31 | 1.0708012 | -0.722 |
| ACTR1B | ILMN_1695821 | 0.0131 | 0.000323 | -3.98 | 0.1744893 | -0.721 |
| NKIRAS2 | ILMN_1653404 | 0.0864 | 0.00665 | -2.88 | -2.5969676 | -0.719 |
| CISD2 | ILMN_1796397 | 0.248 | 0.0443 | -2.09 | -4.2581138 | -0.719 |
| HIGD1A | ILMN_2230016 | 0.00129 | 9.22E-06 | -5.17 | 3.4974916 | -0.718 |
| SNUPN | ILMN_1733932 | 0.00189 | 1.65E-05 | -4.98 | 2.9513649 | -0.718 |
| EP400 | ILMN_1673023 | 0.0341 | 0.00142 | -3.46 | -1.1949462 | -0.718 |
| GRIPAP1 | ILMN_1761176 | 0.0873 | 0.00678 | -2.87 | -2.6145616 | -0.718 |
| RPS6KB2 | ILMN_2364357 | 0.0296 | 0.00109 | -3.55 | -0.952074 | -0.717 |
| INPP1 | ILMN_1667239 | 0.0121 | 0.000282 | -4.03 | 0.3009912 | -0.715 |
| FHOD1 | ILMN_1651776 | 0.00359 | 4.03E-05 | -4.68 | 2.1165292 | -0.714 |
| PKN1 | ILMN_2367707 | 0.0374 | 0.00163 | -3.41 | -1.3201919 | -0.714 |
| NAGK | ILMN_1716547 | 0.0442 | 0.00214 | -3.31 | -1.5703328 | -0.714 |
| COQ9 | ILMN_1756898 | 0.0842 | 0.00633 | -2.9 | -2.5527081 | -0.714 |
| SLC31A1 | ILMN_1804562 | 0.000812 | 4.03E-06 | -5.44 | 4.2732082 | -0.713 |
| CPSF2 | ILMN_2144116 | 0.0801 | 0.00578 | -2.94 | -2.4709725 | -0.713 |
| SIGLEC7 | ILMN_1681415 | 0.204 | 0.0309 | -2.25 | -3.9501176 | -0.713 |
| SS18L2 | ILMN_1796407 | 0.0086 | 0.00016 | -4.22 | 0.8262178 | -0.711 |
| PCBP3 | ILMN_1687216 | 0.0104 | 0.000222 | -4.11 | 0.5242466 | -0.711 |
| KPNA6 | ILMN_1696021 | 0.0811 | 0.00597 | -2.92 | -2.5001502 | -0.711 |
| C1orf131 | ILMN_1805474 | 0.0955 | 0.00797 | -2.81 | -2.7594494 | -0.711 |
| SREBF1 | ILMN_1663035 | 0.00401 | 5.00E-05 | -4.61 | 1.9140013 | -0.71 |
| LFNG | ILMN_2360401 | 0.0123 | 0.000291 | -4.02 | 0.2699326 | -0.71 |
| NCOR2 | ILMN_2340052 | 0.0447 | 0.00218 | -3.3 | -1.5874692 | -0.709 |
| CLPP | ILMN_1725705 | 0.157 | 0.0191 | -2.45 | -3.5343081 | -0.708 |
| CCDC71 | ILMN_1768433 | 0.233 | 0.0391 | -2.14 | -4.1532411 | -0.708 |
| PRDX5 | ILMN_2383975 | 4.01E-05 | 3.21E-08 | -7.03 | 8.798741 | -0.707 |
| BRPF3 | ILMN_1658800 | 0.00584 | 8.72E-05 | -4.42 | 1.3939953 | -0.707 |
| FBXW7 | ILMN_1754279 | 0.00281 | 2.89E-05 | -4.79 | 2.4284003 | -0.706 |
| MBD1 | ILMN_1683595 | 0.00571 | 8.33E-05 | -4.44 | 1.4369537 | -0.706 |
| MCEE | ILMN_1735347 | 0.0173 | 0.000488 | -3.84 | -0.2089893 | -0.706 |
| PGAM4 | ILMN_1706841 | 0.0122 | 0.000286 | -4.02 | 0.2872135 | -0.703 |
| ABR | ILMN_1672878 | 0.0174 | 0.000493 | -3.83 | -0.2175266 | -0.703 |
| SULF2 | ILMN_1667460 | 0.0254 | 0.000871 | -3.63 | -0.7433204 | -0.703 |
| RASAL3 | ILMN_3238803 | 0.0258 | 0.000894 | -3.63 | -0.7673625 | -0.703 |
| CXorf65 | ILMN_1661359 | 0.0601 | 0.00358 | -3.12 | -2.0386683 | -0.703 |
| RCE1 | ILMN_1685002 | 0.109 | 0.0102 | -2.71 | -2.9765382 | -0.701 |
| PLEKHO2 | ILMN_1689968 | 0.00435 | 5.61E-05 | -4.57 | 1.8059632 | -0.7 |
| SMAD6 | ILMN_1767068 | 0.00593 | 8.96E-05 | -4.42 | 1.3692966 | -0.7 |
| YIF1B | ILMN_2363668 | 0.0104 | 0.000221 | -4.11 | 0.5280363 | -0.7 |
| COL7A1 | ILMN_1751161 | 0.0116 | 0.000264 | -4.05 | 0.3630683 | -0.698 |
| SREBF1 | ILMN_2328986 | 0.0187 | 0.000554 | -3.79 | -0.3250437 | -0.698 |
| HVCN1 | ILMN_1815168 | 0.133 | 0.0143 | -2.58 | -3.28018 | -0.698 |
| EFTUD2 | ILMN_1738819 | 0.0193 | 0.000577 | -3.78 | -0.3638188 | -0.697 |
| CAMKK2 | ILMN_2367638 | 0.0429 | 0.00206 | -3.32 | -1.5338041 | -0.697 |
| UPF3B | ILMN_1798163 | 0.0254 | 0.00087 | -3.63 | -0.742286 | -0.694 |
| BRWD1 | ILMN_1801866 | 0.054 | 0.00303 | -3.18 | -1.8864376 | -0.694 |
| MRPL55 | ILMN_2348090 | 0.229 | 0.0379 | -2.16 | -4.1255969 | -0.694 |
| PGAM4 | ILMN_1691104 | 0.0169 | 0.000471 | -3.85 | -0.1746892 | -0.693 |
| EIF2B1 | ILMN_1753716 | 0.019 | 0.000568 | -3.78 | -0.3485903 | -0.693 |
| NCOR2 | ILMN_1698419 | 0.0531 | 0.00295 | -3.19 | -1.8623068 | -0.693 |
| ZC3H12C | ILMN_3237956 | 0.0874 | 0.00681 | -2.87 | -2.6187439 | -0.693 |
| SMG5 | ILMN_2126239 | 0.0925 | 0.0076 | -2.83 | -2.7169199 | -0.693 |
| APEH | ILMN_1718023 | 0.145 | 0.0166 | -2.51 | -3.4107465 | -0.693 |
| HDAC1 | ILMN_1727458 | 0.00113 | 7.32E-06 | -5.24 | 3.7145886 | -0.692 |
| ANKRD33 | ILMN_1667748 | 0.121 | 0.0121 | -2.65 | -3.1281386 | -0.692 |
| GZF1 | ILMN_1763328 | 0.19 | 0.0269 | -2.31 | -3.8309052 | -0.692 |
| LTB | ILMN_2376204 | 0.015 | 0.000395 | -3.91 | -0.0131738 | -0.691 |
| TMEM138 | ILMN_1664761 | 0.0247 | 0.000831 | -3.65 | -0.7001592 | -0.691 |
| TMC6 | ILMN_1794677 | 0.0324 | 0.00129 | -3.49 | -1.10645 | -0.691 |
| UPF3B | ILMN_2397627 | 0.0414 | 0.00194 | -3.35 | -1.4796217 | -0.691 |
| TMEM45B | ILMN_1771120 | 0.17 | 0.022 | -2.4 | -3.6570335 | -0.691 |
| NDUFAF1 | ILMN_1754421 | 0.00544 | 7.69E-05 | -4.47 | 1.5113787 | -0.69 |
| ZNRD1 | ILMN_1692486 | 0.0064 | 0.000103 | -4.37 | 1.2356193 | -0.69 |
| PIK3R6 | ILMN_3246957 | 0.0142 | 0.000363 | -3.94 | 0.0665121 | -0.69 |
| LPXN | ILMN_1742789 | 0.0326 | 0.00131 | -3.49 | -1.1179184 | -0.69 |
| CSNK1E | ILMN_1708858 | 0.061 | 0.00369 | -3.11 | -2.0660325 | -0.69 |
| HMGA1 | ILMN_2311537 | 0.204 | 0.0308 | -2.25 | -3.9475662 | -0.69 |
| LTB | ILMN_2376205 | 0.00383 | 4.57E-05 | -4.64 | 1.9980573 | -0.689 |
| UTP6 | ILMN_1778238 | 0.0356 | 0.00151 | -3.44 | -1.2477597 | -0.688 |
| HES4 | ILMN_1653466 | 0.259 | 0.0481 | -2.05 | -4.3294404 | -0.688 |
| REPIN1 | ILMN_2404385 | 0.0486 | 0.00259 | -3.24 | -1.7443334 | -0.687 |
| CYB561D1 | ILMN_3180557 | 0.0613 | 0.00372 | -3.1 | -2.073477 | -0.687 |
| KCTD13 | ILMN_1786843 | 0.148 | 0.0172 | -2.5 | -3.443634 | -0.687 |
| FAM65A | ILMN_1680037 | 0.04 | 0.00183 | -3.37 | -1.4268834 | -0.686 |
| SEC23IP | ILMN_1690690 | 0.101 | 0.00877 | -2.77 | -2.84516 | -0.686 |
| PFKL | ILMN_1713037 | 0.0142 | 0.00037 | -3.93 | 0.0492665 | -0.685 |
| KCTD5 | ILMN_1672728 | 0.0478 | 0.0025 | -3.25 | -1.7118895 | -0.685 |
| SFR1 | ILMN_1671905 | 0.245 | 0.0432 | -2.1 | -4.2378075 | -0.685 |
| TAX1BP3 | ILMN_1803392 | 0.0242 | 0.000799 | -3.66 | -0.663813 | -0.684 |
| UBTF | ILMN_1806946 | 0.0409 | 0.0019 | -3.35 | -1.4590076 | -0.684 |
| CYB561D1 | ILMN_3256325 | 0.173 | 0.0228 | -2.38 | -3.6889187 | -0.684 |
| MRPS11 | ILMN_2405915 | 0.108 | 0.00991 | -2.72 | -2.9534442 | -0.683 |
| NFE2L1 | ILMN_1739450 | 0.17 | 0.0219 | -2.4 | -3.6543372 | -0.683 |
| RASA3 | ILMN_1654586 | 0.0136 | 0.000342 | -3.96 | 0.1205138 | -0.682 |
| BOD1 | ILMN_1716730 | 0.0142 | 0.000368 | -3.94 | 0.0532599 | -0.682 |
| CTNS | ILMN_1807719 | 0.0168 | 0.000465 | -3.85 | -0.1643421 | -0.682 |
| NIF3L1 | ILMN_1777066 | 0.199 | 0.0294 | -2.27 | -3.9073497 | -0.682 |
| RFC5 | ILMN_1659364 | 0.00187 | 1.62E-05 | -4.98 | 2.9705797 | -0.681 |
| UQCR11 | ILMN_1745049 | 0.0122 | 0.000288 | -4.02 | 0.2816232 | -0.681 |
| PMS2CL | ILMN_2262198 | 0.176 | 0.0235 | -2.37 | -3.7152515 | -0.681 |
| APPL2 | ILMN_1765076 | 0.257 | 0.0472 | -2.06 | -4.3125887 | -0.681 |
| SMARCE1 | ILMN_1747857 | 0.0162 | 0.000444 | -3.87 | -0.1208689 | -0.68 |
| MKL1 | ILMN_1651767 | 0.0949 | 0.00791 | -2.81 | -2.7528235 | -0.68 |
| RSPH3 | ILMN_1788223 | 0.0955 | 0.00798 | -2.81 | -2.760449 | -0.68 |
| GPN2 | ILMN_2205245 | 0.244 | 0.0425 | -2.1 | -4.224575 | -0.678 |
| TBC1D22A | ILMN_3251227 | 0.0126 | 0.000304 | -4 | 0.2303463 | -0.676 |
| ELMO3 | ILMN_1752665 | 0.0119 | 0.000274 | -4.04 | 0.326695 | -0.675 |
| SNRNP25 | ILMN_3238712 | 0.0155 | 0.000417 | -3.89 | -0.0614088 | -0.675 |
| FAAP100 | ILMN_1711823 | 0.0376 | 0.00165 | -3.41 | -1.3287668 | -0.675 |
| ALDH3B1 | ILMN_1728662 | 0.244 | 0.0425 | -2.1 | -4.2239454 | -0.675 |
| SLC7A6OS | ILMN_1692049 | 0.0303 | 0.00114 | -3.54 | -0.9904302 | -0.674 |
| CD9 | ILMN_1695423 | 0.0693 | 0.00456 | -3.03 | -2.2577681 | -0.674 |
| G0S2 | ILMN_1691846 | 0.0864 | 0.00665 | -2.88 | -2.5966307 | -0.674 |
| FAM216A | ILMN_2180371 | 0.102 | 0.0089 | -2.77 | -2.8580896 | -0.674 |
| EPOR | ILMN_2168347 | 0.197 | 0.0287 | -2.28 | -3.8885296 | -0.674 |
| SRP68 | ILMN_1703524 | 0.0277 | 0.000989 | -3.59 | -0.8608181 | -0.673 |
| CYFIP2 | ILMN_2354478 | 0.0135 | 0.000337 | -3.97 | 0.1360649 | -0.672 |
| GPKOW | ILMN_1684197 | 0.171 | 0.0222 | -2.39 | -3.6648073 | -0.672 |
| OPA3 | ILMN_2284591 | 0.0338 | 0.00139 | -3.47 | -1.1764869 | -0.67 |
| HINFP | ILMN_2353697 | 0.00133 | 9.65E-06 | -5.15 | 3.4552814 | -0.669 |
| RNF25 | ILMN_1713486 | 0.00297 | 3.18E-05 | -4.76 | 2.3382303 | -0.669 |
| TTL | ILMN_1764043 | 0.0175 | 0.000499 | -3.83 | -0.2278627 | -0.669 |
| SLC4A7 | ILMN_2200917 | 0.0721 | 0.00484 | -3 | -2.3103655 | -0.669 |
| RRP7A | ILMN_1688178 | 0.00924 | 0.00018 | -4.18 | 0.7171328 | -0.668 |
| TBC1D10C | ILMN_1710434 | 0.0795 | 0.0057 | -2.94 | -2.4581375 | -0.668 |
| AK2 | ILMN_1655645 | 0.0797 | 0.00574 | -2.94 | -2.4641347 | -0.668 |
| NIFK | ILMN_1786189 | 0.00631 | 1.00E-04 | -4.38 | 1.2648675 | -0.667 |
| ZCCHC3 | ILMN_1786852 | 0.00593 | 8.96E-05 | -4.42 | 1.3689424 | -0.665 |
| PAGR1 | ILMN_2194828 | 0.114 | 0.0111 | -2.68 | -3.0550402 | -0.665 |
| BRWD1 | ILMN_1673518 | 0.0398 | 0.00181 | -3.37 | -1.4150575 | -0.663 |
| DIABLO | ILMN_2332990 | 0.0726 | 0.0049 | -3 | -2.3213987 | -0.663 |
| SIN3A | ILMN_1805996 | 0.138 | 0.0152 | -2.55 | -3.3319853 | -0.663 |
| AKAP8L | ILMN_1768962 | 0.0793 | 0.00566 | -2.94 | -2.4522623 | -0.662 |
| RPS19BP1 | ILMN_2177965 | 0.0866 | 0.0067 | -2.88 | -2.6029635 | -0.662 |
| TFAM | ILMN_1715661 | 0.16 | 0.0196 | -2.44 | -3.558003 | -0.662 |
| MYNN | ILMN_1672287 | 0.185 | 0.0256 | -2.33 | -3.7886647 | -0.662 |
| TNFRSF9 | ILMN_1813379 | 0.109 | 0.0101 | -2.72 | -2.9728153 | -0.661 |
| PLEKHO1 | ILMN_1694213 | 0.0356 | 0.00151 | -3.44 | -1.2511527 | -0.66 |
| ARAP3 | ILMN_1812618 | 0.00639 | 0.000103 | -4.37 | 1.2423422 | -0.659 |
| CYC1 | ILMN_1815115 | 0.0619 | 0.00381 | -3.1 | -2.0931651 | -0.659 |
| METTL13 | ILMN_2343624 | 0.0902 | 0.00719 | -2.85 | -2.6674593 | -0.659 |
| DDX46 | ILMN_1727001 | 0.0978 | 0.00827 | -2.8 | -2.7926358 | -0.659 |
| ATG4A | ILMN_3307158 | 0.00057 | 2.22E-06 | -5.63 | 4.8318082 | -0.657 |
| DYNLT3 | ILMN_1681890 | 0.249 | 0.0445 | -2.08 | -4.2636927 | -0.657 |
| MRPL49 | ILMN_1681324 | 0.0245 | 0.000817 | -3.66 | -0.684609 | -0.656 |
| NSF | ILMN_2330845 | 0.0552 | 0.00314 | -3.17 | -1.9189084 | -0.655 |
| SYK | ILMN_2059549 | 0.0211 | 0.000662 | -3.73 | -0.4901686 | -0.654 |
| VEGFA | ILMN_2375879 | 0.0552 | 0.00314 | -3.17 | -1.9187332 | -0.654 |
| SULF2 | ILMN_1686981 | 0.083 | 0.00618 | -2.91 | -2.5313626 | -0.654 |
| PGAM4 | ILMN_1682953 | 0.0844 | 0.00635 | -2.9 | -2.5560635 | -0.654 |
| CBLN3 | ILMN_2053829 | 0.0998 | 0.00856 | -2.78 | -2.8226809 | -0.654 |
| IL18BP | ILMN_2334296 | 0.0847 | 0.0064 | -2.9 | -2.5627848 | -0.653 |
| EIF2B5 | ILMN_1688534 | 0.00627 | 9.83E-05 | -4.38 | 1.2823435 | -0.652 |
| SDCBP2 | ILMN_1705107 | 0.0405 | 0.00186 | -3.36 | -1.4423074 | -0.652 |
| NECAP2 | ILMN_1749011 | 0.185 | 0.0256 | -2.33 | -3.7885654 | -0.652 |
| COQ8A | ILMN_1731064 | 0.248 | 0.0443 | -2.09 | -4.2585555 | -0.652 |
| TNFRSF14 | ILMN_1697409 | 0.0509 | 0.00276 | -3.22 | -1.8019757 | -0.651 |
| AP3M1 | ILMN_2362122 | 0.129 | 0.0136 | -2.6 | -3.2340465 | -0.651 |
| RNF185 | ILMN_1691293 | 0.0229 | 0.000739 | -3.69 | -0.5919729 | -0.65 |
| DEF8 | ILMN_1767509 | 0.14 | 0.0156 | -2.54 | -3.3579482 | -0.649 |
| SEC13 | ILMN_3223181 | 1.80E-05 | 9.43E-09 | -7.44 | 9.9380007 | -0.648 |
| BRPF1 | ILMN_2365549 | 0.0242 | 0.000799 | -3.66 | -0.6642136 | -0.648 |
| HMBOX1 | ILMN_1843949 | 0.0388 | 0.00173 | -3.39 | -1.3733611 | -0.648 |
| PEX16 | ILMN_1701466 | 0.236 | 0.04 | -2.13 | -4.1719265 | -0.648 |
| IRAK2 | ILMN_1745964 | 0.243 | 0.0421 | -2.11 | -4.2149425 | -0.648 |
| SEC13 | ILMN_3297880 | 0.000132 | 1.89E-07 | -6.44 | 7.1405695 | -0.647 |
| PTGES2 | ILMN_2345015 | 0.00227 | 2.11E-05 | -4.9 | 2.7204163 | -0.647 |
| C5orf15 | ILMN_1695917 | 0.00807 | 0.000144 | -4.26 | 0.9237449 | -0.647 |
| COPS4 | ILMN_1726755 | 0.0237 | 0.000773 | -3.68 | -0.6327999 | -0.647 |
| SERPINB6 | ILMN_1712400 | 0.0595 | 0.00353 | -3.12 | -2.0238237 | -0.647 |
| ENO3 | ILMN_1678904 | 0.112 | 0.0106 | -2.7 | -3.016924 | -0.647 |
| TNFRSF10B | ILMN_1699265 | 0.126 | 0.013 | -2.61 | -3.1971224 | -0.647 |
| PRDX5 | ILMN_1711606 | 7.22E-05 | 8.16E-08 | -6.72 | 7.9277906 | -0.646 |
| DDB1 | ILMN_1775937 | 0.144 | 0.0163 | -2.52 | -3.3967328 | -0.646 |
| ZNF627 | ILMN_1708787 | 0.198 | 0.0292 | -2.27 | -3.9027543 | -0.646 |
| ZNF318 | ILMN_1792305 | 0.0474 | 0.00247 | -3.26 | -1.6983254 | -0.645 |
| SUPT16H | ILMN_1781516 | 0.0828 | 0.00616 | -2.91 | -2.5276385 | -0.645 |
| TIMM22 | ILMN_1706959 | 0.0935 | 0.00773 | -2.82 | -2.7317849 | -0.645 |
| WASH7P | ILMN_3239735 | 0.0576 | 0.00336 | -3.14 | -1.9806792 | -0.644 |
| MRPL53 | ILMN_1813682 | 0.0786 | 0.00556 | -2.95 | -2.4363006 | -0.644 |
| ANGEL2 | ILMN_1736340 | 0.235 | 0.0398 | -2.13 | -4.1672761 | -0.644 |
| POLR3F | ILMN_1673966 | 0.00752 | 0.000133 | -4.28 | 1.0020058 | -0.643 |
| ORAI2 | ILMN_1652928 | 0.234 | 0.0395 | -2.14 | -4.1608561 | -0.643 |
| EVA1B | ILMN_1769092 | 0.00367 | 4.13E-05 | -4.67 | 2.0933796 | -0.642 |
| APPBP2 | ILMN_2149766 | 0.24 | 0.0413 | -2.12 | -4.2005634 | -0.641 |
| SPSB2 | ILMN_1787541 | 0.0509 | 0.00277 | -3.22 | -1.803025 | -0.639 |
| MUL1 | ILMN_1675055 | 0.104 | 0.00927 | -2.75 | -2.894006 | -0.639 |
| FARP2 | ILMN_2062687 | 0.141 | 0.0158 | -2.53 | -3.3697344 | -0.639 |
| PCGF1 | ILMN_1757956 | 0.0115 | 0.000257 | -4.06 | 0.3869667 | -0.638 |
| CHERP | ILMN_1798083 | 0.0192 | 0.000575 | -3.78 | -0.3602461 | -0.637 |
| TTC32 | ILMN_2083588 | 0.117 | 0.0114 | -2.67 | -3.0789 | -0.637 |
| CSTB | ILMN_1761797 | 0.0352 | 0.00148 | -3.44 | -1.2337001 | -0.636 |
| ARL16 | ILMN_1679917 | 0.0382 | 0.0017 | -3.4 | -1.356605 | -0.636 |
| MKKS | ILMN_1718718 | 0.0924 | 0.0075 | -2.83 | -2.7054218 | -0.636 |
| DOPEY2 | ILMN_1741711 | 0.0949 | 0.00791 | -2.81 | -2.7528137 | -0.636 |
| CHMP3 | ILMN_2406043 | 0.101 | 0.00883 | -2.77 | -2.851082 | -0.636 |
| MGAT2 | ILMN_2342240 | 0.186 | 0.026 | -2.32 | -3.8028374 | -0.635 |
| NUP62 | ILMN_2323491 | 0.211 | 0.0327 | -2.22 | -4.000532 | -0.635 |
| SAMD4B | ILMN_1753467 | 0.00572 | 8.38E-05 | -4.44 | 1.4313412 | -0.634 |
| PGRMC2 | ILMN_2195236 | 0.148 | 0.0172 | -2.5 | -3.4427791 | -0.634 |
| KISS1R | ILMN_1673521 | 0.248 | 0.0443 | -2.08 | -4.259652 | -0.634 |
| PI4KB | ILMN_1666597 | 0.0092 | 0.000178 | -4.18 | 0.7282112 | -0.633 |
| ELOVL1 | ILMN_1798123 | 0.0537 | 0.003 | -3.19 | -1.8776026 | -0.633 |
| CTCF | ILMN_1786015 | 0.0649 | 0.00411 | -3.07 | -2.1616469 | -0.633 |
| MANBAL | ILMN_1673944 | 0.191 | 0.0273 | -2.3 | -3.8432129 | -0.633 |
| WDR61 | ILMN_1665887 | 0.0237 | 0.000774 | -3.68 | -0.6348917 | -0.632 |
| SEC61A1 | ILMN_1659564 | 0.194 | 0.0281 | -2.29 | -3.8691939 | -0.632 |
| CKAP2L | ILMN_1751776 | 0.0566 | 0.00327 | -3.15 | -1.9561445 | -0.631 |
| ILKAP | ILMN_1684647 | 0.132 | 0.014 | -2.58 | -3.2634133 | -0.631 |
| AMPD3 | ILMN_1731223 | 0.0369 | 0.00158 | -3.42 | -1.2935708 | -0.63 |
| NELFB | ILMN_1742432 | 0.0481 | 0.00253 | -3.25 | -1.7206528 | -0.63 |
| SENP5 | ILMN_3251634 | 0.169 | 0.0217 | -2.4 | -3.6448099 | -0.63 |
| C16orf54 | ILMN_1751061 | 0.0473 | 0.00246 | -3.26 | -1.69462 | -0.629 |
| PRDM2 | ILMN_1779825 | 0.00668 | 0.000109 | -4.35 | 1.1869874 | -0.628 |
| CCNG2 | ILMN_1879480 | 0.173 | 0.0227 | -2.38 | -3.6846214 | -0.628 |
| PIK3R2 | ILMN_1726565 | 0.132 | 0.0141 | -2.58 | -3.2659581 | -0.627 |
| GTF2E1 | ILMN_1655921 | 0.173 | 0.0228 | -2.38 | -3.6896067 | -0.626 |
| SOCS4 | ILMN_2413259 | 0.177 | 0.0239 | -2.36 | -3.7278062 | -0.626 |
| DNAJC7 | ILMN_1663616 | 0.0743 | 0.00509 | -2.99 | -2.3568437 | -0.625 |
| LYRM7 | ILMN_1709936 | 0.0747 | 0.00515 | -2.98 | -2.3669277 | -0.625 |
| VEGFA | ILMN_1803882 | 0.0971 | 0.00816 | -2.8 | -2.7805387 | -0.625 |
| PKM | ILMN_2366634 | 0.00126 | 8.93E-06 | -5.18 | 3.5281868 | -0.624 |
| PEX2 | ILMN_1743299 | 0.15 | 0.0177 | -2.49 | -3.4682433 | -0.624 |
| PTPN22 | ILMN_1695640 | 0.0821 | 0.00607 | -2.92 | -2.5148383 | -0.623 |
| NFKBIB | ILMN_1674152 | 0.106 | 0.00956 | -2.74 | -2.9220574 | -0.623 |
| CTRL | ILMN_1664863 | 0.174 | 0.0229 | -2.38 | -3.693783 | -0.623 |
| GGA3 | ILMN_2402972 | 0.156 | 0.0187 | -2.46 | -3.5170249 | -0.622 |
| TMEM222 | ILMN_1793632 | 0.0342 | 0.00143 | -3.46 | -1.1985033 | -0.621 |
| SEC24A | ILMN_2126832 | 0.0475 | 0.00247 | -3.26 | -1.7011361 | -0.621 |
| ZNF317 | ILMN_1680347 | 0.0524 | 0.00289 | -3.2 | -1.84313 | -0.621 |
| LRRC47 | ILMN_1668484 | 0.173 | 0.0229 | -2.38 | -3.6907084 | -0.621 |
| DPM1 | ILMN_2105308 | 0.224 | 0.0362 | -2.18 | -4.0875254 | -0.619 |
| OPRL1 | ILMN_2400926 | 0.0105 | 0.000228 | -4.1 | 0.4975132 | -0.618 |
| LINC00938 | ILMN_1780773 | 0.0885 | 0.00694 | -2.87 | -2.6356646 | -0.618 |
| DBNL | ILMN_1698307 | 0.182 | 0.025 | -2.34 | -3.767603 | -0.618 |
| SLC24A3 | ILMN_1663519 | 0.0566 | 0.00327 | -3.15 | -1.9539427 | -0.617 |
| MFSD5 | ILMN_1702065 | 0.000943 | 5.33E-06 | -5.35 | 4.012132 | -0.616 |
| CNOT9 | ILMN_2044085 | 0.0176 | 5.00E-04 | -3.83 | -0.2311695 | -0.616 |
| FBXW7 | ILMN_1668634 | 0.00377 | 4.44E-05 | -4.65 | 2.0244249 | -0.615 |
| NISCH | ILMN_1735827 | 0.016 | 0.000436 | -3.88 | -0.1043673 | -0.615 |
| NPAT | ILMN_2120965 | 0.0244 | 0.000809 | -3.66 | -0.6752289 | -0.614 |
| CAMKK2 | ILMN_1743021 | 0.0465 | 0.00237 | -3.27 | -1.6631372 | -0.614 |
| HELQ | ILMN_1720440 | 0.0571 | 0.00332 | -3.15 | -1.9690947 | -0.614 |
| RNF41 | ILMN_1808095 | 0.0885 | 0.00697 | -2.86 | -2.6389457 | -0.614 |
| PADI2 | ILMN_1771223 | 0.256 | 0.047 | -2.06 | -4.3094914 | -0.614 |
| ORC2 | ILMN_2061452 | 0.0305 | 0.00115 | -3.53 | -1.0016308 | -0.613 |
| IDH3G | ILMN_1802706 | 0.0484 | 0.00257 | -3.24 | -1.7366186 | -0.613 |
| QTRT2 | ILMN_2155516 | 0.0582 | 0.00342 | -3.14 | -1.9949965 | -0.613 |
| OPRL1 | ILMN_2400922 | 0.0171 | 0.000478 | -3.84 | -0.1885332 | -0.612 |
| ARHGAP4 | ILMN_1770824 | 0.0182 | 0.000525 | -3.81 | -0.2761324 | -0.612 |
| CATSPER1 | ILMN_1789394 | 0.121 | 0.012 | -2.65 | -3.1260095 | -0.612 |
| MFSD12 | ILMN_1813374 | 0.000985 | 5.96E-06 | -5.31 | 3.9078224 | -0.61 |
| MLF2 | ILMN_1671885 | 0.00401 | 4.99E-05 | -4.61 | 1.9151746 | -0.61 |
| ZNRD1 | ILMN_2398587 | 0.0115 | 0.000259 | -4.06 | 0.3806023 | -0.61 |
| DBNL | ILMN_2376289 | 0.177 | 0.0237 | -2.36 | -3.7234155 | -0.61 |
| RBM42 | ILMN_1670456 | 0.0234 | 0.000761 | -3.68 | -0.6187727 | -0.609 |
| IRAK1 | ILMN_2379130 | 0.0326 | 0.00131 | -3.49 | -1.1220998 | -0.609 |
| CAMK1 | ILMN_2140990 | 0.0784 | 0.00553 | -2.95 | -2.4301668 | -0.609 |
| BLCAP | ILMN_1675612 | 0.185 | 0.0256 | -2.33 | -3.7888851 | -0.609 |
| FASTK | ILMN_2407464 | 0.0662 | 0.00426 | -3.05 | -2.1961448 | -0.608 |
| THUMPD3 | ILMN_1671902 | 0.0726 | 0.00491 | -3 | -2.3230189 | -0.608 |
| RAD9A | ILMN_1684183 | 0.101 | 0.00872 | -2.78 | -2.8399818 | -0.608 |
| SLC25A11 | ILMN_1664168 | 0.0126 | 0.000308 | -4 | 0.2198 | -0.607 |
| NAT1 | ILMN_1743055 | 0.122 | 0.0123 | -2.64 | -3.147404 | -0.607 |
| C17orf58 | ILMN_1712985 | 0.242 | 0.0419 | -2.11 | -4.2112976 | -0.607 |
| MGAT4B | ILMN_1796106 | 0.0332 | 0.00135 | -3.48 | -1.1464754 | -0.606 |
| DIABLO | ILMN_2310589 | 0.0123 | 0.000291 | -4.02 | 0.2698851 | -0.605 |
| SNRPG | ILMN_1683562 | 0.0182 | 0.000528 | -3.81 | -0.281731 | -0.605 |
| UBE2E1 | ILMN_1806778 | 0.00537 | 7.41E-05 | -4.48 | 1.5460056 | -0.604 |
| NGDN | ILMN_2324998 | 0.0971 | 0.00817 | -2.8 | -2.7819161 | -0.604 |
| RNF14 | ILMN_1714461 | 0.256 | 0.0468 | -2.06 | -4.3057549 | -0.604 |
| C6orf223 | ILMN_3248091 | 0.00399 | 4.90E-05 | -4.62 | 1.9319678 | -0.603 |
| HCG26 | ILMN_3236741 | 0.056 | 0.00321 | -3.16 | -1.9388395 | -0.603 |
| RELB | ILMN_1811258 | 0.0614 | 0.00374 | -3.1 | -2.0775681 | -0.603 |
| ZNF146 | ILMN_2054442 | 0.0873 | 0.00678 | -2.87 | -2.6142386 | -0.603 |
| TSPAN3 | ILMN_1790549 | 0.176 | 0.0236 | -2.36 | -3.7197871 | -0.603 |
| ENG | ILMN_1760778 | 0.25 | 0.0449 | -2.08 | -4.271179 | -0.603 |
| NBN | ILMN_1734833 | 0.258 | 0.0475 | -2.05 | -4.3183315 | -0.603 |
| TTC17 | ILMN_1660810 | 0.00533 | 7.30E-05 | -4.48 | 1.559625 | -0.602 |
| YAF2 | ILMN_1765606 | 0.064 | 0.00402 | -3.08 | -2.1419715 | -0.602 |
| C6orf136 | ILMN_1813236 | 0.184 | 0.0254 | -2.33 | -3.7817876 | -0.602 |
| ZNF212 | ILMN_1684964 | 0.198 | 0.0291 | -2.27 | -3.8992599 | -0.602 |
| KCTD11 | ILMN_1777513 | 0.000922 | 4.86E-06 | -5.38 | 4.0979332 | -0.601 |
| HIGD1A | ILMN_1661799 | 0.0023 | 2.22E-05 | -4.88 | 2.6747685 | -0.601 |
| ZNF816 | ILMN_1728710 | 0.0211 | 0.00066 | -3.73 | -0.4879702 | -0.6 |
| ARAP3 | ILMN_3240997 | 0.0282 | 0.00102 | -3.58 | -0.890432 | -0.6 |
| RNF166 | ILMN_1695356 | 0.064 | 0.00402 | -3.08 | -2.1436299 | -0.6 |
| TIGAR | ILMN_1791792 | 0.0879 | 0.00689 | -2.87 | -2.6286863 | -0.6 |
| TICAM2 | ILMN_1651346 | 0.0946 | 0.00787 | -2.82 | -2.7476427 | -0.6 |
| PPM1F | ILMN_2059535 | 0.112 | 0.0107 | -2.69 | -3.0252917 | -0.6 |
| VPS72 | ILMN_1722774 | 0.037 | 0.0016 | -3.42 | -1.3001328 | -0.599 |
| KCTD12 | ILMN_1742332 | 0.0916 | 0.0074 | -2.84 | -2.6926966 | -0.599 |
| PAFAH2 | ILMN_1682919 | 0.0478 | 0.0025 | -3.25 | -1.7113231 | -0.598 |
| CCDC97 | ILMN_1801600 | 0.112 | 0.0108 | -2.69 | -3.0271396 | -0.598 |
| WDR41 | ILMN_1778488 | 0.22 | 0.0351 | -2.19 | -4.0605927 | -0.598 |
| CDC40 | ILMN_1737651 | 0.244 | 0.0427 | -2.1 | -4.2284183 | -0.598 |
| CSE1L | ILMN_1706238 | 0.0902 | 0.0072 | -2.85 | -2.6682004 | -0.596 |
| TMEM208 | ILMN_1700419 | 0.198 | 0.0292 | -2.27 | -3.9029054 | -0.596 |
| LSM1 | ILMN_2218450 | 0.0348 | 0.00146 | -3.45 | -1.221736 | -0.595 |
| ZFYVE26 | ILMN_1798061 | 0.102 | 0.00893 | -2.77 | -2.8605336 | -0.595 |
| RPP21 | ILMN_2186597 | 0.00123 | 8.60E-06 | -5.19 | 3.5634373 | -0.594 |
| PICK1 | ILMN_2373982 | 0.0198 | 0.000605 | -3.76 | -0.4068695 | -0.594 |
| ACAD9 | ILMN_1775579 | 0.0202 | 0.000619 | -3.75 | -0.4277117 | -0.594 |
| OAZ2 | ILMN_3239460 | 0.0486 | 0.00259 | -3.24 | -1.7440227 | -0.594 |
| NUP37 | ILMN_1771903 | 0.0765 | 0.00531 | -2.97 | -2.3943943 | -0.594 |
| VEZF1 | ILMN_1705310 | 0.0972 | 0.0082 | -2.8 | -2.78499 | -0.594 |
| PTAR1 | ILMN_3237679 | 0.126 | 0.0131 | -2.61 | -3.1987942 | -0.594 |
| PAFAH1B1 | ILMN_1722276 | 0.2 | 0.0297 | -2.26 | -3.9176986 | -0.594 |
| BCOR | ILMN_1773117 | 0.00557 | 8.08E-05 | -4.45 | 1.4651667 | -0.593 |
| TMEM205 | ILMN_1730734 | 0.079 | 0.00563 | -2.95 | -2.446726 | -0.593 |
| GTF3C2 | ILMN_2356574 | 0.186 | 0.0259 | -2.32 | -3.8000421 | -0.593 |
| EDRF1 | ILMN_1791656 | 0.19 | 0.0269 | -2.31 | -3.8303295 | -0.593 |
| DENND4B | ILMN_3245066 | 0.0092 | 0.000178 | -4.18 | 0.7263843 | -0.592 |
| DDX52 | ILMN_2366790 | 0.0467 | 0.0024 | -3.27 | -1.6742905 | -0.592 |
| YIPF5 | ILMN_1714756 | 0.0797 | 0.00573 | -2.94 | -2.4633604 | -0.592 |
| NARS | ILMN_1732216 | 0.227 | 0.0374 | -2.16 | -4.11479 | -0.592 |
| SLC4A1AP | ILMN_1750876 | 0.0229 | 0.00074 | -3.69 | -0.593023 | -0.591 |
| CEBPE | ILMN_1779095 | 0.065 | 0.00413 | -3.06 | -2.1682302 | -0.591 |
| BUD13 | ILMN_1749210 | 0.0458 | 0.00229 | -3.29 | -1.6297641 | -0.59 |
| SMIM7 | ILMN_1694759 | 0.0511 | 0.00278 | -3.21 | -1.8072616 | -0.59 |
| TMC8 | ILMN_2211534 | 0.127 | 0.0131 | -2.61 | -3.2021433 | -0.59 |
| CXorf40B | ILMN_2143261 | 0.0331 | 0.00134 | -3.48 | -1.1396869 | -0.589 |
| CEACAM3 | ILMN_1743570 | 0.0974 | 0.00823 | -2.8 | -2.7875323 | -0.589 |
| PPP1CA | ILMN_2377980 | 0.144 | 0.0164 | -2.52 | -3.4001421 | -0.589 |
| SASH3 | ILMN_1697554 | 0.000235 | 5.21E-07 | -6.11 | 6.1929259 | -0.588 |
| RWDD4 | ILMN_1708660 | 0.108 | 0.0098 | -2.73 | -2.9435212 | -0.588 |
| PANK4 | ILMN_1743910 | 0.124 | 0.0126 | -2.63 | -3.1675682 | -0.588 |
| TST | ILMN_1691572 | 0.168 | 0.0215 | -2.4 | -3.6386067 | -0.588 |
| PPHLN1 | ILMN_2322842 | 0.195 | 0.0283 | -2.29 | -3.8758856 | -0.588 |
| TNK2 | ILMN_1669703 | 0.00203 | 1.81E-05 | -4.95 | 2.8631761 | -0.587 |
| IRF5 | ILMN_2312606 | 0.214 | 0.0336 | -2.21 | -4.0229652 | -0.587 |
| CHCHD1 | ILMN_1672149 | 0.0606 | 0.00364 | -3.11 | -2.0517733 | -0.586 |
| PFDN6 | ILMN_1661490 | 0.0697 | 0.00462 | -3.02 | -2.2689126 | -0.586 |
| CTSD | ILMN_1674038 | 0.186 | 0.0261 | -2.32 | -3.8055123 | -0.586 |
| TIA1 | ILMN_2388466 | 0.264 | 0.0497 | -2.03 | -4.3569008 | -0.586 |
| ICAM1 | ILMN_1812226 | 0.107 | 0.00973 | -2.73 | -2.9373078 | -0.585 |
| L3MBTL2 | ILMN_2336109 | 0.0248 | 0.000839 | -3.65 | -0.7086825 | -0.584 |
| FUT4 | ILMN_1792072 | 0.0696 | 0.00461 | -3.02 | -2.2675592 | -0.584 |
| TMEM52B | ILMN_1727606 | 0.0801 | 0.0058 | -2.94 | -2.4744891 | -0.584 |
| SLC37A1 | ILMN_1687495 | 0.22 | 0.0352 | -2.19 | -4.0622211 | -0.584 |
| CD82 | ILMN_2392274 | 0.00576 | 8.49E-05 | -4.43 | 1.4192968 | -0.583 |
| NMD3 | ILMN_1727348 | 0.0507 | 0.00273 | -3.22 | -1.7922254 | -0.583 |
| RSPRY1 | ILMN_1763694 | 0.114 | 0.0111 | -2.68 | -3.0515402 | -0.583 |
| TRIM26 | ILMN_1738704 | 0.00816 | 0.000146 | -4.25 | 0.9106442 | -0.582 |
| EHBP1L1 | ILMN_3244457 | 0.0481 | 0.00253 | -3.25 | -1.7213069 | -0.582 |
| PITPNB | ILMN_1809245 | 0.0297 | 0.00111 | -3.55 | -0.9657651 | -0.581 |
| HSPA14 | ILMN_1797318 | 0.0414 | 0.00195 | -3.35 | -1.48177 | -0.581 |
| RASAL3 | ILMN_1795089 | 0.0454 | 0.00225 | -3.29 | -1.6136401 | -0.581 |
| KIF23 | ILMN_1811472 | 0.0767 | 0.00534 | -2.97 | -2.3992259 | -0.581 |
| ATF6B | ILMN_3194911 | 0.19 | 0.0268 | -2.31 | -3.8297025 | -0.581 |
| STRADA | ILMN_2315694 | 0.2 | 0.0298 | -2.26 | -3.9196863 | -0.581 |
| DDX49 | ILMN_1762225 | 0.0407 | 0.00188 | -3.36 | -1.4495095 | -0.58 |
| ZDHHC13 | ILMN_1785831 | 0.0958 | 0.00801 | -2.81 | -2.7641801 | -0.58 |
| NEMP1 | ILMN_3228822 | 0.149 | 0.0175 | -2.49 | -3.4568223 | -0.58 |
| FAM110A | ILMN_2323944 | 0.0798 | 0.00575 | -2.94 | -2.466029 | -0.579 |
| SRSF5 | ILMN_2378868 | 0.212 | 0.0329 | -2.22 | -4.0045343 | -0.579 |
| MFN1 | ILMN_1664186 | 0.177 | 0.0239 | -2.36 | -3.7307712 | -0.578 |
| COPS5 | ILMN_1736002 | 0.256 | 0.047 | -2.06 | -4.3097335 | -0.578 |
| NFKB1 | ILMN_1714965 | 0.00383 | 4.62E-05 | -4.64 | 1.9879998 | -0.577 |
| MEA1 | ILMN_1727073 | 0.0342 | 0.00143 | -3.46 | -1.2018305 | -0.577 |
| TYK2 | ILMN_1676955 | 0.0484 | 0.00255 | -3.25 | -1.7302156 | -0.577 |
| KIAA0319 | ILMN_1657497 | 0.0902 | 0.00722 | -2.85 | -2.670914 | -0.577 |
| MIB2 | ILMN_2385178 | 0.101 | 0.0088 | -2.77 | -2.8481093 | -0.577 |
| ZKSCAN4 | ILMN_1804571 | 0.102 | 0.00894 | -2.77 | -2.8622873 | -0.577 |
| UBE2Z | ILMN_1783771 | 0.14 | 0.0156 | -2.54 | -3.3567499 | -0.577 |
| PTGS2 | ILMN_2054297 | 0.143 | 0.0162 | -2.52 | -3.3888115 | -0.577 |
| CSRNP2 | ILMN_2187533 | 0.0438 | 0.00212 | -3.31 | -1.5589058 | -0.576 |
| TRNT1 | ILMN_1655924 | 0.14 | 0.0156 | -2.54 | -3.3546161 | -0.576 |
| NMRAL1 | ILMN_1709814 | 0.16 | 0.0195 | -2.45 | -3.5540856 | -0.576 |
| AP5M1 | ILMN_1741331 | 0.192 | 0.0275 | -2.3 | -3.850545 | -0.576 |
| SEPT9 | ILMN_1769118 | 0.0104 | 0.000222 | -4.11 | 0.5244064 | -0.575 |
| CSNK1E | ILMN_2415235 | 0.0169 | 0.000471 | -3.85 | -0.1746409 | -0.574 |
| LRRC25 | ILMN_2150196 | 0.0255 | 0.000876 | -3.63 | -0.7484091 | -0.574 |
| RAB11FIP1 | ILMN_1692219 | 0.043 | 0.00207 | -3.32 | -1.5380442 | -0.574 |
| ZNF484 | ILMN_1683854 | 0.0851 | 0.00646 | -2.89 | -2.5704693 | -0.574 |
| MRPL14 | ILMN_2072603 | 0.00377 | 4.45E-05 | -4.65 | 2.0222787 | -0.573 |
| HADHA | ILMN_1712751 | 0.0319 | 0.00125 | -3.51 | -1.0742594 | -0.573 |
| TIMM21 | ILMN_2182531 | 0.0742 | 0.00508 | -2.99 | -2.3542172 | -0.573 |
| DNASE2 | ILMN_1796245 | 0.0937 | 0.00776 | -2.82 | -2.7349169 | -0.573 |
| TIA1 | ILMN_1778691 | 0.0995 | 0.00851 | -2.79 | -2.817461 | -0.573 |
| EMC6 | ILMN_1758674 | 0.107 | 0.00964 | -2.74 | -2.9291555 | -0.573 |
| FAR1 | ILMN_2143250 | 0.163 | 0.0203 | -2.43 | -3.5872921 | -0.573 |
| SIVA1 | ILMN_1696046 | 0.213 | 0.0332 | -2.22 | -4.0139553 | -0.573 |
| MANBAL | ILMN_2395926 | 0.244 | 0.0426 | -2.1 | -4.2268259 | -0.573 |
| OSCAR | ILMN_1741917 | 0.0937 | 0.00776 | -2.82 | -2.7349429 | -0.572 |
| CD58 | ILMN_2147517 | 0.0208 | 0.000644 | -3.74 | -0.464044 | -0.571 |
| CSE1L | ILMN_1665797 | 0.0567 | 0.00329 | -3.15 | -1.959585 | -0.571 |
| MCM3AP | ILMN_1784766 | 0.0465 | 0.00237 | -3.27 | -1.6624637 | -0.57 |
| DMXL2 | ILMN_1705663 | 0.186 | 0.026 | -2.32 | -3.8035924 | -0.57 |
| TBC1D10A | ILMN_1693726 | 0.0151 | 4.00E-04 | -3.91 | -0.0232075 | -0.569 |
| UQCRC2 | ILMN_1718853 | 0.0227 | 0.000727 | -3.7 | -0.5769133 | -0.569 |
| TMEM208 | ILMN_3239055 | 0.149 | 0.0174 | -2.49 | -3.4539625 | -0.569 |
| AP5M1 | ILMN_2204297 | 0.157 | 0.0191 | -2.46 | -3.5326118 | -0.569 |
| FAM103A1 | ILMN_2151441 | 0.256 | 0.0467 | -2.06 | -4.3032117 | -0.569 |
| BTG2 | ILMN_1770085 | 0.0639 | 0.004 | -3.08 | -2.1370873 | -0.568 |
| PSMA5 | ILMN_1759952 | 0.112 | 0.0107 | -2.69 | -3.0247409 | -0.568 |
| EHD4 | ILMN_1720083 | 0.145 | 0.0166 | -2.51 | -3.4117956 | -0.568 |
| PSMA2 | ILMN_2058512 | 0.16 | 0.0196 | -2.44 | -3.5573122 | -0.568 |
| STT3A | ILMN_1746090 | 0.216 | 0.0339 | -2.21 | -4.0306832 | -0.568 |
| SLC16A5 | ILMN_1803720 | 0.101 | 0.00874 | -2.77 | -2.8415697 | -0.567 |
| MRPL20 | ILMN_2189424 | 0.148 | 0.0172 | -2.5 | -3.4415674 | -0.567 |
| PPP2R1A | ILMN_1810467 | 0.056 | 0.00321 | -3.16 | -1.9380749 | -0.566 |
| CCDC94 | ILMN_1703430 | 0.055 | 0.00312 | -3.17 | -1.9113753 | -0.565 |
| BCL11A | ILMN_2255133 | 0.0998 | 0.00857 | -2.78 | -2.8239343 | -0.565 |
| WASH3P | ILMN_1739199 | 0.112 | 0.0107 | -2.7 | -3.0183768 | -0.564 |
| TBC1D9B | ILMN_1789909 | 0.0125 | 0.000297 | -4.01 | 0.253398 | -0.563 |
| NGDN | ILMN_1690049 | 0.0515 | 0.00282 | -3.21 | -1.8201522 | -0.562 |
| TM9SF4 | ILMN_1674421 | 0.0515 | 0.00283 | -3.21 | -1.822199 | -0.562 |
| UNC13D | ILMN_1773380 | 0.0549 | 0.00311 | -3.17 | -1.9089889 | -0.562 |
| SEC22C | ILMN_2290618 | 0.0726 | 0.0049 | -3 | -2.3212898 | -0.562 |
| NUMA1 | ILMN_2160005 | 0.102 | 0.00887 | -2.77 | -2.8545705 | -0.562 |
| MRPL50 | ILMN_1664833 | 0.19 | 0.0271 | -2.31 | -3.8377588 | -0.562 |
| RGL4 | ILMN_1663422 | 0.192 | 0.0274 | -2.3 | -3.8490915 | -0.562 |
| CDKN2C | ILMN_1656415 | 0.202 | 0.0302 | -2.26 | -3.9322352 | -0.562 |
| SEL1L | ILMN_1726496 | 0.0125 | 3.00E-04 | -4.01 | 0.2436976 | -0.561 |
| MEPCE | ILMN_2180827 | 0.0896 | 0.0071 | -2.86 | -2.6561363 | -0.561 |
| NUP85 | ILMN_1669635 | 0.0921 | 0.00746 | -2.84 | -2.7005693 | -0.561 |
| SPATA5L1 | ILMN_1729179 | 0.171 | 0.0222 | -2.39 | -3.6644125 | -0.561 |
| MRPL17 | ILMN_1797933 | 0.2 | 0.0299 | -2.26 | -3.9219866 | -0.56 |
| TRPT1 | ILMN_2364674 | 0.212 | 0.033 | -2.22 | -4.0074607 | -0.56 |
| ARID2 | ILMN_2182335 | 0.0525 | 0.00291 | -3.2 | -1.8483816 | -0.559 |
| NOA1 | ILMN_1665066 | 0.124 | 0.0126 | -2.63 | -3.1674911 | -0.559 |
| WDR25 | ILMN_1704351 | 0.0609 | 0.00368 | -3.11 | -2.0623464 | -0.558 |
| SNX8 | ILMN_1804051 | 0.0854 | 0.0065 | -2.89 | -2.5758009 | -0.558 |
| GRAMD4 | ILMN_1723706 | 0.102 | 0.00902 | -2.76 | -2.8699787 | -0.558 |
| SRSF5 | ILMN_1761996 | 0.225 | 0.0368 | -2.17 | -4.1012382 | -0.558 |
| MAP3K11 | ILMN_1651788 | 0.0109 | 0.000239 | -4.08 | 0.4549238 | -0.557 |
| TIMP1 | ILMN_1711566 | 0.0563 | 0.00324 | -3.16 | -1.9476898 | -0.557 |
| MAPKAPK5 | ILMN_2322935 | 0.161 | 0.0199 | -2.44 | -3.5699431 | -0.557 |
| GPX3 | ILMN_1726666 | 0.211 | 0.0326 | -2.22 | -3.9980731 | -0.557 |
| STK19 | ILMN_2402363 | 0.244 | 0.0426 | -2.1 | -4.2258937 | -0.557 |
| KAT5 | ILMN_2414366 | 0.0308 | 0.00118 | -3.53 | -1.0235425 | -0.556 |
| PLGLB1 | ILMN_2094416 | 0.129 | 0.0136 | -2.6 | -3.2350992 | -0.556 |
| SEC62 | ILMN_1762003 | 0.152 | 0.018 | -2.48 | -3.4828451 | -0.556 |
| BRI3BP | ILMN_1800619 | 0.169 | 0.0218 | -2.4 | -3.6473871 | -0.556 |
| KLHDC3 | ILMN_1730940 | 0.238 | 0.0404 | -2.13 | -4.1805941 | -0.556 |
| LGALS1 | ILMN_1723978 | 0.242 | 0.0419 | -2.11 | -4.2121806 | -0.556 |
| GTF2F1 | ILMN_1748616 | 0.00202 | 1.80E-05 | -4.95 | 2.8702451 | -0.555 |
| HSBP1 | ILMN_1667030 | 0.0844 | 0.00635 | -2.9 | -2.5553768 | -0.555 |
| AP1S2 | ILMN_2120273 | 0.00416 | 5.23E-05 | -4.6 | 1.8718748 | -0.554 |
| DNAJB12 | ILMN_1712929 | 0.00631 | 9.96E-05 | -4.38 | 1.2699853 | -0.554 |
| MYO1F | ILMN_1681239 | 0.0103 | 0.000214 | -4.12 | 0.5563717 | -0.554 |
| AP4E1 | ILMN_1754531 | 0.0613 | 0.00372 | -3.11 | -2.0716731 | -0.554 |
| TUFM | ILMN_1738369 | 0.00142 | 1.06E-05 | -5.12 | 3.3650038 | -0.553 |
| DGKA | ILMN_2319913 | 0.0801 | 0.00581 | -2.93 | -2.4757834 | -0.553 |
| PKM | ILMN_1775327 | 0.00578 | 8.57E-05 | -4.43 | 1.4105171 | -0.552 |
| ZDHHC16 | ILMN_1763568 | 0.122 | 0.0122 | -2.64 | -3.1380672 | -0.552 |
| MVD | ILMN_1657550 | 0.102 | 0.0089 | -2.77 | -2.8578885 | -0.551 |
| MICB | ILMN_1708006 | 0.0235 | 0.000765 | -3.68 | -0.6238292 | -0.55 |
| LCLAT1 | ILMN_1708081 | 0.0472 | 0.00245 | -3.26 | -1.6910279 | -0.55 |
| SLC9A3R1 | ILMN_1680925 | 0.0525 | 0.0029 | -3.2 | -1.8452049 | -0.55 |
| CRSP8P | ILMN_3244506 | 0.0902 | 0.0072 | -2.85 | -2.6681174 | -0.55 |
| API5 | ILMN_1815051 | 0.0925 | 0.00758 | -2.83 | -2.7138611 | -0.55 |
| SKIV2L2 | ILMN_1651513 | 0.106 | 0.00958 | -2.74 | -2.9240025 | -0.55 |
| USO1 | ILMN_1692121 | 0.0694 | 0.00458 | -3.03 | -2.2607249 | -0.549 |
| UNC119 | ILMN_1664698 | 0.0786 | 0.00558 | -2.95 | -2.4391942 | -0.549 |
| OSCAR | ILMN_2367418 | 0.0795 | 0.0057 | -2.94 | -2.4574826 | -0.549 |
| PIK3R4 | ILMN_1715832 | 0.103 | 0.00913 | -2.76 | -2.881114 | -0.549 |
| AP5M1 | ILMN_3237641 | 0.121 | 0.0121 | -2.64 | -3.1328411 | -0.549 |
| CIAO1 | ILMN_2048793 | 0.139 | 0.0155 | -2.54 | -3.3500724 | -0.549 |
| C19orf52 | ILMN_1726181 | 0.12 | 0.0118 | -2.65 | -3.1104772 | -0.548 |
| MOAP1 | ILMN_1804988 | 0.202 | 0.0302 | -2.26 | -3.931483 | -0.548 |
| LIN37 | ILMN_1656886 | 0.0792 | 0.00565 | -2.95 | -2.4507891 | -0.547 |
| SURF6 | ILMN_1778032 | 0.116 | 0.0114 | -2.67 | -3.0749731 | -0.547 |
| TTLL12 | ILMN_1663113 | 0.00103 | 6.48E-06 | -5.28 | 3.8287763 | -0.546 |
| ZDHHC12 | ILMN_1703370 | 0.229 | 0.0379 | -2.16 | -4.12547 | -0.546 |
| MRPS36 | ILMN_1807095 | 0.00696 | 0.000117 | -4.32 | 1.1168533 | -0.545 |
| RPUSD1 | ILMN_1683082 | 0.0136 | 0.000345 | -3.96 | 0.1142371 | -0.545 |
| SPHK2 | ILMN_1729281 | 0.0332 | 0.00135 | -3.48 | -1.1473809 | -0.545 |
| KAT5 | ILMN_1759789 | 0.0818 | 0.00604 | -2.92 | -2.5104429 | -0.544 |
| GRIPAP1 | ILMN_1719857 | 0.124 | 0.0127 | -2.62 | -3.1754569 | -0.544 |
| PRELID3B | ILMN_1728168 | 0.201 | 0.0301 | -2.26 | -3.9283891 | -0.544 |
| ACTR1A | ILMN_1792314 | 0.247 | 0.0439 | -2.09 | -4.2504856 | -0.544 |
| POLDIP3 | ILMN_1688000 | 0.0147 | 0.000383 | -3.92 | 0.0170274 | -0.543 |
| PAK1 | ILMN_1767365 | 0.0484 | 0.00256 | -3.24 | -1.7337174 | -0.543 |
| AP5Z1 | ILMN_2083559 | 0.053 | 0.00294 | -3.19 | -1.858752 | -0.543 |
| CCS | ILMN_2290998 | 0.139 | 0.0155 | -2.54 | -3.3495289 | -0.543 |
| TAF13 | ILMN_2061327 | 0.0258 | 0.000892 | -3.63 | -0.7649372 | -0.542 |
| ARIH2OS | ILMN_3241670 | 0.0442 | 0.00214 | -3.31 | -1.5701727 | -0.542 |
| MAPK6 | ILMN_1757287 | 0.107 | 0.00969 | -2.73 | -2.9338776 | -0.542 |
| MFSD8 | ILMN_2123119 | 0.109 | 0.0102 | -2.71 | -2.9793634 | -0.542 |
| DIMT1 | ILMN_1803312 | 0.26 | 0.0483 | -2.05 | -4.3318381 | -0.542 |
| E2F4 | ILMN_1761828 | 0.00637 | 0.000101 | -4.37 | 1.2532817 | -0.541 |
| APEX2 | ILMN_1652505 | 0.109 | 0.0101 | -2.72 | -2.9724368 | -0.541 |
| SFT2D1 | ILMN_1734895 | 0.162 | 0.0201 | -2.43 | -3.5774239 | -0.541 |
| ASGR1 | ILMN_1769013 | 0.2 | 0.0297 | -2.27 | -3.9160496 | -0.541 |
| ACTR6 | ILMN_1697585 | 0.222 | 0.0356 | -2.18 | -4.0733522 | -0.541 |
| VHL | ILMN_1738579 | 0.0372 | 0.00161 | -3.41 | -1.308533 | -0.54 |
| CCAR2 | ILMN_1804789 | 0.0768 | 0.00536 | -2.97 | -2.4026238 | -0.54 |
| MCTP1 | ILMN_1654685 | 0.171 | 0.0223 | -2.39 | -3.6672068 | -0.54 |
| PRDX4 | ILMN_2222234 | 0.202 | 0.0303 | -2.26 | -3.9359588 | -0.54 |
| SNX8 | ILMN_1857017 | 0.211 | 0.0326 | -2.22 | -3.99822 | -0.54 |
| CFAP20 | ILMN_2112599 | 0.0118 | 0.000268 | -4.04 | 0.3465408 | -0.539 |
| ZBTB21 | ILMN_1782110 | 0.0709 | 0.00472 | -3.01 | -2.2887558 | -0.539 |
| TRABD | ILMN_1755737 | 0.145 | 0.0166 | -2.51 | -3.4115792 | -0.539 |
| C2orf49 | ILMN_1701131 | 0.149 | 0.0176 | -2.49 | -3.4599277 | -0.539 |
| MLEC | ILMN_1657495 | 0.0546 | 0.00308 | -3.18 | -1.9007335 | -0.538 |
| REV1 | ILMN_1707062 | 0.175 | 0.0234 | -2.37 | -3.7100192 | -0.538 |
| NUP62 | ILMN_1738681 | 0.186 | 0.0261 | -2.32 | -3.8063132 | -0.538 |
| NXT1 | ILMN_1760280 | 0.259 | 0.0481 | -2.05 | -4.3289225 | -0.538 |
| HAX1 | ILMN_2398903 | 0.064 | 0.00402 | -3.08 | -2.142879 | -0.537 |
| UNC119 | ILMN_1806052 | 0.113 | 0.0109 | -2.69 | -3.0398162 | -0.537 |
| CTDSP1 | ILMN_1681678 | 0.168 | 0.0214 | -2.41 | -3.6316009 | -0.537 |
| LRRC25 | ILMN_1766487 | 0.173 | 0.0227 | -2.38 | -3.6854993 | -0.537 |
| DVL3 | ILMN_2137464 | 0.0281 | 0.00102 | -3.58 | -0.8875278 | -0.536 |
| RASGRP2 | ILMN_1794594 | 0.0125 | 0.000297 | -4.01 | 0.2522113 | -0.535 |
| NELFB | ILMN_2399328 | 0.065 | 0.00414 | -3.06 | -2.1696802 | -0.535 |
| SNRNP40 | ILMN_3247064 | 0.0885 | 0.00695 | -2.86 | -2.6368692 | -0.535 |
| TOX2 | ILMN_3298423 | 0.109 | 0.0102 | -2.71 | -2.9763683 | -0.535 |
| WDR55 | ILMN_1678957 | 0.253 | 0.0456 | -2.07 | -4.28388 | -0.535 |
| SSNA1 | ILMN_1715705 | 0.022 | 0.000693 | -3.71 | -0.5330064 | -0.533 |
| DARS | ILMN_1813836 | 0.00613 | 9.48E-05 | -4.4 | 1.3161229 | -0.532 |
| GPR108 | ILMN_2396571 | 0.00701 | 0.000119 | -4.32 | 1.1057107 | -0.531 |
| C17orf49 | ILMN_1763688 | 0.0104 | 0.00022 | -4.11 | 0.5335688 | -0.531 |
| SLC35B3 | ILMN_1682910 | 0.0575 | 0.00335 | -3.14 | -1.9774372 | -0.531 |
| FKBP1A | ILMN_1702237 | 0.0976 | 0.00825 | -2.8 | -2.789703 | -0.531 |
| DGKA | ILMN_2319910 | 0.132 | 0.0142 | -2.58 | -3.2713827 | -0.531 |
| CNPY2 | ILMN_2123402 | 0.171 | 0.0221 | -2.39 | -3.6626595 | -0.531 |
| TTYH3 | ILMN_1692731 | 0.177 | 0.0238 | -2.36 | -3.724099 | -0.531 |
| ZFYVE27 | ILMN_2322972 | 0.218 | 0.0347 | -2.2 | -4.0513932 | -0.531 |
| ATP5SL | ILMN_1809027 | 0.227 | 0.0373 | -2.16 | -4.1137679 | -0.531 |
| RPS6KA4 | ILMN_1756204 | 0.0258 | 0.000894 | -3.63 | -0.7673441 | -0.53 |
| PURB | ILMN_1750079 | 0.245 | 0.043 | -2.1 | -4.2341575 | -0.53 |
| SAP30BP | ILMN_2199022 | 0.0171 | 0.00048 | -3.84 | -0.1930949 | -0.529 |
| EDF1 | ILMN_2246894 | 0.0175 | 0.000496 | -3.83 | -0.2230983 | -0.529 |
| PIP4K2C | ILMN_1787308 | 0.065 | 0.00414 | -3.06 | -2.1697116 | -0.529 |
| RAB33A | ILMN_1724708 | 0.173 | 0.0227 | -2.38 | -3.6841806 | -0.529 |
| SEC22A | ILMN_1775036 | 0.143 | 0.0163 | -2.52 | -3.3918957 | -0.528 |
| RING1 | ILMN_1666399 | 0.018 | 0.000513 | -3.82 | -0.254589 | -0.527 |
| ZMIZ2 | ILMN_1760718 | 0.0268 | 0.000947 | -3.6 | -0.8201486 | -0.527 |
| INIP | ILMN_1688621 | 0.0786 | 0.00555 | -2.95 | -2.4340991 | -0.526 |
| CCPG1 | ILMN_2409290 | 0.138 | 0.0152 | -2.55 | -3.3302916 | -0.526 |
| FCF1 | ILMN_2189870 | 0.0374 | 0.00163 | -3.41 | -1.3183227 | -0.525 |
| NSF | ILMN_1680353 | 0.0649 | 0.00412 | -3.07 | -2.1644666 | -0.525 |
| NDUFAF3 | ILMN_2354515 | 0.142 | 0.0161 | -2.53 | -3.3815713 | -0.525 |
| PDCD2 | ILMN_1797684 | 0.148 | 0.0172 | -2.5 | -3.4440176 | -0.525 |
| TMEM115 | ILMN_1712035 | 0.231 | 0.0385 | -2.15 | -4.1404659 | -0.525 |
| SNRNP40 | ILMN_1799814 | 0.0901 | 0.00717 | -2.85 | -2.6644449 | -0.524 |
| CIB1 | ILMN_1684205 | 0.0964 | 0.00808 | -2.81 | -2.7713961 | -0.524 |
| RCN2 | ILMN_1662129 | 0.149 | 0.0174 | -2.49 | -3.4528962 | -0.524 |
| USP42 | ILMN_2115696 | 0.244 | 0.0425 | -2.1 | -4.224609 | -0.524 |
| SEPT2 | ILMN_2365711 | 0.0876 | 0.00684 | -2.87 | -2.622739 | -0.523 |
| KCTD10 | ILMN_1719064 | 0.138 | 0.0153 | -2.55 | -3.3402868 | -0.523 |
| TMEM50B | ILMN_2047599 | 0.0487 | 0.0026 | -3.24 | -1.7478721 | -0.522 |
| C22orf39 | ILMN_1804884 | 0.2 | 0.0297 | -2.26 | -3.918213 | -0.522 |
| RAB10 | ILMN_1793433 | 0.00745 | 0.000131 | -4.29 | 1.0164367 | -0.521 |
| TRIOBP | ILMN_2370588 | 0.00891 | 0.00017 | -4.2 | 0.7709254 | -0.52 |
| CD58 | ILMN_1785268 | 0.0458 | 0.00227 | -3.29 | -1.6234159 | -0.52 |
| POP4 | ILMN_1696383 | 0.0481 | 0.00253 | -3.25 | -1.7226063 | -0.52 |
| TEPSIN | ILMN_2056687 | 0.187 | 0.0264 | -2.32 | -3.8141369 | -0.52 |
| UBN2 | ILMN_1685855 | 0.196 | 0.0284 | -2.28 | -3.8795473 | -0.52 |
| THAP11 | ILMN_1780699 | 0.0104 | 0.00022 | -4.11 | 0.5306002 | -0.519 |
| STARD3NL | ILMN_2228873 | 0.0786 | 0.00557 | -2.95 | -2.4366585 | -0.519 |
| TRAPPC3 | ILMN_1751627 | 0.177 | 0.0239 | -2.36 | -3.7277477 | -0.518 |
| CFP | ILMN_1658121 | 0.182 | 0.025 | -2.34 | -3.7690333 | -0.518 |
| SQSTM1 | ILMN_1662618 | 0.000329 | 9.30E-07 | -5.92 | 5.6494596 | -0.517 |
| SMIM12 | ILMN_1693226 | 0.0379 | 0.00167 | -3.4 | -1.3407181 | -0.517 |
| FLAD1 | ILMN_1663667 | 0.0734 | 0.00496 | -3 | -2.3332218 | -0.517 |
| TTLL4 | ILMN_1746846 | 0.114 | 0.011 | -2.68 | -3.0451002 | -0.517 |
| TBCC | ILMN_1743352 | 0.169 | 0.0216 | -2.4 | -3.642096 | -0.517 |
| BRAT1 | ILMN_1804498 | 0.0289 | 0.00106 | -3.57 | -0.9224741 | -0.516 |
| DDIT4 | ILMN_1661599 | 0.0338 | 0.0014 | -3.47 | -1.1773212 | -0.516 |
| MUS81 | ILMN_1780937 | 0.0553 | 0.00315 | -3.17 | -1.9204579 | -0.516 |
| GORASP2 | ILMN_1748018 | 0.0784 | 0.00553 | -2.95 | -2.430884 | -0.516 |
| PRKCI | ILMN_1725188 | 0.102 | 0.00902 | -2.76 | -2.869783 | -0.516 |
| TACC1 | ILMN_1770084 | 0.127 | 0.0132 | -2.61 | -3.2091843 | -0.516 |
| SENP5 | ILMN_1675501 | 0.218 | 0.0346 | -2.2 | -4.0494262 | -0.516 |
| TMEM179B | ILMN_1733757 | 0.252 | 0.0455 | -2.07 | -4.2822709 | -0.516 |
| VPS33A | ILMN_1662316 | 0.0248 | 0.000838 | -3.65 | -0.7073853 | -0.515 |
| PKM | ILMN_1672650 | 0.0722 | 0.00485 | -3 | -2.3130263 | -0.514 |
| LZIC | ILMN_1661627 | 0.166 | 0.0211 | -2.41 | -3.6204592 | -0.514 |
| FCF1 | ILMN_2189869 | 0.0571 | 0.00332 | -3.15 | -1.9684702 | -0.513 |
| WNK1 | ILMN_1876924 | 0.0747 | 0.00514 | -2.98 | -2.364496 | -0.513 |
| SUGP2 | ILMN_1711270 | 0.181 | 0.0248 | -2.34 | -3.7614386 | -0.513 |
| BNIP3L | ILMN_2045419 | 0.235 | 0.0397 | -2.14 | -4.1660358 | -0.513 |
| ARL5A | ILMN_1771738 | 0.255 | 0.0463 | -2.06 | -4.2971458 | -0.513 |
| FKRP | ILMN_2368617 | 0.0322 | 0.00128 | -3.5 | -1.0957511 | -0.512 |
| C7orf26 | ILMN_1730048 | 0.0885 | 0.00697 | -2.86 | -2.6387876 | -0.512 |
| GTF2F2 | ILMN_1745798 | 0.161 | 0.0199 | -2.44 | -3.570707 | -0.512 |
| SURF4 | ILMN_1690761 | 0.000913 | 4.77E-06 | -5.38 | 4.1157979 | -0.511 |
| WNK1 | ILMN_1753165 | 0.0181 | 0.000522 | -3.81 | -0.2696587 | -0.511 |
| AP1S2 | ILMN_1766411 | 0.0185 | 0.000541 | -3.8 | -0.303793 | -0.511 |
| GALNS | ILMN_1737949 | 0.0211 | 0.000661 | -3.73 | -0.4891065 | -0.51 |
| MBD1 | ILMN_2352580 | 0.0619 | 0.0038 | -3.1 | -2.090574 | -0.51 |
| TAOK2 | ILMN_1701487 | 0.0876 | 0.00684 | -2.87 | -2.6218247 | -0.51 |
| MRPL22 | ILMN_1663220 | 0.177 | 0.0237 | -2.36 | -3.7217685 | -0.51 |
| VPS4A | ILMN_1708946 | 0.0584 | 0.00343 | -3.13 | -1.9997349 | -0.509 |
| PPP1R11 | ILMN_1747598 | 0.0925 | 0.0076 | -2.83 | -2.7163525 | -0.509 |
| PGAM1 | ILMN_1661366 | 0.208 | 0.0318 | -2.24 | -3.9751081 | -0.509 |
| BORCS6 | ILMN_2225746 | 0.224 | 0.0365 | -2.17 | -4.093252 | -0.509 |
| SLC38A10 | ILMN_2277419 | 0.0458 | 0.0023 | -3.28 | -1.6329436 | -0.508 |
| ADGRE4P | ILMN_3243190 | 0.0866 | 0.0067 | -2.88 | -2.6032866 | -0.508 |
| UBL7 | ILMN_1733991 | 0.187 | 0.0263 | -2.32 | -3.8121951 | -0.508 |
| MUL1 | ILMN_3235168 | 0.228 | 0.0376 | -2.16 | -4.1203685 | -0.508 |
| NEDD4 | ILMN_1751407 | 0.00603 | 9.20E-05 | -4.41 | 1.3437005 | -0.507 |
| SSR2 | ILMN_1783226 | 0.0119 | 0.000274 | -4.04 | 0.3260347 | -0.507 |
| AZIN1 | ILMN_1656682 | 0.0321 | 0.00126 | -3.5 | -1.0848153 | -0.507 |
| TP53I11 | ILMN_2412624 | 0.0553 | 0.00315 | -3.17 | -1.9218283 | -0.507 |
| DRAM1 | ILMN_1669376 | 0.0772 | 0.0054 | -2.96 | -2.4091737 | -0.507 |
| INTS9 | ILMN_1777118 | 0.105 | 0.00938 | -2.75 | -2.9049102 | -0.507 |
| LOC606724 | ILMN_2125747 | 0.116 | 0.0113 | -2.67 | -3.0675398 | -0.507 |
| ANAPC4 | ILMN_1802973 | 0.215 | 0.0337 | -2.21 | -4.0265973 | -0.507 |
| NIP7 | ILMN_1704305 | 0.246 | 0.0435 | -2.09 | -4.2437594 | -0.507 |
| POLD4 | ILMN_1803772 | 0.22 | 0.0351 | -2.19 | -4.0611799 | -0.506 |
| SMARCC2 | ILMN_1773620 | 0.173 | 0.0226 | -2.38 | -3.6813551 | -0.505 |
| SLC39A1 | ILMN_2116714 | 0.175 | 0.0234 | -2.37 | -3.7092849 | -0.505 |
| RAPGEFL1 | ILMN_1758613 | 0.192 | 0.0275 | -2.3 | -3.8503307 | -0.505 |
| RPRD2 | ILMN_3238889 | 0.201 | 0.03 | -2.26 | -3.9252224 | -0.505 |
| RAB11FIP4 | ILMN_1681467 | 0.221 | 0.0354 | -2.19 | -4.0691034 | -0.505 |
| ANAPC5 | ILMN_1723177 | 0.00548 | 7.77E-05 | -4.46 | 1.5014906 | -0.504 |
| C10orf76 | ILMN_1792110 | 0.0298 | 0.00111 | -3.55 | -0.9705294 | -0.504 |
| STARD7 | ILMN_1740819 | 0.077 | 0.00538 | -2.96 | -2.40588 | -0.504 |
| ST3GAL2 | ILMN_1714165 | 0.158 | 0.0192 | -2.45 | -3.53677 | -0.504 |
| CHMP1A | ILMN_1709439 | 0.0183 | 0.000532 | -3.81 | -0.2875908 | -0.503 |
| MNT | ILMN_1792910 | 0.0377 | 0.00166 | -3.4 | -1.3355285 | -0.503 |
| POLR2C | ILMN_1659411 | 0.0566 | 0.00327 | -3.15 | -1.9550605 | -0.503 |
| FAM3A | ILMN_1808356 | 0.0977 | 0.00826 | -2.8 | -2.790786 | -0.503 |
| TCF20 | ILMN_2368068 | 0.119 | 0.0117 | -2.66 | -3.1041731 | -0.503 |
| CARD19 | ILMN_1659189 | 0.163 | 0.0203 | -2.43 | -3.5889198 | -0.503 |
| UBA52 | ILMN_2368576 | 0.163 | 0.0204 | -2.43 | -3.5912279 | -0.503 |
| FBXO42 | ILMN_1756874 | 0.173 | 0.0227 | -2.38 | -3.6858855 | -0.503 |
| RER1 | ILMN_1812067 | 0.175 | 0.0234 | -2.37 | -3.712098 | -0.503 |
| GPATCH2L | ILMN_2265093 | 0.218 | 0.0344 | -2.2 | -4.0444676 | -0.503 |
| FAM104A | ILMN_1807201 | 0.0239 | 0.000785 | -3.67 | -0.6480792 | -0.502 |
| PSMC1 | ILMN_1736353 | 0.0281 | 0.00102 | -3.58 | -0.8863154 | -0.502 |
| ALS2 | ILMN_1750256 | 0.0454 | 0.00223 | -3.29 | -1.6074264 | -0.502 |
| FBXL12 | ILMN_1693438 | 0.185 | 0.0256 | -2.33 | -3.7891618 | -0.502 |
| RARS2 | ILMN_1758214 | 0.258 | 0.0478 | -2.05 | -4.3234311 | -0.502 |
| GRN | ILMN_1811702 | 0.00692 | 0.000116 | -4.33 | 1.1247545 | -0.501 |
| ACO2 | ILMN_1654861 | 0.0543 | 0.00306 | -3.18 | -1.8947994 | -0.501 |
| MYO18A | ILMN_1780560 | 0.0597 | 0.00355 | -3.12 | -2.0305667 | -0.501 |
| PA2G4 | ILMN_1728984 | 0.148 | 0.0172 | -2.5 | -3.4437901 | -0.501 |
| FCRLB | ILMN_1782015 | 0.172 | 0.0225 | -2.38 | -3.6784884 | -0.501 |
| TBP | ILMN_1697117 | 0.223 | 0.0361 | -2.18 | -4.0845358 | -0.501 |
| EPM2AIP1 | ILMN_1682658 | 0.227 | 0.0375 | -2.16 | -4.117111 | -0.501 |
| SLC43A2 | ILMN_1787127 | 0.0404 | 0.00185 | -3.36 | -1.4374791 | -0.5 |
| BCL2L12 | ILMN_2396982 | 0.0608 | 0.00365 | -3.11 | -2.0562028 | -0.5 |
| CSRP1 | ILMN_1811921 | 0.238 | 0.0406 | -2.12 | -4.1858709 | -0.5 |
| PKN1 | ILMN_2367710 | 0.031 | 0.0012 | -3.52 | -1.0362166 | -0.499 |
| STARD10 | ILMN_1717052 | 0.0322 | 0.00127 | -3.5 | -1.0922819 | -0.499 |
| FAM96B | ILMN_1779813 | 0.0739 | 0.00503 | -2.99 | -2.3451586 | -0.499 |
| FAM120B | ILMN_1679641 | 0.198 | 0.0292 | -2.27 | -3.9018208 | -0.499 |
| RAB22A | ILMN_1786976 | 0.213 | 0.0331 | -2.22 | -4.0114833 | -0.499 |
| ZNF322 | ILMN_3251742 | 0.239 | 0.041 | -2.12 | -4.1932191 | -0.499 |
| FASTK | ILMN_1664098 | 0.00705 | 0.000121 | -4.31 | 1.0867931 | -0.498 |
| AFG3L2 | ILMN_2066124 | 0.112 | 0.0107 | -2.69 | -3.0262461 | -0.498 |
| TOB2 | ILMN_1748884 | 0.00672 | 0.000111 | -4.34 | 1.1659045 | -0.497 |
| FDFT1 | ILMN_2144088 | 0.171 | 0.0223 | -2.39 | -3.6677968 | -0.497 |
| FAM60A | ILMN_3272603 | 0.0104 | 0.000225 | -4.1 | 0.5123804 | -0.496 |
| GNL1 | ILMN_1789457 | 0.041 | 0.00192 | -3.35 | -1.4681369 | -0.496 |
| ZNHIT1 | ILMN_1741491 | 0.061 | 0.00368 | -3.11 | -2.0634981 | -0.496 |
| SETDB1 | ILMN_1718207 | 0.174 | 0.0229 | -2.38 | -3.6929771 | -0.496 |
| MDC1 | ILMN_1814122 | 0.179 | 0.0243 | -2.35 | -3.7424604 | -0.496 |
| PTS | ILMN_2162328 | 0.254 | 0.0459 | -2.07 | -4.2894115 | -0.496 |
| MRPL30 | ILMN_1766154 | 0.0627 | 0.0039 | -3.09 | -2.1144729 | -0.495 |
| HMG20A | ILMN_1678290 | 0.0933 | 0.00771 | -2.82 | -2.7298089 | -0.495 |
| SRP9 | ILMN_2099594 | 0.114 | 0.011 | -2.68 | -3.0493597 | -0.495 |
| RNF181 | ILMN_1655340 | 0.257 | 0.0471 | -2.06 | -4.3112742 | -0.495 |
| GGTLC2 | ILMN_2403896 | 0.0925 | 0.00759 | -2.83 | -2.7158849 | -0.494 |
| MYO9B | ILMN_1672547 | 0.0627 | 0.00388 | -3.09 | -2.1116936 | -0.493 |
| RBPJ | ILMN_1726913 | 0.0795 | 0.0057 | -2.94 | -2.4575145 | -0.493 |
| RNPEPL1 | ILMN_1694730 | 0.184 | 0.0254 | -2.33 | -3.7818721 | -0.493 |
| KIAA1143 | ILMN_1752273 | 0.216 | 0.034 | -2.21 | -4.0328485 | -0.493 |
| PHF2 | ILMN_1720476 | 0.00513 | 6.90E-05 | -4.5 | 1.613022 | -0.492 |
| NMNAT1 | ILMN_1692413 | 0.0341 | 0.00142 | -3.46 | -1.1905345 | -0.492 |
| GPATCH2L | ILMN_1699091 | 0.0373 | 0.00162 | -3.41 | -1.3126881 | -0.492 |
| ZSWIM1 | ILMN_1812856 | 0.0626 | 0.00386 | -3.09 | -2.1048198 | -0.492 |
| RNF220 | ILMN_1694504 | 0.202 | 0.0303 | -2.26 | -3.9359427 | -0.492 |
| NPHP4 | ILMN_2219512 | 0.0639 | 0.004 | -3.08 | -2.1375522 | -0.491 |
| RPAP3 | ILMN_1766916 | 0.0931 | 0.00767 | -2.83 | -2.7252953 | -0.491 |
| CCNG2 | ILMN_2228732 | 0.174 | 0.0229 | -2.38 | -3.691881 | -0.491 |
| RGL2 | ILMN_2124386 | 0.000985 | 6.10E-06 | -5.3 | 3.8853722 | -0.49 |
| VPS39 | ILMN_1770388 | 0.13 | 0.0137 | -2.59 | -3.2422104 | -0.49 |
| KCNAB2 | ILMN_2342762 | 0.134 | 0.0146 | -2.57 | -3.2976284 | -0.49 |
| NCKAP5L | ILMN_1763640 | 0.163 | 0.0204 | -2.43 | -3.5914598 | -0.49 |
| SUOX | ILMN_1803745 | 0.249 | 0.0446 | -2.08 | -4.2646333 | -0.49 |
| NADK | ILMN_1758963 | 0.0105 | 0.000229 | -4.1 | 0.4942397 | -0.489 |
| DCAF15 | ILMN_2192620 | 0.0245 | 0.000821 | -3.66 | -0.688853 | -0.489 |
| CERS2 | ILMN_2400500 | 0.0805 | 0.00587 | -2.93 | -2.4849062 | -0.489 |
| RAB5B | ILMN_1752582 | 0.128 | 0.0134 | -2.6 | -3.2189963 | -0.489 |
| NOMO2 | ILMN_1799856 | 0.189 | 0.0267 | -2.31 | -3.8238026 | -0.489 |
| ZNF160 | ILMN_1777049 | 0.236 | 0.04 | -2.13 | -4.1715153 | -0.489 |
| TAPBPL | ILMN_1805449 | 0.0856 | 0.00654 | -2.89 | -2.582234 | -0.488 |
| FBP1 | ILMN_1728799 | 0.133 | 0.0144 | -2.57 | -3.283194 | -0.488 |
| PPP1CC | ILMN_1701855 | 0.244 | 0.0426 | -2.1 | -4.2249774 | -0.488 |
| TMEM55B | ILMN_1770742 | 0.251 | 0.0452 | -2.08 | -4.2758617 | -0.488 |
| PHF1 | ILMN_1752877 | 0.137 | 0.015 | -2.56 | -3.321627 | -0.487 |
| RETSAT | ILMN_1702633 | 0.144 | 0.0164 | -2.52 | -3.3973436 | -0.487 |
| NCF1C | ILMN_2112988 | 0.0244 | 0.000811 | -3.66 | -0.6772269 | -0.486 |
| STK36 | ILMN_1693538 | 0.00828 | 0.000152 | -4.24 | 0.8753806 | -0.485 |
| OLR1 | ILMN_1723035 | 0.0248 | 0.00084 | -3.65 | -0.7103543 | -0.485 |
| FXR2 | ILMN_1738699 | 0.0249 | 0.000847 | -3.64 | -0.7174886 | -0.485 |
| FAM111A | ILMN_2410038 | 0.0429 | 0.00206 | -3.32 | -1.5344164 | -0.485 |
| UHRF1 | ILMN_1786065 | 0.0892 | 0.00705 | -2.86 | -2.6496569 | -0.485 |
| DLEU7 | ILMN_1782881 | 0.101 | 0.0087 | -2.78 | -2.8373735 | -0.485 |
| CCDC12 | ILMN_1725071 | 0.101 | 0.00877 | -2.77 | -2.8453056 | -0.485 |
| NTMT1 | ILMN_2170515 | 0.141 | 0.0159 | -2.53 | -3.3717935 | -0.485 |
| GOLT1B | ILMN_1767837 | 0.199 | 0.0294 | -2.27 | -3.9074354 | -0.485 |
| SSU72 | ILMN_1664956 | 0.000286 | 7.00E-07 | -6.01 | 5.9163063 | -0.484 |
| GRN | ILMN_1724250 | 0.0306 | 0.00117 | -3.53 | -1.0118257 | -0.484 |
| ACAT2 | ILMN_1708672 | 0.0803 | 0.00585 | -2.93 | -2.4811487 | -0.484 |
| TIMM8B | ILMN_1738938 | 0.114 | 0.011 | -2.68 | -3.0496215 | -0.484 |
| CYTH4 | ILMN_1660364 | 0.136 | 0.0149 | -2.56 | -3.312961 | -0.484 |
| STK38L | ILMN_1755792 | 0.151 | 0.018 | -2.48 | -3.4800895 | -0.484 |
| TOR4A | ILMN_1813641 | 0.161 | 0.0198 | -2.44 | -3.5645995 | -0.484 |
| DNLZ | ILMN_2112049 | 0.0537 | 0.00301 | -3.18 | -1.8789133 | -0.483 |
| SMPD1 | ILMN_1757370 | 0.0594 | 0.00352 | -3.13 | -2.0210154 | -0.483 |
| SLC2A5 | ILMN_1671337 | 0.113 | 0.0109 | -2.69 | -3.0354324 | -0.483 |
| PIGH | ILMN_3251246 | 0.117 | 0.0115 | -2.66 | -3.0878756 | -0.483 |
| POLR2I | ILMN_1720542 | 0.0722 | 0.00485 | -3 | -2.3124361 | -0.482 |
| ZMIZ1 | ILMN_1771627 | 0.0246 | 0.000828 | -3.65 | -0.6966986 | -0.481 |
| TXNDC11 | ILMN_1771862 | 0.0628 | 0.0039 | -3.09 | -2.1158303 | -0.481 |
| NDUFS1 | ILMN_1728810 | 0.0819 | 0.00606 | -2.92 | -2.5130765 | -0.481 |
| CDK9 | ILMN_1747556 | 0.109 | 0.0102 | -2.71 | -2.9819563 | -0.481 |
| GTF3C2 | ILMN_1746457 | 0.14 | 0.0158 | -2.54 | -3.3653333 | -0.481 |
| DHRS7B | ILMN_1707901 | 0.167 | 0.0212 | -2.41 | -3.623674 | -0.481 |
| ZEB1-AS1 | ILMN_1829989 | 0.263 | 0.0495 | -2.03 | -4.3527441 | -0.481 |
| INO80E | ILMN_1724406 | 0.0268 | 0.000947 | -3.6 | -0.8207221 | -0.48 |
| GLTP | ILMN_1764380 | 0.0492 | 0.00264 | -3.23 | -1.7594089 | -0.48 |
| WASH1 | ILMN_3245020 | 0.234 | 0.0393 | -2.14 | -4.1574195 | -0.48 |
| HAX1 | ILMN_2289775 | 0.0429 | 0.00205 | -3.33 | -1.5317469 | -0.479 |
| WDR73 | ILMN_1702592 | 0.0588 | 0.00346 | -3.13 | -2.007278 | -0.479 |
| PIGX | ILMN_1769508 | 0.0801 | 0.00579 | -2.94 | -2.4724606 | -0.479 |
| SKIV2L | ILMN_1666512 | 0.0835 | 0.00625 | -2.91 | -2.5408426 | -0.479 |
| MRPS18A | ILMN_1730391 | 0.12 | 0.0119 | -2.65 | -3.1181632 | -0.479 |
| MIB2 | ILMN_2282077 | 0.149 | 0.0176 | -2.49 | -3.4598071 | -0.479 |
| NOTCH1 | ILMN_1729161 | 0.172 | 0.0224 | -2.39 | -3.6728006 | -0.479 |
| VHL | ILMN_2376625 | 0.258 | 0.0476 | -2.05 | -4.3202228 | -0.479 |
| OTUD5 | ILMN_2088847 | 0.0333 | 0.00136 | -3.48 | -1.151863 | -0.478 |
| SEC11A | ILMN_1693490 | 0.0739 | 0.00502 | -2.99 | -2.3435141 | -0.478 |
| GRPEL2 | ILMN_1721138 | 0.0537 | 0.003 | -3.19 | -1.8774133 | -0.477 |
| UFC1 | ILMN_2110281 | 0.0925 | 0.00758 | -2.83 | -2.7142074 | -0.477 |
| DNAJC10 | ILMN_2151541 | 0.127 | 0.0132 | -2.61 | -3.2087082 | -0.477 |
| HCK | ILMN_1791771 | 0.13 | 0.0138 | -2.59 | -3.2494221 | -0.477 |
| BMP2K | ILMN_1811148 | 0.174 | 0.0232 | -2.37 | -3.702736 | -0.477 |
| CERS2 | ILMN_1726108 | 0.0865 | 0.00668 | -2.88 | -2.601089 | -0.476 |
| PHRF1 | ILMN_3245476 | 0.121 | 0.012 | -2.65 | -3.1220014 | -0.476 |
| PTP4A3 | ILMN_3250321 | 0.156 | 0.0189 | -2.46 | -3.5224006 | -0.476 |
| DHX29 | ILMN_1697503 | 0.167 | 0.0213 | -2.41 | -3.6303179 | -0.476 |
| SAP18 | ILMN_1752793 | 0.212 | 0.033 | -2.22 | -4.0070685 | -0.476 |
| P2RY2 | ILMN_1723535 | 0.12 | 0.0119 | -2.65 | -3.1130407 | -0.475 |
| CORO1B | ILMN_1653708 | 0.126 | 0.013 | -2.61 | -3.1969355 | -0.475 |
| SLC25A16 | ILMN_1806032 | 0.252 | 0.0454 | -2.07 | -4.2796383 | -0.475 |
| CORO1B | ILMN_2377019 | 0.00875 | 0.000166 | -4.21 | 0.7955686 | -0.474 |
| SMARCD1 | ILMN_1728845 | 0.0395 | 0.00179 | -3.38 | -1.4036184 | -0.474 |
| GID8 | ILMN_1755909 | 0.15 | 0.0176 | -2.49 | -3.4627575 | -0.474 |
| VMA21 | ILMN_1682694 | 0.227 | 0.0374 | -2.16 | -4.1156446 | -0.474 |
| ALKBH5 | ILMN_1657283 | 0.215 | 0.0337 | -2.21 | -4.026272 | -0.473 |
| CTSZ | ILMN_1666269 | 0.0686 | 0.0045 | -3.03 | -2.2452632 | -0.472 |
| ARF4 | ILMN_1719518 | 0.11 | 0.0104 | -2.71 | -2.9936448 | -0.472 |
| TRIP4 | ILMN_1661173 | 0.143 | 0.0161 | -2.53 | -3.3838687 | -0.472 |
| C9orf78 | ILMN_1697166 | 0.00123 | 8.35E-06 | -5.2 | 3.5912757 | -0.471 |
| CERK | ILMN_1767475 | 0.00677 | 0.000113 | -4.34 | 1.1532097 | -0.471 |
| MTMR14 | ILMN_1659240 | 0.0174 | 0.000492 | -3.84 | -0.214805 | -0.471 |
| DSCR3 | ILMN_1717099 | 0.187 | 0.0263 | -2.32 | -3.8138385 | -0.471 |
| PPP1R7 | ILMN_1808333 | 0.149 | 0.0175 | -2.49 | -3.4585178 | -0.47 |
| ORMDL2 | ILMN_1774708 | 0.165 | 0.0209 | -2.42 | -3.6107775 | -0.47 |
| AP1B1 | ILMN_1781983 | 0.26 | 0.0485 | -2.04 | -4.3354825 | -0.47 |
| KIAA0196 | ILMN_1728676 | 0.153 | 0.0182 | -2.48 | -3.4895486 | -0.469 |
| SGPL1 | ILMN_1761531 | 0.193 | 0.0277 | -2.3 | -3.8569444 | -0.469 |
| PLCG2 | ILMN_1815719 | 0.207 | 0.0315 | -2.24 | -3.9676168 | -0.469 |
| TIAF1 | ILMN_2055634 | 0.26 | 0.0484 | -2.04 | -4.3332178 | -0.469 |
| POLR2G | ILMN_1748438 | 0.134 | 0.0145 | -2.57 | -3.2894386 | -0.468 |
| TWF2 | ILMN_1703305 | 0.0654 | 0.00419 | -3.06 | -2.1791423 | -0.467 |
| HAUS8 | ILMN_3176989 | 0.0131 | 0.000326 | -3.98 | 0.1659483 | -0.466 |
| PPCS | ILMN_1776094 | 0.241 | 0.0416 | -2.11 | -4.2061701 | -0.466 |
| EFR3A | ILMN_1664776 | 0.246 | 0.0436 | -2.09 | -4.2461005 | -0.466 |
| SHC1 | ILMN_1721022 | 0.00369 | 4.28E-05 | -4.66 | 2.0600595 | -0.465 |
| MAP2K2 | ILMN_1657968 | 0.00624 | 9.73E-05 | -4.39 | 1.2916786 | -0.465 |
| GTF2I | ILMN_1673917 | 0.0466 | 0.00239 | -3.27 | -1.6695528 | -0.465 |
| IL16 | ILMN_2290628 | 0.0925 | 0.00753 | -2.83 | -2.7078453 | -0.465 |
| TIMM23 | ILMN_1664231 | 0.162 | 0.02 | -2.43 | -3.5754126 | -0.465 |
| RWDD2A | ILMN_1722156 | 0.00928 | 0.000182 | -4.18 | 0.7057408 | -0.464 |
| POLM | ILMN_1716973 | 0.0565 | 0.00326 | -3.15 | -1.9522917 | -0.464 |
| GRK6 | ILMN_1681802 | 0.168 | 0.0216 | -2.4 | -3.6397312 | -0.464 |
| POM121C | ILMN_3235808 | 0.17 | 0.0221 | -2.39 | -3.659181 | -0.464 |
| STIM2 | ILMN_1738449 | 0.225 | 0.0368 | -2.17 | -4.1003419 | -0.464 |
| NEU1 | ILMN_1763144 | 0.0968 | 0.00814 | -2.8 | -2.7777331 | -0.463 |
| RAB24 | ILMN_1677843 | 0.149 | 0.0175 | -2.49 | -3.4566121 | -0.463 |
| ZBTB22 | ILMN_3241524 | 0.19 | 0.0269 | -2.31 | -3.8332996 | -0.463 |
| FDFT1 | ILMN_1741096 | 0.198 | 0.029 | -2.28 | -3.8972022 | -0.463 |
| DNAJC9 | ILMN_1799516 | 0.256 | 0.047 | -2.06 | -4.309058 | -0.463 |
| INSIG1 | ILMN_1793474 | 0.0888 | 0.007 | -2.86 | -2.6435426 | -0.462 |
| ARHGEF18 | ILMN_1664016 | 0.103 | 0.00911 | -2.76 | -2.8791618 | -0.462 |
| LYL1 | ILMN_2216582 | 0.133 | 0.0143 | -2.57 | -3.2818441 | -0.462 |
| TNFAIP1 | ILMN_1655429 | 0.264 | 0.0497 | -2.03 | -4.3558064 | -0.462 |
| EFHD2 | ILMN_1761463 | 0.000943 | 5.24E-06 | -5.35 | 4.0269678 | -0.461 |
| PUF60 | ILMN_1779404 | 0.0462 | 0.00232 | -3.28 | -1.6415396 | -0.461 |
| ZNF20 | ILMN_1670377 | 0.0735 | 0.00498 | -2.99 | -2.3358722 | -0.461 |
| TMEM86B | ILMN_2049417 | 0.123 | 0.0124 | -2.63 | -3.1562827 | -0.461 |
| HYAL2 | ILMN_1668283 | 0.135 | 0.0148 | -2.56 | -3.3078992 | -0.461 |
| DHX40 | ILMN_2248589 | 0.222 | 0.0358 | -2.18 | -4.0776829 | -0.461 |
| MAD2L1BP | ILMN_1694711 | 0.258 | 0.0479 | -2.05 | -4.3251782 | -0.461 |
| ATG7 | ILMN_1790978 | 0.0465 | 0.00237 | -3.27 | -1.6628129 | -0.46 |
| PHF5A | ILMN_2112402 | 0.0794 | 0.00568 | -2.94 | -2.4547097 | -0.46 |
| GLT8D1 | ILMN_1713290 | 0.144 | 0.0163 | -2.52 | -3.3962097 | -0.46 |
| SH3GL1 | ILMN_1788062 | 0.166 | 0.021 | -2.41 | -3.6174202 | -0.46 |
| LOC100288893 | ILMN_1845157 | 0.255 | 0.0463 | -2.06 | -4.2973085 | -0.46 |
| C1orf52 | ILMN_1742611 | 0.0901 | 0.00716 | -2.85 | -2.6635309 | -0.459 |
| TOP3B | ILMN_1765021 | 0.175 | 0.0234 | -2.37 | -3.7117776 | -0.459 |
| CYTH4 | ILMN_3238196 | 0.19 | 0.0271 | -2.31 | -3.8380449 | -0.459 |
| ZNF182 | ILMN_2337835 | 0.198 | 0.0292 | -2.27 | -3.9014766 | -0.459 |
| SIPA1 | ILMN_1682930 | 0.0189 | 0.000563 | -3.79 | -0.3398279 | -0.458 |
| GUCD1 | ILMN_1764410 | 0.0222 | 0.000701 | -3.71 | -0.5429516 | -0.458 |
| CCDC57 | ILMN_2140342 | 0.114 | 0.0111 | -2.68 | -3.055397 | -0.458 |
| AIP | ILMN_2103841 | 0.122 | 0.0123 | -2.64 | -3.1467059 | -0.458 |
| NDUFB5 | ILMN_1807397 | 0.131 | 0.014 | -2.58 | -3.260984 | -0.458 |
| FEN1 | ILMN_2160929 | 0.147 | 0.0169 | -2.51 | -3.4263543 | -0.458 |
| GRK6 | ILMN_2357015 | 0.181 | 0.0248 | -2.34 | -3.7612729 | -0.458 |
| POLR2F | ILMN_1745885 | 0.00745 | 0.000131 | -4.29 | 1.0148131 | -0.457 |
| P4HB | ILMN_1719303 | 0.0212 | 0.000666 | -3.73 | -0.4962195 | -0.457 |
| FAF2 | ILMN_1670472 | 0.0644 | 0.00405 | -3.07 | -2.1503256 | -0.457 |
| COIL | ILMN_1688034 | 0.152 | 0.0181 | -2.48 | -3.4847552 | -0.457 |
| RNF141 | ILMN_1815010 | 0.207 | 0.0315 | -2.24 | -3.967562 | -0.457 |
| RAB24 | ILMN_1714393 | 0.258 | 0.0479 | -2.05 | -4.3249699 | -0.457 |
| EXOC7 | ILMN_1815012 | 0.065 | 0.00414 | -3.06 | -2.1691403 | -0.456 |
| FAM32A | ILMN_3237241 | 0.0802 | 0.00583 | -2.93 | -2.4790886 | -0.456 |
| YRDC | ILMN_2061732 | 0.0971 | 0.00817 | -2.8 | -2.7812322 | -0.456 |
| SRF | ILMN_1803398 | 0.1 | 0.0086 | -2.78 | -2.8271128 | -0.456 |
| KIAA0753 | ILMN_1680010 | 0.102 | 0.009 | -2.76 | -2.8680776 | -0.456 |
| BRK1 | ILMN_1764500 | 0.123 | 0.0125 | -2.63 | -3.1572505 | -0.456 |
| CCPG1 | ILMN_1794190 | 0.125 | 0.0128 | -2.62 | -3.1806505 | -0.456 |
| SREK1 | ILMN_2373266 | 0.197 | 0.0288 | -2.28 | -3.8905802 | -0.456 |
| CHMP3 | ILMN_1683827 | 0.2 | 0.0298 | -2.26 | -3.9213762 | -0.456 |
| PRAM1 | ILMN_1711493 | 0.212 | 0.033 | -2.22 | -4.0078332 | -0.456 |
| CFDP1 | ILMN_1800837 | 0.222 | 0.0355 | -2.19 | -4.0699462 | -0.456 |
| LOC407835 | ILMN_2198893 | 0.000407 | 1.40E-06 | -5.78 | 5.2656882 | -0.455 |
| GOLGA2 | ILMN_1738821 | 0.0484 | 0.00257 | -3.24 | -1.7354193 | -0.455 |
| SEC61B | ILMN_1801852 | 0.127 | 0.0132 | -2.61 | -3.2092142 | -0.455 |
| MTMR9 | ILMN_1652521 | 0.212 | 0.033 | -2.22 | -4.0082415 | -0.455 |
| TOX2 | ILMN_2082209 | 0.0609 | 0.00368 | -3.11 | -2.0623501 | -0.454 |
| CIAO1 | ILMN_1792837 | 0.117 | 0.0115 | -2.67 | -3.0850058 | -0.454 |
| MAP3K7 | ILMN_1810176 | 0.129 | 0.0135 | -2.6 | -3.227888 | -0.454 |
| PGM2 | ILMN_1673543 | 0.15 | 0.0177 | -2.49 | -3.4657971 | -0.454 |
| AKAP8 | ILMN_1741572 | 0.222 | 0.0355 | -2.18 | -4.0716882 | -0.454 |
| SEMA4A | ILMN_1702787 | 0.0992 | 0.00845 | -2.79 | -2.8112445 | -0.453 |
| DCUN1D5 | ILMN_1726839 | 0.119 | 0.0117 | -2.66 | -3.0997729 | -0.453 |
| TFEB | ILMN_1733616 | 0.134 | 0.0145 | -2.57 | -3.2916971 | -0.453 |
| MED4 | ILMN_1664641 | 0.142 | 0.016 | -2.53 | -3.3804184 | -0.453 |
| ZNF224 | ILMN_1661293 | 0.182 | 0.0249 | -2.34 | -3.7648149 | -0.453 |
| C14orf28 | ILMN_1807031 | 0.253 | 0.0457 | -2.07 | -4.2857679 | -0.453 |
| EDF1 | ILMN_1726169 | 0.0127 | 0.000312 | -3.99 | 0.2066651 | -0.452 |
| ENO1 | ILMN_1710756 | 0.0834 | 0.00622 | -2.91 | -2.5367545 | -0.452 |
| PSMB2 | ILMN_1764794 | 0.129 | 0.0135 | -2.6 | -3.2297083 | -0.452 |
| SLC39A3 | ILMN_2234970 | 0.129 | 0.0136 | -2.6 | -3.2365495 | -0.452 |
| CORO1A | ILMN_1713749 | 0.162 | 0.0202 | -2.43 | -3.5827308 | -0.452 |
| ATXN7L3B | ILMN_1826531 | 0.2 | 0.0296 | -2.27 | -3.9138818 | -0.451 |
| BET1L | ILMN_2060652 | 0.252 | 0.0455 | -2.07 | -4.2824739 | -0.451 |
| QRICH1 | ILMN_1676002 | 0.0658 | 0.00422 | -3.06 | -2.1869812 | -0.45 |
| KXD1 | ILMN_1790951 | 0.0941 | 0.0078 | -2.82 | -2.7399388 | -0.45 |
| SLC15A3 | ILMN_2085862 | 0.00122 | 8.19E-06 | -5.21 | 3.6087601 | -0.449 |
| NFAM1 | ILMN_1791925 | 0.0864 | 0.00664 | -2.88 | -2.5958361 | -0.449 |
| CRY2 | ILMN_1796180 | 0.162 | 0.02 | -2.44 | -3.5738938 | -0.449 |
| EMP3 | ILMN_1765446 | 0.171 | 0.0222 | -2.39 | -3.6642031 | -0.449 |
| LRR1 | ILMN_1778890 | 0.2 | 0.0299 | -2.26 | -3.9230401 | -0.449 |
| VBP1 | ILMN_2223010 | 0.0205 | 0.000629 | -3.75 | -0.4425676 | -0.448 |
| NRDE2 | ILMN_1740165 | 0.218 | 0.0345 | -2.2 | -4.0469569 | -0.448 |
| SF3B2 | ILMN_1775939 | 0.0471 | 0.00244 | -3.26 | -1.6883277 | -0.447 |
| TMED9 | ILMN_1743655 | 0.126 | 0.0131 | -2.61 | -3.1985993 | -0.447 |
| PIN1 | ILMN_1776375 | 0.185 | 0.0258 | -2.33 | -3.7957493 | -0.447 |
| NPHP4 | ILMN_1808417 | 0.207 | 0.0315 | -2.24 | -3.967677 | -0.447 |
| SLC38A10 | ILMN_1759743 | 0.0458 | 0.00229 | -3.29 | -1.629063 | -0.446 |
| VSTM1 | ILMN_1763455 | 0.23 | 0.0384 | -2.15 | -4.1366727 | -0.446 |
| CDK7 | ILMN_1778917 | 0.0193 | 0.000578 | -3.78 | -0.3653978 | -0.445 |
| PARK7 | ILMN_1744713 | 0.00433 | 5.56E-05 | -4.57 | 1.8143592 | -0.444 |
| PHF1 | ILMN_1746968 | 0.00544 | 7.68E-05 | -4.47 | 1.5124137 | -0.444 |
| ATP2A3 | ILMN_1697827 | 0.0959 | 0.00803 | -2.81 | -2.7660591 | -0.444 |
| GAPT | ILMN_1675191 | 0.124 | 0.0127 | -2.62 | -3.1745878 | -0.444 |
| KIAA1429 | ILMN_1813635 | 0.162 | 0.0201 | -2.43 | -3.577723 | -0.444 |
| BSDC1 | ILMN_1734483 | 0.181 | 0.0246 | -2.35 | -3.7547407 | -0.444 |
| RNF4 | ILMN_2142695 | 0.193 | 0.0278 | -2.29 | -3.8605001 | -0.444 |
| NOTCH4 | ILMN_1711157 | 0.254 | 0.0461 | -2.07 | -4.2935284 | -0.443 |
| ZNF189 | ILMN_1806809 | 0.256 | 0.0466 | -2.06 | -4.3017481 | -0.443 |
| LOC643387 | ILMN_3200717 | 0.241 | 0.0415 | -2.12 | -4.2033469 | -0.442 |
| POLR2C | ILMN_1732300 | 0.0812 | 0.00598 | -2.92 | -2.5012596 | -0.441 |
| PTPMT1 | ILMN_3236945 | 0.113 | 0.0108 | -2.69 | -3.0317985 | -0.441 |
| NUP155 | ILMN_1768293 | 0.225 | 0.0368 | -2.17 | -4.1018929 | -0.441 |
| UBE3B | ILMN_1752027 | 0.226 | 0.0372 | -2.16 | -4.1106818 | -0.441 |
| ZNF333 | ILMN_2209299 | 0.245 | 0.0428 | -2.1 | -4.2302839 | -0.441 |
| ZNF333 | ILMN_1759017 | 0.26 | 0.0484 | -2.04 | -4.3346394 | -0.441 |
| PTGES2 | ILMN_2345016 | 0.00373 | 4.37E-05 | -4.65 | 2.0397206 | -0.44 |
| PFN1 | ILMN_1712950 | 0.0106 | 0.000232 | -4.09 | 0.4826235 | -0.44 |
| CTDSP1 | ILMN_1728163 | 0.0384 | 0.00171 | -3.39 | -1.3612647 | -0.44 |
| KCND1 | ILMN_1668586 | 0.0968 | 0.00813 | -2.8 | -2.7774848 | -0.44 |
| BRWD1 | ILMN_1805111 | 0.117 | 0.0114 | -2.67 | -3.0805871 | -0.44 |
| AGO1 | ILMN_1671326 | 0.194 | 0.0279 | -2.29 | -3.8635848 | -0.44 |
| MRPS18C | ILMN_1658416 | 0.119 | 0.0118 | -2.65 | -3.1092063 | -0.439 |
| JMJD8 | ILMN_1687921 | 0.197 | 0.0289 | -2.28 | -3.8926987 | -0.439 |
| PDCD6 | ILMN_1733931 | 0.225 | 0.0368 | -2.17 | -4.1010933 | -0.439 |
| LYRM2 | ILMN_1703132 | 0.256 | 0.0468 | -2.06 | -4.305783 | -0.439 |
| C16orf54 | ILMN_1681032 | 0.0876 | 0.00685 | -2.87 | -2.6229324 | -0.438 |
| TCIRG1 | ILMN_1711994 | 0.0925 | 0.00756 | -2.83 | -2.712473 | -0.438 |
| KIF22 | ILMN_3234884 | 0.147 | 0.0169 | -2.51 | -3.4238793 | -0.438 |
| TESK1 | ILMN_1791067 | 0.222 | 0.0356 | -2.18 | -4.0735981 | -0.438 |
| ATG4A | ILMN_2313782 | 0.0424 | 0.00201 | -3.33 | -1.5125034 | -0.437 |
| ELOF1 | ILMN_1723185 | 0.0426 | 0.00202 | -3.33 | -1.5180937 | -0.437 |
| EIF4A1 | ILMN_3251629 | 0.131 | 0.0139 | -2.59 | -3.2527356 | -0.437 |
| ORC3 | ILMN_1784946 | 0.14 | 0.0157 | -2.54 | -3.3637695 | -0.437 |
| WASH3P | ILMN_1785528 | 0.189 | 0.0268 | -2.31 | -3.8288162 | -0.437 |
| MYBPC3 | ILMN_1781184 | 0.225 | 0.0366 | -2.17 | -4.0964126 | -0.437 |
| PRPF31 | ILMN_1719204 | 0.229 | 0.0381 | -2.15 | -4.1301348 | -0.437 |
| RAB24 | ILMN_2379718 | 0.24 | 0.0412 | -2.12 | -4.1967259 | -0.437 |
| ZNF700 | ILMN_2068435 | 0.245 | 0.0428 | -2.1 | -4.2307994 | -0.437 |
| DENND1C | ILMN_1767020 | 0.129 | 0.0136 | -2.6 | -3.2329574 | -0.436 |
| FAM111A | ILMN_1778845 | 0.167 | 0.0213 | -2.41 | -3.6275014 | -0.436 |
| RPAIN | ILMN_1770339 | 0.238 | 0.0405 | -2.13 | -4.183598 | -0.436 |
| DDA1 | ILMN_1694530 | 0.248 | 0.0441 | -2.09 | -4.2553727 | -0.436 |
| KHNYN | ILMN_1654392 | 0.0928 | 0.00764 | -2.83 | -2.7214975 | -0.435 |
| PPP1CA | ILMN_1695827 | 0.129 | 0.0135 | -2.6 | -3.2304005 | -0.435 |
| ZCCHC9 | ILMN_1723007 | 0.173 | 0.0229 | -2.38 | -3.6909452 | -0.435 |
| NUDT21 | ILMN_1798886 | 0.213 | 0.0331 | -2.22 | -4.0104488 | -0.435 |
| DHX8 | ILMN_1687419 | 0.219 | 0.0349 | -2.19 | -4.0570526 | -0.435 |
| COX5A | ILMN_1704477 | 0.00281 | 2.90E-05 | -4.79 | 2.4245802 | -0.434 |
| OSGEP | ILMN_1753393 | 0.00824 | 0.000151 | -4.24 | 0.8842983 | -0.434 |
| VBP1 | ILMN_1800612 | 0.0182 | 0.000527 | -3.81 | -0.2798249 | -0.434 |
| ADGRG5 | ILMN_1666902 | 0.0336 | 0.00138 | -3.47 | -1.1667626 | -0.434 |
| GPI | ILMN_2173451 | 0.094 | 0.00779 | -2.82 | -2.7388833 | -0.434 |
| ADAM8 | ILMN_1708348 | 0.00168 | 1.35E-05 | -5.04 | 3.1390444 | -0.433 |
| TRIM28 | ILMN_1736575 | 0.00862 | 0.000161 | -4.22 | 0.821275 | -0.433 |
| SIRT2 | ILMN_2398711 | 0.0377 | 0.00166 | -3.4 | -1.3337864 | -0.433 |
| DCUN1D5 | ILMN_2124352 | 0.0745 | 0.00511 | -2.98 | -2.3598652 | -0.433 |
| ITGAL | ILMN_1749591 | 0.0946 | 0.00786 | -2.82 | -2.7471878 | -0.433 |
| ME2 | ILMN_1675186 | 0.1 | 0.00863 | -2.78 | -2.8299666 | -0.433 |
| EIF4G1 | ILMN_1768470 | 0.161 | 0.0198 | -2.44 | -3.5642513 | -0.433 |
| ADM | ILMN_1708934 | 0.00732 | 0.000127 | -4.3 | 1.0437638 | -0.432 |
| SLC4A2 | ILMN_2078389 | 0.0846 | 0.00639 | -2.9 | -2.5609854 | -0.432 |
| RALA | ILMN_1755364 | 0.132 | 0.0141 | -2.58 | -3.2666176 | -0.432 |
| RNF216 | ILMN_1729980 | 0.211 | 0.0326 | -2.22 | -3.9976353 | -0.432 |
| WDR82 | ILMN_1679655 | 0.00554 | 8.02E-05 | -4.45 | 1.4727978 | -0.431 |
| LINC00294 | ILMN_3244521 | 0.108 | 0.0098 | -2.73 | -2.9442447 | -0.431 |
| KIAA1191 | ILMN_2376133 | 0.141 | 0.0159 | -2.53 | -3.371204 | -0.431 |
| PPCDC | ILMN_1704941 | 0.258 | 0.0479 | -2.05 | -4.3242345 | -0.431 |
| NAA38 | ILMN_1691131 | 0.0186 | 0.000547 | -3.8 | -0.3139947 | -0.43 |
| CLN3 | ILMN_2394381 | 0.0279 | 0.001 | -3.58 | -0.8738531 | -0.43 |
| B9D2 | ILMN_1806999 | 0.123 | 0.0125 | -2.63 | -3.1608064 | -0.43 |
| METTL14 | ILMN_2124523 | 0.144 | 0.0164 | -2.52 | -3.4000398 | -0.43 |
| CHFR | ILMN_1653828 | 0.203 | 0.0306 | -2.25 | -3.942037 | -0.43 |
| FERMT3 | ILMN_2366330 | 0.0447 | 0.00219 | -3.3 | -1.5884269 | -0.429 |
| KCNK6 | ILMN_2074773 | 0.147 | 0.0169 | -2.51 | -3.4286733 | -0.429 |
| DVL2 | ILMN_1652163 | 0.153 | 0.0183 | -2.47 | -3.497184 | -0.429 |
| TRIM4 | ILMN_2323385 | 0.172 | 0.0223 | -2.39 | -3.6708458 | -0.429 |
| FBXO46 | ILMN_2350266 | 0.186 | 0.026 | -2.32 | -3.8013198 | -0.429 |
| TMEM189 | ILMN_2162989 | 0.0115 | 0.000259 | -4.06 | 0.3800207 | -0.428 |
| RBMX2 | ILMN_2056551 | 0.103 | 0.00914 | -2.76 | -2.8821758 | -0.428 |
| PAGR1 | ILMN_1727444 | 0.127 | 0.0133 | -2.61 | -3.2146125 | -0.428 |
| TM9SF1 | ILMN_2333440 | 0.204 | 0.0308 | -2.25 | -3.9480028 | -0.428 |
| MCM7 | ILMN_1663195 | 0.213 | 0.0332 | -2.22 | -4.0123402 | -0.428 |
| DPP7 | ILMN_2252309 | 0.216 | 0.034 | -2.2 | -4.03392 | -0.428 |
| CLN3 | ILMN_1669281 | 0.0138 | 0.00035 | -3.95 | 0.1012483 | -0.427 |
| PSMC4 | ILMN_2287888 | 0.0468 | 0.00241 | -3.27 | -1.6778764 | -0.427 |
| G3BP2 | ILMN_2381753 | 0.0486 | 0.00258 | -3.24 | -1.7407854 | -0.427 |
| FKBP2 | ILMN_1807563 | 0.0821 | 0.00608 | -2.92 | -2.516678 | -0.427 |
| PIAS4 | ILMN_1802905 | 0.108 | 0.00992 | -2.72 | -2.954661 | -0.427 |
| RBSN | ILMN_1799890 | 0.11 | 0.0104 | -2.71 | -2.9957459 | -0.427 |
| PHF19 | ILMN_1713249 | 0.122 | 0.0123 | -2.64 | -3.1428661 | -0.427 |
| GGA3 | ILMN_1675982 | 0.158 | 0.0193 | -2.45 | -3.5443322 | -0.427 |
| VAV1 | ILMN_1717334 | 0.165 | 0.0207 | -2.42 | -3.6052521 | -0.427 |
| BCL11A | ILMN_1752899 | 0.231 | 0.0385 | -2.15 | -4.1388158 | -0.427 |
| MRPL54 | ILMN_1658486 | 0.0169 | 0.000472 | -3.85 | -0.1777746 | -0.426 |
| MYADM | ILMN_2308849 | 0.168 | 0.0215 | -2.41 | -3.6357297 | -0.426 |
| UBE3A | ILMN_1764549 | 0.247 | 0.044 | -2.09 | -4.2537633 | -0.426 |
| TRAPPC10 | ILMN_1778464 | 0.0925 | 0.0076 | -2.83 | -2.7170128 | -0.425 |
| CYFIP2 | ILMN_1677200 | 0.108 | 0.00985 | -2.73 | -2.9482048 | -0.425 |
| SYF2 | ILMN_1660186 | 0.115 | 0.0112 | -2.68 | -3.0622263 | -0.425 |
| SLC23A2 | ILMN_1746578 | 0.189 | 0.0268 | -2.31 | -3.828128 | -0.425 |
| GOLGA4 | ILMN_1776297 | 0.192 | 0.0274 | -2.3 | -3.8479914 | -0.425 |
| KANSL3 | ILMN_3187852 | 0.0266 | 0.000937 | -3.61 | -0.8108196 | -0.424 |
| N4BP1 | ILMN_2201966 | 0.0791 | 0.00564 | -2.95 | -2.4490537 | -0.424 |
| GABARAPL2 | ILMN_1796458 | 0.172 | 0.0225 | -2.39 | -3.6766954 | -0.424 |
| ZDHHC5 | ILMN_1679358 | 0.202 | 0.0303 | -2.26 | -3.9349077 | -0.424 |
| SRSF9 | ILMN_1760683 | 0.00401 | 4.97E-05 | -4.61 | 1.9188571 | -0.423 |
| CUX1 | ILMN_2278636 | 0.0168 | 0.000465 | -3.85 | -0.1636739 | -0.423 |
| ZBTB17 | ILMN_1711048 | 0.0466 | 0.00239 | -3.27 | -1.6688588 | -0.423 |
| LRFN3 | ILMN_2103919 | 0.0584 | 0.00343 | -3.14 | -1.9979148 | -0.423 |
| LINC00339 | ILMN_1901198 | 0.0778 | 0.00545 | -2.96 | -2.4184801 | -0.423 |
| TMEM41B | ILMN_1678004 | 0.0835 | 0.00625 | -2.91 | -2.5411508 | -0.423 |
| ULK1 | ILMN_1705144 | 0.19 | 0.027 | -2.31 | -3.833706 | -0.423 |
| SUB1 | ILMN_1736054 | 0.191 | 0.0272 | -2.3 | -3.8408366 | -0.423 |
| RER1 | ILMN_3251317 | 0.205 | 0.031 | -2.25 | -3.9542702 | -0.423 |
| ULK1 | ILMN_1735052 | 0.211 | 0.0325 | -2.22 | -3.9956165 | -0.423 |
| CCDC174 | ILMN_1750144 | 0.253 | 0.0457 | -2.07 | -4.2862597 | -0.423 |
| GTF3C5 | ILMN_1723895 | 0.261 | 0.0487 | -2.04 | -4.3393575 | -0.423 |
| CRAMP1 | ILMN_1660551 | 0.0809 | 0.00594 | -2.93 | -2.4948676 | -0.422 |
| GGA2 | ILMN_1686152 | 0.139 | 0.0155 | -2.54 | -3.3524616 | -0.422 |
| SGTB | ILMN_2109343 | 0.181 | 0.0248 | -2.34 | -3.7596633 | -0.422 |
| HIF1AN | ILMN_1681812 | 0.196 | 0.0286 | -2.28 | -3.884496 | -0.422 |
| ASMTL | ILMN_1804384 | 0.0381 | 0.00169 | -3.4 | -1.3506322 | -0.421 |
| SSBP3 | ILMN_2361163 | 0.225 | 0.0369 | -2.17 | -4.1025572 | -0.421 |
| MAPK8 | ILMN_1849941 | 0.226 | 0.0371 | -2.17 | -4.108574 | -0.421 |
| IFNGR2 | ILMN_1764964 | 0.000473 | 1.76E-06 | -5.71 | 5.052473 | -0.42 |
| PSMB4 | ILMN_1737862 | 0.0858 | 0.00658 | -2.89 | -2.5869297 | -0.42 |
| LUC7L | ILMN_1765371 | 0.0902 | 0.0072 | -2.85 | -2.6688394 | -0.42 |
| G3BP2 | ILMN_1720422 | 0.113 | 0.0109 | -2.69 | -3.0371874 | -0.42 |
| BAK1 | ILMN_1805990 | 0.157 | 0.0191 | -2.46 | -3.5328046 | -0.42 |
| TRIM39 | ILMN_2413517 | 0.158 | 0.0193 | -2.45 | -3.5426892 | -0.42 |
| KLHL36 | ILMN_1703314 | 0.186 | 0.0258 | -2.33 | -3.7971758 | -0.42 |
| MED14 | ILMN_3246388 | 0.24 | 0.0413 | -2.12 | -4.1989567 | -0.42 |
| UBE2M | ILMN_1701331 | 0.0449 | 0.0022 | -3.3 | -1.5954868 | -0.419 |
| LARP1B | ILMN_1765212 | 0.0956 | 0.00799 | -2.81 | -2.7620322 | -0.419 |
| ZNF263 | ILMN_1692620 | 0.104 | 0.00929 | -2.75 | -2.8960996 | -0.419 |
| ICOSLG | ILMN_1675671 | 0.204 | 0.0308 | -2.25 | -3.9489399 | -0.419 |
| SDHD | ILMN_1698487 | 0.238 | 0.0407 | -2.12 | -4.1868463 | -0.419 |
| TMEM8A | ILMN_1741371 | 0.0208 | 0.000645 | -3.74 | -0.4654835 | -0.418 |
| POLR1D | ILMN_1742427 | 0.0269 | 0.00095 | -3.6 | -0.8238932 | -0.418 |
| DAP | ILMN_2112493 | 0.0302 | 0.00114 | -3.54 | -0.9882935 | -0.418 |
| RRP8 | ILMN_2066667 | 0.0795 | 0.00569 | -2.94 | -2.455958 | -0.418 |
| SCO2 | ILMN_1701621 | 0.0889 | 0.00703 | -2.86 | -2.6464228 | -0.418 |
| TERF1 | ILMN_1694847 | 0.0181 | 0.000521 | -3.81 | -0.2684111 | -0.417 |
| TNNT3 | ILMN_1720231 | 0.112 | 0.0106 | -2.7 | -3.0163116 | -0.416 |
| PLPPR2 | ILMN_1667319 | 0.119 | 0.0118 | -2.65 | -3.1096209 | -0.416 |
| TCP11L1 | ILMN_1713174 | 0.148 | 0.0172 | -2.5 | -3.4414987 | -0.416 |
| TPRKB | ILMN_1690307 | 0.149 | 0.0173 | -2.5 | -3.4474844 | -0.416 |
| SNRPC | ILMN_1741997 | 0.198 | 0.0292 | -2.27 | -3.9017764 | -0.416 |
| RPS6KB2 | ILMN_1761175 | 0.019 | 0.000568 | -3.78 | -0.3489885 | -0.415 |
| R3HCC1 | ILMN_1735474 | 0.108 | 0.00981 | -2.73 | -2.9445643 | -0.415 |
| CNIH1 | ILMN_1903568 | 0.256 | 0.0469 | -2.06 | -4.3073755 | -0.414 |
| AXIN1 | ILMN_1766185 | 0.00452 | 5.87E-05 | -4.56 | 1.7635699 | -0.413 |
| CCT7 | ILMN_1703718 | 0.0434 | 0.00209 | -3.32 | -1.5465523 | -0.413 |
| NMD3 | ILMN_2232430 | 0.071 | 0.00473 | -3.01 | -2.2908041 | -0.413 |
| GTSF1 | ILMN_2069632 | 0.091 | 0.0073 | -2.85 | -2.6810303 | -0.413 |
| ATP6V0B | ILMN_1721391 | 0.196 | 0.0284 | -2.28 | -3.8790439 | -0.413 |
| PFN1 | ILMN_2151817 | 0.000101 | 1.38E-07 | -6.54 | 7.4365747 | -0.412 |
| FAM96A | ILMN_1761981 | 0.0118 | 0.00027 | -4.04 | 0.3403486 | -0.412 |
| ATF4 | ILMN_1672128 | 0.0577 | 0.00337 | -3.14 | -1.9831981 | -0.412 |
| NAA35 | ILMN_1810826 | 0.0667 | 0.00432 | -3.05 | -2.2078491 | -0.412 |
| LINC00672 | ILMN_1916702 | 0.0911 | 0.00733 | -2.84 | -2.6846736 | -0.412 |
| KAT2A | ILMN_1782247 | 0.104 | 0.00924 | -2.75 | -2.8918801 | -0.412 |
| EIF4EBP1 | ILMN_1767324 | 0.211 | 0.0325 | -2.22 | -3.9957157 | -0.412 |
| TRAPPC2L | ILMN_1747058 | 0.00937 | 0.000187 | -4.17 | 0.6846692 | -0.411 |
| TRIM39 | ILMN_1736562 | 0.0271 | 0.000962 | -3.6 | -0.8350309 | -0.411 |
| SIPA1 | ILMN_2415536 | 0.047 | 0.00243 | -3.26 | -1.6849617 | -0.411 |
| TNRC6C | ILMN_1746871 | 0.199 | 0.0293 | -2.27 | -3.9068935 | -0.411 |
| POLG2 | ILMN_1671004 | 0.243 | 0.0421 | -2.11 | -4.2151926 | -0.411 |
| TSC1 | ILMN_1797367 | 0.252 | 0.0455 | -2.07 | -4.2817536 | -0.411 |
| MTIF3 | ILMN_1718271 | 0.258 | 0.0478 | -2.05 | -4.3230648 | -0.411 |
| CYLD | ILMN_1775508 | 0.125 | 0.0128 | -2.62 | -3.1783245 | -0.41 |
| DOCK10 | ILMN_1702301 | 0.146 | 0.0168 | -2.51 | -3.4213164 | -0.41 |
| IVNS1ABP | ILMN_1717877 | 0.0458 | 0.0023 | -3.28 | -1.6328413 | -0.409 |
| CD82 | ILMN_1662973 | 0.0552 | 0.00313 | -3.17 | -1.9166823 | -0.409 |
| PRDM2 | ILMN_2258543 | 0.0341 | 0.00141 | -3.46 | -1.1897255 | -0.408 |
| SPPL2A | ILMN_1734229 | 0.0484 | 0.00256 | -3.24 | -1.7337831 | -0.408 |
| BAP1 | ILMN_1768363 | 0.0865 | 0.00666 | -2.88 | -2.5987054 | -0.408 |
| ME2 | ILMN_2048636 | 0.119 | 0.0117 | -2.66 | -3.1039801 | -0.408 |
| DHPS | ILMN_1752967 | 0.245 | 0.0431 | -2.1 | -4.236604 | -0.408 |
| LAMTOR3 | ILMN_1659415 | 0.00158 | 1.24E-05 | -5.07 | 3.2173841 | -0.407 |
| CBY1 | ILMN_1730879 | 0.0291 | 0.00107 | -3.56 | -0.9352665 | -0.407 |
| PFDN1 | ILMN_1717855 | 0.138 | 0.0153 | -2.55 | -3.3379882 | -0.407 |
| VPS37C | ILMN_1789233 | 0.153 | 0.0183 | -2.47 | -3.4956268 | -0.407 |
| AHSA1 | ILMN_1703617 | 0.0575 | 0.00335 | -3.14 | -1.9775286 | -0.405 |
| NUP54 | ILMN_1771835 | 0.0847 | 0.00641 | -2.9 | -2.5635985 | -0.405 |
| HMGN1 | ILMN_2151579 | 0.133 | 0.0143 | -2.58 | -3.2797988 | -0.405 |
| NRBP1 | ILMN_1670096 | 0.139 | 0.0154 | -2.54 | -3.3468491 | -0.405 |
| DLGAP4 | ILMN_1754842 | 0.142 | 0.0161 | -2.53 | -3.3822319 | -0.405 |
| SLC37A4 | ILMN_1678678 | 0.161 | 0.0198 | -2.44 | -3.5633105 | -0.405 |
| CCT7 | ILMN_2341793 | 0.172 | 0.0223 | -2.39 | -3.6705762 | -0.405 |
| SLC30A9 | ILMN_1799128 | 0.225 | 0.0366 | -2.17 | -4.0961974 | -0.405 |
| REV1 | ILMN_2395474 | 0.253 | 0.0457 | -2.07 | -4.2844766 | -0.405 |
| COL4A3BP | ILMN_1680109 | 0.0447 | 0.00219 | -3.3 | -1.588056 | -0.404 |
| CHCHD5 | ILMN_1797530 | 0.1 | 0.00861 | -2.78 | -2.8289003 | -0.404 |
| MFSD2A | ILMN_1789638 | 0.109 | 0.0101 | -2.72 | -2.9702482 | -0.404 |
| CCDC25 | ILMN_1789266 | 0.131 | 0.0138 | -2.59 | -3.2508705 | -0.404 |
| USPL1 | ILMN_1662686 | 0.0458 | 0.00228 | -3.29 | -1.6273048 | -0.403 |
| NADSYN1 | ILMN_1779034 | 0.0554 | 0.00317 | -3.16 | -1.9281185 | -0.403 |
| DPP9 | ILMN_1673069 | 0.0597 | 0.00355 | -3.12 | -2.0289366 | -0.403 |
| ZGPAT | ILMN_2344079 | 0.184 | 0.0253 | -2.33 | -3.77886 | -0.403 |
| MED31 | ILMN_3251451 | 0.185 | 0.0256 | -2.33 | -3.7900227 | -0.403 |
| CSRNP2 | ILMN_3249110 | 0.213 | 0.0331 | -2.22 | -4.0104909 | -0.403 |
| SLC35A4 | ILMN_2105966 | 0.255 | 0.0463 | -2.06 | -4.297116 | -0.403 |
| MTMR12 | ILMN_1802831 | 0.0463 | 0.00234 | -3.28 | -1.6487701 | -0.402 |
| RAVER1 | ILMN_1740395 | 0.0766 | 0.00532 | -2.97 | -2.3968439 | -0.402 |
| PEX13 | ILMN_1683916 | 0.101 | 0.00869 | -2.78 | -2.8365681 | -0.402 |
| POLR2H | ILMN_1689445 | 0.116 | 0.0112 | -2.67 | -3.0653867 | -0.402 |
| SEC23B | ILMN_1657483 | 0.0649 | 0.0041 | -3.07 | -2.1610815 | -0.401 |
| RPIA | ILMN_1714809 | 0.133 | 0.0143 | -2.58 | -3.2769117 | -0.4 |
| PRRC2A | ILMN_2408179 | 0.16 | 0.0197 | -2.44 | -3.5586863 | -0.4 |
| SSBP3 | ILMN_1814165 | 0.195 | 0.0283 | -2.29 | -3.876676 | -0.4 |
| NCKAP1L | ILMN_1674250 | 0.0211 | 0.000662 | -3.73 | -0.4903821 | -0.399 |
| ITGB2 | ILMN_1654396 | 0.0524 | 0.00289 | -3.2 | -1.8430338 | -0.399 |
| IMP3 | ILMN_1733696 | 0.091 | 0.00731 | -2.85 | -2.6820976 | -0.399 |
| TXNDC15 | ILMN_1662848 | 0.0994 | 0.00848 | -2.79 | -2.8151648 | -0.399 |
| MAP4K2 | ILMN_1723625 | 0.142 | 0.016 | -2.53 | -3.3784015 | -0.399 |
| CCDC130 | ILMN_1758633 | 0.198 | 0.0291 | -2.27 | -3.898988 | -0.399 |
| ARID3B | ILMN_1696956 | 0.248 | 0.0443 | -2.08 | -4.2593135 | -0.399 |
| SMC3 | ILMN_1718807 | 0.0927 | 0.00762 | -2.83 | -2.7195199 | -0.398 |
| COMMD2 | ILMN_1683305 | 0.121 | 0.0121 | -2.64 | -3.1326398 | -0.398 |
| PSMB1 | ILMN_1789176 | 0.0336 | 0.00138 | -3.47 | -1.169436 | -0.397 |
| ZFYVE19 | ILMN_1780698 | 0.0381 | 0.00169 | -3.4 | -1.3521359 | -0.397 |
| SGF29 | ILMN_1684789 | 0.11 | 0.0104 | -2.71 | -2.9945718 | -0.397 |
| RABL3 | ILMN_1662306 | 0.238 | 0.0407 | -2.12 | -4.1867514 | -0.397 |
| ARHGAP30 | ILMN_1750805 | 0.0227 | 0.000725 | -3.7 | -0.5746659 | -0.396 |
| TERF2 | ILMN_1768488 | 0.0854 | 0.00651 | -2.89 | -2.5775303 | -0.396 |
| STOML2 | ILMN_1663002 | 0.211 | 0.0325 | -2.22 | -3.9959018 | -0.396 |
| TTF1 | ILMN_1713129 | 0.213 | 0.0331 | -2.22 | -4.0117375 | -0.396 |
| CRIPT | ILMN_1813256 | 0.218 | 0.0347 | -2.2 | -4.0498488 | -0.396 |
| RNASEH1 | ILMN_1726783 | 0.244 | 0.0426 | -2.1 | -4.2260239 | -0.396 |
| ZNF544 | ILMN_1797903 | 0.243 | 0.0421 | -2.11 | -4.2151993 | -0.395 |
| GORASP1 | ILMN_1716821 | 0.259 | 0.0481 | -2.05 | -4.3285492 | -0.395 |
| XAB2 | ILMN_1771057 | 0.00668 | 0.00011 | -4.35 | 1.1812101 | -0.394 |
| C1orf174 | ILMN_3236244 | 0.0278 | 0.000996 | -3.59 | -0.8668582 | -0.394 |
| TFB2M | ILMN_2067708 | 0.0874 | 0.00681 | -2.87 | -2.6183724 | -0.394 |
| NUP160 | ILMN_1652989 | 0.11 | 0.0104 | -2.71 | -2.9935957 | -0.394 |
| DHX38 | ILMN_1735679 | 0.111 | 0.0105 | -2.7 | -3.0041839 | -0.394 |
| EIF2B4 | ILMN_2356672 | 0.113 | 0.0108 | -2.69 | -3.0327929 | -0.394 |
| AAR2 | ILMN_1721225 | 0.15 | 0.0178 | -2.49 | -3.4709108 | -0.394 |
| NLRP12 | ILMN_1758735 | 0.224 | 0.0365 | -2.17 | -4.0932834 | -0.394 |
| TBC1D20 | ILMN_2044572 | 0.0376 | 0.00164 | -3.41 | -1.3279911 | -0.393 |
| ATP23 | ILMN_1767481 | 0.0845 | 0.00637 | -2.9 | -2.557844 | -0.393 |
| SAT2 | ILMN_1746883 | 0.213 | 0.0334 | -2.21 | -4.018931 | -0.393 |
| LCLAT1 | ILMN_3186473 | 0.226 | 0.0372 | -2.16 | -4.1101777 | -0.393 |
| RNF5 | ILMN_2044927 | 0.233 | 0.0392 | -2.14 | -4.1550837 | -0.393 |
| RBM34 | ILMN_1661485 | 0.24 | 0.0412 | -2.12 | -4.1977132 | -0.393 |
| XRCC6 | ILMN_2166506 | 0.0266 | 0.000935 | -3.61 | -0.8089373 | -0.392 |
| ZNF226 | ILMN_2363273 | 0.108 | 0.01 | -2.72 | -2.9615656 | -0.392 |
| ALKBH6 | ILMN_1808792 | 0.219 | 0.0349 | -2.19 | -4.0564361 | -0.392 |
| SLC10A3 | ILMN_1712887 | 0.231 | 0.0385 | -2.15 | -4.1396842 | -0.392 |
| MEAF6 | ILMN_2076940 | 0.129 | 0.0136 | -2.6 | -3.2349427 | -0.391 |
| RAB32 | ILMN_2115434 | 0.142 | 0.016 | -2.53 | -3.380382 | -0.391 |
| MUTYH | ILMN_1714438 | 0.145 | 0.0166 | -2.51 | -3.4092386 | -0.391 |
| MLH1 | ILMN_1788363 | 0.206 | 0.0313 | -2.24 | -3.9624379 | -0.391 |
| EED | ILMN_2347917 | 0.221 | 0.0353 | -2.19 | -4.0661924 | -0.391 |
| EIF5A | ILMN_1794522 | 0.0575 | 0.00335 | -3.14 | -1.9783271 | -0.39 |
| SEC23B | ILMN_2366246 | 0.0972 | 0.0082 | -2.8 | -2.7848929 | -0.39 |
| STRADA | ILMN_2348243 | 0.139 | 0.0154 | -2.55 | -3.3421981 | -0.39 |
| SCAMP2 | ILMN_1654893 | 0.197 | 0.0289 | -2.28 | -3.8926562 | -0.39 |
| BAGE2 | ILMN_2168766 | 0.222 | 0.0356 | -2.18 | -4.0740242 | -0.39 |
| RABGAP1 | ILMN_2061950 | 0.0392 | 0.00176 | -3.38 | -1.3905747 | -0.389 |
| SLC35A2 | ILMN_1742731 | 0.0462 | 0.00232 | -3.28 | -1.642316 | -0.389 |
| ITGB2 | ILMN_2175912 | 0.0497 | 0.00267 | -3.23 | -1.7699298 | -0.389 |
| AKT1 | ILMN_2410909 | 0.0515 | 0.00282 | -3.21 | -1.8216899 | -0.389 |
| BOD1L1 | ILMN_1695856 | 0.26 | 0.0485 | -2.04 | -4.3357681 | -0.389 |
| VPS41 | ILMN_1703379 | 0.0695 | 0.00461 | -3.02 | -2.2660713 | -0.388 |
| ICAM3 | ILMN_2212763 | 0.161 | 0.0198 | -2.44 | -3.5637487 | -0.388 |
| TIMP2 | ILMN_1721876 | 0.199 | 0.0295 | -2.27 | -3.9101614 | -0.388 |
| APTX | ILMN_2317348 | 0.161 | 0.0198 | -2.44 | -3.5640991 | -0.387 |
| SPTY2D1 | ILMN_1728699 | 0.162 | 0.0203 | -2.43 | -3.5854869 | -0.387 |
| BRD7 | ILMN_2082810 | 0.239 | 0.041 | -2.12 | -4.1929456 | -0.387 |
| RNF40 | ILMN_1808712 | 0.0404 | 0.00186 | -3.36 | -1.4398222 | -0.386 |
| TMED4 | ILMN_1804148 | 0.0796 | 0.00571 | -2.94 | -2.4601044 | -0.386 |
| MAP1S | ILMN_1796336 | 0.154 | 0.0184 | -2.47 | -3.4993441 | -0.386 |
| PRKRIP1 | ILMN_1804490 | 0.246 | 0.0434 | -2.09 | -4.2412503 | -0.386 |
| PTK2B | ILMN_2330966 | 0.00475 | 6.25E-05 | -4.54 | 1.705066 | -0.385 |
| DNAJC4 | ILMN_3307827 | 0.0609 | 0.00367 | -3.11 | -2.0591821 | -0.385 |
| TMEM184C | ILMN_3237779 | 0.167 | 0.0212 | -2.41 | -3.6237056 | -0.385 |
| HGS | ILMN_1715994 | 0.197 | 0.0289 | -2.28 | -3.8932607 | -0.385 |
| NCOA5 | ILMN_1770035 | 0.251 | 0.0452 | -2.08 | -4.2761874 | -0.385 |
| ZNF384 | ILMN_1764891 | 0.0285 | 0.00104 | -3.57 | -0.9050907 | -0.384 |
| CFAP70 | ILMN_1784516 | 0.0675 | 0.00439 | -3.04 | -2.2229219 | -0.384 |
| GRK2 | ILMN_3307926 | 0.0183 | 0.000532 | -3.81 | -0.2873555 | -0.383 |
| C1orf122 | ILMN_1786273 | 0.108 | 0.00991 | -2.72 | -2.953682 | -0.383 |
| SGF29 | ILMN_1701477 | 0.172 | 0.0226 | -2.38 | -3.6802606 | -0.383 |
| VIPAS39 | ILMN_1734440 | 0.222 | 0.0356 | -2.18 | -4.0723062 | -0.383 |
| C19orf53 | ILMN_1671374 | 0.026 | 0.000906 | -3.62 | -0.7794329 | -0.382 |
| MARS | ILMN_1799819 | 0.0454 | 0.00225 | -3.29 | -1.612728 | -0.382 |
| DDX56 | ILMN_1679405 | 0.102 | 0.00889 | -2.77 | -2.8574739 | -0.382 |
| TICAM1 | ILMN_1815079 | 0.213 | 0.0333 | -2.21 | -4.0149308 | -0.382 |
| BFAR | ILMN_1814808 | 0.222 | 0.0358 | -2.18 | -4.078717 | -0.382 |
| ATP2A2 | ILMN_1687375 | 0.226 | 0.0369 | -2.17 | -4.1046499 | -0.382 |
| GMPPB | ILMN_2412927 | 0.245 | 0.0432 | -2.1 | -4.237559 | -0.382 |
| YY1 | ILMN_1770892 | 0.00937 | 0.000186 | -4.17 | 0.6859551 | -0.381 |
| MTMR14 | ILMN_2410262 | 0.024 | 0.000789 | -3.67 | -0.6518331 | -0.381 |
| PPHLN1 | ILMN_1791093 | 0.0538 | 0.00302 | -3.18 | -1.882775 | -0.381 |
| SQRDL | ILMN_1667199 | 0.0715 | 0.00477 | -3.01 | -2.2977772 | -0.381 |
| ASMTL | ILMN_1689786 | 0.151 | 0.0179 | -2.48 | -3.4780542 | -0.381 |
| MTERF1 | ILMN_2099249 | 0.165 | 0.0208 | -2.42 | -3.6080257 | -0.381 |
| CLCN7 | ILMN_1694731 | 0.264 | 0.0496 | -2.03 | -4.3543007 | -0.381 |
| SUZ12 | ILMN_1797813 | 0.0653 | 0.00417 | -3.06 | -2.1751609 | -0.38 |
| SNUPN | ILMN_2364535 | 0.0805 | 0.00588 | -2.93 | -2.4867778 | -0.38 |
| NUMA1 | ILMN_1757085 | 0.127 | 0.0131 | -2.61 | -3.1996514 | -0.38 |
| MIOS | ILMN_1680644 | 0.205 | 0.0312 | -2.24 | -3.9584622 | -0.38 |
| INO80B | ILMN_1779040 | 0.219 | 0.0348 | -2.19 | -4.0528527 | -0.38 |
| MEI1 | ILMN_1810254 | 0.164 | 0.0206 | -2.42 | -3.5985979 | -0.379 |
| UBE2Z | ILMN_1692168 | 0.165 | 0.0208 | -2.42 | -3.609169 | -0.379 |
| COPS7B | ILMN_1784785 | 0.187 | 0.0263 | -2.32 | -3.8108267 | -0.379 |
| HCP5 | ILMN_1803945 | 0.21 | 0.0323 | -2.23 | -3.990516 | -0.379 |
| NOSIP | ILMN_1759436 | 0.217 | 0.0342 | -2.2 | -4.0380769 | -0.379 |
| ZNF513 | ILMN_1791069 | 0.264 | 0.0499 | -2.03 | -4.360059 | -0.379 |
| BCL2L13 | ILMN_2181445 | 0.0136 | 0.000343 | -3.96 | 0.1185123 | -0.378 |
| DEPDC5 | ILMN_1658717 | 0.0409 | 0.0019 | -3.35 | -1.4611812 | -0.378 |
| FAM214B | ILMN_1732609 | 0.0458 | 0.00228 | -3.29 | -1.6261269 | -0.378 |
| WDFY1 | ILMN_1676448 | 0.126 | 0.013 | -2.61 | -3.195661 | -0.378 |
| ZBTB24 | ILMN_1659937 | 0.136 | 0.0149 | -2.56 | -3.312562 | -0.378 |
| B4GALT3 | ILMN_1692267 | 0.196 | 0.0286 | -2.28 | -3.8853549 | -0.378 |
| ARHGAP30 | ILMN_2408851 | 0.029 | 0.00107 | -3.56 | -0.9288406 | -0.377 |
| FBRS | ILMN_3245236 | 0.0306 | 0.00116 | -3.53 | -1.0086135 | -0.377 |
| CSK | ILMN_1754121 | 0.0319 | 0.00125 | -3.51 | -1.0745364 | -0.376 |
| IFT52 | ILMN_1673172 | 0.0644 | 0.00406 | -3.07 | -2.1516244 | -0.376 |
| ARMT1 | ILMN_1798108 | 0.122 | 0.0123 | -2.64 | -3.1446553 | -0.376 |
| PRKAB1 | ILMN_1793522 | 0.174 | 0.0231 | -2.37 | -3.6990771 | -0.376 |
| NFATC2IP | ILMN_2379080 | 0.101 | 0.00877 | -2.77 | -2.8444301 | -0.375 |
| SF3B4 | ILMN_1722648 | 0.107 | 0.00974 | -2.73 | -2.9384452 | -0.375 |
| TEN1 | ILMN_3239959 | 0.119 | 0.0117 | -2.66 | -3.1029072 | -0.375 |
| PTPA | ILMN_1729123 | 0.239 | 0.0408 | -2.12 | -4.1887003 | -0.375 |
| ANAPC11 | ILMN_1722102 | 0.0248 | 0.000836 | -3.65 | -0.7052849 | -0.374 |
| SPOCK2 | ILMN_1656287 | 0.0407 | 0.00188 | -3.36 | -1.4488516 | -0.374 |
| PTK2B | ILMN_1732318 | 0.0458 | 0.00227 | -3.29 | -1.6238887 | -0.374 |
| PTGS1 | ILMN_2339835 | 0.124 | 0.0127 | -2.63 | -3.1711215 | -0.374 |
| MRPL10 | ILMN_2396002 | 0.157 | 0.019 | -2.46 | -3.5293888 | -0.374 |
| PCIF1 | ILMN_2232368 | 0.168 | 0.0214 | -2.41 | -3.6320665 | -0.374 |
| CSF1R | ILMN_1686623 | 0.21 | 0.0323 | -2.23 | -3.9883949 | -0.374 |
| FERMT3 | ILMN_2366334 | 0.246 | 0.0435 | -2.09 | -4.2431951 | -0.374 |
| FNBP1 | ILMN_1797342 | 0.0454 | 0.00224 | -3.29 | -1.6095248 | -0.373 |
| TBC1D3C | ILMN_1693802 | 0.0885 | 0.00698 | -2.86 | -2.6406986 | -0.373 |
| C6orf1 | ILMN_2370685 | 0.121 | 0.0121 | -2.64 | -3.1335983 | -0.373 |
| RAB4B | ILMN_2109994 | 0.161 | 0.0199 | -2.44 | -3.5698618 | -0.373 |
| ARPC5L | ILMN_2043816 | 0.249 | 0.0446 | -2.08 | -4.2655198 | -0.373 |
| TVP23B | ILMN_1661593 | 0.261 | 0.049 | -2.04 | -4.3441134 | -0.373 |
| SRPRA | ILMN_1785660 | 0.0462 | 0.00233 | -3.28 | -1.6453396 | -0.372 |
| C16orf74 | ILMN_1806149 | 0.106 | 0.00958 | -2.74 | -2.9238691 | -0.372 |
| HCST | ILMN_1699931 | 0.137 | 0.0151 | -2.55 | -3.3246124 | -0.372 |
| NEDD4 | ILMN_1703140 | 0.163 | 0.0205 | -2.43 | -3.5943962 | -0.372 |
| BAG5 | ILMN_2361695 | 0.17 | 0.0221 | -2.39 | -3.6593493 | -0.372 |
| SDF2 | ILMN_1713978 | 0.00952 | 0.000191 | -4.16 | 0.6628119 | -0.371 |
| PIPSL | ILMN_1676763 | 0.0762 | 0.00528 | -2.97 | -2.3883859 | -0.371 |
| ZSCAN9 | ILMN_1669516 | 0.176 | 0.0235 | -2.37 | -3.7155268 | -0.371 |
| ARMC10 | ILMN_2052598 | 0.185 | 0.0256 | -2.33 | -3.7905965 | -0.371 |
| STIP1 | ILMN_1789510 | 0.227 | 0.0375 | -2.16 | -4.1173065 | -0.371 |
| ZNF562 | ILMN_1672940 | 0.232 | 0.039 | -2.14 | -4.1504278 | -0.371 |
| GFOD2 | ILMN_1744006 | 0.238 | 0.0406 | -2.12 | -4.1856144 | -0.371 |
| FAM110A | ILMN_1782095 | 0.242 | 0.0418 | -2.11 | -4.2101048 | -0.371 |
| SNRPC | ILMN_2086064 | 0.0347 | 0.00146 | -3.45 | -1.2163257 | -0.37 |
| CRTC2 | ILMN_1657771 | 0.0902 | 0.00722 | -2.85 | -2.6702096 | -0.37 |
| TIMM17B | ILMN_1813260 | 0.0911 | 0.00732 | -2.84 | -2.6830546 | -0.37 |
| HMGCL | ILMN_2122420 | 0.236 | 0.0399 | -2.13 | -4.1701048 | -0.37 |
| ALDOA | ILMN_1741148 | 0.0427 | 0.00204 | -3.33 | -1.5235473 | -0.369 |
| UTP23 | ILMN_1774860 | 0.174 | 0.0232 | -2.37 | -3.7031874 | -0.369 |
| NCF1B | ILMN_2106725 | 0.232 | 0.039 | -2.14 | -4.1510862 | -0.369 |
| SERTAD2 | ILMN_1651347 | 0.247 | 0.0438 | -2.09 | -4.2490884 | -0.369 |
| TFIP11 | ILMN_2408102 | 0.0225 | 0.000714 | -3.7 | -0.5604038 | -0.368 |
| SIK3 | ILMN_1732343 | 0.0358 | 0.00152 | -3.43 | -1.2568777 | -0.368 |
| TRMT5 | ILMN_2167922 | 0.11 | 0.0103 | -2.71 | -2.9869807 | -0.368 |
| MED6 | ILMN_1654543 | 0.161 | 0.0198 | -2.44 | -3.5667079 | -0.368 |
| PARVG | ILMN_1695851 | 0.2 | 0.0296 | -2.27 | -3.9140158 | -0.368 |
| RBM33 | ILMN_1741244 | 0.237 | 0.0401 | -2.13 | -4.1742317 | -0.368 |
| IFI30 | ILMN_1807277 | 0.0627 | 0.0039 | -3.09 | -2.1142106 | -0.367 |
| NBPF9 | ILMN_2115490 | 0.0644 | 0.00405 | -3.07 | -2.1503466 | -0.367 |
| RNF34 | ILMN_1786039 | 0.0648 | 0.00408 | -3.07 | -2.1567654 | -0.367 |
| PTPN11 | ILMN_1778236 | 0.0902 | 0.00718 | -2.85 | -2.666326 | -0.367 |
| CMTM3 | ILMN_1705442 | 0.243 | 0.0422 | -2.11 | -4.2176638 | -0.367 |
| ATG4D | ILMN_1801905 | 0.259 | 0.0481 | -2.05 | -4.3278186 | -0.367 |
| COX4I1 | ILMN_1652207 | 0.0604 | 0.00362 | -3.12 | -2.0464246 | -0.366 |
| AZIN1 | ILMN_2327994 | 0.122 | 0.0122 | -2.64 | -3.1405509 | -0.366 |
| MED10 | ILMN_1707631 | 0.14 | 0.0158 | -2.54 | -3.3648149 | -0.366 |
| SEPT2 | ILMN_1748546 | 0.157 | 0.0191 | -2.45 | -3.5339226 | -0.366 |
| GPBP1L1 | ILMN_1662719 | 0.169 | 0.0216 | -2.4 | -3.6431365 | -0.366 |
| CHD8 | ILMN_1806122 | 0.175 | 0.0232 | -2.37 | -3.7049216 | -0.366 |
| ZDHHC23 | ILMN_1736901 | 0.18 | 0.0245 | -2.35 | -3.7522725 | -0.366 |
| PSAP | ILMN_1749109 | 0.00233 | 2.27E-05 | -4.87 | 2.6515922 | -0.365 |
| MRPL28 | ILMN_1694950 | 0.19 | 0.027 | -2.31 | -3.8346162 | -0.365 |
| GIN1 | ILMN_1814622 | 0.189 | 0.0268 | -2.31 | -3.8279697 | -0.364 |
| DOCK4 | ILMN_1801044 | 0.25 | 0.0448 | -2.08 | -4.2692134 | -0.364 |
| UQCR10 | ILMN_2366710 | 0.26 | 0.0485 | -2.04 | -4.3352488 | -0.364 |
| EIF4E2 | ILMN_1738326 | 0.0311 | 0.0012 | -3.52 | -1.0413672 | -0.363 |
| PRKCD | ILMN_1801105 | 0.0323 | 0.00128 | -3.5 | -1.1012607 | -0.363 |
| GPX4 | ILMN_2378952 | 0.0336 | 0.00139 | -3.47 | -1.1708709 | -0.363 |
| ARHGEF1 | ILMN_1772370 | 0.112 | 0.0106 | -2.7 | -3.0171389 | -0.363 |
| GUK1 | ILMN_1758398 | 0.131 | 0.0139 | -2.59 | -3.2554405 | -0.363 |
| TM2D1 | ILMN_2143148 | 0.148 | 0.0171 | -2.5 | -3.4376699 | -0.363 |
| RELA | ILMN_1705266 | 0.258 | 0.0476 | -2.05 | -4.3199216 | -0.363 |
| GMEB1 | ILMN_1727761 | 0.0285 | 0.00104 | -3.57 | -0.904707 | -0.362 |
| PHF1 | ILMN_2385866 | 0.0507 | 0.00273 | -3.22 | -1.7907323 | -0.362 |
| SAE1 | ILMN_1657204 | 0.0564 | 0.00325 | -3.16 | -1.9498071 | -0.362 |
| OSTM1 | ILMN_1720303 | 0.112 | 0.0106 | -2.7 | -3.015102 | -0.362 |
| DDX41 | ILMN_1737344 | 0.26 | 0.0486 | -2.04 | -4.3367326 | -0.362 |
| GNB1 | ILMN_1760320 | 0.0101 | 0.000208 | -4.13 | 0.5833798 | -0.361 |
| LTC4S | ILMN_1668247 | 0.0257 | 0.000886 | -3.63 | -0.7589461 | -0.361 |
| CSNK1G2 | ILMN_1706521 | 0.113 | 0.0109 | -2.69 | -3.0395297 | -0.361 |
| RBBP6 | ILMN_1665135 | 0.205 | 0.0311 | -2.24 | -3.9568656 | -0.361 |
| SEC22C | ILMN_1664051 | 0.231 | 0.0384 | -2.15 | -4.1373979 | -0.361 |
| LAMTOR4 | ILMN_1779735 | 0.263 | 0.0494 | -2.03 | -4.3507934 | -0.361 |
| PPP4C | ILMN_3248975 | 0.0319 | 0.00125 | -3.51 | -1.0738352 | -0.36 |
| CHD4 | ILMN_1658411 | 0.126 | 0.013 | -2.61 | -3.1960189 | -0.36 |
| SYNRG | ILMN_2415467 | 0.193 | 0.0277 | -2.3 | -3.8564173 | -0.36 |
| DHDDS | ILMN_2405642 | 0.0104 | 0.000223 | -4.11 | 0.5169402 | -0.359 |
| PDLIM7 | ILMN_1814985 | 0.124 | 0.0126 | -2.63 | -3.1696799 | -0.359 |
| OTUB1 | ILMN_1723871 | 0.125 | 0.0128 | -2.62 | -3.1784118 | -0.359 |
| HIST1H3D | ILMN_1721127 | 0.213 | 0.0331 | -2.22 | -4.0115434 | -0.359 |
| PSMA4 | ILMN_2198376 | 0.0797 | 0.00573 | -2.94 | -2.4634426 | -0.358 |
| MAML1 | ILMN_1803060 | 0.226 | 0.0369 | -2.17 | -4.1034439 | -0.358 |
| NAA60 | ILMN_1813423 | 0.235 | 0.0397 | -2.14 | -4.1651722 | -0.358 |
| DHX33 | ILMN_1744308 | 0.108 | 0.00982 | -2.73 | -2.9456964 | -0.357 |
| USP8 | ILMN_2094587 | 0.185 | 0.0258 | -2.33 | -3.7947185 | -0.357 |
| EIF3D | ILMN_1739847 | 0.0308 | 0.00118 | -3.53 | -1.0203388 | -0.356 |
| SRSF1 | ILMN_1795341 | 0.168 | 0.0216 | -2.4 | -3.6403738 | -0.356 |
| SNAP23 | ILMN_1748911 | 0.192 | 0.0275 | -2.3 | -3.8520103 | -0.356 |
| KMT5A | ILMN_1651936 | 0.218 | 0.0346 | -2.2 | -4.0497122 | -0.356 |
| PROCR | ILMN_1717262 | 0.224 | 0.0363 | -2.18 | -4.0894938 | -0.355 |
| ARF5 | ILMN_1752340 | 0.244 | 0.0428 | -2.1 | -4.2294968 | -0.355 |
| JUND | ILMN_1810214 | 0.0227 | 0.000726 | -3.7 | -0.5751988 | -0.354 |
| NPTN | ILMN_2336982 | 0.0269 | 0.000952 | -3.6 | -0.8250315 | -0.354 |
| AKT1 | ILMN_2388507 | 0.109 | 0.0102 | -2.71 | -2.9781951 | -0.354 |
| OSBP | ILMN_1706376 | 0.213 | 0.0335 | -2.21 | -4.0201726 | -0.354 |
| RPA1 | ILMN_2049642 | 0.0299 | 0.00112 | -3.54 | -0.9767652 | -0.353 |
| DNAJA2 | ILMN_1770127 | 0.104 | 0.00918 | -2.76 | -2.8859453 | -0.353 |
| TP53RK | ILMN_1764851 | 0.237 | 0.0402 | -2.13 | -4.1760958 | -0.353 |
| ARAP1 | ILMN_1772233 | 0.0796 | 0.00572 | -2.94 | -2.4607775 | -0.352 |
| EMC4 | ILMN_1798874 | 0.0902 | 0.00722 | -2.85 | -2.6703925 | -0.352 |
| CCT5 | ILMN_1706246 | 0.0933 | 0.0077 | -2.82 | -2.7289853 | -0.351 |
| RXRA | ILMN_1687315 | 0.1 | 0.00864 | -2.78 | -2.8319845 | -0.351 |
| TSC2 | ILMN_1714216 | 0.125 | 0.0128 | -2.62 | -3.1802826 | -0.351 |
| CLDN11 | ILMN_1754103 | 0.143 | 0.0162 | -2.52 | -3.3872064 | -0.351 |
| REXO1 | ILMN_1753008 | 0.149 | 0.0175 | -2.49 | -3.4590067 | -0.351 |
| HMGN1 | ILMN_1652123 | 0.24 | 0.0414 | -2.12 | -4.2006304 | -0.351 |
| CRELD2 | ILMN_1748707 | 0.253 | 0.0457 | -2.07 | -4.2846583 | -0.351 |
| STK4 | ILMN_1711383 | 0.109 | 0.0102 | -2.71 | -2.9756794 | -0.35 |
| CS | ILMN_2396410 | 0.264 | 0.0497 | -2.03 | -4.355322 | -0.35 |
| RAB4B | ILMN_1803136 | 0.037 | 0.0016 | -3.42 | -1.3014112 | -0.349 |
| TRIM13 | ILMN_2350114 | 0.0617 | 0.00377 | -3.1 | -2.0843168 | -0.349 |
| DUSP23 | ILMN_1659462 | 0.213 | 0.0334 | -2.21 | -4.0173064 | -0.349 |
| C6orf106 | ILMN_1704253 | 0.257 | 0.0473 | -2.06 | -4.3136442 | -0.349 |
| SARNP | ILMN_1680967 | 0.0428 | 0.00204 | -3.33 | -1.5260641 | -0.348 |
| SRSF6 | ILMN_1697469 | 0.109 | 0.0101 | -2.72 | -2.9707435 | -0.347 |
| U2AF2 | ILMN_2385173 | 0.128 | 0.0134 | -2.6 | -3.2223807 | -0.347 |
| NELFE | ILMN_1765532 | 0.183 | 0.0252 | -2.34 | -3.7757876 | -0.347 |
| TNFRSF1B | ILMN_1764788 | 0.0029 | 3.05E-05 | -4.77 | 2.3766495 | -0.346 |
| SDCBP2 | ILMN_1700310 | 0.0517 | 0.00284 | -3.21 | -1.8263202 | -0.346 |
| SLC9A1 | ILMN_1800425 | 0.0719 | 0.00481 | -3.01 | -2.3053547 | -0.346 |
| PIWIL4 | ILMN_1769606 | 0.0763 | 0.00529 | -2.97 | -2.3905454 | -0.346 |
| CBLL1 | ILMN_2073732 | 0.0826 | 0.00614 | -2.91 | -2.5246142 | -0.346 |
| DENND3 | ILMN_1692742 | 0.164 | 0.0206 | -2.42 | -3.5994442 | -0.346 |
| LYAR | ILMN_1764362 | 0.19 | 0.0271 | -2.3 | -3.8398228 | -0.346 |
| GAPT | ILMN_3242271 | 0.2 | 0.0297 | -2.26 | -3.9173412 | -0.346 |
| ZFPL1 | ILMN_1693039 | 0.22 | 0.0352 | -2.19 | -4.0636362 | -0.346 |
| CSNK1G1 | ILMN_1704713 | 0.111 | 0.0106 | -2.7 | -3.0108622 | -0.345 |
| SAP130 | ILMN_1700044 | 0.17 | 0.022 | -2.39 | -3.6584589 | -0.345 |
| DENND5A | ILMN_1785356 | 0.00258 | 2.58E-05 | -4.83 | 2.5331876 | -0.344 |
| WDR45B | ILMN_1685763 | 0.0871 | 0.00675 | -2.88 | -2.6109178 | -0.344 |
| SF3B5 | ILMN_1689389 | 0.113 | 0.0108 | -2.69 | -3.0308342 | -0.344 |
| TRIOBP | ILMN_1809145 | 0.175 | 0.0232 | -2.37 | -3.7052339 | -0.344 |
| ZDHHC6 | ILMN_2046003 | 0.211 | 0.0325 | -2.23 | -3.9946213 | -0.344 |
| ELF2 | ILMN_1772486 | 0.224 | 0.0363 | -2.18 | -4.0899656 | -0.344 |
| CEP192 | ILMN_1703754 | 0.26 | 0.0486 | -2.04 | -4.3379534 | -0.344 |
| PSAP | ILMN_2355559 | 0.00297 | 3.16E-05 | -4.76 | 2.342631 | -0.343 |
| EFNA4 | ILMN_1755710 | 0.031 | 0.0012 | -3.52 | -1.036943 | -0.343 |
| TOR1A | ILMN_1805812 | 0.0613 | 0.00374 | -3.1 | -2.0767735 | -0.343 |
| C4orf48 | ILMN_3239531 | 0.187 | 0.0263 | -2.32 | -3.8125153 | -0.343 |
| EAF1 | ILMN_1685012 | 0.0398 | 0.0018 | -3.37 | -1.4127988 | -0.342 |
| ATP5B | ILMN_1772132 | 0.0842 | 0.00631 | -2.9 | -2.5503849 | -0.342 |
| NMRK1 | ILMN_1674650 | 0.182 | 0.0249 | -2.34 | -3.7644096 | -0.342 |
| NINJ1 | ILMN_1815086 | 0.00233 | 2.26E-05 | -4.87 | 2.6580757 | -0.341 |
| FRYL | ILMN_1747223 | 0.129 | 0.0135 | -2.6 | -3.2268102 | -0.341 |
| TMEM258 | ILMN_1786759 | 0.0209 | 0.000652 | -3.74 | -0.4759814 | -0.34 |
| LTBP2 | ILMN_3248591 | 0.0873 | 0.00678 | -2.87 | -2.6137364 | -0.34 |
| NBPF10 | ILMN_2155719 | 0.0989 | 0.0084 | -2.79 | -2.8065566 | -0.34 |
| SEPT6 | ILMN_1661342 | 0.101 | 0.00872 | -2.78 | -2.8400469 | -0.34 |
| CREB3 | ILMN_1703072 | 0.123 | 0.0125 | -2.63 | -3.1578759 | -0.34 |
| SLC35B1 | ILMN_1727840 | 0.22 | 0.0352 | -2.19 | -4.0641338 | -0.34 |
| ANXA7 | ILMN_1703791 | 0.257 | 0.0473 | -2.05 | -4.3138452 | -0.34 |
| MRPL36 | ILMN_1800197 | 0.0596 | 0.00354 | -3.12 | -2.0268397 | -0.339 |
| NDUFB8 | ILMN_1661170 | 0.0805 | 0.00587 | -2.93 | -2.4851644 | -0.339 |
| M6PR | ILMN_2084353 | 0.165 | 0.0208 | -2.42 | -3.6069899 | -0.339 |
| AMMECR1L | ILMN_1727023 | 0.229 | 0.0379 | -2.16 | -4.1265933 | -0.339 |
| RAB5C | ILMN_1769665 | 0.0377 | 0.00165 | -3.4 | -1.3328515 | -0.338 |
| SNIP1 | ILMN_1651278 | 0.134 | 0.0145 | -2.57 | -3.2901776 | -0.338 |
| PBX2 | ILMN_1682699 | 0.178 | 0.0241 | -2.36 | -3.7351672 | -0.338 |
| PSMB5 | ILMN_1744649 | 0.218 | 0.0344 | -2.2 | -4.0428319 | -0.338 |
| PNKP | ILMN_1694111 | 0.229 | 0.0381 | -2.15 | -4.1312594 | -0.338 |
| MMS19 | ILMN_2174394 | 0.245 | 0.0429 | -2.1 | -4.2321959 | -0.338 |
| RHOT2 | ILMN_1669310 | 0.139 | 0.0155 | -2.54 | -3.3495746 | -0.337 |
| MRPS10 | ILMN_1663664 | 0.168 | 0.0215 | -2.4 | -3.6370521 | -0.337 |
| RPA1 | ILMN_1795719 | 0.177 | 0.0237 | -2.36 | -3.7220555 | -0.337 |
| PPM1G | ILMN_1806867 | 0.0517 | 0.00284 | -3.21 | -1.8267025 | -0.336 |
| NDUFS5 | ILMN_1776104 | 0.167 | 0.0213 | -2.41 | -3.628951 | -0.336 |
| P2RX1 | ILMN_1758529 | 0.215 | 0.0337 | -2.21 | -4.0260253 | -0.336 |
| PLPP6 | ILMN_1802628 | 0.262 | 0.0492 | -2.04 | -4.3479826 | -0.336 |
| CYTH1 | ILMN_2403852 | 0.0448 | 0.00219 | -3.3 | -1.590878 | -0.335 |
| MAGOH | ILMN_2215656 | 0.0992 | 0.00846 | -2.79 | -2.8124959 | -0.335 |
| CLEC4A | ILMN_2399363 | 0.106 | 0.00948 | -2.74 | -2.9144555 | -0.335 |
| TMEM154 | ILMN_2088124 | 0.149 | 0.0173 | -2.5 | -3.4473545 | -0.335 |
| CBLL1 | ILMN_1705433 | 0.182 | 0.025 | -2.34 | -3.7697828 | -0.335 |
| NCF1 | ILMN_1697309 | 0.186 | 0.026 | -2.32 | -3.8030883 | -0.335 |
| PRR14L | ILMN_3307887 | 0.237 | 0.04 | -2.13 | -4.1730107 | -0.335 |
| RNF4 | ILMN_1687941 | 0.252 | 0.0455 | -2.07 | -4.281397 | -0.335 |
| NUDC | ILMN_2097546 | 0.0313 | 0.00121 | -3.52 | -1.0472516 | -0.334 |
| CTAGE5 | ILMN_1690880 | 0.102 | 0.00896 | -2.76 | -2.8637008 | -0.334 |
| SELT | ILMN_1746368 | 0.133 | 0.0143 | -2.57 | -3.2815715 | -0.334 |
| ATG16L1 | ILMN_1725707 | 0.149 | 0.0174 | -2.49 | -3.4511771 | -0.334 |
| GET4 | ILMN_1701724 | 0.174 | 0.0229 | -2.38 | -3.6937951 | -0.334 |
| GMEB1 | ILMN_2383419 | 0.229 | 0.0381 | -2.15 | -4.1313461 | -0.334 |
| RANBP10 | ILMN_1667306 | 0.231 | 0.0386 | -2.15 | -4.1410459 | -0.333 |
| POFUT2 | ILMN_1775823 | 0.109 | 0.0102 | -2.71 | -2.9789439 | -0.332 |
| BUD31 | ILMN_1710697 | 0.151 | 0.018 | -2.48 | -3.4793174 | -0.332 |
| ATG5 | ILMN_1718265 | 0.163 | 0.0205 | -2.43 | -3.5947819 | -0.332 |
| BRCA1 | ILMN_1738027 | 0.234 | 0.0393 | -2.14 | -4.1576658 | -0.332 |
| TMEM134 | ILMN_2340721 | 0.248 | 0.0443 | -2.09 | -4.2584452 | -0.332 |
| STK11 | ILMN_1751871 | 0.0817 | 0.00602 | -2.92 | -2.5072916 | -0.331 |
| FOXRED1 | ILMN_1762312 | 0.0826 | 0.00614 | -2.91 | -2.5246459 | -0.331 |
| ZSWIM8 | ILMN_1669433 | 0.163 | 0.0205 | -2.43 | -3.5938683 | -0.331 |
| PDLIM7 | ILMN_2396639 | 0.209 | 0.0321 | -2.23 | -3.9832757 | -0.331 |
| VPS26A | ILMN_2344850 | 0.226 | 0.0372 | -2.16 | -4.1101913 | -0.331 |
| RINT1 | ILMN_1784584 | 0.246 | 0.0433 | -2.1 | -4.2403485 | -0.331 |
| SLC52A2 | ILMN_2041577 | 0.257 | 0.0474 | -2.05 | -4.3156961 | -0.331 |
| ATG101 | ILMN_1772527 | 0.0802 | 0.00583 | -2.93 | -2.4786028 | -0.33 |
| SBF1 | ILMN_1703246 | 0.131 | 0.014 | -2.59 | -3.258132 | -0.33 |
| FAM103A1 | ILMN_1763365 | 0.145 | 0.0166 | -2.51 | -3.4112464 | -0.33 |
| POLB | ILMN_1767894 | 0.15 | 0.0178 | -2.49 | -3.4700559 | -0.33 |
| SLC22A18 | ILMN_2382505 | 0.207 | 0.0316 | -2.24 | -3.9698137 | -0.329 |
| NCOA4 | ILMN_1773906 | 0.0362 | 0.00155 | -3.43 | -1.2709838 | -0.328 |
| TMEM214 | ILMN_1716907 | 0.123 | 0.0124 | -2.63 | -3.1516571 | -0.328 |
| BIN3-IT1 | ILMN_1811608 | 0.143 | 0.0161 | -2.53 | -3.385863 | -0.328 |
| ZNF524 | ILMN_1810147 | 0.213 | 0.0334 | -2.21 | -4.0178676 | -0.328 |
| CXXC1 | ILMN_1691276 | 0.0683 | 0.00446 | -3.04 | -2.2364093 | -0.327 |
| NSUN2 | ILMN_1680129 | 0.0842 | 0.00633 | -2.9 | -2.5525908 | -0.327 |
| PRMT1 | ILMN_2347234 | 0.121 | 0.0121 | -2.64 | -3.1320845 | -0.327 |
| TDG | ILMN_1777096 | 0.135 | 0.0148 | -2.56 | -3.3068849 | -0.327 |
| USP39 | ILMN_1659523 | 0.159 | 0.0195 | -2.45 | -3.5501671 | -0.327 |
| CMIP | ILMN_1738075 | 0.176 | 0.0236 | -2.36 | -3.7185864 | -0.327 |
| AP2M1 | ILMN_2402798 | 0.252 | 0.0455 | -2.07 | -4.2817361 | -0.327 |
| IRF2BP2 | ILMN_2394561 | 0.264 | 0.0497 | -2.03 | -4.3569822 | -0.327 |
| UBXN6 | ILMN_1658624 | 0.0186 | 0.000547 | -3.8 | -0.3132964 | -0.326 |
| DAP3 | ILMN_1781680 | 0.0192 | 0.000575 | -3.78 | -0.3597682 | -0.326 |
| ELL | ILMN_1736048 | 0.0778 | 0.00545 | -2.96 | -2.4179827 | -0.326 |
| FAM168A | ILMN_1847308 | 0.0811 | 0.00597 | -2.92 | -2.4995359 | -0.326 |
| TAF1 | ILMN_1664439 | 0.207 | 0.0316 | -2.24 | -3.9699509 | -0.326 |
| DLEU7 | ILMN_3307752 | 0.219 | 0.0348 | -2.2 | -4.0523094 | -0.326 |
| KIAA0141 | ILMN_1762990 | 0.222 | 0.0355 | -2.19 | -4.0716382 | -0.326 |
| ANKLE2 | ILMN_3249608 | 0.231 | 0.0386 | -2.15 | -4.142335 | -0.326 |
| ATXN7L3 | ILMN_1862018 | 0.0158 | 0.000427 | -3.88 | -0.0837013 | -0.325 |
| TAF13 | ILMN_2061318 | 0.156 | 0.0189 | -2.46 | -3.5226453 | -0.325 |
| SENP7 | ILMN_2382354 | 0.202 | 0.0302 | -2.26 | -3.9307749 | -0.325 |
| WBP11 | ILMN_1766435 | 0.241 | 0.0415 | -2.12 | -4.2028826 | -0.325 |
| APH1A | ILMN_1658472 | 0.0484 | 0.00256 | -3.24 | -1.733426 | -0.324 |
| UBA7 | ILMN_1794612 | 0.164 | 0.0206 | -2.42 | -3.6008673 | -0.323 |
| GNL3 | ILMN_2324056 | 0.179 | 0.0244 | -2.35 | -3.7459735 | -0.323 |
| TAF15 | ILMN_2402131 | 0.196 | 0.0285 | -2.28 | -3.8820835 | -0.323 |
| ZNF559 | ILMN_1677785 | 0.262 | 0.0491 | -2.04 | -4.3460038 | -0.323 |
| RALGDS | ILMN_1699856 | 0.0695 | 0.0046 | -3.02 | -2.2642676 | -0.322 |
| SAR1A | ILMN_1657697 | 0.0753 | 0.00521 | -2.98 | -2.3765042 | -0.322 |
| MIS12 | ILMN_1718069 | 0.202 | 0.0304 | -2.25 | -3.9373687 | -0.322 |
| INTS6L | ILMN_2207419 | 0.205 | 0.0311 | -2.24 | -3.9581924 | -0.322 |
| BAG6 | ILMN_1705364 | 0.0209 | 0.00065 | -3.74 | -0.4739234 | -0.321 |
| NIPAL1 | ILMN_1811593 | 0.106 | 0.00951 | -2.74 | -2.9172437 | -0.321 |
| ARHGAP17 | ILMN_1718610 | 0.116 | 0.0113 | -2.67 | -3.0676042 | -0.321 |
| CHMP4B | ILMN_1771233 | 0.136 | 0.0149 | -2.56 | -3.3142917 | -0.321 |
| MAPKAP1 | ILMN_2360229 | 0.172 | 0.0223 | -2.39 | -3.6694784 | -0.321 |
| HMGN2 | ILMN_2058141 | 0.181 | 0.0248 | -2.34 | -3.761461 | -0.321 |
| MCRS1 | ILMN_1784227 | 0.192 | 0.0275 | -2.3 | -3.8505683 | -0.321 |
| DENND5A | ILMN_3245413 | 0.00659 | 0.000107 | -4.36 | 1.2024169 | -0.32 |
| TNPO2 | ILMN_1656066 | 0.0181 | 0.00052 | -3.82 | -0.2661608 | -0.32 |
| RPS6KA1 | ILMN_1715173 | 0.039 | 0.00175 | -3.38 | -1.3837175 | -0.32 |
| DSE | ILMN_1706498 | 0.0766 | 0.00533 | -2.97 | -2.3971757 | -0.32 |
| BAG4 | ILMN_1727996 | 0.132 | 0.014 | -2.58 | -3.2627188 | -0.319 |
| ACAA1 | ILMN_1738921 | 0.155 | 0.0186 | -2.47 | -3.5098677 | -0.319 |
| DNAH1 | ILMN_1685052 | 0.227 | 0.0374 | -2.16 | -4.1158843 | -0.319 |
| GBE1 | ILMN_1789702 | 0.255 | 0.0465 | -2.06 | -4.299415 | -0.319 |
| SLC35A3 | ILMN_1653429 | 0.0513 | 0.0028 | -3.21 | -1.8138269 | -0.318 |
| FZD9 | ILMN_1704398 | 0.178 | 0.0241 | -2.36 | -3.7379745 | -0.318 |
| PCNX3 | ILMN_1783350 | 0.189 | 0.0267 | -2.31 | -3.823811 | -0.318 |
| ZPR1 | ILMN_1753790 | 0.199 | 0.0294 | -2.27 | -3.9079751 | -0.318 |
| PSMC3 | ILMN_1809010 | 0.209 | 0.0321 | -2.23 | -3.9841023 | -0.318 |
| SRXN1 | ILMN_1804822 | 0.23 | 0.0382 | -2.15 | -4.1334512 | -0.318 |
| GAPDH | ILMN_1343295 | 0.027 | 0.000957 | -3.6 | -0.8304909 | -0.317 |
| JTB | ILMN_2206716 | 0.0654 | 0.00419 | -3.06 | -2.1791271 | -0.317 |
| TOM1 | ILMN_2082314 | 0.0786 | 0.00558 | -2.95 | -2.4388643 | -0.317 |
| COMMD8 | ILMN_1765644 | 0.168 | 0.0216 | -2.4 | -3.6399215 | -0.317 |
| AXIN1 | ILMN_1692967 | 0.213 | 0.0334 | -2.21 | -4.0175862 | -0.317 |
| ZDHHC6 | ILMN_1739659 | 0.254 | 0.0461 | -2.07 | -4.2931295 | -0.317 |
| UQCRFS1 | ILMN_1701749 | 0.11 | 0.0103 | -2.71 | -2.9843773 | -0.316 |
| AP2M1 | ILMN_1699598 | 0.127 | 0.0131 | -2.61 | -3.2033835 | -0.316 |
| KIAA1683 | ILMN_2158003 | 0.206 | 0.0314 | -2.24 | -3.964505 | -0.316 |
| ARL6IP5 | ILMN_1769810 | 0.078 | 0.00549 | -2.96 | -2.4242415 | -0.315 |
| TP53BP1 | ILMN_1664440 | 0.147 | 0.0169 | -2.51 | -3.4246583 | -0.315 |
| C14orf2 | ILMN_1652722 | 0.242 | 0.0418 | -2.11 | -4.2097038 | -0.315 |
| GRB2 | ILMN_1748797 | 0.0613 | 0.00373 | -3.1 | -2.0754115 | -0.314 |
| TMCO1 | ILMN_1793829 | 0.0865 | 0.00668 | -2.88 | -2.6014711 | -0.314 |
| EIF4H | ILMN_1776021 | 0.131 | 0.0139 | -2.59 | -3.2518879 | -0.314 |
| PSMC5 | ILMN_1780189 | 0.218 | 0.0346 | -2.2 | -4.0490813 | -0.314 |
| CPSF3L | ILMN_1718013 | 0.263 | 0.0493 | -2.04 | -4.3500131 | -0.314 |
| ATP11C | ILMN_2389810 | 0.162 | 0.0201 | -2.43 | -3.5796526 | -0.313 |
| ZFP91 | ILMN_1665423 | 0.174 | 0.0231 | -2.37 | -3.6995306 | -0.313 |
| RANBP3 | ILMN_1699632 | 0.204 | 0.0308 | -2.25 | -3.9473463 | -0.313 |
| ZNF581 | ILMN_1679093 | 0.21 | 0.0323 | -2.23 | -3.9908603 | -0.313 |
| ACLY | ILMN_2371379 | 0.103 | 0.00914 | -2.76 | -2.8819154 | -0.312 |
| SDHA | ILMN_2051232 | 0.131 | 0.0139 | -2.59 | -3.2542113 | -0.312 |
| TSC22D4 | ILMN_1721636 | 0.158 | 0.0192 | -2.45 | -3.5394991 | -0.312 |
| ARPP19 | ILMN_1772798 | 0.162 | 0.0201 | -2.43 | -3.5788497 | -0.312 |
| LRTOMT | ILMN_3237645 | 0.166 | 0.0211 | -2.41 | -3.6224225 | -0.312 |
| CCDC59 | ILMN_1662318 | 0.239 | 0.0409 | -2.12 | -4.1922466 | -0.312 |
| DOCK2 | ILMN_1799725 | 0.0788 | 0.0056 | -2.95 | -2.4424602 | -0.311 |
| POFUT2 | ILMN_2376667 | 0.0818 | 0.00604 | -2.92 | -2.5101814 | -0.311 |
| DCTN1 | ILMN_2412807 | 0.11 | 0.0103 | -2.71 | -2.9865905 | -0.311 |
| AKR7A3 | ILMN_2145396 | 0.247 | 0.0438 | -2.09 | -4.2499743 | -0.311 |
| NOP10 | ILMN_1815479 | 0.033 | 0.00133 | -3.48 | -1.1356131 | -0.31 |
| FKBP1A | ILMN_2333367 | 0.0407 | 0.00187 | -3.36 | -1.4477652 | -0.31 |
| PRICKLE3 | ILMN_1656942 | 0.041 | 0.00191 | -3.35 | -1.4670095 | -0.31 |
| TRMT112 | ILMN_1690802 | 0.0694 | 0.00458 | -3.03 | -2.2601145 | -0.31 |
| PCYOX1 | ILMN_1679725 | 0.245 | 0.0429 | -2.1 | -4.2327604 | -0.31 |
| EMC7 | ILMN_2150000 | 0.0486 | 0.00259 | -3.24 | -1.7433115 | -0.309 |
| NME3 | ILMN_1669456 | 0.0563 | 0.00324 | -3.16 | -1.9470011 | -0.309 |
| MAP1LC3A | ILMN_1776188 | 0.133 | 0.0144 | -2.57 | -3.2850628 | -0.309 |
| NUPL2 | ILMN_2115154 | 0.19 | 0.0269 | -2.31 | -3.8326549 | -0.309 |
| FIP1L1 | ILMN_1768743 | 0.211 | 0.0326 | -2.22 | -3.9972619 | -0.309 |
| PLEKHJ1 | ILMN_1697701 | 0.212 | 0.033 | -2.22 | -4.0085719 | -0.309 |
| RNF114 | ILMN_3247906 | 0.255 | 0.0465 | -2.06 | -4.300596 | -0.309 |
| PTTG1IP | ILMN_2128750 | 0.0658 | 0.00422 | -3.06 | -2.1861051 | -0.308 |
| ARHGAP9 | ILMN_2382657 | 0.179 | 0.0243 | -2.35 | -3.7430803 | -0.308 |
| GLUL | ILMN_1835017 | 0.184 | 0.0254 | -2.33 | -3.7827085 | -0.308 |
| SMG9 | ILMN_1737005 | 0.231 | 0.0385 | -2.15 | -4.1392209 | -0.308 |
| UBAP2L | ILMN_1814789 | 0.0364 | 0.00156 | -3.43 | -1.276713 | -0.307 |
| GMIP | ILMN_1805693 | 0.0901 | 0.00716 | -2.85 | -2.6632925 | -0.307 |
| PSMA4 | ILMN_1682098 | 0.14 | 0.0157 | -2.54 | -3.3619672 | -0.307 |
| MED8 | ILMN_1736847 | 0.168 | 0.0215 | -2.4 | -3.6390743 | -0.307 |
| OSBPL8 | ILMN_1782459 | 0.21 | 0.0322 | -2.23 | -3.9872725 | -0.307 |
| WDR1 | ILMN_1780036 | 0.224 | 0.0363 | -2.18 | -4.0906026 | -0.307 |
| FMNL1 | ILMN_1782748 | 0.0885 | 0.00695 | -2.86 | -2.6371239 | -0.306 |
| FAM133B | ILMN_1777745 | 0.0998 | 0.00857 | -2.78 | -2.8243282 | -0.306 |
| SIRT2 | ILMN_1723494 | 0.169 | 0.0218 | -2.4 | -3.6499943 | -0.306 |
| MED8 | ILMN_2339705 | 0.221 | 0.0354 | -2.19 | -4.0680695 | -0.306 |
| STK11IP | ILMN_1690085 | 0.0742 | 0.00508 | -2.99 | -2.3536101 | -0.305 |
| VLDLR | ILMN_2361862 | 0.0885 | 0.00696 | -2.86 | -2.6384309 | -0.305 |
| TUBGCP2 | ILMN_1775074 | 0.162 | 0.02 | -2.44 | -3.5750405 | -0.305 |
| KRT10 | ILMN_1716093 | 0.19 | 0.0269 | -2.31 | -3.8316195 | -0.305 |
| ZNF227 | ILMN_1721034 | 0.206 | 0.0312 | -2.24 | -3.9599573 | -0.305 |
| CHPF2 | ILMN_1772991 | 0.0398 | 0.00181 | -3.37 | -1.4179835 | -0.304 |
| FRS2 | ILMN_1911605 | 0.109 | 0.0102 | -2.71 | -2.9763374 | -0.304 |
| TMEM154 | ILMN_1683494 | 0.19 | 0.0271 | -2.3 | -3.8385483 | -0.304 |
| IPPK | ILMN_1732873 | 0.0594 | 0.00351 | -3.13 | -2.0193676 | -0.303 |
| TMEM134 | ILMN_1770977 | 0.258 | 0.048 | -2.05 | -4.3265291 | -0.303 |
| PPP2R5E | ILMN_1666761 | 0.0322 | 0.00128 | -3.5 | -1.0983499 | -0.302 |
| PRUNE1 | ILMN_1728914 | 0.0925 | 0.00753 | -2.83 | -2.7083682 | -0.302 |
| STK40 | ILMN_2075927 | 0.109 | 0.0101 | -2.72 | -2.9718436 | -0.302 |
| ANKRD13A | ILMN_1689908 | 0.128 | 0.0134 | -2.6 | -3.2212306 | -0.302 |
| WDR11 | ILMN_1662896 | 0.134 | 0.0145 | -2.57 | -3.2933887 | -0.302 |
| CBX6 | ILMN_1691930 | 0.227 | 0.0375 | -2.16 | -4.1176886 | -0.302 |
| APOBR | ILMN_1697925 | 0.0422 | 0.00199 | -3.34 | -1.503887 | -0.301 |
| PPP6R1 | ILMN_1794085 | 0.174 | 0.0231 | -2.37 | -3.6991846 | -0.301 |
| SLC15A4 | ILMN_2076463 | 0.185 | 0.0257 | -2.33 | -3.793031 | -0.301 |
| RNF123 | ILMN_1663605 | 0.227 | 0.0375 | -2.16 | -4.1177194 | -0.301 |
| TOP2B | ILMN_1777663 | 0.192 | 0.0276 | -2.3 | -3.852632 | -0.3 |
| TMUB1 | ILMN_1666050 | 0.205 | 0.031 | -2.25 | -3.9555033 | -0.3 |
| GOLGA3 | ILMN_1733511 | 0.212 | 0.0328 | -2.22 | -4.0037331 | -0.3 |
| CYTH1 | ILMN_1694084 | 0.0636 | 0.00397 | -3.08 | -2.1310633 | -0.299 |
| C16orf70 | ILMN_1711703 | 0.187 | 0.0264 | -2.32 | -3.8155146 | -0.299 |
| COX7A2 | ILMN_1701293 | 0.2 | 0.0296 | -2.27 | -3.9132298 | -0.299 |
| NAP1L4 | ILMN_1804327 | 0.212 | 0.0329 | -2.22 | -4.0055951 | -0.299 |
| SYNCRIP | ILMN_1727740 | 0.212 | 0.033 | -2.22 | -4.0082291 | -0.299 |
| GPN2 | ILMN_1721741 | 0.229 | 0.0379 | -2.16 | -4.1274677 | -0.299 |
| INSIG1 | ILMN_1686989 | 0.238 | 0.0406 | -2.13 | -4.18453 | -0.299 |
| RAP2C | ILMN_1773561 | 0.24 | 0.0412 | -2.12 | -4.1974375 | -0.299 |
| BUB3 | ILMN_1693145 | 0.246 | 0.0433 | -2.1 | -4.2394813 | -0.299 |
| EIF2AK1 | ILMN_2156267 | 0.0989 | 0.00841 | -2.79 | -2.8078554 | -0.298 |
| SCNM1 | ILMN_1746598 | 0.228 | 0.0377 | -2.16 | -4.12198 | -0.298 |
| MPLKIP | ILMN_2183885 | 0.256 | 0.0467 | -2.06 | -4.3036142 | -0.298 |
| DAZAP1 | ILMN_1746257 | 0.26 | 0.0486 | -2.04 | -4.3373569 | -0.298 |
| LLGL1 | ILMN_1798212 | 0.166 | 0.0211 | -2.41 | -3.6219662 | -0.297 |
| C1orf123 | ILMN_2082130 | 0.0446 | 0.00218 | -3.3 | -1.5839019 | -0.296 |
| IVNS1ABP | ILMN_2397750 | 0.108 | 0.01 | -2.72 | -2.9617147 | -0.296 |
| CMC2 | ILMN_1783333 | 0.204 | 0.0308 | -2.25 | -3.9481363 | -0.296 |
| ATP6V1C1 | ILMN_1659801 | 0.262 | 0.0491 | -2.04 | -4.3455713 | -0.295 |
| IDH3G | ILMN_1682762 | 0.0332 | 0.00135 | -3.48 | -1.1434661 | -0.294 |
| ADCY7 | ILMN_1655935 | 0.106 | 0.00955 | -2.74 | -2.9207259 | -0.294 |
| IMPDH2 | ILMN_1705737 | 0.12 | 0.012 | -2.65 | -3.1206718 | -0.294 |
| PSMB7 | ILMN_1814156 | 0.165 | 0.0208 | -2.42 | -3.6094655 | -0.294 |
| TSR3 | ILMN_1713884 | 0.213 | 0.0331 | -2.22 | -4.0098992 | -0.294 |
| ARHGAP27 | ILMN_2103362 | 0.221 | 0.0354 | -2.19 | -4.0682522 | -0.294 |
| RILP | ILMN_1800172 | 0.224 | 0.0364 | -2.17 | -4.0920655 | -0.294 |
| ATXN2L | ILMN_2271627 | 0.0456 | 0.00226 | -3.29 | -1.6181877 | -0.293 |
| EEF1B2 | ILMN_1701930 | 0.122 | 0.0123 | -2.64 | -3.1470125 | -0.293 |
| PJA2 | ILMN_1688702 | 0.153 | 0.0183 | -2.47 | -3.4946357 | -0.293 |
| LAMTOR1 | ILMN_1815878 | 0.237 | 0.0402 | -2.13 | -4.1771548 | -0.293 |
| RAB11A | ILMN_1712312 | 0.243 | 0.0423 | -2.11 | -4.2189286 | -0.293 |
| STARD3 | ILMN_1657095 | 0.0233 | 0.000754 | -3.69 | -0.6101087 | -0.292 |
| RAE1 | ILMN_1690259 | 0.0627 | 0.00388 | -3.09 | -2.110121 | -0.292 |
| RAB8B | ILMN_2173004 | 0.124 | 0.0126 | -2.63 | -3.1664518 | -0.292 |
| TSSC4 | ILMN_1802753 | 0.224 | 0.0362 | -2.18 | -4.086372 | -0.292 |
| CPSF3L | ILMN_1762316 | 0.239 | 0.0409 | -2.12 | -4.1921969 | -0.292 |
| APH1A | ILMN_2398388 | 0.245 | 0.0429 | -2.1 | -4.2315272 | -0.292 |
| ST13 | ILMN_1765204 | 0.0482 | 0.00254 | -3.25 | -1.7237696 | -0.291 |
| CORO7 | ILMN_1795949 | 0.0649 | 0.00412 | -3.07 | -2.1649326 | -0.291 |
| TGM2 | ILMN_1679267 | 0.156 | 0.0189 | -2.46 | -3.5239226 | -0.291 |
| C8orf59 | ILMN_1653205 | 0.192 | 0.0275 | -2.3 | -3.8506863 | -0.291 |
| ARHGEF2 | ILMN_1703477 | 0.098 | 0.0083 | -2.79 | -2.795996 | -0.29 |
| MTOR | ILMN_1769031 | 0.185 | 0.0257 | -2.33 | -3.7924683 | -0.29 |
| ADRM1 | ILMN_1760143 | 0.0149 | 0.00039 | -3.92 | 9.48E-05 | -0.289 |
| TOR2A | ILMN_2177832 | 0.0245 | 0.00082 | -3.66 | -0.6876125 | -0.289 |
| NOP10 | ILMN_3240247 | 0.0245 | 0.000821 | -3.66 | -0.6884501 | -0.289 |
| STYX | ILMN_1697024 | 0.026 | 0.000906 | -3.62 | -0.7796855 | -0.289 |
| CCDC115 | ILMN_1730347 | 0.228 | 0.0376 | -2.16 | -4.1193023 | -0.289 |
| HAX1 | ILMN_1750658 | 0.258 | 0.0478 | -2.05 | -4.3230402 | -0.289 |
| UBAC2 | ILMN_1713993 | 0.139 | 0.0156 | -2.54 | -3.3538133 | -0.288 |
| AP2S1 | ILMN_1809957 | 0.154 | 0.0185 | -2.47 | -3.507191 | -0.288 |
| ITFG2 | ILMN_1701244 | 0.162 | 0.0203 | -2.43 | -3.585536 | -0.288 |
| MRPS16 | ILMN_1775744 | 0.182 | 0.0249 | -2.34 | -3.7632279 | -0.288 |
| SNN | ILMN_1788251 | 0.217 | 0.0343 | -2.2 | -4.0405624 | -0.288 |
| TIRAP | ILMN_2400603 | 0.066 | 0.00424 | -3.06 | -2.191057 | -0.287 |
| SENP2 | ILMN_2123567 | 0.0904 | 0.00725 | -2.85 | -2.6742387 | -0.287 |
| AOC2 | ILMN_2405185 | 0.129 | 0.0135 | -2.6 | -3.2289155 | -0.287 |
| PP7080 | ILMN_3239925 | 0.138 | 0.0152 | -2.55 | -3.3350751 | -0.287 |
| UBXN1 | ILMN_1812769 | 0.153 | 0.0182 | -2.48 | -3.4902982 | -0.287 |
| IPO13 | ILMN_1651229 | 0.218 | 0.0347 | -2.2 | -4.050129 | -0.287 |
| ZDHHC18 | ILMN_1668270 | 0.104 | 0.00927 | -2.75 | -2.894447 | -0.286 |
| ARAP1 | ILMN_3269719 | 0.124 | 0.0127 | -2.62 | -3.1762806 | -0.286 |
| PRRT3 | ILMN_1784985 | 0.206 | 0.0312 | -2.24 | -3.9601765 | -0.286 |
| ASB7 | ILMN_1680419 | 0.139 | 0.0154 | -2.54 | -3.3453582 | -0.285 |
| TRRAP | ILMN_1660368 | 0.175 | 0.0232 | -2.37 | -3.7045956 | -0.285 |
| PLEKHM2 | ILMN_1683980 | 0.231 | 0.0384 | -2.15 | -4.1378058 | -0.284 |
| CDC16 | ILMN_2256765 | 0.261 | 0.0487 | -2.04 | -4.339241 | -0.284 |
| ATG16L1 | ILMN_2365881 | 0.168 | 0.0214 | -2.41 | -3.6322668 | -0.282 |
| BTG3 | ILMN_2154836 | 0.256 | 0.0469 | -2.06 | -4.3073987 | -0.282 |
| TOM1 | ILMN_1813148 | 0.19 | 0.0271 | -2.31 | -3.8381144 | -0.281 |
| DERL1 | ILMN_2209748 | 0.066 | 0.00424 | -3.06 | -2.1915308 | -0.28 |
| KDM4A | ILMN_1736650 | 0.11 | 0.0103 | -2.71 | -2.9877736 | -0.28 |
| MADD | ILMN_2328224 | 0.122 | 0.0123 | -2.64 | -3.1448956 | -0.28 |
| HIC2 | ILMN_1652762 | 0.157 | 0.0189 | -2.46 | -3.5264622 | -0.28 |
| TOPBP1 | ILMN_1684929 | 0.193 | 0.0278 | -2.29 | -3.8607551 | -0.28 |
| FLII | ILMN_1737170 | 0.243 | 0.0423 | -2.11 | -4.2191164 | -0.28 |
| BCCIP | ILMN_1771966 | 0.256 | 0.0468 | -2.06 | -4.3052236 | -0.28 |
| SDHA | ILMN_1744210 | 0.0209 | 0.000647 | -3.74 | -0.4697037 | -0.279 |
| FKBP1A | ILMN_1683969 | 0.121 | 0.0121 | -2.64 | -3.1312221 | -0.279 |
| TRMT10B | ILMN_1782513 | 0.225 | 0.0367 | -2.17 | -4.0980057 | -0.279 |
| PSMD7 | ILMN_1776173 | 0.229 | 0.0378 | -2.16 | -4.124684 | -0.279 |
| TYSND1 | ILMN_1775677 | 0.258 | 0.0477 | -2.05 | -4.3215491 | -0.279 |
| ACTG1 | ILMN_2053178 | 0.0753 | 0.0052 | -2.98 | -2.374909 | -0.278 |
| HMG20B | ILMN_1651315 | 0.134 | 0.0145 | -2.57 | -3.2937916 | -0.278 |
| SUCLG1 | ILMN_1779616 | 0.151 | 0.0179 | -2.48 | -3.4758731 | -0.278 |
| EIF4H | ILMN_2304624 | 0.182 | 0.0249 | -2.34 | -3.7641448 | -0.278 |
| BIK | ILMN_1770505 | 0.0516 | 0.00283 | -3.21 | -1.8240397 | -0.277 |
| ATP6V1E1 | ILMN_1798485 | 0.0933 | 0.00771 | -2.82 | -2.7292523 | -0.277 |
| NONO | ILMN_2052790 | 0.11 | 0.0104 | -2.7 | -2.999706 | -0.277 |
| SH2B1 | ILMN_2061185 | 0.226 | 0.037 | -2.17 | -4.1069018 | -0.277 |
| TAF2 | ILMN_1694888 | 0.223 | 0.0359 | -2.18 | -4.0802096 | -0.276 |
| UBE2I | ILMN_1810474 | 0.224 | 0.0364 | -2.17 | -4.0917878 | -0.276 |
| SPOP | ILMN_2397024 | 0.0427 | 0.00203 | -3.33 | -1.5224263 | -0.275 |
| HIVEP3 | ILMN_1864166 | 0.164 | 0.0206 | -2.42 | -3.6013491 | -0.275 |
| SLC15A4 | ILMN_1679731 | 0.218 | 0.0344 | -2.2 | -4.043907 | -0.275 |
| GOLGA6C | ILMN_1780143 | 0.0912 | 0.00736 | -2.84 | -2.6877283 | -0.274 |
| NAB1 | ILMN_1774617 | 0.168 | 0.0215 | -2.41 | -3.6364218 | -0.274 |
| NPTN | ILMN_1792997 | 0.173 | 0.0226 | -2.38 | -3.682049 | -0.274 |
| GPX4 | ILMN_1734353 | 0.18 | 0.0245 | -2.35 | -3.7513874 | -0.274 |
| PUM1 | ILMN_2401155 | 0.0124 | 0.000295 | -4.01 | 0.2601786 | -0.273 |
| MAPK3 | ILMN_2402341 | 0.0686 | 0.0045 | -3.03 | -2.2456077 | -0.272 |
| TAF5 | ILMN_1684802 | 0.133 | 0.0143 | -2.57 | -3.2809901 | -0.272 |
| C15orf39 | ILMN_1793729 | 0.237 | 0.0403 | -2.13 | -4.1788059 | -0.272 |
| PHKA2 | ILMN_1814074 | 0.256 | 0.0468 | -2.06 | -4.3056483 | -0.272 |
| HARS | ILMN_1763523 | 0.0802 | 0.00583 | -2.93 | -2.4786461 | -0.271 |
| TOMM22 | ILMN_1714623 | 0.1 | 0.0086 | -2.78 | -2.8272966 | -0.271 |
| LOC646214 | ILMN_3243351 | 0.131 | 0.0139 | -2.59 | -3.2546736 | -0.271 |
| CNST | ILMN_1670263 | 0.264 | 0.0497 | -2.03 | -4.3553112 | -0.271 |
| ZNF598 | ILMN_2075818 | 0.239 | 0.0409 | -2.12 | -4.1921744 | -0.27 |
| CYBA | ILMN_1744604 | 0.114 | 0.011 | -2.68 | -3.0492863 | -0.269 |
| CYTH2 | ILMN_1721241 | 0.244 | 0.0426 | -2.1 | -4.226531 | -0.269 |
| HIST1H4C | ILMN_2075334 | 0.0686 | 0.00449 | -3.03 | -2.2418933 | -0.268 |
| HMCES | ILMN_1815682 | 0.203 | 0.0306 | -2.25 | -3.9421302 | -0.268 |
| ELF4 | ILMN_1652082 | 0.209 | 0.0321 | -2.23 | -3.9840418 | -0.268 |
| ORM2 | ILMN_1731785 | 0.246 | 0.0436 | -2.09 | -4.2450724 | -0.268 |
| IKBKB | ILMN_1727142 | 0.264 | 0.0497 | -2.03 | -4.3558658 | -0.268 |
| ATP6V1G1 | ILMN_1784523 | 0.0554 | 0.00317 | -3.17 | -1.9260978 | -0.267 |
| U2AF2 | ILMN_1768930 | 0.111 | 0.0105 | -2.7 | -3.0056559 | -0.266 |
| BHLHE40 | ILMN_1768534 | 0.123 | 0.0125 | -2.63 | -3.1589198 | -0.266 |
| GNPTG | ILMN_1764230 | 0.181 | 0.0247 | -2.35 | -3.7567712 | -0.266 |
| PRPF8 | ILMN_1738677 | 0.0425 | 0.00202 | -3.33 | -1.5152025 | -0.265 |
| MICU1 | ILMN_1807540 | 0.218 | 0.0345 | -2.2 | -4.0464987 | -0.265 |
| NBEAL2 | ILMN_1660629 | 0.224 | 0.0364 | -2.17 | -4.0915143 | -0.265 |
| POLRMT | ILMN_1770356 | 0.0939 | 0.00778 | -2.82 | -2.7379077 | -0.264 |
| PSMD5 | ILMN_1720926 | 0.213 | 0.0333 | -2.21 | -4.01603 | -0.264 |
| SUDS3 | ILMN_1757754 | 0.0662 | 0.00426 | -3.05 | -2.1949674 | -0.263 |
| PAK2 | ILMN_1712687 | 0.177 | 0.0239 | -2.36 | -3.7303253 | -0.263 |
| MRE11A | ILMN_1709483 | 0.235 | 0.0395 | -2.14 | -4.1627569 | -0.263 |
| SFI1 | ILMN_1763887 | 0.261 | 0.0488 | -2.04 | -4.3408697 | -0.263 |
| SNU13 | ILMN_1763460 | 0.261 | 0.0488 | -2.04 | -4.3409813 | -0.263 |
| VILL | ILMN_1778650 | 0.126 | 0.0129 | -2.62 | -3.1877644 | -0.262 |
| KDELR2 | ILMN_1724293 | 0.172 | 0.0226 | -2.38 | -3.6801222 | -0.262 |
| TSPAN32 | ILMN_1760449 | 0.225 | 0.0366 | -2.17 | -4.0963562 | -0.262 |
| HMBOX1 | ILMN_1720059 | 0.195 | 0.0282 | -2.29 | -3.8722911 | -0.261 |
| BCAS4 | ILMN_1808059 | 0.2 | 0.0296 | -2.27 | -3.9154656 | -0.261 |
| SCNM1 | ILMN_2375651 | 0.229 | 0.038 | -2.15 | -4.1288569 | -0.261 |
| WDR70 | ILMN_2131392 | 0.246 | 0.0433 | -2.1 | -4.2399291 | -0.261 |
| RBX1 | ILMN_1666670 | 0.0514 | 0.00281 | -3.21 | -1.8165498 | -0.26 |
| MIEF1 | ILMN_1793203 | 0.127 | 0.0133 | -2.61 | -3.2130462 | -0.259 |
| TBC1D20 | ILMN_1742869 | 0.174 | 0.023 | -2.38 | -3.696828 | -0.259 |
| DHRS7 | ILMN_1807455 | 0.261 | 0.0487 | -2.04 | -4.338975 | -0.259 |
| PTPN6 | ILMN_1738675 | 0.144 | 0.0164 | -2.52 | -3.3985077 | -0.258 |
| IRF3 | ILMN_1765649 | 0.203 | 0.0306 | -2.25 | -3.9432537 | -0.258 |
| UPF2 | ILMN_2383693 | 0.255 | 0.0463 | -2.07 | -4.2955495 | -0.258 |
| XRCC6 | ILMN_1743097 | 0.128 | 0.0134 | -2.6 | -3.2228012 | -0.257 |
| SDHAF2 | ILMN_3238633 | 0.207 | 0.0314 | -2.24 | -3.9661168 | -0.257 |
| AOC3 | ILMN_1782086 | 0.22 | 0.035 | -2.19 | -4.0592504 | -0.257 |
| TPD52L2 | ILMN_1699570 | 0.24 | 0.041 | -2.12 | -4.1944294 | -0.257 |
| RNF113A | ILMN_1786388 | 0.248 | 0.0445 | -2.08 | -4.2622053 | -0.257 |
| ATP8B2 | ILMN_1782057 | 0.113 | 0.0108 | -2.69 | -3.0304658 | -0.256 |
| GAPDH | ILMN_1802252 | 0.131 | 0.0139 | -2.59 | -3.2538896 | -0.256 |
| TOMM20 | ILMN_3251472 | 0.132 | 0.0141 | -2.58 | -3.2687792 | -0.256 |
| SNAP29 | ILMN_1659857 | 0.243 | 0.0422 | -2.11 | -4.2174494 | -0.256 |
| RHEB | ILMN_1657949 | 0.258 | 0.0476 | -2.05 | -4.3200522 | -0.256 |
| ATP6V1E1 | ILMN_2339779 | 0.13 | 0.0138 | -2.59 | -3.2458685 | -0.255 |
| TACO1 | ILMN_1685112 | 0.149 | 0.0175 | -2.49 | -3.4547604 | -0.255 |
| AQR | ILMN_1717154 | 0.232 | 0.0388 | -2.14 | -4.14752 | -0.253 |
| EIF1B | ILMN_1679324 | 0.246 | 0.0437 | -2.09 | -4.2465631 | -0.253 |
| ZBTB2 | ILMN_1766247 | 0.264 | 0.0497 | -2.03 | -4.3561555 | -0.253 |
| AURKB | ILMN_1684217 | 0.0154 | 0.00041 | -3.9 | -0.046768 | -0.252 |
| DRG1 | ILMN_1658259 | 0.0193 | 0.000583 | -3.78 | -0.3723122 | -0.252 |
| SYS1 | ILMN_1756590 | 0.226 | 0.0371 | -2.17 | -4.1084792 | -0.252 |
| UBP1 | ILMN_1784410 | 0.236 | 0.0399 | -2.13 | -4.1694366 | -0.252 |
| MTFR1L | ILMN_3264466 | 0.0126 | 0.000304 | -4 | 0.2308414 | -0.251 |
| MYL12B | ILMN_1654016 | 0.028 | 0.00101 | -3.58 | -0.8792725 | -0.251 |
| CALM3 | ILMN_1666385 | 0.0925 | 0.00754 | -2.83 | -2.7092651 | -0.251 |
| PRPF40A | ILMN_1659854 | 0.148 | 0.0171 | -2.5 | -3.4344364 | -0.251 |
| SDF4 | ILMN_1696065 | 0.15 | 0.0177 | -2.49 | -3.4681449 | -0.251 |
| STAP1 | ILMN_1781085 | 0.154 | 0.0184 | -2.47 | -3.4988546 | -0.251 |
| EPN1 | ILMN_1772981 | 0.231 | 0.0387 | -2.15 | -4.1446973 | -0.251 |
| GCC1 | ILMN_1682206 | 0.0414 | 0.00194 | -3.35 | -1.4781329 | -0.249 |
| CS | ILMN_3307648 | 0.0905 | 0.00726 | -2.85 | -2.6754213 | -0.249 |
| TBPL1 | ILMN_1708147 | 0.196 | 0.0285 | -2.28 | -3.8821839 | -0.249 |
| TMEM9B | ILMN_2100815 | 0.0514 | 0.00281 | -3.21 | -1.8163806 | -0.248 |
| INTS5 | ILMN_1796968 | 0.173 | 0.0228 | -2.38 | -3.688179 | -0.248 |
| MYCBP | ILMN_1659620 | 0.0627 | 0.00388 | -3.09 | -2.1108024 | -0.247 |
| ZDHHC4 | ILMN_3307025 | 0.138 | 0.0152 | -2.55 | -3.3341478 | -0.247 |
| ARHGAP9 | ILMN_1663916 | 0.238 | 0.0404 | -2.13 | -4.1815818 | -0.246 |
| CHCHD2 | ILMN_2191681 | 0.0636 | 0.00397 | -3.08 | -2.1314789 | -0.245 |
| FKBP15 | ILMN_1804863 | 0.256 | 0.0467 | -2.06 | -4.3040208 | -0.245 |
| HES5 | ILMN_1794742 | 0.126 | 0.0129 | -2.62 | -3.190168 | -0.244 |
| PRKAG2 | ILMN_2387919 | 0.216 | 0.0341 | -2.2 | -4.035356 | -0.243 |
| TOMM34 | ILMN_1721128 | 0.23 | 0.0382 | -2.15 | -4.1342353 | -0.243 |
| CNBP | ILMN_3250201 | 0.255 | 0.0463 | -2.06 | -4.2967423 | -0.243 |
| RPL36AL | ILMN_2189933 | 0.0341 | 0.00142 | -3.46 | -1.1943845 | -0.242 |
| NDUFA10 | ILMN_2225698 | 0.114 | 0.0111 | -2.68 | -3.0519946 | -0.242 |
| DCAF11 | ILMN_2389376 | 0.193 | 0.0278 | -2.29 | -3.8597432 | -0.242 |
| GOSR1 | ILMN_1798816 | 0.112 | 0.0106 | -2.7 | -3.0156543 | -0.241 |
| ARHGDIA | ILMN_1734742 | 0.138 | 0.0152 | -2.55 | -3.3321525 | -0.241 |
| ACSS1 | ILMN_1752269 | 0.163 | 0.0203 | -2.43 | -3.5866105 | -0.241 |
| C1orf43 | ILMN_1729509 | 0.213 | 0.0334 | -2.21 | -4.0184267 | -0.241 |
| CACTIN | ILMN_2262462 | 0.155 | 0.0186 | -2.47 | -3.5101836 | -0.24 |
| RAC1 | ILMN_2359789 | 0.0576 | 0.00337 | -3.14 | -1.9816232 | -0.239 |
| PHF23 | ILMN_1746135 | 0.12 | 0.0119 | -2.65 | -3.1149585 | -0.239 |
| COX20 | ILMN_1808584 | 0.225 | 0.0365 | -2.17 | -4.0939197 | -0.239 |
| SNX3 | ILMN_1740180 | 0.0446 | 0.00218 | -3.3 | -1.584017 | -0.236 |
| LIPE | ILMN_1670693 | 0.0781 | 0.0055 | -2.96 | -2.4263753 | -0.236 |
| SLC16A3 | ILMN_2364022 | 0.172 | 0.0225 | -2.39 | -3.6767854 | -0.236 |
| GAPDH | ILMN_2038778 | 0.0873 | 0.00679 | -2.87 | -2.6160707 | -0.235 |
| NME6 | ILMN_1768279 | 0.177 | 0.0237 | -2.36 | -3.7215737 | -0.235 |
| F3 | ILMN_2129572 | 0.102 | 0.00889 | -2.77 | -2.8572111 | -0.234 |
| CDK20 | ILMN_1686804 | 0.205 | 0.0309 | -2.25 | -3.9525966 | -0.234 |
| SFSWAP | ILMN_1692575 | 0.234 | 0.0394 | -2.14 | -4.1592901 | -0.234 |
| PPP1R12C | ILMN_1685286 | 0.24 | 0.0413 | -2.12 | -4.2004466 | -0.234 |
| DNAJB12 | ILMN_2333865 | 0.26 | 0.0486 | -2.04 | -4.3376324 | -0.234 |
| TNFRSF19 | ILMN_1704154 | 0.039 | 0.00174 | -3.38 | -1.3819635 | -0.233 |
| ASCC2 | ILMN_1679919 | 0.249 | 0.0446 | -2.08 | -4.2647183 | -0.233 |
| IST1 | ILMN_1740351 | 0.0449 | 0.0022 | -3.3 | -1.5929021 | -0.232 |
| OTUD6B | ILMN_1772703 | 0.194 | 0.028 | -2.29 | -3.8679136 | -0.232 |
| EIF4G1 | ILMN_2370772 | 0.24 | 0.0412 | -2.12 | -4.1968023 | -0.232 |
| TIMM23 | ILMN_1679555 | 0.213 | 0.0334 | -2.21 | -4.0178979 | -0.231 |
| ZNF432 | ILMN_1693788 | 0.222 | 0.0355 | -2.19 | -4.0705984 | -0.231 |
| LOC440895 | ILMN_3250389 | 0.223 | 0.036 | -2.18 | -4.0820697 | -0.231 |
| COQ10B | ILMN_1751615 | 0.233 | 0.0391 | -2.14 | -4.15275 | -0.231 |
| TOR1AIP1 | ILMN_2141941 | 0.245 | 0.0429 | -2.1 | -4.2318809 | -0.231 |
| ANKRD54 | ILMN_1766309 | 0.193 | 0.0279 | -2.29 | -3.8620644 | -0.23 |
| FKBP1A | ILMN_1683658 | 0.245 | 0.0431 | -2.1 | -4.2359344 | -0.23 |
| PRICKLE4 | ILMN_1695868 | 0.224 | 0.0362 | -2.18 | -4.0880923 | -0.229 |
| GTPBP8 | ILMN_2393243 | 0.0611 | 0.0037 | -3.11 | -2.0671118 | -0.228 |
| SERP1 | ILMN_1706817 | 0.13 | 0.0138 | -2.59 | -3.2470365 | -0.228 |
| ODF2 | ILMN_1730698 | 0.154 | 0.0184 | -2.47 | -3.5009462 | -0.228 |
| CLPTM1 | ILMN_1665831 | 0.215 | 0.0338 | -2.21 | -4.0282579 | -0.228 |
| RGS19 | ILMN_1677085 | 0.231 | 0.0387 | -2.15 | -4.1450217 | -0.228 |
| PPP3CB | ILMN_1802669 | 0.195 | 0.0281 | -2.29 | -3.8704839 | -0.227 |
| DNAAF2 | ILMN_1769757 | 0.199 | 0.0293 | -2.27 | -3.9053185 | -0.227 |
| ATP5J | ILMN_2348093 | 0.219 | 0.0348 | -2.19 | -4.0531892 | -0.226 |
| KBTBD4 | ILMN_1687092 | 0.252 | 0.0453 | -2.07 | -4.2775753 | -0.226 |
| METTL6 | ILMN_1661998 | 0.0584 | 0.00343 | -3.14 | -1.9987974 | -0.225 |
| HECTD3 | ILMN_1656902 | 0.186 | 0.0259 | -2.32 | -3.799991 | -0.225 |
| KBTBD4 | ILMN_1687524 | 0.138 | 0.0153 | -2.55 | -3.3398101 | -0.224 |
| WSB1 | ILMN_2415748 | 0.15 | 0.0177 | -2.49 | -3.4659383 | -0.224 |
| GATAD2B | ILMN_1766359 | 0.196 | 0.0286 | -2.28 | -3.8850688 | -0.224 |
| SNRNP200 | ILMN_1705928 | 0.186 | 0.026 | -2.32 | -3.8007504 | -0.223 |
| FDX1L | ILMN_1744628 | 0.0168 | 0.000466 | -3.85 | -0.1647113 | -0.222 |
| C2orf42 | ILMN_1661594 | 0.0929 | 0.00765 | -2.83 | -2.7225961 | -0.222 |
| GRB2 | ILMN_1742521 | 0.178 | 0.0241 | -2.36 | -3.737513 | -0.222 |
| ATP11C | ILMN_1804137 | 0.192 | 0.0275 | -2.3 | -3.8522911 | -0.222 |
| ERH | ILMN_1781795 | 0.21 | 0.0323 | -2.23 | -3.9892782 | -0.221 |
| AFTPH | ILMN_2402766 | 0.0639 | 0.00401 | -3.08 | -2.1400064 | -0.22 |
| DHX30 | ILMN_1795218 | 0.0788 | 0.0056 | -2.95 | -2.4429037 | -0.22 |
| MFSD11 | ILMN_1756152 | 0.153 | 0.0183 | -2.47 | -3.4965604 | -0.22 |
| SPI1 | ILMN_2392043 | 0.243 | 0.0422 | -2.11 | -4.2183981 | -0.22 |
| MDH2 | ILMN_2079004 | 0.0477 | 0.00249 | -3.26 | -1.7053518 | -0.219 |
| PPP2CA | ILMN_2196097 | 0.195 | 0.0282 | -2.29 | -3.8732898 | -0.219 |
| PLEKHA2 | ILMN_1796339 | 0.192 | 0.0276 | -2.3 | -3.8534365 | -0.218 |
| ECI1 | ILMN_2049303 | 0.135 | 0.0146 | -2.57 | -3.3002045 | -0.217 |
| MED9 | ILMN_1794108 | 0.2 | 0.0297 | -2.27 | -3.916601 | -0.217 |
| RAB6A | ILMN_1800871 | 0.246 | 0.0433 | -2.1 | -4.2393836 | -0.217 |
| KANSL2 | ILMN_1713189 | 0.255 | 0.0463 | -2.06 | -4.2967453 | -0.216 |
| NSUN5 | ILMN_2294878 | 0.0668 | 0.00433 | -3.05 | -2.2103971 | -0.215 |
| MAP7D1 | ILMN_1777906 | 0.116 | 0.0113 | -2.67 | -3.0727444 | -0.215 |
| CASC3 | ILMN_1665004 | 0.145 | 0.0166 | -2.51 | -3.4108247 | -0.215 |
| CLIP2 | ILMN_2352190 | 0.208 | 0.0319 | -2.23 | -3.9778986 | -0.215 |
| MBTPS1 | ILMN_1651719 | 0.218 | 0.0344 | -2.2 | -4.0440403 | -0.215 |
| GPR35 | ILMN_1710221 | 0.217 | 0.0342 | -2.2 | -4.0393726 | -0.213 |
| TIRAP | ILMN_2290679 | 0.256 | 0.0469 | -2.06 | -4.3071424 | -0.213 |
| PAPOLA | ILMN_1798354 | 0.0469 | 0.00242 | -3.27 | -1.6808945 | -0.212 |
| LASP1 | ILMN_1665909 | 0.109 | 0.01 | -2.72 | -2.9655373 | -0.211 |
| ZNF415 | ILMN_1664292 | 0.185 | 0.0255 | -2.33 | -3.7862987 | -0.211 |
| PDLIM7 | ILMN_1690125 | 0.196 | 0.0287 | -2.28 | -3.8866585 | -0.211 |
| BTK | ILMN_1662026 | 0.241 | 0.0415 | -2.11 | -4.2043415 | -0.211 |
| HNRNPA1P10 | ILMN_2220283 | 0.0632 | 0.00394 | -3.08 | -2.1233355 | -0.21 |
| YPEL3 | ILMN_1791147 | 0.243 | 0.042 | -2.11 | -4.214524 | -0.21 |
| FBXW11 | ILMN_2394571 | 0.256 | 0.047 | -2.06 | -4.3090847 | -0.21 |
| OS9 | ILMN_2361807 | 0.127 | 0.0131 | -2.61 | -3.2027924 | -0.209 |
| MYH9 | ILMN_2087702 | 0.258 | 0.0479 | -2.05 | -4.3258532 | -0.209 |
| USF1 | ILMN_1777982 | 0.255 | 0.0466 | -2.06 | -4.3013019 | -0.208 |
| AKNA | ILMN_1770673 | 0.222 | 0.0358 | -2.18 | -4.0788844 | -0.207 |
| IFT52 | ILMN_2063500 | 0.0356 | 0.00151 | -3.44 | -1.2506536 | -0.206 |
| CDCA7 | ILMN_1737184 | 0.0767 | 0.00533 | -2.97 | -2.3979202 | -0.206 |
| NDUFA2 | ILMN_3243890 | 0.155 | 0.0186 | -2.47 | -3.5093461 | -0.206 |
| ARPC4 | ILMN_1707336 | 0.162 | 0.0202 | -2.43 | -3.5824498 | -0.206 |
| PDGFRL | ILMN_1680339 | 0.0667 | 0.00432 | -3.05 | -2.2080367 | -0.205 |
| TAF10 | ILMN_1721093 | 0.117 | 0.0115 | -2.66 | -3.0875912 | -0.204 |
| CSNK2B | ILMN_1800461 | 0.172 | 0.0226 | -2.38 | -3.6800808 | -0.203 |
| TM9SF3 | ILMN_1669931 | 0.223 | 0.0361 | -2.18 | -4.0847091 | -0.203 |
| DNTTIP1 | ILMN_1691117 | 0.16 | 0.0196 | -2.44 | -3.5552379 | -0.202 |
| R3HCC1L | ILMN_2158594 | 0.217 | 0.0342 | -2.2 | -4.0388386 | -0.202 |
| SLC35C2 | ILMN_2358914 | 0.138 | 0.0153 | -2.55 | -3.3370154 | -0.201 |
| EIF3CL | ILMN_3238570 | 0.195 | 0.0282 | -2.29 | -3.873728 | -0.201 |
| MYH9 | ILMN_1722872 | 0.218 | 0.0346 | -2.2 | -4.048627 | -0.201 |
| SDHB | ILMN_1667257 | 0.0695 | 0.00461 | -3.02 | -2.2659854 | -0.2 |
| ACSL5 | ILMN_1678517 | 0.149 | 0.0174 | -2.49 | -3.4516378 | -0.2 |
| ZNF76 | ILMN_1804263 | 0.249 | 0.0446 | -2.08 | -4.2649455 | -0.198 |
| EXOC1 | ILMN_1745583 | 0.222 | 0.0356 | -2.18 | -4.0726837 | -0.197 |
| ARPC5 | ILMN_1768394 | 0.0955 | 0.00798 | -2.81 | -2.7606889 | -0.196 |
| RAB1B | ILMN_1664030 | 0.222 | 0.0356 | -2.18 | -4.0725835 | -0.196 |
| URGCP | ILMN_1777811 | 0.167 | 0.0212 | -2.41 | -3.624512 | -0.195 |
| ATP5O | ILMN_1791332 | 0.168 | 0.0214 | -2.41 | -3.6345363 | -0.194 |
| KRBA1 | ILMN_1776631 | 0.127 | 0.0132 | -2.61 | -3.2088737 | -0.193 |
| AKIRIN1 | ILMN_1802799 | 0.0686 | 0.00449 | -3.03 | -2.2426679 | -0.192 |
| C6orf1 | ILMN_2372199 | 0.181 | 0.0246 | -2.35 | -3.7551028 | -0.192 |
| KPNA4 | ILMN_1664756 | 0.211 | 0.0325 | -2.22 | -3.9959504 | -0.192 |
| RPL36AL | ILMN_2189936 | 0.245 | 0.043 | -2.1 | -4.2340519 | -0.192 |
| SZRD1 | ILMN_1707503 | 0.132 | 0.0141 | -2.58 | -3.2667707 | -0.191 |
| DENR | ILMN_1760954 | 0.193 | 0.0278 | -2.29 | -3.8606402 | -0.191 |
| SRP14 | ILMN_2234758 | 0.249 | 0.0446 | -2.08 | -4.264346 | -0.191 |
| NMT2 | ILMN_1656378 | 0.126 | 0.0129 | -2.62 | -3.1886423 | -0.189 |
| CANX | ILMN_2401057 | 0.255 | 0.0466 | -2.06 | -4.3009968 | -0.189 |
| TXNRD2 | ILMN_1657893 | 0.0662 | 0.00427 | -3.05 | -2.1965757 | -0.187 |
| APOBEC3H | ILMN_1664828 | 0.0671 | 0.00436 | -3.04 | -2.2157605 | -0.187 |
| DRG2 | ILMN_1810531 | 0.227 | 0.0375 | -2.16 | -4.1170972 | -0.186 |
| GUCA1B | ILMN_2211728 | 0.149 | 0.0174 | -2.49 | -3.4511487 | -0.185 |
| RNASEK | ILMN_3241136 | 0.0854 | 0.00651 | -2.89 | -2.5771595 | -0.184 |
| CIAPIN1 | ILMN_1735199 | 0.0851 | 0.00646 | -2.89 | -2.5713566 | -0.183 |
| ABCB1 | ILMN_1812070 | 0.173 | 0.0226 | -2.38 | -3.6818671 | -0.181 |
| SERTAD3 | ILMN_1801934 | 0.225 | 0.0366 | -2.17 | -4.0977673 | -0.181 |
| SLC2A3 | ILMN_1775708 | 0.0913 | 0.00737 | -2.84 | -2.6895282 | -0.18 |
| WBP2 | ILMN_1788604 | 0.244 | 0.0425 | -2.1 | -4.2241778 | -0.18 |
| GTPBP10 | ILMN_2383754 | 0.039 | 0.00175 | -3.38 | -1.3840216 | -0.179 |
| ATP5L | ILMN_1812638 | 0.122 | 0.0122 | -2.64 | -3.1379077 | -0.179 |
| COL9A3 | ILMN_1740155 | 0.218 | 0.0346 | -2.2 | -4.0479493 | -0.179 |
| HSPA13 | ILMN_2231985 | 0.239 | 0.0409 | -2.12 | -4.1910943 | -0.179 |
| IK | ILMN_1699362 | 0.0904 | 0.00725 | -2.85 | -2.6741124 | -0.178 |
| RPS10 | ILMN_1686954 | 0.133 | 0.0143 | -2.58 | -3.2785153 | -0.178 |
| RAC2 | ILMN_1709795 | 0.19 | 0.0269 | -2.31 | -3.8304218 | -0.178 |
| TPD52 | ILMN_2381064 | 0.0274 | 0.00098 | -3.59 | -0.851829 | -0.177 |
| ZNF529 | ILMN_1660193 | 0.229 | 0.038 | -2.16 | -4.128004 | -0.177 |
| RAB35 | ILMN_1812571 | 0.246 | 0.0434 | -2.09 | -4.2414032 | -0.177 |
| RNASEK | ILMN_1715698 | 0.0186 | 0.000548 | -3.8 | -0.3161757 | -0.176 |
| DAD1 | ILMN_1731619 | 0.215 | 0.0338 | -2.21 | -4.0275852 | -0.175 |
| DKK3 | ILMN_1739759 | 0.233 | 0.0391 | -2.14 | -4.1528816 | -0.175 |
| CNOT4 | ILMN_1772677 | 0.261 | 0.0488 | -2.04 | -4.3402977 | -0.174 |
| PRPF3 | ILMN_1715392 | 0.15 | 0.0177 | -2.49 | -3.4664821 | -0.173 |
| TMEM230 | ILMN_2404539 | 0.264 | 0.0498 | -2.03 | -4.3581324 | -0.173 |
| PSMD2 | ILMN_1712432 | 0.174 | 0.0232 | -2.37 | -3.7036175 | -0.172 |
| SLC35C1 | ILMN_1680104 | 0.182 | 0.0249 | -2.34 | -3.7639521 | -0.172 |
| RGS10 | ILMN_1733538 | 0.242 | 0.0419 | -2.11 | -4.2108501 | -0.171 |
| GTF2E2 | ILMN_1664931 | 0.125 | 0.0128 | -2.62 | -3.1792005 | -0.17 |
| GPS1 | ILMN_2309228 | 0.17 | 0.022 | -2.4 | -3.6559966 | -0.17 |
| RALY | ILMN_1690610 | 0.2 | 0.0296 | -2.27 | -3.9136544 | -0.17 |
| DHX15 | ILMN_2168449 | 0.202 | 0.0303 | -2.26 | -3.9342964 | -0.17 |
| ZNF177 | ILMN_1787539 | 0.247 | 0.0438 | -2.09 | -4.2487051 | -0.17 |
| GCFC2 | ILMN_1699724 | 0.0876 | 0.00685 | -2.87 | -2.6239355 | -0.168 |
| BTF3 | ILMN_2319414 | 0.142 | 0.016 | -2.53 | -3.3768912 | -0.168 |
| NSUN5P2 | ILMN_1718449 | 0.143 | 0.0163 | -2.52 | -3.3935673 | -0.168 |
| WDR83OS | ILMN_1757914 | 0.166 | 0.021 | -2.41 | -3.616968 | -0.168 |
| PDXDC2P | ILMN_1660277 | 0.187 | 0.0264 | -2.32 | -3.8141585 | -0.168 |
| PTGS1 | ILMN_1665100 | 0.229 | 0.0381 | -2.15 | -4.1304515 | -0.167 |
| PPM1N | ILMN_1803357 | 0.195 | 0.0282 | -2.29 | -3.8732788 | -0.166 |
| SCAND2P | ILMN_1775590 | 0.149 | 0.0173 | -2.5 | -3.4448911 | -0.165 |
| MXD1 | ILMN_2214678 | 0.217 | 0.0343 | -2.2 | -4.0409303 | -0.165 |
| ZNF841 | ILMN_3235593 | 0.24 | 0.0411 | -2.12 | -4.1960119 | -0.165 |
| PPP2CA | ILMN_1722858 | 0.0871 | 0.00675 | -2.88 | -2.6102743 | -0.164 |
| ZNF3 | ILMN_1778560 | 0.109 | 0.0102 | -2.71 | -2.9757123 | -0.157 |
| TRIM41 | ILMN_1813027 | 0.11 | 0.0103 | -2.71 | -2.9868806 | -0.157 |
| NACA | ILMN_2167617 | 0.162 | 0.0202 | -2.43 | -3.5822861 | -0.156 |
| UBA52 | ILMN_1782977 | 0.189 | 0.0266 | -2.31 | -3.8231944 | -0.156 |
| SVBP | ILMN_2050023 | 0.111 | 0.0105 | -2.7 | -3.0046362 | -0.154 |
| URGCP | ILMN_2386818 | 0.222 | 0.0355 | -2.19 | -4.0708643 | -0.153 |
| ACOD1 | ILMN_3193271 | 0.245 | 0.0428 | -2.1 | -4.2304663 | -0.153 |
| ACIN1 | ILMN_1699636 | 0.264 | 0.0496 | -2.03 | -4.354033 | -0.153 |
| RPL24 | ILMN_2160388 | 0.264 | 0.0497 | -2.03 | -4.3569483 | -0.153 |
| PFDN5 | ILMN_1755536 | 0.158 | 0.0192 | -2.45 | -3.5391097 | -0.15 |
| RTFDC1 | ILMN_1771815 | 0.19 | 0.027 | -2.31 | -3.8336066 | -0.15 |
| UBB | ILMN_2191428 | 0.229 | 0.038 | -2.15 | -4.1298068 | -0.15 |
| COX6A1 | ILMN_1783636 | 0.198 | 0.029 | -2.28 | -3.8956214 | -0.149 |
| STOX1 | ILMN_1664014 | 0.249 | 0.0446 | -2.08 | -4.264487 | -0.149 |
| RPL41 | ILMN_2331890 | 0.0528 | 0.00293 | -3.19 | -1.8546374 | -0.146 |
| SLC9A2 | ILMN_1738849 | 0.102 | 0.00888 | -2.77 | -2.8564243 | -0.145 |
| LOC100130298 | ILMN_3270853 | 0.164 | 0.0207 | -2.42 | -3.6034542 | -0.145 |
| SPINT1 | ILMN_1724946 | 0.191 | 0.0273 | -2.3 | -3.8430294 | -0.143 |
| MUSTN1 | ILMN_1773814 | 0.138 | 0.0152 | -2.55 | -3.3345254 | -0.139 |
| UXT | ILMN_1671314 | 0.147 | 0.017 | -2.51 | -3.4290883 | -0.136 |
| CENPT | ILMN_3238375 | 0.255 | 0.0463 | -2.06 | -4.2966228 | -0.136 |
| OTUD6B | ILMN_2215631 | 0.237 | 0.0401 | -2.13 | -4.173876 | -0.135 |
| TYROBP | ILMN_1778977 | 0.0297 | 0.0011 | -3.55 | -0.9619534 | -0.134 |
| PPIL3 | ILMN_2285490 | 0.133 | 0.0142 | -2.58 | -3.276007 | -0.134 |
| DEAF1 | ILMN_1704045 | 0.149 | 0.0175 | -2.49 | -3.4561481 | -0.134 |
| MID2 | ILMN_1760180 | 0.151 | 0.0179 | -2.48 | -3.4750227 | -0.132 |
| ARPC3 | ILMN_1655561 | 0.197 | 0.0287 | -2.28 | -3.8887685 | -0.13 |
| CASP8 | ILMN_1809313 | 0.163 | 0.0204 | -2.43 | -3.5927509 | -0.127 |
| KCNIP3 | ILMN_2350823 | 0.204 | 0.0308 | -2.25 | -3.9478913 | -0.126 |
| RPRD2 | ILMN_1705733 | 0.182 | 0.0249 | -2.34 | -3.7661331 | -0.125 |
| BTF3 | ILMN_1659762 | 0.237 | 0.0403 | -2.13 | -4.1794007 | -0.125 |
| SGSM3 | ILMN_1679614 | 0.2 | 0.0297 | -2.26 | -3.9177574 | -0.124 |
| FAU | ILMN_1664614 | 0.211 | 0.0326 | -2.22 | -3.997068 | -0.124 |
| MIR577 | ILMN_3310870 | 0.237 | 0.0402 | -2.13 | -4.1771252 | -0.123 |
| CACNB1 | ILMN_1763604 | 0.258 | 0.0476 | -2.05 | -4.3199718 | -0.122 |
| IP6K3 | ILMN_3261111 | 0.204 | 0.0307 | -2.25 | -3.9460889 | -0.121 |
| C11orf31 | ILMN_1786872 | 0.219 | 0.0349 | -2.19 | -4.0555916 | -0.119 |
| CRYBB2 | ILMN_1760708 | 0.148 | 0.0172 | -2.5 | -3.4428346 | -0.117 |
| ZNF616 | ILMN_1719219 | 0.256 | 0.0466 | -2.06 | -4.3021309 | -0.113 |
| ATP5EP2 | ILMN_2225887 | 0.257 | 0.0473 | -2.05 | -4.3145342 | -0.111 |
| MYBL2 | ILMN_1709020 | 0.105 | 0.00939 | -2.75 | -2.9062912 | -0.109 |
| ATP5EP2 | ILMN_1756674 | 0.243 | 0.0421 | -2.11 | -4.2152805 | -0.109 |
| ANKRD20A1 | ILMN_1670452 | 0.107 | 0.00977 | -2.73 | -2.9410955 | -0.107 |
| ZSCAN2 | ILMN_1726512 | 0.246 | 0.0436 | -2.09 | -4.2449875 | -0.107 |
| GOLGA6D | ILMN_1708126 | 0.204 | 0.0308 | -2.25 | -3.9472983 | -0.106 |
| PHF19 | ILMN_1745420 | 0.244 | 0.0426 | -2.1 | -4.2260927 | -0.103 |
| PSMG3 | ILMN_1802627 | 0.174 | 0.023 | -2.38 | -3.6971111 | -0.0994 |
| SNORD139 | ILMN_3308310 | 0.238 | 0.0404 | -2.13 | -4.1805003 | -0.093 |
| GATA1 | ILMN_1797251 | 0.174 | 0.0231 | -2.37 | -3.6979726 | -0.0922 |
| FAM129C | ILMN_1664063 | 0.228 | 0.0377 | -2.16 | -4.1216333 | -0.0907 |
| TMEM151A | ILMN_1709659 | 0.252 | 0.0456 | -2.07 | -4.2829588 | -0.0895 |
| ZNF815P | ILMN_3243259 | 0.172 | 0.0224 | -2.39 | -3.6732199 | -0.0861 |
| PDE4DIP | ILMN_1659430 | 0.24 | 0.0412 | -2.12 | -4.197301 | -0.0771 |
