# Supplementary material for: Comprehensive Analysis of Common Different Gene Expression Signatures in the Neutrophils of Sepsis
Source: Biomed Res Int. 2021 Apr 17;2021:6655425. doi: 10.1155/2021/6655425 (PMC8077712; doi:10.1155/2021/6655425)
Supplement: Supplementary 4 [file 6655425.f4.docx]

Table S4. Detailed information of DEGs in GSE49756

| Gene symbol | probe ID | adj.P.Val | P.Value | t Value | B value | logFC |
| --- | --- | --- | --- | --- | --- | --- |
| KRTAP4-1 | ILMN_1780326 | 0.156 | 0.0394 | -2.12 | -4.41343 | -0.0717 |
| OR6Y1 | ILMN_1683046 | 0.179 | 0.048 | -2.03 | -4.5822 | -0.0721 |
| TYROBP | ILMN_1778977 | 0.165 | 0.0427 | -2.08 | -4.48306 | -0.0723 |
| WDR18 | ILMN_1694479 | 0.0887 | 0.0179 | -2.45 | -3.72785 | -0.0735 |
| B2M | ILMN_1725427 | 0.0511 | 0.0085 | -2.75 | -3.06024 | -0.0774 |
| SLC30A6 | ILMN_1699365 | 0.175 | 0.0465 | -2.05 | -4.55521 | -0.0775 |
| COL13A1 | ILMN_2370624 | 0.114 | 0.0253 | -2.31 | -4.02877 | -0.0834 |
| ZC3HC1 | ILMN_1761479 | 0.178 | 0.0477 | -2.03 | -4.57738 | -0.084 |
| SMC3 | ILMN_2182348 | 0.122 | 0.0278 | -2.27 | -4.11253 | -0.0857 |
| ARHGEF17 | ILMN_1754562 | 0.145 | 0.0355 | -2.17 | -4.32371 | -0.0882 |
| KIAA1522 | ILMN_1700384 | 0.172 | 0.0455 | -2.06 | -4.53697 | -0.0882 |
| EFL1 | ILMN_2404629 | 0.154 | 0.0386 | -2.13 | -4.3974 | -0.0899 |
| RUNX1-IT1 | ILMN_3260501 | 0.132 | 0.031 | -2.23 | -4.2064 | -0.0908 |
| SUPT4H1 | ILMN_1766245 | 0.129 | 0.0302 | -2.24 | -4.18491 | -0.092 |
| SARNP | ILMN_1680967 | 0.107 | 0.0231 | -2.35 | -3.95074 | -0.0922 |
| C9orf24 | ILMN_1763695 | 0.157 | 0.0399 | -2.12 | -4.42455 | -0.0925 |
| TSPY26P | ILMN_1737360 | 0.118 | 0.0267 | -2.29 | -4.07644 | -0.0977 |
| TSG101 | ILMN_1747146 | 0.125 | 0.0289 | -2.26 | -4.14586 | -0.0981 |
| DAZAP2 | ILMN_1718988 | 0.0173 | 0.00204 | -3.27 | -1.75297 | -0.1 |
| NR3C1 | ILMN_1668525 | 0.166 | 0.0435 | -2.08 | -4.49793 | -0.1 |
| METTL21A | ILMN_2049364 | 0.145 | 0.0352 | -2.17 | -4.31794 | -0.101 |
| CELF1 | ILMN_2382083 | 0.0441 | 0.00695 | -2.83 | -2.87852 | -0.102 |
| YIF1A | ILMN_1712975 | 0.144 | 0.0352 | -2.17 | -4.31717 | -0.102 |
| PPP2CA | ILMN_2196097 | 0.182 | 0.0492 | -2.02 | -4.60292 | -0.102 |
| NOSIP | ILMN_1759436 | 0.0493 | 0.00807 | -2.77 | -3.01348 | -0.103 |
| ASTE1 | ILMN_1803813 | 0.106 | 0.0229 | -2.35 | -3.94162 | -0.103 |
| PPM1G | ILMN_1765522 | 0.124 | 0.0284 | -2.26 | -4.13184 | -0.103 |
| DCP1B | ILMN_1743992 | 0.15 | 0.037 | -2.15 | -4.36079 | -0.103 |
| ZSCAN2 | ILMN_1726512 | 0.0977 | 0.0205 | -2.4 | -3.84373 | -0.104 |
| ZNF493 | ILMN_1663005 | 0.182 | 0.049 | -2.02 | -4.59973 | -0.104 |
| NONO | ILMN_2052790 | 0.112 | 0.0245 | -2.33 | -4.00312 | -0.106 |
| SH3YL1 | ILMN_1712231 | 0.12 | 0.0273 | -2.28 | -4.09794 | -0.106 |
| HGD | ILMN_2198239 | 0.142 | 0.0344 | -2.18 | -4.29638 | -0.106 |
| PJA1 | ILMN_1667261 | 0.16 | 0.0411 | -2.1 | -4.45031 | -0.106 |
| LAMP2 | ILMN_1752351 | 0.102 | 0.0217 | -2.38 | -3.89614 | -0.107 |
| PSMD2 | ILMN_1712432 | 0.149 | 0.0369 | -2.15 | -4.35809 | -0.107 |
| LMBRD1 | ILMN_1652128 | 0.157 | 0.0398 | -2.12 | -4.42224 | -0.107 |
| UBE2A | ILMN_2307455 | 0.179 | 0.0481 | -2.03 | -4.58495 | -0.107 |
| UBXN1 | ILMN_1812769 | 0.106 | 0.0228 | -2.36 | -3.93842 | -0.108 |
| SORCS1 | ILMN_1680251 | 0.177 | 0.0473 | -2.04 | -4.57001 | -0.108 |
| CSF3R | ILMN_2371280 | 0.0441 | 0.00696 | -2.83 | -2.87885 | -0.109 |
| YIPF3 | ILMN_1673604 | 0.107 | 0.0231 | -2.35 | -3.95001 | -0.109 |
| CDC42BPA | ILMN_1781472 | 0.119 | 0.0269 | -2.29 | -4.08218 | -0.109 |
| AKIRIN1 | ILMN_1802799 | 0.139 | 0.0334 | -2.19 | -4.27257 | -0.109 |
| CST3 | ILMN_1800354 | 0.147 | 0.0361 | -2.16 | -4.33778 | -0.11 |
| SHOC2 | ILMN_2158242 | 0.117 | 0.0261 | -2.3 | -4.05867 | -0.111 |
| S100A11 | ILMN_1750101 | 0.137 | 0.0325 | -2.2 | -4.24866 | -0.113 |
| SPATA9 | ILMN_1685272 | 0.149 | 0.037 | -2.15 | -4.359 | -0.113 |
| ACBD3 | ILMN_1665945 | 0.0511 | 0.00851 | -2.75 | -3.06102 | -0.114 |
| UBE2E3 | ILMN_1733142 | 0.126 | 0.0292 | -2.25 | -4.15571 | -0.114 |
| TXNRD2 | ILMN_1657893 | 0.166 | 0.0432 | -2.08 | -4.4932 | -0.114 |
| ARPC4 | ILMN_1707336 | 0.18 | 0.0483 | -2.03 | -4.58792 | -0.114 |
| RPRD2 | ILMN_1705733 | 0.133 | 0.0313 | -2.22 | -4.2169 | -0.116 |
| ATP6V1E1 | ILMN_2339779 | 0.137 | 0.0326 | -2.2 | -4.252 | -0.116 |
| PORCN | ILMN_1761112 | 0.152 | 0.0381 | -2.14 | -4.38529 | -0.116 |
| KMT5B | ILMN_1692026 | 0.0956 | 0.0199 | -2.41 | -3.8195 | -0.117 |
| RSRC2 | ILMN_2358277 | 0.12 | 0.0272 | -2.28 | -4.09368 | -0.117 |
| EIF2B2 | ILMN_1713380 | 0.128 | 0.0298 | -2.24 | -4.17216 | -0.117 |
| SLC15A4 | ILMN_2076463 | 0.149 | 0.0367 | -2.15 | -4.35343 | -0.117 |
| PPP1R12C | ILMN_1685286 | 0.15 | 0.0371 | -2.15 | -4.36283 | -0.117 |
| ARPC4 | ILMN_2393763 | 0.178 | 0.0474 | -2.04 | -4.57206 | -0.117 |
| YWHAZ | ILMN_1801928 | 0.111 | 0.0245 | -2.33 | -4.00146 | -0.118 |
| IP6K1 | ILMN_3187328 | 0.149 | 0.0367 | -2.15 | -4.35329 | -0.118 |
| SCAF11 | ILMN_3251511 | 0.155 | 0.0394 | -2.12 | -4.41307 | -0.118 |
| HARS2 | ILMN_1715113 | 0.148 | 0.0365 | -2.15 | -4.34949 | -0.119 |
| NIPA2 | ILMN_1720344 | 0.0739 | 0.0141 | -2.55 | -3.513 | -0.12 |
| CHMP7 | ILMN_1795933 | 0.105 | 0.0226 | -2.36 | -3.92983 | -0.12 |
| PPP3CB | ILMN_1802669 | 0.137 | 0.0328 | -2.2 | -4.25553 | -0.12 |
| NOL3 | ILMN_1813925 | 0.143 | 0.0346 | -2.18 | -4.30318 | -0.12 |
| FSIP1 | ILMN_1716925 | 0.0867 | 0.0174 | -2.47 | -3.69892 | -0.121 |
| RAB35 | ILMN_1812571 | 0.0997 | 0.0211 | -2.39 | -3.86924 | -0.121 |
| SULT1A1 | ILMN_2404795 | 0.0993 | 0.021 | -2.39 | -3.86475 | -0.122 |
| HNRNPA2B1 | ILMN_1886493 | 0.145 | 0.0354 | -2.17 | -4.32268 | -0.122 |
| CEP72 | ILMN_1800611 | 0.158 | 0.0405 | -2.11 | -4.43668 | -0.122 |
| LCP1 | ILMN_1662932 | 0.0411 | 0.00632 | -2.86 | -2.79198 | -0.123 |
| RBM23 | ILMN_2363106 | 0.0663 | 0.0122 | -2.61 | -3.38188 | -0.123 |
| ACTR2 | ILMN_2388605 | 0.0758 | 0.0146 | -2.54 | -3.54203 | -0.123 |
| HNRNPA2B1 | ILMN_3273854 | 0.177 | 0.0471 | -2.04 | -4.56659 | -0.123 |
| RLBP1 | ILMN_1751980 | 0.156 | 0.0396 | -2.12 | -4.41842 | -0.124 |
| ANKRD12 | ILMN_1661833 | 0.166 | 0.0434 | -2.08 | -4.49753 | -0.124 |
| CYB5B | ILMN_1684321 | 0.132 | 0.0312 | -2.22 | -4.21203 | -0.125 |
| TERF2IP | ILMN_1657983 | 0.163 | 0.0422 | -2.09 | -4.47341 | -0.125 |
| SRRM2 | ILMN_1734602 | 0.177 | 0.0471 | -2.04 | -4.56714 | -0.125 |
| MRFAP1 | ILMN_2055165 | 0.0179 | 0.00213 | -3.26 | -1.79318 | -0.126 |
| FOXJ3 | ILMN_2128668 | 0.0197 | 0.00241 | -3.21 | -1.90753 | -0.126 |
| SPPL2A | ILMN_1734229 | 0.0576 | 0.01 | -2.69 | -3.2104 | -0.126 |
| SP1 | ILMN_1676010 | 0.114 | 0.0252 | -2.31 | -4.02754 | -0.126 |
| LAMP2 | ILMN_1917290 | 0.117 | 0.0263 | -2.3 | -4.06457 | -0.126 |
| CLDN9 | ILMN_1740276 | 0.123 | 0.0281 | -2.27 | -4.12249 | -0.126 |
| CTU2 | ILMN_1779184 | 0.144 | 0.035 | -2.17 | -4.31327 | -0.126 |
| KIAA0430 | ILMN_1793371 | 0.176 | 0.0468 | -2.04 | -4.56169 | -0.126 |
| RNPS1 | ILMN_1691843 | 0.0811 | 0.0159 | -2.5 | -3.62257 | -0.127 |
| ALG10B | ILMN_1730304 | 0.0903 | 0.0184 | -2.45 | -3.74858 | -0.127 |
| TCEAL8 | ILMN_1656399 | 0.0932 | 0.0192 | -2.43 | -3.78755 | -0.127 |
| ATXN3 | ILMN_2393497 | 0.174 | 0.046 | -2.05 | -4.54614 | -0.127 |
| EXOC1 | ILMN_2347805 | 0.117 | 0.0264 | -2.29 | -4.06764 | -0.128 |
| CCDC174 | ILMN_1750144 | 0.125 | 0.0289 | -2.26 | -4.14651 | -0.128 |
| MYL12B | ILMN_1654016 | 0.0471 | 0.00759 | -2.79 | -2.95819 | -0.129 |
| INTS4 | ILMN_1676233 | 0.0657 | 0.012 | -2.62 | -3.36649 | -0.129 |
| TMEM131 | ILMN_1753608 | 0.0917 | 0.0188 | -2.44 | -3.76821 | -0.129 |
| EGLN2 | ILMN_2354391 | 0.131 | 0.0307 | -2.23 | -4.19879 | -0.129 |
| LAT2 | ILMN_1803560 | 0.148 | 0.0365 | -2.15 | -4.34911 | -0.13 |
| SLC35C2 | ILMN_2358914 | 0.156 | 0.0396 | -2.12 | -4.41881 | -0.13 |
| WAC | ILMN_2323526 | 0.0237 | 0.00308 | -3.12 | -2.13542 | -0.133 |
| SPINT1 | ILMN_2375992 | 0.122 | 0.0278 | -2.27 | -4.11214 | -0.133 |
| LSM7 | ILMN_1678165 | 0.15 | 0.0372 | -2.15 | -4.36374 | -0.133 |
| SF3A2 | ILMN_1754220 | 0.17 | 0.0445 | -2.07 | -4.51792 | -0.133 |
| SMARCA4 | ILMN_1814173 | 0.0426 | 0.00664 | -2.84 | -2.83704 | -0.134 |
| STAG2 | ILMN_1782609 | 0.08 | 0.0156 | -2.51 | -3.60485 | -0.134 |
| PYGM | ILMN_1720849 | 0.0847 | 0.0169 | -2.48 | -3.67288 | -0.134 |
| ZNF580 | ILMN_1653310 | 0.102 | 0.0217 | -2.38 | -3.89766 | -0.134 |
| CERS4 | ILMN_1748057 | 0.106 | 0.0228 | -2.36 | -3.94071 | -0.134 |
| PRCC | ILMN_1802843 | 0.107 | 0.0233 | -2.35 | -3.95674 | -0.134 |
| FAM13B | ILMN_1673478 | 0.112 | 0.0247 | -2.32 | -4.008 | -0.134 |
| SDHB | ILMN_1667257 | 0.0103 | 0.00102 | -3.51 | -1.11288 | -0.135 |
| RBM5 | ILMN_1786893 | 0.0143 | 0.00158 | -3.36 | -1.52118 | -0.135 |
| SPI1 | ILMN_1696463 | 0.15 | 0.037 | -2.15 | -4.36101 | -0.135 |
| RANBP9 | ILMN_2141157 | 0.0709 | 0.0133 | -2.57 | -3.46482 | -0.136 |
| SON | ILMN_1685327 | 0.12 | 0.0273 | -2.28 | -4.09546 | -0.136 |
| SCFD1 | ILMN_1800543 | 0.139 | 0.0333 | -2.19 | -4.26915 | -0.136 |
| NGLY1 | ILMN_1728779 | 0.147 | 0.036 | -2.16 | -4.33582 | -0.136 |
| RBM23 | ILMN_1780756 | 0.0493 | 0.00808 | -2.77 | -3.01382 | -0.137 |
| KMT2E | ILMN_2259633 | 0.0516 | 0.00865 | -2.74 | -3.07619 | -0.137 |
| IER3 | ILMN_1682717 | 0.108 | 0.0235 | -2.34 | -3.96565 | -0.137 |
| C1orf52 | ILMN_1742611 | 0.0799 | 0.0156 | -2.51 | -3.60383 | -0.138 |
| BCR | ILMN_2333146 | 0.0981 | 0.0206 | -2.4 | -3.84975 | -0.138 |
| RHOG | ILMN_1739792 | 0.148 | 0.0363 | -2.16 | -4.34414 | -0.138 |
| ATP5B | ILMN_1772132 | 0.0245 | 0.00324 | -3.11 | -2.18009 | -0.139 |
| MIR503HG | ILMN_1673409 | 0.0408 | 0.00626 | -2.87 | -2.78333 | -0.139 |
| ADRM1 | ILMN_2389013 | 0.0736 | 0.014 | -2.55 | -3.50777 | -0.139 |
| TM9SF3 | ILMN_1669931 | 0.0832 | 0.0165 | -2.49 | -3.65163 | -0.139 |
| GLB1 | ILMN_1735155 | 0.133 | 0.0315 | -2.22 | -4.22074 | -0.139 |
| CXorf38 | ILMN_1697864 | 0.158 | 0.0402 | -2.11 | -4.43047 | -0.14 |
| FBXO28 | ILMN_2045911 | 0.00151 | 8.18E-05 | -4.32 | 1.26395 | -0.141 |
| ATP5J | ILMN_1772929 | 0.151 | 0.0377 | -2.14 | -4.37547 | -0.141 |
| JTB | ILMN_2206716 | 0.155 | 0.039 | -2.13 | -4.40444 | -0.141 |
| TRNP1 | ILMN_1695946 | 0.0356 | 0.00523 | -2.93 | -2.61988 | -0.143 |
| STYX | ILMN_1697024 | 0.057 | 0.00991 | -2.69 | -3.19806 | -0.143 |
| USP39 | ILMN_1659523 | 0.0677 | 0.0125 | -2.6 | -3.40917 | -0.143 |
| FAM193A | ILMN_1651504 | 0.12 | 0.0272 | -2.28 | -4.09301 | -0.143 |
| GANAB | ILMN_1739441 | 0.153 | 0.0385 | -2.13 | -4.39474 | -0.143 |
| EIF4B | ILMN_1655497 | 0.00657 | 0.000568 | -3.7 | -0.56431 | -0.144 |
| ZNF274 | ILMN_1688629 | 0.178 | 0.0476 | -2.04 | -4.57532 | -0.144 |
| LAMP2 | ILMN_1673282 | 0.0149 | 0.00168 | -3.34 | -1.5757 | -0.145 |
| LAPTM5 | ILMN_1772359 | 0.0284 | 0.00389 | -3.04 | -2.34944 | -0.145 |
| LOC730101 | ILMN_3305735 | 0.0737 | 0.014 | -2.55 | -3.50913 | -0.145 |
| IGFBP3 | ILMN_2396875 | 0.104 | 0.0223 | -2.36 | -3.92085 | -0.145 |
| RHOT2 | ILMN_1669310 | 0.133 | 0.0313 | -2.22 | -4.21535 | -0.145 |
| TRIP4 | ILMN_1661173 | 0.146 | 0.0356 | -2.17 | -4.32708 | -0.145 |
| RNF38 | ILMN_1793616 | 0.0744 | 0.0142 | -2.55 | -3.52017 | -0.146 |
| GLG1 | ILMN_1772261 | 0.109 | 0.0237 | -2.34 | -3.97317 | -0.146 |
| NCL | ILMN_2121437 | 0.111 | 0.0244 | -2.33 | -3.99786 | -0.146 |
| DNAJB6 | ILMN_1793770 | 0.121 | 0.0276 | -2.28 | -4.10624 | -0.146 |
| LARP4B | ILMN_1766222 | 0.0858 | 0.0171 | -2.47 | -3.6877 | -0.147 |
| FGFR1OP | ILMN_2411116 | 0.161 | 0.0416 | -2.1 | -4.46002 | -0.147 |
| TRAPPC11 | ILMN_1752086 | 0.0484 | 0.00788 | -2.78 | -2.99135 | -0.148 |
| SLC35A2 | ILMN_1742731 | 0.13 | 0.0304 | -2.23 | -4.18894 | -0.148 |
| MTFR1L | ILMN_3264466 | 0.137 | 0.0327 | -2.2 | -4.25392 | -0.148 |
| SF3B1 | ILMN_1706075 | 0.00169 | 9.51E-05 | -4.28 | 1.12131 | -0.149 |
| FBXO28 | ILMN_1812776 | 0.00878 | 0.000826 | -3.58 | -0.91526 | -0.149 |
| NAA60 | ILMN_1813423 | 0.155 | 0.0392 | -2.12 | -4.41062 | -0.149 |
| GLB1 | ILMN_2397721 | 0.039 | 0.0059 | -2.89 | -2.7296 | -0.15 |
| SECISBP2L | ILMN_1784333 | 0.0719 | 0.0136 | -2.57 | -3.48029 | -0.15 |
| CARD8 | ILMN_2192281 | 0.0993 | 0.0209 | -2.39 | -3.8642 | -0.15 |
| SF3B4 | ILMN_1722648 | 0.0313 | 0.00443 | -2.99 | -2.46877 | -0.151 |
| GOLPH3 | ILMN_1708841 | 0.0454 | 0.00724 | -2.81 | -2.91564 | -0.151 |
| KIAA1551 | ILMN_2229922 | 0.0704 | 0.0132 | -2.58 | -3.45575 | -0.151 |
| GMIP | ILMN_1805693 | 0.0991 | 0.0209 | -2.39 | -3.8608 | -0.151 |
| BRD2 | ILMN_1758918 | 0.14 | 0.0335 | -2.19 | -4.27456 | -0.151 |
| SF3B2 | ILMN_1775939 | 0.0149 | 0.00168 | -3.34 | -1.57377 | -0.152 |
| NFYC | ILMN_1810488 | 0.0199 | 0.00244 | -3.21 | -1.92166 | -0.152 |
| RAD21 | ILMN_1748578 | 0.103 | 0.0219 | -2.37 | -3.90431 | -0.152 |
| DIS3L | ILMN_1795822 | 0.179 | 0.048 | -2.03 | -4.58222 | -0.152 |
| GNAS | ILMN_1769191 | 0.00839 | 0.000782 | -3.6 | -0.86305 | -0.153 |
| ATP5EP2 | ILMN_1756674 | 0.0179 | 0.00213 | -3.26 | -1.79479 | -0.153 |
| ELL | ILMN_1736048 | 0.0241 | 0.00317 | -3.11 | -2.16171 | -0.153 |
| GAK | ILMN_1813775 | 0.035 | 0.00512 | -2.94 | -2.5996 | -0.153 |
| KIAA1586 | ILMN_1722034 | 0.0674 | 0.0124 | -2.6 | -3.40121 | -0.153 |
| WWP1 | ILMN_2215370 | 0.106 | 0.0228 | -2.36 | -3.93804 | -0.153 |
| COPRS | ILMN_1752947 | 0.107 | 0.0232 | -2.35 | -3.95494 | -0.153 |
| ATAD1 | ILMN_1654497 | 0.144 | 0.035 | -2.17 | -4.31224 | -0.153 |
| ZNF841 | ILMN_3235593 | 0.0302 | 0.00422 | -3.01 | -2.42319 | -0.154 |
| OSTF1 | ILMN_1742456 | 0.0558 | 0.00961 | -2.7 | -3.17098 | -0.154 |
| SSR2 | ILMN_1783226 | 0.0751 | 0.0144 | -2.54 | -3.53124 | -0.154 |
| TGOLN2 | ILMN_1651735 | 0.0922 | 0.0189 | -2.43 | -3.77378 | -0.154 |
| NUDC | ILMN_2097546 | 0.146 | 0.0357 | -2.16 | -4.3297 | -0.154 |
| TRIM49B | ILMN_1759504 | 0.153 | 0.0383 | -2.13 | -4.39078 | -0.154 |
| WDR37 | ILMN_2096405 | 0.164 | 0.0423 | -2.09 | -4.47528 | -0.154 |
| IP6K1 | ILMN_1700231 | 0.0222 | 0.00283 | -3.16 | -2.05567 | -0.155 |
| TOR1AIP1 | ILMN_2141941 | 0.0253 | 0.00338 | -3.09 | -2.21964 | -0.155 |
| GOLGA5 | ILMN_1773741 | 0.0752 | 0.0144 | -2.54 | -3.53287 | -0.155 |
| CNOT7 | ILMN_1715886 | 0.161 | 0.0413 | -2.1 | -4.4536 | -0.155 |
| C1QTNF3 | ILMN_1804604 | 0.164 | 0.0424 | -2.09 | -4.47704 | -0.155 |
| ZNF174 | ILMN_1704362 | 0.172 | 0.0455 | -2.06 | -4.53698 | -0.155 |
| SKIV2L | ILMN_1666512 | 0.181 | 0.0488 | -2.02 | -4.59645 | -0.156 |
| N4BP1 | ILMN_2201966 | 0.0886 | 0.0179 | -2.46 | -3.72613 | -0.157 |
| MYL12A | ILMN_1675848 | 0.092 | 0.0188 | -2.43 | -3.77099 | -0.157 |
| RUFY1 | ILMN_1659777 | 0.125 | 0.0287 | -2.26 | -4.1415 | -0.157 |
| CBLL1 | ILMN_2073732 | 0.134 | 0.0318 | -2.21 | -4.2284 | -0.157 |
| CD58 | ILMN_1785268 | 0.147 | 0.0361 | -2.16 | -4.33855 | -0.157 |
| DCP1A | ILMN_1809285 | 0.175 | 0.0464 | -2.05 | -4.55405 | -0.157 |
| SEC16A | ILMN_2126344 | 0.122 | 0.0278 | -2.27 | -4.11195 | -0.158 |
| PTGES3L-AARSD1 | ILMN_1700461 | 0.122 | 0.0279 | -2.27 | -4.11687 | -0.158 |
| AHSA1 | ILMN_1703617 | 0.16 | 0.041 | -2.1 | -4.44868 | -0.158 |
| ILK | ILMN_2364376 | 0.00886 | 0.000838 | -3.57 | -0.92824 | -0.159 |
| BSDC1 | ILMN_1734483 | 0.116 | 0.026 | -2.3 | -4.05329 | -0.159 |
| KPNA3 | ILMN_1708427 | 0.127 | 0.0295 | -2.25 | -4.16331 | -0.159 |
| STAG1 | ILMN_2151048 | 0.163 | 0.0421 | -2.09 | -4.4705 | -0.159 |
| SH2D1A | ILMN_1705892 | 0.17 | 0.0447 | -2.06 | -4.5225 | -0.159 |
| S100A8 | ILMN_1729801 | 0.177 | 0.0473 | -2.04 | -4.56947 | -0.159 |
| ARPC5 | ILMN_1768394 | 0.00062 | 2.55E-05 | -4.68 | 2.37293 | -0.16 |
| SENP2 | ILMN_2123567 | 0.0616 | 0.011 | -2.65 | -3.29241 | -0.16 |
| BAX | ILMN_2321064 | 0.0724 | 0.0137 | -2.56 | -3.48882 | -0.16 |
| GLYR1 | ILMN_1703153 | 0.0883 | 0.0178 | -2.46 | -3.72138 | -0.16 |
| CDK7 | ILMN_1778917 | 0.102 | 0.0219 | -2.37 | -3.90398 | -0.16 |
| LSM10 | ILMN_1751803 | 0.145 | 0.0354 | -2.17 | -4.32154 | -0.16 |
| HAGHL | ILMN_1793201 | 0.15 | 0.0373 | -2.14 | -4.36718 | -0.16 |
| ZMAT3 | ILMN_1865764 | 0.162 | 0.0419 | -2.09 | -4.46773 | -0.16 |
| CLIC1 | ILMN_1756982 | 0.0127 | 0.00136 | -3.41 | -1.37891 | -0.161 |
| OSBPL2 | ILMN_1656482 | 0.0189 | 0.00228 | -3.23 | -1.85886 | -0.161 |
| MFSD11 | ILMN_1756152 | 0.0265 | 0.00359 | -3.07 | -2.2765 | -0.161 |
| PLSCR3 | ILMN_1703433 | 0.099 | 0.0208 | -2.39 | -3.85898 | -0.161 |
| IREB2 | ILMN_1726554 | 0.103 | 0.0221 | -2.37 | -3.91 | -0.161 |
| MBD1 | ILMN_2352580 | 0.153 | 0.0384 | -2.13 | -4.3925 | -0.161 |
| TMEM60 | ILMN_1752213 | 0.161 | 0.0413 | -2.1 | -4.45502 | -0.161 |
| YWHAB | ILMN_2277099 | 0.178 | 0.0476 | -2.04 | -4.57478 | -0.161 |
| KPNA4 | ILMN_1664756 | 0.00321 | 0.000225 | -4 | 0.30634 | -0.162 |
| NCOA6 | ILMN_1695797 | 0.0526 | 0.00888 | -2.73 | -3.0998 | -0.162 |
| MIDN | ILMN_1746408 | 0.116 | 0.0261 | -2.3 | -4.05729 | -0.162 |
| IQCE | ILMN_1689220 | 0.154 | 0.0388 | -2.13 | -4.40052 | -0.162 |
| CD74 | ILMN_2379644 | 0.166 | 0.0432 | -2.08 | -4.49206 | -0.162 |
| PABPC1 | ILMN_1761155 | 0.00943 | 0.000908 | -3.55 | -1.00301 | -0.163 |
| U2SURP | ILMN_2286334 | 0.0252 | 0.00336 | -3.09 | -2.21527 | -0.163 |
| SFSWAP | ILMN_1692575 | 0.0305 | 0.00428 | -3.01 | -2.43587 | -0.163 |
| FXYD5 | ILMN_1704286 | 0.0939 | 0.0194 | -2.42 | -3.79906 | -0.163 |
| HMGN3 | ILMN_1731984 | 0.0987 | 0.0208 | -2.4 | -3.85646 | -0.163 |
| LOC101927066 | ILMN_1850991 | 0.143 | 0.0347 | -2.18 | -4.30399 | -0.163 |
| NDUFS1 | ILMN_1728810 | 0.149 | 0.0367 | -2.15 | -4.35376 | -0.163 |
| MTMR14 | ILMN_1659240 | 0.0785 | 0.0152 | -2.52 | -3.58265 | -0.164 |
| POLG | ILMN_2101930 | 0.0809 | 0.0159 | -2.51 | -3.61818 | -0.164 |
| TRIM11 | ILMN_1718619 | 0.136 | 0.0323 | -2.21 | -4.24233 | -0.164 |
| JMJD7-PLA2G4B | ILMN_1697629 | 0.167 | 0.0436 | -2.08 | -4.50076 | -0.164 |
| SUMO2 | ILMN_2333594 | 0.0563 | 0.00973 | -2.7 | -3.182 | -0.165 |
| OGT | ILMN_1697639 | 0.145 | 0.0355 | -2.17 | -4.32532 | -0.165 |
| ARHGDIB | ILMN_1678143 | 0.177 | 0.0473 | -2.04 | -4.57028 | -0.165 |
| YIPF5 | ILMN_1714756 | 0.00604 | 0.00051 | -3.74 | -0.4626 | -0.166 |
| CHMP1A | ILMN_1709439 | 0.115 | 0.0256 | -2.31 | -4.04161 | -0.166 |
| WIPI2 | ILMN_2321292 | 0.134 | 0.0316 | -2.22 | -4.22461 | -0.166 |
| EXOC8 | ILMN_2083818 | 0.158 | 0.0404 | -2.11 | -4.43645 | -0.166 |
| PRMT1 | ILMN_2347234 | 0.161 | 0.0413 | -2.1 | -4.45524 | -0.166 |
| PCID2 | ILMN_1788024 | 0.181 | 0.0487 | -2.03 | -4.59449 | -0.166 |
| LYPLA2 | ILMN_1764628 | 0.184 | 0.0499 | -2.01 | -4.61515 | -0.166 |
| CSNK1G2 | ILMN_1706521 | 0.0362 | 0.00535 | -2.92 | -2.63945 | -0.167 |
| POLR2C | ILMN_1659411 | 0.051 | 0.00848 | -2.75 | -3.05816 | -0.167 |
| ATXN7L3 | ILMN_1862018 | 0.0525 | 0.00885 | -2.73 | -3.09684 | -0.167 |
| MAMLD1 | ILMN_1680856 | 0.0671 | 0.0123 | -2.6 | -3.39546 | -0.167 |
| HBD | ILMN_1815527 | 0.0732 | 0.0139 | -2.56 | -3.50301 | -0.167 |
| CCDC186 | ILMN_1734010 | 0.136 | 0.0324 | -2.21 | -4.24548 | -0.167 |
| MAU2 | ILMN_1685631 | 0.166 | 0.0434 | -2.08 | -4.49626 | -0.167 |
| GPR35 | ILMN_1710221 | 0.0253 | 0.00338 | -3.09 | -2.22012 | -0.168 |
| ZNF83 | ILMN_2190414 | 0.0376 | 0.00564 | -2.9 | -2.68843 | -0.168 |
| MRVI1 | ILMN_1798493 | 0.139 | 0.0334 | -2.19 | -4.27095 | -0.168 |
| RAPGEF1 | ILMN_1769412 | 0.142 | 0.0343 | -2.18 | -4.29438 | -0.168 |
| NPEPPS | ILMN_2116127 | 0.00647 | 0.000557 | -3.71 | -0.54547 | -0.169 |
| TMED5 | ILMN_1803279 | 0.021 | 0.00263 | -3.18 | -1.98833 | -0.169 |
| NCK1 | ILMN_1698001 | 0.0375 | 0.00562 | -2.91 | -2.68458 | -0.169 |
| FAM104A | ILMN_1807201 | 0.099 | 0.0208 | -2.39 | -3.8596 | -0.169 |
| FAM96B | ILMN_1779813 | 0.145 | 0.0356 | -2.17 | -4.32612 | -0.169 |
| TRIP12 | ILMN_1841620 | 0.177 | 0.0472 | -2.04 | -4.56875 | -0.169 |
| SUSD6 | ILMN_2226917 | 0.0209 | 0.0026 | -3.19 | -1.9779 | -0.17 |
| ZDHHC5 | ILMN_1679358 | 0.0364 | 0.00539 | -2.92 | -2.64626 | -0.17 |
| SEC14L1 | ILMN_1732575 | 0.0366 | 0.00543 | -2.92 | -2.65314 | -0.17 |
| SPI1 | ILMN_2392043 | 0.0703 | 0.0132 | -2.58 | -3.45275 | -0.17 |
| PAPOLA | ILMN_1798354 | 0.0703 | 0.0132 | -2.58 | -3.45432 | -0.17 |
| ANKRD13D | ILMN_1790351 | 0.146 | 0.0356 | -2.16 | -4.32736 | -0.17 |
| CBLL1 | ILMN_1705433 | 0.161 | 0.0416 | -2.1 | -4.45964 | -0.17 |
| CDK12 | ILMN_1707448 | 0.163 | 0.0422 | -2.09 | -4.47193 | -0.17 |
| PPP4R1 | ILMN_2345512 | 0.0232 | 0.003 | -3.13 | -2.11213 | -0.171 |
| MPC1 | ILMN_2226324 | 0.078 | 0.0151 | -2.53 | -3.57423 | -0.171 |
| USP16 | ILMN_2397230 | 0.00463 | 0.00036 | -3.85 | -0.13753 | -0.172 |
| USP8 | ILMN_1715188 | 0.00952 | 0.000922 | -3.54 | -1.01747 | -0.172 |
| IP6K1 | ILMN_2392286 | 0.015 | 0.00169 | -3.34 | -1.58175 | -0.172 |
| RBBP5 | ILMN_1696532 | 0.0362 | 0.00534 | -2.92 | -2.6391 | -0.172 |
| SHKBP1 | ILMN_1765493 | 0.0918 | 0.0188 | -2.44 | -3.76899 | -0.172 |
| MED1 | ILMN_1721729 | 0.115 | 0.0258 | -2.3 | -4.04653 | -0.172 |
| INO80D | ILMN_1856861 | 0.147 | 0.0362 | -2.16 | -4.34125 | -0.172 |
| PSMC2 | ILMN_1768784 | 0.0194 | 0.00235 | -3.22 | -1.88579 | -0.173 |
| SYVN1 | ILMN_1803143 | 0.0839 | 0.0167 | -2.49 | -3.66193 | -0.173 |
| CUL1 | ILMN_1749629 | 0.122 | 0.0281 | -2.27 | -4.12049 | -0.173 |
| C3orf62 | ILMN_1679339 | 0.161 | 0.0412 | -2.1 | -4.45221 | -0.173 |
| ZZEF1 | ILMN_1786396 | 0.0118 | 0.00124 | -3.44 | -1.29452 | -0.174 |
| ARPC1A | ILMN_1759915 | 0.0196 | 0.00239 | -3.22 | -1.90057 | -0.174 |
| TACC3 | ILMN_1724407 | 0.045 | 0.00716 | -2.82 | -2.90444 | -0.174 |
| WDR1 | ILMN_1675844 | 0.0643 | 0.0116 | -2.63 | -3.33898 | -0.174 |
| LSM1 | ILMN_2218450 | 0.0669 | 0.0123 | -2.61 | -3.39195 | -0.174 |
| PDPK1 | ILMN_2387799 | 0.0927 | 0.019 | -2.43 | -3.78039 | -0.174 |
| CHRNB4 | ILMN_3249574 | 0.148 | 0.0366 | -2.15 | -4.35053 | -0.174 |
| ZNF397 | ILMN_1685467 | 0.00551 | 0.000451 | -3.78 | -0.34873 | -0.175 |
| ARPP19 | ILMN_2059505 | 0.0993 | 0.021 | -2.39 | -3.865 | -0.175 |
| ZNF274 | ILMN_2352574 | 0.14 | 0.0337 | -2.19 | -4.27894 | -0.175 |
| CRKL | ILMN_1690122 | 0.00202 | 0.000122 | -4.2 | 0.88216 | -0.176 |
| ZSWIM8 | ILMN_1669433 | 0.0175 | 0.00207 | -3.27 | -1.76759 | -0.176 |
| CD58 | ILMN_2147517 | 0.036 | 0.0053 | -2.93 | -2.63209 | -0.176 |
| RBM10 | ILMN_1765082 | 0.0829 | 0.0164 | -2.49 | -3.64762 | -0.176 |
| COIL | ILMN_1688034 | 0.173 | 0.0456 | -2.06 | -4.53851 | -0.176 |
| GAPDH | ILMN_1343295 | 0.179 | 0.0479 | -2.03 | -4.58127 | -0.176 |
| FOXO4 | ILMN_1712095 | 0.0464 | 0.00746 | -2.8 | -2.94262 | -0.177 |
| NECAP1 | ILMN_1726359 | 0.0501 | 0.00825 | -2.76 | -3.03305 | -0.177 |
| ACAP2 | ILMN_2088825 | 0.0604 | 0.0107 | -2.66 | -3.26804 | -0.177 |
| RPN1 | ILMN_1660533 | 0.101 | 0.0215 | -2.38 | -3.88798 | -0.177 |
| ZDHHC23 | ILMN_1736901 | 0.113 | 0.025 | -2.32 | -4.01942 | -0.177 |
| TSC1 | ILMN_2246510 | 0.179 | 0.0482 | -2.03 | -4.58538 | -0.177 |
| ZSWIM1 | ILMN_1812856 | 0.015 | 0.0017 | -3.33 | -1.58476 | -0.178 |
| SLC2A3 | ILMN_1775708 | 0.0197 | 0.00241 | -3.21 | -1.90914 | -0.178 |
| DCLK3 | ILMN_3246264 | 0.0562 | 0.00971 | -2.7 | -3.17974 | -0.178 |
| PRKAA1 | ILMN_2357577 | 0.058 | 0.0102 | -2.68 | -3.22012 | -0.178 |
| SLAIN2 | ILMN_3234775 | 0.101 | 0.0215 | -2.38 | -3.8869 | -0.178 |
| MTMR3 | ILMN_1803925 | 0.104 | 0.0224 | -2.36 | -3.92336 | -0.178 |
| RGS10 | ILMN_1733538 | 0.159 | 0.0406 | -2.11 | -4.43874 | -0.178 |
| UBE2Q2 | ILMN_1683817 | 0.165 | 0.043 | -2.08 | -4.48932 | -0.178 |
| TMEM63B | ILMN_1728349 | 0.176 | 0.0467 | -2.04 | -4.55837 | -0.178 |
| MANBA | ILMN_1800733 | 0.178 | 0.0477 | -2.03 | -4.57765 | -0.178 |
| INTS4 | ILMN_1660844 | 0.0309 | 0.00436 | -3 | -2.45408 | -0.179 |
| DNAJA2 | ILMN_1770127 | 0.0366 | 0.00544 | -2.92 | -2.65556 | -0.18 |
| ZDHHC18 | ILMN_1668270 | 0.0693 | 0.0129 | -2.59 | -3.43627 | -0.18 |
| FEZ2 | ILMN_1739586 | 0.0908 | 0.0185 | -2.44 | -3.75664 | -0.18 |
| LOC100130705 | ILMN_1873967 | 0.121 | 0.0276 | -2.28 | -4.10598 | -0.18 |
| CD82 | ILMN_1662973 | 0.147 | 0.0359 | -2.16 | -4.33472 | -0.18 |
| COPB1 | ILMN_1699112 | 0.00419 | 0.000317 | -3.89 | -0.01746 | -0.181 |
| C5orf42 | ILMN_1677303 | 0.0202 | 0.00249 | -3.2 | -1.93892 | -0.181 |
| ERF | ILMN_1652749 | 0.0462 | 0.00742 | -2.8 | -2.93728 | -0.181 |
| KRCC1 | ILMN_1745620 | 0.0489 | 0.00797 | -2.77 | -3.00184 | -0.181 |
| NSUN5 | ILMN_2294878 | 0.0669 | 0.0123 | -2.61 | -3.39189 | -0.181 |
| PIAS4 | ILMN_1802905 | 0.0681 | 0.0127 | -2.59 | -3.41798 | -0.181 |
| RNF167 | ILMN_1794726 | 0.015 | 0.00168 | -3.34 | -1.57789 | -0.182 |
| CALM3 | ILMN_1666385 | 0.0444 | 0.00702 | -2.82 | -2.88774 | -0.182 |
| CEPT1 | ILMN_1676588 | 0.0939 | 0.0194 | -2.42 | -3.7989 | -0.182 |
| DMAP1 | ILMN_2328813 | 0.0993 | 0.0209 | -2.39 | -3.86368 | -0.182 |
| PSAP | ILMN_2355559 | 0.151 | 0.0375 | -2.14 | -4.37107 | -0.182 |
| EHD2 | ILMN_1808777 | 0.0155 | 0.00177 | -3.32 | -1.62139 | -0.183 |
| TRIM41 | ILMN_1729495 | 0.0215 | 0.0027 | -3.17 | -2.01476 | -0.183 |
| UBE2E1 | ILMN_1806778 | 0.0663 | 0.0122 | -2.61 | -3.38118 | -0.183 |
| RASA1 | ILMN_1725312 | 0.0867 | 0.0174 | -2.47 | -3.69888 | -0.183 |
| TUG1 | ILMN_1682783 | 0.0215 | 0.0027 | -3.17 | -2.01538 | -0.184 |
| PTPRR | ILMN_1669172 | 0.0302 | 0.00422 | -3.01 | -2.42341 | -0.184 |
| TMEM230 | ILMN_2404539 | 0.0604 | 0.0107 | -2.66 | -3.26748 | -0.184 |
| RCOR3 | ILMN_1682095 | 0.0932 | 0.0192 | -2.43 | -3.78837 | -0.184 |
| IFT52 | ILMN_1673172 | 0.15 | 0.0371 | -2.15 | -4.36311 | -0.184 |
| CMTM6 | ILMN_1696494 | 0.0017 | 9.65E-05 | -4.27 | 1.10782 | -0.185 |
| ZBTB17 | ILMN_1711048 | 0.015 | 0.0017 | -3.33 | -1.58385 | -0.185 |
| ABHD3 | ILMN_1801767 | 0.0334 | 0.00482 | -2.96 | -2.54583 | -0.185 |
| TPSAB1 | ILMN_1676256 | 0.0408 | 0.00625 | -2.87 | -2.78213 | -0.185 |
| VCL | ILMN_2413527 | 0.0677 | 0.0125 | -2.6 | -3.40739 | -0.185 |
| QRFPR | ILMN_2221336 | 0.0969 | 0.0202 | -2.41 | -3.83464 | -0.185 |
| MPC1 | ILMN_1666967 | 0.113 | 0.025 | -2.32 | -4.0186 | -0.185 |
| PPP3CA | ILMN_1670970 | 0.161 | 0.0414 | -2.1 | -4.45617 | -0.185 |
| ZNF500 | ILMN_1700238 | 0.178 | 0.0476 | -2.04 | -4.57486 | -0.185 |
| PUM2 | ILMN_1728305 | 0.000765 | 3.37E-05 | -4.6 | 2.1085 | -0.186 |
| PPP4C | ILMN_3248975 | 0.00146 | 7.81E-05 | -4.34 | 1.30824 | -0.186 |
| PRRC2C | ILMN_1743137 | 0.00166 | 9.33E-05 | -4.28 | 1.13972 | -0.186 |
| ATP5EP2 | ILMN_2225887 | 0.0032 | 0.000224 | -4.01 | 0.30985 | -0.186 |
| APPBP2 | ILMN_2149766 | 0.0255 | 0.00341 | -3.09 | -2.22947 | -0.186 |
| MGEA5 | ILMN_1686750 | 0.0507 | 0.00839 | -2.75 | -3.04878 | -0.186 |
| SYS1 | ILMN_1756590 | 0.0517 | 0.00866 | -2.74 | -3.07694 | -0.186 |
| TMED9 | ILMN_1743655 | 0.0558 | 0.00961 | -2.7 | -3.17058 | -0.186 |
| GNAI2 | ILMN_1775762 | 0.0745 | 0.0142 | -2.55 | -3.52241 | -0.186 |
| SPAST | ILMN_1796738 | 0.0781 | 0.0151 | -2.52 | -3.57698 | -0.186 |
| RALGDS | ILMN_1699856 | 0.0975 | 0.0204 | -2.4 | -3.84212 | -0.186 |
| ZNRD1 | ILMN_2398587 | 0.16 | 0.0411 | -2.1 | -4.45004 | -0.186 |
| SLC16A3 | ILMN_2364022 | 0.00202 | 0.000122 | -4.2 | 0.88802 | -0.187 |
| HS6ST2-AS1 | ILMN_1870609 | 0.0245 | 0.00324 | -3.11 | -2.18111 | -0.187 |
| NPTN | ILMN_2336982 | 0.0414 | 0.00639 | -2.86 | -2.8017 | -0.187 |
| TBRG4 | ILMN_1783728 | 0.0423 | 0.00657 | -2.85 | -2.82679 | -0.187 |
| SLC15A4 | ILMN_1679731 | 0.0494 | 0.00811 | -2.77 | -3.01724 | -0.187 |
| KDM3A | ILMN_1722532 | 0.0609 | 0.0108 | -2.66 | -3.27907 | -0.187 |
| KLF3 | ILMN_2048507 | 0.0989 | 0.0208 | -2.39 | -3.85811 | -0.187 |
| DNAJC7 | ILMN_1663616 | 0.044 | 0.00694 | -2.83 | -2.87681 | -0.188 |
| DCTN1 | ILMN_1793563 | 0.051 | 0.00848 | -2.75 | -3.05793 | -0.188 |
| TUBE1 | ILMN_1764398 | 0.0609 | 0.0108 | -2.66 | -3.27774 | -0.188 |
| TMEM43 | ILMN_1698605 | 0.0663 | 0.0122 | -2.61 | -3.38182 | -0.188 |
| GALNT3 | ILMN_1671039 | 0.0761 | 0.0146 | -2.54 | -3.54708 | -0.188 |
| CBFB | ILMN_1766408 | 0.122 | 0.0279 | -2.27 | -4.11546 | -0.188 |
| COPS7A | ILMN_1758412 | 0.0273 | 0.00372 | -3.06 | -2.30749 | -0.189 |
| CSNK1G1 | ILMN_1704713 | 0.0317 | 0.00451 | -2.99 | -2.48504 | -0.189 |
| EIF3G | ILMN_1689446 | 0.0509 | 0.00845 | -2.75 | -3.05479 | -0.189 |
| CD74 | ILMN_1736567 | 0.0558 | 0.00962 | -2.7 | -3.17123 | -0.189 |
| SARS | ILMN_1786972 | 0.0582 | 0.0102 | -2.68 | -3.22481 | -0.189 |
| DHX8 | ILMN_1687419 | 0.068 | 0.0126 | -2.6 | -3.4142 | -0.189 |
| TMEM115 | ILMN_1712035 | 0.0823 | 0.0162 | -2.5 | -3.63884 | -0.189 |
| WRAP53 | ILMN_1693669 | 0.178 | 0.0474 | -2.04 | -4.57226 | -0.189 |
| NDUFA3 | ILMN_1784641 | 0.18 | 0.0484 | -2.03 | -4.59026 | -0.189 |
| RING1 | ILMN_1666399 | 0.184 | 0.0499 | -2.01 | -4.61614 | -0.189 |
| E2F3 | ILMN_1669502 | 0.0422 | 0.00655 | -2.85 | -2.8244 | -0.19 |
| PPP1R11 | ILMN_1747598 | 0.0558 | 0.0096 | -2.7 | -3.16935 | -0.19 |
| CTDSPL2 | ILMN_1665655 | 0.0657 | 0.012 | -2.62 | -3.36774 | -0.19 |
| MIR1224 | ILMN_3310306 | 0.0906 | 0.0185 | -2.44 | -3.75414 | -0.19 |
| SNN | ILMN_1788251 | 0.117 | 0.0263 | -2.3 | -4.06505 | -0.19 |
| SRXN1 | ILMN_1804822 | 0.155 | 0.0393 | -2.12 | -4.41206 | -0.19 |
| CUL4B | ILMN_2385161 | 0.179 | 0.0479 | -2.03 | -4.58004 | -0.19 |
| EIF3L | ILMN_1762725 | 0.0117 | 0.00122 | -3.45 | -1.27516 | -0.191 |
| WRNIP1 | ILMN_1703036 | 0.052 | 0.00872 | -2.74 | -3.0832 | -0.191 |
| FRYL | ILMN_1747223 | 0.0651 | 0.0118 | -2.62 | -3.35439 | -0.191 |
| RDH5 | ILMN_1773395 | 0.0904 | 0.0184 | -2.44 | -3.75028 | -0.191 |
| RBM26 | ILMN_1740716 | 0.103 | 0.0221 | -2.37 | -3.91042 | -0.191 |
| ZNF45 | ILMN_1771884 | 0.107 | 0.0232 | -2.35 | -3.95524 | -0.191 |
| TOP2B | ILMN_1777663 | 0.127 | 0.0297 | -2.24 | -4.16943 | -0.191 |
| PHF20L1 | ILMN_2361186 | 0.17 | 0.0445 | -2.07 | -4.51911 | -0.191 |
| TRAPPC8 | ILMN_1796240 | 0.00192 | 0.000113 | -4.22 | 0.95662 | -0.192 |
| POGK | ILMN_1756669 | 0.00504 | 0.000404 | -3.82 | -0.24413 | -0.192 |
| DIO3 | ILMN_2075067 | 0.0999 | 0.0211 | -2.39 | -3.87091 | -0.192 |
| EIF2AK1 | ILMN_2156267 | 0.136 | 0.0324 | -2.21 | -4.24651 | -0.192 |
| C9orf78 | ILMN_1697166 | 0.15 | 0.0372 | -2.15 | -4.36526 | -0.192 |
| GLTSCR1L | ILMN_1696127 | 0.00765 | 0.000694 | -3.64 | -0.75147 | -0.193 |
| COL4A3BP | ILMN_1680109 | 0.0194 | 0.00236 | -3.22 | -1.88946 | -0.193 |
| HNRNPA0 | ILMN_1753279 | 0.0678 | 0.0126 | -2.6 | -3.41103 | -0.193 |
| FBXL3 | ILMN_1794187 | 0.0828 | 0.0163 | -2.49 | -3.645 | -0.193 |
| NMRK1 | ILMN_1674650 | 0.0904 | 0.0184 | -2.44 | -3.75131 | -0.193 |
| HCCS | ILMN_1707236 | 0.121 | 0.0276 | -2.28 | -4.10641 | -0.193 |
| NR2C2AP | ILMN_2135798 | 0.152 | 0.038 | -2.14 | -4.38312 | -0.193 |
| TNFRSF1B | ILMN_1764788 | 0.00499 | 0.000398 | -3.82 | -0.23064 | -0.194 |
| SMARCD1 | ILMN_1728845 | 0.0484 | 0.00786 | -2.78 | -2.98966 | -0.194 |
| ERBIN | ILMN_2352401 | 0.0535 | 0.0091 | -2.72 | -3.12178 | -0.194 |
| DDOST | ILMN_1734231 | 0.0645 | 0.0116 | -2.63 | -3.34312 | -0.194 |
| STX3 | ILMN_1659544 | 0.15 | 0.0371 | -2.15 | -4.36154 | -0.194 |
| KAT2B | ILMN_3243142 | 0.155 | 0.0393 | -2.12 | -4.41125 | -0.194 |
| LDB1 | ILMN_1811347 | 0.0104 | 0.00104 | -3.5 | -1.12973 | -0.195 |
| NADSYN1 | ILMN_1779034 | 0.0386 | 0.00581 | -2.89 | -2.71596 | -0.195 |
| TMEM159 | ILMN_2042941 | 0.117 | 0.0262 | -2.3 | -4.06108 | -0.195 |
| FBXW11 | ILMN_2394576 | 0.177 | 0.047 | -2.04 | -4.56539 | -0.195 |
| GPBP1L1 | ILMN_1662719 | 0.0162 | 0.00188 | -3.3 | -1.67905 | -0.196 |
| ACTB | ILMN_2038777 | 0.0263 | 0.00355 | -3.07 | -2.26531 | -0.196 |
| FAM53C | ILMN_1744508 | 0.0439 | 0.00691 | -2.83 | -2.87264 | -0.196 |
| WASH1 | ILMN_1655952 | 0.118 | 0.0266 | -2.29 | -4.07392 | -0.196 |
| METTL25 | ILMN_1745497 | 0.144 | 0.0349 | -2.17 | -4.30936 | -0.196 |
| ZNF787 | ILMN_1791388 | 0.164 | 0.0426 | -2.09 | -4.48065 | -0.196 |
| DENND5A | ILMN_1785356 | 0.0095 | 0.000918 | -3.54 | -1.01311 | -0.197 |
| XRCC5 | ILMN_2105983 | 0.0192 | 0.00232 | -3.22 | -1.87568 | -0.197 |
| SETD2 | ILMN_1769473 | 0.023 | 0.00297 | -3.14 | -2.10284 | -0.197 |
| ARHGAP1 | ILMN_1732060 | 0.0304 | 0.00426 | -3.01 | -2.43266 | -0.197 |
| EIF4G1 | ILMN_2370772 | 0.068 | 0.0126 | -2.6 | -3.41556 | -0.197 |
| AP1G1 | ILMN_2399627 | 0.0803 | 0.0157 | -2.51 | -3.60889 | -0.197 |
| BRWD3 | ILMN_1757794 | 0.106 | 0.023 | -2.35 | -3.94726 | -0.197 |
| POLR2A | ILMN_1782385 | 0.0217 | 0.00273 | -3.17 | -2.02381 | -0.198 |
| GNL2 | ILMN_1761113 | 0.0437 | 0.00688 | -2.83 | -2.86829 | -0.198 |
| LEF1 | ILMN_1679185 | 0.13 | 0.0304 | -2.23 | -4.19106 | -0.198 |
| CXXC5 | ILMN_1745256 | 0.178 | 0.0477 | -2.03 | -4.57767 | -0.198 |
| EPN1 | ILMN_1772981 | 0.000603 | 2.45E-05 | -4.69 | 2.41041 | -0.199 |
| RAB3GAP2 | ILMN_3236423 | 0.0296 | 0.00411 | -3.02 | -2.39977 | -0.199 |
| ELMOD2 | ILMN_1765159 | 0.0532 | 0.00905 | -2.73 | -3.11652 | -0.199 |
| WSB1 | ILMN_2415748 | 0.0659 | 0.012 | -2.62 | -3.37064 | -0.199 |
| AXIN1 | ILMN_1766185 | 0.0894 | 0.0181 | -2.45 | -3.737 | -0.199 |
| CYB5R3 | ILMN_2308582 | 0.000656 | 2.72E-05 | -4.66 | 2.31181 | -0.2 |
| BRAT1 | ILMN_1804498 | 0.0177 | 0.00209 | -3.26 | -1.77573 | -0.2 |
| MTIF3 | ILMN_1718271 | 0.0688 | 0.0128 | -2.59 | -3.42739 | -0.2 |
| DDIAS | ILMN_1790100 | 0.0695 | 0.013 | -2.59 | -3.43851 | -0.2 |
| CHRNA2 | ILMN_1698849 | 0.0954 | 0.0198 | -2.41 | -3.81685 | -0.2 |
| USP16 | ILMN_2397231 | 0.000819 | 3.66E-05 | -4.57 | 2.02802 | -0.201 |
| EIF3D | ILMN_1739847 | 0.00448 | 0.000347 | -3.87 | -0.10053 | -0.201 |
| KMT2E | ILMN_1783606 | 0.0107 | 0.00108 | -3.49 | -1.16756 | -0.201 |
| CS | ILMN_1706057 | 0.0209 | 0.00259 | -3.19 | -1.97676 | -0.201 |
| KBTBD2 | ILMN_1784540 | 0.051 | 0.00846 | -2.75 | -3.05636 | -0.201 |
| CD2 | ILMN_1695025 | 0.0818 | 0.0161 | -2.5 | -3.63006 | -0.201 |
| APLP2 | ILMN_1710482 | 0.154 | 0.0387 | -2.13 | -4.39944 | -0.201 |
| ANKRD17 | ILMN_1712019 | 0.171 | 0.0449 | -2.06 | -4.52653 | -0.201 |
| VSTM4 | ILMN_2415617 | 0.176 | 0.0466 | -2.04 | -4.5581 | -0.201 |
| IST1 | ILMN_1740351 | 1.72E-05 | 2.34E-07 | -6.06 | 6.87069 | -0.202 |
| PPM1B | ILMN_1716862 | 0.0128 | 0.00138 | -3.41 | -1.38969 | -0.202 |
| UNC93B1 | ILMN_2193591 | 0.0248 | 0.00329 | -3.1 | -2.19591 | -0.202 |
| SVIL | ILMN_1671404 | 0.0314 | 0.00446 | -2.99 | -2.474 | -0.202 |
| JKAMP | ILMN_1700276 | 0.0678 | 0.0125 | -2.6 | -3.40964 | -0.202 |
| SLC9A6 | ILMN_1677829 | 0.0874 | 0.0176 | -2.46 | -3.70933 | -0.202 |
| PSAP | ILMN_1749109 | 0.104 | 0.0222 | -2.37 | -3.91628 | -0.202 |
| SRSF4 | ILMN_2175075 | 0.00144 | 7.66E-05 | -4.34 | 1.32624 | -0.203 |
| GUCD1 | ILMN_1764410 | 0.0288 | 0.00397 | -3.03 | -2.36838 | -0.203 |
| NPLOC4 | ILMN_1807600 | 0.171 | 0.0451 | -2.06 | -4.52901 | -0.203 |
| ST8SIA3 | ILMN_1697301 | 0.00777 | 0.000708 | -3.63 | -0.77043 | -0.204 |
| FBRS | ILMN_3245236 | 0.00816 | 0.000755 | -3.61 | -0.83124 | -0.204 |
| GRB2 | ILMN_1748797 | 0.00939 | 0.000903 | -3.55 | -0.99776 | -0.204 |
| FAM13B | ILMN_3258795 | 0.0104 | 0.00104 | -3.5 | -1.1253 | -0.204 |
| PRPF8 | ILMN_1738677 | 0.0136 | 0.00149 | -3.38 | -1.4621 | -0.204 |
| DRAM1 | ILMN_1669376 | 0.0369 | 0.00549 | -2.91 | -2.66302 | -0.204 |
| ZNF844 | ILMN_3238123 | 0.0868 | 0.0174 | -2.47 | -3.7006 | -0.204 |
| DHRS7B | ILMN_1707901 | 0.0953 | 0.0198 | -2.41 | -3.81407 | -0.204 |
| HIGD1A | ILMN_2230016 | 0.133 | 0.0315 | -2.22 | -4.22115 | -0.204 |
| HERC1 | ILMN_1786211 | 0.166 | 0.0434 | -2.08 | -4.49709 | -0.204 |
| ADSL | ILMN_1790757 | 0.0357 | 0.00526 | -2.93 | -2.62399 | -0.205 |
| SEC62 | ILMN_1762003 | 0.0939 | 0.0194 | -2.42 | -3.79897 | -0.205 |
| PNN | ILMN_1721703 | 0.105 | 0.0226 | -2.36 | -3.93318 | -0.205 |
| RASSF5 | ILMN_1813795 | 0.138 | 0.0332 | -2.2 | -4.26604 | -0.205 |
| ADGRE3 | ILMN_2348487 | 0.139 | 0.0335 | -2.19 | -4.27409 | -0.205 |
| VOPP1 | ILMN_2226955 | 0.144 | 0.0349 | -2.17 | -4.3101 | -0.205 |
| SEC23B | ILMN_1657483 | 0.151 | 0.0376 | -2.14 | -4.37494 | -0.205 |
| RASSF5 | ILMN_2362902 | 0.00395 | 0.000294 | -3.92 | 0.05557 | -0.206 |
| DCAF5 | ILMN_1735402 | 0.0247 | 0.00328 | -3.1 | -2.19213 | -0.206 |
| PUF60 | ILMN_1779404 | 0.0786 | 0.0152 | -2.52 | -3.58362 | -0.206 |
| SERBP1 | ILMN_1773968 | 0.104 | 0.0222 | -2.37 | -3.91552 | -0.206 |
| OSBPL8 | ILMN_1782459 | 0.17 | 0.0446 | -2.06 | -4.52037 | -0.206 |
| PPP2R5E | ILMN_1666761 | 0.0127 | 0.00136 | -3.41 | -1.37718 | -0.207 |
| SMG7 | ILMN_1706553 | 0.0189 | 0.00229 | -3.23 | -1.8606 | -0.207 |
| FAM171A1 | ILMN_1749868 | 0.123 | 0.0281 | -2.27 | -4.12231 | -0.207 |
| MRPS27 | ILMN_1711414 | 0.123 | 0.0282 | -2.27 | -4.12489 | -0.207 |
| EP300 | ILMN_1744665 | 0.135 | 0.0321 | -2.21 | -4.23714 | -0.207 |
| QARS | ILMN_1763080 | 0.142 | 0.0344 | -2.18 | -4.2971 | -0.207 |
| TAPBP | ILMN_1742450 | 0.155 | 0.0393 | -2.12 | -4.41166 | -0.207 |
| NRBP1 | ILMN_1670096 | 0.164 | 0.0424 | -2.09 | -4.47777 | -0.207 |
| MDH2 | ILMN_2079004 | 0.00543 | 0.000444 | -3.79 | -0.33326 | -0.208 |
| OSBP | ILMN_1706376 | 0.00613 | 0.00052 | -3.73 | -0.48085 | -0.208 |
| IDS | ILMN_1758626 | 0.00758 | 0.000684 | -3.64 | -0.73778 | -0.208 |
| EIF3J | ILMN_1815345 | 0.0661 | 0.0121 | -2.61 | -3.37558 | -0.208 |
| MTMR10 | ILMN_3251728 | 0.0838 | 0.0166 | -2.49 | -3.66012 | -0.208 |
| RN7SK | ILMN_2074860 | 0.124 | 0.0285 | -2.26 | -4.13389 | -0.208 |
| ZNF32 | ILMN_1680072 | 0.156 | 0.0394 | -2.12 | -4.41438 | -0.208 |
| ARAF | ILMN_1658883 | 0.00859 | 0.000804 | -3.59 | -0.88918 | -0.209 |
| DPP8 | ILMN_2312732 | 0.0523 | 0.00879 | -2.74 | -3.09016 | -0.209 |
| C11orf84 | ILMN_1795564 | 0.0526 | 0.00888 | -2.73 | -3.09985 | -0.209 |
| SCPEP1 | ILMN_1767470 | 0.114 | 0.0252 | -2.31 | -4.02798 | -0.209 |
| NPL | ILMN_1782070 | 0.174 | 0.0461 | -2.05 | -4.54737 | -0.209 |
| SCYL2 | ILMN_1711919 | 1.64E-05 | 2.15E-07 | -6.09 | 6.95104 | -0.21 |
| CASC4 | ILMN_2325574 | 0.0089 | 0.000843 | -3.57 | -0.93398 | -0.21 |
| EIF3B | ILMN_2379469 | 0.0355 | 0.0052 | -2.93 | -2.61406 | -0.21 |
| CLEC16A | ILMN_1781752 | 0.0614 | 0.011 | -2.65 | -3.28919 | -0.21 |
| ARIH2 | ILMN_1792825 | 0.0732 | 0.0139 | -2.56 | -3.50274 | -0.21 |
| GUCA1B | ILMN_2211728 | 0.0819 | 0.0161 | -2.5 | -3.63236 | -0.21 |
| CRY2 | ILMN_1796180 | 0.139 | 0.0334 | -2.19 | -4.27077 | -0.21 |
| POLR2H | ILMN_1689445 | 0.152 | 0.0382 | -2.13 | -4.38703 | -0.21 |
| TPM1 | ILMN_1685339 | 0.184 | 0.0498 | -2.01 | -4.61381 | -0.21 |
| PIAS1 | ILMN_1780598 | 0.00107 | 5.23E-05 | -4.46 | 1.68914 | -0.211 |
| CHST12 | ILMN_1735058 | 0.0159 | 0.00182 | -3.31 | -1.64736 | -0.211 |
| CUL2 | ILMN_1669252 | 0.0462 | 0.00741 | -2.8 | -2.93629 | -0.211 |
| GOLT1B | ILMN_1767837 | 0.0709 | 0.0133 | -2.57 | -3.46398 | -0.211 |
| DPP8 | ILMN_1759801 | 0.095 | 0.0197 | -2.42 | -3.8111 | -0.211 |
| KDM5C | ILMN_1798224 | 0.126 | 0.0291 | -2.25 | -4.15227 | -0.211 |
| GPATCH3 | ILMN_1686929 | 0.135 | 0.0319 | -2.21 | -4.23183 | -0.211 |
| SATB1 | ILMN_1690646 | 0.141 | 0.0339 | -2.19 | -4.28462 | -0.211 |
| PIK3R5 | ILMN_1681067 | 0.164 | 0.0427 | -2.08 | -4.48215 | -0.211 |
| GNB1 | ILMN_1760320 | 0.00173 | 9.86E-05 | -4.27 | 1.08699 | -0.212 |
| CAP1 | ILMN_1797604 | 0.0237 | 0.00308 | -3.12 | -2.13639 | -0.212 |
| TAOK3 | ILMN_3307863 | 0.0284 | 0.0039 | -3.04 | -2.35163 | -0.212 |
| KDM4A | ILMN_1736650 | 0.068 | 0.0126 | -2.6 | -3.41576 | -0.212 |
| PI4KAP1 | ILMN_1656184 | 0.14 | 0.0336 | -2.19 | -4.27777 | -0.212 |
| FAM101B | ILMN_1714418 | 0.155 | 0.0391 | -2.12 | -4.40782 | -0.212 |
| COPB2 | ILMN_1684385 | 0.00765 | 0.000692 | -3.64 | -0.74959 | -0.213 |
| LIME1 | ILMN_2183687 | 0.0144 | 0.00159 | -3.36 | -1.52656 | -0.213 |
| CLPTM1 | ILMN_1665831 | 0.0234 | 0.00304 | -3.13 | -2.12236 | -0.213 |
| CLTA | ILMN_1695420 | 0.027 | 0.00366 | -3.06 | -2.29439 | -0.213 |
| CALM1 | ILMN_1778242 | 0.0554 | 0.00951 | -2.71 | -3.16156 | -0.213 |
| FEN1 | ILMN_2160929 | 0.0558 | 0.00962 | -2.7 | -3.17173 | -0.213 |
| ARPP19 | ILMN_1772798 | 0.109 | 0.0239 | -2.34 | -3.97969 | -0.213 |
| RAD51D | ILMN_2370907 | 0.148 | 0.0365 | -2.15 | -4.34801 | -0.213 |
| GAB1 | ILMN_1781672 | 0.159 | 0.0407 | -2.11 | -4.44223 | -0.213 |
| ACIN1 | ILMN_1699636 | 0.00492 | 0.00039 | -3.83 | -0.2124 | -0.214 |
| MED23 | ILMN_1674506 | 0.0272 | 0.00371 | -3.06 | -2.30577 | -0.214 |
| YY1AP1 | ILMN_1752303 | 0.00112 | 5.52E-05 | -4.45 | 1.63812 | -0.215 |
| MGAT1 | ILMN_1761912 | 0.027 | 0.00366 | -3.06 | -2.29333 | -0.215 |
| ACTR1A | ILMN_1792314 | 0.0646 | 0.0117 | -2.63 | -3.34615 | -0.215 |
| KATNA1 | ILMN_2198393 | 0.0854 | 0.017 | -2.48 | -3.68154 | -0.215 |
| DDX41 | ILMN_1737344 | 0.0871 | 0.0175 | -2.47 | -3.70449 | -0.215 |
| N4BP1 | ILMN_1706225 | 0.132 | 0.0309 | -2.23 | -4.20451 | -0.215 |
| VPS16 | ILMN_1778078 | 0.16 | 0.0409 | -2.1 | -4.4452 | -0.215 |
| MYOM1 | ILMN_1680344 | 0.169 | 0.0442 | -2.07 | -4.51246 | -0.215 |
| RAC2 | ILMN_1709795 | 0.0048 | 0.000379 | -3.84 | -0.18447 | -0.216 |
| ZNF766 | ILMN_1793673 | 0.02 | 0.00246 | -3.2 | -1.92761 | -0.216 |
| TMEM230 | ILMN_1806312 | 0.0241 | 0.00317 | -3.12 | -2.1602 | -0.216 |
| RPL34 | ILMN_1706873 | 0.117 | 0.0263 | -2.3 | -4.06432 | -0.216 |
| ZNF830 | ILMN_3247592 | 0.134 | 0.0318 | -2.22 | -4.228 | -0.216 |
| ZC3H8 | ILMN_1657837 | 0.145 | 0.0353 | -2.17 | -4.32014 | -0.216 |
| MXD1 | ILMN_2214678 | 7.87E-05 | 1.78E-06 | -5.47 | 4.9238 | -0.217 |
| PPP4R1 | ILMN_1724544 | 0.00316 | 0.00022 | -4.01 | 0.3293 | -0.217 |
| MPHOSPH8 | ILMN_2054213 | 0.0237 | 0.00309 | -3.12 | -2.13812 | -0.217 |
| G3BP2 | ILMN_1720422 | 0.039 | 0.00591 | -2.89 | -2.73129 | -0.217 |
| PI4KB | ILMN_1666597 | 0.057 | 0.00992 | -2.69 | -3.19905 | -0.217 |
| AGPS | ILMN_1698209 | 0.11 | 0.024 | -2.33 | -3.98457 | -0.217 |
| RPL29 | ILMN_1771051 | 0.149 | 0.0368 | -2.15 | -4.35572 | -0.217 |
| REL | ILMN_1766085 | 0.149 | 0.0369 | -2.15 | -4.35675 | -0.217 |
| ZNF148 | ILMN_2143822 | 0.00106 | 5.17E-05 | -4.47 | 1.70088 | -0.218 |
| PPM1D | ILMN_1670875 | 0.0112 | 0.00115 | -3.47 | -1.22205 | -0.218 |
| SPINT1 | ILMN_1724946 | 0.0121 | 0.00128 | -3.43 | -1.32018 | -0.218 |
| LIF | ILMN_1738725 | 0.0338 | 0.0049 | -2.96 | -2.5598 | -0.218 |
| APBB1IP | ILMN_1801710 | 0.0429 | 0.00671 | -2.84 | -2.84632 | -0.218 |
| MED13 | ILMN_1806184 | 0.0957 | 0.0199 | -2.41 | -3.81975 | -0.218 |
| POGLUT1 | ILMN_1811104 | 0.155 | 0.039 | -2.12 | -4.4062 | -0.218 |
| PPP4R3B | ILMN_1661650 | 1.22E-05 | 1.41E-07 | -6.21 | 7.35642 | -0.219 |
| ACTB | ILMN_2152131 | 0.000662 | 2.76E-05 | -4.66 | 2.29924 | -0.219 |
| RXRB | ILMN_1774074 | 0.0201 | 0.00247 | -3.2 | -1.93247 | -0.219 |
| NCF1 | ILMN_1697309 | 0.0565 | 0.0098 | -2.7 | -3.1883 | -0.219 |
| PPP6R1 | ILMN_1794085 | 0.0592 | 0.0104 | -2.67 | -3.24596 | -0.219 |
| TBL2 | ILMN_1764489 | 0.115 | 0.0256 | -2.31 | -4.04075 | -0.219 |
| CDC40 | ILMN_1737651 | 0.137 | 0.0326 | -2.2 | -4.25131 | -0.219 |
| ZNF654 | ILMN_1721629 | 0.141 | 0.0341 | -2.18 | -4.28948 | -0.219 |
| GNL1 | ILMN_1789457 | 0.144 | 0.0352 | -2.17 | -4.31613 | -0.219 |
| CD2BP2 | ILMN_1796244 | 0.0382 | 0.00575 | -2.9 | -2.70549 | -0.22 |
| DHDDS | ILMN_1789535 | 0.0409 | 0.00628 | -2.86 | -2.78659 | -0.22 |
| ZNF177 | ILMN_1787539 | 0.0413 | 0.00637 | -2.86 | -2.79864 | -0.22 |
| HELQ | ILMN_1720440 | 0.0829 | 0.0164 | -2.49 | -3.64681 | -0.22 |
| HERC3 | ILMN_1741032 | 0.135 | 0.0321 | -2.21 | -4.2368 | -0.22 |
| S1PR1 | ILMN_3235853 | 0.15 | 0.0372 | -2.15 | -4.36551 | -0.22 |
| PRPF4 | ILMN_1697440 | 0.172 | 0.0454 | -2.06 | -4.53568 | -0.22 |
| TOR1AIP1 | ILMN_1729318 | 0.000765 | 3.38E-05 | -4.6 | 2.10499 | -0.221 |
| CS | ILMN_3307648 | 0.00476 | 0.000374 | -3.84 | -0.17108 | -0.221 |
| POLR2B | ILMN_2088172 | 0.0092 | 0.000879 | -3.56 | -0.97261 | -0.221 |
| HGS | ILMN_1715994 | 0.0146 | 0.00163 | -3.35 | -1.54764 | -0.221 |
| SIRPA | ILMN_1758146 | 0.0257 | 0.00345 | -3.08 | -2.23829 | -0.221 |
| SEC24B | ILMN_1745469 | 0.0263 | 0.00356 | -3.07 | -2.26692 | -0.221 |
| KHSRP | ILMN_1673936 | 0.0684 | 0.0127 | -2.59 | -3.42248 | -0.221 |
| TMEM170B | ILMN_3236428 | 0.136 | 0.0324 | -2.21 | -4.24434 | -0.221 |
| ATXN2L | ILMN_1680380 | 0.173 | 0.0459 | -2.05 | -4.54368 | -0.221 |
| NEFL | ILMN_1659086 | 0.00772 | 0.000703 | -3.63 | -0.76348 | -0.222 |
| ARRDC1 | ILMN_1661492 | 0.0111 | 0.00113 | -3.47 | -1.21102 | -0.222 |
| ITFG1 | ILMN_1693310 | 0.0207 | 0.00257 | -3.19 | -1.96686 | -0.222 |
| CHPF2 | ILMN_1772991 | 0.0215 | 0.0027 | -3.17 | -2.01437 | -0.222 |
| GGNBP2 | ILMN_1725620 | 0.0282 | 0.00387 | -3.04 | -2.34353 | -0.222 |
| VIM | ILMN_2058251 | 0.057 | 0.00992 | -2.69 | -3.19951 | -0.222 |
| CDKN2AIP | ILMN_1773066 | 0.0662 | 0.0121 | -2.61 | -3.37879 | -0.222 |
| FHL3 | ILMN_1703558 | 0.0776 | 0.015 | -2.53 | -3.56946 | -0.222 |
| CEPT1 | ILMN_2356838 | 0.13 | 0.0305 | -2.23 | -4.19261 | -0.222 |
| HK2 | ILMN_2156172 | 0.147 | 0.0359 | -2.16 | -4.33524 | -0.222 |
| TEPSIN | ILMN_2056687 | 0.15 | 0.0374 | -2.14 | -4.3696 | -0.222 |
| ASB3 | ILMN_2358784 | 0.164 | 0.0427 | -2.08 | -4.48219 | -0.222 |
| NCF2 | ILMN_1796642 | 5.54E-05 | 1.13E-06 | -5.61 | 5.35993 | -0.223 |
| TM2D3 | ILMN_1761120 | 0.0204 | 0.00253 | -3.2 | -1.95239 | -0.223 |
| COTL1 | ILMN_1788283 | 0.042 | 0.00652 | -2.85 | -2.82003 | -0.223 |
| TBC1D22A | ILMN_3251227 | 0.061 | 0.0109 | -2.65 | -3.28141 | -0.223 |
| TPRG1L | ILMN_1727479 | 0.0774 | 0.0149 | -2.53 | -3.56559 | -0.223 |
| UQCR10 | ILMN_2366714 | 0.137 | 0.0328 | -2.2 | -4.25476 | -0.223 |
| AKAP10 | ILMN_1718808 | 0.143 | 0.0346 | -2.18 | -4.30256 | -0.223 |
| STT3B | ILMN_2194448 | 0.153 | 0.0384 | -2.13 | -4.39252 | -0.223 |
| ZFR | ILMN_1814113 | 7.49E-05 | 1.64E-06 | -5.5 | 5.00081 | -0.224 |
| CRK | ILMN_1666494 | 0.0375 | 0.0056 | -2.91 | -2.6823 | -0.224 |
| TMEM216 | ILMN_1732577 | 0.0589 | 0.0104 | -2.67 | -3.23938 | -0.224 |
| TRIOBP | ILMN_2370588 | 0.0671 | 0.0123 | -2.6 | -3.3956 | -0.224 |
| ALKBH5 | ILMN_1657283 | 0.0856 | 0.0171 | -2.47 | -3.68538 | -0.224 |
| CORO2A | ILMN_2327974 | 0.138 | 0.0331 | -2.2 | -4.26315 | -0.224 |
| PTBP1 | ILMN_1655154 | 0.00092 | 4.27E-05 | -4.52 | 1.88172 | -0.225 |
| U2AF2 | ILMN_1768930 | 0.0012 | 6.05E-05 | -4.42 | 1.55026 | -0.225 |
| N4BP2L2 | ILMN_1737561 | 0.00169 | 9.56E-05 | -4.27 | 1.11643 | -0.225 |
| XRCC6 | ILMN_1743097 | 0.00568 | 0.000468 | -3.77 | -0.38335 | -0.225 |
| TMED4 | ILMN_1804148 | 0.0122 | 0.00129 | -3.43 | -1.329 | -0.225 |
| DENND5A | ILMN_3245413 | 0.0148 | 0.00166 | -3.34 | -1.56284 | -0.225 |
| TUFM | ILMN_1738369 | 0.0562 | 0.00971 | -2.7 | -3.18032 | -0.225 |
| AGER | ILMN_1729777 | 0.068 | 0.0126 | -2.6 | -3.41496 | -0.225 |
| TBC1D3B | ILMN_2153679 | 0.082 | 0.0161 | -2.5 | -3.63326 | -0.225 |
| RPN2 | ILMN_1693421 | 0.109 | 0.0238 | -2.34 | -3.97732 | -0.225 |
| TMBIM6 | ILMN_1693311 | 0.00521 | 0.000421 | -3.8 | -0.28329 | -0.226 |
| MSL1 | ILMN_3245973 | 0.0171 | 0.002 | -3.28 | -1.73713 | -0.226 |
| CNOT7 | ILMN_1656134 | 0.0314 | 0.00445 | -2.99 | -2.4717 | -0.226 |
| TBC1D23 | ILMN_2228044 | 0.0507 | 0.00837 | -2.76 | -3.04665 | -0.226 |
| RELT | ILMN_1748614 | 0.0532 | 0.00902 | -2.73 | -3.11398 | -0.226 |
| KDM7A | ILMN_3238369 | 0.0658 | 0.012 | -2.62 | -3.36935 | -0.226 |
| PPIL3 | ILMN_2285490 | 0.0978 | 0.0205 | -2.4 | -3.84559 | -0.226 |
| HCST | ILMN_1699931 | 0.12 | 0.0272 | -2.28 | -4.09319 | -0.226 |
| NELFB | ILMN_1742432 | 0.145 | 0.0354 | -2.17 | -4.32172 | -0.226 |
| MBD6 | ILMN_1746012 | 0.00728 | 0.000646 | -3.66 | -0.68483 | -0.227 |
| EOMES | ILMN_1760509 | 0.0162 | 0.00187 | -3.3 | -1.6765 | -0.227 |
| NAP1L4 | ILMN_1804327 | 0.0622 | 0.0111 | -2.65 | -3.30314 | -0.227 |
| SLC12A4 | ILMN_2084059 | 0.0699 | 0.0131 | -2.58 | -3.44563 | -0.227 |
| PSMD1 | ILMN_1702837 | 0.0916 | 0.0187 | -2.44 | -3.76583 | -0.227 |
| XIAP | ILMN_1815361 | 0.101 | 0.0214 | -2.38 | -3.88452 | -0.227 |
| GTF2E2 | ILMN_1664931 | 0.00211 | 0.000129 | -4.18 | 0.83404 | -0.228 |
| WDR55 | ILMN_1678957 | 0.0407 | 0.00624 | -2.87 | -2.78029 | -0.228 |
| WRNIP1 | ILMN_2332105 | 0.0463 | 0.00744 | -2.8 | -2.9401 | -0.228 |
| PMVK | ILMN_1715896 | 0.105 | 0.0227 | -2.36 | -3.93544 | -0.228 |
| DHX33 | ILMN_1744308 | 0.129 | 0.0302 | -2.24 | -4.18323 | -0.228 |
| MAN1A2 | ILMN_1755120 | 0.149 | 0.0369 | -2.15 | -4.35819 | -0.228 |
| KANSL1 | ILMN_1678235 | 0.0142 | 0.00156 | -3.36 | -1.50767 | -0.229 |
| YWHAB | ILMN_1694385 | 0.025 | 0.00332 | -3.1 | -2.20463 | -0.229 |
| STK10 | ILMN_1651692 | 0.0366 | 0.00543 | -2.92 | -2.65355 | -0.229 |
| SUPT6H | ILMN_1758717 | 0.061 | 0.0109 | -2.65 | -3.28105 | -0.229 |
| PAM16 | ILMN_1763884 | 0.106 | 0.0228 | -2.36 | -3.93822 | -0.229 |
| RBM48 | ILMN_1742276 | 0.132 | 0.031 | -2.23 | -4.2071 | -0.229 |
| FAM98A | ILMN_1735453 | 0.17 | 0.0447 | -2.06 | -4.52108 | -0.229 |
| ECHS1 | ILMN_1718132 | 0.174 | 0.046 | -2.05 | -4.54706 | -0.229 |
| EIF4E2 | ILMN_1738326 | 0.00121 | 6.18E-05 | -4.41 | 1.5308 | -0.23 |
| CPNE3 | ILMN_1773576 | 0.0109 | 0.00112 | -3.48 | -1.19876 | -0.23 |
| ZHX2 | ILMN_2184966 | 0.0747 | 0.0143 | -2.55 | -3.52461 | -0.23 |
| TRAF7 | ILMN_2405991 | 0.0935 | 0.0193 | -2.43 | -3.79184 | -0.23 |
| LOC643387 | ILMN_3200717 | 0.102 | 0.0218 | -2.37 | -3.8991 | -0.23 |
| RNASE1 | ILMN_1795183 | 0.161 | 0.0413 | -2.1 | -4.45377 | -0.23 |
| EIF4G2 | ILMN_2380946 | 0.000906 | 4.19E-05 | -4.53 | 1.9009 | -0.231 |
| PICALM | ILMN_1715273 | 0.00092 | 4.27E-05 | -4.52 | 1.88095 | -0.231 |
| ZNF518B | ILMN_1682449 | 0.00443 | 0.000343 | -3.87 | -0.08963 | -0.231 |
| GALNT1 | ILMN_1803977 | 0.108 | 0.0236 | -2.34 | -3.96927 | -0.231 |
| ZNF317 | ILMN_1680347 | 0.12 | 0.0273 | -2.28 | -4.09567 | -0.231 |
| LLGL1 | ILMN_1798212 | 0.132 | 0.031 | -2.23 | -4.20705 | -0.231 |
| DENND1C | ILMN_1767020 | 0.148 | 0.0365 | -2.15 | -4.3482 | -0.231 |
| SEC63 | ILMN_1763989 | 0.153 | 0.0383 | -2.13 | -4.38999 | -0.231 |
| DSCR3 | ILMN_1717099 | 0.0104 | 0.00105 | -3.5 | -1.13627 | -0.232 |
| ARHGAP17 | ILMN_1718610 | 0.0182 | 0.00217 | -3.25 | -1.8135 | -0.232 |
| XRCC6 | ILMN_2166506 | 0.0309 | 0.00436 | -3 | -2.45436 | -0.232 |
| B4GALT3 | ILMN_1692267 | 0.0336 | 0.00486 | -2.96 | -2.55266 | -0.232 |
| ABRACL | ILMN_1720858 | 0.0441 | 0.00696 | -2.83 | -2.87973 | -0.232 |
| CXCR4 | ILMN_2320888 | 0.0498 | 0.00818 | -2.76 | -3.02574 | -0.232 |
| GARS | ILMN_1771026 | 0.0766 | 0.0148 | -2.53 | -3.55573 | -0.232 |
| ABCB7 | ILMN_1687840 | 0.0868 | 0.0174 | -2.47 | -3.70106 | -0.232 |
| NFX1 | ILMN_1744959 | 0.11 | 0.024 | -2.33 | -3.98555 | -0.232 |
| C3orf36 | ILMN_1795936 | 0.157 | 0.04 | -2.11 | -4.42718 | -0.232 |
| ZPR1 | ILMN_1753790 | 0.165 | 0.0428 | -2.08 | -4.48459 | -0.232 |
| PDLIM7 | ILMN_1690125 | 0.0117 | 0.00123 | -3.45 | -1.28274 | -0.233 |
| KDM6B | ILMN_2397521 | 0.0123 | 0.00131 | -3.42 | -1.34653 | -0.233 |
| BCL2L12 | ILMN_2396982 | 0.0874 | 0.0176 | -2.46 | -3.71052 | -0.233 |
| ZMYND11 | ILMN_1754234 | 0.0886 | 0.0179 | -2.46 | -3.72688 | -0.233 |
| PXN | ILMN_1684440 | 0.104 | 0.0223 | -2.37 | -3.91994 | -0.233 |
| MED23 | ILMN_1690999 | 0.132 | 0.0312 | -2.22 | -4.21189 | -0.233 |
| SUZ12 | ILMN_1797813 | 0.0013 | 6.76E-05 | -4.38 | 1.44533 | -0.234 |
| DR1 | ILMN_1759983 | 0.00151 | 8.11E-05 | -4.33 | 1.27194 | -0.234 |
| PTDSS1 | ILMN_1688753 | 0.0243 | 0.00319 | -3.11 | -2.16861 | -0.234 |
| DNAJB12 | ILMN_1712929 | 0.0466 | 0.00751 | -2.8 | -2.94777 | -0.234 |
| SLC15A3 | ILMN_2085862 | 0.0718 | 0.0136 | -2.57 | -3.47969 | -0.234 |
| IARS2 | ILMN_1671207 | 0.0845 | 0.0168 | -2.48 | -3.67084 | -0.234 |
| CD79B | ILMN_1785439 | 0.118 | 0.0266 | -2.29 | -4.07448 | -0.234 |
| SLBP | ILMN_2055700 | 0.121 | 0.0277 | -2.27 | -4.10982 | -0.234 |
| PURB | ILMN_1750079 | 0.125 | 0.0289 | -2.26 | -4.14557 | -0.234 |
| GNLY | ILMN_1790692 | 0.14 | 0.0338 | -2.19 | -4.28098 | -0.234 |
| DNAJB12 | ILMN_2333865 | 0.000721 | 3.12E-05 | -4.62 | 2.17943 | -0.235 |
| TOR1A | ILMN_1805812 | 0.0227 | 0.00291 | -3.15 | -2.08159 | -0.235 |
| RNF19B | ILMN_1682081 | 0.0241 | 0.00316 | -3.12 | -2.15888 | -0.235 |
| TRAPPC10 | ILMN_1778464 | 0.0336 | 0.00485 | -2.96 | -2.54995 | -0.235 |
| GREM1 | ILMN_1752965 | 0.0573 | 0.00999 | -2.69 | -3.20555 | -0.235 |
| RAB33A | ILMN_1724708 | 0.0951 | 0.0197 | -2.42 | -3.81221 | -0.235 |
| HSPA6 | ILMN_1806165 | 0.126 | 0.0291 | -2.25 | -4.15123 | -0.235 |
| GCC2 | ILMN_2408730 | 0.127 | 0.0295 | -2.25 | -4.16452 | -0.235 |
| KRIT1 | ILMN_1811261 | 0.156 | 0.0395 | -2.12 | -4.4163 | -0.235 |
| FDFT1 | ILMN_1741096 | 0.164 | 0.0425 | -2.09 | -4.47908 | -0.235 |
| ARCN1 | ILMN_1699703 | 3.36E-05 | 5.65E-07 | -5.81 | 6.02292 | -0.236 |
| LSP1 | ILMN_1654778 | 0.0181 | 0.00216 | -3.25 | -1.80643 | -0.236 |
| GMEB1 | ILMN_1727761 | 0.0363 | 0.00537 | -2.92 | -2.64303 | -0.236 |
| TRAPPC11 | ILMN_1666827 | 0.0431 | 0.00674 | -2.84 | -2.85044 | -0.236 |
| RAB5B | ILMN_1752582 | 0.0453 | 0.00724 | -2.81 | -2.91481 | -0.236 |
| MATN1 | ILMN_2061116 | 0.0507 | 0.00838 | -2.76 | -3.04741 | -0.236 |
| FLII | ILMN_1737170 | 0.0538 | 0.00918 | -2.72 | -3.12931 | -0.236 |
| GMEB2 | ILMN_1683204 | 0.0651 | 0.0118 | -2.62 | -3.35335 | -0.236 |
| PDCD2 | ILMN_1797684 | 0.0711 | 0.0134 | -2.57 | -3.46885 | -0.236 |
| KDELR2 | ILMN_1724293 | 0.074 | 0.0141 | -2.55 | -3.51419 | -0.236 |
| ANAPC5 | ILMN_1723177 | 0.0757 | 0.0145 | -2.54 | -3.54129 | -0.236 |
| PPP2R3A | ILMN_2376520 | 0.12 | 0.0273 | -2.28 | -4.09741 | -0.236 |
| VPS16 | ILMN_1734736 | 0.125 | 0.0289 | -2.26 | -4.14706 | -0.236 |
| HDC | ILMN_1792323 | 0.169 | 0.0444 | -2.07 | -4.51638 | -0.236 |
| C1QA | ILMN_1737918 | 0.178 | 0.0478 | -2.03 | -4.57847 | -0.236 |
| EIF4EBP2 | ILMN_1728083 | 0.00275 | 0.000183 | -4.07 | 0.50148 | -0.237 |
| DHRS1 | ILMN_1807206 | 0.0127 | 0.00136 | -3.41 | -1.3761 | -0.237 |
| VIM | ILMN_1782538 | 0.0339 | 0.00493 | -2.95 | -2.56561 | -0.237 |
| CEP192 | ILMN_1703754 | 0.0404 | 0.00618 | -2.87 | -2.77151 | -0.237 |
| ZNF160 | ILMN_1777049 | 0.0888 | 0.018 | -2.45 | -3.72885 | -0.237 |
| STRN4 | ILMN_1696190 | 0.0888 | 0.018 | -2.45 | -3.72907 | -0.237 |
| XPR1 | ILMN_3250032 | 0.15 | 0.0374 | -2.14 | -4.36868 | -0.237 |
| SNAP23 | ILMN_1687519 | 0.151 | 0.0376 | -2.14 | -4.37284 | -0.237 |
| SLC25A1 | ILMN_1813671 | 0.18 | 0.0485 | -2.03 | -4.5918 | -0.237 |
| TBL1X | ILMN_1744795 | 0.00402 | 0.000301 | -3.91 | 0.032 | -0.238 |
| OS9 | ILMN_2361807 | 0.00952 | 0.000921 | -3.54 | -1.01673 | -0.238 |
| SLC25A44 | ILMN_1810514 | 0.0405 | 0.0062 | -2.87 | -2.77376 | -0.238 |
| DCTN1 | ILMN_2412807 | 0.0678 | 0.0126 | -2.6 | -3.41055 | -0.238 |
| CCNT1 | ILMN_3239113 | 0.0748 | 0.0143 | -2.55 | -3.52669 | -0.238 |
| SNORA28 | ILMN_3235096 | 0.0833 | 0.0165 | -2.49 | -3.65323 | -0.238 |
| FAAP20 | ILMN_1726989 | 0.11 | 0.024 | -2.33 | -3.98559 | -0.238 |
| SMAGP | ILMN_1804415 | 0.111 | 0.0243 | -2.33 | -3.99561 | -0.238 |
| TNFRSF1A | ILMN_1685005 | 0.116 | 0.0259 | -2.3 | -4.05118 | -0.238 |
| ITM2C | ILMN_2366041 | 0.171 | 0.0449 | -2.06 | -4.52544 | -0.238 |
| CHORDC1 | ILMN_1776337 | 0.173 | 0.0458 | -2.05 | -4.54338 | -0.238 |
| CHST15 | ILMN_1670926 | 0.0174 | 0.00205 | -3.27 | -1.7611 | -0.239 |
| HIGD1A | ILMN_1674522 | 0.0526 | 0.00888 | -2.73 | -3.09931 | -0.239 |
| PRPF31 | ILMN_1719204 | 0.0552 | 0.00947 | -2.71 | -3.15782 | -0.239 |
| CDIP1 | ILMN_1815812 | 0.141 | 0.0341 | -2.18 | -4.28923 | -0.239 |
| SDHAF1 | ILMN_1805720 | 0.158 | 0.04 | -2.11 | -4.42779 | -0.239 |
| ZNF451 | ILMN_1706734 | 0.167 | 0.0435 | -2.08 | -4.49931 | -0.239 |
| PRPF3 | ILMN_1715392 | 0.000614 | 2.51E-05 | -4.69 | 2.38704 | -0.24 |
| PUM1 | ILMN_1783424 | 0.00329 | 0.000233 | -3.99 | 0.27369 | -0.24 |
| LRIF1 | ILMN_1756811 | 0.00336 | 0.000239 | -3.99 | 0.24899 | -0.24 |
| TRIM41 | ILMN_1813027 | 0.00884 | 0.000835 | -3.58 | -0.92453 | -0.24 |
| RAPGEFL1 | ILMN_1758613 | 0.0238 | 0.00311 | -3.12 | -2.1449 | -0.24 |
| SCAMP3 | ILMN_1793712 | 0.0591 | 0.0104 | -2.67 | -3.24372 | -0.24 |
| PRKCB | ILMN_3222974 | 0.0772 | 0.0149 | -2.53 | -3.5624 | -0.24 |
| STAG1 | ILMN_1792435 | 0.0927 | 0.019 | -2.43 | -3.78085 | -0.24 |
| FBXO30 | ILMN_1700232 | 0.0187 | 0.00224 | -3.24 | -1.84162 | -0.241 |
| KPNB1 | ILMN_1703949 | 0.0188 | 0.00226 | -3.23 | -1.85131 | -0.241 |
| AP1G1 | ILMN_2399622 | 0.0268 | 0.00364 | -3.07 | -2.28772 | -0.241 |
| SLC44A2 | ILMN_1771987 | 0.0313 | 0.00443 | -2.99 | -2.46737 | -0.241 |
| PIK3R4 | ILMN_1715832 | 0.0366 | 0.00542 | -2.92 | -2.65221 | -0.241 |
| GIGYF2 | ILMN_1750718 | 0.056 | 0.00964 | -2.7 | -3.1738 | -0.241 |
| TRAPPC4 | ILMN_1814650 | 0.109 | 0.0238 | -2.34 | -3.97798 | -0.241 |
| MAP2K3 | ILMN_1790534 | 0.117 | 0.0262 | -2.3 | -4.05951 | -0.241 |
| AGGF1 | ILMN_2064917 | 0.132 | 0.0311 | -2.22 | -4.21022 | -0.241 |
| PDE3B | ILMN_2131381 | 0.136 | 0.0324 | -2.21 | -4.24597 | -0.241 |
| DTX2 | ILMN_2275098 | 0.00192 | 0.000114 | -4.22 | 0.9531 | -0.242 |
| ADD1 | ILMN_1759252 | 0.0148 | 0.00165 | -3.34 | -1.56059 | -0.242 |
| TSHZ3 | ILMN_1743933 | 0.02 | 0.00246 | -3.2 | -1.92758 | -0.242 |
| SLC31A1 | ILMN_1804562 | 0.043 | 0.00673 | -2.84 | -2.84852 | -0.242 |
| CBX6 | ILMN_1691930 | 0.0648 | 0.0117 | -2.63 | -3.34888 | -0.242 |
| NKAP | ILMN_1675359 | 0.117 | 0.0263 | -2.3 | -4.06485 | -0.242 |
| TRIM26 | ILMN_1738704 | 0.119 | 0.0269 | -2.29 | -4.08431 | -0.242 |
| MED8 | ILMN_2339705 | 0.024 | 0.00315 | -3.12 | -2.1567 | -0.243 |
| TRMT1L | ILMN_1665300 | 0.042 | 0.00651 | -2.85 | -2.8189 | -0.243 |
| SLBP | ILMN_1753353 | 0.046 | 0.00738 | -2.8 | -2.93236 | -0.243 |
| CEP164 | ILMN_1740385 | 0.113 | 0.0248 | -2.32 | -4.01436 | -0.243 |
| OMG | ILMN_1739235 | 0.14 | 0.0337 | -2.19 | -4.27872 | -0.243 |
| FBXO11 | ILMN_1713682 | 5.10E-05 | 1.01E-06 | -5.64 | 5.46304 | -0.244 |
| G3BP2 | ILMN_2381753 | 0.00434 | 0.000333 | -3.88 | -0.06276 | -0.244 |
| CLASP1 | ILMN_1700507 | 0.0118 | 0.00123 | -3.44 | -1.2869 | -0.244 |
| MED12 | ILMN_1793386 | 0.0218 | 0.00275 | -3.17 | -2.03126 | -0.244 |
| TCIRG1 | ILMN_1711994 | 0.0237 | 0.00307 | -3.13 | -2.13225 | -0.244 |
| FCF1 | ILMN_2189870 | 0.0478 | 0.00775 | -2.79 | -2.97624 | -0.244 |
| RAB8A | ILMN_1760858 | 0.0569 | 0.00989 | -2.69 | -3.19622 | -0.244 |
| GPR108 | ILMN_2396571 | 0.0672 | 0.0124 | -2.6 | -3.39621 | -0.244 |
| MEMO1 | ILMN_1741599 | 0.119 | 0.0269 | -2.29 | -4.08356 | -0.244 |
| ZBTB4 | ILMN_1795905 | 0.125 | 0.029 | -2.25 | -4.15013 | -0.244 |
| BAG4 | ILMN_1727996 | 0.127 | 0.0294 | -2.25 | -4.16224 | -0.244 |
| LINC00294 | ILMN_3244521 | 0.16 | 0.0411 | -2.1 | -4.45045 | -0.244 |
| TPM1 | ILMN_2360710 | 0.165 | 0.043 | -2.08 | -4.48798 | -0.244 |
| ANKHD1-EIF4EBP3 | ILMN_1766560 | 0.182 | 0.0489 | -2.02 | -4.59897 | -0.244 |
| EIF3L | ILMN_3240740 | 0.000286 | 9.40E-06 | -4.98 | 3.32675 | -0.245 |
| ZFP91 | ILMN_1665423 | 0.00211 | 0.00013 | -4.18 | 0.82681 | -0.245 |
| CKLF | ILMN_1712389 | 0.0202 | 0.00249 | -3.2 | -1.93872 | -0.245 |
| RTF1 | ILMN_1791039 | 0.0366 | 0.00544 | -2.92 | -2.65512 | -0.245 |
| VRK3 | ILMN_1771697 | 0.0414 | 0.00638 | -2.86 | -2.80085 | -0.245 |
| BAG6 | ILMN_1705364 | 0.0444 | 0.00703 | -2.82 | -2.88817 | -0.245 |
| THAP6 | ILMN_1661424 | 0.0445 | 0.00707 | -2.82 | -2.89317 | -0.245 |
| TIA1 | ILMN_2388466 | 0.0583 | 0.0102 | -2.68 | -3.22599 | -0.245 |
| TTF1 | ILMN_1713129 | 0.0602 | 0.0107 | -2.66 | -3.26417 | -0.245 |
| SENP2 | ILMN_1801121 | 0.0622 | 0.0111 | -2.64 | -3.30408 | -0.245 |
| SRSF10 | ILMN_2354649 | 0.151 | 0.0376 | -2.14 | -4.37316 | -0.245 |
| HMGN5 | ILMN_1749799 | 0.165 | 0.0427 | -2.08 | -4.48354 | -0.245 |
| OTULIN | ILMN_1790062 | 0.0105 | 0.00105 | -3.5 | -1.14083 | -0.246 |
| PDLIM7 | ILMN_2396639 | 0.0305 | 0.00428 | -3.01 | -2.43671 | -0.246 |
| TMEM154 | ILMN_2088124 | 0.041 | 0.0063 | -2.86 | -2.78897 | -0.246 |
| ELOVL1 | ILMN_1798123 | 0.0483 | 0.00785 | -2.78 | -2.98786 | -0.246 |
| GTF2IP1 | ILMN_1815668 | 0.0507 | 0.00839 | -2.75 | -3.04799 | -0.246 |
| C1orf131 | ILMN_1805474 | 0.0646 | 0.0117 | -2.63 | -3.34529 | -0.246 |
| HMBOX1 | ILMN_1720059 | 0.113 | 0.0249 | -2.32 | -4.01473 | -0.246 |
| ITM2C | ILMN_1680453 | 0.17 | 0.0447 | -2.06 | -4.52212 | -0.246 |
| MYH9 | ILMN_1722872 | 0.00225 | 0.000142 | -4.15 | 0.74323 | -0.247 |
| MFSD5 | ILMN_1702065 | 0.0202 | 0.00249 | -3.2 | -1.93801 | -0.247 |
| HIGD1A | ILMN_1661799 | 0.0237 | 0.0031 | -3.12 | -2.14067 | -0.247 |
| GPATCH2L | ILMN_2265093 | 0.0245 | 0.00325 | -3.11 | -2.18298 | -0.247 |
| ZSWIM8 | ILMN_1803743 | 0.0272 | 0.00371 | -3.06 | -2.30594 | -0.247 |
| C16orf70 | ILMN_1711703 | 0.0375 | 0.00562 | -2.91 | -2.68526 | -0.247 |
| TRAPPC11 | ILMN_2338921 | 0.0463 | 0.00744 | -2.8 | -2.93934 | -0.247 |
| FLI1 | ILMN_1665738 | 0.0525 | 0.00884 | -2.73 | -3.0952 | -0.247 |
| PLAGL1 | ILMN_1815121 | 0.0546 | 0.00935 | -2.71 | -3.14568 | -0.247 |
| YY1 | ILMN_2181540 | 0.104 | 0.0223 | -2.37 | -3.91884 | -0.247 |
| MRPL20 | ILMN_2189424 | 0.125 | 0.0289 | -2.26 | -4.14665 | -0.247 |
| SPOCK2 | ILMN_1656287 | 0.158 | 0.0404 | -2.11 | -4.4355 | -0.247 |
| RPIA | ILMN_1714809 | 0.174 | 0.0461 | -2.05 | -4.54797 | -0.247 |
| TKT | ILMN_1736597 | 0.176 | 0.0468 | -2.04 | -4.56106 | -0.247 |
| PSMD7 | ILMN_1776173 | 0.00266 | 0.000175 | -4.08 | 0.54348 | -0.248 |
| STAMBP | ILMN_2391355 | 0.00751 | 0.000674 | -3.65 | -0.72491 | -0.248 |
| CHD4 | ILMN_1658411 | 0.0262 | 0.00353 | -3.08 | -2.26056 | -0.248 |
| DNAJC4 | ILMN_3307827 | 0.0379 | 0.0057 | -2.9 | -2.698 | -0.248 |
| ANKRD13A | ILMN_1689908 | 0.0499 | 0.00819 | -2.76 | -3.02692 | -0.248 |
| AP1G2 | ILMN_1754179 | 0.0551 | 0.00944 | -2.71 | -3.15493 | -0.248 |
| CHD4 | ILMN_1685551 | 0.0663 | 0.0121 | -2.61 | -3.3804 | -0.248 |
| RNF125 | ILMN_1747192 | 0.0939 | 0.0194 | -2.42 | -3.7989 | -0.248 |
| MTOR | ILMN_1769031 | 0.00751 | 0.000675 | -3.65 | -0.72543 | -0.249 |
| VBP1 | ILMN_1800612 | 0.0189 | 0.00227 | -3.23 | -1.85377 | -0.249 |
| C14orf2 | ILMN_1652722 | 0.0229 | 0.00296 | -3.14 | -2.09816 | -0.249 |
| ALDOA | ILMN_1741148 | 0.033 | 0.00475 | -2.97 | -2.531 | -0.249 |
| EVI5L | ILMN_1747281 | 0.079 | 0.0153 | -2.52 | -3.589 | -0.249 |
| PARP1 | ILMN_1686871 | 0.151 | 0.0376 | -2.14 | -4.37317 | -0.249 |
| LXN | ILMN_1723962 | 0.162 | 0.0419 | -2.09 | -4.46713 | -0.249 |
| ACAP2 | ILMN_1766798 | 0.000137 | 3.52E-06 | -5.27 | 4.26721 | -0.25 |
| STAMBP | ILMN_1739253 | 0.00082 | 3.67E-05 | -4.57 | 2.02666 | -0.25 |
| ITGA5 | ILMN_1792679 | 0.00283 | 0.000191 | -4.06 | 0.46333 | -0.25 |
| ZYX | ILMN_1701875 | 0.00525 | 0.000426 | -3.8 | -0.29401 | -0.25 |
| CAPRIN1 | ILMN_1754145 | 0.00532 | 0.000432 | -3.79 | -0.30833 | -0.25 |
| IWS1 | ILMN_1747241 | 0.0313 | 0.00444 | -2.99 | -2.46957 | -0.25 |
| GNL3L | ILMN_1708414 | 0.0342 | 0.00498 | -2.95 | -2.57538 | -0.25 |
| CLTB | ILMN_1674609 | 0.0591 | 0.0104 | -2.67 | -3.24471 | -0.25 |
| AIM1 | ILMN_1688625 | 0.165 | 0.0428 | -2.08 | -4.48518 | -0.25 |
| IPO13 | ILMN_1651229 | 0.176 | 0.0469 | -2.04 | -4.56317 | -0.25 |
| B9D2 | ILMN_1806999 | 0.00662 | 0.000573 | -3.7 | -0.57189 | -0.251 |
| PLRG1 | ILMN_1749634 | 0.00672 | 0.000584 | -3.69 | -0.5911 | -0.251 |
| MAP7D1 | ILMN_1777906 | 0.0118 | 0.00124 | -3.44 | -1.29195 | -0.251 |
| RAE1 | ILMN_1781454 | 0.0372 | 0.00556 | -2.91 | -2.67473 | -0.251 |
| SMARCA5 | ILMN_1713163 | 0.0415 | 0.0064 | -2.86 | -2.80386 | -0.251 |
| METAP1 | ILMN_1785795 | 0.0511 | 0.0085 | -2.75 | -3.06008 | -0.251 |
| SRRT | ILMN_2358202 | 0.0594 | 0.0105 | -2.67 | -3.25096 | -0.251 |
| PLEC | ILMN_1744268 | 0.0973 | 0.0204 | -2.4 | -3.83939 | -0.251 |
| SLC38A10 | ILMN_1759743 | 0.145 | 0.0353 | -2.17 | -4.31867 | -0.251 |
| RRP8 | ILMN_2066667 | 0.024 | 0.00315 | -3.12 | -2.15531 | -0.252 |
| PIGX | ILMN_1769508 | 0.0292 | 0.00404 | -3.03 | -2.38273 | -0.252 |
| NUDT9 | ILMN_1680239 | 0.0473 | 0.00764 | -2.79 | -2.96409 | -0.252 |
| SQSTM1 | ILMN_1662618 | 0.0581 | 0.0102 | -2.68 | -3.22258 | -0.252 |
| SET | ILMN_1742238 | 0.124 | 0.0284 | -2.26 | -4.13248 | -0.252 |
| PTGES2 | ILMN_2345016 | 0.141 | 0.0341 | -2.18 | -4.2895 | -0.252 |
| ZNF830 | ILMN_1795922 | 0.00058 | 2.31E-05 | -4.71 | 2.4667 | -0.253 |
| AP3B1 | ILMN_1768867 | 0.000884 | 4.05E-05 | -4.54 | 1.93295 | -0.253 |
| PSME4 | ILMN_1763540 | 0.0157 | 0.00179 | -3.32 | -1.63513 | -0.253 |
| FBXO18 | ILMN_1729430 | 0.0162 | 0.00186 | -3.3 | -1.67054 | -0.253 |
| UBE2Q1 | ILMN_1776325 | 0.0488 | 0.00795 | -2.78 | -2.99925 | -0.253 |
| SULT1A3 | ILMN_3250899 | 0.0662 | 0.0121 | -2.61 | -3.37863 | -0.253 |
| TMED3 | ILMN_1719316 | 0.0804 | 0.0157 | -2.51 | -3.61049 | -0.253 |
| NFATC2IP | ILMN_2379080 | 0.101 | 0.0214 | -2.38 | -3.88266 | -0.253 |
| AP2M1 | ILMN_2402798 | 0.107 | 0.0233 | -2.35 | -3.95954 | -0.253 |
| TST | ILMN_1691572 | 0.113 | 0.025 | -2.32 | -4.02087 | -0.253 |
| TRAPPC12 | ILMN_1693317 | 0.126 | 0.0293 | -2.25 | -4.15766 | -0.253 |
| TBC1D20 | ILMN_1742869 | 0.017 | 0.00199 | -3.28 | -1.73284 | -0.254 |
| GMEB1 | ILMN_2383419 | 0.0209 | 0.00259 | -3.19 | -1.97574 | -0.254 |
| NBEAL2 | ILMN_1660629 | 0.0844 | 0.0168 | -2.48 | -3.66889 | -0.254 |
| TMEM164 | ILMN_3241091 | 0.0902 | 0.0183 | -2.45 | -3.74712 | -0.254 |
| VCL | ILMN_1795429 | 0.124 | 0.0286 | -2.26 | -4.13772 | -0.254 |
| RBM25 | ILMN_1768117 | 0.000762 | 3.33E-05 | -4.6 | 2.11763 | -0.255 |
| PRR14 | ILMN_1745329 | 0.00229 | 0.000145 | -4.14 | 0.72034 | -0.255 |
| NCOA3 | ILMN_1708805 | 0.00402 | 0.000301 | -3.91 | 0.03131 | -0.255 |
| PPP2R1A | ILMN_1810467 | 0.0115 | 0.0012 | -3.45 | -1.2606 | -0.255 |
| TSC1 | ILMN_1797367 | 0.0554 | 0.00952 | -2.71 | -3.16222 | -0.255 |
| IKZF1 | ILMN_1676575 | 0.112 | 0.0246 | -2.32 | -4.00482 | -0.255 |
| VBP1 | ILMN_2223010 | 0.0144 | 0.0016 | -3.35 | -1.53089 | -0.256 |
| PSMC1 | ILMN_1710406 | 0.015 | 0.00169 | -3.34 | -1.58003 | -0.256 |
| RAB4B | ILMN_2109994 | 0.0258 | 0.00347 | -3.08 | -2.24329 | -0.256 |
| PRELID3B | ILMN_3240893 | 0.0857 | 0.0171 | -2.47 | -3.68586 | -0.256 |
| SCAMP2 | ILMN_1654893 | 0.18 | 0.0485 | -2.03 | -4.59107 | -0.256 |
| NIPBL | ILMN_2264625 | 0.0138 | 0.00151 | -3.37 | -1.47903 | -0.257 |
| DCTN5 | ILMN_1666192 | 0.014 | 0.00153 | -3.37 | -1.49118 | -0.257 |
| KANSL3 | ILMN_3187852 | 0.015 | 0.00168 | -3.34 | -1.57744 | -0.257 |
| ARID1A | ILMN_1797341 | 0.0207 | 0.00257 | -3.19 | -1.97003 | -0.257 |
| CTDSPL2 | ILMN_2077758 | 0.0311 | 0.00439 | -3 | -2.45975 | -0.257 |
| SF1 | ILMN_1712560 | 0.0482 | 0.00783 | -2.78 | -2.98565 | -0.257 |
| NEU1 | ILMN_1763144 | 0.066 | 0.0121 | -2.61 | -3.37414 | -0.257 |
| CXCL14 | ILMN_1748323 | 0.0776 | 0.015 | -2.53 | -3.56901 | -0.257 |
| BAGE2 | ILMN_2168766 | 0.113 | 0.0248 | -2.32 | -4.01394 | -0.257 |
| ARL15 | ILMN_1774161 | 0.123 | 0.0283 | -2.26 | -4.1287 | -0.257 |
| TEFM | ILMN_3307733 | 0.14 | 0.0338 | -2.19 | -4.28145 | -0.257 |
| CCT8 | ILMN_1717868 | 0.0144 | 0.0016 | -3.36 | -1.52948 | -0.258 |
| GLUL | ILMN_1653496 | 0.0245 | 0.00323 | -3.11 | -2.17953 | -0.258 |
| RFWD2 | ILMN_2408001 | 0.0255 | 0.00341 | -3.09 | -2.2272 | -0.258 |
| SACM1L | ILMN_1765019 | 0.0344 | 0.00501 | -2.95 | -2.58118 | -0.258 |
| UBAP2L | ILMN_1814789 | 0.0355 | 0.0052 | -2.93 | -2.61463 | -0.258 |
| ZHX2 | ILMN_1792951 | 0.0588 | 0.0103 | -2.68 | -3.23466 | -0.258 |
| RNF121 | ILMN_1742318 | 0.0701 | 0.0131 | -2.58 | -3.44887 | -0.258 |
| ATP6V1D | ILMN_1797310 | 0.0867 | 0.0173 | -2.47 | -3.69776 | -0.258 |
| AMD1 | ILMN_1667994 | 0.0871 | 0.0175 | -2.47 | -3.70461 | -0.258 |
| DEPDC5 | ILMN_1658717 | 0.177 | 0.047 | -2.04 | -4.56547 | -0.258 |
| CCS | ILMN_1766797 | 0.175 | 0.0463 | -2.05 | -4.55165 | -0.259 |
| GPNMB | ILMN_2407389 | 0.176 | 0.0468 | -2.04 | -4.56154 | -0.259 |
| ZNF428 | ILMN_1652754 | 0.000663 | 2.76E-05 | -4.66 | 2.29643 | -0.26 |
| FIP1L1 | ILMN_1768743 | 0.000814 | 3.63E-05 | -4.57 | 2.03577 | -0.26 |
| SSU72 | ILMN_1664956 | 0.00204 | 0.000124 | -4.19 | 0.87163 | -0.26 |
| THAP12 | ILMN_1655622 | 0.00609 | 0.000515 | -3.74 | -0.47319 | -0.26 |
| PADI2 | ILMN_1771223 | 0.0593 | 0.0105 | -2.67 | -3.24796 | -0.26 |
| FAM200B | ILMN_1675803 | 0.107 | 0.0232 | -2.35 | -3.95475 | -0.26 |
| GATAD2B | ILMN_1766359 | 0.000329 | 1.11E-05 | -4.93 | 3.16544 | -0.261 |
| FAM91A1 | ILMN_1777322 | 0.00186 | 0.000109 | -4.24 | 0.99574 | -0.261 |
| CLTA | ILMN_2345837 | 0.00596 | 0.000499 | -3.75 | -0.44328 | -0.261 |
| OTUD6B | ILMN_1772703 | 0.0366 | 0.00545 | -2.92 | -2.65659 | -0.261 |
| KMT2E | ILMN_2344988 | 0.0557 | 0.00958 | -2.7 | -3.16768 | -0.261 |
| INTS5 | ILMN_1796968 | 0.0877 | 0.0177 | -2.46 | -3.7155 | -0.261 |
| DNAH1 | ILMN_1685052 | 0.143 | 0.0346 | -2.18 | -4.30231 | -0.261 |
| RABL3 | ILMN_1662306 | 0.173 | 0.0458 | -2.05 | -4.54215 | -0.261 |
| PUM1 | ILMN_2401155 | 0.000236 | 7.35E-06 | -5.05 | 3.56233 | -0.262 |
| ATP6V1B2 | ILMN_1787705 | 0.000496 | 1.90E-05 | -4.77 | 2.65457 | -0.262 |
| GLRX | ILMN_1737308 | 0.003 | 0.000206 | -4.03 | 0.39046 | -0.262 |
| VPS37A | ILMN_2161832 | 0.00929 | 0.00089 | -3.55 | -0.98466 | -0.262 |
| HMGN1 | ILMN_2151579 | 0.0567 | 0.00985 | -2.69 | -3.19253 | -0.262 |
| ITGAE | ILMN_1683927 | 0.0917 | 0.0188 | -2.44 | -3.76767 | -0.262 |
| TBCE | ILMN_1725183 | 0.157 | 0.04 | -2.11 | -4.42687 | -0.262 |
| GDF11 | ILMN_1745132 | 0.162 | 0.0416 | -2.1 | -4.46109 | -0.262 |
| STOML2 | ILMN_1663002 | 0.0108 | 0.00109 | -3.49 | -1.17646 | -0.263 |
| RASSF2 | ILMN_2352303 | 0.012 | 0.00126 | -3.44 | -1.30703 | -0.263 |
| WDR37 | ILMN_1796464 | 0.0195 | 0.00238 | -3.22 | -1.89661 | -0.263 |
| SMAP1 | ILMN_2363231 | 0.021 | 0.00262 | -3.18 | -1.98584 | -0.263 |
| SLCO3A1 | ILMN_1706261 | 0.0213 | 0.00266 | -3.18 | -2.00155 | -0.263 |
| SNX29P2 | ILMN_1690064 | 0.154 | 0.0389 | -2.13 | -4.40346 | -0.263 |
| TBC1D20 | ILMN_2044572 | 0.0123 | 0.00131 | -3.42 | -1.34354 | -0.264 |
| LINC00347 | ILMN_1841019 | 0.0199 | 0.00244 | -3.21 | -1.92155 | -0.264 |
| ADAM10 | ILMN_1718946 | 0.0294 | 0.00408 | -3.02 | -2.39247 | -0.264 |
| ZBTB48 | ILMN_1801101 | 0.0411 | 0.00633 | -2.86 | -2.79279 | -0.264 |
| CPEB3 | ILMN_1755954 | 0.1 | 0.0211 | -2.39 | -3.87299 | -0.264 |
| NAA35 | ILMN_1810826 | 0.109 | 0.0237 | -2.34 | -3.97141 | -0.264 |
| CCM2 | ILMN_1652198 | 0.117 | 0.0263 | -2.3 | -4.06552 | -0.264 |
| PSRC1 | ILMN_2315964 | 0.121 | 0.0275 | -2.28 | -4.10301 | -0.264 |
| TAF1L | ILMN_1799939 | 0.153 | 0.0385 | -2.13 | -4.39395 | -0.264 |
| SRPK2 | ILMN_1657451 | 0.163 | 0.0422 | -2.09 | -4.47278 | -0.264 |
| SENP7 | ILMN_2382354 | 0.00204 | 0.000124 | -4.19 | 0.87229 | -0.265 |
| ZNF20 | ILMN_1670377 | 0.0154 | 0.00175 | -3.32 | -1.61393 | -0.265 |
| DDX17 | ILMN_1675124 | 0.0189 | 0.00229 | -3.23 | -1.8607 | -0.265 |
| GCC1 | ILMN_1682206 | 1.72E-05 | 2.33E-07 | -6.07 | 6.8772 | -0.266 |
| SIRT7 | ILMN_2077858 | 0.0038 | 0.000281 | -3.93 | 0.09727 | -0.266 |
| TADA3 | ILMN_1674866 | 0.0106 | 0.00107 | -3.49 | -1.15237 | -0.266 |
| PRUNE1 | ILMN_1728914 | 0.0186 | 0.00222 | -3.24 | -1.83507 | -0.266 |
| TSSC1 | ILMN_2129349 | 0.0238 | 0.00311 | -3.12 | -2.14293 | -0.266 |
| EFR3A | ILMN_1664776 | 0.138 | 0.033 | -2.2 | -4.26258 | -0.266 |
| INTS6L | ILMN_2207419 | 0.154 | 0.0389 | -2.13 | -4.40329 | -0.266 |
| CD93 | ILMN_1704730 | 0.155 | 0.039 | -2.12 | -4.40548 | -0.266 |
| IKBIP | ILMN_1701402 | 0.168 | 0.044 | -2.07 | -4.50942 | -0.266 |
| TFE3 | ILMN_1764826 | 0.00833 | 0.000775 | -3.6 | -0.85477 | -0.267 |
| ZNF830 | ILMN_2154603 | 0.00932 | 0.000894 | -3.55 | -0.98847 | -0.267 |
| SKI | ILMN_1710598 | 0.0121 | 0.00127 | -3.43 | -1.31591 | -0.267 |
| DPP9 | ILMN_1673069 | 0.0222 | 0.00282 | -3.16 | -2.05535 | -0.267 |
| TMEM222 | ILMN_1793632 | 0.035 | 0.00513 | -2.94 | -2.60128 | -0.267 |
| ATP2A3 | ILMN_1697827 | 0.0432 | 0.00677 | -2.84 | -2.85377 | -0.267 |
| ATF6B | ILMN_3194911 | 0.146 | 0.0356 | -2.16 | -4.32787 | -0.267 |
| TMEM186 | ILMN_2230998 | 0.182 | 0.0493 | -2.02 | -4.60446 | -0.267 |
| FBXW11 | ILMN_2394571 | 0.000168 | 4.66E-06 | -5.19 | 3.99849 | -0.268 |
| N4BP2L2 | ILMN_3234089 | 0.00187 | 0.000109 | -4.23 | 0.98992 | -0.268 |
| VOPP1 | ILMN_1757827 | 0.0284 | 0.0039 | -3.04 | -2.35127 | -0.268 |
| ELK1 | ILMN_1654289 | 0.0347 | 0.00507 | -2.94 | -2.591 | -0.268 |
| MYO1G | ILMN_1692295 | 0.0552 | 0.00947 | -2.71 | -3.15774 | -0.268 |
| MAP3K7 | ILMN_2379326 | 0.091 | 0.0186 | -2.44 | -3.75896 | -0.268 |
| FAM133CP | ILMN_3304435 | 0.122 | 0.0281 | -2.27 | -4.12115 | -0.268 |
| PIK3C2A | ILMN_3251440 | 0.127 | 0.0294 | -2.25 | -4.16085 | -0.268 |
| YAF2 | ILMN_1765606 | 0.142 | 0.0343 | -2.18 | -4.29484 | -0.268 |
| PPP1CC | ILMN_1701855 | 0.152 | 0.038 | -2.14 | -4.38254 | -0.268 |
| RNF20 | ILMN_1710758 | 0.00208 | 0.000127 | -4.19 | 0.84985 | -0.269 |
| RTN3 | ILMN_2363065 | 0.0118 | 0.00124 | -3.44 | -1.29145 | -0.269 |
| CRLF3 | ILMN_1660579 | 0.0272 | 0.00371 | -3.06 | -2.30507 | -0.269 |
| MAST3 | ILMN_1738749 | 0.0424 | 0.00659 | -2.85 | -2.82987 | -0.269 |
| ABCA7 | ILMN_1743205 | 0.0695 | 0.013 | -2.59 | -3.43965 | -0.269 |
| TRIM44 | ILMN_1796063 | 0.109 | 0.0238 | -2.34 | -3.97847 | -0.269 |
| KAT2A | ILMN_1782247 | 0.131 | 0.0307 | -2.23 | -4.19954 | -0.269 |
| FBXO21 | ILMN_1745887 | 0.144 | 0.0349 | -2.17 | -4.30933 | -0.269 |
| RPS6KC1 | ILMN_1661000 | 0.16 | 0.0409 | -2.1 | -4.44535 | -0.269 |
| ARFGEF1 | ILMN_1728471 | 3.35E-06 | 2.22E-08 | -6.74 | 9.13864 | -0.27 |
| TFIP11 | ILMN_2408102 | 0.000369 | 1.30E-05 | -4.89 | 3.01994 | -0.27 |
| PIK3CB | ILMN_1763347 | 0.00691 | 0.000603 | -3.68 | -0.62093 | -0.27 |
| ZNF700 | ILMN_1807234 | 0.0328 | 0.00471 | -2.97 | -2.52395 | -0.27 |
| TTC5 | ILMN_1706455 | 0.0413 | 0.00636 | -2.86 | -2.79804 | -0.27 |
| RNGTT | ILMN_1734198 | 0.0674 | 0.0124 | -2.6 | -3.40182 | -0.27 |
| PTPRCAP | ILMN_1672417 | 0.0695 | 0.013 | -2.59 | -3.43943 | -0.27 |
| PEX6 | ILMN_1683279 | 0.14 | 0.0337 | -2.19 | -4.27932 | -0.27 |
| TLN1 | ILMN_1696643 | 0.000938 | 4.40E-05 | -4.52 | 1.85446 | -0.271 |
| ZBTB33 | ILMN_1673138 | 0.0011 | 5.38E-05 | -4.45 | 1.66292 | -0.271 |
| TOP3A | ILMN_2072973 | 0.0037 | 0.00027 | -3.95 | 0.13363 | -0.271 |
| UNC13D | ILMN_1773380 | 0.0115 | 0.00119 | -3.46 | -1.25495 | -0.271 |
| SETD3 | ILMN_1724504 | 0.0327 | 0.00469 | -2.97 | -2.52034 | -0.271 |
| LPCAT4 | ILMN_1674759 | 0.0898 | 0.0182 | -2.45 | -3.74189 | -0.271 |
| IFT122 | ILMN_1742379 | 0.138 | 0.033 | -2.2 | -4.26232 | -0.271 |
| CARD19 | ILMN_1659189 | 0.149 | 0.0368 | -2.15 | -4.35651 | -0.271 |
| SON | ILMN_2247664 | 4.82E-05 | 9.45E-07 | -5.66 | 5.52912 | -0.272 |
| EIF2B4 | ILMN_1687430 | 0.00357 | 0.000258 | -3.96 | 0.17838 | -0.272 |
| GPATCH2L | ILMN_1699091 | 0.0154 | 0.00175 | -3.32 | -1.61439 | -0.272 |
| IPCEF1 | ILMN_1796497 | 0.0477 | 0.00773 | -2.79 | -2.97464 | -0.272 |
| EXOSC9 | ILMN_2312719 | 0.144 | 0.0349 | -2.17 | -4.30865 | -0.272 |
| LBR | ILMN_1810418 | 0.16 | 0.0409 | -2.1 | -4.44652 | -0.272 |
| DYNLL1 | ILMN_2300186 | 0.00153 | 8.30E-05 | -4.32 | 1.24982 | -0.273 |
| TMF1 | ILMN_1661142 | 0.00621 | 0.000528 | -3.73 | -0.49505 | -0.273 |
| CREB1 | ILMN_1841334 | 0.0183 | 0.00218 | -3.25 | -1.81724 | -0.273 |
| WDR1 | ILMN_1780036 | 0.0221 | 0.0028 | -3.16 | -2.04822 | -0.273 |
| HNRNPDL | ILMN_1653432 | 0.0241 | 0.00316 | -3.12 | -2.15947 | -0.273 |
| UBXN11 | ILMN_2376416 | 0.0448 | 0.00711 | -2.82 | -2.89873 | -0.273 |
| CEP295 | ILMN_3235104 | 0.178 | 0.0477 | -2.03 | -4.57728 | -0.273 |
| GTF2F2 | ILMN_1745798 | 0.0144 | 0.00159 | -3.36 | -1.52396 | -0.274 |
| HIF1AN | ILMN_1681812 | 0.0277 | 0.00379 | -3.05 | -2.32424 | -0.274 |
| GRPEL2 | ILMN_1721138 | 0.0508 | 0.00842 | -2.75 | -3.05183 | -0.274 |
| MAPK3 | ILMN_1667260 | 0.0535 | 0.00909 | -2.72 | -3.12101 | -0.274 |
| KIAA0355 | ILMN_1659845 | 0.0973 | 0.0204 | -2.4 | -3.83924 | -0.274 |
| PCGF1 | ILMN_1757956 | 0.159 | 0.0405 | -2.11 | -4.43773 | -0.274 |
| EIF1B | ILMN_1679324 | 0.00393 | 0.000292 | -3.92 | 0.06175 | -0.275 |
| KIAA1551 | ILMN_1726289 | 0.00604 | 0.00051 | -3.74 | -0.4637 | -0.275 |
| CCL5 | ILMN_1773352 | 0.00943 | 0.000908 | -3.55 | -1.00311 | -0.275 |
| RNF166 | ILMN_1695356 | 0.0748 | 0.0143 | -2.55 | -3.52719 | -0.275 |
| DDX55 | ILMN_1797425 | 0.103 | 0.022 | -2.37 | -3.90732 | -0.275 |
| PIGH | ILMN_1798395 | 0.135 | 0.0321 | -2.21 | -4.23812 | -0.275 |
| VPS4B | ILMN_1792587 | 1.35E-06 | 5.96E-09 | -7.13 | 10.40553 | -0.276 |
| CTDSP2 | ILMN_1692962 | 0.00016 | 4.31E-06 | -5.21 | 4.07354 | -0.276 |
| FXYD5 | ILMN_2309848 | 0.00297 | 0.000203 | -4.04 | 0.40394 | -0.276 |
| TNFRSF9 | ILMN_1813379 | 0.00908 | 0.000865 | -3.56 | -0.95769 | -0.276 |
| SLC43A2 | ILMN_1787127 | 0.0297 | 0.00414 | -3.02 | -2.40515 | -0.276 |
| PHF20 | ILMN_1813657 | 0.049 | 0.00799 | -2.77 | -3.00436 | -0.276 |
| HECTD3 | ILMN_1656902 | 0.0658 | 0.012 | -2.62 | -3.36878 | -0.276 |
| LMAN2 | ILMN_1776963 | 0.106 | 0.023 | -2.35 | -3.94735 | -0.276 |
| RNF5P1 | ILMN_2052863 | 0.123 | 0.0284 | -2.26 | -4.12969 | -0.276 |
| LZTR1 | ILMN_1805161 | 0.126 | 0.0291 | -2.25 | -4.15333 | -0.276 |
| STK4 | ILMN_1711383 | 0.00113 | 5.59E-05 | -4.44 | 1.62558 | -0.277 |
| APOC1 | ILMN_1789007 | 0.031 | 0.00438 | -3 | -2.45694 | -0.277 |
| ZNF700 | ILMN_2068435 | 0.0355 | 0.00521 | -2.93 | -2.61601 | -0.277 |
| SEMA4B | ILMN_1672589 | 0.0446 | 0.00708 | -2.82 | -2.89498 | -0.277 |
| WDR47 | ILMN_1741869 | 0.0475 | 0.00767 | -2.79 | -2.96772 | -0.277 |
| SS18 | ILMN_1741782 | 0.0509 | 0.00844 | -2.75 | -3.05394 | -0.277 |
| DEDD | ILMN_1710710 | 0.0513 | 0.00857 | -2.75 | -3.06727 | -0.277 |
| C16orf54 | ILMN_1751061 | 0.0695 | 0.013 | -2.58 | -3.44045 | -0.277 |
| RNF41 | ILMN_1808095 | 0.0743 | 0.0142 | -2.55 | -3.51847 | -0.277 |
| GNL3 | ILMN_2324056 | 0.123 | 0.0281 | -2.27 | -4.12332 | -0.277 |
| FAM160A2 | ILMN_1713402 | 0.154 | 0.0388 | -2.13 | -4.40131 | -0.277 |
| STAM | ILMN_1765409 | 0.184 | 0.0497 | -2.02 | -4.61173 | -0.277 |
| REV1 | ILMN_2395474 | 0.00707 | 0.000622 | -3.67 | -0.64964 | -0.278 |
| CBFA2T3 | ILMN_1657627 | 0.0134 | 0.00146 | -3.39 | -1.44618 | -0.278 |
| DTX2 | ILMN_1744129 | 0.0316 | 0.0045 | -2.99 | -2.48272 | -0.278 |
| ATP1A1 | ILMN_1731783 | 0.0794 | 0.0154 | -2.52 | -3.59478 | -0.278 |
| ZNF304 | ILMN_1656504 | 0.171 | 0.0449 | -2.06 | -4.52591 | -0.278 |
| POC5 | ILMN_2225577 | 0.172 | 0.0456 | -2.06 | -4.53808 | -0.278 |
| PPP2R2A | ILMN_1788961 | 0.00141 | 7.50E-05 | -4.35 | 1.34702 | -0.279 |
| FNBP4 | ILMN_2108938 | 0.00191 | 0.000113 | -4.22 | 0.96108 | -0.279 |
| MOB3A | ILMN_1721344 | 0.00391 | 0.00029 | -3.92 | 0.067 | -0.279 |
| PCNX3 | ILMN_1783350 | 0.0094 | 0.000904 | -3.55 | -0.99921 | -0.279 |
| VPS26A | ILMN_2344850 | 0.0139 | 0.00153 | -3.37 | -1.48895 | -0.279 |
| DHRS7 | ILMN_1807455 | 0.0194 | 0.00235 | -3.22 | -1.88565 | -0.279 |
| FKBP1A | ILMN_1683969 | 0.0258 | 0.00346 | -3.08 | -2.24176 | -0.279 |
| MTMR9 | ILMN_1652521 | 0.0285 | 0.00393 | -3.04 | -2.35789 | -0.279 |
| PPP1R3D | ILMN_1781198 | 0.0427 | 0.00668 | -2.84 | -2.84157 | -0.279 |
| KMT5A | ILMN_1651936 | 0.0456 | 0.0073 | -2.81 | -2.92275 | -0.279 |
| MAPK3 | ILMN_2402341 | 0.0589 | 0.0104 | -2.67 | -3.23835 | -0.279 |
| IGLL1 | ILMN_2393765 | 0.0654 | 0.0119 | -2.62 | -3.35961 | -0.279 |
| P4HA2 | ILMN_1795778 | 0.0902 | 0.0183 | -2.45 | -3.74638 | -0.279 |
| TM6SF1 | ILMN_1750961 | 0.106 | 0.0228 | -2.36 | -3.93981 | -0.279 |
| STK36 | ILMN_1693538 | 0.134 | 0.0316 | -2.22 | -4.22449 | -0.279 |
| SCAF8 | ILMN_1681675 | 1.72E-05 | 2.36E-07 | -6.06 | 6.86347 | -0.28 |
| DHDDS | ILMN_2405642 | 0.0552 | 0.00946 | -2.71 | -3.15674 | -0.28 |
| ATG16L1 | ILMN_1725707 | 0.0633 | 0.0114 | -2.64 | -3.32261 | -0.28 |
| TACR2 | ILMN_1753006 | 0.105 | 0.0226 | -2.36 | -3.93087 | -0.28 |
| LAMTOR3 | ILMN_1659415 | 0.000113 | 2.79E-06 | -5.34 | 4.48985 | -0.281 |
| ACTN4 | ILMN_1725534 | 0.00578 | 0.00048 | -3.76 | -0.40584 | -0.281 |
| LPAR2 | ILMN_1662741 | 0.00591 | 0.000494 | -3.75 | -0.43355 | -0.281 |
| KRI1 | ILMN_2347748 | 0.122 | 0.028 | -2.27 | -4.1172 | -0.281 |
| FAM131A | ILMN_1729217 | 0.182 | 0.049 | -2.02 | -4.60017 | -0.281 |
| PHRF1 | ILMN_3245476 | 0.000765 | 3.37E-05 | -4.6 | 2.10861 | -0.282 |
| UBE4A | ILMN_1739259 | 0.00101 | 4.83E-05 | -4.49 | 1.76396 | -0.282 |
| C15orf39 | ILMN_1793729 | 0.00334 | 0.000237 | -3.99 | 0.2564 | -0.282 |
| SFT2D1 | ILMN_1734895 | 0.00339 | 0.000242 | -3.98 | 0.23633 | -0.282 |
| TTC33 | ILMN_1807088 | 0.00723 | 0.00064 | -3.66 | -0.67583 | -0.282 |
| PSMC1 | ILMN_1736353 | 0.00754 | 0.000678 | -3.65 | -0.72987 | -0.282 |
| LILRB2 | ILMN_2312340 | 0.0106 | 0.00108 | -3.49 | -1.16132 | -0.282 |
| PRR14L | ILMN_3307887 | 0.0237 | 0.00308 | -3.12 | -2.13518 | -0.282 |
| FBP1 | ILMN_1728799 | 0.0605 | 0.0107 | -2.66 | -3.27058 | -0.282 |
| CENPB | ILMN_1664028 | 0.0632 | 0.0114 | -2.64 | -3.32049 | -0.282 |
| PLAUR | ILMN_1691508 | 0.0934 | 0.0193 | -2.43 | -3.79113 | -0.282 |
| CCNT2 | ILMN_1722522 | 0.00463 | 0.000361 | -3.85 | -0.13813 | -0.283 |
| FOXN3 | ILMN_2106902 | 0.0292 | 0.00404 | -3.03 | -2.38308 | -0.283 |
| ATXN1 | ILMN_1800951 | 0.0449 | 0.00715 | -2.82 | -2.90344 | -0.283 |
| BAIAP2 | ILMN_1705922 | 0.0532 | 0.00902 | -2.73 | -3.11373 | -0.283 |
| CSNK1E | ILMN_2415235 | 0.0678 | 0.0126 | -2.6 | -3.41088 | -0.283 |
| DDX52 | ILMN_2366790 | 0.0724 | 0.0137 | -2.56 | -3.48867 | -0.283 |
| KIF22 | ILMN_3234884 | 0.114 | 0.0253 | -2.31 | -4.03109 | -0.283 |
| MSRB3 | ILMN_1676088 | 0.147 | 0.0362 | -2.16 | -4.342 | -0.283 |
| RNF146 | ILMN_1685679 | 0.152 | 0.038 | -2.14 | -4.38349 | -0.283 |
| FBXO8 | ILMN_1672843 | 0.00246 | 0.000159 | -4.11 | 0.63231 | -0.284 |
| SRSF9 | ILMN_1760683 | 0.00428 | 0.000328 | -3.88 | -0.04741 | -0.284 |
| SPN | ILMN_1801040 | 0.0175 | 0.00207 | -3.27 | -1.76935 | -0.284 |
| ATG7 | ILMN_1790978 | 0.0635 | 0.0114 | -2.64 | -3.3254 | -0.284 |
| RAB11B | ILMN_1731135 | 0.0953 | 0.0198 | -2.41 | -3.81434 | -0.284 |
| C6orf136 | ILMN_1813236 | 0.125 | 0.0288 | -2.26 | -4.14309 | -0.284 |
| CPSF3L | ILMN_1762316 | 0.136 | 0.0324 | -2.21 | -4.24484 | -0.284 |
| CCDC112 | ILMN_1761101 | 0.159 | 0.0406 | -2.11 | -4.44014 | -0.284 |
| DSTYK | ILMN_3257566 | 0.164 | 0.0424 | -2.09 | -4.47715 | -0.284 |
| ZNF384 | ILMN_1764891 | 0.000579 | 2.30E-05 | -4.71 | 2.47189 | -0.285 |
| PISD | ILMN_1793934 | 0.00112 | 5.55E-05 | -4.44 | 1.63266 | -0.285 |
| OXR1 | ILMN_1737462 | 0.143 | 0.0345 | -2.18 | -4.29958 | -0.285 |
| POLDIP3 | ILMN_1688000 | 0.000497 | 1.90E-05 | -4.77 | 2.65237 | -0.286 |
| HARS | ILMN_1763523 | 0.00299 | 0.000205 | -4.03 | 0.39494 | -0.286 |
| NKIRAS2 | ILMN_1653404 | 0.0105 | 0.00106 | -3.49 | -1.14943 | -0.286 |
| NCF1C | ILMN_2112988 | 0.0122 | 0.0013 | -3.43 | -1.33379 | -0.286 |
| PROCA1 | ILMN_1663179 | 0.0151 | 0.00172 | -3.33 | -1.59537 | -0.286 |
| RASGRP4 | ILMN_1714650 | 0.0355 | 0.0052 | -2.93 | -2.61499 | -0.286 |
| ARID4B | ILMN_2362982 | 0.0746 | 0.0142 | -2.55 | -3.52338 | -0.286 |
| HAUS8 | ILMN_2365528 | 0.0956 | 0.0199 | -2.41 | -3.81901 | -0.286 |
| LOC286437 | ILMN_1817377 | 0.12 | 0.0272 | -2.28 | -4.09402 | -0.286 |
| ST3GAL2 | ILMN_1714165 | 0.122 | 0.0279 | -2.27 | -4.11645 | -0.286 |
| RFC5 | ILMN_1659364 | 0.135 | 0.0319 | -2.21 | -4.23154 | -0.286 |
| PRSS53 | ILMN_2133187 | 0.149 | 0.0367 | -2.15 | -4.35197 | -0.286 |
| SS18L2 | ILMN_1796407 | 0.153 | 0.0385 | -2.13 | -4.39449 | -0.286 |
| AATK | ILMN_1687609 | 0.021 | 0.00262 | -3.18 | -1.98467 | -0.287 |
| DYM | ILMN_1680130 | 0.023 | 0.00296 | -3.14 | -2.09944 | -0.287 |
| NSF | ILMN_1680687 | 0.0867 | 0.0173 | -2.47 | -3.698 | -0.287 |
| ECH1 | ILMN_1653115 | 0.0941 | 0.0195 | -2.42 | -3.8015 | -0.287 |
| PIK3CD | ILMN_1766275 | 0.0979 | 0.0205 | -2.4 | -3.8473 | -0.287 |
| VPS11 | ILMN_1717781 | 0.104 | 0.0222 | -2.37 | -3.916 | -0.287 |
| STK40 | ILMN_1679311 | 0.11 | 0.0241 | -2.33 | -3.98681 | -0.287 |
| SRSF1 | ILMN_1795341 | 0.00632 | 0.000539 | -3.72 | -0.51604 | -0.288 |
| CTDSP1 | ILMN_1681678 | 0.0233 | 0.00302 | -3.13 | -2.11699 | -0.288 |
| RMI1 | ILMN_1754051 | 0.0418 | 0.00646 | -2.85 | -2.81152 | -0.288 |
| DIAPH1 | ILMN_1661554 | 0.0453 | 0.00722 | -2.81 | -2.91219 | -0.288 |
| RSRC2 | ILMN_2358278 | 0.0745 | 0.0142 | -2.55 | -3.52131 | -0.288 |
| VRK3 | ILMN_2348403 | 0.0758 | 0.0146 | -2.54 | -3.54361 | -0.288 |
| TSC2 | ILMN_1714216 | 0.145 | 0.0355 | -2.17 | -4.32551 | -0.288 |
| ATG4C | ILMN_1700923 | 0.154 | 0.0389 | -2.13 | -4.40386 | -0.288 |
| CDC16 | ILMN_1727389 | 0.17 | 0.0446 | -2.06 | -4.5202 | -0.288 |
| MAPKAPK5 | ILMN_2322935 | 0.173 | 0.0457 | -2.05 | -4.54043 | -0.288 |
| RAB8B | ILMN_2173004 | 4.61E-05 | 8.73E-07 | -5.68 | 5.60557 | -0.289 |
| PTPN11 | ILMN_1778236 | 6.49E-05 | 1.38E-06 | -5.55 | 5.16697 | -0.289 |
| USF1 | ILMN_1777982 | 0.00141 | 7.45E-05 | -4.35 | 1.35281 | -0.289 |
| CD82 | ILMN_2392274 | 0.00529 | 0.000429 | -3.8 | -0.30209 | -0.289 |
| MMS19 | ILMN_3237324 | 0.0222 | 0.00283 | -3.16 | -2.05547 | -0.289 |
| SPECC1 | ILMN_3255061 | 0.0227 | 0.00292 | -3.14 | -2.08436 | -0.289 |
| SYT11 | ILMN_1717934 | 0.0397 | 0.00605 | -2.88 | -2.7519 | -0.289 |
| PCCB | ILMN_1761010 | 0.0509 | 0.00845 | -2.75 | -3.05519 | -0.289 |
| AP3M1 | ILMN_2362122 | 0.0726 | 0.0138 | -2.56 | -3.49248 | -0.289 |
| RBM17 | ILMN_1781906 | 0.145 | 0.0354 | -2.17 | -4.32088 | -0.289 |
| SYNJ1 | ILMN_1701991 | 2.82E-05 | 4.62E-07 | -5.87 | 6.21817 | -0.29 |
| MYH9 | ILMN_2087702 | 0.00035 | 1.21E-05 | -4.91 | 3.08475 | -0.29 |
| CNN2 | ILMN_1770290 | 0.00281 | 0.000188 | -4.06 | 0.47695 | -0.29 |
| ATP2A3 | ILMN_2310909 | 0.031 | 0.00437 | -3 | -2.45542 | -0.29 |
| DHRS4 | ILMN_2185884 | 0.0844 | 0.0168 | -2.48 | -3.66951 | -0.29 |
| ZXDC | ILMN_1743643 | 0.0845 | 0.0168 | -2.48 | -3.67072 | -0.29 |
| DOCK4 | ILMN_1801044 | 0.142 | 0.0344 | -2.18 | -4.29768 | -0.29 |
| AFTPH | ILMN_2402766 | 2.84E-05 | 4.67E-07 | -5.86 | 6.20698 | -0.291 |
| SMG5 | ILMN_2126239 | 0.00346 | 0.000248 | -3.97 | 0.21396 | -0.291 |
| BSG | ILMN_1778374 | 0.0211 | 0.00263 | -3.18 | -1.99121 | -0.291 |
| IMPDH2 | ILMN_1705737 | 0.0568 | 0.00986 | -2.69 | -3.19403 | -0.291 |
| SIRT1 | ILMN_1739083 | 0.0592 | 0.0104 | -2.67 | -3.24549 | -0.291 |
| ZFC3H1 | ILMN_3244096 | 0.0682 | 0.0127 | -2.59 | -3.41854 | -0.291 |
| CREB1 | ILMN_2382758 | 0.161 | 0.0413 | -2.1 | -4.45519 | -0.291 |
| DESI2 | ILMN_1660840 | 0.00196 | 0.000117 | -4.21 | 0.92258 | -0.292 |
| ARHGEF1 | ILMN_2405129 | 0.00213 | 0.000132 | -4.17 | 0.81392 | -0.292 |
| PTPRE | ILMN_1734543 | 0.00396 | 0.000295 | -3.92 | 0.05183 | -0.292 |
| SEPT2 | ILMN_1748546 | 0.0184 | 0.0022 | -3.24 | -1.82524 | -0.292 |
| ATP8B4 | ILMN_1783956 | 0.0215 | 0.00271 | -3.17 | -2.01602 | -0.292 |
| KLHDC3 | ILMN_1730940 | 0.135 | 0.032 | -2.21 | -4.23424 | -0.292 |
| BTN2A1 | ILMN_2364852 | 0.00019 | 5.46E-06 | -5.14 | 3.84575 | -0.293 |
| CRTC2 | ILMN_1657771 | 0.00105 | 5.06E-05 | -4.47 | 1.72009 | -0.293 |
| PRKAR1A | ILMN_1738632 | 0.00224 | 0.00014 | -4.15 | 0.75237 | -0.293 |
| TMEM154 | ILMN_1683494 | 0.0056 | 0.00046 | -3.77 | -0.36726 | -0.293 |
| PRPF40A | ILMN_1659854 | 0.0061 | 0.000516 | -3.74 | -0.47397 | -0.293 |
| ZBTB42 | ILMN_3239445 | 0.0189 | 0.00227 | -3.23 | -1.85338 | -0.293 |
| CAPN1 | ILMN_1705261 | 0.0562 | 0.00972 | -2.7 | -3.18126 | -0.293 |
| MRPS7 | ILMN_1813389 | 0.0617 | 0.011 | -2.65 | -3.29479 | -0.293 |
| TADA3 | ILMN_1792176 | 0.0856 | 0.0171 | -2.47 | -3.68483 | -0.293 |
| DNAL4 | ILMN_1801845 | 0.15 | 0.0373 | -2.14 | -4.36804 | -0.293 |
| C16orf58 | ILMN_1685289 | 0.161 | 0.0414 | -2.1 | -4.4574 | -0.293 |
| ANAPC4 | ILMN_1802973 | 0.18 | 0.0482 | -2.03 | -4.58681 | -0.293 |
| ACAP2 | ILMN_3236270 | 2.49E-05 | 3.92E-07 | -5.91 | 6.37622 | -0.294 |
| UBE3C | ILMN_2181363 | 0.000232 | 7.17E-06 | -5.06 | 3.58483 | -0.294 |
| PCMT1 | ILMN_1671621 | 0.000542 | 2.10E-05 | -4.74 | 2.5602 | -0.294 |
| ARID4B | ILMN_2269564 | 0.0174 | 0.00205 | -3.27 | -1.76063 | -0.294 |
| PCNX4 | ILMN_1680781 | 0.0454 | 0.00726 | -2.81 | -2.91749 | -0.294 |
| DHX15 | ILMN_1754839 | 0.0015 | 8.03E-05 | -4.33 | 1.28152 | -0.295 |
| CRLF3 | ILMN_2155228 | 0.00617 | 0.000524 | -3.73 | -0.48958 | -0.295 |
| ZNRD1 | ILMN_1692486 | 0.00767 | 0.000697 | -3.64 | -0.75545 | -0.295 |
| RRBP1 | ILMN_2360784 | 0.0145 | 0.00161 | -3.35 | -1.53607 | -0.295 |
| PIP4K2C | ILMN_1787308 | 0.0194 | 0.00236 | -3.22 | -1.88964 | -0.295 |
| EIF3A | ILMN_1708164 | 0.0212 | 0.00265 | -3.18 | -1.99669 | -0.295 |
| SEC13 | ILMN_3223181 | 0.0381 | 0.00572 | -2.9 | -2.70192 | -0.295 |
| MEPCE | ILMN_2180827 | 0.0445 | 0.00706 | -2.82 | -2.89171 | -0.295 |
| CPSF2 | ILMN_1673185 | 0.064 | 0.0115 | -2.63 | -3.33327 | -0.295 |
| KIAA1191 | ILMN_2376133 | 0.0992 | 0.0209 | -2.39 | -3.86205 | -0.295 |
| ANKRD54 | ILMN_1766309 | 0.106 | 0.0229 | -2.35 | -3.94144 | -0.295 |
| SP100 | ILMN_2390586 | 0.11 | 0.024 | -2.33 | -3.98317 | -0.295 |
| MIOS | ILMN_1680644 | 0.118 | 0.0266 | -2.29 | -4.0752 | -0.295 |
| GLE1 | ILMN_1754912 | 0.000107 | 2.64E-06 | -5.36 | 4.54405 | -0.296 |
| CRK | ILMN_1803302 | 0.000798 | 3.55E-05 | -4.58 | 2.05909 | -0.296 |
| EIF4A1 | ILMN_1722900 | 0.00281 | 0.000188 | -4.06 | 0.4745 | -0.296 |
| IVNS1ABP | ILMN_1717877 | 0.0116 | 0.00121 | -3.45 | -1.26957 | -0.296 |
| RSRP1 | ILMN_1749915 | 0.0122 | 0.0013 | -3.43 | -1.3349 | -0.296 |
| TRAPPC2L | ILMN_1859908 | 0.0229 | 0.00296 | -3.14 | -2.09852 | -0.296 |
| TBC1D22A | ILMN_2096743 | 0.0975 | 0.0204 | -2.4 | -3.842 | -0.296 |
| GLTP | ILMN_1764380 | 0.0167 | 0.00195 | -3.29 | -1.71209 | -0.297 |
| ZNF592 | ILMN_1671104 | 0.0309 | 0.00434 | -3 | -2.44999 | -0.297 |
| KLF2 | ILMN_1735930 | 0.0425 | 0.00662 | -2.84 | -2.83403 | -0.297 |
| SLC25A20 | ILMN_1667429 | 0.111 | 0.0245 | -2.33 | -4.00096 | -0.297 |
| SAP18 | ILMN_1752793 | 0.134 | 0.0316 | -2.22 | -4.22394 | -0.297 |
| EIF4G2 | ILMN_1761519 | 1.55E-05 | 1.94E-07 | -6.12 | 7.0538 | -0.298 |
| SIK3 | ILMN_1732343 | 0.00129 | 6.72E-05 | -4.38 | 1.45118 | -0.298 |
| SIRPD | ILMN_1769886 | 0.00924 | 0.000884 | -3.56 | -0.97773 | -0.298 |
| POLM | ILMN_1716973 | 0.0264 | 0.00356 | -3.07 | -2.26915 | -0.298 |
| TBL1XR1 | ILMN_1798657 | 0.0529 | 0.00894 | -2.73 | -3.10601 | -0.298 |
| ATP1A1 | ILMN_1775566 | 0.0673 | 0.0124 | -2.6 | -3.39971 | -0.298 |
| SELO | ILMN_1769787 | 0.118 | 0.0265 | -2.29 | -4.07098 | -0.298 |
| KMT2C | ILMN_1725300 | 3.45E-05 | 5.92E-07 | -5.79 | 5.97955 | -0.299 |
| UFC1 | ILMN_2110281 | 0.00202 | 0.000123 | -4.2 | 0.87984 | -0.299 |
| TMEM127 | ILMN_1721563 | 0.00814 | 0.000752 | -3.61 | -0.82722 | -0.299 |
| SLCO3A1 | ILMN_1654735 | 0.00963 | 0.000935 | -3.54 | -1.03045 | -0.299 |
| PLP2 | ILMN_1738767 | 0.0124 | 0.00132 | -3.42 | -1.35466 | -0.299 |
| GTF3C1 | ILMN_1789839 | 0.0159 | 0.00183 | -3.31 | -1.65401 | -0.299 |
| ANKIB1 | ILMN_3241218 | 0.0516 | 0.00865 | -2.74 | -3.07569 | -0.299 |
| ADK | ILMN_2358626 | 0.0985 | 0.0207 | -2.4 | -3.85373 | -0.299 |
| C11orf68 | ILMN_1757847 | 0.00668 | 0.000578 | -3.7 | -0.5814 | -0.3 |
| ICAM3 | ILMN_2212763 | 0.0244 | 0.00322 | -3.11 | -2.1762 | -0.3 |
| SMAD4 | ILMN_1741477 | 0.000192 | 5.54E-06 | -5.14 | 3.83177 | -0.301 |
| EBLN2 | ILMN_2186877 | 0.0015 | 8.08E-05 | -4.33 | 1.27585 | -0.301 |
| TAOK2 | ILMN_1701487 | 0.00316 | 0.000221 | -4.01 | 0.32277 | -0.301 |
| ATP2A2 | ILMN_1687375 | 0.00729 | 0.000647 | -3.66 | -0.68611 | -0.301 |
| MAD2L1BP | ILMN_1694711 | 0.00908 | 0.000865 | -3.56 | -0.95743 | -0.301 |
| DLEU7 | ILMN_1782881 | 0.0653 | 0.0118 | -2.62 | -3.35823 | -0.301 |
| TOR2A | ILMN_2177832 | 0.0968 | 0.0202 | -2.41 | -3.83327 | -0.301 |
| TSTA3 | ILMN_1697777 | 0.102 | 0.0219 | -2.37 | -3.90324 | -0.301 |
| GTF2H1 | ILMN_2157957 | 0.125 | 0.0289 | -2.26 | -4.14545 | -0.301 |
| PMPCA | ILMN_1764239 | 0.143 | 0.0346 | -2.18 | -4.3027 | -0.301 |
| MCEMP1 | ILMN_1762713 | 0.151 | 0.0376 | -2.14 | -4.37471 | -0.301 |
| PKM | ILMN_1672650 | 0.157 | 0.0399 | -2.11 | -4.42513 | -0.301 |
| B3GALT4 | ILMN_1690682 | 0.17 | 0.0446 | -2.07 | -4.5198 | -0.301 |
| TERF2 | ILMN_1768488 | 0.00086 | 3.89E-05 | -4.55 | 1.96957 | -0.302 |
| SH3BGRL3 | ILMN_1737163 | 0.00714 | 0.000631 | -3.67 | -0.66303 | -0.302 |
| SLCO3A1 | ILMN_1678928 | 0.0142 | 0.00157 | -3.36 | -1.51073 | -0.302 |
| PHF23 | ILMN_1746135 | 0.015 | 0.0017 | -3.33 | -1.58575 | -0.302 |
| ADM | ILMN_1708934 | 0.0412 | 0.00635 | -2.86 | -2.79665 | -0.302 |
| MAPKAP1 | ILMN_1691526 | 0.0652 | 0.0118 | -2.62 | -3.35586 | -0.302 |
| AP2A1 | ILMN_1815306 | 0.0938 | 0.0194 | -2.42 | -3.79628 | -0.302 |
| AGL | ILMN_2371825 | 0.171 | 0.0452 | -2.06 | -4.53056 | -0.302 |
| USO1 | ILMN_1692121 | 0.00087 | 3.95E-05 | -4.55 | 1.95529 | -0.303 |
| LSP1 | ILMN_1699836 | 0.00222 | 0.000138 | -4.16 | 0.76577 | -0.303 |
| ACLY | ILMN_2371379 | 0.00867 | 0.000814 | -3.58 | -0.90134 | -0.303 |
| PRKCI | ILMN_1725188 | 0.0114 | 0.00118 | -3.46 | -1.25092 | -0.303 |
| CCNY | ILMN_2261784 | 0.0169 | 0.00197 | -3.28 | -1.72343 | -0.303 |
| TCF20 | ILMN_1666173 | 0.0334 | 0.00482 | -2.96 | -2.54557 | -0.303 |
| MED22 | ILMN_1792860 | 0.0491 | 0.00803 | -2.77 | -3.00872 | -0.303 |
| PCNX4 | ILMN_2111918 | 0.0965 | 0.0201 | -2.41 | -3.82921 | -0.303 |
| SFXN5 | ILMN_1767337 | 0.0132 | 0.00143 | -3.39 | -1.42423 | -0.304 |
| APPL2 | ILMN_1765076 | 0.0394 | 0.00601 | -2.88 | -2.74601 | -0.304 |
| VASP | ILMN_1812001 | 0.0712 | 0.0134 | -2.57 | -3.46992 | -0.304 |
| TP53BP1 | ILMN_1664440 | 0.0714 | 0.0135 | -2.57 | -3.47321 | -0.304 |
| CDA | ILMN_1714592 | 0.165 | 0.043 | -2.08 | -4.48917 | -0.304 |
| SEPT2 | ILMN_2365711 | 0.017 | 0.00198 | -3.28 | -1.72837 | -0.305 |
| POLR1D | ILMN_1742427 | 0.0193 | 0.00235 | -3.22 | -1.88379 | -0.305 |
| GRN | ILMN_1811702 | 0.0884 | 0.0178 | -2.46 | -3.72273 | -0.305 |
| MAP3K2 | ILMN_1696265 | 0.0926 | 0.019 | -2.43 | -3.78009 | -0.305 |
| CYFIP2 | ILMN_1677200 | 0.13 | 0.0303 | -2.24 | -4.18772 | -0.305 |
| NOL11 | ILMN_1688971 | 0.144 | 0.035 | -2.17 | -4.31268 | -0.305 |
| RFWD2 | ILMN_1661002 | 0.00422 | 0.000321 | -3.89 | -0.02773 | -0.306 |
| PEX13 | ILMN_1683916 | 0.0237 | 0.0031 | -3.12 | -2.13958 | -0.306 |
| PPP1R10 | ILMN_1659058 | 0.0383 | 0.00577 | -2.9 | -2.70904 | -0.306 |
| CNOT4 | ILMN_1794324 | 0.0477 | 0.00773 | -2.79 | -2.97405 | -0.306 |
| RC3H2 | ILMN_1710738 | 0.058 | 0.0101 | -2.68 | -3.21882 | -0.306 |
| GBE1 | ILMN_1789702 | 0.0753 | 0.0144 | -2.54 | -3.53512 | -0.306 |
| ANKRD27 | ILMN_1794063 | 0.0785 | 0.0152 | -2.52 | -3.58284 | -0.306 |
| EXOSC9 | ILMN_1721713 | 0.0936 | 0.0193 | -2.42 | -3.79384 | -0.306 |
| GLMN | ILMN_1739397 | 0.112 | 0.0248 | -2.32 | -4.01161 | -0.306 |
| DPF2 | ILMN_1734317 | 0.00329 | 0.000233 | -3.99 | 0.2736 | -0.307 |
| DSTYK | ILMN_1779600 | 0.0149 | 0.00168 | -3.34 | -1.57431 | -0.307 |
| PRKCB | ILMN_1806908 | 0.0218 | 0.00275 | -3.17 | -2.02999 | -0.307 |
| ACO2 | ILMN_1654861 | 0.0491 | 0.00803 | -2.77 | -3.00834 | -0.307 |
| DYX1C1 | ILMN_1812523 | 0.0656 | 0.0119 | -2.62 | -3.36299 | -0.307 |
| RNMT | ILMN_1769637 | 0.113 | 0.0251 | -2.32 | -4.02158 | -0.307 |
| CHST7 | ILMN_1732831 | 0.119 | 0.027 | -2.28 | -4.088 | -0.307 |
| MIB2 | ILMN_2282077 | 0.138 | 0.0331 | -2.2 | -4.2642 | -0.307 |
| DHX15 | ILMN_2168449 | 0.000242 | 7.55E-06 | -5.05 | 3.53573 | -0.308 |
| GSK3B | ILMN_1779376 | 0.000372 | 1.31E-05 | -4.88 | 3.00901 | -0.308 |
| CTDSP1 | ILMN_1728163 | 0.0011 | 5.38E-05 | -4.45 | 1.66231 | -0.308 |
| YY1 | ILMN_1770892 | 0.00854 | 0.000799 | -3.59 | -0.8835 | -0.308 |
| ATP5SL | ILMN_1809027 | 0.0247 | 0.00328 | -3.1 | -2.19199 | -0.308 |
| PDE12 | ILMN_1660305 | 0.089 | 0.018 | -2.45 | -3.73228 | -0.308 |
| LGALS9C | ILMN_1766184 | 0.169 | 0.0443 | -2.07 | -4.51437 | -0.308 |
| SDF2 | ILMN_1713978 | 0.000233 | 7.21E-06 | -5.06 | 3.58067 | -0.309 |
| ZNF160 | ILMN_2312149 | 0.068 | 0.0126 | -2.6 | -3.41584 | -0.309 |
| CHD8 | ILMN_1806122 | 0.00581 | 0.000485 | -3.76 | -0.41597 | -0.31 |
| ODF2 | ILMN_1730698 | 0.0123 | 0.0013 | -3.43 | -1.33716 | -0.31 |
| NUDT3 | ILMN_1724907 | 0.0293 | 0.00406 | -3.03 | -2.38766 | -0.31 |
| YWHAH | ILMN_1728512 | 0.0306 | 0.00429 | -3.01 | -2.43783 | -0.31 |
| USF1 | ILMN_2353240 | 0.0876 | 0.0177 | -2.46 | -3.71371 | -0.31 |
| ATP6V0A2 | ILMN_1700380 | 0.0927 | 0.0191 | -2.43 | -3.78153 | -0.31 |
| IFI30 | ILMN_1807277 | 0.114 | 0.0251 | -2.31 | -4.02481 | -0.31 |
| NSUN2 | ILMN_1680129 | 0.001 | 4.79E-05 | -4.49 | 1.77356 | -0.311 |
| ITM2A | ILMN_2076600 | 0.00112 | 5.54E-05 | -4.44 | 1.63349 | -0.311 |
| SURF4 | ILMN_1690761 | 0.00203 | 0.000123 | -4.2 | 0.87551 | -0.311 |
| PROSC | ILMN_1748908 | 0.00845 | 0.00079 | -3.59 | -0.87315 | -0.311 |
| FOXJ2 | ILMN_1731648 | 0.0609 | 0.0108 | -2.66 | -3.27889 | -0.311 |
| BHLHE40 | ILMN_1768534 | 0.000108 | 2.66E-06 | -5.36 | 4.53715 | -0.312 |
| TMEM41B | ILMN_1678004 | 0.00199 | 0.00012 | -4.2 | 0.90143 | -0.312 |
| DYNC1LI2 | ILMN_1783448 | 0.00234 | 0.000149 | -4.13 | 0.69324 | -0.312 |
| CES2 | ILMN_1696675 | 0.0043 | 0.000329 | -3.88 | -0.05148 | -0.312 |
| MAN2B2 | ILMN_1768510 | 0.00576 | 0.000478 | -3.76 | -0.40199 | -0.312 |
| NDUFAF3 | ILMN_2354515 | 0.0122 | 0.00129 | -3.43 | -1.32878 | -0.312 |
| FAM60A | ILMN_3272603 | 0.0203 | 0.0025 | -3.2 | -1.94318 | -0.312 |
| PGGT1B | ILMN_2156786 | 0.0506 | 0.00836 | -2.76 | -3.04512 | -0.312 |
| MAPRE3 | ILMN_1734290 | 0.0814 | 0.016 | -2.5 | -3.62587 | -0.312 |
| NAAA | ILMN_1668605 | 0.164 | 0.0426 | -2.09 | -4.48099 | -0.312 |
| SPOP | ILMN_2397024 | 2.50E-06 | 1.52E-08 | -6.86 | 9.50611 | -0.313 |
| RAB4B | ILMN_1803136 | 0.000319 | 1.08E-05 | -4.94 | 3.19772 | -0.313 |
| CDC5L | ILMN_1652907 | 0.000699 | 2.98E-05 | -4.63 | 2.22345 | -0.313 |
| TAF2 | ILMN_1694888 | 0.0028 | 0.000187 | -4.06 | 0.48287 | -0.313 |
| ZNF721 | ILMN_1805271 | 0.00338 | 0.000241 | -3.98 | 0.24037 | -0.313 |
| COX19 | ILMN_1656656 | 0.00422 | 0.00032 | -3.89 | -0.02546 | -0.313 |
| GALNS | ILMN_1737949 | 0.00443 | 0.000342 | -3.87 | -0.08772 | -0.313 |
| SPG21 | ILMN_1657423 | 0.0362 | 0.00536 | -2.92 | -2.64153 | -0.313 |
| UNC119B | ILMN_3245351 | 0.0413 | 0.00637 | -2.86 | -2.79863 | -0.313 |
| IL18BP | ILMN_1653575 | 0.0434 | 0.0068 | -2.83 | -2.85835 | -0.313 |
| HMBOX1 | ILMN_1843949 | 0.0805 | 0.0158 | -2.51 | -3.61251 | -0.313 |
| NUDT21 | ILMN_1798886 | 0.115 | 0.0257 | -2.31 | -4.04352 | -0.313 |
| PHF20L1 | ILMN_1732985 | 0.000341 | 1.17E-05 | -4.92 | 3.11955 | -0.314 |
| RALA | ILMN_1755364 | 0.00973 | 0.000952 | -3.53 | -1.04726 | -0.314 |
| SIRPA | ILMN_2372974 | 0.0155 | 0.00177 | -3.32 | -1.6225 | -0.314 |
| SLX4 | ILMN_1732885 | 0.0281 | 0.00384 | -3.05 | -2.33775 | -0.314 |
| PLEKHF1 | ILMN_1708041 | 0.102 | 0.0219 | -2.37 | -3.90349 | -0.314 |
| CES2 | ILMN_2362681 | 0.113 | 0.0249 | -2.32 | -4.01798 | -0.314 |
| DHX16 | ILMN_1716922 | 0.00126 | 6.49E-05 | -4.4 | 1.48428 | -0.315 |
| CEACAM21 | ILMN_1745949 | 0.0161 | 0.00186 | -3.3 | -1.66945 | -0.315 |
| CCM2 | ILMN_1784352 | 0.0388 | 0.00586 | -2.89 | -2.72309 | -0.315 |
| PLAUR | ILMN_2374340 | 0.0394 | 0.00599 | -2.88 | -2.74301 | -0.315 |
| ABLIM1 | ILMN_2396672 | 0.0699 | 0.0131 | -2.58 | -3.44666 | -0.315 |
| UNC119 | ILMN_1664698 | 0.0704 | 0.0132 | -2.58 | -3.45561 | -0.315 |
| ATG4A | ILMN_2313782 | 0.135 | 0.0322 | -2.21 | -4.23892 | -0.315 |
| CASC3 | ILMN_1665004 | 0.00182 | 0.000105 | -4.24 | 1.02456 | -0.316 |
| PTAFR | ILMN_1746836 | 0.013 | 0.0014 | -3.4 | -1.40634 | -0.316 |
| WLS | ILMN_1660549 | 0.0424 | 0.0066 | -2.85 | -2.83142 | -0.316 |
| MAN2A1 | ILMN_2147435 | 0.0917 | 0.0187 | -2.44 | -3.76654 | -0.316 |
| NIPSNAP3A | ILMN_1700159 | 0.102 | 0.0218 | -2.38 | -3.89877 | -0.316 |
| MANBAL | ILMN_1673944 | 0.000115 | 2.86E-06 | -5.33 | 4.46582 | -0.317 |
| FAF2 | ILMN_1670472 | 0.000614 | 2.52E-05 | -4.68 | 2.38551 | -0.317 |
| TRABD | ILMN_1755737 | 0.00717 | 0.000634 | -3.67 | -0.6675 | -0.317 |
| MCM3AP | ILMN_1784766 | 0.0112 | 0.00115 | -3.47 | -1.22661 | -0.317 |
| SLFN11 | ILMN_1752520 | 0.0148 | 0.00166 | -3.34 | -1.56324 | -0.317 |
| SMARCA5 | ILMN_2223130 | 0.0158 | 0.00181 | -3.31 | -1.64304 | -0.317 |
| LOC146880 | ILMN_1678300 | 0.0495 | 0.00813 | -2.77 | -3.01983 | -0.317 |
| SPAST | ILMN_1791547 | 0.084 | 0.0167 | -2.48 | -3.66283 | -0.317 |
| PKM | ILMN_1775327 | 0.0981 | 0.0206 | -2.4 | -3.84935 | -0.317 |
| HYAL2 | ILMN_1668283 | 0.116 | 0.0259 | -2.3 | -4.0508 | -0.317 |
| PLD6 | ILMN_3240586 | 0.166 | 0.0433 | -2.08 | -4.49441 | -0.317 |
| FBXO11 | ILMN_2285112 | 7.32E-06 | 7.27E-08 | -6.4 | 7.99724 | -0.318 |
| CCL5 | ILMN_2098126 | 0.00371 | 0.000272 | -3.94 | 0.12813 | -0.318 |
| GPSM2 | ILMN_2139816 | 0.0399 | 0.00609 | -2.88 | -2.75847 | -0.318 |
| PLAUR | ILMN_2408543 | 0.0673 | 0.0124 | -2.6 | -3.39996 | -0.318 |
| BRWD1 | ILMN_1801866 | 0.0957 | 0.0199 | -2.41 | -3.82086 | -0.318 |
| RAI1 | ILMN_2060770 | 0.1 | 0.0212 | -2.39 | -3.87366 | -0.318 |
| YWHAE | ILMN_1807535 | 0.181 | 0.0487 | -2.03 | -4.59431 | -0.318 |
| TDG | ILMN_1777096 | 2.22E-05 | 3.25E-07 | -5.97 | 6.55657 | -0.319 |
| NAB1 | ILMN_1774617 | 0.00803 | 0.000739 | -3.62 | -0.81051 | -0.319 |
| HIVEP1 | ILMN_1735548 | 0.0488 | 0.00795 | -2.78 | -2.99978 | -0.319 |
| TIMP1 | ILMN_1711566 | 0.122 | 0.028 | -2.27 | -4.11732 | -0.319 |
| RPS6KA1 | ILMN_1715173 | 0.000161 | 4.37E-06 | -5.21 | 4.06011 | -0.32 |
| EXOSC3 | ILMN_1708936 | 0.00078 | 3.46E-05 | -4.59 | 2.08326 | -0.32 |
| GRIPAP1 | ILMN_1761176 | 0.00166 | 9.32E-05 | -4.28 | 1.14075 | -0.32 |
| FAM46B | ILMN_1808011 | 0.00198 | 0.000119 | -4.21 | 0.90934 | -0.32 |
| TCEA2 | ILMN_1665526 | 0.0127 | 0.00137 | -3.41 | -1.38279 | -0.32 |
| SLTM | ILMN_1742224 | 0.0132 | 0.00143 | -3.39 | -1.42425 | -0.32 |
| ERGIC3 | ILMN_2359456 | 0.0185 | 0.00222 | -3.24 | -1.83274 | -0.32 |
| FKBP15 | ILMN_1804863 | 0.0302 | 0.00423 | -3.01 | -2.42609 | -0.32 |
| S100PBP | ILMN_1700685 | 0.0524 | 0.00882 | -2.74 | -3.09359 | -0.32 |
| SMIM7 | ILMN_1694759 | 0.071 | 0.0134 | -2.57 | -3.46633 | -0.32 |
| NT5C | ILMN_1806432 | 0.0992 | 0.0209 | -2.39 | -3.86218 | -0.32 |
| CD79B | ILMN_2366212 | 0.108 | 0.0234 | -2.35 | -3.9615 | -0.32 |
| PKN1 | ILMN_2367707 | 0.147 | 0.0363 | -2.16 | -4.34305 | -0.32 |
| PDXDC1 | ILMN_3243705 | 0.00387 | 0.000286 | -3.93 | 0.07936 | -0.321 |
| CORO2A | ILMN_1813746 | 0.00504 | 0.000404 | -3.82 | -0.24493 | -0.321 |
| NEMF | ILMN_1772489 | 0.0119 | 0.00125 | -3.44 | -1.2976 | -0.321 |
| NOMO1 | ILMN_1678730 | 0.0313 | 0.00442 | -2.99 | -2.4669 | -0.321 |
| PANK4 | ILMN_1743910 | 0.0366 | 0.00544 | -2.92 | -2.65487 | -0.321 |
| CCM2 | ILMN_1692271 | 0.0545 | 0.00931 | -2.71 | -3.14204 | -0.321 |
| AVIL | ILMN_1674265 | 0.13 | 0.0305 | -2.23 | -4.19165 | -0.321 |
| FAM120B | ILMN_1679641 | 5.53E-05 | 1.11E-06 | -5.61 | 5.37254 | -0.322 |
| FXR1 | ILMN_1679640 | 0.000123 | 3.08E-06 | -5.31 | 4.39359 | -0.322 |
| PDE1B | ILMN_1805098 | 0.00345 | 0.000247 | -3.97 | 0.21824 | -0.322 |
| SUGP2 | ILMN_1711270 | 0.0387 | 0.00584 | -2.89 | -2.72068 | -0.322 |
| PKN1 | ILMN_2367710 | 0.0514 | 0.0086 | -2.75 | -3.07053 | -0.322 |
| TMEM51 | ILMN_1674985 | 0.00346 | 0.000248 | -3.97 | 0.2143 | -0.323 |
| CCNY | ILMN_1708991 | 0.0104 | 0.00104 | -3.5 | -1.13281 | -0.323 |
| DPM2 | ILMN_1732049 | 0.0319 | 0.00456 | -2.98 | -2.49431 | -0.323 |
| CNPY4 | ILMN_2252408 | 0.0589 | 0.0104 | -2.67 | -3.23939 | -0.323 |
| CXorf23 | ILMN_1772943 | 0.0764 | 0.0147 | -2.54 | -3.55134 | -0.323 |
| SP3 | ILMN_2389844 | 0.0856 | 0.0171 | -2.47 | -3.68426 | -0.323 |
| RNPC3 | ILMN_2150284 | 0.127 | 0.0295 | -2.25 | -4.16514 | -0.323 |
| ADNP | ILMN_1657993 | 0.000318 | 1.07E-05 | -4.94 | 3.20358 | -0.324 |
| GGA3 | ILMN_1675982 | 0.000366 | 1.28E-05 | -4.89 | 3.02865 | -0.324 |
| ATF4 | ILMN_1672128 | 0.00304 | 0.000209 | -4.03 | 0.37558 | -0.324 |
| OTUD6B | ILMN_2215631 | 0.00931 | 0.000892 | -3.55 | -0.98662 | -0.324 |
| SLC2A1 | ILMN_1659027 | 0.0308 | 0.00434 | -3 | -2.44924 | -0.324 |
| FDFT1 | ILMN_2144088 | 0.0362 | 0.00536 | -2.92 | -2.64207 | -0.324 |
| THAP11 | ILMN_1780699 | 0.0839 | 0.0166 | -2.49 | -3.66134 | -0.324 |
| NME8 | ILMN_1691334 | 0.118 | 0.0267 | -2.29 | -4.07792 | -0.324 |
| RASGRP4 | ILMN_1791955 | 0.0306 | 0.0043 | -3 | -2.44024 | -0.325 |
| ADAM10 | ILMN_2148360 | 0.162 | 0.0418 | -2.09 | -4.46459 | -0.325 |
| STX6 | ILMN_2157951 | 0.000711 | 3.05E-05 | -4.63 | 2.2025 | -0.326 |
| ACTB | ILMN_1777296 | 0.00179 | 0.000103 | -4.25 | 1.04111 | -0.326 |
| RMI1 | ILMN_2151488 | 0.0265 | 0.00358 | -3.07 | -2.27316 | -0.326 |
| NUTF2 | ILMN_1655046 | 0.111 | 0.0243 | -2.33 | -3.99603 | -0.326 |
| ZNF395 | ILMN_1772876 | 0.114 | 0.0254 | -2.31 | -4.03433 | -0.326 |
| NUAK2 | ILMN_1789793 | 0.141 | 0.0339 | -2.19 | -4.28507 | -0.326 |
| PTPRE | ILMN_2383611 | 0.000246 | 7.72E-06 | -5.04 | 3.5148 | -0.327 |
| ARHGAP9 | ILMN_1663916 | 0.000356 | 1.23E-05 | -4.9 | 3.06584 | -0.327 |
| AASDHPPT | ILMN_2096191 | 0.0135 | 0.00146 | -3.39 | -1.4483 | -0.327 |
| LEO1 | ILMN_1801553 | 0.0201 | 0.00247 | -3.2 | -1.9307 | -0.327 |
| U2AF2 | ILMN_2385173 | 0.0338 | 0.0049 | -2.96 | -2.56013 | -0.327 |
| KLF13 | ILMN_1679929 | 0.0589 | 0.0104 | -2.67 | -3.23816 | -0.327 |
| DNAJC17 | ILMN_1703573 | 0.064 | 0.0115 | -2.63 | -3.33377 | -0.327 |
| SLX4IP | ILMN_1715625 | 0.0725 | 0.0138 | -2.56 | -3.49163 | -0.327 |
| POTEE | ILMN_3242016 | 0.0927 | 0.0191 | -2.43 | -3.78202 | -0.327 |
| CD300C | ILMN_1701906 | 0.0996 | 0.021 | -2.39 | -3.86738 | -0.327 |
| SLC25A29 | ILMN_1697544 | 0.147 | 0.0362 | -2.16 | -4.34113 | -0.327 |
| COQ10A | ILMN_1666364 | 0.000675 | 2.85E-05 | -4.65 | 2.26788 | -0.328 |
| EXOSC10 | ILMN_1670796 | 0.00224 | 0.000141 | -4.15 | 0.75124 | -0.328 |
| PSMB5 | ILMN_1744649 | 0.00324 | 0.000228 | -4 | 0.29274 | -0.328 |
| AHCTF1 | ILMN_1747504 | 0.00327 | 0.000231 | -4 | 0.28192 | -0.328 |
| SNRNP40 | ILMN_3247064 | 0.0118 | 0.00123 | -3.44 | -1.288 | -0.328 |
| EPHB1 | ILMN_1692261 | 0.0228 | 0.00293 | -3.14 | -2.08919 | -0.328 |
| DUSP23 | ILMN_1659462 | 0.0291 | 0.00402 | -3.03 | -2.37971 | -0.328 |
| KIF2A | ILMN_1734476 | 0.0363 | 0.00537 | -2.92 | -2.64433 | -0.328 |
| E4F1 | ILMN_1720287 | 0.0644 | 0.0116 | -2.63 | -3.34082 | -0.328 |
| RUNX1 | ILMN_1801504 | 0.0927 | 0.0191 | -2.43 | -3.78132 | -0.328 |
| XYLT2 | ILMN_1799815 | 0.161 | 0.0416 | -2.1 | -4.46024 | -0.328 |
| AIP | ILMN_2103841 | 0.0338 | 0.0049 | -2.96 | -2.56081 | -0.329 |
| NUAK2 | ILMN_2094952 | 0.145 | 0.0353 | -2.17 | -4.32051 | -0.329 |
| IFNGR2 | ILMN_1764964 | 0.000314 | 1.05E-05 | -4.95 | 3.22157 | -0.33 |
| RUFY1 | ILMN_2310968 | 0.00352 | 0.000254 | -3.97 | 0.19103 | -0.33 |
| WASL | ILMN_1666004 | 0.0143 | 0.00159 | -3.36 | -1.52254 | -0.33 |
| STARD10 | ILMN_1717052 | 0.05 | 0.00824 | -2.76 | -3.03155 | -0.33 |
| RAB24 | ILMN_1714393 | 0.0698 | 0.013 | -2.58 | -3.44496 | -0.33 |
| NLRP1 | ILMN_1810045 | 0.0753 | 0.0144 | -2.54 | -3.5348 | -0.33 |
| TMEM69 | ILMN_1681972 | 0.09 | 0.0183 | -2.45 | -3.74444 | -0.33 |
| MSN | ILMN_1659895 | 0.00228 | 0.000144 | -4.15 | 0.72725 | -0.331 |
| FKBP1A | ILMN_2333367 | 0.0114 | 0.00118 | -3.46 | -1.2461 | -0.331 |
| SGF29 | ILMN_1684789 | 0.0163 | 0.00188 | -3.3 | -1.68151 | -0.331 |
| CCL23 | ILMN_1686109 | 0.0189 | 0.00229 | -3.23 | -1.86027 | -0.331 |
| EIF3B | ILMN_1715636 | 0.0345 | 0.00502 | -2.95 | -2.58227 | -0.331 |
| CCDC125 | ILMN_1653480 | 0.0475 | 0.00768 | -2.79 | -2.96836 | -0.331 |
| GPN2 | ILMN_2205245 | 0.0681 | 0.0127 | -2.6 | -3.41747 | -0.331 |
| FAM111A | ILMN_2410038 | 0.0968 | 0.0202 | -2.41 | -3.83423 | -0.331 |
| LASP1 | ILMN_1665909 | 4.78E-05 | 9.24E-07 | -5.66 | 5.55068 | -0.332 |
| ARAP1 | ILMN_1772233 | 0.00174 | 1.00E-04 | -4.26 | 1.07381 | -0.332 |
| GNPAT | ILMN_1699859 | 0.0174 | 0.00205 | -3.27 | -1.75763 | -0.332 |
| SETD1B | ILMN_1813573 | 0.0328 | 0.00471 | -2.97 | -2.52456 | -0.332 |
| ZBTB49 | ILMN_2103388 | 0.0702 | 0.0131 | -2.58 | -3.45172 | -0.332 |
| ZFP91 | ILMN_1703053 | 0.0876 | 0.0177 | -2.46 | -3.714 | -0.332 |
| JUN | ILMN_1806023 | 0.122 | 0.0279 | -2.27 | -4.11709 | -0.332 |
| PDLIM7 | ILMN_1814985 | 0.000687 | 2.91E-05 | -4.64 | 2.24652 | -0.333 |
| EXOC7 | ILMN_1815012 | 0.00259 | 0.00017 | -4.09 | 0.57034 | -0.333 |
| POM121C | ILMN_3235808 | 0.00985 | 0.000968 | -3.53 | -1.06276 | -0.333 |
| SETD1A | ILMN_1714327 | 0.0101 | 0.001 | -3.51 | -1.0959 | -0.333 |
| CTRC | ILMN_1748730 | 0.0124 | 0.00133 | -3.42 | -1.35622 | -0.333 |
| PRKCB | ILMN_1713603 | 0.0235 | 0.00304 | -3.13 | -2.12297 | -0.333 |
| TAF9B | ILMN_1784880 | 0.0336 | 0.00485 | -2.96 | -2.55106 | -0.333 |
| RPS6KB2 | ILMN_2364357 | 0.048 | 0.0078 | -2.78 | -2.98216 | -0.333 |
| COQ9 | ILMN_1756898 | 0.0696 | 0.013 | -2.58 | -3.44178 | -0.333 |
| CASS4 | ILMN_1678061 | 0.145 | 0.0355 | -2.17 | -4.32447 | -0.333 |
| HERC4 | ILMN_1678922 | 6.48E-07 | 2.38E-09 | -7.39 | 11.28912 | -0.334 |
| RAVER1 | ILMN_1740395 | 0.0014 | 7.41E-05 | -4.35 | 1.35789 | -0.334 |
| CIAO1 | ILMN_2048793 | 0.00707 | 0.000623 | -3.67 | -0.65029 | -0.334 |
| PKM | ILMN_2366634 | 0.0463 | 0.00744 | -2.8 | -2.94015 | -0.334 |
| EXOSC10 | ILMN_2402168 | 0.0508 | 0.00841 | -2.75 | -3.05075 | -0.334 |
| WRB | ILMN_2085922 | 0.145 | 0.0356 | -2.17 | -4.32666 | -0.334 |
| LCLAT1 | ILMN_3262936 | 0.00503 | 0.000402 | -3.82 | -0.24013 | -0.335 |
| UBA52 | ILMN_2368576 | 0.0118 | 0.00123 | -3.44 | -1.28911 | -0.335 |
| RPAIN | ILMN_1770339 | 0.042 | 0.00652 | -2.85 | -2.82043 | -0.335 |
| LUC7L | ILMN_1765371 | 0.109 | 0.0238 | -2.34 | -3.97525 | -0.335 |
| CDV3 | ILMN_1673788 | 0.000164 | 4.50E-06 | -5.2 | 4.03156 | -0.336 |
| CDKN1B | ILMN_1722811 | 0.00564 | 0.000465 | -3.77 | -0.37624 | -0.336 |
| SEC13 | ILMN_3297880 | 0.0169 | 0.00198 | -3.28 | -1.72547 | -0.336 |
| TMEM54 | ILMN_2059689 | 0.0509 | 0.00845 | -2.75 | -3.05427 | -0.336 |
| AOC2 | ILMN_2405185 | 0.0588 | 0.0103 | -2.67 | -3.2364 | -0.336 |
| ZNF559 | ILMN_1677785 | 0.0599 | 0.0106 | -2.66 | -3.25941 | -0.336 |
| USP34 | ILMN_1739454 | 1.35E-05 | 1.64E-07 | -6.17 | 7.21088 | -0.337 |
| PRDM2 | ILMN_2258543 | 0.000848 | 3.81E-05 | -4.56 | 1.98983 | -0.337 |
| WDR20 | ILMN_2409706 | 0.012 | 0.00126 | -3.44 | -1.3068 | -0.337 |
| HEATR5B | ILMN_2187487 | 0.0929 | 0.0191 | -2.43 | -3.78423 | -0.337 |
| MTFP1 | ILMN_2355665 | 0.113 | 0.0249 | -2.32 | -4.01464 | -0.337 |
| FOLR3 | ILMN_1730454 | 0.178 | 0.0477 | -2.03 | -4.57742 | -0.337 |
| RABEP1 | ILMN_1719622 | 8.81E-05 | 2.04E-06 | -5.43 | 4.78856 | -0.338 |
| ATF4 | ILMN_2358457 | 0.006 | 0.000504 | -3.74 | -0.45272 | -0.338 |
| ELF2 | ILMN_1772486 | 0.00653 | 0.000563 | -3.71 | -0.55652 | -0.338 |
| PRICKLE3 | ILMN_1656942 | 0.00796 | 0.00073 | -3.62 | -0.79955 | -0.338 |
| WDR33 | ILMN_1716086 | 0.0106 | 0.00107 | -3.49 | -1.1571 | -0.338 |
| VKORC1L1 | ILMN_1802894 | 0.0567 | 0.00984 | -2.69 | -3.19222 | -0.338 |
| MAP3K7 | ILMN_1810176 | 8.68E-07 | 3.51E-09 | -7.28 | 10.91455 | -0.339 |
| BTBD10 | ILMN_1809344 | 1.06E-05 | 1.17E-07 | -6.27 | 7.54168 | -0.339 |
| PPIG | ILMN_1807611 | 0.00168 | 9.48E-05 | -4.28 | 1.12429 | -0.339 |
| YIF1B | ILMN_2363668 | 0.0103 | 0.00103 | -3.51 | -1.1173 | -0.339 |
| CERK | ILMN_1767475 | 0.0155 | 0.00177 | -3.32 | -1.62563 | -0.339 |
| UNC93B1 | ILMN_1654812 | 0.0237 | 0.00308 | -3.13 | -2.13386 | -0.339 |
| LGALS9 | ILMN_2412214 | 0.0498 | 0.00817 | -2.76 | -3.02453 | -0.339 |
| CWC22 | ILMN_1674128 | 0.0589 | 0.0104 | -2.67 | -3.23914 | -0.339 |
| TAPT1 | ILMN_1693882 | 0.143 | 0.0348 | -2.18 | -4.30646 | -0.339 |
| CCNG2 | ILMN_1879480 | 0.154 | 0.0387 | -2.13 | -4.39879 | -0.339 |
| PTK2B | ILMN_1732318 | 0.000667 | 2.79E-05 | -4.65 | 2.28699 | -0.34 |
| SETDB1 | ILMN_1718207 | 0.00316 | 0.000221 | -4.01 | 0.32285 | -0.34 |
| PRDX5 | ILMN_1711606 | 0.0082 | 0.00076 | -3.61 | -0.83657 | -0.34 |
| ARSB | ILMN_2242937 | 0.0511 | 0.00851 | -2.75 | -3.06073 | -0.34 |
| P2RX1 | ILMN_1758529 | 0.0104 | 0.00105 | -3.5 | -1.13576 | -0.341 |
| MON1B | ILMN_1771651 | 0.0139 | 0.00153 | -3.37 | -1.48639 | -0.341 |
| ZNF701 | ILMN_1743067 | 0.0156 | 0.00178 | -3.32 | -1.63026 | -0.341 |
| RNF220 | ILMN_1694504 | 0.0239 | 0.00312 | -3.12 | -2.14647 | -0.341 |
| CHCHD1 | ILMN_1672149 | 0.0531 | 0.009 | -2.73 | -3.11129 | -0.341 |
| STK26 | ILMN_2328776 | 0.151 | 0.0378 | -2.14 | -4.37774 | -0.341 |
| STK40 | ILMN_2075927 | 0.00016 | 4.32E-06 | -5.21 | 4.06977 | -0.342 |
| CDV3 | ILMN_1810977 | 0.000258 | 8.25E-06 | -5.02 | 3.45119 | -0.342 |
| RSBN1L | ILMN_1712027 | 0.0148 | 0.00166 | -3.34 | -1.56146 | -0.342 |
| CDKN1B | ILMN_2196347 | 0.0473 | 0.00763 | -2.79 | -2.96208 | -0.342 |
| PHTF1 | ILMN_1803464 | 0.08 | 0.0156 | -2.51 | -3.6056 | -0.342 |
| CCDC88C | ILMN_3248352 | 0.094 | 0.0195 | -2.42 | -3.79958 | -0.342 |
| GIMAP7 | ILMN_1776678 | 0.136 | 0.0325 | -2.21 | -4.24797 | -0.342 |
| RNF145 | ILMN_1710906 | 1.41E-06 | 6.69E-09 | -7.09 | 10.29495 | -0.343 |
| TMEM87A | ILMN_1716816 | 4.60E-05 | 8.68E-07 | -5.68 | 5.61075 | -0.343 |
| POTEF | ILMN_3241758 | 6.34E-05 | 1.33E-06 | -5.56 | 5.19772 | -0.343 |
| PPM1G | ILMN_1806867 | 0.000319 | 1.08E-05 | -4.94 | 3.19529 | -0.343 |
| MGAT2 | ILMN_2342240 | 0.00471 | 0.000369 | -3.85 | -0.1588 | -0.343 |
| USP22 | ILMN_1666609 | 0.00794 | 0.000728 | -3.62 | -0.79705 | -0.343 |
| IMPDH1 | ILMN_2388363 | 0.102 | 0.0218 | -2.37 | -3.90169 | -0.343 |
| P4HTM | ILMN_3300313 | 0.133 | 0.0314 | -2.22 | -4.21781 | -0.343 |
| SNX6 | ILMN_1696294 | 0.000401 | 1.44E-05 | -4.85 | 2.92134 | -0.344 |
| PBX2 | ILMN_1682699 | 0.00307 | 0.000212 | -4.02 | 0.36132 | -0.344 |
| NCOR2 | ILMN_2256868 | 0.00443 | 0.000343 | -3.87 | -0.09016 | -0.344 |
| DIAPH1 | ILMN_2321416 | 0.00972 | 0.000949 | -3.53 | -1.04475 | -0.344 |
| CYP2D7 | ILMN_2226519 | 0.0139 | 0.00152 | -3.37 | -1.48469 | -0.344 |
| DNMT1 | ILMN_1760201 | 0.0182 | 0.00216 | -3.25 | -1.80946 | -0.344 |
| NDRG3 | ILMN_2385097 | 0.0804 | 0.0157 | -2.51 | -3.61124 | -0.344 |
| VCP | ILMN_1777220 | 0.00211 | 0.00013 | -4.18 | 0.82821 | -0.345 |
| TMEM189 | ILMN_2162989 | 0.00259 | 0.000169 | -4.09 | 0.57439 | -0.345 |
| LSP1 | ILMN_2355225 | 0.0037 | 0.000271 | -3.95 | 0.13134 | -0.345 |
| DDX56 | ILMN_1679405 | 0.0558 | 0.00961 | -2.7 | -3.17065 | -0.345 |
| VEGFB | ILMN_1726981 | 0.0724 | 0.0137 | -2.56 | -3.48828 | -0.345 |
| PLGLB1 | ILMN_1751811 | 0.0847 | 0.0168 | -2.48 | -3.6723 | -0.345 |
| RAI1 | ILMN_1739810 | 0.0967 | 0.0202 | -2.41 | -3.83196 | -0.345 |
| MZF1 | ILMN_1749838 | 0.119 | 0.0268 | -2.29 | -4.08039 | -0.345 |
| PANX2 | ILMN_1694810 | 0.00139 | 7.32E-05 | -4.36 | 1.36982 | -0.346 |
| SRP68 | ILMN_1703524 | 0.0173 | 0.00203 | -3.27 | -1.74846 | -0.346 |
| ALAD | ILMN_1747577 | 0.0331 | 0.00476 | -2.97 | -2.5337 | -0.346 |
| LUZP1 | ILMN_1714159 | 0.0532 | 0.00903 | -2.73 | -3.11475 | -0.346 |
| RNF185 | ILMN_1691293 | 0.07 | 0.0131 | -2.58 | -3.44758 | -0.346 |
| NUMA1 | ILMN_2160005 | 0.0715 | 0.0135 | -2.57 | -3.4751 | -0.346 |
| KIAA0226L | ILMN_2196550 | 0.125 | 0.0287 | -2.26 | -4.14161 | -0.346 |
| MIB2 | ILMN_2385178 | 0.159 | 0.0406 | -2.11 | -4.43942 | -0.346 |
| PPP1R18 | ILMN_1732967 | 0.000161 | 4.36E-06 | -5.21 | 4.06155 | -0.347 |
| BUD13 | ILMN_1749210 | 0.000279 | 9.12E-06 | -4.99 | 3.35565 | -0.347 |
| STX10 | ILMN_1753712 | 0.0432 | 0.00677 | -2.84 | -2.85359 | -0.347 |
| WDR61 | ILMN_1665887 | 0.0799 | 0.0156 | -2.51 | -3.60275 | -0.347 |
| L3MBTL2 | ILMN_3307786 | 0.0908 | 0.0185 | -2.44 | -3.75681 | -0.347 |
| PPARD | ILMN_1674282 | 0.169 | 0.0443 | -2.07 | -4.51438 | -0.347 |
| SREK1 | ILMN_2373266 | 0.00257 | 0.000168 | -4.1 | 0.57994 | -0.348 |
| SEC23B | ILMN_2366246 | 0.00639 | 0.000547 | -3.72 | -0.52925 | -0.348 |
| TXNDC17 | ILMN_1659437 | 0.0151 | 0.00172 | -3.33 | -1.59477 | -0.348 |
| HPSE | ILMN_2092850 | 0.0216 | 0.00271 | -3.17 | -2.01866 | -0.348 |
| PKNOX1 | ILMN_1745365 | 0.049 | 0.00799 | -2.77 | -3.00458 | -0.348 |
| DHX29 | ILMN_2090123 | 0.0538 | 0.00917 | -2.72 | -3.12838 | -0.348 |
| LYSMD3 | ILMN_1766718 | 0.081 | 0.0159 | -2.5 | -3.62098 | -0.348 |
| SLC52A2 | ILMN_2041577 | 0.151 | 0.0377 | -2.14 | -4.37536 | -0.348 |
| MDC1 | ILMN_1814122 | 0.156 | 0.0394 | -2.12 | -4.41453 | -0.348 |
| FLNA | ILMN_1687335 | 0.178 | 0.0477 | -2.03 | -4.57805 | -0.348 |
| TMEM256 | ILMN_2201533 | 0.00128 | 6.65E-05 | -4.39 | 1.46011 | -0.349 |
| KCTD5 | ILMN_1672728 | 0.0017 | 9.63E-05 | -4.27 | 1.10943 | -0.349 |
| NAA10 | ILMN_1721977 | 0.00422 | 0.00032 | -3.89 | -0.0264 | -0.349 |
| REL | ILMN_2124064 | 0.0209 | 0.0026 | -3.18 | -1.97999 | -0.349 |
| ANGEL2 | ILMN_1736340 | 0.0221 | 0.00281 | -3.16 | -2.05003 | -0.349 |
| UBE2C | ILMN_2301083 | 0.0232 | 0.003 | -3.13 | -2.11046 | -0.349 |
| ZSCAN29 | ILMN_1691772 | 0.0356 | 0.00524 | -2.93 | -2.62057 | -0.349 |
| ATF1 | ILMN_1801923 | 0.0411 | 0.00633 | -2.86 | -2.79319 | -0.349 |
| PA2G4 | ILMN_1728984 | 0.045 | 0.00716 | -2.82 | -2.90479 | -0.349 |
| DNASE2 | ILMN_1796245 | 0.0797 | 0.0155 | -2.51 | -3.59928 | -0.349 |
| REXO1 | ILMN_1753008 | 0.000168 | 4.63E-06 | -5.19 | 4.00415 | -0.35 |
| CASP8 | ILMN_1673757 | 0.00107 | 5.23E-05 | -4.46 | 1.68944 | -0.35 |
| ABCB6 | ILMN_2193980 | 0.016 | 0.00185 | -3.31 | -1.66227 | -0.35 |
| AP1B1 | ILMN_1781983 | 0.0319 | 0.00456 | -2.98 | -2.4943 | -0.35 |
| ZBTB2 | ILMN_1766247 | 0.0313 | 0.00444 | -2.99 | -2.46989 | -0.351 |
| FKRP | ILMN_2368617 | 0.0513 | 0.00856 | -2.75 | -3.06604 | -0.351 |
| C7orf43 | ILMN_1770030 | 0.0806 | 0.0158 | -2.51 | -3.61414 | -0.351 |
| NCAPD2 | ILMN_1775008 | 0.00159 | 8.75E-05 | -4.3 | 1.20075 | -0.352 |
| CMIP | ILMN_1738075 | 0.00195 | 0.000116 | -4.21 | 0.93476 | -0.352 |
| KCNK6 | ILMN_2074773 | 0.0904 | 0.0184 | -2.44 | -3.75019 | -0.352 |
| DYNLL1 | ILMN_1795227 | 2.04E-05 | 2.95E-07 | -6 | 6.64793 | -0.353 |
| HCG26 | ILMN_3236741 | 0.0405 | 0.00621 | -2.87 | -2.77544 | -0.353 |
| PMS2CL | ILMN_2262203 | 0.0427 | 0.00667 | -2.84 | -2.84006 | -0.353 |
| ATP2C1 | ILMN_2340565 | 0.0515 | 0.00862 | -2.74 | -3.07258 | -0.353 |
| VPS41 | ILMN_1703379 | 0.00212 | 0.00013 | -4.18 | 0.82473 | -0.354 |
| ZNF385A | ILMN_1786722 | 0.00343 | 0.000246 | -3.98 | 0.22313 | -0.354 |
| CENPBD1 | ILMN_3238740 | 0.0293 | 0.00406 | -3.03 | -2.38796 | -0.354 |
| TFRC | ILMN_1674243 | 0.0314 | 0.00445 | -2.99 | -2.47193 | -0.354 |
| FUT11 | ILMN_1678862 | 0.0427 | 0.00666 | -2.84 | -2.83953 | -0.354 |
| ABCF1 | ILMN_1763875 | 0.0713 | 0.0134 | -2.57 | -3.47189 | -0.354 |
| COL7A1 | ILMN_1751161 | 0.102 | 0.0217 | -2.38 | -3.89397 | -0.354 |
| DOCK2 | ILMN_1799725 | 0.000608 | 2.48E-05 | -4.69 | 2.39835 | -0.355 |
| C2CD2L | ILMN_1778132 | 0.00642 | 0.000551 | -3.71 | -0.53593 | -0.355 |
| GSDMB | ILMN_1666206 | 0.0108 | 0.0011 | -3.48 | -1.18243 | -0.355 |
| HAUS8 | ILMN_1768020 | 0.0226 | 0.00289 | -3.15 | -2.07725 | -0.355 |
| LBHD1 | ILMN_1739345 | 0.0371 | 0.00553 | -2.91 | -2.67012 | -0.355 |
| INO80 | ILMN_3248343 | 0.0416 | 0.00643 | -2.86 | -2.80777 | -0.355 |
| MRFAP1L1 | ILMN_1693702 | 0.049 | 0.008 | -2.77 | -3.00537 | -0.355 |
| GNA12 | ILMN_2216157 | 0.053 | 0.00897 | -2.73 | -3.10869 | -0.355 |
| AMY1B | ILMN_1726327 | 0.121 | 0.0277 | -2.27 | -4.10874 | -0.355 |
| CAB39 | ILMN_1765858 | 1.41E-06 | 6.75E-09 | -7.09 | 10.28525 | -0.356 |
| LOC646214 | ILMN_3243351 | 1.64E-05 | 2.16E-07 | -6.09 | 6.94955 | -0.356 |
| PCIF1 | ILMN_2232368 | 0.0029 | 0.000197 | -4.05 | 0.43376 | -0.356 |
| LILRA3 | ILMN_1661631 | 0.0129 | 0.00139 | -3.4 | -1.39647 | -0.356 |
| TAPBPL | ILMN_1805449 | 0.0331 | 0.00476 | -2.97 | -2.5335 | -0.356 |
| ALKBH1 | ILMN_2375973 | 0.0417 | 0.00645 | -2.85 | -2.81014 | -0.356 |
| MED11 | ILMN_1762639 | 0.05 | 0.00822 | -2.76 | -3.03014 | -0.356 |
| SKP2 | ILMN_1665538 | 0.051 | 0.00848 | -2.75 | -3.05819 | -0.356 |
| CCAR2 | ILMN_1804789 | 0.0612 | 0.0109 | -2.65 | -3.28452 | -0.356 |
| S100PBP | ILMN_1663577 | 0.105 | 0.0225 | -2.36 | -3.92818 | -0.356 |
| CNOT4 | ILMN_1772677 | 0.000127 | 3.22E-06 | -5.3 | 4.35261 | -0.357 |
| OSBPL11 | ILMN_1687410 | 0.00173 | 9.85E-05 | -4.27 | 1.088 | -0.357 |
| SRPRA | ILMN_1785660 | 0.00187 | 0.000109 | -4.23 | 0.9902 | -0.357 |
| SEC24C | ILMN_1676600 | 0.0218 | 0.00275 | -3.16 | -2.03155 | -0.357 |
| RPA1 | ILMN_2049642 | 0.0439 | 0.00691 | -2.83 | -2.8731 | -0.357 |
| GRN | ILMN_1724250 | 0.0495 | 0.00812 | -2.77 | -3.01903 | -0.357 |
| MTERF1 | ILMN_2099249 | 0.0548 | 0.0094 | -2.71 | -3.15058 | -0.357 |
| KCTD5 | ILMN_1652331 | 0.0643 | 0.0116 | -2.63 | -3.33875 | -0.357 |
| PAK2 | ILMN_1712687 | 5.83E-05 | 1.20E-06 | -5.59 | 5.30052 | -0.358 |
| GTF3C2 | ILMN_1746457 | 8.95E-05 | 2.09E-06 | -5.43 | 4.76897 | -0.358 |
| NUP155 | ILMN_1768293 | 0.00245 | 0.000159 | -4.12 | 0.63687 | -0.358 |
| CASP8 | ILMN_2377733 | 0.00266 | 0.000175 | -4.08 | 0.54257 | -0.358 |
| KCTD21 | ILMN_1809708 | 0.0197 | 0.00242 | -3.21 | -1.91188 | -0.358 |
| TECPR1 | ILMN_1767651 | 0.0204 | 0.00253 | -3.19 | -1.95414 | -0.358 |
| RNF146 | ILMN_1682626 | 0.0393 | 0.00597 | -2.88 | -2.74005 | -0.358 |
| LILRA2 | ILMN_1716983 | 0.0563 | 0.00974 | -2.7 | -3.18235 | -0.358 |
| DGKA | ILMN_2319913 | 0.0614 | 0.0109 | -2.65 | -3.28795 | -0.358 |
| HMGCL | ILMN_2122420 | 0.0656 | 0.0119 | -2.62 | -3.3644 | -0.358 |
| RSPH3 | ILMN_1788223 | 0.116 | 0.0259 | -2.3 | -4.05164 | -0.358 |
| ELF4 | ILMN_1652082 | 0.00041 | 1.48E-05 | -4.84 | 2.89179 | -0.359 |
| ABR | ILMN_1655114 | 0.00282 | 0.00019 | -4.06 | 0.46699 | -0.359 |
| CKAP4 | ILMN_1790891 | 0.00414 | 0.000312 | -3.9 | -0.00291 | -0.359 |
| REV1 | ILMN_1707062 | 0.0196 | 0.00239 | -3.21 | -1.9019 | -0.359 |
| BCL9L | ILMN_1743966 | 0.0246 | 0.00325 | -3.11 | -2.18527 | -0.359 |
| EXOSC3 | ILMN_1734194 | 3.41E-05 | 5.77E-07 | -5.8 | 6.00271 | -0.36 |
| VASP | ILMN_1743646 | 0.000567 | 2.24E-05 | -4.72 | 2.49733 | -0.36 |
| ARAP1 | ILMN_3269719 | 0.000672 | 2.82E-05 | -4.65 | 2.27649 | -0.36 |
| UBP1 | ILMN_1784410 | 0.00154 | 8.36E-05 | -4.32 | 1.24384 | -0.36 |
| SLTM | ILMN_2395204 | 0.0037 | 0.000271 | -3.95 | 0.13139 | -0.36 |
| GTF2I | ILMN_1673917 | 0.00483 | 0.000381 | -3.83 | -0.19042 | -0.36 |
| ZDHHC16 | ILMN_1763568 | 0.0228 | 0.00292 | -3.14 | -2.08559 | -0.36 |
| TMEM55A | ILMN_1752117 | 0.0668 | 0.0123 | -2.61 | -3.3889 | -0.36 |
| KCND1 | ILMN_1668586 | 0.0787 | 0.0153 | -2.52 | -3.58612 | -0.36 |
| ANKRD37 | ILMN_1756417 | 0.119 | 0.027 | -2.29 | -4.08541 | -0.36 |
| APOBR | ILMN_1697925 | 2.28E-05 | 3.39E-07 | -5.96 | 6.51361 | -0.361 |
| PFN1 | ILMN_2151817 | 0.000194 | 5.63E-06 | -5.13 | 3.81641 | -0.361 |
| RAB11FIP1 | ILMN_2409596 | 0.00045 | 1.67E-05 | -4.81 | 2.77531 | -0.361 |
| ACOX1 | ILMN_1750158 | 0.00104 | 5.01E-05 | -4.47 | 1.72908 | -0.361 |
| KIAA1147 | ILMN_2355033 | 0.0135 | 0.00148 | -3.38 | -1.45493 | -0.361 |
| PDE7A | ILMN_2278819 | 0.0229 | 0.00295 | -3.14 | -2.09413 | -0.361 |
| BCL2L12 | ILMN_1752953 | 0.0314 | 0.00446 | -2.99 | -2.47341 | -0.361 |
| STK26 | ILMN_1676159 | 0.0339 | 0.00491 | -2.96 | -2.56188 | -0.361 |
| TPM4 | ILMN_2140974 | 0.0926 | 0.019 | -2.43 | -3.77981 | -0.361 |
| TJAP1 | ILMN_1743763 | 0.0137 | 0.00149 | -3.38 | -1.46526 | -0.362 |
| NDE1 | ILMN_1739805 | 0.0267 | 0.00362 | -3.07 | -2.28351 | -0.362 |
| CUX1 | ILMN_1687567 | 0.0318 | 0.00453 | -2.99 | -2.48894 | -0.362 |
| ZCCHC3 | ILMN_1786852 | 0.00161 | 8.91E-05 | -4.3 | 1.18349 | -0.363 |
| TCF20 | ILMN_1660144 | 0.00838 | 0.00078 | -3.6 | -0.86153 | -0.363 |
| CNOT10 | ILMN_2078334 | 0.0101 | 0.00101 | -3.51 | -1.09776 | -0.363 |
| PURA | ILMN_2127328 | 0.0189 | 0.00227 | -3.23 | -1.85519 | -0.363 |
| KCNAB2 | ILMN_2342762 | 0.0442 | 0.00699 | -2.82 | -2.8831 | -0.363 |
| ZNF219 | ILMN_1688295 | 0.0529 | 0.00896 | -2.73 | -3.1076 | -0.363 |
| PGBD3 | ILMN_1737416 | 0.062 | 0.0111 | -2.65 | -3.2998 | -0.363 |
| FKBP2 | ILMN_1807563 | 0.0709 | 0.0133 | -2.57 | -3.46414 | -0.363 |
| ZNF398 | ILMN_2344047 | 0.082 | 0.0161 | -2.5 | -3.63378 | -0.363 |
| ARFGEF1 | ILMN_2221408 | 0.144 | 0.0351 | -2.17 | -4.31582 | -0.363 |
| AMY1C | ILMN_1663313 | 0.00275 | 0.000182 | -4.07 | 0.5051 | -0.364 |
| MFN2 | ILMN_1651385 | 0.00325 | 0.000229 | -4 | 0.29047 | -0.364 |
| PAPOLG | ILMN_1786275 | 0.0237 | 0.00309 | -3.12 | -2.13916 | -0.364 |
| GLT8D1 | ILMN_1713290 | 0.0381 | 0.00573 | -2.9 | -2.70236 | -0.364 |
| FAM53B | ILMN_2053490 | 0.0472 | 0.00762 | -2.79 | -2.96164 | -0.364 |
| FXR2 | ILMN_1738699 | 0.0528 | 0.00891 | -2.73 | -3.10255 | -0.364 |
| CYP4F3 | ILMN_2089484 | 0.103 | 0.022 | -2.37 | -3.90641 | -0.364 |
| E2F4 | ILMN_1761828 | 0.000174 | 4.88E-06 | -5.18 | 3.95435 | -0.365 |
| CUX1 | ILMN_2278636 | 0.00034 | 1.16E-05 | -4.92 | 3.1275 | -0.365 |
| GNPTAB | ILMN_1736757 | 0.000516 | 1.99E-05 | -4.76 | 2.61065 | -0.365 |
| ACTG1 | ILMN_2053178 | 0.000686 | 2.90E-05 | -4.64 | 2.24961 | -0.365 |
| VAV3 | ILMN_1657679 | 0.00242 | 0.000156 | -4.12 | 0.6519 | -0.365 |
| RHOQ | ILMN_1810559 | 0.00963 | 0.000936 | -3.54 | -1.0313 | -0.365 |
| ZNRD1 | ILMN_1722894 | 0.0124 | 0.00132 | -3.42 | -1.35389 | -0.365 |
| EHBP1L1 | ILMN_3244457 | 0.0147 | 0.00164 | -3.35 | -1.55251 | -0.365 |
| CLN3 | ILMN_2394381 | 0.017 | 0.00199 | -3.28 | -1.73358 | -0.365 |
| TCF7L2 | ILMN_1672486 | 0.0232 | 0.003 | -3.13 | -2.11038 | -0.365 |
| NUMA1 | ILMN_1757085 | 0.0534 | 0.00907 | -2.72 | -3.11894 | -0.365 |
| CPSF1 | ILMN_1654545 | 0.0855 | 0.0171 | -2.48 | -3.68309 | -0.365 |
| TMOD2 | ILMN_3250273 | 0.0924 | 0.019 | -2.43 | -3.77698 | -0.365 |
| VPS37C | ILMN_1789233 | 0.000591 | 2.39E-05 | -4.7 | 2.43662 | -0.366 |
| ALS2 | ILMN_1750256 | 0.0086 | 0.000806 | -3.59 | -0.89137 | -0.366 |
| NFXL1 | ILMN_1682197 | 0.0371 | 0.00554 | -2.91 | -2.67137 | -0.366 |
| ZMIZ2 | ILMN_1760718 | 0.0376 | 0.00564 | -2.9 | -2.68876 | -0.366 |
| QPCT | ILMN_1741727 | 0.00275 | 0.000183 | -4.07 | 0.50359 | -0.367 |
| ST3GAL6 | ILMN_1781560 | 0.0162 | 0.00186 | -3.3 | -1.67186 | -0.367 |
| ANKRD13D | ILMN_1672356 | 0.017 | 0.00198 | -3.28 | -1.72696 | -0.367 |
| GRB2 | ILMN_1742521 | 1.72E-05 | 2.34E-07 | -6.06 | 6.87055 | -0.368 |
| RGS19 | ILMN_1677085 | 7.49E-05 | 1.64E-06 | -5.5 | 4.99845 | -0.368 |
| PTK2B | ILMN_2330966 | 7.54E-05 | 1.67E-06 | -5.49 | 4.98164 | -0.368 |
| ZBTB34 | ILMN_1655206 | 0.0016 | 8.83E-05 | -4.3 | 1.19189 | -0.368 |
| CLK4 | ILMN_1695853 | 0.00623 | 0.000531 | -3.73 | -0.50139 | -0.368 |
| TADA2B | ILMN_3239378 | 0.00815 | 0.000753 | -3.61 | -0.82852 | -0.368 |
| CSNK1E | ILMN_1708858 | 0.106 | 0.023 | -2.35 | -3.94599 | -0.368 |
| SGSM2 | ILMN_1779171 | 0.000765 | 3.36E-05 | -4.6 | 2.11112 | -0.369 |
| CMTM2 | ILMN_1799030 | 0.00245 | 0.000158 | -4.12 | 0.64101 | -0.369 |
| NFKBID | ILMN_1763560 | 0.0375 | 0.00562 | -2.91 | -2.68508 | -0.369 |
| ZC3H10 | ILMN_2075847 | 0.0622 | 0.0111 | -2.64 | -3.30417 | -0.369 |
| TPM4 | ILMN_1653180 | 0.0823 | 0.0162 | -2.5 | -3.63769 | -0.369 |
| ARRDC3 | ILMN_2198516 | 0.116 | 0.0261 | -2.3 | -4.05614 | -0.369 |
| PTTG1IP | ILMN_1802251 | 0.00236 | 0.000151 | -4.13 | 0.68331 | -0.37 |
| KIAA0907 | ILMN_1670752 | 4.78E-05 | 9.27E-07 | -5.66 | 5.54757 | -0.371 |
| PAFAH1B1 | ILMN_1722276 | 0.000229 | 7.03E-06 | -5.07 | 3.60477 | -0.371 |
| SEC11A | ILMN_1693490 | 0.00603 | 0.000509 | -3.74 | -0.46145 | -0.371 |
| SH2B2 | ILMN_1669833 | 0.0151 | 0.00172 | -3.33 | -1.59626 | -0.371 |
| NUP214 | ILMN_1666049 | 0.0335 | 0.00483 | -2.96 | -2.54693 | -0.371 |
| RNPEPL1 | ILMN_1694730 | 0.0419 | 0.00649 | -2.85 | -2.8159 | -0.371 |
| ALG6 | ILMN_1771411 | 0.0562 | 0.00971 | -2.7 | -3.18019 | -0.371 |
| BCL2L1 | ILMN_1654118 | 0.117 | 0.0261 | -2.3 | -4.05807 | -0.371 |
| MAPKAPK3 | ILMN_1732452 | 0.00266 | 0.000176 | -4.08 | 0.53929 | -0.372 |
| KIAA2026 | ILMN_2106227 | 0.00988 | 0.000973 | -3.52 | -1.0672 | -0.372 |
| PLEKHA3 | ILMN_1797576 | 0.0396 | 0.00604 | -2.88 | -2.75017 | -0.372 |
| RPUSD1 | ILMN_1683082 | 0.139 | 0.0335 | -2.19 | -4.27362 | -0.372 |
| RPRD2 | ILMN_3238889 | 0.146 | 0.0357 | -2.16 | -4.32966 | -0.372 |
| PRRC2A | ILMN_1760563 | 0.000554 | 2.17E-05 | -4.73 | 2.52536 | -0.373 |
| GLUL | ILMN_1835017 | 0.00194 | 0.000115 | -4.22 | 0.93973 | -0.373 |
| PHKA2 | ILMN_1814074 | 0.00963 | 0.000936 | -3.54 | -1.03161 | -0.373 |
| KLHL28 | ILMN_1693401 | 0.0108 | 0.00111 | -3.48 | -1.18771 | -0.373 |
| KCTD11 | ILMN_1777513 | 0.0564 | 0.00977 | -2.7 | -3.1853 | -0.373 |
| LY6G5C | ILMN_1763467 | 0.0654 | 0.0119 | -2.62 | -3.36048 | -0.373 |
| CDK20 | ILMN_2357730 | 0.0824 | 0.0162 | -2.5 | -3.64006 | -0.373 |
| TARDBP | ILMN_1677532 | 0.105 | 0.0227 | -2.36 | -3.93551 | -0.373 |
| SRPK2 | ILMN_1695468 | 0.154 | 0.0387 | -2.13 | -4.39921 | -0.373 |
| DNAJB14 | ILMN_1657619 | 0.00744 | 0.000664 | -3.65 | -0.7101 | -0.374 |
| IRF3 | ILMN_1765649 | 0.0222 | 0.00282 | -3.16 | -2.05532 | -0.374 |
| CAT | ILMN_1651705 | 0.0276 | 0.00376 | -3.05 | -2.31827 | -0.374 |
| GHRL | ILMN_1696380 | 0.141 | 0.0339 | -2.19 | -4.28492 | -0.374 |
| TRIM39 | ILMN_1736562 | 7.01E-05 | 1.52E-06 | -5.52 | 5.07598 | -0.375 |
| STRADA | ILMN_2315694 | 0.001 | 4.79E-05 | -4.49 | 1.77306 | -0.375 |
| INTS4P2 | ILMN_3280667 | 0.0228 | 0.00293 | -3.14 | -2.0877 | -0.375 |
| PPM1F | ILMN_2059535 | 0.0313 | 0.00444 | -2.99 | -2.47047 | -0.375 |
| UHMK1 | ILMN_2096012 | 0.0377 | 0.00565 | -2.9 | -2.68958 | -0.375 |
| RCN3 | ILMN_1809850 | 0.0702 | 0.0131 | -2.58 | -3.45147 | -0.375 |
| FAM102B | ILMN_1786789 | 0.0704 | 0.0132 | -2.58 | -3.45594 | -0.375 |
| SIPA1 | ILMN_1682930 | 3.08E-05 | 5.12E-07 | -5.84 | 6.11812 | -0.376 |
| EXOC3 | ILMN_1789419 | 9.59E-05 | 2.30E-06 | -5.4 | 4.67583 | -0.377 |
| RNF40 | ILMN_1808712 | 0.00375 | 0.000276 | -3.94 | 0.11265 | -0.377 |
| MUS81 | ILMN_1780937 | 0.0038 | 0.00028 | -3.93 | 0.099 | -0.377 |
| KMT2C | ILMN_1736165 | 0.00574 | 0.000474 | -3.76 | -0.39561 | -0.377 |
| RUNX2 | ILMN_1716651 | 0.0544 | 0.00929 | -2.72 | -3.14068 | -0.377 |
| CLN5 | ILMN_1778203 | 0.0625 | 0.0112 | -2.64 | -3.30959 | -0.377 |
| TMEM52B | ILMN_1727606 | 0.114 | 0.0253 | -2.31 | -4.03133 | -0.377 |
| CTNND1 | ILMN_1696806 | 0.0108 | 0.0011 | -3.48 | -1.18095 | -0.378 |
| FUBP3 | ILMN_2353033 | 0.0194 | 0.00237 | -3.22 | -1.89165 | -0.378 |
| NUPL2 | ILMN_2115154 | 0.0228 | 0.00294 | -3.14 | -2.09134 | -0.378 |
| C12orf10 | ILMN_1777765 | 0.0403 | 0.00615 | -2.87 | -2.76738 | -0.378 |
| FAM89B | ILMN_1804117 | 0.0589 | 0.0104 | -2.67 | -3.23879 | -0.378 |
| TOPBP1 | ILMN_1684929 | 0.000129 | 3.27E-06 | -5.29 | 4.33718 | -0.379 |
| SKIL | ILMN_1736834 | 0.00515 | 0.000415 | -3.81 | -0.27101 | -0.379 |
| ACAP1 | ILMN_1808395 | 0.031 | 0.00437 | -3 | -2.45554 | -0.379 |
| GBF1 | ILMN_2157544 | 0.0589 | 0.0104 | -2.67 | -3.23957 | -0.379 |
| SWSAP1 | ILMN_1707943 | 0.114 | 0.0251 | -2.32 | -4.02428 | -0.379 |
| LOC153577 | ILMN_1899760 | 0.00411 | 0.00031 | -3.9 | 0.00542 | -0.38 |
| RRM2B | ILMN_1698231 | 0.00499 | 0.000398 | -3.82 | -0.2298 | -0.38 |
| PRRC2B | ILMN_3247802 | 0.0114 | 0.00118 | -3.46 | -1.2452 | -0.38 |
| DDB1 | ILMN_1775937 | 0.0135 | 0.00147 | -3.38 | -1.45282 | -0.38 |
| LOC100128398 | ILMN_3265147 | 0.0443 | 0.00701 | -2.82 | -2.88586 | -0.38 |
| AFTPH | ILMN_1767722 | 0.0622 | 0.0112 | -2.64 | -3.30462 | -0.38 |
| C2CD5 | ILMN_1682572 | 0.0921 | 0.0189 | -2.43 | -3.77247 | -0.38 |
| ZNF324 | ILMN_1745784 | 0.154 | 0.0389 | -2.13 | -4.40319 | -0.38 |
| CXXC1 | ILMN_1691276 | 0.0117 | 0.00123 | -3.45 | -1.28431 | -0.381 |
| ANKMY1 | ILMN_1675483 | 0.0158 | 0.00181 | -3.31 | -1.6426 | -0.381 |
| TOLLIP | ILMN_1765523 | 0.0177 | 0.00209 | -3.26 | -1.77686 | -0.381 |
| AP5M1 | ILMN_1741331 | 0.00025 | 7.91E-06 | -5.03 | 3.49154 | -0.382 |
| EDEM3 | ILMN_2065299 | 0.00266 | 0.000176 | -4.08 | 0.5411 | -0.382 |
| COG3 | ILMN_1776154 | 0.00306 | 0.000211 | -4.02 | 0.36582 | -0.382 |
| CLN3 | ILMN_1669281 | 0.0146 | 0.00163 | -3.35 | -1.54933 | -0.382 |
| GMCL1 | ILMN_1670532 | 0.0201 | 0.00247 | -3.2 | -1.93032 | -0.382 |
| KLHL9 | ILMN_1664466 | 0.0299 | 0.00416 | -3.02 | -2.4104 | -0.382 |
| CWC22 | ILMN_3245616 | 0.0677 | 0.0125 | -2.6 | -3.40782 | -0.382 |
| NISCH | ILMN_1735827 | 0.00195 | 0.000116 | -4.22 | 0.93524 | -0.383 |
| NOL12 | ILMN_1759991 | 0.00906 | 0.000861 | -3.57 | -0.95328 | -0.383 |
| JAZF1 | ILMN_1682727 | 0.0187 | 0.00224 | -3.24 | -1.84155 | -0.383 |
| CNOT6L | ILMN_2083833 | 0.0198 | 0.00242 | -3.21 | -1.91391 | -0.383 |
| AKT1 | ILMN_2410909 | 0.0227 | 0.0029 | -3.15 | -2.07884 | -0.383 |
| ZNF12 | ILMN_1784577 | 0.0237 | 0.0031 | -3.12 | -2.14073 | -0.383 |
| ME2 | ILMN_2048636 | 0.0299 | 0.00416 | -3.02 | -2.41157 | -0.383 |
| SIPA1 | ILMN_2415536 | 0.000228 | 6.99E-06 | -5.07 | 3.60927 | -0.384 |
| CHD3 | ILMN_2370414 | 0.00115 | 5.72E-05 | -4.43 | 1.60422 | -0.384 |
| MAP4K2 | ILMN_1723625 | 0.00479 | 0.000378 | -3.84 | -0.1811 | -0.384 |
| WDR41 | ILMN_1778488 | 0.0175 | 0.00206 | -3.27 | -1.7651 | -0.384 |
| CELF1 | ILMN_1686516 | 0.0195 | 0.00238 | -3.22 | -1.89747 | -0.384 |
| CDC42EP3 | ILMN_1736327 | 0.129 | 0.0302 | -2.24 | -4.18434 | -0.384 |
| MYO1F | ILMN_1681239 | 0.000192 | 5.56E-06 | -5.14 | 3.82969 | -0.385 |
| KIF3B | ILMN_1702279 | 0.000267 | 8.63E-06 | -5.01 | 3.40792 | -0.385 |
| LENG1 | ILMN_1679238 | 0.0054 | 0.000441 | -3.79 | -0.32641 | -0.385 |
| TXNRD1 | ILMN_1717056 | 0.0126 | 0.00135 | -3.41 | -1.36985 | -0.385 |
| SENP5 | ILMN_3251634 | 0.023 | 0.00297 | -3.14 | -2.10106 | -0.385 |
| DGKD | ILMN_1765326 | 0.0666 | 0.0122 | -2.61 | -3.38609 | -0.385 |
| ABCF1 | ILMN_2392635 | 0.0732 | 0.0139 | -2.56 | -3.50224 | -0.385 |
| VPS41 | ILMN_2280911 | 0.084 | 0.0167 | -2.48 | -3.66346 | -0.385 |
| ETFB | ILMN_2300970 | 0.156 | 0.0395 | -2.12 | -4.41669 | -0.385 |
| RABGAP1 | ILMN_2061950 | 0.000191 | 5.48E-06 | -5.14 | 3.84204 | -0.386 |
| WDR33 | ILMN_1670172 | 0.00398 | 0.000297 | -3.92 | 0.04527 | -0.386 |
| OGDH | ILMN_1733869 | 0.00484 | 0.000382 | -3.83 | -0.19292 | -0.386 |
| LFNG | ILMN_2268156 | 0.0104 | 0.00104 | -3.5 | -1.12673 | -0.386 |
| TATDN2 | ILMN_1734138 | 0.011 | 0.00112 | -3.48 | -1.20136 | -0.386 |
| SEC22A | ILMN_1775036 | 0.0282 | 0.00386 | -3.04 | -2.34189 | -0.386 |
| BRCC3 | ILMN_1697546 | 0.0674 | 0.0124 | -2.6 | -3.40195 | -0.386 |
| MILR1 | ILMN_1747347 | 0.114 | 0.0252 | -2.31 | -4.02645 | -0.386 |
| MFAP3 | ILMN_1708782 | 0.000425 | 1.55E-05 | -4.83 | 2.84611 | -0.387 |
| EHD1 | ILMN_1651832 | 0.00224 | 0.00014 | -4.15 | 0.75275 | -0.387 |
| CCDC77 | ILMN_2101375 | 0.00494 | 0.000394 | -3.82 | -0.22109 | -0.387 |
| TRAF3IP3 | ILMN_1780397 | 0.00768 | 0.000697 | -3.64 | -0.75657 | -0.387 |
| BMP2K | ILMN_1811148 | 0.0237 | 0.00309 | -3.12 | -2.1386 | -0.387 |
| OLR1 | ILMN_1723035 | 0.0264 | 0.00356 | -3.07 | -2.26812 | -0.387 |
| SCARB1 | ILMN_2183409 | 0.0274 | 0.00373 | -3.06 | -2.31114 | -0.387 |
| MARS | ILMN_1799819 | 2.15E-07 | 5.98E-10 | -7.8 | 12.61767 | -0.388 |
| MLF2 | ILMN_1671885 | 0.00375 | 0.000276 | -3.94 | 0.11282 | -0.388 |
| CRACR2B | ILMN_1745623 | 0.0101 | 0.001 | -3.51 | -1.09729 | -0.388 |
| APAF1 | ILMN_1659463 | 0.0174 | 0.00205 | -3.27 | -1.76021 | -0.388 |
| WDR6 | ILMN_1669484 | 0.0187 | 0.00224 | -3.24 | -1.84215 | -0.388 |
| DCAF10 | ILMN_3235917 | 0.0249 | 0.0033 | -3.1 | -2.19774 | -0.388 |
| LRCH4 | ILMN_1654518 | 0.0264 | 0.00356 | -3.07 | -2.26877 | -0.388 |
| CLCN6 | ILMN_1763036 | 0.0541 | 0.00923 | -2.72 | -3.13472 | -0.388 |
| VGLL4 | ILMN_1768480 | 0.0833 | 0.0165 | -2.49 | -3.65358 | -0.388 |
| LPIN1 | ILMN_1671554 | 0.101 | 0.0215 | -2.38 | -3.88715 | -0.388 |
| TGFBR2 | ILMN_2384241 | 0.00077 | 3.40E-05 | -4.59 | 2.09793 | -0.389 |
| ZFP36L1 | ILMN_1675448 | 0.0178 | 0.00211 | -3.26 | -1.78691 | -0.389 |
| JMJD8 | ILMN_3249366 | 0.0312 | 0.0044 | -3 | -2.46247 | -0.389 |
| SAP30L | ILMN_1843932 | 0.0371 | 0.00553 | -2.91 | -2.67118 | -0.389 |
| ATP2B1 | ILMN_2401933 | 0.0492 | 0.00805 | -2.77 | -3.01134 | -0.389 |
| SCARNA3 | ILMN_3244216 | 0.127 | 0.0294 | -2.25 | -4.16196 | -0.389 |
| HCK | ILMN_1791771 | 0.0315 | 0.00448 | -2.99 | -2.47861 | -0.39 |
| SEL1L | ILMN_1726496 | 0.0346 | 0.00504 | -2.95 | -2.58537 | -0.39 |
| MID1IP1 | ILMN_1668960 | 0.0502 | 0.00828 | -2.76 | -3.03691 | -0.39 |
| TRIOBP | ILMN_1753413 | 0.0558 | 0.00961 | -2.7 | -3.17071 | -0.39 |
| STK38 | ILMN_2152581 | 0.000693 | 2.95E-05 | -4.64 | 2.23327 | -0.391 |
| CMIP | ILMN_1767182 | 0.00236 | 0.000151 | -4.13 | 0.68336 | -0.391 |
| ARL11 | ILMN_1808383 | 0.0389 | 0.00589 | -2.89 | -2.7279 | -0.391 |
| PGAM4 | ILMN_1691104 | 0.0456 | 0.00729 | -2.81 | -2.92176 | -0.391 |
| PGAM1 | ILMN_1661366 | 0.0661 | 0.0121 | -2.61 | -3.37681 | -0.391 |
| ARHGAP25 | ILMN_1777998 | 9.46E-05 | 2.24E-06 | -5.41 | 4.70067 | -0.392 |
| RBM6 | ILMN_1738239 | 0.000693 | 2.95E-05 | -4.64 | 2.2353 | -0.392 |
| NAT1 | ILMN_1743055 | 0.00173 | 9.87E-05 | -4.27 | 1.08641 | -0.392 |
| EVL | ILMN_1730622 | 0.00306 | 0.000211 | -4.03 | 0.36811 | -0.392 |
| BSCL2 | ILMN_1774596 | 0.0066 | 0.00057 | -3.7 | -0.5683 | -0.392 |
| AASDHPPT | ILMN_1698189 | 0.00806 | 0.000742 | -3.62 | -0.81447 | -0.392 |
| TNFRSF10B | ILMN_2331010 | 0.0127 | 0.00136 | -3.41 | -1.37728 | -0.392 |
| CSRNP2 | ILMN_3249110 | 0.0245 | 0.00323 | -3.11 | -2.17926 | -0.392 |
| MCTP1 | ILMN_1654685 | 0.0882 | 0.0178 | -2.46 | -3.72066 | -0.392 |
| NEDD4 | ILMN_1703140 | 0.000894 | 4.12E-05 | -4.54 | 1.91659 | -0.393 |
| TTYH3 | ILMN_1692731 | 0.0362 | 0.00535 | -2.92 | -2.64057 | -0.393 |
| TGM2 | ILMN_1679267 | 0.116 | 0.026 | -2.3 | -4.05376 | -0.393 |
| IVNS1ABP | ILMN_2397750 | 0.00124 | 6.39E-05 | -4.4 | 1.49865 | -0.394 |
| ADAM8 | ILMN_1708348 | 0.0033 | 0.000234 | -3.99 | 0.2682 | -0.394 |
| CYB561D1 | ILMN_3256325 | 0.00953 | 0.000923 | -3.54 | -1.0187 | -0.394 |
| ZNF318 | ILMN_2174729 | 0.0108 | 0.00111 | -3.48 | -1.18632 | -0.394 |
| ACKR2 | ILMN_1763127 | 0.0142 | 0.00157 | -3.36 | -1.51461 | -0.394 |
| LZIC | ILMN_1661627 | 0.0155 | 0.00177 | -3.32 | -1.62511 | -0.394 |
| DGCR14 | ILMN_1754643 | 0.0163 | 0.00188 | -3.3 | -1.68038 | -0.394 |
| MRPL42 | ILMN_2356895 | 0.0189 | 0.00229 | -3.23 | -1.86094 | -0.394 |
| HERC4 | ILMN_1770665 | 0.00106 | 5.13E-05 | -4.47 | 1.70783 | -0.395 |
| TRIM28 | ILMN_1736575 | 0.00156 | 8.51E-05 | -4.31 | 1.22714 | -0.395 |
| AQR | ILMN_1717154 | 0.00517 | 0.000417 | -3.81 | -0.27429 | -0.395 |
| CLCN3 | ILMN_1660837 | 0.00949 | 0.000916 | -3.55 | -1.01112 | -0.395 |
| CERK | ILMN_1721325 | 0.0332 | 0.00477 | -2.97 | -2.53581 | -0.395 |
| PRRC2A | ILMN_2408179 | 0.0362 | 0.00534 | -2.92 | -2.63922 | -0.395 |
| GRK2 | ILMN_3307926 | 1.68E-05 | 2.25E-07 | -6.07 | 6.90808 | -0.396 |
| PAGR1 | ILMN_2194828 | 0.00479 | 0.000378 | -3.84 | -0.18213 | -0.396 |
| AKT1 | ILMN_2388507 | 0.0132 | 0.00143 | -3.39 | -1.42657 | -0.396 |
| SPTAN1 | ILMN_2095133 | 0.0419 | 0.00648 | -2.85 | -2.814 | -0.396 |
| CD302 | ILMN_2070940 | 0.125 | 0.0289 | -2.26 | -4.14518 | -0.396 |
| E2F6 | ILMN_1656196 | 0.138 | 0.0329 | -2.2 | -4.25867 | -0.396 |
| LTN1 | ILMN_1760360 | 4.99E-06 | 4.18E-08 | -6.56 | 8.52967 | -0.397 |
| ZSCAN32 | ILMN_1684591 | 0.00115 | 5.73E-05 | -4.43 | 1.60161 | -0.397 |
| UPF2 | ILMN_1739283 | 0.00562 | 0.000463 | -3.77 | -0.37233 | -0.397 |
| FAM111A | ILMN_1778845 | 0.123 | 0.0282 | -2.27 | -4.12355 | -0.397 |
| CRK | ILMN_1686805 | 1.12E-06 | 4.76E-09 | -7.19 | 10.62239 | -0.398 |
| SH3GL1 | ILMN_1788062 | 0.000587 | 2.36E-05 | -4.71 | 2.4489 | -0.398 |
| MID1IP1 | ILMN_2165473 | 0.0432 | 0.00677 | -2.84 | -2.85446 | -0.398 |
| BLCAP | ILMN_1675612 | 0.0437 | 0.00687 | -2.83 | -2.86774 | -0.398 |
| ARHGAP9 | ILMN_2382657 | 0.000101 | 2.46E-06 | -5.38 | 4.61136 | -0.399 |
| NOTCH1 | ILMN_1729161 | 0.000575 | 2.28E-05 | -4.72 | 2.48145 | -0.399 |
| CBL | ILMN_2181968 | 0.00147 | 7.87E-05 | -4.34 | 1.30021 | -0.399 |
| PTCD1 | ILMN_2225709 | 0.00889 | 0.000842 | -3.57 | -0.93249 | -0.399 |
| SFI1 | ILMN_1763887 | 0.105 | 0.0227 | -2.36 | -3.93512 | -0.399 |
| RAD9A | ILMN_1684183 | 0.0256 | 0.00342 | -3.09 | -2.2313 | -0.4 |
| GRIPAP1 | ILMN_1719857 | 0.0526 | 0.00886 | -2.73 | -3.09742 | -0.4 |
| TCHP | ILMN_1665058 | 0.0605 | 0.0107 | -2.66 | -3.26992 | -0.4 |
| ZSWIM6 | ILMN_1777061 | 5.95E-05 | 1.24E-06 | -5.58 | 5.26851 | -0.401 |
| SH3KBP1 | ILMN_1810782 | 0.00105 | 5.11E-05 | -4.47 | 1.71113 | -0.401 |
| EMP3 | ILMN_1765446 | 0.00758 | 0.000684 | -3.64 | -0.73855 | -0.401 |
| ZNF788 | ILMN_3305466 | 0.0227 | 0.0029 | -3.15 | -2.07971 | -0.401 |
| CTNS | ILMN_1807719 | 0.0391 | 0.00594 | -2.88 | -2.73601 | -0.401 |
| PPP2R5D | ILMN_1780940 | 0.0394 | 0.006 | -2.88 | -2.74519 | -0.401 |
| SHC1 | ILMN_1721022 | 7.93E-08 | 1.72E-10 | -8.16 | 13.81717 | -0.402 |
| EFNA4 | ILMN_1755710 | 0.000345 | 1.18E-05 | -4.91 | 3.1054 | -0.402 |
| CTSC | ILMN_1696347 | 0.00257 | 0.000168 | -4.1 | 0.58171 | -0.402 |
| SLC30A5 | ILMN_1664153 | 0.00949 | 0.000915 | -3.55 | -1.01057 | -0.402 |
| AKAP8L | ILMN_1768962 | 0.0109 | 0.00112 | -3.48 | -1.1947 | -0.402 |
| ZNF282 | ILMN_1668185 | 0.0351 | 0.00515 | -2.94 | -2.60489 | -0.402 |
| TBCC | ILMN_1743352 | 0.0529 | 0.00896 | -2.73 | -3.10716 | -0.402 |
| CCT5 | ILMN_1706246 | 0.00266 | 0.000175 | -4.08 | 0.54422 | -0.403 |
| LTA | ILMN_1795464 | 0.0115 | 0.00119 | -3.46 | -1.25592 | -0.403 |
| FAM168A | ILMN_1847308 | 0.0139 | 0.00153 | -3.37 | -1.48633 | -0.403 |
| ATP2B1 | ILMN_1813399 | 0.0151 | 0.00171 | -3.33 | -1.5894 | -0.403 |
| TMEM79 | ILMN_2090802 | 0.0382 | 0.00575 | -2.9 | -2.70536 | -0.403 |
| PTTG1IP | ILMN_2128750 | 8.80E-05 | 2.03E-06 | -5.43 | 4.79486 | -0.404 |
| ZYX | ILMN_2371169 | 0.00124 | 6.36E-05 | -4.4 | 1.50377 | -0.404 |
| BMP2K | ILMN_2299045 | 0.0127 | 0.00137 | -3.41 | -1.38379 | -0.404 |
| RAB24 | ILMN_1677843 | 0.0154 | 0.00175 | -3.32 | -1.61389 | -0.404 |
| SLC25A29 | ILMN_2350801 | 0.0656 | 0.0119 | -2.62 | -3.36461 | -0.404 |
| RSPRY1 | ILMN_1763694 | 4.78E-06 | 3.87E-08 | -6.58 | 8.60456 | -0.405 |
| WDR82 | ILMN_1679655 | 0.00136 | 7.15E-05 | -4.37 | 1.39184 | -0.405 |
| LRTOMT | ILMN_3237645 | 0.00202 | 0.000123 | -4.2 | 0.87949 | -0.405 |
| F2RL1 | ILMN_2041190 | 0.00771 | 0.000702 | -3.63 | -0.76236 | -0.405 |
| ACOX1 | ILMN_1765767 | 0.01 | 0.000994 | -3.52 | -1.08744 | -0.405 |
| OGG1 | ILMN_2352609 | 0.0137 | 0.0015 | -3.38 | -1.46826 | -0.405 |
| PAGR1 | ILMN_1727444 | 0.0343 | 0.00499 | -2.95 | -2.57751 | -0.405 |
| ZNF362 | ILMN_1686968 | 0.107 | 0.0233 | -2.35 | -3.95691 | -0.405 |
| NSF | ILMN_1680353 | 6.52E-05 | 1.39E-06 | -5.55 | 5.15851 | -0.406 |
| TOR4A | ILMN_1813641 | 0.00914 | 0.000872 | -3.56 | -0.96479 | -0.406 |
| SH3BP2 | ILMN_1801914 | 0.0117 | 0.00122 | -3.45 | -1.27466 | -0.406 |
| STIM1 | ILMN_2064694 | 0.0225 | 0.00287 | -3.15 | -2.06987 | -0.406 |
| EFNA4 | ILMN_1665696 | 0.000412 | 1.49E-05 | -4.84 | 2.88601 | -0.407 |
| ALCAM | ILMN_1670870 | 0.00238 | 0.000152 | -4.13 | 0.67494 | -0.407 |
| ZNF439 | ILMN_1792972 | 0.00875 | 0.000822 | -3.58 | -0.91074 | -0.407 |
| BANF1 | ILMN_2179837 | 0.0253 | 0.00338 | -3.09 | -2.22046 | -0.407 |
| SERPINE1 | ILMN_1744381 | 0.0512 | 0.00853 | -2.75 | -3.06323 | -0.407 |
| KDM3B | ILMN_1706539 | 5.46E-06 | 4.81E-08 | -6.52 | 8.39542 | -0.408 |
| ZNF467 | ILMN_1779015 | 0.000246 | 7.73E-06 | -5.04 | 3.51298 | -0.408 |
| CASP8 | ILMN_1787749 | 0.00092 | 4.28E-05 | -4.52 | 1.87981 | -0.408 |
| KRIT1 | ILMN_2323418 | 0.00999 | 0.000986 | -3.52 | -1.08014 | -0.408 |
| SMG7 | ILMN_2368597 | 0.0174 | 0.00205 | -3.27 | -1.75902 | -0.408 |
| METTL13 | ILMN_2343624 | 0.0313 | 0.00442 | -2.99 | -2.46653 | -0.408 |
| GALNT4 | ILMN_1739297 | 0.0548 | 0.0094 | -2.71 | -3.15094 | -0.408 |
| FRAT2 | ILMN_1788213 | 0.000197 | 5.77E-06 | -5.13 | 3.79355 | -0.409 |
| ARID3A | ILMN_1670130 | 0.00023 | 7.08E-06 | -5.07 | 3.59808 | -0.409 |
| NUDCD3 | ILMN_1796900 | 0.00307 | 0.000212 | -4.02 | 0.36239 | -0.409 |
| TSPO | ILMN_1681679 | 0.00431 | 0.000331 | -3.88 | -0.05684 | -0.409 |
| CSRNP2 | ILMN_2187533 | 0.0328 | 0.00471 | -2.97 | -2.52363 | -0.409 |
| KLF3 | ILMN_1670245 | 0.039 | 0.00592 | -2.89 | -2.73224 | -0.409 |
| CRTC3 | ILMN_3260715 | 0.0563 | 0.00975 | -2.7 | -3.18374 | -0.409 |
| PSG3 | ILMN_1685312 | 0.109 | 0.0236 | -2.34 | -3.97031 | -0.409 |
| HERC4 | ILMN_2370160 | 0.00226 | 0.000143 | -4.15 | 0.73585 | -0.41 |
| MATK | ILMN_2319000 | 0.00262 | 0.000172 | -4.09 | 0.55956 | -0.41 |
| RBM14 | ILMN_1700604 | 0.00368 | 0.000267 | -3.95 | 0.14368 | -0.41 |
| RPS19BP1 | ILMN_2177965 | 0.00505 | 0.000406 | -3.81 | -0.24856 | -0.41 |
| PPWD1 | ILMN_2223380 | 0.00745 | 0.000667 | -3.65 | -0.71416 | -0.41 |
| SLC39A1 | ILMN_2116714 | 0.0162 | 0.00187 | -3.3 | -1.67572 | -0.41 |
| WNK1 | ILMN_1788711 | 0.0309 | 0.00436 | -3 | -2.45407 | -0.41 |
| LGALS12 | ILMN_1776283 | 0.0406 | 0.00622 | -2.87 | -2.77802 | -0.41 |
| ATXN2 | ILMN_1743829 | 7.75E-05 | 1.74E-06 | -5.48 | 4.94537 | -0.411 |
| AOAH | ILMN_1709820 | 0.0659 | 0.012 | -2.61 | -3.37319 | -0.411 |
| GATAD2A | ILMN_1673682 | 5.56E-05 | 1.13E-06 | -5.6 | 5.35416 | -0.412 |
| C7orf26 | ILMN_1730048 | 0.00118 | 5.98E-05 | -4.42 | 1.56102 | -0.412 |
| CBL | ILMN_1716080 | 0.00174 | 9.95E-05 | -4.26 | 1.07814 | -0.412 |
| MAML1 | ILMN_1803060 | 0.00551 | 0.000451 | -3.78 | -0.34768 | -0.412 |
| ZCCHC24 | ILMN_1754660 | 0.00629 | 0.000537 | -3.72 | -0.5119 | -0.412 |
| FNBP1 | ILMN_1797342 | 0.00702 | 0.000616 | -3.68 | -0.64077 | -0.412 |
| DLGAP4 | ILMN_1754842 | 0.0073 | 0.00065 | -3.66 | -0.69011 | -0.412 |
| ME2 | ILMN_1675186 | 0.015 | 0.00169 | -3.34 | -1.57955 | -0.412 |
| LYSMD2 | ILMN_1724493 | 0.0231 | 0.00298 | -3.14 | -2.10525 | -0.412 |
| GPR141 | ILMN_1656818 | 0.0232 | 0.00301 | -3.13 | -2.11279 | -0.412 |
| QTRT2 | ILMN_2155516 | 0.0244 | 0.00322 | -3.11 | -2.17498 | -0.412 |
| KIAA0319 | ILMN_1657497 | 0.0506 | 0.00837 | -2.76 | -3.0457 | -0.412 |
| UBASH3B | ILMN_1653856 | 0.173 | 0.0457 | -2.05 | -4.54122 | -0.412 |
| LTC4S | ILMN_1668247 | 0.00842 | 0.000785 | -3.6 | -0.86738 | -0.413 |
| CALML4 | ILMN_1757210 | 0.00908 | 0.000865 | -3.56 | -0.95745 | -0.413 |
| NUP133 | ILMN_1751338 | 0.0117 | 0.00122 | -3.45 | -1.28138 | -0.413 |
| ZFYVE19 | ILMN_1780698 | 0.0133 | 0.00145 | -3.39 | -1.43899 | -0.413 |
| SPSB2 | ILMN_1787541 | 0.0401 | 0.00613 | -2.87 | -2.76404 | -0.413 |
| USP24 | ILMN_1684594 | 0.0479 | 0.00778 | -2.78 | -2.97963 | -0.413 |
| TSPYL2 | ILMN_1657554 | 0.0548 | 0.0094 | -2.71 | -3.15068 | -0.413 |
| PPP1R21 | ILMN_3271092 | 7.53E-08 | 1.56E-10 | -8.19 | 13.90969 | -0.414 |
| HCP5 | ILMN_1803945 | 2.62E-05 | 4.22E-07 | -5.89 | 6.30372 | -0.414 |
| SP3 | ILMN_1756942 | 0.000101 | 2.43E-06 | -5.38 | 4.6207 | -0.414 |
| PTPN6 | ILMN_1738675 | 0.000216 | 6.51E-06 | -5.09 | 3.67797 | -0.414 |
| HPSE | ILMN_1779547 | 0.00029 | 9.58E-06 | -4.98 | 3.30856 | -0.414 |
| EPRS | ILMN_1783695 | 0.0043 | 0.000329 | -3.88 | -0.05182 | -0.414 |
| NAT10 | ILMN_1705594 | 0.00729 | 0.000648 | -3.66 | -0.68785 | -0.414 |
| PWWP2A | ILMN_1771403 | 0.0104 | 0.00104 | -3.5 | -1.1314 | -0.414 |
| RANBP3 | ILMN_1723689 | 0.0189 | 0.00228 | -3.23 | -1.85657 | -0.414 |
| MIF4GD | ILMN_1743806 | 0.0456 | 0.00731 | -2.81 | -2.92386 | -0.414 |
| ABLIM1 | ILMN_1785424 | 0.0761 | 0.0146 | -2.54 | -3.54774 | -0.414 |
| PGLYRP1 | ILMN_1704870 | 0.151 | 0.0376 | -2.14 | -4.3739 | -0.414 |
| TRIP11 | ILMN_1658144 | 6.00E-05 | 1.25E-06 | -5.58 | 5.25808 | -0.415 |
| PRDX5 | ILMN_2383975 | 0.00307 | 0.000212 | -4.02 | 0.36091 | -0.415 |
| CTSC | ILMN_2242463 | 0.0112 | 0.00116 | -3.47 | -1.22734 | -0.415 |
| ATM | ILMN_2370825 | 0.0622 | 0.0111 | -2.65 | -3.30316 | -0.415 |
| ARHGAP27 | ILMN_2103362 | 1.08E-05 | 1.19E-07 | -6.26 | 7.52327 | -0.416 |
| CPVL | ILMN_2400759 | 0.00413 | 0.000311 | -3.9 | 0.00102 | -0.416 |
| PPP1CA | ILMN_1695827 | 0.00766 | 0.000695 | -3.64 | -0.75358 | -0.416 |
| EIF2B1 | ILMN_1753716 | 0.0185 | 0.00221 | -3.24 | -1.82961 | -0.416 |
| SLC7A6OS | ILMN_1692049 | 0.00129 | 6.72E-05 | -4.38 | 1.45033 | -0.417 |
| OSBPL8 | ILMN_2405078 | 0.00157 | 8.55E-05 | -4.31 | 1.22272 | -0.417 |
| ACTG1 | ILMN_1704961 | 0.00226 | 0.000142 | -4.15 | 0.74044 | -0.417 |
| GMCL1 | ILMN_2194627 | 0.0103 | 0.00103 | -3.5 | -1.12198 | -0.417 |
| MEF2D | ILMN_1763228 | 0.0318 | 0.00454 | -2.98 | -2.48956 | -0.417 |
| AMMECR1L | ILMN_1727023 | 0.00965 | 0.000939 | -3.54 | -1.03391 | -0.418 |
| SMAP1 | ILMN_1768271 | 0.0165 | 0.00192 | -3.29 | -1.69793 | -0.418 |
| VEGFB | ILMN_1722855 | 0.101 | 0.0215 | -2.38 | -3.88803 | -0.418 |
| TXNDC12 | ILMN_1783753 | 0.0211 | 0.00264 | -3.18 | -1.9917 | -0.419 |
| VSIG8 | ILMN_1730740 | 0.147 | 0.0361 | -2.16 | -4.33901 | -0.419 |
| MCM3 | ILMN_2224143 | 0.00013 | 3.31E-06 | -5.29 | 4.32478 | -0.42 |
| PTAR1 | ILMN_3237679 | 0.000145 | 3.78E-06 | -5.25 | 4.19946 | -0.42 |
| CTSZ | ILMN_1666269 | 0.000232 | 7.15E-06 | -5.06 | 3.58791 | -0.42 |
| FBXO3 | ILMN_1749641 | 0.00197 | 0.000118 | -4.21 | 0.91726 | -0.42 |
| ZNF816 | ILMN_1728710 | 0.00502 | 4.00E-04 | -3.82 | -0.2363 | -0.42 |
| RELL2 | ILMN_1652540 | 0.0339 | 0.00493 | -2.95 | -2.56552 | -0.42 |
| EPB41 | ILMN_2323427 | 0.0691 | 0.0129 | -2.59 | -3.43301 | -0.42 |
| SULT1A2 | ILMN_2400372 | 0.0886 | 0.0179 | -2.46 | -3.72575 | -0.42 |
| PLD3 | ILMN_1666976 | 0.000625 | 2.58E-05 | -4.68 | 2.36333 | -0.421 |
| SLC16A5 | ILMN_1755649 | 0.00765 | 0.000694 | -3.64 | -0.75166 | -0.421 |
| CYP27A1 | ILMN_1704985 | 0.0122 | 0.00128 | -3.43 | -1.32572 | -0.421 |
| RAB22A | ILMN_1786976 | 3.80E-05 | 6.71E-07 | -5.76 | 5.85898 | -0.422 |
| GNG2 | ILMN_1807925 | 0.00208 | 0.000127 | -4.19 | 0.84988 | -0.422 |
| ANKRD49 | ILMN_1669259 | 0.004 | 0.000298 | -3.91 | 0.04013 | -0.422 |
| OPRL1 | ILMN_2400926 | 0.0149 | 0.00167 | -3.34 | -1.57008 | -0.422 |
| CYTH4 | ILMN_3238196 | 0.0187 | 0.00224 | -3.24 | -1.83973 | -0.422 |
| CASP3 | ILMN_2388155 | 1.32E-05 | 1.56E-07 | -6.18 | 7.2598 | -0.423 |
| AP1S2 | ILMN_1766411 | 0.000225 | 6.88E-06 | -5.07 | 3.62517 | -0.423 |
| CHAMP1 | ILMN_1729976 | 0.00974 | 0.000953 | -3.53 | -1.0483 | -0.423 |
| LOC146880 | ILMN_1659110 | 0.0197 | 0.00241 | -3.21 | -1.908 | -0.423 |
| S100A6 | ILMN_1713636 | 0.000552 | 2.15E-05 | -4.73 | 2.53535 | -0.424 |
| TGFBR2 | ILMN_1726245 | 0.00104 | 5.04E-05 | -4.47 | 1.7249 | -0.424 |
| KIAA1429 | ILMN_1813635 | 0.00225 | 0.000142 | -4.15 | 0.74455 | -0.424 |
| CDK14 | ILMN_2171295 | 0.0224 | 0.00286 | -3.15 | -2.0657 | -0.424 |
| KCNAB2 | ILMN_1725496 | 0.0286 | 0.00394 | -3.04 | -2.36107 | -0.424 |
| TBC1D10A | ILMN_1693726 | 0.0336 | 0.00486 | -2.96 | -2.55185 | -0.424 |
| FAAP100 | ILMN_1711823 | 0.105 | 0.0225 | -2.36 | -3.92852 | -0.424 |
| PHF2 | ILMN_1720476 | 0.00101 | 4.85E-05 | -4.49 | 1.7617 | -0.425 |
| FPR3 | ILMN_2203271 | 0.00579 | 0.000482 | -3.76 | -0.41067 | -0.425 |
| SPNS3 | ILMN_1668984 | 0.0426 | 0.00664 | -2.84 | -2.83649 | -0.425 |
| WRB | ILMN_1695092 | 0.116 | 0.0259 | -2.3 | -4.05108 | -0.425 |
| FCN1 | ILMN_1668063 | 1.39E-06 | 6.43E-09 | -7.1 | 10.3322 | -0.426 |
| MAD2L1BP | ILMN_1798454 | 4.04E-05 | 7.28E-07 | -5.73 | 5.77948 | -0.426 |
| RNPEP | ILMN_1738347 | 0.0405 | 0.0062 | -2.87 | -2.77406 | -0.426 |
| ARPC1B | ILMN_2085760 | 0.000238 | 7.43E-06 | -5.05 | 3.55179 | -0.427 |
| FAM199X | ILMN_1811121 | 0.00126 | 6.46E-05 | -4.4 | 1.48854 | -0.427 |
| IL6R | ILMN_1696394 | 0.00174 | 9.99E-05 | -4.26 | 1.07417 | -0.427 |
| SLC40A1 | ILMN_1761833 | 0.0603 | 0.0107 | -2.66 | -3.26666 | -0.427 |
| PPP1R21 | ILMN_1667356 | 1.19E-06 | 5.16E-09 | -7.17 | 10.54369 | -0.428 |
| MSL2 | ILMN_3236373 | 0.00184 | 0.000107 | -4.24 | 1.00768 | -0.428 |
| KCNE3 | ILMN_1789387 | 0.0105 | 0.00106 | -3.5 | -1.14859 | -0.428 |
| SSBP3 | ILMN_2361163 | 0.102 | 0.0217 | -2.38 | -3.89763 | -0.428 |
| GAPVD1 | ILMN_1671221 | 0.000144 | 3.71E-06 | -5.26 | 4.21589 | -0.429 |
| DUSP18 | ILMN_1734288 | 0.00476 | 0.000374 | -3.84 | -0.17198 | -0.429 |
| LTN1 | ILMN_3243686 | 0.015 | 0.0017 | -3.33 | -1.58406 | -0.429 |
| LGALS9 | ILMN_1715760 | 0.022 | 0.00279 | -3.16 | -2.04323 | -0.429 |
| BTK | ILMN_1662026 | 0.000194 | 5.64E-06 | -5.13 | 3.81505 | -0.43 |
| PTGES2 | ILMN_2345015 | 0.00161 | 8.91E-05 | -4.3 | 1.18307 | -0.43 |
| CYTH4 | ILMN_1660364 | 0.00464 | 0.000362 | -3.85 | -0.14178 | -0.43 |
| EFHD2 | ILMN_1761463 | 5.53E-05 | 1.11E-06 | -5.61 | 5.37088 | -0.431 |
| PGD | ILMN_1794165 | 0.000434 | 1.59E-05 | -4.82 | 2.82245 | -0.431 |
| MAPKAP1 | ILMN_2360229 | 0.00047 | 1.78E-05 | -4.79 | 2.71571 | -0.431 |
| ABCC5 | ILMN_1651964 | 0.12 | 0.0272 | -2.28 | -4.09328 | -0.431 |
| AP1S2 | ILMN_2120273 | 8.80E-05 | 2.03E-06 | -5.43 | 4.7934 | -0.432 |
| TMEM184B | ILMN_1747460 | 9.59E-05 | 2.28E-06 | -5.4 | 4.68316 | -0.432 |
| RNF25 | ILMN_1713486 | 0.00819 | 0.000759 | -3.61 | -0.83528 | -0.432 |
| UBE3C | ILMN_1704342 | 0.00615 | 0.000522 | -3.73 | -0.48545 | -0.433 |
| ZBTB24 | ILMN_1659937 | 0.0173 | 0.00204 | -3.27 | -1.75435 | -0.433 |
| STK35 | ILMN_1727809 | 0.0442 | 0.00699 | -2.82 | -2.88287 | -0.433 |
| COQ8A | ILMN_1731064 | 0.0831 | 0.0165 | -2.49 | -3.6512 | -0.433 |
| NRROS | ILMN_1746148 | 0.0874 | 0.0176 | -2.46 | -3.70888 | -0.433 |
| AKNA | ILMN_1770673 | 1.64E-05 | 2.18E-07 | -6.08 | 6.93987 | -0.434 |
| SLTM | ILMN_1718815 | 0.00827 | 0.000767 | -3.6 | -0.84584 | -0.434 |
| PGAM1 | ILMN_2112417 | 0.0393 | 0.00598 | -2.88 | -2.74114 | -0.434 |
| CCT3 | ILMN_2334989 | 0.0126 | 0.00134 | -3.42 | -1.36612 | -0.435 |
| NIFK | ILMN_1786189 | 0.0132 | 0.00144 | -3.39 | -1.42988 | -0.435 |
| DGKA | ILMN_2319910 | 0.0177 | 0.00209 | -3.26 | -1.77952 | -0.435 |
| KDM2B | ILMN_1794260 | 0.0197 | 0.0024 | -3.21 | -1.90554 | -0.435 |
| FIBP | ILMN_1657797 | 0.0306 | 0.00429 | -3.01 | -2.43873 | -0.435 |
| NR3C1 | ILMN_2389347 | 0.00197 | 0.000118 | -4.21 | 0.91888 | -0.436 |
| RAC1 | ILMN_2359789 | 1.03E-07 | 2.44E-10 | -8.06 | 13.48177 | -0.437 |
| MNT | ILMN_1792910 | 6.25E-06 | 5.89E-08 | -6.46 | 8.20014 | -0.437 |
| NEDD4 | ILMN_1807881 | 0.000155 | 4.14E-06 | -5.22 | 4.11204 | -0.437 |
| NKTR | ILMN_1721621 | 0.00627 | 0.000535 | -3.72 | -0.5082 | -0.437 |
| GIN1 | ILMN_1814622 | 0.0148 | 0.00165 | -3.34 | -1.55943 | -0.437 |
| RASGRP2 | ILMN_1794594 | 0.0261 | 0.00351 | -3.08 | -2.255 | -0.437 |
| NAPRT | ILMN_1710752 | 0.000199 | 5.85E-06 | -5.12 | 3.7796 | -0.438 |
| TUBB | ILMN_2101885 | 0.0039 | 0.000289 | -3.92 | 0.07122 | -0.438 |
| SKIV2L2 | ILMN_1651513 | 0.0117 | 0.00122 | -3.45 | -1.28176 | -0.438 |
| LOC100288893 | ILMN_1845157 | 0.0154 | 0.00175 | -3.32 | -1.61141 | -0.438 |
| STARD7 | ILMN_1740819 | 0.0243 | 0.00321 | -3.11 | -2.1723 | -0.438 |
| RGL2 | ILMN_2124386 | 0.000126 | 3.18E-06 | -5.3 | 4.36573 | -0.439 |
| GCC2 | ILMN_1678831 | 0.0118 | 0.00124 | -3.44 | -1.29182 | -0.439 |
| NBPF14 | ILMN_1698067 | 0.0585 | 0.0103 | -2.68 | -3.23023 | -0.439 |
| GPR132 | ILMN_1811171 | 0.0663 | 0.0121 | -2.61 | -3.3809 | -0.439 |
| NADK | ILMN_1758963 | 6.30E-05 | 1.32E-06 | -5.56 | 5.20716 | -0.44 |
| CYP4F12 | ILMN_1809384 | 0.000357 | 1.24E-05 | -4.9 | 3.05986 | -0.44 |
| TRIM24 | ILMN_2405031 | 0.000707 | 3.03E-05 | -4.63 | 2.20869 | -0.44 |
| ZNF615 | ILMN_1672135 | 0.00159 | 8.75E-05 | -4.3 | 1.20021 | -0.44 |
| PBX3 | ILMN_1810100 | 0.00241 | 0.000155 | -4.12 | 0.65571 | -0.44 |
| CMTM1 | ILMN_2328363 | 0.0659 | 0.012 | -2.62 | -3.37125 | -0.44 |
| TMEM138 | ILMN_1664761 | 0.00995 | 0.000981 | -3.52 | -1.0751 | -0.441 |
| PPP1R16B | ILMN_1727098 | 0.018 | 0.00214 | -3.25 | -1.80144 | -0.441 |
| NEK6 | ILMN_1660871 | 0.0454 | 0.00724 | -2.81 | -2.91539 | -0.441 |
| LPIN2 | ILMN_1670028 | 2.04E-05 | 2.96E-07 | -6 | 6.64447 | -0.442 |
| FAM220A | ILMN_1717219 | 0.00483 | 0.000381 | -3.83 | -0.1907 | -0.442 |
| STARD3NL | ILMN_2228873 | 0.013 | 0.0014 | -3.4 | -1.40919 | -0.442 |
| NBPF9 | ILMN_2115490 | 0.0194 | 0.00235 | -3.22 | -1.88621 | -0.442 |
| SULT1A1 | ILMN_1656900 | 0.0356 | 0.00523 | -2.93 | -2.6191 | -0.442 |
| STT3A | ILMN_1746090 | 0.00323 | 0.000227 | -4 | 0.2993 | -0.443 |
| FBXL18 | ILMN_3307782 | 0.00646 | 0.000555 | -3.71 | -0.54314 | -0.443 |
| MCMBP | ILMN_1761411 | 0.0111 | 0.00114 | -3.47 | -1.21725 | -0.443 |
| CLDN11 | ILMN_1754103 | 0.0126 | 0.00135 | -3.41 | -1.37124 | -0.443 |
| TP53RK | ILMN_1764851 | 0.0236 | 0.00306 | -3.13 | -2.13014 | -0.443 |
| MGAT4B | ILMN_1796106 | 0.0577 | 0.0101 | -2.68 | -3.21405 | -0.443 |
| BRICD5 | ILMN_1759411 | 0.0823 | 0.0162 | -2.5 | -3.63835 | -0.443 |
| QRICH1 | ILMN_1676002 | 9.39E-05 | 2.22E-06 | -5.41 | 4.71103 | -0.444 |
| CS | ILMN_2396410 | 0.00467 | 0.000366 | -3.85 | -0.1507 | -0.444 |
| FBXL20 | ILMN_1754489 | 0.0528 | 0.00892 | -2.73 | -3.10317 | -0.444 |
| TANC2 | ILMN_3244286 | 0.0548 | 0.00939 | -2.71 | -3.15025 | -0.444 |
| CSRP1 | ILMN_1811921 | 0.0589 | 0.0104 | -2.67 | -3.23983 | -0.444 |
| SCAF4 | ILMN_1659874 | 4.26E-05 | 7.86E-07 | -5.71 | 5.70696 | -0.445 |
| TCF20 | ILMN_2368068 | 0.000554 | 2.16E-05 | -4.73 | 2.52991 | -0.445 |
| ITGAL | ILMN_1749591 | 0.00336 | 0.00024 | -3.98 | 0.24543 | -0.445 |
| PRPSAP1 | ILMN_1768449 | 0.00524 | 0.000425 | -3.8 | -0.29126 | -0.445 |
| SIGLEC5 | ILMN_1652381 | 0.024 | 0.00314 | -3.12 | -2.15255 | -0.445 |
| ARHGAP30 | ILMN_2408851 | 0.000174 | 4.84E-06 | -5.18 | 3.96097 | -0.446 |
| GTF2E1 | ILMN_1655921 | 0.0104 | 0.00105 | -3.5 | -1.13567 | -0.446 |
| NOA1 | ILMN_1665066 | 0.0145 | 0.00161 | -3.35 | -1.53649 | -0.446 |
| LRRC25 | ILMN_2150196 | 1.64E-05 | 2.15E-07 | -6.09 | 6.95229 | -0.447 |
| SLC19A1 | ILMN_1698996 | 0.00423 | 0.000322 | -3.89 | -0.0324 | -0.447 |
| RER1 | ILMN_3251317 | 0.00477 | 0.000375 | -3.84 | -0.17487 | -0.447 |
| PRDX5 | ILMN_1815024 | 0.00682 | 0.000594 | -3.69 | -0.60598 | -0.447 |
| TRIM39 | ILMN_2413517 | 0.0105 | 0.00105 | -3.5 | -1.14253 | -0.447 |
| EBNA1BP2 | ILMN_1768127 | 0.0109 | 0.00111 | -3.48 | -1.19194 | -0.447 |
| PIK3R6 | ILMN_3246957 | 0.0132 | 0.00142 | -3.4 | -1.42155 | -0.447 |
| PRELID1 | ILMN_2179652 | 0.0509 | 0.00844 | -2.75 | -3.05352 | -0.447 |
| STX6 | ILMN_1777915 | 4.12E-05 | 7.45E-07 | -5.73 | 5.75814 | -0.448 |
| DSTYK | ILMN_3180989 | 0.00971 | 0.000948 | -3.53 | -1.0432 | -0.448 |
| TRRAP | ILMN_1660368 | 2.52E-05 | 3.99E-07 | -5.91 | 6.35791 | -0.449 |
| SGK1 | ILMN_1702487 | 0.000693 | 2.94E-05 | -4.64 | 2.23613 | -0.449 |
| SNUPN | ILMN_1733932 | 0.00121 | 6.19E-05 | -4.41 | 1.52943 | -0.449 |
| GOLGA3 | ILMN_1733511 | 0.00038 | 1.35E-05 | -4.87 | 2.98139 | -0.45 |
| MTF1 | ILMN_1763828 | 0.00059 | 2.37E-05 | -4.7 | 2.4424 | -0.45 |
| RABL6 | ILMN_1760628 | 0.0011 | 5.39E-05 | -4.45 | 1.65987 | -0.45 |
| TNNI2 | ILMN_2169261 | 0.00373 | 0.000274 | -3.94 | 0.12222 | -0.45 |
| ZNF318 | ILMN_1792305 | 0.00639 | 0.000548 | -3.72 | -0.53048 | -0.45 |
| PRAM1 | ILMN_1711493 | 0.02 | 0.00246 | -3.21 | -1.92694 | -0.45 |
| CYFIP2 | ILMN_2354478 | 0.0252 | 0.00336 | -3.09 | -2.21501 | -0.451 |
| ITGB2 | ILMN_1654396 | 1.35E-05 | 1.62E-07 | -6.17 | 7.22719 | -0.452 |
| CLDN15 | ILMN_1682226 | 5.45E-05 | 1.09E-06 | -5.62 | 5.39173 | -0.452 |
| KPNA6 | ILMN_1696021 | 0.000274 | 8.91E-06 | -5 | 3.37802 | -0.452 |
| ARHGAP30 | ILMN_1751164 | 0.000306 | 1.02E-05 | -4.96 | 3.25134 | -0.452 |
| MBD1 | ILMN_1683595 | 0.000928 | 4.32E-05 | -4.52 | 1.86983 | -0.452 |
| ZNF189 | ILMN_1806809 | 0.00141 | 7.47E-05 | -4.35 | 1.34977 | -0.452 |
| TNRC6A | ILMN_1714622 | 0.00634 | 0.000542 | -3.72 | -0.52064 | -0.452 |
| PGAM4 | ILMN_1682953 | 0.0426 | 0.00665 | -2.84 | -2.83844 | -0.452 |
| CORO1A | ILMN_1713749 | 0.000547 | 2.13E-05 | -4.74 | 2.54511 | -0.453 |
| C1orf189 | ILMN_1790713 | 0.00118 | 5.97E-05 | -4.42 | 1.56316 | -0.453 |
| CPSF2 | ILMN_2144116 | 0.00297 | 0.000202 | -4.04 | 0.40689 | -0.453 |
| SSBP2 | ILMN_1711608 | 0.0159 | 0.00182 | -3.31 | -1.65187 | -0.453 |
| KIF23 | ILMN_1811472 | 0.0614 | 0.0109 | -2.65 | -3.28778 | -0.453 |
| ZNF217 | ILMN_1755303 | 4.64E-05 | 8.83E-07 | -5.68 | 5.59448 | -0.454 |
| SIN3A | ILMN_1805996 | 0.00058 | 2.30E-05 | -4.71 | 2.46974 | -0.454 |
| GGTLC2 | ILMN_2403896 | 0.0369 | 0.0055 | -2.91 | -2.66476 | -0.454 |
| ORC2 | ILMN_2061452 | 0.0381 | 0.00572 | -2.9 | -2.70187 | -0.454 |
| MYBPH | ILMN_1705297 | 0.0633 | 0.0114 | -2.64 | -3.32206 | -0.454 |
| DCAF15 | ILMN_2192620 | 1.80E-05 | 2.48E-07 | -6.05 | 6.81582 | -0.455 |
| MRFAP1L1 | ILMN_1689774 | 8.81E-05 | 2.04E-06 | -5.43 | 4.78919 | -0.455 |
| HIP1 | ILMN_1701403 | 0.000704 | 3.01E-05 | -4.63 | 2.21503 | -0.455 |
| DEF6 | ILMN_2145280 | 0.00153 | 8.31E-05 | -4.32 | 1.24881 | -0.455 |
| EP400 | ILMN_1673023 | 0.00779 | 0.00071 | -3.63 | -0.77361 | -0.455 |
| GTF2H1 | ILMN_1726693 | 0.0158 | 0.00181 | -3.31 | -1.64518 | -0.455 |
| MYO5A | ILMN_1698225 | 0.025 | 0.00331 | -3.1 | -2.20177 | -0.455 |
| SULT1A2 | ILMN_1798528 | 0.0577 | 0.0101 | -2.68 | -3.2141 | -0.455 |
| ZNF341 | ILMN_2043728 | 0.0774 | 0.0149 | -2.53 | -3.56576 | -0.455 |
| MCCC1 | ILMN_1760174 | 0.00282 | 0.00019 | -4.06 | 0.46722 | -0.456 |
| C19orf25 | ILMN_1655225 | 0.0144 | 0.00159 | -3.36 | -1.52579 | -0.456 |
| SNX18 | ILMN_3229340 | 0.135 | 0.0321 | -2.21 | -4.23615 | -0.456 |
| PHF19 | ILMN_1713249 | 0.0043 | 0.00033 | -3.88 | -0.05425 | -0.457 |
| VEGFA | ILMN_2375879 | 0.0676 | 0.0125 | -2.6 | -3.40495 | -0.457 |
| YWHAG | ILMN_1750145 | 3.22E-05 | 5.37E-07 | -5.82 | 6.07194 | -0.458 |
| CIAO1 | ILMN_1792837 | 0.000774 | 3.42E-05 | -4.59 | 2.09222 | -0.458 |
| TYK2 | ILMN_1676955 | 0.00166 | 9.30E-05 | -4.28 | 1.14268 | -0.458 |
| RAB11FIP4 | ILMN_1681467 | 0.00674 | 0.000587 | -3.69 | -0.59455 | -0.458 |
| WDR20 | ILMN_1707199 | 5.13E-08 | 7.97E-11 | -8.39 | 14.55388 | -0.459 |
| RNF44 | ILMN_1660079 | 9.99E-06 | 1.09E-07 | -6.29 | 7.61074 | -0.459 |
| PARK7 | ILMN_1744713 | 0.000131 | 3.33E-06 | -5.29 | 4.32058 | -0.459 |
| ZDHHC13 | ILMN_1684663 | 0.00196 | 0.000117 | -4.21 | 0.92345 | -0.459 |
| LRRC6 | ILMN_1739683 | 0.0647 | 0.0117 | -2.63 | -3.34804 | -0.459 |
| CTSC | ILMN_1792885 | 0.000543 | 2.10E-05 | -4.74 | 2.55637 | -0.46 |
| ARHGAP4 | ILMN_1770824 | 0.000714 | 3.08E-05 | -4.62 | 2.19324 | -0.46 |
| ZDHHC13 | ILMN_1785831 | 0.00266 | 0.000176 | -4.08 | 0.53657 | -0.46 |
| CHERP | ILMN_1798083 | 0.00352 | 0.000254 | -3.97 | 0.1921 | -0.46 |
| FUCA2 | ILMN_1744914 | 0.00415 | 0.000313 | -3.9 | -0.00452 | -0.46 |
| HK2 | ILMN_1723486 | 0.00724 | 0.000641 | -3.66 | -0.67802 | -0.46 |
| TMEM184C | ILMN_3237779 | 4.80E-05 | 9.33E-07 | -5.66 | 5.54204 | -0.461 |
| FDX1L | ILMN_1744628 | 0.000643 | 2.66E-05 | -4.67 | 2.33199 | -0.461 |
| EDC4 | ILMN_1665212 | 0.0011 | 5.41E-05 | -4.45 | 1.65622 | -0.461 |
| TNNT3 | ILMN_1720231 | 0.0553 | 0.0095 | -2.71 | -3.16006 | -0.461 |
| B3GNT8 | ILMN_1741389 | 0.00424 | 0.000323 | -3.89 | -0.03395 | -0.462 |
| PRDM10 | ILMN_2352295 | 0.0175 | 0.00207 | -3.27 | -1.76759 | -0.462 |
| MAP3K11 | ILMN_1651788 | 3.08E-06 | 2.02E-08 | -6.77 | 9.23124 | -0.463 |
| LRRC25 | ILMN_1766487 | 7.11E-06 | 6.96E-08 | -6.41 | 8.03907 | -0.463 |
| RUNDC1 | ILMN_1733875 | 0.000259 | 8.34E-06 | -5.02 | 3.44073 | -0.463 |
| GXYLT1 | ILMN_1747183 | 0.00312 | 0.000216 | -4.02 | 0.34428 | -0.463 |
| CCND2 | ILMN_2067656 | 0.00811 | 0.000749 | -3.61 | -0.82306 | -0.463 |
| SAP130 | ILMN_1700044 | 0.00041 | 1.48E-05 | -4.85 | 2.89221 | -0.464 |
| MFSD8 | ILMN_2123119 | 0.00159 | 8.76E-05 | -4.3 | 1.19924 | -0.464 |
| APBA3 | ILMN_1686610 | 0.0024 | 0.000154 | -4.12 | 0.66308 | -0.464 |
| CWF19L2 | ILMN_1668498 | 9.59E-05 | 2.29E-06 | -5.4 | 4.67899 | -0.465 |
| ATF4 | ILMN_1783394 | 0.007 | 0.000614 | -3.68 | -0.6366 | -0.465 |
| SLC6A12 | ILMN_1780831 | 0.0251 | 0.00333 | -3.1 | -2.20739 | -0.465 |
| DLEU7 | ILMN_3307752 | 0.0284 | 0.0039 | -3.04 | -2.35072 | -0.465 |
| PLOD1 | ILMN_1684391 | 0.0593 | 0.0105 | -2.67 | -3.24937 | -0.465 |
| DCAF7 | ILMN_1682264 | 4.19E-05 | 7.66E-07 | -5.72 | 5.73078 | -0.466 |
| CLSTN1 | ILMN_2415179 | 0.0182 | 0.00217 | -3.25 | -1.8132 | -0.466 |
| FAM212B | ILMN_1890614 | 0.0302 | 0.00422 | -3.01 | -2.42352 | -0.466 |
| AP3M1 | ILMN_1667086 | 0.0319 | 0.00455 | -2.98 | -2.49219 | -0.466 |
| RFX1 | ILMN_1725787 | 1.66E-05 | 2.22E-07 | -6.08 | 6.92211 | -0.467 |
| ARHGAP30 | ILMN_1750805 | 0.000412 | 1.49E-05 | -4.84 | 2.8835 | -0.467 |
| RETSAT | ILMN_1702633 | 0.00174 | 9.96E-05 | -4.26 | 1.07778 | -0.467 |
| DIDO1 | ILMN_1763605 | 0.0162 | 0.00186 | -3.3 | -1.6714 | -0.467 |
| BRF2 | ILMN_1665554 | 0.0223 | 0.00284 | -3.15 | -2.05922 | -0.467 |
| LSM4 | ILMN_1788099 | 0.122 | 0.0279 | -2.27 | -4.11524 | -0.467 |
| TAF4 | ILMN_1737535 | 0.0592 | 0.0104 | -2.67 | -3.24583 | -0.468 |
| PLCG2 | ILMN_1815719 | 1.28E-05 | 1.52E-07 | -6.19 | 7.2892 | -0.469 |
| ZNF106 | ILMN_2043265 | 0.000294 | 9.73E-06 | -4.97 | 3.29286 | -0.469 |
| FKBP1A | ILMN_1683658 | 0.000139 | 3.57E-06 | -5.27 | 4.25246 | -0.47 |
| TRAK2 | ILMN_1781691 | 0.0108 | 0.0011 | -3.48 | -1.17824 | -0.47 |
| FAM110A | ILMN_2248093 | 0.0259 | 0.00349 | -3.08 | -2.24844 | -0.47 |
| MICALCL | ILMN_1743232 | 0.0399 | 0.00608 | -2.88 | -2.75725 | -0.47 |
| TNIP1 | ILMN_1703650 | 0.00145 | 7.71E-05 | -4.34 | 1.31996 | -0.471 |
| SOS1 | ILMN_1767135 | 0.00315 | 0.000219 | -4.01 | 0.3326 | -0.471 |
| IKBKE | ILMN_1755024 | 0.00713 | 0.00063 | -3.67 | -0.6611 | -0.471 |
| RAB24 | ILMN_2379718 | 0.0123 | 0.0013 | -3.43 | -1.34008 | -0.471 |
| C9orf69 | ILMN_3244065 | 0.0217 | 0.00273 | -3.17 | -2.02369 | -0.471 |
| DOPEY2 | ILMN_1741711 | 0.0326 | 0.00467 | -2.97 | -2.51651 | -0.471 |
| CEP85L | ILMN_1855286 | 0.0356 | 0.00523 | -2.93 | -2.61878 | -0.471 |
| CDC42EP2 | ILMN_1652777 | 0.094 | 0.0195 | -2.42 | -3.79975 | -0.471 |
| MYCBP2 | ILMN_1811972 | 0.0963 | 0.0201 | -2.41 | -3.82762 | -0.471 |
| P2RY10 | ILMN_1784774 | 0.136 | 0.0322 | -2.21 | -4.24096 | -0.471 |
| FKBP2 | ILMN_1674337 | 0.00191 | 0.000113 | -4.22 | 0.96088 | -0.472 |
| ELMO3 | ILMN_1752665 | 0.00194 | 0.000115 | -4.22 | 0.94088 | -0.472 |
| PMS2CL | ILMN_2262198 | 0.00759 | 0.000685 | -3.64 | -0.73957 | -0.472 |
| BCDIN3D | ILMN_1796113 | 0.0253 | 0.00337 | -3.09 | -2.21804 | -0.472 |
| BOK | ILMN_1730032 | 0.069 | 0.0129 | -2.59 | -3.43165 | -0.472 |
| ZNF106 | ILMN_1800912 | 0.000202 | 5.95E-06 | -5.12 | 3.76337 | -0.473 |
| API5 | ILMN_1815051 | 0.00192 | 0.000113 | -4.22 | 0.95469 | -0.473 |
| TUSC2 | ILMN_1804329 | 0.00195 | 0.000115 | -4.22 | 0.93712 | -0.473 |
| VPS36 | ILMN_1802519 | 0.00746 | 0.000668 | -3.65 | -0.71608 | -0.473 |
| RAB24 | ILMN_2278850 | 0.0485 | 0.0079 | -2.78 | -2.99411 | -0.473 |
| TNFSF14 | ILMN_2363392 | 1.09E-05 | 1.21E-07 | -6.26 | 7.50871 | -0.475 |
| ARID3B | ILMN_1696956 | 0.000884 | 4.05E-05 | -4.54 | 1.93197 | -0.475 |
| F2RL1 | ILMN_1673113 | 0.000994 | 4.74E-05 | -4.49 | 1.78291 | -0.475 |
| NBPF10 | ILMN_2155719 | 0.00759 | 0.000685 | -3.64 | -0.74017 | -0.475 |
| NFKBIE | ILMN_1717313 | 0.0088 | 0.000828 | -3.58 | -0.91709 | -0.475 |
| LCLAT1 | ILMN_3186473 | 0.0109 | 0.00111 | -3.48 | -1.19349 | -0.475 |
| RINT1 | ILMN_1784584 | 0.0117 | 0.00122 | -3.45 | -1.27941 | -0.475 |
| NELFB | ILMN_2399328 | 0.0328 | 0.00472 | -2.97 | -2.52525 | -0.475 |
| GDAP2 | ILMN_1789059 | 0.000547 | 2.13E-05 | -4.74 | 2.54632 | -0.476 |
| XKR3 | ILMN_1803609 | 0.00598 | 0.000502 | -3.74 | -0.44903 | -0.477 |
| FAM214B | ILMN_1732609 | 4.45E-06 | 3.41E-08 | -6.62 | 8.72544 | -0.478 |
| ITGB2 | ILMN_2175912 | 1.84E-05 | 2.55E-07 | -6.04 | 6.78897 | -0.478 |
| IRF2BP2 | ILMN_2394561 | 0.000127 | 3.19E-06 | -5.3 | 4.36217 | -0.478 |
| SNRNP40 | ILMN_1799814 | 0.00161 | 8.89E-05 | -4.3 | 1.1849 | -0.478 |
| SLC9A3R1 | ILMN_1680925 | 0.00161 | 8.97E-05 | -4.29 | 1.1766 | -0.478 |
| RBM12B | ILMN_1805778 | 0.00808 | 0.000745 | -3.61 | -0.81795 | -0.478 |
| SIGLEC14 | ILMN_3243061 | 1.35E-05 | 1.62E-07 | -6.17 | 7.22289 | -0.479 |
| SMC3 | ILMN_1718807 | 7.54E-05 | 1.67E-06 | -5.49 | 4.98533 | -0.479 |
| PRDM10 | ILMN_2352293 | 0.000765 | 3.38E-05 | -4.6 | 2.10476 | -0.479 |
| LPGAT1 | ILMN_1687998 | 0.000895 | 4.13E-05 | -4.53 | 1.91374 | -0.479 |
| VEGFA | ILMN_1803882 | 0.094 | 0.0195 | -2.42 | -3.80071 | -0.479 |
| MFAP1 | ILMN_1676799 | 0.000213 | 6.38E-06 | -5.1 | 3.69694 | -0.48 |
| PGAM4 | ILMN_1706841 | 0.00697 | 0.00061 | -3.68 | -0.63194 | -0.48 |
| RPS6KA4 | ILMN_1756204 | 0.00969 | 0.000945 | -3.53 | -1.04021 | -0.48 |
| IER5L | ILMN_1755620 | 0.147 | 0.0361 | -2.16 | -4.33982 | -0.48 |
| HMGA1 | ILMN_2311537 | 0.0255 | 0.00341 | -3.09 | -2.22964 | -0.481 |
| BRPF1 | ILMN_2365549 | 0.00103 | 4.98E-05 | -4.48 | 1.73542 | -0.482 |
| TGFBRAP1 | ILMN_1696870 | 0.00608 | 0.000514 | -3.74 | -0.47026 | -0.482 |
| MECP2 | ILMN_1702715 | 0.00266 | 0.000176 | -4.08 | 0.53875 | -0.483 |
| DUSP6 | ILMN_2396020 | 0.0148 | 0.00166 | -3.34 | -1.5617 | -0.483 |
| TCFL5 | ILMN_1814247 | 0.0153 | 0.00174 | -3.33 | -1.60833 | -0.484 |
| CCPG1 | ILMN_1794190 | 0.0256 | 0.00342 | -3.09 | -2.23128 | -0.484 |
| ORAI1 | ILMN_1724148 | 0.0302 | 0.00422 | -3.01 | -2.42452 | -0.484 |
| MED19 | ILMN_1754553 | 2.57E-05 | 4.10E-07 | -5.9 | 6.33175 | -0.485 |
| CRNKL1 | ILMN_1768640 | 0.000445 | 1.64E-05 | -4.81 | 2.79648 | -0.485 |
| LOC606724 | ILMN_2125747 | 0.00195 | 0.000116 | -4.21 | 0.93342 | -0.485 |
| NOMO2 | ILMN_1799856 | 0.0206 | 0.00255 | -3.19 | -1.96252 | -0.485 |
| TMEM39B | ILMN_2212714 | 0.000164 | 4.50E-06 | -5.2 | 4.03058 | -0.486 |
| TNFAIP1 | ILMN_1655429 | 0.00337 | 0.000241 | -3.98 | 0.24318 | -0.486 |
| HAUS8 | ILMN_3253579 | 0.00598 | 0.000503 | -3.74 | -0.44972 | -0.486 |
| PBDC1 | ILMN_1768176 | 0.0097 | 0.000946 | -3.53 | -1.04124 | -0.486 |
| EHD4 | ILMN_1720083 | 0.01 | 0.000989 | -3.52 | -1.08233 | -0.486 |
| SUPT16H | ILMN_1781516 | 5.26E-06 | 4.53E-08 | -6.54 | 8.45141 | -0.487 |
| TMEM86B | ILMN_2049417 | 0.000671 | 2.81E-05 | -4.65 | 2.27922 | -0.487 |
| KMT2C | ILMN_2408877 | 0.00072 | 3.11E-05 | -4.62 | 2.18242 | -0.487 |
| RHOF | ILMN_1652918 | 0.0799 | 0.0156 | -2.51 | -3.60324 | -0.487 |
| MIEF1 | ILMN_1793203 | 0.0011 | 5.43E-05 | -4.45 | 1.65268 | -0.488 |
| TMEM185A | ILMN_2140389 | 0.0102 | 0.00101 | -3.51 | -1.10618 | -0.488 |
| PTPA | ILMN_1729123 | 0.0109 | 0.00111 | -3.48 | -1.19267 | -0.488 |
| SLC20A1 | ILMN_1672662 | 0.0356 | 0.00522 | -2.93 | -2.61849 | -0.488 |
| CREB3L2 | ILMN_1751097 | 2.46E-07 | 7.05E-10 | -7.75 | 12.46031 | -0.489 |
| TBC1D9B | ILMN_1789909 | 0.00806 | 0.000742 | -3.62 | -0.81436 | -0.489 |
| RPS6KA5 | ILMN_1657515 | 0.115 | 0.0255 | -2.31 | -4.0376 | -0.489 |
| TIMP2 | ILMN_1721876 | 2.26E-05 | 3.36E-07 | -5.96 | 6.52481 | -0.49 |
| SENP5 | ILMN_1675501 | 0.000921 | 4.29E-05 | -4.52 | 1.87845 | -0.49 |
| NUP37 | ILMN_1771903 | 0.00226 | 0.000143 | -4.15 | 0.73748 | -0.49 |
| DAPK1 | ILMN_1708340 | 0.00287 | 0.000194 | -4.05 | 0.44663 | -0.49 |
| CPSF3L | ILMN_1718013 | 0.0107 | 0.00109 | -3.49 | -1.17166 | -0.49 |
| CYB561D1 | ILMN_3180557 | 0.0126 | 0.00135 | -3.41 | -1.37306 | -0.49 |
| CMTM8 | ILMN_1710124 | 0.0229 | 0.00296 | -3.14 | -2.09713 | -0.49 |
| RNF4 | ILMN_2142695 | 2.45E-05 | 3.75E-07 | -5.93 | 6.41862 | -0.491 |
| SH2D3C | ILMN_1775734 | 3.58E-05 | 6.21E-07 | -5.78 | 5.93297 | -0.491 |
| ARHGEF2 | ILMN_1703477 | 5.54E-05 | 1.13E-06 | -5.61 | 5.36046 | -0.491 |
| TOMM34 | ILMN_1721128 | 0.000225 | 6.85E-06 | -5.08 | 3.62922 | -0.491 |
| ZNF140 | ILMN_1727923 | 0.00576 | 0.000478 | -3.76 | -0.4032 | -0.491 |
| DSE | ILMN_1706498 | 9.22E-06 | 9.72E-08 | -6.32 | 7.717 | -0.492 |
| TBC1D3F | ILMN_2068991 | 0.00316 | 0.00022 | -4.01 | 0.32962 | -0.492 |
| ZNF224 | ILMN_1661293 | 0.00463 | 0.00036 | -3.85 | -0.13617 | -0.492 |
| GPR141 | ILMN_2092333 | 0.014 | 0.00154 | -3.37 | -1.49282 | -0.492 |
| GAPT | ILMN_3242271 | 0.0171 | 0.00201 | -3.28 | -1.73978 | -0.492 |
| RPRD1B | ILMN_3243461 | 0.0016 | 8.85E-05 | -4.3 | 1.18962 | -0.493 |
| SUSD3 | ILMN_1785570 | 0.18 | 0.0484 | -2.03 | -4.58949 | -0.493 |
| BTBD7 | ILMN_1687743 | 4.99E-06 | 4.20E-08 | -6.56 | 8.52473 | -0.494 |
| MED14 | ILMN_1739956 | 0.00844 | 0.000788 | -3.6 | -0.87081 | -0.494 |
| ZNF212 | ILMN_1684964 | 0.0229 | 0.00296 | -3.14 | -2.09862 | -0.494 |
| FUT7 | ILMN_1724236 | 0.0513 | 0.00857 | -2.75 | -3.06734 | -0.494 |
| DDX28 | ILMN_1741736 | 0.106 | 0.023 | -2.35 | -3.94589 | -0.494 |
| ANKLE2 | ILMN_3249608 | 0.000345 | 1.18E-05 | -4.91 | 3.10542 | -0.495 |
| CTCF | ILMN_1786015 | 0.00327 | 0.000231 | -4 | 0.28095 | -0.495 |
| SGF29 | ILMN_1701477 | 0.00504 | 0.000405 | -3.82 | -0.24614 | -0.495 |
| BCL2 | ILMN_2246956 | 0.00694 | 0.000606 | -3.68 | -0.62573 | -0.495 |
| CDK13 | ILMN_1778557 | 0.000147 | 3.82E-06 | -5.25 | 4.18892 | -0.496 |
| CD151 | ILMN_2326713 | 0.000533 | 2.06E-05 | -4.75 | 2.57893 | -0.496 |
| CCDC97 | ILMN_1801600 | 4.54E-05 | 8.55E-07 | -5.69 | 5.62594 | -0.497 |
| TTC17 | ILMN_1660810 | 0.00417 | 0.000315 | -3.9 | -0.01135 | -0.497 |
| PITPNM1 | ILMN_1653220 | 0.00422 | 0.000322 | -3.89 | -0.02993 | -0.497 |
| SLC35F5 | ILMN_2206554 | 0.00468 | 0.000367 | -3.85 | -0.15322 | -0.497 |
| PDXP | ILMN_1736441 | 0.0793 | 0.0154 | -2.52 | -3.5934 | -0.497 |
| SUN2 | ILMN_2099301 | 3.85E-05 | 6.81E-07 | -5.75 | 5.84487 | -0.498 |
| GORASP2 | ILMN_1748018 | 0.000177 | 5.01E-06 | -5.17 | 3.92915 | -0.498 |
| MYADM | ILMN_2308849 | 0.000318 | 1.07E-05 | -4.94 | 3.20491 | -0.498 |
| NEDD4 | ILMN_1751407 | 0.00118 | 5.94E-05 | -4.42 | 1.56868 | -0.498 |
| TAF1 | ILMN_2346358 | 0.015 | 0.00169 | -3.34 | -1.57958 | -0.498 |
| TACC1 | ILMN_1770084 | 0.00129 | 6.69E-05 | -4.39 | 1.4551 | -0.5 |
| TIGD7 | ILMN_1676905 | 0.00161 | 8.90E-05 | -4.3 | 1.18395 | -0.5 |
| LGALS1 | ILMN_1723978 | 0.00941 | 0.000906 | -3.55 | -1.00071 | -0.5 |
| TP53I11 | ILMN_2412624 | 0.0164 | 0.00191 | -3.29 | -1.69373 | -0.5 |
| CSE1L | ILMN_1706238 | 0.00191 | 0.000112 | -4.23 | 0.9663 | -0.501 |
| OAZ2 | ILMN_3239460 | 0.00407 | 0.000305 | -3.91 | 0.0184 | -0.501 |
| MKKS | ILMN_1718718 | 0.000182 | 5.19E-06 | -5.16 | 3.8956 | -0.502 |
| SLC4A1AP | ILMN_1750876 | 0.0144 | 0.0016 | -3.35 | -1.53244 | -0.502 |
| TLE4 | ILMN_2103761 | 2.03E-05 | 2.90E-07 | -6 | 6.66624 | -0.504 |
| ZNF763 | ILMN_1727183 | 0.000839 | 3.76E-05 | -4.56 | 2.00343 | -0.504 |
| ALKBH1 | ILMN_1758038 | 0.0015 | 8.07E-05 | -4.33 | 1.27719 | -0.504 |
| SULF2 | ILMN_1686981 | 0.00272 | 0.00018 | -4.08 | 0.51758 | -0.504 |
| COPS7B | ILMN_1784785 | 0.00789 | 0.000723 | -3.62 | -0.79027 | -0.504 |
| JMJD8 | ILMN_1687921 | 0.0107 | 0.00109 | -3.49 | -1.17187 | -0.504 |
| MPPE1 | ILMN_1776515 | 0.0405 | 0.0062 | -2.87 | -2.77452 | -0.504 |
| CUL5 | ILMN_2176955 | 0.00159 | 8.73E-05 | -4.3 | 1.20288 | -0.505 |
| C2orf42 | ILMN_1661594 | 3.07E-06 | 1.98E-08 | -6.78 | 9.25091 | -0.506 |
| CERS2 | ILMN_2400500 | 0.000303 | 1.01E-05 | -4.96 | 3.2611 | -0.506 |
| C10orf76 | ILMN_1792110 | 0.00116 | 5.83E-05 | -4.43 | 1.58541 | -0.506 |
| XPO7 | ILMN_2174884 | 0.00182 | 0.000106 | -4.24 | 1.02218 | -0.506 |
| SPEN | ILMN_1802611 | 0.00227 | 0.000143 | -4.15 | 0.73401 | -0.507 |
| SDAD1 | ILMN_1735360 | 0.00407 | 0.000306 | -3.91 | 0.01743 | -0.507 |
| NACC2 | ILMN_1882590 | 0.0475 | 0.00768 | -2.79 | -2.96844 | -0.507 |
| ZNF576 | ILMN_1687711 | 0.000704 | 3.01E-05 | -4.63 | 2.21497 | -0.508 |
| CSE1L | ILMN_1665797 | 0.000186 | 5.32E-06 | -5.15 | 3.87192 | -0.509 |
| ZBTB47 | ILMN_1699440 | 0.000205 | 6.07E-06 | -5.11 | 3.74531 | -0.509 |
| TBC1D10B | ILMN_1668639 | 0.00103 | 4.97E-05 | -4.48 | 1.73786 | -0.509 |
| MYO9B | ILMN_1672547 | 0.00144 | 7.66E-05 | -4.34 | 1.32634 | -0.509 |
| VPS18 | ILMN_1721575 | 0.00466 | 0.000365 | -3.85 | -0.14825 | -0.509 |
| GSN | ILMN_1801043 | 0.00751 | 0.000675 | -3.65 | -0.72577 | -0.51 |
| BRPF1 | ILMN_1669308 | 0.0294 | 0.00408 | -3.02 | -2.39175 | -0.51 |
| UBTF | ILMN_1806946 | 0.0065 | 0.00056 | -3.71 | -0.55171 | -0.511 |
| TMCC1 | ILMN_1677963 | 0.0394 | 0.006 | -2.88 | -2.74536 | -0.511 |
| PNKD | ILMN_1774604 | 0.0938 | 0.0194 | -2.42 | -3.79667 | -0.511 |
| CSK | ILMN_1754121 | 1.60E-05 | 2.05E-07 | -6.1 | 6.99713 | -0.512 |
| ARAP3 | ILMN_1812618 | 3.44E-05 | 5.84E-07 | -5.8 | 5.99221 | -0.512 |
| TLE4 | ILMN_1696704 | 0.00041 | 1.48E-05 | -4.84 | 2.89101 | -0.512 |
| CERS2 | ILMN_1726108 | 0.000765 | 3.37E-05 | -4.6 | 2.10749 | -0.512 |
| UBQLN4 | ILMN_1789349 | 0.00781 | 0.000712 | -3.63 | -0.776 | -0.512 |
| ETV5 | ILMN_1739222 | 0.0901 | 0.0183 | -2.45 | -3.74501 | -0.512 |
| GRAMD4 | ILMN_1723706 | 0.00328 | 0.000232 | -4 | 0.27898 | -0.513 |
| CREBBP | ILMN_2293692 | 0.00391 | 0.00029 | -3.92 | 0.06685 | -0.513 |
| LST1 | ILMN_1718936 | 0.0088 | 0.000829 | -3.58 | -0.91764 | -0.513 |
| HMOX1 | ILMN_1800512 | 0.0113 | 0.00117 | -3.46 | -1.23548 | -0.513 |
| LUC7L3 | ILMN_2299072 | 0.0193 | 0.00234 | -3.22 | -1.88364 | -0.513 |
| CLEC4A | ILMN_1709204 | 6.60E-05 | 1.42E-06 | -5.54 | 5.14101 | -0.514 |
| TFCP2 | ILMN_1738750 | 0.00286 | 0.000193 | -4.05 | 0.45018 | -0.514 |
| GAPT | ILMN_1675191 | 0.0122 | 0.00129 | -3.43 | -1.32761 | -0.514 |
| MCEE | ILMN_1735347 | 0.0353 | 0.00516 | -2.94 | -2.60798 | -0.514 |
| SPNS2 | ILMN_3301749 | 0.0487 | 0.00793 | -2.78 | -2.99715 | -0.514 |
| DDIT4 | ILMN_1661599 | 4.54E-05 | 8.56E-07 | -5.69 | 5.6249 | -0.515 |
| ACVR1 | ILMN_1760490 | 0.00705 | 0.000619 | -3.68 | -0.64548 | -0.515 |
| SAMD4B | ILMN_1753467 | 0.0246 | 0.00326 | -3.11 | -2.18594 | -0.515 |
| ARL5A | ILMN_1688526 | 0.0474 | 0.00767 | -2.79 | -2.96705 | -0.515 |
| SH3KBP1 | ILMN_1808501 | 4.80E-05 | 9.36E-07 | -5.66 | 5.53869 | -0.516 |
| CYTH2 | ILMN_3188106 | 0.00938 | 0.000901 | -3.55 | -0.99563 | -0.516 |
| RNF125 | ILMN_1821176 | 0.0185 | 0.00221 | -3.24 | -1.82943 | -0.516 |
| TOMM40L | ILMN_2065606 | 0.0189 | 0.00228 | -3.23 | -1.85972 | -0.516 |
| CCDC189 | ILMN_2179726 | 0.0849 | 0.0169 | -2.48 | -3.67482 | -0.516 |
| ADAP2 | ILMN_1763000 | 0.151 | 0.0377 | -2.14 | -4.37627 | -0.516 |
| VEZF1 | ILMN_1705310 | 2.02E-06 | 1.13E-08 | -6.94 | 9.78705 | -0.517 |
| AMY1A | ILMN_2294762 | 0.00959 | 0.000931 | -3.54 | -1.02615 | -0.517 |
| YWHAG | ILMN_2228809 | 9.83E-06 | 1.05E-07 | -6.29 | 7.63869 | -0.518 |
| ATP5D | ILMN_1679178 | 4.23E-05 | 7.78E-07 | -5.71 | 5.71598 | -0.518 |
| PLPPR2 | ILMN_1667319 | 0.000394 | 1.41E-05 | -4.86 | 2.94051 | -0.518 |
| PRPF38A | ILMN_1675626 | 0.00246 | 0.000159 | -4.12 | 0.6346 | -0.518 |
| GLIPR1 | ILMN_1769245 | 0.03 | 0.00419 | -3.01 | -2.4168 | -0.518 |
| ZMIZ1 | ILMN_1771627 | 1.05E-06 | 4.37E-09 | -7.22 | 10.70497 | -0.519 |
| CDC14A | ILMN_1774930 | 4.70E-05 | 8.95E-07 | -5.67 | 5.58122 | -0.519 |
| ADAP1 | ILMN_2047511 | 0.000955 | 4.49E-05 | -4.51 | 1.83481 | -0.519 |
| S100Z | ILMN_1795139 | 0.00787 | 0.00072 | -3.63 | -0.78684 | -0.519 |
| SLC22A18AS | ILMN_1691048 | 0.114 | 0.0254 | -2.31 | -4.03281 | -0.519 |
| TMEM259 | ILMN_1758642 | 4.74E-05 | 9.10E-07 | -5.67 | 5.56615 | -0.52 |
| BPTF | ILMN_1688381 | 0.000407 | 1.46E-05 | -4.85 | 2.90553 | -0.52 |
| TMC8 | ILMN_2211534 | 0.000418 | 1.52E-05 | -4.84 | 2.86557 | -0.52 |
| TSR1 | ILMN_2092232 | 0.000592 | 2.39E-05 | -4.7 | 2.43534 | -0.52 |
| PTPN23 | ILMN_1654318 | 0.00191 | 0.000112 | -4.23 | 0.96839 | -0.52 |
| SF3A3 | ILMN_1705151 | 0.00286 | 0.000193 | -4.05 | 0.45156 | -0.52 |
| PSTPIP1 | ILMN_1703327 | 0.00589 | 0.000492 | -3.75 | -0.42995 | -0.52 |
| GTF3C2 | ILMN_2356574 | 0.00511 | 0.000412 | -3.81 | -0.26312 | -0.521 |
| LIMD2 | ILMN_1673867 | 0.00821 | 0.000761 | -3.61 | -0.8377 | -0.521 |
| ADORA2A | ILMN_1807372 | 0.0228 | 0.00292 | -3.14 | -2.08675 | -0.521 |
| EPOR | ILMN_2168347 | 0.149 | 0.0366 | -2.15 | -4.3517 | -0.521 |
| NAGK | ILMN_1716547 | 0.000271 | 8.77E-06 | -5 | 3.39251 | -0.522 |
| F11R | ILMN_2406532 | 0.00245 | 0.000158 | -4.12 | 0.63976 | -0.522 |
| ARHGEF6 | ILMN_1803423 | 0.0185 | 0.00221 | -3.24 | -1.82896 | -0.522 |
| ITPKB | ILMN_1700432 | 0.0254 | 0.00339 | -3.09 | -2.22227 | -0.522 |
| TRIM52 | ILMN_1754130 | 0.0185 | 0.0022 | -3.24 | -1.82672 | -0.523 |
| CCDC47 | ILMN_1804522 | 0.00183 | 0.000106 | -4.24 | 1.01822 | -0.524 |
| HHEX | ILMN_1762712 | 0.00552 | 0.000453 | -3.78 | -0.35134 | -0.524 |
| SEPT9 | ILMN_1769118 | 4.78E-05 | 9.25E-07 | -5.66 | 5.5503 | -0.525 |
| CFP | ILMN_1658121 | 0.000663 | 2.77E-05 | -4.66 | 2.29534 | -0.525 |
| KLHL22 | ILMN_1705390 | 0.0135 | 0.00147 | -3.38 | -1.45213 | -0.525 |
| CCPG1 | ILMN_2409290 | 0.0213 | 0.00267 | -3.18 | -2.00262 | -0.525 |
| DOCK8 | ILMN_1788931 | 7.54E-05 | 1.66E-06 | -5.49 | 4.98772 | -0.526 |
| LYST | ILMN_1675956 | 0.00594 | 0.000497 | -3.75 | -0.43915 | -0.527 |
| RBBP4 | ILMN_3251085 | 0.0217 | 0.00274 | -3.17 | -2.02856 | -0.527 |
| UPF2 | ILMN_1796305 | 6.25E-06 | 5.85E-08 | -6.46 | 8.20605 | -0.528 |
| ZBTB4 | ILMN_2097793 | 1.43E-05 | 1.75E-07 | -6.15 | 7.15234 | -0.528 |
| CSF1R | ILMN_1686623 | 0.00474 | 0.000372 | -3.84 | -0.16601 | -0.528 |
| TBC1D9B | ILMN_2390227 | 0.0114 | 0.00118 | -3.46 | -1.24553 | -0.528 |
| MED14 | ILMN_3246388 | 3.36E-05 | 5.64E-07 | -5.81 | 6.02522 | -0.529 |
| SLC25A11 | ILMN_1664168 | 0.004 | 0.000299 | -3.91 | 0.03748 | -0.529 |
| SNUPN | ILMN_2364535 | 0.00297 | 0.000203 | -4.04 | 0.40268 | -0.53 |
| RAB36 | ILMN_1733045 | 0.175 | 0.0464 | -2.05 | -4.55406 | -0.53 |
| UPF2 | ILMN_2383693 | 6.78E-06 | 6.48E-08 | -6.44 | 8.10808 | -0.531 |
| NUP160 | ILMN_1652989 | 0.000192 | 5.53E-06 | -5.14 | 3.83451 | -0.531 |
| PRPF4B | ILMN_1736021 | 0.00282 | 0.000189 | -4.06 | 0.47049 | -0.531 |
| HMG20A | ILMN_1678290 | 0.00347 | 0.000249 | -3.97 | 0.20943 | -0.531 |
| TTC13 | ILMN_1745573 | 0.0283 | 0.00388 | -3.04 | -2.34699 | -0.531 |
| TRIM24 | ILMN_1799642 | 0.00375 | 0.000276 | -3.94 | 0.11257 | -0.532 |
| LINC01451 | ILMN_1838767 | 0.016 | 0.00184 | -3.31 | -1.66018 | -0.532 |
| TM9SF4 | ILMN_1674421 | 5.89E-05 | 1.22E-06 | -5.58 | 5.28363 | -0.534 |
| MFSD2A | ILMN_1789638 | 0.00128 | 6.62E-05 | -4.39 | 1.46532 | -0.534 |
| PPP1CA | ILMN_2377980 | 0.0159 | 0.00183 | -3.31 | -1.65392 | -0.534 |
| PARVG | ILMN_1695851 | 0.000368 | 1.29E-05 | -4.89 | 3.02426 | -0.535 |
| ARMT1 | ILMN_1798108 | 0.00164 | 9.12E-05 | -4.29 | 1.16094 | -0.536 |
| BAGE5 | ILMN_2206141 | 0.00705 | 0.000619 | -3.68 | -0.64567 | -0.536 |
| TRERF1 | ILMN_1792076 | 0.00824 | 0.000764 | -3.61 | -0.84136 | -0.536 |
| RAB37 | ILMN_1796136 | 0.0453 | 0.00723 | -2.81 | -2.91359 | -0.536 |
| TFF3 | ILMN_1811387 | 0.0124 | 0.00132 | -3.42 | -1.35336 | -0.537 |
| DAGLB | ILMN_1658885 | 0.0241 | 0.00318 | -3.11 | -2.16311 | -0.537 |
| ARAP3 | ILMN_3240997 | 1.64E-05 | 2.15E-07 | -6.09 | 6.95213 | -0.538 |
| TFEB | ILMN_1733616 | 0.00104 | 5.04E-05 | -4.47 | 1.72432 | -0.538 |
| SSH3 | ILMN_1755234 | 0.00191 | 0.000112 | -4.23 | 0.96576 | -0.538 |
| STX2 | ILMN_1726805 | 0.00396 | 0.000295 | -3.92 | 0.05197 | -0.538 |
| MAP1S | ILMN_1796336 | 4.96E-05 | 9.77E-07 | -5.65 | 5.49773 | -0.539 |
| ZBTB7B | ILMN_1736954 | 0.00963 | 0.000935 | -3.54 | -1.03022 | -0.539 |
| ARHGEF1 | ILMN_1772370 | 0.000476 | 1.81E-05 | -4.78 | 2.70028 | -0.54 |
| SULF2 | ILMN_1667460 | 0.000894 | 4.11E-05 | -4.54 | 1.91929 | -0.54 |
| REPIN1 | ILMN_2404385 | 0.00545 | 0.000446 | -3.78 | -0.33705 | -0.54 |
| DUS2 | ILMN_1811650 | 0.0766 | 0.0148 | -2.53 | -3.55567 | -0.54 |
| APOA2 | ILMN_1688543 | 0.12 | 0.0272 | -2.28 | -4.09298 | -0.54 |
| RAB10 | ILMN_1793433 | 9.64E-09 | 7.82E-12 | -9.09 | 16.77946 | -0.542 |
| SGK1 | ILMN_3305938 | 0.000101 | 2.43E-06 | -5.38 | 4.621 | -0.542 |
| COL18A1 | ILMN_1806733 | 0.0297 | 0.00413 | -3.02 | -2.40506 | -0.542 |
| CCDC12 | ILMN_1725071 | 0.000993 | 4.72E-05 | -4.49 | 1.78582 | -0.543 |
| NCOR1 | ILMN_2186369 | 0.00393 | 0.000291 | -3.92 | 0.06297 | -0.543 |
| FUNDC1 | ILMN_1728540 | 0.000282 | 9.27E-06 | -4.99 | 3.33974 | -0.544 |
| SEC23IP | ILMN_1690690 | 0.000586 | 2.34E-05 | -4.71 | 2.45318 | -0.544 |
| TWF2 | ILMN_1703305 | 0.00146 | 7.83E-05 | -4.34 | 1.30585 | -0.544 |
| ACADM | ILMN_2053679 | 0.00193 | 0.000114 | -4.22 | 0.9489 | -0.544 |
| LBR | ILMN_1768969 | 0.000474 | 1.80E-05 | -4.79 | 2.70448 | -0.545 |
| WRAP73 | ILMN_1711166 | 0.00465 | 0.000363 | -3.85 | -0.14439 | -0.545 |
| C15orf48 | ILMN_1805410 | 0.0532 | 0.00903 | -2.73 | -3.11453 | -0.545 |
| NFKB1 | ILMN_1714965 | 0.000176 | 4.96E-06 | -5.17 | 3.93748 | -0.546 |
| DNAJC10 | ILMN_2151541 | 0.00571 | 0.000472 | -3.76 | -0.39124 | -0.546 |
| WDR46 | ILMN_2212354 | 0.00202 | 0.000122 | -4.2 | 0.88472 | -0.547 |
| SNRNP25 | ILMN_3238712 | 0.0144 | 0.00161 | -3.35 | -1.53413 | -0.547 |
| PHKG2 | ILMN_1669607 | 0.0393 | 0.00598 | -2.88 | -2.741 | -0.547 |
| CORO1B | ILMN_2377019 | 0.0122 | 0.00129 | -3.43 | -1.33156 | -0.549 |
| CIDEB | ILMN_1668910 | 0.0293 | 0.00406 | -3.03 | -2.38857 | -0.549 |
| ASF1B | ILMN_1695414 | 0.036 | 0.0053 | -2.93 | -2.63246 | -0.549 |
| NDUFAF1 | ILMN_1754421 | 1.35E-05 | 1.64E-07 | -6.17 | 7.21125 | -0.55 |
| CYB561A3 | ILMN_2129505 | 0.0213 | 0.00267 | -3.18 | -2.0031 | -0.55 |
| STIP1 | ILMN_1789510 | 1.35E-05 | 1.61E-07 | -6.17 | 7.23026 | -0.551 |
| ADCY7 | ILMN_1655935 | 3.75E-05 | 6.58E-07 | -5.76 | 5.87709 | -0.551 |
| SLC40A1 | ILMN_2053103 | 0.0029 | 0.000196 | -4.05 | 0.4344 | -0.551 |
| PRDX1 | ILMN_2366388 | 0.00374 | 0.000275 | -3.94 | 0.11854 | -0.551 |
| CCS | ILMN_2290998 | 0.0256 | 0.00343 | -3.09 | -2.23411 | -0.551 |
| NUP93 | ILMN_2196569 | 0.00286 | 0.000193 | -4.05 | 0.45286 | -0.552 |
| CORO1B | ILMN_1653708 | 0.00328 | 0.000232 | -4 | 0.27872 | -0.552 |
| MGME1 | ILMN_2176251 | 0.00353 | 0.000255 | -3.96 | 0.18872 | -0.552 |
| MCRIP2 | ILMN_1730523 | 0.0204 | 0.00253 | -3.2 | -1.95293 | -0.552 |
| TNNT3 | ILMN_2334080 | 0.0207 | 0.00257 | -3.19 | -1.96936 | -0.552 |
| BID | ILMN_1763386 | 0.000176 | 4.95E-06 | -5.17 | 3.94037 | -0.553 |
| NMT1 | ILMN_1762678 | 0.00233 | 0.000149 | -4.14 | 0.6981 | -0.553 |
| JAML | ILMN_1778723 | 0.00707 | 0.000622 | -3.67 | -0.64932 | -0.553 |
| IL16 | ILMN_1813572 | 0.051 | 0.00847 | -2.75 | -3.05676 | -0.553 |
| SIGLEC7 | ILMN_1686706 | 0.0345 | 0.00503 | -2.95 | -2.58452 | -0.554 |
| MCM5 | ILMN_1815169 | 0.017 | 0.00199 | -3.28 | -1.72983 | -0.555 |
| S100A4 | ILMN_1688780 | 0.000192 | 5.55E-06 | -5.14 | 3.83153 | -0.556 |
| NFE2L1 | ILMN_1739450 | 0.000202 | 5.97E-06 | -5.12 | 3.76079 | -0.556 |
| SS18L1 | ILMN_1676625 | 0.0132 | 0.00144 | -3.39 | -1.43125 | -0.556 |
| FAM217B | ILMN_1718712 | 0.0184 | 0.0022 | -3.24 | -1.82446 | -0.556 |
| KIAA0753 | ILMN_1680010 | 0.00995 | 0.000981 | -3.52 | -1.07564 | -0.557 |
| FCER1A | ILMN_1688423 | 0.0409 | 0.00628 | -2.86 | -2.78625 | -0.557 |
| FAM110A | ILMN_1782095 | 0.000206 | 6.10E-06 | -5.11 | 3.73946 | -0.558 |
| NFIC | ILMN_1675130 | 0.00058 | 2.31E-05 | -4.71 | 2.46693 | -0.559 |
| UTP6 | ILMN_1778238 | 0.00327 | 0.000231 | -4 | 0.28205 | -0.559 |
| TACSTD2 | ILMN_1739001 | 0.00336 | 0.00024 | -3.98 | 0.24743 | -0.559 |
| NRDE2 | ILMN_1740165 | 0.0108 | 0.0011 | -3.48 | -1.18377 | -0.559 |
| CLEC4A | ILMN_2399363 | 5.46E-06 | 4.77E-08 | -6.52 | 8.40356 | -0.56 |
| BBS4 | ILMN_1762466 | 0.00211 | 0.00013 | -4.18 | 0.82696 | -0.56 |
| CEP19 | ILMN_1873107 | 0.000386 | 1.37E-05 | -4.87 | 2.96498 | -0.561 |
| LCLAT1 | ILMN_1708081 | 0.00251 | 0.000163 | -4.11 | 0.61203 | -0.561 |
| RAB40C | ILMN_1712705 | 0.00465 | 0.000363 | -3.85 | -0.14487 | -0.561 |
| TBC1D10C | ILMN_1710434 | 0.00908 | 0.000865 | -3.56 | -0.95764 | -0.561 |
| ANKRD33 | ILMN_1667748 | 0.0223 | 0.00284 | -3.15 | -2.06083 | -0.561 |
| CSNK2A1 | ILMN_2386355 | 0.000456 | 1.71E-05 | -4.8 | 2.7562 | -0.562 |
| CTSD | ILMN_1674038 | 0.00407 | 0.000306 | -3.91 | 0.01596 | -0.562 |
| PIP5K1C | ILMN_1668514 | 0.00464 | 0.000362 | -3.85 | -0.14246 | -0.562 |
| ZNF692 | ILMN_1800750 | 0.0179 | 0.00212 | -3.26 | -1.79139 | -0.563 |
| RINL | ILMN_1790962 | 0.000619 | 2.54E-05 | -4.68 | 2.37506 | -0.564 |
| DBT | ILMN_1714990 | 0.00477 | 0.000375 | -3.84 | -0.17572 | -0.564 |
| PLGLB1 | ILMN_2094416 | 0.000341 | 1.16E-05 | -4.92 | 3.12354 | -0.565 |
| BCOR | ILMN_1712161 | 0.00166 | 9.25E-05 | -4.29 | 1.14756 | -0.565 |
| DBP | ILMN_1715555 | 0.00209 | 0.000128 | -4.18 | 0.8411 | -0.565 |
| RREB1 | ILMN_3307409 | 0.00534 | 0.000434 | -3.79 | -0.31285 | -0.565 |
| STK11IP | ILMN_1690085 | 0.00753 | 0.000676 | -3.65 | -0.72768 | -0.565 |
| HMOX2 | ILMN_1658807 | 0.00885 | 0.000836 | -3.58 | -0.9254 | -0.565 |
| ARHGAP18 | ILMN_1791186 | 0.00122 | 6.20E-05 | -4.41 | 1.52763 | -0.567 |
| SASH3 | ILMN_1697554 | 1.39E-06 | 6.41E-09 | -7.1 | 10.33547 | -0.568 |
| RPA1 | ILMN_3249261 | 0.000104 | 2.54E-06 | -5.37 | 4.58101 | -0.568 |
| ZKSCAN4 | ILMN_1804571 | 0.00571 | 0.000471 | -3.77 | -0.3897 | -0.568 |
| RNF34 | ILMN_1786039 | 4.66E-10 | 1.02E-13 | -10.4 | 20.91161 | -0.569 |
| NPRL2 | ILMN_2191568 | 0.0065 | 0.00056 | -3.71 | -0.55078 | -0.569 |
| SEMA4A | ILMN_1702787 | 0.000693 | 2.94E-05 | -4.64 | 2.23689 | -0.57 |
| WDR33 | ILMN_2383913 | 0.00318 | 0.000223 | -4.01 | 0.31691 | -0.57 |
| FERMT3 | ILMN_2366330 | 2.23E-05 | 3.28E-07 | -5.97 | 6.54694 | -0.572 |
| PTP4A3 | ILMN_3250321 | 0.00653 | 0.000563 | -3.71 | -0.55665 | -0.572 |
| RNF4 | ILMN_1687941 | 5.07E-06 | 4.29E-08 | -6.55 | 8.50436 | -0.573 |
| CCDC88B | ILMN_1772208 | 2.60E-05 | 4.18E-07 | -5.9 | 6.31426 | -0.573 |
| AAK1 | ILMN_1688755 | 1.60E-05 | 2.06E-07 | -6.1 | 6.99312 | -0.574 |
| SLC16A5 | ILMN_1803720 | 0.00314 | 0.000218 | -4.01 | 0.337 | -0.574 |
| FAM78A | ILMN_1801304 | 0.0179 | 0.00212 | -3.26 | -1.79142 | -0.574 |
| PLAGL2 | ILMN_1786601 | 0.000111 | 2.74E-06 | -5.35 | 4.5066 | -0.575 |
| PECAM1 | ILMN_1689518 | 0.00178 | 0.000103 | -4.25 | 1.04984 | -0.575 |
| ZW10 | ILMN_1712556 | 0.00037 | 1.30E-05 | -4.88 | 3.01564 | -0.576 |
| EXOSC1 | ILMN_2396648 | 0.014 | 0.00153 | -3.37 | -1.49137 | -0.576 |
| GGA3 | ILMN_2402972 | 6.41E-05 | 1.36E-06 | -5.55 | 5.18296 | -0.577 |
| ABR | ILMN_1672878 | 6.77E-07 | 2.58E-09 | -7.37 | 11.21191 | -0.578 |
| FBXW7 | ILMN_1754279 | 2.03E-05 | 2.90E-07 | -6 | 6.6658 | -0.578 |
| P2RY10 | ILMN_2321578 | 0.0722 | 0.0137 | -2.56 | -3.4854 | -0.578 |
| NSUN5P1 | ILMN_2352448 | 0.075 | 0.0144 | -2.54 | -3.53013 | -0.578 |
| HOTAIRM1///HOXA2 | ILMN_1869943 | 0.000309 | 1.03E-05 | -4.95 | 3.23816 | -0.579 |
| TNFAIP2 | ILMN_1727689 | 0.000318 | 1.07E-05 | -4.94 | 3.20363 | -0.58 |
| ZKSCAN1 | ILMN_2139052 | 0.000628 | 2.60E-05 | -4.68 | 2.35499 | -0.58 |
| RASAL3 | ILMN_1795089 | 0.00127 | 6.54E-05 | -4.39 | 1.47694 | -0.581 |
| PAXIP1 | ILMN_1680193 | 0.00256 | 0.000167 | -4.1 | 0.58784 | -0.581 |
| TMOD2 | ILMN_3251132 | 0.00675 | 0.000587 | -3.69 | -0.59584 | -0.581 |
| CHST13 | ILMN_1734707 | 0.0277 | 0.00378 | -3.05 | -2.32206 | -0.581 |
| PEA15 | ILMN_1771376 | 0.00867 | 0.000813 | -3.58 | -0.90036 | -0.582 |
| ERP27 | ILMN_1655261 | 0.00363 | 0.000263 | -3.95 | 0.15783 | -0.583 |
| SGK1 | ILMN_3229324 | 0.000178 | 5.04E-06 | -5.17 | 3.9233 | -0.584 |
| MAN2B1 | ILMN_1759341 | 0.00217 | 0.000135 | -4.17 | 0.79259 | -0.584 |
| SNAPC4 | ILMN_1677484 | 0.0193 | 0.00234 | -3.22 | -1.88285 | -0.585 |
| HDAC1 | ILMN_1727458 | 0.000249 | 7.87E-06 | -5.03 | 3.49604 | -0.586 |
| AP4E1 | ILMN_1754531 | 0.00325 | 0.000229 | -4 | 0.29157 | -0.586 |
| CEACAM3 | ILMN_1743570 | 0.0229 | 0.00295 | -3.14 | -2.09646 | -0.586 |
| NCKAP1L | ILMN_1674250 | 5.40E-08 | 8.66E-11 | -8.37 | 14.4745 | -0.587 |
| ZBTB45 | ILMN_1661484 | 4.99E-06 | 4.17E-08 | -6.56 | 8.53252 | -0.588 |
| ADAM15 | ILMN_1756920 | 0.00159 | 8.79E-05 | -4.3 | 1.19643 | -0.588 |
| DAXX | ILMN_1708077 | 3.92E-06 | 2.83E-08 | -6.67 | 8.90497 | -0.589 |
| VDR | ILMN_2319952 | 5.29E-06 | 4.59E-08 | -6.54 | 8.44073 | -0.589 |
| CD302 | ILMN_1716797 | 0.0148 | 0.00166 | -3.34 | -1.56202 | -0.589 |
| IL6R | ILMN_1754753 | 3.04E-06 | 1.95E-08 | -6.78 | 9.26639 | -0.59 |
| NUDT16L1 | ILMN_1735415 | 0.0334 | 0.00482 | -2.96 | -2.54497 | -0.59 |
| PLAU | ILMN_1656057 | 0.0536 | 0.00912 | -2.72 | -3.12404 | -0.59 |
| PFN1 | ILMN_1712950 | 2.31E-05 | 3.45E-07 | -5.95 | 6.49801 | -0.592 |
| FERMT3 | ILMN_2366334 | 0.000101 | 2.46E-06 | -5.38 | 4.61068 | -0.592 |
| PLEKHO1 | ILMN_1694213 | 0.00158 | 8.67E-05 | -4.31 | 1.20848 | -0.592 |
| LUC7L3 | ILMN_1728180 | 0.00334 | 0.000238 | -3.99 | 0.25443 | -0.592 |
| SHPK | ILMN_2216918 | 0.0178 | 0.0021 | -3.26 | -1.78369 | -0.592 |
| NCOR2 | ILMN_1698419 | 0.000168 | 4.65E-06 | -5.19 | 3.99922 | -0.593 |
| EFTUD2 | ILMN_1738819 | 5.73E-07 | 2.08E-09 | -7.43 | 11.42095 | -0.594 |
| VAV1 | ILMN_1717334 | 2.32E-05 | 3.47E-07 | -5.95 | 6.49357 | -0.594 |
| SPHK2 | ILMN_1729281 | 2.52E-05 | 4.01E-07 | -5.91 | 6.35228 | -0.594 |
| CD300LF | ILMN_2112357 | 4.33E-05 | 8.06E-07 | -5.7 | 5.68284 | -0.594 |
| INPPL1 | ILMN_1728426 | 0.000169 | 4.68E-06 | -5.19 | 3.9933 | -0.594 |
| DENND4B | ILMN_3245066 | 1.32E-07 | 3.16E-10 | -7.98 | 13.23194 | -0.595 |
| SYK | ILMN_2059549 | 0.000229 | 7.05E-06 | -5.07 | 3.60189 | -0.596 |
| TMC6 | ILMN_1794677 | 0.00046 | 1.72E-05 | -4.8 | 2.74669 | -0.596 |
| MYADM | ILMN_2350574 | 0.000984 | 4.67E-05 | -4.5 | 1.79609 | -0.596 |
| ZNF564 | ILMN_2217212 | 0.00297 | 0.000203 | -4.04 | 0.40565 | -0.596 |
| CAMK2G | ILMN_1809695 | 0.00622 | 0.00053 | -3.73 | -0.4989 | -0.596 |
| BID | ILMN_2372413 | 0.00023 | 7.10E-06 | -5.06 | 3.59509 | -0.597 |
| OPA3 | ILMN_2284591 | 0.000516 | 1.98E-05 | -4.76 | 2.61235 | -0.597 |
| ARHGEF18 | ILMN_1664016 | 0.00119 | 6.00E-05 | -4.42 | 1.55886 | -0.597 |
| PAQR7 | ILMN_1746618 | 0.028 | 0.00382 | -3.05 | -2.33314 | -0.597 |
| GDPD3 | ILMN_1774901 | 0.000894 | 4.11E-05 | -4.54 | 1.91847 | -0.598 |
| STK11 | ILMN_1751871 | 0.000238 | 7.42E-06 | -5.05 | 3.55217 | -0.599 |
| LST1 | ILMN_2345353 | 0.000386 | 1.37E-05 | -4.87 | 2.96456 | -0.599 |
| BCL11A | ILMN_1752899 | 0.00605 | 0.000511 | -3.74 | -0.4658 | -0.599 |
| PTPRN2 | ILMN_1728844 | 0.00634 | 0.000542 | -3.72 | -0.52106 | -0.599 |
| SLC2A5 | ILMN_1671337 | 0.0074 | 0.00066 | -3.65 | -0.70492 | -0.599 |
| HINFP | ILMN_2353697 | 5.12E-06 | 4.39E-08 | -6.55 | 8.48326 | -0.6 |
| CDK13 | ILMN_2356068 | 1.57E-05 | 2.00E-07 | -6.11 | 7.02258 | -0.6 |
| RNF121 | ILMN_2356031 | 0.000721 | 3.13E-05 | -4.62 | 2.17829 | -0.601 |
| MAPRE2 | ILMN_1695276 | 0.0022 | 0.000137 | -4.16 | 0.77512 | -0.601 |
| DPEP3 | ILMN_1731275 | 0.0228 | 0.00292 | -3.14 | -2.086 | -0.601 |
| EML2 | ILMN_3240541 | 0.00257 | 0.000168 | -4.1 | 0.58056 | -0.602 |
| TGIF2 | ILMN_1709044 | 0.00798 | 0.000732 | -3.62 | -0.80229 | -0.602 |
| MAP7 | ILMN_1712719 | 0.0167 | 0.00194 | -3.29 | -1.71009 | -0.602 |
| NSUN5 | ILMN_1751958 | 0.0694 | 0.013 | -2.59 | -3.43815 | -0.602 |
| CCR7 | ILMN_1715131 | 3.00E-04 | 9.93E-06 | -4.96 | 3.2734 | -0.604 |
| NCOA5 | ILMN_1770035 | 0.00213 | 0.000131 | -4.18 | 0.81859 | -0.604 |
| FLAD1 | ILMN_1663667 | 0.00651 | 0.000561 | -3.71 | -0.55323 | -0.604 |
| BOD1L1 | ILMN_1695856 | 0.00733 | 0.000653 | -3.66 | -0.69429 | -0.604 |
| ST6GAL1 | ILMN_1756501 | 0.00952 | 0.000921 | -3.54 | -1.01625 | -0.604 |
| ADAP1 | ILMN_3247424 | 0.0107 | 0.00108 | -3.49 | -1.16458 | -0.604 |
| PSME3 | ILMN_1800975 | 0.0218 | 0.00275 | -3.16 | -2.03147 | -0.604 |
| UPB1 | ILMN_1678690 | 0.0376 | 0.00563 | -2.91 | -2.68633 | -0.604 |
| AP5M1 | ILMN_2204297 | 3.96E-05 | 7.10E-07 | -5.74 | 5.80452 | -0.606 |
| SMAD7 | ILMN_2203896 | 0.00117 | 5.90E-05 | -4.42 | 1.5739 | -0.606 |
| GPX3 | ILMN_1726666 | 0.0435 | 0.00682 | -2.83 | -2.86121 | -0.607 |
| CEP19 | ILMN_1665217 | 0.00206 | 0.000126 | -4.19 | 0.85765 | -0.608 |
| TNFSF12 | ILMN_1683700 | 0.00745 | 0.000666 | -3.65 | -0.71293 | -0.61 |
| SLC38A10 | ILMN_2277419 | 0.0172 | 0.00201 | -3.28 | -1.7422 | -0.61 |
| NCOR2 | ILMN_2340052 | 9.23E-05 | 2.17E-06 | -5.41 | 4.73043 | -0.611 |
| PADI4 | ILMN_1807529 | 0.000106 | 2.61E-06 | -5.36 | 4.55386 | -0.611 |
| NHSL2 | ILMN_1748694 | 0.0179 | 0.00213 | -3.26 | -1.79341 | -0.611 |
| NFE2 | ILMN_1711786 | 0.1 | 0.0212 | -2.39 | -3.87501 | -0.611 |
| SIAH1 | ILMN_2380566 | 1.39E-06 | 6.40E-09 | -7.11 | 10.33666 | -0.612 |
| RNF216 | ILMN_1729980 | 0.00398 | 0.000297 | -3.92 | 0.04584 | -0.612 |
| LST1 | ILMN_1688373 | 0.00022 | 6.68E-06 | -5.08 | 3.65369 | -0.614 |
| MTHFR | ILMN_1734830 | 0.0192 | 0.00232 | -3.23 | -1.87416 | -0.614 |
| PTP4A3 | ILMN_2359710 | 0.000331 | 1.12E-05 | -4.93 | 3.15705 | -0.615 |
| FADD | ILMN_1758658 | 0.000394 | 1.40E-05 | -4.86 | 2.94252 | -0.616 |
| RAP1GAP2 | ILMN_2041236 | 1.68E-05 | 2.27E-07 | -6.07 | 6.90256 | -0.618 |
| CYSLTR1 | ILMN_1733276 | 0.00136 | 7.13E-05 | -4.37 | 1.39435 | -0.619 |
| LTBP2 | ILMN_3248591 | 0.00274 | 0.000181 | -4.07 | 0.50988 | -0.619 |
| TNK2 | ILMN_1669703 | 0.00349 | 0.000252 | -3.97 | 0.2013 | -0.619 |
| PSME3 | ILMN_2346573 | 0.0221 | 0.0028 | -3.16 | -2.04798 | -0.62 |
| ANKDD1A | ILMN_1813139 | 4.28E-05 | 7.92E-07 | -5.71 | 5.69951 | -0.622 |
| RASAL3 | ILMN_3238803 | 0.000814 | 3.63E-05 | -4.57 | 2.03582 | -0.622 |
| TOX2 | ILMN_2082209 | 0.00134 | 7.00E-05 | -4.37 | 1.41252 | -0.622 |
| NLRP12 | ILMN_1758735 | 2.33E-05 | 3.49E-07 | -5.95 | 6.48662 | -0.624 |
| ORAI3 | ILMN_1736628 | 0.00755 | 0.00068 | -3.64 | -0.7329 | -0.625 |
| FAM189B | ILMN_1795026 | 0.000932 | 4.36E-05 | -4.52 | 1.86145 | -0.626 |
| TBC1D2B | ILMN_2064606 | 0.0589 | 0.0103 | -2.67 | -3.23681 | -0.626 |
| C16orf74 | ILMN_1806149 | 0.00622 | 0.00053 | -3.73 | -0.49895 | -0.627 |
| RAB37 | ILMN_2255579 | 0.0132 | 0.00143 | -3.39 | -1.42538 | -0.627 |
| EPB41 | ILMN_1667408 | 0.00643 | 0.000552 | -3.71 | -0.53795 | -0.629 |
| ASCC2 | ILMN_1679919 | 5.46E-06 | 4.81E-08 | -6.52 | 8.39537 | -0.63 |
| APEX2 | ILMN_1652505 | 9.59E-05 | 2.29E-06 | -5.4 | 4.67794 | -0.63 |
| BAZ2B | ILMN_1720850 | 0.000586 | 2.35E-05 | -4.71 | 2.45092 | -0.63 |
| AP5M1 | ILMN_3237641 | 0.0011 | 5.44E-05 | -4.45 | 1.65242 | -0.63 |
| HDGF | ILMN_1765621 | 0.000239 | 7.47E-06 | -5.05 | 3.54592 | -0.632 |
| RNF5 | ILMN_2044927 | 0.00131 | 6.82E-05 | -4.38 | 1.43737 | -0.633 |
| ADGRE1 | ILMN_1780601 | 0.00348 | 0.00025 | -3.97 | 0.20686 | -0.633 |
| SLC4A7 | ILMN_2200917 | 0.0221 | 0.0028 | -3.16 | -2.04708 | -0.635 |
| NINJ2 | ILMN_1731745 | 7.62E-05 | 1.70E-06 | -5.49 | 4.96681 | -0.636 |
| PRKCB | ILMN_3299520 | 0.000117 | 2.90E-06 | -5.33 | 4.45292 | -0.64 |
| TRAF3IP2 | ILMN_1701514 | 0.000405 | 1.45E-05 | -4.85 | 2.90945 | -0.64 |
| MGAT3 | ILMN_1853824 | 5.70E-05 | 1.17E-06 | -5.6 | 5.32586 | -0.642 |
| TNFRSF12A | ILMN_1689004 | 0.0213 | 0.00267 | -3.18 | -2.00467 | -0.643 |
| ZNF845 | ILMN_3237579 | 5.08E-05 | 1.01E-06 | -5.64 | 5.46882 | -0.644 |
| TTLL12 | ILMN_1663113 | 0.000153 | 4.05E-06 | -5.23 | 4.1316 | -0.644 |
| NELFCD | ILMN_1663954 | 0.00201 | 0.000121 | -4.2 | 0.89045 | -0.645 |
| GBA2 | ILMN_1674560 | 1.12E-05 | 1.27E-07 | -6.24 | 7.45958 | -0.646 |
| FHOD1 | ILMN_1651776 | 3.24E-05 | 5.43E-07 | -5.82 | 6.06195 | -0.646 |
| PPP1R9B | ILMN_1688865 | 0.000898 | 4.14E-05 | -4.53 | 1.91038 | -0.647 |
| ZBTB46 | ILMN_1710092 | 0.000279 | 9.14E-06 | -4.99 | 3.35307 | -0.648 |
| FAM110A | ILMN_2323944 | 0.000333 | 1.13E-05 | -4.93 | 3.15056 | -0.648 |
| FBXW7 | ILMN_1668634 | 0.000585 | 2.34E-05 | -4.71 | 2.45626 | -0.649 |
| SULF2 | ILMN_2345142 | 0.0015 | 8.04E-05 | -4.33 | 1.28111 | -0.649 |
| LTB | ILMN_2376204 | 0.00116 | 5.83E-05 | -4.43 | 1.58584 | -0.65 |
| TRAF3 | ILMN_2383774 | 0.00745 | 0.000665 | -3.65 | -0.71226 | -0.652 |
| CMTM3 | ILMN_1705442 | 1.50E-06 | 7.49E-09 | -7.06 | 10.18618 | -0.654 |
| PDXK | ILMN_1672504 | 1.98E-05 | 2.78E-07 | -6.01 | 6.70584 | -0.654 |
| IFFO1 | ILMN_2348268 | 8.33E-05 | 1.91E-06 | -5.45 | 4.8514 | -0.656 |
| EIF2B5 | ILMN_1688534 | 0.000309 | 1.03E-05 | -4.95 | 3.2421 | -0.656 |
| PRKCB | ILMN_1716563 | 0.000245 | 7.67E-06 | -5.04 | 3.52053 | -0.657 |
| DCAF12 | ILMN_1786328 | 2.92E-06 | 1.84E-08 | -6.8 | 9.31762 | -0.658 |
| NHSL2 | ILMN_1815644 | 0.00803 | 0.000739 | -3.62 | -0.81023 | -0.659 |
| ESRRA | ILMN_1774272 | 1.70E-07 | 4.32E-10 | -7.89 | 12.92977 | -0.66 |
| DOK2 | ILMN_1791211 | 0.000547 | 2.13E-05 | -4.74 | 2.54496 | -0.66 |
| AGO3 | ILMN_1761049 | 0.00166 | 9.27E-05 | -4.28 | 1.14523 | -0.66 |
| TCF20 | ILMN_1789103 | 0.00171 | 9.73E-05 | -4.27 | 1.09914 | -0.66 |
| SIGLEC10 | ILMN_1655549 | 0.00527 | 0.000427 | -3.8 | -0.2969 | -0.66 |
| ARMC10 | ILMN_2052598 | 0.000156 | 4.17E-06 | -5.22 | 4.1041 | -0.661 |
| TGM3 | ILMN_1786847 | 0.0617 | 0.011 | -2.65 | -3.29398 | -0.661 |
| NSUN5 | ILMN_2408400 | 0.0571 | 0.00995 | -2.69 | -3.20196 | -0.663 |
| BFAR | ILMN_1814808 | 6.51E-05 | 1.39E-06 | -5.55 | 5.16179 | -0.664 |
| VSTM1 | ILMN_1763455 | 0.000282 | 9.23E-06 | -4.99 | 3.34335 | -0.665 |
| FGL2 | ILMN_1693009 | 0.000259 | 8.31E-06 | -5.02 | 3.44382 | -0.666 |
| C7orf25 | ILMN_1751051 | 0.000591 | 2.38E-05 | -4.7 | 2.43815 | -0.667 |
| BMP2K | ILMN_1814200 | 0.00709 | 0.000625 | -3.67 | -0.65412 | -0.668 |
| POLG2 | ILMN_1671004 | 0.00168 | 9.50E-05 | -4.28 | 1.12259 | -0.669 |
| ABCC5 | ILMN_1706531 | 0.00136 | 7.10E-05 | -4.37 | 1.39838 | -0.671 |
| CLCC1 | ILMN_1671933 | 0.00366 | 0.000266 | -3.95 | 0.14928 | -0.671 |
| UGGT1 | ILMN_2360291 | 0.00046 | 1.73E-05 | -4.8 | 2.74511 | -0.672 |
| UPF3B | ILMN_1798163 | 7.34E-07 | 2.93E-09 | -7.33 | 11.08763 | -0.673 |
| FMNL3 | ILMN_2395214 | 4.75E-05 | 9.14E-07 | -5.67 | 5.56111 | -0.674 |
| CEP170 | ILMN_2329885 | 2.92E-06 | 1.84E-08 | -6.8 | 9.32092 | -0.675 |
| SH2D3C | ILMN_2321648 | 1.93E-05 | 2.69E-07 | -6.02 | 6.7363 | -0.676 |
| SRPK2 | ILMN_1669215 | 0.0277 | 0.00379 | -3.05 | -2.32518 | -0.676 |
| PRKCD | ILMN_1801105 | 7.26E-09 | 3.76E-12 | -9.32 | 17.47937 | -0.678 |
| PLEKHO2 | ILMN_1689968 | 1.21E-08 | 1.14E-11 | -8.98 | 16.41706 | -0.678 |
| SMAD7 | ILMN_2203891 | 8.00E-06 | 8.14E-08 | -6.37 | 7.88857 | -0.678 |
| DVL3 | ILMN_2137464 | 3.36E-08 | 4.91E-11 | -8.54 | 15.01969 | -0.679 |
| TMEM218 | ILMN_2395856 | 0.00682 | 0.000594 | -3.69 | -0.60668 | -0.679 |
| PHC1 | ILMN_1796710 | 0.0435 | 0.00683 | -2.83 | -2.86242 | -0.68 |
| CYP4F3 | ILMN_1736190 | 6.77E-07 | 2.56E-09 | -7.37 | 11.21994 | -0.681 |
| OPRL1 | ILMN_2400922 | 0.000866 | 3.93E-05 | -4.55 | 1.96086 | -0.681 |
| NOMO1 | ILMN_2126957 | 0.0028 | 0.000186 | -4.06 | 0.48462 | -0.681 |
| CEBPA | ILMN_1715715 | 0.001 | 4.80E-05 | -4.49 | 1.77158 | -0.682 |
| SPAG7 | ILMN_1684446 | 2.46E-05 | 3.82E-07 | -5.92 | 6.40097 | -0.683 |
| PRCP | ILMN_1769091 | 4.29E-06 | 3.21E-08 | -6.64 | 8.7835 | -0.684 |
| DOCK10 | ILMN_1702301 | 4.48E-05 | 8.40E-07 | -5.69 | 5.64306 | -0.685 |
| S100A4 | ILMN_1684306 | 7.43E-05 | 1.62E-06 | -5.5 | 5.01032 | -0.685 |
| SOCS4 | ILMN_2413259 | 0.000108 | 2.68E-06 | -5.35 | 4.53019 | -0.685 |
| CAMK1 | ILMN_2140990 | 0.00115 | 5.76E-05 | -4.43 | 1.59651 | -0.685 |
| RBM4B | ILMN_1743104 | 0.000759 | 3.32E-05 | -4.6 | 2.12286 | -0.686 |
| REEP5 | ILMN_1758941 | 0.00216 | 0.000134 | -4.17 | 0.79746 | -0.687 |
| BICD2 | ILMN_2380418 | 9.20E-05 | 2.16E-06 | -5.42 | 4.73527 | -0.688 |
| NFATC3 | ILMN_2360028 | 0.00195 | 0.000116 | -4.21 | 0.93374 | -0.69 |
| OXER1 | ILMN_1765061 | 0.000674 | 2.84E-05 | -4.65 | 2.27115 | -0.691 |
| RHOB | ILMN_1802205 | 3.69E-06 | 2.57E-08 | -6.7 | 8.99694 | -0.692 |
| INPP1 | ILMN_1667239 | 0.000148 | 3.89E-06 | -5.24 | 4.17063 | -0.695 |
| HAUS8 | ILMN_3176989 | 3.03E-09 | 1.28E-12 | -9.65 | 18.50541 | -0.696 |
| NLRP12 | ILMN_1716105 | 2.36E-05 | 3.57E-07 | -5.94 | 6.46616 | -0.697 |
| FAH | ILMN_1781536 | 0.000205 | 6.07E-06 | -5.11 | 3.74531 | -0.697 |
| MYPOP | ILMN_1704793 | 5.61E-07 | 1.98E-09 | -7.45 | 11.46572 | -0.698 |
| PSG9 | ILMN_1801776 | 0.0466 | 0.00751 | -2.8 | -2.94821 | -0.698 |
| NDST1 | ILMN_1807283 | 9.63E-05 | 2.31E-06 | -5.4 | 4.67007 | -0.699 |
| C7orf61 | ILMN_2226691 | 0.00091 | 4.21E-05 | -4.53 | 1.89519 | -0.699 |
| RAB24 | ILMN_1799765 | 0.00211 | 0.000129 | -4.18 | 0.83238 | -0.699 |
| FUCA1 | ILMN_1752728 | 0.00492 | 0.000391 | -3.83 | -0.21425 | -0.699 |
| AGO1 | ILMN_1671326 | 0.00013 | 3.30E-06 | -5.29 | 4.3296 | -0.7 |
| TRIM4 | ILMN_2323385 | 2.96E-08 | 3.91E-11 | -8.61 | 15.23826 | -0.701 |
| NOV | ILMN_1787186 | 0.00639 | 0.000548 | -3.72 | -0.53001 | -0.704 |
| PTPN7 | ILMN_1715214 | 9.59E-05 | 2.30E-06 | -5.4 | 4.67602 | -0.706 |
| PCED1B | ILMN_1712431 | 0.0104 | 0.00104 | -3.5 | -1.13061 | -0.706 |
| FAM53B | ILMN_1704571 | 0.0105 | 0.00106 | -3.5 | -1.1492 | -0.706 |
| LYL1 | ILMN_2216582 | 4.46E-05 | 8.33E-07 | -5.69 | 5.65042 | -0.707 |
| PAK2 | ILMN_1659878 | 8.08E-05 | 1.83E-06 | -5.46 | 4.89246 | -0.707 |
| TIAF1 | ILMN_2055634 | 0.00252 | 0.000164 | -4.11 | 0.60566 | -0.707 |
| FCHO1 | ILMN_1654571 | 0.00096 | 4.51E-05 | -4.51 | 1.82925 | -0.709 |
| TNFRSF10B | ILMN_1699265 | 0.000418 | 1.52E-05 | -4.84 | 2.86474 | -0.71 |
| HIC1 | ILMN_1738825 | 0.00316 | 0.00022 | -4.01 | 0.32669 | -0.71 |
| CAMP | ILMN_1688580 | 7.54E-05 | 1.67E-06 | -5.49 | 4.98296 | -0.712 |
| WDFY4 | ILMN_3236551 | 0.00174 | 1.00E-04 | -4.26 | 1.07306 | -0.712 |
| LTB | ILMN_2376205 | 0.00016 | 4.32E-06 | -5.21 | 4.06979 | -0.713 |
| MAP7 | ILMN_2216815 | 0.0241 | 0.00317 | -3.11 | -2.16084 | -0.714 |
| ATP6V0A1 | ILMN_1752579 | 2.49E-05 | 3.89E-07 | -5.92 | 6.38244 | -0.717 |
| PELO | ILMN_1770811 | 7.05E-07 | 2.72E-09 | -7.35 | 11.16071 | -0.719 |
| ZNF146 | ILMN_2054442 | 4.19E-05 | 7.66E-07 | -5.72 | 5.73123 | -0.719 |
| PREB | ILMN_1733930 | 0.000454 | 1.70E-05 | -4.8 | 2.76202 | -0.72 |
| CLC | ILMN_1654875 | 0.000149 | 3.92E-06 | -5.24 | 4.16335 | -0.721 |
| SIAH1 | ILMN_1711627 | 9.64E-09 | 7.27E-12 | -9.11 | 16.84927 | -0.722 |
| ANPEP | ILMN_1763837 | 0.000149 | 3.92E-06 | -5.24 | 4.16493 | -0.723 |
| SLC9A1 | ILMN_1800425 | 3.92E-06 | 2.86E-08 | -6.67 | 8.8958 | -0.725 |
| AGPAT1 | ILMN_1679520 | 0.00185 | 0.000108 | -4.24 | 1.00318 | -0.726 |
| NQO2 | ILMN_1712918 | 2.03E-05 | 2.93E-07 | -6 | 6.65601 | -0.727 |
| RGS14 | ILMN_1696828 | 0.00488 | 0.000387 | -3.83 | -0.20386 | -0.729 |
| CAPG | ILMN_1655821 | 0.000417 | 1.52E-05 | -4.84 | 2.86969 | -0.73 |
| SNAI3 | ILMN_1690262 | 0.000732 | 3.18E-05 | -4.61 | 2.16236 | -0.73 |
| INPP5B | ILMN_1810116 | 0.000372 | 1.31E-05 | -4.88 | 3.0089 | -0.733 |
| KCTD12 | ILMN_2229649 | 0.0117 | 0.00122 | -3.45 | -1.27481 | -0.733 |
| CUEDC1 | ILMN_1676665 | 0.00202 | 0.000123 | -4.2 | 0.87948 | -0.734 |
| LPXN | ILMN_1742789 | 1.39E-06 | 6.24E-09 | -7.11 | 10.36159 | -0.735 |
| SLC35B3 | ILMN_1682910 | 1.22E-05 | 1.42E-07 | -6.21 | 7.35112 | -0.735 |
| AMACR | ILMN_1792741 | 0.00783 | 0.000714 | -3.63 | -0.77873 | -0.735 |
| HILPDA | ILMN_1659990 | 0.0161 | 0.00186 | -3.3 | -1.66926 | -0.735 |
| TOX2 | ILMN_3298423 | 0.00435 | 0.000334 | -3.88 | -0.06593 | -0.736 |
| SPRY2 | ILMN_2089329 | 0.00109 | 5.33E-05 | -4.46 | 1.67186 | -0.737 |
| ZNF641 | ILMN_1731666 | 5.91E-06 | 5.32E-08 | -6.49 | 8.29843 | -0.739 |
| PAK1 | ILMN_1767365 | 0.000259 | 8.29E-06 | -5.02 | 3.44629 | -0.739 |
| SIGLEC5 | ILMN_1740298 | 2.41E-05 | 3.67E-07 | -5.93 | 6.43895 | -0.74 |
| NSF | ILMN_2330845 | 3.96E-05 | 7.09E-07 | -5.74 | 5.80482 | -0.74 |
| STK38L | ILMN_1755792 | 4.82E-05 | 9.47E-07 | -5.66 | 5.52758 | -0.74 |
| RPGRIP1 | ILMN_1803739 | 0.0653 | 0.0118 | -2.62 | -3.35815 | -0.741 |
| KLHL18 | ILMN_1655608 | 2.74E-05 | 4.46E-07 | -5.88 | 6.25086 | -0.742 |
| SIGLEC9 | ILMN_1795236 | 5.53E-05 | 1.12E-06 | -5.61 | 5.36724 | -0.743 |
| HVCN1 | ILMN_1815168 | 0.0127 | 0.00136 | -3.41 | -1.37668 | -0.743 |
| MPEG1 | ILMN_1752355 | 0.0056 | 0.000461 | -3.77 | -0.36887 | -0.745 |
| AP3S2 | ILMN_1731596 | 0.000603 | 2.45E-05 | -4.69 | 2.41166 | -0.746 |
| EIF4ENIF1 | ILMN_1794967 | 0.000502 | 1.93E-05 | -4.77 | 2.64107 | -0.749 |
| FAR1 | ILMN_2143250 | 2.02E-05 | 2.87E-07 | -6 | 6.67546 | -0.751 |
| DUSP6 | ILMN_1677466 | 0.0133 | 0.00145 | -3.39 | -1.43745 | -0.752 |
| BRPF3 | ILMN_1658800 | 5.40E-08 | 8.90E-11 | -8.36 | 14.44837 | -0.753 |
| GNMT | ILMN_1736238 | 0.00146 | 7.82E-05 | -4.34 | 1.3069 | -0.754 |
| CD4 | ILMN_1727284 | 0.000972 | 4.61E-05 | -4.5 | 1.80966 | -0.756 |
| GPAT3 | ILMN_1794875 | 0.00021 | 6.27E-06 | -5.1 | 3.71381 | -0.757 |
| PIK3R2 | ILMN_1726565 | 0.000338 | 1.15E-05 | -4.92 | 3.13485 | -0.757 |
| BRI3BP | ILMN_1797693 | 0.00312 | 0.000216 | -4.02 | 0.34479 | -0.758 |
| PRCP | ILMN_2367215 | 3.73E-06 | 2.61E-08 | -6.7 | 8.98156 | -0.76 |
| RCC2 | ILMN_1720124 | 2.19E-05 | 3.19E-07 | -5.97 | 6.57281 | -0.76 |
| TAX1BP3 | ILMN_1803392 | 0.0138 | 0.00151 | -3.38 | -1.47438 | -0.761 |
| LOC606724 | ILMN_1731804 | 1.41E-06 | 6.61E-09 | -7.1 | 10.306 | -0.762 |
| SIN3B | ILMN_1788315 | 2.49E-05 | 3.90E-07 | -5.92 | 6.3805 | -0.762 |
| GATSL3 | ILMN_2098418 | 5.89E-05 | 1.22E-06 | -5.58 | 5.28405 | -0.762 |
| BCORL1 | ILMN_1767015 | 1.54E-06 | 7.83E-09 | -7.05 | 10.14334 | -0.763 |
| FAM216A | ILMN_2180371 | 0.000672 | 2.83E-05 | -4.65 | 2.2751 | -0.763 |
| NFATC3 | ILMN_1755983 | 0.00016 | 4.31E-06 | -5.21 | 4.07404 | -0.766 |
| RAB37 | ILMN_2243912 | 0.00363 | 0.000264 | -3.95 | 0.1559 | -0.768 |
| RXRA | ILMN_1687315 | 2.20E-09 | 8.28E-13 | -9.78 | 18.92373 | -0.769 |
| PIEZO1 | ILMN_1752249 | 0.000279 | 9.09E-06 | -4.99 | 3.35848 | -0.769 |
| PTPN22 | ILMN_1715885 | 0.00109 | 5.35E-05 | -4.45 | 1.6667 | -0.769 |
| SNRNP25 | ILMN_1801118 | 0.00792 | 0.000726 | -3.62 | -0.79402 | -0.77 |
| CLK1 | ILMN_1652790 | 0.000161 | 4.39E-06 | -5.21 | 4.05622 | -0.772 |
| AP4B1 | ILMN_1669377 | 0.000969 | 4.59E-05 | -4.5 | 1.81422 | -0.772 |
| PGLYRP2 | ILMN_3307921 | 0.00403 | 0.000302 | -3.91 | 0.02848 | -0.772 |
| SENP6 | ILMN_2054233 | 1.56E-05 | 1.96E-07 | -6.11 | 7.04122 | -0.775 |
| BRI3BP | ILMN_1800619 | 0.00223 | 0.00014 | -4.16 | 0.75485 | -0.779 |
| NUP58 | ILMN_2378316 | 0.000155 | 4.14E-06 | -5.22 | 4.11136 | -0.783 |
| SH3BP5L | ILMN_1665384 | 0.00124 | 6.36E-05 | -4.4 | 1.50333 | -0.783 |
| ARRB1 | ILMN_1730620 | 0.000126 | 3.17E-06 | -5.3 | 4.36601 | -0.784 |
| MYO18A | ILMN_1780560 | 0.00045 | 1.67E-05 | -4.81 | 2.77687 | -0.784 |
| FAM213B | ILMN_3243682 | 0.0656 | 0.0119 | -2.62 | -3.36523 | -0.784 |
| PTPN22 | ILMN_1695640 | 0.000446 | 1.65E-05 | -4.81 | 2.79135 | -0.785 |
| ENG | ILMN_1760778 | 0.000207 | 6.18E-06 | -5.11 | 3.7276 | -0.786 |
| PRDX1 | ILMN_2366391 | 0.00181 | 0.000104 | -4.25 | 1.03275 | -0.788 |
| EDRF1 | ILMN_1791656 | 3.20E-08 | 4.52E-11 | -8.56 | 15.09894 | -0.789 |
| CYBB | ILMN_1682312 | 0.00697 | 0.00061 | -3.68 | -0.63148 | -0.789 |
| ZNF182 | ILMN_2337835 | 0.000151 | 3.98E-06 | -5.24 | 4.14863 | -0.792 |
| MED22 | ILMN_1697218 | 0.000705 | 3.02E-05 | -4.63 | 2.21261 | -0.792 |
| PAFAH2 | ILMN_1682919 | 7.34E-07 | 2.91E-09 | -7.33 | 11.0943 | -0.793 |
| RUNX3 | ILMN_1787461 | 1.64E-05 | 2.18E-07 | -6.08 | 6.94053 | -0.795 |
| HVCN1 | ILMN_2365248 | 0.00832 | 0.000772 | -3.6 | -0.8518 | -0.795 |
| ESYT1 | ILMN_1761159 | 0.00039 | 1.39E-05 | -4.86 | 2.95418 | -0.798 |
| AMACR | ILMN_1759670 | 0.00112 | 5.56E-05 | -4.44 | 1.63139 | -0.799 |
| PTP4A3 | ILMN_1662427 | 2.22E-05 | 3.25E-07 | -5.97 | 6.55449 | -0.8 |
| PNPLA1 | ILMN_1808241 | 0.000912 | 4.23E-05 | -4.53 | 1.89108 | -0.8 |
| OSCAR | ILMN_2367418 | 4.45E-06 | 3.43E-08 | -6.62 | 8.71905 | -0.805 |
| SLC23A2 | ILMN_1746578 | 0.000124 | 3.11E-06 | -5.31 | 4.38478 | -0.806 |
| SIGLEC7 | ILMN_1681415 | 0.00144 | 7.68E-05 | -4.34 | 1.32402 | -0.807 |
| TMX2 | ILMN_1799367 | 0.000538 | 2.08E-05 | -4.74 | 2.56933 | -0.808 |
| WNK1 | ILMN_1876924 | 3.78E-06 | 2.67E-08 | -6.69 | 8.9608 | -0.809 |
| ENC1 | ILMN_1779147 | 0.00112 | 5.52E-05 | -4.44 | 1.63742 | -0.812 |
| PGM2 | ILMN_1673543 | 3.97E-05 | 7.15E-07 | -5.74 | 5.79785 | -0.813 |
| TRIOBP | ILMN_1735788 | 0.00124 | 6.37E-05 | -4.4 | 1.50121 | -0.815 |
| MYB | ILMN_1711894 | 0.000103 | 2.52E-06 | -5.37 | 4.58802 | -0.818 |
| C11orf21 | ILMN_3235922 | 0.00115 | 5.72E-05 | -4.43 | 1.60311 | -0.82 |
| PTGDR2 | ILMN_1703326 | 6.89E-06 | 6.68E-08 | -6.43 | 8.07817 | -0.822 |
| WNK1 | ILMN_1753165 | 8.37E-06 | 8.54E-08 | -6.36 | 7.84141 | -0.827 |
| BRI3BP | ILMN_1693410 | 2.87E-05 | 4.73E-07 | -5.86 | 6.19438 | -0.827 |
| OSCAR | ILMN_1741917 | 4.40E-06 | 3.36E-08 | -6.63 | 8.74107 | -0.828 |
| GSE1 | ILMN_1807767 | 0.000198 | 5.80E-06 | -5.12 | 3.78903 | -0.829 |
| THUMPD3 | ILMN_1671902 | 1.17E-05 | 1.33E-07 | -6.23 | 7.41208 | -0.831 |
| PUS7 | ILMN_1779353 | 0.00504 | 0.000405 | -3.82 | -0.24697 | -0.831 |
| VWF | ILMN_1752755 | 0.0282 | 0.00387 | -3.04 | -2.34382 | -0.831 |
| PTP4A3 | ILMN_1769779 | 5.93E-05 | 1.23E-06 | -5.58 | 5.27305 | -0.832 |
| MLEC | ILMN_1657495 | 4.61E-06 | 3.65E-08 | -6.6 | 8.66081 | -0.834 |
| CHD9 | ILMN_1762972 | 5.69E-08 | 1.02E-10 | -8.32 | 14.32064 | -0.835 |
| NUP85 | ILMN_1669635 | 1.00E-04 | 2.41E-06 | -5.38 | 4.63134 | -0.835 |
| NHS | ILMN_1715864 | 0.00161 | 8.95E-05 | -4.3 | 1.17889 | -0.835 |
| HINFP | ILMN_1667453 | 3.68E-06 | 2.52E-08 | -6.71 | 9.01722 | -0.838 |
| ARMC7 | ILMN_1797298 | 6.97E-06 | 6.79E-08 | -6.42 | 8.06271 | -0.838 |
| POLR3K | ILMN_1801664 | 0.0036 | 0.000261 | -3.96 | 0.16527 | -0.838 |
| SIGLEC7 | ILMN_2409384 | 9.39E-05 | 2.22E-06 | -5.41 | 4.70965 | -0.839 |
| KCTD12 | ILMN_1742332 | 0.00125 | 6.45E-05 | -4.4 | 1.49046 | -0.84 |
| ABHD8 | ILMN_1712707 | 0.000346 | 1.19E-05 | -4.91 | 3.09964 | -0.843 |
| KIF3B | ILMN_2081398 | 1.68E-11 | 1.58E-15 | -11.8 | 24.85057 | -0.845 |
| CLCF1 | ILMN_1661197 | 0.00246 | 0.000159 | -4.12 | 0.63579 | -0.846 |
| HOMER3 | ILMN_1811579 | 0.00893 | 0.000846 | -3.57 | -0.9376 | -0.846 |
| SLC24A3 | ILMN_1663519 | 0.000137 | 3.52E-06 | -5.27 | 4.26707 | -0.847 |
| ENO3 | ILMN_1678904 | 0.000565 | 2.23E-05 | -4.72 | 2.50115 | -0.849 |
| PROC | ILMN_1687721 | 0.0101 | 0.001 | -3.51 | -1.09732 | -0.851 |
| FAM65A | ILMN_1680037 | 9.59E-05 | 2.29E-06 | -5.4 | 4.68135 | -0.853 |
| ARHGAP33 | ILMN_1678781 | 0.00522 | 0.000423 | -3.8 | -0.28693 | -0.854 |
| FBXO46 | ILMN_2350266 | 5.48E-06 | 4.85E-08 | -6.52 | 8.38675 | -0.855 |
| TNFSF14 | ILMN_1655414 | 1.87E-07 | 5.10E-10 | -7.84 | 12.77045 | -0.857 |
| LRRC75B | ILMN_1737255 | 0.00213 | 0.000132 | -4.17 | 0.81389 | -0.857 |
| KISS1R | ILMN_1673521 | 7.11E-05 | 1.54E-06 | -5.51 | 5.05805 | -0.858 |
| ZNF322 | ILMN_3251742 | 1.48E-06 | 7.30E-09 | -7.07 | 10.21085 | -0.86 |
| AOC3 | ILMN_1782086 | 6.26E-05 | 1.31E-06 | -5.56 | 5.21548 | -0.864 |
| AMPD3 | ILMN_1774447 | 0.00529 | 0.00043 | -3.8 | -0.30324 | -0.865 |
| DEF8 | ILMN_1767509 | 0.00212 | 0.00013 | -4.18 | 0.82223 | -0.875 |
| ADGRG5 | ILMN_1666902 | 1.20E-05 | 1.39E-07 | -6.21 | 7.3734 | -0.879 |
| SURF6 | ILMN_1778032 | 0.000325 | 1.10E-05 | -4.93 | 3.17872 | -0.88 |
| CEP85L | ILMN_2270100 | 0.000249 | 7.85E-06 | -5.03 | 3.49836 | -0.882 |
| NAT9 | ILMN_1776088 | 0.000341 | 1.17E-05 | -4.92 | 3.12059 | -0.883 |
| PTGS1 | ILMN_2339835 | 4.08E-06 | 3.01E-08 | -6.66 | 8.84674 | -0.89 |
| LFNG | ILMN_2360401 | 1.13E-05 | 1.28E-07 | -6.24 | 7.4525 | -0.891 |
| IDH2 | ILMN_1751753 | 0.000603 | 2.46E-05 | -4.69 | 2.40897 | -0.906 |
| PLCB2 | ILMN_1724066 | 4.61E-05 | 8.75E-07 | -5.68 | 5.60372 | -0.912 |
| RAB11FIP1 | ILMN_1692219 | 1.35E-07 | 3.31E-10 | -7.97 | 13.18749 | -0.913 |
| RSF1 | ILMN_1668834 | 8.18E-05 | 1.87E-06 | -5.46 | 4.87555 | -0.916 |
| ZNF770 | ILMN_1734254 | 6.03E-06 | 5.48E-08 | -6.48 | 8.26827 | -0.93 |
| MKL1 | ILMN_1651767 | 0.00034 | 1.16E-05 | -4.92 | 3.12851 | -0.93 |
| QPRT | ILMN_1700268 | 3.45E-05 | 5.94E-07 | -5.79 | 5.97616 | -0.931 |
| LOC100129034 | ILMN_3256478 | 6.47E-05 | 1.37E-06 | -5.55 | 5.1726 | -0.934 |
| ARRB1 | ILMN_2325168 | 0.000162 | 4.42E-06 | -5.2 | 4.04794 | -0.952 |
| TP53I11 | ILMN_1715669 | 5.51E-06 | 4.92E-08 | -6.51 | 8.37219 | -0.953 |
| TNFSF14 | ILMN_1661343 | 9.60E-06 | 1.02E-07 | -6.3 | 7.67006 | -0.959 |
| SYTL1 | ILMN_1750785 | 7.81E-05 | 1.75E-06 | -5.48 | 4.93577 | -0.961 |
| LEP | ILMN_2207504 | 0.000477 | 1.82E-05 | -4.78 | 2.69724 | -0.964 |
| AMACR | ILMN_2367172 | 0.00222 | 0.000139 | -4.16 | 0.76135 | -0.964 |
| RASA3 | ILMN_1654586 | 6.41E-05 | 1.35E-06 | -5.55 | 5.18478 | -0.967 |
| GPR162 | ILMN_1730816 | 0.00239 | 0.000153 | -4.13 | 0.67075 | -0.968 |
| KCNH4 | ILMN_1800396 | 0.00184 | 0.000107 | -4.24 | 1.01099 | -0.977 |
| GJC2 | ILMN_1723048 | 6.13E-06 | 5.66E-08 | -6.47 | 8.23832 | -0.981 |
| CYP4F3 | ILMN_3251260 | 1.00E-07 | 2.31E-10 | -8.08 | 13.53245 | -0.983 |
| BCL11A | ILMN_2255133 | 0.000586 | 2.35E-05 | -4.71 | 2.45145 | -0.984 |
| KCNK6 | ILMN_1701173 | 7.54E-05 | 1.67E-06 | -5.49 | 4.9814 | -0.988 |
| NFATC3 | ILMN_1800976 | 0.00086 | 3.89E-05 | -4.55 | 1.97087 | -0.992 |
| DBN1 | ILMN_1769926 | 9.85E-06 | 1.06E-07 | -6.29 | 7.63251 | -0.994 |
| BCOR | ILMN_1773117 | 2.61E-07 | 7.63E-10 | -7.73 | 12.38412 | -1 |
| LRRC75A | ILMN_2221784 | 4.08E-06 | 3.02E-08 | -6.66 | 8.84386 | -1 |
| OSGEP | ILMN_1753393 | 1.63E-05 | 2.11E-07 | -6.09 | 6.96939 | -1.01 |
| RTN2 | ILMN_1749115 | 1.98E-05 | 2.77E-07 | -6.01 | 6.70927 | -1.01 |
| ABAT | ILMN_2404407 | 6.89E-06 | 6.62E-08 | -6.43 | 8.08717 | -1.02 |
| GFOD2 | ILMN_1744006 | 1.88E-06 | 1.04E-08 | -6.97 | 9.87396 | -1.04 |
| NCKAP5L | ILMN_1763640 | 6.77E-07 | 2.54E-09 | -7.37 | 11.2266 | -1.05 |
| SH3BP1 | ILMN_1692539 | 6.22E-06 | 5.80E-08 | -6.47 | 8.21482 | -1.05 |
| AKR1A1 | ILMN_2380771 | 0.000866 | 3.93E-05 | -4.55 | 1.96089 | -1.05 |
| PTPN22 | ILMN_2246328 | 0.000969 | 4.58E-05 | -4.5 | 1.8145 | -1.05 |
| TRMT1 | ILMN_1812940 | 9.02E-05 | 2.11E-06 | -5.42 | 4.7561 | -1.07 |
| TESC | ILMN_1750181 | 1.11E-05 | 1.24E-07 | -6.25 | 7.47943 | -1.08 |
| UPF3B | ILMN_2397627 | 4.66E-10 | 1.10E-13 | -10.4 | 20.84554 | -1.09 |
| TMEM45B | ILMN_1771120 | 1.56E-05 | 1.96E-07 | -6.11 | 7.03998 | -1.09 |
| UHRF1 | ILMN_1786065 | 0.00236 | 0.000151 | -4.13 | 0.68587 | -1.1 |
| EEPD1 | ILMN_1811616 | 0.000177 | 5.01E-06 | -5.17 | 3.92863 | -1.11 |
| HMGCR | ILMN_1657395 | 2.46E-07 | 7.06E-10 | -7.75 | 12.45892 | -1.12 |
| XXYLT1 | ILMN_1671116 | 0.00191 | 0.000112 | -4.22 | 0.96247 | -1.12 |
| TNFAIP8L1 | ILMN_1684346 | 0.000257 | 8.22E-06 | -5.02 | 3.45516 | -1.14 |
| AKR1A1 | ILMN_1728047 | 0.00105 | 5.11E-05 | -4.47 | 1.71109 | -1.15 |
| TLR9 | ILMN_1679798 | 9.83E-06 | 1.05E-07 | -6.29 | 7.63851 | -1.18 |
| PRR5L | ILMN_1697491 | 0.00538 | 0.000439 | -3.79 | -0.32289 | -1.18 |
| LFNG | ILMN_1663080 | 2.76E-07 | 8.18E-10 | -7.7 | 12.31734 | -1.19 |
| SSH1 | ILMN_1727671 | 1.74E-06 | 9.42E-09 | -6.99 | 9.96438 | -1.19 |
| SH2B3 | ILMN_1752046 | 4.48E-08 | 6.74E-11 | -8.44 | 14.71516 | -1.2 |
| TNFAIP8L2 | ILMN_1744113 | 1.45E-05 | 1.78E-07 | -6.14 | 7.13691 | -1.2 |
| SLC25A19 | ILMN_1666553 | 1.00E-05 | 1.10E-07 | -6.28 | 7.60207 | -1.22 |
| CEBPE | ILMN_1779095 | 3.38E-05 | 5.71E-07 | -5.8 | 6.0137 | -1.23 |
| MEF2C | ILMN_1742544 | 0.000564 | 2.22E-05 | -4.72 | 2.50505 | -1.23 |
| TICAM2 | ILMN_1651346 | 1.80E-08 | 1.87E-11 | -8.83 | 15.94579 | -1.24 |
| ATP7A | ILMN_1808115 | 9.10E-10 | 2.57E-13 | -10.2 | 20.03658 | -1.26 |
| CKAP2L | ILMN_1751776 | 2.63E-06 | 1.61E-08 | -6.84 | 9.45086 | -1.26 |
| CADM4 | ILMN_1812096 | 1.61E-05 | 2.08E-07 | -6.1 | 6.98323 | -1.27 |
| ABAT | ILMN_1805104 | 1.87E-05 | 2.60E-07 | -6.03 | 6.77036 | -1.28 |
| FAM43A | ILMN_1706015 | 2.52E-10 | 3.56E-14 | -10.8 | 21.9133 | -1.29 |
| P2RY2 | ILMN_2372915 | 2.11E-06 | 1.20E-08 | -6.92 | 9.73154 | -1.29 |
| MARCKSL1 | ILMN_1714433 | 5.48E-06 | 4.87E-08 | -6.52 | 8.38245 | -1.3 |
| CARD9 | ILMN_1712532 | 0.000741 | 3.23E-05 | -4.61 | 2.14864 | -1.37 |
| CD300LB | ILMN_1782741 | 5.61E-07 | 1.95E-09 | -7.45 | 11.47893 | -1.39 |
| SRC | ILMN_1729987 | 5.31E-07 | 1.76E-09 | -7.48 | 11.5791 | -1.42 |
| TGM2 | ILMN_1705750 | 0.00766 | 0.000695 | -3.64 | -0.75344 | -1.42 |
| ARRB1 | ILMN_1819608 | 5.68E-05 | 1.16E-06 | -5.6 | 5.33256 | -1.49 |
| CD33 | ILMN_1747622 | 2.34E-06 | 1.39E-08 | -6.88 | 9.59147 | -1.57 |
| MMP9 | ILMN_1796316 | 3.31E-07 | 1.01E-09 | -7.64 | 12.11301 | -1.69 |
| KLF9 | ILMN_1778523 | 6.71E-08 | 1.30E-10 | 8.25 | 14.08272 | 2.69 |
| CCL20 | ILMN_1657234 | 5.12E-06 | 4.36E-08 | 6.55 | 8.48843 | 2.58 |
| ECHDC3 | ILMN_2072178 | 0.000145 | 3.75E-06 | 5.25 | 4.2059 | 2.46 |
| FCGR1B | ILMN_2261600 | 0.0307 | 0.00432 | 3 | -2.44544 | 2.29 |
| FCGR1A | ILMN_2176063 | 0.0217 | 0.00273 | 3.17 | -2.02462 | 2.28 |
| FKBP5 | ILMN_1778444 | 0.000272 | 8.80E-06 | 5 | 3.38989 | 2.25 |
| IL18R1 | ILMN_1781700 | 7.71E-06 | 7.76E-08 | 6.38 | 7.93395 | 2.17 |
| ANKRD22 | ILMN_1799848 | 0.0164 | 0.0019 | 3.29 | -1.69146 | 1.92 |
| TLR2 | ILMN_1772387 | 0.000603 | 2.45E-05 | 4.69 | 2.41151 | 1.85 |
| FCGR1B | ILMN_2391051 | 0.0341 | 0.00495 | 2.95 | -2.56964 | 1.84 |
| CD69 | ILMN_1651316 | 0.000161 | 4.39E-06 | 5.21 | 4.05629 | 1.78 |
| ZBTB16 | ILMN_2305407 | 0.0227 | 0.00292 | 3.14 | -2.08478 | 1.78 |
| IL18RAP | ILMN_1721762 | 3.44E-05 | 5.88E-07 | 5.8 | 5.98548 | 1.71 |
| NFKBIZ | ILMN_1770260 | 3.04E-08 | 4.15E-11 | 8.59 | 15.18084 | 1.6 |
| MFSD13A | ILMN_1808566 | 0.000401 | 1.44E-05 | 4.85 | 2.92066 | 1.6 |
| IL1B | ILMN_1775501 | 9.07E-06 | 9.39E-08 | 6.33 | 7.75073 | 1.59 |
| ANKRD22 | ILMN_2132599 | 0.0103 | 0.00102 | 3.51 | -1.11154 | 1.59 |
| FBXO6 | ILMN_1701455 | 0.0209 | 0.0026 | 3.19 | -1.97816 | 1.59 |
| SERPING1 | ILMN_1670305 | 0.0224 | 0.00285 | 3.15 | -2.06309 | 1.58 |
| COL9A2 | ILMN_1685122 | 0.000447 | 1.65E-05 | 4.81 | 2.78869 | 1.57 |
| DAAM2 | ILMN_1752668 | 0.000998 | 4.76E-05 | 4.49 | 1.77772 | 1.55 |
| TNFAIP3 | ILMN_1702691 | 1.68E-11 | 1.15E-15 | 11.9 | 25.14405 | 1.53 |
| CD69 | ILMN_2188333 | 0.000419 | 1.53E-05 | 4.84 | 2.86015 | 1.52 |
| NLRP6 | ILMN_1702970 | 1.45E-06 | 7.11E-09 | 7.07 | 10.23542 | 1.48 |
| RIPK2 | ILMN_1758939 | 4.99E-06 | 4.17E-08 | 6.56 | 8.53276 | 1.48 |
| SERPINB2 | ILMN_2150856 | 0.00456 | 0.000354 | 3.86 | -0.1193 | 1.48 |
| TRIM22 | ILMN_1779252 | 0.00274 | 0.000182 | 4.07 | 0.50786 | 1.47 |
| TPK1 | ILMN_1804629 | 3.91E-05 | 6.96E-07 | 5.75 | 5.82266 | 1.43 |
| TPK1 | ILMN_2367063 | 0.000254 | 8.11E-06 | 5.03 | 3.46792 | 1.43 |
| SLC2A11 | ILMN_1748090 | 2.49E-05 | 3.90E-07 | 5.92 | 6.38095 | 1.42 |
| LOC154761 | ILMN_3237627 | 3.00E-05 | 4.97E-07 | 5.84 | 6.14703 | 1.42 |
| IRAK3 | ILMN_1661695 | 0.000147 | 3.84E-06 | 5.25 | 4.18354 | 1.39 |
| SERPINB2 | ILMN_2150851 | 0.0067 | 0.000582 | 3.7 | -0.58707 | 1.38 |
| ERLIN1 | ILMN_1730731 | 0.0125 | 0.00133 | 3.42 | -1.36039 | 1.36 |
| NLRP3 | ILMN_2310896 | 7.63E-06 | 7.64E-08 | 6.39 | 7.94858 | 1.35 |
| TMEM185B | ILMN_2231020 | 0.000212 | 6.36E-06 | 5.1 | 3.6997 | 1.34 |
| SORT1 | ILMN_1707077 | 0.00436 | 0.000335 | 3.88 | -0.06962 | 1.32 |
| PSTPIP2 | ILMN_1713058 | 0.045 | 0.00716 | 2.82 | -2.90441 | 1.31 |
| NTPCR | ILMN_1657446 | 2.49E-08 | 3.05E-11 | 8.68 | 15.47514 | 1.29 |
| TULP2 | ILMN_2091221 | 1.35E-05 | 1.64E-07 | 6.17 | 7.21502 | 1.29 |
| NET1 | ILMN_1758311 | 0.00398 | 0.000297 | 3.92 | 0.04361 | 1.29 |
| TPST1 | ILMN_1651950 | 0.000213 | 6.42E-06 | 5.09 | 3.69168 | 1.28 |
| KRTAP19-6 | ILMN_1784216 | 8.28E-05 | 1.90E-06 | 5.45 | 4.85956 | 1.27 |
| ANXA3 | ILMN_1694548 | 0.0408 | 0.00627 | 2.87 | -2.78397 | 1.27 |
| CLEC4E | ILMN_1771664 | 4.59E-06 | 3.59E-08 | 6.61 | 8.67647 | 1.25 |
| NLRP3 | ILMN_1712026 | 2.33E-05 | 3.51E-07 | 5.95 | 6.48073 | 1.25 |
| FAM65B | ILMN_1726597 | 0.000137 | 3.49E-06 | 5.27 | 4.27482 | 1.25 |
| SAP30 | ILMN_1700896 | 0.00833 | 0.000774 | 3.6 | -0.85369 | 1.25 |
| SOCS1 | ILMN_1774733 | 0.00168 | 9.50E-05 | 4.28 | 1.12247 | 1.23 |
| KDSR | ILMN_2154053 | 0.0125 | 0.00134 | 3.42 | -1.3642 | 1.23 |
| CLEC4D | ILMN_1808979 | 0.000137 | 3.51E-06 | 5.27 | 4.26937 | 1.21 |
| NUDT16 | ILMN_1781996 | 0.014 | 0.00154 | 3.37 | -1.49714 | 1.21 |
| GBP5 | ILMN_2114568 | 0.0375 | 0.0056 | 2.91 | -2.68226 | 1.21 |
| CNIH4 | ILMN_1714759 | 0.00249 | 0.000162 | 4.11 | 0.61862 | 1.2 |
| SNHG7 | ILMN_3227023 | 0.000254 | 8.10E-06 | 5.03 | 3.46926 | 1.18 |
| LACTB | ILMN_1693830 | 0.0024 | 0.000155 | 4.12 | 0.66095 | 1.18 |
| KLHL15 | ILMN_1701648 | 4.78E-06 | 3.87E-08 | 6.58 | 8.60343 | 1.17 |
| ANKRD9 | ILMN_2048607 | 0.000175 | 4.92E-06 | 5.17 | 3.94592 | 1.17 |
| TCAF2 | ILMN_1780798 | 0.000547 | 2.12E-05 | 4.74 | 2.54772 | 1.17 |
| IL1RN | ILMN_1689734 | 0.000762 | 3.34E-05 | 4.6 | 2.11673 | 1.17 |
| ERRFI1 | ILMN_1665510 | 0.000878 | 4.02E-05 | 4.54 | 1.93985 | 1.17 |
| HLX | ILMN_2087646 | 0.0299 | 0.00418 | 3.01 | -2.41426 | 1.17 |
| SNHG9 | ILMN_3238785 | 3.80E-05 | 6.70E-07 | 5.76 | 5.85962 | 1.16 |
| SNHG7 | ILMN_3233205 | 0.00103 | 4.96E-05 | 4.48 | 1.73979 | 1.16 |
| SMAP2 | ILMN_1781468 | 3.68E-06 | 2.51E-08 | 6.71 | 9.02069 | 1.15 |
| ACSL1 | ILMN_1684585 | 1.55E-05 | 1.94E-07 | 6.12 | 7.0507 | 1.14 |
| ABCG1 | ILMN_2329927 | 0.000174 | 4.86E-06 | 5.18 | 3.95878 | 1.14 |
| ACSL4 | ILMN_2391458 | 0.000195 | 5.69E-06 | 5.13 | 3.80611 | 1.14 |
| IL1RN | ILMN_1774874 | 0.000554 | 2.16E-05 | 4.73 | 2.52958 | 1.14 |
| TRIM25 | ILMN_1813625 | 0.0262 | 0.00353 | 3.08 | -2.26095 | 1.14 |
| ACSL4 | ILMN_1683598 | 1.53E-05 | 1.89E-07 | 6.13 | 7.07702 | 1.13 |
| PER1 | ILMN_1653125 | 0.000437 | 1.60E-05 | 4.82 | 2.81568 | 1.13 |
| SAMSN1 | ILMN_1684887 | 0.00058 | 2.31E-05 | 4.71 | 2.46838 | 1.13 |
| TMEM185B | ILMN_2231021 | 0.000932 | 4.36E-05 | 4.52 | 1.86114 | 1.13 |
| NLRP3 | ILMN_1696933 | 0.00307 | 0.000213 | 4.02 | 0.36039 | 1.13 |
| SAMSN1 | ILMN_2171289 | 0.0104 | 0.00104 | 3.5 | -1.13259 | 1.13 |
| STAT1 | ILMN_1690105 | 0.0226 | 0.00289 | 3.15 | -2.0773 | 1.13 |
| STK17B | ILMN_2166534 | 0.00182 | 0.000105 | 4.24 | 1.02309 | 1.12 |
| KDSR | ILMN_2154052 | 0.0263 | 0.00355 | 3.07 | -2.26649 | 1.12 |
| DNAJC25-GNG10 | ILMN_1767809 | 0.0362 | 0.00534 | 2.93 | -2.63781 | 1.11 |
| OBFC1 | ILMN_1789186 | 0.0512 | 0.00855 | 2.75 | -3.06512 | 1.11 |
| CXCL2 | ILMN_1682636 | 4.92E-06 | 4.05E-08 | 6.57 | 8.55945 | 1.1 |
| ACKR3 | ILMN_1798360 | 0.00995 | 0.00098 | 3.52 | -1.07429 | 1.1 |
| SPRY1 | ILMN_2329914 | 2.63E-05 | 4.25E-07 | 5.89 | 6.29861 | 1.09 |
| TOB1 | ILMN_1672004 | 0.00299 | 0.000205 | 4.03 | 0.39456 | 1.09 |
| GNG10 | ILMN_1652003 | 0.023 | 0.00298 | 3.14 | -2.10381 | 1.09 |
| IFIT3 | ILMN_1664543 | 0.0299 | 0.00417 | 3.02 | -2.41357 | 1.09 |
| TIFA | ILMN_1686454 | 0.0778 | 0.015 | 2.53 | -3.57161 | 1.09 |
| GBP1P1 | ILMN_1782487 | 0.111 | 0.0244 | 2.33 | -3.99958 | 1.09 |
| IRAK3 | ILMN_1913678 | 0.00117 | 5.91E-05 | 4.42 | 1.57351 | 1.08 |
| BATF2 | ILMN_1690241 | 0.107 | 0.0231 | 2.35 | -3.95084 | 1.08 |
| SLC25A51 | ILMN_2198499 | 0.00467 | 0.000365 | 3.85 | -0.14912 | 1.07 |
| TSPAN17 | ILMN_1777881 | 0.119 | 0.027 | 2.28 | -4.08774 | 1.07 |
| ABCG1 | ILMN_1794782 | 1.18E-05 | 1.36E-07 | 6.22 | 7.39731 | 1.06 |
| AKAP13 | ILMN_1752247 | 0.00538 | 0.000438 | 3.79 | -0.3213 | 1.06 |
| CASP4 | ILMN_1778059 | 0.0656 | 0.0119 | 2.62 | -3.36459 | 1.06 |
| ZBTB16 | ILMN_2402817 | 0.114 | 0.0253 | 2.31 | -4.02901 | 1.06 |
| RNF122 | ILMN_1691119 | 9.22E-09 | 5.64E-12 | 9.19 | 17.09098 | 1.05 |
| ARG1 | ILMN_1812281 | 0.00152 | 8.25E-05 | 4.32 | 1.25611 | 1.05 |
| SIRT5 | ILMN_1683059 | 0.019 | 0.0023 | 3.23 | -1.86638 | 1.05 |
| SECTM1 | ILMN_1652277 | 0.00973 | 0.000952 | 3.53 | -1.04686 | 1.04 |
| GJB2 | ILMN_1769388 | 0.000975 | 4.63E-05 | 4.5 | 1.80547 | 1.02 |
| CEACAM4 | ILMN_1657455 | 0.00768 | 0.000698 | 3.64 | -0.75677 | 1.02 |
| CD7 | ILMN_1792538 | 0.00041 | 1.48E-05 | 4.84 | 2.89136 | 1.01 |
| ATF3 | ILMN_2374865 | 0.00578 | 0.00048 | 3.76 | -0.40665 | 1.01 |
| CABLES2 | ILMN_1762407 | 0.0067 | 0.000582 | 3.7 | -0.5874 | 1.01 |
| PARP9 | ILMN_2053527 | 0.022 | 0.00279 | 3.16 | -2.04377 | 1.01 |
| PLSCR1 | ILMN_1745242 | 0.0238 | 0.00311 | 3.12 | -2.14449 | 1.01 |
| GFOD1 | ILMN_1778240 | 0.0521 | 0.00874 | 2.74 | -3.08554 | 1.01 |
| ZBTB16 | ILMN_1750496 | 0.0606 | 0.0108 | 2.66 | -3.27247 | 1.01 |
| CCND3 | ILMN_1668721 | 0.000929 | 4.33E-05 | 4.52 | 1.86851 | 1 |
| BLM | ILMN_1709484 | 0.021 | 0.00263 | 3.18 | -1.98787 | 1 |
| NFKBIZ | ILMN_1719695 | 4.33E-07 | 1.40E-09 | 7.55 | 11.80321 | 0.999 |
| EPSTI1 | ILMN_2388547 | 0.0884 | 0.0179 | 2.46 | -3.72406 | 0.999 |
| ITPRIPL2 | ILMN_1751034 | 0.101 | 0.0214 | 2.38 | -3.88498 | 0.997 |
| GJB6 | ILMN_2226223 | 0.000718 | 3.10E-05 | 4.62 | 2.18664 | 0.995 |
| P2RY14 | ILMN_2342835 | 0.175 | 0.0463 | 2.05 | -4.55254 | 0.995 |
| THBS1 | ILMN_1686116 | 0.0128 | 0.00137 | 3.41 | -1.3869 | 0.994 |
| ANXA2R | ILMN_2098616 | 0.0109 | 0.00111 | 3.48 | -1.18855 | 0.993 |
| GNG10 | ILMN_1757074 | 0.00978 | 0.000957 | 3.53 | -1.05252 | 0.991 |
| ABCG1 | ILMN_1695968 | 2.67E-05 | 4.34E-07 | 5.88 | 6.27788 | 0.988 |
| DNAAF1 | ILMN_1776967 | 2.65E-05 | 4.29E-07 | 5.89 | 6.28885 | 0.987 |
| C11orf96 | ILMN_1677402 | 0.000101 | 2.45E-06 | 5.38 | 4.61501 | 0.986 |
| PIK3IP1 | ILMN_1719986 | 0.000554 | 2.17E-05 | 4.73 | 2.52754 | 0.982 |
| C9orf72 | ILMN_2296677 | 1.84E-05 | 2.55E-07 | 6.04 | 6.78849 | 0.978 |
| SYTL3 | ILMN_1720623 | 4.16E-05 | 7.57E-07 | 5.72 | 5.74264 | 0.976 |
| SOCS3 | ILMN_1781001 | 0.0123 | 0.0013 | 3.43 | -1.3396 | 0.976 |
| XBP1 | ILMN_2365465 | 1.64E-06 | 8.52E-09 | 7.02 | 10.06202 | 0.975 |
| SLC7A5 | ILMN_1720373 | 0.000251 | 7.97E-06 | 5.03 | 3.48391 | 0.973 |
| KCNE5 | ILMN_1711650 | 0.0144 | 0.0016 | 3.36 | -1.52782 | 0.972 |
| SLCO4A1 | ILMN_1727200 | 0.039 | 0.0059 | 2.89 | -2.72913 | 0.971 |
| DUSP5 | ILMN_1656501 | 0.00103 | 4.96E-05 | 4.48 | 1.74002 | 0.968 |
| FAM160B1 | ILMN_3182275 | 7.93E-08 | 1.69E-10 | 8.17 | 13.83528 | 0.965 |
| KLF4 | ILMN_2137789 | 0.00382 | 0.000282 | 3.93 | 0.09328 | 0.965 |
| RAB43 | ILMN_1696230 | 1.71E-07 | 4.43E-10 | 7.88 | 12.90631 | 0.963 |
| IL1R2 | ILMN_1758371 | 1.64E-05 | 2.19E-07 | 6.08 | 6.93732 | 0.959 |
| KCNH2 | ILMN_1739987 | 0.000848 | 3.82E-05 | 4.56 | 1.98806 | 0.955 |
| HDAC4 | ILMN_1764396 | 0.0588 | 0.0103 | 2.67 | -3.2353 | 0.951 |
| XBP1 | ILMN_1809433 | 1.11E-06 | 4.65E-09 | 7.2 | 10.64377 | 0.949 |
| EGR2 | ILMN_1743199 | 0.015 | 0.00169 | 3.34 | -1.57874 | 0.948 |
| PFKFB2 | ILMN_1796560 | 0.00183 | 0.000106 | 4.24 | 1.01605 | 0.945 |
| SNHG7 | ILMN_3233229 | 0.0081 | 0.000747 | 3.61 | -0.821 | 0.943 |
| GJB6 | ILMN_1718671 | 0.000601 | 2.43E-05 | 4.7 | 2.41785 | 0.94 |
| RNF144B | ILMN_1752526 | 6.20E-06 | 5.75E-08 | 6.47 | 8.22239 | 0.937 |
| CCNJL | ILMN_1763745 | 0.00126 | 6.48E-05 | 4.4 | 1.48597 | 0.936 |
| STK17B | ILMN_1798543 | 1.30E-06 | 5.67E-09 | 7.14 | 10.45319 | 0.931 |
| SDHAF3 | ILMN_2134039 | 0.00252 | 0.000164 | 4.11 | 0.60515 | 0.928 |
| ETNK1 | ILMN_2244547 | 6.92E-05 | 1.49E-06 | 5.52 | 5.09079 | 0.927 |
| GYPC | ILMN_1682332 | 0.00814 | 0.000752 | 3.61 | -0.82758 | 0.927 |
| FAS | ILMN_1808132 | 0.00854 | 0.000798 | 3.59 | -0.88273 | 0.925 |
| MAD2L2 | ILMN_1669550 | 0.00576 | 0.000478 | 3.76 | -0.40198 | 0.923 |
| PPA1 | ILMN_1805827 | 0.0583 | 0.0102 | 2.68 | -3.22546 | 0.923 |
| STK3 | ILMN_1666453 | 0.1 | 0.0212 | 2.39 | -3.87699 | 0.922 |
| VNN1 | ILMN_1674574 | 0.000346 | 1.19E-05 | 4.91 | 3.10069 | 0.921 |
| IGFBPL1 | ILMN_1677158 | 3.68E-06 | 2.47E-08 | 6.71 | 9.03558 | 0.919 |
| IDI1 | ILMN_1755075 | 0.00144 | 7.67E-05 | 4.34 | 1.32557 | 0.917 |
| LACTB | ILMN_1765132 | 0.0295 | 0.0041 | 3.02 | -2.39622 | 0.917 |
| MIR21 | ILMN_3310840 | 3.68E-06 | 2.55E-08 | 6.7 | 9.00629 | 0.916 |
| HOXA5 | ILMN_1753613 | 0.000478 | 1.82E-05 | 4.78 | 2.69423 | 0.915 |
| CCNJL | ILMN_3237926 | 0.00213 | 0.000132 | 4.17 | 0.81048 | 0.915 |
| SNORD3D | ILMN_3242315 | 1.80E-08 | 1.79E-11 | 8.84 | 15.98762 | 0.912 |
| SNORD3A | ILMN_3239574 | 7.53E-09 | 4.25E-12 | 9.28 | 17.36192 | 0.909 |
| FGD4 | ILMN_1698728 | 0.00202 | 0.000123 | 4.2 | 0.88091 | 0.906 |
| INHBB | ILMN_1685714 | 0.00578 | 0.00048 | 3.76 | -0.40609 | 0.905 |
| ATF6 | ILMN_1703471 | 0.00924 | 0.000884 | 3.56 | -0.97776 | 0.905 |
| STAT1 | ILMN_1691364 | 0.0409 | 0.00628 | 2.86 | -2.78619 | 0.905 |
| SPRY1 | ILMN_1691860 | 4.16E-05 | 7.56E-07 | 5.72 | 5.74375 | 0.904 |
| VAMP5 | ILMN_1809467 | 0.119 | 0.0268 | 2.29 | -4.08123 | 0.901 |
| GYPC | ILMN_1668039 | 0.00492 | 0.000391 | 3.83 | -0.21326 | 0.899 |
| ZNF281 | ILMN_1683127 | 0.000151 | 3.98E-06 | 5.24 | 4.15043 | 0.894 |
| SIPA1L2 | ILMN_1732923 | 0.000142 | 3.66E-06 | 5.26 | 4.22866 | 0.893 |
| C9orf72 | ILMN_1666742 | 1.28E-05 | 1.51E-07 | 6.19 | 7.29322 | 0.892 |
| AKAP13 | ILMN_2396956 | 0.0101 | 0.001 | 3.51 | -1.09759 | 0.89 |
| SLC26A8 | ILMN_1755843 | 0.0366 | 0.00543 | 2.92 | -2.65365 | 0.889 |
| VNN3 | ILMN_2387680 | 5.61E-08 | 9.53E-11 | 8.34 | 14.3825 | 0.887 |
| NLRC5 | ILMN_1716704 | 0.0855 | 0.0171 | 2.48 | -3.68283 | 0.887 |
| ATL3 | ILMN_1751086 | 0.00439 | 0.000338 | 3.87 | -0.07688 | 0.885 |
| CRYL1 | ILMN_1714397 | 1.53E-05 | 1.90E-07 | 6.12 | 7.07144 | 0.884 |
| OLIG2 | ILMN_1727567 | 0.117 | 0.0262 | 2.3 | -4.06008 | 0.875 |
| SC5D | ILMN_1677607 | 1.57E-05 | 2.01E-07 | 6.11 | 7.01862 | 0.874 |
| MAFF | ILMN_1680139 | 0.00276 | 0.000184 | 4.07 | 0.49723 | 0.874 |
| GSTK1 | ILMN_1725241 | 0.0019 | 0.000111 | 4.23 | 0.97466 | 0.872 |
| KCNJ2 | ILMN_1780334 | 0.044 | 0.00693 | 2.83 | -2.8757 | 0.871 |
| CCL4L1 | ILMN_1716276 | 0.000878 | 4.01E-05 | 4.54 | 1.94165 | 0.866 |
| ASPH | ILMN_2352934 | 0.00422 | 0.000321 | 3.89 | -0.02723 | 0.863 |
| LMNB1 | ILMN_2126706 | 0.00895 | 0.000849 | 3.57 | -0.9407 | 0.863 |
| ARID5A | ILMN_1689700 | 0.000217 | 6.58E-06 | 5.09 | 3.66796 | 0.858 |
| IRF1 | ILMN_1708375 | 0.00809 | 0.000746 | 3.61 | -0.81917 | 0.855 |
| UPP1 | ILMN_1798256 | 0.000118 | 2.94E-06 | 5.33 | 4.44021 | 0.853 |
| SESN2 | ILMN_1751598 | 1.22E-05 | 1.42E-07 | 6.21 | 7.35377 | 0.852 |
| CCL3 | ILMN_1671509 | 0.00103 | 4.97E-05 | 4.48 | 1.73778 | 0.852 |
| BMX | ILMN_1796138 | 0.000667 | 2.79E-05 | 4.65 | 2.28783 | 0.851 |
| UBE2L6 | ILMN_1703108 | 0.0254 | 0.00339 | 3.09 | -2.22236 | 0.846 |
| DNAJB9 | ILMN_1773742 | 6.36E-09 | 2.99E-12 | 9.39 | 17.69713 | 0.845 |
| IFITM3 | ILMN_1805750 | 0.00699 | 0.000612 | 3.68 | -0.63459 | 0.845 |
| TBC1D14 | ILMN_1779886 | 0.000994 | 4.74E-05 | 4.49 | 1.78298 | 0.843 |
| PARP9 | ILMN_1731224 | 0.0589 | 0.0104 | 2.67 | -3.23773 | 0.843 |
| DDX21 | ILMN_1735461 | 0.0111 | 0.00114 | 3.47 | -1.21764 | 0.841 |
| PRRG4 | ILMN_1661809 | 0.00707 | 0.000622 | 3.67 | -0.64958 | 0.834 |
| B4GALT5 | ILMN_1685824 | 0.0116 | 0.00121 | 3.45 | -1.26963 | 0.834 |
| ZC3HAV1 | ILMN_1667068 | 0.0402 | 0.00614 | 2.87 | -2.76517 | 0.83 |
| GADD45B | ILMN_1718977 | 1.11E-05 | 1.24E-07 | 6.25 | 7.48252 | 0.827 |
| MT2A | ILMN_1686664 | 4.69E-06 | 3.75E-08 | 6.59 | 8.6341 | 0.825 |
| C9orf72 | ILMN_1762508 | 8.08E-05 | 1.83E-06 | 5.46 | 4.89396 | 0.824 |
| JMY | ILMN_1762080 | 0.0127 | 0.00136 | 3.41 | -1.37818 | 0.823 |
| PFKFB2 | ILMN_1723436 | 0.0312 | 0.00441 | 3 | -2.46381 | 0.819 |
| DTNBP1 | ILMN_1783806 | 0.147 | 0.036 | 2.16 | -4.33673 | 0.819 |
| UBALD2 | ILMN_1775498 | 9.36E-07 | 3.83E-09 | 7.25 | 10.8309 | 0.818 |
| CCL3L1 | ILMN_1773245 | 0.00127 | 6.58E-05 | 4.39 | 1.47029 | 0.818 |
| SDHAF3 | ILMN_1771348 | 0.0188 | 0.00226 | 3.23 | -1.84992 | 0.815 |
| IRS2 | ILMN_2083469 | 2.96E-08 | 3.79E-11 | 8.61 | 15.26743 | 0.813 |
| MAPK14 | ILMN_1788002 | 0.0314 | 0.00446 | 2.99 | -2.4738 | 0.811 |
| CARD16 | ILMN_1726591 | 0.049 | 0.00799 | 2.77 | -3.00469 | 0.809 |
| GBP2 | ILMN_1774077 | 0.00702 | 0.000616 | 3.68 | -0.63979 | 0.808 |
| RAB43 | ILMN_1781182 | 1.60E-06 | 8.20E-09 | 7.03 | 10.09881 | 0.806 |
| GCLM | ILMN_1788547 | 0.083 | 0.0164 | 2.49 | -3.6488 | 0.805 |
| SLC11A1 | ILMN_1735737 | 1.88E-05 | 2.61E-07 | 6.03 | 6.76488 | 0.803 |
| MSL3 | ILMN_1670723 | 4.30E-06 | 3.25E-08 | 6.63 | 8.77204 | 0.799 |
| MSL3 | ILMN_1713156 | 0.000187 | 5.35E-06 | 5.15 | 3.86629 | 0.799 |
| PDE4B | ILMN_1782922 | 6.71E-08 | 1.33E-10 | 8.24 | 14.06497 | 0.798 |
| JADE2 | ILMN_1795285 | 0.00266 | 0.000175 | 4.08 | 0.54373 | 0.797 |
| C19orf12 | ILMN_1664920 | 0.122 | 0.028 | 2.27 | -4.11756 | 0.788 |
| SLC38A1 | ILMN_1769911 | 0.0499 | 0.00821 | 2.76 | -3.02857 | 0.787 |
| DHRS12 | ILMN_1719915 | 0.0208 | 0.00259 | 3.19 | -1.97389 | 0.783 |
| LGALSL | ILMN_1673548 | 0.000346 | 1.19E-05 | 4.91 | 3.10265 | 0.778 |
| CCL3L1 | ILMN_1747355 | 0.00232 | 0.000147 | 4.14 | 0.70945 | 0.775 |
| CREG1 | ILMN_1680624 | 0.00504 | 0.000405 | 3.82 | -0.24683 | 0.774 |
| TAGAP | ILMN_2333774 | 0.00603 | 0.000509 | 3.74 | -0.46138 | 0.773 |
| RGS1 | ILMN_1656011 | 9.00E-06 | 9.27E-08 | 6.33 | 7.76254 | 0.772 |
| PDK4 | ILMN_1684982 | 0.0256 | 0.00343 | 3.09 | -2.23391 | 0.771 |
| NUDT5 | ILMN_1711314 | 0.0576 | 0.0101 | 2.69 | -3.2114 | 0.77 |
| MAPK14 | ILMN_2388090 | 0.115 | 0.0254 | 2.31 | -4.03515 | 0.767 |
| VNN3 | ILMN_1804935 | 1.76E-07 | 4.64E-10 | 7.87 | 12.86189 | 0.761 |
| SNORA64 | ILMN_1709604 | 0.00969 | 0.000945 | 3.53 | -1.04024 | 0.755 |
| IL1A | ILMN_1658483 | 0.0369 | 0.0055 | 2.91 | -2.66628 | 0.755 |
| TNFSF10 | ILMN_1801307 | 0.0736 | 0.014 | 2.55 | -3.50763 | 0.755 |
| TLR5 | ILMN_1722981 | 0.1 | 0.0212 | 2.39 | -3.87436 | 0.754 |
| STAT1 | ILMN_1777325 | 0.11 | 0.024 | 2.33 | -3.9855 | 0.751 |
| CARD16 | ILMN_3252556 | 0.00889 | 0.000843 | 3.57 | -0.93321 | 0.75 |
| IL1R1 | ILMN_1810584 | 0.0187 | 0.00224 | 3.24 | -1.84238 | 0.746 |
| ABHD5 | ILMN_1655702 | 6.06E-06 | 5.56E-08 | 6.48 | 8.25446 | 0.744 |
| ELL2 | ILMN_1655930 | 0.000671 | 2.81E-05 | 4.65 | 2.27987 | 0.744 |
| CCL3L1 | ILMN_2218856 | 0.00316 | 0.000221 | 4.01 | 0.32328 | 0.744 |
| CDKN1A | ILMN_1787212 | 0.0259 | 0.00348 | 3.08 | -2.24664 | 0.742 |
| TMIGD3 | ILMN_1733259 | 0.0747 | 0.0143 | 2.55 | -3.52568 | 0.74 |
| CARD17 | ILMN_1707979 | 0.0259 | 0.00349 | 3.08 | -2.24899 | 0.738 |
| CPD | ILMN_1703074 | 0.0115 | 0.00119 | 3.46 | -1.25775 | 0.737 |
| PLGRKT | ILMN_1709043 | 0.0758 | 0.0146 | 2.54 | -3.54377 | 0.737 |
| ZDHHC19 | ILMN_1766896 | 0.109 | 0.0238 | 2.34 | -3.97808 | 0.734 |
| TAGAP | ILMN_1676408 | 0.0119 | 0.00125 | 3.44 | -1.30262 | 0.733 |
| PPIAL4G | ILMN_1710362 | 3.78E-06 | 2.69E-08 | 6.69 | 8.95512 | 0.731 |
| HMGB2 | ILMN_1654268 | 3.86E-05 | 6.86E-07 | 5.75 | 5.83757 | 0.73 |
| POLR3GL | ILMN_1760667 | 0.00151 | 8.17E-05 | 4.32 | 1.26522 | 0.73 |
| IFITM1 | ILMN_1801246 | 0.000217 | 6.56E-06 | 5.09 | 3.67131 | 0.728 |
| MSL3 | ILMN_3226505 | 0.000452 | 1.69E-05 | 4.81 | 2.76728 | 0.728 |
| EDN1 | ILMN_1682775 | 0.0408 | 0.00627 | 2.87 | -2.78415 | 0.72 |
| PELI1 | ILMN_1679268 | 9.08E-08 | 2.05E-10 | 8.11 | 13.64714 | 0.719 |
| PML | ILMN_1728019 | 0.0419 | 0.00649 | 2.85 | -2.8165 | 0.719 |
| PSEN1 | ILMN_1808548 | 5.31E-07 | 1.78E-09 | 7.48 | 11.57117 | 0.718 |
| CD55 | ILMN_1800540 | 0.000154 | 4.08E-06 | 5.23 | 4.12634 | 0.718 |
| STX11 | ILMN_1720771 | 0.000693 | 2.94E-05 | 4.64 | 2.23786 | 0.718 |
| SPACA6 | ILMN_3247848 | 2.02E-06 | 1.13E-08 | 6.94 | 9.7865 | 0.716 |
| HAL | ILMN_2074748 | 1.67E-06 | 8.89E-09 | 7.01 | 10.02037 | 0.715 |
| DSC2 | ILMN_1663119 | 0.0134 | 0.00146 | 3.39 | -1.4421 | 0.715 |
| CASP1 | ILMN_2326509 | 0.0239 | 0.00313 | 3.12 | -2.15018 | 0.713 |
| FOXC1 | ILMN_1738401 | 0.00376 | 0.000277 | 3.94 | 0.10972 | 0.712 |
| MAP3K8 | ILMN_1741159 | 2.38E-06 | 1.43E-08 | 6.87 | 9.55959 | 0.711 |
| CTSL | ILMN_2374036 | 0.0284 | 0.00391 | 3.04 | -2.35303 | 0.711 |
| C19orf12 | ILMN_1703695 | 0.0983 | 0.0206 | 2.4 | -3.85193 | 0.709 |
| PKD2L1 | ILMN_1789361 | 0.0871 | 0.0175 | 2.47 | -3.70576 | 0.707 |
| TAGAP | ILMN_1739985 | 0.0112 | 0.00115 | 3.47 | -1.22123 | 0.706 |
| KLF10 | ILMN_1659122 | 0.000201 | 5.91E-06 | 5.12 | 3.76976 | 0.705 |
| ASPH | ILMN_1693771 | 0.00844 | 0.000788 | 3.6 | -0.8706 | 0.704 |
| CLEC7A | ILMN_1654504 | 0.041 | 0.0063 | 2.86 | -2.78851 | 0.703 |
| SYAP1 | ILMN_1698470 | 0.000682 | 2.88E-05 | 4.64 | 2.25597 | 0.698 |
| GRB10 | ILMN_1669617 | 0.0224 | 0.00286 | 3.15 | -2.06565 | 0.698 |
| HIPK2 | ILMN_1687440 | 0.0448 | 0.00713 | 2.82 | -2.9006 | 0.698 |
| PUS3 | ILMN_1694147 | 0.1 | 0.0213 | 2.38 | -3.87836 | 0.697 |
| CDC123 | ILMN_1678605 | 0.0483 | 0.00784 | 2.78 | -2.98747 | 0.696 |
| SMAP2 | ILMN_2081682 | 2.38E-06 | 1.42E-08 | 6.87 | 9.56755 | 0.693 |
| MAPK14 | ILMN_1737627 | 0.137 | 0.0328 | 2.2 | -4.25551 | 0.692 |
| ACCS | ILMN_3187680 | 0.019 | 0.0023 | 3.23 | -1.86521 | 0.691 |
| CTSL | ILMN_1812995 | 0.0527 | 0.0089 | 2.73 | -3.10106 | 0.688 |
| PSMB8 | ILMN_2284794 | 0.00423 | 0.000322 | 3.89 | -0.03131 | 0.687 |
| SOCS3 | ILMN_2156250 | 0.03 | 0.00419 | 3.01 | -2.41778 | 0.687 |
| CYP1B1 | ILMN_1693338 | 0.0588 | 0.0103 | 2.67 | -3.23525 | 0.687 |
| SPIDR | ILMN_1887174 | 0.00259 | 0.00017 | 4.09 | 0.57379 | 0.686 |
| TSC22D1 | ILMN_1787567 | 0.000583 | 2.33E-05 | 4.71 | 2.46038 | 0.685 |
| SETDB2 | ILMN_1731644 | 0.101 | 0.0215 | 2.38 | -3.88839 | 0.682 |
| B3GNT5 | ILMN_1702609 | 0.035 | 0.00513 | 2.94 | -2.60116 | 0.68 |
| ETV7 | ILMN_1700671 | 0.149 | 0.0368 | 2.15 | -4.35472 | 0.68 |
| LYPLA1 | ILMN_1666713 | 2.90E-06 | 1.80E-08 | 6.81 | 9.34019 | 0.679 |
| MAK | ILMN_1803984 | 0.000842 | 3.78E-05 | 4.56 | 1.99883 | 0.678 |
| TLR8 | ILMN_1705047 | 0.0029 | 0.000197 | 4.05 | 0.4311 | 0.677 |
| PSMG1 | ILMN_1659285 | 0.0113 | 0.00117 | 3.46 | -1.24034 | 0.677 |
| CSHL1 | ILMN_1680436 | 0.0126 | 0.00134 | 3.42 | -1.36751 | 0.677 |
| GRINA | ILMN_1796490 | 0.048 | 0.0078 | 2.78 | -2.9824 | 0.677 |
| ZC3HAV1 | ILMN_1724837 | 0.106 | 0.0228 | 2.36 | -3.93774 | 0.675 |
| IFIT3 | ILMN_1701789 | 0.0512 | 0.00853 | 2.75 | -3.0636 | 0.674 |
| NFIL3 | ILMN_1707312 | 1.65E-06 | 8.61E-09 | 7.02 | 10.05113 | 0.673 |
| HAL | ILMN_1719988 | 3.68E-06 | 2.50E-08 | 6.71 | 9.02473 | 0.67 |
| CCL3L3 | ILMN_2105573 | 0.00437 | 0.000337 | 3.88 | -0.07313 | 0.67 |
| BCL2A1 | ILMN_1769229 | 0.00464 | 0.000362 | 3.85 | -0.14034 | 0.67 |
| ARL4A | ILMN_1743241 | 0.00533 | 0.000434 | 3.79 | -0.31199 | 0.67 |
| LACTB | ILMN_1703335 | 0.0927 | 0.0191 | 2.43 | -3.78249 | 0.67 |
| SETD9 | ILMN_1757636 | 9.64E-09 | 7.84E-12 | 9.09 | 16.77697 | 0.669 |
| BCL2L11 | ILMN_2359627 | 0.000251 | 7.96E-06 | 5.03 | 3.48535 | 0.669 |
| GCLM | ILMN_2225974 | 0.0778 | 0.015 | 2.53 | -3.57152 | 0.669 |
| DNAJB1 | ILMN_1775304 | 0.00038 | 1.34E-05 | 4.87 | 2.98391 | 0.668 |
| ERI1 | ILMN_3245659 | 0.109 | 0.0236 | 2.34 | -3.97032 | 0.667 |
| MS4A6A | ILMN_1721035 | 0.000356 | 1.23E-05 | 4.9 | 3.06539 | 0.663 |
| H1F0 | ILMN_1757467 | 0.00213 | 0.000132 | 4.17 | 0.81362 | 0.663 |
| MGST2 | ILMN_1802027 | 0.00874 | 0.000821 | 3.58 | -0.90908 | 0.663 |
| HDDC3 | ILMN_1781638 | 0.0369 | 0.0055 | 2.91 | -2.66504 | 0.663 |
| THADA | ILMN_1811624 | 0.059 | 0.0104 | 2.67 | -3.24168 | 0.663 |
| GADD45A | ILMN_1694075 | 3.68E-06 | 2.54E-08 | 6.71 | 9.00987 | 0.662 |
| RALB | ILMN_1676358 | 6.03E-06 | 5.51E-08 | 6.48 | 8.26433 | 0.661 |
| AGPAT2 | ILMN_1732176 | 0.000249 | 7.85E-06 | 5.03 | 3.49905 | 0.659 |
| SMIM3 | ILMN_1684368 | 0.0704 | 0.0132 | 2.58 | -3.45523 | 0.659 |
| CYTIP | ILMN_2092041 | 2.80E-05 | 4.57E-07 | 5.87 | 6.22843 | 0.658 |
| NMI | ILMN_1739541 | 0.00178 | 0.000103 | 4.25 | 1.0473 | 0.658 |
| SRPK1 | ILMN_1798804 | 0.00373 | 0.000273 | 3.94 | 0.12425 | 0.658 |
| CASP1 | ILMN_2326512 | 0.0355 | 0.0052 | 2.93 | -2.61413 | 0.658 |
| RNF115 | ILMN_3247728 | 0.0381 | 0.00573 | 2.9 | -2.70279 | 0.656 |
| STOM | ILMN_1696419 | 0.0607 | 0.0108 | 2.66 | -3.27354 | 0.656 |
| LAP3 | ILMN_1683792 | 0.118 | 0.0267 | 2.29 | -4.07636 | 0.655 |
| PRDM1 | ILMN_2298159 | 0.0529 | 0.00895 | 2.73 | -3.10675 | 0.654 |
| RNU11 | ILMN_3245103 | 0.000277 | 9.04E-06 | 4.99 | 3.36411 | 0.653 |
| NSUN7 | ILMN_1767960 | 0.00402 | 0.000301 | 3.91 | 0.03163 | 0.653 |
| MYLIP | ILMN_1656111 | 7.14E-08 | 1.45E-10 | 8.22 | 13.98296 | 0.652 |
| SNHG8 | ILMN_3309349 | 2.25E-06 | 1.30E-08 | 6.9 | 9.65241 | 0.65 |
| CYTIP | ILMN_3235928 | 5.12E-05 | 1.02E-06 | 5.64 | 5.45633 | 0.649 |
| GK | ILMN_1725471 | 0.022 | 0.00278 | 3.16 | -2.04055 | 0.649 |
| SNHG1 | ILMN_3236713 | 0.000258 | 8.26E-06 | 5.02 | 3.45027 | 0.648 |
| PAPSS1 | ILMN_2224103 | 0.0884 | 0.0178 | 2.46 | -3.72325 | 0.646 |
| MTHFD2 | ILMN_1674706 | 0.0742 | 0.0141 | 2.55 | -3.51622 | 0.645 |
| TIPARP | ILMN_1765578 | 6.71E-08 | 1.28E-10 | 8.25 | 14.09755 | 0.643 |
| SPATA13 | ILMN_1742824 | 0.0418 | 0.00646 | 2.85 | -2.81138 | 0.643 |
| VNN2 | ILMN_1692546 | 0.103 | 0.0221 | 2.37 | -3.91188 | 0.643 |
| IRF7 | ILMN_2349061 | 0.115 | 0.0255 | 2.31 | -4.03675 | 0.643 |
| CHD6 | ILMN_1781816 | 0.0662 | 0.0121 | 2.61 | -3.37974 | 0.642 |
| NOCT | ILMN_1689378 | 0.000282 | 9.25E-06 | 4.99 | 3.34195 | 0.641 |
| SEPHS2 | ILMN_1687824 | 0.0503 | 0.00829 | 2.76 | -3.03748 | 0.639 |
| NCK2 | ILMN_1724718 | 0.00576 | 0.000478 | 3.76 | -0.40273 | 0.637 |
| HAUS4 | ILMN_1771003 | 0.0182 | 0.00216 | 3.25 | -1.80918 | 0.637 |
| SSPN | ILMN_1775486 | 0.0702 | 0.0131 | 2.58 | -3.45108 | 0.637 |
| GADD45A | ILMN_2052208 | 4.33E-07 | 1.41E-09 | 7.55 | 11.79616 | 0.636 |
| RNF115 | ILMN_1811997 | 0.0177 | 0.00209 | 3.26 | -1.77656 | 0.636 |
| APOL6 | ILMN_1687201 | 0.117 | 0.0261 | 2.3 | -4.05852 | 0.636 |
| FOXP1 | ILMN_2250923 | 0.0314 | 0.00447 | 2.99 | -2.47538 | 0.634 |
| PTRH2 | ILMN_2311548 | 0.000155 | 4.12E-06 | 5.23 | 4.11598 | 0.633 |
| XPO6 | ILMN_1755235 | 0.000233 | 7.22E-06 | 5.06 | 3.57878 | 0.633 |
| TSC22D3 | ILMN_2276952 | 0.00314 | 0.000218 | 4.01 | 0.33693 | 0.632 |
| DDX60L | ILMN_3243928 | 0.0764 | 0.0147 | 2.53 | -3.55233 | 0.632 |
| INTS6-AS1 | ILMN_1905075 | 2.52E-05 | 3.98E-07 | 5.91 | 6.36056 | 0.629 |
| HBEGF | ILMN_2121408 | 0.00299 | 0.000205 | 4.03 | 0.39462 | 0.628 |
| LONP2 | ILMN_1691480 | 0.0499 | 0.0082 | 2.76 | -3.02783 | 0.627 |
| KLF10 | ILMN_1667594 | 0.000627 | 2.59E-05 | 4.68 | 2.35968 | 0.626 |
| KIAA0040 | ILMN_1797191 | 0.0148 | 0.00166 | 3.34 | -1.56632 | 0.626 |
| UBE2J1 | ILMN_1713759 | 2.28E-06 | 1.34E-08 | 6.89 | 9.6248 | 0.624 |
| ACSS2 | ILMN_1697510 | 0.000473 | 1.80E-05 | 4.79 | 2.70789 | 0.624 |
| PRKDC | ILMN_1769517 | 0.0798 | 0.0156 | 2.51 | -3.60168 | 0.624 |
| DHRS12 | ILMN_1669177 | 0.0944 | 0.0196 | 2.42 | -3.80509 | 0.622 |
| LIMK2 | ILMN_1660624 | 0.0977 | 0.0205 | 2.4 | -3.8442 | 0.622 |
| ADCY3 | ILMN_1676893 | 0.171 | 0.0448 | 2.06 | -4.52407 | 0.622 |
| STOM | ILMN_1766657 | 0.119 | 0.0269 | 2.29 | -4.08532 | 0.621 |
| PDE4B | ILMN_2340259 | 5.47E-07 | 1.85E-09 | 7.47 | 11.53038 | 0.62 |
| DKFZP586I1420 | ILMN_2216838 | 0.000988 | 4.69E-05 | 4.49 | 1.7917 | 0.618 |
| CLEC12A | ILMN_2292178 | 0.0407 | 0.00624 | 2.87 | -2.77984 | 0.618 |
| PRPS1 | ILMN_1667050 | 0.000253 | 8.03E-06 | 5.03 | 3.47734 | 0.616 |
| DEK | ILMN_1747630 | 0.00756 | 0.000681 | 3.64 | -0.73375 | 0.616 |
| RBM3 | ILMN_1698213 | 6.71E-08 | 1.23E-10 | 8.26 | 14.1355 | 0.613 |
| ADRB2 | ILMN_1695590 | 0.000117 | 2.92E-06 | 5.33 | 4.44707 | 0.613 |
| AUH | ILMN_2231911 | 5.02E-05 | 9.92E-07 | 5.64 | 5.48259 | 0.61 |
| GK | ILMN_2393296 | 0.0257 | 0.00345 | 3.08 | -2.23997 | 0.609 |
| ETS2 | ILMN_1720158 | 0.0266 | 0.0036 | 3.07 | -2.27847 | 0.609 |
| ZSCAN16 | ILMN_1809566 | 0.0401 | 0.00613 | 2.87 | -2.76357 | 0.607 |
| NCOA7 | ILMN_1687768 | 0.133 | 0.0314 | 2.22 | -4.2184 | 0.607 |
| G0S2 | ILMN_1691846 | 0.000275 | 8.95E-06 | 5 | 3.37339 | 0.605 |
| AIM2 | ILMN_1681301 | 0.175 | 0.0463 | 2.05 | -4.55214 | 0.605 |
| VMP1 | ILMN_1692754 | 3.17E-07 | 9.54E-10 | 7.66 | 12.16939 | 0.604 |
| ACOT7 | ILMN_2332250 | 0.0103 | 0.00102 | 3.51 | -1.11285 | 0.604 |
| EIF2AK3 | ILMN_1724984 | 0.0237 | 0.00309 | 3.12 | -2.13794 | 0.604 |
| TNFSF8 | ILMN_1761778 | 0.123 | 0.0282 | 2.27 | -4.12364 | 0.604 |
| CNTNAP3 | ILMN_1717541 | 0.00779 | 0.00071 | 3.63 | -0.77386 | 0.602 |
| HES4 | ILMN_1653466 | 0.122 | 0.028 | 2.27 | -4.11902 | 0.602 |
| ZMYND15 | ILMN_1778136 | 1.34E-05 | 1.59E-07 | 6.18 | 7.24346 | 0.599 |
| KLF10 | ILMN_2411897 | 0.00282 | 0.000189 | 4.06 | 0.47027 | 0.599 |
| SNORD103C | ILMN_3247303 | 0.00233 | 0.000148 | 4.14 | 0.70053 | 0.597 |
| PHC2 | ILMN_1670147 | 0.00552 | 0.000453 | 3.78 | -0.35159 | 0.596 |
| MAP2K1 | ILMN_1694240 | 0.0132 | 0.00143 | 3.39 | -1.42437 | 0.596 |
| GRINA | ILMN_2370872 | 0.0471 | 0.00759 | 2.79 | -2.95762 | 0.596 |
| MBNL1 | ILMN_1807304 | 0.00151 | 8.14E-05 | 4.32 | 1.2686 | 0.595 |
| HIPK2 | ILMN_1912737 | 0.0538 | 0.00918 | 2.72 | -3.1292 | 0.595 |
| MLX | ILMN_1792207 | 0.012 | 0.00126 | 3.44 | -1.30885 | 0.594 |
| SRGAP2 | ILMN_1759549 | 0.13 | 0.0306 | 2.23 | -4.19601 | 0.593 |
| TRIM5 | ILMN_1704972 | 0.107 | 0.0233 | 2.35 | -3.95899 | 0.592 |
| FAR2 | ILMN_2202915 | 0.0106 | 0.00107 | 3.49 | -1.15225 | 0.591 |
| ATPAF1 | ILMN_1790603 | 0.021 | 0.00261 | 3.18 | -1.98301 | 0.591 |
| DSC2 | ILMN_2381257 | 0.0562 | 0.0097 | 2.7 | -3.17907 | 0.591 |
| ODF3B | ILMN_3241446 | 0.000413 | 1.50E-05 | 4.84 | 2.88112 | 0.59 |
| FAM170B | ILMN_1738047 | 0.0499 | 0.0082 | 2.76 | -3.02806 | 0.589 |
| ANKRD36B | ILMN_1780806 | 0.102 | 0.0218 | 2.38 | -3.89809 | 0.589 |
| REXO2 | ILMN_1749009 | 0.00522 | 0.000422 | 3.8 | -0.28613 | 0.588 |
| REPS2 | ILMN_1724668 | 0.00884 | 0.000834 | 3.58 | -0.92317 | 0.588 |
| DHX58 | ILMN_1678422 | 0.101 | 0.0215 | 2.38 | -3.88822 | 0.587 |
| ST8SIA4 | ILMN_2394161 | 0.000288 | 9.50E-06 | 4.98 | 3.31669 | 0.585 |
| DNAJC25 | ILMN_1778161 | 0.0939 | 0.0194 | 2.42 | -3.79778 | 0.585 |
| SPTLC1 | ILMN_1665094 | 0.000147 | 3.85E-06 | 5.25 | 4.18189 | 0.584 |
| STK16 | ILMN_1742520 | 0.00114 | 5.68E-05 | 4.44 | 1.61136 | 0.584 |
| AZIN1 | ILMN_1704550 | 0.000221 | 6.72E-06 | 5.08 | 3.64796 | 0.583 |
| NUCB1 | ILMN_1722634 | 0.0822 | 0.0162 | 2.5 | -3.63659 | 0.583 |
| ZFP36 | ILMN_1720829 | 5.61E-08 | 9.77E-11 | 8.33 | 14.35903 | 0.582 |
| C9orf72 | ILMN_2295252 | 0.00703 | 0.000617 | 3.68 | -0.64224 | 0.582 |
| TMEM119 | ILMN_1738116 | 0.1 | 0.0212 | 2.39 | -3.87724 | 0.581 |
| WDR13 | ILMN_1805512 | 0.0132 | 0.00144 | 3.39 | -1.43131 | 0.579 |
| IRF7 | ILMN_1798181 | 0.119 | 0.027 | 2.28 | -4.08824 | 0.579 |
| EBP | ILMN_2073604 | 0.0116 | 0.0012 | 3.45 | -1.26423 | 0.578 |
| COX14 | ILMN_1804656 | 7.65E-05 | 1.71E-06 | 5.48 | 4.95931 | 0.576 |
| TMLHE | ILMN_1683575 | 8.08E-05 | 1.84E-06 | 5.46 | 4.88937 | 0.575 |
| LOC100128059 | ILMN_1832033 | 0.0336 | 0.00486 | 2.96 | -2.55358 | 0.575 |
| LMTK2 | ILMN_1724315 | 0.178 | 0.0476 | 2.04 | -4.57512 | 0.575 |
| HNRNPA3 | ILMN_1761083 | 4.26E-07 | 1.34E-09 | 7.56 | 11.83952 | 0.574 |
| H2AFY | ILMN_1674034 | 0.000467 | 1.75E-05 | 4.79 | 2.72995 | 0.574 |
| PNPLA8 | ILMN_1680223 | 0.0106 | 0.00107 | 3.49 | -1.16001 | 0.574 |
| TEX30 | ILMN_1682774 | 0.00218 | 0.000135 | 4.17 | 0.78808 | 0.573 |
| IL1RL1 | ILMN_2313672 | 0.0151 | 0.00171 | 3.33 | -1.59092 | 0.572 |
| MRPS34 | ILMN_2210482 | 0.0644 | 0.0116 | 2.63 | -3.3406 | 0.572 |
| MS4A6A | ILMN_2359800 | 0.0037 | 0.00027 | 3.95 | 0.13373 | 0.571 |
| VPS9D1 | ILMN_1693630 | 0.0531 | 0.00899 | 2.73 | -3.11107 | 0.571 |
| TTI2 | ILMN_1760400 | 3.92E-06 | 2.86E-08 | 6.67 | 8.89459 | 0.57 |
| EPG5 | ILMN_1762608 | 0.0874 | 0.0176 | 2.46 | -3.70931 | 0.57 |
| HPS1 | ILMN_2267787 | 0.00086 | 3.89E-05 | 4.55 | 1.96958 | 0.569 |
| CCNL1 | ILMN_1683129 | 0.0239 | 0.00313 | 3.12 | -2.14929 | 0.568 |
| RP9 | ILMN_1687922 | 0.00758 | 0.000683 | 3.64 | -0.73724 | 0.567 |
| DRD3 | ILMN_1738251 | 3.09E-06 | 2.04E-08 | 6.77 | 9.22259 | 0.566 |
| TBC1D15 | ILMN_1803941 | 0.00121 | 6.17E-05 | 4.41 | 1.53199 | 0.566 |
| NUDT16P1 | ILMN_3239937 | 0.138 | 0.0329 | 2.2 | -4.25812 | 0.566 |
| TNFSF13B | ILMN_1758418 | 0.0806 | 0.0158 | 2.51 | -3.6147 | 0.565 |
| OSBPL9 | ILMN_2313856 | 0.00409 | 0.000308 | 3.9 | 0.01089 | 0.564 |
| CITED4 | ILMN_1787691 | 0.0023 | 0.000146 | 4.14 | 0.71814 | 0.563 |
| JADE1 | ILMN_1794473 | 0.00259 | 0.00017 | 4.09 | 0.57367 | 0.563 |
| ST3GAL1 | ILMN_1683313 | 0.000104 | 2.55E-06 | 5.37 | 4.57558 | 0.561 |
| SDF2L1 | ILMN_1749213 | 0.00114 | 5.65E-05 | 4.44 | 1.61492 | 0.56 |
| UHRF2 | ILMN_1691444 | 0.00935 | 0.000898 | 3.55 | -0.99271 | 0.56 |
| TRIM5 | ILMN_2404665 | 0.0823 | 0.0162 | 2.5 | -3.63728 | 0.559 |
| UBE2L6 | ILMN_1769520 | 0.0874 | 0.0176 | 2.46 | -3.70979 | 0.559 |
| PARP4 | ILMN_1776464 | 0.00153 | 8.32E-05 | 4.32 | 1.24794 | 0.558 |
| CST7 | ILMN_1679826 | 0.0902 | 0.0183 | 2.45 | -3.74697 | 0.558 |
| ST3GAL1 | ILMN_2267135 | 0.00422 | 0.000321 | 3.89 | -0.02931 | 0.557 |
| GIPC1 | ILMN_1796177 | 0.0436 | 0.00685 | 2.83 | -2.86535 | 0.556 |
| FAM177A1 | ILMN_2287276 | 0.000103 | 2.50E-06 | 5.37 | 4.59459 | 0.555 |
| OSBPL1A | ILMN_2405602 | 0.00203 | 0.000123 | 4.2 | 0.87566 | 0.554 |
| TET2 | ILMN_1788818 | 0.0122 | 0.00129 | 3.43 | -1.3335 | 0.554 |
| ACAT2 | ILMN_1708672 | 0.0196 | 0.00239 | 3.22 | -1.90088 | 0.552 |
| NR4A3 | ILMN_1781812 | 0.0514 | 0.00859 | 2.75 | -3.06923 | 0.552 |
| IFIT3 | ILMN_2239754 | 0.0567 | 0.00985 | 2.69 | -3.19247 | 0.552 |
| SLA | ILMN_2291954 | 0.12 | 0.0272 | 2.28 | -4.09197 | 0.552 |
| STAT2 | ILMN_1690921 | 0.171 | 0.0452 | 2.06 | -4.53075 | 0.552 |
| RBM33 | ILMN_1741244 | 0.00086 | 3.89E-05 | 4.55 | 1.97106 | 0.551 |
| FCMR | ILMN_1775542 | 0.09 | 0.0183 | 2.45 | -3.74384 | 0.551 |
| PRKAG2 | ILMN_1749419 | 0.0749 | 0.0143 | 2.55 | -3.52813 | 0.55 |
| SNORD13 | ILMN_1892403 | 0.000212 | 6.34E-06 | 5.1 | 3.70316 | 0.549 |
| MEGF9 | ILMN_1658798 | 0.103 | 0.022 | 2.37 | -3.90617 | 0.549 |
| PPP1R15A | ILMN_1659936 | 2.20E-06 | 1.26E-08 | 6.91 | 9.68202 | 0.548 |
| NSUN7 | ILMN_2205882 | 0.0122 | 0.00129 | 3.43 | -1.3269 | 0.548 |
| TSC22D1 | ILMN_1692177 | 0.0196 | 0.00239 | 3.21 | -1.90271 | 0.548 |
| MT1A | ILMN_1691156 | 5.92E-06 | 5.35E-08 | 6.49 | 8.29285 | 0.546 |
| IQCC | ILMN_1774589 | 0.00297 | 0.000202 | 4.04 | 0.40607 | 0.546 |
| SLC31A2 | ILMN_1758938 | 0.0524 | 0.0088 | 2.74 | -3.09177 | 0.546 |
| ZBED1 | ILMN_1694466 | 0.000356 | 1.24E-05 | 4.9 | 3.06296 | 0.545 |
| ARID5A | ILMN_2298567 | 0.00106 | 5.18E-05 | 4.46 | 1.6989 | 0.545 |
| ABHD17C | ILMN_1788416 | 0.12 | 0.0275 | 2.28 | -4.10224 | 0.545 |
| CDK5R1 | ILMN_1730928 | 0.149 | 0.0368 | 2.15 | -4.35473 | 0.545 |
| MBNL1 | ILMN_2313158 | 0.00576 | 0.000477 | 3.76 | -0.39996 | 0.544 |
| JARID2-AS1 | ILMN_1856564 | 5.91E-05 | 1.23E-06 | 5.58 | 5.27836 | 0.542 |
| EEF1A1 | ILMN_3251737 | 4.61E-06 | 3.64E-08 | 6.6 | 8.66268 | 0.541 |
| LOC100419583 | ILMN_1845037 | 0.0189 | 0.00229 | 3.23 | -1.86086 | 0.541 |
| CRY1 | ILMN_2202637 | 0.0339 | 0.00493 | 2.95 | -2.56507 | 0.541 |
| APH1B | ILMN_1767816 | 0.000953 | 4.47E-05 | 4.51 | 1.83793 | 0.54 |
| IDNK | ILMN_1791306 | 0.0105 | 0.00106 | 3.5 | -1.14301 | 0.539 |
| ZDHHC20 | ILMN_1654141 | 0.034 | 0.00494 | 2.95 | -2.56686 | 0.539 |
| TNFSF13B | ILMN_2066858 | 0.065 | 0.0118 | 2.62 | -3.35199 | 0.538 |
| SNHG5 | ILMN_2200659 | 2.49E-08 | 2.93E-11 | 8.69 | 15.5157 | 0.536 |
| CEBPD | ILMN_1782050 | 0.000147 | 3.85E-06 | 5.25 | 4.18215 | 0.536 |
| PRDM8 | ILMN_1802082 | 0.00153 | 8.28E-05 | 4.32 | 1.25281 | 0.535 |
| UBC | ILMN_2252160 | 0.0306 | 0.00429 | 3 | -2.4396 | 0.534 |
| HSPA1B | ILMN_1660436 | 0.113 | 0.0248 | 2.32 | -4.01454 | 0.534 |
| EPCAM | ILMN_2160210 | 0.168 | 0.0438 | 2.07 | -4.50495 | 0.534 |
| TXN | ILMN_1680314 | 0.00596 | 0.000499 | 3.75 | -0.44265 | 0.533 |
| ELF2 | ILMN_2252295 | 0.000408 | 1.47E-05 | 4.85 | 2.90138 | 0.532 |
| AGTRAP | ILMN_1802458 | 0.00408 | 0.000307 | 3.91 | 0.01468 | 0.532 |
| DDX60 | ILMN_1795181 | 0.174 | 0.0461 | 2.05 | -4.54737 | 0.531 |
| TUBA1A | ILMN_1742981 | 0.0188 | 0.00226 | 3.23 | -1.84901 | 0.53 |
| HIST2H2AB | ILMN_1747589 | 0.024 | 0.00315 | 3.12 | -2.15505 | 0.53 |
| YPEL4 | ILMN_1726624 | 0.0619 | 0.0111 | 2.65 | -3.29715 | 0.53 |
| CDS2 | ILMN_1790973 | 0.00344 | 0.000247 | 3.98 | 0.21989 | 0.529 |
| IL1RL2 | ILMN_1762810 | 0.0215 | 0.00271 | 3.17 | -2.01573 | 0.529 |
| UBE2F | ILMN_2164242 | 0.0679 | 0.0126 | 2.6 | -3.41253 | 0.529 |
| DTNBP1 | ILMN_2408240 | 0.0716 | 0.0135 | 2.57 | -3.47613 | 0.529 |
| CLHC1 | ILMN_1805148 | 0.0772 | 0.0149 | 2.53 | -3.56305 | 0.529 |
| SP140 | ILMN_1703263 | 0.0854 | 0.017 | 2.48 | -3.68142 | 0.529 |
| TUBA4A | ILMN_1784300 | 0.0263 | 0.00355 | 3.07 | -2.26631 | 0.527 |
| AP5B1 | ILMN_1717594 | 0.0709 | 0.0133 | 2.57 | -3.46443 | 0.527 |
| UBIAD1 | ILMN_1651872 | 0.0934 | 0.0193 | 2.43 | -3.79083 | 0.525 |
| ASAH1 | ILMN_1684054 | 0.000364 | 1.27E-05 | 4.89 | 3.03674 | 0.524 |
| OSBPL9 | ILMN_2313851 | 0.0032 | 0.000224 | 4.01 | 0.30995 | 0.524 |
| SDHAP1 | ILMN_1666757 | 0.000452 | 1.69E-05 | 4.81 | 2.76806 | 0.523 |
| LIPN | ILMN_3243466 | 0.0016 | 8.81E-05 | 4.3 | 1.19416 | 0.522 |
| SPATA6 | ILMN_1775926 | 0.0506 | 0.00836 | 2.76 | -3.04527 | 0.522 |
| ZNF394 | ILMN_1807514 | 2.23E-08 | 2.50E-11 | 8.74 | 15.66763 | 0.521 |
| CHSY1 | ILMN_1791576 | 0.00428 | 0.000327 | 3.88 | -0.04518 | 0.521 |
| STAT4 | ILMN_1785202 | 0.00503 | 0.000402 | 3.82 | -0.23935 | 0.521 |
| MLLT1 | ILMN_1706590 | 0.0678 | 0.0126 | 2.6 | -3.4115 | 0.52 |
| KYAT3 | ILMN_1778371 | 0.000355 | 1.23E-05 | 4.9 | 3.06845 | 0.518 |
| MMP23A | ILMN_2317701 | 0.015 | 0.00169 | 3.34 | -1.58058 | 0.518 |
| MRPL15 | ILMN_2103720 | 0.0251 | 0.00334 | 3.1 | -2.20873 | 0.518 |
| IFI16 | ILMN_1710937 | 0.139 | 0.0334 | 2.19 | -4.27281 | 0.518 |
| SMARCD3 | ILMN_2309180 | 0.014 | 0.00154 | 3.37 | -1.49553 | 0.517 |
| 2-Mar | ILMN_1703142 | 0.00738 | 0.000658 | 3.66 | -0.70204 | 0.516 |
| IL13RA1 | ILMN_1768505 | 0.000516 | 1.99E-05 | 4.76 | 2.61162 | 0.515 |
| N4BP2L1 | ILMN_2344650 | 0.0214 | 0.00269 | 3.17 | -2.00955 | 0.515 |
| LOC494141 | ILMN_3292784 | 0.0305 | 0.00427 | 3.01 | -2.43451 | 0.515 |
| ZNF165 | ILMN_1806502 | 0.0708 | 0.0133 | 2.57 | -3.46335 | 0.515 |
| TLE1 | ILMN_1751572 | 0.105 | 0.0227 | 2.36 | -3.93553 | 0.514 |
| H2AFY | ILMN_1746171 | 0.00293 | 2.00E-04 | 4.04 | 0.41982 | 0.513 |
| VPS13B | ILMN_2332368 | 0.00789 | 0.000722 | 3.62 | -0.78917 | 0.511 |
| LOC105371763 | ILMN_1873677 | 0.0386 | 0.00583 | 2.89 | -2.71798 | 0.511 |
| AGPAT2 | ILMN_1681081 | 0.039 | 0.0059 | 2.89 | -2.7291 | 0.511 |
| MDM2 | ILMN_1736829 | 0.0056 | 0.00046 | 3.77 | -0.36669 | 0.509 |
| SLC1A5 | ILMN_1707720 | 0.052 | 0.00873 | 2.74 | -3.08413 | 0.509 |
| RPL26L1 | ILMN_2110532 | 5.00E-05 | 9.86E-07 | 5.65 | 5.48814 | 0.508 |
| C5orf30 | ILMN_1677292 | 0.0683 | 0.0127 | 2.59 | -3.42035 | 0.508 |
| ZFAND5 | ILMN_1795228 | 0.00727 | 0.000645 | 3.66 | -0.68313 | 0.507 |
| SKP1 | ILMN_3229770 | 9.64E-09 | 8.17E-12 | 9.08 | 16.73743 | 0.506 |
| TCP11L2 | ILMN_2231051 | 1.09E-05 | 1.22E-07 | 6.25 | 7.50016 | 0.506 |
| SP140 | ILMN_2246882 | 0.0795 | 0.0155 | 2.51 | -3.59703 | 0.506 |
| C4orf32 | ILMN_1700257 | 0.0758 | 0.0146 | 2.54 | -3.54203 | 0.505 |
| FURIN | ILMN_1790228 | 0.0119 | 0.00125 | 3.44 | -1.29836 | 0.504 |
| REPS2 | ILMN_1766425 | 0.0169 | 0.00197 | 3.28 | -1.72351 | 0.503 |
| FAM134B | ILMN_2387952 | 0.155 | 0.0391 | 2.12 | -4.40714 | 0.503 |
| MCUB | ILMN_1801766 | 2.98E-05 | 4.92E-07 | 5.85 | 6.15642 | 0.501 |
| UCHL5 | ILMN_1731612 | 0.00153 | 8.32E-05 | 4.32 | 1.24759 | 0.5 |
| PRPSAP2 | ILMN_1812445 | 0.000559 | 2.20E-05 | 4.73 | 2.51368 | 0.499 |
| ERN1 | ILMN_1731231 | 0.00213 | 0.000132 | 4.17 | 0.81417 | 0.499 |
| PAPSS1 | ILMN_1781819 | 0.0477 | 0.00772 | 2.79 | -2.97337 | 0.499 |
| RNY4 | ILMN_3241021 | 4.90E-06 | 4.01E-08 | 6.57 | 8.57017 | 0.498 |
| ELF2 | ILMN_1691559 | 7.14E-06 | 7.02E-08 | 6.41 | 8.03026 | 0.498 |
| TREM1 | ILMN_1688231 | 8.55E-05 | 1.97E-06 | 5.44 | 4.82475 | 0.498 |
| YOD1 | ILMN_1678919 | 0.0113 | 0.00116 | 3.46 | -1.23422 | 0.498 |
| LGALS8 | ILMN_2353358 | 0.124 | 0.0284 | 2.26 | -4.13223 | 0.498 |
| AGTRAP | ILMN_2307656 | 0.0103 | 0.00102 | 3.51 | -1.11459 | 0.497 |
| IGF2BP3 | ILMN_1807423 | 0.038 | 0.00571 | 2.9 | -2.70021 | 0.497 |
| RPS2 | ILMN_2218277 | 0.00493 | 0.000392 | 3.83 | -0.2166 | 0.496 |
| ARID5A | ILMN_2415157 | 1.57E-05 | 2.00E-07 | 6.11 | 7.02226 | 0.495 |
| RNU4ATAC | ILMN_3240594 | 4.16E-05 | 7.55E-07 | 5.72 | 5.74463 | 0.493 |
| GPR183 | ILMN_1798706 | 0.000144 | 3.73E-06 | 5.26 | 4.21267 | 0.493 |
| KDSR | ILMN_3241164 | 0.167 | 0.0435 | 2.08 | -4.49946 | 0.493 |
| RGMA | ILMN_1717636 | 0.00332 | 0.000236 | 3.99 | 0.26343 | 0.492 |
| PDCD4-AS1 | ILMN_3199489 | 9.18E-06 | 9.59E-08 | 6.32 | 7.72993 | 0.491 |
| AAED1 | ILMN_1717229 | 0.0153 | 0.00174 | 3.33 | -1.60913 | 0.49 |
| NT5C3A | ILMN_1769734 | 0.000911 | 4.22E-05 | 4.53 | 1.89357 | 0.489 |
| GTDC1 | ILMN_1832252 | 0.00126 | 6.49E-05 | 4.4 | 1.48404 | 0.489 |
| TES | ILMN_1746243 | 0.00262 | 0.000172 | 4.09 | 0.55881 | 0.489 |
| IL6 | ILMN_1699651 | 0.00833 | 0.000775 | 3.6 | -0.85476 | 0.489 |
| KLF5 | ILMN_1770293 | 0.182 | 0.0491 | 2.02 | -4.60119 | 0.489 |
| CIRBP | ILMN_1674661 | 9.16E-06 | 9.53E-08 | 6.32 | 7.73673 | 0.485 |
| PDCL3 | ILMN_1667925 | 0.00862 | 0.000808 | 3.59 | -0.89432 | 0.484 |
| BCL6 | ILMN_1737314 | 0.0197 | 0.0024 | 3.21 | -1.90549 | 0.484 |
| IFT20 | ILMN_3235216 | 0.00671 | 0.000583 | 3.7 | -0.5891 | 0.483 |
| RNF10 | ILMN_1697529 | 3.94E-07 | 1.22E-09 | 7.59 | 11.92997 | 0.482 |
| SERINC3 | ILMN_1665065 | 0.000556 | 2.18E-05 | 4.73 | 2.52079 | 0.482 |
| ST3GAL1 | ILMN_1860638 | 0.0102 | 0.00101 | 3.51 | -1.10237 | 0.482 |
| TMEM70 | ILMN_2373010 | 0.0205 | 0.00254 | 3.19 | -1.95897 | 0.482 |
| GSTM2 | ILMN_2201580 | 0.128 | 0.0297 | 2.24 | -4.1709 | 0.481 |
| DLGAP1-AS1 | ILMN_3307940 | 0.00251 | 0.000163 | 4.11 | 0.61223 | 0.48 |
| TMED10P1 | ILMN_2213558 | 0.00239 | 0.000153 | 4.13 | 0.66853 | 0.479 |
| ATPIF1 | ILMN_1727332 | 0.00173 | 9.89E-05 | 4.26 | 1.08387 | 0.478 |
| REPS2 | ILMN_2405797 | 0.0282 | 0.00385 | 3.04 | -2.34069 | 0.478 |
| GBP6 | ILMN_1756953 | 0.17 | 0.0446 | 2.06 | -4.52055 | 0.478 |
| METTL21A | ILMN_3251137 | 0.0441 | 0.00698 | 2.82 | -2.88177 | 0.476 |
| KLF4 | ILMN_1779857 | 0.0125 | 0.00133 | 3.42 | -1.35851 | 0.475 |
| GRASP | ILMN_1705210 | 0.0605 | 0.0107 | 2.66 | -3.26966 | 0.475 |
| RIN1 | ILMN_1660858 | 0.00124 | 6.34E-05 | 4.4 | 1.50648 | 0.474 |
| RNF144A | ILMN_3238326 | 0.0582 | 0.0102 | 2.68 | -3.22375 | 0.474 |
| ABHD2 | ILMN_2403446 | 0.00332 | 0.000236 | 3.99 | 0.26155 | 0.473 |
| ATG14 | ILMN_1678808 | 0.0119 | 0.00125 | 3.44 | -1.30401 | 0.473 |
| BLOC1S6 | ILMN_1733666 | 0.155 | 0.0393 | 2.12 | -4.41079 | 0.473 |
| SRSF3 | ILMN_1723212 | 1.20E-08 | 1.07E-11 | 9 | 16.47833 | 0.472 |
| ZFAS1 | ILMN_3188984 | 3.67E-05 | 6.38E-07 | 5.77 | 5.90644 | 0.471 |
| C12orf57 | ILMN_1812191 | 0.00316 | 0.000221 | 4.01 | 0.32558 | 0.471 |
| SH3GLB1 | ILMN_1766045 | 0.000614 | 2.52E-05 | 4.68 | 2.38507 | 0.47 |
| EEF1B2 | ILMN_1701930 | 0.000287 | 9.44E-06 | 4.98 | 3.32202 | 0.469 |
| CCNH | ILMN_2187830 | 0.00669 | 0.00058 | 3.7 | -0.58411 | 0.469 |
| SLC36A4 | ILMN_2082324 | 0.00745 | 0.000667 | 3.65 | -0.71432 | 0.469 |
| 2-Mar | ILMN_2337789 | 0.0557 | 0.00959 | 2.7 | -3.16841 | 0.469 |
| ING3 | ILMN_2381603 | 2.03E-05 | 2.93E-07 | 6 | 6.65565 | 0.468 |
| ENTPD1 | ILMN_1773125 | 5.73E-05 | 1.18E-06 | 5.59 | 5.31981 | 0.468 |
| USP15 | ILMN_1867588 | 0.000603 | 2.46E-05 | 4.69 | 2.40845 | 0.468 |
| PHC2 | ILMN_1808047 | 0.00579 | 0.000483 | 3.76 | -0.41228 | 0.468 |
| LOC105372881 | ILMN_1867439 | 0.00543 | 0.000444 | 3.79 | -0.33315 | 0.466 |
| RFC2 | ILMN_1655733 | 0.0159 | 0.00182 | 3.31 | -1.65091 | 0.466 |
| RNU5A-1 | ILMN_3237617 | 0.0237 | 0.00309 | 3.12 | -2.13905 | 0.466 |
| YBX3 | ILMN_1782788 | 0.0119 | 0.00126 | 3.44 | -1.30589 | 0.465 |
| GSTM1 | ILMN_2391861 | 0.175 | 0.0463 | 2.05 | -4.55268 | 0.465 |
| PSMG1 | ILMN_1779264 | 0.0499 | 0.00821 | 2.76 | -3.02863 | 0.464 |
| UGCG | ILMN_1736939 | 0.0659 | 0.012 | 2.62 | -3.37124 | 0.464 |
| TCAF2 | ILMN_3184048 | 0.0906 | 0.0185 | 2.44 | -3.75449 | 0.464 |
| CR1 | ILMN_1742601 | 0.12 | 0.0274 | 2.28 | -4.09866 | 0.464 |
| IL1RL1 | ILMN_2242900 | 0.00808 | 0.000744 | 3.61 | -0.81741 | 0.463 |
| TAF6L | ILMN_1727281 | 0.0229 | 0.00295 | 3.14 | -2.0955 | 0.463 |
| MAN1A1 | ILMN_1742187 | 0.0829 | 0.0164 | 2.49 | -3.64714 | 0.463 |
| PLIN2 | ILMN_1801077 | 0.0571 | 0.00993 | 2.69 | -3.2006 | 0.462 |
| SLK | ILMN_2159322 | 0.0612 | 0.0109 | 2.65 | -3.2838 | 0.462 |
| TEK | ILMN_2066151 | 0.069 | 0.0129 | 2.59 | -3.43139 | 0.462 |
| SERPINA1 | ILMN_1764980 | 0.18 | 0.0483 | 2.03 | -4.58719 | 0.462 |
| BEX4 | ILMN_2351638 | 0.000359 | 1.25E-05 | 4.9 | 3.05261 | 0.461 |
| CNNM2 | ILMN_1663975 | 0.0327 | 0.00468 | 2.97 | -2.51913 | 0.461 |
| USP10 | ILMN_1721116 | 0.0141 | 0.00156 | 3.36 | -1.5048 | 0.46 |
| N4BP2L1 | ILMN_1799487 | 0.113 | 0.025 | 2.32 | -4.02069 | 0.46 |
| ISCA1 | ILMN_1715931 | 0.00494 | 0.000393 | 3.82 | -0.21951 | 0.459 |
| SYNJ1 | ILMN_1762303 | 0.00552 | 0.000453 | 3.78 | -0.35242 | 0.459 |
| IAH1 | ILMN_2217329 | 0.00173 | 9.88E-05 | 4.26 | 1.08474 | 0.458 |
| GPR183 | ILMN_2168217 | 0.00459 | 0.000356 | 3.86 | -0.12693 | 0.458 |
| TMEM187 | ILMN_2198185 | 0.0956 | 0.0199 | 2.41 | -3.81932 | 0.458 |
| C9orf72 | ILMN_1741881 | 0.00395 | 0.000294 | 3.92 | 0.05495 | 0.457 |
| MAZ | ILMN_1677997 | 0.0212 | 0.00266 | 3.18 | -1.99991 | 0.457 |
| EMC9 | ILMN_1755677 | 0.111 | 0.0244 | 2.33 | -3.99867 | 0.457 |
| CCNDBP1 | ILMN_1658743 | 1.18E-06 | 5.06E-09 | 7.17 | 10.5636 | 0.456 |
| BPGM | ILMN_1788764 | 0.00612 | 0.000518 | 3.73 | -0.47832 | 0.456 |
| MGAT4A | ILMN_1755643 | 0.0115 | 0.00119 | 3.46 | -1.2567 | 0.456 |
| PGS1 | ILMN_2075051 | 0.0173 | 0.00204 | 3.27 | -1.75339 | 0.456 |
| MS4A6A | ILMN_1797731 | 0.0419 | 0.00648 | 2.85 | -2.81452 | 0.456 |
| CSNK1D | ILMN_2286870 | 0.000226 | 6.90E-06 | 5.07 | 3.62178 | 0.455 |
| SNORD3C | ILMN_3241034 | 0.00187 | 0.000109 | 4.23 | 0.9925 | 0.455 |
| GGH | ILMN_1681754 | 0.0348 | 0.00508 | 2.94 | -2.59317 | 0.455 |
| DTNBP1 | ILMN_1682960 | 0.05 | 0.00823 | 2.76 | -3.03089 | 0.455 |
| MCL1 | ILMN_1756806 | 0.00022 | 6.69E-06 | 5.08 | 3.65238 | 0.454 |
| GPR65 | ILMN_1734740 | 0.0459 | 0.00737 | 2.8 | -2.9306 | 0.454 |
| PDCL3 | ILMN_1702806 | 0.0465 | 0.00748 | 2.8 | -2.94494 | 0.454 |
| VCPIP1 | ILMN_1682180 | 0.0782 | 0.0152 | 2.52 | -3.5791 | 0.453 |
| ENSA | ILMN_2364700 | 5.70E-07 | 2.04E-09 | 7.44 | 11.43826 | 0.452 |
| JADE1 | ILMN_1736015 | 0.0868 | 0.0174 | 2.47 | -3.70036 | 0.452 |
| SLC46A3 | ILMN_1658639 | 0.121 | 0.0277 | 2.27 | -4.11048 | 0.452 |
| C4orf3 | ILMN_2051684 | 0.182 | 0.0489 | 2.02 | -4.59898 | 0.452 |
| ACOT7 | ILMN_1740265 | 0.00473 | 0.000371 | 3.84 | -0.1633 | 0.451 |
| TLR8 | ILMN_1657892 | 0.0379 | 0.0057 | 2.9 | -2.6976 | 0.451 |
| TAOK2 | ILMN_1715613 | 0.0426 | 0.00665 | 2.84 | -2.83821 | 0.451 |
| TSPAN14///NUP58 | ILMN_1873620 | 0.0658 | 0.012 | 2.62 | -3.36915 | 0.451 |
| XRN1 | ILMN_1809086 | 0.121 | 0.0275 | 2.28 | -4.10443 | 0.451 |
| N4BP2 | ILMN_2222101 | 0.000188 | 5.40E-06 | 5.15 | 3.85721 | 0.449 |
| SOD2 | ILMN_1792922 | 0.11 | 0.0241 | 2.33 | -3.98731 | 0.448 |
| WDFY2 | ILMN_1802292 | 0.00145 | 7.74E-05 | 4.34 | 1.3171 | 0.447 |
| VAPA | ILMN_1690822 | 0.00191 | 0.000112 | 4.22 | 0.96247 | 0.446 |
| ACSL3 | ILMN_1654414 | 0.0268 | 0.00364 | 3.07 | -2.28749 | 0.446 |
| SLC35E3 | ILMN_1749521 | 0.0281 | 0.00384 | 3.05 | -2.3362 | 0.446 |
| GPR65 | ILMN_2232121 | 0.0672 | 0.0124 | 2.6 | -3.39732 | 0.446 |
| DUSP12 | ILMN_1767892 | 0.00222 | 0.000139 | 4.16 | 0.76106 | 0.445 |
| SLC22A15 | ILMN_1730639 | 0.0108 | 0.0011 | 3.48 | -1.18379 | 0.445 |
| CHRAC1 | ILMN_1722390 | 0.0328 | 0.00472 | 2.97 | -2.52508 | 0.445 |
| TSPAN14 | ILMN_1785060 | 0.0883 | 0.0178 | 2.46 | -3.72206 | 0.445 |
| ARHGAP24 | ILMN_1801833 | 0.0145 | 0.00162 | 3.35 | -1.53877 | 0.444 |
| TSNAX | ILMN_2211672 | 0.0487 | 0.00794 | 2.78 | -2.99867 | 0.444 |
| SLK | ILMN_1700834 | 0.0771 | 0.0149 | 2.53 | -3.56196 | 0.444 |
| ING1 | ILMN_1662243 | 2.23E-08 | 2.52E-11 | 8.74 | 15.65742 | 0.442 |
| FLVCR2 | ILMN_2204876 | 0.00571 | 0.000471 | 3.77 | -0.38877 | 0.442 |
| RNF213 | ILMN_2289093 | 0.153 | 0.0383 | 2.13 | -4.38986 | 0.442 |
| TATDN3 | ILMN_1744316 | 0.0067 | 0.000582 | 3.7 | -0.58639 | 0.441 |
| TYMP | ILMN_3223126 | 0.0269 | 0.00366 | 3.06 | -2.29279 | 0.441 |
| RNF144A | ILMN_1777660 | 0.0521 | 0.00875 | 2.74 | -3.08618 | 0.441 |
| TXN | ILMN_2038776 | 0.0272 | 0.0037 | 3.06 | -2.30361 | 0.44 |
| ZNF655 | ILMN_2396292 | 0.000159 | 4.26E-06 | 5.22 | 4.08457 | 0.439 |
| LOC102724190 | ILMN_1855346 | 0.00969 | 0.000944 | 3.53 | -1.0392 | 0.439 |
| KIF27 | ILMN_2366795 | 0.117 | 0.0262 | 2.3 | -4.06182 | 0.438 |
| LGALS8 | ILMN_2356654 | 0.156 | 0.0396 | 2.12 | -4.41781 | 0.438 |
| DUSP1 | ILMN_1781285 | 1.77E-07 | 4.75E-10 | 7.86 | 12.83948 | 0.437 |
| TCP11L2 | ILMN_1772780 | 0.00289 | 0.000196 | 4.05 | 0.43629 | 0.437 |
| HEATR3 | ILMN_1808219 | 0.00597 | 0.000501 | 3.75 | -0.44709 | 0.437 |
| MAP4K5 | ILMN_1759030 | 0.0381 | 0.00574 | 2.9 | -2.70378 | 0.435 |
| SLC25A28 | ILMN_1790472 | 0.0653 | 0.0118 | 2.62 | -3.35674 | 0.435 |
| GNAQ | ILMN_1659923 | 0.12 | 0.0274 | 2.28 | -4.10082 | 0.433 |
| GRPEL1 | ILMN_1670817 | 0.0011 | 5.38E-05 | 4.45 | 1.66173 | 0.432 |
| NT5C3A | ILMN_2352121 | 0.0014 | 7.40E-05 | 4.35 | 1.35936 | 0.432 |
| SNORD12 | ILMN_3236049 | 0.0236 | 0.00306 | 3.13 | -2.12978 | 0.432 |
| NOTCH2NL | ILMN_1656254 | 0.0372 | 0.00556 | 2.91 | -2.67557 | 0.432 |
| PRKDC | ILMN_2253648 | 0.0589 | 0.0103 | 2.67 | -3.23722 | 0.432 |
| SESN1 | ILMN_1800626 | 0.0729 | 0.0138 | 2.56 | -3.4966 | 0.432 |
| ING3 | ILMN_1783170 | 0.0824 | 0.0162 | 2.5 | -3.64014 | 0.432 |
| SOD2 | ILMN_2406501 | 0.0308 | 0.00433 | 3 | -2.4467 | 0.431 |
| TES | ILMN_2311796 | 0.0725 | 0.0137 | 2.56 | -3.49136 | 0.431 |
| ERI1 | ILMN_1652716 | 0.165 | 0.0428 | 2.08 | -4.4843 | 0.431 |
| HEBP2 | ILMN_1755077 | 2.59E-05 | 4.14E-07 | 5.9 | 6.32318 | 0.43 |
| SNORD89 | ILMN_3238662 | 0.000132 | 3.37E-06 | 5.29 | 4.30835 | 0.43 |
| TSEN34 | ILMN_1673111 | 0.00113 | 5.63E-05 | 4.44 | 1.61907 | 0.43 |
| ZNF319 | ILMN_1711361 | 0.0241 | 0.00316 | 3.12 | -2.15932 | 0.43 |
| TMEM97 | ILMN_1753890 | 0.000967 | 4.56E-05 | 4.5 | 1.81874 | 0.429 |
| ANKHD1 | ILMN_2341382 | 0.002 | 0.000121 | 4.2 | 0.89612 | 0.429 |
| PSMD9 | ILMN_1732767 | 0.00463 | 0.000361 | 3.85 | -0.13823 | 0.428 |
| TMLHE | ILMN_1677228 | 0.00683 | 0.000595 | 3.69 | -0.60859 | 0.427 |
| SNRNP27 | ILMN_3239621 | 0.083 | 0.0164 | 2.49 | -3.64941 | 0.427 |
| TMEM187 | ILMN_3242211 | 0.0867 | 0.0174 | 2.47 | -3.69911 | 0.427 |
| AGPAT2 | ILMN_2377430 | 0.107 | 0.0233 | 2.35 | -3.95996 | 0.427 |
| ABHD2 | ILMN_1723662 | 0.00601 | 0.000506 | 3.74 | -0.45616 | 0.426 |
| BEND7 | ILMN_3178043 | 0.0309 | 0.00434 | 3 | -2.44967 | 0.426 |
| CSGALNACT1 | ILMN_2055523 | 0.0897 | 0.0182 | 2.45 | -3.74066 | 0.426 |
| SNHG6 | ILMN_3248443 | 4.41E-05 | 8.21E-07 | 5.7 | 5.66416 | 0.425 |
| WTAP | ILMN_1748904 | 0.000967 | 4.56E-05 | 4.5 | 1.81852 | 0.425 |
| RPS9 | ILMN_1749447 | 4.66E-06 | 3.70E-08 | 6.6 | 8.64626 | 0.424 |
| CCNL1 | ILMN_2094776 | 0.00245 | 0.000158 | 4.12 | 0.63929 | 0.424 |
| TCTA | ILMN_1700001 | 0.0223 | 0.00283 | 3.15 | -2.05834 | 0.424 |
| CENPN | ILMN_1720526 | 0.00294 | 2.00E-04 | 4.04 | 0.41873 | 0.423 |
| CRY1 | ILMN_1760593 | 0.078 | 0.0151 | 2.52 | -3.57485 | 0.423 |
| CAB39L | ILMN_1783598 | 0.0906 | 0.0185 | 2.44 | -3.75424 | 0.423 |
| TAF5 | ILMN_1684802 | 0.0935 | 0.0193 | 2.42 | -3.79289 | 0.423 |
| UHRF1BP1L | ILMN_1799601 | 0.000185 | 5.27E-06 | 5.15 | 3.88018 | 0.422 |
| SLC26A8 | ILMN_1672575 | 0.114 | 0.0253 | 2.31 | -4.02972 | 0.422 |
| CASP4 | ILMN_1678454 | 0.161 | 0.0414 | 2.1 | -4.45624 | 0.421 |
| NEK7 | ILMN_1758548 | 0.00316 | 0.000221 | 4.01 | 0.32423 | 0.42 |
| SF3B5 | ILMN_1689389 | 7.21E-05 | 1.57E-06 | 5.51 | 5.04342 | 0.419 |
| BAMBI | ILMN_1691410 | 0.000845 | 3.79E-05 | 4.56 | 1.99492 | 0.419 |
| RMND5A | ILMN_1714093 | 0.00491 | 0.000389 | 3.83 | -0.21028 | 0.419 |
| DUSP22 | ILMN_1730765 | 0.00551 | 0.000452 | 3.78 | -0.34972 | 0.419 |
| NAPA | ILMN_1713285 | 0.0729 | 0.0139 | 2.56 | -3.49812 | 0.419 |
| ZNF252P | ILMN_3243961 | 0.104 | 0.0224 | 2.36 | -3.92479 | 0.418 |
| ISCA1 | ILMN_2219556 | 0.00523 | 0.000423 | 3.8 | -0.2889 | 0.417 |
| MPZL1 | ILMN_2386008 | 0.0374 | 0.00559 | 2.91 | -2.67959 | 0.417 |
| PPP1R3B | ILMN_1712236 | 0.137 | 0.0328 | 2.2 | -4.25604 | 0.417 |
| NSMCE3 | ILMN_1738622 | 0.049 | 0.00801 | 2.77 | -3.00629 | 0.416 |
| 2-Mar | ILMN_1669592 | 0.0661 | 0.0121 | 2.61 | -3.37679 | 0.416 |
| KLHL2 | ILMN_1701837 | 0.0691 | 0.0129 | 2.59 | -3.43242 | 0.416 |
| C7orf50 | ILMN_1718336 | 0.0905 | 0.0184 | 2.44 | -3.75235 | 0.416 |
| SNORD68 | ILMN_2082762 | 0.000744 | 3.24E-05 | 4.61 | 2.14418 | 0.415 |
| BROX | ILMN_1654864 | 0.00359 | 0.00026 | 3.96 | 0.16933 | 0.415 |
| LOC101927531 | ILMN_1844128 | 0.0265 | 0.00358 | 3.07 | -2.27412 | 0.415 |
| GSTM2 | ILMN_1713162 | 0.099 | 0.0208 | 2.39 | -3.85913 | 0.415 |
| BMX | ILMN_1672307 | 0.0979 | 0.0205 | 2.4 | -3.8463 | 0.414 |
| MKNK1 | ILMN_1750429 | 0.118 | 0.0267 | 2.29 | -4.07666 | 0.414 |
| JMY | ILMN_3251620 | 0.0453 | 0.00722 | 2.81 | -2.91246 | 0.413 |
| ABCA1 | ILMN_1766054 | 0.0677 | 0.0125 | 2.6 | -3.40875 | 0.413 |
| HIPK3 | ILMN_2337551 | 0.128 | 0.0298 | 2.24 | -4.17433 | 0.413 |
| ENSA | ILMN_1760779 | 8.96E-05 | 2.09E-06 | 5.43 | 4.76591 | 0.412 |
| ARHGEF37 | ILMN_1869913 | 0.0295 | 0.0041 | 3.02 | -2.39677 | 0.412 |
| CNTRL | ILMN_1758778 | 0.0525 | 0.00884 | 2.73 | -3.095 | 0.412 |
| PNMA3 | ILMN_1783805 | 0.0781 | 0.0151 | 2.52 | -3.57776 | 0.412 |
| ZP3 | ILMN_1662970 | 0.0143 | 0.00158 | 3.36 | -1.52004 | 0.411 |
| SNTB2 | ILMN_1808374 | 0.0436 | 0.00684 | 2.83 | -2.86363 | 0.411 |
| FES | ILMN_1693650 | 0.081 | 0.0159 | 2.5 | -3.61983 | 0.411 |
| HSPA13 | ILMN_3249645 | 0.034 | 0.00494 | 2.95 | -2.56794 | 0.41 |
| SRBD1 | ILMN_1798827 | 0.0455 | 0.00728 | 2.81 | -2.92042 | 0.41 |
| PRMT5 | ILMN_1811955 | 0.0677 | 0.0125 | 2.6 | -3.40891 | 0.41 |
| ODF3B | ILMN_2209614 | 0.0425 | 0.00661 | 2.85 | -2.83261 | 0.409 |
| RPS9 | ILMN_2038772 | 8.96E-05 | 2.10E-06 | 5.42 | 4.76434 | 0.408 |
| SV2A | ILMN_1702009 | 0.00925 | 0.000886 | 3.56 | -0.97972 | 0.408 |
| SCO2 | ILMN_1701621 | 0.0289 | 0.00398 | 3.03 | -2.37127 | 0.408 |
| SOD1 | ILMN_1662438 | 4.82E-05 | 9.42E-07 | 5.66 | 5.5321 | 0.407 |
| SLC25A6 | ILMN_3240838 | 5.85E-05 | 1.21E-06 | 5.59 | 5.29477 | 0.406 |
| DYNLL2 | ILMN_1772796 | 0.0045 | 0.000349 | 3.86 | -0.10598 | 0.406 |
| FGR | ILMN_2272519 | 0.0218 | 0.00276 | 3.16 | -2.03394 | 0.406 |
| SERF1B | ILMN_1655011 | 0.0376 | 0.00564 | 2.9 | -2.68783 | 0.406 |
| RAB33B | ILMN_1727738 | 0.115 | 0.0254 | 2.31 | -4.03525 | 0.406 |
| PLK3 | ILMN_1679979 | 0.00634 | 0.000543 | 3.72 | -0.52197 | 0.405 |
| DKFZP586I1420 | ILMN_1803856 | 0.0071 | 0.000627 | 3.67 | -0.65639 | 0.405 |
| AP3M2 | ILMN_1676946 | 0.0854 | 0.017 | 2.48 | -3.68142 | 0.405 |
| ARMCX6 | ILMN_1716026 | 0.107 | 0.0232 | 2.35 | -3.9561 | 0.405 |
| GTDC1 | ILMN_1762167 | 0.0289 | 0.00399 | 3.03 | -2.37133 | 0.404 |
| PCGF5 | ILMN_1788059 | 0.041 | 0.0063 | 2.86 | -2.78905 | 0.402 |
| APOBEC3A | ILMN_1680192 | 0.0993 | 0.0209 | 2.39 | -3.86472 | 0.402 |
| RAD23B | ILMN_1722662 | 0.00093 | 4.35E-05 | 4.52 | 1.8648 | 0.401 |
| MSH6 | ILMN_1729051 | 0.0109 | 0.00111 | 3.48 | -1.18894 | 0.401 |
| FAM160B1 | ILMN_3231638 | 0.00111 | 5.48E-05 | 4.45 | 1.64417 | 0.4 |
| TSPAN31 | ILMN_1725079 | 0.0211 | 0.00263 | 3.18 | -1.99006 | 0.4 |
| CYSTM1 | ILMN_1761566 | 0.0503 | 0.00831 | 2.76 | -3.03917 | 0.4 |
| HEATR5A | ILMN_3249669 | 0.00934 | 0.000896 | 3.55 | -0.99026 | 0.399 |
| RAB28 | ILMN_2410864 | 0.012 | 0.00126 | 3.44 | -1.30967 | 0.399 |
| CYBRD1 | ILMN_1712305 | 0.0263 | 0.00354 | 3.08 | -2.2624 | 0.399 |
| TYMP | ILMN_3297126 | 0.0516 | 0.00864 | 2.74 | -3.0745 | 0.399 |
| TMEM107 | ILMN_2413331 | 0.00783 | 0.000715 | 3.63 | -0.78001 | 0.398 |
| RAB27A | ILMN_1665859 | 0.0147 | 0.00165 | 3.35 | -1.55632 | 0.398 |
| OBFC1 | ILMN_1906158 | 0.0934 | 0.0193 | 2.43 | -3.79086 | 0.398 |
| MEGF9 | ILMN_2290118 | 0.176 | 0.0468 | 2.04 | -4.56156 | 0.398 |
| EPS15 | ILMN_1665357 | 0.0229 | 0.00294 | 3.14 | -2.09221 | 0.397 |
| OSBPL5 | ILMN_2307032 | 0.0441 | 0.00697 | 2.83 | -2.88033 | 0.397 |
| RMI2 | ILMN_1790537 | 0.158 | 0.0404 | 2.11 | -4.43471 | 0.397 |
| RAX2 | ILMN_1653412 | 0.00141 | 7.45E-05 | 4.35 | 1.35282 | 0.396 |
| RPRD1A | ILMN_2209180 | 0.00321 | 0.000225 | 4 | 0.30533 | 0.396 |
| DYNAP | ILMN_1746670 | 0.0299 | 0.00417 | 3.02 | -2.41382 | 0.396 |
| ATP1B3 | ILMN_1654322 | 0.0613 | 0.0109 | 2.65 | -3.28688 | 0.396 |
| FBXL15 | ILMN_1663379 | 0.0338 | 0.0049 | 2.96 | -2.56047 | 0.395 |
| EAF2 | ILMN_1708798 | 0.00405 | 0.000304 | 3.91 | 0.02339 | 0.394 |
| ATP5S | ILMN_1679188 | 0.0084 | 0.000783 | 3.6 | -0.86492 | 0.394 |
| ADAT1 | ILMN_1657139 | 0.00872 | 0.000819 | 3.58 | -0.90669 | 0.394 |
| 5-Mar | ILMN_2047430 | 0.00182 | 0.000105 | 4.25 | 1.02573 | 0.393 |
| MTSS1 | ILMN_1676980 | 0.0357 | 0.00525 | 2.93 | -2.6234 | 0.392 |
| TRIM9 | ILMN_1763433 | 0.159 | 0.0408 | 2.11 | -4.44362 | 0.392 |
| HPS1 | ILMN_1741483 | 0.0138 | 0.00152 | 3.37 | -1.47995 | 0.391 |
| ACVR1B | ILMN_1763916 | 0.0254 | 0.00339 | 3.09 | -2.22297 | 0.391 |
| RP9P | ILMN_3244444 | 0.0104 | 0.00104 | 3.5 | -1.1336 | 0.39 |
| ZGPAT | ILMN_2344079 | 0.0272 | 0.0037 | 3.06 | -2.30357 | 0.389 |
| NAPB | ILMN_2181125 | 0.0617 | 0.011 | 2.65 | -3.29327 | 0.388 |
| PSMB9 | ILMN_2376108 | 0.141 | 0.0341 | 2.18 | -4.28954 | 0.388 |
| REPS2 | ILMN_1656934 | 0.0589 | 0.0103 | 2.67 | -3.23734 | 0.387 |
| TCN2 | ILMN_1740572 | 0.0716 | 0.0135 | 2.57 | -3.47615 | 0.387 |
| RALBP1 | ILMN_1791840 | 1.87E-06 | 1.02E-08 | 6.97 | 9.88802 | 0.386 |
| ODF2L | ILMN_1702073 | 0.00357 | 0.000259 | 3.96 | 0.17436 | 0.386 |
| HSDL2 | ILMN_1787843 | 0.00488 | 0.000386 | 3.83 | -0.20185 | 0.386 |
| TLE3 | ILMN_2234412 | 0.0688 | 0.0128 | 2.59 | -3.42708 | 0.386 |
| PLK2 | ILMN_1717706 | 0.0426 | 0.00665 | 2.84 | -2.83754 | 0.385 |
| GRAMD1B | ILMN_3237376 | 0.124 | 0.0286 | 2.26 | -4.13859 | 0.385 |
| ARRDC3-AS1 | ILMN_3235196 | 0.00953 | 0.000923 | 3.54 | -1.01824 | 0.384 |
| KYAT3 | ILMN_2244484 | 0.0201 | 0.00248 | 3.2 | -1.93447 | 0.384 |
| RPL41 | ILMN_1710001 | 7.99E-05 | 1.81E-06 | 5.47 | 4.90747 | 0.383 |
| MFSD14B | ILMN_1703229 | 0.0215 | 0.0027 | 3.17 | -2.01316 | 0.383 |
| ACSL3 | ILMN_2360705 | 0.0219 | 0.00277 | 3.16 | -2.03897 | 0.383 |
| IFI6 | ILMN_2347798 | 0.0573 | 0.01 | 2.69 | -3.20644 | 0.383 |
| REPS2 | ILMN_3250972 | 0.093 | 0.0191 | 2.43 | -3.7855 | 0.383 |
| BCAS2 | ILMN_2148452 | 0.112 | 0.0245 | 2.33 | -4.00302 | 0.383 |
| ZAK | ILMN_1768110 | 0.123 | 0.0284 | 2.26 | -4.13004 | 0.383 |
| PPP1CB | ILMN_2405018 | 0.00087 | 3.96E-05 | 4.55 | 1.95461 | 0.382 |
| SLC22A16 | ILMN_1763609 | 0.0867 | 0.0173 | 2.47 | -3.69747 | 0.382 |
| MANF | ILMN_2183510 | 0.00144 | 7.69E-05 | 4.34 | 1.32335 | 0.381 |
| LIN54 | ILMN_1724062 | 0.00251 | 0.000163 | 4.11 | 0.61197 | 0.381 |
| CARD17 | ILMN_3238525 | 0.101 | 0.0214 | 2.38 | -3.88288 | 0.381 |
| PICALM | ILMN_1778709 | 0.0102 | 0.00102 | 3.51 | -1.10727 | 0.38 |
| SNRNP27 | ILMN_2069945 | 0.144 | 0.0351 | 2.17 | -4.3135 | 0.38 |
| PSMB8 | ILMN_1747195 | 0.0625 | 0.0112 | 2.64 | -3.30966 | 0.379 |
| OAS1 | ILMN_1658247 | 0.151 | 0.0375 | 2.14 | -4.37137 | 0.379 |
| N4BP2L2 | ILMN_1797893 | 0.00627 | 0.000534 | 3.72 | -0.50693 | 0.378 |
| AZIN1-AS1 | ILMN_1899940 | 0.0792 | 0.0154 | 2.52 | -3.59202 | 0.378 |
| LCP2 | ILMN_1658962 | 0.0886 | 0.0179 | 2.46 | -3.72629 | 0.378 |
| CRB1 | ILMN_1762139 | 0.0109 | 0.00112 | 3.48 | -1.19698 | 0.377 |
| EAPP | ILMN_3243744 | 0.00045 | 1.67E-05 | 4.81 | 2.77473 | 0.375 |
| FBXO33 | ILMN_1664826 | 0.000892 | 4.09E-05 | 4.54 | 1.92244 | 0.375 |
| GCHFR | ILMN_1694780 | 0.000432 | 1.58E-05 | 4.83 | 2.82977 | 0.374 |
| POLR2J4 | ILMN_1699383 | 0.00214 | 0.000132 | 4.17 | 0.80856 | 0.374 |
| EEF1B2 | ILMN_1685678 | 0.0361 | 0.00532 | 2.93 | -2.63504 | 0.374 |
| KCTD9 | ILMN_1653612 | 0.0516 | 0.00866 | 2.74 | -3.07642 | 0.374 |
| EIF1 | ILMN_1803846 | 0.000198 | 5.80E-06 | 5.12 | 3.78814 | 0.373 |
| TMEM150A | ILMN_2299612 | 0.115 | 0.0256 | 2.31 | -4.04108 | 0.373 |
| SIRT5 | ILMN_1799598 | 0.179 | 0.0481 | 2.03 | -4.58522 | 0.373 |
| UBE2B | ILMN_1663099 | 1.16E-05 | 1.32E-07 | 6.23 | 7.41964 | 0.372 |
| PITPNC1 | ILMN_1670638 | 0.00157 | 8.62E-05 | 4.31 | 1.21489 | 0.372 |
| IDS | ILMN_1798448 | 0.0172 | 0.00202 | 3.27 | -1.74722 | 0.372 |
| PFKFB2 | ILMN_2385298 | 0.119 | 0.0268 | 2.29 | -4.08145 | 0.372 |
| BEX2 | ILMN_2181892 | 0.134 | 0.0316 | 2.22 | -4.22501 | 0.372 |
| IGF2BP2 | ILMN_1702447 | 0.139 | 0.0332 | 2.2 | -4.26669 | 0.372 |
| PTGER4 | ILMN_1795930 | 0.00603 | 0.000509 | 3.74 | -0.46149 | 0.371 |
| HNRNPL | ILMN_2389582 | 4.74E-05 | 9.09E-07 | 5.67 | 5.56642 | 0.37 |
| NXT1 | ILMN_1760280 | 0.0325 | 0.00464 | 2.98 | -2.51055 | 0.37 |
| RWDD2A | ILMN_1829362 | 0.0392 | 0.00596 | 2.88 | -2.73857 | 0.37 |
| ZNF600 | ILMN_1678457 | 0.0561 | 0.00968 | 2.7 | -3.17752 | 0.37 |
| KLRD1 | ILMN_1799134 | 0.00844 | 0.000788 | 3.6 | -0.87037 | 0.369 |
| ZMYM5 | ILMN_2343036 | 0.0296 | 0.00411 | 3.02 | -2.39934 | 0.369 |
| GNE | ILMN_1729417 | 0.0593 | 0.0105 | 2.67 | -3.24925 | 0.369 |
| BEX1 | ILMN_2234697 | 0.105 | 0.0227 | 2.36 | -3.93375 | 0.369 |
| ZNF669 | ILMN_1770172 | 0.000539 | 2.08E-05 | 4.74 | 2.56565 | 0.368 |
| HIST1H2BE | ILMN_1687947 | 0.00784 | 0.000716 | 3.63 | -0.78129 | 0.368 |
| CLN8 | ILMN_1701094 | 0.0572 | 0.00997 | 2.69 | -3.20411 | 0.368 |
| GAB2 | ILMN_1815758 | 0.000656 | 2.72E-05 | 4.66 | 2.31104 | 0.367 |
| CHCHD7 | ILMN_1744138 | 0.0225 | 0.00287 | 3.15 | -2.07039 | 0.367 |
| JMJD1C | ILMN_1764970 | 0.0285 | 0.00392 | 3.04 | -2.35676 | 0.367 |
| RAB20 | ILMN_1708881 | 0.0656 | 0.0119 | 2.62 | -3.36389 | 0.367 |
| FAM172A | ILMN_1654542 | 0.0152 | 0.00173 | 3.33 | -1.60322 | 0.365 |
| RABGEF1 | ILMN_2230579 | 0.129 | 0.0303 | 2.24 | -4.18637 | 0.365 |
| RPS15A | ILMN_2255310 | 9.37E-09 | 6.17E-12 | 9.16 | 17.00549 | 0.364 |
| ATG2A | ILMN_1756006 | 0.0092 | 0.00088 | 3.56 | -0.97355 | 0.364 |
| MRPL18 | ILMN_2230672 | 0.00502 | 0.000401 | 3.82 | -0.23837 | 0.363 |
| MRM3 | ILMN_1689652 | 0.13 | 0.0305 | 2.23 | -4.19429 | 0.363 |
| IFNAR2 | ILMN_1765146 | 0.144 | 0.035 | 2.17 | -4.31167 | 0.363 |
| SCYL3 | ILMN_1743427 | 0.162 | 0.0417 | 2.1 | -4.46223 | 0.363 |
| 8-Mar | ILMN_2336335 | 0.00712 | 0.000628 | 3.67 | -0.65855 | 0.361 |
| AZIN1 | ILMN_2327994 | 0.0456 | 0.00729 | 2.81 | -2.92133 | 0.361 |
| ANAPC15 | ILMN_1726104 | 0.0825 | 0.0163 | 2.49 | -3.64211 | 0.361 |
| GCA | ILMN_1800602 | 0.138 | 0.033 | 2.2 | -4.26234 | 0.361 |
| RNU11 | ILMN_1901419 | 0.0563 | 0.00975 | 2.7 | -3.18334 | 0.36 |
| CD83 | ILMN_1780582 | 0.0565 | 0.00978 | 2.7 | -3.18684 | 0.36 |
| TYMP | ILMN_1690939 | 0.0569 | 0.00988 | 2.69 | -3.19583 | 0.36 |
| STAT3 | ILMN_2410986 | 0.0646 | 0.0117 | 2.63 | -3.34599 | 0.36 |
| MYL5 | ILMN_1746948 | 0.0876 | 0.0176 | 2.46 | -3.71233 | 0.36 |
| PPP2R2B | ILMN_2298365 | 0.00115 | 5.77E-05 | 4.43 | 1.59484 | 0.358 |
| PARP8 | ILMN_1806651 | 0.00192 | 0.000113 | 4.22 | 0.95567 | 0.357 |
| TMCO3 | ILMN_2220739 | 0.0169 | 0.00197 | 3.28 | -1.72185 | 0.357 |
| RPS6KA3 | ILMN_1770822 | 0.0293 | 0.00406 | 3.03 | -2.38864 | 0.357 |
| PLIN2 | ILMN_2138765 | 0.143 | 0.0348 | 2.18 | -4.30732 | 0.356 |
| HMGB2 | ILMN_2219712 | 0.0248 | 0.00329 | 3.1 | -2.19598 | 0.355 |
| THUMPD1 | ILMN_1702946 | 0.0266 | 0.00361 | 3.07 | -2.28024 | 0.355 |
| PYROXD1 | ILMN_3247826 | 0.0617 | 0.011 | 2.65 | -3.29357 | 0.355 |
| TNRC6C | ILMN_1873075 | 0.00222 | 0.000139 | 4.16 | 0.76303 | 0.354 |
| BLOC1S2 | ILMN_1679782 | 0.0313 | 0.00443 | 2.99 | -2.46775 | 0.354 |
| MIGA1 | ILMN_2123559 | 0.0439 | 0.00693 | 2.83 | -2.87516 | 0.354 |
| FCGR2A | ILMN_1706523 | 0.0639 | 0.0115 | 2.63 | -3.33203 | 0.354 |
| MVP | ILMN_2344373 | 0.0791 | 0.0154 | 2.52 | -3.59041 | 0.354 |
| FAM134B | ILMN_2283597 | 0.119 | 0.0269 | 2.29 | -4.08329 | 0.354 |
| ZNF410 | ILMN_1773247 | 0.0146 | 0.00163 | 3.35 | -1.54692 | 0.353 |
| YWHAZ | ILMN_1669286 | 0.0332 | 0.00478 | 2.97 | -2.53827 | 0.353 |
| LOC105377443 | ILMN_1876912 | 0.0737 | 0.014 | 2.55 | -3.51021 | 0.353 |
| KIT | ILMN_1790160 | 0.118 | 0.0265 | 2.29 | -4.07014 | 0.353 |
| IRAK4 | ILMN_1692352 | 0.0474 | 0.00767 | 2.79 | -2.96721 | 0.352 |
| SNORA10 | ILMN_2189842 | 0.0546 | 0.00935 | 2.71 | -3.14569 | 0.352 |
| ARL6IP6 | ILMN_1797964 | 0.0623 | 0.0112 | 2.64 | -3.30539 | 0.352 |
| GSAP | ILMN_3245773 | 0.173 | 0.0458 | 2.05 | -4.54344 | 0.352 |
| PRDX6 | ILMN_1803180 | 0.000817 | 3.65E-05 | 4.57 | 2.03189 | 0.351 |
| MTMR10 | ILMN_1778734 | 0.00374 | 0.000274 | 3.94 | 0.11976 | 0.351 |
| BCLAF1 | ILMN_2357272 | 0.00621 | 0.000528 | 3.73 | -0.49603 | 0.351 |
| ARID5B | ILMN_1721626 | 0.0291 | 0.00403 | 3.03 | -2.38165 | 0.351 |
| GKAP1 | ILMN_1736972 | 0.0426 | 0.00663 | 2.84 | -2.83509 | 0.351 |
| CTBP1 | ILMN_2278235 | 0.112 | 0.0247 | 2.32 | -4.00869 | 0.351 |
| IPO4 | ILMN_1798172 | 0.12 | 0.0274 | 2.28 | -4.0999 | 0.351 |
| IRF9 | ILMN_1745471 | 0.168 | 0.044 | 2.07 | -4.50849 | 0.351 |
| MAX | ILMN_1802457 | 1.09E-05 | 1.20E-07 | 6.26 | 7.51255 | 0.35 |
| RPL14 | ILMN_1726460 | 0.103 | 0.0221 | 2.37 | -3.91121 | 0.35 |
| CHST11 | ILMN_1655880 | 0.165 | 0.043 | 2.08 | -4.48867 | 0.35 |
| TEK | ILMN_1751576 | 0.177 | 0.0473 | 2.04 | -4.56983 | 0.35 |
| H2AFY | ILMN_2373495 | 0.00424 | 0.000323 | 3.89 | -0.0354 | 0.349 |
| H2AFJ | ILMN_1708728 | 0.00596 | 5.00E-04 | 3.75 | -0.44513 | 0.349 |
| RAB27A | ILMN_2329773 | 0.0158 | 0.00181 | 3.31 | -1.64313 | 0.349 |
| RHBDF2 | ILMN_1735792 | 0.158 | 0.0403 | 2.11 | -4.43428 | 0.349 |
| RIMKLB | ILMN_3245707 | 0.174 | 0.0462 | 2.05 | -4.55044 | 0.349 |
| ZCWPW1 | ILMN_1751963 | 0.00158 | 8.66E-05 | 4.31 | 1.20963 | 0.348 |
| LCOR | ILMN_2062381 | 0.0898 | 0.0182 | 2.45 | -3.74119 | 0.348 |
| MIIP | ILMN_1783843 | 0.14 | 0.0335 | 2.19 | -4.27549 | 0.348 |
| CD83 | ILMN_2328666 | 0.144 | 0.0351 | 2.17 | -4.3137 | 0.348 |
| CARD16 | ILMN_1724474 | 0.175 | 0.0464 | 2.05 | -4.55293 | 0.348 |
| MAP4K5 | ILMN_2408908 | 0.0586 | 0.0103 | 2.68 | -3.23095 | 0.347 |
| ACACB | ILMN_1763852 | 0.104 | 0.0225 | 2.36 | -3.92577 | 0.347 |
| RPS16 | ILMN_1651850 | 4.19E-06 | 3.11E-08 | 6.65 | 8.81361 | 0.346 |
| MAX | ILMN_1706546 | 0.00583 | 0.000487 | 3.75 | -0.42031 | 0.346 |
| CYBRD1 | ILMN_2087692 | 0.0661 | 0.0121 | 2.61 | -3.37601 | 0.346 |
| C17orf99 | ILMN_1911047 | 0.0804 | 0.0157 | 2.51 | -3.61004 | 0.346 |
| KREMEN1 | ILMN_1772697 | 0.182 | 0.0491 | 2.02 | -4.60232 | 0.346 |
| NFKBIA | ILMN_1773154 | 2.10E-09 | 6.91E-13 | 9.84 | 19.09559 | 0.345 |
| RPS6KA3 | ILMN_1806294 | 0.00761 | 0.000688 | 3.64 | -0.74333 | 0.345 |
| U2AF1L4 | ILMN_1779177 | 0.0095 | 0.000917 | 3.54 | -1.012 | 0.345 |
| H2AFV | ILMN_1706784 | 0.0124 | 0.00132 | 3.42 | -1.3504 | 0.345 |
| HIST1H2BC | ILMN_1680937 | 0.000713 | 3.07E-05 | 4.62 | 2.19581 | 0.344 |
| SELK | ILMN_2134110 | 0.00289 | 0.000195 | 4.05 | 0.43971 | 0.343 |
| WTAP | ILMN_2260725 | 0.00997 | 0.000984 | 3.52 | -1.07775 | 0.342 |
| TFDP1 | ILMN_2117987 | 0.03 | 0.00418 | 3.01 | -2.41565 | 0.342 |
| IFI27L2 | ILMN_1740319 | 0.0419 | 0.00649 | 2.85 | -2.81656 | 0.342 |
| NATD1 | ILMN_1661735 | 0.0566 | 0.00981 | 2.69 | -3.18934 | 0.341 |
| PXK | ILMN_1815063 | 0.0916 | 0.0187 | 2.44 | -3.76554 | 0.341 |
| REC8 | ILMN_2317751 | 0.171 | 0.045 | 2.06 | -4.52738 | 0.341 |
| RPL26L1 | ILMN_1776586 | 0.0147 | 0.00164 | 3.35 | -1.55436 | 0.34 |
| ATL1 | ILMN_1689251 | 0.0618 | 0.011 | 2.65 | -3.296 | 0.34 |
| AKR1C4 | ILMN_1687757 | 0.119 | 0.0271 | 2.28 | -4.09016 | 0.34 |
| SBDSP1 | ILMN_1765725 | 0.0144 | 0.0016 | 3.35 | -1.53267 | 0.339 |
| TLR4 | ILMN_1706217 | 0.0201 | 0.00247 | 3.2 | -1.93283 | 0.339 |
| NENF | ILMN_2142554 | 0.0372 | 0.00555 | 2.91 | -2.67399 | 0.339 |
| MRPL18 | ILMN_1804479 | 0.0283 | 0.00389 | 3.04 | -2.34815 | 0.338 |
| CTNNAL1 | ILMN_2136446 | 0.143 | 0.0346 | 2.18 | -4.30203 | 0.338 |
| EPB41L4A-AS1 | ILMN_3244583 | 0.00107 | 5.23E-05 | 4.46 | 1.68853 | 0.337 |
| STAT5B | ILMN_1684034 | 0.0334 | 0.00481 | 2.96 | -2.54305 | 0.337 |
| PRMT2 | ILMN_2259119 | 0.0659 | 0.012 | 2.61 | -3.37294 | 0.337 |
| TLR8 | ILMN_1682251 | 0.0751 | 0.0144 | 2.54 | -3.53153 | 0.337 |
| TNFAIP6 | ILMN_1785732 | 0.0874 | 0.0176 | 2.46 | -3.70965 | 0.337 |
| GLS | ILMN_2188722 | 0.0968 | 0.0202 | 2.41 | -3.83325 | 0.337 |
| TMSB10 | ILMN_1812392 | 0.0163 | 0.00189 | 3.3 | -1.68512 | 0.336 |
| HIST1H1A | ILMN_1692831 | 0.0402 | 0.00615 | 2.87 | -2.76656 | 0.336 |
| HIST2H2AC | ILMN_1768973 | 0.00815 | 0.000754 | 3.61 | -0.82906 | 0.335 |
| CMSS1 | ILMN_2215545 | 0.0187 | 0.00225 | 3.24 | -1.84561 | 0.335 |
| FAM72B | ILMN_1688294 | 0.0441 | 0.00696 | 2.83 | -2.87888 | 0.335 |
| NDUFB6 | ILMN_1763147 | 0.0454 | 0.00726 | 2.81 | -2.91744 | 0.335 |
| IL1RAP | ILMN_2357062 | 0.0483 | 0.00786 | 2.78 | -2.98888 | 0.335 |
| PTP4A1 | ILMN_2154654 | 0.0531 | 0.00901 | 2.73 | -3.11276 | 0.335 |
| ZFAS1 | ILMN_3233010 | 0.0899 | 0.0182 | 2.45 | -3.74267 | 0.335 |
| TSTD1 | ILMN_3197097 | 0.015 | 0.0017 | 3.33 | -1.58451 | 0.334 |
| TSC22D3 | ILMN_1748124 | 0.017 | 0.00198 | 3.28 | -1.72883 | 0.334 |
| JUNB | ILMN_2086077 | 0.00124 | 6.34E-05 | 4.4 | 1.50656 | 0.333 |
| ASPRV1 | ILMN_1762284 | 0.00196 | 0.000117 | 4.21 | 0.92882 | 0.333 |
| PTTG1 | ILMN_2042771 | 0.00429 | 0.000328 | 3.88 | -0.04978 | 0.333 |
| SLC9A8 | ILMN_1690625 | 0.129 | 0.0303 | 2.24 | -4.1865 | 0.333 |
| MYO5C | ILMN_1808789 | 0.166 | 0.0432 | 2.08 | -4.49249 | 0.333 |
| BCLAF1 | ILMN_1775192 | 0.00305 | 0.00021 | 4.03 | 0.3734 | 0.332 |
| SLED1 | ILMN_1673322 | 0.00709 | 0.000625 | 3.67 | -0.65454 | 0.332 |
| SLC5A10 | ILMN_2412849 | 0.0569 | 0.00989 | 2.69 | -3.19661 | 0.332 |
| MXI1 | ILMN_1670570 | 0.106 | 0.0228 | 2.36 | -3.94076 | 0.332 |
| AP1AR | ILMN_2207561 | 0.183 | 0.0495 | 2.02 | -4.60837 | 0.332 |
| SNORD31 | ILMN_2072391 | 0.184 | 0.0498 | 2.01 | -4.61427 | 0.332 |
| WDFY2 | ILMN_1880425 | 0.0804 | 0.0157 | 2.51 | -3.61136 | 0.331 |
| TSC22D3 | ILMN_2376403 | 0.0583 | 0.0102 | 2.68 | -3.22626 | 0.33 |
| LOC100130476 | ILMN_3257004 | 0.0766 | 0.0148 | 2.53 | -3.55578 | 0.33 |
| ANXA1 | ILMN_2184184 | 0.00305 | 0.00021 | 4.03 | 0.37181 | 0.329 |
| NSUN4 | ILMN_1716004 | 0.0867 | 0.0173 | 2.47 | -3.69745 | 0.329 |
| INTS12 | ILMN_1725169 | 4.30E-06 | 3.25E-08 | 6.63 | 8.77075 | 0.328 |
| SON | ILMN_1663090 | 0.000628 | 2.60E-05 | 4.68 | 2.35618 | 0.328 |
| SCAND1 | ILMN_2372011 | 0.000964 | 4.54E-05 | 4.51 | 1.82362 | 0.328 |
| JMJD6 | ILMN_2412024 | 0.00199 | 0.000119 | 4.21 | 0.90756 | 0.328 |
| ETNK1 | ILMN_2316778 | 0.00695 | 0.000608 | 3.68 | -0.62837 | 0.328 |
| KIT | ILMN_2229379 | 0.0706 | 0.0133 | 2.58 | -3.45933 | 0.328 |
| MATR3 | ILMN_1660179 | 0.0917 | 0.0187 | 2.44 | -3.76671 | 0.328 |
| ARMC12 | ILMN_1712616 | 0.129 | 0.0302 | 2.24 | -4.1852 | 0.328 |
| DCXR | ILMN_1681437 | 0.000448 | 1.66E-05 | 4.81 | 2.78481 | 0.327 |
| IL1RL1 | ILMN_1697444 | 0.0123 | 0.0013 | 3.43 | -1.3383 | 0.326 |
| RPL28 | ILMN_1673509 | 0.0509 | 0.00845 | 2.75 | -3.05432 | 0.326 |
| C5orf34 | ILMN_1662184 | 0.0566 | 0.00982 | 2.69 | -3.19034 | 0.326 |
| PFKP | ILMN_1805737 | 0.145 | 0.0353 | 2.17 | -4.31882 | 0.326 |
| ICA1 | ILMN_2365569 | 0.0904 | 0.0184 | 2.44 | -3.75161 | 0.325 |
| ZNF586 | ILMN_2274531 | 0.0143 | 0.00158 | 3.36 | -1.519 | 0.324 |
| CYB561 | ILMN_1679721 | 0.0229 | 0.00295 | 3.14 | -2.09475 | 0.324 |
| CASP10 | ILMN_2321720 | 0.0927 | 0.0191 | 2.43 | -3.78211 | 0.324 |
| UTY | ILMN_2264634 | 0.144 | 0.0349 | 2.17 | -4.31061 | 0.324 |
| SPTLC1 | ILMN_1799853 | 0.000971 | 4.60E-05 | 4.5 | 1.81161 | 0.323 |
| PPP1R35 | ILMN_1741475 | 0.0393 | 0.00598 | 2.88 | -2.74099 | 0.323 |
| PNRC1 | ILMN_1789955 | 0.0229 | 0.00295 | 3.14 | -2.09613 | 0.322 |
| TAF8 | ILMN_1697175 | 0.0433 | 0.00679 | 2.84 | -2.85651 | 0.322 |
| SIVA1 | ILMN_1696046 | 0.0778 | 0.015 | 2.53 | -3.57204 | 0.322 |
| LOC100420587 | ILMN_1878670 | 0.145 | 0.0354 | 2.17 | -4.32251 | 0.322 |
| ATP11B | ILMN_2124155 | 0.0794 | 0.0155 | 2.52 | -3.59607 | 0.321 |
| SERPINA1 | ILMN_2256050 | 0.0909 | 0.0186 | 2.44 | -3.75767 | 0.321 |
| SERINC3 | ILMN_1713752 | 0.158 | 0.0401 | 2.11 | -4.42858 | 0.321 |
| ZMPSTE24 | ILMN_1656413 | 0.07 | 0.0131 | 2.58 | -3.44825 | 0.32 |
| TCEA1 | ILMN_2357770 | 0.000174 | 4.88E-06 | 5.18 | 3.95423 | 0.319 |
| PDCD4 | ILMN_1768004 | 0.0107 | 0.00108 | 3.49 | -1.16563 | 0.319 |
| HIST1H4H | ILMN_1751120 | 0.0237 | 0.00307 | 3.13 | -2.13282 | 0.319 |
| IKZF5 | ILMN_3307796 | 0.0356 | 0.00524 | 2.93 | -2.62102 | 0.319 |
| EIF4E3 | ILMN_1796146 | 0.0527 | 0.00889 | 2.73 | -3.10039 | 0.319 |
| SCARNA9 | ILMN_1805064 | 0.00218 | 0.000135 | 4.17 | 0.78775 | 0.318 |
| JPH2 | ILMN_1802434 | 0.00252 | 0.000164 | 4.1 | 0.60417 | 0.318 |
| TBCB | ILMN_1790953 | 0.0298 | 0.00415 | 3.02 | -2.40874 | 0.318 |
| CHPT1 | ILMN_2202940 | 0.0908 | 0.0185 | 2.44 | -3.75714 | 0.318 |
| PITPNC1 | ILMN_1738796 | 0.138 | 0.033 | 2.2 | -4.26042 | 0.318 |
| HPRT1 | ILMN_2056975 | 0.15 | 0.0371 | 2.15 | -4.36344 | 0.318 |
| GRHPR | ILMN_1664798 | 0.0514 | 0.0086 | 2.75 | -3.07014 | 0.317 |
| LCOR | ILMN_1689817 | 0.0925 | 0.019 | 2.43 | -3.7782 | 0.317 |
| BORCS7 | ILMN_2151056 | 0.114 | 0.0252 | 2.31 | -4.02621 | 0.317 |
| MPZL1 | ILMN_1739103 | 0.163 | 0.0421 | 2.09 | -4.46981 | 0.317 |
| SPATA2L | ILMN_1691111 | 0.00765 | 0.000693 | 3.64 | -0.75015 | 0.316 |
| NUP50 | ILMN_1708132 | 0.0135 | 0.00147 | 3.38 | -1.45144 | 0.316 |
| SMCO4 | ILMN_1798270 | 0.129 | 0.0301 | 2.24 | -4.18157 | 0.316 |
| ZCCHC6 | ILMN_1779677 | 0.00505 | 0.000407 | 3.81 | -0.2507 | 0.315 |
| SNHG5 | ILMN_1653794 | 0.0105 | 0.00105 | 3.5 | -1.1425 | 0.315 |
| PHACTR4 | ILMN_1736548 | 0.046 | 0.00737 | 2.8 | -2.93149 | 0.315 |
| BORCS7 | ILMN_1772706 | 0.0874 | 0.0176 | 2.46 | -3.71035 | 0.315 |
| SEZ6 | ILMN_1658809 | 0.11 | 0.0242 | 2.33 | -3.99238 | 0.315 |
| CGRRF1 | ILMN_1681008 | 0.0166 | 0.00193 | 3.29 | -1.70307 | 0.314 |
| SLC5A8 | ILMN_1811221 | 0.0289 | 0.004 | 3.03 | -2.37395 | 0.314 |
| AAMDC | ILMN_1779163 | 0.0536 | 0.00912 | 2.72 | -3.12366 | 0.314 |
| RAB13 | ILMN_1788180 | 0.0675 | 0.0125 | 2.6 | -3.40416 | 0.314 |
| SLC5A9 | ILMN_1748366 | 0.0761 | 0.0146 | 2.54 | -3.54793 | 0.314 |
| SON | ILMN_2236800 | 0.000721 | 3.13E-05 | 4.62 | 2.17796 | 0.313 |
| ERGIC1 | ILMN_1664068 | 0.00529 | 0.000429 | 3.8 | -0.30097 | 0.313 |
| STK24 | ILMN_1695773 | 0.00798 | 0.000733 | 3.62 | -0.80314 | 0.313 |
| GTF2IRD2B | ILMN_1760011 | 0.0138 | 0.00151 | 3.38 | -1.47458 | 0.312 |
| KDM4C | ILMN_1661770 | 0.0345 | 0.00502 | 2.95 | -2.58285 | 0.312 |
| ERCC1 | ILMN_2277676 | 0.0426 | 0.00665 | 2.84 | -2.83793 | 0.312 |
| RICTOR | ILMN_1705828 | 0.0702 | 0.0132 | 2.58 | -3.45236 | 0.312 |
| TMEM126A | ILMN_2217809 | 0.137 | 0.0329 | 2.2 | -4.25751 | 0.312 |
| CEACAM6 | ILMN_1712522 | 0.154 | 0.0386 | 2.13 | -4.39732 | 0.312 |
| TSNAX | ILMN_1713668 | 0.00191 | 0.000112 | 4.23 | 0.96545 | 0.311 |
| FAM160B1 | ILMN_1752927 | 0.036 | 0.00531 | 2.93 | -2.63345 | 0.311 |
| LOC283332 | ILMN_3199608 | 0.056 | 0.00967 | 2.7 | -3.17599 | 0.311 |
| MAFF | ILMN_2322375 | 0.0596 | 0.0105 | 2.67 | -3.2535 | 0.311 |
| PSMB8 | ILMN_2390299 | 0.126 | 0.0292 | 2.25 | -4.15631 | 0.311 |
| MGARP | ILMN_1793025 | 0.143 | 0.0345 | 2.18 | -4.30099 | 0.311 |
| FPR2 | ILMN_2392569 | 0.171 | 0.0452 | 2.06 | -4.53052 | 0.311 |
| TMX4 | ILMN_1702759 | 0.027 | 0.00366 | 3.06 | -2.29404 | 0.31 |
| SNORD16 | ILMN_1669210 | 0.0478 | 0.00775 | 2.78 | -2.97707 | 0.31 |
| LRPAP1 | ILMN_1660341 | 0.0922 | 0.0189 | 2.43 | -3.77427 | 0.31 |
| MT1X | ILMN_1775170 | 0.115 | 0.0255 | 2.31 | -4.03833 | 0.31 |
| OLMALINC | ILMN_3268880 | 0.153 | 0.0382 | 2.13 | -4.38824 | 0.31 |
| ILF2 | ILMN_1745172 | 0.0335 | 0.00484 | 2.96 | -2.5481 | 0.309 |
| TUBA1A | ILMN_2148819 | 0.0548 | 0.00939 | 2.71 | -3.14948 | 0.309 |
| PYGL | ILMN_1696187 | 0.112 | 0.0246 | 2.32 | -4.00638 | 0.309 |
| CKS2 | ILMN_2072296 | 0.00277 | 0.000184 | 4.07 | 0.49422 | 0.308 |
| MPC2 | ILMN_1727813 | 0.00993 | 0.000978 | 3.52 | -1.07251 | 0.308 |
| PPP1R14B | ILMN_1659888 | 0.0365 | 0.00541 | 2.92 | -2.65112 | 0.308 |
| PGGHG | ILMN_1794707 | 0.00919 | 0.000878 | 3.56 | -0.97136 | 0.307 |
| WBP1 | ILMN_2093231 | 0.0148 | 0.00166 | 3.34 | -1.56617 | 0.307 |
| RRAGA | ILMN_1783709 | 0.000445 | 1.64E-05 | 4.81 | 2.79439 | 0.306 |
| ZNF207 | ILMN_1670895 | 0.000732 | 3.19E-05 | 4.61 | 2.16112 | 0.306 |
| HNRNPLL | ILMN_1791575 | 0.0743 | 0.0142 | 2.55 | -3.51824 | 0.306 |
| DPM3 | ILMN_2402674 | 0.112 | 0.0246 | 2.32 | -4.00691 | 0.306 |
| SERPINB1 | ILMN_1679133 | 0.129 | 0.0301 | 2.24 | -4.18197 | 0.306 |
| STAT3 | ILMN_1663618 | 0.0876 | 0.0176 | 2.46 | -3.71243 | 0.305 |
| MEFV | ILMN_1804738 | 0.124 | 0.0285 | 2.26 | -4.13287 | 0.304 |
| PSMA1 | ILMN_1760542 | 0.142 | 0.0343 | 2.18 | -4.29465 | 0.304 |
| SIVA1 | ILMN_1787248 | 0.166 | 0.0434 | 2.08 | -4.49645 | 0.304 |
| RGS18 | ILMN_2101278 | 0.177 | 0.0473 | 2.04 | -4.57068 | 0.304 |
| RPS18 | ILMN_1753534 | 1.60E-05 | 2.07E-07 | 6.1 | 6.99138 | 0.303 |
| LGALS8 | ILMN_2266214 | 0.0378 | 0.00568 | 2.9 | -2.6945 | 0.303 |
| LOC283788 | ILMN_3250345 | 0.0525 | 0.00885 | 2.73 | -3.09686 | 0.303 |
| DCTN6 | ILMN_1744059 | 0.113 | 0.025 | 2.32 | -4.01827 | 0.303 |
| ADAT3 | ILMN_1806275 | 0.126 | 0.0293 | 2.25 | -4.15814 | 0.303 |
| GYG1 | ILMN_2230862 | 0.144 | 0.035 | 2.17 | -4.31245 | 0.303 |
| ECM1 | ILMN_2329735 | 0.063 | 0.0113 | 2.64 | -3.31618 | 0.302 |
| CACNG6 | ILMN_1779043 | 0.114 | 0.0251 | 2.32 | -4.02372 | 0.302 |
| COL16A1 | ILMN_1684554 | 0.115 | 0.0257 | 2.31 | -4.04367 | 0.302 |
| COX7A2L | ILMN_3237665 | 0.00504 | 0.000403 | 3.82 | -0.24256 | 0.301 |
| THOC7 | ILMN_1805028 | 0.015 | 0.00169 | 3.34 | -1.58004 | 0.301 |
| PRPF18 | ILMN_1807243 | 0.0369 | 0.00548 | 2.91 | -2.66264 | 0.301 |
| MAPKAPK2 | ILMN_1774844 | 0.0464 | 0.00746 | 2.8 | -2.94207 | 0.301 |
| ARRB2 | ILMN_1675866 | 0.146 | 0.0358 | 2.16 | -4.33066 | 0.301 |
| FAM126B | ILMN_1779486 | 0.153 | 0.0385 | 2.13 | -4.39431 | 0.301 |
| SPC24 | ILMN_2181432 | 0.0044 | 0.000339 | 3.87 | -0.07968 | 0.3 |
| UGT1A3 | ILMN_2206420 | 0.039 | 0.00591 | 2.89 | -2.73159 | 0.3 |
| ST6GALNAC3 | ILMN_1779061 | 0.0673 | 0.0124 | 2.6 | -3.39989 | 0.3 |
| OR56B1 | ILMN_1724166 | 0.104 | 0.0223 | 2.37 | -3.91876 | 0.3 |
| PTGER2 | ILMN_1703926 | 0.18 | 0.0484 | 2.03 | -4.5893 | 0.3 |
| TOMM5 | ILMN_1808661 | 0.181 | 0.0487 | 2.03 | -4.59467 | 0.3 |
| TSEN34 | ILMN_2368292 | 0.0124 | 0.00132 | 3.42 | -1.34946 | 0.299 |
| HERPUD2 | ILMN_2066348 | 0.0622 | 0.0112 | 2.64 | -3.30449 | 0.299 |
| BBS2 | ILMN_2230035 | 0.108 | 0.0236 | 2.34 | -3.96807 | 0.299 |
| MCRIP1 | ILMN_1733799 | 0.164 | 0.0425 | 2.09 | -4.47871 | 0.299 |
| JAGN1 | ILMN_3238036 | 0.0123 | 0.00131 | 3.42 | -1.34352 | 0.298 |
| MAPK10 | ILMN_2256907 | 0.0164 | 0.00191 | 3.29 | -1.69211 | 0.298 |
| RPL9 | ILMN_2408415 | 0.0372 | 0.00556 | 2.91 | -2.67527 | 0.298 |
| RAB43 | ILMN_2155480 | 0.0453 | 0.00722 | 2.81 | -2.91209 | 0.298 |
| CSRNP1 | ILMN_1703123 | 0.00195 | 0.000116 | 4.22 | 0.93516 | 0.297 |
| OAT | ILMN_1654441 | 0.00614 | 0.00052 | 3.73 | -0.48175 | 0.297 |
| WWP2 | ILMN_1659703 | 0.00956 | 0.000927 | 3.54 | -1.02269 | 0.297 |
| CIPC | ILMN_2097858 | 0.112 | 0.0248 | 2.32 | -4.01161 | 0.297 |
| PSMA6 | ILMN_1704094 | 0.000894 | 4.12E-05 | 4.54 | 1.91712 | 0.296 |
| WDR45 | ILMN_2251279 | 0.00166 | 9.32E-05 | 4.28 | 1.14051 | 0.296 |
| HIST2H2AA3 | ILMN_2144426 | 0.0253 | 0.00337 | 3.09 | -2.21658 | 0.296 |
| PMF1 | ILMN_1756445 | 0.0788 | 0.0153 | 2.52 | -3.58726 | 0.296 |
| HCG27 | ILMN_1746436 | 0.0922 | 0.0189 | 2.43 | -3.77456 | 0.296 |
| CKAP2 | ILMN_1674411 | 0.131 | 0.0309 | 2.23 | -4.20373 | 0.296 |
| ASNS | ILMN_2398107 | 0.162 | 0.0418 | 2.09 | -4.46549 | 0.296 |
| CIPC | ILMN_1788347 | 0.0867 | 0.0174 | 2.47 | -3.69916 | 0.295 |
| LOC105370613 | ILMN_1817816 | 0.108 | 0.0236 | 2.34 | -3.96876 | 0.295 |
| ADORA1 | ILMN_1747227 | 0.114 | 0.0252 | 2.31 | -4.02526 | 0.295 |
| COPS8 | ILMN_2373779 | 0.118 | 0.0267 | 2.29 | -4.07584 | 0.295 |
| ZCCHC14 | ILMN_1743456 | 0.128 | 0.0297 | 2.24 | -4.17015 | 0.295 |
| HIST2H2AA3 | ILMN_1659047 | 0.0121 | 0.00128 | 3.43 | -1.32306 | 0.294 |
| DPYSL2 | ILMN_1672503 | 0.0637 | 0.0115 | 2.63 | -3.32902 | 0.294 |
| FRAT1 | ILMN_1781416 | 0.133 | 0.0313 | 2.22 | -4.21481 | 0.294 |
| COA1 | ILMN_2081335 | 0.0375 | 0.00562 | 2.91 | -2.68436 | 0.293 |
| BMP6 | ILMN_1747650 | 0.0677 | 0.0125 | 2.6 | -3.40741 | 0.293 |
| MTM1 | ILMN_1686985 | 0.0844 | 0.0168 | 2.48 | -3.66927 | 0.293 |
| WIPF1 | ILMN_2262957 | 0.0979 | 0.0205 | 2.4 | -3.84694 | 0.293 |
| FAM86B1 | ILMN_1658861 | 0.0996 | 0.021 | 2.39 | -3.86771 | 0.293 |
| ACER2 | ILMN_3235340 | 0.158 | 0.0401 | 2.11 | -4.4294 | 0.293 |
| PPP1R2 | ILMN_1683044 | 0.000163 | 4.45E-06 | 5.2 | 4.04184 | 0.292 |
| LAIR2 | ILMN_1807491 | 0.0396 | 0.00604 | 2.88 | -2.75082 | 0.292 |
| SBDSP1 | ILMN_2159384 | 0.124 | 0.0287 | 2.26 | -4.14043 | 0.292 |
| RIMKLB | ILMN_1681757 | 0.137 | 0.0326 | 2.2 | -4.25042 | 0.292 |
| GDE1 | ILMN_1728698 | 0.00969 | 0.000945 | 3.53 | -1.04019 | 0.291 |
| EGLN1 | ILMN_1749892 | 0.0217 | 0.00273 | 3.17 | -2.02465 | 0.291 |
| RABGGTB | ILMN_2163796 | 0.0531 | 0.00899 | 2.73 | -3.11054 | 0.291 |
| ZSCAN21 | ILMN_1789410 | 0.0611 | 0.0109 | 2.65 | -3.28288 | 0.291 |
| WTAP | ILMN_2356559 | 0.176 | 0.0467 | 2.04 | -4.55955 | 0.291 |
| MAP3K3 | ILMN_2296697 | 0.0119 | 0.00125 | 3.44 | -1.30111 | 0.29 |
| R3HDM2 | ILMN_1764091 | 0.0247 | 0.00328 | 3.1 | -2.19144 | 0.29 |
| H2AFX | ILMN_2200331 | 0.0891 | 0.0181 | 2.45 | -3.73338 | 0.29 |
| NDUFA7 | ILMN_1675239 | 0.0934 | 0.0193 | 2.43 | -3.79145 | 0.29 |
| LINC01102 | ILMN_3235567 | 0.1 | 0.0212 | 2.39 | -3.87361 | 0.29 |
| TUBA1C | ILMN_1742167 | 0.107 | 0.0232 | 2.35 | -3.95506 | 0.29 |
| CCNH | ILMN_1742250 | 0.0144 | 0.0016 | 3.35 | -1.53113 | 0.289 |
| PRKCZ | ILMN_2386982 | 0.127 | 0.0296 | 2.25 | -4.16586 | 0.289 |
| RBBP6 | ILMN_2309446 | 0.000127 | 3.20E-06 | 5.3 | 4.35707 | 0.288 |
| RNF149 | ILMN_2112524 | 0.0119 | 0.00124 | 3.44 | -1.29665 | 0.287 |
| ACOT9 | ILMN_1658995 | 0.113 | 0.0249 | 2.32 | -4.01722 | 0.287 |
| GET4 | ILMN_1795703 | 0.123 | 0.0283 | 2.26 | -4.1288 | 0.287 |
| SYPL1 | ILMN_1764087 | 0.000272 | 8.82E-06 | 5 | 3.3873 | 0.286 |
| ING2 | ILMN_2085722 | 0.0207 | 0.00257 | 3.19 | -1.96927 | 0.286 |
| MSRB2 | ILMN_1657977 | 0.0902 | 0.0184 | 2.45 | -3.74794 | 0.286 |
| RNF13 | ILMN_1719867 | 0.151 | 0.0377 | 2.14 | -4.37534 | 0.286 |
| RNF13 | ILMN_2339748 | 0.00552 | 0.000454 | 3.78 | -0.35372 | 0.285 |
| TMCO6 | ILMN_2180624 | 0.025 | 0.00331 | 3.1 | -2.20199 | 0.285 |
| INSIG1 | ILMN_1686989 | 0.0334 | 0.00482 | 2.96 | -2.54434 | 0.285 |
| ODF3B | ILMN_1738867 | 0.106 | 0.0228 | 2.36 | -3.93914 | 0.285 |
| SNORD34 | ILMN_2165762 | 0.133 | 0.0315 | 2.22 | -4.22126 | 0.285 |
| CHKB | ILMN_1659054 | 0.0102 | 0.00102 | 3.51 | -1.1081 | 0.284 |
| XKR8 | ILMN_1675556 | 0.16 | 0.0408 | 2.1 | -4.44484 | 0.284 |
| C17orf78 | ILMN_1806330 | 0.0703 | 0.0132 | 2.58 | -3.45441 | 0.283 |
| MAP1LC3A | ILMN_1772958 | 0.08 | 0.0156 | 2.51 | -3.60571 | 0.283 |
| BZW1 | ILMN_1704760 | 0.132 | 0.0312 | 2.22 | -4.21237 | 0.283 |
| RASSF6 | ILMN_2352245 | 0.133 | 0.0315 | 2.22 | -4.22013 | 0.283 |
| KLC1 | ILMN_1654653 | 0.135 | 0.032 | 2.21 | -4.23541 | 0.283 |
| GNG11 | ILMN_1782419 | 0.000673 | 2.83E-05 | 4.65 | 2.27361 | 0.282 |
| USP15 | ILMN_1665557 | 0.0017 | 9.69E-05 | 4.27 | 1.1033 | 0.282 |
| ANAPC1 | ILMN_1659553 | 0.0088 | 0.00083 | 3.58 | -0.91883 | 0.282 |
| RAP1A | ILMN_1809191 | 0.0302 | 0.00421 | 3.01 | -2.42225 | 0.282 |
| MSI2 | ILMN_1804448 | 0.0725 | 0.0137 | 2.56 | -3.48999 | 0.282 |
| C7orf49 | ILMN_1740903 | 0.0939 | 0.0194 | 2.42 | -3.79843 | 0.282 |
| SMCHD1 | ILMN_1808148 | 0.0164 | 0.00191 | 3.29 | -1.69227 | 0.281 |
| MCL1 | ILMN_1803988 | 0.00418 | 0.000317 | 3.89 | -0.01547 | 0.28 |
| CBWD3 | ILMN_3248676 | 0.126 | 0.0291 | 2.25 | -4.15286 | 0.28 |
| METTL26 | ILMN_2369826 | 0.14 | 0.0335 | 2.19 | -4.27538 | 0.28 |
| ADIPOR1 | ILMN_2096322 | 0.00172 | 9.79E-05 | 4.27 | 1.09381 | 0.279 |
| ENTPD6 | ILMN_2091792 | 0.0659 | 0.012 | 2.61 | -3.37335 | 0.279 |
| CSGALNACT2 | ILMN_1799208 | 0.153 | 0.0382 | 2.13 | -4.38853 | 0.279 |
| SKP1 | ILMN_1711766 | 0.000193 | 5.59E-06 | 5.14 | 3.82381 | 0.278 |
| TCTEX1D4 | ILMN_2204526 | 0.158 | 0.0402 | 2.11 | -4.43175 | 0.278 |
| KLRB1 | ILMN_2079655 | 0.000165 | 4.55E-06 | 5.2 | 4.02097 | 0.277 |
| CHRNE | ILMN_1703346 | 0.0871 | 0.0175 | 2.47 | -3.70513 | 0.276 |
| KDM2B | ILMN_1658232 | 0.0447 | 0.0071 | 2.82 | -2.89699 | 0.275 |
| RB1CC1 | ILMN_1736796 | 0.0184 | 0.0022 | 3.24 | -1.82367 | 0.274 |
| APOBEC3F | ILMN_1702706 | 0.0227 | 0.00292 | 3.14 | -2.08444 | 0.274 |
| PLD4 | ILMN_1695490 | 0.046 | 0.00738 | 2.8 | -2.93265 | 0.274 |
| UBE2D3 | ILMN_2241679 | 0.0646 | 0.0117 | 2.63 | -3.34449 | 0.274 |
| PARD6B | ILMN_1745154 | 0.103 | 0.0221 | 2.37 | -3.9106 | 0.274 |
| CLEC2B | ILMN_1784608 | 0.117 | 0.0264 | 2.29 | -4.06798 | 0.274 |
| TRA2B | ILMN_1742798 | 5.53E-05 | 1.11E-06 | 5.61 | 5.37179 | 0.273 |
| GNG5 | ILMN_1701854 | 0.00221 | 0.000138 | 4.16 | 0.76867 | 0.273 |
| SUMO1P1 | ILMN_1785615 | 0.00282 | 0.000189 | 4.06 | 0.46979 | 0.273 |
| LYRM1 | ILMN_1749244 | 0.00633 | 0.000541 | 3.72 | -0.51814 | 0.273 |
| ZFAND2A | ILMN_1694671 | 0.00983 | 0.000964 | 3.53 | -1.05904 | 0.273 |
| PTP4A1 | ILMN_1760575 | 0.0105 | 0.00105 | 3.5 | -1.14261 | 0.273 |
| NDUFB6 | ILMN_2369924 | 0.11 | 0.024 | 2.33 | -3.98543 | 0.273 |
| AGTPBP1 | ILMN_2223922 | 0.132 | 0.031 | 2.22 | -4.20847 | 0.273 |
| NSRP1 | ILMN_1702541 | 0.0172 | 0.00202 | 3.27 | -1.74393 | 0.272 |
| RNF213 | ILMN_1731203 | 0.116 | 0.0258 | 2.3 | -4.04848 | 0.272 |
| C17orf62 | ILMN_1750401 | 0.165 | 0.043 | 2.08 | -4.48837 | 0.272 |
| GMFG | ILMN_1711617 | 0.00072 | 3.11E-05 | 4.62 | 2.18279 | 0.271 |
| CLDN14 | ILMN_1661194 | 0.00117 | 5.92E-05 | 4.42 | 1.57183 | 0.271 |
| SNRPA1 | ILMN_1715179 | 0.0328 | 0.00471 | 2.97 | -2.52393 | 0.271 |
| RFK | ILMN_1690780 | 0.0799 | 0.0156 | 2.51 | -3.60361 | 0.271 |
| RNY3 | ILMN_3239225 | 0.0966 | 0.0202 | 2.41 | -3.83062 | 0.271 |
| TMEM101 | ILMN_1667716 | 0.132 | 0.0312 | 2.22 | -4.21351 | 0.271 |
| STX17 | ILMN_1750896 | 0.147 | 0.0359 | 2.16 | -4.33487 | 0.271 |
| MTMR10 | ILMN_1811064 | 0.159 | 0.0407 | 2.11 | -4.44245 | 0.271 |
| HNRNPC | ILMN_2334587 | 2.46E-05 | 3.80E-07 | 5.92 | 6.4059 | 0.27 |
| CYTIP | ILMN_1746864 | 0.014 | 0.00154 | 3.37 | -1.49755 | 0.27 |
| SLC35G5 | ILMN_1715988 | 0.0678 | 0.0126 | 2.6 | -3.41188 | 0.27 |
| PIGO | ILMN_2379931 | 0.0703 | 0.0132 | 2.58 | -3.453 | 0.27 |
| GP9 | ILMN_1743290 | 0.0967 | 0.0202 | 2.41 | -3.83255 | 0.27 |
| FIS1 | ILMN_1658351 | 0.124 | 0.0285 | 2.26 | -4.1353 | 0.27 |
| ZNF586 | ILMN_2372200 | 0.0139 | 0.00153 | 3.37 | -1.48889 | 0.269 |
| SIVA1 | ILMN_3300972 | 0.0853 | 0.017 | 2.48 | -3.68003 | 0.269 |
| CD74 | ILMN_1761464 | 0.0904 | 0.0184 | 2.44 | -3.75143 | 0.269 |
| SP110 | ILMN_1731418 | 0.0695 | 0.013 | 2.59 | -3.4393 | 0.268 |
| RNU4-1 | ILMN_3309453 | 0.172 | 0.0456 | 2.06 | -4.53804 | 0.268 |
| PDK3 | ILMN_1776582 | 0.0346 | 0.00504 | 2.95 | -2.58603 | 0.267 |
| HNRNPA1P33 | ILMN_2125675 | 0.0536 | 0.00913 | 2.72 | -3.12449 | 0.267 |
| NAGLU | ILMN_1694980 | 0.0654 | 0.0119 | 2.62 | -3.36044 | 0.267 |
| POLR3C | ILMN_1795678 | 0.136 | 0.0322 | 2.21 | -4.24065 | 0.267 |
| RNY1 | ILMN_3237623 | 0.00282 | 0.000189 | 4.06 | 0.46917 | 0.266 |
| SIRPB1 | ILMN_1733997 | 0.00492 | 0.000391 | 3.83 | -0.21509 | 0.266 |
| UNC45A | ILMN_1726434 | 0.0534 | 0.00908 | 2.72 | -3.11986 | 0.266 |
| TXNIP | ILMN_1697448 | 0.0732 | 0.0139 | 2.56 | -3.50201 | 0.266 |
| CLP1 | ILMN_1659976 | 0.0122 | 0.00129 | 3.43 | -1.32933 | 0.265 |
| ARIH1 | ILMN_1694219 | 0.0205 | 0.00253 | 3.19 | -1.95508 | 0.265 |
| HMGB1 | ILMN_2231242 | 0.0541 | 0.00923 | 2.72 | -3.13473 | 0.265 |
| SNORD36C | ILMN_2160160 | 0.0775 | 0.015 | 2.53 | -3.5676 | 0.265 |
| ZNFX1 | ILMN_1745148 | 0.0967 | 0.0202 | 2.41 | -3.83233 | 0.265 |
| 3-Mar | ILMN_1735038 | 0.0868 | 0.0174 | 2.47 | -3.70005 | 0.264 |
| C21orf33 | ILMN_1682812 | 0.109 | 0.0238 | 2.34 | -3.97855 | 0.264 |
| TWF1 | ILMN_1681203 | 0.142 | 0.0344 | 2.18 | -4.29775 | 0.264 |
| LOC103611081 | ILMN_1900235 | 0.174 | 0.0461 | 2.05 | -4.54854 | 0.264 |
| PPP6R2 | ILMN_1670523 | 0.0449 | 0.00714 | 2.82 | -2.90265 | 0.263 |
| DENND2C | ILMN_1815035 | 0.0729 | 0.0138 | 2.56 | -3.49763 | 0.263 |
| C6orf48 | ILMN_2391765 | 0.000753 | 3.29E-05 | 4.6 | 2.13113 | 0.262 |
| RNVU1-7 | ILMN_3244646 | 0.0012 | 6.10E-05 | 4.41 | 1.54217 | 0.262 |
| ATG4B | ILMN_2411915 | 0.0028 | 0.000187 | 4.06 | 0.48171 | 0.262 |
| S100A13 | ILMN_2407168 | 0.00647 | 0.000557 | 3.71 | -0.546 | 0.262 |
| GPC6 | ILMN_1805216 | 0.0136 | 0.00149 | 3.38 | -1.46309 | 0.262 |
| RAB1A | ILMN_2118864 | 0.0342 | 0.00498 | 2.95 | -2.57524 | 0.262 |
| SAC3D1 | ILMN_1776674 | 0.0369 | 0.0055 | 2.91 | -2.66525 | 0.262 |
| FRG1 | ILMN_1734696 | 0.00166 | 9.34E-05 | 4.28 | 1.13834 | 0.261 |
| UQCRHL | ILMN_1718136 | 0.0116 | 0.00121 | 3.45 | -1.26734 | 0.261 |
| XPA | ILMN_1787591 | 0.107 | 0.0232 | 2.35 | -3.95598 | 0.261 |
| S100P | ILMN_1801216 | 0.0095 | 0.000917 | 3.54 | -1.01255 | 0.26 |
| ZNF655 | ILMN_1769673 | 0.0212 | 0.00265 | 3.18 | -1.99789 | 0.26 |
| SLC19A2 | ILMN_2201668 | 0.0236 | 0.00306 | 3.13 | -2.12772 | 0.26 |
| TSEN54 | ILMN_1657509 | 0.0891 | 0.018 | 2.45 | -3.73306 | 0.26 |
| SWI5 | ILMN_1811555 | 0.0966 | 0.0202 | 2.41 | -3.83104 | 0.26 |
| PLCD1 | ILMN_1769394 | 0.15 | 0.0372 | 2.15 | -4.36488 | 0.26 |
| KLHDC2 | ILMN_1741204 | 0.162 | 0.0417 | 2.1 | -4.46211 | 0.26 |
| CYP20A1 | ILMN_2391333 | 0.166 | 0.0433 | 2.08 | -4.49544 | 0.26 |
| RPL17 | ILMN_1655422 | 0.00016 | 4.33E-06 | 5.21 | 4.06824 | 0.259 |
| ARRB2 | ILMN_2395711 | 0.00832 | 0.000772 | 3.6 | -0.85153 | 0.259 |
| UTS2 | ILMN_2236625 | 0.0145 | 0.00162 | 3.35 | -1.53921 | 0.259 |
| CNOT8 | ILMN_1736068 | 0.027 | 0.00367 | 3.06 | -2.29677 | 0.259 |
| VNN2 | ILMN_1758864 | 0.042 | 0.00652 | 2.85 | -2.82042 | 0.259 |
| SPATA31C1 | ILMN_3273268 | 0.0825 | 0.0163 | 2.49 | -3.64142 | 0.259 |
| HIST2H2AA4 | ILMN_3242900 | 0.0362 | 0.00535 | 2.92 | -2.64019 | 0.258 |
| TCP1 | ILMN_1776347 | 0.0419 | 0.0065 | 2.85 | -2.81717 | 0.258 |
| LONRF1 | ILMN_1705953 | 0.094 | 0.0195 | 2.42 | -3.80037 | 0.258 |
| STRA13 | ILMN_1769634 | 0.0963 | 0.0201 | 2.41 | -3.82699 | 0.258 |
| GSTTP2 | ILMN_2246548 | 0.117 | 0.0264 | 2.29 | -4.06905 | 0.258 |
| F8A3 | ILMN_1727984 | 0.119 | 0.0271 | 2.28 | -4.08992 | 0.258 |
| HSPA5 | ILMN_1773865 | 0.132 | 0.0311 | 2.22 | -4.20882 | 0.258 |
| ZSWIM4 | ILMN_1735231 | 0.15 | 0.0373 | 2.14 | -4.36808 | 0.258 |
| ERICH1 | ILMN_1731001 | 0.00107 | 5.20E-05 | 4.46 | 1.69434 | 0.257 |
| JADE1 | ILMN_1655194 | 0.00233 | 0.000148 | 4.14 | 0.69995 | 0.257 |
| TRADD | ILMN_1765851 | 0.0461 | 0.0074 | 2.8 | -2.93464 | 0.257 |
| RAB3IP | ILMN_1803197 | 0.0513 | 0.00855 | 2.75 | -3.06563 | 0.257 |
| DUSP22 | ILMN_1671809 | 0.0612 | 0.0109 | 2.65 | -3.28495 | 0.257 |
| PTS | ILMN_2162328 | 0.128 | 0.0298 | 2.24 | -4.1727 | 0.257 |
| CMSS1 | ILMN_1705753 | 0.165 | 0.0427 | 2.08 | -4.48308 | 0.257 |
| TRAPPC6A | ILMN_1775703 | 0.183 | 0.0495 | 2.02 | -4.60913 | 0.257 |
| PHYKPL | ILMN_1673529 | 0.0622 | 0.0111 | 2.64 | -3.30392 | 0.256 |
| LOC100129550 | ILMN_3236239 | 0.1 | 0.0212 | 2.39 | -3.87421 | 0.256 |
| HIRA | ILMN_1813664 | 0.181 | 0.0487 | 2.03 | -4.59481 | 0.256 |
| SAT1 | ILMN_1753342 | 8.85E-06 | 9.08E-08 | 6.34 | 7.78294 | 0.255 |
| NDFIP2 | ILMN_1677396 | 0.0422 | 0.00656 | 2.85 | -2.82584 | 0.255 |
| CLDND2 | ILMN_2077680 | 0.11 | 0.024 | 2.33 | -3.98436 | 0.255 |
| SLC25A36 | ILMN_1766528 | 0.13 | 0.0303 | 2.23 | -4.18819 | 0.255 |
| CAV2 | ILMN_2360730 | 0.173 | 0.0459 | 2.05 | -4.5439 | 0.255 |
| ITCH | ILMN_1752283 | 0.00222 | 0.000139 | 4.16 | 0.76252 | 0.254 |
| MMD | ILMN_1733937 | 0.0198 | 0.00243 | 3.21 | -1.91545 | 0.254 |
| ZMYM5 | ILMN_1781028 | 0.0334 | 0.00482 | 2.96 | -2.54538 | 0.254 |
| SLC35A2 | ILMN_1798885 | 0.0754 | 0.0145 | 2.54 | -3.53709 | 0.254 |
| SIRT6 | ILMN_1654246 | 0.084 | 0.0167 | 2.48 | -3.66324 | 0.254 |
| PRSS42 | ILMN_1757294 | 0.114 | 0.0253 | 2.31 | -4.03029 | 0.254 |
| PIP4K2A | ILMN_2152465 | 0.0336 | 0.00485 | 2.96 | -2.5512 | 0.253 |
| ADAM17 | ILMN_1908488 | 0.0473 | 0.00765 | 2.79 | -2.96443 | 0.253 |
| TP53INP2 | ILMN_1686906 | 0.069 | 0.0129 | 2.59 | -3.43132 | 0.253 |
| EIF4E3 | ILMN_2225144 | 0.081 | 0.0159 | 2.5 | -3.62025 | 0.253 |
| PTS | ILMN_1720322 | 0.0805 | 0.0157 | 2.51 | -3.61238 | 0.252 |
| RNVU1-18 | ILMN_3236653 | 0.00124 | 6.37E-05 | 4.4 | 1.50152 | 0.251 |
| OAT | ILMN_2068747 | 0.014 | 0.00153 | 3.37 | -1.4902 | 0.251 |
| DYNLT1 | ILMN_1678766 | 0.0654 | 0.0119 | 2.62 | -3.35894 | 0.251 |
| GPAT4 | ILMN_1740752 | 0.0843 | 0.0167 | 2.48 | -3.66686 | 0.251 |
| ANO10 | ILMN_1767111 | 0.15 | 0.0374 | 2.14 | -4.36866 | 0.251 |
| PRKCZ | ILMN_2253286 | 0.151 | 0.0378 | 2.14 | -4.37767 | 0.251 |
| TOR1AIP2 | ILMN_1758057 | 0.167 | 0.0435 | 2.08 | -4.49828 | 0.251 |
| NEK7 | ILMN_1869897 | 0.00888 | 0.000841 | 3.57 | -0.93146 | 0.25 |
| ARL6IP4 | ILMN_1791149 | 0.00978 | 0.000957 | 3.53 | -1.05214 | 0.25 |
| METTL23 | ILMN_2382724 | 0.0547 | 0.00935 | 2.71 | -3.14624 | 0.25 |
| CARD14 | ILMN_1671728 | 0.147 | 0.036 | 2.16 | -4.33595 | 0.25 |
| SRP19 | ILMN_2192032 | 0.00134 | 7.00E-05 | 4.37 | 1.41221 | 0.249 |
| CPEB2-AS1 | ILMN_3294322 | 0.00296 | 0.000201 | 4.04 | 0.4118 | 0.249 |
| RPS7 | ILMN_1750722 | 0.0827 | 0.0163 | 2.49 | -3.64422 | 0.249 |
| PTEN | ILMN_1880406 | 0.0996 | 0.021 | 2.39 | -3.86825 | 0.249 |
| RHOT1 | ILMN_2338480 | 0.0484 | 0.00788 | 2.78 | -2.99139 | 0.248 |
| NUDT2 | ILMN_2349444 | 0.0675 | 0.0125 | 2.6 | -3.40449 | 0.248 |
| DNAJC3 | ILMN_1659843 | 0.104 | 0.0222 | 2.37 | -3.91655 | 0.248 |
| STAT3 | ILMN_2401978 | 0.116 | 0.0261 | 2.3 | -4.05652 | 0.248 |
| PATE1 | ILMN_3235416 | 0.18 | 0.0484 | 2.03 | -4.58889 | 0.248 |
| CSDE1 | ILMN_1683538 | 0.00746 | 0.000668 | 3.65 | -0.71587 | 0.246 |
| LHFP | ILMN_1767448 | 0.0236 | 0.00307 | 3.13 | -2.13057 | 0.246 |
| MRPS24 | ILMN_1802553 | 0.029 | 0.00401 | 3.03 | -2.37667 | 0.246 |
| ZNF331 | ILMN_1711199 | 0.124 | 0.0284 | 2.26 | -4.13195 | 0.246 |
| OSBPL1A | ILMN_1773063 | 0.182 | 0.0492 | 2.02 | -4.60404 | 0.246 |
| PRR13 | ILMN_1795944 | 0.0389 | 0.00588 | 2.89 | -2.72588 | 0.245 |
| MGARP | ILMN_3236160 | 0.183 | 0.0496 | 2.02 | -4.61027 | 0.245 |
| ATRAID | ILMN_1774584 | 0.00838 | 0.00078 | 3.6 | -0.86162 | 0.244 |
| ITGB1BP1 | ILMN_1690099 | 0.0173 | 0.00204 | 3.27 | -1.75373 | 0.244 |
| ST6GALNAC2 | ILMN_1658706 | 0.113 | 0.0251 | 2.32 | -4.02281 | 0.244 |
| C17orf58 | ILMN_1700515 | 0.182 | 0.0492 | 2.02 | -4.60404 | 0.244 |
| RNLS | ILMN_1783873 | 0.0919 | 0.0188 | 2.44 | -3.76971 | 0.243 |
| MAP1LC3B | ILMN_1703244 | 0.0174 | 0.00204 | 3.27 | -1.75545 | 0.242 |
| LY96 | ILMN_1724533 | 0.12 | 0.0274 | 2.28 | -4.10058 | 0.242 |
| CHMP5 | ILMN_1732534 | 0.166 | 0.0434 | 2.08 | -4.49649 | 0.242 |
| SBDS | ILMN_1679045 | 0.184 | 0.0498 | 2.01 | -4.61445 | 0.242 |
| NSFL1C | ILMN_1657624 | 0.0453 | 0.00724 | 2.81 | -2.91479 | 0.241 |
| SLC7A5P2 | ILMN_1788665 | 0.0772 | 0.0149 | 2.53 | -3.56289 | 0.241 |
| DBI | ILMN_1755926 | 0.0404 | 0.00617 | 2.87 | -2.77047 | 0.24 |
| VCPKMT | ILMN_1781102 | 0.0073 | 0.00065 | 3.66 | -0.69033 | 0.239 |
| SCARNA16 | ILMN_3237446 | 0.121 | 0.0277 | 2.27 | -4.11071 | 0.239 |
| SLC9A5 | ILMN_1714023 | 0.147 | 0.0361 | 2.16 | -4.33808 | 0.239 |
| TFDP1 | ILMN_1661717 | 0.183 | 0.0496 | 2.02 | -4.61047 | 0.239 |
| LAPTM4A | ILMN_1745110 | 0.00596 | 5.00E-04 | 3.75 | -0.44523 | 0.238 |
| SYAP1 | ILMN_2089175 | 0.0189 | 0.00228 | 3.23 | -1.85879 | 0.238 |
| ASAH1 | ILMN_1655509 | 0.158 | 0.0401 | 2.11 | -4.42982 | 0.238 |
| EXOSC6 | ILMN_1670218 | 0.158 | 0.0402 | 2.11 | -4.43141 | 0.238 |
| MAT2B | ILMN_1680246 | 0.171 | 0.0449 | 2.06 | -4.52577 | 0.238 |
| EIF1AY | ILMN_1755537 | 0.0194 | 0.00235 | 3.22 | -1.88581 | 0.237 |
| BAG1 | ILMN_1733970 | 0.158 | 0.0404 | 2.11 | -4.43504 | 0.237 |
| ERICH1 | ILMN_2104696 | 0.000575 | 2.27E-05 | 4.72 | 2.48215 | 0.236 |
| ESD | ILMN_1720285 | 0.0065 | 0.00056 | 3.71 | -0.55141 | 0.236 |
| RPL7 | ILMN_1815292 | 0.0954 | 0.0198 | 2.41 | -3.81594 | 0.236 |
| EIF1AX | ILMN_1813240 | 0.101 | 0.0215 | 2.38 | -3.88561 | 0.236 |
| SNHG17 | ILMN_3235326 | 0.14 | 0.0338 | 2.19 | -4.28123 | 0.236 |
| GNRH1 | ILMN_1745991 | 0.148 | 0.0364 | 2.16 | -4.34667 | 0.236 |
| MOSPD3 | ILMN_1805345 | 0.155 | 0.0393 | 2.12 | -4.41086 | 0.235 |
| YWHAQ | ILMN_1674385 | 0.000785 | 3.48E-05 | 4.59 | 2.07604 | 0.234 |
| NTAN1 | ILMN_1815552 | 0.00212 | 0.00013 | 4.18 | 0.82216 | 0.234 |
| UQCRH | ILMN_2232936 | 0.00287 | 0.000195 | 4.05 | 0.44387 | 0.234 |
| AP3S1 | ILMN_2311761 | 0.0166 | 0.00193 | 3.29 | -1.70296 | 0.234 |
| RPS27L | ILMN_1712678 | 0.0175 | 0.00206 | 3.27 | -1.76561 | 0.234 |
| KLRD1 | ILMN_1797988 | 0.152 | 0.0379 | 2.14 | -4.37965 | 0.234 |
| GNG7 | ILMN_1728107 | 0.163 | 0.042 | 2.09 | -4.46855 | 0.234 |
| RNU1-3 | ILMN_3246273 | 0.00144 | 7.65E-05 | 4.34 | 1.32754 | 0.233 |
| TIMM17B | ILMN_1813260 | 0.0366 | 0.00543 | 2.92 | -2.65423 | 0.233 |
| PPP2R3A | ILMN_1656393 | 0.158 | 0.0402 | 2.11 | -4.43201 | 0.233 |
| TROVE2 | ILMN_2241775 | 0.0104 | 0.00105 | 3.5 | -1.13678 | 0.232 |
| ASS1 | ILMN_2395451 | 0.0116 | 0.00121 | 3.45 | -1.27224 | 0.232 |
| ZFPM1 | ILMN_1651438 | 0.0632 | 0.0114 | 2.64 | -3.32028 | 0.232 |
| PARTICL | ILMN_1849399 | 0.14 | 0.0338 | 2.19 | -4.28287 | 0.232 |
| RPL12 | ILMN_1653469 | 0.000216 | 6.51E-06 | 5.09 | 3.67782 | 0.231 |
| RBM4 | ILMN_1712455 | 0.0657 | 0.012 | 2.62 | -3.36773 | 0.231 |
| EMX2OS | ILMN_3241729 | 0.0677 | 0.0125 | 2.6 | -3.40875 | 0.231 |
| RTCA | ILMN_1808305 | 0.161 | 0.0414 | 2.1 | -4.45713 | 0.231 |
| VCPKMT | ILMN_2394193 | 0.011 | 0.00112 | 3.48 | -1.20253 | 0.23 |
| FOSL1 | ILMN_1771841 | 0.0807 | 0.0158 | 2.51 | -3.61637 | 0.23 |
| NAP1L5 | ILMN_1773307 | 0.0996 | 0.021 | 2.39 | -3.86867 | 0.23 |
| SP110 | ILMN_2415144 | 0.117 | 0.0263 | 2.3 | -4.0652 | 0.23 |
| ZNF579 | ILMN_1761722 | 0.137 | 0.0327 | 2.2 | -4.25457 | 0.23 |
| UBE2J2 | ILMN_2387990 | 0.141 | 0.034 | 2.19 | -4.28588 | 0.23 |
| CALCOCO2 | ILMN_1755504 | 0.00478 | 0.000376 | 3.84 | -0.17805 | 0.229 |
| PPM1K | ILMN_2070043 | 0.0539 | 0.0092 | 2.72 | -3.13132 | 0.229 |
| FAM83F | ILMN_1683231 | 0.056 | 0.00967 | 2.7 | -3.176 | 0.229 |
| SNTB2 | ILMN_1786766 | 0.117 | 0.0263 | 2.3 | -4.06397 | 0.229 |
| NUFIP2 | ILMN_1765829 | 0.0307 | 0.00432 | 3 | -2.44534 | 0.228 |
| LAT2 | ILMN_2249720 | 0.0327 | 0.00467 | 2.97 | -2.51726 | 0.228 |
| TRIM9 | ILMN_1719254 | 0.101 | 0.0213 | 2.38 | -3.87992 | 0.228 |
| HSP90B1 | ILMN_2096116 | 0.132 | 0.031 | 2.22 | -4.20854 | 0.228 |
| TSC22D3 | ILMN_1695382 | 0.148 | 0.0366 | 2.15 | -4.34966 | 0.228 |
| MSRB1 | ILMN_1719661 | 0.154 | 0.0386 | 2.13 | -4.39641 | 0.228 |
| RARA | ILMN_1791902 | 0.154 | 0.0389 | 2.13 | -4.40396 | 0.228 |
| PSMB3 | ILMN_1748651 | 0.174 | 0.046 | 2.05 | -4.54557 | 0.228 |
| EIF4EBP3 | ILMN_2197225 | 0.0524 | 0.00881 | 2.74 | -3.09239 | 0.227 |
| LINC01315 | ILMN_1865056 | 0.167 | 0.0437 | 2.07 | -4.50263 | 0.227 |
| DNAJB6 | ILMN_2402416 | 0.000878 | 4.01E-05 | 4.54 | 1.94212 | 0.226 |
| CBX3 | ILMN_1790625 | 0.00419 | 0.000318 | 3.89 | -0.01923 | 0.226 |
| SMIM19 | ILMN_1677385 | 0.0113 | 0.00117 | 3.46 | -1.2394 | 0.226 |
| RABGGTB | ILMN_1790354 | 0.0114 | 0.00118 | 3.46 | -1.24851 | 0.226 |
| DDX43 | ILMN_1813893 | 0.157 | 0.0398 | 2.12 | -4.42222 | 0.226 |
| CA12 | ILMN_1720998 | 0.158 | 0.0404 | 2.11 | -4.43618 | 0.226 |
| DCAF11 | ILMN_2389376 | 0.0152 | 0.00172 | 3.33 | -1.59673 | 0.225 |
| NOP14-AS1 | ILMN_3247844 | 0.101 | 0.0214 | 2.38 | -3.88183 | 0.225 |
| RPA3 | ILMN_1716895 | 0.0105 | 0.00106 | 3.5 | -1.14644 | 0.224 |
| MGAM | ILMN_1714643 | 0.152 | 0.0381 | 2.14 | -4.38534 | 0.224 |
| EEF1B2 | ILMN_2318725 | 3.45E-05 | 5.93E-07 | 5.79 | 5.97695 | 0.223 |
| TOMM7 | ILMN_2087060 | 8.95E-05 | 2.08E-06 | 5.43 | 4.77127 | 0.223 |
| THUMPD1 | ILMN_2108339 | 0.0166 | 0.00194 | 3.29 | -1.70684 | 0.223 |
| TLDC1 | ILMN_1817524 | 0.0711 | 0.0134 | 2.57 | -3.46891 | 0.223 |
| C17orf74 | ILMN_1810107 | 0.152 | 0.038 | 2.14 | -4.38277 | 0.223 |
| MAP2 | ILMN_2283915 | 0.154 | 0.0387 | 2.13 | -4.39883 | 0.223 |
| CKS2 | ILMN_1756326 | 0.0189 | 0.00228 | 3.23 | -1.85782 | 0.222 |
| SCAND1 | ILMN_1795317 | 0.0464 | 0.00746 | 2.8 | -2.94222 | 0.222 |
| PARVB | ILMN_1667592 | 0.15 | 0.0373 | 2.14 | -4.36767 | 0.222 |
| SPCS3 | ILMN_3307700 | 0.00614 | 0.00052 | 3.73 | -0.48226 | 0.221 |
| USPL1 | ILMN_1662686 | 0.13 | 0.0305 | 2.23 | -4.19292 | 0.221 |
| PDE6C | ILMN_1730851 | 0.137 | 0.0328 | 2.2 | -4.25584 | 0.221 |
| FTH1 | ILMN_1683146 | 0.00533 | 0.000433 | 3.79 | -0.31008 | 0.22 |
| HCN4 | ILMN_1790810 | 0.021 | 0.00262 | 3.18 | -1.98432 | 0.22 |
| CALR | ILMN_1736256 | 0.0409 | 0.00628 | 2.86 | -2.78569 | 0.22 |
| DPH3 | ILMN_1679912 | 0.0426 | 0.00664 | 2.84 | -2.83693 | 0.22 |
| INPP5F | ILMN_1813650 | 0.106 | 0.023 | 2.35 | -3.94717 | 0.22 |
| MPP1 | ILMN_1733675 | 0.122 | 0.0281 | 2.27 | -4.1213 | 0.22 |
| RPS4Y2 | ILMN_2191331 | 0.139 | 0.0332 | 2.2 | -4.26707 | 0.22 |
| SPINT3 | ILMN_3236045 | 0.158 | 0.0404 | 2.11 | -4.43447 | 0.22 |
| TOMM7 | ILMN_1674069 | 0.000319 | 1.08E-05 | 4.94 | 3.197 | 0.219 |
| SF3B6 | ILMN_2182120 | 0.0632 | 0.0113 | 2.64 | -3.31987 | 0.219 |
| TAF7 | ILMN_1759460 | 0.000608 | 2.49E-05 | 4.69 | 2.39705 | 0.218 |
| ADIPOQ | ILMN_1775045 | 0.0442 | 0.00699 | 2.82 | -2.88318 | 0.218 |
| TRAF6 | ILMN_1783910 | 0.0501 | 0.00825 | 2.76 | -3.03304 | 0.218 |
| TCP1 | ILMN_1660661 | 0.105 | 0.0227 | 2.36 | -3.93434 | 0.218 |
| ZCCHC7 | ILMN_1744980 | 0.151 | 0.0377 | 2.14 | -4.37615 | 0.218 |
| BIN3 | ILMN_1708485 | 0.161 | 0.0416 | 2.1 | -4.46013 | 0.218 |
| RNF11 | ILMN_1810785 | 0.00252 | 0.000164 | 4.11 | 0.60516 | 0.217 |
| CCDC90B | ILMN_2232166 | 0.0364 | 0.0054 | 2.92 | -2.64852 | 0.217 |
| DEGS1 | ILMN_1667430 | 0.102 | 0.0218 | 2.37 | -3.9012 | 0.217 |
| TMEM106B | ILMN_1726288 | 0.137 | 0.0328 | 2.2 | -4.25661 | 0.217 |
| RPL36 | ILMN_1685088 | 6.76E-05 | 1.45E-06 | 5.53 | 5.11547 | 0.216 |
| LAMTOR5 | ILMN_2220518 | 0.0129 | 0.00139 | 3.4 | -1.39804 | 0.216 |
| CAPNS1 | ILMN_2393254 | 0.0695 | 0.013 | 2.59 | -3.43982 | 0.216 |
| TEX33 | ILMN_1663417 | 0.0711 | 0.0134 | 2.57 | -3.46787 | 0.216 |
| PIK3R6 | ILMN_1783645 | 0.0924 | 0.019 | 2.43 | -3.77709 | 0.216 |
| CXCL16 | ILMN_1728478 | 0.00439 | 0.000339 | 3.87 | -0.07839 | 0.215 |
| ANXA11 | ILMN_2380494 | 0.00443 | 0.000342 | 3.87 | -0.08901 | 0.215 |
| POLR2J | ILMN_1657317 | 0.059 | 0.0104 | 2.67 | -3.24117 | 0.215 |
| ZP3 | ILMN_1805377 | 0.0876 | 0.0176 | 2.46 | -3.71283 | 0.215 |
| CDC42EP4 | ILMN_1745223 | 0.00287 | 0.000194 | 4.05 | 0.44557 | 0.214 |
| H3F3B | ILMN_1695706 | 0.00303 | 0.000208 | 4.03 | 0.37923 | 0.214 |
| RPS8 | ILMN_3243700 | 0.0036 | 0.000261 | 3.96 | 0.16481 | 0.214 |
| MMGT1 | ILMN_1776216 | 0.0123 | 0.0013 | 3.43 | -1.338 | 0.214 |
| HLA-DRB4 | ILMN_1752592 | 0.0893 | 0.0181 | 2.45 | -3.73536 | 0.214 |
| PFKFB4 | ILMN_1653292 | 0.14 | 0.0336 | 2.19 | -4.2767 | 0.214 |
| SLC30A5 | ILMN_1709728 | 0.153 | 0.0384 | 2.13 | -4.39161 | 0.214 |
| TMEM185A | ILMN_1738276 | 0.0269 | 0.00365 | 3.06 | -2.29086 | 0.213 |
| VPS28 | ILMN_1790797 | 0.032 | 0.00457 | 2.98 | -2.49699 | 0.213 |
| MSMO1 | ILMN_2402499 | 0.0493 | 0.00809 | 2.77 | -3.01491 | 0.213 |
| ELANE | ILMN_1706635 | 0.0833 | 0.0165 | 2.49 | -3.65426 | 0.213 |
| C11orf65 | ILMN_1788968 | 0.116 | 0.0259 | 2.3 | -4.05089 | 0.213 |
| SPATS2L | ILMN_1683678 | 0.137 | 0.0328 | 2.2 | -4.25588 | 0.213 |
| TMEM170A | ILMN_2212590 | 0.0453 | 0.00722 | 2.81 | -2.91287 | 0.212 |
| IFRD1 | ILMN_1667561 | 0.11 | 0.024 | 2.33 | -3.98356 | 0.212 |
| MRGPRD | ILMN_1714980 | 0.127 | 0.0296 | 2.25 | -4.16649 | 0.212 |
| ZDHHC3 | ILMN_2201347 | 0.149 | 0.0367 | 2.15 | -4.35355 | 0.212 |
| UQCRFS1 | ILMN_1701749 | 0.0171 | 0.00201 | 3.28 | -1.73973 | 0.211 |
| CNNM3 | ILMN_1662328 | 0.0837 | 0.0166 | 2.49 | -3.65889 | 0.211 |
| ZNF669 | ILMN_2093748 | 0.0977 | 0.0205 | 2.4 | -3.84447 | 0.21 |
| CLEC2D | ILMN_1711702 | 0.148 | 0.0364 | 2.16 | -4.34598 | 0.21 |
| PPBP | ILMN_1767281 | 0.00126 | 6.49E-05 | 4.4 | 1.48425 | 0.209 |
| ING3 | ILMN_2237746 | 0.00534 | 0.000435 | 3.79 | -0.31377 | 0.209 |
| NEO1 | ILMN_1696702 | 0.0116 | 0.00121 | 3.45 | -1.27092 | 0.209 |
| IKZF5 | ILMN_1674024 | 0.0174 | 0.00206 | 3.27 | -1.76244 | 0.208 |
| CTNNBL1 | ILMN_2063925 | 0.173 | 0.0459 | 2.05 | -4.54436 | 0.208 |
| RECQL | ILMN_2380999 | 0.00586 | 0.00049 | 3.75 | -0.42561 | 0.207 |
| MAP3K7CL | ILMN_1699071 | 0.0317 | 0.00452 | 2.99 | -2.48572 | 0.207 |
| PHIP | ILMN_1898682 | 0.11 | 0.0241 | 2.33 | -3.98852 | 0.206 |
| B4GALT1 | ILMN_1766221 | 0.115 | 0.0256 | 2.31 | -4.03975 | 0.206 |
| FAAP20 | ILMN_2097790 | 0.12 | 0.0274 | 2.28 | -4.10076 | 0.206 |
| CELF2 | ILMN_1800638 | 0.146 | 0.0358 | 2.16 | -4.33149 | 0.206 |
| FAM126A | ILMN_1691980 | 0.149 | 0.0367 | 2.15 | -4.35243 | 0.206 |
| MAT2B | ILMN_1811367 | 0.171 | 0.0451 | 2.06 | -4.5292 | 0.206 |
| MOB4 | ILMN_3306168 | 0.0655 | 0.0119 | 2.62 | -3.36157 | 0.205 |
| VDAC3 | ILMN_1729816 | 0.00887 | 0.000839 | 3.57 | -0.92967 | 0.204 |
| RPL12 | ILMN_2116366 | 0.000777 | 3.44E-05 | 4.59 | 2.08789 | 0.203 |
| PSPC1 | ILMN_1724490 | 0.00378 | 0.000279 | 3.94 | 0.10471 | 0.203 |
| CCDC59 | ILMN_1662318 | 0.0289 | 0.00399 | 3.03 | -2.37222 | 0.203 |
| AGTPBP1 | ILMN_1718071 | 0.0319 | 0.00455 | 2.98 | -2.49327 | 0.203 |
| NDUFAF7 | ILMN_2394132 | 0.101 | 0.0213 | 2.38 | -3.88045 | 0.203 |
| FXR1 | ILMN_1704335 | 0.149 | 0.037 | 2.15 | -4.36056 | 0.203 |
| YRDC | ILMN_1736008 | 0.154 | 0.0386 | 2.13 | -4.39594 | 0.203 |
| NDUFA12 | ILMN_1737738 | 0.00771 | 0.000701 | 3.63 | -0.76169 | 0.202 |
| HLA-F | ILMN_1762861 | 0.0301 | 0.0042 | 3.01 | -2.41912 | 0.202 |
| DGCR11 | ILMN_3236211 | 0.0491 | 0.00802 | 2.77 | -3.00741 | 0.202 |
| CYP20A1 | ILMN_1718177 | 0.0724 | 0.0137 | 2.56 | -3.48792 | 0.202 |
| IGF2R | ILMN_1807662 | 0.117 | 0.0264 | 2.29 | -4.06669 | 0.201 |
| EIF3I | ILMN_1671291 | 0.132 | 0.0311 | 2.22 | -4.21122 | 0.201 |
| EEF1B2 | ILMN_1694587 | 6.47E-06 | 6.12E-08 | 6.45 | 8.1633 | 0.2 |
| SEC22B | ILMN_1784238 | 0.131 | 0.0306 | 2.23 | -4.19684 | 0.2 |
| HINT2 | ILMN_1697820 | 0.179 | 0.0479 | 2.03 | -4.57999 | 0.2 |
| GABARAPL1 | ILMN_2151281 | 0.0573 | 0.01 | 2.69 | -3.20679 | 0.199 |
| ZNF773 | ILMN_1685365 | 0.14 | 0.0336 | 2.19 | -4.27799 | 0.199 |
| C1orf43 | ILMN_1660602 | 0.153 | 0.0383 | 2.13 | -4.38968 | 0.199 |
| EIF5 | ILMN_2318430 | 0.0447 | 0.00709 | 2.82 | -2.89651 | 0.198 |
| WDR45 | ILMN_1756146 | 0.0616 | 0.011 | 2.65 | -3.29225 | 0.198 |
| HLA-C | ILMN_1721113 | 0.00202 | 0.000122 | 4.2 | 0.88721 | 0.197 |
| SOD2 | ILMN_2336781 | 0.0659 | 0.012 | 2.62 | -3.3706 | 0.197 |
| INAFM1 | ILMN_1703316 | 0.0672 | 0.0124 | 2.6 | -3.39677 | 0.197 |
| POLE3 | ILMN_1785198 | 0.101 | 0.0213 | 2.38 | -3.88001 | 0.197 |
| RPL13A | ILMN_1713369 | 0.000469 | 1.77E-05 | 4.79 | 2.72239 | 0.196 |
| RPL13AP6 | ILMN_1789809 | 0.0115 | 0.0012 | 3.45 | -1.25976 | 0.196 |
| EZR | ILMN_3272378 | 0.067 | 0.0123 | 2.61 | -3.39305 | 0.196 |
| MIR1275 | ILMN_3309839 | 0.0748 | 0.0143 | 2.55 | -3.52747 | 0.196 |
| PGRMC1 | ILMN_1684771 | 0.136 | 0.0323 | 2.21 | -4.24308 | 0.196 |
| KIR3DL2 | ILMN_2190842 | 0.183 | 0.0494 | 2.02 | -4.60725 | 0.196 |
| RPSAP9 | ILMN_3208973 | 0.00236 | 0.000151 | 4.13 | 0.68368 | 0.195 |
| KRT75 | ILMN_1721247 | 0.0993 | 0.0209 | 2.39 | -3.86404 | 0.195 |
| MGARP | ILMN_2072101 | 0.109 | 0.0238 | 2.34 | -3.97746 | 0.195 |
| HSPA2 | ILMN_1766499 | 0.114 | 0.0253 | 2.31 | -4.02878 | 0.195 |
| HERPUD2 | ILMN_1785158 | 0.0394 | 0.00601 | 2.88 | -2.74567 | 0.194 |
| SPATA7 | ILMN_1652409 | 0.13 | 0.0305 | 2.23 | -4.19304 | 0.194 |
| TRIM66 | ILMN_3235584 | 0.0551 | 0.00945 | 2.71 | -3.15582 | 0.193 |
| RPS6 | ILMN_1808939 | 2.46E-05 | 3.77E-07 | 5.93 | 6.41362 | 0.192 |
| USP3 | ILMN_1725862 | 0.129 | 0.0302 | 2.24 | -4.1845 | 0.192 |
| FCHO2 | ILMN_1670322 | 0.0465 | 0.00748 | 2.8 | -2.94434 | 0.191 |
| HLA-DRA | ILMN_2157441 | 0.0531 | 0.00901 | 2.73 | -3.11232 | 0.191 |
| EMC6 | ILMN_2405592 | 0.0255 | 0.00341 | 3.09 | -2.22946 | 0.19 |
| RWDD1 | ILMN_2316806 | 0.14 | 0.0338 | 2.19 | -4.2826 | 0.19 |
| SECISBP2 | ILMN_1736481 | 0.056 | 0.00966 | 2.7 | -3.17579 | 0.189 |
| U2AF1 | ILMN_1772113 | 0.0028 | 0.000187 | 4.06 | 0.48363 | 0.188 |
| MAF1 | ILMN_1713985 | 0.0888 | 0.018 | 2.45 | -3.7296 | 0.188 |
| SNORD32A | ILMN_1743217 | 0.121 | 0.0275 | 2.28 | -4.10358 | 0.187 |
| HSPE1 | ILMN_1803775 | 0.123 | 0.0283 | 2.26 | -4.12774 | 0.187 |
| LOC101928532 | ILMN_1823582 | 0.0901 | 0.0183 | 2.45 | -3.74581 | 0.186 |
| LMAN1 | ILMN_1715814 | 0.116 | 0.0259 | 2.3 | -4.05193 | 0.186 |
| ENHO | ILMN_3247320 | 0.168 | 0.044 | 2.07 | -4.50906 | 0.186 |
| APMAP | ILMN_1674394 | 0.17 | 0.0447 | 2.06 | -4.52143 | 0.186 |
| EIF3J-AS1 | ILMN_1689749 | 0.111 | 0.0243 | 2.33 | -3.99591 | 0.185 |
| CTNNB1 | ILMN_1757350 | 0.135 | 0.0321 | 2.21 | -4.23662 | 0.185 |
| C5orf64 | ILMN_1674116 | 0.15 | 0.0374 | 2.14 | -4.36859 | 0.185 |
| TMEM234 | ILMN_1673752 | 0.155 | 0.0391 | 2.12 | -4.40749 | 0.185 |
| UBE2D3 | ILMN_2320853 | 0.00314 | 0.000218 | 4.01 | 0.33634 | 0.184 |
| PPP6R2 | ILMN_1655922 | 0.0511 | 0.0085 | 2.75 | -3.05962 | 0.184 |
| RPRD1A | ILMN_1764207 | 0.102 | 0.0218 | 2.38 | -3.89809 | 0.184 |
| TIPRL | ILMN_1781457 | 0.0112 | 0.00116 | 3.47 | -1.22812 | 0.183 |
| IRX1 | ILMN_1735353 | 0.124 | 0.0286 | 2.26 | -4.13774 | 0.183 |
| PRPF6 | ILMN_1659259 | 0.148 | 0.0365 | 2.15 | -4.34843 | 0.183 |
| FKBP2 | ILMN_1678080 | 0.166 | 0.0434 | 2.08 | -4.4967 | 0.183 |
| PHIP | ILMN_1788689 | 0.0179 | 0.00212 | 3.26 | -1.79133 | 0.182 |
| UBR5 | ILMN_1741253 | 0.13 | 0.0305 | 2.23 | -4.19194 | 0.182 |
| NKG7 | ILMN_1682993 | 0.177 | 0.047 | 2.04 | -4.56534 | 0.182 |
| PSMA6 | ILMN_2151818 | 0.00324 | 0.000228 | 4 | 0.29496 | 0.181 |
| AATK | ILMN_1699334 | 0.078 | 0.0151 | 2.52 | -3.57565 | 0.181 |
| ZNF24 | ILMN_1654357 | 0.099 | 0.0208 | 2.39 | -3.85956 | 0.181 |
| SLC25A37 | ILMN_1715969 | 0.112 | 0.0246 | 2.32 | -4.00422 | 0.181 |
| SLC45A4 | ILMN_2391976 | 0.0647 | 0.0117 | 2.63 | -3.34694 | 0.18 |
| HBB | ILMN_2100437 | 0.159 | 0.0405 | 2.11 | -4.43855 | 0.18 |
| DNPEP | ILMN_1691393 | 0.162 | 0.0419 | 2.09 | -4.4663 | 0.18 |
| RPS17 | ILMN_2207539 | 0.00193 | 0.000114 | 4.22 | 0.95177 | 0.179 |
| SCAND1 | ILMN_1794230 | 0.019 | 0.0023 | 3.23 | -1.86718 | 0.179 |
| BLOC1S6 | ILMN_2105033 | 0.126 | 0.0292 | 2.25 | -4.15541 | 0.179 |
| DRD4 | ILMN_2112915 | 0.164 | 0.0426 | 2.09 | -4.48084 | 0.179 |
| TRIM13 | ILMN_2262275 | 0.0766 | 0.0148 | 2.53 | -3.55569 | 0.178 |
| DEFA1B | ILMN_2102721 | 0.0971 | 0.0203 | 2.4 | -3.83718 | 0.178 |
| LLPH | ILMN_2122176 | 0.103 | 0.0221 | 2.37 | -3.91209 | 0.178 |
| STRAP | ILMN_1731194 | 0.0297 | 0.00414 | 3.02 | -2.40569 | 0.177 |
| SHROOM4 | ILMN_2206188 | 0.0825 | 0.0163 | 2.49 | -3.64078 | 0.177 |
| EZR | ILMN_1795937 | 0.114 | 0.0252 | 2.31 | -4.02517 | 0.176 |
| SNORA6 | ILMN_3245365 | 0.142 | 0.0343 | 2.18 | -4.29356 | 0.176 |
| SDPR | ILMN_1715991 | 0.0492 | 0.00806 | 2.77 | -3.01168 | 0.175 |
| EVI2A | ILMN_2369018 | 0.107 | 0.0232 | 2.35 | -3.95484 | 0.175 |
| CHMP2B | ILMN_1741176 | 0.156 | 0.0394 | 2.12 | -4.41391 | 0.175 |
| AZI2 | ILMN_1733680 | 0.161 | 0.0414 | 2.1 | -4.45716 | 0.175 |
| PMPCB | ILMN_1728660 | 0.178 | 0.0475 | 2.04 | -4.57349 | 0.175 |
| PSMB6 | ILMN_1666409 | 0.0962 | 0.02 | 2.41 | -3.8252 | 0.174 |
| RBM4 | ILMN_1709042 | 0.13 | 0.0305 | 2.23 | -4.19249 | 0.174 |
| FPR1 | ILMN_2092118 | 0.0142 | 0.00157 | 3.36 | -1.50962 | 0.173 |
| PAIP2 | ILMN_1782094 | 0.132 | 0.0312 | 2.22 | -4.21268 | 0.173 |
| TUBB1 | ILMN_1710280 | 0.0199 | 0.00244 | 3.21 | -1.91979 | 0.172 |
| SRSF5 | ILMN_2378868 | 0.0604 | 0.0107 | 2.66 | -3.2679 | 0.172 |
| ING2 | ILMN_1671265 | 0.11 | 0.0242 | 2.33 | -3.99131 | 0.172 |
| MAGEA2 | ILMN_1684607 | 0.123 | 0.0282 | 2.27 | -4.1258 | 0.172 |
| MED10 | ILMN_1707631 | 0.123 | 0.0284 | 2.26 | -4.13041 | 0.172 |
| DHX40 | ILMN_1653047 | 0.163 | 0.0421 | 2.09 | -4.47112 | 0.172 |
| RPS15A | ILMN_2337241 | 0.0105 | 0.00105 | 3.5 | -1.1387 | 0.171 |
| ADIPOR1 | ILMN_1688322 | 0.0646 | 0.0117 | 2.63 | -3.34421 | 0.171 |
| METRNL | ILMN_2342066 | 0.162 | 0.0418 | 2.09 | -4.46464 | 0.171 |
| ZNF174 | ILMN_1719202 | 0.163 | 0.042 | 2.09 | -4.46965 | 0.171 |
| RPS6 | ILMN_1656791 | 0.0035 | 0.000253 | 3.97 | 0.19767 | 0.17 |
| LCN2 | ILMN_1692223 | 0.0582 | 0.0102 | 2.68 | -3.22401 | 0.17 |
| NUCB2 | ILMN_1655913 | 0.0787 | 0.0153 | 2.52 | -3.58499 | 0.17 |
| RHOA | ILMN_1781290 | 0.0134 | 0.00146 | 3.39 | -1.44391 | 0.169 |
| TRA2A | ILMN_1731043 | 0.0276 | 0.00377 | 3.05 | -2.32099 | 0.169 |
| TRMT6 | ILMN_1765085 | 0.131 | 0.0307 | 2.23 | -4.19961 | 0.169 |
| SLC22A4 | ILMN_2050911 | 0.161 | 0.0413 | 2.1 | -4.45473 | 0.169 |
| TMEM120A | ILMN_1654516 | 0.164 | 0.0426 | 2.09 | -4.48141 | 0.169 |
| EIF5 | ILMN_1815733 | 0.0218 | 0.00275 | 3.16 | -2.03214 | 0.168 |
| RPS27A | ILMN_1755883 | 0.0442 | 0.00698 | 2.82 | -2.88238 | 0.168 |
| RNF41 | ILMN_1700345 | 0.132 | 0.0312 | 2.22 | -4.21389 | 0.168 |
| PDCD4 | ILMN_2396272 | 0.0523 | 0.00879 | 2.74 | -3.08995 | 0.167 |
| POMP | ILMN_1693287 | 0.0839 | 0.0166 | 2.49 | -3.66133 | 0.167 |
| C19orf52 | ILMN_1726181 | 0.155 | 0.0392 | 2.12 | -4.41016 | 0.167 |
| ZNF207 | ILMN_1778177 | 0.0583 | 0.0102 | 2.68 | -3.2262 | 0.166 |
| PRR15 | ILMN_2203768 | 0.103 | 0.0221 | 2.37 | -3.91138 | 0.166 |
| DPH3 | ILMN_2349610 | 0.126 | 0.0292 | 2.25 | -4.15655 | 0.166 |
| RPL21P28 | ILMN_3283775 | 0.00199 | 0.00012 | 4.2 | 0.90266 | 0.165 |
| RPL10A | ILMN_1808041 | 0.00729 | 0.000647 | 3.66 | -0.68702 | 0.165 |
| MAFF | ILMN_1700413 | 0.0921 | 0.0189 | 2.43 | -3.77248 | 0.165 |
| PQLC1 | ILMN_1798620 | 0.0943 | 0.0195 | 2.42 | -3.80348 | 0.165 |
| ARMC5 | ILMN_1697088 | 0.143 | 0.0347 | 2.18 | -4.30525 | 0.165 |
| OPRM1 | ILMN_1803261 | 0.158 | 0.0404 | 2.11 | -4.43579 | 0.165 |
| RPS4Y1 | ILMN_1783142 | 0.00398 | 0.000297 | 3.92 | 0.04487 | 0.164 |
| EVI2A | ILMN_1733579 | 0.0564 | 0.00976 | 2.7 | -3.1846 | 0.164 |
| PTPN12 | ILMN_1695509 | 0.165 | 0.0427 | 2.08 | -4.4838 | 0.164 |
| GNS | ILMN_1744517 | 0.115 | 0.0256 | 2.31 | -4.03885 | 0.163 |
| NUFIP2 | ILMN_2102693 | 0.129 | 0.0301 | 2.24 | -4.18087 | 0.163 |
| UBL3 | ILMN_1810729 | 0.00437 | 0.000336 | 3.88 | -0.0715 | 0.162 |
| ISCU | ILMN_1735432 | 0.0111 | 0.00114 | 3.47 | -1.21454 | 0.162 |
| MMADHC | ILMN_3248966 | 0.0177 | 0.00209 | 3.26 | -1.77628 | 0.162 |
| RPSA | ILMN_2411723 | 0.0664 | 0.0122 | 2.61 | -3.3839 | 0.162 |
| TSC22D1 | ILMN_2412380 | 0.184 | 0.0498 | 2.02 | -4.61324 | 0.161 |
| RPLP0 | ILMN_1709880 | 0.019 | 0.0023 | 3.23 | -1.86714 | 0.16 |
| MSRA | ILMN_2228180 | 0.0458 | 0.00734 | 2.81 | -2.92779 | 0.16 |
| OSCP1 | ILMN_2273572 | 0.0705 | 0.0132 | 2.58 | -3.45855 | 0.16 |
| NAMPT | ILMN_1653871 | 0.0708 | 0.0133 | 2.57 | -3.46316 | 0.16 |
| RPS26 | ILMN_2209027 | 0.0761 | 0.0146 | 2.54 | -3.54727 | 0.16 |
| HACL1 | ILMN_1723414 | 0.174 | 0.0461 | 2.05 | -4.54847 | 0.16 |
| MIR302C | ILMN_3308265 | 0.101 | 0.0214 | 2.38 | -3.88449 | 0.159 |
| HSPE1 | ILMN_2092536 | 0.156 | 0.0394 | 2.12 | -4.41492 | 0.159 |
| ADAM17 | ILMN_2121068 | 0.167 | 0.0436 | 2.08 | -4.50004 | 0.159 |
| APOM | ILMN_1731941 | 0.127 | 0.0297 | 2.24 | -4.16937 | 0.158 |
| MMGT1 | ILMN_3237986 | 0.147 | 0.0361 | 2.16 | -4.33773 | 0.158 |
| RPS13 | ILMN_1777344 | 0.00415 | 0.000313 | 3.9 | -0.00435 | 0.157 |
| RPS27 | ILMN_1696839 | 0.0427 | 0.00666 | 2.84 | -2.84003 | 0.157 |
| RBM18 | ILMN_1802355 | 0.0691 | 0.0129 | 2.59 | -3.43239 | 0.157 |
| CHP1 | ILMN_1779401 | 0.0419 | 0.00648 | 2.85 | -2.81462 | 0.156 |
| FGL1 | ILMN_1803597 | 0.16 | 0.0408 | 2.1 | -4.44451 | 0.156 |
| RPL32 | ILMN_1663799 | 0.00885 | 0.000836 | 3.58 | -0.9261 | 0.155 |
| AQP9 | ILMN_1715068 | 0.0826 | 0.0163 | 2.49 | -3.64244 | 0.155 |
| GNA13 | ILMN_1758906 | 0.0874 | 0.0176 | 2.46 | -3.70942 | 0.155 |
| GLUL | ILMN_1765208 | 0.132 | 0.0312 | 2.22 | -4.21162 | 0.155 |
| MMADHC | ILMN_2090558 | 0.00723 | 0.00064 | 3.66 | -0.67637 | 0.153 |
| RPL19P12 | ILMN_3194217 | 0.024 | 0.00315 | 3.12 | -2.15528 | 0.153 |
| EMC6 | ILMN_1758674 | 0.0251 | 0.00333 | 3.1 | -2.20637 | 0.153 |
| ZNF586 | ILMN_1666727 | 0.171 | 0.0448 | 2.06 | -4.52408 | 0.153 |
| RPS20 | ILMN_1701596 | 0.00113 | 5.61E-05 | 4.44 | 1.62194 | 0.152 |
| RPS14 | ILMN_2338785 | 0.0117 | 0.00123 | 3.45 | -1.28217 | 0.152 |
| TCEB1 | ILMN_2066020 | 0.042 | 0.00652 | 2.85 | -2.82017 | 0.152 |
| ARL4A | ILMN_1704842 | 0.162 | 0.0417 | 2.09 | -4.46346 | 0.152 |
| LSM3 | ILMN_2229242 | 0.126 | 0.0291 | 2.25 | -4.15309 | 0.151 |
| COX16 | ILMN_1706305 | 0.16 | 0.041 | 2.1 | -4.44893 | 0.151 |
| YBX1 | ILMN_2124769 | 0.18 | 0.0483 | 2.03 | -4.58811 | 0.151 |
| MMADHC | ILMN_1810759 | 0.00914 | 0.000872 | 3.56 | -0.96481 | 0.149 |
| CRBN | ILMN_1668582 | 0.081 | 0.0159 | 2.5 | -3.61956 | 0.149 |
| PSMC3 | ILMN_1809010 | 0.152 | 0.0381 | 2.14 | -4.38459 | 0.149 |
| ENY2 | ILMN_2166865 | 0.014 | 0.00154 | 3.37 | -1.4961 | 0.148 |
| UVSSA | ILMN_1810225 | 0.179 | 0.048 | 2.03 | -4.5824 | 0.148 |
| RPL39 | ILMN_1737015 | 0.0017 | 9.65E-05 | 4.27 | 1.10713 | 0.147 |
| LRAT | ILMN_1673491 | 0.115 | 0.0256 | 2.31 | -4.04002 | 0.147 |
| IFI6 | ILMN_1687384 | 0.139 | 0.0333 | 2.19 | -4.26933 | 0.147 |
| PIGM | ILMN_1799860 | 0.177 | 0.0471 | 2.04 | -4.56734 | 0.147 |
| BCL10 | ILMN_1716446 | 0.177 | 0.0473 | 2.04 | -4.57007 | 0.147 |
| RPS5 | ILMN_1707810 | 0.0259 | 0.00348 | 3.08 | -2.24728 | 0.146 |
| TTC32 | ILMN_2083593 | 0.149 | 0.0367 | 2.15 | -4.35204 | 0.146 |
| C18orf21 | ILMN_1805998 | 0.154 | 0.0387 | 2.13 | -4.39812 | 0.146 |
| RPL10A | ILMN_2154566 | 0.0181 | 0.00215 | 3.25 | -1.8039 | 0.145 |
| SRSF6 | ILMN_1697469 | 0.165 | 0.043 | 2.08 | -4.48851 | 0.145 |
| PIP4K2A | ILMN_3236637 | 0.0507 | 0.00839 | 2.75 | -3.04811 | 0.144 |
| PPM1A | ILMN_1903159 | 0.154 | 0.0388 | 2.13 | -4.40033 | 0.144 |
| GSPT1 | ILMN_1750130 | 0.0325 | 0.00464 | 2.98 | -2.51044 | 0.143 |
| SSR4 | ILMN_1680403 | 0.0609 | 0.0108 | 2.66 | -3.27773 | 0.143 |
| H3F3C | ILMN_1769705 | 0.148 | 0.0366 | 2.15 | -4.3505 | 0.143 |
| MRPL33 | ILMN_1726417 | 0.148 | 0.0365 | 2.15 | -4.34885 | 0.142 |
| MBOAT1 | ILMN_2278550 | 0.166 | 0.0434 | 2.08 | -4.49667 | 0.142 |
| CSNK1A1 | ILMN_1785988 | 0.117 | 0.0263 | 2.3 | -4.06568 | 0.139 |
| TAAR9 | ILMN_1762024 | 0.132 | 0.0311 | 2.22 | -4.20911 | 0.139 |
| SLC11A1 | ILMN_1741165 | 0.15 | 0.0371 | 2.15 | -4.36265 | 0.139 |
| VNN2 | ILMN_1678939 | 0.155 | 0.039 | 2.12 | -4.40579 | 0.139 |
| SRSF5 | ILMN_1761996 | 0.125 | 0.0288 | 2.26 | -4.14396 | 0.138 |
| LRP10 | ILMN_1670272 | 0.0668 | 0.0123 | 2.61 | -3.39062 | 0.137 |
| CRCP | ILMN_2381537 | 0.0704 | 0.0132 | 2.58 | -3.45666 | 0.137 |
| GAB2 | ILMN_1665964 | 0.0963 | 0.0201 | 2.41 | -3.82765 | 0.137 |
| SNORD100 | ILMN_1673181 | 0.166 | 0.0433 | 2.08 | -4.49395 | 0.137 |
| RPS3A | ILMN_2139943 | 0.00755 | 0.00068 | 3.64 | -0.73286 | 0.136 |
| EIF3K | ILMN_1694057 | 0.0541 | 0.00923 | 2.72 | -3.13414 | 0.136 |
| CTBS | ILMN_2144574 | 0.0718 | 0.0136 | 2.57 | -3.47895 | 0.136 |
| HSPD1 | ILMN_1766713 | 0.0958 | 0.0199 | 2.41 | -3.82149 | 0.136 |
| RPL41 | ILMN_2331890 | 0.011 | 0.00113 | 3.47 | -1.20685 | 0.135 |
| RPS27A | ILMN_2048326 | 0.0284 | 0.00391 | 3.04 | -2.35438 | 0.135 |
| LGALS14 | ILMN_1698318 | 0.159 | 0.0407 | 2.11 | -4.44276 | 0.135 |
| RPL9 | ILMN_1750507 | 0.0106 | 0.00107 | 3.49 | -1.16046 | 0.134 |
| TLK2 | ILMN_1663486 | 0.0794 | 0.0155 | 2.52 | -3.5954 | 0.134 |
| BASP1 | ILMN_1651826 | 0.119 | 0.027 | 2.28 | -4.08657 | 0.134 |
| DEFA1B | ILMN_1679357 | 0.152 | 0.038 | 2.14 | -4.38371 | 0.134 |
| SARAF | ILMN_1780141 | 0.172 | 0.0453 | 2.06 | -4.53383 | 0.133 |
| RPL38 | ILMN_1765043 | 0.0037 | 0.000271 | 3.95 | 0.13285 | 0.132 |
| RPS15 | ILMN_2219134 | 0.0968 | 0.0202 | 2.41 | -3.8338 | 0.132 |
| GINM1 | ILMN_2220403 | 0.137 | 0.0326 | 2.2 | -4.25034 | 0.132 |
| GNPNAT1 | ILMN_1686235 | 0.165 | 0.043 | 2.08 | -4.48791 | 0.132 |
| RPLP1 | ILMN_2386530 | 0.0259 | 0.00348 | 3.08 | -2.24756 | 0.131 |
| RPL11 | ILMN_2114876 | 0.0327 | 0.00468 | 2.97 | -2.5191 | 0.131 |
| RPL7A | ILMN_1740749 | 0.0591 | 0.0104 | 2.67 | -3.24495 | 0.131 |
| RPS4X | ILMN_1810577 | 0.0287 | 0.00396 | 3.03 | -2.36543 | 0.13 |
| CHIC2 | ILMN_1679428 | 0.0562 | 0.0097 | 2.7 | -3.17875 | 0.13 |
| COX17 | ILMN_2187718 | 0.133 | 0.0314 | 2.22 | -4.21843 | 0.127 |
| GTF2H5 | ILMN_1739497 | 0.0448 | 0.00712 | 2.82 | -2.89936 | 0.126 |
| RPL31 | ILMN_1754195 | 0.0187 | 0.00224 | 3.24 | -1.84007 | 0.125 |
| RPS4X | ILMN_2166831 | 0.0663 | 0.0122 | 2.61 | -3.3823 | 0.125 |
| MLLT11 | ILMN_1759097 | 0.151 | 0.0377 | 2.14 | -4.37638 | 0.125 |
| ACTR10 | ILMN_1798254 | 0.127 | 0.0294 | 2.25 | -4.16161 | 0.124 |
| PDHA2 | ILMN_2068951 | 0.172 | 0.0455 | 2.06 | -4.53745 | 0.124 |
| RPS3A | ILMN_1673638 | 0.039 | 0.00592 | 2.89 | -2.73312 | 0.122 |
| PAN3 | ILMN_1681304 | 0.0428 | 0.00669 | 2.84 | -2.8439 | 0.121 |
| CAMLG | ILMN_1714599 | 0.0672 | 0.0124 | 2.6 | -3.39634 | 0.121 |
| PSMG4 | ILMN_3224204 | 0.0745 | 0.0142 | 2.55 | -3.52185 | 0.12 |
| SERINC1 | ILMN_1741613 | 0.0571 | 0.00996 | 2.69 | -3.20244 | 0.119 |
| KDM6A | ILMN_1654488 | 0.0908 | 0.0185 | 2.44 | -3.75706 | 0.119 |
| PCMTD1 | ILMN_1737426 | 0.158 | 0.0404 | 2.11 | -4.43538 | 0.119 |
| FAM187B | ILMN_3236452 | 0.184 | 0.0499 | 2.01 | -4.61497 | 0.119 |
| ARF1 | ILMN_2330948 | 0.0528 | 0.00891 | 2.73 | -3.10276 | 0.118 |
| PSMD6 | ILMN_1779633 | 0.0917 | 0.0188 | 2.44 | -3.76721 | 0.117 |
| MRPL10 | ILMN_1695472 | 0.178 | 0.0477 | 2.03 | -4.57675 | 0.117 |
| UBE2D3 | ILMN_2292963 | 0.148 | 0.0363 | 2.16 | -4.34455 | 0.116 |
| RPS3A | ILMN_1657722 | 0.0512 | 0.00853 | 2.75 | -3.06306 | 0.115 |
| RPL24 | ILMN_2160388 | 0.071 | 0.0134 | 2.57 | -3.46729 | 0.115 |
| RPL27 | ILMN_1656807 | 0.0956 | 0.0199 | 2.41 | -3.81908 | 0.115 |
| RPS29 | ILMN_1738243 | 0.0673 | 0.0124 | 2.6 | -3.40012 | 0.114 |
| PIP4K2A | ILMN_1911042 | 0.14 | 0.0338 | 2.19 | -4.2822 | 0.114 |
| CTBS | ILMN_2144573 | 0.0681 | 0.0126 | 2.6 | -3.41712 | 0.113 |
| UBE2D3 | ILMN_2320850 | 0.0439 | 0.00691 | 2.83 | -2.87238 | 0.112 |
| CGGBP1 | ILMN_2387090 | 0.0427 | 0.00666 | 2.84 | -2.83993 | 0.11 |
| SLMAP | ILMN_1783120 | 0.115 | 0.0256 | 2.31 | -4.04066 | 0.11 |
| PFDN5 | ILMN_2356284 | 0.155 | 0.0393 | 2.12 | -4.41112 | 0.11 |
| RPLP1 | ILMN_2247594 | 0.101 | 0.0216 | 2.38 | -3.89105 | 0.109 |
| CWF19L1 | ILMN_1651886 | 0.11 | 0.0241 | 2.33 | -3.98933 | 0.108 |
| UBQLN1 | ILMN_1688622 | 0.143 | 0.0347 | 2.18 | -4.3054 | 0.108 |
| RDM1 | ILMN_1786361 | 0.168 | 0.0438 | 2.07 | -4.50538 | 0.108 |
| SNRPB2 | ILMN_1690706 | 0.0385 | 0.0058 | 2.89 | -2.71435 | 0.106 |
| EIF3H | ILMN_1683660 | 0.063 | 0.0113 | 2.64 | -3.31654 | 0.106 |
| RPL11 | ILMN_1672446 | 0.0928 | 0.0191 | 2.43 | -3.78363 | 0.106 |
| RPL13AP20 | ILMN_1660376 | 0.113 | 0.0249 | 2.32 | -4.01755 | 0.105 |
| TCEB2 | ILMN_1733927 | 0.143 | 0.0345 | 2.18 | -4.29958 | 0.103 |
| IDS | ILMN_1815445 | 0.16 | 0.0409 | 2.1 | -4.44521 | 0.102 |
| RPL18A | ILMN_2141452 | 0.0868 | 0.0174 | 2.47 | -3.70057 | 0.101 |
| RPL13AP5 | ILMN_3297317 | 0.109 | 0.0238 | 2.34 | -3.97756 | 0.0989 |
| RPL19 | ILMN_1701832 | 0.0215 | 0.00271 | 3.17 | -2.01743 | 0.0987 |
| RPL35 | ILMN_2142815 | 0.148 | 0.0364 | 2.15 | -4.34694 | 0.0983 |
| ANXA7 | ILMN_1703791 | 0.161 | 0.0412 | 2.1 | -4.45257 | 0.0932 |
| RPS29 | ILMN_1694742 | 0.143 | 0.0346 | 2.18 | -4.30262 | 0.0927 |
| RPL21 | ILMN_2290808 | 0.0944 | 0.0196 | 2.42 | -3.80471 | 0.0908 |
| RPL38 | ILMN_2343775 | 0.0524 | 0.00881 | 2.74 | -3.09285 | 0.088 |
| RPL30 | ILMN_1754303 | 0.0982 | 0.0206 | 2.4 | -3.85096 | 0.0878 |
| RPS27 | ILMN_1660498 | 0.0578 | 0.0101 | 2.68 | -3.21507 | 0.0877 |
| COX7C | ILMN_1798189 | 0.155 | 0.0392 | 2.12 | -4.41 | 0.0872 |
| RPS12 | ILMN_1782621 | 0.0975 | 0.0204 | 2.4 | -3.84182 | 0.0866 |
| SLC35C2 | ILMN_1754235 | 0.128 | 0.0297 | 2.24 | -4.17094 | 0.085 |
| EEF1A1 | ILMN_1343291 | 0.0887 | 0.0179 | 2.46 | -3.72734 | 0.0841 |
| IFITM2 | ILMN_1673352 | 0.029 | 0.00401 | 3.03 | -2.37588 | 0.0839 |
| RPL27A | ILMN_1713086 | 0.157 | 0.0399 | 2.11 | -4.42535 | 0.068 |
