# Supplementary material for: Comprehensive Analysis of Common Different Gene Expression Signatures in the Neutrophils of Sepsis
Source: Biomed Res Int. 2021 Apr 17;2021:6655425. doi: 10.1155/2021/6655425 (PMC8077712; doi:10.1155/2021/6655425)
Supplement: Supplementary 5 [file 6655425.f5.docx]

Table S5. Detailed information of DEGs in GSE49757

| Gene symbol | probe ID | adj.P.Val | P.Value | t Value | B value | logFC |
| --- | --- | --- | --- | --- | --- | --- |
| RPL6 | ILMN_1712155 | 0.13 | 0.0405 | -2.1 | -4.36574 | -0.0766 |
| SF3B4 | ILMN_1722648 | 0.145 | 0.0476 | -2.03 | -4.50221 | -0.0795 |
| RPS29 | ILMN_1738243 | 0.11 | 0.0315 | -2.21 | -4.15207 | -0.0799 |
| RPS13 | ILMN_1777344 | 0.112 | 0.0326 | -2.19 | -4.18036 | -0.0823 |
| RAB3GAP1 | ILMN_1739876 | 0.139 | 0.0445 | -2.06 | -4.4457 | -0.0825 |
| ARPC5 | ILMN_1768394 | 0.116 | 0.0344 | -2.17 | -4.22753 | -0.0829 |
| ORM2 | ILMN_1731785 | 0.0916 | 0.0242 | -2.32 | -3.92369 | -0.0849 |
| TYROBP | ILMN_1778977 | 0.118 | 0.0353 | -2.16 | -4.24801 | -0.0866 |
| UBXN1 | ILMN_1812769 | 0.146 | 0.0479 | -2.02 | -4.50809 | -0.0872 |
| RPS17 | ILMN_2207533 | 0.0866 | 0.0223 | -2.35 | -3.85441 | -0.0873 |
| RPL6 | ILMN_1690494 | 0.104 | 0.0289 | -2.24 | -4.07863 | -0.089 |
| RPS11 | ILMN_1740587 | 0.0878 | 0.0229 | -2.34 | -3.87577 | -0.0892 |
| IDS | ILMN_1815445 | 0.143 | 0.0464 | -2.04 | -4.48189 | -0.0894 |
| RPL41 | ILMN_2331890 | 0.057 | 0.0122 | -2.59 | -3.32343 | -0.0906 |
| PRR14 | ILMN_1745329 | 0.131 | 0.041 | -2.09 | -4.37737 | -0.0911 |
| MRPS22 | ILMN_1655377 | 0.129 | 0.04 | -2.1 | -4.35618 | -0.0915 |
| RPS27A | ILMN_2048326 | 0.0782 | 0.0192 | -2.41 | -3.72325 | -0.0917 |
| COPB2 | ILMN_1684385 | 0.138 | 0.0444 | -2.06 | -4.44364 | -0.0923 |
| MTPN | ILMN_2180682 | 0.117 | 0.0349 | -2.16 | -4.23822 | -0.0926 |
| RPS25 | ILMN_1746516 | 0.0848 | 0.0217 | -2.36 | -3.82856 | -0.0942 |
| CHCHD2 | ILMN_2191681 | 0.147 | 0.0483 | -2.02 | -4.51555 | -0.0956 |
| SARNP | ILMN_1680967 | 0.111 | 0.032 | -2.2 | -4.16525 | -0.0959 |
| PSMD6 | ILMN_2155998 | 0.111 | 0.0323 | -2.2 | -4.17233 | -0.096 |
| FBXO11 | ILMN_2285112 | 0.108 | 0.0309 | -2.22 | -4.13425 | -0.0961 |
| GNLY | ILMN_2256295 | 0.114 | 0.0334 | -2.18 | -4.2005 | -0.0967 |
| SLC2A3 | ILMN_1775708 | 0.109 | 0.0312 | -2.21 | -4.14242 | -0.0969 |
| OST4 | ILMN_3247452 | 0.0456 | 0.00877 | -2.72 | -3.02927 | -0.0971 |
| RAC1 | ILMN_2359789 | 0.103 | 0.0287 | -2.25 | -4.07143 | -0.0976 |
| RPS8 | ILMN_3243700 | 0.147 | 0.0483 | -2.02 | -4.51562 | -0.0976 |
| ATP5L | ILMN_1812638 | 0.119 | 0.0357 | -2.15 | -4.25832 | -0.098 |
| ENY2 | ILMN_2166865 | 0.134 | 0.0426 | -2.08 | -4.40928 | -0.0987 |
| CYB5B | ILMN_1684321 | 0.138 | 0.0442 | -2.06 | -4.44087 | -0.099 |
| PCNP | ILMN_1807994 | 0.0607 | 0.0133 | -2.56 | -3.40229 | -0.0992 |
| TOMM7 | ILMN_1674069 | 0.109 | 0.0312 | -2.21 | -4.14251 | -0.1 |
| RPL17 | ILMN_1658283 | 0.0592 | 0.0129 | -2.57 | -3.37222 | -0.101 |
| NSA2 | ILMN_2103295 | 0.0748 | 0.0181 | -2.44 | -3.66911 | -0.101 |
| PAFAH1B1 | ILMN_1722276 | 0.143 | 0.0467 | -2.03 | -4.48629 | -0.101 |
| BTF3 | ILMN_1659762 | 0.0549 | 0.0116 | -2.61 | -3.2777 | -0.102 |
| RALY | ILMN_2403994 | 0.146 | 0.0478 | -2.02 | -4.50642 | -0.102 |
| CMTM6 | ILMN_1696494 | 0.0795 | 0.0197 | -2.4 | -3.74503 | -0.103 |
| SF3B1 | ILMN_1706075 | 0.0797 | 0.0198 | -2.4 | -3.75035 | -0.103 |
| SUPT4H1 | ILMN_1766245 | 0.119 | 0.0356 | -2.15 | -4.2571 | -0.104 |
| SRSF2 | ILMN_1696407 | 0.119 | 0.0356 | -2.15 | -4.25741 | -0.104 |
| SNX3 | ILMN_1740180 | 0.0751 | 0.0182 | -2.43 | -3.67542 | -0.105 |
| COX5B | ILMN_1663512 | 0.0815 | 0.0205 | -2.39 | -3.77784 | -0.105 |
| NDUFA1 | ILMN_1784286 | 0.0869 | 0.0224 | -2.35 | -3.85807 | -0.105 |
| SRSF5 | ILMN_1761996 | 0.0998 | 0.0275 | -2.26 | -4.03363 | -0.105 |
| RPS15A | ILMN_1787949 | 0.118 | 0.0351 | -2.16 | -4.2446 | -0.105 |
| CREBBP | ILMN_1809583 | 0.122 | 0.037 | -2.14 | -4.28853 | -0.105 |
| PPP1R2 | ILMN_1683044 | 0.0891 | 0.0233 | -2.33 | -3.89285 | -0.106 |
| SUSD6 | ILMN_2226917 | 0.118 | 0.0352 | -2.16 | -4.24729 | -0.106 |
| CNOT4 | ILMN_1772677 | 0.121 | 0.0366 | -2.14 | -4.28063 | -0.106 |
| COX7A2 | ILMN_1701293 | 0.119 | 0.0358 | -2.15 | -4.26194 | -0.107 |
| MED6 | ILMN_1654543 | 0.144 | 0.047 | -2.03 | -4.4924 | -0.107 |
| SDHB | ILMN_1667257 | 0.0557 | 0.0118 | -2.6 | -3.2965 | -0.108 |
| RPL11 | ILMN_1672446 | 0.0713 | 0.0168 | -2.46 | -3.60737 | -0.108 |
| IK | ILMN_1699362 | 0.145 | 0.0474 | -2.03 | -4.49988 | -0.108 |
| GNB1 | ILMN_1760320 | 0.0796 | 0.0197 | -2.4 | -3.74675 | -0.109 |
| DHX15 | ILMN_1754839 | 0.127 | 0.0393 | -2.11 | -4.341 | -0.109 |
| MMADHC | ILMN_1810759 | 0.0147 | 0.00177 | -3.28 | -1.58094 | -0.11 |
| FXR1 | ILMN_1679640 | 0.0674 | 0.0155 | -2.5 | -3.53706 | -0.11 |
| TMEM170A | ILMN_2212590 | 0.134 | 0.0423 | -2.08 | -4.40294 | -0.11 |
| PSMB4 | ILMN_1737862 | 0.139 | 0.0446 | -2.05 | -4.44846 | -0.11 |
| RPL13AP20 | ILMN_1660376 | 0.0374 | 0.00667 | -2.82 | -2.78449 | -0.111 |
| TMCO1 | ILMN_1793829 | 0.1 | 0.0275 | -2.26 | -4.03605 | -0.111 |
| ATG12 | ILMN_2188204 | 0.147 | 0.0483 | -2.02 | -4.51429 | -0.111 |
| SAR1A | ILMN_1657697 | 0.0861 | 0.0222 | -2.35 | -3.84788 | -0.112 |
| MMADHC | ILMN_2090558 | 0.0171 | 0.00221 | -3.21 | -1.78104 | -0.113 |
| CXCL8 | ILMN_1666733 | 0.101 | 0.028 | -2.26 | -4.0487 | -0.113 |
| FAM32A | ILMN_3237241 | 0.126 | 0.0388 | -2.12 | -4.32883 | -0.113 |
| PRPF4 | ILMN_1697440 | 0.134 | 0.0423 | -2.08 | -4.4029 | -0.113 |
| RPL18 | ILMN_2230624 | 0.0157 | 0.00195 | -3.25 | -1.66719 | -0.114 |
| UBE2Z | ILMN_1692168 | 0.113 | 0.0332 | -2.18 | -4.19722 | -0.114 |
| NDUFA3 | ILMN_1784641 | 0.065 | 0.0148 | -2.52 | -3.49451 | -0.115 |
| FOXO4 | ILMN_1712095 | 0.119 | 0.0357 | -2.15 | -4.25976 | -0.116 |
| YY1AP1 | ILMN_1752303 | 0.0411 | 0.00761 | -2.77 | -2.90228 | -0.117 |
| GRB2 | ILMN_1742521 | 0.0931 | 0.0247 | -2.31 | -3.94348 | -0.117 |
| ATP5B | ILMN_1772132 | 0.0489 | 0.0098 | -2.68 | -3.12819 | -0.118 |
| AFTPH | ILMN_2342841 | 0.113 | 0.0329 | -2.19 | -4.18951 | -0.118 |
| MYH9 | ILMN_2087702 | 0.118 | 0.0352 | -2.16 | -4.24622 | -0.118 |
| EIF3L | ILMN_1762725 | 0.122 | 0.0369 | -2.14 | -4.28673 | -0.118 |
| TSPYL1 | ILMN_1779014 | 0.0398 | 0.00724 | -2.79 | -2.85732 | -0.119 |
| SNHG5 | ILMN_2200659 | 0.0456 | 0.00876 | -2.72 | -3.02887 | -0.119 |
| RPS3A | ILMN_1657722 | 0.0754 | 0.0183 | -2.43 | -3.67962 | -0.119 |
| POLR2J3 | ILMN_1677138 | 0.106 | 0.0297 | -2.23 | -4.10224 | -0.119 |
| RAN | ILMN_3307930 | 0.143 | 0.0464 | -2.04 | -4.48151 | -0.119 |
| RPL35A | ILMN_1756360 | 0.0309 | 0.00506 | -2.92 | -2.53519 | -0.12 |
| EIF3L | ILMN_3240740 | 0.0417 | 0.00775 | -2.76 | -2.9192 | -0.12 |
| RPS19 | ILMN_1784717 | 0.0482 | 0.00958 | -2.68 | -3.10829 | -0.12 |
| BRD2 | ILMN_1758918 | 0.0592 | 0.0129 | -2.57 | -3.37313 | -0.12 |
| EMC6 | ILMN_1758674 | 0.0668 | 0.0154 | -2.5 | -3.5274 | -0.12 |
| CGGBP1 | ILMN_1752631 | 0.0678 | 0.0157 | -2.49 | -3.54545 | -0.12 |
| CCNG1 | ILMN_1694491 | 0.074 | 0.0178 | -2.44 | -3.65593 | -0.12 |
| TFIP11 | ILMN_2408102 | 0.134 | 0.0424 | -2.08 | -4.40522 | -0.12 |
| ATP5J | ILMN_1772929 | 0.0553 | 0.0117 | -2.61 | -3.28828 | -0.121 |
| RPS3A | ILMN_1673638 | 0.0973 | 0.0265 | -2.28 | -4.00202 | -0.121 |
| ITM2B | ILMN_1751708 | 0.0988 | 0.0271 | -2.27 | -4.02143 | -0.121 |
| UBE2M | ILMN_1701331 | 0.0247 | 0.00375 | -3.03 | -2.26502 | -0.122 |
| GTF2E2 | ILMN_1664931 | 0.034 | 0.00581 | -2.87 | -2.66005 | -0.122 |
| SNRPB2 | ILMN_1690706 | 0.0398 | 0.00723 | -2.79 | -2.8568 | -0.122 |
| UBE3C | ILMN_2181363 | 0.102 | 0.0284 | -2.25 | -4.06125 | -0.122 |
| EPN1 | ILMN_1772981 | 0.0272 | 0.00428 | -2.98 | -2.38291 | -0.123 |
| USP16 | ILMN_2397230 | 0.0774 | 0.019 | -2.42 | -3.71288 | -0.123 |
| GNAS | ILMN_1769191 | 0.0366 | 0.00645 | -2.83 | -2.75429 | -0.124 |
| ZDHHC6 | ILMN_2046003 | 0.0388 | 0.007 | -2.8 | -2.82813 | -0.124 |
| IDS | ILMN_1758626 | 0.0645 | 0.0146 | -2.52 | -3.48356 | -0.124 |
| RPL30 | ILMN_1754303 | 0.0387 | 0.00696 | -2.8 | -2.82214 | -0.125 |
| RPL32 | ILMN_1663799 | 0.0487 | 0.00971 | -2.68 | -3.1206 | -0.125 |
| SON | ILMN_2247664 | 0.0222 | 0.00323 | -3.08 | -2.12709 | -0.126 |
| MMADHC | ILMN_3248966 | 0.0242 | 0.00365 | -3.04 | -2.23955 | -0.126 |
| XPR1 | ILMN_2104106 | 0.0519 | 0.0107 | -2.64 | -3.20296 | -0.126 |
| MED30 | ILMN_1671603 | 0.0638 | 0.0144 | -2.53 | -3.46796 | -0.126 |
| NDUFAF3 | ILMN_2354515 | 0.0998 | 0.0275 | -2.26 | -4.03322 | -0.126 |
| COPG1 | ILMN_1708204 | 0.118 | 0.0354 | -2.16 | -4.25219 | -0.126 |
| ZSWIM1 | ILMN_1812856 | 0.14 | 0.0454 | -2.05 | -4.46234 | -0.126 |
| SKI | ILMN_1710598 | 0.149 | 0.0494 | -2.01 | -4.53405 | -0.126 |
| NRBP1 | ILMN_1670096 | 0.143 | 0.0463 | -2.04 | -4.47936 | -0.127 |
| ACLY | ILMN_2371379 | 0.144 | 0.0473 | -2.03 | -4.49682 | -0.127 |
| NECAP2 | ILMN_1749011 | 0.0361 | 0.00635 | -2.84 | -2.74022 | -0.128 |
| MAP3K11 | ILMN_1651788 | 0.064 | 0.0145 | -2.52 | -3.47417 | -0.128 |
| RAB5B | ILMN_1752582 | 0.0943 | 0.0252 | -2.3 | -3.95945 | -0.128 |
| HNRNPD | ILMN_1751368 | 0.127 | 0.0389 | -2.12 | -4.33192 | -0.128 |
| RPL11 | ILMN_2114876 | 0.0368 | 0.0065 | -2.83 | -2.76161 | -0.129 |
| FGFR1OP2 | ILMN_1657436 | 0.0499 | 0.0101 | -2.66 | -3.15721 | -0.129 |
| SENP2 | ILMN_1801121 | 0.0668 | 0.0154 | -2.5 | -3.52623 | -0.129 |
| WAC | ILMN_2323526 | 0.0712 | 0.0168 | -2.47 | -3.60495 | -0.129 |
| SRP19 | ILMN_2192032 | 0.0794 | 0.0197 | -2.4 | -3.74328 | -0.129 |
| OTULIN | ILMN_1790062 | 0.0996 | 0.0274 | -2.27 | -4.03012 | -0.129 |
| HSD17B11 | ILMN_1735367 | 0.129 | 0.0398 | -2.11 | -4.35187 | -0.129 |
| SRSF5 | ILMN_2378868 | 0.0235 | 0.00349 | -3.05 | -2.1987 | -0.13 |
| PSMD6 | ILMN_1779633 | 0.0281 | 0.00447 | -2.96 | -2.42417 | -0.13 |
| RAP2C | ILMN_1773561 | 0.0416 | 0.00773 | -2.76 | -2.91653 | -0.13 |
| ARHGAP9 | ILMN_2382657 | 0.0422 | 0.00787 | -2.76 | -2.93254 | -0.13 |
| GCC1 | ILMN_1682206 | 0.0272 | 0.00427 | -2.98 | -2.38116 | -0.131 |
| DHX15 | ILMN_2168449 | 0.0393 | 0.00714 | -2.79 | -2.84487 | -0.131 |
| SERP1 | ILMN_1706817 | 0.0941 | 0.0251 | -2.3 | -3.95727 | -0.131 |
| SYVN1 | ILMN_1803143 | 0.115 | 0.0338 | -2.18 | -4.21155 | -0.131 |
| RAB5A | ILMN_1805800 | 0.0315 | 0.00519 | -2.91 | -2.55867 | -0.132 |
| CCDC174 | ILMN_1750144 | 0.0354 | 0.00618 | -2.85 | -2.71515 | -0.132 |
| NSUN2 | ILMN_1680129 | 0.0513 | 0.0105 | -2.65 | -3.18643 | -0.132 |
| SEC24C | ILMN_1676600 | 0.0635 | 0.0143 | -2.53 | -3.46231 | -0.132 |
| DECR1 | ILMN_1720838 | 0.0847 | 0.0216 | -2.36 | -3.82657 | -0.132 |
| CEBPZ | ILMN_1744147 | 0.105 | 0.0296 | -2.23 | -4.09856 | -0.132 |
| CLPTM1L | ILMN_1752802 | 0.15 | 0.0499 | -2 | -4.54172 | -0.132 |
| NACA | ILMN_2167617 | 0.00967 | 0.00101 | -3.47 | -1.05741 | -0.133 |
| DIABLO | ILMN_2310589 | 0.0328 | 0.00553 | -2.89 | -2.61605 | -0.133 |
| POLDIP3 | ILMN_1688000 | 0.0344 | 0.00591 | -2.86 | -2.67578 | -0.133 |
| DCAF12 | ILMN_1786328 | 0.0585 | 0.0127 | -2.58 | -3.35619 | -0.133 |
| SBF2 | ILMN_2123665 | 0.104 | 0.029 | -2.24 | -4.08061 | -0.133 |
| PLAC8 | ILMN_2093343 | 0.126 | 0.0384 | -2.12 | -4.3209 | -0.133 |
| VTI1B | ILMN_1693136 | 0.127 | 0.039 | -2.11 | -4.33395 | -0.133 |
| KIF5B | ILMN_1788160 | 0.134 | 0.0423 | -2.08 | -4.40391 | -0.133 |
| FAM91A1 | ILMN_1777322 | 0.147 | 0.0482 | -2.02 | -4.51386 | -0.133 |
| RPL36AL | ILMN_2189936 | 0.0198 | 0.00271 | -3.14 | -1.96923 | -0.134 |
| PIP4K2A | ILMN_1911042 | 0.0579 | 0.0125 | -2.58 | -3.34361 | -0.134 |
| KCTD5 | ILMN_1672728 | 0.09 | 0.0236 | -2.33 | -3.90348 | -0.134 |
| SLC35F5 | ILMN_2206554 | 0.103 | 0.0286 | -2.25 | -4.06928 | -0.134 |
| DCTN1 | ILMN_2412807 | 0.115 | 0.034 | -2.17 | -4.21733 | -0.134 |
| MOB4 | ILMN_1813594 | 0.144 | 0.0471 | -2.03 | -4.49449 | -0.134 |
| TMBIM4 | ILMN_1664750 | 0.0132 | 0.00153 | -3.33 | -1.44409 | -0.135 |
| SVBP | ILMN_2050023 | 0.0239 | 0.00357 | -3.04 | -2.22014 | -0.135 |
| NSA2 | ILMN_1694259 | 0.0254 | 0.0039 | -3.01 | -2.29874 | -0.135 |
| LARP7 | ILMN_2392717 | 0.0337 | 0.00574 | -2.87 | -2.64849 | -0.135 |
| CAMLG | ILMN_1714599 | 0.0475 | 0.00934 | -2.69 | -3.08535 | -0.135 |
| CANX | ILMN_2401057 | 0.132 | 0.0416 | -2.09 | -4.38834 | -0.135 |
| FBXL12 | ILMN_1693438 | 0.14 | 0.045 | -2.05 | -4.45515 | -0.135 |
| UFC1 | ILMN_2110281 | 0.144 | 0.0472 | -2.03 | -4.4962 | -0.135 |
| KBTBD4 | ILMN_1687092 | 0.149 | 0.0493 | -2.01 | -4.5313 | -0.135 |
| MAP3K7 | ILMN_1810176 | 0.0115 | 0.00126 | -3.4 | -1.26795 | -0.136 |
| LSM5 | ILMN_1737947 | 0.0945 | 0.0253 | -2.3 | -3.96219 | -0.136 |
| ZBTB17 | ILMN_1711048 | 0.102 | 0.0282 | -2.25 | -4.05552 | -0.136 |
| FBL | ILMN_1719205 | 0.114 | 0.0333 | -2.18 | -4.19989 | -0.136 |
| ADSL | ILMN_1790757 | 0.115 | 0.0341 | -2.17 | -4.21917 | -0.136 |
| SLC43A2 | ILMN_1787127 | 0.125 | 0.0382 | -2.12 | -4.31555 | -0.136 |
| ARF5 | ILMN_1752340 | 0.034 | 0.00582 | -2.87 | -2.66175 | -0.137 |
| ATP5EP2 | ILMN_1756674 | 0.0499 | 0.0101 | -2.66 | -3.15629 | -0.137 |
| PSMC1 | ILMN_1736353 | 0.0794 | 0.0196 | -2.4 | -3.742 | -0.137 |
| PEX16 | ILMN_1745655 | 0.0971 | 0.0264 | -2.28 | -3.99872 | -0.137 |
| EP300 | ILMN_1744665 | 0.119 | 0.0355 | -2.16 | -4.25383 | -0.137 |
| RPS6KA1 | ILMN_1715173 | 0.128 | 0.0397 | -2.11 | -4.34917 | -0.137 |
| TAGLN2 | ILMN_2090105 | 0.14 | 0.0454 | -2.05 | -4.46231 | -0.137 |
| ARAF | ILMN_1658883 | 0.144 | 0.0468 | -2.03 | -4.488 | -0.137 |
| TBPL1 | ILMN_1708147 | 0.0135 | 0.00157 | -3.33 | -1.46971 | -0.138 |
| MDH1 | ILMN_1656913 | 0.0299 | 0.00485 | -2.93 | -2.49679 | -0.138 |
| NCKAP1L | ILMN_1674250 | 0.0489 | 0.0098 | -2.68 | -3.12881 | -0.138 |
| MED10 | ILMN_1707631 | 0.088 | 0.0229 | -2.34 | -3.87759 | -0.138 |
| PHF20L1 | ILMN_1732985 | 0.0965 | 0.0261 | -2.29 | -3.98896 | -0.138 |
| COMMD2 | ILMN_1683305 | 0.107 | 0.0302 | -2.22 | -4.11626 | -0.138 |
| GNL2 | ILMN_1761113 | 0.122 | 0.0367 | -2.14 | -4.28264 | -0.138 |
| OSTC | ILMN_1776005 | 0.142 | 0.046 | -2.04 | -4.47321 | -0.138 |
| RBMS1 | ILMN_1666444 | 0.0577 | 0.0124 | -2.58 | -3.33938 | -0.139 |
| SZRD1 | ILMN_1707503 | 0.0606 | 0.0133 | -2.56 | -3.39946 | -0.139 |
| MICU2 | ILMN_1738346 | 0.0615 | 0.0136 | -2.55 | -3.41892 | -0.139 |
| UBP1 | ILMN_1784410 | 0.0621 | 0.0138 | -2.54 | -3.43154 | -0.139 |
| BUD13 | ILMN_1749210 | 0.0695 | 0.0163 | -2.48 | -3.57717 | -0.139 |
| WDR20 | ILMN_1707199 | 0.0795 | 0.0197 | -2.4 | -3.74611 | -0.139 |
| FDFT1 | ILMN_1741096 | 0.147 | 0.0484 | -2.02 | -4.51633 | -0.139 |
| ZNF274 | ILMN_1688629 | 0.0388 | 0.00701 | -2.8 | -2.82899 | -0.14 |
| RNF181 | ILMN_1655340 | 0.0723 | 0.0172 | -2.46 | -3.62799 | -0.14 |
| POLR2C | ILMN_1659411 | 0.0675 | 0.0156 | -2.5 | -3.53969 | -0.141 |
| ATG3 | ILMN_1769566 | 0.113 | 0.0332 | -2.18 | -4.19627 | -0.141 |
| ACIN1 | ILMN_1699636 | 0.133 | 0.042 | -2.08 | -4.39733 | -0.141 |
| CNOT2 | ILMN_1772651 | 0.149 | 0.0492 | -2.01 | -4.53072 | -0.141 |
| MED1 | ILMN_1721729 | 0.0186 | 0.00248 | -3.17 | -1.88862 | -0.142 |
| RBSN | ILMN_1799890 | 0.0211 | 0.00298 | -3.11 | -2.05423 | -0.142 |
| EMC6 | ILMN_2405592 | 0.0316 | 0.00523 | -2.91 | -2.56594 | -0.142 |
| FEZ2 | ILMN_2403946 | 0.0691 | 0.0161 | -2.48 | -3.56937 | -0.142 |
| YME1L1 | ILMN_1734316 | 0.0882 | 0.023 | -2.34 | -3.88163 | -0.142 |
| RFWD2 | ILMN_2408001 | 0.123 | 0.0374 | -2.13 | -4.29797 | -0.142 |
| ECD | ILMN_2206645 | 0.13 | 0.0404 | -2.1 | -4.36333 | -0.142 |
| VIMP | ILMN_1787680 | 0.13 | 0.0406 | -2.1 | -4.36749 | -0.142 |
| SHMT2 | ILMN_1661264 | 0.134 | 0.0422 | -2.08 | -4.40024 | -0.142 |
| NACA | ILMN_2167616 | 0.00977 | 0.00102 | -3.47 | -1.07112 | -0.143 |
| DPM1 | ILMN_1658992 | 0.0268 | 0.00416 | -2.99 | -2.35844 | -0.143 |
| OCIAD1 | ILMN_1799604 | 0.0332 | 0.00563 | -2.88 | -2.63086 | -0.143 |
| MOB1A | ILMN_2125562 | 0.0697 | 0.0164 | -2.48 | -3.58197 | -0.143 |
| CD2 | ILMN_1695025 | 0.122 | 0.0369 | -2.14 | -4.28595 | -0.143 |
| C14orf166 | ILMN_1696708 | 0.0207 | 0.0029 | -3.12 | -2.02938 | -0.144 |
| BTF3 | ILMN_2319414 | 0.00553 | 0.000453 | -3.73 | -0.31846 | -0.145 |
| PUM1 | ILMN_1783424 | 0.059 | 0.0128 | -2.57 | -3.36884 | -0.145 |
| LBH | ILMN_2315979 | 0.0911 | 0.024 | -2.32 | -3.91716 | -0.145 |
| SNRPC | ILMN_2086064 | 0.114 | 0.0334 | -2.18 | -4.20053 | -0.145 |
| EIF2S3 | ILMN_1665717 | 0.00941 | 0.000967 | -3.49 | -1.02165 | -0.146 |
| ING3 | ILMN_2237746 | 0.0847 | 0.0216 | -2.36 | -3.82501 | -0.146 |
| FDFT1 | ILMN_2144088 | 0.14 | 0.0451 | -2.05 | -4.45792 | -0.146 |
| DPM1 | ILMN_2105308 | 0.0282 | 0.00448 | -2.96 | -2.42509 | -0.147 |
| ITCH | ILMN_1752283 | 0.0465 | 0.00903 | -2.71 | -3.05553 | -0.147 |
| ATP5J | ILMN_1652806 | 0.0615 | 0.0136 | -2.55 | -3.41878 | -0.147 |
| U2SURP | ILMN_2286334 | 0.0756 | 0.0183 | -2.43 | -3.68278 | -0.147 |
| WDR82 | ILMN_1679655 | 0.112 | 0.0326 | -2.19 | -4.18067 | -0.147 |
| SKIL | ILMN_1736834 | 0.116 | 0.0343 | -2.17 | -4.22528 | -0.147 |
| SNHG5 | ILMN_1653794 | 0.118 | 0.0353 | -2.16 | -4.24971 | -0.147 |
| BCAP29 | ILMN_2342084 | 0.127 | 0.0388 | -2.12 | -4.33024 | -0.147 |
| APH1A | ILMN_1658472 | 0.0166 | 0.00211 | -3.23 | -1.73962 | -0.148 |
| SPI1 | ILMN_1696463 | 0.034 | 0.00582 | -2.87 | -2.66195 | -0.148 |
| MAGOH | ILMN_2215656 | 0.0442 | 0.00842 | -2.73 | -2.99272 | -0.148 |
| GAPDH | ILMN_2038778 | 0.0503 | 0.0102 | -2.66 | -3.16627 | -0.148 |
| OSTC | ILMN_3236156 | 0.0526 | 0.0109 | -2.64 | -3.22186 | -0.148 |
| ZZEF1 | ILMN_1786396 | 0.0634 | 0.0142 | -2.53 | -3.45898 | -0.148 |
| ZC3H15 | ILMN_2062112 | 0.0638 | 0.0144 | -2.53 | -3.46764 | -0.148 |
| GSK3B | ILMN_1779376 | 0.0859 | 0.0221 | -2.36 | -3.84387 | -0.148 |
| HACD2 | ILMN_2170353 | 0.112 | 0.0327 | -2.19 | -4.1842 | -0.148 |
| TMEM8A | ILMN_1741371 | 0.124 | 0.0378 | -2.13 | -4.30643 | -0.148 |
| AP2M1 | ILMN_2402798 | 0.133 | 0.042 | -2.08 | -4.39671 | -0.148 |
| FUBP3 | ILMN_2353033 | 0.135 | 0.0427 | -2.07 | -4.41069 | -0.148 |
| ARPC1B | ILMN_2085760 | 0.0523 | 0.0108 | -2.64 | -3.21605 | -0.149 |
| KMT2E | ILMN_1783606 | 0.133 | 0.0421 | -2.08 | -4.39832 | -0.149 |
| SDF4 | ILMN_1696065 | 0.0411 | 0.00761 | -2.77 | -2.9027 | -0.15 |
| ISOC1 | ILMN_1764861 | 0.0473 | 0.00926 | -2.7 | -3.07798 | -0.15 |
| QRICH1 | ILMN_1676002 | 0.0549 | 0.0116 | -2.61 | -3.2774 | -0.15 |
| CTSH | ILMN_2390853 | 0.0848 | 0.0217 | -2.36 | -3.82734 | -0.15 |
| RPA3 | ILMN_1716895 | 0.0896 | 0.0235 | -2.33 | -3.89851 | -0.15 |
| RWDD1 | ILMN_2316806 | 0.0947 | 0.0254 | -2.3 | -3.9649 | -0.15 |
| SETDB1 | ILMN_1718207 | 0.106 | 0.0298 | -2.23 | -4.10265 | -0.15 |
| MTMR14 | ILMN_1659240 | 0.142 | 0.0461 | -2.04 | -4.47495 | -0.15 |
| CDC14A | ILMN_1774930 | 0.147 | 0.0483 | -2.02 | -4.5143 | -0.15 |
| YWHAB | ILMN_1694385 | 0.0312 | 0.00512 | -2.92 | -2.5453 | -0.151 |
| ZSWIM6 | ILMN_1777061 | 0.0325 | 0.00546 | -2.89 | -2.60333 | -0.151 |
| C1orf52 | ILMN_1742611 | 0.0422 | 0.00787 | -2.76 | -2.93242 | -0.151 |
| TRAPPC4 | ILMN_1814650 | 0.0766 | 0.0187 | -2.42 | -3.70062 | -0.151 |
| GMEB1 | ILMN_1727761 | 0.13 | 0.0404 | -2.1 | -4.36363 | -0.151 |
| RAB4B | ILMN_1803136 | 0.0321 | 0.00536 | -2.9 | -2.58691 | -0.152 |
| MZF1 | ILMN_1749838 | 0.0837 | 0.0213 | -2.37 | -3.8127 | -0.152 |
| CRLF3 | ILMN_2155228 | 0.0861 | 0.0222 | -2.35 | -3.84791 | -0.152 |
| RNF41 | ILMN_1808095 | 0.106 | 0.0299 | -2.23 | -4.10623 | -0.152 |
| PRPF40A | ILMN_1659854 | 0.112 | 0.0323 | -2.2 | -4.17372 | -0.152 |
| DDB1 | ILMN_1775937 | 0.149 | 0.0494 | -2.01 | -4.53355 | -0.152 |
| IGBP1 | ILMN_1717165 | 0.0349 | 0.00604 | -2.86 | -2.69554 | -0.153 |
| RAB8B | ILMN_2173004 | 0.0388 | 0.00699 | -2.8 | -2.82585 | -0.153 |
| SPTY2D1 | ILMN_1728699 | 0.0457 | 0.0088 | -2.72 | -3.03249 | -0.153 |
| BFAR | ILMN_1814808 | 0.046 | 0.00889 | -2.71 | -3.04156 | -0.153 |
| EIF4G2 | ILMN_1761519 | 0.0491 | 0.00987 | -2.67 | -3.13444 | -0.153 |
| FEZ2 | ILMN_1739586 | 0.0527 | 0.0109 | -2.63 | -3.22701 | -0.153 |
| SMS | ILMN_1694305 | 0.0809 | 0.0202 | -2.39 | -3.76789 | -0.153 |
| PPCS | ILMN_2396996 | 0.116 | 0.0344 | -2.17 | -4.22777 | -0.153 |
| ZNF800 | ILMN_1688346 | 0.022 | 0.00316 | -3.09 | -2.10867 | -0.154 |
| ANXA7 | ILMN_1703791 | 0.0315 | 0.00522 | -2.91 | -2.56303 | -0.154 |
| CALM3 | ILMN_1666385 | 0.0335 | 0.00569 | -2.88 | -2.64181 | -0.154 |
| PPP1R18 | ILMN_1732967 | 0.0856 | 0.022 | -2.36 | -3.83948 | -0.154 |
| NDUFS7 | ILMN_1669966 | 0.048 | 0.00946 | -2.69 | -3.09722 | -0.155 |
| MOB4 | ILMN_3229859 | 0.0492 | 0.00991 | -2.67 | -3.13861 | -0.155 |
| HIST1H2BK | ILMN_1796179 | 0.1 | 0.0276 | -2.26 | -4.03862 | -0.155 |
| CXXC1 | ILMN_1691276 | 0.112 | 0.0326 | -2.19 | -4.18012 | -0.155 |
| DCAF15 | ILMN_2192620 | 0.114 | 0.0336 | -2.18 | -4.2065 | -0.155 |
| RFWD3 | ILMN_1687107 | 0.128 | 0.0394 | -2.11 | -4.34305 | -0.155 |
| APH1A | ILMN_2398388 | 0.149 | 0.0492 | -2.01 | -4.53095 | -0.155 |
| EIF3D | ILMN_1739847 | 0.0117 | 0.00129 | -3.39 | -1.28955 | -0.156 |
| CCNT2 | ILMN_1722522 | 0.0174 | 0.00225 | -3.2 | -1.79973 | -0.156 |
| SDF4 | ILMN_2378257 | 0.0209 | 0.00294 | -3.11 | -2.04202 | -0.156 |
| PJA2 | ILMN_1688702 | 0.0344 | 0.00592 | -2.86 | -2.67713 | -0.156 |
| RAB5C | ILMN_1769665 | 0.038 | 0.0068 | -2.81 | -2.80151 | -0.156 |
| PRPF3 | ILMN_1715392 | 0.043 | 0.00809 | -2.75 | -2.95768 | -0.156 |
| DNAJB11 | ILMN_1753243 | 0.0515 | 0.0105 | -2.65 | -3.19076 | -0.156 |
| DNAJC14 | ILMN_1785177 | 0.0569 | 0.0122 | -2.59 | -3.32083 | -0.156 |
| ARHGEF2 | ILMN_1703477 | 0.105 | 0.0295 | -2.23 | -4.09467 | -0.156 |
| FAM96A | ILMN_1761981 | 0.113 | 0.0331 | -2.19 | -4.19463 | -0.156 |
| ZNF266 | ILMN_1753782 | 0.115 | 0.0338 | -2.18 | -4.21304 | -0.156 |
| LOC148413 | ILMN_3244323 | 0.143 | 0.0463 | -2.04 | -4.47933 | -0.156 |
| TCF20 | ILMN_2368068 | 0.143 | 0.0465 | -2.04 | -4.48248 | -0.156 |
| HLA-DRB1 | ILMN_1715169 | 0.0349 | 0.00605 | -2.85 | -2.69654 | -0.157 |
| MRPL51 | ILMN_2097421 | 0.0521 | 0.0107 | -2.64 | -3.20772 | -0.157 |
| DNAJC7 | ILMN_1663616 | 0.0902 | 0.0237 | -2.33 | -3.90714 | -0.157 |
| CNOT7 | ILMN_1715886 | 0.101 | 0.0279 | -2.26 | -4.04573 | -0.157 |
| NUCB2 | ILMN_1655913 | 0.137 | 0.0439 | -2.06 | -4.43375 | -0.157 |
| HLA-DRA | ILMN_1689655 | 0.0151 | 0.00185 | -3.27 | -1.62051 | -0.158 |
| FAM199X | ILMN_1811121 | 0.0468 | 0.00911 | -2.7 | -3.06323 | -0.158 |
| ZSCAN32 | ILMN_1684591 | 0.0594 | 0.013 | -2.57 | -3.37889 | -0.158 |
| ELMOD2 | ILMN_1765159 | 0.0825 | 0.0208 | -2.38 | -3.79162 | -0.158 |
| PSMA4 | ILMN_2198376 | 0.0866 | 0.0224 | -2.35 | -3.85508 | -0.158 |
| ATP5EP2 | ILMN_2225887 | 0.0286 | 0.00457 | -2.96 | -2.44239 | -0.159 |
| ATF6B | ILMN_3273641 | 0.0818 | 0.0206 | -2.38 | -3.78318 | -0.159 |
| JKAMP | ILMN_1700276 | 0.0849 | 0.0217 | -2.36 | -3.82947 | -0.159 |
| HPCAL1 | ILMN_1764850 | 0.086 | 0.0221 | -2.35 | -3.84623 | -0.159 |
| PIGT | ILMN_1738759 | 0.11 | 0.0318 | -2.2 | -4.15936 | -0.159 |
| RBMS1 | ILMN_2358541 | 0.139 | 0.0445 | -2.06 | -4.44607 | -0.159 |
| PPP4C | ILMN_3248975 | 0.00394 | 0.00028 | -3.88 | 0.12812 | -0.16 |
| LYAR | ILMN_2221564 | 0.0167 | 0.00212 | -3.22 | -1.74417 | -0.16 |
| SPI1 | ILMN_2392043 | 0.041 | 0.00756 | -2.77 | -2.8971 | -0.16 |
| MRPL13 | ILMN_1671158 | 0.124 | 0.0377 | -2.13 | -4.3041 | -0.16 |
| NDUFAB1 | ILMN_2179018 | 0.145 | 0.0475 | -2.03 | -4.50092 | -0.16 |
| LSM3 | ILMN_1719032 | 0.0354 | 0.00617 | -2.85 | -2.71392 | -0.161 |
| UBE3B | ILMN_1752027 | 0.0524 | 0.0108 | -2.64 | -3.21795 | -0.161 |
| IER3 | ILMN_1682717 | 0.0535 | 0.0112 | -2.62 | -3.24656 | -0.161 |
| HLA-F | ILMN_1762861 | 0.056 | 0.0119 | -2.6 | -3.30288 | -0.161 |
| CSNK1G2 | ILMN_1706521 | 0.0738 | 0.0177 | -2.44 | -3.6522 | -0.161 |
| HOOK3 | ILMN_1741464 | 0.145 | 0.0477 | -2.03 | -4.50411 | -0.161 |
| BUD31 | ILMN_1710697 | 0.00371 | 0.00026 | -3.9 | 0.19999 | -0.162 |
| EXOC7 | ILMN_1815012 | 0.0521 | 0.0107 | -2.64 | -3.20961 | -0.162 |
| EIF2A | ILMN_1733305 | 0.0729 | 0.0174 | -2.45 | -3.6376 | -0.162 |
| EIF2AK1 | ILMN_2156267 | 0.0762 | 0.0186 | -2.43 | -3.6933 | -0.162 |
| AGGF1 | ILMN_2064917 | 0.106 | 0.0301 | -2.23 | -4.11156 | -0.162 |
| SRSF1 | ILMN_1795341 | 0.0183 | 0.00242 | -3.18 | -1.86314 | -0.163 |
| FBRS | ILMN_3245236 | 0.0322 | 0.00538 | -2.9 | -2.59124 | -0.163 |
| UBE2V2 | ILMN_1770515 | 0.0378 | 0.00676 | -2.81 | -2.79662 | -0.163 |
| HMG20B | ILMN_1651315 | 0.0675 | 0.0156 | -2.5 | -3.53943 | -0.163 |
| TIGD7 | ILMN_1676905 | 0.0719 | 0.0171 | -2.46 | -3.61889 | -0.163 |
| USF1 | ILMN_1777982 | 0.0754 | 0.0183 | -2.43 | -3.67953 | -0.163 |
| CD74 | ILMN_1761464 | 0.107 | 0.0304 | -2.22 | -4.11965 | -0.163 |
| IL2RG | ILMN_1794386 | 0.112 | 0.0327 | -2.19 | -4.18361 | -0.163 |
| DCTN3 | ILMN_1762281 | 0.127 | 0.039 | -2.11 | -4.33373 | -0.163 |
| TNPO2 | ILMN_1656066 | 0.0235 | 0.00348 | -3.05 | -2.19667 | -0.164 |
| KANSL2 | ILMN_1713189 | 0.068 | 0.0158 | -2.49 | -3.54902 | -0.164 |
| LPAR2 | ILMN_1662741 | 0.0849 | 0.0217 | -2.36 | -3.83026 | -0.164 |
| BRWD1 | ILMN_1673518 | 0.0962 | 0.026 | -2.29 | -3.98527 | -0.164 |
| PIN1 | ILMN_1776375 | 0.106 | 0.0298 | -2.23 | -4.10346 | -0.164 |
| RELA | ILMN_1705266 | 0.13 | 0.0404 | -2.1 | -4.36488 | -0.164 |
| NDUFB11 | ILMN_1749709 | 0.018 | 0.00235 | -3.19 | -1.83924 | -0.165 |
| DDT | ILMN_1690982 | 0.0559 | 0.0119 | -2.6 | -3.30058 | -0.165 |
| SS18 | ILMN_1805646 | 0.0925 | 0.0245 | -2.31 | -3.93562 | -0.165 |
| PTGES3 | ILMN_1719749 | 0.122 | 0.0367 | -2.14 | -4.28295 | -0.165 |
| JTB | ILMN_2206716 | 0.00177 | 9.72E-05 | -4.2 | 1.12087 | -0.166 |
| STK40 | ILMN_2075927 | 0.0489 | 0.00979 | -2.68 | -3.1275 | -0.166 |
| ATP5G2 | ILMN_1660577 | 0.0644 | 0.0146 | -2.52 | -3.48071 | -0.166 |
| ARAP1 | ILMN_3269719 | 0.0684 | 0.0159 | -2.49 | -3.55686 | -0.166 |
| ITFG1 | ILMN_1693310 | 0.0737 | 0.0177 | -2.44 | -3.6515 | -0.166 |
| BRMS1 | ILMN_1719165 | 0.0968 | 0.0262 | -2.28 | -3.99386 | -0.166 |
| SH3GL1 | ILMN_1788062 | 0.109 | 0.0314 | -2.21 | -4.14852 | -0.166 |
| NEDD9 | ILMN_1758719 | 0.121 | 0.0366 | -2.14 | -4.28073 | -0.166 |
| DERL2 | ILMN_1877068 | 0.14 | 0.0451 | -2.05 | -4.45699 | -0.166 |
| DERL2 | ILMN_1761969 | 0.0089 | 0.000894 | -3.51 | -0.94881 | -0.167 |
| MANBAL | ILMN_1673944 | 0.0106 | 0.00114 | -3.43 | -1.17072 | -0.167 |
| DR1 | ILMN_1759983 | 0.0649 | 0.0148 | -2.52 | -3.49273 | -0.167 |
| CNOT7 | ILMN_1656134 | 0.0704 | 0.0166 | -2.47 | -3.59312 | -0.167 |
| RBMS1 | ILMN_2358540 | 0.13 | 0.0405 | -2.1 | -4.36639 | -0.167 |
| PARP8 | ILMN_1806651 | 0.14 | 0.0454 | -2.05 | -4.46189 | -0.167 |
| PSMA4 | ILMN_1682098 | 0.147 | 0.0485 | -2.02 | -4.51903 | -0.167 |
| RPL36AL | ILMN_2189933 | 0.0048 | 0.000375 | -3.79 | -0.14269 | -0.168 |
| UXT | ILMN_1671314 | 0.00703 | 0.000642 | -3.62 | -0.64284 | -0.168 |
| DNTTIP1 | ILMN_1691117 | 0.0135 | 0.00158 | -3.32 | -1.47325 | -0.168 |
| EIF4G2 | ILMN_2380946 | 0.0209 | 0.00295 | -3.11 | -2.04643 | -0.168 |
| COPS5 | ILMN_1736002 | 0.027 | 0.00421 | -2.98 | -2.36987 | -0.168 |
| NPTN | ILMN_2336982 | 0.0318 | 0.00529 | -2.9 | -2.57587 | -0.168 |
| SSR2 | ILMN_1783226 | 0.0347 | 0.006 | -2.86 | -2.68954 | -0.168 |
| TIMM17B | ILMN_1813260 | 0.0482 | 0.00954 | -2.69 | -3.10433 | -0.168 |
| NDUFA4 | ILMN_1751258 | 0.0515 | 0.0105 | -2.65 | -3.19257 | -0.168 |
| ATF1 | ILMN_1801923 | 0.0898 | 0.0236 | -2.33 | -3.90201 | -0.168 |
| TVP23B | ILMN_1751492 | 0.12 | 0.0361 | -2.15 | -4.26705 | -0.168 |
| LSM3 | ILMN_2229242 | 0.0272 | 0.00428 | -2.98 | -2.38325 | -0.169 |
| RAB5C | ILMN_2399140 | 0.0386 | 0.00694 | -2.8 | -2.8195 | -0.169 |
| TIMM22 | ILMN_1706959 | 0.0483 | 0.00961 | -2.68 | -3.11086 | -0.169 |
| CKS2 | ILMN_1756326 | 0.0552 | 0.0117 | -2.61 | -3.28431 | -0.169 |
| LPCAT1 | ILMN_1702171 | 0.0913 | 0.0241 | -2.32 | -3.92031 | -0.169 |
| ALKBH1 | ILMN_2375973 | 0.0962 | 0.0259 | -2.29 | -3.98445 | -0.169 |
| ALG13 | ILMN_1761456 | 0.141 | 0.0455 | -2.05 | -4.46518 | -0.169 |
| E2F4 | ILMN_1761828 | 0.0313 | 0.00515 | -2.91 | -2.55103 | -0.17 |
| RASSF5 | ILMN_2362902 | 0.047 | 0.00918 | -2.7 | -3.07019 | -0.17 |
| DHX36 | ILMN_2100000 | 0.0802 | 0.02 | -2.4 | -3.75787 | -0.17 |
| PATL1 | ILMN_1680782 | 0.0908 | 0.0239 | -2.32 | -3.91407 | -0.17 |
| PLEK | ILMN_1795762 | 0.123 | 0.0372 | -2.13 | -4.29436 | -0.17 |
| TACR3 | ILMN_1658369 | 0.127 | 0.0389 | -2.12 | -4.33078 | -0.17 |
| MTHFS | ILMN_1772302 | 0.13 | 0.0408 | -2.09 | -4.3727 | -0.17 |
| ARCN1 | ILMN_1699703 | 0.0104 | 0.00111 | -3.44 | -1.14712 | -0.171 |
| OSBP | ILMN_1706376 | 0.0171 | 0.0022 | -3.21 | -1.77733 | -0.171 |
| MED31 | ILMN_3251451 | 0.0374 | 0.00666 | -2.82 | -2.78332 | -0.171 |
| INIP | ILMN_1688621 | 0.0527 | 0.0109 | -2.63 | -3.22478 | -0.171 |
| EIF4A3 | ILMN_1667043 | 0.0731 | 0.0175 | -2.45 | -3.64005 | -0.171 |
| SCNM1 | ILMN_2375651 | 0.0877 | 0.0228 | -2.34 | -3.87167 | -0.171 |
| CXXC5 | ILMN_3307729 | 0.092 | 0.0244 | -2.31 | -3.93034 | -0.171 |
| CCT2 | ILMN_1679382 | 0.123 | 0.0374 | -2.13 | -4.29772 | -0.171 |
| TADA2B | ILMN_3239378 | 0.135 | 0.043 | -2.07 | -4.41655 | -0.171 |
| WAC | ILMN_2374692 | 0.0115 | 0.00126 | -3.4 | -1.26805 | -0.172 |
| TIAL1 | ILMN_1796855 | 0.0125 | 0.00142 | -3.36 | -1.37611 | -0.172 |
| CDC5L | ILMN_1652907 | 0.013 | 0.00149 | -3.34 | -1.42059 | -0.172 |
| CALCOCO2 | ILMN_1755504 | 0.0465 | 0.00904 | -2.71 | -3.05626 | -0.172 |
| IREB2 | ILMN_1726554 | 0.0631 | 0.0141 | -2.53 | -3.45163 | -0.172 |
| NCOA6 | ILMN_1695797 | 0.0797 | 0.0198 | -2.4 | -3.75005 | -0.172 |
| QKI | ILMN_2336280 | 0.139 | 0.0447 | -2.05 | -4.4492 | -0.172 |
| GAPDH | ILMN_1802252 | 0.0519 | 0.0107 | -2.64 | -3.2033 | -0.173 |
| TRIP11 | ILMN_1658144 | 0.0615 | 0.0136 | -2.55 | -3.42015 | -0.173 |
| GNPAT | ILMN_1699859 | 0.0729 | 0.0174 | -2.45 | -3.63759 | -0.173 |
| ZNF430 | ILMN_1780026 | 0.0798 | 0.0198 | -2.4 | -3.7515 | -0.173 |
| UQCR10 | ILMN_2366714 | 0.0918 | 0.0243 | -2.32 | -3.9274 | -0.173 |
| DDX50 | ILMN_1712320 | 0.111 | 0.0319 | -2.2 | -4.1633 | -0.173 |
| ZC3H7A | ILMN_2212690 | 0.131 | 0.0411 | -2.09 | -4.37945 | -0.173 |
| SNX14 | ILMN_2361570 | 0.0184 | 0.00244 | -3.18 | -1.8707 | -0.174 |
| CCZ1B | ILMN_1713322 | 0.0192 | 0.0026 | -3.15 | -1.93206 | -0.174 |
| RAB4B | ILMN_2109994 | 0.0529 | 0.011 | -2.63 | -3.23063 | -0.174 |
| PSMG4 | ILMN_3224204 | 0.131 | 0.0413 | -2.09 | -4.38206 | -0.174 |
| THAP12 | ILMN_1655622 | 0.133 | 0.042 | -2.08 | -4.39664 | -0.174 |
| GGPS1 | ILMN_1692276 | 0.00228 | 0.000136 | -4.1 | 0.80393 | -0.175 |
| MED4 | ILMN_1664641 | 0.00555 | 0.000456 | -3.73 | -0.32458 | -0.175 |
| PPIL3 | ILMN_1770020 | 0.0203 | 0.00282 | -3.13 | -2.00429 | -0.175 |
| IMPDH2 | ILMN_1705737 | 0.022 | 0.00319 | -3.08 | -2.11638 | -0.175 |
| C18orf21 | ILMN_1805998 | 0.0414 | 0.00767 | -2.77 | -2.90999 | -0.175 |
| ANAPC11 | ILMN_1722102 | 0.0697 | 0.0163 | -2.48 | -3.58154 | -0.175 |
| STX11 | ILMN_1720771 | 0.0755 | 0.0183 | -2.43 | -3.68036 | -0.175 |
| NXF1 | ILMN_2358652 | 0.0765 | 0.0187 | -2.42 | -3.69831 | -0.175 |
| ABRACL | ILMN_1720858 | 0.0952 | 0.0255 | -2.3 | -3.97049 | -0.175 |
| PPCS | ILMN_1776094 | 0.0997 | 0.0274 | -2.27 | -4.03153 | -0.175 |
| MTIF3 | ILMN_1718271 | 0.104 | 0.029 | -2.24 | -4.07923 | -0.175 |
| RPA1 | ILMN_2049642 | 0.106 | 0.03 | -2.23 | -4.11082 | -0.175 |
| COIL | ILMN_1688034 | 0.109 | 0.0312 | -2.21 | -4.14199 | -0.175 |
| COPS4 | ILMN_1726755 | 0.135 | 0.043 | -2.07 | -4.41733 | -0.175 |
| TMEM14B | ILMN_1685258 | 0.137 | 0.0437 | -2.06 | -4.42972 | -0.175 |
| SH2B2 | ILMN_1669833 | 0.0482 | 0.00955 | -2.69 | -3.10518 | -0.176 |
| LILRB2 | ILMN_2312340 | 0.0517 | 0.0106 | -2.65 | -3.19618 | -0.176 |
| ACP1 | ILMN_2344956 | 0.0609 | 0.0134 | -2.55 | -3.40611 | -0.176 |
| ANAPC5 | ILMN_1723177 | 0.0629 | 0.0141 | -2.54 | -3.44847 | -0.176 |
| USP1 | ILMN_1696975 | 0.0763 | 0.0186 | -2.42 | -3.69449 | -0.176 |
| TMEM208 | ILMN_1700419 | 0.132 | 0.0413 | -2.09 | -4.38343 | -0.176 |
| CCT7 | ILMN_2341793 | 0.0167 | 0.00212 | -3.22 | -1.74519 | -0.177 |
| DNAJB12 | ILMN_1758545 | 0.0415 | 0.00771 | -2.77 | -2.91414 | -0.177 |
| SMG9 | ILMN_1737005 | 0.0674 | 0.0156 | -2.5 | -3.53752 | -0.177 |
| DIABLO | ILMN_1665335 | 0.1 | 0.0276 | -2.26 | -4.03786 | -0.177 |
| ARID1A | ILMN_1797341 | 0.103 | 0.0288 | -2.25 | -4.07374 | -0.177 |
| TMEM219 | ILMN_1737644 | 0.111 | 0.032 | -2.2 | -4.164 | -0.177 |
| CCZ1 | ILMN_1708604 | 0.0498 | 0.0101 | -2.66 | -3.15357 | -0.178 |
| BRD7 | ILMN_2082810 | 0.0545 | 0.0115 | -2.62 | -3.26758 | -0.178 |
| DENND3 | ILMN_1692742 | 0.0635 | 0.0143 | -2.53 | -3.46054 | -0.178 |
| CPSF2 | ILMN_1673185 | 0.096 | 0.0259 | -2.29 | -3.98245 | -0.178 |
| TESK1 | ILMN_1791067 | 0.111 | 0.032 | -2.2 | -4.16451 | -0.178 |
| LASP1 | ILMN_1665909 | 0.121 | 0.0365 | -2.14 | -4.27859 | -0.178 |
| SUPT6H | ILMN_1758717 | 0.123 | 0.0374 | -2.13 | -4.29801 | -0.178 |
| C1GALT1C1 | ILMN_2401730 | 0.128 | 0.0394 | -2.11 | -4.3433 | -0.178 |
| USP16 | ILMN_2397231 | 0.00433 | 0.000324 | -3.83 | -0.00676 | -0.179 |
| SPRTN | ILMN_1655485 | 0.0244 | 0.00368 | -3.03 | -2.24734 | -0.179 |
| FCN1 | ILMN_1668063 | 0.0311 | 0.00509 | -2.92 | -2.54119 | -0.179 |
| PDCD2 | ILMN_1797684 | 0.0568 | 0.0121 | -2.59 | -3.31814 | -0.179 |
| PELO | ILMN_1770811 | 0.124 | 0.0378 | -2.13 | -4.30649 | -0.179 |
| DDIT3 | ILMN_1676984 | 0.136 | 0.0434 | -2.07 | -4.42488 | -0.179 |
| TRIP4 | ILMN_1661173 | 0.0198 | 0.00272 | -3.14 | -1.97198 | -0.18 |
| EIF4E2 | ILMN_1738326 | 0.0287 | 0.00457 | -2.96 | -2.44415 | -0.18 |
| GMPPB | ILMN_2412927 | 0.0612 | 0.0135 | -2.55 | -3.4114 | -0.18 |
| ANP32B | ILMN_1684293 | 0.0749 | 0.0181 | -2.44 | -3.67008 | -0.18 |
| ZCCHC8 | ILMN_1792092 | 0.0874 | 0.0227 | -2.34 | -3.86778 | -0.18 |
| SLC35A5 | ILMN_1709817 | 0.0944 | 0.0253 | -2.3 | -3.96103 | -0.18 |
| SNUPN | ILMN_2364535 | 0.114 | 0.0333 | -2.18 | -4.19993 | -0.18 |
| ARHGAP9 | ILMN_1663916 | 0.00837 | 0.000817 | -3.54 | -0.86565 | -0.181 |
| KXD1 | ILMN_1790951 | 0.0127 | 0.00144 | -3.35 | -1.38861 | -0.181 |
| VAMP7 | ILMN_1690524 | 0.0138 | 0.00162 | -3.32 | -1.49522 | -0.181 |
| STAMBP | ILMN_1739253 | 0.0163 | 0.00206 | -3.23 | -1.71665 | -0.181 |
| USP34 | ILMN_1739454 | 0.0167 | 0.00214 | -3.22 | -1.75272 | -0.181 |
| MOB4 | ILMN_3306168 | 0.0182 | 0.0024 | -3.18 | -1.85888 | -0.181 |
| SPCS1 | ILMN_1665280 | 0.0448 | 0.00856 | -2.73 | -3.00736 | -0.181 |
| MED12 | ILMN_1793386 | 0.0625 | 0.0139 | -2.54 | -3.44021 | -0.181 |
| LDHA | ILMN_1807106 | 0.0765 | 0.0187 | -2.42 | -3.69833 | -0.181 |
| BAG5 | ILMN_1728514 | 0.114 | 0.0333 | -2.18 | -4.19964 | -0.181 |
| CEP350 | ILMN_1742400 | 0.147 | 0.0484 | -2.02 | -4.51625 | -0.181 |
| GOLPH3 | ILMN_1708841 | 0.009 | 0.000906 | -3.51 | -0.9619 | -0.182 |
| RARA | ILMN_1659206 | 0.0454 | 0.00871 | -2.72 | -3.02324 | -0.182 |
| PPWD1 | ILMN_2223380 | 0.0632 | 0.0142 | -2.53 | -3.45472 | -0.182 |
| ZNF721 | ILMN_1805271 | 0.0712 | 0.0168 | -2.47 | -3.60489 | -0.182 |
| CDC25B | ILMN_2338323 | 0.132 | 0.0413 | -2.09 | -4.38368 | -0.182 |
| SOCS4 | ILMN_2413259 | 0.143 | 0.0466 | -2.04 | -4.48463 | -0.182 |
| RBM42 | ILMN_1670456 | 0.0158 | 0.00197 | -3.25 | -1.67612 | -0.183 |
| NAP1L1 | ILMN_1699208 | 0.0212 | 0.003 | -3.1 | -2.05999 | -0.183 |
| ZFAND3 | ILMN_1720053 | 0.0315 | 0.00519 | -2.91 | -2.55847 | -0.183 |
| VOPP1 | ILMN_1757827 | 0.117 | 0.0349 | -2.16 | -4.23951 | -0.183 |
| PEX16 | ILMN_1701466 | 0.0038 | 0.000268 | -3.89 | 0.17123 | -0.184 |
| SSU72 | ILMN_1664956 | 0.00962 | 0.000999 | -3.48 | -1.05146 | -0.184 |
| COX5A | ILMN_1704477 | 0.0136 | 0.0016 | -3.32 | -1.48433 | -0.184 |
| CD74 | ILMN_1736567 | 0.0158 | 0.00196 | -3.25 | -1.67346 | -0.184 |
| ACAP2 | ILMN_1766798 | 0.0229 | 0.00337 | -3.06 | -2.16614 | -0.184 |
| CPNE3 | ILMN_1773576 | 0.0543 | 0.0114 | -2.62 | -3.26344 | -0.184 |
| HIP1 | ILMN_1701403 | 0.12 | 0.0363 | -2.15 | -4.27195 | -0.184 |
| SPCS2 | ILMN_1809488 | 0.00503 | 0.000401 | -3.77 | -0.20634 | -0.185 |
| CBLL1 | ILMN_2073732 | 0.0152 | 0.00187 | -3.27 | -1.63131 | -0.185 |
| APPBP2 | ILMN_2149766 | 0.0241 | 0.00362 | -3.04 | -2.23199 | -0.185 |
| COG3 | ILMN_2129715 | 0.0439 | 0.00833 | -2.74 | -2.9831 | -0.185 |
| STX10 | ILMN_1753712 | 0.14 | 0.0452 | -2.05 | -4.45963 | -0.185 |
| ACBD3 | ILMN_1665945 | 0.00274 | 0.000176 | -4.02 | 0.56298 | -0.186 |
| RGL2 | ILMN_2124386 | 0.0164 | 0.00207 | -3.23 | -1.72246 | -0.186 |
| RAB2B | ILMN_1753002 | 0.0195 | 0.00267 | -3.14 | -1.95501 | -0.186 |
| GRIPAP1 | ILMN_1761176 | 0.0306 | 0.005 | -2.92 | -2.52425 | -0.186 |
| GATAD2B | ILMN_1766359 | 0.0349 | 0.00605 | -2.85 | -2.69579 | -0.186 |
| RPP25L | ILMN_1683175 | 0.0722 | 0.0172 | -2.46 | -3.62539 | -0.186 |
| SP4 | ILMN_2145997 | 0.0834 | 0.0212 | -2.37 | -3.80791 | -0.186 |
| TAF1L | ILMN_1799939 | 0.107 | 0.0305 | -2.22 | -4.12433 | -0.186 |
| IP6K2 | ILMN_3185709 | 0.123 | 0.0375 | -2.13 | -4.30099 | -0.186 |
| FAM206A | ILMN_2191929 | 0.143 | 0.0463 | -2.04 | -4.47944 | -0.186 |
| SHC1 | ILMN_1721022 | 0.0316 | 0.00524 | -2.91 | -2.56678 | -0.187 |
| MRPL21 | ILMN_2348050 | 0.0548 | 0.0116 | -2.61 | -3.27583 | -0.187 |
| NDUFA10 | ILMN_2225698 | 0.096 | 0.0259 | -2.29 | -3.98132 | -0.187 |
| SYNRG | ILMN_1805247 | 0.11 | 0.0318 | -2.2 | -4.15933 | -0.187 |
| P2RX1 | ILMN_1758529 | 0.125 | 0.0381 | -2.12 | -4.31412 | -0.187 |
| AKR7A3 | ILMN_2145396 | 0.127 | 0.0392 | -2.11 | -4.33821 | -0.187 |
| MEPCE | ILMN_2180827 | 0.149 | 0.0492 | -2.01 | -4.53096 | -0.187 |
| VIMP | ILMN_1803744 | 0.149 | 0.0493 | -2.01 | -4.53172 | -0.187 |
| NUDT2 | ILMN_2349444 | 0.149 | 0.0493 | -2.01 | -4.53212 | -0.187 |
| HCP5 | ILMN_1803945 | 0.00497 | 0.000395 | -3.77 | -0.19096 | -0.188 |
| GPR137 | ILMN_1804332 | 0.0315 | 0.00521 | -2.91 | -2.56205 | -0.188 |
| SEPT9 | ILMN_1769118 | 0.033 | 0.00558 | -2.88 | -2.62336 | -0.188 |
| TP53BP1 | ILMN_1664440 | 0.0344 | 0.00594 | -2.86 | -2.67958 | -0.188 |
| FAM193A | ILMN_1651504 | 0.0381 | 0.00682 | -2.81 | -2.80446 | -0.188 |
| PPFIA1 | ILMN_2382126 | 0.0952 | 0.0256 | -2.29 | -3.97154 | -0.188 |
| LRRC47 | ILMN_1668484 | 0.106 | 0.0299 | -2.23 | -4.10644 | -0.188 |
| DENND1C | ILMN_1767020 | 0.122 | 0.0367 | -2.14 | -4.28251 | -0.188 |
| PANK2 | ILMN_2373177 | 0.141 | 0.0456 | -2.05 | -4.46661 | -0.188 |
| NBPF3 | ILMN_2265995 | 0.15 | 0.0499 | -2 | -4.5419 | -0.188 |
| SENP5 | ILMN_1675501 | 0.0574 | 0.0123 | -2.59 | -3.33234 | -0.189 |
| NAA38 | ILMN_1691131 | 0.114 | 0.0334 | -2.18 | -4.20178 | -0.189 |
| CERS2 | ILMN_1726108 | 0.128 | 0.0394 | -2.11 | -4.34272 | -0.189 |
| CMC2 | ILMN_1783333 | 0.131 | 0.0409 | -2.09 | -4.37463 | -0.189 |
| POLR2C | ILMN_1732300 | 0.0585 | 0.0127 | -2.58 | -3.35581 | -0.19 |
| UBAC2 | ILMN_1713993 | 0.0586 | 0.0127 | -2.58 | -3.35865 | -0.19 |
| TMOD3 | ILMN_1809484 | 0.0919 | 0.0243 | -2.32 | -3.9283 | -0.19 |
| TOR1AIP1 | ILMN_2141941 | 0.0317 | 0.00526 | -2.91 | -2.57004 | -0.191 |
| STK10 | ILMN_1651692 | 0.0635 | 0.0143 | -2.53 | -3.4607 | -0.191 |
| RASGRP4 | ILMN_1714650 | 0.0663 | 0.0152 | -2.51 | -3.5173 | -0.191 |
| TAOK3 | ILMN_3307863 | 0.0702 | 0.0165 | -2.47 | -3.58857 | -0.191 |
| KMT2C | ILMN_2408877 | 0.0918 | 0.0243 | -2.32 | -3.92663 | -0.191 |
| F2RL1 | ILMN_1673113 | 0.143 | 0.0464 | -2.04 | -4.48113 | -0.191 |
| TNFRSF1B | ILMN_1764788 | 0.00895 | 9.00E-04 | -3.51 | -0.95495 | -0.192 |
| TERF2 | ILMN_1768488 | 0.0223 | 0.00324 | -3.08 | -2.13216 | -0.192 |
| CXCR4 | ILMN_1801584 | 0.0261 | 0.00404 | -3 | -2.33074 | -0.192 |
| CTSC | ILMN_1696347 | 0.0426 | 0.00797 | -2.75 | -2.94417 | -0.192 |
| CXorf40A | ILMN_2064655 | 0.0486 | 0.0097 | -2.68 | -3.11927 | -0.192 |
| RGS2 | ILMN_2197365 | 0.0694 | 0.0162 | -2.48 | -3.57569 | -0.192 |
| WRNIP1 | ILMN_1703036 | 0.0784 | 0.0193 | -2.41 | -3.72708 | -0.192 |
| SPEN | ILMN_1802611 | 0.0832 | 0.0211 | -2.37 | -3.80346 | -0.192 |
| LPXN | ILMN_1742789 | 0.104 | 0.0289 | -2.24 | -4.07786 | -0.192 |
| AGAP6 | ILMN_2209578 | 0.147 | 0.0485 | -2.02 | -4.51784 | -0.192 |
| POLR2J2 | ILMN_3241970 | 0.0527 | 0.0109 | -2.63 | -3.22613 | -0.193 |
| GNL3L | ILMN_1708414 | 0.103 | 0.0287 | -2.25 | -4.07201 | -0.193 |
| ZFYVE27 | ILMN_2322972 | 0.137 | 0.0437 | -2.06 | -4.42976 | -0.193 |
| SRP9 | ILMN_1759883 | 0.138 | 0.0444 | -2.06 | -4.44385 | -0.193 |
| ZNF428 | ILMN_1652754 | 0.00972 | 0.00101 | -3.47 | -1.06459 | -0.194 |
| MLH1 | ILMN_1788363 | 0.0675 | 0.0156 | -2.5 | -3.53884 | -0.194 |
| CHERP | ILMN_1798083 | 0.0809 | 0.0202 | -2.39 | -3.7676 | -0.194 |
| SIAH1 | ILMN_2380566 | 0.0834 | 0.0212 | -2.37 | -3.80745 | -0.194 |
| PFKFB4 | ILMN_1653292 | 0.125 | 0.0382 | -2.12 | -4.31681 | -0.194 |
| SEC11A | ILMN_1693490 | 0.0413 | 0.00766 | -2.77 | -2.90819 | -0.195 |
| RING1 | ILMN_1666399 | 0.046 | 0.00888 | -2.71 | -3.041 | -0.195 |
| AIP | ILMN_2103841 | 0.0795 | 0.0197 | -2.4 | -3.74605 | -0.195 |
| RSRC2 | ILMN_2358278 | 0.112 | 0.0325 | -2.19 | -4.17938 | -0.195 |
| GDPD3 | ILMN_1774901 | 0.13 | 0.0408 | -2.09 | -4.37205 | -0.195 |
| TMEM123 | ILMN_1724139 | 0.147 | 0.0486 | -2.02 | -4.52012 | -0.195 |
| C2orf49 | ILMN_1701131 | 0.00391 | 0.000278 | -3.88 | 0.13727 | -0.196 |
| MCRS1 | ILMN_1784227 | 0.0293 | 0.00471 | -2.95 | -2.47101 | -0.196 |
| KTN1 | ILMN_2339202 | 0.0295 | 0.00476 | -2.94 | -2.47918 | -0.196 |
| RAVER1 | ILMN_1740395 | 0.0443 | 0.00843 | -2.73 | -2.99367 | -0.196 |
| VASP | ILMN_1743646 | 0.0481 | 0.00953 | -2.69 | -3.10342 | -0.196 |
| EFTUD2 | ILMN_1738819 | 0.0749 | 0.0181 | -2.44 | -3.6705 | -0.196 |
| TGFBR2 | ILMN_2384241 | 0.0828 | 0.0209 | -2.38 | -3.79766 | -0.196 |
| ZNF613 | ILMN_1671895 | 0.0889 | 0.0233 | -2.33 | -3.89072 | -0.196 |
| GABPA | ILMN_1750029 | 0.0952 | 0.0255 | -2.3 | -3.97079 | -0.196 |
| EPC1 | ILMN_1746276 | 0.135 | 0.0429 | -2.07 | -4.41486 | -0.196 |
| STRN4 | ILMN_1696190 | 0.138 | 0.0444 | -2.06 | -4.44372 | -0.196 |
| LIMS1 | ILMN_1733176 | 0.15 | 0.0499 | -2 | -4.54249 | -0.196 |
| AP2S1 | ILMN_1809957 | 0.00521 | 0.00042 | -3.75 | -0.24814 | -0.197 |
| FAM46C | ILMN_1713266 | 0.0196 | 0.00269 | -3.14 | -1.96061 | -0.197 |
| DDX47 | ILMN_1814859 | 0.0422 | 0.00789 | -2.76 | -2.93457 | -0.197 |
| ZCCHC7 | ILMN_1744980 | 0.0534 | 0.0111 | -2.63 | -3.24281 | -0.197 |
| RAB3IP | ILMN_1803197 | 0.0548 | 0.0116 | -2.61 | -3.2747 | -0.197 |
| MAP4K2 | ILMN_1723625 | 0.0925 | 0.0245 | -2.31 | -3.93621 | -0.197 |
| PDS5B | ILMN_1675695 | 0.123 | 0.0371 | -2.14 | -4.29228 | -0.197 |
| RSRC2 | ILMN_2358277 | 0.0129 | 0.00148 | -3.35 | -1.41254 | -0.198 |
| SNRPG | ILMN_1683562 | 0.0158 | 0.00197 | -3.25 | -1.67728 | -0.198 |
| CCDC59 | ILMN_1662318 | 0.0201 | 0.00278 | -3.13 | -1.99144 | -0.198 |
| MIS12 | ILMN_1718069 | 0.0243 | 0.00366 | -3.03 | -2.24201 | -0.198 |
| SMARCA5 | ILMN_2223130 | 0.0547 | 0.0115 | -2.61 | -3.27262 | -0.198 |
| TGFBR3 | ILMN_1784287 | 0.0761 | 0.0185 | -2.43 | -3.68983 | -0.198 |
| NSUN3 | ILMN_1676280 | 0.0789 | 0.0195 | -2.41 | -3.73653 | -0.198 |
| SENP5 | ILMN_3251634 | 0.0874 | 0.0227 | -2.34 | -3.86812 | -0.198 |
| MBD1 | ILMN_2352580 | 0.0898 | 0.0236 | -2.33 | -3.90077 | -0.198 |
| DHX29 | ILMN_2090123 | 0.102 | 0.0282 | -2.25 | -4.05661 | -0.198 |
| PPP1R11 | ILMN_1747598 | 0.0271 | 0.00423 | -2.98 | -2.37238 | -0.199 |
| WSB1 | ILMN_2415748 | 0.0307 | 0.00502 | -2.92 | -2.52893 | -0.199 |
| RNF4 | ILMN_2142695 | 0.0341 | 0.00585 | -2.87 | -2.66653 | -0.199 |
| TUFM | ILMN_1738369 | 0.0463 | 0.00897 | -2.71 | -3.04946 | -0.199 |
| RBM15 | ILMN_1666739 | 0.0686 | 0.016 | -2.49 | -3.56016 | -0.199 |
| TARS | ILMN_1685480 | 0.0693 | 0.0162 | -2.48 | -3.57386 | -0.199 |
| PDLIM7 | ILMN_1814985 | 0.0715 | 0.0169 | -2.46 | -3.61028 | -0.199 |
| CYB561D1 | ILMN_3180557 | 0.134 | 0.0422 | -2.08 | -4.40004 | -0.199 |
| FOXN3 | ILMN_2106902 | 0.149 | 0.0494 | -2.01 | -4.53421 | -0.199 |
| PPHLN1 | ILMN_1791093 | 0.00742 | 0.000694 | -3.59 | -0.71489 | -0.2 |
| CGRRF1 | ILMN_1681008 | 0.0615 | 0.0136 | -2.55 | -3.41807 | -0.2 |
| C10orf76 | ILMN_1792110 | 0.0717 | 0.017 | -2.46 | -3.61347 | -0.2 |
| NMD3 | ILMN_2232430 | 0.0765 | 0.0187 | -2.42 | -3.69844 | -0.2 |
| ZNHIT3 | ILMN_1812478 | 0.0812 | 0.0203 | -2.39 | -3.77258 | -0.2 |
| PSME1 | ILMN_1806017 | 0.0883 | 0.0231 | -2.34 | -3.88237 | -0.2 |
| ELOVL5 | ILMN_2174369 | 0.102 | 0.0282 | -2.25 | -4.05784 | -0.2 |
| MARS | ILMN_1799819 | 0.00237 | 0.000144 | -4.08 | 0.75334 | -0.201 |
| RAB21 | ILMN_1746856 | 0.00296 | 0.000196 | -3.99 | 0.465 | -0.201 |
| ZNF384 | ILMN_1764891 | 0.0159 | 0.00199 | -3.24 | -1.68785 | -0.201 |
| FBXO18 | ILMN_1729430 | 0.0167 | 0.00213 | -3.22 | -1.74805 | -0.201 |
| THAP1 | ILMN_1757272 | 0.022 | 0.00318 | -3.08 | -2.11291 | -0.201 |
| LAMTOR3 | ILMN_1659415 | 0.038 | 0.0068 | -2.81 | -2.80174 | -0.201 |
| USP7 | ILMN_1729319 | 0.0521 | 0.0107 | -2.64 | -3.20698 | -0.201 |
| KAT2A | ILMN_1782247 | 0.128 | 0.0398 | -2.11 | -4.35103 | -0.201 |
| TRIM41 | ILMN_1729495 | 0.00683 | 0.000617 | -3.63 | -0.60573 | -0.202 |
| ING2 | ILMN_1671265 | 0.0218 | 0.00313 | -3.09 | -2.09824 | -0.202 |
| G3BP2 | ILMN_1720422 | 0.0235 | 0.00348 | -3.05 | -2.19715 | -0.202 |
| PSMD10 | ILMN_1776102 | 0.0324 | 0.00543 | -2.89 | -2.59942 | -0.202 |
| SETD3 | ILMN_1724504 | 0.0461 | 0.00892 | -2.71 | -3.04479 | -0.202 |
| OMA1 | ILMN_1670079 | 0.0526 | 0.0109 | -2.63 | -3.22286 | -0.202 |
| CLEC16A | ILMN_1781752 | 0.0619 | 0.0137 | -2.54 | -3.4289 | -0.202 |
| PTS | ILMN_1720322 | 0.0717 | 0.017 | -2.46 | -3.61355 | -0.202 |
| TMUB1 | ILMN_1666050 | 0.0925 | 0.0245 | -2.31 | -3.93598 | -0.202 |
| KRT23 | ILMN_1791545 | 0.146 | 0.048 | -2.02 | -4.51009 | -0.202 |
| GPATCH2L | ILMN_2265093 | 0.146 | 0.0481 | -2.02 | -4.51169 | -0.202 |
| WIPI1 | ILMN_1781386 | 0.147 | 0.0484 | -2.02 | -4.51601 | -0.202 |
| ATP5O | ILMN_1791332 | 0.00175 | 9.54E-05 | -4.21 | 1.13874 | -0.203 |
| FOXJ3 | ILMN_2128668 | 0.00385 | 0.000272 | -3.89 | 0.15529 | -0.203 |
| DERL1 | ILMN_2209748 | 0.00496 | 0.000394 | -3.77 | -0.1881 | -0.203 |
| C1orf174 | ILMN_3236244 | 0.0213 | 0.00303 | -3.1 | -2.06858 | -0.203 |
| HMGN1 | ILMN_2151579 | 0.0659 | 0.0151 | -2.51 | -3.51175 | -0.203 |
| WDR20 | ILMN_2409706 | 0.0807 | 0.0202 | -2.39 | -3.76551 | -0.203 |
| FKBP1A | ILMN_2333367 | 0.0974 | 0.0265 | -2.28 | -4.00317 | -0.203 |
| SETD1B | ILMN_1813573 | 0.106 | 0.03 | -2.23 | -4.10856 | -0.203 |
| UQCR11 | ILMN_1745049 | 0.122 | 0.0371 | -2.14 | -4.29106 | -0.203 |
| RFWD2 | ILMN_1661002 | 0.0141 | 0.00167 | -3.3 | -1.52771 | -0.204 |
| KLHL5 | ILMN_1706687 | 0.024 | 0.00358 | -3.04 | -2.22272 | -0.204 |
| LOC606724 | ILMN_2125747 | 0.0632 | 0.0141 | -2.53 | -3.45419 | -0.204 |
| LIAS | ILMN_2393341 | 0.0687 | 0.016 | -2.49 | -3.56237 | -0.204 |
| SGSM2 | ILMN_1779171 | 0.0728 | 0.0174 | -2.45 | -3.63516 | -0.204 |
| GLRX5 | ILMN_1796165 | 0.0967 | 0.0262 | -2.28 | -3.99236 | -0.204 |
| CERS2 | ILMN_2400500 | 0.12 | 0.0359 | -2.15 | -4.26454 | -0.204 |
| PTK2 | ILMN_1714364 | 0.142 | 0.046 | -2.04 | -4.47307 | -0.204 |
| HMGCS1 | ILMN_1797728 | 0.143 | 0.0463 | -2.04 | -4.47922 | -0.204 |
| BUB3 | ILMN_1778764 | 0.149 | 0.0497 | -2.01 | -4.53846 | -0.204 |
| TMEM167A | ILMN_3251560 | 0.0039 | 0.000277 | -3.88 | 0.14057 | -0.205 |
| FAM214B | ILMN_1732609 | 0.00555 | 0.000456 | -3.73 | -0.32467 | -0.205 |
| RHOT2 | ILMN_1669310 | 0.0309 | 0.00506 | -2.92 | -2.53448 | -0.205 |
| MYO1F | ILMN_1681239 | 0.0488 | 0.00976 | -2.68 | -3.12453 | -0.205 |
| ZNF263 | ILMN_1692620 | 0.0716 | 0.0169 | -2.46 | -3.61167 | -0.205 |
| SLK | ILMN_2159322 | 0.0935 | 0.0249 | -2.31 | -3.94926 | -0.205 |
| RLF | ILMN_1801156 | 0.107 | 0.0306 | -2.22 | -4.12591 | -0.205 |
| RAB24 | ILMN_1714393 | 0.109 | 0.0312 | -2.21 | -4.14469 | -0.205 |
| C19orf38 | ILMN_1728392 | 0.149 | 0.0494 | -2.01 | -4.5342 | -0.205 |
| PPP4R3B | ILMN_1661650 | 0.000501 | 1.80E-05 | -4.69 | 2.7137 | -0.206 |
| DNAJB12 | ILMN_2333865 | 0.0301 | 0.00489 | -2.93 | -2.50389 | -0.206 |
| PEX13 | ILMN_1683916 | 0.0496 | 0.01 | -2.67 | -3.14881 | -0.206 |
| SIAH1 | ILMN_1711627 | 0.054 | 0.0113 | -2.62 | -3.25731 | -0.206 |
| TRAPPC12 | ILMN_1693317 | 0.0575 | 0.0124 | -2.59 | -3.33598 | -0.206 |
| MFSD8 | ILMN_2123119 | 0.08 | 0.0199 | -2.4 | -3.75388 | -0.206 |
| GPAA1 | ILMN_1769702 | 0.0944 | 0.0253 | -2.3 | -3.96149 | -0.206 |
| DNAL4 | ILMN_1801845 | 0.0048 | 0.000375 | -3.79 | -0.14185 | -0.207 |
| TLE4 | ILMN_2103761 | 0.0328 | 0.00553 | -2.89 | -2.615 | -0.207 |
| STAG1 | ILMN_2151048 | 0.0497 | 0.0101 | -2.67 | -3.15173 | -0.207 |
| SQSTM1 | ILMN_1662618 | 0.0801 | 0.02 | -2.4 | -3.75643 | -0.207 |
| LIAS | ILMN_1736077 | 0.0986 | 0.027 | -2.27 | -4.019 | -0.207 |
| TSPYL2 | ILMN_2347298 | 0.107 | 0.0305 | -2.22 | -4.12477 | -0.207 |
| NUDC | ILMN_2097546 | 0.022 | 0.00317 | -3.09 | -2.11196 | -0.208 |
| SH2D3C | ILMN_1775734 | 0.0285 | 0.00455 | -2.96 | -2.43837 | -0.208 |
| LPAR2 | ILMN_3236220 | 0.0365 | 0.00643 | -2.83 | -2.75128 | -0.208 |
| PPP2R5E | ILMN_1666761 | 0.0402 | 0.00735 | -2.78 | -2.87155 | -0.208 |
| RNASEH1 | ILMN_1726783 | 0.0473 | 0.00927 | -2.7 | -3.07911 | -0.208 |
| SEC22C | ILMN_2290618 | 0.0475 | 0.00933 | -2.69 | -3.08495 | -0.208 |
| RPS6KB2 | ILMN_1761175 | 0.0523 | 0.0108 | -2.64 | -3.21602 | -0.208 |
| UGP2 | ILMN_2389155 | 0.072 | 0.0171 | -2.46 | -3.6201 | -0.208 |
| STYX | ILMN_1697024 | 0.01 | 0.00106 | -3.46 | -1.10278 | -0.209 |
| MAPK3 | ILMN_2402341 | 0.0183 | 0.00241 | -3.18 | -1.86239 | -0.209 |
| NXF1 | ILMN_1705783 | 0.0434 | 0.00819 | -2.74 | -2.9688 | -0.209 |
| CNPY2 | ILMN_3243471 | 0.0552 | 0.0117 | -2.61 | -3.28378 | -0.209 |
| RAB28 | ILMN_2293992 | 0.121 | 0.0365 | -2.14 | -4.277 | -0.209 |
| PHKA2 | ILMN_1814074 | 0.127 | 0.0391 | -2.11 | -4.33632 | -0.209 |
| STK38L | ILMN_1755792 | 0.142 | 0.0459 | -2.04 | -4.47187 | -0.209 |
| CHD4 | ILMN_1658411 | 0.0293 | 0.0047 | -2.95 | -2.46881 | -0.21 |
| TAF15 | ILMN_2402131 | 0.0316 | 0.00524 | -2.91 | -2.56705 | -0.21 |
| ARHGEF1 | ILMN_1772370 | 0.0452 | 0.00866 | -2.72 | -3.01804 | -0.21 |
| CORO1A | ILMN_1713749 | 0.0612 | 0.0135 | -2.55 | -3.41176 | -0.21 |
| MIB1 | ILMN_1788832 | 0.0739 | 0.0178 | -2.44 | -3.65432 | -0.21 |
| PMPCA | ILMN_1764239 | 0.11 | 0.0318 | -2.2 | -4.16096 | -0.21 |
| PIP5K1A | ILMN_1769869 | 0.135 | 0.0427 | -2.07 | -4.41182 | -0.21 |
| HIKESHI | ILMN_1656463 | 0.142 | 0.0459 | -2.04 | -4.47216 | -0.21 |
| SHFM1 | ILMN_1794505 | 0.145 | 0.0477 | -2.02 | -4.5052 | -0.21 |
| CASZ1 | ILMN_1655191 | 0.147 | 0.0488 | -2.02 | -4.52273 | -0.21 |
| CWC15 | ILMN_1713482 | 0.00727 | 0.000675 | -3.6 | -0.6893 | -0.211 |
| LINC00623 | ILMN_3234142 | 0.0362 | 0.00638 | -2.84 | -2.74448 | -0.211 |
| CRBN | ILMN_2225735 | 0.0434 | 0.00817 | -2.74 | -2.96648 | -0.211 |
| SURF4 | ILMN_1690761 | 0.0466 | 0.00907 | -2.7 | -3.0595 | -0.211 |
| SYNRG | ILMN_2415467 | 0.0493 | 0.00994 | -2.67 | -3.14111 | -0.211 |
| SP4 | ILMN_1721081 | 0.0697 | 0.0164 | -2.48 | -3.58172 | -0.211 |
| UPF2 | ILMN_1796305 | 0.0834 | 0.0212 | -2.37 | -3.80819 | -0.211 |
| PDS5B | ILMN_1746696 | 0.0886 | 0.0232 | -2.34 | -3.88612 | -0.211 |
| KLHL5 | ILMN_2342437 | 0.105 | 0.0294 | -2.24 | -4.09104 | -0.211 |
| SSH3 | ILMN_1755234 | 0.118 | 0.0353 | -2.16 | -4.24998 | -0.211 |
| DPH2 | ILMN_2375418 | 0.12 | 0.036 | -2.15 | -4.26687 | -0.211 |
| ZNF189 | ILMN_1806809 | 0.0013 | 6.30E-05 | -4.33 | 1.52791 | -0.212 |
| ATG4B | ILMN_2411915 | 0.00403 | 0.000292 | -3.87 | 0.09135 | -0.212 |
| NDUFB5 | ILMN_1807397 | 0.0122 | 0.00137 | -3.37 | -1.34126 | -0.212 |
| UBXN6 | ILMN_1658624 | 0.0135 | 0.00158 | -3.32 | -1.4769 | -0.212 |
| PRKAR1A | ILMN_1738632 | 0.0194 | 0.00265 | -3.15 | -1.94693 | -0.212 |
| NDUFS5 | ILMN_1776104 | 0.0359 | 0.00628 | -2.84 | -2.73039 | -0.212 |
| KRIT1 | ILMN_2323418 | 0.069 | 0.0161 | -2.48 | -3.56823 | -0.212 |
| EHD1 | ILMN_1651832 | 0.102 | 0.0282 | -2.25 | -4.05701 | -0.212 |
| PHACTR2 | ILMN_1790533 | 0.111 | 0.032 | -2.2 | -4.16514 | -0.212 |
| CCNT2 | ILMN_2357682 | 0.114 | 0.0337 | -2.18 | -4.20897 | -0.212 |
| NDE1 | ILMN_1739805 | 0.115 | 0.0338 | -2.18 | -4.213 | -0.212 |
| KIF22 | ILMN_3234884 | 0.132 | 0.0416 | -2.09 | -4.38844 | -0.212 |
| MOB1A | ILMN_3242011 | 0.00675 | 0.000609 | -3.64 | -0.59328 | -0.213 |
| MRPL21 | ILMN_1744835 | 0.0221 | 0.00321 | -3.08 | -2.12233 | -0.213 |
| SECISBP2L | ILMN_1784333 | 0.0541 | 0.0114 | -2.62 | -3.2597 | -0.213 |
| RCOR3 | ILMN_1682095 | 0.0774 | 0.019 | -2.42 | -3.71236 | -0.213 |
| DIMT1 | ILMN_1803312 | 0.105 | 0.0297 | -2.23 | -4.10166 | -0.213 |
| FBXO8 | ILMN_1672843 | 0.00852 | 0.000836 | -3.53 | -0.8874 | -0.214 |
| MRPL43 | ILMN_2258774 | 0.0315 | 0.00522 | -2.91 | -2.56389 | -0.214 |
| DAP3 | ILMN_1781680 | 0.0412 | 0.00762 | -2.77 | -2.90401 | -0.214 |
| SH3KBP1 | ILMN_1808501 | 0.0552 | 0.0117 | -2.61 | -3.28641 | -0.214 |
| MALT1 | ILMN_1730986 | 0.0694 | 0.0162 | -2.48 | -3.57612 | -0.214 |
| RWDD4 | ILMN_1708660 | 0.0903 | 0.0238 | -2.32 | -3.90853 | -0.214 |
| MRPS28 | ILMN_1718424 | 0.112 | 0.0327 | -2.19 | -4.18483 | -0.214 |
| F2RL1 | ILMN_2041190 | 0.127 | 0.0388 | -2.12 | -4.33045 | -0.214 |
| C20orf24 | ILMN_2283388 | 0.14 | 0.0451 | -2.05 | -4.45772 | -0.214 |
| RGS19 | ILMN_1677085 | 0.00383 | 0.00027 | -3.89 | 0.16334 | -0.215 |
| AP2S1 | ILMN_1662426 | 0.00539 | 0.00044 | -3.74 | -0.29135 | -0.215 |
| CXCR4 | ILMN_2320888 | 0.0207 | 0.00289 | -3.12 | -2.02759 | -0.215 |
| PI4KB | ILMN_1666597 | 0.0244 | 0.00369 | -3.03 | -2.25009 | -0.215 |
| ING2 | ILMN_2085722 | 0.0252 | 0.00384 | -3.02 | -2.28553 | -0.215 |
| CD47 | ILMN_1771333 | 0.0263 | 0.00406 | -3 | -2.33595 | -0.215 |
| KIAA0355 | ILMN_1659845 | 0.0552 | 0.0117 | -2.61 | -3.2857 | -0.215 |
| MKKS | ILMN_1718718 | 0.075 | 0.0181 | -2.43 | -3.67307 | -0.215 |
| TMEM214 | ILMN_1716907 | 0.0818 | 0.0206 | -2.38 | -3.7823 | -0.215 |
| SMIM14 | ILMN_1713892 | 0.0895 | 0.0235 | -2.33 | -3.89679 | -0.215 |
| CD300A | ILMN_1693552 | 0.0962 | 0.0259 | -2.29 | -3.98393 | -0.215 |
| CMIP | ILMN_1767182 | 0.144 | 0.0471 | -2.03 | -4.49377 | -0.215 |
| SCYL2 | ILMN_1711919 | 6.72E-05 | 1.00E-06 | -5.5 | 5.4549 | -0.216 |
| TTC32 | ILMN_2083588 | 0.0588 | 0.0128 | -2.57 | -3.36223 | -0.216 |
| DUSP23 | ILMN_1659462 | 0.0643 | 0.0146 | -2.52 | -3.47971 | -0.216 |
| CCNY | ILMN_2261784 | 0.0667 | 0.0153 | -2.5 | -3.52577 | -0.216 |
| RAB8A | ILMN_1760858 | 0.0964 | 0.0261 | -2.29 | -3.98835 | -0.216 |
| HIKESHI | ILMN_1781419 | 0.104 | 0.0291 | -2.24 | -4.08479 | -0.216 |
| GPC2 | ILMN_1651642 | 0.108 | 0.0308 | -2.22 | -4.13095 | -0.216 |
| SPATA5L1 | ILMN_1729179 | 0.00155 | 8.19E-05 | -4.25 | 1.28206 | -0.217 |
| GTF2F2 | ILMN_1745798 | 0.00685 | 0.000619 | -3.63 | -0.6091 | -0.217 |
| FEM1C | ILMN_1703330 | 0.0147 | 0.00177 | -3.28 | -1.58087 | -0.217 |
| LRRC8B | ILMN_1838313 | 0.0301 | 0.00488 | -2.93 | -2.50235 | -0.217 |
| ACO2 | ILMN_1654861 | 0.0596 | 0.013 | -2.57 | -3.38253 | -0.217 |
| SRSF6 | ILMN_1697469 | 0.0598 | 0.0131 | -2.56 | -3.38554 | -0.217 |
| CDV3 | ILMN_1673788 | 0.0652 | 0.0149 | -2.51 | -3.49907 | -0.217 |
| EIF3J | ILMN_1815345 | 0.0967 | 0.0262 | -2.28 | -3.99248 | -0.217 |
| MAPK1 | ILMN_2235283 | 0.109 | 0.0311 | -2.21 | -4.14096 | -0.217 |
| NEMF | ILMN_1772489 | 0.0199 | 0.00274 | -3.14 | -1.97786 | -0.218 |
| MAT2B | ILMN_1811367 | 0.0407 | 0.0075 | -2.78 | -2.88946 | -0.218 |
| SENP7 | ILMN_2382354 | 0.041 | 0.00756 | -2.77 | -2.89657 | -0.218 |
| PPRC1 | ILMN_1796210 | 0.135 | 0.0429 | -2.07 | -4.41505 | -0.218 |
| SEC61B | ILMN_1801852 | 0.000565 | 2.09E-05 | -4.65 | 2.56788 | -0.219 |
| DDX18 | ILMN_1756220 | 0.0248 | 0.00378 | -3.02 | -2.271 | -0.219 |
| WDR45B | ILMN_1685763 | 0.0272 | 0.00427 | -2.98 | -2.38091 | -0.219 |
| AP5M1 | ILMN_3237641 | 0.04 | 0.00729 | -2.79 | -2.86444 | -0.219 |
| CYFIP2 | ILMN_1677200 | 0.064 | 0.0144 | -2.53 | -3.47211 | -0.219 |
| SCAND2P | ILMN_1713746 | 0.069 | 0.0161 | -2.48 | -3.568 | -0.219 |
| FRS2 | ILMN_1911605 | 0.0953 | 0.0256 | -2.29 | -3.97276 | -0.219 |
| EED | ILMN_2347917 | 0.0959 | 0.0258 | -2.29 | -3.98065 | -0.219 |
| EDF1 | ILMN_1726169 | 0.00248 | 0.000154 | -4.06 | 0.69032 | -0.22 |
| UBE4A | ILMN_1739259 | 0.00394 | 0.000281 | -3.88 | 0.12759 | -0.22 |
| SUZ12 | ILMN_1797813 | 0.0102 | 0.00108 | -3.45 | -1.12043 | -0.22 |
| SIRT2 | ILMN_1723494 | 0.0447 | 0.00854 | -2.73 | -3.0053 | -0.22 |
| UBE2V1 | ILMN_1726107 | 0.0547 | 0.0115 | -2.61 | -3.27321 | -0.22 |
| PDLIM7 | ILMN_2396639 | 0.0814 | 0.0204 | -2.39 | -3.77624 | -0.22 |
| RASSF5 | ILMN_1813795 | 0.0829 | 0.0209 | -2.38 | -3.79793 | -0.22 |
| MTFR1L | ILMN_3264466 | 0.0975 | 0.0266 | -2.28 | -4.00562 | -0.22 |
| ZBTB2 | ILMN_1766247 | 0.109 | 0.0313 | -2.21 | -4.14722 | -0.22 |
| NDUFA9 | ILMN_1760741 | 0.132 | 0.0415 | -2.09 | -4.38682 | -0.22 |
| AKT1 | ILMN_2388507 | 0.135 | 0.0429 | -2.07 | -4.4139 | -0.22 |
| SSR2 | ILMN_1785037 | 0.0155 | 0.00191 | -3.26 | -1.6495 | -0.221 |
| TIAL1 | ILMN_1717745 | 0.0169 | 0.00218 | -3.21 | -1.76842 | -0.221 |
| ZBTB4 | ILMN_2097793 | 0.0185 | 0.00245 | -3.17 | -1.87578 | -0.221 |
| TOP2B | ILMN_1777663 | 0.0592 | 0.0129 | -2.57 | -3.37328 | -0.221 |
| RPAIN | ILMN_1770339 | 0.0629 | 0.0141 | -2.54 | -3.44832 | -0.221 |
| PPP1R7 | ILMN_1808333 | 0.0645 | 0.0146 | -2.52 | -3.48463 | -0.221 |
| REV1 | ILMN_1707062 | 0.077 | 0.0189 | -2.42 | -3.70645 | -0.221 |
| VPS4A | ILMN_1708946 | 0.109 | 0.0311 | -2.21 | -4.14135 | -0.221 |
| SPAST | ILMN_2373556 | 0.144 | 0.0469 | -2.03 | -4.48942 | -0.221 |
| USO1 | ILMN_1692121 | 0.00114 | 5.33E-05 | -4.38 | 1.68594 | -0.222 |
| KDM2A | ILMN_3237966 | 0.00212 | 0.000124 | -4.13 | 0.89462 | -0.222 |
| STAMBP | ILMN_2391355 | 0.00808 | 0.000781 | -3.56 | -0.82404 | -0.222 |
| C5orf15 | ILMN_1695917 | 0.0153 | 0.00189 | -3.26 | -1.63917 | -0.222 |
| SMUG1 | ILMN_1804642 | 0.0159 | 0.00199 | -3.25 | -1.68666 | -0.222 |
| CRELD2 | ILMN_1748707 | 0.0166 | 0.00211 | -3.23 | -1.74073 | -0.222 |
| COX4I1 | ILMN_1652207 | 0.0265 | 0.0041 | -2.99 | -2.34508 | -0.222 |
| ZFP36L1 | ILMN_1675448 | 0.0624 | 0.0139 | -2.54 | -3.43897 | -0.222 |
| CEP83 | ILMN_1799113 | 0.0634 | 0.0142 | -2.53 | -3.45915 | -0.222 |
| PDXDC1 | ILMN_3243705 | 0.0852 | 0.0218 | -2.36 | -3.83436 | -0.222 |
| PAM16 | ILMN_1763884 | 0.0853 | 0.0219 | -2.36 | -3.83668 | -0.222 |
| MRPL42 | ILMN_2356895 | 0.108 | 0.0307 | -2.22 | -4.13061 | -0.222 |
| PPP1R8 | ILMN_2321485 | 0.134 | 0.0425 | -2.08 | -4.40667 | -0.222 |
| RBCK1 | ILMN_2406313 | 0.139 | 0.0448 | -2.05 | -4.45085 | -0.222 |
| GTF2IP1 | ILMN_1815668 | 0.15 | 0.0498 | -2.01 | -4.54091 | -0.222 |
| ZNF274 | ILMN_2352574 | 0.0112 | 0.00122 | -3.41 | -1.23817 | -0.223 |
| FERMT3 | ILMN_2366330 | 0.047 | 0.00919 | -2.7 | -3.07124 | -0.223 |
| BOLA2 | ILMN_1782633 | 0.0825 | 0.0208 | -2.38 | -3.79253 | -0.223 |
| CNPY2 | ILMN_2123402 | 0.0906 | 0.0238 | -2.32 | -3.91126 | -0.223 |
| DDHD2 | ILMN_1705871 | 0.0943 | 0.0252 | -2.3 | -3.95934 | -0.223 |
| ZC3H12A | ILMN_1672295 | 0.112 | 0.0326 | -2.19 | -4.18076 | -0.223 |
| TRIM39 | ILMN_1736562 | 0.00885 | 0.000884 | -3.52 | -0.93892 | -0.224 |
| SQRDL | ILMN_1667199 | 0.0109 | 0.00117 | -3.42 | -1.20024 | -0.224 |
| SYS1 | ILMN_1756590 | 0.026 | 0.004 | -3 | -2.32267 | -0.224 |
| OTUD5 | ILMN_2088847 | 0.043 | 0.00808 | -2.75 | -2.95667 | -0.224 |
| CKAP4 | ILMN_1790891 | 0.0477 | 0.00938 | -2.69 | -3.08951 | -0.224 |
| ZNF211 | ILMN_1811195 | 0.0615 | 0.0136 | -2.55 | -3.4193 | -0.224 |
| CHORDC1 | ILMN_1776337 | 0.0724 | 0.0173 | -2.45 | -3.62947 | -0.224 |
| S1PR1 | ILMN_1653504 | 0.0746 | 0.018 | -2.44 | -3.6652 | -0.224 |
| SRSF10 | ILMN_1677162 | 0.0926 | 0.0246 | -2.31 | -3.93721 | -0.224 |
| ZFP91 | ILMN_1665423 | 0.00667 | 0.000597 | -3.64 | -0.57533 | -0.225 |
| RNF20 | ILMN_1710758 | 0.0289 | 0.00462 | -2.95 | -2.45219 | -0.225 |
| RBPJ | ILMN_2316844 | 0.0584 | 0.0126 | -2.58 | -3.35396 | -0.225 |
| SSFA2 | ILMN_1742260 | 0.064 | 0.0144 | -2.53 | -3.47115 | -0.225 |
| WDR75 | ILMN_1801869 | 0.085 | 0.0218 | -2.36 | -3.83207 | -0.225 |
| KIAA1429 | ILMN_1741841 | 0.1 | 0.0277 | -2.26 | -4.03951 | -0.225 |
| LINC00116 | ILMN_1693685 | 0.115 | 0.0339 | -2.18 | -4.21423 | -0.225 |
| SDHD | ILMN_1698487 | 0.000872 | 3.74E-05 | -4.48 | 2.01984 | -0.226 |
| SYNCRIP | ILMN_1727740 | 0.00272 | 0.000173 | -4.03 | 0.58027 | -0.226 |
| PPHLN1 | ILMN_2322842 | 0.00672 | 0.000604 | -3.64 | -0.58584 | -0.226 |
| YRDC | ILMN_2061732 | 0.00781 | 0.000744 | -3.57 | -0.77867 | -0.226 |
| QKI | ILMN_1773485 | 0.0142 | 0.00169 | -3.3 | -1.53685 | -0.226 |
| SUPT16H | ILMN_1781516 | 0.0271 | 0.00422 | -2.98 | -2.37107 | -0.226 |
| ARFIP1 | ILMN_2403906 | 0.0321 | 0.00536 | -2.9 | -2.58772 | -0.226 |
| FKBP1A | ILMN_1683658 | 0.0368 | 0.00652 | -2.83 | -2.76374 | -0.226 |
| CDV3 | ILMN_1810977 | 0.038 | 0.00681 | -2.81 | -2.80238 | -0.226 |
| EIF1AX | ILMN_1813240 | 0.0828 | 0.0209 | -2.38 | -3.7968 | -0.226 |
| JUN | ILMN_1806023 | 0.0931 | 0.0247 | -2.31 | -3.94289 | -0.226 |
| TCEANC2 | ILMN_1673544 | 0.113 | 0.0332 | -2.18 | -4.19701 | -0.226 |
| DAGLB | ILMN_1658885 | 0.126 | 0.0384 | -2.12 | -4.32128 | -0.226 |
| PRELID3B | ILMN_1728168 | 0.000369 | 1.14E-05 | -4.82 | 3.1403 | -0.227 |
| CS | ILMN_1706057 | 0.00233 | 0.000141 | -4.09 | 0.77265 | -0.227 |
| GUK1 | ILMN_1758398 | 0.00391 | 0.000277 | -3.88 | 0.1393 | -0.227 |
| DCTN5 | ILMN_1666192 | 0.00711 | 0.000652 | -3.61 | -0.65687 | -0.227 |
| SAP130 | ILMN_1700044 | 0.0518 | 0.0106 | -2.65 | -3.19865 | -0.227 |
| COTL1 | ILMN_1788283 | 0.0571 | 0.0122 | -2.59 | -3.32665 | -0.227 |
| ADGRE3 | ILMN_1755873 | 0.0633 | 0.0142 | -2.53 | -3.45614 | -0.227 |
| TFEB | ILMN_1733616 | 0.0713 | 0.0168 | -2.46 | -3.60752 | -0.227 |
| TCF20 | ILMN_1660144 | 0.0952 | 0.0256 | -2.29 | -3.97121 | -0.227 |
| C19orf25 | ILMN_1655225 | 0.0977 | 0.0267 | -2.28 | -4.00762 | -0.227 |
| SDF2 | ILMN_1713978 | 0.00181 | 1.00E-04 | -4.19 | 1.09233 | -0.228 |
| MRPS18C | ILMN_1658416 | 0.00194 | 0.000109 | -4.17 | 1.01004 | -0.228 |
| VEZF1 | ILMN_1705310 | 0.00421 | 0.00031 | -3.85 | 0.03368 | -0.228 |
| TAOK2 | ILMN_1701487 | 0.00634 | 0.000545 | -3.67 | -0.4914 | -0.228 |
| PAK2 | ILMN_1712687 | 0.00844 | 0.000826 | -3.54 | -0.87661 | -0.228 |
| CSNK1G1 | ILMN_1704713 | 0.0152 | 0.00187 | -3.27 | -1.62703 | -0.228 |
| LSM12 | ILMN_2092693 | 0.0316 | 0.00525 | -2.91 | -2.56777 | -0.228 |
| CARD19 | ILMN_1659189 | 0.0489 | 0.00977 | -2.68 | -3.12598 | -0.228 |
| ZNF106 | ILMN_2043265 | 0.0634 | 0.0142 | -2.53 | -3.45803 | -0.228 |
| SPATA2 | ILMN_1681135 | 0.0684 | 0.0159 | -2.49 | -3.55623 | -0.228 |
| HSPB11 | ILMN_1681340 | 0.113 | 0.0328 | -2.19 | -4.18652 | -0.228 |
| PSMB2 | ILMN_1764794 | 0.115 | 0.034 | -2.17 | -4.21751 | -0.228 |
| UBE2Z | ILMN_1743086 | 0.126 | 0.0387 | -2.12 | -4.32712 | -0.228 |
| PGK1 | ILMN_1755749 | 0.00226 | 0.000134 | -4.11 | 0.81968 | -0.229 |
| BAZ1A | ILMN_1742230 | 0.00578 | 0.00048 | -3.71 | -0.37331 | -0.229 |
| KIF2A | ILMN_1734476 | 0.00679 | 0.000613 | -3.63 | -0.59921 | -0.229 |
| RBBP5 | ILMN_1696532 | 0.0112 | 0.00122 | -3.41 | -1.2376 | -0.229 |
| SIN3A | ILMN_1805996 | 0.065 | 0.0148 | -2.52 | -3.49568 | -0.229 |
| NFIC | ILMN_1675130 | 0.0705 | 0.0166 | -2.47 | -3.59464 | -0.229 |
| PSME3 | ILMN_1800975 | 0.0939 | 0.025 | -2.3 | -3.95381 | -0.229 |
| BZW1 | ILMN_1704760 | 0.0954 | 0.0256 | -2.29 | -3.97414 | -0.229 |
| BABAM1 | ILMN_2396813 | 0.132 | 0.0415 | -2.09 | -4.38672 | -0.229 |
| STARD7 | ILMN_1740819 | 0.138 | 0.0441 | -2.06 | -4.43872 | -0.229 |
| GRB2 | ILMN_1748797 | 0.00161 | 8.60E-05 | -4.24 | 1.23545 | -0.23 |
| UBA3 | ILMN_2324157 | 0.0271 | 0.00424 | -2.98 | -2.37489 | -0.23 |
| NDUFC1 | ILMN_1733603 | 0.0344 | 0.00593 | -2.86 | -2.67883 | -0.23 |
| SH3KBP1 | ILMN_1810782 | 0.0485 | 0.00967 | -2.68 | -3.11657 | -0.23 |
| TGFBR2 | ILMN_1726245 | 0.0499 | 0.0101 | -2.66 | -3.15733 | -0.23 |
| PTK2B | ILMN_2330966 | 0.0744 | 0.0179 | -2.44 | -3.66187 | -0.23 |
| RNF123 | ILMN_1663605 | 0.075 | 0.0181 | -2.44 | -3.67243 | -0.23 |
| PPM1F | ILMN_2059535 | 0.0878 | 0.0229 | -2.34 | -3.87541 | -0.23 |
| GNLY | ILMN_1790692 | 0.00862 | 0.000855 | -3.53 | -0.90771 | -0.231 |
| AP5M1 | ILMN_2204297 | 0.0107 | 0.00115 | -3.43 | -1.17867 | -0.231 |
| PRPF18 | ILMN_1807243 | 0.0416 | 0.00773 | -2.76 | -2.91625 | -0.231 |
| LSM12 | ILMN_1734428 | 0.0482 | 0.00958 | -2.68 | -3.10801 | -0.231 |
| FKBP1A | ILMN_1683969 | 0.0495 | 0.00999 | -2.67 | -3.14572 | -0.231 |
| PFKL | ILMN_1713037 | 0.064 | 0.0144 | -2.53 | -3.47223 | -0.231 |
| PITPNM1 | ILMN_1653220 | 0.0796 | 0.0198 | -2.4 | -3.74781 | -0.231 |
| DBNDD2 | ILMN_1730612 | 0.00206 | 0.000119 | -4.14 | 0.93369 | -0.232 |
| CES2 | ILMN_1696675 | 0.00647 | 0.00057 | -3.66 | -0.53168 | -0.232 |
| REV1 | ILMN_2395474 | 0.0237 | 0.00353 | -3.05 | -2.20875 | -0.232 |
| DAP | ILMN_2112493 | 0.036 | 0.00631 | -2.84 | -2.73444 | -0.232 |
| DNAJB14 | ILMN_2415898 | 0.0507 | 0.0103 | -2.66 | -3.17442 | -0.232 |
| NME4 | ILMN_1800634 | 0.0667 | 0.0153 | -2.5 | -3.5247 | -0.232 |
| LZIC | ILMN_1661627 | 0.0718 | 0.017 | -2.46 | -3.61638 | -0.232 |
| CCDC94 | ILMN_1703430 | 0.0807 | 0.0202 | -2.39 | -3.76555 | -0.232 |
| CASP8 | ILMN_1673757 | 0.13 | 0.0404 | -2.1 | -4.36456 | -0.232 |
| SLC35E1 | ILMN_1752333 | 0.00557 | 0.000458 | -3.73 | -0.32883 | -0.233 |
| PRPF38A | ILMN_1675626 | 0.00689 | 0.000624 | -3.63 | -0.61642 | -0.233 |
| CD82 | ILMN_1662973 | 0.011 | 0.0012 | -3.42 | -1.21761 | -0.233 |
| CREB1 | ILMN_1841334 | 0.016 | 0.00201 | -3.24 | -1.6972 | -0.233 |
| YY1 | ILMN_1770892 | 0.0185 | 0.00246 | -3.17 | -1.87885 | -0.233 |
| ADPRM | ILMN_1702526 | 0.0325 | 0.00545 | -2.89 | -2.60256 | -0.233 |
| RBM6 | ILMN_1738239 | 0.0514 | 0.0105 | -2.65 | -3.18823 | -0.233 |
| CCS | ILMN_1766797 | 0.0687 | 0.016 | -2.49 | -3.56141 | -0.233 |
| DEFA4 | ILMN_1753347 | 0.0983 | 0.0269 | -2.27 | -4.01421 | -0.233 |
| ACAP2 | ILMN_2088825 | 0.00238 | 0.000145 | -4.08 | 0.7454 | -0.234 |
| RBM4 | ILMN_1712455 | 0.0098 | 0.00102 | -3.47 | -1.07501 | -0.234 |
| MRPL10 | ILMN_2396002 | 0.0156 | 0.00193 | -3.26 | -1.65911 | -0.234 |
| DPYD | ILMN_1795715 | 0.0835 | 0.0212 | -2.37 | -3.80985 | -0.234 |
| BZW1 | ILMN_1793846 | 0.111 | 0.032 | -2.2 | -4.16425 | -0.234 |
| ZNF561 | ILMN_1717049 | 0.135 | 0.0431 | -2.07 | -4.41945 | -0.234 |
| LOC153577 | ILMN_1899760 | 0.138 | 0.044 | -2.06 | -4.43638 | -0.234 |
| SNORA3B | ILMN_3238078 | 0.0494 | 0.00997 | -2.67 | -3.14422 | -0.235 |
| COMMD1 | ILMN_1761242 | 0.0649 | 0.0148 | -2.52 | -3.4933 | -0.235 |
| MRPS30 | ILMN_1726743 | 0.077 | 0.0188 | -2.42 | -3.7058 | -0.235 |
| MRPL24 | ILMN_1695576 | 0.0802 | 0.02 | -2.4 | -3.75679 | -0.235 |
| GSDMB | ILMN_1666206 | 0.0917 | 0.0242 | -2.32 | -3.92521 | -0.235 |
| NOA1 | ILMN_1665066 | 0.096 | 0.0259 | -2.29 | -3.98161 | -0.235 |
| SOD2 | ILMN_1792922 | 0.114 | 0.0336 | -2.18 | -4.20809 | -0.235 |
| PGM3 | ILMN_1693620 | 0.128 | 0.0398 | -2.11 | -4.35077 | -0.235 |
| C6orf136 | ILMN_1813236 | 0.145 | 0.0478 | -2.02 | -4.50586 | -0.235 |
| TM2D1 | ILMN_2143148 | 0.00137 | 6.88E-05 | -4.3 | 1.44518 | -0.236 |
| ZC3H7A | ILMN_1693227 | 0.00163 | 8.80E-05 | -4.23 | 1.21433 | -0.236 |
| GOLGA3 | ILMN_1733511 | 0.00917 | 0.000931 | -3.5 | -0.98667 | -0.236 |
| GLG1 | ILMN_1772261 | 0.0114 | 0.00125 | -3.4 | -1.2596 | -0.236 |
| RCC2 | ILMN_1720124 | 0.0622 | 0.0139 | -2.54 | -3.43562 | -0.236 |
| PHF19 | ILMN_1713249 | 0.064 | 0.0144 | -2.53 | -3.47031 | -0.236 |
| POLR1D | ILMN_1742427 | 0.0721 | 0.0172 | -2.46 | -3.62383 | -0.236 |
| TECPR1 | ILMN_1767651 | 0.0835 | 0.0212 | -2.37 | -3.81078 | -0.236 |
| SMARCE1 | ILMN_1747857 | 0.104 | 0.0292 | -2.24 | -4.08597 | -0.236 |
| PDIA6 | ILMN_1680626 | 0.111 | 0.0322 | -2.2 | -4.17131 | -0.236 |
| TBP | ILMN_1697117 | 0.0188 | 0.00251 | -3.17 | -1.89923 | -0.237 |
| TOMM20 | ILMN_1679796 | 0.032 | 0.00535 | -2.9 | -2.58533 | -0.237 |
| APBB1IP | ILMN_1801710 | 0.0613 | 0.0135 | -2.55 | -3.41331 | -0.237 |
| PBDC1 | ILMN_1768176 | 0.0654 | 0.0149 | -2.51 | -3.50142 | -0.237 |
| OTUD6B-AS1 | ILMN_1876124 | 0.12 | 0.0362 | -2.15 | -4.27164 | -0.237 |
| DOCK8 | ILMN_1788931 | 0.124 | 0.0378 | -2.13 | -4.30729 | -0.237 |
| MYNN | ILMN_1672287 | 0.000384 | 1.21E-05 | -4.81 | 3.09114 | -0.238 |
| U2AF2 | ILMN_1768930 | 0.000477 | 1.67E-05 | -4.72 | 2.78313 | -0.238 |
| ZCCHC9 | ILMN_1723007 | 0.0188 | 0.00251 | -3.17 | -1.89951 | -0.238 |
| PLEKHO2 | ILMN_1689968 | 0.0315 | 0.00522 | -2.91 | -2.56289 | -0.238 |
| APEX1 | ILMN_1661886 | 0.0319 | 0.00531 | -2.9 | -2.57831 | -0.238 |
| YY1 | ILMN_2181540 | 0.042 | 0.00782 | -2.76 | -2.92719 | -0.238 |
| SMAP1 | ILMN_2363231 | 0.0426 | 0.00799 | -2.75 | -2.94625 | -0.238 |
| CNPY4 | ILMN_2252408 | 0.0815 | 0.0204 | -2.39 | -3.77694 | -0.238 |
| WDR54 | ILMN_1658289 | 0.0819 | 0.0206 | -2.38 | -3.78462 | -0.238 |
| NMNAT1 | ILMN_1692413 | 0.0902 | 0.0237 | -2.33 | -3.90735 | -0.238 |
| AHSP | ILMN_1696512 | 0.115 | 0.034 | -2.17 | -4.21776 | -0.238 |
| ST13P4 | ILMN_2098437 | 0.126 | 0.0384 | -2.12 | -4.32178 | -0.238 |
| MGAT3 | ILMN_1853824 | 0.145 | 0.0477 | -2.02 | -4.505 | -0.238 |
| NDUFB8 | ILMN_1661170 | 0.00282 | 0.000184 | -4.01 | 0.52207 | -0.239 |
| SLC30A5 | ILMN_1709728 | 0.0391 | 0.00708 | -2.8 | -2.83793 | -0.239 |
| UCHL3 | ILMN_1660111 | 0.0761 | 0.0185 | -2.43 | -3.69101 | -0.239 |
| ARMC7 | ILMN_1797298 | 0.092 | 0.0244 | -2.31 | -3.92988 | -0.239 |
| TRAPPC6B | ILMN_2272967 | 0.0945 | 0.0253 | -2.3 | -3.96276 | -0.239 |
| MRPS36 | ILMN_1807095 | 0.105 | 0.0296 | -2.23 | -4.09938 | -0.239 |
| TNC | ILMN_2145670 | 0.109 | 0.0313 | -2.21 | -4.14563 | -0.239 |
| QRICH1 | ILMN_2385191 | 0.11 | 0.0318 | -2.2 | -4.15934 | -0.239 |
| GUSBP11 | ILMN_3235718 | 0.115 | 0.0339 | -2.17 | -4.21525 | -0.239 |
| HECTD4 | ILMN_3244516 | 0.125 | 0.0381 | -2.12 | -4.31377 | -0.239 |
| G3BP2 | ILMN_2381753 | 0.000206 | 5.02E-06 | -5.05 | 3.92218 | -0.24 |
| CASC4 | ILMN_2325574 | 0.0169 | 0.00217 | -3.22 | -1.76701 | -0.24 |
| SLC16A6 | ILMN_1729691 | 0.0296 | 0.00476 | -2.94 | -2.48091 | -0.24 |
| PANK2 | ILMN_1708095 | 0.0434 | 0.00821 | -2.74 | -2.97063 | -0.24 |
| ITM2C | ILMN_1680453 | 0.0482 | 0.00959 | -2.68 | -3.10873 | -0.24 |
| RBX1 | ILMN_1666670 | 0.0022 | 0.000129 | -4.12 | 0.85251 | -0.241 |
| LOC646214 | ILMN_3243351 | 0.00253 | 0.000157 | -4.06 | 0.66922 | -0.241 |
| SRP9 | ILMN_2099594 | 0.00778 | 0.000738 | -3.57 | -0.77142 | -0.241 |
| ZNF329 | ILMN_1689059 | 0.0111 | 0.0012 | -3.42 | -1.21982 | -0.241 |
| CCDC77 | ILMN_2101375 | 0.0369 | 0.00654 | -2.83 | -2.76626 | -0.241 |
| DHX40 | ILMN_2248589 | 0.0389 | 0.00702 | -2.8 | -2.83021 | -0.241 |
| C22orf39 | ILMN_1804884 | 0.0671 | 0.0155 | -2.5 | -3.532 | -0.241 |
| C14orf28 | ILMN_1807031 | 0.0723 | 0.0172 | -2.46 | -3.62767 | -0.241 |
| GNL3 | ILMN_1806106 | 0.0858 | 0.022 | -2.36 | -3.84281 | -0.241 |
| HOOK3 | ILMN_2062001 | 0.0872 | 0.0226 | -2.35 | -3.86459 | -0.241 |
| VASP | ILMN_1812001 | 0.12 | 0.0359 | -2.15 | -4.26412 | -0.241 |
| ZNF598 | ILMN_2075818 | 0.00238 | 0.000145 | -4.08 | 0.7434 | -0.242 |
| ATF4 | ILMN_1672128 | 0.0167 | 0.00213 | -3.22 | -1.747 | -0.242 |
| CD79B | ILMN_2366212 | 0.0453 | 0.00869 | -2.72 | -3.02164 | -0.242 |
| SREK1 | ILMN_2373266 | 0.0469 | 0.00916 | -2.7 | -3.06823 | -0.242 |
| SPAST | ILMN_1796738 | 0.0575 | 0.0124 | -2.59 | -3.33594 | -0.242 |
| AZIN1 | ILMN_1704550 | 0.0761 | 0.0185 | -2.43 | -3.69112 | -0.242 |
| VIPAS39 | ILMN_1734440 | 0.103 | 0.0285 | -2.25 | -4.06454 | -0.242 |
| ELMO3 | ILMN_1752665 | 0.11 | 0.0319 | -2.2 | -4.16121 | -0.242 |
| MRPL19 | ILMN_1771149 | 0.113 | 0.0329 | -2.19 | -4.19005 | -0.242 |
| ENO1-AS1 | ILMN_1896149 | 0.114 | 0.0335 | -2.18 | -4.20527 | -0.242 |
| ZNF772 | ILMN_1738124 | 0.118 | 0.0352 | -2.16 | -4.24667 | -0.242 |
| KIF1C | ILMN_1796749 | 0.127 | 0.0388 | -2.12 | -4.33058 | -0.242 |
| KCTD13 | ILMN_1786843 | 0.00652 | 0.000578 | -3.65 | -0.54493 | -0.243 |
| HMGN1 | ILMN_1652123 | 0.019 | 0.00255 | -3.16 | -1.91274 | -0.243 |
| ARL16 | ILMN_1679917 | 0.0355 | 0.0062 | -2.85 | -2.71815 | -0.243 |
| PIWIL4 | ILMN_1769606 | 0.0531 | 0.0111 | -2.63 | -3.23673 | -0.243 |
| ADGRE1 | ILMN_1780601 | 0.0931 | 0.0248 | -2.31 | -3.9443 | -0.243 |
| PLOD3 | ILMN_1774836 | 0.114 | 0.0337 | -2.18 | -4.21053 | -0.243 |
| RPL34 | ILMN_1774823 | 0.00206 | 0.000119 | -4.14 | 0.9327 | -0.244 |
| GNLY | ILMN_1708779 | 0.00926 | 0.000947 | -3.49 | -1.00271 | -0.244 |
| DRG1 | ILMN_1658259 | 0.0113 | 0.00124 | -3.41 | -1.24848 | -0.244 |
| MGAT2 | ILMN_2342240 | 0.016 | 0.00201 | -3.24 | -1.69444 | -0.244 |
| RAB24 | ILMN_1677843 | 0.0248 | 0.00377 | -3.02 | -2.26947 | -0.244 |
| UFM1 | ILMN_1757646 | 0.0294 | 0.00473 | -2.94 | -2.47408 | -0.244 |
| CHTF18 | ILMN_1756705 | 0.0627 | 0.014 | -2.54 | -3.4449 | -0.244 |
| RPA1 | ILMN_3249261 | 0.144 | 0.0469 | -2.03 | -4.49078 | -0.244 |
| RCE1 | ILMN_1685002 | 0.00165 | 8.93E-05 | -4.23 | 1.20045 | -0.245 |
| GTF2F1 | ILMN_1748616 | 0.00194 | 0.000109 | -4.17 | 1.01367 | -0.245 |
| TMED7 | ILMN_2186482 | 0.0156 | 0.00193 | -3.26 | -1.65865 | -0.245 |
| RABL3 | ILMN_1662306 | 0.021 | 0.00297 | -3.11 | -2.05246 | -0.245 |
| TAF1 | ILMN_2346358 | 0.104 | 0.0291 | -2.24 | -4.08462 | -0.245 |
| SQLE | ILMN_2041293 | 0.127 | 0.0388 | -2.12 | -4.32967 | -0.245 |
| CCL23 | ILMN_1686109 | 0.046 | 0.00888 | -2.71 | -3.04059 | -0.246 |
| ZDHHC13 | ILMN_1785831 | 0.0473 | 0.00926 | -2.7 | -3.07769 | -0.246 |
| NDUFV1 | ILMN_1786718 | 0.0621 | 0.0138 | -2.54 | -3.43313 | -0.246 |
| TAF13 | ILMN_2061327 | 0.0683 | 0.0158 | -2.49 | -3.55393 | -0.246 |
| DHX40 | ILMN_1653047 | 0.0748 | 0.018 | -2.44 | -3.66822 | -0.246 |
| SLC24A3 | ILMN_1663519 | 0.108 | 0.0308 | -2.22 | -4.1318 | -0.246 |
| ZNF446 | ILMN_1743767 | 0.139 | 0.0448 | -2.05 | -4.45069 | -0.246 |
| ELF4 | ILMN_1652082 | 0.00781 | 0.000741 | -3.57 | -0.77571 | -0.247 |
| NOD2 | ILMN_1762594 | 0.0366 | 0.00647 | -2.83 | -2.75701 | -0.247 |
| TRAF7 | ILMN_2405991 | 0.0903 | 0.0238 | -2.33 | -3.90787 | -0.247 |
| PHF1 | ILMN_1752877 | 0.0998 | 0.0275 | -2.26 | -4.03368 | -0.247 |
| TM6SF1 | ILMN_1750961 | 0.106 | 0.0299 | -2.23 | -4.10784 | -0.247 |
| HDAC7 | ILMN_3266186 | 0.106 | 0.0301 | -2.23 | -4.11248 | -0.247 |
| TMEM106C | ILMN_1692511 | 0.147 | 0.0484 | -2.02 | -4.51683 | -0.247 |
| MSL2 | ILMN_3236373 | 0.00104 | 4.72E-05 | -4.41 | 1.79972 | -0.248 |
| CS | ILMN_3307648 | 0.00133 | 6.59E-05 | -4.32 | 1.48639 | -0.248 |
| SSR1 | ILMN_1750693 | 0.00257 | 0.000161 | -4.05 | 0.64646 | -0.248 |
| TMEM91 | ILMN_2211065 | 0.0119 | 0.00132 | -3.38 | -1.30932 | -0.248 |
| CCDC69 | ILMN_1657680 | 0.0197 | 0.00269 | -3.14 | -1.96216 | -0.248 |
| CITED2 | ILMN_1663092 | 0.029 | 0.00464 | -2.95 | -2.45651 | -0.248 |
| BIRC3 | ILMN_1776181 | 0.0465 | 0.00904 | -2.71 | -3.05696 | -0.248 |
| DEF8 | ILMN_1656718 | 0.062 | 0.0138 | -2.54 | -3.43078 | -0.248 |
| ATP6V1H | ILMN_1689473 | 0.0649 | 0.0148 | -2.52 | -3.49245 | -0.248 |
| POFUT1 | ILMN_1776076 | 0.0683 | 0.0158 | -2.49 | -3.55358 | -0.248 |
| NMB | ILMN_2347592 | 0.127 | 0.0389 | -2.12 | -4.33105 | -0.248 |
| UBE3C | ILMN_1704342 | 0.0353 | 0.00614 | -2.85 | -2.70932 | -0.249 |
| PLPPR2 | ILMN_1667319 | 0.0376 | 0.00672 | -2.82 | -2.7903 | -0.249 |
| GAR1 | ILMN_2412549 | 0.0574 | 0.0123 | -2.59 | -3.33304 | -0.249 |
| MRE11A | ILMN_1709483 | 0.0786 | 0.0194 | -2.41 | -3.73066 | -0.249 |
| CDV3 | ILMN_1710597 | 0.0852 | 0.0218 | -2.36 | -3.83457 | -0.249 |
| LOC440895 | ILMN_3250389 | 0.135 | 0.0428 | -2.07 | -4.41228 | -0.249 |
| CDK19 | ILMN_1676891 | 0.143 | 0.0463 | -2.04 | -4.47991 | -0.249 |
| NUP54 | ILMN_2061405 | 0.00926 | 0.000947 | -3.49 | -1.00281 | -0.25 |
| NAA10 | ILMN_1721977 | 0.0337 | 0.00573 | -2.87 | -2.64771 | -0.25 |
| CTSC | ILMN_1792885 | 0.0445 | 0.00848 | -2.73 | -2.99983 | -0.25 |
| QKI | ILMN_1690476 | 0.0557 | 0.0118 | -2.6 | -3.29546 | -0.25 |
| ODF2 | ILMN_1730698 | 0.059 | 0.0128 | -2.57 | -3.3675 | -0.25 |
| ANPEP | ILMN_1763837 | 0.12 | 0.0359 | -2.15 | -4.26415 | -0.25 |
| IPO8 | ILMN_1753164 | 0.125 | 0.0382 | -2.12 | -4.31547 | -0.25 |
| GTPBP8 | ILMN_1660698 | 0.0746 | 0.018 | -2.44 | -3.66455 | -0.251 |
| LCMT1 | ILMN_2336186 | 0.119 | 0.0358 | -2.15 | -4.26207 | -0.251 |
| ZPR1 | ILMN_1753790 | 0.139 | 0.0447 | -2.05 | -4.45004 | -0.251 |
| RBMX2 | ILMN_2056551 | 0.00275 | 0.000177 | -4.02 | 0.55778 | -0.252 |
| TAF15 | ILMN_1678707 | 0.00428 | 0.000318 | -3.84 | 0.0102 | -0.252 |
| C1orf50 | ILMN_1801941 | 0.0344 | 0.00593 | -2.86 | -2.67765 | -0.252 |
| SNORD68 | ILMN_2082762 | 0.0465 | 0.00904 | -2.71 | -3.05612 | -0.252 |
| S100A4 | ILMN_1688780 | 0.059 | 0.0128 | -2.57 | -3.36577 | -0.252 |
| CD79A | ILMN_1659227 | 0.0646 | 0.0147 | -2.52 | -3.48699 | -0.252 |
| CASP8 | ILMN_2377733 | 0.0649 | 0.0148 | -2.52 | -3.49119 | -0.252 |
| ABHD15 | ILMN_1769390 | 0.102 | 0.0282 | -2.25 | -4.0561 | -0.252 |
| DPAGT1 | ILMN_1692760 | 0.142 | 0.0461 | -2.04 | -4.47494 | -0.252 |
| ALDOA | ILMN_1736700 | 0.00201 | 0.000115 | -4.15 | 0.96537 | -0.253 |
| SPHK2 | ILMN_1729281 | 0.04 | 0.00731 | -2.79 | -2.86632 | -0.253 |
| NAP1L1 | ILMN_1705876 | 0.056 | 0.0119 | -2.6 | -3.30128 | -0.253 |
| WRAP73 | ILMN_1711166 | 0.078 | 0.0192 | -2.41 | -3.72059 | -0.253 |
| SNRNP25 | ILMN_3238712 | 0.149 | 0.0497 | -2.01 | -4.53813 | -0.253 |
| CYBA | ILMN_1744604 | 0.000316 | 9.23E-06 | -4.88 | 3.34369 | -0.254 |
| ELL | ILMN_1736048 | 0.000877 | 3.77E-05 | -4.48 | 2.01325 | -0.254 |
| TRAPPC10 | ILMN_1778464 | 0.00276 | 0.000178 | -4.02 | 0.55369 | -0.254 |
| JUND | ILMN_1810214 | 0.0064 | 0.000556 | -3.66 | -0.50979 | -0.254 |
| EPRS | ILMN_1783695 | 0.022 | 0.00317 | -3.09 | -2.11069 | -0.254 |
| IFFO1 | ILMN_2348268 | 0.039 | 0.00707 | -2.8 | -2.83673 | -0.254 |
| TCP11L1 | ILMN_1713174 | 0.0615 | 0.0136 | -2.55 | -3.41759 | -0.254 |
| MCU | ILMN_1672759 | 0.0897 | 0.0235 | -2.33 | -3.89958 | -0.254 |
| PLBD1 | ILMN_1707286 | 0.103 | 0.0288 | -2.25 | -4.07352 | -0.254 |
| FEM1B | ILMN_1744239 | 0.106 | 0.03 | -2.23 | -4.10852 | -0.254 |
| PTPN9 | ILMN_1789504 | 0.134 | 0.0423 | -2.08 | -4.40272 | -0.254 |
| UBE2K | ILMN_1782954 | 0.00161 | 8.60E-05 | -4.24 | 1.23554 | -0.255 |
| ZDHHC18 | ILMN_1668270 | 0.011 | 0.00119 | -3.42 | -1.21361 | -0.255 |
| ANGEL2 | ILMN_1736340 | 0.0297 | 0.0048 | -2.94 | -2.48723 | -0.255 |
| G3BP2 | ILMN_2381758 | 0.0315 | 0.00521 | -2.91 | -2.56183 | -0.255 |
| LYAR | ILMN_1764362 | 0.0483 | 0.0096 | -2.68 | -3.10971 | -0.255 |
| USP42 | ILMN_2115696 | 0.0536 | 0.0112 | -2.62 | -3.24847 | -0.255 |
| AEBP2 | ILMN_1803376 | 0.0785 | 0.0194 | -2.41 | -3.72925 | -0.255 |
| PIGP | ILMN_1764871 | 0.0944 | 0.0253 | -2.3 | -3.96104 | -0.255 |
| FAM156B | ILMN_3244110 | 0.145 | 0.0474 | -2.03 | -4.49993 | -0.255 |
| DLEC1 | ILMN_2401987 | 0.0121 | 0.00136 | -3.37 | -1.33509 | -0.256 |
| SELK | ILMN_2134110 | 0.0182 | 0.0024 | -3.18 | -1.85742 | -0.256 |
| KMT2C | ILMN_2295183 | 0.0689 | 0.0161 | -2.48 | -3.56647 | -0.256 |
| TTC33 | ILMN_1807088 | 0.0721 | 0.0171 | -2.46 | -3.62335 | -0.256 |
| C19orf60 | ILMN_3268564 | 0.0815 | 0.0205 | -2.39 | -3.77806 | -0.256 |
| PPP1CC | ILMN_1701855 | 0.131 | 0.0411 | -2.09 | -4.37957 | -0.256 |
| DEAF1 | ILMN_1658902 | 0.143 | 0.0464 | -2.04 | -4.48156 | -0.256 |
| LYZ | ILMN_1815205 | 0.0103 | 0.00109 | -3.45 | -1.13308 | -0.257 |
| RAB33A | ILMN_1724708 | 0.0152 | 0.00186 | -3.27 | -1.62628 | -0.257 |
| SGTA | ILMN_1677800 | 0.0252 | 0.00386 | -3.02 | -2.29094 | -0.257 |
| METTL18 | ILMN_1734915 | 0.0831 | 0.021 | -2.38 | -3.80142 | -0.257 |
| LMAN2L | ILMN_1755221 | 0.0919 | 0.0243 | -2.32 | -3.92899 | -0.257 |
| DENND5A | ILMN_1785356 | 7.42E-05 | 1.15E-06 | -5.46 | 5.32674 | -0.258 |
| FAM104A | ILMN_1807201 | 0.0065 | 0.000575 | -3.65 | -0.54037 | -0.258 |
| RPS6KB2 | ILMN_2364357 | 0.00808 | 0.000781 | -3.56 | -0.824 | -0.258 |
| TTC17 | ILMN_1660810 | 0.0365 | 0.00644 | -2.83 | -2.75332 | -0.258 |
| MEI1 | ILMN_1810254 | 0.0831 | 0.021 | -2.38 | -3.80167 | -0.258 |
| DDX49 | ILMN_1762225 | 0.129 | 0.0401 | -2.1 | -4.35814 | -0.258 |
| GRIPAP1 | ILMN_1719857 | 0.144 | 0.0472 | -2.03 | -4.49598 | -0.258 |
| DIABLO | ILMN_2332990 | 0.00832 | 0.000811 | -3.54 | -0.8594 | -0.259 |
| BRPF1 | ILMN_2365549 | 0.0139 | 0.00164 | -3.31 | -1.5094 | -0.259 |
| ZFPL1 | ILMN_1693039 | 0.0225 | 0.00328 | -3.07 | -2.1425 | -0.259 |
| SLCO3A1 | ILMN_1654735 | 0.0259 | 0.00398 | -3.01 | -2.31872 | -0.259 |
| TRIM39 | ILMN_2413517 | 0.0269 | 0.00418 | -2.99 | -2.36269 | -0.259 |
| ZNF106 | ILMN_1800912 | 0.0527 | 0.0109 | -2.63 | -3.22552 | -0.259 |
| ACOT8 | ILMN_1679600 | 0.0586 | 0.0127 | -2.58 | -3.3576 | -0.259 |
| FAM60A | ILMN_3272603 | 0.0716 | 0.0169 | -2.46 | -3.61138 | -0.259 |
| TSPYL2 | ILMN_1657554 | 0.125 | 0.0382 | -2.12 | -4.31715 | -0.259 |
| PDK3 | ILMN_1776582 | 0.135 | 0.0428 | -2.07 | -4.4131 | -0.259 |
| SRSF9 | ILMN_1760683 | 0.000371 | 1.15E-05 | -4.82 | 3.13278 | -0.26 |
| SFSWAP | ILMN_1692575 | 0.00335 | 0.000228 | -3.94 | 0.32352 | -0.26 |
| PRKRA | ILMN_1758474 | 0.00636 | 0.000548 | -3.67 | -0.49542 | -0.26 |
| IMPDH1 | ILMN_1676515 | 0.0099 | 0.00104 | -3.46 | -1.08742 | -0.26 |
| ARAP3 | ILMN_1812618 | 0.0141 | 0.00168 | -3.3 | -1.53226 | -0.26 |
| VBP1 | ILMN_2223010 | 0.0221 | 0.00321 | -3.08 | -2.12167 | -0.26 |
| LRRC8D | ILMN_1763409 | 0.0469 | 0.00915 | -2.7 | -3.06715 | -0.26 |
| BOP1 | ILMN_1715583 | 0.0953 | 0.0256 | -2.29 | -3.97282 | -0.26 |
| PPIB | ILMN_1703622 | 0.0983 | 0.0269 | -2.27 | -4.01458 | -0.26 |
| ADPRHL2 | ILMN_1811480 | 0.0997 | 0.0274 | -2.27 | -4.03125 | -0.26 |
| OPTN | ILMN_2381899 | 0.104 | 0.0291 | -2.24 | -4.08482 | -0.26 |
| ETV6 | ILMN_1789596 | 0.105 | 0.0296 | -2.23 | -4.0971 | -0.26 |
| PIM3 | ILMN_1789781 | 0.148 | 0.0488 | -2.01 | -4.52404 | -0.26 |
| SFT2D1 | ILMN_1734895 | 9.42E-05 | 1.63E-06 | -5.37 | 4.99099 | -0.261 |
| TWSG1 | ILMN_2214144 | 0.0102 | 0.00108 | -3.45 | -1.12264 | -0.261 |
| ZNHIT3 | ILMN_1750044 | 0.0186 | 0.00248 | -3.17 | -1.88885 | -0.261 |
| ARMC10 | ILMN_2052598 | 0.0202 | 0.0028 | -3.13 | -1.99874 | -0.261 |
| ASCC2 | ILMN_1679919 | 0.0242 | 0.00365 | -3.04 | -2.2386 | -0.261 |
| BZW2 | ILMN_1676548 | 0.0522 | 0.0108 | -2.64 | -3.21259 | -0.261 |
| PITPNB | ILMN_1809245 | 0.0738 | 0.0177 | -2.44 | -3.65317 | -0.261 |
| MYO9B | ILMN_1672547 | 0.0881 | 0.023 | -2.34 | -3.88055 | -0.261 |
| RFXANK | ILMN_1804113 | 0.127 | 0.0393 | -2.11 | -4.34043 | -0.261 |
| DEF8 | ILMN_1656185 | 0.00607 | 0.000513 | -3.69 | -0.43365 | -0.262 |
| ITGAE | ILMN_1683927 | 0.0173 | 0.00224 | -3.21 | -1.7927 | -0.262 |
| ATF4 | ILMN_2358457 | 0.0314 | 0.00517 | -2.91 | -2.55457 | -0.262 |
| ZBTB21 | ILMN_1782110 | 0.0504 | 0.0102 | -2.66 | -3.16764 | -0.262 |
| AKAP8L | ILMN_1768962 | 0.0939 | 0.025 | -2.3 | -3.95365 | -0.262 |
| PTPN11 | ILMN_1778236 | 0.00105 | 4.81E-05 | -4.41 | 1.78238 | -0.263 |
| PSMC6 | ILMN_1655316 | 0.00293 | 0.000193 | -3.99 | 0.47828 | -0.263 |
| CBWD5 | ILMN_2150352 | 0.0593 | 0.013 | -2.57 | -3.37643 | -0.263 |
| GPATCH2L | ILMN_1699091 | 0.0995 | 0.0273 | -2.27 | -4.02866 | -0.263 |
| LXN | ILMN_1723962 | 0.108 | 0.0306 | -2.22 | -4.12671 | -0.263 |
| HERC4 | ILMN_1678922 | 0.00046 | 1.56E-05 | -4.74 | 2.8476 | -0.264 |
| LSM14B | ILMN_1777725 | 0.0448 | 0.00856 | -2.73 | -3.00787 | -0.264 |
| PTPMT1 | ILMN_3236945 | 0.0638 | 0.0144 | -2.53 | -3.46809 | -0.264 |
| MRPL42 | ILMN_2356890 | 0.0902 | 0.0237 | -2.33 | -3.9063 | -0.264 |
| LUC7L2 | ILMN_1747099 | 0.129 | 0.0399 | -2.1 | -4.35412 | -0.264 |
| GOLGA4 | ILMN_1776297 | 0.13 | 0.0405 | -2.1 | -4.36589 | -0.264 |
| LSM1 | ILMN_2218450 | 0.000778 | 3.21E-05 | -4.53 | 2.16386 | -0.265 |
| LINC00493 | ILMN_1803953 | 0.00243 | 0.000149 | -4.07 | 0.71872 | -0.265 |
| NGDN | ILMN_2324998 | 0.00309 | 0.000206 | -3.97 | 0.41497 | -0.265 |
| CALML4 | ILMN_1757210 | 0.0152 | 0.00188 | -3.27 | -1.63261 | -0.265 |
| CWC22 | ILMN_1674128 | 0.0313 | 0.00513 | -2.91 | -2.54774 | -0.265 |
| POC5 | ILMN_2225577 | 0.0684 | 0.0159 | -2.49 | -3.55538 | -0.265 |
| BTN3A1 | ILMN_1687888 | 0.094 | 0.0251 | -2.3 | -3.95513 | -0.265 |
| UBTF | ILMN_2394264 | 0.131 | 0.0411 | -2.09 | -4.37765 | -0.265 |
| C8orf59 | ILMN_1653205 | 0.00046 | 1.57E-05 | -4.73 | 2.84308 | -0.266 |
| PTPN6 | ILMN_1738675 | 0.00197 | 0.000112 | -4.16 | 0.99137 | -0.266 |
| ZNF518A | ILMN_1742541 | 0.014 | 0.00166 | -3.31 | -1.51694 | -0.266 |
| PHF1 | ILMN_2385866 | 0.0241 | 0.00361 | -3.04 | -2.22871 | -0.266 |
| ALKBH7 | ILMN_1761764 | 0.0355 | 0.0062 | -2.85 | -2.71817 | -0.266 |
| CYLD | ILMN_1775508 | 0.0592 | 0.0129 | -2.57 | -3.37242 | -0.266 |
| SUV39H1 | ILMN_1781479 | 0.0613 | 0.0135 | -2.55 | -3.41336 | -0.266 |
| GCC2 | ILMN_2408730 | 0.0974 | 0.0266 | -2.28 | -4.00446 | -0.266 |
| GDAP2 | ILMN_1789059 | 0.115 | 0.0338 | -2.18 | -4.21283 | -0.266 |
| DNAJB14 | ILMN_1668979 | 0.127 | 0.0389 | -2.12 | -4.33096 | -0.266 |
| AKAP10 | ILMN_1718808 | 0.145 | 0.0474 | -2.03 | -4.49988 | -0.266 |
| DDX41 | ILMN_1737344 | 0.0141 | 0.00169 | -3.3 | -1.53519 | -0.267 |
| NIF3L1 | ILMN_1777066 | 0.0149 | 0.00181 | -3.28 | -1.59941 | -0.267 |
| STT3A | ILMN_1746090 | 0.0297 | 0.0048 | -2.94 | -2.48798 | -0.267 |
| HELQ | ILMN_1720440 | 0.0338 | 0.00578 | -2.87 | -2.65473 | -0.267 |
| FAM156A | ILMN_1658160 | 0.106 | 0.0299 | -2.23 | -4.10753 | -0.267 |
| CTAGE5 | ILMN_2314080 | 0.108 | 0.031 | -2.21 | -4.13788 | -0.267 |
| TNFRSF4 | ILMN_2112256 | 0.112 | 0.0324 | -2.19 | -4.17589 | -0.267 |
| TMEM258 | ILMN_1786759 | 0.000144 | 2.89E-06 | -5.21 | 4.44815 | -0.268 |
| EFHD2 | ILMN_1761463 | 0.000195 | 4.52E-06 | -5.08 | 4.02243 | -0.268 |
| TMED7 | ILMN_1672405 | 0.00628 | 0.000536 | -3.68 | -0.47491 | -0.268 |
| CD86 | ILMN_1782560 | 0.0405 | 0.00744 | -2.78 | -2.88198 | -0.268 |
| GEMIN4 | ILMN_1770206 | 0.0973 | 0.0265 | -2.28 | -4.00107 | -0.268 |
| ANKMY2 | ILMN_1784292 | 0.113 | 0.0331 | -2.19 | -4.19351 | -0.268 |
| PLAUR | ILMN_1691508 | 0.00533 | 0.000434 | -3.74 | -0.2781 | -0.269 |
| CBLL1 | ILMN_1705433 | 0.0104 | 0.00111 | -3.44 | -1.14821 | -0.269 |
| PIM2 | ILMN_1748283 | 0.0475 | 0.00934 | -2.69 | -3.08554 | -0.269 |
| FERMT3 | ILMN_2366334 | 0.049 | 0.00984 | -2.67 | -3.13224 | -0.269 |
| QPRT | ILMN_1700268 | 0.104 | 0.0293 | -2.24 | -4.08793 | -0.269 |
| TRIM4 | ILMN_2323385 | 0.000839 | 3.55E-05 | -4.5 | 2.07013 | -0.27 |
| TMEM256 | ILMN_2201533 | 0.00149 | 7.76E-05 | -4.27 | 1.33256 | -0.27 |
| SIK3 | ILMN_1732343 | 0.00412 | 0.000301 | -3.86 | 0.06061 | -0.27 |
| CDK7 | ILMN_1778917 | 0.00616 | 0.000522 | -3.68 | -0.45027 | -0.27 |
| ANKRA2 | ILMN_2185563 | 0.00672 | 0.000604 | -3.64 | -0.58541 | -0.27 |
| PP7080 | ILMN_1836218 | 0.0525 | 0.0109 | -2.64 | -3.21927 | -0.27 |
| ZNF585A | ILMN_1737211 | 0.115 | 0.0342 | -2.17 | -4.22146 | -0.27 |
| PARK7 | ILMN_1744713 | 0.000344 | 1.05E-05 | -4.85 | 3.22611 | -0.271 |
| IVNS1ABP | ILMN_2397750 | 0.00129 | 6.25E-05 | -4.33 | 1.53673 | -0.271 |
| ZNF581 | ILMN_1679093 | 0.00592 | 0.000495 | -3.7 | -0.40105 | -0.271 |
| ZNF160 | ILMN_2312149 | 0.0317 | 0.00527 | -2.9 | -2.57232 | -0.271 |
| VAV1 | ILMN_1717334 | 0.0406 | 0.00746 | -2.78 | -2.88505 | -0.271 |
| GTF3C2 | ILMN_2356574 | 0.0423 | 0.0079 | -2.76 | -2.93574 | -0.271 |
| ARHGEF18 | ILMN_1664016 | 0.049 | 0.00983 | -2.67 | -3.13093 | -0.271 |
| METTL21B | ILMN_1723846 | 0.0612 | 0.0135 | -2.55 | -3.41154 | -0.271 |
| RAB11B | ILMN_1731135 | 0.0815 | 0.0204 | -2.39 | -3.77741 | -0.271 |
| ZNF17 | ILMN_1751692 | 0.0948 | 0.0254 | -2.3 | -3.96634 | -0.271 |
| GTF3C5 | ILMN_1723895 | 0.0991 | 0.0272 | -2.27 | -4.02526 | -0.271 |
| IMPA1 | ILMN_1758811 | 0.0012 | 5.69E-05 | -4.36 | 1.62386 | -0.272 |
| TVP23B | ILMN_1661593 | 0.00242 | 0.000148 | -4.08 | 0.72655 | -0.272 |
| GRK2 | ILMN_3307926 | 0.00759 | 0.000714 | -3.58 | -0.74112 | -0.272 |
| RRP8 | ILMN_2066667 | 0.0116 | 0.00127 | -3.4 | -1.27561 | -0.272 |
| SASS6 | ILMN_2089458 | 0.0342 | 0.00589 | -2.86 | -2.67242 | -0.272 |
| DCPS | ILMN_1740737 | 0.0544 | 0.0114 | -2.62 | -3.26561 | -0.272 |
| ATXN1L | ILMN_3193798 | 0.0974 | 0.0265 | -2.28 | -4.00329 | -0.272 |
| TAF6L | ILMN_1727281 | 0.11 | 0.0314 | -2.21 | -4.14936 | -0.272 |
| BAZ2B | ILMN_1720850 | 0.129 | 0.0401 | -2.1 | -4.35755 | -0.272 |
| CD82 | ILMN_2392274 | 0.000501 | 1.80E-05 | -4.69 | 2.71366 | -0.273 |
| NINJ1 | ILMN_1815086 | 7.00E-04 | 2.79E-05 | -4.57 | 2.29707 | -0.273 |
| KIAA0556 | ILMN_1753426 | 0.00781 | 0.000744 | -3.57 | -0.77905 | -0.273 |
| PHF1 | ILMN_1746968 | 0.00983 | 0.00103 | -3.47 | -1.07843 | -0.273 |
| SUN2 | ILMN_2099301 | 0.0167 | 0.00213 | -3.22 | -1.74834 | -0.273 |
| SEC22A | ILMN_1775036 | 0.02 | 0.00276 | -3.13 | -1.98457 | -0.273 |
| TYMS | ILMN_1806040 | 0.0485 | 0.00966 | -2.68 | -3.11607 | -0.273 |
| MAPKAPK5 | ILMN_1699082 | 0.0786 | 0.0194 | -2.41 | -3.73109 | -0.273 |
| PAX3 | ILMN_1707314 | 0.0826 | 0.0208 | -2.38 | -3.79375 | -0.273 |
| HAX1 | ILMN_2289775 | 0.132 | 0.0414 | -2.09 | -4.38379 | -0.273 |
| MANBAL | ILMN_2395926 | 0.000805 | 3.37E-05 | -4.51 | 2.11891 | -0.274 |
| MEA1 | ILMN_1727073 | 0.0394 | 0.00715 | -2.79 | -2.84627 | -0.274 |
| PTK2B | ILMN_1732318 | 0.0612 | 0.0135 | -2.55 | -3.41233 | -0.274 |
| KDM7A | ILMN_3238369 | 0.107 | 0.0303 | -2.22 | -4.11746 | -0.274 |
| CCNY | ILMN_1708991 | 0.00504 | 0.000403 | -3.77 | -0.20977 | -0.275 |
| MAPRE2 | ILMN_1695276 | 0.0229 | 0.00335 | -3.07 | -2.1607 | -0.275 |
| RASGRP2 | ILMN_1794594 | 0.0342 | 0.00587 | -2.87 | -2.66961 | -0.275 |
| FAM189B | ILMN_1795026 | 0.0957 | 0.0258 | -2.29 | -3.97786 | -0.275 |
| NBPF14 | ILMN_1698067 | 0.149 | 0.0492 | -2.01 | -4.52994 | -0.275 |
| DNAJA2 | ILMN_1770127 | 0.000649 | 2.54E-05 | -4.6 | 2.38627 | -0.276 |
| TMF1 | ILMN_1661142 | 0.00192 | 0.000108 | -4.17 | 1.02545 | -0.276 |
| SRSF7 | ILMN_1778836 | 0.00842 | 0.000823 | -3.54 | -0.87269 | -0.276 |
| CAT | ILMN_1651705 | 0.018 | 0.00237 | -3.19 | -1.84431 | -0.276 |
| TMEM50B | ILMN_2047599 | 0.0183 | 0.00242 | -3.18 | -1.86574 | -0.276 |
| SEMA4A | ILMN_1702787 | 0.0228 | 0.00333 | -3.07 | -2.15715 | -0.276 |
| TMEM86B | ILMN_2049417 | 0.0307 | 0.00502 | -2.92 | -2.52811 | -0.276 |
| PLEKHA3 | ILMN_1797576 | 0.0389 | 0.00703 | -2.8 | -2.83155 | -0.276 |
| SGK1 | ILMN_1702487 | 0.0931 | 0.0248 | -2.31 | -3.9441 | -0.276 |
| LINC01451 | ILMN_1838767 | 0.121 | 0.0363 | -2.15 | -4.2733 | -0.276 |
| PTPRA | ILMN_1762766 | 0.0308 | 0.00503 | -2.92 | -2.53049 | -0.277 |
| LSP1 | ILMN_1654778 | 0.037 | 0.00657 | -2.82 | -2.77034 | -0.277 |
| COMMD3 | ILMN_1690392 | 0.0425 | 0.00796 | -2.75 | -2.94235 | -0.277 |
| DCAKD | ILMN_1811648 | 0.0619 | 0.0137 | -2.55 | -3.42695 | -0.277 |
| FMC1 | ILMN_3238680 | 0.101 | 0.0279 | -2.26 | -4.04688 | -0.277 |
| MRPL11 | ILMN_1690371 | 0.116 | 0.0345 | -2.17 | -4.2296 | -0.277 |
| BRPF1 | ILMN_1669308 | 0.0292 | 0.00467 | -2.95 | -2.46299 | -0.278 |
| PER2 | ILMN_1738095 | 0.034 | 0.00581 | -2.87 | -2.66003 | -0.278 |
| TNFSF14 | ILMN_1655414 | 0.0374 | 0.00665 | -2.82 | -2.78163 | -0.278 |
| ETV3 | ILMN_1703180 | 0.048 | 0.00947 | -2.69 | -3.09773 | -0.278 |
| NRDE2 | ILMN_1740165 | 0.0534 | 0.0112 | -2.63 | -3.24369 | -0.278 |
| PGGT1B | ILMN_2156786 | 0.106 | 0.03 | -2.23 | -4.10911 | -0.278 |
| TFAM | ILMN_1715661 | 0.107 | 0.0303 | -2.22 | -4.11877 | -0.278 |
| TMEM87A | ILMN_1716816 | 0.00012 | 2.27E-06 | -5.28 | 4.67684 | -0.279 |
| SCNM1 | ILMN_1746598 | 0.00148 | 7.62E-05 | -4.27 | 1.34924 | -0.279 |
| AMPD3 | ILMN_1665307 | 0.00638 | 0.000553 | -3.67 | -0.5039 | -0.279 |
| THAP11 | ILMN_1780699 | 0.0175 | 0.00228 | -3.2 | -1.80981 | -0.279 |
| NMD3 | ILMN_1727348 | 0.0187 | 0.0025 | -3.17 | -1.89429 | -0.279 |
| AHR | ILMN_2162799 | 0.0479 | 0.00944 | -2.69 | -3.0956 | -0.279 |
| LPGAT1 | ILMN_1687998 | 0.059 | 0.0128 | -2.57 | -3.36841 | -0.279 |
| PPTC7 | ILMN_1800855 | 0.0835 | 0.0212 | -2.37 | -3.81047 | -0.279 |
| AMPD3 | ILMN_1731223 | 0.0944 | 0.0252 | -2.3 | -3.96055 | -0.279 |
| NADSYN1 | ILMN_1779034 | 0.000172 | 3.85E-06 | -5.13 | 4.17326 | -0.28 |
| ALDOA | ILMN_1741148 | 0.000718 | 2.90E-05 | -4.56 | 2.26112 | -0.28 |
| ZHX2 | ILMN_2184966 | 0.00461 | 0.000352 | -3.81 | -0.08477 | -0.28 |
| API5 | ILMN_1815051 | 0.00507 | 0.000407 | -3.76 | -0.21927 | -0.28 |
| TRIM28 | ILMN_1736575 | 0.00638 | 0.000553 | -3.67 | -0.5039 | -0.28 |
| C4orf48 | ILMN_3239531 | 0.0583 | 0.0126 | -2.58 | -3.35194 | -0.28 |
| AKT1 | ILMN_2410909 | 0.0625 | 0.0139 | -2.54 | -3.44135 | -0.28 |
| STAP1 | ILMN_1781085 | 0.0728 | 0.0174 | -2.45 | -3.63491 | -0.28 |
| GNG8 | ILMN_1693269 | 0.0825 | 0.0208 | -2.38 | -3.79265 | -0.28 |
| MED13 | ILMN_1806184 | 0.109 | 0.031 | -2.21 | -4.13911 | -0.28 |
| PRDX1 | ILMN_2366388 | 0.134 | 0.0421 | -2.08 | -4.39976 | -0.28 |
| AKNA | ILMN_1770673 | 0.000805 | 3.37E-05 | -4.51 | 2.11798 | -0.281 |
| SASH3 | ILMN_1697554 | 0.00246 | 0.000152 | -4.07 | 0.70241 | -0.281 |
| PSMG2 | ILMN_2088410 | 0.0142 | 0.0017 | -3.3 | -1.53975 | -0.281 |
| ADGRE3 | ILMN_2348487 | 0.0147 | 0.00178 | -3.28 | -1.58144 | -0.281 |
| GALC | ILMN_1799744 | 0.0197 | 0.00271 | -3.14 | -1.9674 | -0.281 |
| EHD4 | ILMN_1720083 | 0.0389 | 0.00703 | -2.8 | -2.83162 | -0.281 |
| PADI4 | ILMN_1807529 | 0.0449 | 0.00859 | -2.72 | -3.01119 | -0.281 |
| COQ9 | ILMN_1756898 | 0.0469 | 0.00917 | -2.7 | -3.06963 | -0.281 |
| BBX | ILMN_1745415 | 0.056 | 0.0119 | -2.6 | -3.30317 | -0.281 |
| RAP2A | ILMN_1677404 | 0.0802 | 0.02 | -2.4 | -3.75735 | -0.281 |
| MAT2B | ILMN_1680246 | 0.0877 | 0.0228 | -2.34 | -3.87274 | -0.281 |
| THAP1 | ILMN_2301955 | 0.119 | 0.0358 | -2.15 | -4.26037 | -0.281 |
| INSIG2 | ILMN_1676629 | 0.0169 | 0.00217 | -3.22 | -1.76505 | -0.282 |
| BORCS8-MEF2B | ILMN_2068202 | 0.0202 | 0.0028 | -3.13 | -1.99967 | -0.282 |
| NDUFS3 | ILMN_1756355 | 0.021 | 0.00297 | -3.11 | -2.0513 | -0.282 |
| SNRNP40 | ILMN_3247064 | 0.0439 | 0.00831 | -2.74 | -2.98129 | -0.282 |
| FOPNL | ILMN_1790650 | 0.0454 | 0.00871 | -2.72 | -3.02364 | -0.282 |
| VTA1 | ILMN_1813010 | 0.0508 | 0.0104 | -2.65 | -3.17752 | -0.282 |
| OGG1 | ILMN_2352609 | 0.0532 | 0.0111 | -2.63 | -3.23904 | -0.282 |
| OXER1 | ILMN_1765061 | 0.0561 | 0.012 | -2.6 | -3.30517 | -0.282 |
| UEVLD | ILMN_1711452 | 0.0643 | 0.0146 | -2.52 | -3.47891 | -0.282 |
| FCAR | ILMN_2379967 | 0.0868 | 0.0224 | -2.35 | -3.85715 | -0.282 |
| BRAF | ILMN_1652472 | 0.141 | 0.0457 | -2.04 | -4.469 | -0.282 |
| TSC22D2 | ILMN_2124187 | 0.000315 | 9.18E-06 | -4.89 | 3.34961 | -0.283 |
| IVNS1ABP | ILMN_1717877 | 0.000598 | 2.26E-05 | -4.63 | 2.49702 | -0.283 |
| AP5M1 | ILMN_1741331 | 0.000603 | 2.28E-05 | -4.63 | 2.48661 | -0.283 |
| LSP1 | ILMN_1699836 | 0.0248 | 0.00377 | -3.02 | -2.26866 | -0.283 |
| EEF1B2 | ILMN_1701930 | 0.0675 | 0.0156 | -2.49 | -3.54081 | -0.283 |
| EDRF1 | ILMN_1791656 | 0.0869 | 0.0225 | -2.35 | -3.85922 | -0.283 |
| SETD2 | ILMN_1769473 | 0.00531 | 0.000431 | -3.75 | -0.27262 | -0.284 |
| CDK13 | ILMN_2356068 | 0.0167 | 0.00213 | -3.22 | -1.74933 | -0.284 |
| HAUS8 | ILMN_3253579 | 0.0241 | 0.00362 | -3.04 | -2.23263 | -0.284 |
| TRIM13 | ILMN_2350114 | 0.0574 | 0.0123 | -2.59 | -3.33108 | -0.284 |
| CLDN11 | ILMN_1754103 | 0.079 | 0.0195 | -2.4 | -3.73788 | -0.284 |
| MTO1 | ILMN_2380740 | 0.0794 | 0.0197 | -2.4 | -3.74361 | -0.284 |
| ZNF529 | ILMN_1660193 | 0.114 | 0.0336 | -2.18 | -4.20741 | -0.284 |
| PRPF31 | ILMN_1719204 | 0.00135 | 6.69E-05 | -4.31 | 1.47261 | -0.285 |
| DEGS1 | ILMN_1780058 | 0.00272 | 0.000174 | -4.03 | 0.57244 | -0.285 |
| INO80E | ILMN_1724406 | 0.0207 | 0.00291 | -3.12 | -2.03362 | -0.285 |
| PDIA3P1 | ILMN_2075436 | 0.0515 | 0.0105 | -2.65 | -3.19271 | -0.285 |
| TIMM8B | ILMN_1738938 | 0.0575 | 0.0124 | -2.59 | -3.33447 | -0.285 |
| PPP3CA | ILMN_1670970 | 0.0645 | 0.0146 | -2.52 | -3.4835 | -0.285 |
| TRAK1 | ILMN_2289924 | 0.0656 | 0.015 | -2.51 | -3.5069 | -0.285 |
| PTMA | ILMN_1759954 | 0.127 | 0.039 | -2.11 | -4.33449 | -0.285 |
| EHBP1L1 | ILMN_3244457 | 0.00228 | 0.000137 | -4.1 | 0.79854 | -0.286 |
| CCL5 | ILMN_2098126 | 0.00448 | 0.000339 | -3.82 | -0.0482 | -0.286 |
| NDUFAF1 | ILMN_1754421 | 0.00862 | 0.00085 | -3.53 | -0.90302 | -0.286 |
| YES1 | ILMN_1691466 | 0.0227 | 0.00331 | -3.07 | -2.15078 | -0.286 |
| TRAF3IP2 | ILMN_1701514 | 0.0262 | 0.00405 | -3 | -2.33415 | -0.286 |
| CYP4F3 | ILMN_3251260 | 0.112 | 0.0324 | -2.19 | -4.17645 | -0.286 |
| CCL5 | ILMN_1773352 | 0.00128 | 6.15E-05 | -4.34 | 1.55088 | -0.287 |
| EDF1 | ILMN_2246894 | 0.0019 | 0.000106 | -4.18 | 1.0385 | -0.287 |
| C1orf131 | ILMN_1805474 | 0.00228 | 0.000136 | -4.1 | 0.80404 | -0.287 |
| SS18L2 | ILMN_1796407 | 0.0123 | 0.00139 | -3.37 | -1.35346 | -0.287 |
| NUDCD3 | ILMN_1796900 | 0.0501 | 0.0102 | -2.66 | -3.16274 | -0.287 |
| MAP3K7 | ILMN_2379326 | 0.138 | 0.0443 | -2.06 | -4.44146 | -0.287 |
| BBS4 | ILMN_1762466 | 0.00776 | 0.000735 | -3.58 | -0.76779 | -0.288 |
| ICAM3 | ILMN_2212763 | 0.0126 | 0.00143 | -3.36 | -1.38418 | -0.288 |
| SLC25A11 | ILMN_1664168 | 0.0527 | 0.011 | -2.63 | -3.22744 | -0.288 |
| CREBL2 | ILMN_1660223 | 0.0844 | 0.0215 | -2.37 | -3.82158 | -0.288 |
| NUP54 | ILMN_1771835 | 0.102 | 0.0283 | -2.25 | -4.05854 | -0.288 |
| UBTF | ILMN_1806946 | 0.13 | 0.0406 | -2.1 | -4.3681 | -0.288 |
| PSMD4 | ILMN_3307799 | 0.149 | 0.0493 | -2.01 | -4.53216 | -0.288 |
| TXNDC17 | ILMN_1659437 | 0.000542 | 1.97E-05 | -4.67 | 2.62592 | -0.289 |
| MCM3AP | ILMN_1784766 | 0.00766 | 0.000721 | -3.58 | -0.75032 | -0.289 |
| NUP62 | ILMN_1738681 | 0.0139 | 0.00164 | -3.31 | -1.5108 | -0.289 |
| ENO1 | ILMN_1710756 | 0.00022 | 5.48E-06 | -5.03 | 3.83893 | -0.29 |
| POLR2F | ILMN_1745885 | 0.000859 | 3.64E-05 | -4.49 | 2.04453 | -0.29 |
| SRP68 | ILMN_1703524 | 0.0064 | 0.000557 | -3.66 | -0.51081 | -0.29 |
| C2orf47 | ILMN_1795007 | 0.00705 | 0.000644 | -3.62 | -0.64584 | -0.29 |
| MID1IP1 | ILMN_1668960 | 0.049 | 0.00985 | -2.67 | -3.13289 | -0.29 |
| HGSNAT | ILMN_1662413 | 0.0878 | 0.0229 | -2.34 | -3.87429 | -0.29 |
| PPP3CA | ILMN_2044226 | 0.11 | 0.0315 | -2.21 | -4.15142 | -0.29 |
| PPP2R1A | ILMN_1810467 | 0.000158 | 3.31E-06 | -5.17 | 4.31724 | -0.291 |
| ADM | ILMN_1708934 | 0.00055 | 2.02E-05 | -4.66 | 2.60435 | -0.291 |
| SIPA1 | ILMN_2415536 | 0.00383 | 0.00027 | -3.89 | 0.1637 | -0.291 |
| TYK2 | ILMN_1676955 | 0.0129 | 0.00147 | -3.35 | -1.40881 | -0.291 |
| PIK3AP1 | ILMN_1652787 | 0.0133 | 0.00155 | -3.33 | -1.45496 | -0.291 |
| FBXO42 | ILMN_1756874 | 0.0156 | 0.00194 | -3.25 | -1.66266 | -0.291 |
| SLC9A1 | ILMN_1800425 | 0.0298 | 0.00483 | -2.94 | -2.49283 | -0.291 |
| EXOSC10 | ILMN_2402168 | 0.0314 | 0.00517 | -2.91 | -2.55484 | -0.291 |
| TERF1 | ILMN_2357382 | 0.0353 | 0.00614 | -2.85 | -2.70925 | -0.291 |
| FBXO46 | ILMN_2350266 | 0.036 | 0.00631 | -2.84 | -2.73467 | -0.291 |
| CASP8 | ILMN_1787749 | 0.0412 | 0.00762 | -2.77 | -2.90374 | -0.291 |
| YAF2 | ILMN_1765606 | 0.0833 | 0.0211 | -2.37 | -3.80488 | -0.291 |
| GZF1 | ILMN_1763328 | 0.106 | 0.0298 | -2.23 | -4.10298 | -0.291 |
| FCF1 | ILMN_2189869 | 0.106 | 0.0299 | -2.23 | -4.10683 | -0.291 |
| BRD7P3 | ILMN_2147920 | 0.108 | 0.0308 | -2.22 | -4.13215 | -0.291 |
| PIK3R4 | ILMN_1715832 | 0.138 | 0.0444 | -2.06 | -4.44385 | -0.291 |
| ANKRD49 | ILMN_1669259 | 3.51E-05 | 4.22E-07 | -5.73 | 6.27961 | -0.292 |
| SLC35A1 | ILMN_1741440 | 0.00192 | 0.000108 | -4.17 | 1.02561 | -0.292 |
| PBX2 | ILMN_1682699 | 0.00201 | 0.000114 | -4.15 | 0.9691 | -0.292 |
| FNTA | ILMN_1746494 | 0.00944 | 0.000975 | -3.48 | -1.02932 | -0.292 |
| TMEM209 | ILMN_1748926 | 0.0551 | 0.0117 | -2.61 | -3.28239 | -0.292 |
| TWISTNB | ILMN_1781121 | 0.104 | 0.029 | -2.24 | -4.08147 | -0.292 |
| PFN1 | ILMN_2151817 | 0.0011 | 5.11E-05 | -4.39 | 1.72665 | -0.293 |
| NSF | ILMN_1680353 | 0.00192 | 0.000108 | -4.17 | 1.02475 | -0.293 |
| ST3GAL2 | ILMN_1714165 | 0.0152 | 0.00186 | -3.27 | -1.6216 | -0.293 |
| ADAM12 | ILMN_1705689 | 0.0187 | 0.00251 | -3.17 | -1.89777 | -0.293 |
| HP1BP3 | ILMN_1701169 | 0.0359 | 0.00627 | -2.84 | -2.72928 | -0.293 |
| HLA-DPB1 | ILMN_1749070 | 0.0459 | 0.00886 | -2.71 | -3.03822 | -0.293 |
| RFXANK | ILMN_2399497 | 0.059 | 0.0128 | -2.57 | -3.36695 | -0.293 |
| DBR1 | ILMN_1678680 | 0.104 | 0.0289 | -2.24 | -4.07703 | -0.293 |
| TRIM26 | ILMN_1738704 | 0.108 | 0.0307 | -2.22 | -4.13067 | -0.293 |
| SLC4A7 | ILMN_2200917 | 0.138 | 0.044 | -2.06 | -4.43704 | -0.293 |
| CTSC | ILMN_2242463 | 0.00348 | 0.000239 | -3.93 | 0.27743 | -0.294 |
| DOCK2 | ILMN_1799725 | 0.0065 | 0.000576 | -3.65 | -0.54145 | -0.294 |
| RPIA | ILMN_1714809 | 0.032 | 0.00533 | -2.9 | -2.583 | -0.294 |
| OXR1 | ILMN_1737462 | 0.0532 | 0.0111 | -2.63 | -3.23882 | -0.294 |
| SEC22C | ILMN_1664051 | 0.0922 | 0.0244 | -2.31 | -3.93184 | -0.294 |
| ZNHIT1 | ILMN_1741491 | 0.00272 | 0.000173 | -4.03 | 0.57935 | -0.295 |
| ZNF845 | ILMN_3237579 | 0.00312 | 0.00021 | -3.97 | 0.3992 | -0.295 |
| ARHGEF3 | ILMN_1781010 | 0.00788 | 0.000753 | -3.57 | -0.78998 | -0.295 |
| CPSF2 | ILMN_2144116 | 0.0224 | 0.00326 | -3.08 | -2.137 | -0.295 |
| EFNA4 | ILMN_1665696 | 0.0569 | 0.0122 | -2.59 | -3.3224 | -0.295 |
| MAP2K2 | ILMN_1657968 | 0.00462 | 0.000354 | -3.81 | -0.08851 | -0.296 |
| RAB24 | ILMN_2379718 | 0.00771 | 0.000727 | -3.58 | -0.75828 | -0.296 |
| DHX40 | ILMN_2325008 | 0.0111 | 0.00121 | -3.41 | -1.22495 | -0.296 |
| UTP23 | ILMN_1774860 | 0.0198 | 0.00271 | -3.14 | -1.96987 | -0.296 |
| CACNB4 | ILMN_1673503 | 0.0667 | 0.0153 | -2.5 | -3.52464 | -0.296 |
| ZFP91 | ILMN_1703053 | 0.071 | 0.0167 | -2.47 | -3.60047 | -0.296 |
| PLOD1 | ILMN_1684391 | 0.0915 | 0.0241 | -2.32 | -3.92213 | -0.296 |
| HIBCH | ILMN_1656977 | 0.0985 | 0.027 | -2.27 | -4.01746 | -0.296 |
| BRWD3 | ILMN_1757794 | 0.000863 | 3.67E-05 | -4.49 | 2.03676 | -0.297 |
| CYP4F3 | ILMN_1736190 | 0.00898 | 0.000903 | -3.51 | -0.95898 | -0.297 |
| ZNF140 | ILMN_1727923 | 0.0152 | 0.00187 | -3.27 | -1.62856 | -0.297 |
| MCOLN2 | ILMN_1660462 | 0.0327 | 0.0055 | -2.89 | -2.61069 | -0.297 |
| IL18BP | ILMN_1653575 | 0.0489 | 0.00979 | -2.68 | -3.1278 | -0.297 |
| GTF2E1 | ILMN_1655921 | 0.0716 | 0.0169 | -2.46 | -3.61115 | -0.297 |
| CBLN3 | ILMN_2053829 | 0.097 | 0.0263 | -2.28 | -3.9975 | -0.297 |
| ATP6V0A1 | ILMN_1752579 | 0.113 | 0.0329 | -2.19 | -4.18981 | -0.297 |
| CEP19 | ILMN_1873107 | 0.00138 | 6.97E-05 | -4.3 | 1.43351 | -0.298 |
| NCL | ILMN_2121437 | 0.00352 | 0.000243 | -3.92 | 0.26392 | -0.298 |
| UBN2 | ILMN_1685855 | 0.105 | 0.0294 | -2.24 | -4.09237 | -0.298 |
| GIN1 | ILMN_1814622 | 0.0155 | 0.00192 | -3.26 | -1.65402 | -0.299 |
| CTCF | ILMN_1786015 | 0.0229 | 0.00336 | -3.07 | -2.16292 | -0.299 |
| JAZF1 | ILMN_1682727 | 0.0231 | 0.00341 | -3.06 | -2.17827 | -0.299 |
| MTMR4 | ILMN_1771019 | 0.0529 | 0.011 | -2.63 | -3.23189 | -0.299 |
| CARS2 | ILMN_1765222 | 0.138 | 0.0443 | -2.06 | -4.44197 | -0.299 |
| ZNF585A | ILMN_1764415 | 8.75E-05 | 1.45E-06 | -5.4 | 5.10226 | -0.3 |
| ARHGAP4 | ILMN_1770824 | 0.00643 | 0.000561 | -3.66 | -0.51728 | -0.3 |
| PIGX | ILMN_1769508 | 0.0129 | 0.00147 | -3.35 | -1.40583 | -0.3 |
| FAM110A | ILMN_1782095 | 0.0148 | 0.00179 | -3.28 | -1.58666 | -0.3 |
| SGF29 | ILMN_1701477 | 0.0249 | 0.0038 | -3.02 | -2.27626 | -0.3 |
| INPP5K | ILMN_1712423 | 0.0442 | 0.00841 | -2.73 | -2.99187 | -0.3 |
| ARFIP1 | ILMN_1768399 | 0.0932 | 0.0248 | -2.31 | -3.94501 | -0.3 |
| VDAC1 | ILMN_2175601 | 0.00028 | 7.66E-06 | -4.94 | 3.52045 | -0.301 |
| TMEM55A | ILMN_1752117 | 0.0235 | 0.00349 | -3.05 | -2.19874 | -0.301 |
| COBLL1 | ILMN_1761260 | 0.0735 | 0.0176 | -2.45 | -3.64782 | -0.301 |
| NUTF2 | ILMN_1655046 | 0.0952 | 0.0255 | -2.3 | -3.96999 | -0.301 |
| RBPJ | ILMN_1708537 | 0.000519 | 1.87E-05 | -4.68 | 2.67482 | -0.302 |
| PSMC4 | ILMN_1687887 | 0.00345 | 0.000237 | -3.93 | 0.28623 | -0.302 |
| PTS | ILMN_2162328 | 0.00398 | 0.000285 | -3.87 | 0.11398 | -0.302 |
| PCCB | ILMN_1761010 | 0.00883 | 0.000881 | -3.52 | -0.93553 | -0.302 |
| SPAG7 | ILMN_1684446 | 0.0139 | 0.00164 | -3.31 | -1.50722 | -0.302 |
| MVD | ILMN_1657550 | 0.0469 | 0.00915 | -2.7 | -3.06751 | -0.302 |
| SCAMP1 | ILMN_1729058 | 0.0649 | 0.0148 | -2.52 | -3.49174 | -0.302 |
| VPS11 | ILMN_1717781 | 0.102 | 0.0284 | -2.25 | -4.062 | -0.302 |
| SPAG1 | ILMN_1712773 | 0.145 | 0.0475 | -2.03 | -4.50069 | -0.302 |
| PEX2 | ILMN_1743299 | 0.000463 | 1.58E-05 | -4.73 | 2.83332 | -0.303 |
| RASSF5 | ILMN_1669189 | 0.000639 | 2.49E-05 | -4.6 | 2.40447 | -0.303 |
| RAB11FIP2 | ILMN_2072541 | 0.00689 | 0.000625 | -3.63 | -0.61709 | -0.303 |
| PAICS | ILMN_2392546 | 0.00783 | 0.000746 | -3.57 | -0.78233 | -0.303 |
| MILR1 | ILMN_1747347 | 0.0494 | 0.00998 | -2.67 | -3.14457 | -0.303 |
| SIPA1 | ILMN_1682930 | 0.00131 | 6.46E-05 | -4.32 | 1.50517 | -0.304 |
| BTN2A1 | ILMN_2364852 | 0.00234 | 0.000141 | -4.09 | 0.77016 | -0.304 |
| GLTP | ILMN_1764380 | 0.00553 | 0.000453 | -3.73 | -0.31899 | -0.304 |
| RGL4 | ILMN_1663422 | 0.0194 | 0.00264 | -3.15 | -1.94597 | -0.304 |
| MLLT6 | ILMN_1718734 | 0.0274 | 0.0043 | -2.98 | -2.38912 | -0.304 |
| MLEC | ILMN_1657495 | 0.0279 | 0.00441 | -2.97 | -2.41162 | -0.304 |
| RAB11FIP1 | ILMN_1692219 | 0.036 | 0.0063 | -2.84 | -2.7334 | -0.304 |
| ABHD8 | ILMN_1712707 | 0.064 | 0.0144 | -2.53 | -3.47098 | -0.304 |
| ARRDC2 | ILMN_1655612 | 0.0648 | 0.0147 | -2.52 | -3.48987 | -0.304 |
| PKN1 | ILMN_2367707 | 0.105 | 0.0294 | -2.24 | -4.09182 | -0.304 |
| COPRS | ILMN_1752947 | 0.0074 | 0.000692 | -3.59 | -0.7117 | -0.305 |
| CHCHD5 | ILMN_1797530 | 0.00785 | 0.000749 | -3.57 | -0.78607 | -0.305 |
| HSPA5 | ILMN_1773865 | 0.0279 | 0.00442 | -2.97 | -2.41337 | -0.305 |
| SNHG12 | ILMN_3249059 | 0.0485 | 0.00965 | -2.68 | -3.11444 | -0.305 |
| MRPL49 | ILMN_1681324 | 0.0563 | 0.012 | -2.6 | -3.30891 | -0.305 |
| SGK1 | ILMN_3305938 | 0.0997 | 0.0274 | -2.27 | -4.03049 | -0.305 |
| CCDC12 | ILMN_1725071 | 0.128 | 0.0397 | -2.11 | -4.34839 | -0.305 |
| DNAJC4 | ILMN_3307827 | 0.00558 | 0.00046 | -3.72 | -0.33216 | -0.306 |
| ZSCAN18 | ILMN_1654946 | 0.00646 | 0.000567 | -3.66 | -0.52737 | -0.306 |
| CUX1 | ILMN_1687567 | 0.0121 | 0.00136 | -3.37 | -1.33595 | -0.306 |
| CXCL1 | ILMN_1787897 | 0.0188 | 0.00252 | -3.16 | -1.90234 | -0.306 |
| TCEAL9 | ILMN_1679838 | 0.0239 | 0.00358 | -3.04 | -2.22088 | -0.306 |
| TNFSF14 | ILMN_1661343 | 0.08 | 0.0199 | -2.4 | -3.75364 | -0.306 |
| TRNT1 | ILMN_1655924 | 0.123 | 0.0373 | -2.13 | -4.29668 | -0.306 |
| SEC61G | ILMN_1787026 | 8.05E-06 | 5.29E-08 | -6.29 | 8.26173 | -0.307 |
| ERLEC1 | ILMN_1724376 | 0.00125 | 6.00E-05 | -4.34 | 1.57403 | -0.307 |
| MTOR | ILMN_1769031 | 0.00155 | 8.15E-05 | -4.25 | 1.28578 | -0.307 |
| ST6GAL1 | ILMN_1756501 | 0.00639 | 0.000555 | -3.67 | -0.50674 | -0.307 |
| UBAC1 | ILMN_1807044 | 0.0168 | 0.00215 | -3.22 | -1.75806 | -0.307 |
| TRAK2 | ILMN_1781691 | 0.0246 | 0.00373 | -3.03 | -2.25823 | -0.307 |
| OLR1 | ILMN_1723035 | 0.0583 | 0.0126 | -2.58 | -3.35109 | -0.307 |
| CYC1 | ILMN_1815115 | 0.00147 | 7.55E-05 | -4.28 | 1.35843 | -0.308 |
| ZNF317 | ILMN_1680347 | 0.00634 | 0.000545 | -3.67 | -0.49129 | -0.308 |
| SEC23A | ILMN_1790311 | 0.0129 | 0.00148 | -3.35 | -1.41442 | -0.308 |
| PICK1 | ILMN_2373982 | 0.0941 | 0.0251 | -2.3 | -3.95726 | -0.308 |
| ADAM8 | ILMN_1708348 | 0.000798 | 3.32E-05 | -4.52 | 2.132 | -0.309 |
| TUSC2 | ILMN_1804329 | 0.00294 | 0.000193 | -3.99 | 0.47597 | -0.309 |
| CYB561D2 | ILMN_1738718 | 0.0193 | 0.00263 | -3.15 | -1.94148 | -0.309 |
| ZBTB7B | ILMN_1736954 | 0.0215 | 0.00306 | -3.1 | -2.07848 | -0.309 |
| TRAPPC13 | ILMN_1726930 | 0.0399 | 0.00728 | -2.79 | -2.86327 | -0.309 |
| METTL14 | ILMN_2124523 | 0.05 | 0.0102 | -2.66 | -3.1598 | -0.309 |
| SLAIN2 | ILMN_3234775 | 0.0584 | 0.0126 | -2.58 | -3.35316 | -0.309 |
| PLXNB2 | ILMN_1763447 | 0.0802 | 0.02 | -2.4 | -3.75728 | -0.309 |
| SLC35B1 | ILMN_1727840 | 2.23E-05 | 2.33E-07 | -5.89 | 6.8475 | -0.31 |
| DNAJB14 | ILMN_1657619 | 0.0124 | 0.0014 | -3.36 | -1.36375 | -0.31 |
| TACC1 | ILMN_1770084 | 0.016 | 0.00202 | -3.24 | -1.69802 | -0.31 |
| KIAA0513 | ILMN_1693233 | 0.0591 | 0.0129 | -2.57 | -3.36971 | -0.31 |
| KANSL1L | ILMN_3251616 | 0.0844 | 0.0215 | -2.37 | -3.82177 | -0.31 |
| CD58 | ILMN_1785268 | 4.36E-05 | 5.46E-07 | -5.66 | 6.03355 | -0.311 |
| ANKRD54 | ILMN_1766309 | 0.0306 | 0.00498 | -2.93 | -2.52139 | -0.311 |
| KDM1B | ILMN_1781537 | 0.0322 | 0.00539 | -2.9 | -2.59304 | -0.311 |
| NTMT1 | ILMN_2170515 | 0.0324 | 0.00542 | -2.89 | -2.59783 | -0.311 |
| DCUN1D5 | ILMN_2124352 | 0.0374 | 0.00667 | -2.82 | -2.78414 | -0.311 |
| RANBP3 | ILMN_1699632 | 0.0565 | 0.0121 | -2.6 | -3.3138 | -0.311 |
| DENND5A | ILMN_3245413 | 1.29E-05 | 1.03E-07 | -6.11 | 7.62869 | -0.312 |
| ZNF160 | ILMN_1777049 | 0.00654 | 0.00058 | -3.65 | -0.54835 | -0.312 |
| SIRT6 | ILMN_1654246 | 0.0227 | 0.00331 | -3.07 | -2.15064 | -0.312 |
| PSMB10 | ILMN_1683026 | 0.0268 | 0.00416 | -2.99 | -2.3572 | -0.312 |
| IMPDH1 | ILMN_2388363 | 0.0878 | 0.0229 | -2.34 | -3.87427 | -0.312 |
| LCLAT1 | ILMN_3186473 | 0.0986 | 0.027 | -2.27 | -4.01988 | -0.312 |
| SEC61G | ILMN_2367020 | 2.18E-05 | 2.18E-07 | -5.91 | 6.90828 | -0.313 |
| PLAUR | ILMN_2408543 | 0.0202 | 0.00279 | -3.13 | -1.99548 | -0.313 |
| ZNF654 | ILMN_1744471 | 0.0212 | 0.00299 | -3.11 | -2.05917 | -0.313 |
| SCOC | ILMN_1768050 | 0.0341 | 0.00584 | -2.87 | -2.66522 | -0.313 |
| NAGK | ILMN_1716547 | 0.119 | 0.0356 | -2.15 | -4.25703 | -0.313 |
| MFAP1 | ILMN_1676799 | 0.000207 | 5.06E-06 | -5.05 | 3.91501 | -0.314 |
| UPF3B | ILMN_1798163 | 0.00189 | 0.000106 | -4.18 | 1.0413 | -0.314 |
| VBP1 | ILMN_1800612 | 0.00423 | 0.000313 | -3.85 | 0.02505 | -0.314 |
| SLC35B3 | ILMN_1682910 | 0.00638 | 0.000552 | -3.67 | -0.50207 | -0.314 |
| AKAP8 | ILMN_1741572 | 0.011 | 0.00119 | -3.42 | -1.20965 | -0.314 |
| PBX3 | ILMN_1810100 | 0.0234 | 0.00346 | -3.06 | -2.18989 | -0.314 |
| MYCBP2 | ILMN_1811972 | 0.0869 | 0.0225 | -2.35 | -3.86069 | -0.314 |
| CD58 | ILMN_2147517 | 2.44E-05 | 2.60E-07 | -5.86 | 6.74083 | -0.315 |
| LBHD1 | ILMN_1739345 | 0.000315 | 9.14E-06 | -4.89 | 3.35352 | -0.315 |
| IFNGR2 | ILMN_1764964 | 0.00093 | 4.11E-05 | -4.46 | 1.93203 | -0.315 |
| LINC00936 | ILMN_1795835 | 0.0197 | 0.00271 | -3.14 | -1.96706 | -0.315 |
| TERF1 | ILMN_1694847 | 0.0311 | 0.00509 | -2.92 | -2.54053 | -0.315 |
| CENPT | ILMN_3238375 | 0.0419 | 0.00781 | -2.76 | -2.9255 | -0.315 |
| FAR1 | ILMN_2143250 | 0.0469 | 0.00918 | -2.7 | -3.06983 | -0.315 |
| BHLHE40 | ILMN_1768534 | 0.00272 | 0.000174 | -4.03 | 0.57605 | -0.316 |
| PIPOX | ILMN_1739050 | 0.00311 | 0.000208 | -3.97 | 0.40722 | -0.316 |
| E4F1 | ILMN_1720287 | 0.00378 | 0.000265 | -3.9 | 0.1794 | -0.316 |
| GGA2 | ILMN_1686152 | 0.0813 | 0.0204 | -2.39 | -3.77453 | -0.316 |
| PIK3CB | ILMN_1763347 | 0.0195 | 0.00266 | -3.15 | -1.95179 | -0.317 |
| CDC42SE2 | ILMN_2313926 | 0.0242 | 0.00364 | -3.04 | -2.23726 | -0.317 |
| SNRPC | ILMN_1741997 | 0.0336 | 0.00571 | -2.88 | -2.64396 | -0.317 |
| SGK1 | ILMN_3229324 | 0.105 | 0.0295 | -2.24 | -4.09415 | -0.317 |
| BMI1 | ILMN_1700915 | 0.00205 | 0.000118 | -4.14 | 0.93714 | -0.318 |
| ZNF146 | ILMN_2054442 | 0.0066 | 0.000586 | -3.65 | -0.55772 | -0.318 |
| ICOSLG | ILMN_1675671 | 0.022 | 0.00316 | -3.09 | -2.10877 | -0.318 |
| IGFL3 | ILMN_1679897 | 0.0259 | 0.00398 | -3.01 | -2.31793 | -0.318 |
| RPRD2 | ILMN_3238889 | 0.0953 | 0.0256 | -2.29 | -3.97194 | -0.318 |
| BRCC3 | ILMN_1697546 | 0.106 | 0.0298 | -2.23 | -4.1039 | -0.318 |
| CTDSP1 | ILMN_1728163 | 0.000281 | 7.76E-06 | -4.93 | 3.50793 | -0.319 |
| GPR108 | ILMN_2396571 | 0.000923 | 4.06E-05 | -4.46 | 1.94361 | -0.319 |
| AQR | ILMN_1717154 | 0.0181 | 0.00239 | -3.18 | -1.85244 | -0.319 |
| TMEM109 | ILMN_2092756 | 0.0521 | 0.0107 | -2.64 | -3.20843 | -0.319 |
| SERPINB9 | ILMN_1742052 | 0.118 | 0.0351 | -2.16 | -4.24401 | -0.319 |
| SMPD1 | ILMN_1757370 | 0.122 | 0.037 | -2.14 | -4.28932 | -0.319 |
| GAPT | ILMN_3242271 | 0.0172 | 0.00222 | -3.21 | -1.7875 | -0.32 |
| NR1D2 | ILMN_1676844 | 0.128 | 0.0394 | -2.11 | -4.3436 | -0.32 |
| MCM3 | ILMN_2224143 | 0.000338 | 1.01E-05 | -4.86 | 3.25773 | -0.321 |
| UAP1 | ILMN_1742461 | 0.00272 | 0.000174 | -4.03 | 0.57545 | -0.321 |
| ZNRD1 | ILMN_2398587 | 0.0111 | 0.0012 | -3.41 | -1.22193 | -0.321 |
| DHX33 | ILMN_1744308 | 0.0403 | 0.00739 | -2.78 | -2.87653 | -0.321 |
| CDC23 | ILMN_1799688 | 0.0787 | 0.0194 | -2.41 | -3.73233 | -0.321 |
| TCTN1 | ILMN_1685124 | 0.149 | 0.0493 | -2.01 | -4.5315 | -0.321 |
| IDH3G | ILMN_1802706 | 0.00271 | 0.000172 | -4.03 | 0.58293 | -0.322 |
| CDK13 | ILMN_1778557 | 0.0101 | 0.00107 | -3.45 | -1.11415 | -0.322 |
| SCO2 | ILMN_1701621 | 0.0442 | 0.00842 | -2.73 | -2.9932 | -0.322 |
| C1orf61 | ILMN_1759652 | 0.103 | 0.0287 | -2.25 | -4.07131 | -0.322 |
| WRNIP1 | ILMN_2332105 | 0.000239 | 6.15E-06 | -5 | 3.72946 | -0.323 |
| TSHZ3 | ILMN_1743933 | 0.000335 | 1.00E-05 | -4.86 | 3.2673 | -0.323 |
| GLE1 | ILMN_1694323 | 0.0525 | 0.0109 | -2.64 | -3.21958 | -0.323 |
| NKAP | ILMN_2067980 | 0.0618 | 0.0137 | -2.55 | -3.42508 | -0.323 |
| EIF5A | ILMN_1794522 | 0.000632 | 2.45E-05 | -4.61 | 2.42105 | -0.324 |
| PARVG | ILMN_1695851 | 0.00696 | 0.000632 | -3.62 | -0.62804 | -0.324 |
| MKRN2 | ILMN_2056760 | 0.0114 | 0.00125 | -3.4 | -1.26069 | -0.324 |
| AURKA | ILMN_2357438 | 0.0816 | 0.0205 | -2.39 | -3.77908 | -0.324 |
| GLT8D1 | ILMN_1713290 | 0.0829 | 0.021 | -2.38 | -3.79879 | -0.324 |
| CCT5 | ILMN_1706246 | 0.0872 | 0.0226 | -2.35 | -3.86496 | -0.324 |
| DRAM1 | ILMN_1669376 | 0.000953 | 4.24E-05 | -4.45 | 1.90201 | -0.325 |
| PTPRJ | ILMN_1731589 | 0.0117 | 0.0013 | -3.39 | -1.29124 | -0.325 |
| OPRL1 | ILMN_2400922 | 0.0129 | 0.00148 | -3.35 | -1.41279 | -0.325 |
| BCAP29 | ILMN_1697642 | 0.019 | 0.00255 | -3.16 | -1.91307 | -0.325 |
| ERGIC3 | ILMN_2359456 | 0.0374 | 0.00667 | -2.82 | -2.78397 | -0.325 |
| MUTYH | ILMN_1714438 | 0.0391 | 0.00709 | -2.8 | -2.83858 | -0.325 |
| ATP11C | ILMN_1661428 | 0.0457 | 0.00882 | -2.72 | -3.03426 | -0.325 |
| SIRT1 | ILMN_1739083 | 0.0482 | 0.00956 | -2.68 | -3.10639 | -0.325 |
| ENO3 | ILMN_1678904 | 0.049 | 0.00984 | -2.67 | -3.13246 | -0.325 |
| CPPED1 | ILMN_1662865 | 0.0491 | 0.00988 | -2.67 | -3.13613 | -0.325 |
| SEC23B | ILMN_1657483 | 0.000633 | 2.46E-05 | -4.6 | 2.41709 | -0.326 |
| LTN1 | ILMN_1760360 | 0.00135 | 6.68E-05 | -4.31 | 1.47373 | -0.326 |
| ZHX2 | ILMN_1792951 | 0.00255 | 0.000159 | -4.05 | 0.65693 | -0.326 |
| DCUN1D5 | ILMN_1726839 | 0.00463 | 0.000356 | -3.8 | -0.09481 | -0.326 |
| TMEM222 | ILMN_1793632 | 0.00669 | 6.00E-04 | -3.64 | -0.58018 | -0.326 |
| KCNJ8 | ILMN_1735779 | 0.0152 | 0.00188 | -3.27 | -1.63281 | -0.326 |
| C1GALT1 | ILMN_1662578 | 0.0579 | 0.0125 | -2.58 | -3.34384 | -0.326 |
| CASS4 | ILMN_1678061 | 0.121 | 0.0365 | -2.14 | -4.27805 | -0.326 |
| TSC22D2 | ILMN_1685574 | 0.00471 | 0.000365 | -3.8 | -0.11841 | -0.327 |
| QTRT2 | ILMN_2155516 | 0.0101 | 0.00106 | -3.45 | -1.1109 | -0.327 |
| POM121C | ILMN_3235808 | 0.0103 | 0.00109 | -3.45 | -1.13617 | -0.327 |
| POLB | ILMN_1767894 | 0.0213 | 0.00303 | -3.1 | -2.06934 | -0.327 |
| LINC00294 | ILMN_3244521 | 0.0438 | 0.0083 | -2.74 | -2.98052 | -0.327 |
| CTSH | ILMN_1752451 | 0.0492 | 0.0099 | -2.67 | -3.13793 | -0.327 |
| TRIB3 | ILMN_1787815 | 0.146 | 0.048 | -2.02 | -4.50954 | -0.327 |
| RFXANK | ILMN_1780987 | 0.00181 | 9.98E-05 | -4.19 | 1.09611 | -0.328 |
| TIA1 | ILMN_1778691 | 0.0212 | 0.00301 | -3.1 | -2.06487 | -0.328 |
| NAAA | ILMN_1668605 | 0.0327 | 0.00551 | -2.89 | -2.61158 | -0.328 |
| SMS | ILMN_2225318 | 0.0442 | 0.0084 | -2.73 | -2.99129 | -0.328 |
| NAB1 | ILMN_1774617 | 0.00882 | 0.000878 | -3.52 | -0.93298 | -0.329 |
| DLEU7 | ILMN_3307752 | 0.00989 | 0.00104 | -3.46 | -1.08531 | -0.329 |
| CCDC97 | ILMN_1801600 | 0.0144 | 0.00172 | -3.29 | -1.55208 | -0.329 |
| RBM48 | ILMN_1742276 | 0.0361 | 0.00633 | -2.84 | -2.7368 | -0.329 |
| MNT | ILMN_1792910 | 0.00149 | 7.75E-05 | -4.27 | 1.33362 | -0.33 |
| PPP2R2D | ILMN_1805652 | 0.00848 | 0.000832 | -3.53 | -0.88259 | -0.33 |
| TPI1P2 | ILMN_1684114 | 0.00958 | 0.000993 | -3.48 | -1.04593 | -0.33 |
| NR3C1 | ILMN_2389347 | 0.0214 | 0.00304 | -3.1 | -2.07221 | -0.33 |
| BTN2A1 | ILMN_1783392 | 0.0232 | 0.00342 | -3.06 | -2.17999 | -0.33 |
| CD79B | ILMN_1785439 | 0.0405 | 0.00742 | -2.78 | -2.88001 | -0.33 |
| INPP5B | ILMN_1810116 | 0.0619 | 0.0137 | -2.54 | -3.42873 | -0.33 |
| ARHGAP12 | ILMN_2101810 | 0.0259 | 0.00399 | -3 | -2.31974 | -0.331 |
| SFI1 | ILMN_1763887 | 0.0678 | 0.0157 | -2.49 | -3.54433 | -0.331 |
| SLC37A1 | ILMN_1687495 | 0.0878 | 0.0228 | -2.34 | -3.87341 | -0.331 |
| PPIG | ILMN_2212156 | 0.136 | 0.0433 | -2.07 | -4.4223 | -0.331 |
| PRCP | ILMN_2367215 | 0.000407 | 1.30E-05 | -4.79 | 3.02046 | -0.332 |
| TOPBP1 | ILMN_1684929 | 0.0015 | 7.82E-05 | -4.27 | 1.32475 | -0.332 |
| CEP19 | ILMN_1665217 | 0.00479 | 0.000373 | -3.79 | -0.13833 | -0.332 |
| FAM98C | ILMN_1771870 | 0.0438 | 0.00828 | -2.74 | -2.97819 | -0.332 |
| BTN3A2 | ILMN_1700067 | 0.0519 | 0.0106 | -2.64 | -3.20233 | -0.332 |
| PRCP | ILMN_1769091 | 0.000237 | 5.98E-06 | -5.01 | 3.75536 | -0.333 |
| DDIT4 | ILMN_1661599 | 0.00204 | 0.000117 | -4.15 | 0.94523 | -0.333 |
| CFDP1 | ILMN_1800837 | 0.0139 | 0.00165 | -3.31 | -1.51377 | -0.333 |
| ZDHHC13 | ILMN_1684663 | 0.0141 | 0.00168 | -3.3 | -1.52926 | -0.333 |
| INTS6L | ILMN_2207419 | 0.0335 | 0.00568 | -2.88 | -2.6397 | -0.333 |
| DDX46 | ILMN_1727001 | 0.0449 | 0.0086 | -2.72 | -3.01164 | -0.333 |
| TCF20 | ILMN_1666173 | 0.0481 | 0.00951 | -2.69 | -3.10203 | -0.333 |
| SH3BP2 | ILMN_1801914 | 0.0549 | 0.0116 | -2.61 | -3.27772 | -0.333 |
| SNAPC4 | ILMN_1677484 | 0.0766 | 0.0187 | -2.42 | -3.70104 | -0.333 |
| ZNRD1 | ILMN_1692486 | 0.00164 | 8.84E-05 | -4.23 | 1.20945 | -0.334 |
| IRF2BP2 | ILMN_2394561 | 0.00944 | 0.000973 | -3.48 | -1.02745 | -0.334 |
| TNFAIP6 | ILMN_1785732 | 0.0175 | 0.00227 | -3.2 | -1.8065 | -0.334 |
| C16orf54 | ILMN_1751061 | 0.0188 | 0.00251 | -3.17 | -1.89943 | -0.334 |
| PKN1 | ILMN_2367710 | 0.0201 | 0.00279 | -3.13 | -1.99351 | -0.334 |
| SHPK | ILMN_2216918 | 0.0737 | 0.0177 | -2.44 | -3.6509 | -0.334 |
| CCDC47 | ILMN_1804522 | 0.00157 | 8.30E-05 | -4.25 | 1.26946 | -0.335 |
| AXIN1 | ILMN_1766185 | 0.00255 | 0.00016 | -4.05 | 0.65597 | -0.335 |
| PGRMC2 | ILMN_2195236 | 0.00425 | 0.000316 | -3.84 | 0.01765 | -0.335 |
| LYL1 | ILMN_2216582 | 0.00711 | 0.000651 | -3.61 | -0.65513 | -0.335 |
| EBAG9 | ILMN_1791896 | 0.0117 | 0.00129 | -3.39 | -1.28511 | -0.335 |
| OAZ2 | ILMN_3239460 | 0.0366 | 0.00645 | -2.83 | -2.75463 | -0.335 |
| YIF1B | ILMN_2363668 | 0.000819 | 3.45E-05 | -4.51 | 2.09727 | -0.336 |
| NIP7 | ILMN_1704305 | 0.00149 | 7.72E-05 | -4.27 | 1.33745 | -0.336 |
| SLC30A5 | ILMN_1664153 | 0.00526 | 0.000426 | -3.75 | -0.26073 | -0.336 |
| PIGH | ILMN_3251246 | 0.00563 | 0.000466 | -3.72 | -0.34564 | -0.336 |
| FANCD2 | ILMN_2235137 | 0.00602 | 0.000507 | -3.69 | -0.42393 | -0.336 |
| ZNF226 | ILMN_1692133 | 0.044 | 0.00834 | -2.74 | -2.98492 | -0.336 |
| GMCL1 | ILMN_2194627 | 0.0836 | 0.0213 | -2.37 | -3.81221 | -0.336 |
| KLHL18 | ILMN_1655608 | 0.0967 | 0.0262 | -2.28 | -3.99235 | -0.336 |
| RNF5P1 | ILMN_2052863 | 0.00463 | 0.000357 | -3.8 | -0.09812 | -0.337 |
| MLF2 | ILMN_1671885 | 0.00668 | 0.000599 | -3.64 | -0.57809 | -0.337 |
| TMEM189-UBE2V1 | ILMN_1749212 | 0.024 | 0.00359 | -3.04 | -2.22513 | -0.337 |
| RAB24 | ILMN_1799765 | 0.0325 | 0.00545 | -2.89 | -2.60201 | -0.337 |
| CCDC57 | ILMN_2140342 | 0.0607 | 0.0133 | -2.56 | -3.4015 | -0.337 |
| S100Z | ILMN_1795139 | 0.1 | 0.0276 | -2.26 | -4.03871 | -0.337 |
| PSMC4 | ILMN_2287888 | 0.000877 | 3.78E-05 | -4.48 | 2.00993 | -0.338 |
| ETNK1 | ILMN_1696028 | 0.00226 | 0.000134 | -4.11 | 0.82229 | -0.338 |
| SYK | ILMN_2059549 | 0.00647 | 0.000568 | -3.66 | -0.52885 | -0.338 |
| PLAUR | ILMN_2374340 | 0.00944 | 0.000973 | -3.48 | -1.02755 | -0.338 |
| NSF | ILMN_2330845 | 0.0117 | 0.00129 | -3.39 | -1.288 | -0.338 |
| HYAL2 | ILMN_1668283 | 0.033 | 0.00559 | -2.88 | -2.62501 | -0.338 |
| SLC40A1 | ILMN_1761833 | 0.144 | 0.0468 | -2.03 | -4.48905 | -0.338 |
| FCGR2B | ILMN_1660027 | 8.23E-05 | 1.33E-06 | -5.42 | 5.18413 | -0.339 |
| XKR8 | ILMN_1675556 | 0.0215 | 0.00308 | -3.1 | -2.08457 | -0.339 |
| LOC100288893 | ILMN_1845157 | 0.0252 | 0.00385 | -3.02 | -2.28699 | -0.339 |
| PMS2CL | ILMN_1673337 | 0.0664 | 0.0152 | -2.5 | -3.51952 | -0.339 |
| HLA-DRB3 | ILMN_1717261 | 0.000261 | 7.00E-06 | -4.96 | 3.60665 | -0.34 |
| REL | ILMN_1766085 | 0.00248 | 0.000154 | -4.06 | 0.691 | -0.34 |
| ABR | ILMN_1672878 | 0.0029 | 0.00019 | -4 | 0.49133 | -0.34 |
| ZNF12 | ILMN_1784577 | 0.00436 | 0.000328 | -3.83 | -0.01823 | -0.34 |
| C7orf26 | ILMN_1730048 | 0.00623 | 0.000529 | -3.68 | -0.46281 | -0.34 |
| GAPT | ILMN_1675191 | 0.00673 | 0.000605 | -3.64 | -0.5883 | -0.34 |
| DENND4B | ILMN_3245066 | 2.12E-05 | 2.08E-07 | -5.92 | 6.95256 | -0.341 |
| SGMS1 | ILMN_1740505 | 0.00503 | 0.000401 | -3.77 | -0.20627 | -0.341 |
| S100A4 | ILMN_1684306 | 0.0255 | 0.00391 | -3.01 | -2.30117 | -0.341 |
| RUFY1 | ILMN_2310968 | 0.0471 | 0.00923 | -2.7 | -3.07461 | -0.341 |
| ECHS1 | ILMN_1718132 | 0.0565 | 0.0121 | -2.6 | -3.31361 | -0.341 |
| TAGAP | ILMN_1676408 | 0.124 | 0.0379 | -2.13 | -4.30882 | -0.341 |
| TIGAR | ILMN_1791792 | 0.000343 | 1.04E-05 | -4.85 | 3.23324 | -0.342 |
| ARAP3 | ILMN_3240997 | 0.0021 | 0.000122 | -4.13 | 0.90969 | -0.342 |
| ZCCHC3 | ILMN_1786852 | 0.0341 | 0.00586 | -2.87 | -2.66839 | -0.342 |
| BCL9L | ILMN_1743966 | 0.038 | 0.00681 | -2.81 | -2.80229 | -0.342 |
| MAP7 | ILMN_1712719 | 0.0848 | 0.0217 | -2.36 | -3.82787 | -0.342 |
| UBN2 | ILMN_3248928 | 0.134 | 0.0424 | -2.08 | -4.40498 | -0.342 |
| RAB11FIP1 | ILMN_2409596 | 0.00819 | 0.000794 | -3.55 | -0.83965 | -0.343 |
| RALGAPA1 | ILMN_1741391 | 0.011 | 0.00119 | -3.42 | -1.21504 | -0.343 |
| SGF29 | ILMN_1684789 | 0.0139 | 0.00163 | -3.31 | -1.50418 | -0.343 |
| AIG1 | ILMN_1797974 | 0.043 | 0.00809 | -2.75 | -2.95696 | -0.343 |
| GAR1 | ILMN_3263329 | 0.0521 | 0.0107 | -2.64 | -3.20993 | -0.343 |
| AKR7A2 | ILMN_1677043 | 0.000261 | 7.00E-06 | -4.96 | 3.60646 | -0.344 |
| DEGS1 | ILMN_1667430 | 0.000828 | 3.49E-05 | -4.5 | 2.08554 | -0.344 |
| CLDN15 | ILMN_1682226 | 0.00151 | 7.92E-05 | -4.26 | 1.31304 | -0.344 |
| PIK3C2A | ILMN_2166093 | 0.0189 | 0.00253 | -3.16 | -1.90561 | -0.344 |
| XPO7 | ILMN_2174884 | 0.0221 | 0.00321 | -3.08 | -2.12334 | -0.344 |
| DFFA | ILMN_1667213 | 0.0304 | 0.00495 | -2.93 | -2.51511 | -0.344 |
| NME1 | ILMN_1741133 | 0.0459 | 0.00887 | -2.71 | -3.03965 | -0.344 |
| AZIN1 | ILMN_2327994 | 0.00548 | 0.000449 | -3.73 | -0.30956 | -0.345 |
| CDKN1A | ILMN_1784602 | 0.0201 | 0.00277 | -3.13 | -1.98861 | -0.345 |
| ZNF559 | ILMN_1677785 | 0.0239 | 0.00357 | -3.04 | -2.22022 | -0.345 |
| AMPD2 | ILMN_1786899 | 0.0362 | 0.00637 | -2.84 | -2.7426 | -0.345 |
| SLC2A14 | ILMN_1668865 | 0.0395 | 0.00718 | -2.79 | -2.8508 | -0.345 |
| SORD | ILMN_2285375 | 0.0492 | 0.00992 | -2.67 | -3.13897 | -0.345 |
| TAPT1 | ILMN_1693882 | 0.0679 | 0.0157 | -2.49 | -3.54644 | -0.345 |
| LINC00938 | ILMN_1780773 | 0.00055 | 2.03E-05 | -4.66 | 2.5984 | -0.346 |
| WDR53 | ILMN_1724986 | 0.0279 | 0.00441 | -2.97 | -2.41102 | -0.346 |
| EIF2B1 | ILMN_1753716 | 0.0296 | 0.00478 | -2.94 | -2.48373 | -0.346 |
| MYB | ILMN_1711894 | 0.039 | 0.00705 | -2.8 | -2.83363 | -0.346 |
| NSUN5 | ILMN_1751958 | 0.0712 | 0.0168 | -2.47 | -3.60461 | -0.346 |
| RASSF1 | ILMN_1734205 | 0.085 | 0.0218 | -2.36 | -3.83231 | -0.346 |
| PLCG2 | ILMN_1815719 | 0.00526 | 0.000425 | -3.75 | -0.25885 | -0.347 |
| USP42 | ILMN_1752575 | 0.0288 | 0.00461 | -2.95 | -2.45046 | -0.347 |
| RASSF1 | ILMN_1683239 | 0.072 | 0.0171 | -2.46 | -3.62074 | -0.347 |
| RNF138 | ILMN_1655165 | 0.0187 | 0.0025 | -3.17 | -1.89576 | -0.348 |
| EIF2B5 | ILMN_1688534 | 0.0762 | 0.0186 | -2.43 | -3.69274 | -0.348 |
| DSE | ILMN_1706498 | 2.10E-05 | 2.04E-07 | -5.93 | 6.97301 | -0.349 |
| DLGAP4 | ILMN_1754842 | 0.00434 | 0.000326 | -3.83 | -0.01129 | -0.349 |
| SLCO3A1 | ILMN_1678928 | 0.0121 | 0.00136 | -3.37 | -1.33555 | -0.349 |
| HINT1 | ILMN_1807710 | 0.0297 | 0.00479 | -2.94 | -2.48642 | -0.349 |
| CCT7 | ILMN_1662954 | 0.0522 | 0.0108 | -2.64 | -3.21115 | -0.349 |
| FLVCR1-AS1 | ILMN_3301042 | 0.0973 | 0.0265 | -2.28 | -4.00139 | -0.349 |
| PRKCD | ILMN_1801105 | 0.000797 | 3.31E-05 | -4.52 | 2.13659 | -0.35 |
| SLC15A3 | ILMN_2085862 | 0.00369 | 0.000257 | -3.91 | 0.20814 | -0.35 |
| NIPSNAP3A | ILMN_1700159 | 0.0317 | 0.00526 | -2.91 | -2.56974 | -0.35 |
| RPP25L | ILMN_2391345 | 0.0548 | 0.0116 | -2.61 | -3.27611 | -0.35 |
| SSBP3 | ILMN_1814165 | 0.0655 | 0.015 | -2.51 | -3.50403 | -0.35 |
| C9orf66 | ILMN_1717248 | 0.0399 | 0.00727 | -2.79 | -2.86092 | -0.351 |
| TOX2 | ILMN_2082209 | 0.0089 | 0.000893 | -3.51 | -0.94839 | -0.352 |
| SLC9A3R1 | ILMN_1680925 | 0.017 | 0.00219 | -3.21 | -1.77229 | -0.352 |
| VEGFB | ILMN_1726981 | 0.0206 | 0.00288 | -3.12 | -2.02235 | -0.352 |
| CEP57 | ILMN_1791568 | 0.0517 | 0.0106 | -2.65 | -3.19746 | -0.352 |
| GMCL1 | ILMN_1670532 | 0.0823 | 0.0207 | -2.38 | -3.7899 | -0.352 |
| COPS6 | ILMN_1764431 | 0.131 | 0.0412 | -2.09 | -4.38057 | -0.352 |
| TMEM189 | ILMN_2162989 | 0.00034 | 1.02E-05 | -4.85 | 3.24558 | -0.353 |
| SUCLG1 | ILMN_1779616 | 0.00245 | 0.000151 | -4.07 | 0.70882 | -0.353 |
| LST1 | ILMN_1718936 | 0.00775 | 0.000731 | -3.58 | -0.76346 | -0.353 |
| SLC2A1 | ILMN_1659027 | 0.00918 | 0.000933 | -3.5 | -0.98833 | -0.353 |
| MBD1 | ILMN_1683595 | 0.0107 | 0.00115 | -3.43 | -1.18338 | -0.353 |
| ZNF318 | ILMN_1792305 | 0.0246 | 0.00372 | -3.03 | -2.25699 | -0.353 |
| PTPN6 | ILMN_1664122 | 0.0463 | 0.00895 | -2.71 | -3.04792 | -0.353 |
| C17orf49 | ILMN_1763688 | 3.75E-06 | 1.84E-08 | -6.57 | 9.27053 | -0.354 |
| IRF2BP2 | ILMN_1671005 | 0.000897 | 3.90E-05 | -4.47 | 1.98174 | -0.354 |
| SLCO3A1 | ILMN_1706261 | 0.01 | 0.00106 | -3.46 | -1.10287 | -0.354 |
| HCK | ILMN_1791771 | 0.0119 | 0.00133 | -3.38 | -1.31448 | -0.354 |
| LUC7L | ILMN_1765371 | 0.0169 | 0.00217 | -3.22 | -1.76382 | -0.354 |
| COG5 | ILMN_2399877 | 0.0321 | 0.00536 | -2.9 | -2.58761 | -0.354 |
| TMC6 | ILMN_1794677 | 0.0606 | 0.0133 | -2.56 | -3.39906 | -0.354 |
| TMC8 | ILMN_2211534 | 0.101 | 0.0278 | -2.26 | -4.04391 | -0.354 |
| AZIN1 | ILMN_1656682 | 5.88E-05 | 8.04E-07 | -5.56 | 5.66503 | -0.355 |
| P4HB | ILMN_1719303 | 0.000191 | 4.39E-06 | -5.09 | 4.04829 | -0.355 |
| GNG2 | ILMN_1807925 | 0.00722 | 0.000667 | -3.61 | -0.67865 | -0.355 |
| C9orf85 | ILMN_1806758 | 0.0306 | 0.00499 | -2.92 | -2.5221 | -0.355 |
| VSTM1 | ILMN_1763455 | 0.00185 | 0.000103 | -4.18 | 1.06842 | -0.356 |
| CWF19L2 | ILMN_1668498 | 0.00781 | 0.000743 | -3.57 | -0.77829 | -0.356 |
| CNOT10 | ILMN_2078334 | 0.0148 | 0.00179 | -3.28 | -1.58695 | -0.356 |
| GID4 | ILMN_2113016 | 0.0198 | 0.00272 | -3.14 | -1.97165 | -0.356 |
| CLPP | ILMN_1725705 | 0.0387 | 0.00696 | -2.8 | -2.82216 | -0.356 |
| TMEM51 | ILMN_1674985 | 0.003 | 0.000199 | -3.99 | 0.44971 | -0.357 |
| ABI3 | ILMN_1755658 | 0.0205 | 0.00286 | -3.12 | -2.01685 | -0.357 |
| PMS2CL | ILMN_2262198 | 0.0688 | 0.016 | -2.48 | -3.56431 | -0.357 |
| RPP21 | ILMN_2186597 | 0.000129 | 2.49E-06 | -5.25 | 4.58927 | -0.358 |
| NCOA4 | ILMN_1773906 | 0.00399 | 0.000287 | -3.87 | 0.10736 | -0.358 |
| POLR2I | ILMN_1720542 | 0.00413 | 0.000303 | -3.86 | 0.05668 | -0.358 |
| MRPL42P5 | ILMN_2042343 | 0.00926 | 0.000946 | -3.49 | -1.00104 | -0.358 |
| HOPX | ILMN_2316236 | 0.116 | 0.0344 | -2.17 | -4.22795 | -0.358 |
| LGALS9C | ILMN_1766184 | 0.139 | 0.0449 | -2.05 | -4.45347 | -0.358 |
| SNN | ILMN_1788251 | 0.000207 | 5.07E-06 | -5.05 | 3.91259 | -0.359 |
| RNF19B | ILMN_1682081 | 0.000494 | 1.75E-05 | -4.7 | 2.73686 | -0.359 |
| DENND4A | ILMN_1759327 | 0.00431 | 0.000323 | -3.84 | -0.00278 | -0.359 |
| BNIP3L | ILMN_1718961 | 0.00488 | 0.000383 | -3.78 | -0.16283 | -0.359 |
| MEF2D | ILMN_1763228 | 0.0139 | 0.00165 | -3.31 | -1.51378 | -0.359 |
| RINL | ILMN_1790962 | 0.0168 | 0.00215 | -3.22 | -1.75874 | -0.359 |
| ABCB7 | ILMN_1687840 | 0.019 | 0.00257 | -3.16 | -1.91978 | -0.359 |
| WNK1 | ILMN_1753165 | 0.0266 | 0.00413 | -2.99 | -2.35234 | -0.359 |
| PRSS53 | ILMN_2133187 | 0.0423 | 0.00791 | -2.76 | -2.9366 | -0.359 |
| MIR155HG | ILMN_3248910 | 0.0489 | 0.00978 | -2.68 | -3.12693 | -0.359 |
| PRDX1 | ILMN_2366391 | 0.0723 | 0.0172 | -2.46 | -3.62755 | -0.359 |
| HIGD1A | ILMN_1661799 | 0.000416 | 1.36E-05 | -4.77 | 2.97953 | -0.36 |
| MAP3K6 | ILMN_1694539 | 0.0131 | 0.00151 | -3.34 | -1.43353 | -0.36 |
| RPUSD1 | ILMN_1683082 | 0.0399 | 0.00727 | -2.79 | -2.86181 | -0.36 |
| CTDSPL2 | ILMN_1665655 | 0.00792 | 0.000759 | -3.56 | -0.79771 | -0.361 |
| SET | ILMN_1742238 | 0.015 | 0.00183 | -3.27 | -1.60835 | -0.361 |
| COG3 | ILMN_1776154 | 0.0167 | 0.00212 | -3.22 | -1.74565 | -0.361 |
| NUDCD2 | ILMN_1751589 | 0.0325 | 0.00545 | -2.89 | -2.60249 | -0.361 |
| HNMT | ILMN_1790881 | 0.00282 | 0.000184 | -4.01 | 0.52124 | -0.362 |
| SSBP3 | ILMN_2361163 | 0.0278 | 0.00439 | -2.97 | -2.40659 | -0.362 |
| CDH2 | ILMN_1779228 | 0.0757 | 0.0184 | -2.43 | -3.68428 | -0.362 |
| SERPINB6 | ILMN_1712400 | 2.43E-05 | 2.57E-07 | -5.87 | 6.75046 | -0.363 |
| SKIV2L2 | ILMN_1651513 | 0.000198 | 4.69E-06 | -5.07 | 3.98563 | -0.363 |
| PEA15 | ILMN_1771376 | 0.000701 | 2.81E-05 | -4.57 | 2.29108 | -0.363 |
| PHF2 | ILMN_1720476 | 0.000923 | 4.05E-05 | -4.46 | 1.94576 | -0.363 |
| C17orf58 | ILMN_2398926 | 0.00195 | 0.00011 | -4.17 | 1.00614 | -0.363 |
| LINC00672 | ILMN_1916702 | 0.0271 | 0.00423 | -2.98 | -2.37405 | -0.363 |
| FAM60A | ILMN_3196019 | 0.0406 | 0.00745 | -2.78 | -2.88332 | -0.363 |
| SMARCB1 | ILMN_1758823 | 5.94E-05 | 8.19E-07 | -5.55 | 5.64655 | -0.364 |
| CHD9 | ILMN_1762972 | 0.00329 | 0.000223 | -3.95 | 0.34411 | -0.364 |
| ZNF641 | ILMN_1731666 | 0.0073 | 0.00068 | -3.6 | -0.69556 | -0.364 |
| NUP160 | ILMN_1652989 | 8.68E-05 | 1.43E-06 | -5.4 | 5.11806 | -0.365 |
| RBM34 | ILMN_1661485 | 0.000331 | 9.87E-06 | -4.87 | 3.28067 | -0.365 |
| DDX5 | ILMN_1819304 | 0.0296 | 0.00476 | -2.94 | -2.48065 | -0.365 |
| SS18L1 | ILMN_1676625 | 0.0322 | 0.0054 | -2.9 | -2.59408 | -0.365 |
| SFR1 | ILMN_1671905 | 0.0047 | 0.000365 | -3.8 | -0.1175 | -0.366 |
| NCF1C | ILMN_2112988 | 0.0114 | 0.00124 | -3.4 | -1.25268 | -0.366 |
| MESDC1 | ILMN_1781565 | 0.0304 | 0.00495 | -2.93 | -2.51529 | -0.366 |
| ARL5B | ILMN_2120022 | 0.0318 | 0.00528 | -2.9 | -2.57443 | -0.366 |
| USP37 | ILMN_1686319 | 0.0482 | 0.00958 | -2.68 | -3.10849 | -0.366 |
| NGLY1 | ILMN_1728779 | 0.00238 | 0.000145 | -4.08 | 0.74381 | -0.367 |
| CX3CR1 | ILMN_2088437 | 0.0418 | 0.00779 | -2.76 | -2.92342 | -0.367 |
| RNU2-1 | ILMN_2057836 | 0.0645 | 0.0146 | -2.52 | -3.48317 | -0.367 |
| FCER1A | ILMN_1688423 | 0.0874 | 0.0227 | -2.34 | -3.86863 | -0.367 |
| CEP85L | ILMN_2270100 | 0.146 | 0.048 | -2.02 | -4.50993 | -0.367 |
| HIGD1A | ILMN_1674522 | 0.000265 | 7.14E-06 | -4.96 | 3.5871 | -0.368 |
| BCORL1 | ILMN_1767015 | 0.00626 | 0.000533 | -3.68 | -0.46929 | -0.368 |
| KPNA2 | ILMN_1721868 | 0.00752 | 0.000705 | -3.59 | -0.729 | -0.368 |
| SEC23IP | ILMN_1690690 | 0.0104 | 0.00111 | -3.44 | -1.15122 | -0.368 |
| CCS | ILMN_2290998 | 0.0175 | 0.00229 | -3.2 | -1.81359 | -0.368 |
| NXT1 | ILMN_1760280 | 0.0196 | 0.00268 | -3.14 | -1.95958 | -0.368 |
| TOX2 | ILMN_3298423 | 0.0577 | 0.0124 | -2.58 | -3.33977 | -0.368 |
| FHOD1 | ILMN_1651776 | 0.00133 | 6.59E-05 | -4.32 | 1.4858 | -0.369 |
| HNRNPA1P10 | ILMN_2220283 | 0.00908 | 0.000918 | -3.5 | -0.97387 | -0.369 |
| RSF1 | ILMN_1668834 | 0.013 | 0.0015 | -3.34 | -1.42611 | -0.369 |
| FUNDC1 | ILMN_1728540 | 0.0175 | 0.00227 | -3.2 | -1.805 | -0.369 |
| PSMA2 | ILMN_2058512 | 0.0189 | 0.00254 | -3.16 | -1.90962 | -0.369 |
| USP48 | ILMN_1738572 | 0.0191 | 0.00259 | -3.16 | -1.9277 | -0.369 |
| GOLGA1 | ILMN_1807152 | 0.0195 | 0.00265 | -3.15 | -1.94925 | -0.369 |
| TBC1D10C | ILMN_1710434 | 0.0201 | 0.00279 | -3.13 | -1.99378 | -0.369 |
| CD47 | ILMN_2356991 | 0.028 | 0.00444 | -2.97 | -2.41734 | -0.369 |
| AGO1 | ILMN_1671326 | 0.0431 | 0.00812 | -2.75 | -2.96011 | -0.369 |
| BRI3BP | ILMN_1797693 | 0.147 | 0.0487 | -2.02 | -4.5221 | -0.369 |
| XPNPEP1 | ILMN_1669015 | 0.000129 | 2.49E-06 | -5.25 | 4.58959 | -0.37 |
| PFKP | ILMN_1805737 | 0.00303 | 0.000202 | -3.98 | 0.43678 | -0.37 |
| NELFCD | ILMN_1663954 | 0.0138 | 0.00162 | -3.32 | -1.4953 | -0.37 |
| BTBD7 | ILMN_1687743 | 0.0252 | 0.00385 | -3.02 | -2.28864 | -0.37 |
| MRPL50 | ILMN_1664833 | 0.00173 | 9.41E-05 | -4.21 | 1.15115 | -0.371 |
| PIEZO1 | ILMN_1752249 | 0.0133 | 0.00154 | -3.33 | -1.45352 | -0.371 |
| ARIH2OS | ILMN_3241670 | 0.0375 | 0.00669 | -2.82 | -2.78741 | -0.371 |
| FFAR3 | ILMN_1802735 | 0.0755 | 0.0183 | -2.43 | -3.68137 | -0.371 |
| GPR84 | ILMN_1785345 | 0.139 | 0.0447 | -2.05 | -4.44993 | -0.371 |
| ESCO1 | ILMN_1682233 | 0.0083 | 0.000808 | -3.54 | -0.85578 | -0.372 |
| CKS1B | ILMN_1719256 | 0.0118 | 0.00131 | -3.39 | -1.30032 | -0.372 |
| ARL5B | ILMN_1680465 | 0.0328 | 0.00553 | -2.89 | -2.61503 | -0.372 |
| THAP8 | ILMN_1790109 | 0.0355 | 0.00621 | -2.85 | -2.71976 | -0.372 |
| CDA | ILMN_1714592 | 0.0398 | 0.00726 | -2.79 | -2.85974 | -0.372 |
| HMBOX1 | ILMN_1843949 | 0.0418 | 0.00779 | -2.76 | -2.92347 | -0.372 |
| VEGFA | ILMN_1803882 | 0.0445 | 0.00848 | -2.73 | -2.99927 | -0.372 |
| TAF2 | ILMN_1694888 | 0.000168 | 3.68E-06 | -5.14 | 4.21745 | -0.373 |
| CSK | ILMN_1754121 | 0.000196 | 4.59E-06 | -5.08 | 4.00663 | -0.373 |
| PSMC4 | ILMN_2395389 | 0.000409 | 1.33E-05 | -4.78 | 2.99643 | -0.373 |
| TPI1 | ILMN_2181191 | 0.000835 | 3.53E-05 | -4.5 | 2.07565 | -0.373 |
| SNORD14A | ILMN_1799381 | 0.00361 | 0.00025 | -3.91 | 0.2353 | -0.373 |
| WNK1 | ILMN_1876924 | 0.00419 | 0.000309 | -3.85 | 0.03826 | -0.373 |
| ASB6 | ILMN_1806705 | 0.00837 | 0.000817 | -3.54 | -0.8659 | -0.373 |
| RNF166 | ILMN_1695356 | 0.0211 | 0.00298 | -3.11 | -2.05516 | -0.373 |
| RHBDF2 | ILMN_1691717 | 0.0522 | 0.0108 | -2.64 | -3.21188 | -0.373 |
| CCNG2 | ILMN_1747244 | 0.0547 | 0.0115 | -2.61 | -3.27284 | -0.373 |
| KDELR2 | ILMN_1724293 | 0.00012 | 2.24E-06 | -5.28 | 4.68687 | -0.374 |
| CUX1 | ILMN_2278636 | 0.000471 | 1.64E-05 | -4.72 | 2.80218 | -0.374 |
| TIA1 | ILMN_1712634 | 0.00335 | 0.000228 | -3.94 | 0.32332 | -0.374 |
| ATXN7L2 | ILMN_1664177 | 0.00649 | 0.000572 | -3.66 | -0.53529 | -0.374 |
| EMP3 | ILMN_1765446 | 0.0122 | 0.00138 | -3.37 | -1.34857 | -0.374 |
| SPAST | ILMN_1791547 | 0.0272 | 0.00427 | -2.98 | -2.38151 | -0.374 |
| NUP85 | ILMN_1669635 | 0.0434 | 0.00818 | -2.74 | -2.96725 | -0.374 |
| PRDM2 | ILMN_2258543 | 0.00973 | 0.00101 | -3.47 | -1.0656 | -0.375 |
| DNAJC9 | ILMN_1799516 | 0.0137 | 0.00161 | -3.32 | -1.49282 | -0.375 |
| PREB | ILMN_1733930 | 0.0169 | 0.00218 | -3.22 | -1.76768 | -0.375 |
| ZBTB45 | ILMN_1661484 | 0.00663 | 0.00059 | -3.65 | -0.56494 | -0.376 |
| ZMIZ2 | ILMN_1760718 | 0.0119 | 0.00133 | -3.38 | -1.3192 | -0.376 |
| HINFP | ILMN_2353697 | 0.0175 | 0.00227 | -3.2 | -1.80809 | -0.376 |
| AXIN1 | ILMN_1692967 | 0.0334 | 0.00566 | -2.88 | -2.6371 | -0.376 |
| PDIA3P1 | ILMN_2075440 | 6.90E-05 | 1.05E-06 | -5.49 | 5.41181 | -0.377 |
| SREBF1 | ILMN_2328986 | 0.0523 | 0.0108 | -2.64 | -3.21597 | -0.377 |
| RAB37 | ILMN_1796136 | 0.0576 | 0.0124 | -2.58 | -3.33779 | -0.377 |
| COL18A1 | ILMN_1806733 | 0.125 | 0.0384 | -2.12 | -4.31993 | -0.377 |
| REL | ILMN_2124064 | 0.000797 | 3.31E-05 | -4.52 | 2.13518 | -0.378 |
| SMC4 | ILMN_2330861 | 0.00107 | 4.93E-05 | -4.4 | 1.76048 | -0.378 |
| OXSM | ILMN_1683992 | 0.0071 | 0.000649 | -3.61 | -0.65318 | -0.378 |
| RNF5 | ILMN_2044927 | 0.0117 | 0.0013 | -3.39 | -1.29252 | -0.378 |
| KRI1 | ILMN_2347748 | 0.0125 | 0.00141 | -3.36 | -1.37186 | -0.378 |
| RNMT | ILMN_1769637 | 0.0138 | 0.00162 | -3.31 | -1.49899 | -0.378 |
| ADGRG5 | ILMN_1666902 | 0.0335 | 0.00569 | -2.88 | -2.64085 | -0.378 |
| UTP6 | ILMN_1778238 | 0.0352 | 0.00612 | -2.85 | -2.70696 | -0.378 |
| MYADM | ILMN_2308849 | 0.0384 | 0.0069 | -2.81 | -2.8145 | -0.378 |
| AP2A1 | ILMN_2359211 | 0.0598 | 0.0131 | -2.56 | -3.38603 | -0.378 |
| GNA15 | ILMN_1773963 | 0.00416 | 0.000305 | -3.85 | 0.04891 | -0.379 |
| RASGRP1 | ILMN_1768958 | 0.00428 | 0.000318 | -3.84 | 0.00998 | -0.379 |
| CNPY3 | ILMN_1812967 | 0.0101 | 0.00107 | -3.45 | -1.1111 | -0.379 |
| RAB11FIP4 | ILMN_1681467 | 0.0173 | 0.00224 | -3.21 | -1.79323 | -0.379 |
| NADK | ILMN_1758963 | 0.00667 | 0.000597 | -3.64 | -0.57555 | -0.38 |
| ERC1 | ILMN_1852384 | 0.0129 | 0.00148 | -3.35 | -1.41126 | -0.38 |
| ARHGAP21 | ILMN_1811592 | 0.0407 | 0.00751 | -2.78 | -2.89049 | -0.38 |
| JCHAIN | ILMN_2105441 | 0.0888 | 0.0233 | -2.33 | -3.88926 | -0.38 |
| ANAPC16 | ILMN_1738955 | 4.07E-05 | 5.02E-07 | -5.69 | 6.11298 | -0.381 |
| DVL3 | ILMN_2137464 | 0.00906 | 0.000913 | -3.5 | -0.96909 | -0.381 |
| GNMT | ILMN_1736238 | 0.0219 | 0.00314 | -3.09 | -2.10169 | -0.381 |
| GTPBP8 | ILMN_2393243 | 0.0272 | 0.00427 | -2.98 | -2.38129 | -0.381 |
| SLC25A29 | ILMN_1697544 | 0.0743 | 0.0179 | -2.44 | -3.65996 | -0.381 |
| COQ8A | ILMN_1731064 | 0.139 | 0.0445 | -2.06 | -4.4452 | -0.381 |
| TBC1D3F | ILMN_2068991 | 0.0317 | 0.00526 | -2.91 | -2.57051 | -0.382 |
| TBL2 | ILMN_1764489 | 0.13 | 0.0405 | -2.1 | -4.36694 | -0.382 |
| LRRC25 | ILMN_2150196 | 0.000388 | 1.22E-05 | -4.81 | 3.08044 | -0.383 |
| HCG26 | ILMN_3236741 | 0.0147 | 0.00176 | -3.29 | -1.57563 | -0.383 |
| MAPKAPK5 | ILMN_2322935 | 0.0206 | 0.00287 | -3.12 | -2.02147 | -0.383 |
| HDAC1 | ILMN_1727458 | 0.000426 | 1.41E-05 | -4.76 | 2.94575 | -0.384 |
| TIA1 | ILMN_2388466 | 0.000817 | 3.44E-05 | -4.51 | 2.10035 | -0.384 |
| IL2RB | ILMN_1684349 | 0.00484 | 0.000378 | -3.79 | -0.15112 | -0.384 |
| TMCC1 | ILMN_1677963 | 0.0315 | 0.00522 | -2.91 | -2.56406 | -0.384 |
| NSUN5P1 | ILMN_2352448 | 0.107 | 0.0306 | -2.22 | -4.12613 | -0.384 |
| TAGAP | ILMN_1739985 | 0.112 | 0.0325 | -2.19 | -4.1795 | -0.384 |
| RBBP8 | ILMN_2363621 | 0.00204 | 0.000117 | -4.14 | 0.9434 | -0.386 |
| NOP2 | ILMN_1723158 | 0.00441 | 0.000332 | -3.83 | -0.0302 | -0.386 |
| MIOS | ILMN_1680644 | 0.00484 | 0.00038 | -3.78 | -0.15477 | -0.386 |
| GTF3C1 | ILMN_1789839 | 0.0134 | 0.00156 | -3.33 | -1.46398 | -0.386 |
| PPP1R9B | ILMN_1688865 | 0.0152 | 0.00188 | -3.27 | -1.63229 | -0.386 |
| UFSP2 | ILMN_1756311 | 0.0189 | 0.00253 | -3.16 | -1.9063 | -0.386 |
| RAB37 | ILMN_2255579 | 0.0498 | 0.0101 | -2.66 | -3.15464 | -0.386 |
| WBP11 | ILMN_1766435 | 9.23E-05 | 1.57E-06 | -5.38 | 5.02558 | -0.387 |
| FRYL | ILMN_1747223 | 0.00266 | 0.000169 | -4.04 | 0.60361 | -0.387 |
| SAMD3 | ILMN_1659158 | 0.00463 | 0.000356 | -3.8 | -0.09477 | -0.387 |
| EFCAB3 | ILMN_1726437 | 0.00908 | 0.000919 | -3.5 | -0.97493 | -0.387 |
| VAMP4 | ILMN_1761363 | 0.00997 | 0.00105 | -3.46 | -1.09599 | -0.387 |
| BNIP3L | ILMN_2045419 | 0.0168 | 0.00215 | -3.22 | -1.75777 | -0.387 |
| EIF4EBP1 | ILMN_1767324 | 0.0254 | 0.00389 | -3.01 | -2.29817 | -0.387 |
| FLAD1 | ILMN_1663667 | 0.0315 | 0.0052 | -2.91 | -2.56029 | -0.387 |
| RHBDF2 | ILMN_2373062 | 0.0694 | 0.0163 | -2.48 | -3.5762 | -0.387 |
| EXOSC1 | ILMN_2396648 | 0.00337 | 0.000229 | -3.94 | 0.31549 | -0.388 |
| GPR141 | ILMN_1656818 | 0.00996 | 0.00105 | -3.46 | -1.0951 | -0.388 |
| MRPS17 | ILMN_1804851 | 0.00998 | 0.00105 | -3.46 | -1.09764 | -0.388 |
| NFKBIB | ILMN_1674152 | 0.0148 | 0.00178 | -3.28 | -1.58586 | -0.388 |
| CCDC189 | ILMN_2179726 | 0.0384 | 0.00691 | -2.81 | -2.81543 | -0.388 |
| RUNDC1 | ILMN_1733875 | 6.13E-05 | 8.70E-07 | -5.54 | 5.5899 | -0.389 |
| TMEM154 | ILMN_2088124 | 0.00368 | 0.000256 | -3.91 | 0.21293 | -0.389 |
| C3orf58 | ILMN_1797372 | 0.0064 | 0.000557 | -3.66 | -0.51028 | -0.389 |
| SUOX | ILMN_2383455 | 0.00809 | 0.000782 | -3.55 | -0.82577 | -0.389 |
| ZNF22 | ILMN_2117904 | 0.0213 | 0.00302 | -3.1 | -2.06628 | -0.389 |
| U2AF2 | ILMN_2385173 | 0.0421 | 0.00785 | -2.76 | -2.9308 | -0.389 |
| CAMK2G | ILMN_1809695 | 0.0558 | 0.0118 | -2.6 | -3.29703 | -0.389 |
| IMP3 | ILMN_1733696 | 0.000716 | 2.89E-05 | -4.56 | 2.26434 | -0.39 |
| STIM2 | ILMN_1738449 | 0.00377 | 0.000265 | -3.9 | 0.18097 | -0.39 |
| RNF216 | ILMN_1729980 | 0.00865 | 0.00086 | -3.52 | -0.91333 | -0.39 |
| VEGFA | ILMN_2375879 | 0.0115 | 0.00127 | -3.4 | -1.27064 | -0.39 |
| SREK1 | ILMN_1720088 | 0.0167 | 0.00212 | -3.22 | -1.74509 | -0.39 |
| SLC2A6 | ILMN_1778321 | 0.0519 | 0.0107 | -2.64 | -3.20259 | -0.39 |
| PTMS | ILMN_1721046 | 0.00661 | 0.000588 | -3.65 | -0.56094 | -0.391 |
| CSNK2A1 | ILMN_2386355 | 0.0142 | 0.00169 | -3.3 | -1.53717 | -0.391 |
| ATXN2L | ILMN_2271627 | 0.0368 | 0.00651 | -2.83 | -2.76221 | -0.391 |
| MYADM | ILMN_2350574 | 0.0426 | 0.00798 | -2.75 | -2.94547 | -0.391 |
| PRPSAP2 | ILMN_2116661 | 0.000465 | 1.60E-05 | -4.73 | 2.82358 | -0.392 |
| KDM3A | ILMN_1722532 | 0.00944 | 0.000972 | -3.48 | -1.02693 | -0.392 |
| EXOSC9 | ILMN_1721713 | 0.0574 | 0.0123 | -2.59 | -3.33275 | -0.392 |
| FAM214A | ILMN_1700733 | 0.0787 | 0.0194 | -2.41 | -3.73191 | -0.392 |
| SPPL2A | ILMN_1734229 | 6.26E-08 | 2.69E-11 | -8.3 | 15.50132 | -0.393 |
| BCL2L12 | ILMN_2396982 | 0.000739 | 3.03E-05 | -4.54 | 2.21971 | -0.393 |
| GBA2 | ILMN_1674560 | 0.00215 | 0.000126 | -4.12 | 0.87869 | -0.393 |
| KIAA1143 | ILMN_1752273 | 0.000634 | 2.46E-05 | -4.6 | 2.41401 | -0.394 |
| APEX2 | ILMN_1652505 | 0.00201 | 0.000114 | -4.15 | 0.96795 | -0.394 |
| TMEM154 | ILMN_1683494 | 0.00299 | 0.000198 | -3.99 | 0.4555 | -0.394 |
| ITFG2 | ILMN_1701244 | 0.0136 | 0.00159 | -3.32 | -1.48063 | -0.394 |
| GAB1 | ILMN_1781672 | 0.0445 | 0.00849 | -2.73 | -3.00048 | -0.394 |
| TRAF1 | ILMN_1698218 | 0.0514 | 0.0105 | -2.65 | -3.18949 | -0.394 |
| DARS | ILMN_1813836 | 0.000923 | 4.06E-05 | -4.46 | 1.94338 | -0.395 |
| P2RY13 | ILMN_1664525 | 0.00265 | 0.000168 | -4.04 | 0.60893 | -0.395 |
| HAUS8 | ILMN_1768020 | 0.00595 | 0.000498 | -3.7 | -0.40723 | -0.395 |
| ADARB1 | ILMN_2319326 | 0.0853 | 0.0219 | -2.36 | -3.8366 | -0.395 |
| GPR65 | ILMN_1734740 | 0.00597 | 0.000502 | -3.7 | -0.41476 | -0.396 |
| CYFIP2 | ILMN_2354478 | 0.0112 | 0.00122 | -3.41 | -1.23742 | -0.396 |
| OTUD6B | ILMN_2215631 | 0.0184 | 0.00244 | -3.18 | -1.87071 | -0.396 |
| CD300LF | ILMN_2112357 | 0.0225 | 0.00329 | -3.07 | -2.1441 | -0.396 |
| MRPL54 | ILMN_1658486 | 0.00032 | 9.43E-06 | -4.88 | 3.32404 | -0.397 |
| FBXW7 | ILMN_1668634 | 0.000953 | 4.25E-05 | -4.45 | 1.89995 | -0.397 |
| ZNF654 | ILMN_2271894 | 0.00421 | 0.000311 | -3.85 | 0.03061 | -0.397 |
| QPCT | ILMN_1741727 | 0.000106 | 1.91E-06 | -5.32 | 4.8397 | -0.398 |
| SEC23B | ILMN_2366246 | 0.000158 | 3.30E-06 | -5.17 | 4.3216 | -0.398 |
| SLC35E1 | ILMN_3251379 | 6.47E-06 | 3.90E-08 | -6.37 | 8.55144 | -0.399 |
| SIN3B | ILMN_1788315 | 0.00637 | 0.00055 | -3.67 | -0.4984 | -0.399 |
| ESRRA | ILMN_1774272 | 0.00983 | 0.00103 | -3.47 | -1.0785 | -0.399 |
| STAP1 | ILMN_3247998 | 0.0498 | 0.0101 | -2.66 | -3.15418 | -0.399 |
| TXNDC12 | ILMN_1783753 | 0.00126 | 6.05E-05 | -4.34 | 1.56679 | -0.4 |
| ARL15 | ILMN_2202967 | 0.00801 | 0.000771 | -3.56 | -0.81251 | -0.4 |
| RALGAPA1 | ILMN_2324574 | 0.016 | 0.00202 | -3.24 | -1.69794 | -0.4 |
| KCNK6 | ILMN_2074773 | 0.0355 | 0.00619 | -2.85 | -2.71771 | -0.4 |
| AAGAB | ILMN_3237396 | 0.000894 | 3.88E-05 | -4.47 | 1.98666 | -0.401 |
| STK36 | ILMN_1693538 | 0.00272 | 0.000174 | -4.03 | 0.57341 | -0.401 |
| ARSB | ILMN_2242937 | 0.00398 | 0.000286 | -3.87 | 0.11081 | -0.401 |
| CLC | ILMN_1654875 | 0.00697 | 0.000634 | -3.62 | -0.63042 | -0.401 |
| SMAP1 | ILMN_1768271 | 0.0097 | 0.00101 | -3.47 | -1.06096 | -0.401 |
| PIGA | ILMN_1705985 | 0.0128 | 0.00145 | -3.35 | -1.39598 | -0.401 |
| BRI3BP | ILMN_1693410 | 0.0178 | 0.00234 | -3.19 | -1.83239 | -0.401 |
| JAML | ILMN_1778723 | 0.00459 | 0.00035 | -3.81 | -0.07969 | -0.402 |
| RPS19BP1 | ILMN_2177965 | 0.00796 | 0.000765 | -3.56 | -0.80457 | -0.402 |
| RASA3 | ILMN_1654586 | 0.0154 | 0.0019 | -3.26 | -1.64528 | -0.402 |
| FAM65A | ILMN_1680037 | 0.0302 | 0.00491 | -2.93 | -2.50823 | -0.402 |
| G0S2 | ILMN_1691846 | 0.0381 | 0.00683 | -2.81 | -2.80554 | -0.402 |
| FAM117B | ILMN_1739942 | 0.119 | 0.0356 | -2.15 | -4.25696 | -0.402 |
| CMIP | ILMN_1738075 | 0.00209 | 0.000121 | -4.14 | 0.91467 | -0.403 |
| CWC22 | ILMN_3245616 | 0.00303 | 0.000201 | -3.98 | 0.43849 | -0.403 |
| LSP1 | ILMN_2355225 | 0.00634 | 0.000544 | -3.67 | -0.48875 | -0.403 |
| CEACAM3 | ILMN_1743570 | 0.00634 | 0.000545 | -3.67 | -0.49089 | -0.403 |
| CNPY3 | ILMN_1669674 | 0.0119 | 0.00132 | -3.38 | -1.31144 | -0.403 |
| FAM220A | ILMN_2116811 | 0.0335 | 0.00569 | -2.88 | -2.64124 | -0.403 |
| SIRT2 | ILMN_2398711 | 0.0543 | 0.0114 | -2.62 | -3.2626 | -0.403 |
| TMEM138 | ILMN_1664761 | 0.0654 | 0.0149 | -2.51 | -3.50202 | -0.403 |
| NFKBIB | ILMN_1690473 | 0.000288 | 8.03E-06 | -4.92 | 3.47648 | -0.405 |
| PIGH | ILMN_1798395 | 0.00282 | 0.000183 | -4.01 | 0.52497 | -0.405 |
| BTN3A1 | ILMN_1802708 | 0.0588 | 0.0128 | -2.57 | -3.36225 | -0.405 |
| CCL4L1 | ILMN_1716276 | 0.133 | 0.0418 | -2.08 | -4.39337 | -0.405 |
| ZNF816 | ILMN_1728710 | 0.00862 | 0.00085 | -3.53 | -0.90275 | -0.406 |
| TACSTD2 | ILMN_1739001 | 0.0545 | 0.0115 | -2.62 | -3.2676 | -0.406 |
| DEF8 | ILMN_1767509 | 0.0561 | 0.012 | -2.6 | -3.30575 | -0.406 |
| RAB37 | ILMN_2243912 | 0.0625 | 0.0139 | -2.54 | -3.43942 | -0.406 |
| HCST | ILMN_2396991 | 0.000597 | 2.25E-05 | -4.63 | 2.5005 | -0.407 |
| C17orf58 | ILMN_1712985 | 0.00697 | 0.000634 | -3.62 | -0.63142 | -0.407 |
| FCHO1 | ILMN_1654571 | 0.0872 | 0.0226 | -2.35 | -3.86448 | -0.407 |
| TIMP1 | ILMN_1711566 | 0.0032 | 0.000215 | -3.96 | 0.37553 | -0.408 |
| XPR1 | ILMN_3250032 | 0.00498 | 0.000397 | -3.77 | -0.1948 | -0.408 |
| CTDSPL2 | ILMN_2077758 | 0.0328 | 0.00554 | -2.89 | -2.6174 | -0.408 |
| FAM117B | ILMN_3244607 | 0.0423 | 0.00792 | -2.76 | -2.93807 | -0.408 |
| CRTC2 | ILMN_1657771 | 7.92E-06 | 5.00E-08 | -6.31 | 8.31511 | -0.409 |
| CKAP2L | ILMN_1751776 | 0.00473 | 0.000367 | -3.8 | -0.12358 | -0.409 |
| PSMD10 | ILMN_2281128 | 0.0129 | 0.00147 | -3.35 | -1.4086 | -0.409 |
| TAPBPL | ILMN_1805449 | 0.0246 | 0.00373 | -3.03 | -2.25788 | -0.41 |
| MPEG1 | ILMN_1752355 | 0.0641 | 0.0145 | -2.52 | -3.47467 | -0.41 |
| EBNA1BP2 | ILMN_1768127 | 0.0699 | 0.0164 | -2.47 | -3.58495 | -0.41 |
| FCGR2B | ILMN_2382403 | 0.00145 | 7.41E-05 | -4.28 | 1.37554 | -0.411 |
| LOC407835 | ILMN_2198893 | 4.07E-06 | 2.05E-08 | -6.54 | 9.16781 | -0.412 |
| COX20 | ILMN_1808584 | 0.00298 | 0.000197 | -3.99 | 0.46059 | -0.412 |
| SF3A3 | ILMN_1705151 | 0.00515 | 0.000414 | -3.76 | -0.23427 | -0.412 |
| ASUN | ILMN_2196335 | 0.0152 | 0.00186 | -3.27 | -1.62424 | -0.412 |
| UTP14C | ILMN_1686645 | 0.026 | 0.004 | -3 | -2.32338 | -0.412 |
| HCST | ILMN_1699931 | 0.000265 | 7.16E-06 | -4.96 | 3.58547 | -0.413 |
| SNAP23 | ILMN_1748911 | 0.00211 | 0.000123 | -4.13 | 0.90168 | -0.413 |
| NCOR1 | ILMN_2186369 | 0.025 | 0.00382 | -3.02 | -2.28017 | -0.413 |
| ZNF654 | ILMN_1721629 | 0.00272 | 0.000173 | -4.03 | 0.58029 | -0.414 |
| TIMM21 | ILMN_1748916 | 0.0073 | 0.000681 | -3.6 | -0.69683 | -0.414 |
| HSPA13 | ILMN_2231985 | 0.0104 | 0.00111 | -3.44 | -1.14711 | -0.414 |
| ACTR6 | ILMN_1697585 | 0.000339 | 1.02E-05 | -4.86 | 3.25256 | -0.415 |
| WDR41 | ILMN_1778488 | 0.00195 | 0.000111 | -4.16 | 1.00028 | -0.415 |
| SDHAF1 | ILMN_2070355 | 0.00231 | 0.00014 | -4.09 | 0.78155 | -0.415 |
| ZNF562 | ILMN_1672940 | 0.0315 | 0.00522 | -2.91 | -2.56263 | -0.415 |
| ZNF654 | ILMN_2367233 | 0.00309 | 0.000206 | -3.97 | 0.41432 | -0.416 |
| CAMP | ILMN_1688580 | 0.00407 | 0.000296 | -3.86 | 0.07814 | -0.416 |
| ADAP1 | ILMN_2047511 | 0.0014 | 7.10E-05 | -4.3 | 1.41668 | -0.417 |
| ZNF45 | ILMN_1771884 | 0.0023 | 0.000138 | -4.1 | 0.7908 | -0.417 |
| ATP23 | ILMN_1767481 | 0.0066 | 0.000586 | -3.65 | -0.55848 | -0.417 |
| FAM216A | ILMN_2180371 | 0.0146 | 0.00176 | -3.29 | -1.57373 | -0.417 |
| POLR2H | ILMN_1689445 | 5.64E-07 | 1.20E-09 | -7.29 | 11.88122 | -0.418 |
| CXorf65 | ILMN_1661359 | 0.00164 | 8.87E-05 | -4.23 | 1.20683 | -0.418 |
| SOS1 | ILMN_1767135 | 0.00647 | 0.000569 | -3.66 | -0.53008 | -0.418 |
| GTF3C6 | ILMN_1691578 | 0.000928 | 4.09E-05 | -4.46 | 1.93528 | -0.419 |
| METTL25 | ILMN_1745497 | 0.00403 | 0.00029 | -3.87 | 0.09555 | -0.419 |
| AGPAT1 | ILMN_1679520 | 0.00619 | 0.000525 | -3.68 | -0.45497 | -0.419 |
| B9D2 | ILMN_1806999 | 4.91E-07 | 1.01E-09 | -7.34 | 12.04465 | -0.42 |
| ANP32AP1 | ILMN_2056002 | 0.0016 | 8.57E-05 | -4.24 | 1.23865 | -0.42 |
| INPPL1 | ILMN_1728426 | 0.0065 | 0.000575 | -3.65 | -0.54093 | -0.42 |
| NCF1 | ILMN_1697309 | 0.00823 | 0.000798 | -3.55 | -0.84468 | -0.42 |
| ENC1 | ILMN_1779147 | 0.0277 | 0.00438 | -2.97 | -2.40434 | -0.42 |
| CSTA | ILMN_1669888 | 0.033 | 0.00559 | -2.88 | -2.62542 | -0.42 |
| LST1 | ILMN_1688373 | 0.000465 | 1.60E-05 | -4.73 | 2.82306 | -0.421 |
| GMDS | ILMN_1711227 | 0.00577 | 0.000479 | -3.71 | -0.37131 | -0.422 |
| POLR2D | ILMN_1792672 | 0.00757 | 0.00071 | -3.59 | -0.73636 | -0.423 |
| EAF1 | ILMN_1685012 | 0.000122 | 2.33E-06 | -5.27 | 4.65295 | -0.424 |
| CNOT9 | ILMN_2044085 | 0.000297 | 8.31E-06 | -4.91 | 3.44391 | -0.424 |
| LRR1 | ILMN_1715616 | 0.000946 | 4.20E-05 | -4.45 | 1.91073 | -0.425 |
| CISD2 | ILMN_1796397 | 0.0131 | 0.00151 | -3.34 | -1.43311 | -0.425 |
| PUS7 | ILMN_1779353 | 0.0732 | 0.0175 | -2.45 | -3.6431 | -0.425 |
| ELMSAN1 | ILMN_1763091 | 2.21E-05 | 2.29E-07 | -5.9 | 6.8621 | -0.426 |
| NCF1B | ILMN_2106725 | 0.0167 | 0.00213 | -3.22 | -1.74718 | -0.426 |
| ITGB2 | ILMN_1654396 | 9.76E-05 | 1.71E-06 | -5.35 | 4.94642 | -0.427 |
| TJAP1 | ILMN_1743763 | 0.000407 | 1.29E-05 | -4.79 | 3.02434 | -0.427 |
| AP1S2 | ILMN_1766411 | 0.00042 | 1.38E-05 | -4.77 | 2.9648 | -0.427 |
| KIAA0753 | ILMN_1680010 | 0.0297 | 0.00479 | -2.94 | -2.48558 | -0.427 |
| AMPD2 | ILMN_1701589 | 0.0306 | 0.00498 | -2.92 | -2.52174 | -0.427 |
| RELB | ILMN_1811258 | 0.00117 | 5.51E-05 | -4.37 | 1.65512 | -0.428 |
| INPP1 | ILMN_1667239 | 0.0119 | 0.00133 | -3.38 | -1.31618 | -0.428 |
| SDCBP2 | ILMN_1705107 | 0.0491 | 0.00987 | -2.67 | -3.13459 | -0.429 |
| EDC4 | ILMN_1665212 | 0.00201 | 0.000114 | -4.15 | 0.97025 | -0.43 |
| MZT1 | ILMN_3243945 | 0.0028 | 0.000182 | -4.01 | 0.53377 | -0.43 |
| LSM4 | ILMN_1788099 | 0.0473 | 0.00929 | -2.7 | -3.08109 | -0.43 |
| IFIH1 | ILMN_1781373 | 0.0834 | 0.0212 | -2.37 | -3.80725 | -0.43 |
| SNAP23 | ILMN_1687519 | 0.00503 | 0.000401 | -3.77 | -0.20432 | -0.431 |
| PDCD2 | ILMN_1758915 | 0.0059 | 0.000493 | -3.7 | -0.39667 | -0.431 |
| TBC1D9B | ILMN_2390227 | 0.00775 | 0.000732 | -3.58 | -0.76423 | -0.431 |
| TRIOBP | ILMN_1809145 | 0.0269 | 0.00418 | -2.99 | -2.36301 | -0.431 |
| EGLN1 | ILMN_1749892 | 0.0288 | 0.00461 | -2.95 | -2.45013 | -0.431 |
| ALCAM | ILMN_1670870 | 0.00128 | 6.22E-05 | -4.33 | 1.5414 | -0.432 |
| TIMM21 | ILMN_2182531 | 0.00359 | 0.000248 | -3.92 | 0.24375 | -0.432 |
| FBP1 | ILMN_1728799 | 0.00711 | 0.000653 | -3.61 | -0.65773 | -0.433 |
| MTIF2 | ILMN_1765520 | 0.0469 | 0.00914 | -2.7 | -3.06618 | -0.433 |
| ZMIZ1 | ILMN_1771627 | 0.000112 | 2.07E-06 | -5.3 | 4.76467 | -0.434 |
| GPR65 | ILMN_2232121 | 0.0072 | 0.000664 | -3.61 | -0.67452 | -0.434 |
| ZNF484 | ILMN_1683854 | 0.00992 | 0.00104 | -3.46 | -1.08951 | -0.434 |
| RPGR | ILMN_2336803 | 0.0104 | 0.0011 | -3.44 | -1.14205 | -0.434 |
| AK4 | ILMN_1764090 | 0.0232 | 0.00342 | -3.06 | -2.17992 | -0.434 |
| LRRC25 | ILMN_1766487 | 0.000421 | 1.38E-05 | -4.77 | 2.9627 | -0.435 |
| ICAM2 | ILMN_1786823 | 0.00517 | 0.000416 | -3.76 | -0.2385 | -0.435 |
| IL16 | ILMN_2290628 | 0.0223 | 0.00325 | -3.08 | -2.13359 | -0.436 |
| FAM234A | ILMN_1810055 | 0.0246 | 0.00373 | -3.03 | -2.25867 | -0.436 |
| CEP131 | ILMN_1746206 | 0.0561 | 0.012 | -2.6 | -3.30588 | -0.436 |
| PGM1 | ILMN_1800659 | 8.33E-05 | 1.36E-06 | -5.42 | 5.16536 | -0.437 |
| TRMT10C | ILMN_1672565 | 0.00757 | 0.00071 | -3.59 | -0.73646 | -0.438 |
| AP4E1 | ILMN_1754531 | 0.0235 | 0.00349 | -3.05 | -2.19958 | -0.438 |
| ZNF234 | ILMN_2103397 | 0.00274 | 0.000176 | -4.02 | 0.56314 | -0.439 |
| RELT | ILMN_1748614 | 0.00352 | 0.000243 | -3.92 | 0.26286 | -0.439 |
| TNNI2 | ILMN_2169261 | 0.00417 | 0.000306 | -3.85 | 0.04661 | -0.439 |
| ANP32C | ILMN_1655868 | 0.00478 | 0.000372 | -3.79 | -0.13494 | -0.439 |
| PADI2 | ILMN_1771223 | 0.000409 | 1.32E-05 | -4.78 | 3.00668 | -0.44 |
| ATP2C1 | ILMN_1758784 | 0.0201 | 0.00279 | -3.13 | -1.99353 | -0.441 |
| NME8 | ILMN_1691334 | 0.0473 | 0.00928 | -2.7 | -3.08021 | -0.441 |
| ZNF324 | ILMN_1745784 | 0.00535 | 0.000436 | -3.74 | -0.28287 | -0.442 |
| OMA1 | ILMN_2094938 | 0.0375 | 0.00669 | -2.82 | -2.7868 | -0.442 |
| SEL1L | ILMN_1726496 | 0.0139 | 0.00165 | -3.31 | -1.51261 | -0.443 |
| HMGA1 | ILMN_2311537 | 0.0468 | 0.0091 | -2.7 | -3.06247 | -0.443 |
| PRDX5 | ILMN_1711606 | 0.000765 | 3.15E-05 | -4.53 | 2.1813 | -0.444 |
| LTN1 | ILMN_3243686 | 0.00302 | 0.000201 | -3.98 | 0.44156 | -0.444 |
| LYRM7 | ILMN_1709936 | 0.0207 | 0.00291 | -3.11 | -2.0342 | -0.444 |
| CYP27A1 | ILMN_1704985 | 0.0285 | 0.00455 | -2.96 | -2.4385 | -0.444 |
| CDK2AP2 | ILMN_1690653 | 0.0331 | 0.00561 | -2.88 | -2.62877 | -0.444 |
| TMEM19 | ILMN_1693333 | 0.00335 | 0.000228 | -3.94 | 0.31996 | -0.445 |
| ERO1A | ILMN_1744963 | 0.00394 | 0.000282 | -3.88 | 0.1246 | -0.445 |
| VAMP4 | ILMN_1804676 | 0.00395 | 0.000282 | -3.88 | 0.12183 | -0.445 |
| FLVCR1 | ILMN_1661596 | 0.0458 | 0.00885 | -2.71 | -3.03731 | -0.445 |
| ARID2 | ILMN_1795247 | 0.000788 | 3.27E-05 | -4.52 | 2.14807 | -0.446 |
| CFAP20 | ILMN_2112599 | 9.36E-05 | 1.61E-06 | -5.37 | 5.00377 | -0.447 |
| RASSF7 | ILMN_1733110 | 0.00282 | 0.000184 | -4.01 | 0.51991 | -0.447 |
| ZNF432 | ILMN_1693788 | 0.00484 | 0.00038 | -3.78 | -0.15479 | -0.447 |
| NOMO1 | ILMN_1678730 | 0.0119 | 0.00133 | -3.38 | -1.31385 | -0.447 |
| CPOX | ILMN_3240389 | 0.0152 | 0.00186 | -3.27 | -1.62566 | -0.447 |
| WARS | ILMN_2337655 | 0.0962 | 0.026 | -2.29 | -3.98552 | -0.447 |
| TIAL1 | ILMN_2351309 | 0.00711 | 0.000654 | -3.61 | -0.65943 | -0.448 |
| ME2 | ILMN_1675186 | 0.0167 | 0.00213 | -3.22 | -1.74975 | -0.448 |
| ZNF653 | ILMN_1696276 | 0.0254 | 0.00389 | -3.01 | -2.29625 | -0.448 |
| APOBEC3G | ILMN_1802106 | 0.104 | 0.0292 | -2.24 | -4.08738 | -0.448 |
| ARL6IP5 | ILMN_1769810 | 0.00259 | 0.000162 | -4.05 | 0.63948 | -0.449 |
| ATF4 | ILMN_1783394 | 0.00178 | 9.76E-05 | -4.2 | 1.11666 | -0.45 |
| CCL19 | ILMN_1769129 | 2.20E-05 | 2.23E-07 | -5.91 | 6.88679 | -0.451 |
| PRKCI | ILMN_1725188 | 8.56E-05 | 1.40E-06 | -5.41 | 5.13486 | -0.451 |
| ALKBH5 | ILMN_1657283 | 0.000172 | 3.85E-06 | -5.13 | 4.17309 | -0.451 |
| NQO2 | ILMN_1712918 | 0.000553 | 2.04E-05 | -4.66 | 2.59136 | -0.451 |
| CDC42EP3 | ILMN_1736327 | 0.0353 | 0.00616 | -2.85 | -2.71202 | -0.451 |
| NINJ2 | ILMN_1731745 | 0.000407 | 1.30E-05 | -4.79 | 3.02001 | -0.452 |
| HIVEP2 | ILMN_1745447 | 0.00122 | 5.77E-05 | -4.36 | 1.61189 | -0.452 |
| CNGB1 | ILMN_1702383 | 0.00267 | 0.00017 | -4.03 | 0.59887 | -0.452 |
| BRF2 | ILMN_1665554 | 0.00561 | 0.000464 | -3.72 | -0.34012 | -0.452 |
| ARL5A | ILMN_2332558 | 0.105 | 0.0296 | -2.23 | -4.09669 | -0.452 |
| LST1 | ILMN_2345353 | 0.000662 | 2.60E-05 | -4.59 | 2.36202 | -0.453 |
| ABCF1 | ILMN_1763875 | 0.0022 | 0.00013 | -4.12 | 0.85003 | -0.454 |
| RHOQ | ILMN_1810559 | 0.00348 | 0.000239 | -3.93 | 0.2791 | -0.454 |
| TNFAIP8 | ILMN_2414325 | 0.00434 | 0.000326 | -3.83 | -0.01204 | -0.454 |
| CDKN2C | ILMN_1656415 | 0.0141 | 0.00167 | -3.3 | -1.52785 | -0.454 |
| PLAGL2 | ILMN_1786601 | 0.0101 | 0.00107 | -3.45 | -1.11338 | -0.455 |
| ITGB2 | ILMN_2175912 | 7.77E-05 | 1.22E-06 | -5.45 | 5.26616 | -0.457 |
| SLC31A1 | ILMN_1804562 | 0.000666 | 2.63E-05 | -4.59 | 2.35368 | -0.457 |
| NUP58 | ILMN_2378316 | 0.00155 | 8.22E-05 | -4.25 | 1.27841 | -0.457 |
| RAB28 | ILMN_2410864 | 0.00531 | 0.000431 | -3.74 | -0.27292 | -0.457 |
| CYB5D1 | ILMN_1670925 | 0.00722 | 0.000668 | -3.61 | -0.67885 | -0.457 |
| SMAD6 | ILMN_1767068 | 0.00282 | 0.000184 | -4.01 | 0.52337 | -0.458 |
| TPI1 | ILMN_1707627 | 0.000438 | 1.46E-05 | -4.75 | 2.90658 | -0.459 |
| PFN1 | ILMN_1712950 | 0.000626 | 2.41E-05 | -4.61 | 2.43455 | -0.46 |
| MAN2B1 | ILMN_1759341 | 0.00228 | 0.000137 | -4.1 | 0.79964 | -0.46 |
| PSG3 | ILMN_1685312 | 0.00276 | 0.000179 | -4.02 | 0.54961 | -0.46 |
| CS | ILMN_2396410 | 0.000112 | 2.06E-06 | -5.3 | 4.76864 | -0.461 |
| HIGD1A | ILMN_2230016 | 0.000195 | 4.53E-06 | -5.08 | 4.01975 | -0.461 |
| SEC13 | ILMN_3223181 | 0.000198 | 4.71E-06 | -5.07 | 3.98255 | -0.461 |
| ME2 | ILMN_2048636 | 0.00992 | 0.00104 | -3.46 | -1.09011 | -0.461 |
| CTRL | ILMN_1664863 | 0.0616 | 0.0136 | -2.55 | -3.42155 | -0.461 |
| SCARF1 | ILMN_2390946 | 0.0215 | 0.00306 | -3.1 | -2.07867 | -0.462 |
| MRPL17 | ILMN_1797933 | 0.00398 | 0.000285 | -3.87 | 0.11323 | -0.463 |
| AOAH | ILMN_1709820 | 0.0133 | 0.00154 | -3.33 | -1.45257 | -0.463 |
| ASF1B | ILMN_1695414 | 0.0586 | 0.0127 | -2.58 | -3.35709 | -0.463 |
| CHST11 | ILMN_1655880 | 0.00428 | 0.000319 | -3.84 | 0.00669 | -0.464 |
| TNK2 | ILMN_1669703 | 0.0592 | 0.0129 | -2.57 | -3.37457 | -0.464 |
| MED19 | ILMN_1754553 | 0.000531 | 1.93E-05 | -4.67 | 2.64762 | -0.465 |
| SLC38A10 | ILMN_2277419 | 0.00304 | 0.000202 | -3.98 | 0.43436 | -0.465 |
| TMEM189-UBE2V1 | ILMN_1677446 | 0.00385 | 0.000272 | -3.89 | 0.15673 | -0.465 |
| BCL7B | ILMN_1754199 | 0.015 | 0.00182 | -3.28 | -1.60432 | -0.465 |
| SUOX | ILMN_1803745 | 0.0162 | 0.00204 | -3.24 | -1.71056 | -0.465 |
| IL16 | ILMN_1813572 | 0.0242 | 0.00364 | -3.04 | -2.23642 | -0.465 |
| RSPH3 | ILMN_1788223 | 0.025 | 0.00381 | -3.02 | -2.27881 | -0.465 |
| DCLRE1C | ILMN_1754211 | 0.0281 | 0.00446 | -2.96 | -2.42062 | -0.465 |
| STARD10 | ILMN_1717052 | 0.00143 | 7.28E-05 | -4.29 | 1.39259 | -0.466 |
| NAT1 | ILMN_1743055 | 0.0437 | 0.00827 | -2.74 | -2.97727 | -0.466 |
| TRIM24 | ILMN_1799642 | 0.0314 | 0.00516 | -2.91 | -2.55245 | -0.467 |
| FAM110A | ILMN_2323944 | 0.00152 | 7.99E-05 | -4.26 | 1.30546 | -0.468 |
| CECR5 | ILMN_1654690 | 0.00999 | 0.00105 | -3.46 | -1.09937 | -0.468 |
| AP1S2 | ILMN_2120273 | 5.97E-05 | 8.33E-07 | -5.55 | 5.63128 | -0.469 |
| LGALS1 | ILMN_1723978 | 0.000365 | 1.12E-05 | -4.83 | 3.16105 | -0.469 |
| STK11IP | ILMN_1690085 | 0.00143 | 7.31E-05 | -4.29 | 1.38913 | -0.469 |
| TRAPPC13 | ILMN_1658439 | 0.00302 | 2.00E-04 | -3.98 | 0.44509 | -0.469 |
| NPRL2 | ILMN_2191568 | 0.008 | 0.000769 | -3.56 | -0.81006 | -0.469 |
| SURF6 | ILMN_1778032 | 0.0114 | 0.00124 | -3.4 | -1.25411 | -0.469 |
| DPEP3 | ILMN_1731275 | 0.108 | 0.0307 | -2.22 | -4.12826 | -0.469 |
| LCLAT1 | ILMN_1708081 | 0.00385 | 0.000272 | -3.89 | 0.15655 | -0.47 |
| NCOR2 | ILMN_2340052 | 0.00155 | 8.20E-05 | -4.25 | 1.28024 | -0.471 |
| PRDX5 | ILMN_1815024 | 0.0037 | 0.000259 | -3.9 | 0.20378 | -0.471 |
| SLAMF9 | ILMN_2227248 | 0.0115 | 0.00126 | -3.4 | -1.26443 | -0.471 |
| ELK1 | ILMN_1654289 | 0.000872 | 3.74E-05 | -4.48 | 2.02089 | -0.472 |
| NPHP4 | ILMN_2219512 | 0.00179 | 9.84E-05 | -4.2 | 1.10959 | -0.474 |
| CERK | ILMN_1767475 | 1.60E-06 | 4.46E-09 | -6.95 | 10.62417 | -0.475 |
| HNMT | ILMN_1751789 | 0.00197 | 0.000112 | -4.16 | 0.9917 | -0.475 |
| SMPD2 | ILMN_1672176 | 0.0235 | 0.00348 | -3.05 | -2.19721 | -0.475 |
| ID2 | ILMN_1793990 | 0.00154 | 8.07E-05 | -4.26 | 1.2958 | -0.476 |
| PLAGL1 | ILMN_2356955 | 0.00944 | 0.000974 | -3.48 | -1.02878 | -0.476 |
| FOXRED1 | ILMN_1762312 | 0.0112 | 0.00122 | -3.41 | -1.23341 | -0.476 |
| NCOR2 | ILMN_1698419 | 0.00184 | 0.000102 | -4.19 | 1.07719 | -0.477 |
| BTN3A2 | ILMN_1676528 | 0.0129 | 0.00148 | -3.34 | -1.41719 | -0.477 |
| MYO5A | ILMN_1698225 | 0.023 | 0.00339 | -3.06 | -2.17225 | -0.477 |
| CERK | ILMN_1721325 | 0.00123 | 5.85E-05 | -4.35 | 1.59842 | -0.478 |
| MITD1 | ILMN_1677133 | 0.00741 | 0.000692 | -3.59 | -0.71249 | -0.478 |
| CSE1L | ILMN_1665797 | 0.000402 | 1.27E-05 | -4.79 | 3.04278 | -0.479 |
| RXRA | ILMN_1687315 | 2.58E-06 | 1.10E-08 | -6.71 | 9.7648 | -0.481 |
| NFKB2 | ILMN_2390859 | 0.000248 | 6.46E-06 | -4.98 | 3.68315 | -0.481 |
| MEF2C | ILMN_1742544 | 0.0832 | 0.0211 | -2.37 | -3.80372 | -0.481 |
| MYPOP | ILMN_1704793 | 0.000198 | 4.69E-06 | -5.07 | 3.98626 | -0.482 |
| GALNS | ILMN_1737949 | 0.00248 | 0.000153 | -4.06 | 0.69238 | -0.483 |
| C15orf48 | ILMN_2389064 | 0.00641 | 0.000559 | -3.66 | -0.51366 | -0.483 |
| FNBP1 | ILMN_1797342 | 0.000744 | 3.06E-05 | -4.54 | 2.21112 | -0.485 |
| ANKRD37 | ILMN_1756417 | 0.0735 | 0.0176 | -2.45 | -3.64743 | -0.485 |
| NOMO2 | ILMN_1799856 | 0.00417 | 0.000307 | -3.85 | 0.04385 | -0.486 |
| ATG7 | ILMN_1790978 | 5.39E-05 | 7.08E-07 | -5.59 | 5.7855 | -0.487 |
| CCNG2 | ILMN_2228732 | 0.00697 | 0.000634 | -3.62 | -0.63137 | -0.487 |
| PLOD2 | ILMN_1771599 | 0.02 | 0.00276 | -3.13 | -1.98559 | -0.487 |
| CAMKK2 | ILMN_1743021 | 0.0348 | 0.00603 | -2.86 | -2.69361 | -0.487 |
| PLAU | ILMN_1656057 | 0.0526 | 0.0109 | -2.63 | -3.22367 | -0.487 |
| ORC3 | ILMN_1784946 | 0.00466 | 0.00036 | -3.8 | -0.10553 | -0.488 |
| FCGR2B | ILMN_1804174 | 0.00373 | 0.000261 | -3.9 | 0.1935 | -0.489 |
| PTGES2 | ILMN_2345016 | 0.0059 | 0.000493 | -3.7 | -0.39749 | -0.492 |
| SLC4A1AP | ILMN_1750876 | 0.00638 | 0.000553 | -3.67 | -0.50359 | -0.492 |
| PTGS2 | ILMN_2054297 | 0.00971 | 0.00101 | -3.47 | -1.06219 | -0.492 |
| CXCR4 | ILMN_2246410 | 0.031 | 0.00507 | -2.92 | -2.53699 | -0.492 |
| CFAP36 | ILMN_1792456 | 2.04E-05 | 1.91E-07 | -5.95 | 7.03687 | -0.493 |
| RAB24 | ILMN_2278850 | 6.62E-05 | 9.66E-07 | -5.51 | 5.48938 | -0.493 |
| NELL2 | ILMN_1725417 | 0.000353 | 1.08E-05 | -4.84 | 3.19717 | -0.493 |
| NOL12 | ILMN_2151368 | 0.00113 | 5.25E-05 | -4.38 | 1.70122 | -0.493 |
| AGL | ILMN_2371825 | 0.00454 | 0.000345 | -3.82 | -0.06431 | -0.493 |
| DFNA5 | ILMN_1670145 | 0.00503 | 0.000402 | -3.77 | -0.20777 | -0.493 |
| GPR141 | ILMN_2092333 | 0.00686 | 0.00062 | -3.63 | -0.61076 | -0.493 |
| AMACR | ILMN_1792741 | 0.0281 | 0.00445 | -2.97 | -2.42003 | -0.493 |
| ANKRD13A | ILMN_1689908 | 0.000433 | 1.44E-05 | -4.76 | 2.92381 | -0.494 |
| NUP62 | ILMN_2323491 | 0.0083 | 0.000807 | -3.54 | -0.85432 | -0.495 |
| CDC42SE2 | ILMN_1735594 | 0.0119 | 0.00132 | -3.38 | -1.31144 | -0.495 |
| EP400 | ILMN_1673023 | 0.000145 | 2.93E-06 | -5.2 | 4.43372 | -0.496 |
| BID | ILMN_2259495 | 0.0131 | 0.00151 | -3.34 | -1.43471 | -0.496 |
| SLC25A29 | ILMN_2350801 | 0.0207 | 0.00291 | -3.12 | -2.03212 | -0.496 |
| LGALS12 | ILMN_1776283 | 0.000102 | 1.82E-06 | -5.34 | 4.88404 | -0.497 |
| PTP4A3 | ILMN_3250321 | 0.00126 | 6.04E-05 | -4.34 | 1.56827 | -0.497 |
| DYNLT3 | ILMN_1681890 | 0.0149 | 0.00181 | -3.28 | -1.59803 | -0.497 |
| CD36 | ILMN_1796094 | 0.129 | 0.0402 | -2.1 | -4.35894 | -0.498 |
| ELMSAN1 | ILMN_2412922 | 0.000141 | 2.80E-06 | -5.22 | 4.47841 | -0.499 |
| PGAM4 | ILMN_2102515 | 0.0214 | 0.00305 | -3.1 | -2.07537 | -0.499 |
| TNFSF12 | ILMN_1683700 | 0.00488 | 0.000384 | -3.78 | -0.16587 | -0.5 |
| SEC13 | ILMN_3297880 | 0.000168 | 3.67E-06 | -5.14 | 4.22079 | -0.501 |
| STK11 | ILMN_1751871 | 0.00726 | 0.000673 | -3.6 | -0.68582 | -0.501 |
| ASUN | ILMN_2196337 | 0.00401 | 0.000289 | -3.87 | 0.09941 | -0.502 |
| GCH1 | ILMN_2335813 | 0.00568 | 0.000471 | -3.72 | -0.3546 | -0.502 |
| STARD3NL | ILMN_2228873 | 0.002 | 0.000114 | -4.15 | 0.97353 | -0.504 |
| PMS2CL | ILMN_2262203 | 0.00932 | 0.000955 | -3.49 | -1.01028 | -0.504 |
| TMOD2 | ILMN_3251132 | 0.1 | 0.0275 | -2.26 | -4.03492 | -0.504 |
| LIPT1 | ILMN_2343105 | 0.00325 | 0.000219 | -3.95 | 0.35718 | -0.505 |
| MRPL30 | ILMN_1766154 | 0.00459 | 0.000351 | -3.81 | -0.08014 | -0.505 |
| CTDSP1 | ILMN_1681678 | 9.61E-06 | 6.99E-08 | -6.22 | 7.99572 | -0.506 |
| NFKB2 | ILMN_1799062 | 4.61E-05 | 5.83E-07 | -5.65 | 5.97023 | -0.506 |
| SREBF1 | ILMN_1663035 | 0.00176 | 9.63E-05 | -4.2 | 1.12979 | -0.507 |
| TBC1D10A | ILMN_1693726 | 0.000369 | 1.14E-05 | -4.82 | 3.14204 | -0.508 |
| ZNF503 | ILMN_1787265 | 0.00031 | 8.94E-06 | -4.89 | 3.37377 | -0.509 |
| ID3 | ILMN_1732296 | 0.00287 | 0.000188 | -4 | 0.50116 | -0.509 |
| P2RY10 | ILMN_2321578 | 0.0406 | 0.00748 | -2.78 | -2.88666 | -0.509 |
| DBN1 | ILMN_1769926 | 0.000695 | 2.76E-05 | -4.57 | 2.30619 | -0.51 |
| ACBD5 | ILMN_1693250 | 0.0096 | 0.000994 | -3.48 | -1.04761 | -0.51 |
| GHRL | ILMN_1696380 | 0.0338 | 0.00577 | -2.87 | -2.6534 | -0.51 |
| FUT11 | ILMN_1678862 | 0.000913 | 3.98E-05 | -4.46 | 1.96125 | -0.511 |
| HMOX2 | ILMN_1658807 | 0.0139 | 0.00164 | -3.31 | -1.50687 | -0.512 |
| TAX1BP3 | ILMN_1803392 | 0.023 | 0.00338 | -3.06 | -2.17071 | -0.512 |
| ALG10B | ILMN_1730304 | 0.000302 | 8.50E-06 | -4.91 | 3.42187 | -0.514 |
| PPP1R12B | ILMN_1756289 | 0.000494 | 1.76E-05 | -4.7 | 2.73421 | -0.514 |
| CD300LB | ILMN_1782741 | 0.0241 | 0.00361 | -3.04 | -2.22856 | -0.514 |
| RFC5 | ILMN_1659364 | 0.000801 | 3.34E-05 | -4.52 | 2.12694 | -0.516 |
| AP4B1 | ILMN_1669377 | 0.00174 | 9.51E-05 | -4.21 | 1.14148 | -0.516 |
| NUP37 | ILMN_1771903 | 0.00638 | 0.000552 | -3.67 | -0.50279 | -0.516 |
| UBE2O | ILMN_1746579 | 0.0092 | 0.000937 | -3.5 | -0.99262 | -0.516 |
| PRDX5 | ILMN_2383975 | 0.000465 | 1.60E-05 | -4.73 | 2.82473 | -0.517 |
| TBC1D9B | ILMN_1789909 | 0.0193 | 0.00263 | -3.15 | -1.93953 | -0.517 |
| PIK3C2A | ILMN_3251440 | 0.000143 | 2.86E-06 | -5.21 | 4.45666 | -0.518 |
| VEGFB | ILMN_1722855 | 0.022 | 0.00319 | -3.08 | -2.11649 | -0.518 |
| YIPF5 | ILMN_1714756 | 6.16E-05 | 8.78E-07 | -5.54 | 5.58047 | -0.519 |
| ZNRD1 | ILMN_1722894 | 0.000499 | 1.78E-05 | -4.7 | 2.72264 | -0.519 |
| C11orf21 | ILMN_3235922 | 0.0172 | 0.00222 | -3.21 | -1.78782 | -0.519 |
| KIF23 | ILMN_1811472 | 0.019 | 0.00256 | -3.16 | -1.91608 | -0.519 |
| ARHGEF17 | ILMN_1754562 | 2.52E-05 | 2.78E-07 | -5.85 | 6.67813 | -0.52 |
| HAUS8 | ILMN_3176989 | 0.00144 | 7.35E-05 | -4.28 | 1.38363 | -0.52 |
| TRMT1 | ILMN_1812940 | 0.00455 | 0.000345 | -3.81 | -0.06623 | -0.52 |
| TMEM52B | ILMN_1727606 | 0.0116 | 0.00127 | -3.4 | -1.27595 | -0.52 |
| GOLT1B | ILMN_1767837 | 0.000946 | 4.19E-05 | -4.45 | 1.91208 | -0.521 |
| FCRLB | ILMN_1782015 | 0.000417 | 1.36E-05 | -4.77 | 2.97463 | -0.522 |
| FAM43A | ILMN_1706015 | 0.0342 | 0.00589 | -2.86 | -2.67172 | -0.522 |
| ICAM1 | ILMN_1812226 | 0.00094 | 4.16E-05 | -4.45 | 1.91918 | -0.523 |
| KISS1R | ILMN_1673521 | 0.027 | 0.0042 | -2.99 | -2.36586 | -0.523 |
| HSPA14 | ILMN_1797318 | 0.000194 | 4.49E-06 | -5.09 | 4.02781 | -0.524 |
| LIPT1 | ILMN_1717524 | 0.00434 | 0.000325 | -3.83 | -0.00961 | -0.524 |
| PKM | ILMN_2366634 | 5.39E-05 | 7.05E-07 | -5.59 | 5.78972 | -0.526 |
| AK4 | ILMN_1798249 | 0.00711 | 0.000653 | -3.61 | -0.65816 | -0.526 |
| TNFAIP8L2 | ILMN_1744113 | 0.0119 | 0.00133 | -3.38 | -1.31313 | -0.527 |
| PGAM4 | ILMN_1691104 | 0.00015 | 3.09E-06 | -5.19 | 4.38239 | -0.528 |
| VDR | ILMN_2319952 | 3.09E-05 | 3.62E-07 | -5.78 | 6.42565 | -0.529 |
| YEATS2 | ILMN_1676899 | 0.0043 | 0.000321 | -3.84 | 0.00219 | -0.529 |
| GTPBP8 | ILMN_2286514 | 0.00697 | 0.000635 | -3.62 | -0.63215 | -0.53 |
| PSME2 | ILMN_1786612 | 0.03 | 0.00487 | -2.93 | -2.50002 | -0.53 |
| ATG4A | ILMN_3307158 | 0.000505 | 1.81E-05 | -4.69 | 2.70453 | -0.531 |
| FICD | ILMN_1778064 | 0.000468 | 1.61E-05 | -4.73 | 2.81421 | -0.532 |
| ORC2 | ILMN_2061452 | 0.00278 | 0.00018 | -4.02 | 0.54258 | -0.532 |
| ABAT | ILMN_2404407 | 0.022 | 0.00318 | -3.08 | -2.1147 | -0.532 |
| APBA3 | ILMN_1686610 | 6.19E-06 | 3.57E-08 | -6.4 | 8.63763 | -0.533 |
| PHKG2 | ILMN_1669607 | 0.00934 | 0.000958 | -3.49 | -1.01331 | -0.533 |
| ITGAL | ILMN_1749591 | 0.0012 | 5.68E-05 | -4.36 | 1.62667 | -0.534 |
| FAM111A | ILMN_2410038 | 0.0106 | 0.00114 | -3.43 | -1.17196 | -0.534 |
| SSBP2 | ILMN_1711608 | 0.00728 | 0.000677 | -3.6 | -0.69155 | -0.536 |
| CAMKK2 | ILMN_2367638 | 0.0341 | 0.00584 | -2.87 | -2.66477 | -0.536 |
| MAP7 | ILMN_2216815 | 0.0486 | 0.00969 | -2.68 | -3.11863 | -0.536 |
| DNAAF2 | ILMN_1769757 | 0.000164 | 3.51E-06 | -5.15 | 4.26305 | -0.537 |
| KIF3B | ILMN_1702279 | 0.000384 | 1.20E-05 | -4.81 | 3.09474 | -0.538 |
| PTP4A3 | ILMN_1662427 | 0.00417 | 0.000307 | -3.85 | 0.04473 | -0.539 |
| LRRK2 | ILMN_2226015 | 0.0116 | 0.00128 | -3.39 | -1.28353 | -0.539 |
| HMGCR | ILMN_1657395 | 0.00207 | 0.000119 | -4.14 | 0.92717 | -0.54 |
| NCKIPSD | ILMN_1736623 | 9.15E-05 | 1.55E-06 | -5.38 | 5.03852 | -0.541 |
| PLAGL1 | ILMN_1815121 | 0.000263 | 7.07E-06 | -4.96 | 3.5974 | -0.542 |
| LYRM7 | ILMN_3238623 | 0.0383 | 0.00688 | -2.81 | -2.81147 | -0.542 |
| BRI3BP | ILMN_1800619 | 0.0102 | 0.00108 | -3.45 | -1.12515 | -0.544 |
| CLEC4A | ILMN_1709204 | 1.07E-05 | 8.12E-08 | -6.18 | 7.85263 | -0.545 |
| EIF4ENIF1 | ILMN_1794967 | 0.000986 | 4.42E-05 | -4.43 | 1.86277 | -0.545 |
| BAD | ILMN_1738652 | 0.00148 | 7.62E-05 | -4.27 | 1.34995 | -0.545 |
| FAM220A | ILMN_1717219 | 0.00162 | 8.69E-05 | -4.23 | 1.22566 | -0.545 |
| MPC1 | ILMN_1666967 | 0.000409 | 1.32E-05 | -4.78 | 3.0022 | -0.546 |
| CSE1L | ILMN_1706238 | 3.97E-05 | 4.86E-07 | -5.7 | 6.14391 | -0.547 |
| FBXW7 | ILMN_1754279 | 6.46E-06 | 3.81E-08 | -6.38 | 8.5739 | -0.548 |
| BCL11A | ILMN_1752899 | 0.000166 | 3.56E-06 | -5.15 | 4.24807 | -0.548 |
| UBE2E1 | ILMN_1806778 | 1.65E-06 | 5.46E-09 | -6.89 | 10.43141 | -0.549 |
| ACTR1B | ILMN_1695821 | 0.00505 | 0.000405 | -3.76 | -0.21373 | -0.549 |
| NBN | ILMN_1734833 | 0.000208 | 5.09E-06 | -5.05 | 3.90821 | -0.55 |
| FAM168A | ILMN_1847308 | 0.000806 | 3.38E-05 | -4.51 | 2.11511 | -0.551 |
| GADD45G | ILMN_1651498 | 0.0059 | 0.000493 | -3.7 | -0.39691 | -0.553 |
| NANOS3 | ILMN_1804007 | 0.000322 | 9.55E-06 | -4.87 | 3.31185 | -0.554 |
| UBE2E1 | ILMN_2371685 | 0.000787 | 3.26E-05 | -4.52 | 2.15051 | -0.555 |
| KCTD12 | ILMN_1742332 | 0.0133 | 0.00154 | -3.33 | -1.4535 | -0.555 |
| HVCN1 | ILMN_1815168 | 0.044 | 0.00835 | -2.74 | -2.98574 | -0.555 |
| MIF | ILMN_1807074 | 0.00143 | 7.29E-05 | -4.29 | 1.39073 | -0.556 |
| PDLIM5 | ILMN_2361478 | 0.00055 | 2.01E-05 | -4.66 | 2.608 | -0.557 |
| AP3S2 | ILMN_1731596 | 0.00401 | 0.000288 | -3.87 | 0.10178 | -0.557 |
| WARS | ILMN_1727271 | 0.049 | 0.00984 | -2.67 | -3.13169 | -0.558 |
| PSG9 | ILMN_1801776 | 0.000883 | 3.82E-05 | -4.48 | 1.99979 | -0.559 |
| PECAM1 | ILMN_1689518 | 0.00458 | 0.000349 | -3.81 | -0.07481 | -0.559 |
| CLEC4A | ILMN_2399363 | 1.60E-06 | 4.46E-09 | -6.95 | 10.62495 | -0.56 |
| MPC1 | ILMN_2226324 | 1.87E-05 | 1.73E-07 | -5.97 | 7.13088 | -0.56 |
| PDLIM5 | ILMN_2412281 | 0.00055 | 2.03E-05 | -4.66 | 2.59871 | -0.56 |
| VSIG8 | ILMN_1730740 | 0.0125 | 0.00141 | -3.36 | -1.37086 | -0.561 |
| NFKBID | ILMN_1763560 | 0.0017 | 9.22E-05 | -4.22 | 1.16986 | -0.562 |
| ADORA2A | ILMN_1807372 | 0.037 | 0.00657 | -2.82 | -2.77101 | -0.562 |
| BID | ILMN_1763386 | 0.000252 | 6.66E-06 | -4.98 | 3.65378 | -0.563 |
| KCTD12 | ILMN_2229649 | 0.00668 | 6.00E-04 | -3.64 | -0.57912 | -0.564 |
| FPR3 | ILMN_2203271 | 0.027 | 0.00421 | -2.99 | -2.36971 | -0.564 |
| CEBPE | ILMN_1779095 | 0.0136 | 0.00159 | -3.32 | -1.47973 | -0.565 |
| LEO1 | ILMN_1801553 | 0.000409 | 1.33E-05 | -4.78 | 3.00049 | -0.566 |
| P2RY10 | ILMN_1784774 | 0.0181 | 0.00238 | -3.18 | -1.85067 | -0.566 |
| ZNF318 | ILMN_2174729 | 0.000494 | 1.75E-05 | -4.7 | 2.73783 | -0.567 |
| IDH2 | ILMN_1751753 | 0.00189 | 0.000106 | -4.18 | 1.04132 | -0.567 |
| BCOR | ILMN_1712161 | 0.0061 | 0.000516 | -3.69 | -0.44007 | -0.567 |
| ARID2 | ILMN_2182335 | 0.000202 | 4.82E-06 | -5.07 | 3.96149 | -0.568 |
| CYSLTR1 | ILMN_1733276 | 0.00099 | 4.45E-05 | -4.43 | 1.85663 | -0.568 |
| PCBP3 | ILMN_1687216 | 0.000349 | 1.07E-05 | -4.84 | 3.20823 | -0.569 |
| TRIOBP | ILMN_2370588 | 0.00123 | 5.87E-05 | -4.35 | 1.59538 | -0.57 |
| NFKB1 | ILMN_1714965 | 4.64E-06 | 2.43E-08 | -6.5 | 9.00468 | -0.571 |
| HINFP | ILMN_1667453 | 0.0072 | 0.000664 | -3.61 | -0.67434 | -0.572 |
| CTSZ | ILMN_1666269 | 0.000178 | 4.01E-06 | -5.12 | 4.13448 | -0.573 |
| ABCF1 | ILMN_2392635 | 0.00488 | 0.000385 | -3.78 | -0.16734 | -0.573 |
| ASCL2 | ILMN_1723412 | 0.0111 | 0.00121 | -3.41 | -1.22566 | -0.574 |
| TGM3 | ILMN_1786847 | 0.0197 | 0.0027 | -3.14 | -1.96513 | -0.574 |
| AGAP3 | ILMN_1795918 | 0.0448 | 0.00856 | -2.73 | -3.00748 | -0.574 |
| IL18BP | ILMN_2334296 | 0.0035 | 0.000241 | -3.93 | 0.27077 | -0.575 |
| NOL12 | ILMN_1759991 | 0.000102 | 1.82E-06 | -5.34 | 4.88628 | -0.576 |
| FAM118A | ILMN_1809147 | 0.00533 | 0.000434 | -3.74 | -0.27896 | -0.577 |
| NAT9 | ILMN_1776088 | 0.0221 | 0.00319 | -3.08 | -2.11804 | -0.577 |
| DOCK10 | ILMN_1702301 | 0.000136 | 2.67E-06 | -5.23 | 4.52234 | -0.578 |
| EIF1AD | ILMN_1717834 | 0.000732 | 2.98E-05 | -4.55 | 2.23603 | -0.578 |
| MKL1 | ILMN_1651767 | 0.0102 | 0.00108 | -3.45 | -1.12236 | -0.578 |
| P4HA1 | ILMN_1693334 | 6.71E-05 | 9.97E-07 | -5.5 | 5.45981 | -0.579 |
| PTGES2 | ILMN_2345015 | 0.00183 | 0.000101 | -4.19 | 1.08424 | -0.579 |
| AK4 | ILMN_2338038 | 0.000749 | 3.08E-05 | -4.54 | 2.20407 | -0.58 |
| ETFB | ILMN_2300970 | 0.0038 | 0.000268 | -3.89 | 0.17075 | -0.58 |
| ZBTB46 | ILMN_1710092 | 0.0139 | 0.00164 | -3.31 | -1.50956 | -0.582 |
| TSPAN13 | ILMN_2130525 | 0.0181 | 0.00239 | -3.18 | -1.85296 | -0.584 |
| SIGLEC7 | ILMN_2409384 | 0.000863 | 3.68E-05 | -4.49 | 2.03543 | -0.585 |
| CCDC88B | ILMN_1772208 | 0.000604 | 2.29E-05 | -4.62 | 2.48271 | -0.586 |
| LOC284023 | ILMN_1739325 | 0.00441 | 0.000332 | -3.83 | -0.02978 | -0.586 |
| NUDT16L1 | ILMN_1735415 | 0.00974 | 0.00102 | -3.47 | -1.06825 | -0.589 |
| THUMPD3 | ILMN_1671902 | 0.00648 | 0.000571 | -3.66 | -0.53424 | -0.591 |
| PAK1 | ILMN_1767365 | 0.000554 | 2.05E-05 | -4.66 | 2.58933 | -0.592 |
| FAM110A | ILMN_2248093 | 0.00862 | 0.000854 | -3.53 | -0.90731 | -0.593 |
| NOMO1 | ILMN_2126957 | 0.0153 | 0.00189 | -3.26 | -1.63969 | -0.594 |
| PRR7 | ILMN_1677509 | 0.000409 | 1.31E-05 | -4.78 | 3.01219 | -0.597 |
| PGAM4 | ILMN_1706841 | 2.30E-06 | 9.20E-09 | -6.75 | 9.9328 | -0.599 |
| PTP4A3 | ILMN_2359710 | 0.000162 | 3.43E-06 | -5.16 | 4.28424 | -0.599 |
| ARRDC1 | ILMN_1661492 | 0.000443 | 1.50E-05 | -4.75 | 2.88421 | -0.599 |
| CD79B | ILMN_1710017 | 0.000677 | 2.67E-05 | -4.58 | 2.33676 | -0.599 |
| PTGS2 | ILMN_1677511 | 0.00862 | 0.000855 | -3.53 | -0.90747 | -0.599 |
| BCL2L12 | ILMN_1752953 | 0.000376 | 1.17E-05 | -4.82 | 3.11885 | -0.6 |
| RBM4B | ILMN_1743104 | 0.00243 | 0.00015 | -4.07 | 0.71531 | -0.603 |
| UPF3B | ILMN_2397627 | 0.00377 | 0.000265 | -3.9 | 0.18238 | -0.603 |
| SPG21 | ILMN_1657423 | 0.0064 | 0.000557 | -3.66 | -0.51032 | -0.603 |
| BTN3A3 | ILMN_2373831 | 0.0217 | 0.00311 | -3.09 | -2.09398 | -0.603 |
| HIC1 | ILMN_1738825 | 0.0673 | 0.0155 | -2.5 | -3.53409 | -0.603 |
| PGAM4 | ILMN_1682953 | 9.61E-06 | 7.03E-08 | -6.21 | 7.98931 | -0.604 |
| SULF2 | ILMN_1667460 | 0.000369 | 1.13E-05 | -4.83 | 3.14837 | -0.605 |
| CD59 | ILMN_2333687 | 6.00E-04 | 2.27E-05 | -4.63 | 2.49308 | -0.606 |
| SNUPN | ILMN_1733932 | 0.000384 | 1.20E-05 | -4.81 | 3.09291 | -0.608 |
| TSPAN13 | ILMN_1669881 | 0.0284 | 0.00452 | -2.96 | -2.43295 | -0.608 |
| AMACR | ILMN_1759670 | 0.00675 | 0.000608 | -3.64 | -0.59267 | -0.609 |
| TMEM205 | ILMN_1730734 | 9.14E-05 | 1.53E-06 | -5.38 | 5.05392 | -0.61 |
| CLIC4 | ILMN_1671250 | 0.000409 | 1.32E-05 | -4.78 | 3.00604 | -0.61 |
| HK2 | ILMN_2156172 | 0.00055 | 2.02E-05 | -4.66 | 2.60289 | -0.612 |
| ZW10 | ILMN_1712556 | 0.000116 | 2.16E-06 | -5.29 | 4.72238 | -0.613 |
| BID | ILMN_2372413 | 0.000342 | 1.03E-05 | -4.85 | 3.23728 | -0.613 |
| ASB6 | ILMN_2397776 | 0.00169 | 9.15E-05 | -4.22 | 1.17741 | -0.613 |
| GPNMB | ILMN_2407389 | 0.00496 | 0.000393 | -3.77 | -0.18736 | -0.613 |
| LCLAT1 | ILMN_3262936 | 0.00237 | 0.000144 | -4.08 | 0.74979 | -0.614 |
| PTPN22 | ILMN_1715885 | 0.00228 | 0.000136 | -4.1 | 0.80689 | -0.615 |
| PGAM1 | ILMN_2112417 | 8.05E-06 | 5.39E-08 | -6.29 | 8.24363 | -0.616 |
| LONP1 | ILMN_1766125 | 0.00123 | 5.84E-05 | -4.35 | 1.60013 | -0.616 |
| PKM | ILMN_1672650 | 0.000186 | 4.25E-06 | -5.1 | 4.08078 | -0.618 |
| ETFB | ILMN_1729374 | 0.00475 | 0.00037 | -3.79 | -0.12936 | -0.618 |
| LGMN | ILMN_1698019 | 0.025 | 0.00381 | -3.02 | -2.27779 | -0.619 |
| NOCT | ILMN_1689378 | 2.88E-05 | 3.28E-07 | -5.8 | 6.51815 | -0.62 |
| TMX2 | ILMN_1799367 | 0.000191 | 4.40E-06 | -5.09 | 4.04765 | -0.62 |
| CYB561A3 | ILMN_2129505 | 0.00984 | 0.00103 | -3.47 | -1.08016 | -0.621 |
| ATP2C1 | ILMN_2340565 | 0.00262 | 0.000165 | -4.04 | 0.62557 | -0.623 |
| CITED4 | ILMN_1787691 | 0.000168 | 3.62E-06 | -5.15 | 4.2318 | -0.624 |
| TIFA | ILMN_1686454 | 0.09 | 0.0237 | -2.33 | -3.90441 | -0.624 |
| TCF7L2 | ILMN_1672486 | 9.15E-05 | 1.54E-06 | -5.38 | 5.04745 | -0.625 |
| EPHB1 | ILMN_1692261 | 0.000126 | 2.40E-06 | -5.26 | 4.62462 | -0.625 |
| RNF121 | ILMN_2356031 | 0.00148 | 7.65E-05 | -4.27 | 1.34558 | -0.626 |
| FAM111A | ILMN_1778845 | 0.0107 | 0.00114 | -3.43 | -1.17464 | -0.626 |
| PRDX4 | ILMN_2222234 | 0.000269 | 7.27E-06 | -4.95 | 3.5707 | -0.627 |
| ZNF615 | ILMN_1672135 | 3.97E-05 | 4.87E-07 | -5.7 | 6.14272 | -0.629 |
| FARP2 | ILMN_2062687 | 6.96E-05 | 1.07E-06 | -5.48 | 5.39203 | -0.631 |
| SAR1B | ILMN_1736888 | 3.96E-07 | 6.41E-10 | -7.46 | 12.47837 | -0.632 |
| BCL2L2 | ILMN_1746561 | 0.00034 | 1.02E-05 | -4.86 | 3.24879 | -0.633 |
| GATSL3 | ILMN_2098418 | 0.00409 | 0.000298 | -3.86 | 0.07016 | -0.634 |
| RCOR1 | ILMN_1743421 | 0.000295 | 8.24E-06 | -4.92 | 3.452 | -0.636 |
| PIGA | ILMN_2367186 | 0.00213 | 0.000124 | -4.13 | 0.88867 | -0.636 |
| PDE9A | ILMN_2306540 | 0.119 | 0.0355 | -2.16 | -4.25362 | -0.636 |
| WDR61 | ILMN_1665887 | 5.29E-06 | 2.88E-08 | -6.45 | 8.84381 | -0.638 |
| TRIOBP | ILMN_1735788 | 0.000576 | 2.15E-05 | -4.64 | 2.54319 | -0.638 |
| LTB | ILMN_2376204 | 0.00114 | 5.30E-05 | -4.38 | 1.69083 | -0.639 |
| C17orf58 | ILMN_1700515 | 0.00131 | 6.46E-05 | -4.32 | 1.50567 | -0.639 |
| STAMBPL1 | ILMN_1682799 | 0.0275 | 0.00433 | -2.98 | -2.39347 | -0.641 |
| ATG4A | ILMN_2313782 | 6.35E-05 | 9.10E-07 | -5.53 | 5.54707 | -0.642 |
| DENND4A | ILMN_2041161 | 0.000437 | 1.46E-05 | -4.75 | 2.9121 | -0.643 |
| FKBP2 | ILMN_1674337 | 5.86E-05 | 7.97E-07 | -5.56 | 5.67292 | -0.646 |
| GBE1 | ILMN_1789702 | 0.000409 | 1.32E-05 | -4.78 | 3.0044 | -0.646 |
| RCSD1 | ILMN_1749006 | 0.0255 | 0.00391 | -3.01 | -2.30162 | -0.647 |
| KCNK6 | ILMN_1701173 | 8.75E-05 | 1.44E-06 | -5.4 | 5.10694 | -0.649 |
| LTB | ILMN_2376205 | 0.000281 | 7.78E-06 | -4.93 | 3.50605 | -0.649 |
| ZNF395 | ILMN_1772876 | 0.000472 | 1.65E-05 | -4.72 | 2.796 | -0.649 |
| PTPN22 | ILMN_1695640 | 0.000143 | 2.87E-06 | -5.21 | 4.45335 | -0.651 |
| NBN | ILMN_2358041 | 0.000162 | 3.44E-06 | -5.16 | 4.28134 | -0.651 |
| ID2 | ILMN_2086095 | 7.97E-06 | 5.12E-08 | -6.3 | 8.29175 | -0.652 |
| TNFRSF10B | ILMN_1699265 | 0.000298 | 8.38E-06 | -4.91 | 3.43596 | -0.652 |
| FGL2 | ILMN_1693009 | 7.77E-05 | 1.22E-06 | -5.45 | 5.26642 | -0.654 |
| MTFP1 | ILMN_2355665 | 0.00407 | 0.000296 | -3.86 | 0.07842 | -0.655 |
| P2RY6 | ILMN_1660031 | 6.87E-05 | 1.04E-06 | -5.49 | 5.42068 | -0.658 |
| PLEKHO1 | ILMN_1694213 | 0.000239 | 6.10E-06 | -5 | 3.73666 | -0.658 |
| SMAD3 | ILMN_1682738 | 2.82E-05 | 3.20E-07 | -5.81 | 6.54336 | -0.66 |
| HILPDA | ILMN_1659990 | 0.0117 | 0.00129 | -3.39 | -1.28607 | -0.661 |
| UHRF1 | ILMN_1786065 | 0.0265 | 0.00411 | -2.99 | -2.3482 | -0.661 |
| PKM | ILMN_1775327 | 6.19E-06 | 3.54E-08 | -6.4 | 8.64413 | -0.662 |
| UNC119 | ILMN_1664698 | 2.10E-05 | 2.05E-07 | -5.93 | 6.96781 | -0.663 |
| WDR25 | ILMN_1704351 | 0.000312 | 9.03E-06 | -4.89 | 3.36502 | -0.663 |
| CLIC4 | ILMN_2063584 | 0.00116 | 5.45E-05 | -4.37 | 1.66505 | -0.663 |
| PPP2R5D | ILMN_1780940 | 0.0015 | 7.79E-05 | -4.27 | 1.32896 | -0.663 |
| SMAD7 | ILMN_2203896 | 0.000246 | 6.41E-06 | -4.99 | 3.6906 | -0.664 |
| MCEE | ILMN_1735347 | 0.000909 | 3.96E-05 | -4.47 | 1.96594 | -0.664 |
| PLCB2 | ILMN_1724066 | 0.00282 | 0.000183 | -4.01 | 0.52474 | -0.664 |
| KCTD11 | ILMN_1777513 | 0.000252 | 6.67E-06 | -4.98 | 3.65283 | -0.665 |
| NFXL1 | ILMN_1682197 | 0.00309 | 0.000206 | -3.97 | 0.41581 | -0.666 |
| ABAT | ILMN_1805104 | 0.00646 | 0.000566 | -3.66 | -0.52558 | -0.666 |
| CCT6B | ILMN_1699610 | 5.51E-05 | 7.30E-07 | -5.59 | 5.75646 | -0.667 |
| ANAPC10 | ILMN_1770378 | 0.000168 | 3.65E-06 | -5.14 | 4.2245 | -0.668 |
| PGAM1 | ILMN_1661366 | 7.66E-06 | 4.77E-08 | -6.32 | 8.35995 | -0.67 |
| AK4 | ILMN_1843198 | 1.87E-05 | 1.74E-07 | -5.97 | 7.12606 | -0.67 |
| LRRK2 | ILMN_1776649 | 0.00046 | 1.56E-05 | -4.73 | 2.84456 | -0.675 |
| SMAD7 | ILMN_2203891 | 0.00048 | 1.69E-05 | -4.71 | 2.77356 | -0.675 |
| STXBP6 | ILMN_1750912 | 5.57E-05 | 7.46E-07 | -5.58 | 5.73534 | -0.676 |
| VLDLR | ILMN_2361862 | 4.70E-06 | 2.49E-08 | -6.49 | 8.98111 | -0.678 |
| BNIP3 | ILMN_1724658 | 0.00102 | 4.59E-05 | -4.42 | 1.82666 | -0.678 |
| VGLL4 | ILMN_1768480 | 9.76E-05 | 1.71E-06 | -5.35 | 4.94854 | -0.679 |
| GJA5 | ILMN_1702972 | 0.000171 | 3.82E-06 | -5.13 | 4.18214 | -0.679 |
| ANKDD1A | ILMN_1813139 | 0.000196 | 4.60E-06 | -5.08 | 4.00502 | -0.679 |
| SIGLEC7 | ILMN_1686706 | 0.000569 | 2.12E-05 | -4.65 | 2.5572 | -0.679 |
| HDGF | ILMN_1765621 | 6.84E-06 | 4.17E-08 | -6.35 | 8.48848 | -0.681 |
| SENP6 | ILMN_2054233 | 0.00032 | 9.43E-06 | -4.88 | 3.32356 | -0.682 |
| SCD | ILMN_1689329 | 0.00123 | 5.87E-05 | -4.35 | 1.59545 | -0.683 |
| OSM | ILMN_1780546 | 6.19E-06 | 3.46E-08 | -6.4 | 8.66641 | -0.684 |
| SLC35B2 | ILMN_1789001 | 0.000409 | 1.32E-05 | -4.78 | 3.00474 | -0.684 |
| SIGLEC7 | ILMN_1681415 | 0.000859 | 3.65E-05 | -4.49 | 2.04421 | -0.685 |
| ADAP1 | ILMN_3247424 | 0.00133 | 6.56E-05 | -4.32 | 1.49053 | -0.685 |
| HK2 | ILMN_1723486 | 0.001 | 4.52E-05 | -4.43 | 1.84188 | -0.687 |
| LOC646938 | ILMN_1689876 | 5.97E-05 | 8.31E-07 | -5.55 | 5.63345 | -0.689 |
| MRPL14 | ILMN_2072603 | 0.000206 | 5.02E-06 | -5.05 | 3.92222 | -0.69 |
| BCL11A | ILMN_1659800 | 1.65E-06 | 5.30E-09 | -6.9 | 10.46056 | -0.692 |
| ATF5 | ILMN_1669113 | 0.000665 | 2.62E-05 | -4.59 | 2.35678 | -0.692 |
| FUT4 | ILMN_1792072 | 5.34E-05 | 6.91E-07 | -5.6 | 5.80879 | -0.695 |
| ALDOC | ILMN_1755974 | 0.000135 | 2.64E-06 | -5.23 | 4.53386 | -0.696 |
| C6orf223 | ILMN_3248091 | 0.000202 | 4.84E-06 | -5.07 | 3.95673 | -0.696 |
| TLR9 | ILMN_1679798 | 0.00092 | 4.03E-05 | -4.46 | 1.95034 | -0.696 |
| FMNL3 | ILMN_2395214 | 4.91E-07 | 9.64E-10 | -7.35 | 12.08861 | -0.698 |
| TESC | ILMN_1750181 | 2.74E-05 | 3.09E-07 | -5.82 | 6.57522 | -0.698 |
| RBPJ | ILMN_1726913 | 0.000407 | 1.29E-05 | -4.79 | 3.02312 | -0.698 |
| NEDD4 | ILMN_1703140 | 8.05E-06 | 5.30E-08 | -6.29 | 8.25979 | -0.699 |
| NKIRAS1 | ILMN_1664216 | 0.00403 | 0.000292 | -3.87 | 0.09006 | -0.701 |
| SAR1B | ILMN_2394296 | 1.60E-06 | 4.88E-09 | -6.92 | 10.53794 | -0.703 |
| UNC119 | ILMN_1806052 | 2.18E-05 | 2.19E-07 | -5.91 | 6.90333 | -0.703 |
| RFX5 | ILMN_1741200 | 5.54E-05 | 7.38E-07 | -5.58 | 5.74623 | -0.703 |
| TNNT3 | ILMN_2334080 | 0.000501 | 1.79E-05 | -4.7 | 2.71506 | -0.705 |
| NFKBIE | ILMN_1717313 | 0.000239 | 6.12E-06 | -5 | 3.73393 | -0.71 |
| P2RY14 | ILMN_2342835 | 0.126 | 0.0386 | -2.12 | -4.32566 | -0.712 |
| SULF2 | ILMN_2345142 | 0.000369 | 1.14E-05 | -4.82 | 3.14326 | -0.713 |
| IKBKE | ILMN_1755024 | 0.00145 | 7.44E-05 | -4.28 | 1.37187 | -0.713 |
| INTS9 | ILMN_1777118 | 4.06E-08 | 8.07E-12 | -8.62 | 16.64954 | -0.714 |
| PAICS | ILMN_1773760 | 0.000169 | 3.72E-06 | -5.14 | 4.20549 | -0.714 |
| PTP4A3 | ILMN_1769779 | 0.000483 | 1.70E-05 | -4.71 | 2.76466 | -0.715 |
| ENO2 | ILMN_1765796 | 0.00017 | 3.78E-06 | -5.13 | 4.19236 | -0.717 |
| GSE1 | ILMN_1807767 | 0.00588 | 0.00049 | -3.7 | -0.39159 | -0.718 |
| TGIF2 | ILMN_1709044 | 0.000146 | 2.98E-06 | -5.2 | 4.41894 | -0.719 |
| AK2 | ILMN_1716053 | 5.75E-05 | 7.74E-07 | -5.57 | 5.70067 | -0.72 |
| SULF2 | ILMN_1686981 | 0.000304 | 8.68E-06 | -4.9 | 3.40173 | -0.72 |
| TM9SF4 | ILMN_1674421 | 4.08E-08 | 1.12E-11 | -8.53 | 16.33945 | -0.721 |
| ATP7A | ILMN_1808115 | 5.34E-05 | 6.94E-07 | -5.6 | 5.80448 | -0.723 |
| THOC1 | ILMN_1802157 | 0.00114 | 5.34E-05 | -4.38 | 1.68459 | -0.723 |
| FAM213B | ILMN_3243682 | 0.113 | 0.0329 | -2.19 | -4.1889 | -0.724 |
| IER5 | ILMN_1721833 | 1.19E-05 | 9.15E-08 | -6.14 | 7.73778 | -0.729 |
| BRPF3 | ILMN_1658800 | 4.49E-06 | 2.29E-08 | -6.51 | 9.05959 | -0.73 |
| ORM1 | ILMN_1696584 | 0.000156 | 3.25E-06 | -5.18 | 4.33493 | -0.73 |
| AK2 | ILMN_1670542 | 4.26E-05 | 5.31E-07 | -5.67 | 6.05984 | -0.733 |
| LYSMD2 | ILMN_1724493 | 0.00535 | 0.000436 | -3.74 | -0.28386 | -0.734 |
| OPA3 | ILMN_2284591 | 0.00108 | 4.99E-05 | -4.4 | 1.74761 | -0.735 |
| BCOR | ILMN_1773117 | 1.41E-05 | 1.15E-07 | -6.08 | 7.52291 | -0.736 |
| MIB2 | ILMN_2282077 | 0.000168 | 3.61E-06 | -5.15 | 4.23419 | -0.736 |
| TNFRSF10B | ILMN_2331010 | 0.000607 | 2.31E-05 | -4.62 | 2.47536 | -0.744 |
| GPI | ILMN_2173451 | 6.82E-05 | 1.03E-06 | -5.49 | 5.43136 | -0.745 |
| HVCN1 | ILMN_2365248 | 0.0388 | 0.00699 | -2.8 | -2.82687 | -0.748 |
| AGO3 | ILMN_1761049 | 1.24E-05 | 9.70E-08 | -6.13 | 7.68197 | -0.749 |
| RAP1GAP2 | ILMN_2041236 | 0.000721 | 2.92E-05 | -4.55 | 2.2539 | -0.749 |
| P2RY2 | ILMN_1723535 | 4.59E-10 | 3.05E-14 | -10.1 | 21.93817 | -0.75 |
| PTPN22 | ILMN_2246328 | 0.000102 | 1.82E-06 | -5.34 | 4.884 | -0.75 |
| SH2B3 | ILMN_1752046 | 0.000228 | 5.75E-06 | -5.02 | 3.79276 | -0.752 |
| SNRNP25 | ILMN_1801118 | 0.0311 | 0.00509 | -2.92 | -2.54145 | -0.752 |
| TBC1D22A | ILMN_2096743 | 2.92E-05 | 3.34E-07 | -5.8 | 6.50087 | -0.753 |
| CLCF1 | ILMN_1661197 | 0.0011 | 5.11E-05 | -4.39 | 1.72553 | -0.754 |
| SH3BP1 | ILMN_1692539 | 0.000161 | 3.39E-06 | -5.16 | 4.29513 | -0.755 |
| TNFAIP2 | ILMN_1727689 | 5.51E-05 | 7.28E-07 | -5.59 | 5.75905 | -0.758 |
| POLR3K | ILMN_1801664 | 0.0281 | 0.00446 | -2.96 | -2.42214 | -0.76 |
| DUSP2 | ILMN_1712959 | 0.000281 | 7.72E-06 | -4.93 | 3.513 | -0.763 |
| TNIP1 | ILMN_1703650 | 1.34E-07 | 9.78E-11 | -7.96 | 14.27279 | -0.764 |
| ADARB2 | ILMN_1749493 | 1.70E-05 | 1.47E-07 | -6.02 | 7.28864 | -0.764 |
| CD33 | ILMN_1747622 | 0.00275 | 0.000177 | -4.02 | 0.56087 | -0.765 |
| PDE6D | ILMN_1790680 | 2.77E-06 | 1.32E-08 | -6.66 | 9.58818 | -0.766 |
| PTGS1 | ILMN_2339835 | 6.71E-05 | 9.92E-07 | -5.5 | 5.46485 | -0.766 |
| BCKDHA | ILMN_1735979 | 0.000543 | 1.98E-05 | -4.67 | 2.62219 | -0.766 |
| C15orf48 | ILMN_1805410 | 0.000274 | 7.48E-06 | -4.94 | 3.54348 | -0.767 |
| LFNG | ILMN_1663080 | 0.000121 | 2.30E-06 | -5.27 | 4.66555 | -0.768 |
| FAM210A | ILMN_1658110 | 0.00864 | 0.000858 | -3.52 | -0.91175 | -0.77 |
| RPAP3 | ILMN_1766916 | 0.00101 | 4.54E-05 | -4.43 | 1.83718 | -0.773 |
| EEPD1 | ILMN_1811616 | 0.00247 | 0.000153 | -4.07 | 0.69623 | -0.773 |
| LRRC75B | ILMN_1737255 | 0.00936 | 0.000961 | -3.49 | -1.01568 | -0.775 |
| CARD9 | ILMN_1712532 | 0.00495 | 0.000392 | -3.77 | -0.18422 | -0.777 |
| LYST | ILMN_1675956 | 0.000203 | 4.91E-06 | -5.06 | 3.94355 | -0.778 |
| PRPSAP1 | ILMN_1768449 | 9.80E-08 | 5.97E-11 | -8.09 | 14.74339 | -0.779 |
| ZNF770 | ILMN_1734254 | 3.90E-06 | 1.94E-08 | -6.56 | 9.22023 | -0.782 |
| TNF | ILMN_1728106 | 0.0441 | 0.00838 | -2.73 | -2.98835 | -0.782 |
| DNAJC10 | ILMN_2151541 | 6.47E-06 | 3.86E-08 | -6.37 | 8.56296 | -0.783 |
| ZDHHC23 | ILMN_1736901 | 6.91E-05 | 1.05E-06 | -5.49 | 5.40759 | -0.783 |
| FOSB | ILMN_1751607 | 0.00883 | 0.00088 | -3.52 | -0.93507 | -0.783 |
| NOTCH4 | ILMN_1711157 | 9.60E-06 | 6.83E-08 | -6.22 | 8.0169 | -0.785 |
| XXYLT1 | ILMN_1671116 | 0.0152 | 0.00188 | -3.27 | -1.63337 | -0.786 |
| TBC1D2B | ILMN_2064606 | 0.000219 | 5.42E-06 | -5.03 | 3.84872 | -0.788 |
| RPGRIP1 | ILMN_1803739 | 0.0317 | 0.00527 | -2.91 | -2.5715 | -0.79 |
| BCL11A | ILMN_2255133 | 2.51E-05 | 2.71E-07 | -5.85 | 6.70027 | -0.791 |
| C7orf61 | ILMN_2226691 | 0.00265 | 0.000167 | -4.04 | 0.61313 | -0.792 |
| LEP | ILMN_2207504 | 0.00819 | 0.000794 | -3.55 | -0.83992 | -0.794 |
| AK2 | ILMN_1655645 | 0.000102 | 1.79E-06 | -5.34 | 4.9009 | -0.796 |
| RHOF | ILMN_1652918 | 0.000515 | 1.86E-05 | -4.69 | 2.68263 | -0.798 |
| SIGLEC10 | ILMN_1655549 | 0.000276 | 7.53E-06 | -4.94 | 3.53694 | -0.815 |
| SIK1 | ILMN_3235647 | 0.0032 | 0.000216 | -3.96 | 0.37213 | -0.82 |
| GJC2 | ILMN_1723048 | 0.000238 | 6.04E-06 | -5 | 3.74647 | -0.826 |
| AMACR | ILMN_2367172 | 0.00409 | 0.000298 | -3.86 | 0.07016 | -0.83 |
| TBC1D22A | ILMN_3251227 | 2.63E-06 | 1.13E-08 | -6.7 | 9.73239 | -0.831 |
| RTN2 | ILMN_1749115 | 0.000252 | 6.66E-06 | -4.98 | 3.65293 | -0.834 |
| TRAF3 | ILMN_2383774 | 0.000302 | 8.55E-06 | -4.91 | 3.4171 | -0.838 |
| SEC24A | ILMN_2126832 | 2.07E-06 | 7.61E-09 | -6.8 | 10.11381 | -0.848 |
| EGLN3 | ILMN_1667626 | 7.05E-07 | 1.59E-09 | -7.22 | 11.61053 | -0.857 |
| SIK1 | ILMN_1717639 | 0.00425 | 0.000315 | -3.84 | 0.01922 | -0.859 |
| TICAM2 | ILMN_1651346 | 2.07E-06 | 7.48E-09 | -6.81 | 10.13025 | -0.867 |
| RUNX3 | ILMN_1787461 | 3.96E-07 | 6.57E-10 | -7.45 | 12.45433 | -0.868 |
| COLEC12 | ILMN_1689088 | 2.30E-06 | 9.41E-09 | -6.75 | 9.91114 | -0.868 |
| LIF | ILMN_1738725 | 8.26E-05 | 1.34E-06 | -5.42 | 5.17716 | -0.871 |
| LFNG | ILMN_2360401 | 1.74E-05 | 1.55E-07 | -6 | 7.23707 | -0.873 |
| METTL6 | ILMN_1661998 | 2.95E-09 | 3.91E-13 | -9.44 | 19.52295 | -0.883 |
| SYTL1 | ILMN_1750785 | 5.94E-05 | 8.15E-07 | -5.56 | 5.65164 | -0.884 |
| ARRB1 | ILMN_1730620 | 0.00031 | 8.90E-06 | -4.89 | 3.3784 | -0.885 |
| MIB2 | ILMN_2385178 | 0.00052 | 1.88E-05 | -4.68 | 2.66988 | -0.895 |
| CFP | ILMN_1658121 | 2.15E-06 | 8.14E-09 | -6.79 | 10.04948 | -0.905 |
| SLC25A19 | ILMN_1666553 | 2.35E-06 | 9.80E-09 | -6.74 | 9.87247 | -0.907 |
| SERPINE1 | ILMN_1744381 | 2.67E-05 | 2.99E-07 | -5.83 | 6.60901 | -0.916 |
| EML2 | ILMN_3240541 | 8.68E-06 | 5.87E-08 | -6.26 | 8.16154 | -0.922 |
| GCH1 | ILMN_1812759 | 0.000245 | 6.34E-06 | -4.99 | 3.70025 | -0.922 |
| MARCKSL1 | ILMN_1714433 | 0.00046 | 1.57E-05 | -4.73 | 2.84205 | -0.93 |
| SLC2A5 | ILMN_1671337 | 1.65E-05 | 1.40E-07 | -6.03 | 7.33416 | -0.932 |
| GBP4 | ILMN_1771385 | 0.0271 | 0.00422 | -2.98 | -2.37096 | -0.951 |
| AMPD3 | ILMN_1774447 | 6.19E-06 | 3.51E-08 | -6.4 | 8.65296 | -0.958 |
| GPNMB | ILMN_1801205 | 0.00197 | 0.000112 | -4.16 | 0.99177 | -0.958 |
| IL1A | ILMN_1658483 | 0.00102 | 4.60E-05 | -4.42 | 1.82569 | -0.973 |
| ARRB1 | ILMN_1819608 | 0.0133 | 0.00154 | -3.33 | -1.45351 | -0.986 |
| MCTP1 | ILMN_1654685 | 2.17E-06 | 8.34E-09 | -6.78 | 10.02704 | -0.992 |
| PDXP | ILMN_1736441 | 9.61E-06 | 7.13E-08 | -6.21 | 7.97641 | -1 |
| CLIC4 | ILMN_2063586 | 2.77E-06 | 1.21E-08 | -6.68 | 9.66992 | -1.01 |
| CYBB | ILMN_1682312 | 2.10E-05 | 2.02E-07 | -5.93 | 6.98204 | -1.02 |
| C15orf48 | ILMN_1654696 | 0.000186 | 4.26E-06 | -5.1 | 4.07763 | -1.02 |
| TESK2 | ILMN_1654370 | 0.000302 | 8.56E-06 | -4.91 | 3.41581 | -1.02 |
| FAM162A | ILMN_1803647 | 0.000701 | 2.82E-05 | -4.57 | 2.28807 | -1.02 |
| FAM26F | ILMN_2066849 | 0.00229 | 0.000138 | -4.1 | 0.79474 | -1.03 |
| CADM4 | ILMN_1812096 | 4.91E-07 | 8.87E-10 | -7.37 | 12.16767 | -1.04 |
| TFRC | ILMN_1674243 | 0.000132 | 2.56E-06 | -5.24 | 4.56379 | -1.04 |
| DMXL2 | ILMN_1705663 | 1.64E-05 | 1.38E-07 | -6.04 | 7.34766 | -1.05 |
| ARRB1 | ILMN_2325168 | 0.000627 | 2.42E-05 | -4.61 | 2.43195 | -1.05 |
| TNFAIP8L1 | ILMN_1684346 | 0.00323 | 0.000218 | -3.96 | 0.36468 | -1.06 |
| TNFRSF9 | ILMN_1813379 | 1.47E-07 | 1.27E-10 | -7.89 | 14.02462 | -1.07 |
| KIF3B | ILMN_2081398 | 3.41E-07 | 4.95E-10 | -7.53 | 12.72479 | -1.09 |
| UPB1 | ILMN_1678690 | 0.00015 | 3.07E-06 | -5.19 | 4.3896 | -1.09 |
| AKR1A1 | ILMN_1728047 | 0.00116 | 5.46E-05 | -4.37 | 1.66324 | -1.1 |
| AKR1A1 | ILMN_2380771 | 7.00E-04 | 2.80E-05 | -4.57 | 2.29362 | -1.11 |
| COL13A1 | ILMN_2370624 | 2.28E-07 | 2.42E-10 | -7.72 | 13.40859 | -1.13 |
| P2RY2 | ILMN_2372915 | 2.28E-07 | 2.28E-10 | -7.73 | 13.46681 | -1.19 |
| TMEM268 | ILMN_1803652 | 0.00032 | 9.37E-06 | -4.88 | 3.33012 | -1.19 |
| LRRC75A | ILMN_2221784 | 0.000595 | 2.24E-05 | -4.63 | 2.50542 | -1.19 |
| TCFL5 | ILMN_1814247 | 4.91E-07 | 9.57E-10 | -7.35 | 12.09572 | -1.23 |
| PNPLA1 | ILMN_1808241 | 1.61E-05 | 1.34E-07 | -6.04 | 7.37134 | -1.23 |
| PRR5L | ILMN_1697491 | 0.00142 | 7.21E-05 | -4.29 | 1.40175 | -1.26 |
| MCRIP2 | ILMN_1730523 | 6.26E-08 | 3.32E-11 | -8.24 | 15.3027 | -1.29 |
| GPR162 | ILMN_1730816 | 0.000127 | 2.43E-06 | -5.26 | 4.61113 | -1.36 |
| SRC | ILMN_1729987 | 1.80E-07 | 1.67E-10 | -7.81 | 13.75962 | -1.37 |
| CD4 | ILMN_1727284 | 9.61E-06 | 7.14E-08 | -6.21 | 7.97554 | -1.4 |
| PTGES | ILMN_1713829 | 0.00649 | 0.000573 | -3.66 | -0.53649 | -1.48 |
| KCNH4 | ILMN_1800396 | 4.97E-05 | 6.39E-07 | -5.62 | 5.88395 | -1.5 |
| MMP9 | ILMN_1796316 | 9.36E-06 | 6.48E-08 | -6.24 | 8.06695 | -1.65 |
| TGM2 | ILMN_1705750 | 0.0123 | 0.00138 | -3.37 | -1.3504 | -1.75 |
| IL18R1 | ILMN_1781700 | 3.96E-07 | 6.56E-10 | 7.45 | 12.45657 | 2.48 |
| TLR2 | ILMN_1772387 | 9.47E-06 | 6.65E-08 | 6.23 | 8.04258 | 2.35 |
| ECHDC3 | ILMN_2072178 | 6.95E-05 | 1.06E-06 | 5.48 | 5.39733 | 2.35 |
| FKBP5 | ILMN_1778444 | 2.66E-05 | 2.96E-07 | 5.83 | 6.61819 | 2.24 |
| TPST1 | ILMN_1651950 | 4.08E-08 | 1.35E-11 | 8.48 | 16.15861 | 2.22 |
| KLF9 | ILMN_1778523 | 1.02E-05 | 7.64E-08 | 6.19 | 7.90996 | 2.05 |
| RAB3IL1 | ILMN_1741632 | 0.00012 | 2.27E-06 | 5.27 | 4.67472 | 1.81 |
| IL18RAP | ILMN_1721762 | 0.00022 | 5.50E-06 | 5.03 | 3.83581 | 1.69 |
| CCND3 | ILMN_1668721 | 9.80E-08 | 6.50E-11 | 8.06 | 14.66216 | 1.58 |
| NLRP6 | ILMN_1702970 | 7.92E-06 | 5.04E-08 | 6.3 | 8.30705 | 1.54 |
| SLC16A10 | ILMN_1782938 | 0.000134 | 2.61E-06 | 5.24 | 4.54304 | 1.5 |
| ABHD17C | ILMN_1788416 | 1.68E-05 | 1.44E-07 | 6.02 | 7.30759 | 1.46 |
| ZBTB16 | ILMN_2305407 | 0.00563 | 0.000466 | 3.72 | -0.34484 | 1.46 |
| SLC2A11 | ILMN_1748090 | 0.000369 | 1.14E-05 | 4.82 | 3.14301 | 1.45 |
| DAAM2 | ILMN_1752668 | 0.000471 | 1.64E-05 | 4.72 | 2.79883 | 1.44 |
| TRIM25 | ILMN_1813625 | 0.000407 | 1.29E-05 | 4.79 | 3.02429 | 1.43 |
| HAMP | ILMN_1729188 | 0.064 | 0.0144 | 2.53 | -3.47162 | 1.4 |
| OLIG2 | ILMN_1727567 | 0.0016 | 8.58E-05 | 4.24 | 1.23846 | 1.38 |
| TSC22D1 | ILMN_1692177 | 1.35E-06 | 3.58E-09 | 7 | 10.83409 | 1.36 |
| SPATA13 | ILMN_1742824 | 9.36E-06 | 6.51E-08 | 6.24 | 8.06248 | 1.36 |
| GCLM | ILMN_1788547 | 0.000916 | 4.00E-05 | 4.46 | 1.95631 | 1.36 |
| NTPCR | ILMN_1657446 | 2.09E-05 | 2.00E-07 | 5.94 | 6.99364 | 1.34 |
| TBC1D14 | ILMN_1779886 | 1.37E-05 | 1.10E-07 | 6.1 | 7.56131 | 1.31 |
| PRKDC | ILMN_1769517 | 2.94E-05 | 3.39E-07 | 5.79 | 6.4869 | 1.3 |
| TPK1 | ILMN_2367063 | 0.00283 | 0.000185 | 4.01 | 0.51781 | 1.26 |
| GFOD1 | ILMN_1778240 | 0.00114 | 5.29E-05 | 4.38 | 1.69286 | 1.23 |
| MOB3C | ILMN_1798288 | 0.000196 | 4.62E-06 | 5.08 | 4.00082 | 1.22 |
| SIRT5 | ILMN_1683059 | 0.000202 | 4.85E-06 | 5.06 | 3.95499 | 1.22 |
| SIPA1L2 | ILMN_1732923 | 1.65E-06 | 5.41E-09 | 6.9 | 10.44024 | 1.19 |
| SLC25A51 | ILMN_2198499 | 0.000569 | 2.12E-05 | 4.65 | 2.55675 | 1.17 |
| SMAP2 | ILMN_1781468 | 1.41E-05 | 1.14E-07 | 6.09 | 7.52833 | 1.16 |
| MFSD13A | ILMN_1808566 | 0.00265 | 0.000167 | 4.04 | 0.61086 | 1.16 |
| IL1R2 | ILMN_1758371 | 6.64E-05 | 9.73E-07 | 5.51 | 5.48258 | 1.14 |
| PROK2 | ILMN_1775257 | 0.00107 | 4.90E-05 | 4.4 | 1.76475 | 1.14 |
| PAOX | ILMN_2278265 | 0.00177 | 9.71E-05 | 4.2 | 1.12169 | 1.14 |
| SMA4 | ILMN_3238814 | 0.00403 | 0.000292 | 3.87 | 0.09001 | 1.14 |
| SORT1 | ILMN_1707077 | 0.00398 | 0.000285 | 3.87 | 0.11206 | 1.13 |
| PRKDC | ILMN_2334121 | 0.000221 | 5.54E-06 | 5.03 | 3.8277 | 1.12 |
| RNF14 | ILMN_1812581 | 0.00276 | 0.000179 | 4.02 | 0.54956 | 1.12 |
| KRTAP19-6 | ILMN_1784216 | 0.0106 | 0.00114 | 3.43 | -1.17127 | 1.11 |
| EFNA1 | ILMN_2371055 | 0.072 | 0.0171 | 2.46 | -3.62008 | 1.11 |
| CEBPD | ILMN_1782050 | 6.34E-07 | 1.39E-09 | 7.25 | 11.74126 | 1.1 |
| CRYL1 | ILMN_1714397 | 9.60E-06 | 6.88E-08 | 6.22 | 8.01077 | 1.09 |
| STK17B | ILMN_2166534 | 1.19E-05 | 9.23E-08 | 6.14 | 7.7302 | 1.09 |
| ST6GALNAC3 | ILMN_2127379 | 0.000112 | 2.04E-06 | 5.3 | 4.77627 | 1.09 |
| SLC26A8 | ILMN_1755843 | 0.00208 | 0.000121 | 4.14 | 0.91838 | 1.09 |
| LINC00152 | ILMN_3230508 | 0.00316 | 0.000213 | 3.96 | 0.38669 | 1.09 |
| TSC22D1 | ILMN_1787567 | 2.07E-06 | 7.62E-09 | 6.8 | 10.11262 | 1.08 |
| ACSL4 | ILMN_2391458 | 0.000953 | 4.25E-05 | 4.45 | 1.89941 | 1.08 |
| ERLIN1 | ILMN_1730731 | 0.026 | 0.00401 | 3 | -2.32506 | 1.08 |
| MGST2 | ILMN_1802027 | 3.63E-06 | 1.76E-08 | 6.58 | 9.31341 | 1.07 |
| CLEC4E | ILMN_1771664 | 3.01E-05 | 3.50E-07 | 5.78 | 6.45827 | 1.07 |
| NAIP | ILMN_1760189 | 0.00205 | 0.000118 | 4.14 | 0.94076 | 1.06 |
| CD177 | ILMN_3251610 | 0.0536 | 0.0112 | 2.62 | -3.24764 | 1.06 |
| FAM65B | ILMN_1726597 | 0.0012 | 5.67E-05 | 4.36 | 1.62797 | 1.04 |
| NAIP | ILMN_2260082 | 0.00421 | 0.000311 | 3.85 | 0.03087 | 1.04 |
| CEACAM4 | ILMN_1657455 | 0.00466 | 0.000361 | 3.8 | -0.1071 | 1.04 |
| CLEC4D | ILMN_1808979 | 0.00827 | 0.000803 | 3.55 | -0.8503 | 1.04 |
| CCNJL | ILMN_1763745 | 0.00813 | 0.000786 | 3.55 | -0.83062 | 1.03 |
| IRS2 | ILMN_2083469 | 2.94E-07 | 3.31E-10 | 7.63 | 13.10931 | 1.02 |
| RNF144A | ILMN_1777660 | 2.52E-05 | 2.74E-07 | 5.85 | 6.69259 | 1.02 |
| SC5D | ILMN_1677607 | 0.00025 | 6.56E-06 | 4.98 | 3.66741 | 1.01 |
| TSC22D1 | ILMN_2412380 | 0.000408 | 1.30E-05 | 4.79 | 3.01631 | 1.01 |
| CCL20 | ILMN_1657234 | 0.0321 | 0.00537 | 2.9 | -2.58906 | 1.01 |
| ANXA3 | ILMN_1694548 | 0.0496 | 0.01 | 2.67 | -3.14917 | 1.01 |
| ZNF281 | ILMN_1683127 | 0.000108 | 1.96E-06 | 5.32 | 4.81774 | 1 |
| ACSL4 | ILMN_1683598 | 0.000223 | 5.61E-06 | 5.02 | 3.81685 | 1 |
| LMNB1 | ILMN_2126706 | 0.00504 | 0.000403 | 3.77 | -0.2096 | 0.995 |
| SMIM3 | ILMN_1684368 | 0.000442 | 1.49E-05 | 4.75 | 2.89018 | 0.993 |
| BATF | ILMN_1668822 | 0.0433 | 0.00815 | 2.74 | -2.96386 | 0.99 |
| ARID5A | ILMN_1689700 | 1.83E-05 | 1.66E-07 | 5.98 | 7.16815 | 0.989 |
| XPO6 | ILMN_1755235 | 2.07E-06 | 7.70E-09 | 6.8 | 10.10338 | 0.98 |
| TPK1 | ILMN_1804629 | 0.00948 | 0.00098 | 3.48 | -1.03387 | 0.98 |
| PRKDC | ILMN_2253648 | 9.06E-05 | 1.51E-06 | 5.39 | 5.06609 | 0.977 |
| MIR21 | ILMN_3310840 | 1.09E-05 | 8.28E-08 | 6.17 | 7.83399 | 0.976 |
| TMIGD3 | ILMN_1733259 | 0.00107 | 4.89E-05 | 4.4 | 1.76743 | 0.972 |
| VNN1 | ILMN_1674574 | 0.00059 | 2.21E-05 | 4.63 | 2.5162 | 0.971 |
| KDSR | ILMN_2154053 | 0.00733 | 0.000684 | 3.6 | -0.70129 | 0.964 |
| KDSR | ILMN_2154052 | 0.00637 | 0.000549 | 3.67 | -0.49708 | 0.96 |
| SLCO4A1 | ILMN_1727200 | 0.00908 | 0.000916 | 3.5 | -0.9718 | 0.948 |
| PLSCR1 | ILMN_1745242 | 0.00583 | 0.000486 | 3.71 | -0.38326 | 0.944 |
| INHBB | ILMN_1685714 | 0.00138 | 6.98E-05 | 4.3 | 1.43196 | 0.943 |
| NLRP3 | ILMN_1696933 | 0.0181 | 0.00238 | 3.18 | -1.84984 | 0.939 |
| KIT | ILMN_1790160 | 1.79E-05 | 1.61E-07 | 5.99 | 7.19785 | 0.937 |
| MKNK1 | ILMN_1750429 | 0.000274 | 7.46E-06 | 4.94 | 3.54537 | 0.925 |
| MIAT | ILMN_1864900 | 0.0283 | 0.00451 | 2.96 | -2.43151 | 0.922 |
| GCLM | ILMN_2225974 | 0.000173 | 3.88E-06 | 5.13 | 4.16691 | 0.916 |
| SERPINB2 | ILMN_2150851 | 0.0313 | 0.00514 | 2.91 | -2.54984 | 0.91 |
| CYP1B1 | ILMN_1693338 | 0.00463 | 0.000357 | 3.8 | -0.09686 | 0.909 |
| TLR5 | ILMN_1722981 | 0.0292 | 0.00467 | 2.95 | -2.46257 | 0.907 |
| KIT | ILMN_2229379 | 7.89E-05 | 1.26E-06 | 5.44 | 5.23984 | 0.891 |
| SAMSN1 | ILMN_2171289 | 0.0177 | 0.00231 | 3.2 | -1.82157 | 0.891 |
| CDK5RAP2 | ILMN_2415529 | 0.0193 | 0.00263 | 3.15 | -1.9418 | 0.89 |
| TSC22D3 | ILMN_2276952 | 2.30E-06 | 9.47E-09 | 6.75 | 9.90543 | 0.886 |
| ADORA3 | ILMN_1730710 | 1.73E-06 | 5.84E-09 | 6.87 | 10.36699 | 0.882 |
| CCNJL | ILMN_3237926 | 0.0337 | 0.00574 | 2.87 | -2.64933 | 0.876 |
| IL1RL1 | ILMN_2313672 | 2.39E-05 | 2.51E-07 | 5.87 | 6.77653 | 0.875 |
| GRB10 | ILMN_1669617 | 0.00104 | 4.74E-05 | 4.41 | 1.7967 | 0.87 |
| SPIDR | ILMN_1887174 | 0.00015 | 3.07E-06 | 5.19 | 4.38784 | 0.866 |
| TCAF2 | ILMN_1780798 | 0.00214 | 0.000125 | 4.13 | 0.88458 | 0.865 |
| MGST1 | ILMN_2355168 | 0.00814 | 0.000787 | 3.55 | -0.83189 | 0.863 |
| FAM170B | ILMN_1738047 | 5.86E-05 | 7.94E-07 | 5.56 | 5.6761 | 0.862 |
| ATP2B4 | ILMN_2367753 | 0.0162 | 0.00204 | 3.24 | -1.70827 | 0.862 |
| 2-Mar | ILMN_2337789 | 0.00154 | 8.10E-05 | 4.26 | 1.29243 | 0.861 |
| FNIP2 | ILMN_1819783 | 0.0403 | 0.00738 | 2.78 | -2.87446 | 0.86 |
| ACVR1B | ILMN_2243308 | 0.000805 | 3.38E-05 | 4.51 | 2.11713 | 0.859 |
| ZDHHC19 | ILMN_1766896 | 0.0272 | 0.00428 | 2.98 | -2.38296 | 0.859 |
| CASP4 | ILMN_1778059 | 0.0742 | 0.0179 | 2.44 | -3.65928 | 0.856 |
| RNF144B | ILMN_1752526 | 0.00226 | 0.000134 | 4.1 | 0.81642 | 0.849 |
| RNF144A | ILMN_3238326 | 0.000244 | 6.29E-06 | 4.99 | 3.7072 | 0.848 |
| STK17B | ILMN_1798543 | 3.08E-07 | 3.67E-10 | 7.61 | 13.00935 | 0.846 |
| DDX60L | ILMN_3243928 | 0.0013 | 6.38E-05 | 4.33 | 1.51725 | 0.846 |
| IL1R1 | ILMN_1810584 | 1.50E-05 | 1.24E-07 | 6.06 | 7.4474 | 0.842 |
| CBS | ILMN_1804735 | 0.00307 | 0.000205 | 3.98 | 0.42328 | 0.842 |
| SYTL3 | ILMN_1720623 | 0.000175 | 3.95E-06 | 5.12 | 4.15085 | 0.838 |
| ACCS | ILMN_3187680 | 0.00311 | 0.000209 | 3.97 | 0.40459 | 0.838 |
| CD163 | ILMN_2379599 | 0.0483 | 0.0096 | 2.68 | -3.11008 | 0.838 |
| ANKRD9 | ILMN_2048607 | 0.000106 | 1.91E-06 | 5.32 | 4.84165 | 0.836 |
| ATF6 | ILMN_1703471 | 0.0252 | 0.00386 | 3.02 | -2.28918 | 0.835 |
| PER1 | ILMN_1653125 | 0.000631 | 2.44E-05 | 4.61 | 2.42353 | 0.834 |
| TMEM185B | ILMN_2231020 | 0.0103 | 0.0011 | 3.44 | -1.13753 | 0.833 |
| HIPK2 | ILMN_1687440 | 1.78E-05 | 1.60E-07 | 6 | 7.20746 | 0.832 |
| F5 | ILMN_1709233 | 0.000392 | 1.23E-05 | 4.8 | 3.06829 | 0.832 |
| ZNF252P | ILMN_3243961 | 0.000169 | 3.72E-06 | 5.14 | 4.20664 | 0.831 |
| ATPAF1 | ILMN_1790603 | 0.00466 | 0.00036 | 3.8 | -0.10513 | 0.83 |
| TMEM185B | ILMN_2231021 | 0.00462 | 0.000354 | 3.81 | -0.09013 | 0.829 |
| SLA | ILMN_2291954 | 0.0158 | 0.00198 | 3.25 | -1.68016 | 0.825 |
| SERPINB2 | ILMN_2150856 | 0.0451 | 0.00865 | 2.72 | -3.01664 | 0.824 |
| RGMA | ILMN_1717636 | 9.36E-05 | 1.60E-06 | 5.37 | 5.00677 | 0.823 |
| CARD16 | ILMN_1726591 | 0.00144 | 7.37E-05 | 4.28 | 1.38038 | 0.822 |
| 2-Mar | ILMN_1703142 | 0.00135 | 6.69E-05 | 4.31 | 1.47185 | 0.821 |
| KCNE5 | ILMN_1711650 | 0.00648 | 0.000571 | 3.66 | -0.53363 | 0.821 |
| ELL2 | ILMN_1655930 | 0.00252 | 0.000156 | 4.06 | 0.67382 | 0.819 |
| THADA | ILMN_1811624 | 0.00228 | 0.000136 | 4.1 | 0.80773 | 0.818 |
| GLMP | ILMN_1698243 | 0.0205 | 0.00286 | 3.12 | -2.0171 | 0.813 |
| HSPA1B | ILMN_1660436 | 0.0325 | 0.00546 | 2.89 | -2.60461 | 0.813 |
| MSL3 | ILMN_1713156 | 0.00272 | 0.000174 | 4.03 | 0.57699 | 0.811 |
| SETDB2 | ILMN_1731644 | 0.0135 | 0.00158 | 3.32 | -1.47196 | 0.811 |
| SOWAHC | ILMN_1724040 | 0.0207 | 0.00291 | 3.12 | -2.03216 | 0.81 |
| SLC11A1 | ILMN_1735737 | 0.00371 | 0.000259 | 3.9 | 0.2015 | 0.808 |
| SERINC3 | ILMN_1665065 | 0.000221 | 5.54E-06 | 5.03 | 3.82822 | 0.803 |
| ABCG1 | ILMN_2329927 | 2.77E-06 | 1.29E-08 | 6.66 | 9.60618 | 0.802 |
| STEAP4 | ILMN_1772036 | 0.0787 | 0.0195 | 2.41 | -3.73393 | 0.802 |
| ATP2B4 | ILMN_1680579 | 0.00911 | 0.000924 | 3.5 | -0.97998 | 0.8 |
| CD44 | ILMN_1778625 | 0.00937 | 0.000962 | 3.49 | -1.01693 | 0.8 |
| SMAP2 | ILMN_2081682 | 2.20E-05 | 2.26E-07 | 5.9 | 6.87378 | 0.795 |
| CTSL | ILMN_2374036 | 0.00628 | 0.000536 | 3.68 | -0.47576 | 0.795 |
| NUDT16 | ILMN_1781996 | 0.0398 | 0.00724 | 2.79 | -2.85771 | 0.795 |
| SAV1 | ILMN_2050654 | 0.00753 | 0.000706 | 3.59 | -0.73034 | 0.794 |
| FNIP2 | ILMN_3237329 | 0.0369 | 0.00653 | 2.83 | -2.76571 | 0.792 |
| ATL3 | ILMN_1751086 | 0.00351 | 0.000242 | 3.92 | 0.26542 | 0.787 |
| TOB1 | ILMN_1672004 | 0.0164 | 0.00208 | 3.23 | -1.72469 | 0.782 |
| TTPAL | ILMN_1744442 | 0.00333 | 0.000226 | 3.95 | 0.3301 | 0.781 |
| LOC154761 | ILMN_3237627 | 0.000579 | 2.16E-05 | 4.64 | 2.53674 | 0.78 |
| MAT2A | ILMN_1737298 | 4.83E-05 | 6.15E-07 | 5.63 | 5.91957 | 0.769 |
| HDDC3 | ILMN_1781638 | 0.0131 | 0.00151 | 3.34 | -1.43077 | 0.769 |
| ITPRIPL2 | ILMN_1751034 | 0.0217 | 0.00311 | 3.09 | -2.09404 | 0.765 |
| OSBPL1A | ILMN_2405602 | 0.00111 | 5.16E-05 | 4.39 | 1.71717 | 0.763 |
| NLRP3 | ILMN_1712026 | 0.019 | 0.00257 | 3.16 | -1.92067 | 0.762 |
| SDHAF3 | ILMN_2134039 | 0.0291 | 0.00466 | 2.95 | -2.46035 | 0.758 |
| TTPAL | ILMN_2364131 | 0.00458 | 0.000349 | 3.81 | -0.07466 | 0.755 |
| IFI16 | ILMN_1710937 | 0.00242 | 0.000149 | 4.07 | 0.72168 | 0.748 |
| CECR6 | ILMN_1702229 | 0.0159 | 0.00199 | 3.25 | -1.68573 | 0.747 |
| FIGNL2 | ILMN_1777759 | 0.034 | 0.00583 | 2.87 | -2.66274 | 0.747 |
| BLM | ILMN_1709484 | 0.0569 | 0.0122 | 2.59 | -3.32089 | 0.747 |
| IL1RL1 | ILMN_2242900 | 2.51E-05 | 2.71E-07 | 5.85 | 6.70131 | 0.746 |
| OLIG1 | ILMN_1666089 | 0.0411 | 0.00759 | 2.77 | -2.90002 | 0.746 |
| CD7 | ILMN_1792538 | 2.03E-06 | 7.02E-09 | 6.83 | 10.19183 | 0.744 |
| SSPN | ILMN_1775486 | 0.0243 | 0.00366 | 3.03 | -2.24228 | 0.744 |
| CARD16 | ILMN_1724474 | 0.00181 | 1.00E-04 | 4.19 | 1.09235 | 0.741 |
| BLVRB | ILMN_1797793 | 0.0175 | 0.00228 | 3.2 | -1.81051 | 0.741 |
| MPP1 | ILMN_1733675 | 1.60E-06 | 4.64E-09 | 6.94 | 10.58727 | 0.74 |
| PSMG1 | ILMN_1659285 | 0.0123 | 0.00139 | 3.37 | -1.35315 | 0.74 |
| PARP4 | ILMN_1776464 | 2.06E-05 | 1.95E-07 | 5.94 | 7.01617 | 0.739 |
| NLRP3 | ILMN_2310896 | 0.026 | 0.00402 | 3 | -2.32659 | 0.739 |
| TLE3 | ILMN_2234412 | 0.00125 | 6.02E-05 | 4.34 | 1.57168 | 0.738 |
| SAMSN1 | ILMN_1684887 | 0.0183 | 0.00241 | 3.18 | -1.86183 | 0.737 |
| CASP5 | ILMN_1722158 | 0.0905 | 0.0238 | 2.32 | -3.91018 | 0.736 |
| ST6GALNAC3 | ILMN_1779061 | 0.00638 | 0.000553 | 3.67 | -0.50361 | 0.732 |
| IRAK3 | ILMN_1661695 | 0.00451 | 0.000341 | 3.82 | -0.05509 | 0.729 |
| CPD | ILMN_1703074 | 0.00218 | 0.000128 | 4.12 | 0.8625 | 0.728 |
| CARD16 | ILMN_3252556 | 0.0023 | 0.000138 | 4.1 | 0.78872 | 0.728 |
| ARID5A | ILMN_2298567 | 2.20E-05 | 2.25E-07 | 5.9 | 6.87843 | 0.727 |
| MSL3 | ILMN_1670723 | 0.00102 | 4.64E-05 | 4.42 | 1.81664 | 0.726 |
| H2AFY | ILMN_1674034 | 2.77E-06 | 1.27E-08 | 6.67 | 9.62455 | 0.725 |
| ST3GAL4 | ILMN_2204545 | 0.0748 | 0.0181 | 2.44 | -3.66869 | 0.724 |
| SLC31A2 | ILMN_1758938 | 0.000182 | 4.14E-06 | 5.11 | 4.10399 | 0.723 |
| ETS2 | ILMN_1720158 | 0.0127 | 0.00144 | 3.35 | -1.38955 | 0.723 |
| PKD2L1 | ILMN_1789361 | 0.0526 | 0.0109 | 2.64 | -3.22134 | 0.723 |
| OSBPL9 | ILMN_2313856 | 4.44E-05 | 5.60E-07 | 5.66 | 6.01001 | 0.722 |
| CR1 | ILMN_1742601 | 0.0207 | 0.0029 | 3.12 | -2.02872 | 0.722 |
| 2-Mar | ILMN_1669592 | 0.00507 | 0.000407 | 3.76 | -0.21834 | 0.72 |
| CDK5RAP2 | ILMN_1655990 | 0.0163 | 0.00206 | 3.23 | -1.71777 | 0.72 |
| CTSL | ILMN_1812995 | 0.0226 | 0.0033 | 3.07 | -2.1491 | 0.719 |
| PSMG1 | ILMN_1779264 | 0.0015 | 7.82E-05 | 4.27 | 1.32481 | 0.717 |
| KCNH2 | ILMN_1739987 | 0.00202 | 0.000116 | 4.15 | 0.95628 | 0.716 |
| ARHGEF40 | ILMN_1705743 | 0.00272 | 0.000174 | 4.03 | 0.57673 | 0.715 |
| CYB5R1 | ILMN_1729237 | 3.41E-07 | 4.31E-10 | 7.56 | 12.8569 | 0.713 |
| KIF27 | ILMN_2366795 | 0.00447 | 0.000337 | 3.82 | -0.04365 | 0.712 |
| MGLL | ILMN_1657708 | 0.0634 | 0.0142 | 2.53 | -3.45692 | 0.712 |
| MRPL15 | ILMN_2103720 | 9.15E-05 | 1.55E-06 | 5.38 | 5.04193 | 0.711 |
| C11orf96 | ILMN_1677402 | 0.000877 | 3.78E-05 | 4.48 | 2.01073 | 0.709 |
| MSL3 | ILMN_3226505 | 0.00428 | 0.00032 | 3.84 | 0.0059 | 0.707 |
| HAUS4 | ILMN_1771003 | 0.0241 | 0.00361 | 3.04 | -2.22872 | 0.707 |
| ACSL1 | ILMN_1684585 | 0.00559 | 0.000461 | 3.72 | -0.33498 | 0.706 |
| ARRDC4 | ILMN_2184064 | 0.0229 | 0.00335 | 3.07 | -2.16082 | 0.706 |
| RNF13 | ILMN_1719867 | 7.47E-05 | 1.16E-06 | 5.46 | 5.31605 | 0.704 |
| SERPING1 | ILMN_1670305 | 0.0936 | 0.0249 | 2.3 | -3.95022 | 0.703 |
| COL9A2 | ILMN_1685122 | 0.0703 | 0.0165 | 2.47 | -3.59111 | 0.699 |
| GRASP | ILMN_1705210 | 0.0195 | 0.00266 | 3.15 | -1.95194 | 0.698 |
| LPCAT3 | ILMN_1805225 | 0.0407 | 0.00751 | 2.78 | -2.89035 | 0.698 |
| ERI1 | ILMN_1652716 | 0.111 | 0.0322 | 2.2 | -4.17106 | 0.694 |
| ANGPTL4 | ILMN_1707727 | 0.0885 | 0.0231 | 2.34 | -3.88486 | 0.693 |
| GDF15 | ILMN_2188862 | 0.148 | 0.049 | 2.01 | -4.52779 | 0.693 |
| UBALD2 | ILMN_1775498 | 7.50E-07 | 1.74E-09 | 7.19 | 11.5233 | 0.692 |
| MLLT1 | ILMN_1706590 | 0.041 | 0.00757 | 2.77 | -2.89758 | 0.692 |
| IFT88 | ILMN_1669366 | 4.79E-06 | 2.57E-08 | 6.48 | 8.95118 | 0.69 |
| ABHD5 | ILMN_1655702 | 1.72E-05 | 1.50E-07 | 6.01 | 7.26752 | 0.69 |
| MAPK14 | ILMN_2388090 | 0.041 | 0.00756 | 2.77 | -2.89634 | 0.686 |
| H2AFY | ILMN_1746171 | 4.91E-07 | 1.00E-09 | 7.34 | 12.05225 | 0.685 |
| MT2A | ILMN_1686664 | 0.129 | 0.04 | 2.1 | -4.35463 | 0.685 |
| ABCG1 | ILMN_1794782 | 2.77E-06 | 1.32E-08 | 6.66 | 9.58598 | 0.684 |
| IRAK3 | ILMN_1913678 | 0.00428 | 0.000319 | 3.84 | 0.00814 | 0.684 |
| RGS1 | ILMN_1656011 | 1.65E-06 | 5.32E-09 | 6.9 | 10.45566 | 0.681 |
| AKAP13 | ILMN_1752247 | 0.00419 | 0.000309 | 3.85 | 0.03775 | 0.681 |
| ADCY3 | ILMN_1676893 | 0.0585 | 0.0126 | 2.58 | -3.35492 | 0.681 |
| FGD4 | ILMN_1698728 | 0.0235 | 0.0035 | 3.05 | -2.20004 | 0.679 |
| ITGAD | ILMN_1681945 | 0.00021 | 5.18E-06 | 5.05 | 3.89125 | 0.678 |
| HLX | ILMN_2087646 | 0.064 | 0.0144 | 2.53 | -3.47055 | 0.678 |
| DHRS13 | ILMN_1790781 | 0.0848 | 0.0217 | 2.36 | -3.829 | 0.678 |
| TRIM22 | ILMN_1779252 | 0.0503 | 0.0102 | 2.66 | -3.16512 | 0.677 |
| PEX5 | ILMN_1660232 | 0.0327 | 0.00551 | 2.89 | -2.61164 | 0.675 |
| CREG1 | ILMN_1680624 | 0.00139 | 7.01E-05 | 4.3 | 1.4278 | 0.672 |
| CARD17 | ILMN_1707979 | 0.0255 | 0.00391 | 3.01 | -2.30225 | 0.671 |
| STK3 | ILMN_1666453 | 0.11 | 0.0318 | 2.2 | -4.15891 | 0.667 |
| IQCC | ILMN_1774589 | 0.00394 | 0.000281 | 3.88 | 0.12697 | 0.666 |
| FES | ILMN_1693650 | 0.00665 | 0.000593 | 3.64 | -0.56855 | 0.665 |
| MAPK14 | ILMN_1737627 | 0.039 | 0.00706 | 2.8 | -2.83526 | 0.663 |
| SYAP1 | ILMN_1698470 | 0.000304 | 8.66E-06 | 4.9 | 3.40426 | 0.66 |
| MAPK14 | ILMN_1788002 | 0.0237 | 0.00353 | 3.05 | -2.20803 | 0.659 |
| SLC26A6 | ILMN_1785252 | 0.0147 | 0.00177 | 3.28 | -1.57924 | 0.658 |
| VPS9D1 | ILMN_1693630 | 0.026 | 0.004 | 3 | -2.323 | 0.658 |
| PRKDC | ILMN_1797499 | 0.0241 | 0.00362 | 3.04 | -2.23086 | 0.656 |
| NUDT5 | ILMN_1711314 | 0.0519 | 0.0107 | 2.64 | -3.20334 | 0.656 |
| CD163 | ILMN_1722622 | 0.0761 | 0.0185 | 2.43 | -3.69115 | 0.655 |
| VMP1 | ILMN_1692754 | 1.28E-05 | 1.01E-07 | 6.12 | 7.64042 | 0.652 |
| ERV3-1 | ILMN_2118663 | 0.0013 | 6.35E-05 | 4.33 | 1.52156 | 0.651 |
| ERI1 | ILMN_3245659 | 0.0559 | 0.0119 | 2.6 | -3.30067 | 0.651 |
| FAM110B | ILMN_1679217 | 0.00646 | 0.000566 | 3.66 | -0.52628 | 0.649 |
| SLC36A1 | ILMN_2124471 | 6.46E-06 | 3.81E-08 | 6.38 | 8.57453 | 0.648 |
| CD55 | ILMN_1800540 | 0.000649 | 2.54E-05 | 4.6 | 2.38553 | 0.648 |
| SNTB2 | ILMN_1808374 | 0.00421 | 0.000312 | 3.85 | 0.02955 | 0.647 |
| IFITM1 | ILMN_1801246 | 0.000422 | 1.39E-05 | 4.77 | 2.95706 | 0.645 |
| GSTM1 | ILMN_2391861 | 7.95E-05 | 1.28E-06 | 5.43 | 5.22509 | 0.643 |
| DLGAP1-AS1 | ILMN_3307940 | 8.05E-06 | 5.34E-08 | 6.29 | 8.25187 | 0.642 |
| ARHGEF35 | ILMN_2214473 | 0.0128 | 0.00146 | 3.35 | -1.39971 | 0.642 |
| IFITM3 | ILMN_1805750 | 0.00702 | 0.00064 | 3.62 | -0.63996 | 0.639 |
| MYO10 | ILMN_2232712 | 0.0189 | 0.00254 | 3.16 | -1.90749 | 0.638 |
| ACSL3 | ILMN_2360705 | 0.000252 | 6.67E-06 | 4.98 | 3.65153 | 0.634 |
| HAL | ILMN_2074748 | 0.00047 | 1.63E-05 | 4.72 | 2.80772 | 0.634 |
| CD177 | ILMN_1774071 | 0.0784 | 0.0193 | 2.41 | -3.72751 | 0.634 |
| TPST2 | ILMN_2329679 | 0.00449 | 0.00034 | 3.82 | -0.05151 | 0.632 |
| STOX2 | ILMN_1803256 | 0.0846 | 0.0216 | 2.36 | -3.82408 | 0.632 |
| NFE2L3 | ILMN_2049766 | 0.00918 | 0.000934 | 3.5 | -0.99004 | 0.631 |
| ABCB9 | ILMN_2343048 | 0.0784 | 0.0193 | 2.41 | -3.72621 | 0.631 |
| RAB43 | ILMN_1781182 | 0.00143 | 7.26E-05 | 4.29 | 1.39504 | 0.63 |
| HECW2 | ILMN_1775268 | 0.00626 | 0.000532 | 3.68 | -0.46885 | 0.63 |
| PIK3C2B | ILMN_2117323 | 0.0148 | 0.0018 | 3.28 | -1.59375 | 0.63 |
| PODXL2 | ILMN_1657347 | 0.00666 | 0.000595 | 3.64 | -0.57143 | 0.629 |
| HOXA5 | ILMN_1753613 | 0.00128 | 6.21E-05 | 4.33 | 1.54184 | 0.628 |
| KIAA0040 | ILMN_1797191 | 0.014 | 0.00167 | 3.31 | -1.52266 | 0.627 |
| CHI3L1 | ILMN_3307868 | 0.000701 | 2.81E-05 | 4.57 | 2.28943 | 0.626 |
| RAB43 | ILMN_1696230 | 0.00277 | 0.000179 | 4.02 | 0.54755 | 0.626 |
| C1RL | ILMN_1733288 | 0.0365 | 0.00643 | 2.83 | -2.75156 | 0.626 |
| ADAM9 | ILMN_1727524 | 0.00411 | 0.000301 | 3.86 | 0.06333 | 0.624 |
| CST7 | ILMN_1679826 | 0.0385 | 0.00693 | 2.8 | -2.81849 | 0.619 |
| NTSR1 | ILMN_1778831 | 0.0429 | 0.00804 | 2.75 | -2.95199 | 0.619 |
| NUDT16P1 | ILMN_3239937 | 0.0507 | 0.0103 | 2.66 | -3.17566 | 0.616 |
| H3F3C | ILMN_2061979 | 0.0205 | 0.00286 | 3.12 | -2.01821 | 0.615 |
| SELPLG | ILMN_1734855 | 7.81E-05 | 1.23E-06 | 5.44 | 5.25719 | 0.614 |
| DNTTIP2 | ILMN_1708345 | 0.0588 | 0.0127 | 2.57 | -3.36195 | 0.614 |
| PAG1 | ILMN_2055156 | 0.00259 | 0.000163 | 4.05 | 0.63672 | 0.613 |
| SGSH | ILMN_2133675 | 0.00703 | 0.000642 | 3.62 | -0.64253 | 0.612 |
| SPINK1 | ILMN_1787266 | 0.107 | 0.0306 | 2.22 | -4.12582 | 0.61 |
| PAG1 | ILMN_1673640 | 0.0177 | 0.0023 | 3.2 | -1.81976 | 0.607 |
| USP10 | ILMN_1721116 | 0.000423 | 1.39E-05 | 4.77 | 2.95395 | 0.606 |
| CD22 | ILMN_1792075 | 0.0869 | 0.0225 | 2.35 | -3.85976 | 0.606 |
| NSMAF | ILMN_1716596 | 0.000607 | 2.31E-05 | 4.62 | 2.47568 | 0.605 |
| HAL | ILMN_1719988 | 0.00136 | 6.80E-05 | 4.31 | 1.45607 | 0.603 |
| TMEM106B | ILMN_2067607 | 0.000441 | 1.48E-05 | 4.75 | 2.89532 | 0.601 |
| DNAJB5 | ILMN_1794056 | 0.00759 | 0.000713 | 3.58 | -0.74024 | 0.601 |
| TNPO1 | ILMN_1786347 | 1.60E-06 | 4.82E-09 | 6.93 | 10.55108 | 0.6 |
| ANXA11 | ILMN_1807003 | 0.00341 | 0.000232 | 3.94 | 0.30397 | 0.6 |
| TMEM119 | ILMN_1738116 | 0.015 | 0.00182 | 3.28 | -1.60581 | 0.6 |
| CD14 | ILMN_1740015 | 0.052 | 0.0107 | 2.64 | -3.2061 | 0.6 |
| ZBTB16 | ILMN_2402817 | 0.104 | 0.0292 | 2.24 | -4.08601 | 0.599 |
| TAS2R39 | ILMN_1759475 | 0.00459 | 0.00035 | 3.81 | -0.07834 | 0.598 |
| TPST2 | ILMN_1695432 | 0.0121 | 0.00136 | 3.37 | -1.33439 | 0.598 |
| SLC7A11 | ILMN_1655229 | 0.075 | 0.0182 | 2.43 | -3.6736 | 0.596 |
| HCG27 | ILMN_1746436 | 6.64E-05 | 9.77E-07 | 5.51 | 5.47887 | 0.595 |
| ACACB | ILMN_1763852 | 0.00345 | 0.000237 | 3.93 | 0.28731 | 0.594 |
| SLA | ILMN_2345898 | 0.0138 | 0.00162 | 3.32 | -1.49636 | 0.593 |
| SDHAF3 | ILMN_1771348 | 0.0963 | 0.026 | 2.29 | -3.98653 | 0.593 |
| ZMYND15 | ILMN_1778136 | 2.15E-05 | 2.13E-07 | 5.92 | 6.93331 | 0.592 |
| RAB27A | ILMN_1665859 | 0.00129 | 6.29E-05 | 4.33 | 1.53029 | 0.592 |
| GJB6 | ILMN_2226223 | 0.0275 | 0.00433 | 2.98 | -2.3937 | 0.592 |
| SAP30 | ILMN_1700896 | 0.11 | 0.0315 | 2.21 | -4.15129 | 0.592 |
| ANO10 | ILMN_1767111 | 0.000304 | 8.70E-06 | 4.9 | 3.40058 | 0.59 |
| CABLES2 | ILMN_1762407 | 0.0557 | 0.0118 | 2.6 | -3.29537 | 0.59 |
| PAG1 | ILMN_1736806 | 0.0135 | 0.00157 | 3.32 | -1.47113 | 0.589 |
| PFKFB2 | ILMN_1796560 | 0.0189 | 0.00253 | 3.16 | -1.90673 | 0.588 |
| TSC22D3 | ILMN_2376403 | 3.64E-05 | 4.39E-07 | 5.72 | 6.24181 | 0.586 |
| BMP6 | ILMN_1747650 | 1.60E-06 | 4.70E-09 | 6.93 | 10.57372 | 0.584 |
| DYSF | ILMN_1810420 | 0.0113 | 0.00124 | 3.41 | -1.24852 | 0.584 |
| LSMEM1 | ILMN_1804895 | 5.97E-05 | 8.35E-07 | 5.55 | 5.62806 | 0.582 |
| MPRIP | ILMN_1774547 | 0.00481 | 0.000376 | 3.79 | -0.14455 | 0.58 |
| CTSA | ILMN_1719286 | 0.0298 | 0.00481 | 2.94 | -2.49023 | 0.58 |
| OSBPL9 | ILMN_2313851 | 0.00014 | 2.76E-06 | 5.22 | 4.48937 | 0.578 |
| ARID5A | ILMN_2415157 | 0.000117 | 2.18E-06 | 5.29 | 4.71409 | 0.577 |
| PDK4 | ILMN_1684982 | 0.0619 | 0.0137 | 2.55 | -3.42778 | 0.575 |
| C1orf74 | ILMN_1786470 | 0.0151 | 0.00185 | 3.27 | -1.61802 | 0.574 |
| TSPAN31 | ILMN_1725079 | 0.00103 | 4.67E-05 | 4.42 | 1.81066 | 0.573 |
| CASP1 | ILMN_2326512 | 0.00702 | 0.00064 | 3.62 | -0.63915 | 0.573 |
| SESN2 | ILMN_1751598 | 0.0116 | 0.00128 | 3.39 | -1.27755 | 0.573 |
| RNASE6 | ILMN_1780533 | 0.0122 | 0.00138 | 3.37 | -1.34717 | 0.571 |
| TNFRSF21 | ILMN_1699695 | 0.0129 | 0.00148 | 3.35 | -1.41231 | 0.571 |
| TXN | ILMN_1680314 | 0.00484 | 0.00038 | 3.78 | -0.15571 | 0.57 |
| CD99L2 | ILMN_2388142 | 0.000616 | 2.36E-05 | 4.62 | 2.45378 | 0.569 |
| LOC100128059 | ILMN_1832033 | 0.00326 | 0.00022 | 3.95 | 0.35371 | 0.569 |
| PLPP2 | ILMN_1675523 | 0.00498 | 0.000396 | 3.77 | -0.19311 | 0.569 |
| THBS1 | ILMN_1686116 | 0.00397 | 0.000284 | 3.88 | 0.11617 | 0.568 |
| RABGEF1 | ILMN_2230577 | 0.0073 | 0.000679 | 3.6 | -0.69476 | 0.568 |
| TM4SF19 | ILMN_2413644 | 0.0988 | 0.0271 | 2.27 | -4.02234 | 0.568 |
| GPX1 | ILMN_1787412 | 0.0266 | 0.00412 | 2.99 | -2.35013 | 0.567 |
| NRIP3 | ILMN_1759563 | 0.119 | 0.0357 | 2.15 | -4.25983 | 0.567 |
| PXK | ILMN_1815063 | 0.00227 | 0.000135 | 4.1 | 0.81394 | 0.566 |
| LIPN | ILMN_3243466 | 0.000303 | 8.59E-06 | 4.9 | 3.41228 | 0.564 |
| SNORD12 | ILMN_3236049 | 0.000485 | 1.71E-05 | 4.71 | 2.75972 | 0.564 |
| PPP1R3B | ILMN_1712236 | 0.0185 | 0.00247 | 3.17 | -1.8829 | 0.564 |
| SLA | ILMN_1667371 | 0.0192 | 0.00261 | 3.15 | -1.934 | 0.564 |
| CD68 | ILMN_2267914 | 0.0712 | 0.0168 | 2.47 | -3.60379 | 0.564 |
| TUBA4A | ILMN_1784300 | 0.000898 | 3.90E-05 | 4.47 | 1.97971 | 0.563 |
| LOC105372881 | ILMN_1867439 | 0.000879 | 3.80E-05 | 4.48 | 2.00624 | 0.562 |
| GSTM2 | ILMN_2201580 | 0.000494 | 1.76E-05 | 4.7 | 2.73418 | 0.561 |
| SH3BP5 | ILMN_1814773 | 0.0116 | 0.00128 | 3.39 | -1.27817 | 0.561 |
| TDRD9 | ILMN_1811110 | 0.0221 | 0.00321 | 3.08 | -2.12231 | 0.56 |
| TNFSF13B | ILMN_1758418 | 0.0637 | 0.0143 | 2.53 | -3.46621 | 0.56 |
| TSPAN17 | ILMN_1777881 | 0.144 | 0.0469 | 2.03 | -4.48975 | 0.56 |
| ZBTB16 | ILMN_1750496 | 0.048 | 0.00949 | 2.69 | -3.0994 | 0.559 |
| PNP | ILMN_2172174 | 0.0758 | 0.0184 | 2.43 | -3.68584 | 0.559 |
| AKAP13 | ILMN_2396956 | 0.00128 | 6.19E-05 | 4.34 | 1.54517 | 0.557 |
| ZNF438 | ILMN_1678494 | 0.0117 | 0.00129 | 3.39 | -1.28618 | 0.557 |
| ATL1 | ILMN_3255124 | 0.000739 | 3.02E-05 | 4.55 | 2.22162 | 0.556 |
| TXN | ILMN_2038776 | 0.00555 | 0.000455 | 3.73 | -0.32377 | 0.556 |
| MERTK | ILMN_2138589 | 0.0239 | 0.00356 | 3.04 | -2.21733 | 0.556 |
| HIPK2 | ILMN_1912737 | 0.0102 | 0.00108 | 3.45 | -1.12047 | 0.554 |
| RNF122 | ILMN_1691119 | 3.32E-05 | 3.91E-07 | 5.75 | 6.35113 | 0.552 |
| ANKRD23 | ILMN_1747151 | 0.00233 | 0.000141 | 4.09 | 0.77236 | 0.551 |
| GPX1 | ILMN_1749662 | 0.000524 | 1.90E-05 | 4.68 | 2.66133 | 0.55 |
| NDC80 | ILMN_1664511 | 0.0267 | 0.00415 | 2.99 | -2.35501 | 0.548 |
| RGS18 | ILMN_2101278 | 0.00162 | 8.70E-05 | 4.23 | 1.225 | 0.547 |
| ITPKC | ILMN_1714945 | 0.0793 | 0.0196 | 2.4 | -3.74055 | 0.547 |
| ARMCX6 | ILMN_1716026 | 0.00107 | 4.88E-05 | 4.41 | 1.76851 | 0.546 |
| PLXDC2 | ILMN_1861376 | 0.00014 | 2.76E-06 | 5.22 | 4.49067 | 0.545 |
| POR | ILMN_1677768 | 0.0406 | 0.00746 | 2.78 | -2.88484 | 0.542 |
| VNN3 | ILMN_2387680 | 0.0029 | 0.00019 | 4 | 0.49167 | 0.541 |
| AGPAT2 | ILMN_1732176 | 0.00186 | 0.000104 | 4.18 | 1.06075 | 0.54 |
| CSNK1D | ILMN_2286870 | 0.00022 | 5.44E-06 | 5.03 | 3.84504 | 0.539 |
| PLD1 | ILMN_1719696 | 0.0129 | 0.00147 | 3.35 | -1.4074 | 0.539 |
| MAFG | ILMN_1692260 | 0.0339 | 0.00578 | 2.87 | -2.65603 | 0.537 |
| TRIM9 | ILMN_1763433 | 0.0487 | 0.00973 | 2.68 | -3.12179 | 0.537 |
| EMP1 | ILMN_1801616 | 0.0523 | 0.0108 | 2.64 | -3.21477 | 0.536 |
| TNFSF13B | ILMN_2066858 | 0.0554 | 0.0117 | 2.61 | -3.28974 | 0.536 |
| MRVI1-AS1 | ILMN_3181347 | 0.0492 | 0.00991 | 2.67 | -3.1382 | 0.532 |
| PRDM8 | ILMN_1802082 | 0.00411 | 3.00E-04 | 3.86 | 0.06445 | 0.531 |
| WDSUB1 | ILMN_1735275 | 0.0354 | 0.00616 | 2.85 | -2.71262 | 0.531 |
| KLHL2 | ILMN_1701837 | 0.00929 | 0.000952 | 3.49 | -1.00744 | 0.53 |
| LOC494141 | ILMN_3292784 | 0.0209 | 0.00293 | 3.11 | -2.03956 | 0.53 |
| CXCR1 | ILMN_1662524 | 0.000297 | 8.34E-06 | 4.91 | 3.43988 | 0.529 |
| ARID5B | ILMN_1721626 | 0.000409 | 1.33E-05 | 4.78 | 2.99722 | 0.529 |
| DTNBP1 | ILMN_2408240 | 0.0263 | 0.00406 | 3 | -2.33646 | 0.529 |
| ITPR1 | ILMN_1789505 | 0.0151 | 0.00184 | 3.27 | -1.61393 | 0.528 |
| HSPA1A | ILMN_1789074 | 0.0315 | 0.00522 | 2.91 | -2.563 | 0.526 |
| CR1 | ILMN_2388112 | 0.0737 | 0.0177 | 2.45 | -3.64991 | 0.526 |
| H2AFY | ILMN_2373495 | 2.77E-06 | 1.23E-08 | 6.68 | 9.6528 | 0.524 |
| RALB | ILMN_1676358 | 0.000927 | 4.08E-05 | 4.46 | 1.93764 | 0.524 |
| UGCG | ILMN_1736939 | 0.0325 | 0.00547 | 2.89 | -2.60566 | 0.524 |
| RPS6KA2 | ILMN_1702501 | 0.0935 | 0.0249 | 2.31 | -3.94919 | 0.524 |
| TFPI | ILMN_1662619 | 0.0117 | 0.0013 | 3.39 | -1.29729 | 0.523 |
| BRSK1 | ILMN_2185845 | 0.0159 | 0.00198 | 3.25 | -1.6822 | 0.523 |
| SRPK1 | ILMN_1798804 | 0.000988 | 4.43E-05 | 4.43 | 1.85974 | 0.521 |
| ATL1 | ILMN_2381476 | 0.00905 | 0.000912 | 3.51 | -0.96752 | 0.521 |
| TEK | ILMN_2066151 | 0.0361 | 0.00633 | 2.84 | -2.73729 | 0.52 |
| ANG | ILMN_1760727 | 0.00844 | 0.000827 | 3.54 | -0.87678 | 0.519 |
| CMSS1 | ILMN_1705753 | 0.00166 | 8.97E-05 | 4.23 | 1.19603 | 0.518 |
| CYBRD1 | ILMN_1712305 | 0.0615 | 0.0136 | 2.55 | -3.42019 | 0.518 |
| DLEU2 | ILMN_1784046 | 0.0718 | 0.017 | 2.46 | -3.61519 | 0.517 |
| RAB27A | ILMN_2329773 | 0.00644 | 0.000562 | 3.66 | -0.51927 | 0.516 |
| FPR2 | ILMN_2392569 | 0.0111 | 0.0012 | 3.41 | -1.22312 | 0.516 |
| SUMF1 | ILMN_1753472 | 0.0167 | 0.00213 | 3.22 | -1.74678 | 0.516 |
| CARD17 | ILMN_3238525 | 0.0413 | 0.00766 | 2.77 | -2.90806 | 0.516 |
| TMEM187 | ILMN_2198185 | 0.0212 | 0.003 | 3.1 | -2.06062 | 0.514 |
| RNF217 | ILMN_1900520 | 0.0835 | 0.0212 | 2.37 | -3.81033 | 0.514 |
| MBNL1 | ILMN_1807304 | 0.00132 | 6.51E-05 | 4.32 | 1.4984 | 0.513 |
| KLHL6 | ILMN_1768814 | 0.0165 | 0.00209 | 3.23 | -1.73085 | 0.512 |
| GALK1 | ILMN_1803194 | 0.00135 | 6.66E-05 | 4.31 | 1.47582 | 0.511 |
| PARD6A | ILMN_1654112 | 0.0637 | 0.0143 | 2.53 | -3.46582 | 0.511 |
| CDC123 | ILMN_1678605 | 0.0803 | 0.02 | 2.4 | -3.75923 | 0.511 |
| TCN2 | ILMN_1740572 | 0.0583 | 0.0126 | 2.58 | -3.3513 | 0.51 |
| CDK5R1 | ILMN_1730928 | 0.121 | 0.0363 | 2.15 | -4.27361 | 0.508 |
| ZBED1 | ILMN_1694466 | 0.00135 | 6.76E-05 | 4.31 | 1.46275 | 0.507 |
| ATL1 | ILMN_1689251 | 0.0139 | 0.00165 | 3.31 | -1.51354 | 0.507 |
| TBC1D15 | ILMN_1803941 | 0.00195 | 0.00011 | 4.16 | 1.00125 | 0.505 |
| CD59 | ILMN_1724789 | 0.0531 | 0.0111 | 2.63 | -3.23667 | 0.505 |
| CD163 | ILMN_1733270 | 0.138 | 0.0444 | 2.06 | -4.44346 | 0.505 |
| TDRD9 | ILMN_2169490 | 0.0703 | 0.0165 | 2.47 | -3.5917 | 0.504 |
| SDC2 | ILMN_1784553 | 0.00295 | 0.000194 | 3.99 | 0.47178 | 0.503 |
| KAT8 | ILMN_1804679 | 0.0053 | 0.000429 | 3.75 | -0.26858 | 0.502 |
| RABGEF1 | ILMN_2230579 | 0.0254 | 0.0039 | 3.01 | -2.29877 | 0.502 |
| GJB6 | ILMN_1718671 | 0.00634 | 0.000544 | 3.67 | -0.48939 | 0.501 |
| EMILIN2 | ILMN_1697268 | 0.0121 | 0.00135 | 3.38 | -1.33164 | 0.501 |
| KIAA1211L | ILMN_1776121 | 0.0274 | 0.00431 | 2.98 | -2.39107 | 0.501 |
| LOC102724190 | ILMN_1855346 | 0.00952 | 0.000985 | 3.48 | -1.03847 | 0.5 |
| HDAC4 | ILMN_1764396 | 0.0797 | 0.0198 | 2.4 | -3.74949 | 0.5 |
| MBNL1 | ILMN_2313158 | 0.00633 | 0.000542 | 3.67 | -0.48488 | 0.499 |
| MAP2K1 | ILMN_1694240 | 0.0159 | 0.00199 | 3.25 | -1.68612 | 0.499 |
| YPEL4 | ILMN_1726624 | 0.052 | 0.0107 | 2.64 | -3.2047 | 0.499 |
| RAPGEF1 | ILMN_1678799 | 0.121 | 0.0365 | 2.14 | -4.27808 | 0.499 |
| CLEC5A | ILMN_1780465 | 0.129 | 0.0401 | 2.1 | -4.35699 | 0.499 |
| CSF2RA | ILMN_1661196 | 7.56E-05 | 1.18E-06 | 5.45 | 5.30043 | 0.498 |
| IL13RA1 | ILMN_1768505 | 0.008 | 0.00077 | 3.56 | -0.81161 | 0.498 |
| LINC00152 | ILMN_3220934 | 0.0307 | 0.00501 | 2.92 | -2.52657 | 0.498 |
| ZNF250 | ILMN_1757230 | 0.0183 | 0.00243 | 3.18 | -1.86694 | 0.497 |
| ARG1 | ILMN_1812281 | 0.0422 | 0.00789 | 2.76 | -2.93494 | 0.496 |
| ENTPD1 | ILMN_1773125 | 0.000736 | 3.00E-05 | 4.55 | 2.22713 | 0.494 |
| TSPAN14 | ILMN_1785060 | 0.00531 | 0.00043 | 3.75 | -0.2706 | 0.493 |
| IGF2R | ILMN_1807662 | 1.73E-05 | 1.53E-07 | 6.01 | 7.24759 | 0.491 |
| RGS12 | ILMN_1722834 | 0.121 | 0.0363 | 2.15 | -4.27246 | 0.491 |
| TMCO3 | ILMN_2220739 | 0.000165 | 3.54E-06 | 5.15 | 4.25343 | 0.49 |
| CASP1 | ILMN_2326509 | 0.0202 | 0.00279 | 3.13 | -1.99594 | 0.49 |
| DKFZP586I1420 | ILMN_2216838 | 0.00622 | 0.000528 | 3.68 | -0.46125 | 0.489 |
| DGAT2 | ILMN_1681520 | 0.0448 | 0.00855 | 2.73 | -3.00676 | 0.489 |
| NLRC4 | ILMN_1796976 | 0.0482 | 0.00957 | 2.68 | -3.10723 | 0.489 |
| HLX | ILMN_1686862 | 0.117 | 0.0348 | 2.16 | -4.23695 | 0.489 |
| MMP25 | ILMN_1717207 | 0.000104 | 1.86E-06 | 5.33 | 4.86579 | 0.487 |
| PLEKHB2 | ILMN_1697652 | 0.0138 | 0.00163 | 3.31 | -1.50186 | 0.487 |
| MLX | ILMN_1792207 | 0.00665 | 0.000593 | 3.64 | -0.56931 | 0.486 |
| ATP2B4 | ILMN_1664772 | 0.0762 | 0.0186 | 2.43 | -3.69299 | 0.486 |
| LRRC41 | ILMN_1712755 | 0.00727 | 0.000674 | 3.6 | -0.68706 | 0.485 |
| CSF2RA | ILMN_2376455 | 0.000328 | 9.74E-06 | 4.87 | 3.29336 | 0.484 |
| SLC46A3 | ILMN_1658639 | 0.0801 | 0.02 | 2.4 | -3.75628 | 0.484 |
| CEP68 | ILMN_1808500 | 0.112 | 0.0325 | 2.19 | -4.17957 | 0.484 |
| PAPSS2 | ILMN_2410929 | 0.00915 | 0.000928 | 3.5 | -0.98423 | 0.481 |
| D2HGDH | ILMN_2206272 | 0.00116 | 5.47E-05 | 4.37 | 1.66233 | 0.48 |
| PIK3IP1 | ILMN_1719986 | 0.00282 | 0.000184 | 4.01 | 0.52079 | 0.48 |
| PLXDC2 | ILMN_1753312 | 0.000101 | 1.78E-06 | 5.34 | 4.9089 | 0.479 |
| THRA | ILMN_1661683 | 0.0496 | 0.01 | 2.67 | -3.14995 | 0.479 |
| RIN2 | ILMN_1769546 | 0.0635 | 0.0143 | 2.53 | -3.46101 | 0.478 |
| GTDC1 | ILMN_1762167 | 0.0161 | 0.00203 | 3.24 | -1.70266 | 0.476 |
| LINC00426 | ILMN_3249175 | 0.0441 | 0.00836 | 2.73 | -2.98704 | 0.476 |
| RPL39L | ILMN_1712413 | 0.0447 | 0.00854 | 2.73 | -3.00542 | 0.476 |
| AGPAT2 | ILMN_1681081 | 0.0221 | 0.0032 | 3.08 | -2.11978 | 0.475 |
| ELF2 | ILMN_2252295 | 0.0076 | 0.000715 | 3.58 | -0.74288 | 0.474 |
| RPRD1A | ILMN_2209180 | 0.000146 | 2.98E-06 | 5.2 | 4.41706 | 0.473 |
| PTGER2 | ILMN_1703926 | 0.125 | 0.0382 | 2.12 | -4.31539 | 0.472 |
| CHKA | ILMN_1691151 | 0.139 | 0.0449 | 2.05 | -4.45365 | 0.472 |
| NFIL3 | ILMN_1707312 | 9.15E-05 | 1.55E-06 | 5.38 | 5.03746 | 0.471 |
| PGGHG | ILMN_1794707 | 0.000168 | 3.68E-06 | 5.14 | 4.2174 | 0.471 |
| FAM172A | ILMN_1654542 | 0.016 | 0.00201 | 3.24 | -1.69625 | 0.471 |
| CNIH4 | ILMN_1714759 | 0.0817 | 0.0205 | 2.38 | -3.78133 | 0.471 |
| RNF13 | ILMN_2339748 | 6.90E-06 | 4.26E-08 | 6.35 | 8.46929 | 0.47 |
| RN7SK | ILMN_1739423 | 0.00137 | 6.86E-05 | 4.31 | 1.44792 | 0.47 |
| IDNK | ILMN_1791306 | 0.03 | 0.00486 | 2.93 | -2.49934 | 0.47 |
| CLDN14 | ILMN_1661194 | 4.51E-06 | 2.33E-08 | 6.51 | 9.04396 | 0.468 |
| PRPS1 | ILMN_1667050 | 0.00606 | 0.000511 | 3.69 | -0.43012 | 0.468 |
| MCL1 | ILMN_1756806 | 0.00102 | 4.64E-05 | 4.42 | 1.81772 | 0.467 |
| CLDN14 | ILMN_2328575 | 0.00716 | 0.000659 | 3.61 | -0.66619 | 0.466 |
| PARD6A | ILMN_2323848 | 0.0383 | 0.00687 | 2.81 | -2.81011 | 0.466 |
| ITGAX | ILMN_1726175 | 0.000868 | 3.71E-05 | 4.49 | 2.02842 | 0.464 |
| GTDC1 | ILMN_1762741 | 0.0236 | 0.00351 | 3.05 | -2.20416 | 0.464 |
| ASPH | ILMN_2352934 | 0.0468 | 0.00912 | 2.7 | -3.06458 | 0.464 |
| NPC1 | ILMN_1713505 | 0.0865 | 0.0223 | 2.35 | -3.85351 | 0.464 |
| DDX27 | ILMN_1676759 | 0.00161 | 8.63E-05 | 4.24 | 1.23235 | 0.463 |
| COMMD4 | ILMN_2096654 | 0.0666 | 0.0153 | 2.5 | -3.52193 | 0.463 |
| APH1B | ILMN_1862217 | 0.000429 | 1.42E-05 | 4.76 | 2.93613 | 0.462 |
| NSUN4 | ILMN_1716004 | 0.0139 | 0.00164 | 3.31 | -1.50833 | 0.462 |
| ANKRD28 | ILMN_2232520 | 0.105 | 0.0297 | 2.23 | -4.09955 | 0.462 |
| PTRH2 | ILMN_2311548 | 0.000281 | 7.77E-06 | 4.93 | 3.5068 | 0.461 |
| GBA | ILMN_1755123 | 0.0656 | 0.015 | 2.51 | -3.5068 | 0.461 |
| SLC51A | ILMN_1789096 | 0.109 | 0.0313 | 2.21 | -4.14697 | 0.46 |
| ST3GAL1 | ILMN_1683313 | 0.00194 | 0.000109 | 4.17 | 1.01442 | 0.459 |
| PAPSS1 | ILMN_2224103 | 0.0869 | 0.0225 | 2.35 | -3.86006 | 0.459 |
| DNASE1L1 | ILMN_2380967 | 0.00488 | 0.000384 | 3.78 | -0.16482 | 0.458 |
| LOC100129550 | ILMN_3236239 | 0.00221 | 0.00013 | 4.11 | 0.84535 | 0.457 |
| LHFPL2 | ILMN_1747744 | 0.0319 | 0.00531 | 2.9 | -2.57958 | 0.457 |
| SLC25A25 | ILMN_1791728 | 0.0138 | 0.00162 | 3.31 | -1.49836 | 0.456 |
| ATP6V1F | ILMN_2099783 | 0.0162 | 0.00203 | 3.24 | -1.70613 | 0.456 |
| CYSTM1 | ILMN_1761566 | 0.00789 | 0.000755 | 3.57 | -0.79324 | 0.455 |
| FURIN | ILMN_1790228 | 0.00962 | 0.000997 | 3.48 | -1.05003 | 0.455 |
| UBC | ILMN_2252160 | 0.0361 | 0.00633 | 2.84 | -2.73668 | 0.455 |
| DHRS12 | ILMN_1669177 | 0.0481 | 0.0095 | 2.69 | -3.10123 | 0.455 |
| POLR3GL | ILMN_1760667 | 0.0031 | 0.000208 | 3.97 | 0.40877 | 0.452 |
| KDSR | ILMN_3241164 | 0.054 | 0.0113 | 2.62 | -3.25609 | 0.452 |
| RFESD | ILMN_1802162 | 0.0233 | 0.00344 | 3.06 | -2.18501 | 0.451 |
| CYBRD1 | ILMN_2087692 | 0.073 | 0.0175 | 2.45 | -3.63913 | 0.451 |
| GNAQ | ILMN_1659923 | 0.0761 | 0.0185 | 2.43 | -3.68948 | 0.451 |
| PCYOX1 | ILMN_2113535 | 0.011 | 0.00119 | 3.42 | -1.21155 | 0.45 |
| MBP | ILMN_2331544 | 0.000196 | 4.60E-06 | 5.08 | 4.00521 | 0.449 |
| SPTSSA | ILMN_1699676 | 0.0732 | 0.0175 | 2.45 | -3.64286 | 0.449 |
| KLF6 | ILMN_1735014 | 9.01E-07 | 2.15E-09 | 7.14 | 11.32205 | 0.448 |
| MED20 | ILMN_1777526 | 0.00032 | 9.43E-06 | 4.88 | 3.32336 | 0.447 |
| COLGALT1 | ILMN_1727043 | 0.00789 | 0.000755 | 3.57 | -0.79291 | 0.447 |
| TSHZ1 | ILMN_1718907 | 0.0129 | 0.00148 | 3.34 | -1.41564 | 0.447 |
| CIPC | ILMN_1788347 | 0.000438 | 1.46E-05 | 4.75 | 2.90671 | 0.446 |
| SNORD89 | ILMN_3238662 | 0.00151 | 7.92E-05 | 4.26 | 1.31356 | 0.445 |
| PPFIBP2 | ILMN_1675656 | 0.0107 | 0.00114 | 3.43 | -1.17576 | 0.445 |
| METTL4 | ILMN_1738173 | 0.0132 | 0.00153 | 3.33 | -1.44494 | 0.445 |
| MGST1 | ILMN_1781952 | 0.0424 | 0.00793 | 2.75 | -2.93933 | 0.445 |
| CLEC7A | ILMN_1654504 | 0.0434 | 0.00821 | 2.74 | -2.97022 | 0.445 |
| RASA2 | ILMN_1703688 | 0.00135 | 6.68E-05 | 4.31 | 1.47341 | 0.443 |
| CYTIP | ILMN_2092041 | 0.00276 | 0.000178 | 4.02 | 0.55178 | 0.443 |
| TSC22D3 | ILMN_1748124 | 0.000154 | 3.20E-06 | 5.18 | 4.35074 | 0.441 |
| MCM2 | ILMN_1681503 | 0.0602 | 0.0132 | 2.56 | -3.39272 | 0.441 |
| ATPIF1 | ILMN_1727332 | 0.00852 | 0.000837 | 3.53 | -0.88792 | 0.44 |
| PLEKHB2 | ILMN_2297710 | 0.0117 | 0.00131 | 3.39 | -1.29913 | 0.44 |
| CD44 | ILMN_2348788 | 0.0823 | 0.0207 | 2.38 | -3.79 | 0.44 |
| CREB5 | ILMN_1728677 | 0.093 | 0.0247 | 2.31 | -3.9422 | 0.44 |
| IL7R | ILMN_2342579 | 0.00017 | 3.75E-06 | 5.14 | 4.19808 | 0.439 |
| ARMCX6 | ILMN_2414533 | 0.00125 | 5.95E-05 | 4.35 | 1.58174 | 0.439 |
| EXOC6 | ILMN_1651628 | 0.019 | 0.00257 | 3.16 | -1.91937 | 0.439 |
| ATP13A3 | ILMN_1663684 | 0.028 | 0.00444 | 2.97 | -2.41763 | 0.439 |
| CDH26 | ILMN_1765255 | 0.0456 | 0.00878 | 2.72 | -3.03087 | 0.439 |
| ZADH2 | ILMN_1795063 | 0.00224 | 0.000132 | 4.11 | 0.83178 | 0.438 |
| UNC45A | ILMN_1726434 | 0.00257 | 0.000161 | 4.05 | 0.6478 | 0.438 |
| LONP2 | ILMN_1691480 | 0.0267 | 0.00415 | 2.99 | -2.35605 | 0.438 |
| PELI1 | ILMN_1679268 | 0.000308 | 8.83E-06 | 4.9 | 3.38624 | 0.437 |
| ASAH1 | ILMN_1684054 | 0.000113 | 2.09E-06 | 5.3 | 4.75466 | 0.436 |
| CYTIP | ILMN_3235928 | 0.00055 | 2.01E-05 | 4.66 | 2.60728 | 0.436 |
| PITPNC1 | ILMN_1670638 | 0.000879 | 3.80E-05 | 4.48 | 2.00555 | 0.436 |
| CCNH | ILMN_1742250 | 0.00121 | 5.73E-05 | 4.36 | 1.61782 | 0.436 |
| CLEC12A | ILMN_1663142 | 0.00261 | 0.000164 | 4.04 | 0.6294 | 0.436 |
| XRN2 | ILMN_1727617 | 0.0295 | 0.00475 | 2.94 | -2.47792 | 0.436 |
| TMEM187 | ILMN_3242211 | 0.0719 | 0.0171 | 2.46 | -3.61897 | 0.436 |
| DNAAF1 | ILMN_1776967 | 0.114 | 0.0335 | 2.18 | -4.20461 | 0.436 |
| TREM1 | ILMN_1688231 | 0.000311 | 9.00E-06 | 4.89 | 3.36794 | 0.435 |
| TMEM181 | ILMN_1710078 | 0.000348 | 1.06E-05 | 4.84 | 3.21351 | 0.435 |
| GSR | ILMN_1775182 | 0.035 | 0.00608 | 2.85 | -2.70116 | 0.435 |
| PPT1 | ILMN_1669273 | 1.48E-05 | 1.21E-07 | 6.07 | 7.46731 | 0.434 |
| MLX | ILMN_2401618 | 0.00728 | 0.000677 | 3.6 | -0.69195 | 0.434 |
| FPR2 | ILMN_1740875 | 0.013 | 0.0015 | 3.34 | -1.42366 | 0.434 |
| YIPF1 | ILMN_1803564 | 0.11 | 0.0317 | 2.2 | -4.15741 | 0.434 |
| ABHD2 | ILMN_1723662 | 0.000615 | 2.36E-05 | 4.62 | 2.45628 | 0.433 |
| MGAM | ILMN_1714643 | 0.00125 | 5.97E-05 | 4.35 | 1.57996 | 0.433 |
| C9orf72 | ILMN_1762508 | 0.00524 | 0.000423 | 3.75 | -0.25477 | 0.433 |
| SLF2 | ILMN_3233135 | 0.00601 | 0.000506 | 3.69 | -0.42154 | 0.433 |
| SPACA6 | ILMN_3247848 | 0.00407 | 0.000296 | 3.86 | 0.07796 | 0.432 |
| SLC12A9 | ILMN_1695962 | 0.0518 | 0.0106 | 2.64 | -3.19946 | 0.432 |
| TMEM106B | ILMN_1726288 | 0.00048 | 1.68E-05 | 4.71 | 2.77462 | 0.431 |
| TMEM2 | ILMN_1784661 | 0.00155 | 8.16E-05 | 4.25 | 1.2849 | 0.431 |
| PPP1R3D | ILMN_1781198 | 0.014 | 0.00167 | 3.31 | -1.52288 | 0.431 |
| HN1 | ILMN_2384591 | 0.0161 | 0.00203 | 3.24 | -1.70428 | 0.43 |
| SV2A | ILMN_1702009 | 0.0149 | 0.00181 | 3.28 | -1.59748 | 0.429 |
| FNDC3A | ILMN_1705111 | 0.0162 | 0.00204 | 3.24 | -1.70805 | 0.429 |
| PEX11G | ILMN_1710000 | 0.0186 | 0.00247 | 3.17 | -1.88501 | 0.429 |
| ACOT13 | ILMN_2098743 | 0.00484 | 0.00038 | 3.78 | -0.15572 | 0.428 |
| ZC3H18 | ILMN_1658834 | 0.0135 | 0.00158 | 3.32 | -1.47476 | 0.428 |
| DCTN6 | ILMN_1744059 | 0.00186 | 0.000103 | 4.18 | 1.06191 | 0.427 |
| ELF2 | ILMN_1691559 | 0.0117 | 0.00129 | 3.39 | -1.28805 | 0.427 |
| SH3GLB1 | ILMN_1766045 | 0.00123 | 5.85E-05 | 4.35 | 1.59878 | 0.426 |
| RFPL4A | ILMN_3227341 | 0.00868 | 0.000863 | 3.52 | -0.91689 | 0.426 |
| NECTIN2 | ILMN_1718303 | 0.071 | 0.0167 | 2.47 | -3.60068 | 0.426 |
| IL4R | ILMN_1652185 | 0.144 | 0.0472 | 2.03 | -4.4947 | 0.426 |
| IDI1 | ILMN_1755075 | 0.115 | 0.0341 | 2.17 | -4.21874 | 0.425 |
| TMCC3 | ILMN_1685493 | 0.00801 | 0.000772 | 3.56 | -0.81398 | 0.424 |
| SPRY1 | ILMN_2329914 | 0.0217 | 0.00311 | 3.09 | -2.09405 | 0.424 |
| GBA | ILMN_2364110 | 0.0682 | 0.0158 | 2.49 | -3.55221 | 0.424 |
| BST1 | ILMN_1770161 | 0.0818 | 0.0206 | 2.38 | -3.78275 | 0.424 |
| PCNX1 | ILMN_1740010 | 0.00109 | 5.02E-05 | 4.4 | 1.74181 | 0.422 |
| HMGB2 | ILMN_1654268 | 0.0057 | 0.000473 | 3.72 | -0.35939 | 0.422 |
| TET2 | ILMN_1788818 | 0.022 | 0.00317 | 3.09 | -2.11006 | 0.422 |
| SNORD12C | ILMN_3249286 | 0.00869 | 0.000865 | 3.52 | -0.91905 | 0.421 |
| FUS | ILMN_2306066 | 0.0247 | 0.00375 | 3.03 | -2.26404 | 0.421 |
| RHOT1 | ILMN_1785762 | 0.059 | 0.0128 | 2.57 | -3.3675 | 0.421 |
| MGAT5 | ILMN_1816244 | 0.00771 | 0.000727 | 3.58 | -0.75824 | 0.42 |
| SHB | ILMN_1732612 | 0.0242 | 0.00365 | 3.04 | -2.23967 | 0.42 |
| ECT2 | ILMN_1717173 | 0.0509 | 0.0104 | 2.65 | -3.18014 | 0.42 |
| C3AR1 | ILMN_1787529 | 0.018 | 0.00236 | 3.19 | -1.84303 | 0.419 |
| RFC2 | ILMN_1655733 | 0.0434 | 0.00818 | 2.74 | -2.9675 | 0.418 |
| MAML3 | ILMN_1795499 | 0.0551 | 0.0117 | 2.61 | -3.28302 | 0.418 |
| GBGT1 | ILMN_1652906 | 0.0182 | 0.0024 | 3.18 | -1.85798 | 0.417 |
| TNFSF8 | ILMN_1761778 | 0.0429 | 0.00805 | 2.75 | -2.95276 | 0.417 |
| NEDD8 | ILMN_1785711 | 0.00367 | 0.000256 | 3.91 | 0.21513 | 0.416 |
| JADE2 | ILMN_1795285 | 0.0181 | 0.00237 | 3.19 | -1.84732 | 0.416 |
| IL36G | ILMN_2158713 | 0.0595 | 0.013 | 2.57 | -3.38 | 0.416 |
| NET1 | ILMN_1758311 | 0.0645 | 0.0146 | 2.52 | -3.48491 | 0.416 |
| TNS3 | ILMN_1667893 | 0.0967 | 0.0262 | 2.28 | -3.99177 | 0.416 |
| CD68 | ILMN_2359907 | 0.0429 | 0.00807 | 2.75 | -2.95464 | 0.415 |
| PAPSS1 | ILMN_1781819 | 0.0473 | 0.00927 | 2.7 | -3.0787 | 0.415 |
| PTPRE | ILMN_1756814 | 0.059 | 0.0128 | 2.57 | -3.36766 | 0.415 |
| PUS3 | ILMN_1694147 | 0.134 | 0.0425 | 2.08 | -4.40717 | 0.415 |
| KIAA1211L | ILMN_3243366 | 0.0456 | 0.00878 | 2.72 | -3.03088 | 0.414 |
| OASL | ILMN_1681721 | 0.113 | 0.0328 | 2.19 | -4.18661 | 0.414 |
| THRA | ILMN_1718182 | 0.0292 | 0.00469 | 2.95 | -2.46591 | 0.413 |
| APH1B | ILMN_1767816 | 0.000615 | 2.36E-05 | 4.62 | 2.45632 | 0.412 |
| ALDH3A2 | ILMN_1794825 | 0.0046 | 0.000352 | 3.81 | -0.08344 | 0.412 |
| TNFAIP3 | ILMN_1702691 | 0.0086 | 0.000846 | 3.53 | -0.89851 | 0.412 |
| SIRT5 | ILMN_1799598 | 0.0117 | 0.0013 | 3.39 | -1.29655 | 0.412 |
| TMLHE | ILMN_1707002 | 0.0177 | 0.00232 | 3.19 | -1.82495 | 0.412 |
| MAZ | ILMN_1677997 | 0.0263 | 0.00408 | 3 | -2.33948 | 0.411 |
| MS4A6A | ILMN_1797731 | 0.11 | 0.0316 | 2.21 | -4.15438 | 0.411 |
| STXBP5 | ILMN_1684402 | 0.000863 | 3.67E-05 | 4.49 | 2.03879 | 0.41 |
| ABCG1 | ILMN_1658176 | 0.00405 | 0.000294 | 3.86 | 0.08428 | 0.41 |
| GSTK1 | ILMN_1725241 | 0.015 | 0.00183 | 3.27 | -1.60958 | 0.41 |
| RBM47 | ILMN_1689046 | 0.0249 | 0.00379 | 3.02 | -2.27274 | 0.409 |
| E2F2 | ILMN_1777233 | 0.148 | 0.0489 | 2.01 | -4.526 | 0.409 |
| IL17RA | ILMN_1728724 | 0.011 | 0.00119 | 3.42 | -1.2116 | 0.408 |
| PHC2 | ILMN_1670147 | 0.0977 | 0.0266 | 2.28 | -4.00714 | 0.408 |
| GLA | ILMN_1766637 | 0.0347 | 0.006 | 2.86 | -2.68865 | 0.407 |
| ATPIF1 | ILMN_1685978 | 0.0035 | 0.000241 | 3.93 | 0.27048 | 0.406 |
| RBM47 | ILMN_3224926 | 0.00994 | 0.00104 | 3.46 | -1.09217 | 0.406 |
| PPP2R2B | ILMN_2298365 | 0.000145 | 2.92E-06 | 5.21 | 4.43698 | 0.405 |
| IKBIP | ILMN_2324994 | 0.0169 | 0.00217 | 3.22 | -1.76569 | 0.405 |
| ABCG1 | ILMN_1695968 | 0.0244 | 0.00368 | 3.03 | -2.24676 | 0.405 |
| CAPN15 | ILMN_1763739 | 0.0914 | 0.0241 | 2.32 | -3.921 | 0.405 |
| MAPKAPK2 | ILMN_1774844 | 0.00212 | 0.000123 | 4.13 | 0.8974 | 0.404 |
| VAPA | ILMN_1690822 | 0.00726 | 0.000672 | 3.6 | -0.6845 | 0.404 |
| CASP2 | ILMN_2410540 | 0.0111 | 0.00121 | 3.41 | -1.22976 | 0.404 |
| KLHL8 | ILMN_2189222 | 0.0307 | 0.00502 | 2.92 | -2.52777 | 0.404 |
| ZNF320 | ILMN_2103480 | 0.0469 | 0.00917 | 2.7 | -3.06917 | 0.404 |
| MBP | ILMN_2398939 | 0.000439 | 1.47E-05 | 4.75 | 2.90171 | 0.403 |
| POLR1E | ILMN_2110167 | 0.00368 | 0.000256 | 3.91 | 0.21308 | 0.403 |
| TUBA1A | ILMN_1742981 | 0.0359 | 0.00627 | 2.84 | -2.72862 | 0.403 |
| ARHGEF11 | ILMN_1733861 | 0.0361 | 0.00635 | 2.84 | -2.7396 | 0.403 |
| RNASE1 | ILMN_2333670 | 0.0718 | 0.017 | 2.46 | -3.61562 | 0.403 |
| S1PR4 | ILMN_1784737 | 0.00794 | 0.000762 | 3.56 | -0.80111 | 0.402 |
| FAM45A | ILMN_1691760 | 0.0142 | 0.0017 | 3.3 | -1.54376 | 0.402 |
| SWAP70 | ILMN_1785175 | 0.00236 | 0.000143 | 4.09 | 0.75974 | 0.401 |
| SLC22A1 | ILMN_1715742 | 0.0277 | 0.00438 | 2.97 | -2.40508 | 0.401 |
| CES1 | ILMN_2359945 | 0.0304 | 0.00494 | 2.93 | -2.51415 | 0.401 |
| ACTN1 | ILMN_2232177 | 0.00046 | 1.57E-05 | 4.73 | 2.83989 | 0.4 |
| BEST1 | ILMN_1718982 | 0.000439 | 1.47E-05 | 4.75 | 2.90247 | 0.399 |
| ERLIN2 | ILMN_1669729 | 0.021 | 0.00297 | 3.11 | -2.05059 | 0.399 |
| TNPO1 | ILMN_1837935 | 0.000202 | 4.82E-06 | 5.07 | 3.96073 | 0.397 |
| MCL1 | ILMN_1803988 | 0.000344 | 1.04E-05 | 4.85 | 3.22901 | 0.397 |
| SP100 | ILMN_1690920 | 0.00862 | 0.00085 | 3.53 | -0.90266 | 0.397 |
| EIF4EBP3 | ILMN_2197225 | 0.00157 | 8.35E-05 | 4.25 | 1.263 | 0.396 |
| GRAMD1B | ILMN_3237376 | 0.0341 | 0.00584 | 2.87 | -2.66456 | 0.395 |
| H6PD | ILMN_1721136 | 0.0776 | 0.0191 | 2.41 | -3.71631 | 0.395 |
| TAZ | ILMN_2358733 | 0.00646 | 0.000566 | 3.66 | -0.5252 | 0.394 |
| FAM207A | ILMN_2104924 | 0.0167 | 0.00212 | 3.22 | -1.74261 | 0.394 |
| CHSY1 | ILMN_1791576 | 0.0389 | 0.00702 | 2.8 | -2.83015 | 0.394 |
| PITPNC1 | ILMN_1738796 | 0.0601 | 0.0132 | 2.56 | -3.39077 | 0.394 |
| ABHD2 | ILMN_2403446 | 0.00421 | 0.000312 | 3.85 | 0.02928 | 0.393 |
| TMLHE | ILMN_1683575 | 0.0083 | 0.000808 | 3.54 | -0.85559 | 0.393 |
| BCAT1 | ILMN_1766169 | 0.00888 | 0.000889 | 3.51 | -0.94454 | 0.393 |
| SLC8B1 | ILMN_1701655 | 0.0092 | 0.000938 | 3.5 | -0.99352 | 0.393 |
| NTN3 | ILMN_1656040 | 0.0702 | 0.0165 | 2.47 | -3.58865 | 0.393 |
| CCNH | ILMN_2187830 | 0.0164 | 0.00207 | 3.23 | -1.72095 | 0.392 |
| ACSL3 | ILMN_1666096 | 0.0362 | 0.00637 | 2.84 | -2.74282 | 0.392 |
| RALGAPA2 | ILMN_1726333 | 0.0476 | 0.00937 | 2.69 | -3.08881 | 0.392 |
| IGFLR1 | ILMN_1786426 | 0.0801 | 0.0199 | 2.4 | -3.75503 | 0.392 |
| FBXL5 | ILMN_1673370 | 0.108 | 0.0307 | 2.22 | -4.12831 | 0.392 |
| MS4A6A | ILMN_1721035 | 0.023 | 0.00339 | 3.06 | -2.17174 | 0.391 |
| FAM134B | ILMN_1811330 | 0.0265 | 0.00411 | 2.99 | -2.34793 | 0.391 |
| ARRDC4 | ILMN_1660544 | 0.104 | 0.0292 | 2.24 | -4.08553 | 0.391 |
| VNN3 | ILMN_1804935 | 0.0171 | 0.0022 | 3.21 | -1.77978 | 0.39 |
| DENND1A | ILMN_1727315 | 0.0294 | 0.00472 | 2.94 | -2.47331 | 0.39 |
| CTNNAL1 | ILMN_1721901 | 0.0341 | 0.00584 | 2.87 | -2.66421 | 0.39 |
| GAA | ILMN_2410783 | 0.0152 | 0.00187 | 3.27 | -1.6277 | 0.389 |
| C1orf162 | ILMN_1754894 | 0.0314 | 0.00517 | 2.91 | -2.55446 | 0.389 |
| LOC401052 | ILMN_1791423 | 0.056 | 0.0119 | 2.6 | -3.30353 | 0.389 |
| SYN2 | ILMN_1781060 | 0.0231 | 0.00341 | 3.06 | -2.17713 | 0.388 |
| CHST1 | ILMN_1677719 | 0.0281 | 0.00446 | 2.96 | -2.42204 | 0.388 |
| BMX | ILMN_1796138 | 0.097 | 0.0263 | 2.28 | -3.99724 | 0.388 |
| SCNN1A | ILMN_1713995 | 0.108 | 0.031 | 2.21 | -4.13666 | 0.388 |
| SERINC3 | ILMN_1815656 | 0.00251 | 0.000156 | 4.06 | 0.67896 | 0.387 |
| GJB2 | ILMN_1769388 | 0.0481 | 0.00949 | 2.69 | -3.09993 | 0.387 |
| NDFIP1 | ILMN_1751452 | 0.0774 | 0.019 | 2.42 | -3.71197 | 0.386 |
| RPL39L | ILMN_2108357 | 0.129 | 0.0403 | 2.1 | -4.36253 | 0.386 |
| MIR23A | ILMN_3309224 | 0.149 | 0.0493 | 2.01 | -4.53136 | 0.386 |
| ZNF821 | ILMN_2151075 | 0.0127 | 0.00144 | 3.35 | -1.39154 | 0.385 |
| HPS1 | ILMN_2267787 | 0.0198 | 0.00271 | 3.14 | -1.96978 | 0.385 |
| ODF2L | ILMN_1702073 | 0.0326 | 0.00549 | 2.89 | -2.60923 | 0.385 |
| CLCN7 | ILMN_1694731 | 0.137 | 0.0439 | 2.06 | -4.43476 | 0.385 |
| KLHL12 | ILMN_2164081 | 0.000245 | 6.33E-06 | 4.99 | 3.70114 | 0.384 |
| ABHD4 | ILMN_1700633 | 0.00131 | 6.46E-05 | 4.32 | 1.5051 | 0.383 |
| AGO2 | ILMN_1695719 | 0.00841 | 0.000821 | 3.54 | -0.87046 | 0.383 |
| CMSS1 | ILMN_2215545 | 0.0117 | 0.0013 | 3.39 | -1.29724 | 0.383 |
| FAM19A2 | ILMN_1691876 | 0.0807 | 0.0201 | 2.39 | -3.76459 | 0.383 |
| ZNF746 | ILMN_1706342 | 0.000205 | 4.97E-06 | 5.06 | 3.93116 | 0.382 |
| PHF13 | ILMN_1761560 | 0.00421 | 0.00031 | 3.85 | 0.03447 | 0.382 |
| LGALSL | ILMN_1673548 | 0.0204 | 0.00284 | 3.12 | -2.01114 | 0.382 |
| JMJD1C | ILMN_2410742 | 0.00135 | 6.75E-05 | 4.31 | 1.46352 | 0.381 |
| ZDHHC20 | ILMN_1654141 | 0.048 | 0.00947 | 2.69 | -3.09766 | 0.381 |
| ZCWPW1 | ILMN_1751963 | 0.000635 | 2.47E-05 | 4.6 | 2.41085 | 0.38 |
| GADD45B | ILMN_1718977 | 0.015 | 0.00183 | 3.27 | -1.60885 | 0.38 |
| LINC00173 | ILMN_3311190 | 0.0336 | 0.00572 | 2.88 | -2.64585 | 0.38 |
| IL1RL2 | ILMN_1762810 | 0.0771 | 0.0189 | 2.42 | -3.70833 | 0.38 |
| LIX1L | ILMN_1708098 | 0.0878 | 0.0228 | 2.34 | -3.8741 | 0.38 |
| HP | ILMN_1812433 | 0.118 | 0.0351 | 2.16 | -4.24356 | 0.38 |
| ADIPOR1 | ILMN_1688322 | 1.08E-06 | 2.71E-09 | 7.08 | 11.10131 | 0.379 |
| DIRC2 | ILMN_1793743 | 0.000721 | 2.93E-05 | 4.55 | 2.2521 | 0.379 |
| FCMR | ILMN_1775542 | 0.00488 | 0.000385 | 3.78 | -0.16723 | 0.379 |
| MGAT5 | ILMN_3238058 | 0.00789 | 0.000754 | 3.57 | -0.79158 | 0.378 |
| CTDP1 | ILMN_2407589 | 0.0109 | 0.00118 | 3.42 | -1.20343 | 0.378 |
| RIMKLB | ILMN_3245707 | 0.0297 | 0.00478 | 2.94 | -2.48461 | 0.378 |
| MCOLN1 | ILMN_1764383 | 0.00727 | 0.000674 | 3.6 | -0.68778 | 0.377 |
| EBPL | ILMN_1805922 | 0.0877 | 0.0228 | 2.34 | -3.87258 | 0.377 |
| PIR | ILMN_1761247 | 0.144 | 0.0469 | 2.03 | -4.48933 | 0.377 |
| TNPO1 | ILMN_1763627 | 0.0141 | 0.00168 | 3.3 | -1.53269 | 0.376 |
| C9orf72 | ILMN_2296677 | 0.0673 | 0.0155 | 2.5 | -3.53577 | 0.376 |
| HOXC6 | ILMN_1794492 | 0.023 | 0.00337 | 3.06 | -2.16807 | 0.375 |
| NEK6 | ILMN_1660871 | 0.0832 | 0.0211 | 2.37 | -3.80438 | 0.375 |
| KIF21B | ILMN_2163819 | 0.0874 | 0.0227 | 2.34 | -3.86869 | 0.375 |
| B4GALT4 | ILMN_2380688 | 0.144 | 0.0472 | 2.03 | -4.49615 | 0.375 |
| CIPC | ILMN_2097858 | 0.00181 | 9.95E-05 | 4.19 | 1.09896 | 0.374 |
| TPP1 | ILMN_1729234 | 0.0185 | 0.00246 | 3.17 | -1.87866 | 0.374 |
| POC1A | ILMN_1780667 | 0.0833 | 0.0211 | 2.37 | -3.80519 | 0.374 |
| UHRF2 | ILMN_1691444 | 0.0834 | 0.0212 | 2.37 | -3.80722 | 0.374 |
| DLX4 | ILMN_1771113 | 0.00911 | 0.000924 | 3.5 | -0.97997 | 0.373 |
| AGFG1 | ILMN_1792497 | 0.0131 | 0.00152 | 3.34 | -1.43582 | 0.373 |
| YBX3 | ILMN_1782788 | 0.0452 | 0.00866 | 2.72 | -3.01794 | 0.373 |
| FRMD3 | ILMN_1698725 | 0.0539 | 0.0113 | 2.62 | -3.25413 | 0.373 |
| NCLN | ILMN_3237632 | 0.00926 | 0.000947 | 3.49 | -1.00219 | 0.372 |
| RBM47 | ILMN_3306730 | 0.024 | 0.00358 | 3.04 | -2.22269 | 0.372 |
| CDK5R1 | ILMN_2062271 | 0.0875 | 0.0227 | 2.34 | -3.86913 | 0.372 |
| LCOR | ILMN_1689817 | 0.00254 | 0.000158 | 4.05 | 0.66359 | 0.371 |
| EPS15 | ILMN_1665357 | 0.0037 | 0.000259 | 3.9 | 0.20338 | 0.371 |
| ERN1 | ILMN_1731231 | 0.00638 | 0.000552 | 3.67 | -0.50221 | 0.371 |
| OLFM4 | ILMN_2116877 | 0.0231 | 0.00341 | 3.06 | -2.17717 | 0.371 |
| MDM2 | ILMN_1736829 | 0.034 | 0.00581 | 2.87 | -2.65953 | 0.371 |
| AGPAT2 | ILMN_2377430 | 0.0827 | 0.0209 | 2.38 | -3.79469 | 0.371 |
| YIPF1 | ILMN_2052163 | 0.0881 | 0.023 | 2.34 | -3.87978 | 0.371 |
| ZCCHC6 | ILMN_1779677 | 6.19E-06 | 3.56E-08 | 6.4 | 8.63917 | 0.37 |
| ZNF107 | ILMN_1766974 | 0.0147 | 0.00177 | 3.28 | -1.58024 | 0.37 |
| TUBA1C | ILMN_1742167 | 0.0167 | 0.00214 | 3.22 | -1.75171 | 0.37 |
| YPEL2 | ILMN_1677098 | 0.0328 | 0.00554 | 2.89 | -2.61644 | 0.37 |
| GAA | ILMN_1765801 | 0.0794 | 0.0197 | 2.4 | -3.74351 | 0.37 |
| HK3 | ILMN_1670302 | 0.00412 | 0.000302 | 3.86 | 0.05942 | 0.369 |
| SLC7A5P2 | ILMN_1671355 | 0.0255 | 0.00392 | 3.01 | -2.30394 | 0.369 |
| ADGRE5 | ILMN_1676718 | 0.0324 | 0.00543 | 2.89 | -2.59928 | 0.369 |
| ENTPD6 | ILMN_2091792 | 0.0794 | 0.0196 | 2.4 | -3.74217 | 0.369 |
| FYB | ILMN_2280548 | 0.108 | 0.031 | 2.21 | -4.13645 | 0.369 |
| HS1BP3 | ILMN_1738237 | 0.00234 | 0.000142 | 4.09 | 0.76819 | 0.368 |
| DICER1 | ILMN_2349831 | 0.083 | 0.021 | 2.38 | -3.80005 | 0.368 |
| KEAP1 | ILMN_2410771 | 0.0972 | 0.0264 | 2.28 | -4.00049 | 0.368 |
| HAVCR2 | ILMN_1693826 | 0.00055 | 2.02E-05 | 4.66 | 2.60038 | 0.367 |
| GPR34 | ILMN_1701947 | 0.101 | 0.028 | 2.26 | -4.05026 | 0.367 |
| TLR4 | ILMN_1706217 | 0.00667 | 0.000597 | 3.64 | -0.57451 | 0.366 |
| ARL11 | ILMN_1808383 | 0.0337 | 0.00574 | 2.87 | -2.64905 | 0.366 |
| WDR13 | ILMN_1805512 | 0.072 | 0.0171 | 2.46 | -3.61959 | 0.366 |
| CXCL16 | ILMN_1728478 | 9.35E-07 | 2.29E-09 | 7.12 | 11.26026 | 0.365 |
| TEK | ILMN_1751576 | 0.0926 | 0.0246 | 2.31 | -3.93712 | 0.365 |
| OASL | ILMN_1674811 | 0.123 | 0.0373 | 2.13 | -4.29608 | 0.365 |
| ADAT1 | ILMN_1657139 | 0.0027 | 0.000172 | 4.03 | 0.58762 | 0.364 |
| RNF213 | ILMN_2289093 | 0.135 | 0.0428 | 2.07 | -4.41192 | 0.364 |
| ZFAND5 | ILMN_1795228 | 0.00216 | 0.000126 | 4.12 | 0.87363 | 0.363 |
| SLC38A1 | ILMN_1769911 | 0.0207 | 0.00289 | 3.12 | -2.02672 | 0.363 |
| MBOAT1 | ILMN_1764082 | 0.0292 | 0.00468 | 2.95 | -2.46417 | 0.363 |
| FNDC3A | ILMN_2362581 | 0.0368 | 0.00652 | 2.83 | -2.76384 | 0.363 |
| GADD45A | ILMN_1694075 | 0.0571 | 0.0122 | 2.59 | -3.3259 | 0.363 |
| C16orf72 | ILMN_1773407 | 0.00767 | 0.000723 | 3.58 | -0.75284 | 0.362 |
| PTEN | ILMN_1701134 | 0.0162 | 0.00204 | 3.24 | -1.70899 | 0.362 |
| SEL1L3 | ILMN_1797822 | 0.0219 | 0.00314 | 3.09 | -2.10356 | 0.362 |
| USP32 | ILMN_1804743 | 0.0608 | 0.0134 | 2.56 | -3.40438 | 0.362 |
| DNASE1L1 | ILMN_1681695 | 0.0256 | 0.00394 | 3.01 | -2.30872 | 0.361 |
| SPIRE1 | ILMN_1757845 | 0.0635 | 0.0143 | 2.53 | -3.4625 | 0.361 |
| LAT2 | ILMN_2249720 | 0.00014 | 2.78E-06 | 5.22 | 4.48356 | 0.36 |
| AFF1 | ILMN_2129015 | 0.0056 | 0.000463 | 3.72 | -0.33871 | 0.36 |
| FRAT1 | ILMN_1781416 | 0.0332 | 0.00562 | 2.88 | -2.63033 | 0.36 |
| PHC2 | ILMN_1808047 | 0.0464 | 0.00899 | 2.71 | -3.05153 | 0.36 |
| SERAC1 | ILMN_1812169 | 0.0235 | 0.00348 | 3.05 | -2.19561 | 0.359 |
| TAZ | ILMN_1707791 | 0.0304 | 0.00495 | 2.93 | -2.51536 | 0.359 |
| ITGAM | ILMN_1685009 | 0.0318 | 0.00529 | 2.9 | -2.57578 | 0.358 |
| CD14 | ILMN_2396444 | 0.112 | 0.0324 | 2.19 | -4.17587 | 0.358 |
| PTEN | ILMN_1880406 | 0.00628 | 0.000535 | 3.68 | -0.47325 | 0.357 |
| EXTL3 | ILMN_1778226 | 0.00862 | 0.000852 | 3.53 | -0.90497 | 0.357 |
| TGIF1 | ILMN_1702211 | 0.0516 | 0.0106 | 2.65 | -3.19454 | 0.357 |
| FPR1 | ILMN_2092118 | 0.000239 | 6.14E-06 | 5 | 3.73103 | 0.356 |
| SLC11A1 | ILMN_1741165 | 0.00227 | 0.000135 | 4.1 | 0.80939 | 0.355 |
| TES | ILMN_1746243 | 0.0409 | 0.00754 | 2.77 | -2.89394 | 0.355 |
| PRPSAP2 | ILMN_1812445 | 0.0209 | 0.00294 | 3.11 | -2.04394 | 0.354 |
| RPL32 | ILMN_1782167 | 0.0244 | 0.00368 | 3.03 | -2.24759 | 0.354 |
| PPP1R3B | ILMN_2131103 | 0.054 | 0.0113 | 2.62 | -3.25521 | 0.354 |
| STK16 | ILMN_1742520 | 0.0588 | 0.0127 | 2.57 | -3.36129 | 0.354 |
| NUCB1 | ILMN_1722634 | 0.0651 | 0.0148 | 2.51 | -3.49604 | 0.354 |
| HIPK3 | ILMN_1746941 | 0.112 | 0.0326 | 2.19 | -4.18062 | 0.354 |
| ZNF626 | ILMN_2290732 | 0.0432 | 0.00814 | 2.75 | -2.96258 | 0.353 |
| IL7R | ILMN_1691341 | 0.000322 | 9.52E-06 | 4.88 | 3.31468 | 0.352 |
| PYGL | ILMN_1696187 | 0.0228 | 0.00334 | 3.07 | -2.15749 | 0.352 |
| MCM6 | ILMN_1798654 | 0.0315 | 0.00522 | 2.91 | -2.56374 | 0.352 |
| MS4A6A | ILMN_2359800 | 0.0548 | 0.0116 | 2.61 | -3.27479 | 0.352 |
| TCAF2 | ILMN_1740818 | 0.0959 | 0.0258 | 2.29 | -3.98059 | 0.352 |
| 8-Mar | ILMN_2336335 | 0.0024 | 0.000147 | 4.08 | 0.73471 | 0.351 |
| RMND5A | ILMN_1714093 | 0.0158 | 0.00196 | 3.25 | -1.67293 | 0.351 |
| C9orf72 | ILMN_1666742 | 0.0319 | 0.00531 | 2.9 | -2.57963 | 0.351 |
| ADIPOR1 | ILMN_2096322 | 2.77E-06 | 1.25E-08 | 6.67 | 9.6415 | 0.35 |
| ST3GAL1 | ILMN_1860638 | 0.0411 | 0.00761 | 2.77 | -2.90197 | 0.35 |
| SYAP1 | ILMN_2089175 | 0.00403 | 0.000291 | 3.87 | 0.09391 | 0.349 |
| MAN2A2 | ILMN_1815148 | 0.0092 | 0.000938 | 3.5 | -0.99404 | 0.349 |
| PSMA1 | ILMN_1760542 | 0.0105 | 0.00111 | 3.44 | -1.15319 | 0.349 |
| PGS1 | ILMN_2075051 | 0.0348 | 0.00602 | 2.86 | -2.69266 | 0.349 |
| C9orf72 | ILMN_2295252 | 0.0612 | 0.0135 | 2.55 | -3.4117 | 0.349 |
| CR1 | ILMN_1767193 | 0.0974 | 0.0265 | 2.28 | -4.00349 | 0.349 |
| KEAP1 | ILMN_2410772 | 0.108 | 0.0306 | 2.22 | -4.1274 | 0.349 |
| LONRF3 | ILMN_1720452 | 0.147 | 0.0486 | 2.02 | -4.51947 | 0.349 |
| PKNOX1 | ILMN_1745365 | 0.0132 | 0.00153 | 3.33 | -1.4431 | 0.347 |
| ATP1A1 | ILMN_1775566 | 0.0784 | 0.0193 | 2.41 | -3.72612 | 0.347 |
| LILRA5 | ILMN_2266595 | 0.129 | 0.0403 | 2.1 | -4.36112 | 0.347 |
| ATP1B3 | ILMN_1654322 | 0.00462 | 0.000354 | 3.81 | -0.0904 | 0.346 |
| TUBA1B | ILMN_1800261 | 0.00626 | 0.000533 | 3.68 | -0.46931 | 0.346 |
| ZNF600 | ILMN_1678457 | 0.0185 | 0.00247 | 3.17 | -1.88255 | 0.346 |
| DKFZP586I1420 | ILMN_1803856 | 0.0481 | 0.0095 | 2.69 | -3.10107 | 0.346 |
| GYPC | ILMN_1668039 | 0.103 | 0.0284 | 2.25 | -4.06367 | 0.346 |
| CLK1 | ILMN_1652790 | 0.00493 | 0.00039 | 3.78 | -0.17945 | 0.345 |
| C9orf106 | ILMN_1777477 | 0.0399 | 0.00727 | 2.79 | -2.86197 | 0.344 |
| TMLHE | ILMN_1677228 | 0.0403 | 0.00738 | 2.78 | -2.87471 | 0.344 |
| EVI2B | ILMN_2042651 | 0.0574 | 0.0123 | 2.59 | -3.33124 | 0.344 |
| PDCD4-AS1 | ILMN_3199489 | 0.000939 | 4.15E-05 | 4.45 | 1.92101 | 0.343 |
| ST6GALNAC6 | ILMN_1691736 | 0.0367 | 0.00649 | 2.83 | -2.76009 | 0.343 |
| TMEM70 | ILMN_1739032 | 0.0416 | 0.00774 | 2.76 | -2.91738 | 0.343 |
| AP1AR | ILMN_2207561 | 0.0831 | 0.021 | 2.38 | -3.80168 | 0.343 |
| EPS15L1 | ILMN_1708369 | 0.134 | 0.0426 | 2.08 | -4.40944 | 0.343 |
| RPRD1A | ILMN_1764207 | 0.00205 | 0.000118 | 4.14 | 0.93635 | 0.342 |
| BCL6 | ILMN_1737314 | 0.0089 | 0.000892 | 3.51 | -0.94701 | 0.342 |
| RASSF2 | ILMN_1812139 | 0.0183 | 0.00242 | 3.18 | -1.86359 | 0.342 |
| FAM134B | ILMN_2387952 | 0.0429 | 0.00804 | 2.75 | -2.95226 | 0.342 |
| LY6G5C | ILMN_1763467 | 0.116 | 0.0346 | 2.17 | -4.23099 | 0.342 |
| PRMT5 | ILMN_1811955 | 0.129 | 0.04 | 2.1 | -4.35573 | 0.342 |
| ITGAX | ILMN_2254635 | 0.00102 | 4.60E-05 | 4.42 | 1.8247 | 0.341 |
| SLC35E3 | ILMN_1749521 | 0.0213 | 0.00302 | 3.1 | -2.06667 | 0.341 |
| RNF175 | ILMN_1741281 | 0.0253 | 0.00387 | 3.02 | -2.29309 | 0.341 |
| TTC9 | ILMN_3243117 | 0.0313 | 0.00515 | 2.91 | -2.55142 | 0.341 |
| ASPH | ILMN_1693771 | 0.127 | 0.0391 | 2.11 | -4.33601 | 0.341 |
| LAIR1 | ILMN_1768598 | 0.142 | 0.0458 | 2.04 | -4.47052 | 0.341 |
| HTATIP2 | ILMN_1664303 | 0.00225 | 0.000133 | 4.11 | 0.82408 | 0.339 |
| ASPRV1 | ILMN_1762284 | 0.014 | 0.00166 | 3.31 | -1.51818 | 0.339 |
| FRAT1 | ILMN_1736180 | 0.0153 | 0.00189 | 3.26 | -1.63913 | 0.339 |
| INTS6-AS1 | ILMN_1905075 | 0.019 | 0.00257 | 3.16 | -1.92024 | 0.339 |
| OSBPL5 | ILMN_2307032 | 0.0352 | 0.00612 | 2.85 | -2.70714 | 0.339 |
| PPARG | ILMN_1800225 | 0.0811 | 0.0203 | 2.39 | -3.77124 | 0.339 |
| ANKRD28 | ILMN_1697594 | 0.0878 | 0.0229 | 2.34 | -3.87564 | 0.338 |
| ITK | ILMN_1699160 | 0.0053 | 0.000429 | 3.75 | -0.26821 | 0.337 |
| ZAK | ILMN_1768110 | 0.0472 | 0.00923 | 2.7 | -3.07552 | 0.337 |
| STAT5B | ILMN_1684034 | 0.0237 | 0.00352 | 3.05 | -2.2074 | 0.336 |
| FBXO30 | ILMN_2168992 | 0.0248 | 0.00376 | 3.03 | -2.26636 | 0.336 |
| FAM179A | ILMN_3247182 | 0.0765 | 0.0187 | 2.42 | -3.69849 | 0.336 |
| SNHG7 | ILMN_3227023 | 0.101 | 0.0279 | 2.26 | -4.04648 | 0.336 |
| BCAP31 | ILMN_1812403 | 0.000239 | 6.08E-06 | 5 | 3.73976 | 0.335 |
| PPP1CB | ILMN_2405018 | 0.00488 | 0.000385 | 3.78 | -0.16628 | 0.335 |
| RNF149 | ILMN_2112524 | 0.00595 | 0.000499 | 3.7 | -0.40918 | 0.335 |
| FAR2 | ILMN_1694596 | 0.0972 | 0.0264 | 2.28 | -4.00065 | 0.335 |
| SOD1 | ILMN_1662438 | 0.000208 | 5.12E-06 | 5.05 | 3.90249 | 0.334 |
| CDS2 | ILMN_1790973 | 0.0178 | 0.00232 | 3.19 | -1.8281 | 0.334 |
| SERINC3 | ILMN_1713752 | 0.0191 | 0.00259 | 3.16 | -1.92551 | 0.334 |
| LCOR | ILMN_2062381 | 0.00479 | 0.000374 | 3.79 | -0.13917 | 0.333 |
| NEDD1 | ILMN_2206151 | 0.00718 | 0.000662 | 3.61 | -0.67102 | 0.333 |
| ARHGAP19 | ILMN_1688953 | 0.0146 | 0.00176 | 3.29 | -1.57405 | 0.333 |
| CXCR2 | ILMN_1680397 | 0.0182 | 0.00239 | 3.18 | -1.85446 | 0.333 |
| RAB36 | ILMN_1733045 | 0.0412 | 0.00763 | 2.77 | -2.90468 | 0.333 |
| ZBTB43 | ILMN_1731113 | 0.0647 | 0.0147 | 2.52 | -3.48801 | 0.333 |
| JMY | ILMN_1762080 | 0.127 | 0.0391 | 2.11 | -4.33617 | 0.333 |
| LMO2 | ILMN_1800078 | 0.0188 | 0.00253 | 3.16 | -1.90479 | 0.332 |
| NEDD9 | ILMN_1743619 | 0.0507 | 0.0103 | 2.66 | -3.17566 | 0.332 |
| PLEC | ILMN_1736792 | 0.0592 | 0.0129 | 2.57 | -3.37264 | 0.332 |
| GGH | ILMN_1681754 | 0.0796 | 0.0198 | 2.4 | -3.7482 | 0.332 |
| RAB43 | ILMN_2155480 | 0.0525 | 0.0109 | 2.64 | -3.2192 | 0.331 |
| ZNF493 | ILMN_2278653 | 0.0558 | 0.0119 | 2.6 | -3.29802 | 0.331 |
| SCML1 | ILMN_2340908 | 0.0762 | 0.0186 | 2.43 | -3.69296 | 0.331 |
| SIRPB1 | ILMN_1733997 | 0.00644 | 0.000564 | 3.66 | -0.52171 | 0.33 |
| RAX2 | ILMN_1653412 | 0.0345 | 0.00596 | 2.86 | -2.68302 | 0.33 |
| GPX8 | ILMN_3242038 | 0.0361 | 0.00634 | 2.84 | -2.73869 | 0.329 |
| ZMYM5 | ILMN_1781028 | 0.00615 | 0.000521 | 3.69 | -0.44835 | 0.328 |
| MAML2 | ILMN_1765729 | 0.0192 | 0.0026 | 3.15 | -1.93198 | 0.328 |
| NUP153 | ILMN_1705907 | 0.000162 | 3.44E-06 | 5.16 | 4.27981 | 0.327 |
| SLC22A15 | ILMN_1730639 | 0.0901 | 0.0237 | 2.33 | -3.90561 | 0.327 |
| JMJD1C | ILMN_1764970 | 0.000984 | 4.40E-05 | 4.44 | 1.86585 | 0.326 |
| TMEM159 | ILMN_1655876 | 0.0089 | 0.000893 | 3.51 | -0.94794 | 0.326 |
| ERLIN2 | ILMN_1700549 | 0.0306 | 0.00499 | 2.92 | -2.52227 | 0.326 |
| PCM1 | ILMN_2042595 | 0.00131 | 6.43E-05 | 4.32 | 1.5096 | 0.325 |
| SNORD12B | ILMN_3240002 | 0.00895 | 9.00E-04 | 3.51 | -0.95524 | 0.324 |
| PFKFB2 | ILMN_2385298 | 0.0731 | 0.0175 | 2.45 | -3.64017 | 0.324 |
| SDSL | ILMN_1750674 | 0.0848 | 0.0217 | 2.36 | -3.82767 | 0.324 |
| MXRA7 | ILMN_1743836 | 0.0374 | 0.00667 | 2.82 | -2.78448 | 0.323 |
| TSPAN14///NUP58 | ILMN_1873620 | 0.0506 | 0.0103 | 2.66 | -3.17139 | 0.323 |
| MERTK | ILMN_1770610 | 0.0516 | 0.0105 | 2.65 | -3.1941 | 0.323 |
| TMEM234 | ILMN_1673752 | 0.00592 | 0.000496 | 3.7 | -0.40263 | 0.322 |
| SGK3 | ILMN_2366703 | 0.00862 | 0.000854 | 3.53 | -0.90677 | 0.322 |
| TUBA1A | ILMN_2148819 | 0.0213 | 0.00302 | 3.1 | -2.0661 | 0.322 |
| TMEM62 | ILMN_1745807 | 0.128 | 0.0395 | 2.11 | -4.34569 | 0.322 |
| TP53I13 | ILMN_1666178 | 0.000182 | 4.14E-06 | 5.11 | 4.10528 | 0.321 |
| PCM1 | ILMN_1690487 | 0.00136 | 6.82E-05 | 4.31 | 1.45358 | 0.321 |
| FAM160B1 | ILMN_1752927 | 0.00254 | 0.000158 | 4.05 | 0.66284 | 0.321 |
| GADD45A | ILMN_2052208 | 0.0507 | 0.0103 | 2.66 | -3.17487 | 0.321 |
| ELMOD3 | ILMN_3190834 | 0.113 | 0.0332 | 2.18 | -4.19608 | 0.321 |
| KIAA1551 | ILMN_1726289 | 0.00146 | 7.47E-05 | 4.28 | 1.36821 | 0.32 |
| NATD1 | ILMN_1661735 | 0.0475 | 0.00934 | 2.69 | -3.08547 | 0.32 |
| SERPINA1 | ILMN_2256050 | 0.0567 | 0.0121 | 2.59 | -3.31695 | 0.32 |
| ADAT3 | ILMN_1806275 | 0.0919 | 0.0243 | 2.32 | -3.92838 | 0.32 |
| RNF130 | ILMN_1729294 | 0.000108 | 1.97E-06 | 5.31 | 4.81073 | 0.318 |
| NSFL1C | ILMN_1657624 | 0.00666 | 0.000595 | 3.64 | -0.57284 | 0.318 |
| DIP2B | ILMN_1755589 | 0.00061 | 2.33E-05 | 4.62 | 2.46799 | 0.317 |
| GNS | ILMN_1744517 | 0.00157 | 8.35E-05 | 4.25 | 1.26313 | 0.317 |
| RHOT1 | ILMN_1731484 | 0.0656 | 0.015 | 2.51 | -3.50753 | 0.317 |
| PPP2R3A | ILMN_1656393 | 0.00519 | 0.000418 | 3.75 | -0.24465 | 0.316 |
| OLMALINC | ILMN_3268880 | 0.102 | 0.0283 | 2.25 | -4.05985 | 0.316 |
| CTNNAL1 | ILMN_2136446 | 0.104 | 0.0289 | 2.24 | -4.07708 | 0.316 |
| MSH6 | ILMN_1729051 | 0.0242 | 0.00364 | 3.04 | -2.23799 | 0.315 |
| MKNK1 | ILMN_1725738 | 0.0588 | 0.0127 | 2.57 | -3.36157 | 0.315 |
| DICER1 | ILMN_1772692 | 0.0774 | 0.019 | 2.42 | -3.71345 | 0.315 |
| CLEC2B | ILMN_1784608 | 0.00359 | 0.000249 | 3.92 | 0.24023 | 0.314 |
| RGPD1 | ILMN_2116827 | 0.00488 | 0.000384 | 3.78 | -0.16373 | 0.314 |
| C9orf72 | ILMN_1741881 | 0.0205 | 0.00286 | 3.12 | -2.01844 | 0.314 |
| ZNF7 | ILMN_2137066 | 0.0645 | 0.0146 | 2.52 | -3.48247 | 0.314 |
| ZC3H3 | ILMN_1702389 | 0.0679 | 0.0157 | 2.49 | -3.54705 | 0.314 |
| ARMCX3 | ILMN_2334760 | 0.0847 | 0.0216 | 2.36 | -3.82551 | 0.314 |
| MBTD1 | ILMN_2101650 | 0.0911 | 0.024 | 2.32 | -3.91844 | 0.314 |
| TXNIP | ILMN_1697448 | 9.36E-05 | 1.61E-06 | 5.37 | 5.00115 | 0.313 |
| C9orf64 | ILMN_1777318 | 0.00958 | 0.000992 | 3.48 | -1.04492 | 0.313 |
| MAGED4B | ILMN_2316278 | 0.0158 | 0.00198 | 3.25 | -1.68016 | 0.313 |
| SMCHD1 | ILMN_1808148 | 0.025 | 0.00381 | 3.02 | -2.27951 | 0.313 |
| TSEN34 | ILMN_2368292 | 0.0333 | 0.00565 | 2.88 | -2.63434 | 0.313 |
| PACSIN2 | ILMN_3307772 | 0.0492 | 0.00992 | 2.67 | -3.13949 | 0.313 |
| TRIM27 | ILMN_1655482 | 0.077 | 0.0188 | 2.42 | -3.70615 | 0.313 |
| SKAP2 | ILMN_1657129 | 6.62E-05 | 9.62E-07 | 5.51 | 5.49316 | 0.312 |
| C10orf12 | ILMN_1665508 | 0.0219 | 0.00315 | 3.09 | -2.10447 | 0.312 |
| LRPAP1 | ILMN_1660341 | 0.0229 | 0.00336 | 3.06 | -2.16484 | 0.312 |
| CALHM2 | ILMN_1766200 | 0.0353 | 0.00616 | 2.85 | -2.71219 | 0.312 |
| MX2 | ILMN_2231928 | 0.069 | 0.0161 | 2.48 | -3.56793 | 0.312 |
| KLHL8 | ILMN_1714083 | 0.113 | 0.0333 | 2.18 | -4.19795 | 0.312 |
| TBC1D7 | ILMN_1661622 | 0.134 | 0.0426 | 2.08 | -4.40851 | 0.312 |
| RGPD1 | ILMN_1746428 | 0.0221 | 0.00321 | 3.08 | -2.12164 | 0.311 |
| HRH2 | ILMN_1722411 | 0.138 | 0.0444 | 2.06 | -4.44313 | 0.311 |
| NDEL1 | ILMN_2397199 | 0.0108 | 0.00116 | 3.43 | -1.18788 | 0.31 |
| KIAA0319L | ILMN_1683277 | 0.0156 | 0.00194 | 3.25 | -1.66332 | 0.31 |
| METTL23 | ILMN_1688727 | 0.0277 | 0.00438 | 2.97 | -2.40539 | 0.31 |
| FKBP11 | ILMN_1787345 | 0.0479 | 0.00943 | 2.69 | -3.09376 | 0.31 |
| FLCN | ILMN_1655525 | 0.0635 | 0.0143 | 2.53 | -3.4608 | 0.31 |
| CD68 | ILMN_1714861 | 0.125 | 0.0382 | 2.12 | -4.31587 | 0.31 |
| AP3D1 | ILMN_1764945 | 0.0131 | 0.00151 | 3.34 | -1.42987 | 0.309 |
| BIN1 | ILMN_2309245 | 0.0145 | 0.00174 | 3.29 | -1.56297 | 0.309 |
| IQSEC1 | ILMN_1808299 | 0.0169 | 0.00217 | 3.22 | -1.76603 | 0.309 |
| USP15 | ILMN_1665557 | 0.00111 | 5.18E-05 | 4.39 | 1.71335 | 0.308 |
| FLVCR2 | ILMN_2204876 | 0.0164 | 0.00207 | 3.23 | -1.72249 | 0.308 |
| ADIPOQ | ILMN_1775045 | 0.02 | 0.00275 | 3.13 | -1.98279 | 0.307 |
| SNORA17A | ILMN_3243129 | 0.0592 | 0.0129 | 2.57 | -3.37423 | 0.307 |
| AGFG1 | ILMN_3260345 | 0.12 | 0.0361 | 2.15 | -4.2674 | 0.307 |
| APOC1 | ILMN_1789007 | 0.135 | 0.0429 | 2.07 | -4.41583 | 0.307 |
| DYNLT1 | ILMN_1678766 | 0.0128 | 0.00145 | 3.35 | -1.39735 | 0.306 |
| ANKRD6 | ILMN_2178587 | 0.0239 | 0.00357 | 3.04 | -2.21871 | 0.306 |
| AHNAK | ILMN_1714567 | 0.0431 | 0.00812 | 2.75 | -2.96025 | 0.306 |
| CSNK1D | ILMN_1720708 | 0.000579 | 2.17E-05 | 4.64 | 2.53434 | 0.305 |
| HIST2H3D | ILMN_1664706 | 0.0441 | 0.00837 | 2.73 | -2.98814 | 0.305 |
| MVP | ILMN_1803277 | 0.0764 | 0.0186 | 2.42 | -3.69672 | 0.305 |
| MRPL57 | ILMN_2203807 | 0.083 | 0.021 | 2.38 | -3.79985 | 0.305 |
| ATP1A1 | ILMN_1731783 | 0.0971 | 0.0264 | 2.28 | -3.99838 | 0.305 |
| ENHO | ILMN_3247320 | 0.0272 | 0.00427 | 2.98 | -2.38122 | 0.304 |
| LY96 | ILMN_1724533 | 0.0272 | 0.00427 | 2.98 | -2.38187 | 0.304 |
| PTPN1 | ILMN_1681591 | 0.0432 | 0.00813 | 2.75 | -2.96191 | 0.304 |
| LINC00092 | ILMN_3249172 | 0.0852 | 0.0218 | 2.36 | -3.83389 | 0.304 |
| FAM172A | ILMN_3241099 | 0.145 | 0.0477 | 2.02 | -4.5045 | 0.303 |
| FAM160B1 | ILMN_3231638 | 0.00344 | 0.000235 | 3.93 | 0.29462 | 0.302 |
| MGAT4A | ILMN_1755643 | 0.00403 | 0.000292 | 3.87 | 0.09042 | 0.302 |
| IPP | ILMN_1789106 | 0.0193 | 0.00262 | 3.15 | -1.93689 | 0.302 |
| ATL1 | ILMN_3181411 | 0.0489 | 0.00978 | 2.68 | -3.12669 | 0.302 |
| LAIR2 | ILMN_1807491 | 0.118 | 0.0353 | 2.16 | -4.2489 | 0.302 |
| ZNF684 | ILMN_1751393 | 0.14 | 0.0452 | 2.05 | -4.45941 | 0.302 |
| RB1CC1 | ILMN_1736796 | 0.00845 | 0.000828 | 3.54 | -0.87841 | 0.301 |
| ZNF646 | ILMN_1675479 | 0.00862 | 0.000855 | 3.53 | -0.9081 | 0.301 |
| WDR74 | ILMN_2270845 | 0.0211 | 0.00299 | 3.11 | -2.05799 | 0.301 |
| ADGRE5 | ILMN_2413508 | 0.0576 | 0.0124 | 2.59 | -3.33738 | 0.301 |
| FAH | ILMN_1781536 | 0.0814 | 0.0204 | 2.39 | -3.77594 | 0.301 |
| TSC22D3 | ILMN_1695382 | 0.0964 | 0.026 | 2.29 | -3.98766 | 0.301 |
| TNFRSF10B | ILMN_1812915 | 0.119 | 0.0358 | 2.15 | -4.26065 | 0.301 |
| ZNFX1 | ILMN_1745148 | 0.00149 | 7.69E-05 | 4.27 | 1.34056 | 0.3 |
| PRR21 | ILMN_1704043 | 0.011 | 0.00118 | 3.42 | -1.20896 | 0.3 |
| ADAMTSL4 | ILMN_1687035 | 0.0229 | 0.00336 | 3.07 | -2.16398 | 0.3 |
| ODF3B | ILMN_3241446 | 0.0247 | 0.00374 | 3.03 | -2.26178 | 0.3 |
| FAM189A1 | ILMN_1886092 | 0.0391 | 0.00709 | 2.8 | -2.83869 | 0.3 |
| RAB3IP | ILMN_2291619 | 0.0959 | 0.0258 | 2.29 | -3.98078 | 0.3 |
| ECM1 | ILMN_2329735 | 0.121 | 0.0363 | 2.15 | -4.27271 | 0.3 |
| WWP2 | ILMN_1659703 | 0.000102 | 1.82E-06 | 5.34 | 4.88584 | 0.299 |
| ERLIN2 | ILMN_2378664 | 0.00886 | 0.000886 | 3.51 | -0.94126 | 0.299 |
| DDAH2 | ILMN_1770787 | 0.0622 | 0.0138 | 2.54 | -3.43408 | 0.299 |
| SLC22A4 | ILMN_1685057 | 0.00962 | 0.000998 | 3.48 | -1.05136 | 0.298 |
| EPB41L3 | ILMN_2109197 | 0.0151 | 0.00185 | 3.27 | -1.62032 | 0.298 |
| HOXA6 | ILMN_1815570 | 0.0484 | 0.00963 | 2.68 | -3.11278 | 0.298 |
| TMEM191A | ILMN_3228529 | 0.0523 | 0.0108 | 2.64 | -3.21606 | 0.298 |
| TMEM70 | ILMN_2373010 | 0.0978 | 0.0267 | 2.28 | -4.0092 | 0.298 |
| RHBDD2 | ILMN_1809437 | 0.0171 | 0.00222 | 3.21 | -1.78447 | 0.297 |
| METTL23 | ILMN_2280731 | 0.0406 | 0.00747 | 2.78 | -2.88558 | 0.297 |
| ZNF75A | ILMN_1666552 | 0.0535 | 0.0112 | 2.62 | -3.24589 | 0.297 |
| ZNF839 | ILMN_1777487 | 0.0685 | 0.0159 | 2.49 | -3.55844 | 0.297 |
| SLC5A9 | ILMN_1748366 | 0.111 | 0.032 | 2.2 | -4.16638 | 0.297 |
| MAP4K5 | ILMN_1759030 | 0.0214 | 0.00304 | 3.1 | -2.07362 | 0.296 |
| ATP6AP1 | ILMN_1697694 | 0.0314 | 0.00518 | 2.91 | -2.55603 | 0.295 |
| TALDO1 | ILMN_1670998 | 0.0759 | 0.0184 | 2.43 | -3.68749 | 0.295 |
| TOP1P1 | ILMN_2086952 | 0.0874 | 0.0227 | 2.34 | -3.86781 | 0.295 |
| ALPL | ILMN_1701603 | 0.105 | 0.0297 | 2.23 | -4.10039 | 0.295 |
| MALSU1 | ILMN_1739798 | 0.106 | 0.0301 | 2.23 | -4.11383 | 0.295 |
| G6PD | ILMN_1697559 | 0.00952 | 0.000984 | 3.48 | -1.03767 | 0.294 |
| ZMPSTE24 | ILMN_1656413 | 0.054 | 0.0113 | 2.62 | -3.25626 | 0.294 |
| HIST2H2BE | ILMN_1732071 | 0.0549 | 0.0116 | 2.61 | -3.2782 | 0.294 |
| TPP2 | ILMN_1667657 | 0.104 | 0.0291 | 2.24 | -4.08353 | 0.294 |
| LAIR2 | ILMN_2323933 | 0.145 | 0.0476 | 2.03 | -4.50174 | 0.294 |
| FCER1G | ILMN_2123743 | 0.00918 | 0.000934 | 3.5 | -0.98986 | 0.293 |
| C17orf62 | ILMN_1750401 | 0.0687 | 0.016 | 2.49 | -3.5621 | 0.293 |
| MSL3 | ILMN_3232573 | 0.129 | 0.0401 | 2.1 | -4.35853 | 0.293 |
| UBXN2B | ILMN_3236556 | 0.0145 | 0.00174 | 3.29 | -1.56458 | 0.292 |
| FAM134A | ILMN_3251572 | 0.0656 | 0.015 | 2.51 | -3.50724 | 0.292 |
| LOC102724002 | ILMN_1839719 | 0.0175 | 0.00228 | 3.2 | -1.80969 | 0.291 |
| ZNF33A | ILMN_2352590 | 0.0321 | 0.00535 | 2.9 | -2.586 | 0.291 |
| SNORD3A | ILMN_3239574 | 0.0392 | 0.0071 | 2.8 | -2.84078 | 0.291 |
| FBXO32 | ILMN_1703955 | 0.064 | 0.0145 | 2.52 | -3.47364 | 0.291 |
| GPRASP1 | ILMN_1787627 | 0.0675 | 0.0156 | 2.5 | -3.53852 | 0.291 |
| FAR2 | ILMN_2202915 | 0.105 | 0.0295 | 2.23 | -4.0965 | 0.291 |
| PQLC1 | ILMN_1798620 | 0.0147 | 0.00177 | 3.29 | -1.57768 | 0.29 |
| REPS2 | ILMN_3250143 | 0.0908 | 0.0239 | 2.32 | -3.91373 | 0.29 |
| CAPRIN2 | ILMN_1681118 | 0.0413 | 0.00764 | 2.77 | -2.90637 | 0.289 |
| SOS2 | ILMN_1764414 | 0.0761 | 0.0185 | 2.43 | -3.69088 | 0.289 |
| SIGLEC6 | ILMN_1685630 | 0.106 | 0.0301 | 2.23 | -4.11122 | 0.289 |
| AFF1 | ILMN_1673119 | 0.129 | 0.04 | 2.1 | -4.35642 | 0.289 |
| CLEC12A | ILMN_2403228 | 0.077 | 0.0189 | 2.42 | -3.70669 | 0.288 |
| RIMBP3 | ILMN_1732988 | 0.111 | 0.0321 | 2.2 | -4.16702 | 0.288 |
| ARFGAP3 | ILMN_1731287 | 0.127 | 0.039 | 2.11 | -4.33418 | 0.288 |
| CYTIP | ILMN_1746864 | 0.00312 | 0.000209 | 3.97 | 0.40241 | 0.287 |
| CEP41 | ILMN_1797209 | 0.00898 | 0.000904 | 3.51 | -0.95911 | 0.287 |
| PACSIN2 | ILMN_1702396 | 0.0209 | 0.00293 | 3.11 | -2.04091 | 0.287 |
| FAM27C | ILMN_3293843 | 0.0352 | 0.00612 | 2.85 | -2.70628 | 0.287 |
| PLIN3 | ILMN_1660021 | 0.0598 | 0.0131 | 2.56 | -3.38517 | 0.287 |
| KIF1BP | ILMN_1745813 | 0.0803 | 0.02 | 2.39 | -3.75963 | 0.287 |
| GTPBP1 | ILMN_1811378 | 0.0868 | 0.0224 | 2.35 | -3.85665 | 0.287 |
| PPP1R35 | ILMN_1741475 | 0.00962 | 0.001 | 3.48 | -1.05245 | 0.286 |
| DHX34 | ILMN_1747506 | 0.0171 | 0.00221 | 3.21 | -1.78071 | 0.286 |
| SNORD3D | ILMN_3242315 | 0.0414 | 0.00768 | 2.77 | -2.91084 | 0.286 |
| TREML2 | ILMN_1740864 | 0.097 | 0.0263 | 2.28 | -3.99647 | 0.286 |
| UQCRH | ILMN_1792138 | 0.113 | 0.0331 | 2.19 | -4.19459 | 0.286 |
| KATNBL1 | ILMN_1739854 | 0.019 | 0.00257 | 3.16 | -1.91984 | 0.285 |
| TDP2 | ILMN_1785821 | 0.0277 | 0.00438 | 2.97 | -2.40418 | 0.285 |
| FGGY | ILMN_1737604 | 0.0215 | 0.00307 | 3.1 | -2.08191 | 0.284 |
| CAPNS1 | ILMN_2393254 | 0.0327 | 0.00552 | 2.89 | -2.61308 | 0.284 |
| ST3GAL1 | ILMN_2267135 | 0.121 | 0.0364 | 2.14 | -4.275 | 0.284 |
| ARHGEF11 | ILMN_2347380 | 0.128 | 0.0396 | 2.11 | -4.34807 | 0.284 |
| C21orf33 | ILMN_1737588 | 0.0107 | 0.00114 | 3.43 | -1.17657 | 0.283 |
| IGF2BP2 | ILMN_1702447 | 0.0282 | 0.00448 | 2.96 | -2.42492 | 0.282 |
| SLC10A3 | ILMN_1712887 | 0.0338 | 0.00577 | 2.87 | -2.65378 | 0.282 |
| DIP2B | ILMN_2180352 | 0.00102 | 4.64E-05 | 4.42 | 1.81683 | 0.281 |
| EVI2A | ILMN_2369018 | 0.00158 | 8.39E-05 | 4.25 | 1.25938 | 0.281 |
| RNF103 | ILMN_1692199 | 0.0526 | 0.0109 | 2.63 | -3.2226 | 0.281 |
| EVI2A | ILMN_1662747 | 0.0579 | 0.0125 | 2.58 | -3.343 | 0.281 |
| ATP1B1 | ILMN_1730291 | 0.0625 | 0.0139 | 2.54 | -3.44148 | 0.281 |
| CD44 | ILMN_1803429 | 0.129 | 0.0401 | 2.1 | -4.35869 | 0.281 |
| NAGLU | ILMN_1694980 | 0.141 | 0.0454 | 2.05 | -4.46354 | 0.28 |
| UNKL | ILMN_2126408 | 0.018 | 0.00236 | 3.19 | -1.84024 | 0.279 |
| PRF1 | ILMN_1740633 | 0.0313 | 0.00514 | 2.91 | -2.55018 | 0.279 |
| METRNL | ILMN_2258004 | 0.0326 | 0.00548 | 2.89 | -2.60786 | 0.279 |
| DNAJB5 | ILMN_1663722 | 0.13 | 0.0405 | 2.1 | -4.36559 | 0.279 |
| LATS2 | ILMN_1703412 | 0.014 | 0.00167 | 3.31 | -1.5228 | 0.278 |
| IL1RL1 | ILMN_1697444 | 0.0599 | 0.0131 | 2.56 | -3.3885 | 0.278 |
| DUSP10 | ILMN_1759175 | 0.125 | 0.0381 | 2.12 | -4.31475 | 0.278 |
| RNASE1 | ILMN_1795183 | 0.136 | 0.0434 | 2.07 | -4.42459 | 0.278 |
| GLB1L | ILMN_2217513 | 0.0236 | 0.00351 | 3.05 | -2.20396 | 0.277 |
| CSGALNACT2 | ILMN_1799208 | 0.0785 | 0.0193 | 2.41 | -3.72898 | 0.277 |
| FOXD4L4 | ILMN_3238576 | 0.097 | 0.0263 | 2.28 | -3.99757 | 0.277 |
| RNF24 | ILMN_1717809 | 0.101 | 0.0278 | 2.26 | -4.04386 | 0.277 |
| MAP1LC3B | ILMN_1703244 | 0.00208 | 0.00012 | 4.14 | 0.92007 | 0.276 |
| MIR1185-1 | ILMN_3309164 | 0.0124 | 0.0014 | 3.36 | -1.36005 | 0.276 |
| TCTA | ILMN_1700001 | 0.0753 | 0.0183 | 2.43 | -3.67805 | 0.276 |
| YOD1 | ILMN_1678919 | 0.0863 | 0.0223 | 2.35 | -3.85106 | 0.276 |
| FAM133CP | ILMN_3304435 | 0.123 | 0.0375 | 2.13 | -4.30001 | 0.276 |
| MYD88 | ILMN_1738523 | 0.000153 | 3.16E-06 | 5.18 | 4.36232 | 0.275 |
| LPP | ILMN_1651254 | 0.022 | 0.00317 | 3.08 | -2.11212 | 0.275 |
| EXT1 | ILMN_2129927 | 0.114 | 0.0336 | 2.18 | -4.20594 | 0.275 |
| WASF2 | ILMN_1684051 | 0.0107 | 0.00115 | 3.43 | -1.18127 | 0.274 |
| HNRNPA3 | ILMN_1761083 | 0.0119 | 0.00133 | 3.38 | -1.31325 | 0.274 |
| RTCA | ILMN_2059294 | 0.0329 | 0.00555 | 2.89 | -2.61902 | 0.274 |
| BORCS7 | ILMN_2151056 | 0.0405 | 0.00743 | 2.78 | -2.88107 | 0.274 |
| KCNG2 | ILMN_1735062 | 0.0641 | 0.0145 | 2.52 | -3.47563 | 0.274 |
| MAFF | ILMN_2322375 | 0.0795 | 0.0197 | 2.4 | -3.74534 | 0.274 |
| VPS8 | ILMN_2415170 | 0.00601 | 0.000506 | 3.69 | -0.42188 | 0.273 |
| GRSF1 | ILMN_1806601 | 0.018 | 0.00236 | 3.19 | -1.84203 | 0.273 |
| LYPLA1 | ILMN_1666713 | 0.0598 | 0.0131 | 2.56 | -3.38462 | 0.273 |
| CHCHD7 | ILMN_1744138 | 0.0972 | 0.0264 | 2.28 | -3.99973 | 0.273 |
| SLC9A4 | ILMN_2112730 | 0.135 | 0.0431 | 2.07 | -4.41785 | 0.273 |
| MACF1 | ILMN_2301624 | 0.137 | 0.0437 | 2.06 | -4.43045 | 0.273 |
| OGFOD3 | ILMN_1811991 | 0.0187 | 0.00249 | 3.17 | -1.89095 | 0.272 |
| RNF146 | ILMN_1685679 | 0.0337 | 0.00575 | 2.87 | -2.64988 | 0.272 |
| CELF2 | ILMN_2400947 | 0.0832 | 0.0211 | 2.37 | -3.80285 | 0.272 |
| TSEN34 | ILMN_1673111 | 0.108 | 0.0308 | 2.22 | -4.1332 | 0.272 |
| COG5 | ILMN_1721535 | 0.112 | 0.0323 | 2.2 | -4.17372 | 0.272 |
| GNPTG | ILMN_1764230 | 0.0315 | 0.00521 | 2.91 | -2.56143 | 0.271 |
| CYB561 | ILMN_1679721 | 0.041 | 0.00757 | 2.77 | -2.89791 | 0.271 |
| MVP | ILMN_2344373 | 0.0613 | 0.0135 | 2.55 | -3.41323 | 0.271 |
| MANSC1 | ILMN_1652490 | 0.105 | 0.0293 | 2.24 | -4.08961 | 0.271 |
| MOSPD1 | ILMN_1727798 | 0.111 | 0.0322 | 2.2 | -4.17089 | 0.271 |
| C19orf35 | ILMN_1674926 | 0.0686 | 0.016 | 2.49 | -3.56003 | 0.27 |
| RAB33B | ILMN_1727738 | 0.0879 | 0.0229 | 2.34 | -3.87685 | 0.27 |
| LRRC26 | ILMN_1680757 | 0.0998 | 0.0274 | 2.27 | -4.03265 | 0.27 |
| IFRD1 | ILMN_1667561 | 0.0389 | 0.00702 | 2.8 | -2.83063 | 0.269 |
| MMP23A | ILMN_2317701 | 0.0955 | 0.0257 | 2.29 | -3.9753 | 0.269 |
| MRRF | ILMN_2415949 | 0.112 | 0.0328 | 2.19 | -4.18564 | 0.269 |
| TP53INP2 | ILMN_1686906 | 0.0264 | 0.00408 | 3 | -2.34149 | 0.268 |
| SLAMF6 | ILMN_2196078 | 0.0445 | 0.00848 | 2.73 | -2.99977 | 0.268 |
| KRT75 | ILMN_1721247 | 0.0537 | 0.0112 | 2.62 | -3.25062 | 0.268 |
| ATG9A | ILMN_2410975 | 0.0561 | 0.0119 | 2.6 | -3.30458 | 0.268 |
| KYAT3 | ILMN_2244484 | 0.072 | 0.0171 | 2.46 | -3.62146 | 0.268 |
| HPS1 | ILMN_1741483 | 0.0883 | 0.0231 | 2.34 | -3.88298 | 0.268 |
| DUSP16 | ILMN_1764361 | 0.0937 | 0.025 | 2.3 | -3.95175 | 0.268 |
| SGK3 | ILMN_1747020 | 0.048 | 0.00948 | 2.69 | -3.09902 | 0.267 |
| FLCN | ILMN_1814952 | 0.075 | 0.0181 | 2.43 | -3.67313 | 0.267 |
| CLN8 | ILMN_1701094 | 0.0766 | 0.0187 | 2.42 | -3.70006 | 0.267 |
| SFPQ | ILMN_1769931 | 0.124 | 0.0377 | 2.13 | -4.30483 | 0.267 |
| ACSL3 | ILMN_1654414 | 0.131 | 0.0412 | 2.09 | -4.38151 | 0.267 |
| HNRNPM | ILMN_1745385 | 0.00634 | 0.000545 | 3.67 | -0.49019 | 0.266 |
| MEFV | ILMN_2115752 | 0.0473 | 0.00927 | 2.7 | -3.07894 | 0.266 |
| LAMP1 | ILMN_1782292 | 0.119 | 0.0356 | 2.15 | -4.25748 | 0.266 |
| PIK3CD | ILMN_1766275 | 0.0275 | 0.00432 | 2.98 | -2.39329 | 0.265 |
| SIRPA | ILMN_2372974 | 0.0836 | 0.0213 | 2.37 | -3.81237 | 0.265 |
| SLC5A8 | ILMN_1811221 | 0.0985 | 0.0269 | 2.27 | -4.01654 | 0.265 |
| KLHL12 | ILMN_1676075 | 0.0184 | 0.00243 | 3.18 | -1.87013 | 0.264 |
| DUSP28 | ILMN_1741780 | 0.0348 | 0.00602 | 2.86 | -2.6927 | 0.264 |
| FBXL2 | ILMN_1688639 | 0.0515 | 0.0105 | 2.65 | -3.19106 | 0.264 |
| ZFYVE16 | ILMN_1651769 | 0.0926 | 0.0246 | 2.31 | -3.93708 | 0.264 |
| ZNF429 | ILMN_1695413 | 0.129 | 0.0399 | 2.1 | -4.35377 | 0.264 |
| ARMCX3 | ILMN_2334765 | 0.129 | 0.0403 | 2.1 | -4.36214 | 0.264 |
| CANT1 | ILMN_1664012 | 0.0131 | 0.00151 | 3.34 | -1.43041 | 0.263 |
| PRKCZ | ILMN_2386982 | 0.0632 | 0.0141 | 2.53 | -3.45415 | 0.263 |
| UNG | ILMN_1683120 | 0.0974 | 0.0266 | 2.28 | -4.00452 | 0.263 |
| SNTB2 | ILMN_1786766 | 0.106 | 0.0301 | 2.23 | -4.11376 | 0.263 |
| GPS1 | ILMN_1769960 | 0.113 | 0.0329 | 2.19 | -4.18834 | 0.263 |
| ZNF407 | ILMN_1741300 | 0.022 | 0.00318 | 3.08 | -2.11424 | 0.262 |
| JARID2-AS1 | ILMN_1856564 | 0.0361 | 0.00633 | 2.84 | -2.7367 | 0.262 |
| USF2 | ILMN_2324672 | 0.098 | 0.0268 | 2.28 | -4.01189 | 0.262 |
| CCDC6 | ILMN_1745904 | 0.103 | 0.0288 | 2.24 | -4.07462 | 0.262 |
| ZNF254 | ILMN_1657729 | 0.132 | 0.0416 | 2.09 | -4.38864 | 0.262 |
| TBCB | ILMN_1790953 | 0.0235 | 0.00347 | 3.05 | -2.19342 | 0.261 |
| ATP11B | ILMN_1658884 | 0.0276 | 0.00435 | 2.97 | -2.39915 | 0.261 |
| RHBDD2 | ILMN_2406410 | 0.04 | 0.0073 | 2.79 | -2.86552 | 0.261 |
| ZEB2 | ILMN_1688698 | 0.0699 | 0.0164 | 2.47 | -3.58497 | 0.261 |
| JADE3 | ILMN_1790518 | 0.0542 | 0.0114 | 2.62 | -3.26201 | 0.26 |
| CD164 | ILMN_1783852 | 0.0618 | 0.0137 | 2.55 | -3.42515 | 0.26 |
| PREX1 | ILMN_1777342 | 0.00448 | 0.000339 | 3.82 | -0.04766 | 0.259 |
| TMX4 | ILMN_1702759 | 0.0371 | 0.00659 | 2.82 | -2.77274 | 0.259 |
| PRR13 | ILMN_1795944 | 0.0477 | 0.0094 | 2.69 | -3.09113 | 0.259 |
| GSDMB | ILMN_2260756 | 0.129 | 0.0399 | 2.1 | -4.35405 | 0.259 |
| TGIF1 | ILMN_2318643 | 0.0615 | 0.0136 | 2.55 | -3.41948 | 0.258 |
| PCGF6 | ILMN_1656574 | 0.0916 | 0.0242 | 2.32 | -3.92421 | 0.258 |
| PRKCZ | ILMN_1697267 | 0.111 | 0.032 | 2.2 | -4.16469 | 0.258 |
| TIAM2 | ILMN_2358560 | 0.00532 | 0.000433 | 3.74 | -0.27573 | 0.257 |
| GMFG | ILMN_1711617 | 0.0017 | 9.22E-05 | 4.22 | 1.17038 | 0.256 |
| S100P | ILMN_1801216 | 0.00303 | 0.000202 | 3.98 | 0.43631 | 0.256 |
| MBP | ILMN_1672660 | 0.00925 | 0.000944 | 3.49 | -0.99957 | 0.256 |
| C5AR2 | ILMN_1669317 | 0.0407 | 0.0075 | 2.78 | -2.88975 | 0.256 |
| CACNG6 | ILMN_1779043 | 0.0787 | 0.0194 | 2.41 | -3.73358 | 0.256 |
| LOC401357 | ILMN_2072598 | 0.0814 | 0.0204 | 2.39 | -3.77618 | 0.256 |
| DCUN1D3 | ILMN_1665455 | 0.109 | 0.0312 | 2.21 | -4.14379 | 0.256 |
| RAP1GDS1 | ILMN_1806266 | 0.114 | 0.0337 | 2.18 | -4.20832 | 0.256 |
| NAB2 | ILMN_1721922 | 0.149 | 0.0496 | 2.01 | -4.53774 | 0.256 |
| SYTL2 | ILMN_2336609 | 0.0362 | 0.00638 | 2.84 | -2.74447 | 0.255 |
| SNRPD1 | ILMN_1768393 | 0.0964 | 0.026 | 2.29 | -3.9872 | 0.255 |
| VPS13B | ILMN_2268409 | 0.108 | 0.0307 | 2.22 | -4.13017 | 0.255 |
| LPP | ILMN_1839019 | 0.0177 | 0.00232 | 3.19 | -1.8256 | 0.254 |
| PRMT2 | ILMN_2259119 | 0.13 | 0.0404 | 2.1 | -4.36419 | 0.254 |
| SSH2 | ILMN_1672834 | 0.0489 | 0.0098 | 2.68 | -3.12848 | 0.253 |
| TBC1D32 | ILMN_2228196 | 0.0666 | 0.0153 | 2.5 | -3.52323 | 0.253 |
| IRF2BPL | ILMN_1804396 | 0.075 | 0.0181 | 2.43 | -3.67318 | 0.253 |
| TPD52L2 | ILMN_1699570 | 0.00644 | 0.000563 | 3.66 | -0.5207 | 0.252 |
| RPL26L1 | ILMN_2110532 | 0.0128 | 0.00145 | 3.35 | -1.39678 | 0.252 |
| ZFP36 | ILMN_1720829 | 0.0372 | 0.0066 | 2.82 | -2.77444 | 0.252 |
| BORCS7 | ILMN_1772706 | 0.081 | 0.0203 | 2.39 | -3.76953 | 0.252 |
| SHISA5 | ILMN_2139100 | 0.0816 | 0.0205 | 2.39 | -3.78004 | 0.252 |
| NUP98 | ILMN_1684074 | 0.14 | 0.0451 | 2.05 | -4.45623 | 0.252 |
| SKAP2 | ILMN_2125010 | 0.000569 | 2.11E-05 | 4.65 | 2.56032 | 0.251 |
| ATP6V0E1 | ILMN_1715635 | 0.0131 | 0.00151 | 3.34 | -1.43267 | 0.251 |
| APP | ILMN_2404065 | 0.0154 | 0.0019 | 3.26 | -1.64355 | 0.251 |
| IL9R | ILMN_1794686 | 0.134 | 0.0424 | 2.08 | -4.40505 | 0.251 |
| CKAP2 | ILMN_1674411 | 0.138 | 0.0442 | 2.06 | -4.4405 | 0.251 |
| ERICH1 | ILMN_2104696 | 0.000736 | 3.00E-05 | 4.55 | 2.22854 | 0.25 |
| CHPT1 | ILMN_1729112 | 0.00628 | 0.000536 | 3.68 | -0.47589 | 0.25 |
| ADAM17 | ILMN_2121068 | 0.0135 | 0.00157 | 3.32 | -1.47007 | 0.25 |
| TMEM80 | ILMN_1708482 | 0.0216 | 0.00309 | 3.09 | -2.08681 | 0.25 |
| DENND4C | ILMN_1669599 | 0.0231 | 0.0034 | 3.06 | -2.17579 | 0.25 |
| PRKAA1 | ILMN_1783889 | 0.0241 | 0.0036 | 3.04 | -2.22765 | 0.25 |
| FAM50A | ILMN_1725130 | 0.0515 | 0.0105 | 2.65 | -3.19051 | 0.25 |
| ATXN1 | ILMN_2153332 | 0.104 | 0.0291 | 2.24 | -4.0828 | 0.25 |
| PTPRC | ILMN_1804279 | 0.115 | 0.034 | 2.17 | -4.21764 | 0.25 |
| CTDP1 | ILMN_1815759 | 0.144 | 0.0468 | 2.03 | -4.48922 | 0.25 |
| SEC62 | ILMN_1762003 | 0.0454 | 0.00872 | 2.72 | -3.02422 | 0.249 |
| UBXN7 | ILMN_1733116 | 0.0762 | 0.0186 | 2.43 | -3.6926 | 0.249 |
| MCHR2 | ILMN_2383150 | 0.0744 | 0.0179 | 2.44 | -3.66126 | 0.248 |
| LSR | ILMN_1749396 | 0.0888 | 0.0232 | 2.33 | -3.88913 | 0.248 |
| NSMCE1 | ILMN_1697962 | 0.135 | 0.0431 | 2.07 | -4.41878 | 0.248 |
| TIPARP | ILMN_1765578 | 0.0186 | 0.00248 | 3.17 | -1.88604 | 0.247 |
| MBOAT7 | ILMN_1722218 | 0.0194 | 0.00265 | 3.15 | -1.94785 | 0.247 |
| EML3 | ILMN_1772644 | 0.0212 | 0.00301 | 3.1 | -2.06396 | 0.247 |
| TUBB2A | ILMN_2038775 | 0.0872 | 0.0226 | 2.35 | -3.86449 | 0.247 |
| SFXN3 | ILMN_1662910 | 0.0897 | 0.0235 | 2.33 | -3.90029 | 0.247 |
| LOC101928596 | ILMN_1837348 | 0.114 | 0.0334 | 2.18 | -4.20254 | 0.247 |
| CA4 | ILMN_1695157 | 0.118 | 0.0352 | 2.16 | -4.24669 | 0.247 |
| TK2 | ILMN_1766814 | 0.139 | 0.0446 | 2.06 | -4.44686 | 0.247 |
| METRNL | ILMN_2342066 | 0.008 | 0.000771 | 3.56 | -0.81175 | 0.246 |
| EEF1D | ILMN_1782543 | 0.0127 | 0.00144 | 3.35 | -1.39016 | 0.246 |
| METTL23 | ILMN_2382724 | 0.0204 | 0.00284 | 3.12 | -2.01066 | 0.246 |
| PCBP2 | ILMN_3251155 | 0.0205 | 0.00285 | 3.12 | -2.01415 | 0.246 |
| VNN2 | ILMN_1758864 | 0.146 | 0.0478 | 2.02 | -4.50688 | 0.246 |
| ETS1 | ILMN_1687538 | 0.0481 | 0.00953 | 2.69 | -3.10355 | 0.245 |
| DSTYK | ILMN_1779600 | 0.148 | 0.0491 | 2.01 | -4.52944 | 0.245 |
| METTL9 | ILMN_1726421 | 0.0828 | 0.0209 | 2.38 | -3.79731 | 0.244 |
| KIR3DL2 | ILMN_2190842 | 0.13 | 0.0406 | 2.1 | -4.3675 | 0.244 |
| BIN1 | ILMN_1674160 | 0.053 | 0.011 | 2.63 | -3.23349 | 0.243 |
| ZNF33B | ILMN_1742935 | 0.0967 | 0.0262 | 2.28 | -3.9927 | 0.243 |
| NLN | ILMN_1742089 | 0.124 | 0.0376 | 2.13 | -4.30358 | 0.243 |
| TMEM11 | ILMN_2175265 | 0.141 | 0.0455 | 2.05 | -4.46503 | 0.243 |
| ZFC3H1 | ILMN_3244096 | 0.00361 | 0.000251 | 3.91 | 0.23294 | 0.242 |
| ANTXR2 | ILMN_1812926 | 0.0486 | 0.00969 | 2.68 | -3.11822 | 0.242 |
| MDM4 | ILMN_1746020 | 0.0733 | 0.0176 | 2.45 | -3.64468 | 0.242 |
| GNG7 | ILMN_1728107 | 0.112 | 0.0323 | 2.2 | -4.17421 | 0.242 |
| LEPROT | ILMN_1661537 | 0.0178 | 0.00232 | 3.19 | -1.82798 | 0.241 |
| ZFAS1 | ILMN_3188984 | 0.0517 | 0.0106 | 2.65 | -3.19655 | 0.241 |
| TGFBRAP1 | ILMN_1696870 | 0.113 | 0.0331 | 2.19 | -4.19323 | 0.241 |
| IGFBPL1 | ILMN_1677158 | 0.132 | 0.0413 | 2.09 | -4.38339 | 0.241 |
| SECISBP2 | ILMN_1736481 | 0.0155 | 0.00191 | 3.26 | -1.64901 | 0.24 |
| ITGAV | ILMN_2169439 | 0.0389 | 0.00704 | 2.8 | -2.83258 | 0.24 |
| LMNA | ILMN_1696749 | 0.117 | 0.035 | 2.16 | -4.24073 | 0.24 |
| ADAM17 | ILMN_1765779 | 0.142 | 0.046 | 2.04 | -4.47457 | 0.24 |
| TMEM42 | ILMN_1760245 | 0.0786 | 0.0194 | 2.41 | -3.73009 | 0.239 |
| KYAT3 | ILMN_1778371 | 0.0931 | 0.0247 | 2.31 | -3.94316 | 0.239 |
| RMI1 | ILMN_1754051 | 0.0628 | 0.014 | 2.54 | -3.44625 | 0.238 |
| PAF1 | ILMN_1669508 | 0.0879 | 0.0229 | 2.34 | -3.87727 | 0.238 |
| LSM2 | ILMN_2070300 | 0.129 | 0.0401 | 2.1 | -4.35812 | 0.238 |
| EDARADD | ILMN_1761820 | 0.144 | 0.0469 | 2.03 | -4.49091 | 0.238 |
| ACOT9 | ILMN_2367070 | 0.0489 | 0.00981 | 2.67 | -3.12945 | 0.237 |
| ANKRD39 | ILMN_1710979 | 0.0612 | 0.0135 | 2.55 | -3.41145 | 0.237 |
| TTLL1 | ILMN_2372795 | 0.064 | 0.0144 | 2.53 | -3.47244 | 0.237 |
| DEK | ILMN_1747630 | 0.0985 | 0.027 | 2.27 | -4.01719 | 0.237 |
| FAM53C | ILMN_1744508 | 0.0144 | 0.00173 | 3.29 | -1.55613 | 0.236 |
| UBE2B | ILMN_1663099 | 0.0275 | 0.00434 | 2.97 | -2.39661 | 0.236 |
| P2RY8 | ILMN_1768284 | 0.0713 | 0.0168 | 2.47 | -3.60581 | 0.236 |
| CELF2 | ILMN_1800638 | 0.113 | 0.0328 | 2.19 | -4.18692 | 0.236 |
| IFT88 | ILMN_2373755 | 0.116 | 0.0345 | 2.17 | -4.22974 | 0.236 |
| HLA-DRB4 | ILMN_1679617 | 0.139 | 0.0446 | 2.06 | -4.44686 | 0.236 |
| CSF2RA | ILMN_1721204 | 0.0116 | 0.00127 | 3.4 | -1.2751 | 0.235 |
| USP3 | ILMN_1725862 | 0.0278 | 0.0044 | 2.97 | -2.40899 | 0.235 |
| GAB2 | ILMN_1815758 | 0.0528 | 0.011 | 2.63 | -3.22821 | 0.235 |
| UNC93B1 | ILMN_2193591 | 0.122 | 0.0368 | 2.14 | -4.28503 | 0.235 |
| ZEB2 | ILMN_1820767 | 0.137 | 0.0436 | 2.06 | -4.42943 | 0.235 |
| RPA2 | ILMN_1753582 | 0.0353 | 0.00615 | 2.85 | -2.71174 | 0.234 |
| LOC105274304 | ILMN_1902929 | 0.0656 | 0.015 | 2.51 | -3.50706 | 0.234 |
| CFAP58 | ILMN_2100287 | 0.0702 | 0.0165 | 2.47 | -3.58971 | 0.234 |
| SP110 | ILMN_1672661 | 0.105 | 0.0294 | 2.24 | -4.09209 | 0.234 |
| B3GAT1 | ILMN_1794072 | 0.11 | 0.0318 | 2.2 | -4.16025 | 0.234 |
| USF2 | ILMN_1756696 | 0.112 | 0.0325 | 2.19 | -4.17742 | 0.234 |
| HSP90AA1 | ILMN_1691097 | 0.134 | 0.0422 | 2.08 | -4.40116 | 0.234 |
| ZNF32 | ILMN_1695362 | 0.138 | 0.0442 | 2.06 | -4.44025 | 0.234 |
| RHEB | ILMN_1657949 | 0.149 | 0.0496 | 2.01 | -4.53659 | 0.234 |
| ANXA11 | ILMN_1685170 | 0.00689 | 0.000624 | 3.63 | -0.61582 | 0.233 |
| RIT1 | ILMN_1656335 | 0.0191 | 0.00258 | 3.16 | -1.92214 | 0.233 |
| SIL1 | ILMN_1678729 | 0.0732 | 0.0175 | 2.45 | -3.64326 | 0.233 |
| DCLK1 | ILMN_1731616 | 0.131 | 0.0409 | 2.09 | -4.37493 | 0.233 |
| HEBP2 | ILMN_1755077 | 0.00908 | 0.00092 | 3.5 | -0.97588 | 0.232 |
| HNRNPM | ILMN_3269405 | 0.0187 | 0.00251 | 3.17 | -1.89722 | 0.232 |
| LONRF1 | ILMN_1705953 | 0.0488 | 0.00975 | 2.68 | -3.12382 | 0.232 |
| RNF213 | ILMN_1731203 | 0.101 | 0.0278 | 2.26 | -4.04461 | 0.232 |
| AK3 | ILMN_1778173 | 0.138 | 0.0443 | 2.06 | -4.4422 | 0.232 |
| PER3 | ILMN_1660986 | 0.144 | 0.0471 | 2.03 | -4.49364 | 0.232 |
| RALBP1 | ILMN_1791840 | 0.0122 | 0.00137 | 3.37 | -1.34275 | 0.231 |
| IFITM4P | ILMN_1770071 | 0.0636 | 0.0143 | 2.53 | -3.46396 | 0.231 |
| IL1RAP | ILMN_2357062 | 0.0784 | 0.0193 | 2.41 | -3.72729 | 0.231 |
| NFKBIA | ILMN_1773154 | 0.0102 | 0.00108 | 3.45 | -1.12145 | 0.23 |
| MIR345 | ILMN_3310386 | 0.0239 | 0.00357 | 3.04 | -2.21959 | 0.23 |
| KLHL24 | ILMN_1678671 | 0.0484 | 0.00962 | 2.68 | -3.11213 | 0.23 |
| NACA2 | ILMN_2158548 | 0.0728 | 0.0174 | 2.45 | -3.63453 | 0.23 |
| PPL | ILMN_1806030 | 0.0675 | 0.0156 | 2.49 | -3.54062 | 0.229 |
| ABLIM1 | ILMN_1731610 | 0.0986 | 0.027 | 2.27 | -4.0183 | 0.229 |
| B3GNTL1 | ILMN_1702817 | 0.116 | 0.0342 | 2.17 | -4.22319 | 0.229 |
| ZEB1-AS1 | ILMN_1829989 | 0.148 | 0.049 | 2.01 | -4.52712 | 0.229 |
| AAMDC | ILMN_1779163 | 0.0832 | 0.0211 | 2.37 | -3.8044 | 0.228 |
| RNU4-1 | ILMN_3309453 | 0.131 | 0.0411 | 2.09 | -4.37804 | 0.228 |
| TMEM126B | ILMN_2145518 | 0.134 | 0.0426 | 2.08 | -4.40947 | 0.228 |
| FZD7 | ILMN_2108823 | 0.0441 | 0.00838 | 2.73 | -2.98912 | 0.227 |
| RNA5S9 | ILMN_3234762 | 0.0458 | 0.00885 | 2.71 | -3.03736 | 0.227 |
| WDR91 | ILMN_1652223 | 0.0809 | 0.0202 | 2.39 | -3.76735 | 0.227 |
| SRI | ILMN_1699525 | 0.148 | 0.0491 | 2.01 | -4.52952 | 0.227 |
| GRK5 | ILMN_1728830 | 0.0206 | 0.00288 | 3.12 | -2.0246 | 0.226 |
| SLC22A4 | ILMN_2050911 | 0.0399 | 0.00727 | 2.79 | -2.86102 | 0.226 |
| CD53 | ILMN_1662843 | 0.0535 | 0.0112 | 2.62 | -3.2457 | 0.226 |
| VRK2 | ILMN_1750088 | 0.054 | 0.0113 | 2.62 | -3.25772 | 0.226 |
| FOXO1 | ILMN_1738816 | 0.097 | 0.0263 | 2.28 | -3.99549 | 0.226 |
| TALDO1 | ILMN_1746588 | 0.00213 | 0.000124 | 4.13 | 0.89128 | 0.225 |
| IAH1 | ILMN_2217329 | 0.0279 | 0.00441 | 2.97 | -2.4106 | 0.225 |
| YBX1 | ILMN_2124769 | 0.0301 | 0.00489 | 2.93 | -2.50429 | 0.225 |
| FBXO30 | ILMN_1700232 | 0.0519 | 0.0107 | 2.64 | -3.20301 | 0.225 |
| ERGIC1 | ILMN_1664068 | 0.0523 | 0.0108 | 2.64 | -3.21423 | 0.225 |
| STK24 | ILMN_1695773 | 0.107 | 0.0306 | 2.22 | -4.12594 | 0.225 |
| STK17A | ILMN_1776428 | 0.113 | 0.033 | 2.19 | -4.19223 | 0.225 |
| HSDL2 | ILMN_1787843 | 0.122 | 0.037 | 2.14 | -4.28879 | 0.225 |
| WLS | ILMN_1671260 | 0.123 | 0.0373 | 2.13 | -4.29699 | 0.225 |
| IFNGR1 | ILMN_1675939 | 0.00663 | 0.000591 | 3.64 | -0.5659 | 0.224 |
| C7orf49 | ILMN_1740903 | 0.0795 | 0.0197 | 2.4 | -3.7458 | 0.224 |
| TRMT44 | ILMN_1798129 | 0.104 | 0.0291 | 2.24 | -4.08406 | 0.224 |
| USP4 | ILMN_1773505 | 0.0463 | 0.00898 | 2.71 | -3.05096 | 0.223 |
| CPNE1 | ILMN_2276000 | 0.097 | 0.0263 | 2.28 | -3.99732 | 0.223 |
| IRF8 | ILMN_1666594 | 0.103 | 0.0288 | 2.25 | -4.07379 | 0.223 |
| C9orf142 | ILMN_1761138 | 0.105 | 0.0296 | 2.23 | -4.0977 | 0.223 |
| EGLN2 | ILMN_1768773 | 0.00716 | 0.000659 | 3.61 | -0.66637 | 0.222 |
| ENSA | ILMN_2364700 | 0.00827 | 0.000804 | 3.55 | -0.85081 | 0.222 |
| OR11H1 | ILMN_2072401 | 0.00941 | 0.000968 | 3.49 | -1.02234 | 0.222 |
| IKZF2 | ILMN_1893909 | 0.0348 | 0.00602 | 2.86 | -2.69219 | 0.222 |
| CCDC93 | ILMN_3248263 | 0.103 | 0.0286 | 2.25 | -4.06716 | 0.222 |
| CPEB2-AS1 | ILMN_3294322 | 0.00792 | 0.000758 | 3.56 | -0.79708 | 0.221 |
| BEST4 | ILMN_1796685 | 0.097 | 0.0263 | 2.28 | -3.9973 | 0.221 |
| COL16A1 | ILMN_1684554 | 0.0985 | 0.0269 | 2.27 | -4.01626 | 0.221 |
| C5orf47 | ILMN_1718030 | 0.116 | 0.0344 | 2.17 | -4.22738 | 0.221 |
| CDKN2D | ILMN_1748883 | 0.14 | 0.0452 | 2.05 | -4.45921 | 0.221 |
| TXNDC5 | ILMN_1788108 | 0.0241 | 0.00362 | 3.04 | -2.23185 | 0.22 |
| THNSL2 | ILMN_2173294 | 0.0673 | 0.0155 | 2.5 | -3.53547 | 0.22 |
| RPS6KA3 | ILMN_1806294 | 0.115 | 0.034 | 2.17 | -4.21771 | 0.22 |
| HMGB2 | ILMN_2219712 | 0.143 | 0.0465 | 2.04 | -4.48243 | 0.22 |
| VPS8 | ILMN_1678268 | 0.0198 | 0.00272 | 3.14 | -1.97211 | 0.219 |
| CBLB | ILMN_1685580 | 0.108 | 0.0308 | 2.22 | -4.13092 | 0.219 |
| PACS1 | ILMN_1716488 | 0.108 | 0.031 | 2.21 | -4.13659 | 0.219 |
| GALK2 | ILMN_1732514 | 0.115 | 0.0341 | 2.17 | -4.22044 | 0.219 |
| EPM2A | ILMN_1719468 | 0.119 | 0.0358 | 2.15 | -4.26178 | 0.219 |
| ZXDC | ILMN_2348512 | 0.122 | 0.037 | 2.14 | -4.28938 | 0.219 |
| VCP | ILMN_1777220 | 0.124 | 0.0376 | 2.13 | -4.30304 | 0.219 |
| MSI2 | ILMN_1804448 | 0.137 | 0.0438 | 2.06 | -4.43223 | 0.219 |
| REXO2 | ILMN_1749009 | 0.145 | 0.0474 | 2.03 | -4.49832 | 0.219 |
| TRADD | ILMN_1765851 | 0.0175 | 0.00228 | 3.2 | -1.80903 | 0.218 |
| WDR45 | ILMN_2251279 | 0.021 | 0.00297 | 3.11 | -2.05138 | 0.218 |
| LRP10 | ILMN_1670272 | 0.0211 | 0.00298 | 3.11 | -2.05464 | 0.218 |
| KLHL21 | ILMN_1692785 | 0.0588 | 0.0128 | 2.57 | -3.36318 | 0.218 |
| APP | ILMN_2404063 | 0.0654 | 0.0149 | 2.51 | -3.50219 | 0.218 |
| NDRG1 | ILMN_1809931 | 0.0737 | 0.0177 | 2.45 | -3.65036 | 0.218 |
| RNF115 | ILMN_1811997 | 0.111 | 0.0322 | 2.2 | -4.17037 | 0.218 |
| CPQ | ILMN_1713031 | 0.0656 | 0.015 | 2.51 | -3.50688 | 0.217 |
| TRIB1 | ILMN_1803811 | 0.0688 | 0.016 | 2.48 | -3.5647 | 0.217 |
| TMEM120A | ILMN_1654516 | 0.0858 | 0.022 | 2.36 | -3.84224 | 0.217 |
| ADAM19 | ILMN_1713751 | 0.0878 | 0.0229 | 2.34 | -3.87545 | 0.217 |
| KIAA0226L | ILMN_2196550 | 0.0962 | 0.026 | 2.29 | -3.98489 | 0.217 |
| SPPL3 | ILMN_1657405 | 0.114 | 0.0335 | 2.18 | -4.20527 | 0.217 |
| UPF1 | ILMN_1688011 | 0.0117 | 0.0013 | 3.39 | -1.29522 | 0.216 |
| HIST2H2AC | ILMN_1768973 | 0.0404 | 0.00741 | 2.78 | -2.87867 | 0.216 |
| G6PD | ILMN_2347949 | 0.072 | 0.0171 | 2.46 | -3.6217 | 0.216 |
| MGST3 | ILMN_1751956 | 0.0335 | 0.00568 | 2.88 | -2.64021 | 0.215 |
| RAB1A | ILMN_2118864 | 0.072 | 0.0171 | 2.46 | -3.62068 | 0.215 |
| CAPNS1 | ILMN_1655418 | 0.0822 | 0.0207 | 2.38 | -3.78765 | 0.215 |
| SMIM4 | ILMN_3245869 | 0.127 | 0.0393 | 2.11 | -4.34139 | 0.215 |
| IKBKG | ILMN_1707308 | 0.00159 | 8.44E-05 | 4.24 | 1.25354 | 0.214 |
| UQCRHL | ILMN_1718136 | 0.0464 | 0.009 | 2.71 | -3.05219 | 0.214 |
| ATP8B4 | ILMN_1783956 | 0.0957 | 0.0257 | 2.29 | -3.97748 | 0.214 |
| HSPBAP1 | ILMN_1797031 | 0.131 | 0.041 | 2.09 | -4.37656 | 0.214 |
| NDEL1 | ILMN_1705064 | 0.0132 | 0.00153 | 3.33 | -1.44699 | 0.213 |
| VPS28 | ILMN_2339863 | 0.0133 | 0.00155 | 3.33 | -1.45605 | 0.213 |
| CPEB4 | ILMN_1722025 | 0.0911 | 0.024 | 2.32 | -3.91734 | 0.213 |
| TCAF2 | ILMN_1778202 | 0.0967 | 0.0262 | 2.29 | -3.99152 | 0.213 |
| GMPR2 | ILMN_1677919 | 0.0555 | 0.0118 | 2.61 | -3.29082 | 0.212 |
| BRMS1L | ILMN_2225511 | 0.105 | 0.0297 | 2.23 | -4.10159 | 0.212 |
| TOR1AIP2 | ILMN_2113938 | 0.134 | 0.0421 | 2.08 | -4.39968 | 0.212 |
| LINC01003 | ILMN_3248282 | 0.149 | 0.0495 | 2.01 | -4.53587 | 0.212 |
| RPS9 | ILMN_2038772 | 0.0229 | 0.00335 | 3.07 | -2.16083 | 0.211 |
| HCFC1R1 | ILMN_1757877 | 0.0694 | 0.0163 | 2.48 | -3.57633 | 0.211 |
| EXO5 | ILMN_2117569 | 0.116 | 0.0346 | 2.17 | -4.23196 | 0.211 |
| GAB2 | ILMN_1665964 | 0.0158 | 0.00197 | 3.25 | -1.67772 | 0.209 |
| NOL8 | ILMN_1689747 | 0.0192 | 0.00261 | 3.15 | -1.93229 | 0.209 |
| DBI | ILMN_1755926 | 0.0235 | 0.00348 | 3.05 | -2.19617 | 0.209 |
| RBM38 | ILMN_2404049 | 0.0388 | 0.00699 | 2.8 | -2.82577 | 0.209 |
| NYAP2 | ILMN_1781890 | 0.0954 | 0.0256 | 2.29 | -3.9738 | 0.209 |
| ANXA11 | ILMN_2380494 | 0.0157 | 0.00195 | 3.25 | -1.66771 | 0.208 |
| AUTS2 | ILMN_1749081 | 0.0185 | 0.00246 | 3.17 | -1.87999 | 0.208 |
| ANXA2 | ILMN_2409167 | 0.0249 | 0.0038 | 3.02 | -2.27526 | 0.208 |
| INAFM1 | ILMN_1703316 | 0.0434 | 0.00818 | 2.74 | -2.96733 | 0.208 |
| ZP3 | ILMN_1805377 | 0.0486 | 0.00969 | 2.68 | -3.11817 | 0.208 |
| DUSP22 | ILMN_1671809 | 0.0618 | 0.0137 | 2.55 | -3.42602 | 0.208 |
| TMEM156 | ILMN_2095660 | 0.0738 | 0.0177 | 2.44 | -3.65347 | 0.208 |
| RHBDL2 | ILMN_2053538 | 0.114 | 0.0334 | 2.18 | -4.20198 | 0.208 |
| WBP1L | ILMN_1658830 | 0.0725 | 0.0173 | 2.45 | -3.63082 | 0.207 |
| RANGAP1 | ILMN_1662198 | 0.115 | 0.0338 | 2.18 | -4.21286 | 0.207 |
| FBXW4 | ILMN_1671427 | 0.135 | 0.0428 | 2.07 | -4.41224 | 0.207 |
| FAAP20 | ILMN_2097790 | 0.129 | 0.0402 | 2.1 | -4.36027 | 0.206 |
| GABPB1 | ILMN_1761147 | 0.145 | 0.0476 | 2.03 | -4.50296 | 0.206 |
| HSCB | ILMN_2184789 | 0.147 | 0.0484 | 2.02 | -4.51657 | 0.206 |
| GABARAPL1 | ILMN_2151281 | 0.0221 | 0.00322 | 3.08 | -2.1243 | 0.205 |
| ANGPTL4 | ILMN_2386444 | 0.0592 | 0.0129 | 2.57 | -3.37211 | 0.205 |
| PHYKPL | ILMN_1673529 | 0.136 | 0.0433 | 2.07 | -4.42194 | 0.205 |
| CWF19L1 | ILMN_1651886 | 0.014 | 0.00166 | 3.31 | -1.5217 | 0.204 |
| AP1G1 | ILMN_2399622 | 0.0297 | 0.0048 | 2.94 | -2.48763 | 0.204 |
| UQCRH | ILMN_2232936 | 0.0388 | 0.00699 | 2.8 | -2.82677 | 0.204 |
| STAT3 | ILMN_1663618 | 0.113 | 0.0328 | 2.19 | -4.18707 | 0.204 |
| CSF2RA | ILMN_2376458 | 0.00692 | 0.000628 | 3.63 | -0.62277 | 0.203 |
| CIRBP | ILMN_1674661 | 0.052 | 0.0107 | 2.64 | -3.20636 | 0.203 |
| NUFIP2 | ILMN_1765829 | 0.0824 | 0.0208 | 2.38 | -3.79079 | 0.203 |
| RRP12 | ILMN_1767253 | 0.0859 | 0.0221 | 2.36 | -3.84405 | 0.203 |
| MPZ | ILMN_1810937 | 0.131 | 0.0412 | 2.09 | -4.37966 | 0.203 |
| ZFYVE16 | ILMN_2140999 | 0.144 | 0.0468 | 2.03 | -4.48756 | 0.203 |
| PPP3R1 | ILMN_1796962 | 0.0116 | 0.00127 | 3.4 | -1.27533 | 0.202 |
| TTI2 | ILMN_1760400 | 0.0374 | 0.00665 | 2.82 | -2.78099 | 0.202 |
| SUMO1P1 | ILMN_1785615 | 0.0463 | 0.00896 | 2.71 | -3.04811 | 0.202 |
| MFSD1 | ILMN_1789751 | 0.0552 | 0.0117 | 2.61 | -3.2842 | 0.202 |
| DNM2 | ILMN_1656822 | 0.0575 | 0.0124 | 2.59 | -3.33493 | 0.202 |
| TSTD1 | ILMN_3197097 | 0.0871 | 0.0226 | 2.35 | -3.86299 | 0.202 |
| HSP90AA1 | ILMN_2373515 | 0.145 | 0.0473 | 2.03 | -4.49731 | 0.202 |
| FAM177A1 | ILMN_1763603 | 0.0881 | 0.023 | 2.34 | -3.87982 | 0.201 |
| UQCRC1 | ILMN_1671191 | 0.144 | 0.047 | 2.03 | -4.49192 | 0.201 |
| DYNC1H1 | ILMN_1780302 | 0.0195 | 0.00266 | 3.15 | -1.95215 | 0.2 |
| CIR1 | ILMN_1671516 | 0.0217 | 0.00311 | 3.09 | -2.09301 | 0.2 |
| PEX11B | ILMN_1678546 | 0.0228 | 0.00333 | 3.07 | -2.15634 | 0.2 |
| HNRNPA2B1 | ILMN_3273854 | 0.0235 | 0.00348 | 3.05 | -2.19632 | 0.2 |
| ENSA | ILMN_1760779 | 0.0457 | 0.00881 | 2.72 | -3.03345 | 0.2 |
| KAT6A | ILMN_2095840 | 0.0688 | 0.016 | 2.48 | -3.56307 | 0.2 |
| ZNF786 | ILMN_1713706 | 0.0741 | 0.0178 | 2.44 | -3.65707 | 0.2 |
| THEMIS2 | ILMN_1735143 | 0.0625 | 0.014 | 2.54 | -3.44207 | 0.199 |
| FTSJ3 | ILMN_1811692 | 0.14 | 0.045 | 2.05 | -4.45594 | 0.199 |
| KDM5B | ILMN_1755727 | 0.00775 | 0.000733 | 3.58 | -0.76487 | 0.198 |
| RNY1 | ILMN_3237623 | 0.0256 | 0.00393 | 3.01 | -2.30639 | 0.198 |
| ARL6IP1 | ILMN_1708416 | 0.0306 | 0.00499 | 2.92 | -2.52258 | 0.198 |
| TMPPE | ILMN_1774211 | 0.0667 | 0.0153 | 2.5 | -3.52422 | 0.198 |
| TPD52L2 | ILMN_2323633 | 0.106 | 0.0299 | 2.23 | -4.10585 | 0.198 |
| PAFAH1B2 | ILMN_1743319 | 0.107 | 0.0305 | 2.22 | -4.12314 | 0.198 |
| ZNF672 | ILMN_1669094 | 0.124 | 0.0377 | 2.13 | -4.3061 | 0.198 |
| RHOT1 | ILMN_1678504 | 0.14 | 0.0449 | 2.05 | -4.45426 | 0.198 |
| DOCK5 | ILMN_1752455 | 0.0379 | 0.00679 | 2.81 | -2.79958 | 0.197 |
| PCDHB9 | ILMN_2047885 | 0.11 | 0.0317 | 2.2 | -4.1579 | 0.197 |
| HIST1H2BH | ILMN_1688666 | 0.135 | 0.0431 | 2.07 | -4.41848 | 0.197 |
| AKR1B1 | ILMN_1701731 | 0.0315 | 0.00521 | 2.91 | -2.56251 | 0.196 |
| HNRNPM | ILMN_3192791 | 0.0686 | 0.016 | 2.49 | -3.55999 | 0.196 |
| ETS1 | ILMN_2122103 | 0.071 | 0.0167 | 2.47 | -3.60131 | 0.196 |
| NPIPB5 | ILMN_3246766 | 0.144 | 0.0472 | 2.03 | -4.49616 | 0.196 |
| SSTR2 | ILMN_2152257 | 0.146 | 0.0481 | 2.02 | -4.51187 | 0.196 |
| GDI1 | ILMN_1734153 | 0.05 | 0.0101 | 2.66 | -3.15894 | 0.195 |
| TGIF1 | ILMN_2318638 | 0.0955 | 0.0257 | 2.29 | -3.97487 | 0.195 |
| SCAP | ILMN_1677534 | 0.101 | 0.0278 | 2.26 | -4.04413 | 0.195 |
| TRIM9 | ILMN_1719254 | 0.114 | 0.0337 | 2.18 | -4.20938 | 0.195 |
| CALD1 | ILMN_1717990 | 0.143 | 0.0466 | 2.04 | -4.48537 | 0.195 |
| SLC12A6 | ILMN_1767992 | 0.0202 | 0.0028 | 3.13 | -1.99697 | 0.194 |
| ALX1 | ILMN_3236443 | 0.0787 | 0.0194 | 2.41 | -3.7336 | 0.194 |
| NT5C | ILMN_1806432 | 0.124 | 0.0379 | 2.13 | -4.31071 | 0.194 |
| ASMTL | ILMN_1732615 | 0.132 | 0.0414 | 2.09 | -4.38549 | 0.194 |
| CCT6P1 | ILMN_2050617 | 0.0117 | 0.0013 | 3.39 | -1.29294 | 0.193 |
| VNN2 | ILMN_1678939 | 0.117 | 0.0349 | 2.16 | -4.24014 | 0.193 |
| HNRNPA2B1 | ILMN_2369682 | 0.00458 | 0.000349 | 3.81 | -0.07489 | 0.192 |
| TMX4 | ILMN_2204754 | 0.125 | 0.038 | 2.13 | -4.31176 | 0.192 |
| MED29 | ILMN_1728360 | 0.0277 | 0.00438 | 2.97 | -2.40449 | 0.191 |
| EFCAB14 | ILMN_1697597 | 0.0362 | 0.00636 | 2.84 | -2.74165 | 0.191 |
| SLC4A5 | ILMN_2273224 | 0.0613 | 0.0135 | 2.55 | -3.41444 | 0.191 |
| ERICH1 | ILMN_1731001 | 0.0148 | 0.00178 | 3.28 | -1.5857 | 0.19 |
| CEP162 | ILMN_1781174 | 0.0625 | 0.0139 | 2.54 | -3.44034 | 0.19 |
| NPIPB3 | ILMN_1656868 | 0.0918 | 0.0243 | 2.32 | -3.92703 | 0.19 |
| EDEM3 | ILMN_2065299 | 0.13 | 0.0404 | 2.1 | -4.36425 | 0.19 |
| FOXN2 | ILMN_1736510 | 0.0218 | 0.00312 | 3.09 | -2.0971 | 0.189 |
| FRAT2 | ILMN_1788213 | 0.0375 | 0.0067 | 2.82 | -2.78874 | 0.189 |
| DCXR | ILMN_1681437 | 0.0444 | 0.00846 | 2.73 | -2.99712 | 0.189 |
| MS4A3 | ILMN_1695530 | 0.0592 | 0.0129 | 2.57 | -3.3735 | 0.189 |
| MET | ILMN_1715175 | 0.0593 | 0.0129 | 2.57 | -3.3753 | 0.189 |
| SPC24 | ILMN_2181432 | 0.0723 | 0.0172 | 2.46 | -3.62767 | 0.189 |
| DCAF8 | ILMN_3244963 | 0.0813 | 0.0204 | 2.39 | -3.77438 | 0.189 |
| CHD7 | ILMN_1677376 | 0.0207 | 0.00291 | 3.12 | -2.03189 | 0.188 |
| STAT3 | ILMN_2401978 | 0.103 | 0.0286 | 2.25 | -4.0693 | 0.188 |
| MTURN | ILMN_1672605 | 0.129 | 0.0403 | 2.1 | -4.36133 | 0.188 |
| STX12 | ILMN_1773901 | 0.0531 | 0.0111 | 2.63 | -3.23613 | 0.187 |
| GNG5 | ILMN_1701854 | 0.0718 | 0.017 | 2.46 | -3.61708 | 0.187 |
| IKBKAP | ILMN_2211189 | 0.117 | 0.0348 | 2.16 | -4.23791 | 0.187 |
| RXRG | ILMN_1750624 | 0.134 | 0.0426 | 2.08 | -4.40912 | 0.187 |
| SLC25A3 | ILMN_1782890 | 0.00628 | 0.000536 | 3.68 | -0.47429 | 0.186 |
| AP1G1 | ILMN_2399627 | 0.0689 | 0.0161 | 2.48 | -3.56617 | 0.186 |
| ITGA7 | ILMN_1791409 | 0.13 | 0.0407 | 2.1 | -4.37011 | 0.186 |
| KIAA1551 | ILMN_2229922 | 0.0194 | 0.00264 | 3.15 | -1.94307 | 0.185 |
| DPM3 | ILMN_2376408 | 0.104 | 0.0291 | 2.24 | -4.08311 | 0.185 |
| CBX6 | ILMN_1691930 | 0.123 | 0.0374 | 2.13 | -4.29803 | 0.185 |
| TUBB4B | ILMN_1780769 | 0.127 | 0.0389 | 2.12 | -4.3319 | 0.185 |
| SVIL | ILMN_1690754 | 0.134 | 0.0423 | 2.08 | -4.40379 | 0.185 |
| TMEM50A | ILMN_1745368 | 0.0489 | 0.00979 | 2.68 | -3.12722 | 0.184 |
| RNF19A | ILMN_1812327 | 0.126 | 0.0384 | 2.12 | -4.32182 | 0.184 |
| LILRB3 | ILMN_1784884 | 0.145 | 0.0474 | 2.03 | -4.49885 | 0.184 |
| PABPC5 | ILMN_1796926 | 0.0526 | 0.0109 | 2.63 | -3.22432 | 0.183 |
| CNEP1R1 | ILMN_1653134 | 0.0556 | 0.0118 | 2.6 | -3.29284 | 0.183 |
| SCARNA16 | ILMN_3237446 | 0.112 | 0.0325 | 2.19 | -4.17854 | 0.183 |
| VPS28 | ILMN_1790797 | 0.0426 | 0.00798 | 2.75 | -2.94545 | 0.182 |
| ELF1 | ILMN_1664010 | 0.0148 | 0.00179 | 3.28 | -1.58717 | 0.181 |
| IRX1 | ILMN_1735353 | 0.0441 | 0.00838 | 2.73 | -2.9886 | 0.181 |
| C5AR1 | ILMN_1689836 | 0.0562 | 0.012 | 2.6 | -3.30737 | 0.181 |
| RAB7A | ILMN_1716524 | 0.0877 | 0.0228 | 2.34 | -3.87148 | 0.181 |
| MYLIP | ILMN_1656111 | 0.106 | 0.03 | 2.23 | -4.10921 | 0.181 |
| PRDX3 | ILMN_2395974 | 0.11 | 0.0314 | 2.21 | -4.14993 | 0.181 |
| UBN1 | ILMN_2399503 | 0.123 | 0.0374 | 2.13 | -4.29901 | 0.181 |
| GNA13 | ILMN_2176037 | 0.127 | 0.0392 | 2.11 | -4.33874 | 0.181 |
| FAM83F | ILMN_1683231 | 0.0887 | 0.0232 | 2.33 | -3.88814 | 0.18 |
| USP38 | ILMN_1704876 | 0.123 | 0.0375 | 2.13 | -4.30002 | 0.18 |
| PATL2 | ILMN_3306672 | 0.11 | 0.0317 | 2.2 | -4.15711 | 0.179 |
| OIP5-AS1 | ILMN_3297455 | 0.112 | 0.0323 | 2.2 | -4.1742 | 0.179 |
| MTMR10 | ILMN_1778734 | 0.105 | 0.0297 | 2.23 | -4.10148 | 0.178 |
| PRAF2 | ILMN_1720578 | 0.04 | 0.0073 | 2.79 | -2.86579 | 0.177 |
| HIST2H2AA4 | ILMN_3242900 | 0.0683 | 0.0158 | 2.49 | -3.55332 | 0.177 |
| OR2T6 | ILMN_1720617 | 0.147 | 0.0485 | 2.02 | -4.51787 | 0.177 |
| HIST2H2AA3 | ILMN_1659047 | 0.0673 | 0.0155 | 2.5 | -3.53452 | 0.176 |
| DYNC1I2 | ILMN_1773847 | 0.0737 | 0.0177 | 2.44 | -3.6509 | 0.176 |
| PRDX6 | ILMN_1803180 | 0.0795 | 0.0197 | 2.4 | -3.74563 | 0.176 |
| ATAD1 | ILMN_1654497 | 0.0806 | 0.0201 | 2.39 | -3.76346 | 0.176 |
| WDR37 | ILMN_1796464 | 0.11 | 0.0316 | 2.21 | -4.15477 | 0.176 |
| ADGRE5 | ILMN_1673363 | 0.148 | 0.0489 | 2.01 | -4.52461 | 0.176 |
| RABAC1 | ILMN_2207363 | 0.0466 | 0.00905 | 2.71 | -3.0578 | 0.175 |
| H2AFJ | ILMN_1708728 | 0.113 | 0.033 | 2.19 | -4.19025 | 0.175 |
| SESTD1 | ILMN_1724495 | 0.115 | 0.034 | 2.17 | -4.21698 | 0.175 |
| DHPS | ILMN_1687279 | 0.075 | 0.0181 | 2.43 | -3.67274 | 0.174 |
| FAM131A | ILMN_1729217 | 0.116 | 0.0346 | 2.17 | -4.23142 | 0.174 |
| SHKBP1 | ILMN_1765493 | 0.125 | 0.0382 | 2.12 | -4.31726 | 0.174 |
| H3F3C | ILMN_1769705 | 0.0127 | 0.00144 | 3.35 | -1.38778 | 0.172 |
| BTBD2 | ILMN_3238274 | 0.0298 | 0.00481 | 2.94 | -2.48974 | 0.172 |
| BASP1 | ILMN_1651826 | 0.0203 | 0.00282 | 3.13 | -2.00489 | 0.171 |
| GMPR2 | ILMN_2365595 | 0.131 | 0.0412 | 2.09 | -4.38138 | 0.171 |
| CPSF3 | ILMN_2087528 | 0.138 | 0.044 | 2.06 | -4.43688 | 0.171 |
| ARGLU1 | ILMN_1788468 | 0.0407 | 0.00748 | 2.78 | -2.88736 | 0.17 |
| TROVE2 | ILMN_2241775 | 0.117 | 0.0349 | 2.16 | -4.24013 | 0.17 |
| KIAA0922 | ILMN_1668469 | 0.0679 | 0.0157 | 2.49 | -3.54759 | 0.169 |
| UTS2 | ILMN_2236625 | 0.0937 | 0.025 | 2.3 | -3.95164 | 0.169 |
| SNX17 | ILMN_1732810 | 0.116 | 0.0346 | 2.17 | -4.23123 | 0.169 |
| RPS16 | ILMN_1651850 | 0.0161 | 0.00202 | 3.24 | -1.69867 | 0.168 |
| DGCR11 | ILMN_3236211 | 0.047 | 0.0092 | 2.7 | -3.07179 | 0.168 |
| KCNK3 | ILMN_1768483 | 0.123 | 0.0373 | 2.13 | -4.29575 | 0.168 |
| CD53 | ILMN_2413808 | 0.147 | 0.0486 | 2.02 | -4.51926 | 0.168 |
| RFTN1 | ILMN_1800787 | 0.00711 | 0.000653 | 3.61 | -0.65849 | 0.167 |
| SEC14L1 | ILMN_2391912 | 0.0182 | 0.0024 | 3.18 | -1.85739 | 0.167 |
| MSRA | ILMN_2228180 | 0.0547 | 0.0115 | 2.61 | -3.27318 | 0.167 |
| RGPD2 | ILMN_2056074 | 0.103 | 0.0285 | 2.25 | -4.06693 | 0.167 |
| SDCBP | ILMN_2363591 | 0.129 | 0.0403 | 2.1 | -4.36138 | 0.167 |
| C1orf56 | ILMN_1780268 | 0.0861 | 0.0222 | 2.35 | -3.84719 | 0.166 |
| GINS4 | ILMN_1807501 | 0.117 | 0.0349 | 2.16 | -4.24016 | 0.166 |
| EVI5 | ILMN_1746314 | 0.13 | 0.0404 | 2.1 | -4.36446 | 0.166 |
| LCP2 | ILMN_1658962 | 0.145 | 0.0476 | 2.03 | -4.50212 | 0.166 |
| EVI2B | ILMN_1763452 | 0.0273 | 0.00428 | 2.98 | -2.38459 | 0.165 |
| USP4 | ILMN_1798712 | 0.0493 | 0.00993 | 2.67 | -3.14066 | 0.165 |
| ENTPD3 | ILMN_2087941 | 0.064 | 0.0145 | 2.52 | -3.4741 | 0.165 |
| ZNF473 | ILMN_2346649 | 0.0749 | 0.0181 | 2.44 | -3.67058 | 0.165 |
| DPH3 | ILMN_2261973 | 0.121 | 0.0366 | 2.14 | -4.27891 | 0.165 |
| RNF149 | ILMN_1665877 | 0.0341 | 0.00585 | 2.87 | -2.66623 | 0.164 |
| UBE2W | ILMN_1717420 | 0.0275 | 0.00432 | 2.98 | -2.39265 | 0.163 |
| RHOA | ILMN_1781290 | 0.0498 | 0.0101 | 2.66 | -3.15316 | 0.163 |
| MRPL24 | ILMN_2398995 | 0.0552 | 0.0117 | 2.61 | -3.2853 | 0.163 |
| UBE2D2 | ILMN_1699503 | 0.059 | 0.0128 | 2.57 | -3.36828 | 0.163 |
| MEX3A | ILMN_3239177 | 0.112 | 0.0325 | 2.19 | -4.17903 | 0.163 |
| RAB31 | ILMN_1660691 | 0.0647 | 0.0147 | 2.52 | -3.48876 | 0.162 |
| RPS9 | ILMN_1749447 | 0.0715 | 0.0169 | 2.46 | -3.61021 | 0.162 |
| CDC42SE1 | ILMN_1769027 | 0.0857 | 0.022 | 2.36 | -3.84164 | 0.162 |
| NDUFA12 | ILMN_1737738 | 0.0272 | 0.00427 | 2.98 | -2.38214 | 0.161 |
| LAMTOR1 | ILMN_1815878 | 0.0756 | 0.0183 | 2.43 | -3.68264 | 0.161 |
| NRDC | ILMN_1800897 | 0.0634 | 0.0142 | 2.53 | -3.45756 | 0.16 |
| MCUB | ILMN_1801766 | 0.0769 | 0.0188 | 2.42 | -3.70445 | 0.16 |
| SEMA4D | ILMN_1687533 | 0.0931 | 0.0248 | 2.31 | -3.94404 | 0.16 |
| GIGYF2 | ILMN_1750718 | 0.097 | 0.0263 | 2.28 | -3.9965 | 0.16 |
| LTA4H | ILMN_1690342 | 0.102 | 0.0281 | 2.26 | -4.05326 | 0.16 |
| HBP1 | ILMN_2160764 | 0.017 | 0.00219 | 3.21 | -1.77418 | 0.159 |
| TOP1 | ILMN_2192316 | 0.131 | 0.0411 | 2.09 | -4.37921 | 0.159 |
| WDR45 | ILMN_1756146 | 0.0691 | 0.0161 | 2.48 | -3.57001 | 0.158 |
| HNRNPA2B1 | ILMN_1886493 | 0.0721 | 0.0171 | 2.46 | -3.62338 | 0.158 |
| DOPEY2 | ILMN_2180239 | 0.02 | 0.00276 | 3.13 | -1.98646 | 0.157 |
| HBP1 | ILMN_1685415 | 0.0429 | 0.00806 | 2.75 | -2.95383 | 0.157 |
| LYRM1 | ILMN_1749244 | 0.111 | 0.0319 | 2.2 | -4.16308 | 0.157 |
| BSDC1 | ILMN_1734483 | 0.0464 | 0.00899 | 2.71 | -3.0519 | 0.155 |
| METTL22 | ILMN_1658290 | 0.12 | 0.036 | 2.15 | -4.26561 | 0.155 |
| CTNNA1 | ILMN_1804854 | 0.129 | 0.0401 | 2.1 | -4.35858 | 0.155 |
| EVI2A | ILMN_1733579 | 0.027 | 0.00422 | 2.98 | -2.37025 | 0.154 |
| ACOT9 | ILMN_1658995 | 0.0895 | 0.0235 | 2.33 | -3.89755 | 0.154 |
| DKC1 | ILMN_1671257 | 0.0986 | 0.027 | 2.27 | -4.01877 | 0.154 |
| NKG7 | ILMN_1682993 | 0.112 | 0.0326 | 2.19 | -4.18168 | 0.154 |
| S100A13 | ILMN_2407168 | 0.0729 | 0.0174 | 2.45 | -3.63757 | 0.153 |
| CHIC2 | ILMN_1679428 | 0.0196 | 0.00268 | 3.14 | -1.95949 | 0.152 |
| EWSR1 | ILMN_2413251 | 0.138 | 0.0444 | 2.06 | -4.44373 | 0.152 |
| ZRANB2 | ILMN_1703015 | 0.127 | 0.0391 | 2.11 | -4.3369 | 0.151 |
| RNU1-3 | ILMN_3246273 | 0.118 | 0.0354 | 2.16 | -4.25133 | 0.15 |
| MAGEL2 | ILMN_1657478 | 0.139 | 0.0446 | 2.06 | -4.44712 | 0.15 |
| LAPTM4A | ILMN_1745110 | 0.146 | 0.0479 | 2.02 | -4.50843 | 0.15 |
| FXYD5 | ILMN_1704286 | 0.0374 | 0.00665 | 2.82 | -2.78182 | 0.149 |
| MED25 | ILMN_1811823 | 0.0601 | 0.0132 | 2.56 | -3.39117 | 0.149 |
| COX7A2L | ILMN_3237665 | 0.0691 | 0.0162 | 2.48 | -3.57082 | 0.149 |
| SNX22 | ILMN_2080760 | 0.0951 | 0.0255 | 2.3 | -3.96863 | 0.149 |
| MRPL23 | ILMN_1806123 | 0.11 | 0.0315 | 2.21 | -4.1516 | 0.149 |
| SYPL1 | ILMN_1764087 | 0.0364 | 0.0064 | 2.83 | -2.74757 | 0.148 |
| DPY19L1 | ILMN_1791296 | 0.0796 | 0.0198 | 2.4 | -3.74812 | 0.148 |
| ZMAT2 | ILMN_1745343 | 0.0579 | 0.0125 | 2.58 | -3.34395 | 0.147 |
| CREBRF | ILMN_2195821 | 0.0869 | 0.0225 | 2.35 | -3.86046 | 0.147 |
| APPL1 | ILMN_1763730 | 0.103 | 0.0288 | 2.24 | -4.07383 | 0.147 |
| GPC6 | ILMN_1805216 | 0.127 | 0.039 | 2.11 | -4.33481 | 0.147 |
| NTAN1 | ILMN_1815552 | 0.0534 | 0.0111 | 2.63 | -3.24236 | 0.146 |
| IQGAP1 | ILMN_1803819 | 0.0599 | 0.0131 | 2.56 | -3.3881 | 0.146 |
| SNORD13 | ILMN_1892403 | 0.0649 | 0.0148 | 2.52 | -3.49384 | 0.146 |
| AVPR2 | ILMN_1698324 | 0.115 | 0.0341 | 2.17 | -4.21959 | 0.146 |
| RNF19A | ILMN_2381197 | 0.0881 | 0.023 | 2.34 | -3.87985 | 0.145 |
| SNRNP35 | ILMN_1772845 | 0.0861 | 0.0221 | 2.35 | -3.84691 | 0.144 |
| STK24 | ILMN_1655163 | 0.0337 | 0.00573 | 2.87 | -2.64816 | 0.143 |
| CSF3R | ILMN_2323172 | 0.0915 | 0.0242 | 2.32 | -3.92233 | 0.142 |
| KMT2B | ILMN_1715968 | 0.0936 | 0.025 | 2.3 | -3.95073 | 0.142 |
| CD6 | ILMN_1746565 | 0.114 | 0.0336 | 2.18 | -4.20749 | 0.142 |
| SAFB | ILMN_1722059 | 0.12 | 0.0361 | 2.15 | -4.2675 | 0.142 |
| PQLC3 | ILMN_1814213 | 0.126 | 0.0384 | 2.12 | -4.32125 | 0.142 |
| TNPO3 | ILMN_1683811 | 0.111 | 0.0323 | 2.2 | -4.17228 | 0.141 |
| CDK2AP1 | ILMN_3245559 | 0.0434 | 0.00821 | 2.74 | -2.97025 | 0.14 |
| DNAJB6 | ILMN_2402416 | 0.0809 | 0.0202 | 2.39 | -3.7683 | 0.14 |
| TMEM140 | ILMN_1736863 | 0.0675 | 0.0156 | 2.5 | -3.53952 | 0.138 |
| COASY | ILMN_1753498 | 0.0939 | 0.0251 | 2.3 | -3.95406 | 0.138 |
| RIC8A | ILMN_1770733 | 0.1 | 0.0276 | 2.26 | -4.03701 | 0.138 |
| ANKS1A | ILMN_1813669 | 0.112 | 0.0324 | 2.19 | -4.17515 | 0.138 |
| SERTAD3 | ILMN_1801934 | 0.0986 | 0.027 | 2.27 | -4.01931 | 0.137 |
| RBM12 | ILMN_2329834 | 0.0737 | 0.0177 | 2.44 | -3.65125 | 0.136 |
| RPS4Y2 | ILMN_2191331 | 0.141 | 0.0457 | 2.04 | -4.46775 | 0.135 |
| CBX3 | ILMN_1790625 | 0.0697 | 0.0163 | 2.48 | -3.57991 | 0.134 |
| DDX59 | ILMN_1748077 | 0.133 | 0.0419 | 2.08 | -4.39578 | 0.134 |
| IFITM2 | ILMN_1673352 | 0.0149 | 0.00181 | 3.28 | -1.59856 | 0.133 |
| CORO7 | ILMN_1795949 | 0.143 | 0.0467 | 2.03 | -4.48596 | 0.133 |
| CHURC1 | ILMN_1798177 | 0.104 | 0.0291 | 2.24 | -4.08224 | 0.132 |
| CDC42SE1 | ILMN_2349138 | 0.135 | 0.0429 | 2.07 | -4.41454 | 0.132 |
| UBE2A | ILMN_2307455 | 0.0541 | 0.0113 | 2.62 | -3.25873 | 0.131 |
| STRN | ILMN_1749882 | 0.127 | 0.039 | 2.11 | -4.33362 | 0.131 |
| JADE1 | ILMN_1655194 | 0.149 | 0.0494 | 2.01 | -4.53298 | 0.131 |
| SUMO3 | ILMN_1725642 | 0.127 | 0.0393 | 2.11 | -4.3413 | 0.13 |
| ITGB1BP1 | ILMN_1690099 | 0.144 | 0.0472 | 2.03 | -4.49582 | 0.13 |
| LAMP2 | ILMN_1917290 | 0.0481 | 0.00951 | 2.69 | -3.10182 | 0.129 |
| TUG1 | ILMN_1682783 | 0.0866 | 0.0223 | 2.35 | -3.85487 | 0.129 |
| LEMD2 | ILMN_1680860 | 0.12 | 0.0361 | 2.15 | -4.26872 | 0.129 |
| CFD | ILMN_1777190 | 0.145 | 0.0478 | 2.02 | -4.50563 | 0.128 |
| SKP1 | ILMN_3229770 | 0.13 | 0.0404 | 2.1 | -4.36489 | 0.127 |
| GRK5 | ILMN_2096719 | 0.135 | 0.0429 | 2.07 | -4.41451 | 0.125 |
| EWSR1 | ILMN_1727041 | 0.0865 | 0.0223 | 2.35 | -3.8527 | 0.124 |
| GTF2H5 | ILMN_1739497 | 0.15 | 0.0499 | 2 | -4.54302 | 0.124 |
| UBXN4 | ILMN_1781097 | 0.12 | 0.0359 | 2.15 | -4.26375 | 0.123 |
| PLAA | ILMN_1810387 | 0.135 | 0.0427 | 2.07 | -4.41054 | 0.122 |
| DYNLRB1 | ILMN_1703564 | 0.0651 | 0.0149 | 2.51 | -3.49754 | 0.119 |
| ANKRD11 | ILMN_2108709 | 0.126 | 0.0386 | 2.12 | -4.32421 | 0.115 |
| TRPC4AP | ILMN_2402805 | 0.0854 | 0.0219 | 2.36 | -3.83803 | 0.111 |
| S100PBP | ILMN_2294274 | 0.144 | 0.0472 | 2.03 | -4.49621 | 0.111 |
| HNRNPA2B1 | ILMN_3178792 | 0.102 | 0.0281 | 2.26 | -4.05198 | 0.11 |
| LAMP2 | ILMN_2279961 | 0.0569 | 0.0122 | 2.59 | -3.3211 | 0.109 |
| ARNTL | ILMN_2405305 | 0.0979 | 0.0267 | 2.28 | -4.01021 | 0.107 |
| S100A11 | ILMN_1750101 | 0.0629 | 0.0141 | 2.54 | -3.44805 | 0.105 |
| ERP29 | ILMN_2323048 | 0.139 | 0.0449 | 2.05 | -4.45247 | 0.105 |
| FTH1P3 | ILMN_2173835 | 0.144 | 0.0472 | 2.03 | -4.49591 | 0.1 |
| UBC | ILMN_2038773 | 0.077 | 0.0188 | 2.42 | -3.70549 | 0.0961 |
| ZNF207 | ILMN_1778177 | 0.135 | 0.0427 | 2.07 | -4.41137 | 0.0954 |
| FTH1 | ILMN_1683146 | 0.112 | 0.0328 | 2.19 | -4.18602 | 0.0919 |
| H3F3A | ILMN_1699015 | 0.0742 | 0.0178 | 2.44 | -3.65823 | 0.0771 |
