# Supplementary material for: Comprehensive Analysis of Common Different Gene Expression Signatures in the Neutrophils of Sepsis
Source: Biomed Res Int. 2021 Apr 17;2021:6655425. doi: 10.1155/2021/6655425 (PMC8077712; doi:10.1155/2021/6655425)
Supplement: Supplementary 6 [file 6655425.f6.docx]

Table S6. Detailed information of DEGs in GSE6535

| Gene symbol | probe ID | adj.P.Val | P.Value | t Value | B value | logFC |
| --- | --- | --- | --- | --- | --- | --- |
| TM4SF1 | X75684 | 0.0910034 | 0.00101 | 3.4161976 | -0.82393 | 1.4102844 |
| NDUFC2 | NM_004549 | 0.0097313 | 6.35E-06 | 4.8225805 | 3.6179 | 1.3037208 |
| FAM167A | AF124366 | 0.010442 | 1.02E-05 | 4.7008129 | 3.19931 | 1.27293 |
| PLOD2 | NM_000935 | 0.1951426 | 0.00772 | 2.7371207 | -2.54479 | 1.2684727 |
| MRPS18A | NM_018135 | 0.0879607 | 0.00086 | 3.461701 | -0.687 | 1.2650823 |
| CDK5RAP2 | NM_018249 | 0.0153369 | 2.23E-05 | 4.4955673 | 2.50708 | 1.2621273 |
| RPL27 | NM_000988 | 0.0760431 | 0.000623 | 3.5569833 | -0.40996 | 1.235838 |
| LAIR1 | NM_002287 | 0.0393048 | 0.000135 | 4.0046147 | 0.92632 | 1.2219598 |
| EPB41L4A-AS1 | AB002437 | 0.0123772 | 1.46E-05 | 4.6076608 | 2.883 | 1.2131434 |
| IGFBP7 | NM_001553 | 0.0337456 | 0.000103 | 4.0799896 | 1.16162 | 1.2113217 |
| CYP1B1 | NM_000104 | 0.066993 | 0.000388 | 3.6987622 | 0.00153 | 1.1848395 |
| HBZ | NM_005332 | 0.1824064 | 0.00674 | 2.7801402 | -2.44034 | 1.1757136 |
| PRDX4 | NM_006406 | 0.0966792 | 0.00128 | 3.3379876 | -1.02837 | 1.1688209 |
| SOD1 | NM_000454 | 0.066993 | 0.000407 | 3.6852141 | -0.03828 | 1.1639128 |
| SSBP1 | NM_003143 | 0.2213313 | 0.0113 | 2.5929079 | -2.87912 | 1.1383063 |
| CTSC | NM_001814 | 0.010442 | 1.06E-05 | 4.6908879 | 3.16544 | 1.1314363 |
| MT1L | NM_002450 | 0.0676754 | 0.000419 | 3.6779649 | -0.0642 | 1.1303482 |
| FASTK | NM_006712 | 0.1094396 | 0.00182 | 3.2228458 | -1.3333 | 1.1240748 |
| CANX | L10284 | 0.0656815 | 0.000337 | 3.7402371 | 0.12401 | 1.109466 |
| ITGA7 | NM_002206 | 0.019989 | 3.86E-05 | 4.3497274 | 2.02599 | 1.1051747 |
| CBX5 | NM_012117 | 0.0538499 | 0.000222 | 3.8641276 | 0.48957 | 1.0966508 |
| UQCRH | NM_006004 | 0.2287376 | 0.0126 | 2.551746 | -2.97049 | 1.0957377 |
| RPL11 | NM_000975 | 0.1443034 | 0.00374 | 2.9846493 | -1.95043 | 1.0955054 |
| KPNA2 | NM_002266 | 0.178177 | 0.00637 | 2.7992444 | -2.40252 | 1.0887386 |
| FKBP3 | NM_002013 | 0.0288486 | 6.96E-05 | 4.1944893 | 1.50127 | 1.0857248 |
| FST | NM_006350 | 0.1967103 | 0.00789 | 2.7226133 | -2.58301 | 1.0856043 |
| ABCC4 | NM_005845 | 0.019989 | 3.75E-05 | 4.3573107 | 2.05077 | 1.0829536 |
| NDUFS5 | NM_004552 | 0.0908794 | 0.000975 | 3.4196418 | -0.79758 | 1.0819131 |
| NAE1 | NM_003905 | 0.0317356 | 9.18E-05 | 4.1121778 | 1.26296 | 1.0758751 |
| AFP | NM_001134 | 0.3085383 | 0.0275 | 2.2441909 | -3.61313 | 1.0745446 |
| DBI | M14200 | 0.0393432 | 0.000137 | 4.0023335 | 0.9085 | 1.0732362 |
| TFRC | NM_003234 | 0.2027285 | 0.00859 | 2.6919623 | -2.65406 | 1.0716586 |
| CDKN3 | NM_005192 | 0.019989 | 3.77E-05 | 4.3556481 | 2.04534 | 1.0702592 |
| COX16 | NM_016468 | 0.1335098 | 0.00293 | 3.0674659 | -1.7408 | 1.0649006 |
| PSMB7 | NM_002799 | 0.0418925 | 0.000148 | 3.9778642 | 0.8435 | 1.0506587 |
| ACADM | NM_000016 | 0.0309222 | 8.13E-05 | 4.145947 | 1.36981 | 1.0455425 |
| RPS27L | NM_015920 | 0.0834675 | 0.000761 | 3.496272 | -0.58266 | 1.042835 |
| C16orf59 | AK023971 | 0.0438052 | 0.000169 | 3.9404329 | 0.72821 | 1.0414424 |
| NDUFA8 | NM_014222 | 0.066993 | 0.000362 | 3.7198719 | 0.06375 | 1.0391619 |
| XBP1 | NM_005080 | 0.1193371 | 0.00229 | 3.1474368 | -1.53221 | 1.0363575 |
| PFN2 | NM_002628 | 0.1093261 | 0.0018 | 3.2261231 | -1.32457 | 1.0357232 |
| LRPAP1 | NM_002337 | 0.0097313 | 7.05E-06 | 4.795731 | 3.52511 | 1.0297953 |
| ANXA1 | NM_000700 | 0.0544513 | 0.000239 | 3.8408471 | 0.42501 | 1.0278327 |
| KRT8 | D28446 | 0.3010066 | 0.0249 | 2.2836639 | -3.53529 | 1.0233717 |
| CKS2 | NM_001827 | 0.1987867 | 0.00813 | 2.7118365 | -2.60807 | 1.0008963 |
| ATP1A2 | NM_000702 | 0.1093261 | 0.00179 | 3.2265139 | -1.32353 | 0.9968301 |
| CD177 | NM_020406 | 0.3580951 | 0.0422 | 2.0638168 | -3.95749 | 0.996717 |
| SPTSSA | AL080066 | 0.0734366 | 0.000549 | 3.5951445 | -0.30032 | 0.9933005 |
| SLC25A5 | NM_001152 | 0.1071625 | 0.00169 | 3.245167 | -1.27373 | 0.9918489 |
| PRNP | NM_000311 | 0.1049394 | 0.00153 | 3.2767115 | -1.18903 | 0.9914482 |
| SERPINB2 | NM_002575 | 0.2656183 | 0.0187 | 2.3979518 | -3.30109 | 0.9877164 |
| RETN | NM_020415 | 0.0889842 | 0.000907 | 3.4419295 | -0.73543 | 0.9848369 |
| HDDC2 | NM_016063 | 0.1765189 | 0.00621 | 2.8084823 | -2.38048 | 0.9844895 |
| TNNI3 | NM_000363 | 0.066993 | 0.000371 | 3.7123553 | 0.04157 | 0.9837287 |
| SEC11A | NM_014300 | 0.2620958 | 0.0174 | 2.4263072 | -3.23025 | 0.9776485 |
| TYR | NM_000372 | 0.0834675 | 0.000763 | 3.5023272 | -0.57941 | 0.9713423 |
| MRPL19 | NM_014763 | 0.1275193 | 0.00259 | 3.1092448 | -1.62997 | 0.9666471 |
| HSP90B1 | D16892 | 0.178177 | 0.00638 | 2.7993585 | -2.4029 | 0.9611808 |
| ANXA2 | NM_004039 | 0.2701844 | 0.0194 | 2.3850743 | -3.32752 | 0.9567674 |
| NR5A1 | NM_004959 | 0.0266909 | 5.58E-05 | 4.2495659 | 1.70106 | 0.9557852 |
| EID1 | NM_014335 | 0.0309222 | 8.45E-05 | 4.1352669 | 1.33595 | 0.9543637 |
| DROSHA | NM_013235 | 0.0277983 | 6.27E-05 | 4.2204176 | 1.59882 | 0.952202 |
| LGALS1 | NM_002305 | 0.2026998 | 0.00854 | 2.6939885 | -2.64938 | 0.9513676 |
| OIT3 | AF075085 | 0.066993 | 0.000386 | 3.7004476 | 0.00648 | 0.9491938 |
| RSL24D1 | NM_016304 | 0.066993 | 0.000378 | 3.7068913 | 0.02546 | 0.9465316 |
| CPSF3 | NM_016207 | 0.0436668 | 0.000161 | 3.9581144 | 0.76938 | 0.939408 |
| ECI2 | NM_006117 | 0.2091461 | 0.00919 | 2.6674866 | -2.71031 | 0.9389344 |
| HCFC1R1 | NM_017885 | 0.0918593 | 0.00107 | 3.3912186 | -0.87683 | 0.9378714 |
| SENP3-EIF4A1///SNORA67///EIF4A1 | D17188 | 0.1251516 | 0.00251 | 3.1174215 | -1.61038 | 0.9315159 |
| ANLN | NM_018685 | 0.1325955 | 0.00284 | 3.0764472 | -1.71615 | 0.9257292 |
| KIAA1143 | AB032969 | 0.029729 | 7.33E-05 | 4.1746827 | 1.46116 | 0.9247784 |
| IGFBP3 | NM_000598 | 0.3085383 | 0.0279 | 2.2387912 | -3.62377 | 0.9245579 |
| ATOX1 | NM_004045 | 0.0309222 | 8.02E-05 | 4.1496449 | 1.38154 | 0.918901 |
| MTX2 | NM_006554 | 0.026394 | 5.37E-05 | 4.2654347 | 1.73417 | 0.9168143 |
| PSMA5 | NM_002790 | 0.1016932 | 0.00139 | 3.3071899 | -1.1066 | 0.9156427 |
| TIMM44 | NM_006351 | 0.1301758 | 0.00273 | 3.0898067 | -1.68178 | 0.9073981 |
| YWHAG | NM_012479 | 0.1144986 | 0.00201 | 3.1898998 | -1.42065 | 0.9071492 |
| NDUFAF4 | NM_014165 | 0.3111067 | 0.0291 | 2.2217196 | -3.64503 | 0.9055379 |
| UPF3A | AK001069 | 0.0153369 | 2.21E-05 | 4.4981636 | 2.51572 | 0.9031878 |
| VRK1 | NM_003384 | 0.0196561 | 3.37E-05 | 4.3859265 | 2.14453 | 0.8966576 |
| HSP90AA1 | X15183 | 0.1796562 | 0.00656 | 2.7885667 | -2.42791 | 0.8963437 |
| FKBP4 | NM_002014 | 0.1071625 | 0.00164 | 3.255293 | -1.24661 | 0.8914229 |
| BRIX1 | NM_018321 | 0.2193009 | 0.011 | 2.6028183 | -2.85687 | 0.8906816 |
| SPARC | NM_003118 | 0.3280313 | 0.0345 | 2.1494275 | -3.79757 | 0.89066 |
| PEBP1 | NM_002567 | 0.2794145 | 0.0212 | 2.3494543 | -3.40168 | 0.8897261 |
| MIR100HG | AK021552 | 0.1571156 | 0.00459 | 2.9148525 | -2.11656 | 0.8895161 |
| DHCR24 | AF261758 | 0.1734578 | 0.00589 | 2.8295621 | -2.32477 | 0.8893673 |
| FKBP2 | NM_004470 | 0.1071625 | 0.00167 | 3.250951 | -1.26065 | 0.8870258 |
| APOH | NM_000042 | 0.151322 | 0.00418 | 2.945945 | -2.0457 | 0.8864387 |
| ITIH2 | NM_002216 | 0.1997513 | 0.00829 | 2.7047399 | -2.62452 | 0.8855256 |
| ATP5A1 | NM_004046 | 0.1478914 | 0.00398 | 2.9628491 | -2.00365 | 0.8846747 |
| BCAT1 | AK025615 | 0.1127896 | 0.00193 | 3.2023988 | -1.38759 | 0.8841384 |
| SEC61G | NM_014302 | 0.1443034 | 0.00374 | 2.9835491 | -1.9519 | 0.8835939 |
| NUP133 | NM_018230 | 0.0357556 | 0.000116 | 4.0473712 | 1.05945 | 0.883277 |
| SNORD46 | X96646 | 0.1024046 | 0.00142 | 3.30228 | -1.11992 | 0.8804484 |
| PRDX1 | NM_002574 | 0.3193544 | 0.0314 | 2.1886935 | -3.7223 | 0.8797066 |
| BORA | AK026277 | 0.0080989 | 2.17E-06 | 5.0980725 | 4.56746 | 0.878754 |
| TBC1D22B | AK021840 | 0.0908794 | 0.000967 | 3.4221113 | -0.7907 | 0.8782404 |
| LOC642852 | AL390181 | 0.0516526 | 0.00021 | 3.8777434 | 0.53674 | 0.8780366 |
| ANXA2P1 | M62896 | 0.1947265 | 0.00767 | 2.7330574 | -2.55866 | 0.8774933 |
| FGF2 | NM_002006 | 0.0317356 | 8.99E-05 | 4.1181841 | 1.28192 | 0.8772812 |
| GUSB | NM_000181 | 0.0114923 | 1.29E-05 | 4.6391662 | 2.98959 | 0.8761788 |
| ZNF205 | NM_003456 | 0.2069105 | 0.00891 | 2.6793907 | -2.68329 | 0.8726253 |
| MRPL15 | NM_014175 | 0.1093261 | 0.0018 | 3.2258936 | -1.32518 | 0.8702678 |
| TNFRSF11B | NM_002546 | 0.066993 | 0.000397 | 3.6924526 | -0.01703 | 0.869589 |
| CDKN2C | NM_001262 | 0.0544513 | 0.000234 | 3.8464333 | 0.44188 | 0.8678359 |
| PRSS2 | NM_002770 | 0.2069707 | 0.009 | 2.6752291 | -2.69256 | 0.8670479 |
| ABLIM3 | NM_014945 | 0.0544513 | 0.000228 | 3.856048 | 0.46515 | 0.8626632 |
| NDUFS4 | NM_002495 | 0.1514416 | 0.0042 | 2.9445086 | -2.04926 | 0.8579021 |
| ARID1B | Y08266 | 0.2213313 | 0.0113 | 2.5915133 | -2.88219 | 0.8570066 |
| PAK4 | NM_005884 | 0.0566565 | 0.00026 | 3.8185571 | 0.3523 | 0.8534227 |
| EXOSC4 | NM_019037 | 0.0730455 | 0.000518 | 3.6126156 | -0.24985 | 0.8525263 |
| SMO | NM_005631 | 0.019989 | 3.84E-05 | 4.350625 | 2.02892 | 0.8518648 |
| PSMB6 | D29012 | 0.1230069 | 0.0024 | 3.1320378 | -1.57239 | 0.8511854 |
| FAM105A | AF052146 | 0.0317356 | 9.23E-05 | 4.1107349 | 1.2584 | 0.8507593 |
| SEPP1 | NM_005410 | 0.3224138 | 0.0327 | 2.1717048 | -3.75501 | 0.8499455 |
| MRPL27 | NM_016504 | 0.1723798 | 0.00574 | 2.8361633 | -2.31411 | 0.8495743 |
| PROS1 | NM_000313 | 0.0676754 | 0.000421 | 3.6750944 | -0.06795 | 0.8461894 |
| KLHL35 | AL050370 | 0.0879607 | 0.000868 | 3.4556839 | -0.69693 | 0.8452952 |
| PCNA | NM_002592 | 0.2435112 | 0.0145 | 2.4989675 | -3.08573 | 0.8421154 |
| SERPINB7 | NM_003784 | 0.1071625 | 0.00168 | 3.2480262 | -1.26608 | 0.8419141 |
| MYO19 | AK026518 | 0.0910034 | 0.001 | 3.4103208 | -0.82348 | 0.8407912 |
| PSMD1 | NM_002807 | 0.0695964 | 0.000444 | 3.6591636 | -0.11454 | 0.8402897 |
| ABRACL | AF116682 | 0.0695964 | 0.00044 | 3.6616891 | -0.10716 | 0.8399628 |
| SERBP1 | NM_015640 | 0.066993 | 0.00036 | 3.7209091 | 0.06681 | 0.8384954 |
| MRPL36 | AF151109 | 0.1405463 | 0.0034 | 3.0197067 | -1.85929 | 0.8369713 |
| KRT19 | NM_002276 | 0.3746676 | 0.0477 | 2.0106993 | -4.03704 | 0.8366514 |
| MFSD10 | NM_001120 | 0.0842718 | 0.000781 | 3.4881505 | -0.6056 | 0.8363552 |
| IGFBP4 | M62403 | 0.3311124 | 0.035 | 2.1430831 | -3.80962 | 0.8358957 |
| PPIH | NM_006347 | 0.1178713 | 0.00219 | 3.1611877 | -1.49621 | 0.8354662 |
| MAP1B | NM_005909 | 0.3043244 | 0.0266 | 2.2581837 | -3.58614 | 0.834937 |
| CYCS | D00265 | 0.2620958 | 0.0175 | 2.4240071 | -3.24631 | 0.8332615 |
| SLC39A8 | AL049963 | 0.0803722 | 0.000684 | 3.5285833 | -0.49101 | 0.8325869 |
| MRPL3 | NM_007208 | 0.1753086 | 0.00599 | 2.8211637 | -2.35014 | 0.831331 |
| SORT1 | AK000757 | 0.1753086 | 0.00602 | 2.8194331 | -2.35429 | 0.831317 |
| ATP5C1 | NM_005174 | 0.0578532 | 0.000273 | 3.8024875 | 0.30961 | 0.830807 |
| UQCRB | NM_006294 | 0.1071625 | 0.00167 | 3.2504765 | -1.25952 | 0.8300824 |
| MRPL32 | AF161401 | 0.209542 | 0.00942 | 2.6591707 | -2.72955 | 0.8293065 |
| CMAS | NM_018686 | 0.1093081 | 0.00176 | 3.2349805 | -1.30566 | 0.8289245 |
| CADM1 | NM_014333 | 0.0966792 | 0.00121 | 3.3519188 | -0.9846 | 0.8269849 |
| LOC115110 | AL359943 | 0.0788364 | 0.000655 | 3.5420852 | -0.45254 | 0.8268339 |
| BLOC1S1 | NM_001487 | 0.0869908 | 0.000839 | 3.4661825 | -0.66746 | 0.8266105 |
| FBXO7 | NM_012179 | 0.0730455 | 0.000532 | 3.6047028 | -0.27273 | 0.8246854 |
| ITM2A | NM_004867 | 0.2396323 | 0.014 | 2.5123069 | -3.04651 | 0.8242429 |
| CACNA1E | NM_000721 | 0.2743887 | 0.0203 | 2.3662256 | -3.36709 | 0.8237541 |
| MRPL33 | NM_004891 | 0.0730455 | 0.000503 | 3.6216389 | -0.22371 | 0.8226205 |
| CALD1 | AK021691 | 0.1885217 | 0.00722 | 2.754594 | -2.50819 | 0.821986 |
| EEF1A2 | NM_001958 | 0.1159976 | 0.00208 | 3.1788851 | -1.4497 | 0.8217171 |
| UQCRC2 | NM_003366 | 0.1045557 | 0.00151 | 3.282592 | -1.17317 | 0.8211882 |
| GTPBP4 | NM_012341 | 0.0730455 | 0.000523 | 3.6100031 | -0.25741 | 0.8203218 |
| PSMA6 | X59417 | 0.1348737 | 0.00298 | 3.0604968 | -1.75703 | 0.8201115 |
| KRT18 | L32537 | 0.2942233 | 0.024 | 2.3000545 | -3.50231 | 0.8196941 |
| ACYP1 | NM_001107 | 0.1178713 | 0.0022 | 3.1623258 | -1.49728 | 0.8185786 |
| SNRPC | NM_003093 | 0.2689628 | 0.0192 | 2.3882553 | -3.32134 | 0.8153464 |
| MDH1 | NM_005917 | 0.1443034 | 0.00372 | 2.9853772 | -1.94731 | 0.8151316 |
| CYB5R3 | NM_007326 | 0.1325955 | 0.00284 | 3.0760231 | -1.71724 | 0.8146219 |
| NDUFS6 | NM_004553 | 0.1753086 | 0.00607 | 2.816008 | -2.36249 | 0.8135682 |
| CST7 | NM_003650 | 0.3115609 | 0.0292 | 2.2189181 | -3.66354 | 0.8130664 |
| LDHA | NM_005566 | 0.2267728 | 0.0119 | 2.5712341 | -2.92736 | 0.8106125 |
| ZBTB32 | NM_014383 | 0.1716402 | 0.00564 | 2.8419931 | -2.30006 | 0.8072096 |
| YWHAQ | NM_006826 | 0.2070132 | 0.00903 | 2.673912 | -2.69558 | 0.8064259 |
| TFPI2 | NM_006528 | 0.2879379 | 0.0231 | 2.3148372 | -3.4718 | 0.8059656 |
| MRPS18C | NM_016067 | 0.1753086 | 0.00603 | 2.8183332 | -2.35692 | 0.8055522 |
| AKR1C1 | NM_001353 | 0.2451134 | 0.0148 | 2.4888344 | -3.10763 | 0.8055435 |
| EBNA1BP2 | NM_006824 | 0.0834675 | 0.000755 | 3.4985195 | -0.57631 | 0.8051443 |
| PRKXP1 | Y10483 | 0.0601794 | 0.00029 | 3.784439 | 0.25558 | 0.8046366 |
| RPS7 | NM_001011 | 0.0966792 | 0.00118 | 3.3591916 | -0.96465 | 0.8026926 |
| HSPA4 | AB023420 | 0.1071625 | 0.00163 | 3.2570853 | -1.2418 | 0.8006347 |
| MLANA | NM_005511 | 0.1920931 | 0.0075 | 2.7409287 | -2.54025 | 0.8002016 |
| LIPF | NM_004190 | 0.0746481 | 0.000597 | 3.5700521 | -0.37251 | 0.7986324 |
| PHF10 | AL133094 | 0.1244795 | 0.00248 | 3.1208657 | -1.60144 | 0.7983879 |
| UMPS | NM_000373 | 0.1401609 | 0.00337 | 3.0208349 | -1.86085 | 0.7971968 |
| MAST4 | AB002301 | 0.1168911 | 0.00212 | 3.1729987 | -1.46519 | 0.7970649 |
| EEFSEC | AF268872 | 0.2426145 | 0.0143 | 2.5027833 | -3.07764 | 0.7960924 |
| XPOT | NM_007235 | 0.1136814 | 0.00196 | 3.1988339 | -1.39703 | 0.7943848 |
| DAB2 | NM_001343 | 0.2310131 | 0.0129 | 2.5429382 | -2.9899 | 0.7942492 |
| IFT27 | NM_006860 | 0.0966792 | 0.00119 | 3.3583669 | -0.96691 | 0.7942484 |
| PERP | AF317550 | 0.2226083 | 0.0114 | 2.5875828 | -2.89097 | 0.7941399 |
| GYG1 | NM_004130 | 0.2720497 | 0.0198 | 2.3760377 | -3.34676 | 0.7941211 |
| PSMB5 | NM_002797 | 0.31075 | 0.0287 | 2.2270633 | -3.64758 | 0.7937916 |
| SNRPD2 | NM_004597 | 0.3507537 | 0.0401 | 2.085815 | -3.91695 | 0.7932805 |
| FAM86C1 | NM_018172 | 0.1072342 | 0.0017 | 3.2465614 | -1.27481 | 0.7910825 |
| ZCCHC17 | NM_016505 | 0.0894504 | 0.000925 | 3.436015 | -0.75195 | 0.7907923 |
| CTSL | NM_001912 | 0.1373817 | 0.00319 | 3.0388106 | -1.81384 | 0.7901277 |
| ACAT2 | NM_005891 | 0.1178713 | 0.00219 | 3.1617266 | -1.4948 | 0.790015 |
| RUVBL2 | NM_006666 | 0.1509582 | 0.00413 | 2.9528812 | -2.03208 | 0.7890459 |
| GHR | Z11849 | 0.0803722 | 0.000685 | 3.5284552 | -0.49138 | 0.7887667 |
| PATZ1 | AF254084 | 0.269815 | 0.0193 | 2.3862452 | -3.32553 | 0.7885328 |
| DPP4 | NM_001935 | 0.0811766 | 0.000714 | 3.5156154 | -0.52787 | 0.7882825 |
| SNORA68 | Y11162 | 0.2145573 | 0.0101 | 2.6344063 | -2.78566 | 0.7882601 |
| PAK1IP1 | NM_017906 | 0.2052959 | 0.00879 | 2.6845204 | -2.67151 | 0.7881837 |
| GADD45A | NM_001924 | 0.1287193 | 0.00267 | 3.0968388 | -1.66365 | 0.7871954 |
| UQCC2 | AF086234 | 0.3385119 | 0.0372 | 2.1180067 | -3.85694 | 0.786077 |
| TFDP2 | NM_006286 | 0.1753086 | 0.0061 | 2.8142576 | -2.36668 | 0.7832417 |
| HK2 | AF086198 | 0.1301758 | 0.00273 | 3.0898171 | -1.68175 | 0.7828825 |
| ATP5I | NM_007100 | 0.1365237 | 0.00313 | 3.0438729 | -1.79946 | 0.7827233 |
| KIF1BP | AB033105 | 0.0974657 | 0.00131 | 3.327458 | -1.05147 | 0.7826359 |
| NDUFA6 | NM_002490 | 0.16832 | 0.00537 | 2.8593309 | -2.25815 | 0.7822517 |
| GCSH | NM_004483 | 0.2727714 | 0.02 | 2.3744691 | -3.34802 | 0.7816985 |
| APOA1 | NM_000039 | 0.3125149 | 0.0297 | 2.2119937 | -3.67706 | 0.7795108 |
| PDIA5 | NM_006810 | 0.1061802 | 0.00157 | 3.269961 | -1.20721 | 0.779507 |
| MCFD2 | M23161 | 0.1428571 | 0.00362 | 2.9957162 | -1.92268 | 0.7787506 |
| LMAN1 | NM_005570 | 0.2446803 | 0.0147 | 2.4925641 | -3.09958 | 0.777729 |
| CDK1 | NM_001786 | 0.3774739 | 0.0496 | 1.9924064 | -4.08642 | 0.7748197 |
| FOXK1 | AK022628 | 0.1127896 | 0.00193 | 3.2033374 | -1.3851 | 0.7735834 |
| C14orf2 | NM_004894 | 0.0915477 | 0.00104 | 3.3995201 | -0.85343 | 0.771719 |
| FKBP5 | NM_004117 | 0.2743887 | 0.0203 | 2.3661087 | -3.36733 | 0.7715237 |
| MB | NM_005368 | 0.2498927 | 0.0156 | 2.4704021 | -3.14701 | 0.7706039 |
| NQO1 | NM_000903 | 0.3283045 | 0.0346 | 2.1485044 | -3.79933 | 0.7686973 |
| MRPS10 | NM_018141 | 0.2267728 | 0.0119 | 2.5719433 | -2.92579 | 0.7682524 |
| RBMS3 | NM_014483 | 0.1042374 | 0.00149 | 3.2871402 | -1.16089 | 0.7682241 |
| NDUFA2 | NM_002488 | 0.0811766 | 0.000711 | 3.5169836 | -0.52399 | 0.76798 |
| IARS2 | NM_018060 | 0.0566565 | 0.000264 | 3.8161059 | 0.33928 | 0.7658253 |
| COX7B | NM_001866 | 0.1997513 | 0.00829 | 2.7047633 | -2.62447 | 0.7654389 |
| VEGFB | NM_003377 | 0.1049394 | 0.00152 | 3.2800247 | -1.18009 | 0.7649642 |
| MYO10 | AF234532 | 0.0153369 | 1.99E-05 | 4.5261296 | 2.60905 | 0.7643358 |
| SNORA64 | Y11158 | 0.3164131 | 0.0307 | 2.1987095 | -3.7029 | 0.7617646 |
| ARV1 | AL122047 | 0.0915477 | 0.00105 | 3.3953983 | -0.86484 | 0.7604246 |
| ZNF22 | NM_006963 | 0.2267728 | 0.0119 | 2.5711179 | -2.92762 | 0.7594986 |
| ALCAM | NM_001627 | 0.1408906 | 0.00345 | 3.0112279 | -1.88225 | 0.7587949 |
| SHFM1 | NM_006304 | 0.3045913 | 0.0266 | 2.2570738 | -3.58835 | 0.75861 |
| NDUFB8 | NM_005004 | 0.1159976 | 0.00209 | 3.1764652 | -1.45607 | 0.7549562 |
| TOP2A | NM_001067 | 0.3086243 | 0.028 | 2.2368875 | -3.62752 | 0.7547906 |
| RPS23 | NM_001025 | 0.3125149 | 0.0297 | 2.2118755 | -3.67729 | 0.7542571 |
| AKR1A1 | NM_006066 | 0.1517071 | 0.00423 | 2.9420307 | -2.05541 | 0.7523686 |
| NDUFA4L2 | NM_020142 | 0.1424297 | 0.00356 | 3.0038673 | -1.90501 | 0.7520022 |
| SNRPD1 | NM_006938 | 0.2946149 | 0.024 | 2.2989377 | -3.50456 | 0.7519324 |
| NDUFA4 | NM_002489 | 0.3038871 | 0.0261 | 2.2658448 | -3.57091 | 0.751832 |
| IKBKE | NM_014002 | 0.0924214 | 0.0011 | 3.3833498 | -0.89814 | 0.7502205 |
| CCT3 | NM_005998 | 0.2272024 | 0.0122 | 2.5620887 | -2.94764 | 0.7501029 |
| POLR2G | NM_002696 | 0.0966792 | 0.00127 | 3.3359456 | -1.02831 | 0.7488488 |
| MET | AK025784 | 0.1024046 | 0.00142 | 3.3010291 | -1.12331 | 0.7483116 |
| NDUFA13 | NM_015965 | 0.240531 | 0.0141 | 2.5078083 | -3.06672 | 0.7475781 |
| HPGD | NM_000860 | 0.3029261 | 0.0257 | 2.2721693 | -3.55829 | 0.7475413 |
| CCDC53 | NM_016053 | 0.0309222 | 8.38E-05 | 4.1375801 | 1.34328 | 0.7473902 |
| PTPN1 | NM_002827 | 0.1094396 | 0.00183 | 3.2203445 | -1.33996 | 0.7466576 |
| SUPT3H | NM_003599 | 0.0656815 | 0.000338 | 3.7398874 | 0.12298 | 0.7464522 |
| HEXB | NM_000521 | 0.2114224 | 0.00973 | 2.6464929 | -2.75822 | 0.7464413 |
| NARS2 | AK027094 | 0.0858696 | 0.000802 | 3.4802263 | -0.62795 | 0.7456931 |
| SERPINB6 | NM_004568 | 0.1723798 | 0.00574 | 2.8356535 | -2.31533 | 0.7451226 |
| AIMP1 | NM_004757 | 0.0910034 | 0.000988 | 3.4155386 | -0.80899 | 0.7442201 |
| ANKRD36 | AB046861 | 0.2065213 | 0.00887 | 2.6804364 | -2.6806 | 0.7432961 |
| SERPINF1 | NM_002615 | 0.3010319 | 0.0251 | 2.2823855 | -3.53719 | 0.7427029 |
| ZNF32 | U69645 | 0.0452843 | 0.000177 | 3.9270197 | 0.68708 | 0.7421583 |
| CHD6 | AK026022 | 0.1121916 | 0.00191 | 3.2061524 | -1.37764 | 0.74191 |
| FOXD1 | NM_004472 | 0.1716402 | 0.00564 | 2.8429535 | -2.29854 | 0.7418422 |
| CSRP2 | NM_001321 | 0.1504983 | 0.0041 | 2.9527547 | -2.02878 | 0.740935 |
| EIF2S2 | NM_003908 | 0.1087525 | 0.00174 | 3.2369862 | -1.2956 | 0.7405145 |
| PHLDA1 | NM_007350 | 0.1071625 | 0.00169 | 3.2465686 | -1.27236 | 0.7392771 |
| MRM2 | NM_013393 | 0.136725 | 0.00316 | 3.0408574 | -1.80713 | 0.7392352 |
| REXO2 | NM_015523 | 0.2763247 | 0.0207 | 2.359442 | -3.3806 | 0.738007 |
| ZNF133 | NM_003434 | 0.2226083 | 0.0114 | 2.5870262 | -2.89221 | 0.7369085 |
| SNRPA1 | NM_003090 | 0.2594182 | 0.017 | 2.4357777 | -3.2214 | 0.7346303 |
| CYP27B1 | NM_000785 | 0.2170877 | 0.0107 | 2.6122204 | -2.83585 | 0.734596 |
| FAM81A | AL110257 | 0.1548857 | 0.00446 | 2.9238729 | -2.10032 | 0.7345715 |
| CHML | AK000933 | 0.1478914 | 0.00398 | 2.9623505 | -2.00489 | 0.7325302 |
| SLC25A3 | NM_002635 | 0.2545873 | 0.0164 | 2.450516 | -3.19007 | 0.7318794 |
| SMC3 | NM_005445 | 0.1525419 | 0.00432 | 2.934916 | -2.07303 | 0.7318445 |
| NAA20 | NM_016100 | 0.0737062 | 0.000573 | 3.5824803 | -0.3368 | 0.730922 |
| TPX2 | AB024704 | 0.1794227 | 0.00652 | 2.7919084 | -2.42061 | 0.7299166 |
| OR7E24 | Y10529 | 0.2543051 | 0.0162 | 2.4548299 | -3.18086 | 0.728461 |
| SEMA3F | NM_004186 | 0.1719083 | 0.00567 | 2.8409398 | -2.30339 | 0.7282633 |
| RHOBTB3 | NM_014899 | 0.2701297 | 0.0193 | 2.3854648 | -3.32716 | 0.7281129 |
| MRPL40 | NM_003776 | 0.2069707 | 0.009 | 2.6748844 | -2.69335 | 0.7273682 |
| FXR1 | NM_005087 | 0.1509582 | 0.00413 | 2.9500452 | -2.03552 | 0.725326 |
| PSMA4 | NM_002789 | 0.1684102 | 0.00543 | 2.8552545 | -2.26802 | 0.7232355 |
| TCEAL9 | NM_016303 | 0.2369994 | 0.0136 | 2.5211551 | -3.03764 | 0.7224932 |
| CRLS1 | NM_019095 | 0.0730455 | 0.000524 | 3.6092623 | -0.25955 | 0.7211794 |
| ERICH1 | AF161437 | 0.1093261 | 0.00178 | 3.2299741 | -1.31431 | 0.7211026 |
| FAM98A | AL049943 | 0.0966792 | 0.00126 | 3.3393334 | -1.01905 | 0.7206629 |
| NDUFB2 | NM_004546 | 0.1609168 | 0.00476 | 2.9010834 | -2.15637 | 0.7206211 |
| PARD3 | NM_019619 | 0.2026998 | 0.00857 | 2.6927292 | -2.65229 | 0.7200159 |
| MFSD12 | AF218008 | 0.2545873 | 0.0164 | 2.4509051 | -3.18924 | 0.7192281 |
| UTP18 | NM_016001 | 0.0975587 | 0.00132 | 3.3253356 | -1.05725 | 0.7187819 |
| BBOX1 | NM_003986 | 0.3434435 | 0.0382 | 2.1075721 | -3.86167 | 0.7154367 |
| SSR4 | NM_006280 | 0.2170877 | 0.0106 | 2.6151407 | -2.82918 | 0.7153536 |
| PTPN11 | D13540 | 0.1016932 | 0.00139 | 3.3082712 | -1.10366 | 0.7151456 |
| TSC22D1 | NM_006022 | 0.1600943 | 0.00469 | 2.9065607 | -2.14293 | 0.7132723 |
| ERH | NM_004450 | 0.1334554 | 0.00289 | 3.0701742 | -1.73225 | 0.7131365 |
| MIS18A | NM_018944 | 0.178177 | 0.00641 | 2.7986533 | -2.40524 | 0.7110298 |
| TRMT112 | NM_016404 | 0.199617 | 0.00825 | 2.7065087 | -2.62042 | 0.7108841 |
| CATX-1 | AF083117 | 0.3479607 | 0.0396 | 2.096127 | -3.87339 | 0.7100029 |
| MACF1 | NM_012090 | 0.1159976 | 0.00209 | 3.1772402 | -1.45403 | 0.7096289 |
| KLRK1 | NM_007360 | 0.0889842 | 0.000904 | 3.4447542 | -0.73087 | 0.709289 |
| POLR3K | NM_016310 | 0.2004149 | 0.00833 | 2.7030717 | -2.62838 | 0.7092112 |
| OXCT1 | NM_000436 | 0.1571156 | 0.00458 | 2.9176416 | -2.1189 | 0.7090618 |
| CFAP97 | AB037851 | 0.0834675 | 0.000749 | 3.5010085 | -0.56927 | 0.7084092 |
| MEA1 | NM_014623 | 0.1277833 | 0.00261 | 3.1044298 | -1.64403 | 0.7068724 |
| LOC642533 | AF086148 | 0.1728702 | 0.00581 | 2.8314337 | -2.32548 | 0.7065344 |
| USMG5 | AL110185 | 0.2720497 | 0.0199 | 2.3743224 | -3.35032 | 0.706333 |
| CARMIL1 | Y10210 | 0.2443825 | 0.0146 | 2.4986125 | -3.07583 | 0.7052288 |
| ARL3 | NM_004311 | 0.0730455 | 0.000488 | 3.631048 | -0.19641 | 0.7052002 |
| ZNF677 | AK026366 | 0.199617 | 0.00826 | 2.7061016 | -2.62137 | 0.7030916 |
| UACA | AB046781 | 0.2092626 | 0.00922 | 2.6663925 | -2.71281 | 0.7027181 |
| EMC2 | NM_014673 | 0.1260689 | 0.00253 | 3.1141313 | -1.61891 | 0.7022981 |
| ADARB2-AS1 | AF086274 | 0.2380542 | 0.0138 | 2.5164826 | -3.04784 | 0.7014385 |
| CRYBB1 | NM_001887 | 0.1071625 | 0.00162 | 3.2601657 | -1.23354 | 0.7014173 |
| GPR84 | NM_020370 | 0.2650109 | 0.0186 | 2.4007324 | -3.29527 | 0.7005526 |
| GPNMB | NM_002510 | 0.2643568 | 0.0181 | 2.4111847 | -3.27333 | 0.6991452 |
| TTC9 | D86980 | 0.1071625 | 0.00166 | 3.2519785 | -1.2555 | 0.6981605 |
| GMNN | NM_015895 | 0.3197006 | 0.032 | 2.1818298 | -3.73554 | 0.6978704 |
| PSMC2 | NM_002803 | 0.1723798 | 0.00574 | 2.8357892 | -2.31501 | 0.6970418 |
| RPS6 | NM_001010 | 0.1631182 | 0.00494 | 2.8885601 | -2.18702 | 0.6964861 |
| ZC2HC1A | NM_016010 | 0.094966 | 0.00115 | 3.369297 | -0.93687 | 0.6962266 |
| RPL22 | AF113701 | 0.313933 | 0.0301 | 2.2077486 | -3.68371 | 0.69618 |
| MRPS28 | NM_014018 | 0.1159976 | 0.00208 | 3.1794744 | -1.44814 | 0.6959721 |
| CD3D | NM_000732 | 0.3246594 | 0.0333 | 2.1649049 | -3.76805 | 0.6956756 |
| PBDC1 | NM_016500 | 0.0730455 | 0.000505 | 3.6207263 | -0.22636 | 0.6950887 |
| POLR2H | U37689 | 0.2647025 | 0.0184 | 2.4066061 | -3.27096 | 0.6945067 |
| RRBP1 | NM_004587 | 0.1239768 | 0.00243 | 3.12784 | -1.58331 | 0.6944123 |
| LOXL1 | NM_005576 | 0.2925605 | 0.0238 | 2.3037194 | -3.48259 | 0.6942035 |
| GINS2 | NM_016095 | 0.1525928 | 0.00432 | 2.9341524 | -2.07492 | 0.69386 |
| STOML2 | NM_013442 | 0.243156 | 0.0144 | 2.5004926 | -3.08261 | 0.6937093 |
| ZNF195 | NM_007152 | 0.1239768 | 0.00244 | 3.1273409 | -1.58647 | 0.6928235 |
| RABGGTB | NM_004582 | 0.196827 | 0.00791 | 2.7219183 | -2.58463 | 0.6927579 |
| PTTG3P | AF095289 | 0.3010319 | 0.0251 | 2.2814272 | -3.53977 | 0.6887464 |
| COX5B | NM_001862 | 0.1476893 | 0.00392 | 2.9680248 | -1.99074 | 0.6887335 |
| PTPRB | NM_002837 | 0.1287193 | 0.00266 | 3.0998373 | -1.65943 | 0.688547 |
| MRPL39 | NM_017446 | 0.2763447 | 0.0207 | 2.3591919 | -3.3806 | 0.6881084 |
| MATR3 | AJ224166 | 0.1753086 | 0.00608 | 2.8164649 | -2.35426 | 0.6879647 |
| LSM8 | NM_016200 | 0.1515459 | 0.00421 | 2.9430541 | -2.05287 | 0.6860294 |
| LOC101927253 | AK000454 | 0.3110118 | 0.029 | 2.2227989 | -3.65517 | 0.6858725 |
| TSPAN3 | NM_005724 | 0.3773425 | 0.0494 | 1.9955261 | -4.07856 | 0.6858186 |
| IFI30 | NM_006332 | 0.2294155 | 0.0126 | 2.5492989 | -2.97589 | 0.6855566 |
| PCDH9 | NM_020403 | 0.1184091 | 0.00223 | 3.1576943 | -1.50735 | 0.6855476 |
| ZNF639 | NM_016331 | 0.16832 | 0.00539 | 2.8591175 | -2.25202 | 0.6854967 |
| RPL10 | NM_006013 | 0.2093914 | 0.00923 | 2.6656865 | -2.71443 | 0.6853881 |
| ODAM | NM_017855 | 0.2318019 | 0.013 | 2.5391271 | -2.99828 | 0.6843852 |
| UPP1 | NM_003364 | 0.1630642 | 0.00492 | 2.8897845 | -2.18403 | 0.6841544 |
| PSMD14 | NM_005805 | 0.3515263 | 0.0404 | 2.0825151 | -3.92305 | 0.6829249 |
| IPO5 | NM_002271 | 0.2285794 | 0.0124 | 2.5561143 | -2.96085 | 0.6828345 |
| PRSS1 | NM_002769 | 0.2759402 | 0.0206 | 2.3614467 | -3.36437 | 0.6823671 |
| QARS | NM_005051 | 0.3085383 | 0.0277 | 2.2404427 | -3.62126 | 0.6815869 |
| SERPINA3 | NM_001085 | 0.1504442 | 0.00409 | 2.9535508 | -2.0268 | 0.681556 |
| ISY1-RAB43 | AB032986 | 0.1753086 | 0.00606 | 2.8170079 | -2.3601 | 0.6807978 |
| FRMPD1 | NM_014907 | 0.1408906 | 0.00346 | 3.0095963 | -1.88637 | 0.6803931 |
| SUCLG1 | AF104921 | 0.2809852 | 0.0219 | 2.3363118 | -3.42863 | 0.6800318 |
| VWA5A | NM_014622 | 0.0811766 | 0.000701 | 3.5246713 | -0.50857 | 0.6795103 |
| EEF1E1 | NM_004280 | 0.2520986 | 0.0158 | 2.4637267 | -3.16157 | 0.6781782 |
| HBS1L | NM_006620 | 0.0910034 | 0.001 | 3.411763 | -0.81948 | 0.6780775 |
| OR7E47P | X87825 | 0.226759 | 0.0118 | 2.5735499 | -2.92222 | 0.6780401 |
| ATP5O | NM_001697 | 0.3438323 | 0.0384 | 2.1045309 | -3.88216 | 0.6770803 |
| PRORY | AK026367 | 0.1408906 | 0.00346 | 3.0101055 | -1.88508 | 0.6764719 |
| ALDOC | NM_005165 | 0.216553 | 0.0104 | 2.6220199 | -2.81367 | 0.6758837 |
| TCEB2 | NM_007108 | 0.3194439 | 0.0317 | 2.1849342 | -3.72956 | 0.6753671 |
| MS4A4A | NM_016650 | 0.3634278 | 0.0445 | 2.0403967 | -4.00022 | 0.675336 |
| IDNK | AF086281 | 0.2942233 | 0.024 | 2.3003252 | -3.48941 | 0.6753279 |
| AHSA1 | NM_012111 | 0.2272024 | 0.0121 | 2.5641802 | -2.94301 | 0.6741248 |
| CDK6 | AK000660 | 0.1899878 | 0.00735 | 2.7483065 | -2.52295 | 0.6729847 |
| APOC3 | NM_000040 | 0.1844686 | 0.0069 | 2.7707591 | -2.47009 | 0.6724653 |
| ANKRD53 | AK022118 | 0.3773425 | 0.049 | 1.9980349 | -4.07527 | 0.6717467 |
| MANF | NM_006010 | 0.1723798 | 0.00574 | 2.8376865 | -2.31201 | 0.6716017 |
| POLD3 | D26018 | 0.119209 | 0.00228 | 3.1487052 | -1.5289 | 0.6702348 |
| RASSF8 | NM_007211 | 0.2136442 | 0.00994 | 2.6387935 | -2.77571 | 0.6682301 |
| ETF1 | NM_004730 | 0.2656183 | 0.0187 | 2.3982452 | -3.30047 | 0.6680517 |
| RPL27 | U25752 | 0.3626634 | 0.044 | 2.0450476 | -3.99177 | 0.6677699 |
| RPL18 | NM_000979 | 0.2643568 | 0.0182 | 2.4103228 | -3.27475 | 0.6669636 |
| TMOD2 | AK025346 | 0.209542 | 0.00928 | 2.6638359 | -2.71866 | 0.6665267 |
| NUTF2 | NM_005796 | 0.2367932 | 0.0135 | 2.5236148 | -3.03227 | 0.6651174 |
| PCCB | NM_000532 | 0.2532532 | 0.016 | 2.4593663 | -3.17117 | 0.6643351 |
| ID3 | X69111 | 0.3124692 | 0.0297 | 2.2141775 | -3.67036 | 0.6642335 |
| VMA21 | AK025798 | 0.1759761 | 0.00617 | 2.8105363 | -2.37558 | 0.6641276 |
| NUP210 | AB020713 | 0.1897029 | 0.00732 | 2.7497156 | -2.51965 | 0.6636172 |
| URI1 | NM_003796 | 0.1482191 | 0.00402 | 2.9603906 | -2.01098 | 0.6635899 |
| UQCR10 | NM_013387 | 0.1415865 | 0.00349 | 3.0071985 | -1.89242 | 0.6624527 |
| CEBPZOS | AK001814 | 0.0695964 | 0.000444 | 3.6592972 | -0.11415 | 0.6621962 |
| TCEA1 | NM_006756 | 0.1239768 | 0.00244 | 3.1258581 | -1.58847 | 0.6605379 |
| HS3ST3A1 | NM_006042 | 0.2621913 | 0.0175 | 2.4235531 | -3.24727 | 0.6603884 |
| CCT7 | NM_006429 | 0.3650662 | 0.0449 | 2.0367119 | -4.00691 | 0.6599573 |
| TOMM7 | NM_019059 | 0.2709425 | 0.0195 | 2.3819202 | -3.33453 | 0.658855 |
| PRSS50 | NM_013270 | 0.268249 | 0.0191 | 2.3900125 | -3.31767 | 0.6579471 |
| HOXD4 | X04706 | 0.2451134 | 0.0148 | 2.4891448 | -3.10717 | 0.6578254 |
| TPRKB | NM_016058 | 0.1973623 | 0.00795 | 2.7199782 | -2.58915 | 0.6575875 |
| GNG11 | NM_004126 | 0.2396323 | 0.014 | 2.5110808 | -3.05961 | 0.6570741 |
| SLC26A2 | NM_000112 | 0.1705821 | 0.00556 | 2.8472252 | -2.28743 | 0.6562412 |
| HAUS3 | AF040964 | 0.1928529 | 0.00756 | 2.7380022 | -2.5471 | 0.6562231 |
| PYCR2 | NM_013328 | 0.2110673 | 0.00966 | 2.649084 | -2.75232 | 0.6558292 |
| RAVER2 | NM_018211 | 0.1093261 | 0.00178 | 3.2293333 | -1.31602 | 0.6555516 |
| ZNF154 | U20648 | 0.2093914 | 0.00925 | 2.6673365 | -2.71142 | 0.6547789 |
| FGFR1 | AK001052 | 0.3038871 | 0.0259 | 2.2683665 | -3.56588 | 0.6547466 |
| UQCR11 | NM_006830 | 0.0966792 | 0.00122 | 3.3484915 | -0.99399 | 0.6547249 |
| ASPM | NM_018123 | 0.185185 | 0.00701 | 2.7659352 | -2.48203 | 0.6545873 |
| GAPDH | NM_002046 | 0.3206611 | 0.0324 | 2.1759831 | -3.7468 | 0.6540779 |
| PRDX3 | NM_006793 | 0.1478914 | 0.00397 | 2.9632675 | -2.00261 | 0.6540076 |
| NUDT1 | NM_002452 | 0.1671388 | 0.00524 | 2.8685939 | -2.22913 | 0.6529197 |
| CRTC3 | AK024981 | 0.1824064 | 0.00676 | 2.7779968 | -2.45298 | 0.6526194 |
| PRKCA | NM_002737 | 0.0566565 | 0.000263 | 3.813291 | 0.34203 | 0.6514927 |
| RPL22L1 | AL110170 | 0.2720497 | 0.0198 | 2.3764953 | -3.34581 | 0.6507676 |
| MRPS21 | AF182417 | 0.303108 | 0.0258 | 2.2702112 | -3.5622 | 0.6500117 |
| CYP19A1 | NM_000103 | 0.2720497 | 0.0198 | 2.3766659 | -3.34546 | 0.6499409 |
| PODXL | NM_005397 | 0.3748949 | 0.0479 | 2.0082302 | -4.05708 | 0.6498801 |
| COL6A3 | NM_004369 | 0.300855 | 0.0249 | 2.2848656 | -3.53288 | 0.6496743 |
| C12orf29 | AL049382 | 0.147805 | 0.00394 | 2.9657689 | -1.99637 | 0.6496016 |
| BRINP1 | NM_014618 | 0.0945914 | 0.00114 | 3.3767227 | -0.92563 | 0.6494703 |
| HIKESHI | NM_016411 | 0.1529701 | 0.00436 | 2.9314226 | -2.08167 | 0.6494531 |
| SGSH | NM_000199 | 0.0730455 | 0.000527 | 3.6076495 | -0.26421 | 0.6492849 |
| ITPR3 | NM_002224 | 0.2272024 | 0.0122 | 2.5620679 | -2.94768 | 0.6487099 |
| DCAF8 | AK026213 | 0.3628601 | 0.0441 | 2.0447027 | -3.99133 | 0.6478764 |
| VAT1 | NM_006373 | 0.2643568 | 0.0179 | 2.4149039 | -3.26551 | 0.6476311 |
| IBTK | AF235049 | 0.1482191 | 0.00402 | 2.9593123 | -2.01246 | 0.6473622 |
| UQCRQ | NM_014402 | 0.2974046 | 0.0244 | 2.2921866 | -3.51817 | 0.6464373 |
| MTDH | AK000745 | 0.1930182 | 0.00758 | 2.7372039 | -2.54896 | 0.6464346 |
| CTAG1B | NM_001327 | 0.2794145 | 0.0213 | 2.3481216 | -3.40442 | 0.6453026 |
| SMARCC2 | NM_003075 | 0.136725 | 0.00315 | 3.0426115 | -1.80418 | 0.6452934 |
| ENC1 | NM_003633 | 0.1635158 | 0.00496 | 2.887951 | -2.18946 | 0.6450003 |
| CAB39 | NM_016289 | 0.1061339 | 0.00156 | 3.2719268 | -1.20192 | 0.6435289 |
| CD63 | NM_001780 | 0.1686339 | 0.00545 | 2.8542109 | -2.27054 | 0.6430323 |
| MED7 | NM_004270 | 0.0746481 | 0.000604 | 3.5667381 | -0.38201 | 0.6417905 |
| TMEM230 | NM_014145 | 0.1525419 | 0.0043 | 2.9359027 | -2.07059 | 0.641336 |
| MPHOSPH10 | X98494 | 0.3182598 | 0.0311 | 2.1926245 | -3.7147 | 0.6412578 |
| ARL1 | NM_001177 | 0.2369994 | 0.0136 | 2.5228733 | -3.03362 | 0.6407521 |
| CHML | NM_001821 | 0.268249 | 0.0191 | 2.3906744 | -3.31586 | 0.6391358 |
| A2M | NM_000014 | 0.3774739 | 0.0497 | 1.9913784 | -4.08825 | 0.639089 |
| B4GAT1 | NM_006876 | 0.1393572 | 0.00329 | 3.0275199 | -1.84102 | 0.6386228 |
| HAUS1 | AK024747 | 0.1071625 | 0.00161 | 3.2620218 | -1.22855 | 0.6381529 |
| BLVRA | NM_000712 | 0.1678959 | 0.0053 | 2.864575 | -2.2463 | 0.6379769 |
| MMACHC | AL080062 | 0.1692126 | 0.00548 | 2.8533708 | -2.27341 | 0.6377907 |
| HIGD1A | NM_014056 | 0.2451134 | 0.0148 | 2.4880907 | -3.10945 | 0.6377639 |
| MGP | NM_000900 | 0.3124692 | 0.0295 | 2.2153478 | -3.67052 | 0.6375629 |
| PATZ1 | AF242522 | 0.1355343 | 0.00301 | 3.0564426 | -1.76739 | 0.6360842 |
| MYBBP1A | NM_014520 | 0.1408906 | 0.00343 | 3.0134405 | -1.87666 | 0.6346294 |
| HIF3A | AK021737 | 0.1648871 | 0.00503 | 2.88209 | -2.20281 | 0.6343102 |
| RPL19 | NM_000981 | 0.3626634 | 0.0437 | 2.0480433 | -3.98632 | 0.634208 |
| LBX1 | NM_006562 | 0.3182598 | 0.0311 | 2.1931409 | -3.7137 | 0.6341166 |
| POLR2K | NM_005034 | 0.1935489 | 0.00761 | 2.7365637 | -2.55092 | 0.6331398 |
| BBS7 | NM_018190 | 0.3043244 | 0.0264 | 2.260026 | -3.58248 | 0.6330443 |
| CDC37 | NM_007065 | 0.1624342 | 0.00488 | 2.8927736 | -2.17672 | 0.6319429 |
| CUL5 | NM_003478 | 0.147805 | 0.00394 | 2.9661522 | -1.99541 | 0.6317871 |
| CTTN | NM_005231 | 0.3626634 | 0.044 | 2.0456693 | -3.98957 | 0.6316811 |
| PNO1 | NM_020143 | 0.0746481 | 0.000604 | 3.566581 | -0.38246 | 0.6315964 |
| NDUFA1 | NM_004541 | 0.1424297 | 0.00355 | 3.0013409 | -1.90719 | 0.6312184 |
| ATP5F1 | NM_001688 | 0.2620958 | 0.0174 | 2.4257632 | -3.2426 | 0.6310851 |
| P4HA2 | NM_004199 | 0.1649028 | 0.00509 | 2.8779708 | -2.21286 | 0.6298889 |
| SIL1 | AJ299442 | 0.209542 | 0.0094 | 2.659214 | -2.72922 | 0.6281297 |
| NDUFA7 | NM_005001 | 0.1548069 | 0.00445 | 2.9246889 | -2.09831 | 0.6280415 |
| ELAC2 | NM_018127 | 0.2272024 | 0.0122 | 2.563383 | -2.94477 | 0.6273389 |
| PFKL | NM_002626 | 0.3029261 | 0.0256 | 2.2727938 | -3.55704 | 0.6268463 |
| OXA1L | NM_005015 | 0.2107941 | 0.00958 | 2.6520786 | -2.7455 | 0.6266397 |
| SNRPG | NM_003096 | 0.3194439 | 0.0315 | 2.1873272 | -3.72494 | 0.6262907 |
| GDA | NM_004293 | 0.2161336 | 0.0103 | 2.6240699 | -2.80904 | 0.6262412 |
| ZFHX3 | AL133108 | 0.2756699 | 0.0206 | 2.3650172 | -3.34409 | 0.6262234 |
| ZNHIT2 | NM_014205 | 0.2479891 | 0.0152 | 2.4794533 | -3.12784 | 0.625999 |
| SARS | NM_006513 | 0.2069707 | 0.00898 | 2.6759555 | -2.69089 | 0.6252732 |
| RPL39 | NM_001000 | 0.2144484 | 0.01 | 2.6353159 | -2.78359 | 0.624543 |
| FYN | NM_002037 | 0.0966792 | 0.00125 | 3.3411805 | -1.014 | 0.6240963 |
| PSMD6 | NM_014814 | 0.1087525 | 0.00175 | 3.2363446 | -1.29964 | 0.6237721 |
| PCBP4 | NM_020418 | 0.2015371 | 0.0084 | 2.7001151 | -2.63522 | 0.6232922 |
| SPOCK1 | NM_004598 | 0.3090079 | 0.0282 | 2.2339607 | -3.6325 | 0.6230878 |
| RIN3 | AK021762 | 0.2266344 | 0.0118 | 2.5756165 | -2.91762 | 0.6229749 |
| FAM118B | AK024756 | 0.1853789 | 0.00703 | 2.7639847 | -2.48608 | 0.622974 |
| ITGAE | NM_002208 | 0.1024046 | 0.00142 | 3.3010053 | -1.12337 | 0.6214662 |
| RPF1 | AK022537 | 0.1669655 | 0.00519 | 2.8708152 | -2.23027 | 0.6207565 |
| TTC37 | NM_014639 | 0.1557142 | 0.00449 | 2.9212976 | -2.10667 | 0.620113 |
| PCK1 | NM_002591 | 0.2647025 | 0.0184 | 2.4073365 | -3.28017 | 0.6197682 |
| PCMT1 | NM_005389 | 0.0544513 | 0.000233 | 3.848316 | 0.44757 | 0.6194287 |
| CUTA | NM_015921 | 0.3438323 | 0.0383 | 2.1052842 | -3.88075 | 0.6193813 |
| TMEM109 | AF131803 | 0.3073227 | 0.0271 | 2.2502214 | -3.60193 | 0.6192188 |
| DDX27 | NM_017895 | 0.3194439 | 0.0316 | 2.186578 | -3.72638 | 0.6184574 |
| CRYZ | NM_001889 | 0.151322 | 0.00418 | 2.9457636 | -2.04615 | 0.6172493 |
| CA9 | NM_001216 | 0.2318019 | 0.013 | 2.5392863 | -2.99793 | 0.6172359 |
| UBAP2 | NM_018449 | 0.1987867 | 0.00812 | 2.7125012 | -2.60653 | 0.6167794 |
| HHEX | NM_002729 | 0.1144986 | 0.00201 | 3.1898264 | -1.42084 | 0.6166688 |
| UBE2K | NM_005339 | 0.1509582 | 0.00413 | 2.949699 | -2.03638 | 0.6160268 |
| SHMT2 | NM_005412 | 0.359785 | 0.0426 | 2.0602403 | -3.96081 | 0.6159763 |
| COX6B1 | NM_001863 | 0.1606636 | 0.00474 | 2.9023738 | -2.15321 | 0.6158242 |
| A1CF | NM_014576 | 0.2184262 | 0.0108 | 2.6079716 | -2.83589 | 0.6152448 |
| H2AFY2 | NM_018649 | 0.0915477 | 0.00105 | 3.3981687 | -0.86346 | 0.6149636 |
| PFKFB1 | NM_002625 | 0.2492343 | 0.0155 | 2.4707933 | -3.14669 | 0.6144838 |
| VEGFC | NM_005429 | 0.2121653 | 0.00983 | 2.6426449 | -2.76696 | 0.6141613 |
| PREB | NM_013388 | 0.2195749 | 0.0111 | 2.5999289 | -2.86342 | 0.6137154 |
| ANK2 | AF131823 | 0.2734453 | 0.0201 | 2.3710468 | -3.35614 | 0.613013 |
| GLI3 | NM_000168 | 0.1049394 | 0.00153 | 3.2771614 | -1.18781 | 0.6127574 |
| TNS2 | AB028998 | 0.1341301 | 0.00295 | 3.0649108 | -1.74733 | 0.6124792 |
| ZC4H2 | NM_018684 | 0.3317432 | 0.0353 | 2.1404771 | -3.81456 | 0.6123368 |
| RPS26 | NM_001029 | 0.3516452 | 0.0405 | 2.0811342 | -3.92561 | 0.6122027 |
| EIF2B2 | NM_014239 | 0.2266344 | 0.0118 | 2.5763129 | -2.91608 | 0.6119198 |
| CRYGA | NM_014617 | 0.2352335 | 0.0133 | 2.5286423 | -3.02127 | 0.6106308 |
| HEXA | NM_000520 | 0.2318019 | 0.013 | 2.5396572 | -2.99711 | 0.6102327 |
| SMARCA1 | NM_003069 | 0.3124692 | 0.0294 | 2.2162974 | -3.66788 | 0.6097957 |
| PYGO1 | AL049925 | 0.1810254 | 0.00664 | 2.786089 | -2.43503 | 0.6091346 |
| FRMD6-AS1 | AK022437 | 0.2643568 | 0.0179 | 2.4163262 | -3.26251 | 0.6084092 |
| RFC3 | NM_002915 | 0.3575256 | 0.042 | 2.0658001 | -3.93958 | 0.6071542 |
| SEMA3G | NM_020163 | 0.2647025 | 0.0185 | 2.403206 | -3.29008 | 0.6067726 |
| HCCS | NM_005333 | 0.1514416 | 0.0042 | 2.9439501 | -2.05065 | 0.6066246 |
| DSP | NM_004415 | 0.209542 | 0.00943 | 2.6580293 | -2.73193 | 0.6066242 |
| PTPN23 | AB025194 | 0.1408906 | 0.00342 | 3.0154063 | -1.8731 | 0.6066035 |
| PSMB2 | NM_002794 | 0.2809852 | 0.022 | 2.3349673 | -3.43138 | 0.6063833 |
| UFL1 | AB018319 | 0.1144986 | 0.00199 | 3.1941461 | -1.41157 | 0.6063563 |
| TRMT6 | NM_015939 | 0.1671388 | 0.00523 | 2.8682153 | -2.23659 | 0.6062602 |
| HARS | NM_002109 | 0.1366355 | 0.00314 | 3.0426355 | -1.80261 | 0.6058187 |
| LOC100131508 | AF130064 | 0.2828206 | 0.0223 | 2.3295997 | -3.44235 | 0.6056986 |
| SNTB2 | NM_006750 | 0.1794227 | 0.00651 | 2.7913864 | -2.42122 | 0.6056978 |
| RBX1 | NM_014248 | 0.353342 | 0.0408 | 2.0779541 | -3.93148 | 0.6055727 |
| POLR2L | U37690 | 0.3203134 | 0.0321 | 2.1797501 | -3.73955 | 0.6055154 |
| IL6ST | NM_002184 | 0.2192333 | 0.0109 | 2.6045539 | -2.85298 | 0.6054623 |
| SSB | J04205 | 0.2283232 | 0.0124 | 2.5600541 | -2.95202 | 0.6053499 |
| MMRN1 | NM_007351 | 0.3085383 | 0.0279 | 2.2374271 | -3.6272 | 0.6050198 |
| TMEM208 | NM_014187 | 0.2656183 | 0.0187 | 2.3984601 | -3.30002 | 0.6048785 |
| CLMP | AK026068 | 0.2308232 | 0.0127 | 2.5463946 | -2.98229 | 0.6044176 |
| MRPL16 | NM_017840 | 0.3194439 | 0.0316 | 2.1860535 | -3.7274 | 0.6043834 |
| ST8SIA1 | NM_003034 | 0.1987867 | 0.00812 | 2.7133526 | -2.60494 | 0.6040073 |
| MRPS2 | NM_016034 | 0.2786639 | 0.021 | 2.3519216 | -3.3966 | 0.603997 |
| ASXL1 | AB023195 | 0.2272024 | 0.0122 | 2.5642319 | -2.94287 | 0.6029624 |
| GSTM3 | NM_000849 | 0.3361507 | 0.0366 | 2.125036 | -3.84278 | 0.6027815 |
| SNORA62 | L07383 | 0.3272746 | 0.0339 | 2.1575031 | -3.78219 | 0.602584 |
| PDLIM1 | U90878 | 0.3022342 | 0.0254 | 2.2762295 | -3.55018 | 0.6024121 |
| ENO2 | NM_001975 | 0.3774739 | 0.0497 | 1.9921017 | -4.08696 | 0.6023715 |
| SLC35E2B | AB007916 | 0.0924214 | 0.0011 | 3.3822433 | -0.9012 | 0.6013878 |
| EPX | NM_000502 | 0.323085 | 0.0329 | 2.1701804 | -3.75707 | 0.6013243 |
| CEBPG | NM_001806 | 0.0897462 | 0.000933 | 3.4333901 | -0.75927 | 0.6012474 |
| AP3S2 | AL109702 | 0.2484966 | 0.0154 | 2.4744201 | -3.12802 | 0.6007432 |
| SNORA67 | Y11161 | 0.2198619 | 0.0111 | 2.5983196 | -2.86696 | 0.6006434 |
| RPL35A | AK021571 | 0.3532797 | 0.0407 | 2.0787324 | -3.93004 | 0.6002077 |
| ARL6IP4 | NM_016638 | 0.1408906 | 0.00344 | 3.0117114 | -1.88103 | 0.5988954 |
| PRPF40A | NM_017892 | 0.228885 | 0.0126 | 2.5505375 | -2.97316 | 0.5984274 |
| GBAS | NM_001483 | 0.2177491 | 0.0108 | 2.6090801 | -2.84281 | 0.5982774 |
| HPN | NM_002151 | 0.1244042 | 0.00246 | 3.1238314 | -1.59373 | 0.5979296 |
| MRPS35 | AF182422 | 0.2794145 | 0.0213 | 2.3473991 | -3.4059 | 0.5978083 |
| SYT7 | Y19237 | 0.3247015 | 0.0333 | 2.1645502 | -3.76872 | 0.5975224 |
| CD44 | M59040 | 0.2396323 | 0.014 | 2.5095819 | -3.06287 | 0.5967262 |
| CWC15 | NM_016403 | 0.1987867 | 0.00811 | 2.7129817 | -2.60541 | 0.5967206 |
| CTNNBL1 | AK024761 | 0.2717214 | 0.0197 | 2.3784731 | -3.34124 | 0.5965003 |
| PPIG | NM_004792 | 0.2647025 | 0.0183 | 2.4068964 | -3.28234 | 0.5962387 |
| NUP205 | D86978 | 0.3389515 | 0.0373 | 2.1166965 | -3.85939 | 0.5956264 |
| OGG1 | NM_016819 | 0.1920931 | 0.00752 | 2.7418538 | -2.53904 | 0.5955734 |
| EFNB2 | NM_004093 | 0.3341966 | 0.0361 | 2.1315576 | -3.8025 | 0.5954141 |
| ADCY1 | L05500 | 0.1357556 | 0.00304 | 3.0536746 | -1.77446 | 0.5953022 |
| CCDC47 | NM_020198 | 0.2446803 | 0.0147 | 2.4909305 | -3.10331 | 0.5951778 |
| PMS2P4 | D38438 | 0.1037891 | 0.00146 | 3.2931904 | -1.14712 | 0.5951411 |
| ATP5E | NM_006886 | 0.1335098 | 0.00292 | 3.0674325 | -1.73927 | 0.5948786 |
| CEBPZ | NM_005760 | 0.2845523 | 0.0226 | 2.3242033 | -3.45335 | 0.5933353 |
| HUWE1 | NM_005703 | 0.2794639 | 0.0213 | 2.3467589 | -3.40722 | 0.5932582 |
| ACAT1 | NM_000019 | 0.2352335 | 0.0134 | 2.5279956 | -3.02269 | 0.5931355 |
| PKIG | NM_007066 | 0.3010319 | 0.0251 | 2.282139 | -3.52584 | 0.5930055 |
| DMKN | AF086315 | 0.3575256 | 0.042 | 2.0658948 | -3.93941 | 0.5929671 |
| WDR76 | AK023035 | 0.1725725 | 0.00579 | 2.8339304 | -2.32025 | 0.592936 |
| SSRP1 | NM_003146 | 0.3194439 | 0.0317 | 2.185725 | -3.72633 | 0.5925462 |
| RAB38 | AF235022 | 0.1824064 | 0.00675 | 2.7789285 | -2.45077 | 0.5925391 |
| COPS2 | NM_004236 | 0.1335098 | 0.00292 | 3.0675037 | -1.73909 | 0.5925349 |
| C1orf112 | NM_018186 | 0.16832 | 0.00537 | 2.8592527 | -2.25833 | 0.592474 |
| CNRIP1 | AL110235 | 0.1526166 | 0.00433 | 2.9334509 | -2.07666 | 0.5916116 |
| MCM2 | NM_004526 | 0.3712602 | 0.0468 | 2.0184422 | -4.03989 | 0.5909914 |
| RPL38 | NM_000999 | 0.3566103 | 0.0415 | 2.0702725 | -3.94563 | 0.5908887 |
| ARHGAP28 | AK024298 | 0.3469379 | 0.0391 | 2.0960322 | -3.89799 | 0.5907175 |
| RPS19 | NM_001022 | 0.1529701 | 0.00437 | 2.9304014 | -2.0842 | 0.5906996 |
| MAP3K6 | NM_004672 | 0.1851786 | 0.00698 | 2.7668016 | -2.47943 | 0.5897627 |
| PATZ1 | AF254082 | 0.3712044 | 0.0466 | 2.0198048 | -4.03744 | 0.5896305 |
| DCK | NM_000788 | 0.1525002 | 0.00428 | 2.9376179 | -2.06634 | 0.589469 |
| MRPL12 | NM_002949 | 0.3024516 | 0.0255 | 2.2754707 | -3.5517 | 0.5886265 |
| PDGFC | NM_016205 | 0.2272024 | 0.0121 | 2.5649029 | -2.94138 | 0.5877432 |
| EMC8 | NM_006067 | 0.3566103 | 0.0416 | 2.0691893 | -3.94762 | 0.5868493 |
| RPL37 | NM_000997 | 0.3751013 | 0.0483 | 2.0047444 | -4.06444 | 0.5866194 |
| GARS | NM_002047 | 0.2159984 | 0.0103 | 2.6254625 | -2.80603 | 0.5865462 |
| SUCLG2 | AF131748 | 0.2170877 | 0.0107 | 2.6114154 | -2.83756 | 0.5862782 |
| FAM216A | NM_013300 | 0.259265 | 0.0169 | 2.437244 | -3.21829 | 0.5861953 |
| WDR33 | NM_018383 | 0.1401609 | 0.00338 | 3.0191156 | -1.86373 | 0.5861413 |
| VNN1 | NM_004666 | 0.3665028 | 0.0452 | 2.0336598 | -4.01244 | 0.5860736 |
| SNRNP40 | NM_004814 | 0.2845523 | 0.0225 | 2.3252247 | -3.45127 | 0.585875 |
| TCEB3-AS1 | AL049675 | 0.209542 | 0.00943 | 2.6578908 | -2.73224 | 0.585421 |
| POLR2B | NM_000938 | 0.1317922 | 0.0028 | 3.0813849 | -1.70346 | 0.5843646 |
| GALNT1 | NM_020474 | 0.1184091 | 0.00223 | 3.1564621 | -1.50859 | 0.5835621 |
| MYL12A | NM_006471 | 0.1529701 | 0.00438 | 2.9300753 | -2.085 | 0.5833193 |
| OBSL1 | AB014557 | 0.1373817 | 0.00318 | 3.0394859 | -1.81213 | 0.5832783 |
| KDM1A | AB011173 | 0.3082737 | 0.0273 | 2.2475934 | -3.60566 | 0.5830099 |
| MAGED2 | U92544 | 0.2620958 | 0.0174 | 2.426199 | -3.24168 | 0.5828114 |
| TPST2 | NM_003595 | 0.2826415 | 0.0222 | 2.3306709 | -3.44016 | 0.5827781 |
| ZMAT2 | X97303 | 0.2210906 | 0.0112 | 2.5934872 | -2.87777 | 0.5826479 |
| EHHADH | NM_001966 | 0.1357556 | 0.00304 | 3.0534906 | -1.77493 | 0.5824176 |
| SNORD7 | AJ007733 | 0.2110673 | 0.00967 | 2.6496571 | -2.75122 | 0.5822818 |
| MCM6 | NM_005915 | 0.2202622 | 0.0111 | 2.5965724 | -2.87087 | 0.5822182 |
| MVD | NM_002461 | 0.2974046 | 0.0244 | 2.292328 | -3.51788 | 0.5819823 |
| ZNF331 | NM_018555 | 0.3613235 | 0.0431 | 2.0546079 | -3.97221 | 0.5817436 |
| XPO5 | AF298880 | 0.2028552 | 0.00862 | 2.6909249 | -2.65645 | 0.5817074 |
| TIMM17A | NM_006335 | 0.3111067 | 0.0291 | 2.2209555 | -3.65955 | 0.5812217 |
| ORC5 | NM_002553 | 0.2451134 | 0.0148 | 2.4892839 | -3.10666 | 0.5810622 |
| DNAJC12 | AF176012 | 0.2020687 | 0.00847 | 2.6972999 | -2.64173 | 0.5797053 |
| ASUN | AK001499 | 0.1624342 | 0.00487 | 2.8931758 | -2.17574 | 0.5796712 |
| ANGEL1 | AL137268 | 0.1334554 | 0.0029 | 3.0697358 | -1.73337 | 0.579622 |
| FMC1 | AF161386 | 0.2308232 | 0.0128 | 2.5437068 | -2.98821 | 0.5795289 |
| CKAP5 | D43948 | 0.3182598 | 0.0311 | 2.1936586 | -3.71269 | 0.5794346 |
| PUM3 | NM_014878 | 0.2773083 | 0.0209 | 2.3552202 | -3.38981 | 0.5794237 |
| CD99 | NM_002414 | 0.3720621 | 0.0469 | 2.017053 | -4.04238 | 0.5782997 |
| C5 | NM_001735 | 0.1557142 | 0.0045 | 2.9207587 | -2.108 | 0.5782977 |
| LTBP1 | NM_000627 | 0.2080014 | 0.00912 | 2.6703839 | -2.70367 | 0.5782504 |
| CUEDC2 | AF086406 | 0.1728702 | 0.00582 | 2.8311025 | -2.32628 | 0.5782334 |
| EEF1A1 | NM_001402 | 0.2752302 | 0.0204 | 2.365305 | -3.368 | 0.5780918 |
| CYSLTR2 | NM_020377 | 0.2272024 | 0.0121 | 2.5645528 | -2.94218 | 0.5779227 |
| EIF2B4 | AF112207 | 0.1428571 | 0.00362 | 2.9956683 | -1.9228 | 0.5778627 |
| SLC25A4 | NM_001151 | 0.2375251 | 0.0137 | 2.5196065 | -3.04102 | 0.5773409 |
| DDRGK1 | AF086090 | 0.313933 | 0.0301 | 2.2064233 | -3.68792 | 0.5768346 |
| PON2 | NM_000305 | 0.1469889 | 0.00386 | 2.9730979 | -1.97806 | 0.5768191 |
| CLASRP | NM_007056 | 0.2282012 | 0.0123 | 2.5594342 | -2.95351 | 0.5767468 |
| C5orf24 | AF085880 | 0.2250644 | 0.0116 | 2.5805987 | -2.90654 | 0.5766788 |
| GLRX5 | NM_016417 | 0.1987867 | 0.0081 | 2.7150218 | -2.60147 | 0.5765698 |
| SLC22A5 | NM_003060 | 0.2502473 | 0.0156 | 2.4685448 | -3.15126 | 0.5763509 |
| GATB | NM_004564 | 0.3244949 | 0.0332 | 2.1663966 | -3.76519 | 0.5761097 |
| CFAP44 | NM_018338 | 0.2620958 | 0.0175 | 2.4265111 | -3.24029 | 0.5756484 |
| VRK2 | NM_006296 | 0.1478914 | 0.00397 | 2.963125 | -2.00296 | 0.5756203 |
| FAN1 | NM_014967 | 0.1671388 | 0.00524 | 2.8676881 | -2.23787 | 0.5753645 |
| NCBP1 | NM_002486 | 0.2142895 | 0.00999 | 2.636857 | -2.7801 | 0.5750933 |
| ALG3 | NM_005787 | 0.2215551 | 0.0113 | 2.5925745 | -2.87995 | 0.5747787 |
| APOM | NM_019101 | 0.2320508 | 0.0131 | 2.5355624 | -3.0061 | 0.5744609 |
| RGS3 | U27655 | 0.1630642 | 0.00491 | 2.8903121 | -2.18274 | 0.5744538 |
| SEPHS1 | NM_012247 | 0.2446332 | 0.0146 | 2.4945775 | -3.09502 | 0.5740722 |
| CYP51A1 | NM_000786 | 0.3738079 | 0.0474 | 2.0134697 | -4.04656 | 0.5737788 |
| CCDC167 | AF161383 | 0.144977 | 0.00377 | 2.9812676 | -1.95761 | 0.5732264 |
| EXOSC7 | D29958 | 0.1823807 | 0.0067 | 2.7811438 | -2.44552 | 0.5731752 |
| FAM206A | NM_017832 | 0.1268203 | 0.00257 | 3.1095382 | -1.63081 | 0.5726738 |
| TMEM258 | NM_014206 | 0.3010626 | 0.0252 | 2.2801939 | -3.54224 | 0.5726616 |
| NUDT15 | NM_018283 | 0.1908746 | 0.00741 | 2.7452267 | -2.53018 | 0.5725715 |
| TBRG4 | NM_004749 | 0.2672473 | 0.019 | 2.3929545 | -3.3111 | 0.5711713 |
| IRS1 | NM_005544 | 0.2069707 | 0.00896 | 2.6776463 | -2.67833 | 0.5709154 |
| FBXL17 | AL133602 | 0.2643568 | 0.018 | 2.4144758 | -3.26641 | 0.5707117 |
| NDUFA3 | NM_004542 | 0.331172 | 0.0351 | 2.1424573 | -3.81081 | 0.5706465 |
| FRRS1L | AF155065 | 0.277449 | 0.0209 | 2.356439 | -3.38571 | 0.5698281 |
| CHST4 | NM_005769 | 0.3687369 | 0.0458 | 2.0283466 | -4.01982 | 0.5697235 |
| DDX1 | NM_004939 | 0.2479891 | 0.0153 | 2.4777071 | -3.13183 | 0.5696604 |
| ABI2 | AF085867 | 0.3634278 | 0.0444 | 2.0409285 | -3.99926 | 0.5696116 |
| CPNE3 | NM_003909 | 0.1172416 | 0.00215 | 3.1679404 | -1.47848 | 0.5681343 |
| FER1L4 | AK026266 | 0.1185909 | 0.00224 | 3.15626 | -1.5111 | 0.5679854 |
| PATZ1 | AF254085 | 0.1728702 | 0.00586 | 2.8306954 | -2.32878 | 0.5677512 |
| ITGA2 | NM_002203 | 0.3773425 | 0.0492 | 1.9958622 | -4.08028 | 0.5677414 |
| MTAP | AK024734 | 0.1885666 | 0.00723 | 2.7539467 | -2.50971 | 0.5677205 |
| EIF3H | NM_003756 | 0.3409934 | 0.0378 | 2.1112326 | -3.86963 | 0.5676065 |
| MLLT1 | AL365410 | 0.2136407 | 0.00992 | 2.6392453 | -2.77468 | 0.567161 |
| LARGE1 | NM_004737 | 0.3085383 | 0.0276 | 2.24351 | -3.61447 | 0.5671224 |
| EHBP1 | AB020710 | 0.2205635 | 0.0112 | 2.5955559 | -2.87315 | 0.5666906 |
| SLC41A3 | NM_017836 | 0.1716402 | 0.00565 | 2.8417012 | -2.30076 | 0.5665161 |
| FTSJ3 | NM_017647 | 0.2330828 | 0.0132 | 2.5328092 | -3.01214 | 0.5663283 |
| GABRG3 | S82769 | 0.2159984 | 0.0103 | 2.6257722 | -2.80533 | 0.5660676 |
| CRLF1 | NM_004750 | 0.3029261 | 0.0257 | 2.2722473 | -3.55746 | 0.5656439 |
| DKFZP586B0319 | AL050097 | 0.2176181 | 0.0108 | 2.6097054 | -2.84141 | 0.5653992 |
| TWIST1 | NM_000474 | 0.2485591 | 0.0155 | 2.4721676 | -3.14374 | 0.5651777 |
| MRPL58 | NM_001545 | 0.1301758 | 0.00275 | 3.0871647 | -1.68859 | 0.5648277 |
| GCN1 | U77700 | 0.3396034 | 0.0374 | 2.1151311 | -3.86233 | 0.5645225 |
| FKRP | AK022638 | 0.3341989 | 0.0361 | 2.1300227 | -3.83433 | 0.5641593 |
| AASDH | AF161436 | 0.1408906 | 0.00346 | 3.0101264 | -1.88503 | 0.5639891 |
| CCDC88A | NM_018084 | 0.3278751 | 0.0341 | 2.1549504 | -3.78706 | 0.5628721 |
| NDUFA9 | NM_005002 | 0.2145573 | 0.0101 | 2.6342855 | -2.78593 | 0.5621874 |
| IGF2BP1 | AK022784 | 0.2647025 | 0.0184 | 2.4049182 | -3.28649 | 0.5612443 |
| STEAP3 | NM_018234 | 0.2484966 | 0.0154 | 2.4746202 | -3.13797 | 0.5611703 |
| BTF3 | NM_001207 | 0.3603638 | 0.0429 | 2.0564014 | -3.97107 | 0.5601299 |
| TYRP1 | NM_000550 | 0.2435112 | 0.0145 | 2.4985921 | -3.08654 | 0.5601071 |
| UAP1 | NM_003115 | 0.1906509 | 0.00739 | 2.7465619 | -2.52705 | 0.5601038 |
| ACBD3 | AK025520 | 0.1773235 | 0.0063 | 2.8032052 | -2.39308 | 0.5595344 |
| POLR1D | NM_015972 | 0.238651 | 0.0138 | 2.5146621 | -3.05181 | 0.5593071 |
| DCTPP1 | AK024843 | 0.2578232 | 0.0168 | 2.4418284 | -3.20824 | 0.5583744 |
| HMGXB3 | D83778 | 0.209542 | 0.00946 | 2.6570241 | -2.73422 | 0.5583485 |
| FAM20A | AL133105 | 0.2545873 | 0.0163 | 2.4513666 | -3.18825 | 0.5583202 |
| ST3GAL3 | NM_006279 | 0.2516358 | 0.0158 | 2.4641333 | -3.16097 | 0.5582687 |
| RRM1 | NM_001033 | 0.359785 | 0.0426 | 2.0597716 | -3.96386 | 0.5572793 |
| UBE2N | NM_003348 | 0.2395218 | 0.0139 | 2.5118523 | -3.05793 | 0.5572765 |
| LEFTY2 | NM_003240 | 0.2483691 | 0.0153 | 2.4767848 | -3.13381 | 0.5568031 |
| CLGN | NM_004362 | 0.1365237 | 0.00313 | 3.0436916 | -1.79992 | 0.5564022 |
| LINC00527 | AJ011409 | 0.2620958 | 0.0175 | 2.4241065 | -3.2461 | 0.5553984 |
| FANCG | NM_004629 | 0.2662736 | 0.0189 | 2.3957454 | -3.30528 | 0.5546644 |
| EEF1B2 | S81522 | 0.3280313 | 0.0344 | 2.1511122 | -3.79347 | 0.5535717 |
| CBR1 | NM_001757 | 0.3755392 | 0.0484 | 2.0033947 | -4.05211 | 0.5524467 |
| SRP14 | NM_003134 | 0.1678959 | 0.00531 | 2.8633495 | -2.2484 | 0.5521792 |
| ATP5J | NM_001685 | 0.243156 | 0.0144 | 2.5008007 | -3.08194 | 0.5520294 |
| DUSP7 | AL110157 | 0.243156 | 0.0144 | 2.500857 | -3.07132 | 0.5515605 |
| NFKBIA | NM_020529 | 0.3477033 | 0.0393 | 2.0940675 | -3.90164 | 0.5507641 |
| CENPK | AY009151 | 0.2159984 | 0.0102 | 2.6278195 | -2.80057 | 0.5503898 |
| RSRC1 | NM_016625 | 0.2688191 | 0.0192 | 2.3888816 | -3.32003 | 0.5501858 |
| LARP1 | AB018274 | 0.2954527 | 0.0241 | 2.2971939 | -3.50808 | 0.5501419 |
| HMGCS1 | NM_002130 | 0.280592 | 0.0217 | 2.3390677 | -3.42299 | 0.5496296 |
| NOP16 | NM_016391 | 0.1893913 | 0.0073 | 2.7509243 | -2.51681 | 0.548864 |
| SETD6 | AK024801 | 0.336789 | 0.0367 | 2.1231746 | -3.84723 | 0.5486206 |
| MRPS16 | NM_016065 | 0.2272024 | 0.0121 | 2.5660306 | -2.93891 | 0.5484825 |
| SFI1 | AB011114 | 0.1221433 | 0.00238 | 3.1352577 | -1.564 | 0.5481575 |
| DST | X75692 | 0.2801282 | 0.0214 | 2.3449986 | -3.41083 | 0.5478944 |
| TRMT44 | AK022953 | 0.243028 | 0.0143 | 2.5031178 | -3.07655 | 0.5474119 |
| ACSM3 | NM_005622 | 0.3749151 | 0.0481 | 2.006984 | -4.05815 | 0.5470761 |
| SMYD5 | U50383 | 0.2308232 | 0.0128 | 2.5458552 | -2.98348 | 0.5468049 |
| PLIN2 | NM_001122 | 0.3746676 | 0.0478 | 2.0090373 | -4.05676 | 0.546485 |
| FZD5 | AK024850 | 0.2594182 | 0.017 | 2.4380967 | -3.2047 | 0.5455423 |
| NMT1 | AF043324 | 0.151322 | 0.00418 | 2.9457094 | -2.04629 | 0.5455229 |
| ADAM22 | AL133090 | 0.3706609 | 0.0462 | 2.0235921 | -4.03062 | 0.5446008 |
| ANAPC11 | NM_016476 | 0.246904 | 0.0151 | 2.4819766 | -3.12264 | 0.5444706 |
| POLK | NM_016218 | 0.2193009 | 0.011 | 2.6022096 | -2.85824 | 0.5432689 |
| GRID1 | AB033046 | 0.2118902 | 0.00976 | 2.6452532 | -2.76104 | 0.5430727 |
| FIGN | NM_018086 | 0.2226083 | 0.0114 | 2.5881343 | -2.88977 | 0.5426774 |
| EMP2 | NM_001424 | 0.2709425 | 0.0196 | 2.3821794 | -3.32203 | 0.5424316 |
| TBCB | NM_001281 | 0.2150352 | 0.0101 | 2.6323015 | -2.79042 | 0.5422045 |
| PALD1 | AB033100 | 0.2159377 | 0.0102 | 2.6286309 | -2.79873 | 0.5417406 |
| OBSCN | AB046859 | 0.231417 | 0.0129 | 2.541647 | -2.99274 | 0.5399913 |
| SLC37A4 | NM_001467 | 0.3193544 | 0.0313 | 2.1906439 | -3.7177 | 0.5399497 |
| PLP2 | NM_002668 | 0.2794145 | 0.0213 | 2.3474786 | -3.40574 | 0.5398867 |
| RAD1 | NM_002853 | 0.209542 | 0.00935 | 2.663393 | -2.72041 | 0.5396375 |
| RPE | AJ224326 | 0.359785 | 0.0427 | 2.0595543 | -3.96317 | 0.5395643 |
| MUTYH | NM_012222 | 0.2711364 | 0.0196 | 2.3804473 | -3.33714 | 0.539356 |
| ZBTB38 | AK025985 | 0.228628 | 0.0125 | 2.5534459 | -2.96674 | 0.5391521 |
| SPG7 | NM_003119 | 0.2872702 | 0.023 | 2.316578 | -3.46886 | 0.5379952 |
| BMP5 | M60314 | 0.2727714 | 0.02 | 2.3722902 | -3.34283 | 0.537977 |
| DPM3 | NM_018973 | 0.3614109 | 0.0433 | 2.0527087 | -3.97676 | 0.5378622 |
| HEMGN | AF130060 | 0.2121611 | 0.00982 | 2.6432761 | -2.76553 | 0.5370487 |
| COG5 | NM_006348 | 0.1087525 | 0.00175 | 3.2351078 | -1.30062 | 0.5369328 |
| ADAM12 | NM_003474 | 0.2516358 | 0.0158 | 2.4641646 | -3.1609 | 0.5365479 |
| SCP2 | NM_002979 | 0.1071625 | 0.00169 | 3.2459216 | -1.27172 | 0.5362637 |
| SUB1 | NM_006713 | 0.3038871 | 0.026 | 2.2671307 | -3.56834 | 0.5359884 |
| PARM1 | NM_015393 | 0.304273 | 0.0263 | 2.2617766 | -3.579 | 0.535983 |
| KCNMB4 | NM_014505 | 0.2322068 | 0.0131 | 2.5349497 | -3.00745 | 0.5359037 |
| ETFA | NM_000126 | 0.3479119 | 0.0395 | 2.0918288 | -3.9058 | 0.5355705 |
| CYP4B1 | NM_000779 | 0.3040447 | 0.0263 | 2.2627466 | -3.57707 | 0.5355283 |
| TBKBP1 | NM_014726 | 0.2845523 | 0.0225 | 2.3247998 | -3.45213 | 0.535376 |
| RPL17 | NM_000985 | 0.3193544 | 0.0314 | 2.1888125 | -3.72207 | 0.5353004 |
| GAPDHS | NM_014364 | 0.2320508 | 0.0131 | 2.5365478 | -2.9938 | 0.5342928 |
| UXT | NM_004182 | 0.2630092 | 0.0177 | 2.4201601 | -3.25443 | 0.5340475 |
| ENDOU | NM_006025 | 0.1602362 | 0.0047 | 2.9051801 | -2.14632 | 0.5339443 |
| NRG2 | NM_013982 | 0.2662736 | 0.0189 | 2.3954571 | -3.3063 | 0.5337347 |
| CCDC136 | AL133027 | 0.1365237 | 0.00311 | 3.0466461 | -1.79392 | 0.533462 |
| MAP3K4 | NM_005922 | 0.2850785 | 0.0227 | 2.3222841 | -3.45607 | 0.5331746 |
| ULK3 | AL117482 | 0.3280313 | 0.0345 | 2.1502441 | -3.79512 | 0.5330409 |
| SMC2 | NM_006444 | 0.3774739 | 0.0496 | 1.9938125 | -4.08039 | 0.5329677 |
| USP7 | NM_003470 | 0.151322 | 0.00418 | 2.9455448 | -2.04669 | 0.5325883 |
| SBF1 | U93181 | 0.3124692 | 0.0296 | 2.2146059 | -3.67036 | 0.5320241 |
| RPL21 | NM_000982 | 0.3628601 | 0.0442 | 2.0432573 | -3.99503 | 0.5318165 |
| RPS6KB1 | NM_003161 | 0.178177 | 0.00635 | 2.8001625 | -2.40033 | 0.531561 |
| COL11A1 | NM_001854 | 0.3404852 | 0.0377 | 2.1142481 | -3.84744 | 0.5314577 |
| MFGE8 | NM_005928 | 0.2546144 | 0.0164 | 2.4520218 | -3.18592 | 0.5308357 |
| MRPS33 | NM_016071 | 0.3328823 | 0.0358 | 2.1333841 | -3.82798 | 0.530493 |
| MRPS14 | AL049705 | 0.3478862 | 0.0394 | 2.0932584 | -3.90315 | 0.5299484 |
| DIAPH2 | NM_006729 | 0.1390339 | 0.00325 | 3.0324747 | -1.82992 | 0.5296351 |
| RPL36AL | NM_001001 | 0.1428571 | 0.00359 | 2.9974591 | -1.91696 | 0.5294351 |
| PDS5B | NM_015032 | 0.2213313 | 0.0113 | 2.591849 | -2.88144 | 0.5293401 |
| PDS5A | AB014548 | 0.3014722 | 0.0253 | 2.2787983 | -3.54504 | 0.5292989 |
| AP2B1 | NM_001282 | 0.1802386 | 0.0066 | 2.7868979 | -2.43188 | 0.5292052 |
| TRAPPC2L | NM_016209 | 0.2647025 | 0.0185 | 2.4026751 | -3.2912 | 0.529048 |
| TOM1L1 | NM_005486 | 0.2069105 | 0.00891 | 2.6788003 | -2.68436 | 0.5288359 |
| TCERG1 | NM_006706 | 0.363318 | 0.0444 | 2.0416771 | -3.9979 | 0.5286596 |
| ITGB1BP1 | NM_004763 | 0.3113636 | 0.0291 | 2.2200607 | -3.6613 | 0.5285286 |
| STIL | NM_003035 | 0.3603638 | 0.0429 | 2.0572795 | -3.96621 | 0.5280049 |
| ACER3 | NM_018367 | 0.2647025 | 0.0185 | 2.4030149 | -3.29048 | 0.5276932 |
| PSMA2 | NM_002787 | 0.336082 | 0.0365 | 2.1255343 | -3.84278 | 0.5273286 |
| SLC10A3 | NM_019848 | 0.2195749 | 0.0111 | 2.5992328 | -2.86491 | 0.5268799 |
| INTU | AB033110 | 0.2502473 | 0.0156 | 2.4687182 | -3.15088 | 0.5267319 |
| COL4A4 | NM_000092 | 0.2479891 | 0.0152 | 2.4782467 | -3.13067 | 0.5264551 |
| PRIM1 | NM_000946 | 0.3194439 | 0.0316 | 2.1864229 | -3.72585 | 0.526073 |
| KDELR2 | NM_006854 | 0.3589156 | 0.0424 | 2.061748 | -3.96128 | 0.5257985 |
| DHX9 | NM_001357 | 0.3613235 | 0.0432 | 2.0537455 | -3.97592 | 0.5256811 |
| ZZZ3 | AL080063 | 0.2942233 | 0.024 | 2.2997398 | -3.50294 | 0.5249009 |
| ZDHHC1 | U90653 | 0.313933 | 0.0299 | 2.2098748 | -3.67958 | 0.5246159 |
| SAV1 | AK023071 | 0.2042334 | 0.00871 | 2.6869835 | -2.66553 | 0.5243192 |
| ZDHHC14 | AK024637 | 0.3124692 | 0.0295 | 2.2158576 | -3.66792 | 0.5241046 |
| FBXO25 | AF174605 | 0.2643568 | 0.0181 | 2.4126211 | -3.26992 | 0.5240828 |
| DHX36 | AF217190 | 0.2647025 | 0.0183 | 2.4062842 | -3.28363 | 0.5234479 |
| CDYL | NM_004824 | 0.2320508 | 0.0131 | 2.5369523 | -3.00305 | 0.5231423 |
| NDUFAF3 | AL049955 | 0.3110118 | 0.0289 | 2.2233519 | -3.65486 | 0.522395 |
| KMT2D | U80756 | 0.2176181 | 0.0107 | 2.6100331 | -2.84067 | 0.5220838 |
| SERPINA1 | X05826 | 0.2131997 | 0.00989 | 2.6404306 | -2.77199 | 0.5212752 |
| DUSP4 | NM_001394 | 0.2484966 | 0.0154 | 2.4738101 | -3.13996 | 0.5211326 |
| NDFIP2 | AB032991 | 0.3038871 | 0.026 | 2.2681929 | -3.55242 | 0.5208076 |
| MIEF1 | NM_019008 | 0.2159984 | 0.0103 | 2.6256029 | -2.80558 | 0.5198508 |
| ATP5J2 | NM_004889 | 0.2169791 | 0.0106 | 2.6164427 | -2.82624 | 0.5198505 |
| ZNF644 | NM_016620 | 0.3102192 | 0.0286 | 2.2282474 | -3.64526 | 0.5195529 |
| PARL | NM_018622 | 0.3457833 | 0.039 | 2.0980792 | -3.88016 | 0.5194736 |
| ANP32D | NM_012404 | 0.2159984 | 0.0103 | 2.6249061 | -2.80715 | 0.5190637 |
| FOXF2 | NM_001452 | 0.1301758 | 0.00273 | 3.0888765 | -1.68418 | 0.5188115 |
| ACO1 | M58510 | 0.2828256 | 0.0223 | 2.3293203 | -3.44292 | 0.5180587 |
| NT5E | NM_002526 | 0.2643568 | 0.0181 | 2.4122642 | -3.27027 | 0.5177026 |
| USP13 | NM_003940 | 0.2803326 | 0.0216 | 2.341909 | -3.41717 | 0.5177 |
| MESDC2 | D42039 | 0.3321963 | 0.0355 | 2.1380967 | -3.81815 | 0.5165405 |
| PSMB10 | NM_002801 | 0.209542 | 0.00938 | 2.6600987 | -2.7272 | 0.5164196 |
| KIF2A | NM_004520 | 0.2624415 | 0.0176 | 2.4228695 | -3.24872 | 0.5161731 |
| ARID2 | AB046777 | 0.1975204 | 0.00797 | 2.7192097 | -2.59094 | 0.5160701 |
| CCDC149 | AK023472 | 0.2110673 | 0.00967 | 2.6488247 | -2.75291 | 0.51574 |
| C17orf58 | AK026583 | 0.2202622 | 0.0111 | 2.5964586 | -2.87112 | 0.5156722 |
| MRPL24 | AK024570 | 0.3182598 | 0.0311 | 2.1943461 | -3.69739 | 0.5145775 |
| FEM1B | NM_015322 | 0.1443034 | 0.00371 | 2.9860671 | -1.94558 | 0.5145369 |
| DFNA5 | NM_004403 | 0.2647025 | 0.0185 | 2.4020326 | -3.29254 | 0.5141135 |
| ARMC10 | AF275808 | 0.2809852 | 0.0219 | 2.3364783 | -3.42774 | 0.5140956 |
| MUC1 | NM_002456 | 0.323085 | 0.0329 | 2.1697651 | -3.75873 | 0.5139473 |
| FARP1 | NM_005766 | 0.216553 | 0.0104 | 2.6217014 | -2.81439 | 0.5135768 |
| WDR46 | NM_005452 | 0.3481988 | 0.0396 | 2.0912397 | -3.89193 | 0.5132509 |
| DUSP3 | AL049417 | 0.3645243 | 0.0447 | 2.0379663 | -4.00463 | 0.5132394 |
| CCDC91 | NM_018318 | 0.2266344 | 0.0118 | 2.5762712 | -2.90643 | 0.5132046 |
| ZNF706 | NM_016096 | 0.2786639 | 0.021 | 2.3523267 | -3.39577 | 0.5131312 |
| SRP9 | NM_003133 | 0.3182598 | 0.0311 | 2.1927194 | -3.71451 | 0.5128194 |
| C11orf58 | NM_014267 | 0.2460024 | 0.0149 | 2.4854984 | -3.11504 | 0.5124083 |
| GSPT1 | NM_002094 | 0.3201262 | 0.0321 | 2.1802464 | -3.73859 | 0.5122549 |
| PDE4D | NM_006203 | 0.228628 | 0.0125 | 2.5543722 | -2.9647 | 0.5120889 |
| SAGE1 | NM_018666 | 0.2451928 | 0.0149 | 2.4874372 | -3.11086 | 0.5117519 |
| EXOSC10 | NM_002685 | 0.350072 | 0.0399 | 2.0881195 | -3.89769 | 0.5116479 |
| DOCK6 | AK022412 | 0.2272024 | 0.0121 | 2.5653979 | -2.94029 | 0.5111871 |
| IPO8 | NM_006390 | 0.2099189 | 0.00953 | 2.6542764 | -2.74049 | 0.5111621 |
| LMBR1L | NM_018113 | 0.2809852 | 0.022 | 2.3349397 | -3.43088 | 0.5109342 |
| MCM4 | X74794 | 0.249461 | 0.0156 | 2.470113 | -3.14815 | 0.510802 |
| FEZ1 | NM_005103 | 0.3329858 | 0.0359 | 2.1339142 | -3.82509 | 0.5107317 |
| WDR43 | D26488 | 0.365912 | 0.045 | 2.0356999 | -4.00766 | 0.5103771 |
| STEAP2 | AK026813 | 0.324739 | 0.0333 | 2.163913 | -3.76994 | 0.5094807 |
| LAMTOR5 | NM_006402 | 0.2272024 | 0.0122 | 2.5633984 | -2.94471 | 0.5093773 |
| FAM96B | NM_016062 | 0.3029261 | 0.0257 | 2.2717997 | -3.55903 | 0.5088865 |
| CYP20A1 | AF183412 | 0.3665028 | 0.0452 | 2.0344454 | -4.00881 | 0.5084818 |
| LSM6 | NM_007080 | 0.2272024 | 0.0122 | 2.5618292 | -2.94821 | 0.507951 |
| PMPCB | NM_004279 | 0.199617 | 0.00826 | 2.7064451 | -2.62057 | 0.5078129 |
| SLF1 | AL050298 | 0.2643568 | 0.0179 | 2.4157727 | -3.26368 | 0.5077298 |
| B3GALT1 | AF117222 | 0.3205045 | 0.0323 | 2.1780569 | -3.74196 | 0.5073693 |
| TMEM254-AS1 | AK021673 | 0.3566103 | 0.0416 | 2.0695755 | -3.93267 | 0.5065608 |
| IFI27L2 | AF208232 | 0.1956321 | 0.0078 | 2.7267075 | -2.57347 | 0.5064749 |
| PHAX | AF086448 | 0.2756699 | 0.0206 | 2.3639209 | -3.35844 | 0.5061642 |
| TOMM22 | NM_020243 | 0.3360025 | 0.0365 | 2.1260545 | -3.8418 | 0.5061271 |
| WARS2 | NM_015836 | 0.3614109 | 0.0433 | 2.0522247 | -3.97869 | 0.5055493 |
| PNMAL1 | NM_018215 | 0.2287376 | 0.0126 | 2.551514 | -2.97101 | 0.5052192 |
| MYO9A | NM_006901 | 0.2424114 | 0.0142 | 2.5048037 | -3.07308 | 0.5051087 |
| HNRNPH1 | NM_005520 | 0.2737103 | 0.0202 | 2.3691603 | -3.36102 | 0.504854 |
| ALX4 | AF294629 | 0.3749151 | 0.0481 | 2.0057054 | -4.06272 | 0.5048439 |
| NDUFB7 | NM_004146 | 0.3509719 | 0.0402 | 2.0839698 | -3.92036 | 0.5046933 |
| SCAMP1 | NM_004866 | 0.2226698 | 0.0114 | 2.586534 | -2.89331 | 0.5044218 |
| FOXM1 | U74612 | 0.3029261 | 0.0256 | 2.2733384 | -3.55528 | 0.5039689 |
| POMP | NM_015932 | 0.353342 | 0.0408 | 2.0778548 | -3.93166 | 0.5037851 |
| MRM1 | AK026231 | 0.3681749 | 0.0457 | 2.0290748 | -4.02073 | 0.5035183 |
| FUT8 | NM_004480 | 0.2184262 | 0.0108 | 2.6071251 | -2.84721 | 0.5030351 |
| U2AF1 | NM_006758 | 0.2643568 | 0.0182 | 2.4100863 | -3.27525 | 0.5026187 |
| RPS27A | NM_002954 | 0.3120187 | 0.0294 | 2.2170681 | -3.66715 | 0.5025844 |
| MITD1 | AL161992 | 0.1920931 | 0.00752 | 2.7399107 | -2.54263 | 0.501982 |
| MSI1 | NM_002442 | 0.3010319 | 0.025 | 2.2826265 | -3.53737 | 0.5016549 |
| SEPT4 | AK022400 | 0.2809852 | 0.0219 | 2.3353507 | -3.4306 | 0.5015065 |
| HIST1H2BN | NM_003520 | 0.3010319 | 0.0251 | 2.2809835 | -3.54066 | 0.5008503 |
| DTL | NM_016448 | 0.3193544 | 0.0314 | 2.1885308 | -3.72261 | 0.5008004 |
| PARPBP | NM_017915 | 0.2193009 | 0.0109 | 2.6033784 | -2.85562 | 0.5007764 |
| EML4 | NM_019063 | 0.2446332 | 0.0147 | 2.4930391 | -3.09875 | 0.5007156 |
| CCDC34 | AF155108 | 0.2426145 | 0.0143 | 2.503006 | -3.07716 | 0.5007108 |
| OSBPL3 | AY008372 | 0.2626411 | 0.0176 | 2.4222412 | -3.24968 | 0.5006595 |
| KIF15 | NM_020242 | 0.3280313 | 0.0343 | 2.152879 | -3.79011 | 0.5003724 |
| PDSS1 | NM_014317 | 0.2057018 | 0.00882 | 2.6825522 | -2.67573 | 0.4993689 |
| COMMD3 | NM_012071 | 0.2352335 | 0.0134 | 2.5279019 | -3.02289 | 0.4985688 |
| EDN1 | NM_001955 | 0.2528444 | 0.0159 | 2.4608896 | -3.16791 | 0.4984134 |
| SRF | NM_003131 | 0.1824064 | 0.00676 | 2.7783053 | -2.45224 | 0.4980189 |
| ZNF280D | NM_017661 | 0.3029261 | 0.0257 | 2.2724114 | -3.55713 | 0.4975756 |
| ZNF280C | AK000102 | 0.2159984 | 0.0103 | 2.6271379 | -2.80211 | 0.4975602 |
| SKP1 | D17032 | 0.3097259 | 0.0284 | 2.2301032 | -3.64161 | 0.4972602 |
| HACL1 | NM_012260 | 0.2288093 | 0.0126 | 2.5510288 | -2.97208 | 0.4969643 |
| UFM1 | NM_016617 | 0.3164281 | 0.0308 | 2.1977248 | -3.70481 | 0.4969038 |
| ARL6IP5 | NM_006407 | 0.3010319 | 0.025 | 2.2823238 | -3.53797 | 0.4967589 |
| HIST1H2AI | NM_003509 | 0.2159377 | 0.0102 | 2.6296881 | -2.79634 | 0.4967241 |
| KIAA0101 | NM_014736 | 0.2593074 | 0.017 | 2.4372865 | -3.2071 | 0.4964225 |
| GMDS | NM_001500 | 0.2541018 | 0.0161 | 2.4575725 | -3.17472 | 0.496394 |
| GRK2 | NM_001619 | 0.3593343 | 0.0425 | 2.0606515 | -3.96329 | 0.4962621 |
| SMC5 | AB011166 | 0.2146583 | 0.0101 | 2.6336911 | -2.78728 | 0.4956119 |
| EMX2 | X68880 | 0.3280313 | 0.0342 | 2.1538092 | -3.78923 | 0.4951334 |
| KLHDC10 | D87454 | 0.243156 | 0.0143 | 2.5011392 | -3.08121 | 0.4948264 |
| SRP68 | NM_014230 | 0.3043244 | 0.0265 | 2.2584458 | -3.58562 | 0.4944181 |
| ATP13A3 | AK024639 | 0.2727714 | 0.02 | 2.3711907 | -3.35681 | 0.4943147 |
| TIMM10B | NM_012192 | 0.1365237 | 0.00312 | 3.0448711 | -1.79691 | 0.4941072 |
| RPRM | NM_019845 | 0.209542 | 0.00945 | 2.658677 | -2.72192 | 0.4930894 |
| SCAND1 | NM_016558 | 0.2479891 | 0.0152 | 2.4788042 | -3.12947 | 0.4930414 |
| HAUS2 | NM_018097 | 0.3043244 | 0.0265 | 2.2595632 | -3.5834 | 0.4928604 |
| TMEM163 | AL122044 | 0.3082737 | 0.0274 | 2.2449442 | -3.61237 | 0.4927967 |
| TAF1C | NM_005679 | 0.1571156 | 0.00458 | 2.9141346 | -2.12431 | 0.4926494 |
| DCAF7 | NM_005828 | 0.3272746 | 0.0339 | 2.1570749 | -3.78301 | 0.4925042 |
| R3HDM2 | NM_014925 | 0.1266667 | 0.00256 | 3.1108135 | -1.62751 | 0.4920177 |
| GRPEL1 | AF070525 | 0.3027304 | 0.0255 | 2.2748376 | -3.55296 | 0.4919809 |
| GOLT1B | NM_016072 | 0.2375251 | 0.0137 | 2.5206246 | -3.03867 | 0.4919125 |
| CLINT1 | NM_014666 | 0.3327453 | 0.0358 | 2.1337699 | -3.82725 | 0.4918206 |
| PCYT1A | NM_005017 | 0.2743887 | 0.0203 | 2.3659463 | -3.36767 | 0.4913563 |
| TIA1 | AF090093 | 0.315105 | 0.0304 | 2.2020415 | -3.69644 | 0.4911452 |
| P4HB | NM_000918 | 0.323085 | 0.0329 | 2.1699461 | -3.75839 | 0.4893356 |
| TFPI | NM_006287 | 0.3194439 | 0.0317 | 2.1854487 | -3.72856 | 0.4889116 |
| CELF4 | AF248650 | 0.3702521 | 0.0462 | 2.0254579 | -4.01066 | 0.4889025 |
| GYS2 | S70004 | 0.2643568 | 0.0182 | 2.4101283 | -3.27555 | 0.488871 |
| EME1 | AK021607 | 0.3040447 | 0.0262 | 2.2638099 | -3.57496 | 0.4886519 |
| NAA35 | AK025266 | 0.3773425 | 0.0494 | 1.99461 | -4.08137 | 0.4886123 |
| HPSE2 | AF282887 | 0.3194439 | 0.0315 | 2.1875763 | -3.72446 | 0.4880935 |
| COX6A2 | NM_005205 | 0.2250644 | 0.0116 | 2.5808149 | -2.90606 | 0.4876818 |
| ZDHHC9 | NM_016032 | 0.3601247 | 0.0428 | 2.0583523 | -3.96425 | 0.4876815 |
| SLC30A7 | AK023089 | 0.2701844 | 0.0194 | 2.3842342 | -3.32972 | 0.4875258 |
| TIMMDC1 | NM_016589 | 0.2270895 | 0.012 | 2.5684222 | -2.9336 | 0.4874518 |
| DNAAF2 | NM_018139 | 0.3706609 | 0.0464 | 2.0226788 | -4.03116 | 0.4867902 |
| SLC39A4 | NM_017767 | 0.3327453 | 0.0357 | 2.1345851 | -3.82571 | 0.486567 |
| POLR2I | NM_006233 | 0.3085383 | 0.0279 | 2.2376657 | -3.62673 | 0.4863866 |
| PCOLCE2 | NM_013363 | 0.2826284 | 0.0222 | 2.3309623 | -3.43957 | 0.4863219 |
| MGST2 | NM_002413 | 0.238944 | 0.0139 | 2.5134714 | -3.0544 | 0.4853853 |
| PDHX | NM_003477 | 0.31075 | 0.0286 | 2.2272978 | -3.64712 | 0.4850164 |
| MST1 | L11924 | 0.3605723 | 0.043 | 2.0554318 | -3.97284 | 0.4840787 |
| SLC9A5 | AK021876 | 0.3119209 | 0.0293 | 2.2181462 | -3.66427 | 0.4831808 |
| USP39 | NM_006590 | 0.2320339 | 0.013 | 2.5373872 | -3.0021 | 0.4825284 |
| RFC4 | NM_002916 | 0.3452894 | 0.0388 | 2.0992246 | -3.89205 | 0.4815948 |
| MIIP | AK022500 | 0.2643025 | 0.0178 | 2.4185357 | -3.25748 | 0.4814346 |
| POLR3G | NM_006467 | 0.2809852 | 0.0218 | 2.3371689 | -3.42688 | 0.4814002 |
| GFOD1 | NM_018988 | 0.2447209 | 0.0148 | 2.490209 | -3.10487 | 0.4810764 |
| ZNF232 | NM_014519 | 0.1519177 | 0.00425 | 2.9402403 | -2.05985 | 0.481064 |
| S100A13 | NM_005979 | 0.3200275 | 0.032 | 2.1808229 | -3.73748 | 0.4810467 |
| RAB32 | NM_006834 | 0.3073227 | 0.0271 | 2.2501926 | -3.60199 | 0.4807903 |
| MYLK | AK025953 | 0.3111067 | 0.0291 | 2.2214371 | -3.64558 | 0.4807487 |
| DNTT | M26147 | 0.2846386 | 0.0226 | 2.3236896 | -3.45439 | 0.480349 |
| RPS11 | NM_001015 | 0.3073227 | 0.027 | 2.2508903 | -3.60061 | 0.4803212 |
| RASA1 | NM_002890 | 0.3614109 | 0.0433 | 2.0520963 | -3.97893 | 0.4801488 |
| PKP2 | NM_004572 | 0.2646371 | 0.0182 | 2.4094261 | -3.27622 | 0.4800032 |
| MAPRE1 | NM_012325 | 0.2803326 | 0.0216 | 2.3414137 | -3.41818 | 0.4798733 |
| CFI | NM_000204 | 0.3746162 | 0.0477 | 2.0108723 | -4.0512 | 0.4794342 |
| GNG5 | NM_005274 | 0.2709425 | 0.0196 | 2.3804913 | -3.33751 | 0.4793896 |
| NUP107 | NM_020401 | 0.3078065 | 0.0272 | 2.2478204 | -3.60669 | 0.4793575 |
| SLC16A10 | AF116652 | 0.313933 | 0.03 | 2.2083003 | -3.68426 | 0.4792157 |
| BCAS3 | NM_017679 | 0.3124692 | 0.0296 | 2.2134315 | -3.67426 | 0.4791591 |
| DIMT1 | NM_014473 | 0.3626634 | 0.0438 | 2.0485035 | -3.98219 | 0.4791416 |
| PSMG2 | NM_020232 | 0.2850785 | 0.0228 | 2.3207398 | -3.4604 | 0.4791228 |
| SIK2 | AB018324 | 0.1956321 | 0.00778 | 2.7280697 | -2.5703 | 0.4789031 |
| GRINA | AL157442 | 0.2571992 | 0.0167 | 2.4437753 | -3.20442 | 0.478566 |
| CSTF3 | NM_001326 | 0.313933 | 0.0301 | 2.2071894 | -3.68562 | 0.4785619 |
| MTA1 | NM_004689 | 0.3280313 | 0.0344 | 2.1509156 | -3.79474 | 0.4784819 |
| PLK4 | NM_014264 | 0.3010319 | 0.025 | 2.2825482 | -3.53752 | 0.4782174 |
| LINC00662 | AK022299 | 0.3164281 | 0.0307 | 2.1990645 | -3.70056 | 0.4780907 |
| CCT8L2 | NM_014406 | 0.359785 | 0.0427 | 2.0583564 | -3.96749 | 0.477966 |
| EXOSC5 | NM_020158 | 0.2479891 | 0.0152 | 2.4778356 | -3.13155 | 0.4772569 |
| TNFAIP8 | NM_014350 | 0.2647025 | 0.0185 | 2.4025106 | -3.29113 | 0.4771663 |
| MAN1B1-AS1 | AK023312 | 0.1669655 | 0.00521 | 2.8700422 | -2.23215 | 0.4761445 |
| NGDN | AK022215 | 0.3193544 | 0.0313 | 2.1909137 | -3.71632 | 0.4758095 |
| GGCX | NM_000821 | 0.2167984 | 0.0105 | 2.6200477 | -2.81812 | 0.4754546 |
| PRKG2 | NM_006259 | 0.3688033 | 0.0458 | 2.0284752 | -4.01843 | 0.4753954 |
| NUP62 | NM_016553 | 0.2905882 | 0.0235 | 2.3074102 | -3.48744 | 0.4750425 |
| PARS2 | AK025585 | 0.3438323 | 0.0383 | 2.1054313 | -3.8795 | 0.4742546 |
| FAM35A | AK022978 | 0.3729978 | 0.0472 | 2.015621 | -4.0427 | 0.4734204 |
| FAM131B | NM_014690 | 0.2723729 | 0.0199 | 2.37318 | -3.35269 | 0.4734062 |
| MIR17HG | AK024240 | 0.259265 | 0.0169 | 2.438545 | -3.21486 | 0.4731077 |
| CD59 | M34671 | 0.3085383 | 0.0279 | 2.2378471 | -3.62638 | 0.4729519 |
| SIKE1 | AK024821 | 0.2318019 | 0.013 | 2.5399269 | -2.99652 | 0.4728625 |
| PHKA2 | NM_000292 | 0.259265 | 0.0169 | 2.4371057 | -3.21859 | 0.4727225 |
| TIGAR | NM_020375 | 0.2210906 | 0.0112 | 2.5941018 | -2.8764 | 0.4726377 |
| TBCE | NM_003193 | 0.301343 | 0.0252 | 2.280069 | -3.54183 | 0.4721047 |
| CYP1A2 | NM_000761 | 0.2557873 | 0.0165 | 2.4464451 | -3.19874 | 0.471888 |
| CHRD | AF209930 | 0.1947325 | 0.00768 | 2.7325572 | -2.55982 | 0.4718658 |
| APPL1 | NM_012096 | 0.2545873 | 0.0162 | 2.4539034 | -3.18284 | 0.4716505 |
| DAD1 | NM_001344 | 0.3662968 | 0.0451 | 2.0346675 | -4.01061 | 0.4714277 |
| SNRPB2 | NM_003092 | 0.3224138 | 0.0327 | 2.1717391 | -3.75495 | 0.470968 |
| PAM16 | NM_016069 | 0.3205045 | 0.0324 | 2.1764575 | -3.74588 | 0.4703226 |
| ACACB | NM_001093 | 0.3278751 | 0.0341 | 2.1554587 | -3.78428 | 0.47027 |
| TRMT12 | NM_017956 | 0.2484966 | 0.0153 | 2.475951 | -3.13537 | 0.4682549 |
| KCNMA1 | NM_002247 | 0.3472285 | 0.0392 | 2.0952652 | -3.89942 | 0.4681094 |
| ARL6IP1 | D31885 | 0.3634278 | 0.0445 | 2.0405456 | -3.99995 | 0.4676309 |
| TRIM68 | NM_018073 | 0.3722816 | 0.047 | 2.0171194 | -4.04001 | 0.4676065 |
| PIH1D1 | NM_017916 | 0.2620958 | 0.0175 | 2.4243571 | -3.24557 | 0.4674279 |
| ETFB | NM_001985 | 0.3197006 | 0.032 | 2.1816207 | -3.73595 | 0.4670584 |
| ZNF691 | NM_015911 | 0.2541322 | 0.0161 | 2.4566947 | -3.17659 | 0.4670547 |
| MPC2 | NM_015415 | 0.3194439 | 0.0318 | 2.184379 | -3.73063 | 0.4670052 |
| ISCA1 | AF038186 | 0.2803326 | 0.0216 | 2.3421669 | -3.41664 | 0.4666648 |
| LAIR2 | NM_002288 | 0.3755392 | 0.0485 | 2.00257 | -4.06832 | 0.4663709 |
| IPO11 | NM_016338 | 0.3022342 | 0.0254 | 2.2763147 | -3.55001 | 0.4660012 |
| ALYREF | AF047002 | 0.3236611 | 0.033 | 2.1687594 | -3.76066 | 0.4655782 |
| TMEM214 | NM_017727 | 0.2720497 | 0.0199 | 2.3749285 | -3.34906 | 0.4655583 |
| ASNS | NM_001673 | 0.2801189 | 0.0214 | 2.345291 | -3.41023 | 0.4648639 |
| UBA6 | NM_018227 | 0.258383 | 0.0168 | 2.4399718 | -3.2125 | 0.4646466 |
| ZFYVE1 | AF251025 | 0.2680931 | 0.0191 | 2.3908329 | -3.31596 | 0.4643762 |
| EMC1 | D42044 | 0.3378305 | 0.0369 | 2.1208988 | -3.8515 | 0.4643488 |
| SLC6A20 | AJ276207 | 0.313933 | 0.0301 | 2.2073531 | -3.68611 | 0.4639809 |
| NME3 | NM_002513 | 0.3615416 | 0.0434 | 2.0515349 | -3.97995 | 0.4639257 |
| DHRS11 | AK026196 | 0.3755392 | 0.0485 | 2.0041304 | -4.0608 | 0.4638121 |
| CCDC6 | AK024913 | 0.3616157 | 0.0434 | 2.0516407 | -3.9787 | 0.4636079 |
| ASPH | NM_004318 | 0.2849424 | 0.0227 | 2.322502 | -3.45681 | 0.4635409 |
| SDF2L1 | AB043007 | 0.3752209 | 0.0483 | 2.0044194 | -4.06502 | 0.4629994 |
| THOC1 | NM_005131 | 0.3203134 | 0.0322 | 2.1798703 | -3.73759 | 0.4624774 |
| PSMB3 | NM_002795 | 0.228628 | 0.0125 | 2.5538103 | -2.96594 | 0.4623131 |
| EXOSC9 | NM_005033 | 0.3538348 | 0.0409 | 2.0770156 | -3.93321 | 0.461745 |
| ALLC | NM_018436 | 0.3182598 | 0.0311 | 2.192984 | -3.714 | 0.4608876 |
| TCF12 | NM_003205 | 0.3626634 | 0.0439 | 2.0460136 | -3.99001 | 0.4607965 |
| GPATCH2 | NM_018040 | 0.2528444 | 0.0159 | 2.4614979 | -3.16634 | 0.4607796 |
| CDV3 | NM_017548 | 0.3010626 | 0.0252 | 2.2804058 | -3.54182 | 0.4602256 |
| SEPT3 | NM_019106 | 0.2254599 | 0.0117 | 2.5795551 | -2.90886 | 0.4599254 |
| ALDH3A2 | NM_000382 | 0.2528444 | 0.0159 | 2.4606446 | -3.16844 | 0.459905 |
| LOC101928076 | AF143325 | 0.3039961 | 0.0261 | 2.2651764 | -3.57154 | 0.4597353 |
| PAK5 | AB040812 | 0.3131136 | 0.0298 | 2.2106976 | -3.67959 | 0.4594494 |
| RNF181 | NM_016494 | 0.1871402 | 0.00713 | 2.7592039 | -2.49734 | 0.4585379 |
| PTDSS1 | NM_014754 | 0.359785 | 0.0427 | 2.0586758 | -3.96691 | 0.4580932 |
| LUC7L2 | NM_016019 | 0.2643568 | 0.0181 | 2.4122027 | -3.27119 | 0.4580012 |
| PEAK1 | AK025943 | 0.3335417 | 0.036 | 2.1321132 | -3.83038 | 0.4575358 |
| EEA1 | NM_003566 | 0.2720497 | 0.0199 | 2.374695 | -3.34955 | 0.4575112 |
| C1orf54 | AK026874 | 0.228628 | 0.0125 | 2.553584 | -2.96644 | 0.4567906 |
| ARL1 | AK001489 | 0.3749151 | 0.0481 | 2.0060489 | -4.06211 | 0.4562936 |
| ELL2 | NM_012081 | 0.2479891 | 0.0152 | 2.4785993 | -3.12991 | 0.4562368 |
| ADCY3 | NM_004036 | 0.3370512 | 0.0368 | 2.122375 | -3.84873 | 0.4561433 |
| MPI | NM_002435 | 0.3258905 | 0.0336 | 2.1608229 | -3.77585 | 0.4560741 |
| PROC | NM_000312 | 0.3102192 | 0.0286 | 2.2284089 | -3.64494 | 0.4560636 |
| WDR5 | AK024484 | 0.243838 | 0.0145 | 2.4963747 | -3.09153 | 0.4553098 |
| GK2 | X78712 | 0.3194746 | 0.0319 | 2.1826658 | -3.73393 | 0.4549951 |
| CA14 | NM_012113 | 0.2270895 | 0.012 | 2.5692006 | -2.93187 | 0.454958 |
| HDAC6 | NM_006044 | 0.1785669 | 0.00645 | 2.794907 | -2.41284 | 0.4549359 |
| KLK15 | X75363 | 0.3773425 | 0.0491 | 1.9974667 | -4.07629 | 0.4543094 |
| CLDN7 | AJ011497 | 0.3680992 | 0.0456 | 2.0298557 | -4.01931 | 0.4542258 |
| KAT2A | AF029777 | 0.3203134 | 0.0322 | 2.1800103 | -3.73732 | 0.4542238 |
| CAD | NM_004341 | 0.3749151 | 0.0481 | 2.0066971 | -4.05983 | 0.4538123 |
| PTPN3 | NM_002829 | 0.1648871 | 0.00507 | 2.8792124 | -2.20983 | 0.4533165 |
| KHSRP | NM_003685 | 0.3773425 | 0.0493 | 1.9951555 | -4.08154 | 0.4529114 |
| SLURP1 | NM_020427 | 0.3318914 | 0.0354 | 2.1389379 | -3.81748 | 0.4524176 |
| SGK3 | NM_013257 | 0.3074274 | 0.0271 | 2.2492645 | -3.60383 | 0.4523649 |
| SLC5A6 | AF069307 | 0.3761225 | 0.0487 | 2.0009966 | -4.07113 | 0.4522201 |
| USP35 | AB037793 | 0.3437553 | 0.0383 | 2.1057203 | -3.87994 | 0.45192 |
| TIPRL | AL049670 | 0.3110118 | 0.0288 | 2.2247947 | -3.65203 | 0.4515542 |
| GFRA1 | NM_005264 | 0.2803326 | 0.0215 | 2.3433356 | -3.41371 | 0.4514968 |
| RFC1 | NM_002913 | 0.2308232 | 0.0128 | 2.5437087 | -2.9882 | 0.451333 |
| SNX9 | NM_016224 | 0.3711607 | 0.0465 | 2.0211203 | -4.03507 | 0.4512829 |
| ELK4 | AK024944 | 0.27389 | 0.0202 | 2.3683886 | -3.36262 | 0.4504903 |
| COX7A1 | NM_001864 | 0.3355812 | 0.0364 | 2.1272256 | -3.83866 | 0.4497264 |
| ROCK2 | NM_004850 | 0.3194439 | 0.0316 | 2.187222 | -3.7243 | 0.4494176 |
| ARL4A | NM_005738 | 0.232248 | 0.0131 | 2.5345244 | -3.00838 | 0.4490326 |
| EIF6 | NM_002212 | 0.2643568 | 0.0181 | 2.4119159 | -3.27179 | 0.448847 |
| GRSF1 | NM_002092 | 0.3381006 | 0.037 | 2.1194321 | -3.85426 | 0.4483243 |
| GABPB1 | NM_005254 | 0.2484966 | 0.0154 | 2.4734223 | -3.14104 | 0.4479348 |
| UBQLN1 | NM_013438 | 0.2599509 | 0.0171 | 2.4327347 | -3.22785 | 0.4477926 |
| TRDN | NM_006073 | 0.3339724 | 0.036 | 2.1315831 | -3.81762 | 0.4475922 |
| BDP1 | AF298152 | 0.2193009 | 0.0109 | 2.6035318 | -2.85527 | 0.4472541 |
| RBP1 | NM_002899 | 0.2647025 | 0.0184 | 2.4054295 | -3.28542 | 0.4471442 |
| AHCYL1 | NM_014121 | 0.2922072 | 0.0237 | 2.3046348 | -3.48074 | 0.4470509 |
| PPP4R4 | AB046842 | 0.3385119 | 0.0372 | 2.1176654 | -3.85758 | 0.4468094 |
| PDE6C | NM_006204 | 0.2905662 | 0.0235 | 2.3097162 | -3.46949 | 0.4465525 |
| HINFP | AL080201 | 0.3162879 | 0.0307 | 2.1991038 | -3.70214 | 0.4465514 |
| EFCAB14 | NM_014774 | 0.1648871 | 0.00505 | 2.8818363 | -2.20436 | 0.4462914 |
| SH3GL1P3 | X99662 | 0.3085383 | 0.0277 | 2.241379 | -3.60655 | 0.4461039 |
| PSMD9 | NM_002813 | 0.2545873 | 0.0162 | 2.453343 | -3.18404 | 0.4458965 |
| ERCC1 | NM_001983 | 0.2794145 | 0.0212 | 2.3485839 | -3.40347 | 0.4457216 |
| MYEOV | AJ223366 | 0.3681749 | 0.0457 | 2.0289843 | -4.02089 | 0.4456535 |
| NTN1 | NM_004822 | 0.3428732 | 0.0381 | 2.1078504 | -3.87596 | 0.445614 |
| MITF | AL117653 | 0.3278751 | 0.0341 | 2.1546998 | -3.78753 | 0.4444943 |
| SLC5A3 | NM_006933 | 0.2978777 | 0.0245 | 2.2907863 | -3.52098 | 0.444234 |
| PIGH | NM_004569 | 0.2846386 | 0.0226 | 2.3232353 | -3.45532 | 0.4441375 |
| UFSP2 | NM_018359 | 0.3449886 | 0.0387 | 2.1006959 | -3.88931 | 0.4423333 |
| OR5E1P | X89671 | 0.3626634 | 0.044 | 2.0456423 | -3.99069 | 0.4417683 |
| CD2AP | AF146277 | 0.3267413 | 0.0338 | 2.1584155 | -3.78045 | 0.4417216 |
| PDF | AF239156 | 0.3124692 | 0.0296 | 2.2131601 | -3.67479 | 0.4408945 |
| SMAD3 | NM_015400 | 0.3543398 | 0.041 | 2.0758367 | -3.93538 | 0.440327 |
| HSPB11 | NM_016126 | 0.3164281 | 0.0308 | 2.1975424 | -3.70517 | 0.4402815 |
| PEPD | NM_000285 | 0.3747788 | 0.0479 | 2.0084482 | -4.05781 | 0.4402487 |
| PAFAH2 | NM_000437 | 0.3450765 | 0.0387 | 2.1002984 | -3.89005 | 0.4399864 |
| DCTN6 | NM_006571 | 0.3545081 | 0.041 | 2.0754317 | -3.93613 | 0.4396766 |
| MUS81 | AK024665 | 0.209542 | 0.00947 | 2.6562996 | -2.73588 | 0.4395979 |
| KIZ | NM_018474 | 0.3194439 | 0.0318 | 2.1833793 | -3.73256 | 0.439582 |
| RAB9A | NM_004251 | 0.2546144 | 0.0164 | 2.4495932 | -3.19203 | 0.43862 |
| RAD51D | NM_002878 | 0.2974046 | 0.0244 | 2.2919564 | -3.51863 | 0.4384566 |
| ABCB6 | NM_005689 | 0.2763447 | 0.0207 | 2.3582172 | -3.38363 | 0.4362608 |
| SACS | NM_014363 | 0.3603638 | 0.0429 | 2.0562337 | -3.97137 | 0.4362025 |
| IRF2BPL | AF075110 | 0.3626634 | 0.0437 | 2.04808 | -3.98625 | 0.4361637 |
| CPSF1 | NM_013291 | 0.2801753 | 0.0214 | 2.3446525 | -3.41154 | 0.4361314 |
| RDH14 | AF237952 | 0.3088677 | 0.0282 | 2.2341514 | -3.63365 | 0.4358941 |
| HEPH | NM_014799 | 0.2961077 | 0.0242 | 2.2954425 | -3.51161 | 0.4357446 |
| SIAE | AF303378 | 0.3280313 | 0.0344 | 2.1513118 | -3.79399 | 0.4355687 |
| PLEC | Z36817 | 0.324739 | 0.0334 | 2.1634053 | -3.77091 | 0.4353669 |
| ATRAID | NM_016085 | 0.3327453 | 0.0357 | 2.1350163 | -3.8249 | 0.4351042 |
| PLTP | NM_006227 | 0.3681749 | 0.0457 | 2.0294721 | -4.01779 | 0.4338354 |
| TMED4 | D17216 | 0.3280313 | 0.0344 | 2.1505885 | -3.79536 | 0.4337809 |
| CSNK2A2 | NM_001896 | 0.3043244 | 0.0265 | 2.2594337 | -3.58366 | 0.4335471 |
| GPN3 | NM_016301 | 0.3664199 | 0.0451 | 2.0347111 | -4.00945 | 0.4321141 |
| INTS8 | NM_017864 | 0.300855 | 0.0249 | 2.2842404 | -3.53413 | 0.4315223 |
| TCF4 | NM_003199 | 0.3365459 | 0.0367 | 2.1241171 | -3.84451 | 0.4308107 |
| PGS1 | AL359590 | 0.3014722 | 0.0252 | 2.2793302 | -3.54331 | 0.4306908 |
| BCL7C | NM_004765 | 0.3038871 | 0.026 | 2.2661719 | -3.57025 | 0.4300383 |
| NAXD | NM_018210 | 0.1358036 | 0.00305 | 3.0537007 | -1.77595 | 0.4296319 |
| ADSL | NM_000026 | 0.3159474 | 0.0306 | 2.199927 | -3.70054 | 0.4294203 |
| C12orf10 | AF289485 | 0.2021841 | 0.0085 | 2.6957188 | -2.64539 | 0.4292298 |
| GLRX3 | NM_006541 | 0.3318914 | 0.0353 | 2.1398004 | -3.81493 | 0.4292081 |
| MGAT4B | NM_014275 | 0.3777714 | 0.0498 | 1.9904634 | -4.08987 | 0.4279041 |
| HKDC1 | AK026414 | 0.3381006 | 0.037 | 2.1194326 | -3.85426 | 0.427107 |
| NKTR | NM_005385 | 0.3280313 | 0.0345 | 2.1495339 | -3.79737 | 0.4268525 |
| HIGD1B | NM_016438 | 0.3712366 | 0.0467 | 2.0193408 | -4.03827 | 0.4262963 |
| DUSP3 | NM_004090 | 0.3273889 | 0.0339 | 2.15671 | -3.7837 | 0.4261097 |
| SOX9 | NM_000346 | 0.3729978 | 0.0472 | 2.0149579 | -4.04615 | 0.4256631 |
| RAB3B | AK002107 | 0.308177 | 0.0273 | 2.2470523 | -3.60821 | 0.4254924 |
| FXN | NM_000144 | 0.3205045 | 0.0323 | 2.1770731 | -3.7447 | 0.4252844 |
| OSGEPL1 | AJ295148 | 0.3340626 | 0.0361 | 2.1306537 | -3.83313 | 0.4250254 |
| BMP7 | NM_001719 | 0.2783535 | 0.021 | 2.3531431 | -3.39409 | 0.4247579 |
| MRPS22 | NM_020191 | 0.3515263 | 0.0404 | 2.08306 | -3.92104 | 0.424683 |
| SLC45A2 | NM_016180 | 0.3577372 | 0.0421 | 2.0646799 | -3.9559 | 0.4243266 |
| PHC1 | U89277 | 0.3258905 | 0.0336 | 2.1617737 | -3.77224 | 0.4241412 |
| RBM6 | NM_005777 | 0.331172 | 0.0351 | 2.1420202 | -3.81164 | 0.4240896 |
| PIGF | NM_002643 | 0.2647025 | 0.0185 | 2.4038884 | -3.28865 | 0.4239671 |
| COX8A | NM_004074 | 0.3088677 | 0.0282 | 2.2342541 | -3.63345 | 0.4235592 |
| EP400 | U80743 | 0.3144731 | 0.0303 | 2.2052106 | -3.68864 | 0.4233711 |
| C12orf65 | AK025908 | 0.2051866 | 0.00877 | 2.6843764 | -2.67154 | 0.4227345 |
| CNIH1 | AK022936 | 0.2170877 | 0.0107 | 2.6117623 | -2.83678 | 0.4226106 |
| SCO1 | NM_004589 | 0.3280313 | 0.0345 | 2.1497232 | -3.79701 | 0.4222751 |
| RAB27B | NM_004163 | 0.3628601 | 0.0442 | 2.0431866 | -3.99516 | 0.4221569 |
| DYRK4 | AF263541 | 0.2850785 | 0.0228 | 2.3207278 | -3.46042 | 0.4217467 |
| STX18 | NM_016930 | 0.2426145 | 0.0143 | 2.5030109 | -3.07714 | 0.4217403 |
| EPB41L2 | NM_001431 | 0.3634278 | 0.0444 | 2.0409305 | -3.99925 | 0.421057 |
| PPM1J | AL133644 | 0.3614109 | 0.0433 | 2.0523735 | -3.96405 | 0.4208945 |
| ZNF131 | U09410 | 0.2809852 | 0.022 | 2.3341128 | -3.43313 | 0.4193992 |
| ZWINT | NM_007057 | 0.3681749 | 0.0457 | 2.0291251 | -4.02063 | 0.4189216 |
| ABL1 | NM_005157 | 0.3746162 | 0.0476 | 2.0106231 | -4.05392 | 0.4188339 |
| CHST3 | NM_004273 | 0.3746162 | 0.0477 | 2.0108987 | -4.03773 | 0.4182968 |
| PRKAR2A | NM_004157 | 0.2793127 | 0.0211 | 2.3503651 | -3.39981 | 0.4182802 |
| U2SURP | AB002330 | 0.3184583 | 0.0312 | 2.1921367 | -3.71564 | 0.417949 |
| ARHGEF16 | NM_014448 | 0.3706609 | 0.0463 | 2.0227328 | -4.03217 | 0.4177338 |
| RRAS | NM_006270 | 0.313933 | 0.0301 | 2.2073925 | -3.6844 | 0.4169647 |
| IFFO2 | AK024480 | 0.3280313 | 0.0344 | 2.1505779 | -3.79538 | 0.4165367 |
| FNBP1L | NM_017737 | 0.3738079 | 0.0474 | 2.0129308 | -4.04978 | 0.4158487 |
| TRIP11 | AF007217 | 0.3251175 | 0.0335 | 2.1624778 | -3.77269 | 0.4157767 |
| MPG | NM_002434 | 0.3085383 | 0.0279 | 2.2382976 | -3.62549 | 0.4156585 |
| KARS | NM_005548 | 0.3401834 | 0.0376 | 2.1136132 | -3.86517 | 0.4155987 |
| ARHGAP5-AS1 | AF147421 | 0.3078065 | 0.0272 | 2.2482876 | -3.60504 | 0.4154765 |
| ANXA9 | NM_003568 | 0.3280313 | 0.0344 | 2.1510781 | -3.79443 | 0.4153559 |
| SIX2 | NM_016932 | 0.3194439 | 0.0316 | 2.1858163 | -3.72785 | 0.4149981 |
| MED1 | NM_004774 | 0.2599033 | 0.0171 | 2.4334166 | -3.22641 | 0.4144875 |
| NR2F1 | NM_005654 | 0.3430221 | 0.0381 | 2.1078761 | -3.87494 | 0.4143378 |
| PQBP1 | NM_005710 | 0.3110118 | 0.029 | 2.2221859 | -3.65714 | 0.4136936 |
| ZNF473 | AB032967 | 0.300855 | 0.0249 | 2.284574 | -3.53346 | 0.413607 |
| SPAG7 | NM_004890 | 0.3614109 | 0.0433 | 2.0518867 | -3.97931 | 0.4133282 |
| ITFG1 | AF212247 | 0.1725725 | 0.00578 | 2.8335755 | -2.32033 | 0.4126614 |
| BLOC1S5 | AK025442 | 0.3764415 | 0.0487 | 2.0007773 | -4.05677 | 0.4122865 |
| SLC25A15 | NM_014252 | 0.3370512 | 0.0368 | 2.1226417 | -3.84728 | 0.4122157 |
| ALDH1L2 | AF086109 | 0.3249955 | 0.0334 | 2.1637595 | -3.76845 | 0.411788 |
| PHTF2 | AL050389 | 0.2453463 | 0.0149 | 2.4868594 | -3.11211 | 0.4109922 |
| TPP2 | NM_003291 | 0.3327453 | 0.0357 | 2.1352987 | -3.82436 | 0.4099403 |
| ZNF692 | NM_017865 | 0.291334 | 0.0236 | 2.3055789 | -3.49115 | 0.4098727 |
| HAUS5 | AB020648 | 0.3194439 | 0.0318 | 2.1835322 | -3.73226 | 0.4094589 |
| NRGN | NM_006176 | 0.3194439 | 0.0317 | 2.1853979 | -3.72866 | 0.4093182 |
| SMPD1 | NM_000543 | 0.3614109 | 0.0432 | 2.0531161 | -3.97707 | 0.4084725 |
| MTRR | NM_002454 | 0.2446803 | 0.0147 | 2.4906125 | -3.104 | 0.4081633 |
| WBSCR22 | NM_017528 | 0.3609133 | 0.043 | 2.0548254 | -3.97395 | 0.4078731 |
| CLTB | NM_007097 | 0.3194439 | 0.0316 | 2.1869631 | -3.7248 | 0.4078032 |
| FJX1 | NM_014344 | 0.3452894 | 0.0388 | 2.0992347 | -3.89203 | 0.4076748 |
| BLOC1S4 | NM_018366 | 0.2599509 | 0.0172 | 2.4321703 | -3.22905 | 0.4072762 |
| GRINA | U44954 | 0.3773425 | 0.0492 | 1.9965198 | -4.07911 | 0.4064548 |
| C1orf109 | NM_017850 | 0.313933 | 0.03 | 2.2083268 | -3.68421 | 0.4062162 |
| KLHL13 | AB037730 | 0.3144522 | 0.0302 | 2.2046931 | -3.69128 | 0.4055567 |
| ZSCAN29 | AK023525 | 0.2899332 | 0.0233 | 2.3106846 | -3.48081 | 0.4053779 |
| RNF113A | NM_006978 | 0.2620958 | 0.0174 | 2.4270187 | -3.22875 | 0.4041714 |
| DLD | NM_000108 | 0.3706609 | 0.0463 | 2.0225143 | -4.03256 | 0.4037442 |
| C2orf47 | AK026208 | 0.2479891 | 0.0152 | 2.4784573 | -3.13022 | 0.4036773 |
| HCN3 | AB040968 | 0.3278751 | 0.0341 | 2.1547137 | -3.78751 | 0.4035647 |
| LAMTOR2 | NM_014017 | 0.3508149 | 0.0402 | 2.0846194 | -3.91916 | 0.4033957 |
| HEMK1 | NM_016173 | 0.3706609 | 0.0463 | 2.0231004 | -4.0315 | 0.402716 |
| TRIM41 | AL137593 | 0.2169791 | 0.0106 | 2.6160571 | -2.82711 | 0.4025179 |
| TAX1BP1 | NM_006024 | 0.3508149 | 0.0402 | 2.0848608 | -3.91872 | 0.4025075 |
| CYB5R1 | NM_016243 | 0.3435153 | 0.0382 | 2.1064313 | -3.87861 | 0.4020027 |
| CSDE1 | NM_007158 | 0.2446332 | 0.0147 | 2.4923901 | -3.10016 | 0.4016949 |
| RAB39A | X99962 | 0.3040447 | 0.0263 | 2.262819 | -3.57693 | 0.401369 |
| MKS1 | NM_017777 | 0.3207744 | 0.0325 | 2.1752549 | -3.7482 | 0.4003553 |
| COTL1 | L08436 | 0.3688275 | 0.0459 | 2.0270001 | -4.02447 | 0.4002337 |
| SFRP2 | AF311912 | 0.3201262 | 0.0321 | 2.1809101 | -3.73647 | 0.4000715 |
| ORC2 | NM_006190 | 0.3438323 | 0.0384 | 2.1042395 | -3.8827 | 0.4000595 |
| CACNA1C | AL359562 | 0.2905662 | 0.0234 | 2.3098213 | -3.48196 | 0.3993029 |
| KIAA1462 | AB040895 | 0.3355812 | 0.0364 | 2.1268492 | -3.84031 | 0.3992266 |
| C14orf169 | AK025455 | 0.3557339 | 0.0413 | 2.0733412 | -3.93896 | 0.3989385 |
| MCTS1 | NM_014060 | 0.2643568 | 0.018 | 2.4125923 | -3.27037 | 0.3980843 |
| SCAPER | AB040887 | 0.2484966 | 0.0153 | 2.4756078 | -3.13635 | 0.3978682 |
| MIF4GD | AF225422 | 0.3038871 | 0.0261 | 2.2660296 | -3.57054 | 0.3968888 |
| EDEM2 | NM_018217 | 0.3280313 | 0.0344 | 2.150731 | -3.79509 | 0.3968317 |
| PRKRA | NM_003690 | 0.3119209 | 0.0293 | 2.2178579 | -3.66561 | 0.3965987 |
| CXCL14 | NM_004887 | 0.302174 | 0.0254 | 2.2768797 | -3.54888 | 0.3965483 |
| DHX32 | NM_018180 | 0.3566103 | 0.0416 | 2.0697056 | -3.94667 | 0.3960145 |
| PAXIP1 | U80735 | 0.2602754 | 0.0172 | 2.4317198 | -3.21885 | 0.3958929 |
| UTP15 | AK022849 | 0.2436881 | 0.0145 | 2.4979512 | -3.08793 | 0.3942509 |
| MCM8 | AK022738 | 0.3448075 | 0.0387 | 2.1012378 | -3.8883 | 0.3938481 |
| HIPK2 | NM_014075 | 0.3580951 | 0.0422 | 2.064122 | -3.95589 | 0.3913187 |
| POP4 | NM_006627 | 0.2484966 | 0.0154 | 2.4748651 | -3.13794 | 0.3892727 |
| HNRNPL | NM_001533 | 0.2816924 | 0.0221 | 2.3325618 | -3.4363 | 0.3875569 |
| RFXANK | NM_003721 | 0.3755392 | 0.0485 | 2.0030461 | -4.06634 | 0.3867667 |
| SPDEF | NM_012391 | 0.3575195 | 0.0419 | 2.0668881 | -3.95082 | 0.386253 |
| AP3B1 | NM_003664 | 0.3479119 | 0.0394 | 2.0929887 | -3.90365 | 0.3859755 |
| HOXB13 | U81599 | 0.3583478 | 0.0422 | 2.0636185 | -3.95681 | 0.3859718 |
| MCPH1 | AK022909 | 0.303108 | 0.0258 | 2.2706307 | -3.56136 | 0.3859568 |
| MRPS7 | NM_015971 | 0.3360897 | 0.0366 | 2.1250595 | -3.84368 | 0.3855295 |
| GNAT1 | NM_000172 | 0.3712044 | 0.0466 | 2.0202871 | -4.03657 | 0.3837042 |
| NEK6 | NM_014397 | 0.3085383 | 0.0276 | 2.2421351 | -3.61792 | 0.3828477 |
| HAS3 | NM_005329 | 0.360428 | 0.0429 | 2.0558012 | -3.97216 | 0.3813836 |
| ADPRHL2 | NM_017825 | 0.3115609 | 0.0292 | 2.218854 | -3.66366 | 0.3802727 |
| MAFG | NM_002359 | 0.3309551 | 0.035 | 2.1437124 | -3.80843 | 0.3800608 |
| FBXO4 | NM_018007 | 0.2794145 | 0.0213 | 2.3473773 | -3.40595 | 0.3799646 |
| ATL3 | AL117600 | 0.3773425 | 0.0491 | 1.9969073 | -4.07842 | 0.3792746 |
| CEP104 | NM_014704 | 0.3205045 | 0.0323 | 2.178401 | -3.74129 | 0.3790852 |
| LRRC8D | NM_018103 | 0.3280313 | 0.0344 | 2.1514052 | -3.79381 | 0.3788756 |
| CD1B | NM_001764 | 0.3675937 | 0.0455 | 2.0310272 | -4.01611 | 0.3784515 |
| CLEC11A | NM_002975 | 0.3712602 | 0.0467 | 2.0188505 | -4.03915 | 0.3784477 |
| RNF219 | AK023511 | 0.3693438 | 0.046 | 2.0259143 | -4.02643 | 0.3782528 |
| RUNX3 | NM_004350 | 0.367254 | 0.0454 | 2.0329813 | -4.0103 | 0.3768019 |
| BMP15 | NM_005448 | 0.2170877 | 0.0107 | 2.6126511 | -2.83478 | 0.3758229 |
| WDR12 | NM_018256 | 0.3566103 | 0.0415 | 2.0703776 | -3.94544 | 0.3758008 |
| ZNF510 | NM_014930 | 0.2906127 | 0.0235 | 2.3071099 | -3.48805 | 0.3743989 |
| ARHGAP35 | NM_004491 | 0.3761225 | 0.0487 | 2.0009708 | -4.07118 | 0.3733286 |
| ORMDL1 | NM_016467 | 0.3085383 | 0.0277 | 2.2410059 | -3.62015 | 0.371221 |
| SUPV3L1 | NM_003171 | 0.3141369 | 0.0302 | 2.2056549 | -3.68941 | 0.3689006 |
| LRP12 | NM_013437 | 0.3381006 | 0.037 | 2.1202289 | -3.85181 | 0.3687239 |
| IFT172 | AL110218 | 0.2599033 | 0.0171 | 2.4338046 | -3.22559 | 0.3683279 |
| DDC | NM_000790 | 0.3738079 | 0.0474 | 2.0132658 | -4.03452 | 0.3679022 |
| CHMP4C | AK000049 | 0.3438323 | 0.0384 | 2.1042394 | -3.8827 | 0.3667052 |
| ARHGEF12 | AF090906 | 0.303332 | 0.0258 | 2.2696542 | -3.56331 | 0.3665866 |
| MAPK7 | NM_002749 | 0.3203134 | 0.0322 | 2.1788852 | -3.74121 | 0.3655003 |
| C17orf80 | NM_017941 | 0.3441857 | 0.0385 | 2.1029235 | -3.88516 | 0.3644796 |
| ANXA4 | NM_001153 | 0.3755225 | 0.0484 | 2.0035124 | -4.06664 | 0.3636328 |
| DPY19L2P2 | AL049437 | 0.3473364 | 0.0392 | 2.0947244 | -3.90042 | 0.3635088 |
| MIS18BP1 | NM_018353 | 0.3438323 | 0.0383 | 2.1047926 | -3.88167 | 0.3632019 |
| GTF2F2 | NM_004128 | 0.3039961 | 0.0261 | 2.2653305 | -3.57124 | 0.3619032 |
| RAP1GDS1 | AF215923 | 0.2656183 | 0.0188 | 2.3974458 | -3.30215 | 0.3618615 |
| KCTD20 | AL049282 | 0.3291297 | 0.0347 | 2.1469078 | -3.80236 | 0.3613385 |
| FNIP2 | AB040883 | 0.3614109 | 0.0433 | 2.0519597 | -3.97918 | 0.3609656 |
| CLPP | NM_006012 | 0.313933 | 0.0301 | 2.2066873 | -3.6874 | 0.3599278 |
| KLF8 | NM_007250 | 0.3774739 | 0.0495 | 1.9930297 | -4.08532 | 0.3597608 |
| CLDN11 | AJ245902 | 0.3318914 | 0.0354 | 2.138974 | -3.81741 | 0.3589648 |
| ZFYVE21 | AK001921 | 0.3575256 | 0.042 | 2.0655829 | -3.95425 | 0.3572243 |
| FOXJ2 | NM_018416 | 0.2905662 | 0.0235 | 2.3079383 | -3.48637 | 0.3558233 |
| LOC100505874 | AK021893 | 0.3738079 | 0.0474 | 2.0124054 | -4.05073 | 0.3539981 |
| NLRP2 | NM_017852 | 0.3577372 | 0.0421 | 2.0645699 | -3.95611 | 0.3535457 |
| NDUFAF5 | AK025977 | 0.3194439 | 0.0318 | 2.1844016 | -3.73058 | 0.3531711 |
| CARS | AF288207 | 0.3645243 | 0.0448 | 2.0375937 | -4.00531 | 0.3530622 |
| POLD4 | AF179890 | 0.3626634 | 0.044 | 2.0455606 | -3.99084 | 0.3511308 |
| LIMK1 | NM_002314 | 0.3150436 | 0.0304 | 2.2025835 | -3.69538 | 0.3511148 |
| TBC1D19 | NM_018317 | 0.3280313 | 0.0345 | 2.1498359 | -3.7959 | 0.3506343 |
| SV2B | NM_014848 | 0.3479119 | 0.0395 | 2.0919654 | -3.90555 | 0.3504263 |
| FBXO18 | AL133069 | 0.3567936 | 0.0417 | 2.0684698 | -3.94895 | 0.3500408 |
| ATPAF2 | AF070584 | 0.3327453 | 0.0357 | 2.1355501 | -3.82389 | 0.3497737 |
| MRFAP1L1 | AF155654 | 0.3532978 | 0.0408 | 2.0785099 | -3.93045 | 0.348532 |
| ZNF207 | NM_003457 | 0.3085383 | 0.0277 | 2.2410066 | -3.62015 | 0.3474628 |
| HNRNPD | NM_002138 | 0.3626634 | 0.044 | 2.0449612 | -3.99193 | 0.3469346 |
| FANCF | AF181994 | 0.3479607 | 0.0396 | 2.0912313 | -3.90691 | 0.3441165 |
| SLC25A12 | NM_003705 | 0.3722816 | 0.047 | 2.0162852 | -4.04376 | 0.342901 |
| TTC31 | AK022850 | 0.3591218 | 0.0424 | 2.0613018 | -3.9621 | 0.3428052 |
| TRAPPC3 | NM_014408 | 0.3145556 | 0.0303 | 2.2036887 | -3.69323 | 0.3422984 |
| NSUN2 | NM_017755 | 0.3751013 | 0.0482 | 2.0050079 | -4.06397 | 0.3416035 |
| BTBD1 | NM_017942 | 0.371507 | 0.0468 | 2.0178971 | -4.04087 | 0.3409691 |
| ELK1 | AF000672 | 0.3109985 | 0.0287 | 2.2257763 | -3.65011 | 0.3407777 |
| DLX4 | NM_001934 | 0.3554176 | 0.0412 | 2.0737249 | -3.93928 | 0.3407752 |
| SLC14A1 | NM_015865 | 0.3327453 | 0.0357 | 2.1346576 | -3.82557 | 0.3391941 |
| TM4SF20 | AK026453 | 0.3478862 | 0.0394 | 2.0932483 | -3.90316 | 0.3378964 |
| PSPC1 | NM_018282 | 0.3665028 | 0.0452 | 2.0339846 | -4.01185 | 0.3355999 |
| CINP | NM_016550 | 0.3516452 | 0.0405 | 2.0812883 | -3.92532 | 0.3283988 |
| ZNF74 | NM_003426 | 0.3244949 | 0.0331 | 2.1669216 | -3.76331 | 0.3236865 |
| LOC149351 | AK022898 | 0.3572139 | 0.0418 | 2.0677623 | -3.94921 | 0.3196459 |
| SACM1L | NM_014016 | 0.3749151 | 0.0482 | -2.0055163 | -4.06306 | -0.2931017 |
| ACTN1 | NM_001102 | 0.3712044 | 0.0466 | -2.0199718 | -4.03714 | -0.3215254 |
| SLC6A12 | NM_003044 | 0.3749151 | 0.0482 | -2.0059141 | -4.06122 | -0.3232847 |
| NOS1AP | AB007933 | 0.3165437 | 0.0308 | -2.1969951 | -3.70623 | -0.3336276 |
| RXRB | X65463 | 0.3566103 | 0.0417 | -2.0690776 | -3.94783 | -0.3364917 |
| TSNAX-DISC1 | AF230316 | 0.3626634 | 0.044 | -2.0453366 | -3.99125 | -0.3368571 |
| ATG16L1 | NM_017974 | 0.3722816 | 0.047 | -2.0169869 | -4.02686 | -0.3391572 |
| BRSK2 | AF020089 | 0.3656227 | 0.045 | -2.0358548 | -4.00846 | -0.3413023 |
| RAPGEF6 | NM_016340 | 0.3645243 | 0.0448 | -2.0375522 | -4.00538 | -0.3435723 |
| INIP | AF161411 | 0.3515263 | 0.0404 | -2.081882 | -3.92423 | -0.3461668 |
| MAPKAPK3 | NM_004635 | 0.3448075 | 0.0386 | -2.1014618 | -3.88788 | -0.3464318 |
| CRYGB | NM_005210 | 0.3452894 | 0.0389 | -2.0990957 | -3.89229 | -0.3495297 |
| TIMM10 | NM_012456 | 0.3751013 | 0.0482 | -2.004927 | -4.06411 | -0.3501282 |
| RBM22 | NM_018047 | 0.3566103 | 0.0415 | -2.0708471 | -3.94457 | -0.3518352 |
| TRADD | L41690 | 0.3681749 | 0.0457 | -2.0286286 | -4.02153 | -0.3521018 |
| IL36G | NM_019618 | 0.327424 | 0.034 | -2.1563153 | -3.78445 | -0.3532033 |
| TRAK1 | NM_014965 | 0.3396034 | 0.0374 | -2.1156299 | -3.86044 | -0.3599708 |
| CASZ1 | NM_017766 | 0.3777714 | 0.0498 | -1.9907712 | -4.08818 | -0.3627161 |
| CPXM1 | NM_019609 | 0.3040447 | 0.0263 | -2.2625885 | -3.57739 | -0.3628253 |
| TBC1D28 | AL137747 | 0.3645243 | 0.0447 | -2.0382729 | -4.00408 | -0.3634903 |
| NODAL | NM_018055 | 0.3272746 | 0.0339 | -2.1575499 | -3.78121 | -0.3642255 |
| JTB | NM_006694 | 0.343986 | 0.0385 | -2.1033881 | -3.88429 | -0.365231 |
| CHTOP | NM_015607 | 0.3244949 | 0.0331 | -2.166542 | -3.76491 | -0.3662695 |
| EFHC1 | NM_018100 | 0.3010319 | 0.0251 | -2.2812962 | -3.54003 | -0.3663814 |
| APC | S67789 | 0.3038871 | 0.026 | -2.2668564 | -3.55625 | -0.3667378 |
| FGF22 | AB021925 | 0.3401834 | 0.0376 | -2.1136234 | -3.86516 | -0.3705675 |
| DYNLT1 | NM_006519 | 0.3642508 | 0.0446 | -2.0391402 | -4.0025 | -0.3734515 |
| SEC22B | AK023270 | 0.3557339 | 0.0413 | -2.0731278 | -3.94038 | -0.374807 |
| OMP | NM_006189 | 0.3589156 | 0.0424 | -2.0617708 | -3.96124 | -0.3750758 |
| MGC70870 | D16888 | 0.3124692 | 0.0297 | -2.2127879 | -3.67551 | -0.3755196 |
| SIGLEC8 | NM_014442 | 0.3115634 | 0.0292 | -2.2186146 | -3.66413 | -0.3759402 |
| PHF1 | NM_002636 | 0.3043244 | 0.0264 | -2.2602055 | -3.58213 | -0.3762766 |
| RAB11FIP3 | NM_014700 | 0.3515263 | 0.0404 | -2.0819767 | -3.92405 | -0.3800815 |
| RBM7 | NM_016090 | 0.3518277 | 0.0405 | -2.0807098 | -3.92639 | -0.3801056 |
| VPS50 | NM_017667 | 0.3345979 | 0.0362 | -2.1294795 | -3.83442 | -0.3803651 |
| ZDHHC2 | NM_016353 | 0.3739709 | 0.0475 | -2.012 | -4.05145 | -0.3808033 |
| ZFYVE9 | NM_004799 | 0.305155 | 0.0267 | -2.2560679 | -3.59035 | -0.3808184 |
| F11R | NM_016946 | 0.3246296 | 0.0332 | -2.165522 | -3.76686 | -0.3814812 |
| TMEM55B | AL137727 | 0.3317432 | 0.0352 | -2.1408076 | -3.81393 | -0.3815552 |
| PSORS1C1 | NM_014068 | 0.311454 | 0.0292 | -2.2197044 | -3.662 | -0.3820079 |
| SPAG5-AS1 | AL157421 | 0.3773425 | 0.0494 | -1.9944042 | -4.08287 | -0.3821242 |
| SLC22A7 | NM_006672 | 0.363318 | 0.0444 | -2.0417414 | -3.99778 | -0.3826461 |
| PLEKHG3 | AB011171 | 0.3566103 | 0.0417 | -2.0690873 | -3.94781 | -0.3829314 |
| SNU13 | NM_005008 | 0.3321963 | 0.0355 | -2.1375614 | -3.82008 | -0.3829687 |
| STARD10 | AF151810 | 0.3774739 | 0.0496 | -1.9925163 | -4.08623 | -0.3840288 |
| ADAM28 | NM_014265 | 0.2763447 | 0.0207 | -2.357955 | -3.38417 | -0.3843506 |
| C11orf54 | NM_014039 | 0.3645243 | 0.0448 | -2.037647 | -4.00521 | -0.3855466 |
| KIF3C | NM_002254 | 0.3194439 | 0.0318 | -2.1836462 | -3.73204 | -0.3858571 |
| CWC25 | NM_017748 | 0.3124692 | 0.0294 | -2.2160128 | -3.66922 | -0.3859332 |
| DGCR11 | L77561 | 0.3738079 | 0.0474 | -2.0123783 | -4.05077 | -0.3864014 |
| EDEM1 | NM_014674 | 0.3770675 | 0.0489 | -2.0000651 | -4.05594 | -0.3868715 |
| BMPR1A | NM_004329 | 0.350072 | 0.0399 | -2.0873689 | -3.91407 | -0.3869038 |
| LAG3 | NM_002286 | 0.2283232 | 0.0124 | -2.5577421 | -2.95725 | -0.3896736 |
| U2AF1 | AK023589 | 0.324739 | 0.0334 | -2.1637652 | -3.77023 | -0.3899902 |
| C1orf21 | AF035282 | 0.3613235 | 0.0431 | -2.0538927 | -3.97565 | -0.3905697 |
| XPNPEP3 | AL365514 | 0.3280313 | 0.0343 | -2.1528149 | -3.79023 | -0.3912397 |
| CUEDC1 | NM_017949 | 0.3348034 | 0.0363 | -2.1284141 | -3.83736 | -0.3913246 |
| SH3BP5-AS1 | AL133111 | 0.3159474 | 0.0306 | -2.1997814 | -3.70082 | -0.3945505 |
| C2orf72 | AF086098 | 0.3280313 | 0.0345 | -2.1498329 | -3.7959 | -0.3960944 |
| FAM118A | NM_017911 | 0.3554176 | 0.0412 | -2.0738933 | -3.93897 | -0.3961624 |
| UBL3 | NM_007106 | 0.3124692 | 0.0297 | -2.212752 | -3.67558 | -0.3967893 |
| GPR6 | NM_005284 | 0.3436777 | 0.0382 | -2.106023 | -3.87937 | -0.3975177 |
| FGF14 | NM_004115 | 0.3773295 | 0.049 | -1.9980881 | -4.07631 | -0.4008581 |
| CYP4F12 | AB035130 | 0.3747788 | 0.0478 | -2.0086179 | -4.05751 | -0.4016553 |
| BIN3 | NM_018688 | 0.3675937 | 0.0455 | -2.0308019 | -4.0176 | -0.4017908 |
| HPCAL1 | NM_002149 | 0.3318914 | 0.0353 | -2.1399306 | -3.8156 | -0.4039639 |
| FLT1 | NM_002019 | 0.2283232 | 0.0123 | -2.5585484 | -2.95547 | -0.4044717 |
| DOCK4 | AF085922 | 0.3223169 | 0.0327 | -2.1731688 | -3.73878 | -0.4048429 |
| ACKR4 | NM_016557 | 0.3666929 | 0.0452 | -2.0336231 | -4.01142 | -0.4049461 |
| MYO5B | AB032945 | 0.2850785 | 0.0227 | -2.3217061 | -3.45843 | -0.4050626 |
| GDI1 | NM_001493 | 0.2845523 | 0.0225 | -2.3255846 | -3.45053 | -0.4052933 |
| AKT1 | NM_005163 | 0.3088677 | 0.0282 | -2.23353 | -3.63488 | -0.4059105 |
| ELF2 | NM_006874 | 0.3759926 | 0.0486 | -2.0016107 | -4.07003 | -0.4063146 |
| COL11A2 | J04974 | 0.3073227 | 0.027 | -2.2513253 | -3.59975 | -0.408385 |
| SPOCK2 | NM_014767 | 0.2954527 | 0.0241 | -2.2985738 | -3.50402 | -0.4085433 |
| PHTF1 | NM_006608 | 0.2794145 | 0.0212 | -2.3499315 | -3.38879 | -0.4087178 |
| LOC101928565 | AF085892 | 0.161646 | 0.00482 | -2.8965203 | -2.16755 | -0.40909 |
| WLS | AK026744 | 0.2935111 | 0.0239 | -2.3015142 | -3.49936 | -0.4091318 |
| CRK | NM_016823 | 0.3773425 | 0.0493 | -1.9952595 | -4.08135 | -0.4092336 |
| SULT1A1 | NM_001055 | 0.2845523 | 0.0225 | -2.32574 | -3.45022 | -0.4092403 |
| ERBB4 | AF007153 | 0.3706609 | 0.0464 | -2.0222769 | -4.03299 | -0.4099358 |
| TMCC3 | AB032971 | 0.3085383 | 0.0276 | -2.2418324 | -3.61852 | -0.4101035 |
| PPP1R12A | NM_002480 | 0.2656183 | 0.0187 | -2.398176 | -3.30062 | -0.4109359 |
| MAP4K4 | NM_004834 | 0.2835748 | 0.0224 | -2.3277478 | -3.44612 | -0.4110737 |
| ST6GALNAC2 | NM_006456 | 0.3318914 | 0.0354 | -2.1390219 | -3.81732 | -0.4119829 |
| AQP5 | NM_001651 | 0.3759926 | 0.0486 | -2.0018557 | -4.06847 | -0.4122955 |
| CSF3 | NM_000759 | 0.1885217 | 0.00722 | -2.7545681 | -2.50825 | -0.4127445 |
| YPEL3 | AK022409 | 0.3774739 | 0.0497 | -1.9922639 | -4.08553 | -0.4134189 |
| IHH | L38517 | 0.34431 | 0.0386 | -2.1023587 | -3.88621 | -0.4137887 |
| LRRC20 | NM_018239 | 0.3243113 | 0.0331 | -2.1674758 | -3.76312 | -0.4138964 |
| LOC103344931 | AL049387 | 0.3712602 | 0.0468 | -2.0185039 | -4.03978 | -0.4195289 |
| ALDH16A1 | AK024182 | 0.3749151 | 0.048 | -2.0070444 | -4.06033 | -0.420083 |
| MAD2L1 | NM_002358 | 0.361962 | 0.0435 | -2.0504464 | -3.98088 | -0.4215881 |
| GNL3 | NM_014366 | 0.3712044 | 0.0466 | -2.0202365 | -4.03666 | -0.422095 |
| METTL13 | NM_015935 | 0.359785 | 0.0427 | -2.0582133 | -3.96775 | -0.4224295 |
| JMJD1C | AB037801 | 0.3777714 | 0.0498 | -1.9907656 | -4.08934 | -0.4228385 |
| LOC100129503 | AF218021 | 0.3413368 | 0.0378 | -2.1116857 | -3.8658 | -0.4245734 |
| CCR5 | NM_000579 | 0.3533006 | 0.0408 | -2.0787089 | -3.92907 | -0.4252127 |
| TSHB | NM_000549 | 0.3626634 | 0.0438 | -2.0480864 | -3.98408 | -0.4254771 |
| USP48 | NM_018391 | 0.3010319 | 0.0251 | -2.2819114 | -3.5388 | -0.425799 |
| PPP2R3B | NM_013239 | 0.3626634 | 0.0438 | -2.0469784 | -3.98826 | -0.4265481 |
| KTI12///TXNDC12 | L31901 | 0.2272024 | 0.0122 | -2.5627354 | -2.94621 | -0.4267685 |
| BOK | AF174487 | 0.3088677 | 0.0281 | -2.235197 | -3.63085 | -0.4267846 |
| SNX2 | NM_003100 | 0.3043244 | 0.0265 | -2.2589817 | -3.58456 | -0.4280621 |
| KLK12 | NM_019598 | 0.3626634 | 0.0439 | -2.0465767 | -3.98899 | -0.4290835 |
| UBR4 | AK024105 | 0.3345979 | 0.0362 | -2.130102 | -3.83228 | -0.4314371 |
| PIGX | AK000529 | 0.2816924 | 0.0221 | -2.3327582 | -3.4359 | -0.4322824 |
| LINC00939 | AL137270 | 0.3120035 | 0.0293 | -2.2173238 | -3.66666 | -0.4329433 |
| GCNT3 | NM_004751 | 0.3628601 | 0.0441 | -2.0440744 | -3.99354 | -0.433038 |
| ONECUT1 | U96173 | 0.2484966 | 0.0154 | -2.4736462 | -3.14056 | -0.433473 |
| BTG1 | NM_001731 | 0.3039474 | 0.0261 | -2.2655095 | -3.57157 | -0.4335692 |
| HSPC324 | AF161442 | 0.3281211 | 0.0345 | -2.1495023 | -3.79653 | -0.433882 |
| UBXN2B | U79282 | 0.2647025 | 0.0183 | -2.4061055 | -3.284 | -0.4339895 |
| MAP2 | NM_002374 | 0.359785 | 0.0427 | -2.0588847 | -3.96548 | -0.4348433 |
| FBRSL1 | AB046765 | 0.3030424 | 0.0257 | -2.2713258 | -3.55998 | -0.4351106 |
| CYB5D1 | AK022811 | 0.3006448 | 0.0248 | -2.2859896 | -3.53062 | -0.4352264 |
| FAM214A | AB037791 | 0.2845523 | 0.0226 | -2.3241684 | -3.45342 | -0.4376592 |
| TRPM5 | AF177473 | 0.3457326 | 0.0389 | -2.0981374 | -3.89407 | -0.4378094 |
| RNF166 | AK026860 | 0.3193544 | 0.0314 | -2.1891253 | -3.72146 | -0.4379691 |
| EPHB4 | NM_004444 | 0.3110118 | 0.0288 | -2.2243304 | -3.65294 | -0.4380213 |
| PNISR | AL080186 | 0.3085383 | 0.0279 | -2.2379549 | -3.62616 | -0.4386828 |
| GMDS-AS1 | AK023629 | 0.3712044 | 0.0465 | -2.0206877 | -4.03585 | -0.438761 |
| KCNQ1DN | AB039920 | 0.3088603 | 0.0281 | -2.2356001 | -3.6308 | -0.439051 |
| MKRN1 | NM_013446 | 0.3280313 | 0.0342 | -2.1536094 | -3.78961 | -0.4391101 |
| DUSP6 | NM_001946 | 0.3205045 | 0.0324 | -2.1764102 | -3.74597 | -0.4404717 |
| CEP85L | AL133101 | 0.2809852 | 0.0219 | -2.3369988 | -3.42723 | -0.441096 |
| MYOZ3 | AF052497 | 0.3566103 | 0.0416 | -2.0704853 | -3.93008 | -0.4413391 |
| PHF23 | AK026537 | 0.2905662 | 0.0235 | -2.3080148 | -3.48622 | -0.4414041 |
| KLHL41 | NM_006063 | 0.2594182 | 0.017 | -2.4357776 | -3.2214 | -0.4414118 |
| DDX6 | AK021715 | 0.1914137 | 0.00745 | -2.7436452 | -2.53388 | -0.4414258 |
| STRN | AF085351 | 0.314285 | 0.0302 | -2.2052012 | -3.69029 | -0.441446 |
| TMEM204 | AK024551 | 0.3110118 | 0.029 | -2.2228135 | -3.65591 | -0.4415766 |
| LOC284561 | AK023809 | 0.2626411 | 0.0176 | -2.4216799 | -3.25123 | -0.4429168 |
| MBP | NM_002385 | 0.246904 | 0.0151 | -2.482237 | -3.12208 | -0.4436295 |
| TMC7 | AF088010 | 0.3365039 | 0.0367 | -2.1243796 | -3.84402 | -0.443981 |
| KIAA0430 | AB007890 | 0.2643568 | 0.0179 | -2.41548 | -3.26429 | -0.4440163 |
| FRMD4B | AB023230 | 0.3749151 | 0.0482 | -2.0056772 | -4.06277 | -0.444585 |
| PLEKHS1 | AK027190 | 0.3450765 | 0.0388 | -2.1000597 | -3.89049 | -0.4447636 |
| CFAP45 | NM_012337 | 0.313933 | 0.0301 | -2.207374 | -3.68606 | -0.4450883 |
| PRO0471 | AF111846 | 0.2019304 | 0.00845 | -2.6981154 | -2.63985 | -0.4454819 |
| SNTG1 | NM_018967 | 0.2743887 | 0.0203 | -2.3666092 | -3.3663 | -0.4458566 |
| TMX4 | AB032988 | 0.2516217 | 0.0158 | -2.464813 | -3.15951 | -0.4460904 |
| SPEG | NM_005876 | 0.324739 | 0.0333 | -2.1639945 | -3.76979 | -0.4469933 |
| CYLC1 | Z22780 | 0.3688275 | 0.0459 | -2.0269061 | -4.02464 | -0.4470249 |
| MAN2A2 | NM_018621 | 0.3549118 | 0.0411 | -2.07474 | -3.93741 | -0.4473213 |
| ZZEF1 | AB007859 | 0.2647025 | 0.0184 | -2.4045352 | -3.2873 | -0.4482288 |
| CC2D2B | AK022203 | 0.2571992 | 0.0167 | -2.4436715 | -3.20464 | -0.4485898 |
| CMKLR1 | NM_004072 | 0.3164281 | 0.0307 | -2.19835 | -3.70279 | -0.4488466 |
| PBX2 | NM_002586 | 0.3085383 | 0.0275 | -2.2445795 | -3.61236 | -0.4492857 |
| HIST1H3A | NM_003529 | 0.313933 | 0.0301 | -2.2065178 | -3.68773 | -0.4495673 |
| RUFY2 | NM_017987 | 0.3773425 | 0.0493 | -1.99547 | -4.08098 | -0.4495936 |
| NFE2L1 | U08853 | 0.3262899 | 0.0337 | -2.1602358 | -3.77609 | -0.4497234 |
| DLG4 | NM_001365 | 0.3043244 | 0.0265 | -2.2586296 | -3.58526 | -0.4510994 |
| F2RL1 | NM_005242 | 0.3085383 | 0.0279 | -2.2382667 | -3.62555 | -0.4520345 |
| CLDN16 | NM_006580 | 0.3246594 | 0.0333 | -2.1650862 | -3.7677 | -0.4521278 |
| ARHGAP42 | AF143327 | 0.2925605 | 0.0238 | -2.3036153 | -3.4945 | -0.4521314 |
| OAZ1 | NM_004152 | 0.2320508 | 0.0131 | -2.5356135 | -3.00599 | -0.4522983 |
| SIPA1L1 | NM_015556 | 0.16832 | 0.00539 | -2.8581642 | -2.26097 | -0.452583 |
| NUP50 | NM_007172 | 0.3085383 | 0.0277 | -2.2408527 | -3.61971 | -0.4527654 |
| BACH1 | NM_001186 | 0.3729978 | 0.0472 | -2.0148258 | -4.04638 | -0.4529421 |
| ABAT | NM_000663 | 0.1725683 | 0.00576 | -2.8347061 | -2.31761 | -0.4538921 |
| ZMYM3 | NM_005096 | 0.3454668 | 0.0389 | -2.0986713 | -3.89308 | -0.455162 |
| HMGB2 | X62534 | 0.3706609 | 0.0463 | -2.0227576 | -4.03212 | -0.4575956 |
| NFAT5 | AK001067 | 0.3029261 | 0.0256 | -2.2724324 | -3.55777 | -0.4581018 |
| PACSIN2 | NM_007229 | 0.2188274 | 0.0109 | -2.6060008 | -2.84973 | -0.4586127 |
| STX3 | NM_004177 | 0.3598596 | 0.0428 | -2.0576612 | -3.96876 | -0.4590971 |
| HOXC13 | NM_017410 | 0.2484966 | 0.0154 | -2.4747605 | -3.13817 | -0.4602239 |
| GPIHBP1 | AF088057 | 0.2809852 | 0.0218 | -2.3373701 | -3.42647 | -0.4605523 |
| DIO2 | NM_013989 | 0.2435112 | 0.0145 | -2.4979187 | -3.08819 | -0.4607803 |
| GNG7 | AK024465 | 0.3575195 | 0.0419 | -2.0665635 | -3.95141 | -0.4616235 |
| LUZP1 | AK025345 | 0.3124692 | 0.0296 | -2.2131378 | -3.67483 | -0.4618861 |
| OLFML2B | AL050137 | 0.3489952 | 0.0398 | -2.0890244 | -3.911 | -0.462844 |
| CLCN4 | NM_001830 | 0.3702521 | 0.0462 | -2.0246459 | -4.02762 | -0.4636488 |
| OGFRL1 | AK024732 | 0.3085383 | 0.0278 | -2.2390766 | -3.62395 | -0.4638745 |
| BBC3 | U82987 | 0.2267728 | 0.0119 | -2.5708368 | -2.92824 | -0.4641876 |
| C11orf86 | AK026328 | 0.2727714 | 0.02 | -2.3718726 | -3.3554 | -0.4642963 |
| CARD9 | AF311287 | 0.2545873 | 0.0163 | -2.4519766 | -3.18695 | -0.4645924 |
| MOS | NM_005372 | 0.3321045 | 0.0354 | -2.138189 | -3.81889 | -0.4646166 |
| SERHL2 | NM_014509 | 0.185185 | 0.00701 | -2.7649693 | -2.48376 | -0.4656883 |
| HNRNPM | AK022050 | 0.3321963 | 0.0355 | -2.1388955 | -3.81369 | -0.4659027 |
| POU3F3 | NM_006236 | 0.209542 | 0.00949 | -2.6557045 | -2.73723 | -0.4666206 |
| ABCC5 | AL359600 | 0.2545873 | 0.0162 | -2.4537434 | -3.18318 | -0.4668391 |
| GPR22 | NM_005295 | 0.3194439 | 0.0315 | -2.1878768 | -3.72388 | -0.4669818 |
| KIAA1109 | AL137254 | 0.3700237 | 0.0461 | -2.0249182 | -4.02823 | -0.4670677 |
| EPS15 | NM_001981 | 0.2484966 | 0.0155 | -2.4725953 | -3.14282 | -0.4671099 |
| TRERF1 | AJ277276 | 0.2718275 | 0.0197 | -2.3780274 | -3.34216 | -0.4681939 |
| TSPOAP1-AS1 | AK000271 | 0.3773425 | 0.0494 | -1.9943909 | -4.0829 | -0.4682327 |
| FAAH | NM_001441 | 0.2689628 | 0.0192 | -2.3886611 | -3.32005 | -0.4684175 |
| RAPGEF2 | NM_014247 | 0.3327453 | 0.0357 | -2.1348039 | -3.8253 | -0.4684567 |
| ACTN1 | AK022224 | 0.3479119 | 0.0395 | -2.0920678 | -3.90536 | -0.4686113 |
| HAX1 | NM_006118 | 0.1699968 | 0.00551 | -2.8501287 | -2.28042 | -0.468839 |
| NLGN3 | NM_018977 | 0.2773083 | 0.0209 | -2.3552029 | -3.38985 | -0.4695191 |
| CDH23 | AY010111 | 0.3327453 | 0.0358 | -2.1337718 | -3.82725 | -0.4695943 |
| CCL8 | NM_005623 | 0.359785 | 0.0427 | -2.0595652 | -3.96204 | -0.4696004 |
| MAGEB4 | NM_002367 | 0.3745212 | 0.0476 | -2.0109793 | -4.05328 | -0.4701488 |
| MEGF6 | AB011539 | 0.16832 | 0.00538 | -2.8586424 | -2.25981 | -0.4701597 |
| RELL1 | AK025431 | 0.3115609 | 0.0292 | -2.2191326 | -3.66312 | -0.4701889 |
| MMP27 | AF195192 | 0.228628 | 0.0125 | -2.5531662 | -2.96736 | -0.4703751 |
| TUBGCP5 | AK023934 | 0.302174 | 0.0254 | -2.2776129 | -3.54674 | -0.4705682 |
| SCUBE2 | AF086390 | 0.37702 | 0.0488 | -1.9993663 | -4.07404 | -0.471 |
| ARHGAP31 | AB033030 | 0.3010319 | 0.0251 | -2.282826 | -3.53492 | -0.4712067 |
| DDX5 | AL133585 | 0.3746162 | 0.0477 | -2.0102159 | -4.05465 | -0.4713059 |
| HYPM | AF049615 | 0.3774739 | 0.0497 | -1.9918349 | -4.08744 | -0.4734445 |
| NEU1 | NM_000434 | 0.2973659 | 0.0244 | -2.293065 | -3.5164 | -0.4740091 |
| KIF21B | NM_017596 | 0.2620958 | 0.0175 | -2.4248556 | -3.24452 | -0.4740139 |
| IRS2 | NM_003749 | 0.3772426 | 0.0489 | -1.9985557 | -4.07548 | -0.4742017 |
| SEC14L5 | AB007880 | 0.3489952 | 0.0398 | -2.0891245 | -3.91082 | -0.474437 |
| XRCC6 | AF052148 | 0.3110118 | 0.0289 | -2.224074 | -3.65344 | -0.474795 |
| PPP6R2 | NM_014678 | 0.2446803 | 0.0147 | -2.4913757 | -3.10235 | -0.4749202 |
| MTERF1 | NM_006980 | 0.2446332 | 0.0147 | -2.4924616 | -3.1 | -0.4750491 |
| EIF2AK2 | NM_002759 | 0.3748949 | 0.0479 | -2.0077561 | -4.05905 | -0.4751667 |
| GEN1 | AK025489 | 0.2643568 | 0.018 | -2.4131072 | -3.26929 | -0.4752269 |
| DCTN5 | AK022200 | 0.2828206 | 0.0222 | -2.3298158 | -3.44191 | -0.4755855 |
| IVNS1ABP | AK023363 | 0.3773425 | 0.0492 | -1.9964088 | -4.07817 | -0.4756128 |
| ATF6 | AK025316 | 0.3382709 | 0.0371 | -2.1185929 | -3.85584 | -0.4758686 |
| PNMAL2 | AB033009 | 0.3029261 | 0.0256 | -2.2734457 | -3.55574 | -0.47604 |
| RRM2B | AB036063 | 0.3038871 | 0.026 | -2.2673425 | -3.56792 | -0.4764416 |
| CCDC170 | AK026958 | 0.2643568 | 0.0181 | -2.4115679 | -3.27214 | -0.4766701 |
| SPG11 | AK025092 | 0.3090079 | 0.0283 | -2.2321788 | -3.63753 | -0.4771085 |
| PARP8 | AK024961 | 0.3073227 | 0.027 | -2.2507876 | -3.60081 | -0.4775275 |
| CHMP1B | NM_020412 | 0.2794639 | 0.0213 | -2.3465027 | -3.40774 | -0.4775401 |
| HIST1H4I | NM_003495 | 0.3626634 | 0.0439 | -2.0465431 | -3.98905 | -0.4775826 |
| EYA3 | AK022005 | 0.2195372 | 0.011 | -2.6027252 | -2.84781 | -0.4779479 |
| THSD1 | NM_018676 | 0.3085383 | 0.0275 | -2.2472697 | -3.59163 | -0.4780412 |
| FAM193A | NM_003704 | 0.3085383 | 0.0277 | -2.2415927 | -3.61899 | -0.4791451 |
| MFSD9 | U79290 | 0.3749151 | 0.048 | -2.0067762 | -4.06081 | -0.4792182 |
| PPARA | AF270490 | 0.3774739 | 0.0496 | -1.9922573 | -4.08669 | -0.479592 |
| MAP4K4 | NM_017792 | 0.2756699 | 0.0206 | -2.3609071 | -3.37808 | -0.4796056 |
| UHMK1 | AL137257 | 0.2905662 | 0.0234 | -2.3089317 | -3.48436 | -0.4797165 |
| STAT5A | NM_003152 | 0.2656183 | 0.0188 | -2.3975909 | -3.30184 | -0.480261 |
| SPSB4 | AF086345 | 0.3246296 | 0.0332 | -2.1655627 | -3.76679 | -0.4805535 |
| PPCDC | AF182419 | 0.3014722 | 0.0253 | -2.2784205 | -3.54579 | -0.480962 |
| ZNF552 | AK023769 | 0.303108 | 0.0258 | -2.2709651 | -3.56001 | -0.4811155 |
| PRUNE2 | AB002365 | 0.3283045 | 0.0346 | -2.1483973 | -3.79953 | -0.4813995 |
| RYR1 | J05200 | 0.3090079 | 0.0283 | -2.2317986 | -3.63828 | -0.4815683 |
| TAS2R5 | NM_018980 | 0.3090079 | 0.0283 | -2.2315398 | -3.63879 | -0.4817718 |
| PAPD4 | AF086115 | 0.3353624 | 0.0364 | -2.1312458 | -3.78533 | -0.4828461 |
| TTC12 | AK027161 | 0.3746676 | 0.0478 | -2.0099614 | -4.05283 | -0.4830047 |
| CCDC40 | NM_017950 | 0.3038871 | 0.026 | -2.2683574 | -3.55209 | -0.4837543 |
| ANKRD12 | AF317425 | 0.2809852 | 0.0219 | -2.3354647 | -3.43036 | -0.4849755 |
| DCAF13 | NM_014156 | 0.3479119 | 0.0395 | -2.0916518 | -3.90613 | -0.4851391 |
| TMEM127 | NM_017849 | 0.3473364 | 0.0392 | -2.0951393 | -3.89866 | -0.4852941 |
| RASGRP3 | NM_015376 | 0.1832726 | 0.00681 | -2.775284 | -2.45939 | -0.4853209 |
| PRR5L | AK024275 | 0.2548941 | 0.0164 | -2.4487627 | -3.1938 | -0.4853882 |
| FMNL1 | AJ008114 | 0.3194439 | 0.0316 | -2.1866196 | -3.7263 | -0.4854057 |
| NPAS3 | AF164438 | 0.3310351 | 0.035 | -2.143396 | -3.80903 | -0.4858752 |
| TICAM2 | AB002444 | 0.3360897 | 0.0366 | -2.1249245 | -3.84393 | -0.4859769 |
| RBP2 | NM_004164 | 0.331136 | 0.0351 | -2.1432751 | -3.80835 | -0.4861789 |
| CHIC1 | Y11897 | 0.3618138 | 0.0435 | -2.0504301 | -3.98197 | -0.4865967 |
| DMTN | NM_001978 | 0.2272024 | 0.0121 | -2.5669867 | -2.92705 | -0.4869463 |
| ATXN7 | NM_000333 | 0.2666235 | 0.0189 | -2.3941791 | -3.30898 | -0.4869901 |
| ST8SIA4 | NM_005668 | 0.2701297 | 0.0194 | -2.3849023 | -3.32833 | -0.4877347 |
| DENND5A | AL117448 | 0.3749151 | 0.0481 | -2.0059687 | -4.06225 | -0.4877893 |
| COMMD6 | AF086081 | 0.3086525 | 0.028 | -2.2381459 | -3.62265 | -0.4878057 |
| ZC3HAV1 | NM_020119 | 0.1891073 | 0.00727 | -2.7519598 | -2.51438 | -0.4880761 |
| ACPP | NM_001099 | 0.3280313 | 0.0343 | -2.1525293 | -3.79167 | -0.4882767 |
| FBXL20 | AK024690 | 0.2019304 | 0.00845 | -2.6980126 | -2.64009 | -0.4884039 |
| FOXI1 | NM_012188 | 0.2643568 | 0.0182 | -2.4102371 | -3.27532 | -0.4885829 |
| ANAPC10 | NM_014885 | 0.3262899 | 0.0337 | -2.1597354 | -3.77793 | -0.489182 |
| FOXO3 | NM_001455 | 0.1851786 | 0.00699 | -2.7659925 | -2.48134 | -0.4895564 |
| SLC22A31 | AL137382 | 0.2168013 | 0.0105 | -2.6196321 | -2.81905 | -0.4900541 |
| POLQ | NM_014125 | 0.3246594 | 0.0333 | -2.1648242 | -3.7682 | -0.4907541 |
| NPAS3 | AK002000 | 0.3685252 | 0.0458 | -2.0284005 | -4.02085 | -0.4910852 |
| C7orf43 | NM_018275 | 0.3111067 | 0.0291 | -2.2209245 | -3.65961 | -0.4913679 |
| DCTN1 | U73799 | 0.3166299 | 0.0308 | -2.1965902 | -3.70701 | -0.4915345 |
| TSC22D4 | AJ133115 | 0.3566103 | 0.0416 | -2.0696385 | -3.9468 | -0.4918811 |
| MICAL3 | AB037785 | 0.3225 | 0.0328 | -2.1713716 | -3.75565 | -0.4920916 |
| N4BP2L1 | U50528 | 0.3040447 | 0.0262 | -2.2635245 | -3.57483 | -0.4925088 |
| COQ8A | AL137511 | 0.359785 | 0.0427 | -2.0579471 | -3.96824 | -0.4926397 |
| LOC101928524 | AL137400 | 0.2107941 | 0.00961 | -2.6518154 | -2.74631 | -0.492758 |
| DISC1 | AL049973 | 0.3205045 | 0.0323 | -2.1770457 | -3.74475 | -0.4928082 |
| ATG2A | AB007864 | 0.1851786 | 0.00699 | -2.7664505 | -2.48026 | -0.4938365 |
| PPP1R14D | NM_017726 | 0.313933 | 0.0301 | -2.2073881 | -3.68524 | -0.4940167 |
| CLK1 | NM_004071 | 0.1806156 | 0.00662 | -2.7856364 | -2.43487 | -0.4945071 |
| MIATNB | AK026502 | 0.2539975 | 0.0161 | -2.4575796 | -3.17499 | -0.4949728 |
| NCKAP5L | AB046822 | 0.3774739 | 0.0496 | -1.9931108 | -4.08403 | -0.4955866 |
| SETD1B | AB028999 | 0.3738079 | 0.0473 | -2.0133658 | -4.049 | -0.4956154 |
| KCNRG | AF086244 | 0.3380223 | 0.037 | -2.1201562 | -3.8529 | -0.496226 |
| KLHL24 | AK024270 | 0.2367505 | 0.0135 | -2.5242561 | -3.03087 | -0.4963729 |
| ROM1 | NM_000327 | 0.3082737 | 0.0274 | -2.246337 | -3.59618 | -0.4965239 |
| ABCG1 | NM_004915 | 0.2308232 | 0.0128 | -2.5458931 | -2.98339 | -0.4966098 |
| NFIL3 | NM_005384 | 0.3755225 | 0.0484 | -2.0035501 | -4.06657 | -0.4968844 |
| ZBTB39 | NM_014830 | 0.1421506 | 0.00353 | -3.0036598 | -1.90134 | -0.4976207 |
| SLC8A1 | X91221 | 0.3773425 | 0.0494 | -1.9943785 | -4.08292 | -0.4987056 |
| PEX7 | NM_000288 | 0.3124692 | 0.0296 | -2.2140997 | -3.65987 | -0.498937 |
| DGKI | NM_004717 | 0.3309003 | 0.035 | -2.1439968 | -3.80789 | -0.5002303 |
| HIST1H4C | NM_003542 | 0.2210906 | 0.0112 | -2.5935948 | -2.87753 | -0.5004533 |
| BMP2K | AK021725 | 0.3144522 | 0.0303 | -2.204518 | -3.69162 | -0.5004866 |
| PCDH1 | NM_002587 | 0.2992676 | 0.0246 | -2.2886389 | -3.5253 | -0.5014143 |
| LINC00963 | AK024177 | 0.2136442 | 0.00995 | -2.6383911 | -2.77662 | -0.5018675 |
| HOXA7 | NM_006896 | 0.3193544 | 0.0313 | -2.1900153 | -3.71974 | -0.501947 |
| STAT2 | NM_005419 | 0.3508149 | 0.0402 | -2.0849776 | -3.91749 | -0.5019622 |
| R3HCC1L | AF050198 | 0.3097467 | 0.0285 | -2.2298353 | -3.64214 | -0.5020848 |
| EEPD1 | AF161370 | 0.3040447 | 0.0262 | -2.2636198 | -3.57534 | -0.5031783 |
| ABHD2 | NM_007011 | 0.3085383 | 0.0278 | -2.240231 | -3.62168 | -0.504008 |
| DERL1 | NM_018630 | 0.16832 | 0.00536 | -2.8598701 | -2.25684 | -0.5043186 |
| LOC388692 | AK023642 | 0.2954527 | 0.0241 | -2.2975196 | -3.50679 | -0.5047929 |
| LOC101929340 | AK000493 | 0.3179406 | 0.031 | -2.1946419 | -3.71079 | -0.5053684 |
| LINC00894 | U66048 | 0.1908746 | 0.00741 | -2.7451511 | -2.53036 | -0.5054418 |
| CRYGD | NM_006891 | 0.2643568 | 0.0179 | -2.4165764 | -3.26121 | -0.5058065 |
| RAPGEF1 | AK023760 | 0.3438323 | 0.0384 | -2.1041844 | -3.8828 | -0.5058259 |
| PTEN | AK021619 | 0.3318914 | 0.0354 | -2.1388239 | -3.81769 | -0.5058541 |
| BTBD18 | AK000995 | 0.2318019 | 0.013 | -2.5384863 | -2.99968 | -0.5062747 |
| GPLD1 | NM_001503 | 0.3630552 | 0.0443 | -2.0435838 | -3.99112 | -0.5064552 |
| PTBP3 | NM_005156 | 0.1631182 | 0.00494 | -2.8884404 | -2.18731 | -0.5065304 |
| FEM1C | AK025265 | 0.2195749 | 0.011 | -2.6001535 | -2.86285 | -0.5066076 |
| SLC6A16 | NM_014037 | 0.1394857 | 0.00333 | -3.0248767 | -1.85064 | -0.506677 |
| TMEM62 | AK027028 | 0.2091382 | 0.00918 | -2.6679464 | -2.70925 | -0.5069612 |
| PELI2 | AF302502 | 0.343986 | 0.0385 | -2.1033771 | -3.88431 | -0.5073268 |
| FAM63A | NM_018379 | 0.2169791 | 0.0105 | -2.6173185 | -2.82427 | -0.5075516 |
| LOC101927905 | AL080194 | 0.216553 | 0.0104 | -2.6219805 | -2.81376 | -0.5077912 |
| TMOD2 | NM_014548 | 0.2468015 | 0.015 | -2.483887 | -3.11829 | -0.5079688 |
| TBC1D14 | AB037743 | 0.2754326 | 0.0205 | -2.3627257 | -3.37433 | -0.5079822 |
| CCDC146 | AB040938 | 0.313933 | 0.0301 | -2.2063605 | -3.68804 | -0.5082587 |
| KBTBD11 | NM_014867 | 0.3479119 | 0.0395 | -2.0920951 | -3.90531 | -0.5085211 |
| ESRRA | NM_004451 | 0.177547 | 0.00632 | -2.8022267 | -2.39541 | -0.5095319 |
| ERO1B | AK001865 | 0.3085383 | 0.0276 | -2.2431863 | -3.61585 | -0.5099599 |
| BOD1L1 | AK025965 | 0.2159984 | 0.0103 | -2.6252363 | -2.8064 | -0.5099893 |
| RGS14 | NM_006480 | 0.3285866 | 0.0346 | -2.1478199 | -3.80063 | -0.5102997 |
| MAPK1 | AL157438 | 0.330546 | 0.0349 | -2.1446645 | -3.80662 | -0.5104684 |
| B9D1 | AL137568 | 0.2647025 | 0.0184 | -2.4042921 | -3.2878 | -0.5107648 |
| KIAA1109 | AB037792 | 0.1421506 | 0.00353 | -3.0048606 | -1.89969 | -0.5113231 |
| EVI2A | NM_014210 | 0.3603638 | 0.0429 | -2.0561513 | -3.97152 | -0.511593 |
| DLG5 | NM_004747 | 0.3244949 | 0.0332 | -2.1665851 | -3.76396 | -0.5117443 |
| TCF7L1 | U15553 | 0.3773295 | 0.049 | -1.9982638 | -4.076 | -0.5119101 |
| SMG6 | NM_017575 | 0.2266344 | 0.0118 | -2.575887 | -2.91702 | -0.5123364 |
| TGFBR2 | NM_003242 | 0.2270895 | 0.012 | -2.5691471 | -2.93199 | -0.5128435 |
| ADCYAP1 | NM_001117 | 0.1365237 | 0.0031 | -3.0465741 | -1.79257 | -0.5129273 |
| NWD1 | AK026798 | 0.3014722 | 0.0253 | -2.2788553 | -3.53239 | -0.5144631 |
| PLSCR4 | NM_020353 | 0.3255226 | 0.0335 | -2.1626393 | -3.77059 | -0.5153808 |
| ITGB1BP2 | NM_012278 | 0.2803326 | 0.0215 | -2.3430418 | -3.41485 | -0.5156078 |
| SOAT1 | L21934 | 0.3386892 | 0.0372 | -2.1172343 | -3.85839 | -0.5159401 |
| MAP3K10 | NM_002446 | 0.3413368 | 0.0378 | -2.1112384 | -3.85484 | -0.5167119 |
| C8B | NM_000066 | 0.3073227 | 0.0271 | -2.2497806 | -3.60281 | -0.5172353 |
| PDZD2 | AB002298 | 0.209542 | 0.00941 | -2.6610181 | -2.72582 | -0.5172516 |
| RET | X15262 | 0.1457029 | 0.0038 | -2.9787186 | -1.964 | -0.5172765 |
| NEBL | NM_006393 | 0.209542 | 0.0095 | -2.6553684 | -2.738 | -0.5175437 |
| MYD88 | NM_002468 | 0.3073227 | 0.027 | -2.2507345 | -3.60092 | -0.5183056 |
| USP53 | AB037771 | 0.3575195 | 0.0419 | -2.0670829 | -3.94939 | -0.5187018 |
| SERINC2 | NM_018565 | 0.3773425 | 0.0491 | -1.9974213 | -4.0775 | -0.5190518 |
| PRKD2 | NM_016457 | 0.3110118 | 0.0289 | -2.2229995 | -3.65555 | -0.5192579 |
| BCL3 | NM_005178 | 0.3634278 | 0.0445 | -2.0406812 | -3.99971 | -0.5192763 |
| OR52K3P | AF143328 | 0.2310131 | 0.0129 | -2.5433557 | -2.9889 | -0.5194434 |
| TMEM106A | U25750 | 0.1871708 | 0.00715 | -2.7583545 | -2.49934 | -0.5197955 |
| PPFIA1 | NM_003626 | 0.1642821 | 0.00499 | -2.8857147 | -2.19491 | -0.5211908 |
| GAB1 | NM_002039 | 0.3543398 | 0.041 | -2.0759514 | -3.93517 | -0.5216921 |
| ABI3 | NM_016428 | 0.209542 | 0.00944 | -2.6598718 | -2.72843 | -0.522499 |
| IP6K1 | D87452 | 0.209542 | 0.00932 | -2.6624663 | -2.72179 | -0.5226771 |
| FBRS | AK022551 | 0.2535685 | 0.016 | -2.458559 | -3.1729 | -0.5228538 |
| DGKD | NM_003648 | 0.2437795 | 0.0145 | -2.4968099 | -3.09059 | -0.5228964 |
| GLIPR2 | AJ011129 | 0.330546 | 0.0349 | -2.1446642 | -3.80662 | -0.5231226 |
| HOXD3 | NM_006898 | 0.2647025 | 0.0184 | -2.4056689 | -3.28409 | -0.5242156 |
| BID | NM_001196 | 0.3110093 | 0.0288 | -2.225203 | -3.65123 | -0.5249521 |
| IRX4 | NM_016358 | 0.3193544 | 0.0314 | -2.1893299 | -3.72107 | -0.5252688 |
| BPIFA1 | NM_016583 | 0.3710943 | 0.0465 | -2.0217621 | -4.03281 | -0.5270875 |
| TNFSF8 | NM_001244 | 0.2626411 | 0.0176 | -2.4222153 | -3.2501 | -0.5271176 |
| WNT4 | AY009398 | 0.199617 | 0.00825 | -2.707511 | -2.61847 | -0.527499 |
| KRT23 | AF075083 | 0.3010319 | 0.0251 | -2.2807558 | -3.54112 | -0.5276338 |
| USP15 | AF106069 | 0.1705821 | 0.00557 | -2.8466051 | -2.28893 | -0.5284057 |
| STAT3 | AK024535 | 0.1443034 | 0.00374 | -2.9841768 | -1.95032 | -0.5288225 |
| VWA1 | AL137722 | 0.3205045 | 0.0324 | -2.1764097 | -3.74598 | -0.5288481 |
| ADAM2 | NM_001464 | 0.3088677 | 0.0282 | -2.2349317 | -3.61856 | -0.5300577 |
| BMP2K | AK021620 | 0.2451134 | 0.0149 | -2.4892109 | -3.1066 | -0.5316864 |
| DKFZP761C1711 | AL137340 | 0.2267728 | 0.0119 | -2.5711736 | -2.92749 | -0.5327079 |
| MKLN1 | AF086386 | 0.2380542 | 0.0138 | -2.5170576 | -3.03625 | -0.5334047 |
| DAZAP2 | NM_014764 | 0.2121611 | 0.00979 | -2.6441435 | -2.76356 | -0.5341205 |
| CFC1 | AF312769 | 0.3327453 | 0.0357 | -2.1350299 | -3.82487 | -0.5344865 |
| SUPT20H | NM_017569 | 0.2532532 | 0.016 | -2.4595126 | -3.17086 | -0.5355737 |
| PXN | NM_002859 | 0.3085383 | 0.0277 | -2.2413337 | -3.6195 | -0.5357579 |
| CDC42SE2 | NM_020240 | 0.3614109 | 0.0433 | -2.0521867 | -3.97876 | -0.536344 |
| SBF2 | AK022478 | 0.3111897 | 0.0291 | -2.2205281 | -3.66039 | -0.5383819 |
| ZNF546 | AF087985 | 0.1107084 | 0.00187 | -3.2139319 | -1.35923 | -0.5407271 |
| NAPA | NM_003827 | 0.1753086 | 0.00601 | -2.8200239 | -2.35287 | -0.5407549 |
| CBL | NM_005188 | 0.2599509 | 0.0172 | -2.4320627 | -3.22928 | -0.5409336 |
| P2RY1 | AK026659 | 0.3107928 | 0.0287 | -2.226767 | -3.64816 | -0.5411685 |
| CPQ | NM_006102 | 0.2846386 | 0.0226 | -2.3233765 | -3.45503 | -0.5421771 |
| FGF19 | NM_005117 | 0.2417482 | 0.0142 | -2.5055372 | -3.07166 | -0.5423431 |
| MEF2D | AK027180 | 0.2516217 | 0.0158 | -2.4648529 | -3.15943 | -0.5424822 |
| STAT3 | NM_003150 | 0.33608 | 0.0365 | -2.1261739 | -3.84064 | -0.5428073 |
| ABCG2 | AF151530 | 0.3182598 | 0.0311 | -2.1938821 | -3.69901 | -0.5438864 |
| DLL4 | NM_019074 | 0.16832 | 0.00542 | -2.8563002 | -2.26549 | -0.5451201 |
| CEACAM3 | NM_001815 | 0.3085383 | 0.0278 | -2.2398013 | -3.62253 | -0.5459692 |
| CHSY1 | NM_014918 | 0.1897029 | 0.00733 | -2.7493407 | -2.52053 | -0.5465574 |
| MIER1 | AB046830 | 0.1773235 | 0.00628 | -2.8041167 | -2.3909 | -0.5473633 |
| ARFIP1 | NM_014447 | 0.2076372 | 0.00908 | -2.6719177 | -2.70015 | -0.5474745 |
| LINC00588 | AL080200 | 0.2743887 | 0.0202 | -2.3690442 | -3.34876 | -0.5478945 |
| PDE1C | NM_005020 | 0.2597229 | 0.017 | -2.434782 | -3.22351 | -0.547998 |
| CPN2 | J05158 | 0.2751458 | 0.0204 | -2.3645684 | -3.37052 | -0.5480968 |
| CD226 | NM_006566 | 0.3109985 | 0.0287 | -2.2275147 | -3.64433 | -0.5486584 |
| LMCD1 | NM_014583 | 0.3194746 | 0.0319 | -2.18344 | -3.7316 | -0.5490107 |
| TP53INP2 | AL137597 | 0.1172416 | 0.00216 | -3.1669501 | -1.48109 | -0.549137 |
| EPPK1 | AK000090 | 0.3194439 | 0.0317 | -2.18569 | -3.7281 | -0.5500639 |
| ADPRH | NM_001125 | 0.2643568 | 0.018 | -2.4158089 | -3.26283 | -0.5501317 |
| SPIB | AK025419 | 0.1844686 | 0.0069 | -2.7706068 | -2.47045 | -0.5502618 |
| LOC401052 | AK022260 | 0.303108 | 0.0258 | -2.2711147 | -3.55972 | -0.5504266 |
| HIVEP1 | NM_002114 | 0.3043244 | 0.0266 | -2.2582088 | -3.58609 | -0.55054 |
| UBE2D3 | AF116686 | 0.3669312 | 0.0453 | -2.0327678 | -4.01405 | -0.5510732 |
| LOC100508226///MAGI1-IT1///MAGI1 | AL050129 | 0.2647025 | 0.0184 | -2.4059754 | -3.27228 | -0.5512886 |
| CLIP4 | AK024722 | 0.3205045 | 0.0323 | -2.1769685 | -3.7449 | -0.5514023 |
| ICAM3 | NM_002162 | 0.3754834 | 0.0483 | -2.0039263 | -4.0659 | -0.5514336 |
| PTPA | X73478 | 0.3165437 | 0.0308 | -2.1983648 | -3.68891 | -0.5514753 |
| EN2 | NM_001427 | 0.2169791 | 0.0105 | -2.6179661 | -2.82293 | -0.5526776 |
| CD46 | M58050 | 0.1705821 | 0.00558 | -2.8461564 | -2.29001 | -0.5543363 |
| OR10J1 | NM_012351 | 0.2308232 | 0.0128 | -2.5458418 | -2.98344 | -0.5544842 |
| N4BP2L2 | NM_014887 | 0.2314605 | 0.0129 | -2.5412133 | -2.99369 | -0.5553418 |
| B2M | NM_004048 | 0.3510252 | 0.0403 | -2.0837031 | -3.92086 | -0.5562462 |
| MVP | NM_017458 | 0.2308232 | 0.0128 | -2.5439343 | -2.98771 | -0.5566497 |
| CSF1R | NM_005211 | 0.2446332 | 0.0146 | -2.4942313 | -3.09617 | -0.5573997 |
| C20orf181 | U63828 | 0.215197 | 0.0101 | -2.6315064 | -2.79222 | -0.5575268 |
| TNXB | Y17867 | 0.1956321 | 0.00775 | -2.7302236 | -2.56571 | -0.5578879 |
| TLCD2 | AK000038 | 0.3090079 | 0.0283 | -2.231565 | -3.63874 | -0.5582548 |
| ZNFX1 | AK022226 | 0.3088677 | 0.0281 | -2.2350468 | -3.63189 | -0.5582551 |
| TET2 | AB046766 | 0.3681749 | 0.0457 | -2.028753 | -4.02131 | -0.5584781 |
| LOC105375531 | AK000794 | 0.1728702 | 0.00583 | -2.8305873 | -2.32752 | -0.5595162 |
| PGAP1 | AL050078 | 0.1529701 | 0.00437 | -2.9306035 | -2.0837 | -0.5599744 |
| CAMK2G | AK026309 | 0.1956321 | 0.00779 | -2.7274588 | -2.57172 | -0.5604735 |
| SCN10A | NM_006514 | 0.1947414 | 0.00769 | -2.7328856 | -2.55951 | -0.560799 |
| EGR2 | NM_000399 | 0.3321963 | 0.0355 | -2.1379569 | -3.81746 | -0.5620446 |
| KIAA0232 | D86985 | 0.3401834 | 0.0376 | -2.1136184 | -3.86516 | -0.5625287 |
| PBXIP1 | NM_020524 | 0.1671388 | 0.00527 | -2.8661084 | -2.24171 | -0.5628549 |
| SHANK2 | AF131790 | 0.3205045 | 0.0323 | -2.1769813 | -3.74488 | -0.5629374 |
| ARF1 | AK023803 | 0.1704895 | 0.00554 | -2.8480721 | -2.28539 | -0.5629397 |
| RIPK1 | NM_003804 | 0.1794227 | 0.0065 | -2.7928974 | -2.41826 | -0.5631787 |
| CYHR1 | AB007965 | 0.1394857 | 0.00332 | -3.0236833 | -1.85074 | -0.5637403 |
| IGSF9B | AB028953 | 0.2643568 | 0.0182 | -2.4116053 | -3.26053 | -0.5640868 |
| ZCCHC2 | AK000229 | 0.2860137 | 0.0228 | -2.3196817 | -3.46196 | -0.5655943 |
| PDE4B | NM_002600 | 0.2872899 | 0.0231 | -2.3154765 | -3.47109 | -0.5659015 |
| ERV3-2 | AB040899 | 0.3082737 | 0.0274 | -2.2454967 | -3.61128 | -0.5667599 |
| B2M | AK022379 | 0.3586346 | 0.0423 | -2.0626789 | -3.95957 | -0.5676392 |
| HMGCS2 | NM_005518 | 0.2369994 | 0.0136 | -2.5213624 | -3.03719 | -0.5676724 |
| ELOVL5 | AF111849 | 0.2069707 | 0.00897 | -2.6760875 | -2.69059 | -0.568236 |
| CREB1 | M34356 | 0.1244795 | 0.00248 | -3.1207839 | -1.60165 | -0.5690043 |
| MSRB1 | NM_016332 | 0.3041762 | 0.0263 | -2.2621592 | -3.57824 | -0.569072 |
| PSMA1 | NM_002786 | 0.1862536 | 0.00709 | -2.7614034 | -2.49216 | -0.5697262 |
| EIF4A1 | U79273 | 0.2753954 | 0.0204 | -2.3636367 | -3.37244 | -0.5702185 |
| M1AP | Y12839 | 0.2705987 | 0.0195 | -2.3836199 | -3.33055 | -0.5718333 |
| LRP10 | AF131760 | 0.3045913 | 0.0266 | -2.2574347 | -3.58763 | -0.5721487 |
| HIST1H4H | NM_003543 | 0.2159984 | 0.0103 | -2.6268244 | -2.80282 | -0.572652 |
| CNNM1 | NM_020348 | 0.1037891 | 0.00146 | -3.292558 | -1.14624 | -0.5727126 |
| C15orf39 | NM_015492 | 0.2588746 | 0.0169 | -2.4389152 | -3.21475 | -0.5730592 |
| RASL12 | NM_016563 | 0.1753086 | 0.00611 | -2.8150194 | -2.36556 | -0.5743795 |
| PSMD6-AS2 | AK023371 | 0.3082737 | 0.0274 | -2.2451756 | -3.61192 | -0.5744935 |
| APAF1 | NM_013229 | 0.1753086 | 0.00605 | -2.8172111 | -2.35961 | -0.5752322 |
| ZER1 | NM_006336 | 0.3125149 | 0.0297 | -2.2122208 | -3.67662 | -0.5760773 |
| ANKRD33B | AK023999 | 0.151322 | 0.00418 | -2.9467131 | -2.04495 | -0.5766348 |
| VPS9D1 | NM_004913 | 0.2803326 | 0.0216 | -2.3415823 | -3.41784 | -0.5768138 |
| C1QTNF5///MFRP | AL110261 | 0.3051984 | 0.0267 | -2.2557595 | -3.59096 | -0.5772291 |
| MTHFS | AL109717 | 0.2546144 | 0.0164 | -2.449509 | -3.19221 | -0.5774784 |
| ZNF200 | NM_003454 | 0.2308232 | 0.0128 | -2.5451651 | -2.985 | -0.5778538 |
| ST3GAL1 | NM_003033 | 0.1278422 | 0.00262 | -3.103406 | -1.64668 | -0.578148 |
| RPS6KA2 | X85106 | 0.2380542 | 0.0138 | -2.5162938 | -3.04825 | -0.5788603 |
| CRTAC1 | NM_018058 | 0.2809852 | 0.022 | -2.3357492 | -3.42923 | -0.5790745 |
| SGK1 | NM_005627 | 0.2195749 | 0.011 | -2.600622 | -2.8618 | -0.5798325 |
| NOD2 | AF178930 | 0.3086243 | 0.028 | -2.2364542 | -3.62912 | -0.5799563 |
| PTOV1-AS2 | AL050131 | 0.3479119 | 0.0395 | -2.0915758 | -3.90627 | -0.5801561 |
| KIAA0825 | AB020632 | 0.1394857 | 0.00333 | -3.0254308 | -1.84924 | -0.5807165 |
| WDCP | AK025598 | 0.3774739 | 0.0497 | -1.9920598 | -4.0859 | -0.5807507 |
| TRANK1 | AB002340 | 0.2542212 | 0.0162 | -2.4556083 | -3.1792 | -0.5812831 |
| ABHD3 | AF007152 | 0.1478914 | 0.00399 | -2.9617028 | -2.00651 | -0.5818459 |
| KRT75 | NM_004693 | 0.2169791 | 0.0105 | -2.6179691 | -2.8228 | -0.5818761 |
| NEK7 | AL080111 | 0.2435112 | 0.0145 | -2.4983159 | -3.08733 | -0.5834042 |
| ATG4B | AL050288 | 0.2484966 | 0.0154 | -2.475219 | -3.13718 | -0.5837442 |
| LOC101927830///TMLHE-AS1 | L23867 | 0.2159377 | 0.0102 | -2.6298289 | -2.79617 | -0.5838995 |
| ZCCHC2 | NM_017742 | 0.2754326 | 0.0205 | -2.3631103 | -3.37353 | -0.5843751 |
| KRT37 | NM_003770 | 0.1474008 | 0.00389 | -2.9723595 | -1.98243 | -0.5846781 |
| HES2 | NM_019089 | 0.2318019 | 0.013 | -2.5385985 | -2.99944 | -0.5851445 |
| CRTAM | NM_019604 | 0.2604757 | 0.0172 | -2.4311052 | -3.22014 | -0.5856164 |
| HIST1H1T | NM_005323 | 0.209542 | 0.00931 | -2.6627295 | -2.72119 | -0.5858558 |
| ARNTL | NM_001178 | 0.2380542 | 0.0137 | -2.5178505 | -3.04485 | -0.5866072 |
| OCLM | AF142063 | 0.2267728 | 0.0119 | -2.5751045 | -2.91876 | -0.5868647 |
| PPP1R12B | AB007972 | 0.1191227 | 0.00227 | -3.1520122 | -1.52221 | -0.5876136 |
| PLXDC2 | AL080095 | 0.359785 | 0.0427 | -2.058139 | -3.96789 | -0.5882634 |
| BCL2A1 | NM_004049 | 0.1885666 | 0.00724 | -2.7543345 | -2.50931 | -0.5913904 |
| TMEM55A | AL359591 | 0.1244795 | 0.00247 | -3.1227347 | -1.59658 | -0.591605 |
| KIAA0319 | NM_014809 | 0.3088677 | 0.0282 | -2.2334798 | -3.63498 | -0.5936765 |
| KANSL1L | AL133053 | 0.2957918 | 0.0242 | -2.2973274 | -3.49491 | -0.5939121 |
| IFIT5 | NM_012420 | 0.3085383 | 0.0279 | -2.2385867 | -3.62492 | -0.593946 |
| KLF6 | AL117595 | 0.2543051 | 0.0162 | -2.4548625 | -3.18079 | -0.5947196 |
| VAMP2 | NM_014232 | 0.1365237 | 0.00309 | -3.047572 | -1.79003 | -0.5949324 |
| TMCC1 | AB018322 | 0.0737062 | 0.000571 | -3.5832767 | -0.33451 | -0.5949472 |
| RHBDD3 | AL050346 | 0.315681 | 0.0305 | -2.2009522 | -3.69855 | -0.5951359 |
| CDH9 | NM_016279 | 0.3040447 | 0.0263 | -2.2629766 | -3.57662 | -0.5956757 |
| PSD4 | NM_012455 | 0.246904 | 0.0151 | -2.4822195 | -3.12211 | -0.5965359 |
| UBE2D3 | NM_003340 | 0.119209 | 0.00228 | -3.1492049 | -1.52759 | -0.5989833 |
| R3HCC1 | AL050297 | 0.0859672 | 0.000815 | -3.4750034 | -0.64266 | -0.5993434 |
| PIK3CA | AK021510 | 0.1671388 | 0.00526 | -2.8661911 | -2.2415 | -0.5995414 |
| NKX3-1 | NM_006167 | 0.1361806 | 0.00306 | -3.0508493 | -1.78167 | -0.5995485 |
| TENM2 | AB032953 | 0.16832 | 0.00542 | -2.8561806 | -2.26578 | -0.5999872 |
| RNF217 | AK023584 | 0.096519 | 0.00117 | -3.3616544 | -0.95788 | -0.6009156 |
| SSSCA1-AS1 | AF085877 | 0.3082737 | 0.0274 | -2.2461278 | -3.61003 | -0.6010263 |
| SLC22A15 | AL353933 | 0.2545873 | 0.0163 | -2.4518762 | -3.18687 | -0.6011057 |
| MFN2 | NM_014874 | 0.1107084 | 0.00187 | -3.2136733 | -1.35769 | -0.6011817 |
| SETX | NM_015046 | 0.2578232 | 0.0168 | -2.4411323 | -3.21004 | -0.6019822 |
| CYP4F3 | NM_000896 | 0.2599033 | 0.0171 | -2.4341725 | -3.22481 | -0.6020491 |
| AVPR1B | NM_000707 | 0.16832 | 0.00542 | -2.8560223 | -2.26616 | -0.602136 |
| STMND1 | AK026805 | 0.3746676 | 0.0478 | -2.010483 | -4.02215 | -0.6022202 |
| LGALS8-AS1 | AK026321 | 0.3085383 | 0.0277 | -2.2421362 | -3.60444 | -0.6023262 |
| DHX34 | NM_014681 | 0.3109985 | 0.0287 | -2.226881 | -3.64638 | -0.6031067 |
| FOXP2 | AF086040 | 0.3508168 | 0.0402 | -2.0851835 | -3.9031 | -0.6037142 |
| SLC7A4 | NM_004173 | 0.1408906 | 0.00345 | -3.0122133 | -1.88116 | -0.6037437 |
| SSTR5 | NM_001053 | 0.1723798 | 0.00573 | -2.838569 | -2.30989 | -0.6054876 |
| PARP16 | NM_017851 | 0.2647025 | 0.0185 | -2.4025732 | -3.291 | -0.6057354 |
| C1GALT1 | AK023557 | 0.1548069 | 0.00444 | -2.9252736 | -2.09686 | -0.6063403 |
| ADAR | NM_001111 | 0.0908794 | 0.000976 | -3.4194793 | -0.79803 | -0.6072296 |
| TMEM216 | NM_016499 | 0.246904 | 0.0151 | -2.481737 | -3.12315 | -0.6073804 |
| NCK2 | NM_003581 | 0.1139781 | 0.00197 | -3.195973 | -1.4046 | -0.6086227 |
| TXNDC11 | NM_015914 | 0.3043244 | 0.0264 | -2.2605871 | -3.58137 | -0.608774 |
| RPGR | NM_000328 | 0.1094396 | 0.00182 | -3.2246072 | -1.33322 | -0.6089333 |
| LOC105373460 | AL117577 | 0.3630552 | 0.0443 | -2.0424432 | -3.99651 | -0.609815 |
| PGGHG | AK026288 | 0.2709425 | 0.0195 | -2.3814657 | -3.33548 | -0.6110745 |
| NECAB2 | AF070637 | 0.2069707 | 0.00894 | -2.6775205 | -2.6873 | -0.6110765 |
| TRPC4 | NM_016179 | 0.2905662 | 0.0235 | -2.3091868 | -3.48324 | -0.6114835 |
| DDX60 | NM_017631 | 0.3321963 | 0.0355 | -2.1370098 | -3.82113 | -0.6118496 |
| SIGLEC1 | AK024462 | 0.3515263 | 0.0404 | -2.0820939 | -3.92383 | -0.6135454 |
| ADGRL4 | AF192403 | 0.2662736 | 0.0189 | -2.3955765 | -3.30563 | -0.6135552 |
| CLCA2 | NM_006536 | 0.228628 | 0.0125 | -2.5558331 | -2.96137 | -0.6143568 |
| KRT1 | NM_006121 | 0.2272024 | 0.0121 | -2.5652752 | -2.93073 | -0.6150404 |
| PCNX2 | NM_014801 | 0.2091866 | 0.0092 | -2.6677509 | -2.70996 | -0.6158793 |
| ZNF71 | AF269249 | 0.3626634 | 0.0438 | -2.0486004 | -3.98201 | -0.6169683 |
| ITGA5 | NM_002205 | 0.0918653 | 0.00108 | -3.3869626 | -0.88817 | -0.6173617 |
| RBMY3AP | U94386 | 0.1443034 | 0.00374 | -2.9837394 | -1.95142 | -0.6188761 |
| ATRX | L33813 | 0.3712044 | 0.0466 | -2.019754 | -4.03753 | -0.6198123 |
| ZNF551 | X52354 | 0.1844686 | 0.00691 | -2.7730722 | -2.4584 | -0.6205074 |
| SPAG6 | NM_012443 | 0.3145073 | 0.0303 | -2.2039833 | -3.69266 | -0.6209786 |
| MSC | NM_005098 | 0.1016932 | 0.00138 | -3.3129058 | -1.09651 | -0.6214386 |
| EIF4E2 | AK027239 | 0.1476893 | 0.00392 | -2.9692012 | -1.98248 | -0.6221335 |
| LOC102723769 | AK001448 | 0.3022342 | 0.0254 | -2.2770565 | -3.53541 | -0.6224447 |
| P2RY10 | NM_014499 | 0.2229435 | 0.0115 | -2.5852982 | -2.89606 | -0.622795 |
| 7-Mar | AK023400 | 0.0966792 | 0.00122 | -3.3490642 | -0.99242 | -0.6239219 |
| CEP68 | AF090099 | 0.178177 | 0.00642 | -2.7962076 | -2.40975 | -0.6242404 |
| SRRM2 | X97301 | 0.2170877 | 0.0106 | -2.6132763 | -2.83337 | -0.6252421 |
| TXNIP | NM_006472 | 0.2396323 | 0.014 | -2.5097545 | -3.06249 | -0.6258287 |
| GPR27 | NM_018971 | 0.3088677 | 0.0282 | -2.2334131 | -3.63511 | -0.6277665 |
| KRT20 | X73502 | 0.1042896 | 0.0015 | -3.2845924 | -1.16777 | -0.6291626 |
| UBR2 | AB002347 | 0.2672473 | 0.019 | -2.3925206 | -3.31244 | -0.6312106 |
| DTWD1 | AL122040 | 0.2093914 | 0.00926 | -2.6648355 | -2.71637 | -0.6330184 |
| GAREM2 | AB015349 | 0.1428571 | 0.00362 | -2.9946904 | -1.92392 | -0.6330389 |
| ELL | AF157562 | 0.1287193 | 0.00265 | -3.0994152 | -1.65699 | -0.6349416 |
| IRF7 | NM_004031 | 0.3420498 | 0.038 | -2.1090847 | -3.87365 | -0.6350013 |
| SIRPG | NM_018556 | 0.1609168 | 0.00477 | -2.9005771 | -2.15761 | -0.6357978 |
| HECW2 | AB037722 | 0.2069707 | 0.00898 | -2.6757379 | -2.69139 | -0.6369677 |
| ZBTB18 | NM_006352 | 0.2239887 | 0.0115 | -2.58388 | -2.89925 | -0.6370502 |
| TAP2 | NM_000544 | 0.2396323 | 0.014 | -2.510743 | -3.06018 | -0.6379433 |
| TPCN2 | AL137479 | 0.1428571 | 0.00364 | -2.9933273 | -1.92735 | -0.6396184 |
| RAPGEF1 | NM_005312 | 0.1397749 | 0.00335 | -3.023348 | -1.85451 | -0.640184 |
| ABHD5 | AF007132 | 0.2028552 | 0.00862 | -2.6908177 | -2.6567 | -0.6414151 |
| FPR2 | NM_001462 | 0.343986 | 0.0384 | -2.1036057 | -3.88388 | -0.6416185 |
| SNRK | NM_017719 | 0.1334554 | 0.00288 | -3.0720397 | -1.72746 | -0.6434006 |
| USP34 | AK024341 | 0.1187233 | 0.00225 | -3.1531451 | -1.51728 | -0.6434091 |
| MED12L | AF087980 | 0.2545873 | 0.0163 | -2.4529493 | -3.18427 | -0.6445769 |
| ACTR2 | AK025051 | 0.3098293 | 0.0285 | -2.2292453 | -3.6433 | -0.645256 |
| SARS | AK022339 | 0.1071625 | 0.00169 | -3.2466033 | -1.27227 | -0.645754 |
| KMT2C | AF264750 | 0.0730455 | 0.000527 | -3.6079643 | -0.2633 | -0.645913 |
| POC1A | AL117629 | 0.257508 | 0.0167 | -2.4425659 | -3.20699 | -0.6460241 |
| PAEP | NM_002571 | 0.2272024 | 0.0121 | -2.5666146 | -2.93761 | -0.646136 |
| AGL | NM_000028 | 0.0908794 | 0.000971 | -3.4226008 | -0.79257 | -0.6477966 |
| GPR132 | NM_013345 | 0.1759761 | 0.00618 | -2.8101896 | -2.37641 | -0.6485533 |
| NT5C2 | NM_012229 | 0.16832 | 0.00535 | -2.8606909 | -2.25485 | -0.649477 |
| COL9A2 | M95610 | 0.1796562 | 0.00656 | -2.7886583 | -2.4277 | -0.649645 |
| MBNL1 | AK021883 | 0.1606636 | 0.00474 | -2.9025354 | -2.15281 | -0.6498326 |
| CCND3 | AK022376 | 0.209542 | 0.00943 | -2.6596813 | -2.72863 | -0.6499408 |
| BAZ2B | NM_013450 | 0.2287376 | 0.0125 | -2.55218 | -2.96954 | -0.6503914 |
| ANKRD44 | AL133087 | 0.1824064 | 0.00673 | -2.7798701 | -2.44854 | -0.6505653 |
| SH2B2 | AB000520 | 0.1753086 | 0.00609 | -2.8150006 | -2.3649 | -0.650723 |
| MAP3K14 | AJ008158 | 0.1824064 | 0.00672 | -2.7801111 | -2.44797 | -0.6536207 |
| TINAG | NM_014464 | 0.1393572 | 0.00327 | -3.0291299 | -1.83693 | -0.6538423 |
| PARP11 | NM_020367 | 0.1643915 | 0.005 | -2.8848714 | -2.19697 | -0.6541829 |
| CD4 | M12807 | 0.2193009 | 0.011 | -2.6020454 | -2.85861 | -0.654635 |
| CCDC126 | AK026684 | 0.2354058 | 0.0134 | -2.5272384 | -3.02434 | -0.6549098 |
| NFKBIB | NM_002503 | 0.3029261 | 0.0255 | -2.2742748 | -3.55409 | -0.6549557 |
| FGD3 | AK000004 | 0.2599033 | 0.0171 | -2.4332456 | -3.22677 | -0.6557513 |
| FAM8A1 | NM_016255 | 0.2701297 | 0.0194 | -2.3850386 | -3.32804 | -0.656701 |
| GNLY | NM_012483 | 0.238651 | 0.0138 | -2.5153056 | -3.05026 | -0.6572301 |
| YPEL5 | NM_016061 | 0.1987867 | 0.00811 | -2.7130763 | -2.60519 | -0.6575807 |
| BCAS1 | NM_003657 | 0.2033553 | 0.00866 | -2.6898055 | -2.65935 | -0.6578895 |
| U2AF1 | AK022152 | 0.2656183 | 0.0187 | -2.3981265 | -3.30072 | -0.6588968 |
| CHST15 | NM_014863 | 0.1832726 | 0.00681 | -2.7753944 | -2.45913 | -0.6598669 |
| GPSM1 | AL117478 | 0.3085383 | 0.0277 | -2.2412511 | -3.61967 | -0.6604176 |
| OVGP1 | NM_002557 | 0.1961094 | 0.00785 | -2.7247593 | -2.57802 | -0.6610091 |
| WDR47 | NM_014969 | 0.1178713 | 0.0022 | -3.1645136 | -1.49367 | -0.6611402 |
| RUNX2 | L40992 | 0.1394857 | 0.00331 | -3.0248935 | -1.84768 | -0.6614161 |
| SIRPB1 | NM_006065 | 0.2354058 | 0.0134 | -2.5269109 | -3.02506 | -0.6619712 |
| ARNT | AL137290 | 0.0884947 | 0.00089 | -3.4478922 | -0.71875 | -0.6624257 |
| RIMS2 | NM_014677 | 0.3073227 | 0.0271 | -2.2501747 | -3.60131 | -0.6625536 |
| LRWD1 | AL133057 | 0.082735 | 0.000736 | -3.5064497 | -0.55386 | -0.6630679 |
| EPB42 | NM_000119 | 0.1144986 | 0.00201 | -3.1907763 | -1.42046 | -0.6637201 |
| SYNE2 | AL080133 | 0.1244795 | 0.00249 | -3.1200794 | -1.60348 | -0.6642275 |
| CDK5R1 | NM_003885 | 0.2070132 | 0.00902 | -2.6749596 | -2.69346 | -0.664518 |
| ATG16L2 | AK024423 | 0.1753086 | 0.00611 | -2.8136925 | -2.36803 | -0.6651935 |
| GCC1 | AK025688 | 0.3137915 | 0.0299 | -2.2100409 | -3.68007 | -0.6674055 |
| THBS4 | NM_003248 | 0.1428571 | 0.0036 | -2.9967811 | -1.91866 | -0.6680606 |
| TNFRSF9 | NM_001561 | 0.1847348 | 0.00694 | -2.7688777 | -2.47453 | -0.6691123 |
| ZNF641 | AF086127 | 0.3070166 | 0.0269 | -2.25258 | -3.59726 | -0.6691206 |
| LAMA3 | AK024889 | 0.3095921 | 0.0284 | -2.2310062 | -3.63908 | -0.6694162 |
| MKNK2 | Z25424 | 0.0752196 | 0.000613 | -3.5622716 | -0.39482 | -0.6700996 |
| NET1 | NM_005863 | 0.3318914 | 0.0354 | -2.1391076 | -3.81624 | -0.6715725 |
| GIMAP4 | NM_018326 | 0.1606636 | 0.00473 | -2.903141 | -2.15132 | -0.6719695 |
| RUNDC3A | NM_006695 | 0.3193544 | 0.0313 | -2.1902913 | -3.70593 | -0.6720738 |
| PPP4R1 | NM_005134 | 0.1365237 | 0.0031 | -3.0465898 | -1.79253 | -0.6724361 |
| C8A | NM_000562 | 0.2107941 | 0.00962 | -2.6508178 | -2.74837 | -0.6732303 |
| DUSP1 | AJ227912 | 0.3571044 | 0.0418 | -2.067696 | -3.95037 | -0.6746551 |
| EGLN1 | AJ227859 | 0.1428571 | 0.00363 | -2.9946727 | -1.9253 | -0.6747362 |
| LOC101928574 | AL137602 | 0.1287193 | 0.00265 | -3.0998966 | -1.65749 | -0.6757297 |
| PSEN1 | NM_007318 | 0.2021841 | 0.0085 | -2.6957034 | -2.64542 | -0.6779966 |
| TRIP12 | D28476 | 0.1172048 | 0.00214 | -3.1698694 | -1.47342 | -0.6781006 |
| PTPRA | NM_002836 | 0.1393572 | 0.00328 | -3.0275894 | -1.84084 | -0.6795537 |
| FAM149A | AL080065 | 0.2463438 | 0.015 | -2.4846292 | -3.11692 | -0.6795556 |
| LOC105378763 | AF279783 | 0.1749624 | 0.00596 | -2.8244469 | -2.34375 | -0.6808103 |
| IL6R | NM_000565 | 0.2720497 | 0.0199 | -2.3742109 | -3.35055 | -0.6812632 |
| GPR12 | NM_005288 | 0.2107941 | 0.00962 | -2.6513582 | -2.73818 | -0.6849566 |
| EI24 | NM_004879 | 0.209542 | 0.00947 | -2.6566277 | -2.73513 | -0.6863595 |
| SCLY | NM_016510 | 0.0587622 | 0.00028 | -3.7946528 | 0.28614 | -0.6876518 |
| WASF2 | AB026542 | 0.1264871 | 0.00255 | -3.112159 | -1.62402 | -0.6880297 |
| CACNB4 | NM_000726 | 0.1791174 | 0.00648 | -2.7941754 | -2.41522 | -0.6885629 |
| FMNL1 | AJ008123 | 0.1671388 | 0.00525 | -2.867224 | -2.239 | -0.6887944 |
| ZNF217 | NM_006526 | 0.3674314 | 0.0454 | -2.0342828 | -3.96216 | -0.68927 |
| GRAMD1C | NM_017577 | 0.066993 | 0.000362 | -3.7217002 | 0.06422 | -0.6905135 |
| EGFL6 | NM_015507 | 0.3207744 | 0.0324 | -2.1768615 | -3.73019 | -0.6925986 |
| SERINC3 | NM_006811 | 0.0803722 | 0.000684 | -3.5286495 | -0.49083 | -0.6935678 |
| MARCKS | NM_002356 | 0.0884947 | 0.000888 | -3.4485253 | -0.71698 | -0.6941604 |
| IDS | NM_000202 | 0.0915477 | 0.00104 | -3.4003314 | -0.8543 | -0.6969168 |
| FOXP1 | AK026898 | 0.0734366 | 0.00054 | -3.6002723 | -0.28552 | -0.6989689 |
| PGPEP1 | NM_017712 | 0.0437326 | 0.000166 | -3.9448919 | 0.74191 | -0.700453 |
| LAMA2 | NM_000426 | 0.2993592 | 0.0247 | -2.2879884 | -3.52661 | -0.7012262 |
| SETX | AK024331 | 0.0879607 | 0.000867 | -3.4559139 | -0.69628 | -0.7015292 |
| CD300A | AF020314 | 0.2169791 | 0.0106 | -2.616421 | -2.82629 | -0.7015906 |
| NEU2 | NM_005383 | 0.1042896 | 0.00149 | -3.2855498 | -1.16518 | -0.7023039 |
| GPN2 | NM_018066 | 0.3124692 | 0.0295 | -2.2169628 | -3.66493 | -0.7023566 |
| TNFAIP6 | NM_007115 | 0.332511 | 0.0356 | -2.1363964 | -3.82229 | -0.7042865 |
| RPS6KA5 | NM_004755 | 0.0918593 | 0.00108 | -3.3884075 | -0.88417 | -0.7044736 |
| FRY | U50534 | 0.0730455 | 0.000507 | -3.6195247 | -0.22984 | -0.7049955 |
| ELMO1 | NM_014800 | 0.0646961 | 0.000321 | -3.7551847 | 0.16839 | -0.7076452 |
| NFATC2 | AK025758 | 0.1349172 | 0.00299 | -3.05958 | -1.75937 | -0.7087001 |
| EGR3 | NM_004430 | 0.1071625 | 0.00167 | -3.2508261 | -1.26098 | -0.7107455 |
| IL1RL2 | NM_003854 | 0.1987867 | 0.00814 | -2.7116636 | -2.60847 | -0.7110811 |
| VASP | NM_003370 | 0.2643568 | 0.0182 | -2.4092472 | -3.2774 | -0.7114681 |
| ZNF451 | AK023072 | 0.2014285 | 0.00839 | -2.7024122 | -2.63063 | -0.7128118 |
| IFI16 | NM_005531 | 0.1987867 | 0.00812 | -2.7124943 | -2.60654 | -0.7139211 |
| ICAM2 | NM_000873 | 0.198031 | 0.00802 | -2.7168428 | -2.59644 | -0.7143775 |
| KCNC4 | NM_004978 | 0.1389367 | 0.00324 | -3.0335844 | -1.82111 | -0.7147844 |
| HAL | NM_002108 | 0.2756699 | 0.0206 | -2.3606742 | -3.37856 | -0.7162282 |
| SLA | NM_006748 | 0.3006479 | 0.0248 | -2.2862391 | -3.52947 | -0.7210411 |
| TBC1D22A | AK000851 | 0.0889842 | 0.000915 | -3.4408076 | -0.74189 | -0.7211029 |
| TAGAP | AK025272 | 0.1699968 | 0.00552 | -2.8496615 | -2.28155 | -0.7241263 |
| OAF | AK021720 | 0.3634278 | 0.0444 | -2.0415396 | -3.98369 | -0.7271052 |
| AVPR2 | NM_000054 | 0.1094396 | 0.00181 | -3.2231824 | -1.3324 | -0.7315582 |
| SUPT20H | AK021457 | 0.0737062 | 0.000571 | -3.5855214 | -0.33216 | -0.736855 |
| GNG7 | NM_005145 | 0.066993 | 0.000372 | -3.7112787 | 0.03839 | -0.7371807 |
| LOC401320 | AL137445 | 0.2545873 | 0.0163 | -2.4518955 | -3.18683 | -0.7371919 |
| SSH2 | AF086010 | 0.1071625 | 0.00164 | -3.2554624 | -1.24616 | -0.7372166 |
| DCHS1 | AK021852 | 0.1172416 | 0.00216 | -3.1675269 | -1.4816 | -0.7373455 |
| BST1 | NM_004334 | 0.340974 | 0.0377 | -2.1115657 | -3.86901 | -0.7394823 |
| KCNJ15 | NM_002243 | 0.3159474 | 0.0306 | -2.2010916 | -3.68438 | -0.74333 |
| TGFBR1 | NM_004612 | 0.2727714 | 0.0201 | -2.3711589 | -3.35688 | -0.7439867 |
| SGCD | AF010236 | 0.0918593 | 0.00107 | -3.3991727 | -0.86852 | -0.7449139 |
| SLC25A37 | S94541 | 0.2121611 | 0.00982 | -2.6430776 | -2.76598 | -0.7454577 |
| LRMP | NM_006152 | 0.1478914 | 0.00399 | -2.9624556 | -2.00584 | -0.7458962 |
| THBD | NM_000361 | 0.2282012 | 0.0123 | -2.5597359 | -2.95284 | -0.7491226 |
| KLK5 | NM_012427 | 0.259265 | 0.0169 | -2.4382964 | -3.20475 | -0.749329 |
| SEC14L1 | NM_003003 | 0.1525002 | 0.00427 | -2.9382163 | -2.06486 | -0.7504628 |
| C5orf15 | NM_020199 | 0.0966792 | 0.00127 | -3.3367071 | -1.02623 | -0.7512463 |
| FLOT2 | NM_004475 | 0.2195749 | 0.011 | -2.5995798 | -2.86413 | -0.7530818 |
| CAMK1D | AL137430 | 0.1071625 | 0.00163 | -3.2566762 | -1.2429 | -0.7532522 |
| LOC101930164 | AK026751 | 0.1615383 | 0.0048 | -2.8989733 | -2.16253 | -0.754328 |
| ARHGAP25 | NM_014882 | 0.2150352 | 0.0101 | -2.6322036 | -2.79065 | -0.7561213 |
| WDR45 | NM_007075 | 0.066993 | 0.000407 | -3.6849086 | -0.03918 | -0.7565468 |
| RUNX2 | AL353944 | 0.1474008 | 0.00389 | -2.970069 | -1.98563 | -0.7593278 |
| LBR | NM_002296 | 0.1443034 | 0.00369 | -2.9885234 | -1.93942 | -0.7596393 |
| NSDHL | NM_015922 | 0.1759761 | 0.00616 | -2.8109035 | -2.3747 | -0.760252 |
| CHST11 | NM_018413 | 0.0908794 | 0.00095 | -3.4276982 | -0.77515 | -0.7608388 |
| CCNJL | AK024228 | 0.1365237 | 0.00308 | -3.0501462 | -1.78501 | -0.7637568 |
| NADK | AK023114 | 0.1355343 | 0.00301 | -3.0570412 | -1.76586 | -0.7656844 |
| SSTR3 | NM_001051 | 0.3712366 | 0.0467 | -2.0206313 | -4.01931 | -0.7672603 |
| PRKDC | NM_006904 | 0.2285794 | 0.0124 | -2.556056 | -2.96098 | -0.7696052 |
| KCNK7 | NM_005714 | 0.2267728 | 0.0119 | -2.5705078 | -2.92897 | -0.7697484 |
| LOC101928474 | AK000958 | 0.0730455 | 0.000532 | -3.6051096 | -0.27155 | -0.7710967 |
| RNF130 | NM_018434 | 0.1197727 | 0.0023 | -3.1453279 | -1.53772 | -0.7714667 |
| AKAP13 | NM_006738 | 0.0468849 | 0.000186 | -3.9131954 | 0.64477 | -0.7718779 |
| RGS1 | NM_002922 | 0.0910034 | 0.001 | -3.4109777 | -0.82166 | -0.7728895 |
| LCORL | AL133031 | 0.1443034 | 0.0037 | -2.9876855 | -1.94152 | -0.7738549 |
| IFRD1 | NM_001550 | 0.1145655 | 0.00203 | -3.1873394 | -1.42741 | -0.7741047 |
| BRPF1 | NM_004634 | 0.3749151 | 0.048 | -2.008865 | -4.0381 | -0.775612 |
| PTGFR | NM_000959 | 0.2884872 | 0.0232 | -2.3140811 | -3.47271 | -0.7797009 |
| SMCHD1 | AB014550 | 0.0879607 | 0.000872 | -3.4543081 | -0.70078 | -0.7847331 |
| GVINP1 | AK023435 | 0.1172048 | 0.00214 | -3.1692422 | -1.47506 | -0.788631 |
| GJB6 | NM_006783 | 0.0317598 | 9.53E-05 | -4.1018472 | 1.23038 | -0.7892918 |
| MX1 | NM_002462 | 0.2872899 | 0.023 | -2.3160339 | -3.46996 | -0.7896604 |
| FAM126B | AK001843 | 0.0966792 | 0.00126 | -3.3392762 | -1.01921 | -0.7905729 |
| MS4A7 | AF309653 | 0.3075627 | 0.0272 | -2.249109 | -3.60341 | -0.7906339 |
| TNFRSF1B | NM_001066 | 0.2021841 | 0.0085 | -2.6960458 | -2.64463 | -0.7911471 |
| MAP3K14 | AJ008144 | 0.1071625 | 0.00163 | -3.2581207 | -1.23902 | -0.7922134 |
| NLRP3 | NM_004895 | 0.0544513 | 0.000239 | -3.8405825 | 0.42421 | -0.792594 |
| LRRK2 | AK026776 | 0.3360897 | 0.0366 | -2.124896 | -3.84399 | -0.7928868 |
| FMNL1 | NM_005892 | 0.2051866 | 0.00877 | -2.6845362 | -2.67117 | -0.796037 |
| RICTOR | AK024327 | 0.080922 | 0.000694 | -3.5244649 | -0.50273 | -0.7965648 |
| ECE1 | NM_001397 | 0.0803722 | 0.000682 | -3.5297315 | -0.48775 | -0.7968762 |
| PLEKHO1 | NM_016274 | 0.0858696 | 0.000805 | -3.4788481 | -0.63183 | -0.7978394 |
| PELI1 | AF302505 | 0.142152 | 0.00353 | -3.0029275 | -1.90319 | -0.7982523 |
| FMNL1 | AJ008131 | 0.1024046 | 0.00143 | -3.2988286 | -1.12927 | -0.8006567 |
| TSPAN2 | AK022144 | 0.2545873 | 0.0163 | -2.4527322 | -3.18534 | -0.8008387 |
| NCF2 | NM_000433 | 0.2643568 | 0.0181 | -2.4129029 | -3.25811 | -0.801514 |
| GARNL3 | AK025650 | 0.324739 | 0.0334 | -2.1639155 | -3.76906 | -0.802386 |
| HSPA6 | NM_002155 | 0.2720497 | 0.0198 | -2.3752996 | -3.34829 | -0.8072849 |
| PPP1R15A | NM_014330 | 0.130982 | 0.00277 | -3.0842734 | -1.69603 | -0.8081713 |
| CASS4 | NM_020356 | 0.0644756 | 0.000314 | -3.7610324 | 0.18578 | -0.8102381 |
| CPPED1 | NM_018340 | 0.1393572 | 0.00328 | -3.0276507 | -1.84069 | -0.811493 |
| ARHGAP15 | NM_018460 | 0.2144484 | 0.01 | -2.6353911 | -2.78342 | -0.8115815 |
| NCOA2 | NM_006540 | 0.0436668 | 0.00016 | -3.9577284 | 0.77487 | -0.8132238 |
| PPP1R10 | NM_002714 | 0.0966792 | 0.00128 | -3.3333451 | -1.03541 | -0.8137098 |
| PISD | AL050371 | 0.161646 | 0.00482 | -2.8965647 | -2.16744 | -0.8207608 |
| ALKBH5 | AK000315 | 0.1920931 | 0.00752 | -2.7402283 | -2.54189 | -0.8217116 |
| KIAA1551 | NM_018169 | 0.2272024 | 0.0122 | -2.56207 | -2.94768 | -0.8231633 |
| APLN | NM_017413 | 0.2643568 | 0.0182 | -2.4102755 | -3.27485 | -0.82363 |
| PTPRC | NM_002838 | 0.3340626 | 0.0361 | -2.1306171 | -3.8332 | -0.8251902 |
| SLC2A3 | NM_006931 | 0.2551724 | 0.0165 | -2.4480053 | -3.19542 | -0.8265202 |
| RRAGA | NM_006570 | 0.0098039 | 7.88E-06 | -4.7673449 | 3.42731 | -0.8265984 |
| TREML2 | AK023755 | 0.1624342 | 0.00486 | -2.8936529 | -2.17457 | -0.8270442 |
| CEACAM5 | M29540 | 0.1464852 | 0.00384 | -2.9749602 | -1.9734 | -0.8273218 |
| CCR3 | NM_001837 | 0.2387573 | 0.0139 | -2.5141193 | -3.05299 | -0.8314381 |
| CXorf21 | AK021639 | 0.0879607 | 0.000857 | -3.4611226 | -0.6851 | -0.8332196 |
| CDK3 | NM_001258 | 0.0913797 | 0.00103 | -3.4031431 | -0.84339 | -0.8342567 |
| UBE2D1 | NM_003338 | 0.1956321 | 0.00781 | -2.7265236 | -2.5739 | -0.8366763 |
| IFI44 | NM_006417 | 0.3150854 | 0.0304 | -2.2022979 | -3.69594 | -0.8381819 |
| BTG2 | NM_006763 | 0.1728702 | 0.00586 | -2.831315 | -2.32059 | -0.8389248 |
| IFIT3 | AF026943 | 0.3085383 | 0.028 | -2.2372429 | -3.62757 | -0.8393088 |
| CXCL8 | M17017 | 0.2713773 | 0.0197 | -2.3789826 | -3.34064 | -0.843495 |
| OSM | NM_020530 | 0.1765189 | 0.00622 | -2.8074882 | -2.38286 | -0.8440827 |
| ANKRD2 | AJ249975 | 0.2629566 | 0.0177 | -2.4212462 | -3.25177 | -0.8464435 |
| MAP3K14 | AJ008151 | 0.0966792 | 0.0012 | -3.3539784 | -0.97895 | -0.8466987 |
| H3F3A | NM_002107 | 0.094966 | 0.00115 | -3.3681343 | -0.94007 | -0.8496461 |
| CELF2 | NM_006561 | 0.1728702 | 0.00585 | -2.8289964 | -2.33134 | -0.8500393 |
| SOD2 | D17152 | 0.1719441 | 0.00568 | -2.8393726 | -2.30638 | -0.8515656 |
| PIM2 | NM_006875 | 0.0788364 | 0.000654 | -3.5439577 | -0.45108 | -0.8528664 |
| CASP8 | NM_001228 | 0.0859672 | 0.000811 | -3.4764525 | -0.63858 | -0.8532734 |
| SMAP2 | AL137764 | 0.2694389 | 0.0193 | -2.3870894 | -3.32377 | -0.8548522 |
| PTPRE | NM_006504 | 0.1187233 | 0.00225 | -3.1528066 | -1.51817 | -0.8552752 |
| CXCR4 | NM_003467 | 0.0908794 | 0.000979 | -3.4184895 | -0.80078 | -0.8560333 |
| CSRNP1 | AL117565 | 0.0966792 | 0.00128 | -3.3348225 | -1.03416 | -0.856625 |
| FAM49A | AK001942 | 0.2214344 | 0.0113 | -2.5924108 | -2.87077 | -0.8569144 |
| COL1A2 | L47668 | 0.3039961 | 0.0262 | -2.2644274 | -3.57373 | -0.8645162 |
| IFNA8 | K01900 | 0.1212124 | 0.00234 | -3.1417137 | -1.54908 | -0.8671015 |
| NPC2 | NM_006432 | 0.1610191 | 0.00478 | -2.8997325 | -2.15968 | -0.8710067 |
| ZNF226 | NM_016444 | 0.2541018 | 0.0161 | -2.4590416 | -3.17066 | -0.8738978 |
| SERPINA1 | NM_000295 | 0.1417552 | 0.0035 | -3.0060632 | -1.89528 | -0.8760338 |
| SH3PXD2B | AB037716 | 0.066993 | 0.000369 | -3.71383 | 0.04592 | -0.8773082 |
| RABGAP1L | NM_014857 | 0.0503883 | 0.000202 | -3.8886785 | 0.57 | -0.8831133 |
| IFI44L | NM_006820 | 0.3401834 | 0.0376 | -2.1137031 | -3.86405 | -0.8838573 |
| SH2B3 | NM_005475 | 0.199617 | 0.00824 | -2.7072352 | -2.61874 | -0.8855372 |
| PAPOLG | AF075029 | 0.1975424 | 0.00798 | -2.7193522 | -2.59101 | -0.885935 |
| G0S2 | NM_015714 | 0.1853789 | 0.00704 | -2.7635876 | -2.48701 | -0.8869926 |
| NABP1 | AK026486 | 0.3110118 | 0.0289 | -2.2238907 | -3.6538 | -0.8897617 |
| IGF2R | S80797 | 0.1144986 | 0.00201 | -3.1896887 | -1.4212 | -0.8918388 |
| PAK3 | AF070581 | 0.1221433 | 0.00237 | -3.1418506 | -1.54782 | -0.895045 |
| LOC105375026 | AL117529 | 0.3074274 | 0.0272 | -2.2490427 | -3.60427 | -0.9059279 |
| NLRP1 | NM_014922 | 0.0357556 | 0.000117 | -4.0447861 | 1.05138 | -0.9103353 |
| MYO15B | AK021565 | 0.2794145 | 0.0212 | -2.3485825 | -3.40347 | -0.9134858 |
| FGL2 | NM_006682 | 0.176956 | 0.00625 | -2.8060779 | -2.38623 | -0.9139551 |
| EFHC2 | AK026496 | 0.0910034 | 0.00101 | -3.4074114 | -0.83156 | -0.9140735 |
| KMT2B | NM_014727 | 0.2367505 | 0.0135 | -2.528345 | -2.98007 | -0.9149807 |
| IFIT1 | NM_001548 | 0.2709425 | 0.0196 | -2.3825456 | -3.32092 | -0.9154257 |
| HLA-F | NM_018950 | 0.1525419 | 0.00431 | -2.935571 | -2.07141 | -0.9166671 |
| DUSP1 | NM_004417 | 0.178177 | 0.00641 | -2.7969399 | -2.40801 | -0.9216271 |
| SMOC2 | AJ249902 | 0.1971121 | 0.00793 | -2.7209157 | -2.58697 | -0.9244333 |
| NIFK | AK027097 | 0.3090079 | 0.0283 | -2.2352982 | -3.58905 | -0.9389342 |
| ARGLU1 | NM_018011 | 0.2446332 | 0.0147 | -2.4925475 | -3.09982 | -0.9416925 |
| SLC22A1 | NM_003057 | 0.0966792 | 0.00129 | -3.3319855 | -1.03912 | -0.9479566 |
| AQP9 | AF016495 | 0.279121 | 0.0211 | -2.3514767 | -3.397 | -0.9543944 |
| MBOAT7 | S82470 | 0.1107084 | 0.00188 | -3.2121027 | -1.36186 | -0.9564077 |
| ZNF25 | X52350 | 0.0966792 | 0.0012 | -3.3538188 | -0.97939 | -0.9566005 |
| SCGB2A1 | NM_002407 | 0.1753086 | 0.00604 | -2.818982 | -2.35609 | -0.9639237 |
| CASP4 | NM_001225 | 0.0730455 | 0.000477 | -3.6374748 | -0.17774 | -0.9652671 |
| IRF1 | NM_002198 | 0.1571156 | 0.00458 | -2.9156453 | -2.12164 | -0.9722315 |
| SULF2 | AB033073 | 0.0153369 | 2.27E-05 | -4.4912194 | 2.4926 | -0.9736736 |
| KIAA1257 | AB033083 | 0.1277833 | 0.00261 | -3.1044254 | -1.64404 | -0.9777363 |
| CKAP4 | NM_006825 | 0.0910034 | 0.000992 | -3.4142339 | -0.81261 | -0.9825318 |
| XPO6 | AB002368 | 0.0730455 | 0.000473 | -3.6398273 | -0.17089 | -0.9869742 |
| CLEC7A | AY009090 | 0.0737062 | 0.000581 | -3.5782505 | -0.34896 | -0.9906109 |
| GPR65 | NM_003608 | 0.0836963 | 0.000771 | -3.4920321 | -0.59465 | -1.004214 |
| MBNL1 | AJ227863 | 0.0283192 | 6.68E-05 | -4.2002966 | 1.54292 | -1.0064031 |
| DIO3OS | AF305836 | 0.2369215 | 0.0136 | -2.5228639 | -3.03391 | -1.0118955 |
| H3F3AP4///H3F3A | D17130 | 0.1037891 | 0.00147 | -3.2897135 | -1.15393 | -1.0126566 |
| STK17B | NM_004226 | 0.1159976 | 0.00209 | -3.1776591 | -1.45292 | -1.0196647 |
| RGS2 | NM_002923 | 0.331172 | 0.0351 | -2.1420361 | -3.81161 | -1.020273 |
| MX2 | M30818 | 0.1065048 | 0.00159 | -3.2661754 | -1.21739 | -1.0225416 |
| PKP4 | NM_003628 | 0.2961077 | 0.0242 | -2.2958301 | -3.5102 | -1.0228843 |
| CLIC1 | NM_001288 | 0.0153369 | 2.30E-05 | -4.4877248 | 2.48097 | -1.0346729 |
| LAPTM5 | NM_006762 | 0.1655217 | 0.00512 | -2.8760658 | -2.21749 | -1.0349816 |
| KALRN | NM_007064 | 0.1393722 | 0.00329 | -3.0267205 | -1.84305 | -1.0380823 |
| HCP5 | NM_006674 | 0.0737062 | 0.000584 | -3.5764928 | -0.35401 | -1.0480437 |
| MCL1 | AF118124 | 0.0358996 | 0.000121 | -4.034591 | 1.01956 | -1.0497489 |
| OSGIN2 | NM_004337 | 0.0097313 | 7.12E-06 | -4.7931932 | 3.51636 | -1.0547588 |
| SELL | NM_000655 | 0.2905662 | 0.0235 | -2.3082372 | -3.48516 | -1.0587875 |
| IFIT2 | AF026944 | 0.1301758 | 0.00275 | -3.0874362 | -1.68789 | -1.0697117 |
| PTPN6 | NM_002831 | 0.1107084 | 0.00188 | -3.2114799 | -1.36351 | -1.0748278 |
| RAF1 | NM_002880 | 0.0277983 | 6.38E-05 | -4.2126026 | 1.58231 | -1.0794437 |
| CTSS | NM_004079 | 0.1139781 | 0.00197 | -3.1967765 | -1.40247 | -1.089856 |
| KLF2 | NM_016270 | 0.0966792 | 0.00127 | -3.3369434 | -1.02558 | -1.0900318 |
| HCAR3 | NM_006018 | 0.1794227 | 0.00653 | -2.790592 | -2.4231 | -1.1076718 |
| KCNQ3 | NM_004519 | 0.1093261 | 0.00178 | -3.2302463 | -1.31589 | -1.113212 |
| DOCK4 | NM_014705 | 0.0097313 | 7.30E-06 | -4.7869666 | 3.49488 | -1.1167152 |
| ZBP1 | AJ300575 | 0.0277983 | 6.40E-05 | -4.2117093 | 1.57945 | -1.1281701 |
| ACSL1 | NM_001995 | 0.1325955 | 0.00284 | -3.076549 | -1.71589 | -1.1489662 |
| STARD13-AS | AL137709 | 0.209542 | 0.00948 | -2.6560358 | -2.73648 | -1.1572813 |
| CAMK2D | AF071569 | 0.2755652 | 0.0205 | -2.3649302 | -3.3449 | -1.1604789 |
| BTNL8 | AK025111 | 0.0153369 | 2.26E-05 | -4.4923004 | 2.4962 | -1.1669001 |
| FPR1 | NM_002029 | 0.1443034 | 0.00371 | -2.9868503 | -1.94362 | -1.1743919 |
| ARAP1 | AB018325 | 0.0097313 | 4.46E-06 | -4.9125806 | 3.93086 | -1.1986411 |
| PTGS2 | NM_000963 | 0.0097313 | 7.11E-06 | -4.7936806 | 3.51804 | -1.2074844 |
| MXD1 | NM_002357 | 0.066993 | 0.000383 | -3.7048489 | 0.01461 | -1.2176916 |
| TMEM123 | AL110202 | 0.0860796 | 0.000826 | -3.4711422 | -0.65352 | -1.2221351 |
| ZFP36 | NM_003407 | 0.0737062 | 0.00057 | -3.5840633 | -0.33224 | -1.2352304 |
| BNIP3L | NM_004331 | 5.63E-05 | 3.02E-09 | -6.6426012 | 10.39411 | -1.2373198 |
| PREX1 | AB037836 | 0.0357556 | 0.000114 | -4.0507318 | 1.06995 | -1.2396713 |
| CYTH4 | NM_013385 | 0.0734366 | 0.00055 | -3.5950146 | -0.3007 | -1.2796964 |
| FUS | NM_004960 | 0.0730455 | 0.000486 | -3.6321946 | -0.19308 | -1.3222613 |
| TREM1 | NM_018643 | 0.0105716 | 1.13E-05 | -4.6735708 | 3.10645 | -1.3514076 |
| FBXL5 | AF157323 | 0.0889842 | 0.000915 | -3.4394867 | -0.74225 | -1.3779463 |
| QPCT | NM_012413 | 0.0097313 | 4.68E-06 | -4.9005058 | 3.8887 | -1.4120226 |
| LITAF | NM_004862 | 0.0437326 | 0.000165 | -3.9463896 | 0.74651 | -1.4823606 |
| MME | NM_007289 | 0.0021339 | 3.43E-07 | -5.5440759 | 6.20301 | -1.6628944 |
| CHI3L1 | NM_001276 | 0.0002775 | 2.97E-08 | -6.1198825 | 8.36991 | -1.7527005 |
| EVI2B | NM_006495 | 0.010442 | 1.03E-05 | -4.6970557 | 3.18648 | -1.8288686 |
