# Supplementary material for: Comprehensive Analysis of Common Different Gene Expression Signatures in the Neutrophils of Sepsis
Source: Biomed Res Int. 2021 Apr 17;2021:6655425. doi: 10.1155/2021/6655425 (PMC8077712; doi:10.1155/2021/6655425)
Supplement: Supplementary 7 [file 6655425.f7.docx]

Table S7. Detailed information of DEGs in GSE5772

| Gene symbol | probe ID | adj.P.Val | P.Value | t Value | B value | logFC |
| --- | --- | --- | --- | --- | --- | --- |
| ZSCAN9 | AF024699 | 0.0111961 | 7.26E-05 | 4.1794333 | 1.483026 | 1.47356803 |
| LAIR1 | NM_002287 | 0.0156667 | 0.000152 | 3.9620866 | 0.822532 | 1.40762317 |
| DNAH10 | AJ132089 | 0.113181 | 0.00691 | 2.7693085 | -2.527838 | 1.38399656 |
| RPL11 | NM_000975 | 0.0150557 | 0.000139 | 3.9770743 | 0.894201 | 1.36822733 |
| PSMB7 | NM_002799 | 0.0013805 | 1.04E-06 | 5.2310032 | 5.338311 | 1.30016332 |
| NDUFC2 | NM_004549 | 0.0008296 | 8.89E-08 | 5.7891731 | 7.59634 | 1.29133324 |
| SSBP1 | NM_003143 | 0.0275811 | 0.000399 | 3.7068152 | -0.029716 | 1.29090592 |
| RPL18A | L05093 | 0.054419 | 0.00147 | 3.2777014 | -1.20567 | 1.28310458 |
| SENP3-EIF4A1///SNORA67///EIF4A1 | D17188 | 0.0042569 | 9.64E-06 | 4.6823046 | 3.301401 | 1.2765668 |
| NDUFS4 | NM_002495 | 0.002974 | 4.62E-06 | 4.8602956 | 3.971726 | 1.26057081 |
| XBP1 | NM_005080 | 0.0063444 | 2.60E-05 | 4.4197587 | 2.399427 | 1.18503846 |
| CDK5RAP2 | NM_018249 | 0.0008875 | 1.46E-07 | 5.6854355 | 7.13931 | 1.18392751 |
| CYP1B1 | NM_000104 | 0.0186103 | 0.000207 | 3.8744941 | 0.546942 | 1.18025965 |
| HSP90AA1 | X15183 | 0.01599 | 0.000157 | 3.936943 | 0.773491 | 1.16679063 |
| PPDPF | AK024699 | 0.0651052 | 0.0022 | 3.1507156 | -1.565148 | 1.15881823 |
| C16orf59 | AK023971 | 0.004513 | 1.21E-05 | 4.6333387 | 3.09779 | 1.15381729 |
| PMEL | NM_006928 | 0.0756665 | 0.00307 | 3.039503 | -1.867217 | 1.13160104 |
| KRT8 | D28446 | 0.0939476 | 0.00474 | 2.8922188 | -2.248032 | 1.13119414 |
| AFP | NM_001134 | 0.0827977 | 0.00367 | 2.9791759 | -2.041051 | 1.12518036 |
| MRPS18A | NM_018135 | 0.0584922 | 0.00174 | 3.2281732 | -1.349627 | 1.11131259 |
| PYHIN1 | AK024890 | 0.2702815 | 0.0421 | 2.1076977 | -3.865237 | 1.10440497 |
| CST7 | NM_003650 | 0.1123742 | 0.0068 | 2.7653349 | -2.582495 | 1.09852057 |
| SORT1 | AK000757 | 0.0094827 | 5.34E-05 | 4.2297998 | 1.742941 | 1.09821349 |
| RPL27 | NM_000988 | 0.0357683 | 0.000653 | 3.5229122 | -0.515226 | 1.09664234 |
| RPS27L | NM_015920 | 0.0063444 | 2.61E-05 | 4.426348 | 2.400035 | 1.09559588 |
| APOH | NM_000042 | 0.0044912 | 1.08E-05 | 4.6498811 | 3.196791 | 1.09152881 |
| PSMB6 | D29012 | 0.0030828 | 4.96E-06 | 4.8430031 | 3.91069 | 1.08885082 |
| YWHAG | NM_012479 | 0.0137785 | 0.000111 | 4.0400394 | 1.087824 | 1.07917226 |
| EID1 | NM_014335 | 0.0009647 | 3.62E-07 | 5.4711294 | 6.299837 | 1.07789428 |
| PSMA6 | X59417 | 0.0037227 | 7.71E-06 | 4.7387673 | 3.507112 | 1.07763864 |
| KIAA1143 | AB032969 | 0.000986 | 4.75E-07 | 5.4072771 | 6.049793 | 1.07511787 |
| TM4SF1 | X75684 | 0.0303122 | 0.000461 | 3.6388759 | -0.182122 | 1.07294859 |
| ATP5A1 | NM_004046 | 0.0245148 | 0.000334 | 3.7260391 | 0.100666 | 1.07177268 |
| RPL3 | NM_000967 | 0.1863506 | 0.0193 | 2.3813704 | -3.421568 | 1.06486351 |
| DROSHA | NM_013235 | 0.0008875 | 2.16E-07 | 5.6053878 | 6.763865 | 1.06051478 |
| ITGA7 | NM_002206 | 0.0087173 | 4.73E-05 | 4.2914793 | 1.867111 | 1.05328772 |
| FCHSD2 | NM_014824 | 0.2222798 | 0.0281 | 2.2330716 | -3.738235 | 1.05064051 |
| FAM167A | AF124366 | 0.0018869 | 2.22E-06 | 5.0500318 | 4.640509 | 1.04582154 |
| ATP5I | NM_007100 | 0.0015575 | 1.64E-06 | 5.127444 | 4.903844 | 1.03593912 |
| GAREM1 | AK025263 | 0.0901352 | 0.00444 | 2.9797462 | -2.061111 | 1.03430603 |
| KPNA2 | NM_002266 | 0.0759704 | 0.00313 | 3.0330003 | -1.884375 | 1.03230588 |
| SNRPC | NM_003093 | 0.0719307 | 0.00282 | 3.0688575 | -1.791255 | 1.02198426 |
| OIT3 | AF075085 | 0.004513 | 1.20E-05 | 4.6254872 | 3.10041 | 1.01878452 |
| SLC25A5 | NM_001152 | 0.0155686 | 0.000147 | 3.9873342 | 0.856394 | 1.01862543 |
| IGFBP7 | NM_001553 | 0.0109317 | 6.84E-05 | 4.1697736 | 1.526724 | 1.01429521 |
| HBZ | NM_005332 | 0.0756724 | 0.0031 | 3.0364273 | -1.883426 | 1.01321962 |
| GADD45A | NM_001924 | 0.004513 | 1.18E-05 | 4.6205902 | 3.119173 | 1.00989392 |
| HSPD1 | NM_002156 | 0.0833651 | 0.00374 | 2.9773431 | -2.04137 | 1.00987165 |
| HDDC2 | NM_016063 | 0.0150499 | 0.000138 | 3.9735319 | 0.88526 | 1.00789399 |
| ATP5O | NM_001697 | 0.0249978 | 0.000342 | 3.7239836 | 0.087867 | 1.00471446 |
| BRS3 | NM_001727 | 0.1688731 | 0.0156 | 2.5064636 | -3.0083 | 1.0040483 |
| CTSC | NM_001814 | 0.0015575 | 1.67E-06 | 5.1019495 | 4.904979 | 1.00395661 |
| LRPAP1 | NM_002337 | 0.0010003 | 5.71E-07 | 5.3767273 | 5.889073 | 0.99622076 |
| TRMT112 | NM_016404 | 0.0063444 | 2.25E-05 | 4.4592311 | 2.52985 | 0.99463097 |
| HSPA4 | AB023420 | 0.0063444 | 2.48E-05 | 4.4388568 | 2.443379 | 0.99339824 |
| MRPL33 | NM_004891 | 0.0014968 | 1.36E-06 | 5.1768077 | 5.088126 | 0.98960185 |
| SNRPD1 | NM_006938 | 0.0430649 | 0.000928 | 3.4177875 | -0.814974 | 0.98740269 |
| LSM8 | NM_016200 | 0.0010003 | 6.75E-07 | 5.3328117 | 5.729001 | 0.98443633 |
| LGALS1 | NM_002305 | 0.0766741 | 0.00323 | 3.0202001 | -1.924188 | 0.97973201 |
| ANXA1 | NM_000700 | 0.0323271 | 0.000542 | 3.579752 | -0.336033 | 0.97877827 |
| BRIX1 | NM_018321 | 0.0113659 | 7.86E-05 | 4.1300189 | 1.39799 | 0.9764168 |
| SOD1 | NM_000454 | 0.054419 | 0.00143 | 3.2851376 | -1.202984 | 0.96624003 |
| PTPN11 | D13540 | 0.0023334 | 3.50E-06 | 4.9421575 | 4.222899 | 0.96339563 |
| BACE2 | AK023647 | 0.1624289 | 0.0143 | 2.5302884 | -2.957536 | 0.95892526 |
| SPARC | NM_003118 | 0.0584922 | 0.00175 | 3.2217183 | -1.380348 | 0.94755717 |
| TBC1D22B | AK021840 | 0.007146 | 3.33E-05 | 4.3594893 | 2.176361 | 0.94538456 |
| UQCRH | NM_006004 | 0.1856094 | 0.0191 | 2.3844245 | -3.439502 | 0.94337367 |
| HSP90B1 | D16892 | 0.0456285 | 0.00103 | 3.3976103 | -0.892525 | 0.94053594 |
| TFRC | NM_003234 | 0.0683784 | 0.00253 | 3.104576 | -1.694909 | 0.9404829 |
| YWHAQ | NM_006826 | 0.0628051 | 0.00206 | 3.1722345 | -1.51433 | 0.9388927 |
| ZBTB32 | NM_014383 | 0.0133459 | 0.000104 | 4.063093 | 1.153771 | 0.93820197 |
| NDUFS5 | NM_004552 | 0.0665112 | 0.00236 | 3.1250589 | -1.645382 | 0.93428168 |
| CBX5 | NM_012117 | 0.0086874 | 4.50E-05 | 4.2821558 | 1.903963 | 0.92791142 |
| NDUFS6 | NM_004553 | 0.0321337 | 0.000525 | 3.5904865 | -0.306414 | 0.92755793 |
| PRDX4 | NM_006406 | 0.0143077 | 0.000122 | 4.0069347 | 0.997283 | 0.92581693 |
| TNNI3 | NM_000363 | 0.0156288 | 0.00015 | 3.9520501 | 0.814455 | 0.92309486 |
| CDKN3 | NM_005192 | 0.0042569 | 9.81E-06 | 4.6807759 | 3.288446 | 0.92007446 |
| SPTSSA | AL080066 | 0.0063444 | 2.72E-05 | 4.4201951 | 2.361751 | 0.9191368 |
| LOC642852 | AL390181 | 0.0133459 | 0.000104 | 4.0522679 | 1.143502 | 0.91374189 |
| LOC115110 | AL359943 | 0.0169332 | 0.000171 | 3.9158651 | 0.701434 | 0.91281421 |
| MRPS10 | NM_018141 | 0.0394144 | 0.000805 | 3.4659991 | -0.677341 | 0.90709738 |
| MEA1 | NM_014623 | 0.0037227 | 7.78E-06 | 4.7364028 | 3.501905 | 0.90600621 |
| CKS2 | NM_001827 | 0.0640443 | 0.00215 | 3.1558827 | -1.562658 | 0.90590522 |
| ANXA2 | NM_004039 | 0.1326671 | 0.0095 | 2.6490869 | -2.842934 | 0.90255609 |
| ATP1A2 | NM_000702 | 0.0548516 | 0.00154 | 3.2620417 | -1.267891 | 0.89741962 |
| UPF3A | AK001069 | 0.0018869 | 2.17E-06 | 5.0518908 | 4.664803 | 0.89530461 |
| MLANA | NM_005511 | 0.005506 | 1.59E-05 | 4.5743704 | 2.85279 | 0.89513143 |
| ATOX1 | NM_004045 | 0.0021188 | 2.84E-06 | 4.9768214 | 4.419521 | 0.89228493 |
| CANX | L10284 | 0.0479056 | 0.00114 | 3.3548653 | -1.011237 | 0.89040503 |
| CACNA1E | NM_000721 | 0.0669542 | 0.0024 | 3.118839 | -1.654436 | 0.8897582 |
| DBI | M14200 | 0.0063444 | 2.28E-05 | 4.4562358 | 2.51935 | 0.88622907 |
| NR5A1 | NM_004959 | 0.0064573 | 2.93E-05 | 4.3983101 | 2.295421 | 0.88284225 |
| RPSA | NM_002295 | 0.2539274 | 0.0375 | 2.1105314 | -3.997166 | 0.8777499 |
| PSMA5 | NM_002790 | 0.0111961 | 7.54E-05 | 4.1454577 | 1.439891 | 0.87575626 |
| ABCC4 | NM_005845 | 0.0133459 | 0.000103 | 4.0577982 | 1.153916 | 0.87261785 |
| CD2BP2 | NM_006110 | 0.0704124 | 0.00267 | 3.12872 | -1.626712 | 0.87194052 |
| MYO19 | AK026518 | 0.0105279 | 6.37E-05 | 4.1845465 | 1.587919 | 0.86916078 |
| FKBP2 | NM_004470 | 0.0357683 | 0.00065 | 3.5362199 | -0.487794 | 0.86161211 |
| MCFD2 | M23161 | 0.0124946 | 9.17E-05 | 4.0959492 | 1.262765 | 0.86077905 |
| SSR4 | NM_006280 | 0.054419 | 0.00145 | 3.2789913 | -1.218823 | 0.85945874 |
| NDUFA6 | NM_002490 | 0.0465561 | 0.00108 | 3.3718405 | -0.956128 | 0.85460444 |
| HNRNPA1 | NM_002136 | 0.1740706 | 0.0167 | 2.4367574 | -3.302421 | 0.85319697 |
| BTF3 | X74070 | 0.038955 | 0.000778 | 3.4737476 | -0.66186 | 0.85208526 |
| UPP1 | NM_003364 | 0.0086874 | 4.42E-05 | 4.29401 | 1.92449 | 0.8498283 |
| RRBP1 | NM_004587 | 0.0086874 | 4.49E-05 | 4.2804733 | 1.904782 | 0.84883521 |
| ERH | NM_004450 | 0.0103334 | 6.20E-05 | 4.1879506 | 1.606677 | 0.84573729 |
| PRNP | NM_000311 | 0.047064 | 0.00111 | 3.3679979 | -0.972719 | 0.84180333 |
| CCT3 | NM_005998 | 0.067073 | 0.00241 | 3.1151587 | -1.677041 | 0.83871461 |
| TFF1 | NM_003225 | 0.1382055 | 0.0106 | 2.6100844 | -2.934054 | 0.83733941 |
| SHFM1 | NM_006304 | 0.0846647 | 0.00386 | 2.9612917 | -2.087163 | 0.83540034 |
| MRPS18C | NM_016067 | 0.0281913 | 0.000416 | 3.6574037 | -0.105557 | 0.83361546 |
| MAOB | AF088019 | 0.1737714 | 0.0166 | 2.4897855 | -3.089167 | 0.83086692 |
| BLOC1S1 | NM_001487 | 0.0275811 | 4.00E-04 | 3.6658089 | -0.079083 | 0.82655328 |
| SEC61G | NM_014302 | 0.0518214 | 0.00127 | 3.3208174 | -1.108546 | 0.82653656 |
| CALD1 | AK021691 | 0.054419 | 0.00145 | 3.2823594 | -1.205414 | 0.82559471 |
| NDUFA13 | NM_015965 | 0.066734 | 0.00237 | 3.1251751 | -1.64708 | 0.82548877 |
| ZNF205 | NM_003456 | 0.0664961 | 0.00235 | 3.1350039 | -1.621431 | 0.82463804 |
| ANLN | NM_018685 | 0.0756578 | 0.00306 | 3.0408468 | -1.880107 | 0.82135159 |
| MET | AK025784 | 0.0273716 | 0.000389 | 3.683094 | -0.034592 | 0.82110498 |
| ISY1-RAB43 | AB032986 | 0.0143077 | 0.000121 | 4.0171732 | 1.01599 | 0.81971102 |
| RPL15 | NM_002948 | 0.1000418 | 0.00547 | 2.8445412 | -2.377714 | 0.81772045 |
| VNN1 | NM_004666 | 0.0620976 | 0.00199 | 3.1792798 | -1.497504 | 0.81626402 |
| PSMA4 | NM_002789 | 0.0186103 | 0.000208 | 3.8610318 | 0.522599 | 0.81535696 |
| PRSS50 | NM_013270 | 0.0321337 | 0.00051 | 3.5987022 | -0.281652 | 0.8105866 |
| RETN | NM_020415 | 0.1297693 | 0.00906 | 2.6794304 | -2.68698 | 0.8056211 |
| SERPINB7 | NM_003784 | 0.0439456 | 0.000966 | 3.4114574 | -0.843789 | 0.80416881 |
| UQCRB | NM_006294 | 0.0543013 | 0.00141 | 3.2879223 | -1.186611 | 0.80288924 |
| PCOLCE2 | NM_013363 | 0.1757233 | 0.0171 | 2.4463832 | -3.154116 | 0.79975352 |
| CCT6A | NM_001762 | 0.1109174 | 0.00665 | 2.77462 | -2.550771 | 0.79950412 |
| IFT27 | NM_006860 | 0.0087707 | 4.84E-05 | 4.2561652 | 1.834488 | 0.79349075 |
| FAM81A | AL110257 | 0.0390066 | 0.000783 | 3.4694751 | -0.67005 | 0.79090097 |
| RAB7A | NM_018533 | 0.203751 | 0.0234 | 2.3420224 | -3.313548 | 0.79046733 |
| CYCS | D00265 | 0.0829795 | 0.00369 | 2.9778718 | -2.036994 | 0.78983033 |
| SEC11A | NM_014300 | 0.0521155 | 0.00131 | 3.3223765 | -1.092614 | 0.7889821 |
| ABRACL | AF116682 | 0.0135713 | 0.000109 | 4.0590667 | 1.119072 | 0.78800395 |
| LRP1 | NM_002332 | 0.0583001 | 0.00171 | 3.2297322 | -1.346321 | 0.78560721 |
| FOXK1 | AK022628 | 0.0386078 | 0.000759 | 3.4786626 | -0.642977 | 0.78545621 |
| SEMA3G | NM_020163 | 0.0321337 | 0.000531 | 3.5940683 | -0.315095 | 0.78457224 |
| NDUFA2 | NM_002488 | 0.0063444 | 2.55E-05 | 4.4322003 | 2.420215 | 0.78369896 |
| RPS23 | NM_001025 | 0.2117172 | 0.0252 | 2.2739933 | -3.660777 | 0.78359819 |
| NDUFB8 | NM_005004 | 0.0156288 | 0.00015 | 3.9459723 | 0.808441 | 0.78290445 |
| ACADM | NM_000016 | 0.0074031 | 3.53E-05 | 4.3441856 | 2.122977 | 0.78193512 |
| CYB5R3 | NM_007326 | 0.0321216 | 0.000508 | 3.6087828 | -0.269176 | 0.78119132 |
| ABLIM3 | NM_014945 | 0.0111961 | 7.58E-05 | 4.1458634 | 1.436839 | 0.78090451 |
| GYG1 | NM_004130 | 0.133833 | 0.00972 | 2.6385042 | -2.878291 | 0.78066977 |
| ZCCHC17 | NM_016505 | 0.0095862 | 5.55E-05 | 4.230601 | 1.717765 | 0.78056357 |
| ERICH1 | AF161437 | 0.0116187 | 8.30E-05 | 4.115161 | 1.350829 | 0.77746519 |
| AHSA1 | NM_012111 | 0.0521629 | 0.00131 | 3.3209444 | -1.109577 | 0.77704996 |
| PEBP1 | NM_002567 | 0.1452704 | 0.0117 | 2.5715171 | -3.044006 | 0.77369867 |
| HPGD | NM_000860 | 0.1028461 | 0.00579 | 2.827204 | -2.403739 | 0.77202488 |
| RSL24D1 | NM_016304 | 0.038955 | 0.000779 | 3.4889856 | -0.640387 | 0.77049302 |
| PSMB5 | NM_002797 | 0.1219105 | 0.00803 | 2.7077282 | -2.693613 | 0.76924966 |
| PCNA | NM_002592 | 0.0321337 | 0.000522 | 3.5976682 | -0.296307 | 0.76871678 |
| NEDD8 | NM_006156 | 0.054419 | 0.00149 | 3.2743654 | -1.236291 | 0.76761994 |
| SLC25A3 | NM_002635 | 0.101408 | 0.00563 | 2.8356948 | -2.391526 | 0.76416889 |
| EBNA1BP2 | NM_006824 | 0.0152194 | 0.000143 | 3.9639863 | 0.854244 | 0.76364669 |
| FBXO7 | NM_012179 | 0.0124914 | 9.10E-05 | 4.0918423 | 1.265983 | 0.76246883 |
| BCAT1 | AK025615 | 0.0231878 | 0.000286 | 3.7810338 | 0.243608 | 0.76215795 |
| PTPRB | AL080103 | 0.1456966 | 0.0117 | 2.617997 | -2.793677 | 0.76207708 |
| UQCRC2 | NM_003366 | 0.0244857 | 0.000328 | 3.7294604 | 0.105679 | 0.76130426 |
| UQCRQ | NM_014402 | 0.0756724 | 0.00309 | 3.0351262 | -1.877161 | 0.76110863 |
| SLC25A6 | AF076617 | 0.1559788 | 0.0135 | 2.5172933 | -3.168459 | 0.76091639 |
| UBE2T | NM_014176 | 0.0369575 | 0.000699 | 3.5060414 | -0.566628 | 0.76004446 |
| SNRPD2 | NM_004597 | 0.1626794 | 0.0145 | 2.4945286 | -3.185317 | 0.75942759 |
| PRKXP1 | Y10483 | 0.0094827 | 5.39E-05 | 4.2340669 | 1.740946 | 0.7588549 |
| CRLS1 | NM_019095 | 0.0035339 | 6.25E-06 | 4.7977409 | 3.696209 | 0.75718511 |
| RPS26 | NM_001029 | 0.0996693 | 0.00537 | 2.8492282 | -2.356471 | 0.75630372 |
| SDHB | NM_003000 | 0.0063444 | 2.14E-05 | 4.4754038 | 2.578751 | 0.75459926 |
| TOMM7 | NM_019059 | 0.0558319 | 0.00159 | 3.2532691 | -1.293859 | 0.75104991 |
| CCDC53 | NM_016053 | 0.0013885 | 1.19E-06 | 5.2023677 | 5.219994 | 0.74797787 |
| MRPL19 | NM_014763 | 0.0186103 | 0.000208 | 3.8604129 | 0.524755 | 0.74704501 |
| STOML2 | NM_013442 | 0.0657251 | 0.00226 | 3.1391735 | -1.599401 | 0.74680961 |
| COX16 | NM_016468 | 0.054419 | 0.00149 | 3.2727744 | -1.239248 | 0.74617194 |
| FARSA | NM_004461 | 0.1335036 | 0.00964 | 2.6464447 | -2.828374 | 0.74281062 |
| MAP1B | NM_005909 | 0.0995472 | 0.00535 | 2.8497197 | -2.372635 | 0.74132701 |
| ATP5G1 | NM_005175 | 0.1176582 | 0.0075 | 2.7341438 | -2.630475 | 0.74041988 |
| CD3D | NM_000732 | 0.1842801 | 0.0187 | 2.3941034 | -3.416892 | 0.73975481 |
| RPL22 | AF113701 | 0.0110071 | 6.96E-05 | 4.1939408 | 1.525292 | 0.73915605 |
| SNTB2 | NM_006750 | 0.0133588 | 0.000106 | 4.0464011 | 1.126603 | 0.73894046 |
| TXNL1 | NM_004786 | 0.0439456 | 0.00097 | 3.4139137 | -0.837574 | 0.73798175 |
| TBCA | NM_004607 | 0.1029763 | 0.00581 | 2.8205414 | -2.445686 | 0.73751087 |
| VAT1 | NM_006373 | 0.0620976 | 0.00199 | 3.1801736 | -1.49505 | 0.73725734 |
| TMEM208 | NM_014187 | 0.0185338 | 2.00E-04 | 3.8875648 | 0.577381 | 0.73641163 |
| FASTK | NM_006712 | 0.1695325 | 0.0158 | 2.4586774 | -3.288759 | 0.73585161 |
| TMEM230 | NM_014145 | 0.0103334 | 6.17E-05 | 4.2018998 | 1.621662 | 0.73473705 |
| POLR2G | NM_002696 | 0.0321337 | 0.000526 | 3.5858617 | -0.32346 | 0.73374193 |
| IGFBP3 | NM_000598 | 0.113181 | 0.00692 | 2.7625724 | -2.58043 | 0.73241059 |
| ACAT2 | NM_005891 | 0.023401 | 0.000292 | 3.764871 | 0.220935 | 0.73226845 |
| FST | NM_006350 | 0.0861666 | 0.00398 | 2.9567814 | -2.077942 | 0.73199394 |
| KRT19 | NM_002276 | 0.0979995 | 0.00519 | 2.8637549 | -2.329779 | 0.73124555 |
| HAUS1 | AK024747 | 0.0063444 | 2.34E-05 | 4.4497295 | 2.496671 | 0.73112552 |
| HIGD1A | NM_014056 | 0.0469649 | 0.0011 | 3.3666143 | -0.964599 | 0.73107862 |
| ANXA2P1 | M62896 | 0.2479036 | 0.0355 | 2.1332255 | -3.966805 | 0.7289075 |
| EEF1A2 | NM_001958 | 0.072697 | 0.00289 | 3.0589304 | -1.822961 | 0.72844425 |
| REXO2 | NM_015523 | 0.0585081 | 0.00177 | 3.2217425 | -1.378675 | 0.72596112 |
| MFSD12 | AF218008 | 0.0421719 | 0.000893 | 3.428411 | -0.788414 | 0.72500621 |
| HDAC2 | NM_001527 | 0.0439569 | 0.000973 | 3.4104578 | -0.836493 | 0.72345479 |
| ULK3 | AL117482 | 0.0412635 | 0.000855 | 3.4821701 | -0.673898 | 0.72249075 |
| ARL3 | NM_004311 | 0.0143077 | 0.000122 | 4.0180412 | 1.007993 | 0.71686701 |
| AKR1A1 | NM_006066 | 0.0187031 | 0.000212 | 3.8546376 | 0.506457 | 0.71679853 |
| PRSS2 | NM_002770 | 0.0360761 | 0.000671 | 3.517194 | -0.536807 | 0.71589572 |
| SNRPG | NM_003096 | 0.1071441 | 0.00623 | 2.802025 | -2.466102 | 0.71404357 |
| LOC221272 | AK025312 | 0.208278 | 0.0243 | 2.3111041 | -3.486935 | 0.71285938 |
| C14orf2 | NM_004894 | 0.0148125 | 0.000133 | 3.9834208 | 0.920654 | 0.7124663 |
| XPO5 | AF298880 | 0.004289 | 1.01E-05 | 4.6853764 | 3.261335 | 0.71092576 |
| EEF1E1 | NM_004280 | 0.0332742 | 0.000581 | 3.5640957 | -0.399091 | 0.71038749 |
| KRT18 | L32537 | 0.065773 | 0.00228 | 3.1393469 | -1.594577 | 0.71005122 |
| FAM105A | AF052146 | 0.0063444 | 2.59E-05 | 4.438198 | 2.408971 | 0.70874397 |
| COX7B | NM_001866 | 0.0939886 | 0.00481 | 2.888151 | -2.267532 | 0.70840709 |
| VRK1 | NM_003384 | 0.0063444 | 2.61E-05 | 4.4234579 | 2.397595 | 0.70829374 |
| LDHA | NM_005566 | 0.1268613 | 0.00856 | 2.6833252 | -2.781623 | 0.70821831 |
| MFSD10 | NM_001120 | 0.0825583 | 0.00364 | 2.9825786 | -2.02474 | 0.70758591 |
| NDUFA3 | NM_004542 | 0.054419 | 0.00149 | 3.2729096 | -1.226372 | 0.70614973 |
| MDH1 | NM_005917 | 0.1000418 | 0.00545 | 2.8448066 | -2.376813 | 0.70554643 |
| RPL18 | NM_000979 | 0.0551533 | 0.00155 | 3.2699131 | -1.235494 | 0.70552577 |
| SUCLG1 | AF104921 | 0.0244857 | 0.000327 | 3.730403 | 0.108604 | 0.70516813 |
| ATP5C1 | NM_005174 | 0.0275811 | 0.000399 | 3.6885184 | -0.047285 | 0.70387673 |
| GHSR | NM_004122 | 0.0143077 | 0.000121 | 4.0331059 | 1.028617 | 0.70337593 |
| PSMD1 | NM_002807 | 0.0363239 | 0.000683 | 3.5104552 | -0.548875 | 0.70323129 |
| OR5H1 | X64988 | 0.1697769 | 0.0158 | 2.4823042 | -3.099157 | 0.69799289 |
| CRYBB1 | NM_001887 | 0.0175438 | 0.000181 | 3.9056681 | 0.65086 | 0.69743137 |
| SMC3 | NM_005445 | 0.1000418 | 0.00544 | 2.8466641 | -2.363542 | 0.69600457 |
| C1orf68 | AF005081 | 0.2714443 | 0.0425 | 2.0573079 | -4.095354 | 0.69536962 |
| FKBP5 | NM_004117 | 0.1737714 | 0.0166 | 2.4378337 | -3.346066 | 0.69500032 |
| CA9 | NM_001216 | 0.0116187 | 8.34E-05 | 4.1117208 | 1.342547 | 0.69402589 |
| SERPINB6 | NM_004568 | 0.0777448 | 0.0033 | 3.0150717 | -1.939715 | 0.69070739 |
| COX5B | NM_001862 | 0.0345677 | 0.000622 | 3.539946 | -0.457808 | 0.69019781 |
| PPIH | NM_006347 | 0.0187031 | 0.000212 | 3.8645286 | 0.514933 | 0.68894963 |
| PFKL | NM_002626 | 0.095687 | 0.00498 | 2.8762961 | -2.297557 | 0.68878545 |
| EMC2 | NM_014673 | 0.0291837 | 0.000437 | 3.647377 | -0.145089 | 0.68845444 |
| NEFM | NM_005382 | 0.2434182 | 0.0339 | 2.1923026 | -3.567923 | 0.68748448 |
| PTGS1 | AF129755 | 0.2224184 | 0.0282 | 2.2646365 | -3.502442 | 0.68707283 |
| ZNF677 | AK026366 | 0.0939476 | 0.00476 | 2.8894 | -2.26334 | 0.6853576 |
| NQO1 | NM_000903 | 0.2268907 | 0.0295 | 2.2111359 | -3.798321 | 0.68477543 |
| PTPN1 | NM_002827 | 0.0439456 | 0.000965 | 3.4035321 | -0.8654 | 0.68398929 |
| ATP5G3 | NM_001689 | 0.2186316 | 0.027 | 2.2455667 | -3.742441 | 0.6808181 |
| MANF | NM_006010 | 0.0207741 | 0.000246 | 3.8243378 | 0.378036 | 0.67981634 |
| HHEX | NM_002729 | 0.0095639 | 5.48E-05 | 4.2314685 | 1.727208 | 0.67812649 |
| CWC15 | NM_016403 | 0.0215921 | 0.00026 | 3.8009293 | 0.325562 | 0.67463788 |
| RPL38 | NM_000999 | 0.1310062 | 0.00925 | 2.656258 | -2.826451 | 0.67454846 |
| NAA20 | NM_016100 | 0.0184852 | 0.000196 | 3.8820737 | 0.580615 | 0.67452196 |
| NME1 | NM_000269 | 0.178499 | 0.0175 | 2.420063 | -3.350172 | 0.6726273 |
| DDX21 | U41387 | 0.1146701 | 0.00712 | 2.7505516 | -2.609662 | 0.67161278 |
| ZNHIT2 | NM_014205 | 0.0521155 | 0.0013 | 3.3312579 | -1.063242 | 0.67149481 |
| ZNF133 | NM_003434 | 0.1295479 | 0.00896 | 2.6684467 | -2.816528 | 0.67100231 |
| EMC8 | NM_006067 | 0.0996693 | 0.0054 | 2.8503558 | -2.345847 | 0.66968226 |
| CHD6 | AK026022 | 0.0660645 | 0.00231 | 3.1323164 | -1.633088 | 0.66921876 |
| DNPH1 | NM_006443 | 0.065408 | 0.00223 | 3.1419709 | -1.605862 | 0.66887005 |
| ZC4H2 | NM_018684 | 0.0903792 | 0.00447 | 2.9147534 | -2.192141 | 0.66871774 |
| NFKBIA | NM_020529 | 0.0763139 | 0.00318 | 3.0263665 | -1.908579 | 0.66864096 |
| NDUFB10 | NM_004548 | 0.0430649 | 0.000927 | 3.4264721 | -0.793016 | 0.668481 |
| MAPRE1 | NM_012325 | 0.0201093 | 0.000235 | 3.8253589 | 0.410021 | 0.66846691 |
| ST6GAL1 | U67847 | 0.0586227 | 0.00179 | 3.2587207 | -1.294936 | 0.66792208 |
| MRPS21 | AF182417 | 0.2237543 | 0.0287 | 2.2226218 | -3.751262 | 0.66732517 |
| BTF3 | NM_001207 | 0.1019438 | 0.0057 | 2.8315164 | -2.401911 | 0.66672002 |
| ITGAE | NM_002208 | 0.0036705 | 6.91E-06 | 4.7632149 | 3.605873 | 0.66587431 |
| CHML | AK000933 | 0.0465561 | 0.00108 | 3.3697464 | -0.96507 | 0.66568759 |
| DYNLL1 | NM_003746 | 0.1274252 | 0.00868 | 2.6816139 | -2.756051 | 0.66501343 |
| MVB12B | AK024432 | 0.2654784 | 0.0407 | 2.1029337 | -3.690452 | 0.66266007 |
| DPP4 | NM_001935 | 0.0624872 | 0.00203 | 3.1712419 | -1.524696 | 0.66258448 |
| LINC00527 | AJ011409 | 0.0696501 | 0.00261 | 3.0915407 | -1.734893 | 0.66218381 |
| RPL36 | NM_015414 | 0.2842376 | 0.0469 | 2.0138535 | -4.16681 | 0.6606143 |
| SIKE1 | AK024821 | 0.0063444 | 2.65E-05 | 4.4267576 | 2.385725 | 0.65788172 |
| TCEA1 | NM_006756 | 0.0235571 | 0.000298 | 3.7513697 | 0.186892 | 0.65685731 |
| DCAF6 | NM_018442 | 0.0728907 | 0.0029 | 3.0598175 | -1.823051 | 0.65585619 |
| CDC37 | NM_007065 | 0.0214045 | 0.000257 | 3.8047153 | 0.337378 | 0.65528234 |
| TTC9 | D86980 | 0.0306289 | 0.000468 | 3.6217242 | -0.216952 | 0.65410861 |
| APOC3 | NM_000040 | 0.0186103 | 0.000205 | 3.8637634 | 0.531632 | 0.65288799 |
| SDF2L1 | AB043007 | 0.0235781 | 0.000301 | 3.7577333 | 0.195091 | 0.65193554 |
| ARL6IP5 | NM_006407 | 0.0298582 | 0.000451 | 3.6364028 | -0.175492 | 0.65186605 |
| DYNC1I1 | NM_004411 | 0.2462336 | 0.0346 | 2.1715273 | -3.688704 | 0.65088605 |
| ATP5F1 | NM_001688 | 0.0833651 | 0.00374 | 2.9758031 | -2.02749 | 0.65038664 |
| PHB | NM_002634 | 0.1439362 | 0.0114 | 2.5783895 | -3.028526 | 0.65022982 |
| FXR1 | NM_005087 | 0.0551226 | 0.00155 | 3.2664195 | -1.258179 | 0.64908185 |
| QARS | NM_005051 | 0.1510389 | 0.0127 | 2.5387324 | -3.109425 | 0.64828642 |
| PYCR2 | NM_013328 | 0.0418652 | 0.000882 | 3.4381328 | -0.764425 | 0.64684735 |
| MS4A4A | NM_016650 | 0.2082866 | 0.0244 | 2.2934369 | -3.600473 | 0.64581298 |
| MRPL16 | NM_017840 | 0.1282245 | 0.00878 | 2.6744687 | -2.793229 | 0.64544662 |
| NACA | NM_005594 | 0.0321337 | 0.000517 | 3.5987181 | -0.290184 | 0.64415331 |
| TPM2 | NM_003289 | 0.1001885 | 0.00549 | 2.8461903 | -2.366056 | 0.64317907 |
| TECTA | AF143331 | 0.1892138 | 0.02 | 2.3982502 | -3.202693 | 0.64315581 |
| TRPS1 | NM_014112 | 0.0545224 | 0.00152 | 3.2669932 | -1.24848 | 0.64265505 |
| DDX27 | NM_017895 | 0.0728907 | 0.0029 | 3.0598122 | -1.815247 | 0.64229753 |
| NUP133 | NM_018230 | 0.0064573 | 2.94E-05 | 4.4021871 | 2.293174 | 0.64186312 |
| CUTA | NM_015921 | 0.0465561 | 0.00108 | 3.3767985 | -0.947505 | 0.64106325 |
| EXOSC4 | NM_019037 | 0.0583001 | 0.00172 | 3.2340862 | -1.33516 | 0.64034152 |
| TPRKB | NM_016058 | 0.0120918 | 8.75E-05 | 4.1027574 | 1.306279 | 0.64022186 |
| UACA | AB046781 | 0.0235571 | 0.000297 | 3.761636 | 0.207192 | 0.64019068 |
| PDS5B | NM_015032 | 0.0307001 | 0.000472 | 3.6230329 | -0.210982 | 0.63751263 |
| NTS | NM_006183 | 0.2636624 | 0.0403 | 2.0801591 | -4.053258 | 0.63597596 |
| MVD | NM_002461 | 0.054419 | 0.00151 | 3.2722922 | -1.238471 | 0.63433941 |
| CDH20 | AF217289 | 0.1043401 | 0.00594 | 2.879255 | -2.262098 | 0.63394388 |
| SNORA64 | Y11158 | 0.1719556 | 0.0162 | 2.4476635 | -3.302449 | 0.6333892 |
| MT1L | NM_002450 | 0.1338297 | 0.0097 | 2.6426395 | -2.847777 | 0.63253304 |
| POLR2L | U37690 | 0.0813926 | 0.00353 | 2.9910706 | -1.992927 | 0.63099899 |
| TMEM258 | NM_014206 | 0.1469586 | 0.012 | 2.559113 | -3.072991 | 0.630276 |
| XPOT | NM_007235 | 0.0521155 | 0.00131 | 3.3162941 | -1.119828 | 0.62928422 |
| EIF2S2 | NM_003908 | 0.0866583 | 0.00403 | 2.9456424 | -2.127354 | 0.6287775 |
| RSRC1 | NM_016625 | 0.0323271 | 0.000549 | 3.5758064 | -0.353409 | 0.62730628 |
| IFI30 | NM_006332 | 0.2186316 | 0.0273 | 2.2414707 | -3.764214 | 0.62673622 |
| TCEAL9 | NM_016303 | 0.054419 | 0.00146 | 3.2767907 | -1.217942 | 0.62659763 |
| SNORD46 | X96646 | 0.0741176 | 0.00299 | 3.0646266 | -1.803492 | 0.6260844 |
| EIF4E | NM_001968 | 0.1529547 | 0.013 | 2.5305609 | -3.128014 | 0.62590134 |
| GUSB | NM_000181 | 0.0148125 | 0.000134 | 3.9812152 | 0.913482 | 0.6258496 |
| PRDX3 | NM_006793 | 0.033557 | 0.000593 | 3.5553696 | -0.414397 | 0.62528849 |
| SEPP1 | NM_005410 | 0.2261208 | 0.0293 | 2.2127736 | -3.809876 | 0.62491231 |
| MMRN1 | NM_007351 | 0.1063442 | 0.0061 | 2.8074244 | -2.461308 | 0.62412784 |
| ACAT1 | NM_000019 | 0.0456285 | 0.00103 | 3.3866416 | -0.919724 | 0.62368166 |
| ZNF644 | NM_016620 | 0.0766741 | 0.00322 | 3.02492 | -1.90726 | 0.62336312 |
| PATZ1 | AF254082 | 0.1703464 | 0.016 | 2.4538658 | -3.299401 | 0.62270759 |
| SAMSN1 | AF222927 | 0.2498502 | 0.0363 | 2.1234647 | -3.987739 | 0.62262112 |
| GRK2 | NM_001619 | 0.1757233 | 0.017 | 2.4353538 | -3.277283 | 0.62241848 |
| PPIG | NM_004792 | 0.1109174 | 0.00666 | 2.7758807 | -2.547822 | 0.62233818 |
| NUTF2 | NM_005796 | 0.1166385 | 0.00737 | 2.7423481 | -2.611173 | 0.621908 |
| RARS | NM_002887 | 0.072626 | 0.00288 | 3.0614463 | -1.817892 | 0.6193053 |
| RPLP0 | NM_001002 | 0.0667428 | 0.00239 | 3.1303361 | -1.611094 | 0.61908547 |
| RNU5A-1 | K03167 | 0.2932519 | 0.0497 | 1.9913743 | -4.199162 | 0.61894386 |
| PAPPA | AF085834 | 0.216106 | 0.0263 | 2.2922185 | -3.397241 | 0.61748857 |
| PRKD3 | NM_005813 | 0.0393257 | 0.000799 | 3.475107 | -0.652556 | 0.61707504 |
| NDUFA7 | NM_005001 | 0.0607189 | 0.00189 | 3.1946325 | -1.460156 | 0.61590113 |
| SPP1 | NM_000582 | 0.2464471 | 0.0347 | 2.1428944 | -3.946922 | 0.61464897 |
| APOM | NM_019101 | 0.0342728 | 0.00061 | 3.5432704 | -0.454283 | 0.6142523 |
| RPL10 | AB019572 | 0.2380332 | 0.0324 | 2.1718179 | -3.901811 | 0.61346991 |
| MRPS14 | AL049705 | 0.0111961 | 7.36E-05 | 4.1656423 | 1.470293 | 0.61248881 |
| VANGL2 | AB033041 | 0.0944724 | 0.00486 | 2.8876918 | -2.243478 | 0.61145713 |
| LSM3 | NM_014463 | 0.2256009 | 0.0291 | 2.2184168 | -3.769374 | 0.60976 |
| ECI2 | NM_006117 | 0.0586227 | 0.00179 | 3.2135487 | -1.402997 | 0.60968885 |
| GCN1 | U77700 | 0.1103533 | 0.00659 | 2.779489 | -2.538969 | 0.60937514 |
| PCMT1 | NM_005389 | 0.0063444 | 2.27E-05 | 4.4549361 | 2.522262 | 0.60919612 |
| MAP2K6 | U39657 | 0.0997665 | 0.00541 | 2.8480049 | -2.360189 | 0.60917992 |
| FAM98A | AL049943 | 0.0321337 | 0.000517 | 3.5975481 | -0.290786 | 0.60915128 |
| HEXB | NM_000521 | 0.1472547 | 0.0121 | 2.5589091 | -3.062948 | 0.60879544 |
| C19orf53 | NM_014047 | 0.0934903 | 0.00471 | 2.8942894 | -2.242775 | 0.6082956 |
| ATP5J | NM_001685 | 0.0722098 | 0.00284 | 3.0629149 | -1.81155 | 0.60817797 |
| ATP1B3 | NM_001679 | 0.2278065 | 0.0297 | 2.2087248 | -3.80316 | 0.60806571 |
| TNC | NM_002160 | 0.1537559 | 0.0131 | 2.5270434 | -3.136399 | 0.60720949 |
| STIP1 | NM_006819 | 0.228869 | 0.03 | 2.2070703 | -3.776465 | 0.6071194 |
| POLR3K | NM_016310 | 0.0186103 | 0.000207 | 3.8644051 | 0.52906 | 0.60691828 |
| PABPC3 | AF132026 | 0.2342629 | 0.0315 | 2.1827526 | -3.844494 | 0.60631929 |
| CD59 | M34671 | 0.0244156 | 0.000317 | 3.7344535 | 0.130845 | 0.60586819 |
| CUEDC2 | AF086406 | 0.0241346 | 0.000312 | 3.7489766 | 0.168453 | 0.60580243 |
| SNORA68 | Y11162 | 0.2117172 | 0.0251 | 2.278731 | -3.607053 | 0.60549031 |
| SMU1 | NM_018225 | 0.1166352 | 0.00735 | 2.7435695 | -2.598235 | 0.605362 |
| ETF1 | NM_004730 | 0.1463265 | 0.0119 | 2.5646432 | -3.050139 | 0.60486174 |
| RPL29P2 | AF085886 | 0.2825576 | 0.046 | 2.0220747 | -4.166419 | 0.60466609 |
| UCHL5 | NM_015984 | 0.123648 | 0.00824 | 2.6992118 | -2.724172 | 0.60414635 |
| MCM2 | NM_004526 | 0.0586227 | 0.00179 | 3.2162871 | -1.399339 | 0.60312683 |
| MB | NM_005368 | 0.1268613 | 0.00855 | 2.695928 | -2.681804 | 0.60297227 |
| MRPL36 | AF151109 | 0.0231308 | 0.000284 | 3.7777285 | 0.249415 | 0.60285546 |
| MRPS2 | NM_016034 | 0.0942217 | 0.00483 | 2.8873371 | -2.242555 | 0.60220491 |
| BBOX1 | NM_003986 | 0.2431472 | 0.0338 | 2.1834452 | -3.57419 | 0.60153354 |
| ADAM22 | AL133090 | 0.1624289 | 0.0143 | 2.4938149 | -3.221456 | 0.60137226 |
| SNRPA1 | NM_003090 | 0.1432404 | 0.0113 | 2.5851885 | -2.991864 | 0.60110173 |
| MYL12A | NM_006471 | 0.0424602 | 0.000903 | 3.4236866 | -0.806566 | 0.60022474 |
| MIXL1 | AF211891 | 0.0901352 | 0.00445 | 2.9211842 | -2.170702 | 0.59892091 |
| C14orf119 | NM_017924 | 0.032585 | 0.000557 | 3.5796781 | -0.347951 | 0.59707884 |
| MRPL27 | NM_016504 | 0.0660645 | 0.0023 | 3.1479414 | -1.593772 | 0.59682374 |
| ANXA5 | NM_001154 | 0.1646272 | 0.0149 | 2.4800722 | -3.251714 | 0.59635036 |
| CRTC3 | AK024981 | 0.0360761 | 0.000669 | 3.5218391 | -0.524766 | 0.59614595 |
| FAM131B | NM_014690 | 0.0409279 | 0.000842 | 3.4588621 | -0.699338 | 0.59556387 |
| ITIH2 | NM_002216 | 0.1040257 | 0.0059 | 2.8182295 | -2.434331 | 0.59546769 |
| MGST2 | NM_002413 | 0.0182969 | 0.000191 | 3.8869601 | 0.59704 | 0.59521987 |
| RPL5 | NM_000969 | 0.2139928 | 0.0257 | 2.2734659 | -3.660442 | 0.5950554 |
| BAMBI | NM_012342 | 0.0717801 | 0.0028 | 3.0744686 | -1.771464 | 0.59500326 |
| REPIN1 | NM_013400 | 0.0876152 | 0.00418 | 2.9514655 | -2.075757 | 0.59471446 |
| ICAM5 | NM_003259 | 0.1776292 | 0.0173 | 2.42029 | -3.385244 | 0.59466172 |
| LIPF | NM_004190 | 0.0628051 | 0.00205 | 3.1725373 | -1.525363 | 0.59429784 |
| TOP2A | NM_001067 | 0.2017761 | 0.0229 | 2.3121291 | -3.604651 | 0.59338915 |
| SDHAF3 | NM_020186 | 0.0436292 | 0.000944 | 3.4100719 | -0.846339 | 0.59230823 |
| ETFB | NM_001985 | 0.0583001 | 0.00172 | 3.2228658 | -1.380584 | 0.59204426 |
| NRG2 | NM_013982 | 0.1503974 | 0.0126 | 2.5428575 | -3.110262 | 0.59202264 |
| PREB | NM_013388 | 0.0399787 | 0.000818 | 3.4705974 | -0.676982 | 0.59193551 |
| GATB | NM_004564 | 0.1175962 | 0.00749 | 2.7421811 | -2.583141 | 0.59178142 |
| DDR1-AS1 | AL137624 | 0.1942796 | 0.021 | 2.3783878 | -3.239604 | 0.59172028 |
| MED7 | NM_004270 | 0.0064573 | 2.89E-05 | 4.4204792 | 2.309266 | 0.59167643 |
| SNX3 | NM_003795 | 0.1394995 | 0.0108 | 2.6018187 | -2.963893 | 0.59044548 |
| ASNS | NM_001673 | 0.0321337 | 0.00053 | 3.5947376 | -0.308108 | 0.59043667 |
| SLC25A36 | NM_018155 | 0.0466165 | 0.00109 | 3.3787911 | -0.943939 | 0.59037038 |
| SCAND1 | NM_016558 | 0.0426242 | 0.000909 | 3.4275271 | -0.804964 | 0.58991691 |
| SUB1 | NM_006713 | 0.0776572 | 0.00329 | 3.0184277 | -1.932296 | 0.58989764 |
| ADARB2-AS1 | AF086274 | 0.1667366 | 0.0152 | 2.472207 | -3.269847 | 0.58904573 |
| MYL3 | NM_000258 | 0.0587844 | 0.0018 | 3.2116656 | -1.400881 | 0.58797975 |
| COX5A | NM_004255 | 0.2382319 | 0.0325 | 2.1707955 | -3.852908 | 0.58637989 |
| DHX36 | AF217190 | 0.0251191 | 0.000346 | 3.7155577 | 0.063827 | 0.58631631 |
| AXL | NM_001699 | 0.1133673 | 0.00696 | 2.766223 | -2.563321 | 0.58567487 |
| HARS | NM_002109 | 0.0087173 | 4.76E-05 | 4.2738523 | 1.856435 | 0.58488694 |
| MPC2 | NM_015415 | 0.0466173 | 0.00109 | 3.3767803 | -0.947505 | 0.58321271 |
| EIF3H | NM_003756 | 0.2268822 | 0.0295 | 2.2093509 | -3.829528 | 0.58289864 |
| ARID5B | M73837 | 0.0879934 | 0.00422 | 2.9401484 | -2.096334 | 0.58242969 |
| ODAM | NM_017855 | 0.156062 | 0.0135 | 2.5175989 | -3.166752 | 0.58234927 |
| CPSF6 | NM_007007 | 0.1128359 | 0.00686 | 2.7664256 | -2.579592 | 0.58021326 |
| RPL19 | NM_000981 | 0.2019645 | 0.023 | 2.3120119 | -3.592386 | 0.57994424 |
| WARS2 | NM_015836 | 0.0874494 | 0.00416 | 2.9425508 | -2.115052 | 0.57922216 |
| CCDC47 | NM_020198 | 0.0667358 | 0.00238 | 3.1218583 | -1.646282 | 0.57914418 |
| BST2 | NM_004335 | 0.1897973 | 0.0203 | 2.3612297 | -3.500719 | 0.57887536 |
| RAB32 | NM_006834 | 0.0596571 | 0.00183 | 3.2115513 | -1.400963 | 0.57819018 |
| MRPL32 | AF161401 | 0.0939476 | 0.00478 | 2.8922346 | -2.266283 | 0.57705432 |
| CCT7 | NM_006429 | 0.2396123 | 0.0328 | 2.1663546 | -3.899786 | 0.57692397 |
| ARL6IP1 | D31885 | 0.1297693 | 0.00903 | 2.6654755 | -2.804551 | 0.57621081 |
| NUDT1 | NM_002452 | 0.0245148 | 0.00033 | 3.7273059 | 0.098995 | 0.57599746 |
| TIMM17A | NM_006335 | 0.0608758 | 0.00191 | 3.1965809 | -1.453625 | 0.57590404 |
| FRMPD1 | NM_014907 | 0.0618873 | 0.00197 | 3.1835678 | -1.479512 | 0.57512189 |
| POP7 | NM_005837 | 0.0572249 | 0.00165 | 3.2395633 | -1.323525 | 0.57466072 |
| IL6ST | NM_002184 | 0.076013 | 0.00314 | 3.0340868 | -1.883169 | 0.5736813 |
| RRP9 | NM_004704 | 0.1694233 | 0.0157 | 2.4657606 | -3.235812 | 0.57244699 |
| PALD1 | AB033100 | 0.0617907 | 0.00196 | 3.1862345 | -1.479578 | 0.57121919 |
| C11orf58 | NM_014267 | 0.0478854 | 0.00114 | 3.3542287 | -1.003248 | 0.57119786 |
| RPS6 | NM_001010 | 0.1095633 | 0.00647 | 2.7852287 | -2.524772 | 0.57111104 |
| HCCS | NM_005333 | 0.0063444 | 2.40E-05 | 4.4498848 | 2.473889 | 0.57068881 |
| FGG | NM_000509 | 0.1242184 | 0.00831 | 2.6996771 | -2.713303 | 0.5706812 |
| ATP5J2 | NM_004889 | 0.0456285 | 0.00103 | 3.3886054 | -0.911635 | 0.57063626 |
| SERBP1 | NM_015640 | 0.0951116 | 0.00493 | 2.8800938 | -2.288274 | 0.57011236 |
| LMBR1L | NM_018113 | 0.0063444 | 2.41E-05 | 4.4991542 | 2.468626 | 0.56988213 |
| EEF1G | NM_001404 | 0.0678818 | 0.00249 | 3.1257309 | -1.613825 | 0.56950052 |
| GAPDH | NM_002046 | 0.2147504 | 0.0259 | 2.2630161 | -3.707476 | 0.56913378 |
| PERP | AF317550 | 0.0939476 | 0.0048 | 2.8879296 | -2.276152 | 0.56851425 |
| VEGFB | NM_003377 | 0.0572242 | 0.00164 | 3.240884 | -1.327035 | 0.56685466 |
| SLC4A8 | AL137310 | 0.1646272 | 0.0149 | 2.4953546 | -3.121603 | 0.56669179 |
| NDUFB5 | NM_002492 | 0.242405 | 0.0337 | 2.1549861 | -3.924155 | 0.56657318 |
| HAUS3 | AF040964 | 0.1373219 | 0.0105 | 2.6116491 | -2.941259 | 0.56620661 |
| FKBP11 | NM_016594 | 0.0583001 | 0.00173 | 3.2275905 | -1.361063 | 0.56493209 |
| RCN1 | NM_002901 | 0.1292596 | 0.00893 | 2.668949 | -2.806238 | 0.56480656 |
| UFM1 | NM_016617 | 0.0682418 | 0.00251 | 3.114455 | -1.670317 | 0.56472489 |
| LAIR2 | NM_002288 | 0.1934196 | 0.0208 | 2.3593261 | -3.394701 | 0.56469397 |
| MITD1 | AL161992 | 0.0323271 | 0.000539 | 3.5957775 | -0.312993 | 0.56433261 |
| ARL4D | NM_001661 | 0.2586407 | 0.039 | 2.1087762 | -3.859867 | 0.56392767 |
| SRP68 | NM_014230 | 0.0497502 | 0.00119 | 3.3405478 | -1.050643 | 0.56310288 |
| NDUFB7 | NM_004146 | 0.1482563 | 0.0122 | 2.5554766 | -3.059688 | 0.56273279 |
| MIPEP | NM_005932 | 0.180387 | 0.0179 | 2.4300884 | -3.219795 | 0.56151359 |
| C1orf112 | NM_018186 | 0.1302889 | 0.00919 | 2.6724599 | -2.7375 | 0.56072346 |
| ACO1 | M58510 | 0.0944724 | 0.00485 | 2.8857038 | -2.274077 | 0.56035779 |
| ANKRD36 | AB046861 | 0.2186316 | 0.0271 | 2.2451274 | -3.730412 | 0.55907613 |
| APPL1 | NM_012096 | 0.0700937 | 0.00266 | 3.0935468 | -1.738071 | 0.5586006 |
| TSPAN6 | NM_003270 | 0.0473321 | 0.00112 | 3.3772806 | -0.930066 | 0.55818059 |
| ENO2 | NM_001975 | 0.1734173 | 0.0165 | 2.4411429 | -3.337504 | 0.5573626 |
| DENR | AF038554 | 0.1352168 | 0.00996 | 2.6310917 | -2.874953 | 0.55694379 |
| ALDH9A1 | NM_000696 | 0.054029 | 0.00138 | 3.3018255 | -1.152719 | 0.55693676 |
| PRPF40B | AL137459 | 0.2464837 | 0.0351 | 2.1373272 | -3.972892 | 0.55645427 |
| PDIA5 | NM_006810 | 0.054419 | 0.0015 | 3.275157 | -1.232156 | 0.55628126 |
| RCN2 | NM_002902 | 0.1537559 | 0.0131 | 2.5300547 | -3.127 | 0.55594546 |
| DAD1 | NM_001344 | 0.1259171 | 0.00846 | 2.6897957 | -2.756276 | 0.55522394 |
| WISP2 | NM_003881 | 0.0381459 | 0.00074 | 3.4984082 | -0.596126 | 0.55518361 |
| PDLIM1 | U90878 | 0.0958373 | 0.005 | 2.8757944 | -2.299139 | 0.55470683 |
| CRYGA | NM_014617 | 0.1099701 | 0.00652 | 2.7830149 | -2.521178 | 0.55401947 |
| BABAM1 | NM_014173 | 0.0836864 | 0.00378 | 2.9691552 | -2.050618 | 0.55355077 |
| NDUFA1 | NM_004541 | 0.0722098 | 0.00285 | 3.0616942 | -1.821862 | 0.55302638 |
| SLC1A4 | NM_003038 | 0.1943126 | 0.0211 | 2.3694741 | -3.370389 | 0.55294238 |
| TPX2 | AB024704 | 0.0886388 | 0.00431 | 2.9288766 | -2.165448 | 0.55228724 |
| SKP1 | D17032 | 0.0654211 | 0.00224 | 3.1426532 | -1.591082 | 0.55164632 |
| CPNE3 | NM_003909 | 0.0567509 | 0.00162 | 3.2457163 | -1.314937 | 0.5510102 |
| EXOSC7 | D29958 | 0.054419 | 0.00148 | 3.277424 | -1.222484 | 0.55050853 |
| POMP | NM_015932 | 0.1302889 | 0.00918 | 2.6620494 | -2.812294 | 0.5482657 |
| GHR | Z11849 | 0.0572249 | 0.00166 | 3.244225 | -1.319697 | 0.54804302 |
| SELT | NM_016275 | 0.0585008 | 0.00175 | 3.2220971 | -1.373398 | 0.54790699 |
| BMP7 | NM_001719 | 0.0323271 | 0.00055 | 3.5834 | -0.341987 | 0.54789494 |
| GP1BA | NM_000173 | 0.118422 | 0.00761 | 2.7768177 | -2.457192 | 0.54730539 |
| LOC642533 | AF086148 | 0.1432404 | 0.0113 | 2.5839905 | -3.014773 | 0.54620561 |
| CKAP5 | D43948 | 0.0757208 | 0.00311 | 3.034503 | -1.88778 | 0.54590343 |
| ANAPC5 | NM_016237 | 0.0363239 | 0.000681 | 3.5348982 | -0.498501 | 0.54545988 |
| ANAPC11 | NM_016476 | 0.0894193 | 0.00439 | 2.9172974 | -2.192048 | 0.54541437 |
| CTNNAL1 | NM_003798 | 0.1253368 | 0.00841 | 2.6930041 | -2.728997 | 0.54442762 |
| ZNHIT3 | L40410 | 0.0833651 | 0.00375 | 2.9724321 | -2.051136 | 0.5442882 |
| RPN2 | NM_002951 | 0.2576088 | 0.0385 | 2.0998224 | -4.016764 | 0.54403625 |
| MGST3 | NM_004528 | 0.1626794 | 0.0144 | 2.4907806 | -3.228273 | 0.54386042 |
| SLMAP | NM_007159 | 0.0620976 | 0.002 | 3.1792798 | -1.491259 | 0.54357894 |
| APOB | NM_000384 | 0.1335036 | 0.00964 | 2.6424337 | -2.868623 | 0.54331399 |
| UCHL3 | NM_006002 | 0.0719307 | 0.00282 | 3.0675782 | -1.808319 | 0.5429296 |
| C1D | NM_006333 | 0.077671 | 0.00329 | 3.0162291 | -1.92849 | 0.54255562 |
| DCK | NM_000788 | 0.0362556 | 0.000676 | 3.5338254 | -0.503727 | 0.54183818 |
| NUCB2 | NM_005013 | 0.1957791 | 0.0215 | 2.3406865 | -3.506237 | 0.54053303 |
| CCDC88A | NM_018084 | 0.1341506 | 0.00978 | 2.6383632 | -2.847109 | 0.54049417 |
| UQCR11 | NM_006830 | 0.0360761 | 0.000668 | 3.5173545 | -0.534052 | 0.54029823 |
| PFN2 | NM_002628 | 0.1013524 | 0.00559 | 2.8359944 | -2.398863 | 0.53917168 |
| RPS19BP1 | AK025423 | 0.0832389 | 0.00373 | 2.9753764 | -2.05194 | 0.53882927 |
| ETFA | NM_000126 | 0.2257704 | 0.0291 | 2.2143364 | -3.818533 | 0.53846792 |
| TTYH1 | AF177909 | 0.1673799 | 0.0154 | 2.4952858 | -3.072984 | 0.53832522 |
| HCFC1R1 | NM_017885 | 0.0144552 | 0.000128 | 4.0124925 | 0.97501 | 0.53768383 |
| PRMT1 | NM_001536 | 0.1137923 | 0.007 | 2.758114 | -2.572291 | 0.53692448 |
| ATL3 | AL117600 | 0.0199832 | 0.000232 | 3.837053 | 0.433552 | 0.53519607 |
| HSPA5 | AF216292 | 0.1943221 | 0.0212 | 2.3427113 | -3.540846 | 0.53508478 |
| MRPL52 | AK000450 | 0.1875708 | 0.0196 | 2.3751137 | -3.4464 | 0.53507977 |
| WBSCR22 | NM_017528 | 0.0738979 | 0.00298 | 3.0532176 | -1.826486 | 0.53395624 |
| CEBPZOS | AK001814 | 0.0175438 | 0.000181 | 3.8977972 | 0.64062 | 0.53367314 |
| PPM1G | NM_002707 | 0.038767 | 0.000764 | 3.491467 | -0.627356 | 0.53340462 |
| EFCAB14 | NM_014774 | 0.0170066 | 0.000172 | 3.939347 | 0.714094 | 0.53314983 |
| KCNJ12 | AK024229 | 0.0845864 | 0.00384 | 2.9704297 | -2.035875 | 0.53286975 |
| ACBD3 | AK025520 | 0.0360761 | 0.000664 | 3.5227422 | -0.513992 | 0.53279603 |
| RPS27 | NM_001030 | 0.2244411 | 0.0288 | 2.2185796 | -3.798993 | 0.53276244 |
| NLRC4 | AL389934 | 0.2809458 | 0.0456 | 2.0266148 | -4.16784 | 0.53243176 |
| TMOD2 | AK025346 | 0.1784419 | 0.0175 | 2.4181105 | -3.389376 | 0.5320667 |
| PGRMC2 | NM_006320 | 0.0924658 | 0.00465 | 2.9113473 | -2.162934 | 0.53080578 |
| KDELR2 | NM_006854 | 0.1677163 | 0.0154 | 2.4710641 | -3.246856 | 0.53052047 |
| NOP58 | NM_015934 | 0.1848142 | 0.019 | 2.3887066 | -3.428498 | 0.53035688 |
| RPS21 | NM_001024 | 0.2287293 | 0.0299 | 2.2032237 | -3.82997 | 0.53028701 |
| NUDT21 | NM_007006 | 0.1979702 | 0.0221 | 2.3281691 | -3.557814 | 0.52980071 |
| HINFP | AL080201 | 0.0419922 | 0.000886 | 3.436429 | -0.775338 | 0.52969527 |
| UQCRC1 | NM_003365 | 0.1870449 | 0.0196 | 2.3751472 | -3.470236 | 0.5287913 |
| NREP | NM_004772 | 0.0674912 | 0.00244 | 3.1208454 | -1.649343 | 0.52849154 |
| TMEM254-AS1 | AK021673 | 0.1897973 | 0.0202 | 2.3631513 | -3.483142 | 0.52838599 |
| CPSF3 | NM_016207 | 0.0454418 | 0.00102 | 3.3952322 | -0.890453 | 0.52801619 |
| RAB20 | NM_017817 | 0.1369265 | 0.0103 | 2.619293 | -2.931141 | 0.52775841 |
| RAG2 | AF080577 | 0.1041076 | 0.00591 | 2.8508958 | -2.274395 | 0.52768614 |
| DUSP3 | AL049417 | 0.1302889 | 0.00917 | 2.6644522 | -2.796578 | 0.52762537 |
| CLINT1 | NM_014666 | 0.1128359 | 0.00687 | 2.7646371 | -2.575346 | 0.5265465 |
| PCDH9 | NM_020403 | 0.0326659 | 0.00056 | 3.5909132 | -0.328372 | 0.52614681 |
| FMC1 | AF161386 | 0.0669542 | 0.0024 | 3.1223915 | -1.648099 | 0.52460431 |
| PAK4 | NM_005884 | 0.0061444 | 1.91E-05 | 4.5835122 | 2.671135 | 0.52433751 |
| BBS7 | NM_018190 | 0.257798 | 0.0387 | 2.0962587 | -4.049498 | 0.52402087 |
| AP2B1 | NM_001282 | 0.044787 | 0.000998 | 3.4077997 | -0.859969 | 0.52385664 |
| DDB1 | NM_001923 | 0.0992823 | 0.00534 | 2.8595895 | -2.307134 | 0.52287141 |
| TAF6 | NM_005641 | 0.1517484 | 0.0129 | 2.5401807 | -3.092608 | 0.52252292 |
| ARL4C | NM_005737 | 0.1697769 | 0.0158 | 2.4567906 | -3.293464 | 0.52236323 |
| NAE1 | NM_003905 | 0.1153155 | 0.0072 | 2.7509721 | -2.61709 | 0.52199636 |
| ADCY1 | L05500 | 0.0497502 | 0.00119 | 3.3410616 | -1.050781 | 0.52197068 |
| ADAM29 | NM_014269 | 0.2267949 | 0.0294 | 2.2127139 | -3.80539 | 0.52136285 |
| PLP2 | NM_002668 | 0.1405632 | 0.0109 | 2.5954661 | -2.978936 | 0.52112798 |
| GRPEL1 | AF070525 | 0.0829795 | 0.0037 | 2.9785019 | -2.028343 | 0.52018953 |
| CLMP | AK026068 | 0.0555997 | 0.00158 | 3.2521752 | -1.299776 | 0.5201243 |
| EBP | NM_006579 | 0.1882468 | 0.0198 | 2.3712863 | -3.455644 | 0.51981683 |
| DHX9 | NM_001357 | 0.1719792 | 0.0163 | 2.4482633 | -3.310046 | 0.51844247 |
| OR7E47P | X87825 | 0.0484521 | 0.00116 | 3.3602804 | -0.986177 | 0.51801332 |
| CTSL | NM_001912 | 0.1298985 | 0.00909 | 2.6683018 | -2.797121 | 0.51768735 |
| CCDC149 | AK023472 | 0.0391908 | 0.000792 | 3.4709394 | -0.668863 | 0.51600033 |
| RPL21 | NM_000982 | 0.2510003 | 0.0366 | 2.1191245 | -3.996174 | 0.51592608 |
| UBE2K | NM_005339 | 0.0685225 | 0.00254 | 3.101239 | -1.701837 | 0.51573073 |
| POLK | NM_016218 | 0.0894193 | 0.00438 | 2.9252316 | -2.150166 | 0.51545526 |
| GMNN | NM_015895 | 0.0939476 | 0.0048 | 2.8897608 | -2.263796 | 0.51528286 |
| COL6A2 | M20777 | 0.1819598 | 0.0182 | 2.403593 | -3.385814 | 0.51491034 |
| SNRNP25 | AK026593 | 0.0521099 | 0.00128 | 3.3304055 | -1.080988 | 0.5145379 |
| GALNT1 | NM_020474 | 0.0812173 | 0.00349 | 2.9946906 | -1.991904 | 0.51345669 |
| HK2 | AF086198 | 0.1882468 | 0.0198 | 2.3702816 | -3.491636 | 0.51312281 |
| PSMA2 | NM_002787 | 0.2364436 | 0.0321 | 2.1746065 | -3.899223 | 0.51260443 |
| RDH11 | NM_016026 | 0.1776438 | 0.0174 | 2.4202653 | -3.374367 | 0.51179183 |
| SPAG7 | NM_004890 | 0.0924658 | 0.00462 | 2.9028163 | -2.222017 | 0.5109415 |
| EHBP1 | AB020710 | 0.1331129 | 0.00956 | 2.6462327 | -2.849769 | 0.51050444 |
| TMX1 | AL080080 | 0.054419 | 0.00145 | 3.2840903 | -1.191468 | 0.51048067 |
| BEX3 | NM_014380 | 0.2635994 | 0.0402 | 2.079946 | -4.071599 | 0.51024866 |
| GRIK1 | NM_000830 | 0.1382055 | 0.0106 | 2.6116282 | -2.939608 | 0.51012479 |
| NCAPG2 | NM_017760 | 0.2558741 | 0.038 | 2.1056836 | -3.991328 | 0.51010652 |
| CLASP2 | AB014527 | 0.2927986 | 0.0494 | 2.0108388 | -3.972044 | 0.50989724 |
| MRPS35 | AF182422 | 0.0657251 | 0.00226 | 3.143029 | -1.593577 | 0.50909554 |
| UQCC2 | AF086234 | 0.1614049 | 0.0142 | 2.4979596 | -3.211599 | 0.50871396 |
| COPS6 | NM_006833 | 0.1013524 | 0.0056 | 2.836281 | -2.398363 | 0.50869478 |
| CAB39 | NM_016289 | 0.0868362 | 0.00409 | 2.9421681 | -2.120351 | 0.50863173 |
| SRSF1 | NM_006924 | 0.1839197 | 0.0186 | 2.3961222 | -3.413147 | 0.50861063 |
| SGSH | NM_000199 | 0.0244857 | 0.000327 | 3.730577 | 0.11814 | 0.5084468 |
| ATP6AP2 | NM_005765 | 0.1897973 | 0.0203 | 2.3599046 | -3.516107 | 0.50840759 |
| ALCAM | NM_001627 | 0.1222957 | 0.00807 | 2.7072664 | -2.714325 | 0.50789481 |
| VEGFC | NM_005429 | 0.0302775 | 0.000459 | 3.6371214 | -0.185515 | 0.507636 |
| ATP13A3 | AK024639 | 0.0503178 | 0.00121 | 3.3417047 | -1.051422 | 0.50726791 |
| SSRP1 | NM_003146 | 0.0757024 | 0.0031 | 3.0385144 | -1.872376 | 0.50699232 |
| NUCB1 | NM_006184 | 0.136426 | 0.0102 | 2.6196827 | -2.912262 | 0.50662815 |
| ARL6IP4 | NM_016638 | 0.0768832 | 0.00324 | 3.0196565 | -1.93423 | 0.50641468 |
| MCOLN3 | NM_018298 | 0.2606622 | 0.0394 | 2.1117523 | -3.734007 | 0.50594812 |
| PAM16 | NM_016069 | 0.1185939 | 0.00766 | 2.7272598 | -2.656895 | 0.50568272 |
| COX4I1 | D17129 | 0.2464837 | 0.035 | 2.1387327 | -3.968112 | 0.50514723 |
| MCM4 | X74794 | 0.0260319 | 0.000364 | 3.7069938 | 0.027905 | 0.50511446 |
| TBKBP1 | NM_014726 | 0.0945578 | 0.00488 | 2.8855199 | -2.257743 | 0.50495263 |
| NELFE | NM_002904 | 0.0879057 | 0.00421 | 2.9374063 | -2.136014 | 0.50462797 |
| C12orf65 | AK025908 | 0.0244156 | 0.000319 | 3.754384 | 0.163148 | 0.50439653 |
| TXN | NM_003329 | 0.0894193 | 0.00439 | 2.9171904 | -2.192322 | 0.50434258 |
| ELL2 | NM_012081 | 0.0291837 | 0.000438 | 3.6452147 | -0.14867 | 0.50432403 |
| RBM26 | AF273052 | 0.0815565 | 0.00356 | 2.9981292 | -1.949009 | 0.50350229 |
| DCUN1D4 | D87466 | 0.0393257 | 0.000797 | 3.4921295 | -0.627655 | 0.50315449 |
| KLHL35 | AL050370 | 0.2287293 | 0.03 | 2.2031428 | -3.841128 | 0.50288909 |
| TYMS | NM_001071 | 0.2107166 | 0.025 | 2.2777041 | -3.687827 | 0.50250263 |
| TIMM44 | NM_006351 | 0.0169041 | 0.000169 | 3.9213874 | 0.70688 | 0.50233356 |
| SRF | NM_003131 | 0.054206 | 0.0014 | 3.3026385 | -1.15639 | 0.50231395 |
| RPL35A | AK021571 | 0.2556137 | 0.0379 | 2.1083522 | -3.969663 | 0.50185757 |
| NARS2 | AK027094 | 0.0146238 | 0.00013 | 3.9991743 | 0.952611 | 0.50177734 |
| PLA2G2E | NM_014589 | 0.053044 | 0.00134 | 3.3134082 | -1.117663 | 0.50158671 |
| SLC30A7 | AK023089 | 0.0677732 | 0.00246 | 3.1160489 | -1.674783 | 0.50107726 |
| MST1R | NM_002447 | 0.1949713 | 0.0213 | 2.3421706 | -3.549166 | 0.50079223 |
| CRBN | NM_016302 | 0.0521155 | 0.00129 | 3.3271154 | -1.068261 | 0.500725 |
| PSMD6 | NM_014814 | 0.054419 | 0.00149 | 3.2815677 | -1.219592 | 0.50069061 |
| FGF2 | NM_002006 | 0.033557 | 0.000592 | 3.5628761 | -0.416063 | 0.50010601 |
| PSMG2 | NM_020232 | 0.1410211 | 0.011 | 2.5918125 | -2.997393 | 0.50009974 |
| RUVBL2 | NM_006666 | 0.0823866 | 0.00363 | 2.9845095 | -2.012063 | 0.50008945 |
| BCAS3 | NM_017679 | 0.111606 | 0.00671 | 2.7823701 | -2.507368 | 0.49984943 |
| SSR1 | NM_003144 | 0.065773 | 0.00228 | 3.1365157 | -1.606611 | 0.49970817 |
| MIS18BP1 | NM_018353 | 0.0298582 | 0.000451 | 3.6354179 | -0.170476 | 0.49967286 |
| SLC16A3 | NM_004207 | 0.1788119 | 0.0176 | 2.4156003 | -3.395512 | 0.49950407 |
| ATIC | NM_004044 | 0.2181298 | 0.0269 | 2.2474099 | -3.749355 | 0.49939102 |
| SMARCC2 | NM_003075 | 0.0989712 | 0.00529 | 2.8618923 | -2.319426 | 0.49938647 |
| SUMO3 | NM_006936 | 0.1132586 | 0.00694 | 2.7761336 | -2.49652 | 0.49922065 |
| ARID2 | AB046777 | 0.0198493 | 0.000229 | 3.8492869 | 0.452971 | 0.49909555 |
| SMAD5 | AK001036 | 0.136426 | 0.0102 | 2.6207486 | -2.909434 | 0.49859788 |
| UFL1 | AB018319 | 0.0323271 | 0.000551 | 3.5875619 | -0.334024 | 0.49831976 |
| ZBED5 | AF205600 | 0.2227307 | 0.0284 | 2.2448418 | -3.514405 | 0.49790601 |
| CBR1 | NM_001757 | 0.0640443 | 0.00214 | 3.1641952 | -1.541504 | 0.49782945 |
| PDCD2 | NM_002598 | 0.0939476 | 0.00476 | 2.892744 | -2.256228 | 0.49782217 |
| CITED2 | NM_006079 | 0.2145555 | 0.0258 | 2.2644777 | -3.702828 | 0.49773004 |
| CXCL14 | NM_004887 | 0.0539101 | 0.00138 | 3.3025782 | -1.148712 | 0.4974857 |
| HIST1H3C | NM_003531 | 0.0829795 | 0.00371 | 2.977207 | -2.022342 | 0.4968641 |
| RIN3 | AK021762 | 0.1875708 | 0.0196 | 2.3764642 | -3.441795 | 0.49663509 |
| RHOD | NM_014578 | 0.2429637 | 0.0338 | 2.1567729 | -3.889623 | 0.49654293 |
| URI1 | NM_003796 | 0.0373686 | 0.000711 | 3.5033115 | -0.579515 | 0.49569016 |
| PELP1 | U88153 | 0.2311159 | 0.0305 | 2.2091374 | -3.658306 | 0.49555783 |
| NDUFAF4 | NM_014165 | 0.0571642 | 0.00164 | 3.2505936 | -1.298662 | 0.4949035 |
| PSMD7 | NM_002811 | 0.1624289 | 0.0144 | 2.4962065 | -3.182297 | 0.49390459 |
| CENPU | AK027121 | 0.1289463 | 0.00886 | 2.6754768 | -2.760189 | 0.49340319 |
| ABCC2 | NM_000392 | 0.1242184 | 0.0083 | 2.6959886 | -2.741559 | 0.49319187 |
| GBAS | NM_001483 | 0.1490718 | 0.0123 | 2.5523063 | -3.07689 | 0.4930318 |
| CEBPZ | NM_005760 | 0.1419554 | 0.0111 | 2.58883 | -3.003561 | 0.49245333 |
| BBX | NM_020235 | 0.1184174 | 0.00758 | 2.729559 | -2.651259 | 0.4913858 |
| PTGDR | AK026202 | 0.2108916 | 0.025 | 2.29797 | -3.433973 | 0.49137063 |
| MRPS30 | NM_016640 | 0.118422 | 0.00761 | 2.7284108 | -2.654029 | 0.49129571 |
| BZW2 | NM_014038 | 0.0683784 | 0.00253 | 3.1027207 | -1.705711 | 0.49117275 |
| TMED4 | D17216 | 0.0982563 | 0.00521 | 2.8643343 | -2.311876 | 0.49080126 |
| DONSON | NM_017613 | 0.0412635 | 0.000859 | 3.465434 | -0.710493 | 0.49061087 |
| MRM2 | NM_013393 | 0.0886015 | 0.00429 | 2.9294406 | -2.146455 | 0.4904509 |
| CDK1 | NM_001786 | 0.2396502 | 0.0329 | 2.165451 | -3.902551 | 0.49036972 |
| EEFSEC | AF268872 | 0.0924658 | 0.00462 | 2.9189187 | -2.159199 | 0.49030573 |
| NRBP2 | AL137662 | 0.1762363 | 0.0172 | 2.4389828 | -3.242048 | 0.48993856 |
| ITPR3 | NM_002224 | 0.1064568 | 0.00612 | 2.8067485 | -2.44402 | 0.48942158 |
| EPRS | NM_004446 | 0.1434215 | 0.0113 | 2.5836886 | -3.004922 | 0.48819544 |
| CTAG1B | NM_001327 | 0.1991527 | 0.0224 | 2.321994 | -3.570779 | 0.4881136 |
| LOC100288911 | AF075027 | 0.2579868 | 0.0389 | 2.1210499 | -3.697316 | 0.48767565 |
| FBXO25 | AF174605 | 0.0677732 | 0.00246 | 3.1158511 | -1.675315 | 0.48713557 |
| SLCO4A1 | NM_016354 | 0.2538409 | 0.0374 | 2.126763 | -3.826816 | 0.48699144 |
| RFXANK | NM_003721 | 0.0444226 | 0.000988 | 3.4201578 | -0.824793 | 0.48697294 |
| PSMD14 | NM_005805 | 0.2218047 | 0.028 | 2.2321703 | -3.769738 | 0.48674765 |
| BET1 | NM_005868 | 0.0521629 | 0.00131 | 3.3182008 | -1.106834 | 0.48663363 |
| SLC16A10 | AF116652 | 0.1297693 | 0.00902 | 2.6678132 | -2.798775 | 0.48654029 |
| AGGF1 | NM_018046 | 0.1934196 | 0.0208 | 2.3574516 | -3.465574 | 0.48593739 |
| 15-Sep | NM_004261 | 0.0663274 | 0.00233 | 3.1292881 | -1.634931 | 0.48588829 |
| NOX3 | AF190122 | 0.2139928 | 0.0257 | 2.2722606 | -3.630001 | 0.48456979 |
| GCSAML-AS1 | AK002105 | 0.278065 | 0.0446 | 2.0565211 | -3.873469 | 0.48420635 |
| ADH5 | NM_000671 | 0.0186103 | 0.000203 | 3.873399 | 0.555183 | 0.48369026 |
| TBCB | NM_001281 | 0.0182969 | 0.000192 | 3.8971323 | 0.604505 | 0.48351209 |
| COLGALT1 | AK025982 | 0.0697057 | 0.00263 | 3.0929531 | -1.728098 | 0.48317673 |
| UBA52 | NM_003333 | 0.1468345 | 0.012 | 2.5620429 | -3.045559 | 0.48298242 |
| ABCA5 | AJ275973 | 0.1236078 | 0.0082 | 2.7003273 | -2.740279 | 0.48275342 |
| BLVRA | NM_000712 | 0.0819837 | 0.0036 | 2.9847227 | -2.026061 | 0.48176127 |
| PSMC6 | NM_002806 | 0.1819598 | 0.0182 | 2.4009238 | -3.427532 | 0.48166744 |
| FKBP3 | NM_002013 | 0.1943126 | 0.0212 | 2.3499773 | -3.493687 | 0.48141399 |
| SAYSD1 | NM_018322 | 0.1578715 | 0.0138 | 2.5144154 | -3.129346 | 0.48029154 |
| ECT2 | NM_018098 | 0.1402432 | 0.0109 | 2.6010586 | -2.944168 | 0.48023788 |
| ATRNL1 | AB011106 | 0.0741176 | 0.00299 | 3.0485356 | -1.844198 | 0.48020946 |
| GSTK1 | NM_015917 | 0.0939476 | 0.00478 | 2.8887231 | -2.265398 | 0.48004195 |
| ZFYVE1 | AF251025 | 0.1144311 | 0.00709 | 2.7594875 | -2.570613 | 0.47960629 |
| DHX15 | NM_001358 | 0.2558899 | 0.0381 | 2.106127 | -3.987516 | 0.4788178 |
| DNAJC4 | NM_005528 | 0.0894193 | 0.00439 | 2.9343945 | -2.143365 | 0.47872112 |
| CHD1L | NM_004284 | 0.1450954 | 0.0116 | 2.5737265 | -3.028563 | 0.47858581 |
| LOC100131508 | AF130064 | 0.2842376 | 0.0469 | 2.0129033 | -4.208093 | 0.47800839 |
| B4GAT1 | NM_006876 | 0.0545484 | 0.00152 | 3.2715643 | -1.243872 | 0.47725474 |
| CWC22 | AB046824 | 0.0706847 | 0.0027 | 3.0826608 | -1.753551 | 0.47698343 |
| LEFTY2 | NM_003240 | 0.1468345 | 0.012 | 2.5675452 | -3.009511 | 0.47697156 |
| TST | NM_003312 | 0.0618873 | 0.00197 | 3.1858795 | -1.475771 | 0.47681477 |
| FBXL17 | AL133602 | 0.2062857 | 0.0239 | 2.3066692 | -3.554726 | 0.47676001 |
| NDUFV1 | NM_007103 | 0.0870407 | 0.00414 | 2.9411049 | -2.124784 | 0.47578513 |
| PTTG1 | NM_004219 | 0.2856754 | 0.0474 | 2.0108768 | -4.165949 | 0.47555894 |
| COL9A3 | NM_001853 | 0.1149659 | 0.00717 | 2.7495701 | -2.612083 | 0.47539221 |
| MNAT1 | NM_002431 | 0.0712292 | 0.00276 | 3.0741849 | -1.78227 | 0.4743086 |
| SORCS3 | AB028982 | 0.2924442 | 0.0493 | 2.0092214 | -3.931573 | 0.47426314 |
| DDRGK1 | AF086090 | 0.2227424 | 0.0284 | 2.2316316 | -3.746204 | 0.47329066 |
| MRPL15 | NM_014175 | 0.1461041 | 0.0118 | 2.5719697 | -3.020774 | 0.4724877 |
| PTTG2 | AF095288 | 0.2789904 | 0.045 | 2.0328719 | -4.153839 | 0.47170049 |
| CPSF4 | NM_006693 | 0.2479036 | 0.0355 | 2.1338538 | -3.950084 | 0.47110048 |
| PSMD9 | NM_002813 | 0.0510337 | 0.00124 | 3.3314703 | -1.073372 | 0.47053554 |
| AHCY | NM_000687 | 0.1253368 | 0.00841 | 2.6972342 | -2.719273 | 0.46982911 |
| CMAS | NM_018686 | 0.2396502 | 0.0329 | 2.1656061 | -3.890691 | 0.4692886 |
| SUFU | NM_016169 | 0.2472731 | 0.0353 | 2.1454552 | -3.845304 | 0.4687114 |
| LYAR | NM_017816 | 0.044233 | 0.000981 | 3.4117605 | -0.852055 | 0.46861478 |
| CCT8 | NM_006585 | 0.1096833 | 0.00648 | 2.7880585 | -2.500286 | 0.46805346 |
| RABGGTB | NM_004582 | 0.2135865 | 0.0256 | 2.2682938 | -3.694972 | 0.46788877 |
| DCTN6 | NM_006571 | 0.0866583 | 0.00403 | 2.9472055 | -2.107375 | 0.46767636 |
| SLC9A5 | AK021876 | 0.098928 | 0.00527 | 2.8560307 | -2.356911 | 0.46732174 |
| IFFO2 | AK024480 | 0.0869648 | 0.00411 | 2.9532126 | -2.058479 | 0.46706355 |
| UQCRFS1 | NM_006003 | 0.2117172 | 0.0252 | 2.2740435 | -3.694528 | 0.46652685 |
| TPST2 | NM_003595 | 0.2777453 | 0.0445 | 2.0411027 | -4.091827 | 0.46645327 |
| MYBBP1A | NM_014520 | 0.0389174 | 0.000772 | 3.4967649 | -0.612663 | 0.46643723 |
| FEM1B | NM_015322 | 0.0231308 | 0.000284 | 3.768153 | 0.233026 | 0.46628758 |
| PMM1 | NM_002676 | 0.0762261 | 0.00316 | 3.0351207 | -1.891542 | 0.46622276 |
| HBS1L | NM_006620 | 0.1101431 | 0.00654 | 2.7847045 | -2.498692 | 0.46569024 |
| CNPY2 | NM_014255 | 0.1739053 | 0.0167 | 2.4377685 | -3.312046 | 0.465256 |
| SLC11A2 | AB004857 | 0.1455237 | 0.0117 | 2.592217 | -2.887617 | 0.46513929 |
| PPIP5K2 | NM_015216 | 0.1064568 | 0.00613 | 2.8078838 | -2.442058 | 0.46498665 |
| ATP5E | NM_006886 | 0.0776724 | 0.0033 | 3.0125833 | -1.951759 | 0.46481887 |
| SMO | NM_005631 | 0.01599 | 0.000158 | 3.9472559 | 0.783704 | 0.46449656 |
| IL10RB | NM_000628 | 0.2153734 | 0.026 | 2.2601454 | -3.725851 | 0.46444007 |
| NOC3L | AK022882 | 0.047064 | 0.0011 | 3.3722674 | -0.960363 | 0.46361396 |
| MRPL51 | NM_016497 | 0.2702815 | 0.0421 | 2.061928 | -4.086663 | 0.46315932 |
| UXT | NM_004182 | 0.1949713 | 0.0213 | 2.340424 | -3.556173 | 0.46303183 |
| NAA60 | AK024216 | 0.0660645 | 0.00231 | 3.1334599 | -1.617113 | 0.46286469 |
| MAP3K6 | NM_004672 | 0.0696501 | 0.00262 | 3.1056047 | -1.691992 | 0.46227227 |
| PNO1 | NM_020143 | 0.0063444 | 2.31E-05 | 4.473651 | 2.510898 | 0.46187885 |
| HLA-DPA1 | M27487 | 0.2418742 | 0.0335 | 2.1683619 | -3.785639 | 0.46117333 |
| CD63 | NM_001780 | 0.1989551 | 0.0223 | 2.3215444 | -3.586316 | 0.46055563 |
| EML2 | NM_012155 | 0.054419 | 0.00149 | 3.2740066 | -1.238833 | 0.46019172 |
| SRP14 | NM_003134 | 0.098963 | 0.00528 | 2.8531568 | -2.363633 | 0.45894206 |
| MLLT1 | AL365410 | 0.1660109 | 0.0151 | 2.4761928 | -3.238904 | 0.45888695 |
| UQCR10 | NM_013387 | 0.136007 | 0.0102 | 2.6214521 | -2.928458 | 0.45863311 |
| CIC | AB002304 | 0.0852702 | 0.00391 | 2.9584792 | -2.078869 | 0.45835071 |
| TK1 | NM_003258 | 0.0628051 | 0.00206 | 3.1766628 | -1.493146 | 0.4581526 |
| RPS6KB1 | NM_003161 | 0.065408 | 0.00224 | 3.1482011 | -1.589226 | 0.45804371 |
| GSTM3 | NM_000849 | 0.1013524 | 0.0056 | 2.8370527 | -2.378551 | 0.45760861 |
| CYB5B | AB009282 | 0.2464837 | 0.0347 | 2.1410865 | -3.965516 | 0.45752732 |
| ANP32D | NM_012404 | 0.0706847 | 0.0027 | 3.0830618 | -1.745401 | 0.45666159 |
| NXPH3 | AB032985 | 0.216106 | 0.0263 | 2.2877649 | -3.393659 | 0.45660226 |
| PRPF40A | NM_017892 | 0.1989551 | 0.0223 | 2.3253356 | -3.581939 | 0.45658995 |
| INPP5E | NM_019892 | 0.0719307 | 0.00283 | 3.0731138 | -1.784981 | 0.45595481 |
| GIPR | NM_000164 | 0.1839197 | 0.0186 | 2.3983101 | -3.383062 | 0.45573992 |
| IRF2BPL | AF075110 | 0.1646272 | 0.0148 | 2.4829555 | -3.222993 | 0.45529272 |
| NDUFV2 | M22538 | 0.2227424 | 0.0284 | 2.2254899 | -3.794992 | 0.4547392 |
| SLF1 | AL050298 | 0.0882579 | 0.00426 | 2.9386023 | -2.119521 | 0.45470549 |
| POLR3G | NM_006467 | 0.0947211 | 0.00491 | 2.884433 | -2.278324 | 0.45424714 |
| ALDH1L1 | NM_012190 | 0.2479036 | 0.0355 | 2.1469612 | -3.791428 | 0.4540649 |
| ATP6V1D | NM_015994 | 0.0842358 | 0.00382 | 2.9665533 | -2.066396 | 0.45327145 |
| ZC2HC1A | NM_016010 | 0.0819837 | 0.0036 | 2.9869723 | -2.00566 | 0.45286314 |
| NGDN | AK022215 | 0.1813691 | 0.0181 | 2.4099197 | -3.358159 | 0.45268235 |
| UTP6 | NM_018428 | 0.0386078 | 0.000758 | 3.4893838 | -0.608368 | 0.45264334 |
| HMGCS1 | NM_002130 | 0.0706847 | 0.0027 | 3.0851735 | -1.7574 | 0.45241938 |
| HUWE1 | NM_005703 | 0.1893475 | 0.0201 | 2.3674229 | -3.461625 | 0.45238971 |
| LTA4H | NM_000895 | 0.1497708 | 0.0125 | 2.5494474 | -3.073089 | 0.45221788 |
| EEA1 | NM_003566 | 0.0996693 | 0.00538 | 2.8506675 | -2.370912 | 0.45219549 |
| NUP37 | AK026271 | 0.1269501 | 0.00858 | 2.6845624 | -2.759146 | 0.45216245 |
| ISX | AK025181 | 0.1789884 | 0.0177 | 2.4584573 | -3.096529 | 0.45077451 |
| PRKX | NM_005044 | 0.0762261 | 0.00316 | 3.0404568 | -1.859521 | 0.44955385 |
| NUP62 | NM_016553 | 0.0235781 | 3.00E-04 | 3.7743786 | 0.212687 | 0.44930432 |
| TUBB3 | NM_006086 | 0.2321878 | 0.0308 | 2.1914866 | -3.863583 | 0.44892616 |
| MAP3K4 | NM_005922 | 0.1054485 | 0.00603 | 2.8240021 | -2.39035 | 0.44887416 |
| ZNF131 | U09410 | 0.054419 | 0.00145 | 3.2822418 | -1.21929 | 0.44806117 |
| FKBP4 | NM_002014 | 0.1158476 | 0.00726 | 2.7502153 | -2.583076 | 0.44797742 |
| UBA6 | NM_018227 | 0.054419 | 0.00143 | 3.2843517 | -1.211905 | 0.44760226 |
| NUP205 | D86978 | 0.1166984 | 0.00738 | 2.7384507 | -2.639099 | 0.44755627 |
| CALM1 | NM_006888 | 0.1670347 | 0.0153 | 2.4695316 | -3.265194 | 0.44747972 |
| MBL1P | AF019382 | 0.1399213 | 0.0108 | 2.6283149 | -2.777924 | 0.44747745 |
| AARS | NM_001605 | 0.2842707 | 0.047 | 2.0116644 | -4.211544 | 0.44746356 |
| RGS10 | AF045229 | 0.1314749 | 0.00937 | 2.6540787 | -2.821052 | 0.44705322 |
| CADM1 | NM_014333 | 0.052779 | 0.00133 | 3.313841 | -1.119068 | 0.44641987 |
| ELAC2 | NM_018127 | 0.1410211 | 0.011 | 2.5962765 | -2.955178 | 0.44589509 |
| KCNC1 | NM_004976 | 0.0473321 | 0.00112 | 3.36725 | -0.966198 | 0.44582147 |
| LSM6 | NM_007080 | 0.1694233 | 0.0157 | 2.4644111 | -3.239523 | 0.44578785 |
| SMYD5 | U50383 | 0.0886388 | 0.00431 | 2.9261699 | -2.170995 | 0.44553864 |
| HYAL4 | NM_012269 | 0.278402 | 0.0447 | 2.0490379 | -3.983924 | 0.44502756 |
| TIA1 | AF090093 | 0.1942796 | 0.021 | 2.3480709 | -3.525789 | 0.44487538 |
| PAK5 | AB040812 | 0.0972664 | 0.0051 | 2.8733815 | -2.298943 | 0.44463082 |
| CD44 | M59040 | 0.275319 | 0.0438 | 2.0431181 | -4.14133 | 0.44407689 |
| ASXL1 | AB023195 | 0.060664 | 0.00187 | 3.2092576 | -1.391507 | 0.44406133 |
| MDH2 | NM_005918 | 0.2498502 | 0.0362 | 2.1247792 | -3.983254 | 0.44388271 |
| EEF1A1 | NM_001402 | 0.1307831 | 0.00923 | 2.6699067 | -2.74296 | 0.44375507 |
| SF3B3 | D13642 | 0.1699197 | 0.0159 | 2.4588411 | -3.252439 | 0.4437501 |
| PCYT1A | NM_005017 | 0.0681429 | 0.0025 | 3.107243 | -1.702758 | 0.44312528 |
| EMC4 | NM_016454 | 0.2028811 | 0.0231 | 2.3101161 | -3.58341 | 0.44236198 |
| ACTL6A | NM_004301 | 0.2464837 | 0.035 | 2.1426285 | -3.916177 | 0.44220109 |
| WDR43 | D26488 | 0.0373686 | 0.000715 | 3.4991552 | -0.586991 | 0.44219437 |
| CCDC18-AS1 | AK021668 | 0.293878 | 0.0499 | 1.9864202 | -4.254461 | 0.4420197 |
| PCOLCE | NM_002593 | 0.1373219 | 0.0105 | 2.6141299 | -2.934392 | 0.44199296 |
| EML4 | NM_019063 | 0.1040257 | 0.0059 | 2.8159563 | -2.439219 | 0.43954313 |
| RGS3 | U27655 | 0.1645099 | 0.0147 | 2.4896396 | -3.183718 | 0.43952438 |
| WDR75 | AK022581 | 0.1223311 | 0.00808 | 2.7102508 | -2.688209 | 0.43942655 |
| ACOT13 | NM_018473 | 0.2139928 | 0.0257 | 2.267031 | -3.708101 | 0.43914469 |
| AKAP11 | NM_016248 | 0.1678083 | 0.0155 | 2.4759778 | -3.176273 | 0.4391392 |
| RHOBTB3 | NM_014899 | 0.1357279 | 0.0101 | 2.6266099 | -2.905778 | 0.43876818 |
| DNTT | NM_004088 | 0.1450954 | 0.0116 | 2.5753025 | -3.014059 | 0.43863975 |
| DYNC1H1 | AB002323 | 0.1827906 | 0.0184 | 2.4068847 | -3.337482 | 0.43859201 |
| GSDMD | AK022212 | 0.2364436 | 0.032 | 2.1754454 | -3.896594 | 0.43815026 |
| LOC101927253 | AK000454 | 0.2636624 | 0.0404 | 2.108318 | -3.699718 | 0.4380828 |
| ZNF22 | NM_006963 | 0.0516181 | 0.00127 | 3.3272135 | -1.084221 | 0.43791495 |
| RPA3 | NM_002947 | 0.2636624 | 0.0403 | 2.0855479 | -4.014436 | 0.43723001 |
| COX6B1 | NM_001863 | 0.1842801 | 0.0188 | 2.3910571 | -3.435993 | 0.4369303 |
| SLURP1 | NM_020427 | 0.1394995 | 0.0108 | 2.6049474 | -2.935203 | 0.43689866 |
| UBQLN1 | NM_013438 | 0.0386078 | 0.000756 | 3.4862199 | -0.626598 | 0.4362136 |
| OXA1L | NM_005015 | 0.2070505 | 0.0241 | 2.2957767 | -3.610334 | 0.4358438 |
| SNRNP40 | NM_004814 | 0.2702815 | 0.0421 | 2.0600548 | -4.120825 | 0.43578087 |
| IL12RB1 | NM_005535 | 0.2574197 | 0.0384 | 2.110247 | -3.895272 | 0.435727 |
| ITGB1BP1 | NM_004763 | 0.1391336 | 0.0107 | 2.6082123 | -2.927522 | 0.43539474 |
| DKC1 | NM_001363 | 0.251584 | 0.0369 | 2.1182351 | -3.991761 | 0.43486374 |
| IKBKE | NM_014002 | 0.0621876 | 0.00201 | 3.1839466 | -1.493427 | 0.43474298 |
| ORC5 | NM_002553 | 0.0572249 | 0.00165 | 3.2559617 | -1.289045 | 0.4346596 |
| RPL39 | NM_001000 | 0.2623379 | 0.0399 | 2.0839599 | -4.023017 | 0.43458739 |
| NUP107 | NM_020401 | 0.0375133 | 0.000722 | 3.5060301 | -0.579248 | 0.43453398 |
| PTTG3P | AF095289 | 0.2278065 | 0.0297 | 2.207959 | -3.827488 | 0.43444658 |
| OR8G2 | X89669 | 0.2815203 | 0.0457 | 2.0322072 | -4.043175 | 0.43427174 |
| CNR1 | NM_001840 | 0.0766741 | 0.00322 | 3.0399377 | -1.851559 | 0.43319038 |
| NENF | NM_013349 | 0.2382257 | 0.0325 | 2.169662 | -3.89517 | 0.43303261 |
| RNF219 | AK023511 | 0.0572249 | 0.00166 | 3.2485557 | -1.30108 | 0.43269552 |
| DHCR24 | AF261758 | 0.1789481 | 0.0176 | 2.4184675 | -3.35217 | 0.43241042 |
| PEAK1 | AK025943 | 0.1819598 | 0.0182 | 2.4039048 | -3.406199 | 0.43206512 |
| MUS81 | AK024665 | 0.0584456 | 0.00174 | 3.2305933 | -1.344736 | 0.43191234 |
| TMEM57 | NM_018202 | 0.1695325 | 0.0157 | 2.4589212 | -3.288219 | 0.43165698 |
| POLD3 | D26018 | 0.023401 | 0.000292 | 3.7788846 | 0.235629 | 0.43089804 |
| ARHGAP1 | NM_004308 | 0.1789481 | 0.0176 | 2.4208277 | -3.333763 | 0.43077921 |
| GPR180 | AK024059 | 0.0696501 | 0.00262 | 3.097926 | -1.71043 | 0.42979502 |
| ALLC | NM_018436 | 0.216106 | 0.0264 | 2.2565661 | -3.718215 | 0.42948727 |
| MTDH | AK000745 | 0.2606622 | 0.0394 | 2.0883566 | -4.06467 | 0.42903819 |
| DNTTIP2 | NM_014597 | 0.2922265 | 0.0493 | 1.9936873 | -4.222319 | 0.428883 |
| ARV1 | AL122047 | 0.0924658 | 0.00466 | 2.9024215 | -2.215652 | 0.42884741 |
| HS3ST3A1 | NM_006042 | 0.274267 | 0.0434 | 2.0519629 | -4.012559 | 0.42883683 |
| TSPAN31 | NM_005981 | 0.054419 | 0.00151 | 3.2958332 | -1.186138 | 0.42857034 |
| HIF3A | AK021737 | 0.0815565 | 0.00355 | 2.9980885 | -1.974139 | 0.42856758 |
| RBM6 | NM_005777 | 0.1103533 | 0.00658 | 2.7793672 | -2.547928 | 0.42838349 |
| CA14 | NM_012113 | 0.0799947 | 0.00342 | 3.0083721 | -1.94568 | 0.42768097 |
| KDM1A | AB011173 | 0.1314749 | 0.00934 | 2.6556792 | -2.836783 | 0.42765481 |
| TRAPPC12 | NM_016030 | 0.1559788 | 0.0135 | 2.5191072 | -3.152665 | 0.4269933 |
| LUC7L2 | NM_016019 | 0.101777 | 0.00568 | 2.830899 | -2.420128 | 0.42639186 |
| LENG9 | AF211976 | 0.0944724 | 0.00485 | 2.8993813 | -2.214292 | 0.42590515 |
| MLLT6 | S72604 | 0.1267468 | 0.00853 | 2.6868571 | -2.763296 | 0.4255726 |
| KRAS | NM_004985 | 0.203751 | 0.0234 | 2.3051172 | -3.60678 | 0.42538894 |
| EEF1B2 | S81522 | 0.233966 | 0.0314 | 2.1868327 | -3.855074 | 0.42522554 |
| MRPL40 | NM_003776 | 0.1270806 | 0.0086 | 2.683302 | -2.771905 | 0.42505654 |
| FTSJ3 | NM_017647 | 0.0939476 | 0.0048 | 2.8901859 | -2.254489 | 0.42493653 |
| RPL30 | NM_000989 | 0.1292596 | 0.00893 | 2.6726591 | -2.787144 | 0.42473909 |
| CCNB1 | M25753 | 0.2328881 | 0.0311 | 2.1874681 | -3.87354 | 0.42470885 |
| DCAF7 | NM_005828 | 0.1979702 | 0.0221 | 2.3345401 | -3.461307 | 0.42460077 |
| CENPK | AY009151 | 0.0643604 | 0.00218 | 3.1549595 | -1.553668 | 0.42438807 |
| COMT | NM_000754 | 0.2916847 | 0.0491 | 1.9929796 | -4.246045 | 0.42433362 |
| RTF1 | D87440 | 0.0829795 | 0.00371 | 2.9840893 | -2.011103 | 0.42414631 |
| IPO11 | NM_016338 | 0.0833651 | 0.00376 | 2.9781344 | -2.016122 | 0.42411616 |
| NOMO3///NOMO2 | D17082 | 0.127518 | 0.0087 | 2.6779238 | -2.784986 | 0.42385027 |
| CXXC1 | NM_014593 | 0.1391535 | 0.0107 | 2.6041011 | -2.958302 | 0.42340867 |
| IPO8 | NM_006390 | 0.0555843 | 0.00157 | 3.2558716 | -1.286588 | 0.42337442 |
| PDHX | NM_003477 | 0.0357683 | 0.000653 | 3.5379821 | -0.478323 | 0.42331295 |
| ABL1 | NM_005157 | 0.0845947 | 0.00385 | 2.9706939 | -2.044629 | 0.42325173 |
| AMMECR1 | AJ009817 | 0.2049276 | 0.0236 | 2.3106642 | -3.521841 | 0.42311925 |
| MESDC2 | D42039 | 0.0478854 | 0.00113 | 3.3640019 | -0.98388 | 0.42224017 |
| DDOST | NM_005216 | 0.0989871 | 0.00529 | 2.8613623 | -2.311639 | 0.42205311 |
| SIL1 | AJ299442 | 0.0861216 | 0.00397 | 2.969123 | -1.994491 | 0.42201072 |
| FARP1 | NM_005766 | 0.0801519 | 0.00344 | 3.0269882 | -1.872696 | 0.42200083 |
| ATXN1 | NM_000332 | 0.1505447 | 0.0126 | 2.5432364 | -3.077391 | 0.42181152 |
| RHD | NM_016124 | 0.1767397 | 0.0172 | 2.4255954 | -3.338151 | 0.42133364 |
| HOXD4 | X04706 | 0.2225464 | 0.0282 | 2.2294053 | -3.771708 | 0.42126359 |
| DUS1L | AF202637 | 0.0976212 | 0.00514 | 2.866669 | -2.29501 | 0.42108631 |
| PSMB4 | NM_002796 | 0.1646272 | 0.0148 | 2.4849713 | -3.206132 | 0.42104076 |
| HEMGN | AF130060 | 0.1461041 | 0.0118 | 2.568482 | -3.03053 | 0.4209751 |
| KIZ | NM_018474 | 0.0866784 | 0.00405 | 2.9503755 | -2.11034 | 0.42081006 |
| WDR33 | NM_018383 | 0.0886015 | 0.0043 | 2.9325174 | -2.141293 | 0.42069559 |
| ZBTB38 | AK025985 | 0.2010691 | 0.0228 | 2.3173446 | -3.567019 | 0.42052846 |
| MAP2K1 | NM_002755 | 0.1447684 | 0.0116 | 2.5787452 | -3.005584 | 0.41994217 |
| ECH1 | NM_001398 | 0.2070505 | 0.024 | 2.2935651 | -3.653094 | 0.41946347 |
| BAG2 | NM_004282 | 0.2825576 | 0.046 | 2.0242091 | -4.128438 | 0.41928617 |
| SCN2A | AL137498 | 0.192428 | 0.0207 | 2.359963 | -3.436697 | 0.41915859 |
| GLRX2 | NM_016066 | 0.0915925 | 0.00454 | 2.9107135 | -2.203431 | 0.41898141 |
| SPG7 | NM_003119 | 0.2278065 | 0.0297 | 2.2076252 | -3.817543 | 0.41883347 |
| SPCS1 | NM_014041 | 0.2778201 | 0.0445 | 2.0362801 | -4.163351 | 0.41878987 |
| TAL2 | NM_005421 | 0.2512732 | 0.0368 | 2.137401 | -3.710505 | 0.41863765 |
| MLEC | NM_014730 | 0.113181 | 0.00692 | 2.7657305 | -2.554865 | 0.41854348 |
| MYCBP | NM_012333 | 0.2186316 | 0.0273 | 2.2439782 | -3.742117 | 0.41839243 |
| NMRK1 | NM_017881 | 0.1212433 | 0.00793 | 2.7117098 | -2.703846 | 0.41823803 |
| MRPL4 | NM_015956 | 0.1803885 | 0.0179 | 2.4093551 | -3.396362 | 0.41815116 |
| TFPI | NM_006287 | 0.1674253 | 0.0154 | 2.4687052 | -3.265997 | 0.41809898 |
| CFAP97 | AB037851 | 0.1285813 | 0.00883 | 2.6798652 | -2.778648 | 0.41805379 |
| EFCC1 | AK022115 | 0.1456688 | 0.0117 | 2.6163036 | -2.790155 | 0.41803818 |
| LOX | NM_002317 | 0.1000418 | 0.00547 | 2.8558111 | -2.300604 | 0.41798588 |
| GNAS-AS1 | AJ251759 | 0.1369265 | 0.0103 | 2.6245127 | -2.88931 | 0.41767609 |
| CD8B | NM_004931 | 0.1943126 | 0.0211 | 2.3460827 | -3.540867 | 0.41732994 |
| COX8A | NM_004074 | 0.1568827 | 0.0136 | 2.512845 | -3.178025 | 0.41730639 |
| COPS2 | NM_004236 | 0.0829795 | 0.0037 | 2.9804137 | -2.032795 | 0.41720544 |
| TIGAR | NM_020375 | 0.1064568 | 0.00615 | 2.8048458 | -2.476763 | 0.41695435 |
| CDKN2C | NM_001262 | 0.1101729 | 0.00655 | 2.7813913 | -2.542945 | 0.41693571 |
| HKDC1 | AK026414 | 0.0615071 | 0.00194 | 3.1962175 | -1.45458 | 0.41612537 |
| MCM5 | NM_006739 | 0.1373219 | 0.0104 | 2.6130879 | -2.927462 | 0.41612034 |
| PON2 | NM_000305 | 0.0546804 | 0.00153 | 3.2713717 | -1.232444 | 0.41606803 |
| ERP29 | NM_006817 | 0.2382257 | 0.0325 | 2.1705512 | -3.891487 | 0.41455165 |
| HDAC6 | NM_006044 | 0.0663274 | 0.00233 | 3.1291801 | -1.635223 | 0.41448276 |
| H2AFZ | NM_002106 | 0.2411032 | 0.0333 | 2.1681864 | -3.773588 | 0.41448097 |
| EPCAM | NM_002354 | 0.1025138 | 0.00575 | 2.8334075 | -2.399064 | 0.41410112 |
| WBP4 | NM_007187 | 0.1863506 | 0.0193 | 2.3817163 | -3.444121 | 0.41401044 |
| NME4 | NM_005009 | 0.282682 | 0.0462 | 2.0207334 | -4.179906 | 0.41398267 |
| SERPINA1 | X05826 | 0.141683 | 0.0111 | 2.596219 | -2.954776 | 0.4139624 |
| MYO10 | AF234532 | 0.0186518 | 0.00021 | 3.8625133 | 0.515228 | 0.41383312 |
| GALK1 | NM_000154 | 0.1624289 | 0.0143 | 2.5006964 | -3.147592 | 0.41379789 |
| SLC35E2B | AB007916 | 0.1079621 | 0.00631 | 2.7941983 | -2.511348 | 0.41356529 |
| PCBP4 | NM_020418 | 0.1479027 | 0.0122 | 2.557076 | -3.066778 | 0.41355306 |
| GRINA | AL157442 | 0.2078456 | 0.0242 | 2.2888948 | -3.666235 | 0.41349567 |
| VAMP8 | NM_003761 | 0.1965399 | 0.0217 | 2.3343673 | -3.557086 | 0.41347803 |
| PSMB3 | NM_002795 | 0.0685241 | 0.00254 | 3.0990212 | -1.713878 | 0.41280502 |
| CDKAL1 | NM_017774 | 0.0465561 | 0.00108 | 3.3773524 | -0.943755 | 0.41247028 |
| IPO5 | NM_002271 | 0.2244411 | 0.0288 | 2.2207081 | -3.790246 | 0.41232998 |
| CD274 | NM_014143 | 0.216106 | 0.0263 | 2.2583302 | -3.702662 | 0.41215074 |
| ARMC10 | AF275808 | 0.1373219 | 0.0104 | 2.6215634 | -2.875339 | 0.41157142 |
| TMEM109 | AF131803 | 0.1957791 | 0.0215 | 2.3395751 | -3.544557 | 0.41155513 |
| ATRAID | NM_016085 | 0.2558741 | 0.038 | 2.1047897 | -4.019874 | 0.4115065 |
| ANKRD39 | NM_016466 | 0.1788119 | 0.0176 | 2.4222551 | -3.318451 | 0.41140041 |
| PHKA2 | NM_000292 | 0.1507633 | 0.0126 | 2.5481502 | -3.03017 | 0.41121718 |
| ACOT8 | NM_005469 | 0.148227 | 0.0122 | 2.557284 | -3.064697 | 0.41106461 |
| RAB9A | NM_004251 | 0.1216492 | 0.00798 | 2.7160576 | -2.692597 | 0.41079701 |
| MRPS36 | AF086034 | 0.2314641 | 0.0307 | 2.1942318 | -3.846249 | 0.41078374 |
| PDGFC | NM_016205 | 0.1236078 | 0.00822 | 2.7072488 | -2.713351 | 0.41063202 |
| PRKAA1 | NM_006251 | 0.1989551 | 0.0223 | 2.3279677 | -3.5041 | 0.41061137 |
| RECQL | NM_002907 | 0.0868362 | 0.00406 | 2.9556706 | -2.085468 | 0.41043145 |
| MRFAP1L1 | AF155654 | 0.0882579 | 0.00426 | 2.9306429 | -2.142123 | 0.41001527 |
| RPF1 | AK022537 | 0.1314509 | 0.00931 | 2.6589634 | -2.828386 | 0.40967925 |
| CCDC124 | AF086307 | 0.2825576 | 0.046 | 2.0221897 | -4.165188 | 0.40967008 |
| VPS28 | NM_016208 | 0.0940173 | 0.00482 | 2.8869496 | -2.278644 | 0.40965132 |
| PCCB | NM_000532 | 0.1440794 | 0.0115 | 2.5775857 | -3.029991 | 0.40947188 |
| SERPINE2 | M17783 | 0.2647036 | 0.0406 | 2.07615 | -4.076808 | 0.40913456 |
| MRPS31 | NM_005830 | 0.0713541 | 0.00276 | 3.0898751 | -1.704736 | 0.40881902 |
| JKAMP | NM_016475 | 0.2437347 | 0.034 | 2.1557685 | -3.847984 | 0.4085478 |
| INTS8 | NM_017864 | 0.0861216 | 0.00396 | 2.9556949 | -2.087277 | 0.40790632 |
| AZI2 | AK025592 | 0.1948683 | 0.0213 | 2.3427165 | -3.537911 | 0.40766608 |
| RNF181 | NM_016494 | 0.0939476 | 0.0048 | 2.8876912 | -2.276503 | 0.40717088 |
| GTF2H3 | NM_001516 | 0.0273904 | 0.000391 | 3.6786147 | -0.041765 | 0.40708588 |
| PSMC2 | NM_002803 | 0.291372 | 0.049 | 1.995687 | -4.222548 | 0.40690382 |
| NFASC | AB018299 | 0.0639603 | 0.00212 | 3.1635287 | -1.545262 | 0.40681981 |
| RUNX1T1 | NM_004349 | 0.2934384 | 0.0497 | 1.9895508 | -4.206029 | 0.40656221 |
| TIMP1 | NM_003254 | 0.271293 | 0.0425 | 2.0568514 | -4.086795 | 0.40653372 |
| CYP1A2 | NM_000761 | 0.1672424 | 0.0153 | 2.4695682 | -3.253607 | 0.4053028 |
| POLR2B | NM_000938 | 0.102283 | 0.00573 | 2.8296913 | -2.423483 | 0.40529653 |
| MSL3 | NM_006800 | 0.2596136 | 0.0392 | 2.0911387 | -4.048182 | 0.4052193 |
| TNFRSF11B | NM_002546 | 0.140492 | 0.0109 | 2.596448 | -2.976368 | 0.40519798 |
| KIF2A | NM_004520 | 0.1568827 | 0.0136 | 2.5154739 | -3.16933 | 0.40515774 |
| KLHL8 | AB037799 | 0.2028811 | 0.0231 | 2.3187678 | -3.53077 | 0.40510177 |
| DPP3 | AJ271216 | 0.2329418 | 0.0311 | 2.1933313 | -3.788387 | 0.40486298 |
| AIMP1 | NM_004757 | 0.1064568 | 0.00614 | 2.8069499 | -2.471852 | 0.40472577 |
| HEBP1 | NM_015987 | 0.1502516 | 0.0125 | 2.557906 | -3.029273 | 0.40452182 |
| PAFAH2 | NM_000437 | 0.0678009 | 0.00248 | 3.1234597 | -1.629651 | 0.4043332 |
| TMPO | NM_003276 | 0.2078456 | 0.0242 | 2.2906304 | -3.658296 | 0.40420618 |
| ZNF692 | NM_017865 | 0.0674912 | 0.00244 | 3.1206193 | -1.65748 | 0.40416419 |
| BEX1 | NM_018476 | 0.2448205 | 0.0343 | 2.1479358 | -3.938016 | 0.40372884 |
| CLTB | NM_007097 | 0.1310062 | 0.00926 | 2.6670268 | -2.780803 | 0.4030356 |
| AARS2 | AB033096 | 0.1144311 | 0.00709 | 2.7547477 | -2.599517 | 0.40291374 |
| COX7A1 | NM_001864 | 0.1681304 | 0.0155 | 2.4689101 | -3.240695 | 0.40283457 |
| NDUFAF3 | AL049955 | 0.282682 | 0.0462 | 2.0216567 | -4.160668 | 0.40272623 |
| RPL10 | NM_006013 | 0.1400503 | 0.0109 | 2.6132293 | -2.89464 | 0.4025838 |
| GAS5 | AL110141 | 0.2463376 | 0.0347 | 2.1435018 | -3.934106 | 0.40249425 |
| MRM1 | AK026231 | 0.1788119 | 0.0176 | 2.4169954 | -3.369064 | 0.4023434 |
| CDYL | NM_004824 | 0.1982129 | 0.0222 | 2.3271675 | -3.569926 | 0.40226512 |
| HERPUD1 | NM_014685 | 0.1503974 | 0.0126 | 2.5435264 | -3.087836 | 0.40173053 |
| ARHGEF12 | NM_015313 | 0.2278065 | 0.0297 | 2.2072393 | -3.819246 | 0.40134335 |
| CENPN | NM_018455 | 0.266287 | 0.041 | 2.0726484 | -4.057012 | 0.40102963 |
| PSMB10 | NM_002801 | 0.2181298 | 0.0269 | 2.2483538 | -3.724619 | 0.40095354 |
| KAT2A | AF029777 | 0.1297693 | 0.00904 | 2.6844079 | -2.654397 | 0.40039139 |
| EXT1 | NM_000127 | 0.1326671 | 0.00948 | 2.6581982 | -2.791208 | 0.4003342 |
| EXOC4 | AF132734 | 0.11382 | 0.00701 | 2.7660898 | -2.526188 | 0.40016732 |
| MRPS33 | NM_016071 | 0.1843199 | 0.0188 | 2.3900021 | -3.448402 | 0.40016043 |
| KHSRP | NM_003685 | 0.2857242 | 0.0475 | 2.0079579 | -4.217254 | 0.39981429 |
| MCM6 | NM_005915 | 0.1352168 | 0.00995 | 2.6341689 | -2.877419 | 0.399602 |
| DNAJC11 | NM_018198 | 0.1037841 | 0.00587 | 2.8475643 | -2.308773 | 0.39903321 |
| CARNS1 | AB037815 | 0.2688753 | 0.0416 | 2.0899282 | -3.771746 | 0.398905 |
| TOP1 | J03250 | 0.1388895 | 0.0106 | 2.6060828 | -2.953693 | 0.39815247 |
| MRPS22 | NM_020191 | 0.216408 | 0.0265 | 2.2544657 | -3.721629 | 0.39812207 |
| EPM2AIP1 | NM_014805 | 0.0821699 | 0.00361 | 2.9951793 | -1.967865 | 0.39797899 |
| SNRPB2 | NM_003092 | 0.2058797 | 0.0238 | 2.2967758 | -3.637517 | 0.39775198 |
| DLD | NM_000108 | 0.1741324 | 0.0167 | 2.4361908 | -3.347052 | 0.39758903 |
| CPVL | AF282617 | 0.067079 | 0.00242 | 3.1257537 | -1.631222 | 0.39724827 |
| SNX9 | NM_016224 | 0.1391336 | 0.0107 | 2.6039139 | -2.95902 | 0.39718938 |
| RAP2B | NM_002886 | 0.054419 | 0.0015 | 3.2768761 | -1.222295 | 0.39690487 |
| EMP2 | NM_001424 | 0.0868362 | 0.00407 | 2.9496377 | -2.105354 | 0.39688684 |
| HSPB11 | NM_016126 | 0.1338536 | 0.00972 | 2.6411336 | -2.871 | 0.39677976 |
| PLOD1 | NM_000302 | 0.1373219 | 0.0104 | 2.6175923 | -2.905716 | 0.39674005 |
| MACF1 | NM_012090 | 0.1866425 | 0.0194 | 2.3820705 | -3.363688 | 0.3961311 |
| TRIM37 | AB020705 | 0.1943126 | 0.0212 | 2.3446203 | -3.534637 | 0.39609029 |
| FAM216A | NM_013300 | 0.2343631 | 0.0316 | 2.1844465 | -3.846807 | 0.39608054 |
| PDSS1 | NM_014317 | 0.038955 | 0.000776 | 3.493077 | -0.619768 | 0.39591867 |
| PGM1 | NM_002633 | 0.1964853 | 0.0217 | 2.3344928 | -3.547122 | 0.39556748 |
| PTRH2 | NM_016077 | 0.1346767 | 0.00984 | 2.6376134 | -2.869545 | 0.39486517 |
| IARS2 | NM_018060 | 0.0868362 | 0.00409 | 2.9484364 | -2.108429 | 0.39446882 |
| PARPBP | NM_017915 | 0.0321337 | 0.00052 | 3.6191335 | -0.258429 | 0.393926 |
| P2RX2 | NM_016318 | 0.1942796 | 0.0211 | 2.3476763 | -3.504644 | 0.39389461 |
| HIST1H2BN | NM_003520 | 0.0756724 | 0.00308 | 3.039609 | -1.859408 | 0.39389242 |
| SLC43A3 | NM_014096 | 0.216106 | 0.0262 | 2.2601527 | -3.709073 | 0.39385499 |
| COL4A4 | NM_000092 | 0.2364436 | 0.0321 | 2.1891079 | -3.713125 | 0.39381824 |
| PATZ1 | AF242522 | 0.1667447 | 0.0152 | 2.4735462 | -3.263168 | 0.39334928 |
| UBE2M | NM_003969 | 0.1202127 | 0.00785 | 2.7206339 | -2.672895 | 0.39331726 |
| SMC5 | AB011166 | 0.1173046 | 0.00746 | 2.741219 | -2.604919 | 0.39317816 |
| DUSP10 | AB026436 | 0.0946907 | 0.00489 | 2.892392 | -2.22811 | 0.39298246 |
| CPS1 | NM_001875 | 0.2032033 | 0.0232 | 2.3092932 | -3.596611 | 0.39285464 |
| CBX1 | NM_006807 | 0.1852395 | 0.019 | 2.3858224 | -3.425042 | 0.39276999 |
| ORMDL1 | NM_016467 | 0.1184467 | 0.00763 | 2.7293823 | -2.642213 | 0.39248472 |
| VPS4A | NM_013245 | 0.1373219 | 0.0105 | 2.6192694 | -2.901413 | 0.39120955 |
| HYOU1 | NM_006389 | 0.1888942 | 0.02 | 2.370258 | -3.476214 | 0.39104159 |
| PIH1D1 | NM_017916 | 0.1697769 | 0.0159 | 2.4553421 | -3.30736 | 0.39070041 |
| ADSL | NM_000026 | 0.0829795 | 0.00368 | 2.9795407 | -2.033239 | 0.39025341 |
| CCDC91 | NM_018318 | 0.0586227 | 0.00179 | 3.2196734 | -1.38027 | 0.3901375 |
| CYP51A1 | NM_000786 | 0.1789884 | 0.0177 | 2.4203904 | -3.333232 | 0.38986436 |
| IFT20 | AF070643 | 0.2099044 | 0.0247 | 2.2844672 | -3.656242 | 0.38974964 |
| LARS2 | NM_015340 | 0.1185939 | 0.00768 | 2.7430043 | -2.5325 | 0.38970761 |
| CAND1 | NM_018448 | 0.1166385 | 0.00736 | 2.7407677 | -2.633457 | 0.38944516 |
| CLASRP | NM_007056 | 0.221853 | 0.028 | 2.2347573 | -3.755743 | 0.38934095 |
| RHOQ | NM_012249 | 0.0868362 | 0.00409 | 2.9486733 | -2.107823 | 0.38881114 |
| CYB5R1 | NM_016243 | 0.1897973 | 0.0203 | 2.3630823 | -3.460021 | 0.38874837 |
| TNFRSF12A | NM_016639 | 0.2678224 | 0.0413 | 2.0684592 | -4.104868 | 0.38867829 |
| PKIG | NM_007066 | 0.2720567 | 0.0427 | 2.058063 | -4.048139 | 0.38850563 |
| GPNMB | NM_002510 | 0.1468345 | 0.012 | 2.5639392 | -3.050301 | 0.38849472 |
| QDPR | NM_000320 | 0.0678009 | 0.00248 | 3.1292666 | -1.621979 | 0.38784496 |
| MGAT4B | NM_014275 | 0.1776431 | 0.0174 | 2.4222937 | -3.377494 | 0.3871748 |
| HMGXB4 | AL079310 | 0.1703464 | 0.016 | 2.4541356 | -3.287724 | 0.38694855 |
| CYSLTR2 | NM_020377 | 0.2401191 | 0.0331 | 2.1661809 | -3.844731 | 0.38648594 |
| ALG3 | NM_005787 | 0.1002555 | 0.0055 | 2.8464253 | -2.347555 | 0.38646232 |
| KMT2A | AF272377 | 0.1184174 | 0.0076 | 2.7488875 | -2.554395 | 0.38634203 |
| MRPS11 | AK027059 | 0.1185939 | 0.00766 | 2.7265877 | -2.667752 | 0.38626187 |
| TIMMDC1 | NM_016589 | 0.1140546 | 0.00704 | 2.7588727 | -2.580653 | 0.38615559 |
| HELZ | NM_014877 | 0.1264755 | 0.0085 | 2.6955796 | -2.723315 | 0.38595689 |
| PGS1 | AL359590 | 0.1175962 | 0.00749 | 2.7328298 | -2.643237 | 0.3850865 |
| SMIM7 | AK025602 | 0.145209 | 0.0116 | 2.5784263 | -2.995236 | 0.38502468 |
| OGG1 | NM_016819 | 0.1289463 | 0.00887 | 2.6969882 | -2.666648 | 0.3845994 |
| MRPL24 | AK024570 | 0.1029763 | 0.00581 | 2.8267066 | -2.405346 | 0.38456053 |
| CRLF1 | NM_004750 | 0.1184174 | 0.00758 | 2.7326375 | -2.644043 | 0.38455451 |
| ABCC1 | NM_004996 | 0.1839549 | 0.0186 | 2.3987479 | -3.39238 | 0.38441498 |
| SLC27A2 | NM_003645 | 0.1967942 | 0.0219 | 2.3408177 | -3.46112 | 0.38430366 |
| HSD17B4 | NM_000414 | 0.1788119 | 0.0175 | 2.4202736 | -3.34826 | 0.38430101 |
| KLHL9 | AB037775 | 0.1119967 | 0.00676 | 2.7765468 | -2.509953 | 0.38402797 |
| AADAT | NM_016228 | 0.1419554 | 0.0111 | 2.5950394 | -2.935966 | 0.38299195 |
| CD2AP | AF146277 | 0.1970083 | 0.0219 | 2.3323379 | -3.524283 | 0.38283208 |
| PTS | NM_000317 | 0.1075002 | 0.00627 | 2.8004502 | -2.470276 | 0.38277985 |
| CAMSAP3 | AB040976 | 0.2017761 | 0.0229 | 2.3161506 | -3.529858 | 0.38253018 |
| DNAJC15 | NM_013238 | 0.1458999 | 0.0118 | 2.5736098 | -3.026109 | 0.38223371 |
| EIF1B | NM_005875 | 0.2186316 | 0.0272 | 2.2442545 | -3.741555 | 0.38187771 |
| CEP290 | NM_014684 | 0.1542309 | 0.0132 | 2.5277674 | -3.111595 | 0.38185953 |
| TAF1C | NM_005679 | 0.0660645 | 0.00231 | 3.139668 | -1.592616 | 0.38171382 |
| PTDSS1 | NM_014754 | 0.2206434 | 0.0277 | 2.237117 | -3.745882 | 0.3816655 |
| EMC7 | NM_020154 | 0.1630289 | 0.0145 | 2.4929736 | -3.209247 | 0.38103594 |
| LOC105377443 | U79300 | 0.2686691 | 0.0415 | 2.0942071 | -3.721754 | 0.38100778 |
| COX7A2L | NM_004718 | 0.1863662 | 0.0193 | 2.3801966 | -3.45939 | 0.38098477 |
| CHEK1 | NM_001274 | 0.0852088 | 0.0039 | 2.9675269 | -2.061589 | 0.38087355 |
| FRG1 | NM_004477 | 0.2512732 | 0.0368 | 2.1178434 | -3.997701 | 0.38062749 |
| SCO1 | NM_004589 | 0.2079212 | 0.0243 | 2.2912027 | -3.621959 | 0.38028336 |
| MAGOH | NM_002370 | 0.1510389 | 0.0127 | 2.5418262 | -3.068511 | 0.3800315 |
| MYEOV | AJ223366 | 0.2103445 | 0.0249 | 2.279952 | -3.670068 | 0.37988072 |
| DCXR | NM_016286 | 0.2606008 | 0.0394 | 2.090499 | -4.045084 | 0.37981466 |
| EIF2B4 | AF112207 | 0.0585081 | 0.00176 | 3.2565806 | -1.298882 | 0.37965305 |
| STARD9 | AK025589 | 0.2317714 | 0.0307 | 2.1978135 | -3.793079 | 0.37873014 |
| HMGB1 | AL110194 | 0.1468345 | 0.012 | 2.5694004 | -3.014935 | 0.37861639 |
| PTPN4 | NM_002830 | 0.1892138 | 0.02 | 2.3703845 | -3.441447 | 0.37855514 |
| TRMT11 | AF182423 | 0.0307001 | 0.000472 | 3.6320865 | -0.202535 | 0.37840115 |
| CYB561A3 | AK024953 | 0.0990149 | 0.00532 | 2.8591434 | -2.334511 | 0.37839769 |
| MPC1 | NM_016098 | 0.2510017 | 0.0367 | 2.1230374 | -3.952989 | 0.37827804 |
| IRS1 | NM_005544 | 0.2099182 | 0.0248 | 2.2871031 | -3.587447 | 0.37737946 |
| ZWINT | NM_007057 | 0.1999595 | 0.0226 | 2.3189492 | -3.5888 | 0.3773415 |
| ACYP1 | NM_001107 | 0.1533235 | 0.0131 | 2.5333142 | -3.098219 | 0.37730838 |
| SH3GL1P3 | X99662 | 0.0795162 | 0.0034 | 3.0180631 | -1.919806 | 0.37679733 |
| ADK | NM_006721 | 0.1888942 | 0.02 | 2.3664359 | -3.500641 | 0.37663106 |
| H2AFX | NM_002105 | 0.1863506 | 0.0193 | 2.3919288 | -3.389513 | 0.37658193 |
| C17orf58 | AK026583 | 0.1222888 | 0.00807 | 2.708885 | -2.701004 | 0.37553151 |
| NR2F1 | NM_005654 | 0.0319727 | 0.000504 | 3.614324 | -0.259116 | 0.37532566 |
| AAK1 | NM_014911 | 0.2866196 | 0.0478 | 2.009996 | -4.120142 | 0.37521567 |
| ZNRD1 | NM_014596 | 0.1045094 | 0.00595 | 2.8172948 | -2.437422 | 0.37488983 |
| NCAPH2 | AL021682 | 0.2464837 | 0.035 | 2.147799 | -3.812388 | 0.37421923 |
| SCARA3 | NM_016240 | 0.1158476 | 0.00726 | 2.7601728 | -2.562745 | 0.37377242 |
| B3GALT1 | AF117222 | 0.1613774 | 0.0142 | 2.5177167 | -3.061426 | 0.3727291 |
| SMKR1 | AF086124 | 0.2342629 | 0.0316 | 2.2003341 | -3.655496 | 0.37247463 |
| TPP2 | NM_003291 | 0.0812343 | 0.00352 | 2.9994405 | -1.96884 | 0.3722354 |
| ACOX2 | NM_003500 | 0.1432404 | 0.0113 | 2.5895099 | -2.95968 | 0.37217016 |
| GGNBP2 | AK024883 | 0.1274252 | 0.00865 | 2.6828517 | -2.753116 | 0.37214238 |
| ATP6V1H | NM_015941 | 0.0498021 | 0.0012 | 3.3522619 | -1.0257 | 0.37214126 |
| GUSBP3 | L40520 | 0.2448205 | 0.0343 | 2.1494564 | -3.930981 | 0.37179048 |
| RPS19 | NM_001022 | 0.1489447 | 0.0123 | 2.5608375 | -3.051566 | 0.37178087 |
| TTC37 | NM_014639 | 0.2818304 | 0.0457 | 2.026191 | -4.152277 | 0.37167003 |
| PARL | NM_018622 | 0.1449966 | 0.0116 | 2.5808993 | -2.979057 | 0.37160489 |
| GNAI1 | NM_002069 | 0.1567849 | 0.0136 | 2.5183035 | -3.131918 | 0.3714563 |
| RABL2B | NM_007081 | 0.1480668 | 0.0122 | 2.5715809 | -2.916142 | 0.37112746 |
| APOL3 | NM_014349 | 0.2382257 | 0.0325 | 2.1733504 | -3.855197 | 0.37073891 |
| SEC61B | NM_006808 | 0.2761466 | 0.0439 | 2.04253 | -4.113616 | 0.3707034 |
| CXADR | NM_001338 | 0.247639 | 0.0354 | 2.1374456 | -3.900466 | 0.37022756 |
| POP5 | NM_015918 | 0.1359438 | 0.0101 | 2.6291346 | -2.868611 | 0.37011374 |
| PROS1 | NM_000313 | 0.060664 | 0.00187 | 3.2045052 | -1.434489 | 0.36989458 |
| SNHG4 | AJ224170 | 0.2636624 | 0.0403 | 2.0971637 | -3.80349 | 0.36979461 |
| RDX | NM_002906 | 0.0712292 | 0.00275 | 3.0874321 | -1.745747 | 0.36966623 |
| CAMLG | NM_001745 | 0.2464837 | 0.0349 | 2.1395281 | -3.968575 | 0.36957077 |
| DSP | NM_004415 | 0.1124338 | 0.00681 | 2.7723106 | -2.539144 | 0.36949902 |
| HSD17B10 | NM_004493 | 0.2694432 | 0.0418 | 2.0657414 | -4.088497 | 0.36935669 |
| MOXD1 | AY007239 | 0.1849838 | 0.019 | 2.3896219 | -3.424582 | 0.36860434 |
| PSMG1 | NM_003720 | 0.1814882 | 0.0181 | 2.4069418 | -3.378092 | 0.36857793 |
| SLC39A8 | AL049963 | 0.1550215 | 0.0133 | 2.5267164 | -3.078141 | 0.36850571 |
| HEXA | NM_000520 | 0.2855321 | 0.0473 | 2.0113377 | -4.166233 | 0.36841441 |
| USE1 | NM_018467 | 0.2636624 | 0.0403 | 2.0832458 | -4.039312 | 0.36834924 |
| IER3IP1 | NM_016097 | 0.1567931 | 0.0136 | 2.516904 | -3.156183 | 0.36791337 |
| C3 | NM_000064 | 0.2577555 | 0.0385 | 2.1020317 | -3.952282 | 0.36737757 |
| DNAJC27 | AF178983 | 0.2206434 | 0.0277 | 2.2449466 | -3.668465 | 0.36705251 |
| USP11 | NM_004651 | 0.216408 | 0.0265 | 2.2564842 | -3.69165 | 0.366857 |
| TRMT6 | NM_015939 | 0.1184174 | 0.0076 | 2.7302478 | -2.639996 | 0.36595353 |
| MRPS18B | NM_014046 | 0.1508131 | 0.0127 | 2.5498368 | -3.058744 | 0.36570402 |
| PRKAR2A | NM_004157 | 0.1976163 | 0.022 | 2.3306607 | -3.551143 | 0.36550097 |
| ART1 | NM_004314 | 0.2229331 | 0.0284 | 2.2282119 | -3.748016 | 0.36511193 |
| DUSP7 | AL110157 | 0.1109815 | 0.00667 | 2.7866711 | -2.478718 | 0.36501173 |
| HSPG2 | NM_005529 | 0.2397153 | 0.033 | 2.1791 | -3.746613 | 0.36472631 |
| DPM3 | NM_018973 | 0.1987813 | 0.0223 | 2.3265541 | -3.569532 | 0.36458712 |
| USP7 | NM_003470 | 0.1082155 | 0.00633 | 2.7948872 | -2.492405 | 0.36425133 |
| NDUFAF1 | NM_016013 | 0.150826 | 0.0127 | 2.5438415 | -3.085104 | 0.3642016 |
| C9orf78 | NM_016520 | 0.0939476 | 0.00476 | 2.8966048 | -2.24013 | 0.36389826 |
| DNMT3A | AF067972 | 0.2020607 | 0.023 | 2.3104185 | -3.608984 | 0.36381809 |
| NUDT15 | NM_018283 | 0.0996693 | 0.0054 | 2.8598414 | -2.319111 | 0.36346217 |
| COMMD4 | NM_017828 | 0.1789884 | 0.0177 | 2.4161016 | -3.368763 | 0.3630527 |
| BCL7C | NM_004765 | 0.0281913 | 0.000414 | 3.6627945 | -0.098096 | 0.3628216 |
| ZMAT2 | X97303 | 0.2336706 | 0.0313 | 2.1923131 | -3.811461 | 0.36278682 |
| EBAG9 | NM_004215 | 0.1572019 | 0.0137 | 2.512849 | -3.167241 | 0.36255953 |
| FIBP | NM_004214 | 0.1550215 | 0.0133 | 2.5276519 | -3.109946 | 0.36228619 |
| PNN | NM_002687 | 0.2464837 | 0.0349 | 2.1407195 | -3.952161 | 0.36222952 |
| RBMS3 | NM_014483 | 0.1124471 | 0.00681 | 2.7727416 | -2.547381 | 0.36199722 |
| SPOCK1 | NM_004598 | 0.2225464 | 0.0282 | 2.2327028 | -3.72447 | 0.36198727 |
| FBXO18 | AL133069 | 0.1052729 | 0.00602 | 2.8119506 | -2.450126 | 0.36167103 |
| SUCLG2 | AF131748 | 0.1943126 | 0.0212 | 2.3460013 | -3.50695 | 0.3615541 |
| SDC3 | NM_014654 | 0.1722851 | 0.0163 | 2.4589443 | -3.223721 | 0.36123499 |
| RND1 | NM_014470 | 0.1976163 | 0.022 | 2.3356801 | -3.473296 | 0.3610308 |
| S100A13 | NM_005979 | 0.2181298 | 0.0269 | 2.2482576 | -3.736089 | 0.36011868 |
| TBRG4 | NM_004749 | 0.204763 | 0.0236 | 2.3059553 | -3.586964 | 0.35977334 |
| COL4A6 | U04845 | 0.1808089 | 0.018 | 2.4316721 | -3.187917 | 0.35969978 |
| EFNA5 | AK025909 | 0.1877777 | 0.0197 | 2.4297547 | -3.211408 | 0.35934008 |
| POMT1 | NM_007171 | 0.1236078 | 0.00823 | 2.7011673 | -2.728856 | 0.35893792 |
| GAL | NM_015973 | 0.257798 | 0.0387 | 2.0972738 | -4.021647 | 0.35851933 |
| PDS5A | AB014548 | 0.2744064 | 0.0434 | 2.0474709 | -4.129975 | 0.35810285 |
| POLR2H | U37689 | 0.2647036 | 0.0406 | 2.0789342 | -4.038136 | 0.35768118 |
| TNS2 | AB028998 | 0.2579868 | 0.0389 | 2.0977294 | -3.989895 | 0.35735769 |
| EGFL7 | NM_016215 | 0.2935387 | 0.0498 | 1.9897651 | -4.215416 | 0.35696823 |
| GPN3 | NM_016301 | 0.1450954 | 0.0116 | 2.5789676 | -3.003789 | 0.35593148 |
| NMT1 | AF043324 | 0.1042627 | 0.00593 | 2.8195505 | -2.432115 | 0.35550681 |
| UBL4A | NM_014235 | 0.1170195 | 0.00742 | 2.7417716 | -2.61289 | 0.35543434 |
| PMF1 | NM_007221 | 0.2084829 | 0.0244 | 2.2881153 | -3.653157 | 0.35522621 |
| MTA3 | AB033092 | 0.1791112 | 0.0178 | 2.4188902 | -3.32529 | 0.3551986 |
| BAG4 | AL049466 | 0.2117172 | 0.0252 | 2.2782821 | -3.633672 | 0.35495109 |
| RNF7 | NM_014245 | 0.2244411 | 0.0289 | 2.2189723 | -3.807257 | 0.35476893 |
| ALG5 | NM_013338 | 0.1612765 | 0.0142 | 2.5043598 | -3.162314 | 0.35460059 |
| EARS2 | AF088053 | 0.1435988 | 0.0114 | 2.5851354 | -2.980567 | 0.35449891 |
| CYP4B1 | NM_000779 | 0.2227424 | 0.0284 | 2.2409363 | -3.596782 | 0.35431713 |
| DIABLO | NM_019887 | 0.0827977 | 0.00367 | 2.9809813 | -2.029492 | 0.354023 |
| HIKESHI | NM_016411 | 0.1376482 | 0.0105 | 2.6168432 | -2.916786 | 0.35389142 |
| NPTX2 | U26662 | 0.1624289 | 0.0144 | 2.5070069 | -3.054501 | 0.35374032 |
| ASPM | NM_018123 | 0.1302889 | 0.00917 | 2.6650264 | -2.774214 | 0.35334295 |
| MRPL28 | NM_006428 | 0.2365292 | 0.0321 | 2.1754925 | -3.882664 | 0.35268134 |
| THAP11 | L10378 | 0.1472547 | 0.0121 | 2.5641909 | -3.01727 | 0.35252365 |
| EDEM2 | NM_018217 | 0.2825757 | 0.046 | 2.0226749 | -4.172795 | 0.35221157 |
| CPSF1 | NM_013291 | 0.1852395 | 0.0191 | 2.3869913 | -3.442686 | 0.35218264 |
| CPZ | NM_003652 | 0.0459359 | 0.00105 | 3.4038658 | -0.868207 | 0.35192135 |
| TFIP11 | NM_012143 | 0.1697769 | 0.0159 | 2.4619977 | -3.265043 | 0.3519056 |
| CENPH | AB035124 | 0.1774842 | 0.0173 | 2.4225955 | -3.367519 | 0.35188492 |
| SDHD | NM_003002 | 0.1839197 | 0.0186 | 2.3932396 | -3.443549 | 0.35159535 |
| PMPCB | NM_004279 | 0.1842801 | 0.0187 | 2.3922908 | -3.444191 | 0.35143577 |
| PIGF | NM_002643 | 0.0793322 | 0.00339 | 3.0083591 | -1.966349 | 0.35120226 |
| CSNK2A2 | NM_001896 | 0.1166352 | 0.00733 | 2.7438066 | -2.626068 | 0.35095374 |
| TOMM22 | NM_020243 | 0.2095508 | 0.0246 | 2.2906532 | -3.61496 | 0.35089965 |
| TTR | NM_000371 | 0.0924658 | 0.00463 | 2.9002343 | -2.244801 | 0.35049666 |
| RPL17 | NM_000985 | 0.2490787 | 0.0359 | 2.1336145 | -3.889786 | 0.3502916 |
| RRP15 | NM_016052 | 0.2411486 | 0.0333 | 2.1595985 | -3.914103 | 0.3502101 |
| TFB2M | AK026835 | 0.0869648 | 0.00412 | 2.9561339 | -2.055077 | 0.34957226 |
| SIRT4 | NM_012240 | 0.1883208 | 0.0198 | 2.3735931 | -3.458129 | 0.34956079 |
| GAPVD1 | AB040954 | 0.216106 | 0.0265 | 2.2635935 | -3.655164 | 0.34947539 |
| TINF2 | NM_012461 | 0.1163321 | 0.0073 | 2.7452997 | -2.622452 | 0.3491097 |
| MFAP1 | NM_005926 | 0.1521116 | 0.0129 | 2.5395851 | -3.061288 | 0.34901119 |
| KCTD9 | NM_017634 | 0.0861086 | 0.00395 | 2.9581039 | -2.082341 | 0.34899058 |
| NAPEPLD | AK000801 | 0.156062 | 0.0135 | 2.526778 | -3.088871 | 0.34874562 |
| MTMR8 | NM_017677 | 0.278402 | 0.0447 | 2.0569593 | -3.803013 | 0.34874497 |
| GGH | NM_003878 | 0.2095508 | 0.0246 | 2.2856616 | -3.644875 | 0.34840085 |
| MAF | X79984 | 0.2015295 | 0.0229 | 2.3414444 | -3.337032 | 0.34810417 |
| PIK3R2 | NM_005027 | 0.1719792 | 0.0163 | 2.4567743 | -3.190459 | 0.34810231 |
| UBA5 | AK026904 | 0.0705335 | 0.00269 | 3.0956455 | -1.716694 | 0.34735127 |
| AP3B1 | NM_003664 | 0.1158476 | 0.00724 | 2.7461261 | -2.611171 | 0.34731423 |
| NTN1 | NM_004822 | 0.2279568 | 0.0298 | 2.2079447 | -3.791165 | 0.34730576 |
| SLC45A2 | NM_016180 | 0.2505713 | 0.0365 | 2.1241227 | -3.963608 | 0.34725011 |
| PRKAB1 | NM_006253 | 0.1341506 | 0.00977 | 2.6449527 | -2.841843 | 0.34721822 |
| NT5E | NM_002526 | 0.1054485 | 0.00604 | 2.8197704 | -2.40738 | 0.3472082 |
| UBE2N | NM_003348 | 0.0583001 | 0.00173 | 3.2382353 | -1.333229 | 0.34719962 |
| FAM118B | AK024756 | 0.2349399 | 0.0317 | 2.1874098 | -3.753713 | 0.34688762 |
| SEC22B | NM_004892 | 0.1013524 | 0.00559 | 2.8346283 | -2.392701 | 0.34668003 |
| EIF2AK3 | NM_004836 | 0.2704563 | 0.0421 | 2.0621767 | -4.071843 | 0.34662221 |
| SERPINA3 | NM_001085 | 0.2340873 | 0.0314 | 2.1863691 | -3.854903 | 0.34658783 |
| GTPBP4 | NM_012341 | 0.1341506 | 0.00978 | 2.6468083 | -2.827683 | 0.34620506 |
| GPATCH8 | AB011125 | 0.2558741 | 0.038 | 2.1059778 | -4.002909 | 0.3459735 |
| TSC22D1 | NM_006022 | 0.2236067 | 0.0286 | 2.2260959 | -3.739311 | 0.34590736 |
| SLC25A4 | NM_001151 | 0.2769804 | 0.0442 | 2.0436818 | -4.099391 | 0.34527872 |
| MGP | NM_000900 | 0.2787014 | 0.0448 | 2.0332865 | -4.144558 | 0.3451466 |
| TMEM165 | NM_018475 | 0.1302889 | 0.00915 | 2.6659906 | -2.80256 | 0.34506368 |
| TNFAIP8 | NM_014350 | 0.2342629 | 0.0316 | 2.1857985 | -3.805554 | 0.34452499 |
| SAC3D1 | NM_013299 | 0.0766741 | 0.0032 | 3.0297639 | -1.889014 | 0.34448516 |
| SLC39A6 | NM_012319 | 0.1028461 | 0.00579 | 2.8257189 | -2.433271 | 0.34439766 |
| RSF1 | NM_016578 | 0.2184318 | 0.027 | 2.2465172 | -3.728365 | 0.34435003 |
| UTP18 | NM_016001 | 0.1512545 | 0.0128 | 2.539428 | -3.096176 | 0.34426438 |
| GMPR2 | NM_016576 | 0.2020999 | 0.023 | 2.312078 | -3.601571 | 0.34418346 |
| SRP19 | NM_003135 | 0.2824824 | 0.0459 | 2.0229319 | -4.187076 | 0.34382661 |
| RAB11FIP5 | AB020664 | 0.1738254 | 0.0167 | 2.4514112 | -3.172995 | 0.34351581 |
| SUPT3H | NM_003599 | 0.0876152 | 0.00418 | 2.9535979 | -2.081344 | 0.34312073 |
| RFC1 | NM_002913 | 0.0585081 | 0.00177 | 3.2319476 | -1.338379 | 0.34310041 |
| SHQ1 | NM_018130 | 0.1462109 | 0.0119 | 2.570984 | -3.001866 | 0.34302962 |
| HSBP1 | NM_001537 | 0.1722845 | 0.0163 | 2.4466218 | -3.313657 | 0.34302612 |
| CSTF2 | NM_001325 | 0.1845572 | 0.0189 | 2.3909747 | -3.399695 | 0.34289777 |
| MRPS17 | NM_015969 | 0.25461 | 0.0376 | 2.1086034 | -4.02458 | 0.34237759 |
| PIGH | NM_004569 | 0.1436534 | 0.0114 | 2.5838701 | -2.98366 | 0.34236571 |
| RFC4 | NM_002916 | 0.1550215 | 0.0133 | 2.5264113 | -3.113411 | 0.34212932 |
| LIPT1 | NM_015929 | 0.1667447 | 0.0152 | 2.4757883 | -3.226003 | 0.34208196 |
| GPR37L1 | NM_004767 | 0.282682 | 0.0463 | 2.0383771 | -3.857965 | 0.34194056 |
| RIOK2 | NM_018343 | 0.2462336 | 0.0346 | 2.1519166 | -3.850377 | 0.34154224 |
| ANGEL1 | AL137268 | 0.0572249 | 0.00166 | 3.2486575 | -1.307644 | 0.34154135 |
| PQBP1 | NM_005710 | 0.2499157 | 0.0363 | 2.1234709 | -3.973102 | 0.34147867 |
| POLD2 | NM_006230 | 0.163448 | 0.0146 | 2.4944947 | -3.16127 | 0.34139854 |
| ACSM3 | NM_005622 | 0.1745981 | 0.0168 | 2.4369554 | -3.299763 | 0.34125069 |
| F3 | NM_001993 | 0.2752204 | 0.0437 | 2.0590756 | -3.96588 | 0.34102939 |
| AKAP8 | NM_005858 | 0.0827977 | 0.00366 | 2.9886379 | -1.991082 | 0.340822 |
| TMED5 | NM_016040 | 0.1646272 | 0.0149 | 2.483787 | -3.197892 | 0.34070487 |
| CHDH | AJ272267 | 0.1839197 | 0.0186 | 2.4023285 | -3.32026 | 0.34037038 |
| GRSF1 | NM_002092 | 0.150826 | 0.0127 | 2.5418824 | -3.110059 | 0.34031381 |
| SERPIND1 | NM_000185 | 0.2662583 | 0.0409 | 2.0726601 | -4.082388 | 0.33976908 |
| HEXA | S76984 | 0.2437347 | 0.034 | 2.154383 | -3.915631 | 0.33963174 |
| TRAPPC2L | NM_016209 | 0.136162 | 0.0102 | 2.6279109 | -2.881584 | 0.33931197 |
| CDC45 | NM_003504 | 0.2550855 | 0.0378 | 2.1064127 | -4.019815 | 0.33920581 |
| TMEM254 | AF147339 | 0.2244403 | 0.0288 | 2.2374604 | -3.633344 | 0.33882522 |
| INCENP | NM_020238 | 0.1000418 | 0.00547 | 2.8436003 | -2.379835 | 0.33803675 |
| ZSCAN29 | AK023525 | 0.1132586 | 0.00694 | 2.7687431 | -2.539461 | 0.33771312 |
| PPP1R2P9 | AB044137 | 0.2938924 | 0.0499 | 1.9990824 | -3.997979 | 0.33766406 |
| SERPINB2 | NM_002575 | 0.2186316 | 0.0271 | 2.2501795 | -3.6992 | 0.3371497 |
| DUSP3 | NM_004090 | 0.222299 | 0.0281 | 2.2424936 | -3.594391 | 0.33686015 |
| NASP | NM_002482 | 0.2410863 | 0.0333 | 2.1603508 | -3.91262 | 0.33630843 |
| POLD1 | NM_002691 | 0.1139144 | 0.00703 | 2.7679134 | -2.51307 | 0.33557317 |
| CIB1 | NM_006384 | 0.2314641 | 0.0306 | 2.1969884 | -3.812172 | 0.33556696 |
| AASDH | AF161436 | 0.0868362 | 0.00407 | 2.9542016 | -2.096377 | 0.33551076 |
| KLHL28 | NM_017658 | 0.2043777 | 0.0235 | 2.308132 | -3.569514 | 0.33520147 |
| MRPL3 | NM_007208 | 0.2710079 | 0.0423 | 2.0604213 | -4.086055 | 0.33498502 |
| RPP38 | NM_006414 | 0.1747585 | 0.0169 | 2.4330924 | -3.343956 | 0.33482472 |
| PKLR | M15465 | 0.2460278 | 0.0345 | 2.1520835 | -3.821645 | 0.33437173 |
| MYH10 | M69181 | 0.1507633 | 0.0126 | 2.5427492 | -3.088973 | 0.33432777 |
| CLEC10A | NM_006344 | 0.2152969 | 0.026 | 2.2812271 | -3.501519 | 0.33405122 |
| ORMDL2 | NM_014182 | 0.0988601 | 0.00526 | 2.8588411 | -2.342125 | 0.3339987 |
| RHAG | NM_000324 | 0.2070505 | 0.0241 | 2.2948165 | -3.613786 | 0.3338894 |
| GINS2 | NM_016095 | 0.1862647 | 0.0192 | 2.3838848 | -3.458392 | 0.33320824 |
| TARDBP | NM_007375 | 0.2218047 | 0.0279 | 2.2365469 | -3.740625 | 0.33317895 |
| SARS | NM_006513 | 0.2463376 | 0.0347 | 2.1448351 | -3.949443 | 0.33240192 |
| NINL | AB023197 | 0.2500105 | 0.0364 | 2.1229423 | -3.984791 | 0.3323867 |
| GPSM2 | NM_013296 | 0.1863506 | 0.0193 | 2.3843466 | -3.434463 | 0.33228234 |
| CSRP2 | NM_001321 | 0.1737714 | 0.0166 | 2.4411143 | -3.303421 | 0.33221026 |
| SMC2 | NM_006444 | 0.2099044 | 0.0248 | 2.2843971 | -3.622492 | 0.33206554 |
| BDP1 | AF298152 | 0.1376482 | 0.0105 | 2.6149874 | -2.921812 | 0.33201973 |
| ARHGAP28 | AK024298 | 0.2763419 | 0.044 | 2.0658716 | -3.789074 | 0.33180175 |
| KCNMB4 | NM_014505 | 0.2619357 | 0.0397 | 2.08522 | -4.060527 | 0.33160143 |
| ACTR8 | AK026232 | 0.1103533 | 0.00659 | 2.780658 | -2.53626 | 0.33153709 |
| SNORD7 | AJ007733 | 0.1819598 | 0.0182 | 2.4151575 | -3.292433 | 0.33140503 |
| KIF1BP | AB033105 | 0.2822876 | 0.0459 | 2.0241121 | -4.18238 | 0.33134299 |
| C12orf10 | AF289485 | 0.1979702 | 0.0221 | 2.32774 | -3.570311 | 0.33126708 |
| DST | X75692 | 0.2327579 | 0.0311 | 2.1892713 | -3.866008 | 0.33115012 |
| EFCAB2 | AK001393 | 0.282682 | 0.0463 | 2.0215707 | -4.158409 | 0.33110707 |
| PARD6G | AB043635 | 0.1064568 | 0.00614 | 2.8245099 | -2.375553 | 0.33104197 |
| CHCHD3 | NM_017812 | 0.216106 | 0.0264 | 2.2568149 | -3.717706 | 0.33094409 |
| TIMM21 | NM_014177 | 0.2285263 | 0.0299 | 2.2068065 | -3.780138 | 0.3308662 |
| RIPPLY3 | NM_018962 | 0.1490718 | 0.0124 | 2.5556596 | -3.047196 | 0.33069069 |
| KANSL2 | NM_017822 | 0.1965399 | 0.0217 | 2.3386435 | -3.507569 | 0.33042089 |
| PPFIA2 | AL157453 | 0.212861 | 0.0255 | 2.2713694 | -3.675915 | 0.33026595 |
| SCCPDH | NM_016002 | 0.2099044 | 0.0247 | 2.2852794 | -3.631964 | 0.33000707 |
| FASTKD2 | NM_014929 | 0.248582 | 0.0357 | 2.1372789 | -3.905913 | 0.32978272 |
| GIPC1 | NM_005716 | 0.2070505 | 0.0241 | 2.2993087 | -3.533236 | 0.32924026 |
| NOX1 | NM_007052 | 0.2763419 | 0.044 | 2.0487122 | -3.977669 | 0.32897119 |
| GALE | NM_000403 | 0.0766741 | 0.00321 | 3.0370971 | -1.862675 | 0.32876747 |
| SYT8 | AL137708 | 0.2498502 | 0.0362 | 2.127499 | -3.94646 | 0.32800251 |
| ISOC1 | NM_016048 | 0.0639603 | 0.00212 | 3.1724338 | -1.518274 | 0.32788101 |
| TFAM | NM_003201 | 0.1887147 | 0.0199 | 2.3689461 | -3.492936 | 0.32784079 |
| KIN | AJ005273 | 0.257798 | 0.0386 | 2.1017808 | -3.980224 | 0.32779056 |
| PRKG2 | NM_006259 | 0.271026 | 0.0424 | 2.0756029 | -3.916329 | 0.32760078 |
| LINC01152 | D43770 | 0.228869 | 0.03 | 2.2051429 | -3.818549 | 0.32757335 |
| ENG | NM_000118 | 0.2101009 | 0.0248 | 2.2816097 | -3.642442 | 0.32745158 |
| BAD | U66879 | 0.0924658 | 0.00464 | 2.9046272 | -2.227618 | 0.32736523 |
| RRP36 | AF161371 | 0.1897973 | 0.0203 | 2.3629396 | -3.482918 | 0.32715306 |
| C19orf24 | NM_017914 | 0.244903 | 0.0343 | 2.1474734 | -3.94991 | 0.32684201 |
| TRIM44 | NM_017583 | 0.0917737 | 0.00456 | 2.9169686 | -2.147856 | 0.32614235 |
| TSPAN13 | NM_014399 | 0.2463049 | 0.0346 | 2.1511217 | -3.807159 | 0.3258046 |
| CUL1 | NM_003592 | 0.2491679 | 0.0359 | 2.1296998 | -3.968429 | 0.32544819 |
| RCSD1 | AK025425 | 0.2152969 | 0.026 | 2.2609448 | -3.723346 | 0.32522678 |
| ETFDH | NM_004453 | 0.1146701 | 0.00713 | 2.7552387 | -2.570411 | 0.32441912 |
| SLC39A4 | NM_017767 | 0.2464837 | 0.0351 | 2.1402835 | -3.934511 | 0.32420657 |
| SLC25A13 | NM_014251 | 0.1052729 | 0.00601 | 2.8207412 | -2.394486 | 0.32386676 |
| CWF19L1 | NM_018294 | 0.1790875 | 0.0177 | 2.4193639 | -3.324275 | 0.32359287 |
| LOC90246 | AK023635 | 0.2788112 | 0.0449 | 2.0568499 | -3.803889 | 0.32337499 |
| PLGRKT | NM_018465 | 0.2567844 | 0.0383 | 2.1025998 | -4.010427 | 0.32318095 |
| VMA21 | AK025798 | 0.2928173 | 0.0495 | 1.9925387 | -4.185269 | 0.32285978 |
| MED1 | NM_004774 | 0.1982129 | 0.0222 | 2.3268901 | -3.548991 | 0.32191927 |
| NRGN | NM_006176 | 0.2381674 | 0.0324 | 2.171014 | -3.891544 | 0.3215272 |
| PARD3 | NM_019619 | 0.2538409 | 0.0375 | 2.111852 | -3.99162 | 0.3206987 |
| VPS51 | NM_013265 | 0.1706246 | 0.016 | 2.4545595 | -3.295579 | 0.32042546 |
| NRN1 | NM_016588 | 0.216989 | 0.0267 | 2.2550323 | -3.692966 | 0.32002073 |
| POLRMT | NM_005035 | 0.1667447 | 0.0153 | 2.4802911 | -3.179428 | 0.31951407 |
| SCAPER | AB040887 | 0.1236078 | 0.00823 | 2.7028998 | -2.705637 | 0.31931842 |
| UBXN4 | D87684 | 0.2537037 | 0.0374 | 2.1111857 | -3.997781 | 0.31928563 |
| KARS | NM_005548 | 0.1352168 | 0.00996 | 2.635055 | -2.865227 | 0.31916128 |
| UQCC1 | NM_018244 | 0.1338095 | 0.00969 | 2.6521121 | -2.759672 | 0.31889813 |
| EIF6 | NM_002212 | 0.1314509 | 0.00931 | 2.674622 | -2.689928 | 0.31876809 |
| P4HA2 | NM_004199 | 0.1410211 | 0.011 | 2.6020234 | -2.897059 | 0.31823661 |
| C1S | NM_001734 | 0.2498502 | 0.0362 | 2.1278151 | -3.957605 | 0.31771731 |
| ASF1A | NM_014034 | 0.2488785 | 0.0358 | 2.1406106 | -3.787843 | 0.31752873 |
| DCTD | NM_001921 | 0.1456688 | 0.0117 | 2.573614 | -2.984809 | 0.31710232 |
| CYP20A1 | AF183412 | 0.2227267 | 0.0283 | 2.2385882 | -3.602328 | 0.31688788 |
| CDV3 | NM_017548 | 0.2932053 | 0.0496 | 1.9885676 | -4.240323 | 0.31686342 |
| C5 | NM_001735 | 0.2036611 | 0.0233 | 2.3078934 | -3.587372 | 0.31682108 |
| TAF11 | NM_005643 | 0.1602764 | 0.014 | 2.5037297 | -3.187287 | 0.31655105 |
| FKBP9 | AL050187 | 0.2736918 | 0.0431 | 2.0512414 | -4.107837 | 0.31648432 |
| PIK3CB | NM_006219 | 0.2686691 | 0.0415 | 2.0674626 | -4.077306 | 0.31632414 |
| GID8 | AK025775 | 0.2267949 | 0.0294 | 2.2152697 | -3.760147 | 0.31613988 |
| PPP2R5D | NM_006245 | 0.0716512 | 0.00279 | 3.0788304 | -1.747222 | 0.31603955 |
| NDUFS1 | NM_005006 | 0.1526012 | 0.013 | 2.5332153 | -3.12074 | 0.31559158 |
| CCDC136 | AL133027 | 0.2099044 | 0.0247 | 2.3024184 | -3.460441 | 0.31542804 |
| MELK | NM_014791 | 0.1996764 | 0.0225 | 2.3218101 | -3.568946 | 0.31493819 |
| CENPC | NM_001812 | 0.1550215 | 0.0133 | 2.5265981 | -3.10189 | 0.31480619 |
| GABPB1 | NM_005254 | 0.1420887 | 0.0112 | 2.5910973 | -2.98713 | 0.31476684 |
| PRSS3 | NM_002771 | 0.216408 | 0.0265 | 2.2552636 | -3.707278 | 0.31474849 |
| AKAP1 | NM_003488 | 0.1398508 | 0.0108 | 2.6146704 | -2.870203 | 0.31459986 |
| MRPL47 | NM_020409 | 0.216106 | 0.0264 | 2.2650139 | -3.600194 | 0.31449911 |
| SAFB2 | NM_014649 | 0.2181298 | 0.0269 | 2.2478248 | -3.747562 | 0.31446709 |
| FAM86C1 | NM_018172 | 0.2191653 | 0.0274 | 2.2463585 | -3.706913 | 0.31444087 |
| TRAPPC3 | NM_014408 | 0.0953161 | 0.00495 | 2.8810755 | -2.286794 | 0.31440746 |
| TTC3 | D84294 | 0.1297693 | 0.00906 | 2.6650742 | -2.815129 | 0.31418916 |
| YRDC | AK027129 | 0.1166385 | 0.00736 | 2.7470719 | -2.59118 | 0.31390856 |
| UBR5-AS1///UBR5 | AF090904 | 0.1362564 | 0.0102 | 2.6319456 | -2.851791 | 0.31361294 |
| PPP4R4 | AB046842 | 0.1751502 | 0.0169 | 2.4458852 | -3.199834 | 0.31290148 |
| IDH3B | NM_006899 | 0.2020999 | 0.023 | 2.3120352 | -3.59089 | 0.3125703 |
| MRPS7 | NM_015971 | 0.2206434 | 0.0277 | 2.2399315 | -3.723541 | 0.31216941 |
| IFI27L2 | AF208232 | 0.1227852 | 0.00813 | 2.7161136 | -2.656611 | 0.31206455 |
| ATR | NM_001184 | 0.2921387 | 0.0492 | 1.9983213 | -4.136993 | 0.31174502 |
| UBTF | NM_014233 | 0.172577 | 0.0164 | 2.4461152 | -3.303337 | 0.31142869 |
| GATAD1 | AK026142 | 0.2717866 | 0.0427 | 2.0591537 | -4.03092 | 0.31140551 |
| MPI | NM_002435 | 0.1216492 | 0.008 | 2.7113461 | -2.685227 | 0.31103618 |
| UCHL1 | NM_004181 | 0.1803885 | 0.0179 | 2.4260856 | -3.241656 | 0.31076131 |
| DTL | NM_016448 | 0.1334078 | 0.00962 | 2.6450544 | -2.842183 | 0.31040134 |
| GOLT1B | NM_016072 | 0.2606622 | 0.0394 | 2.092355 | -3.998106 | 0.31034975 |
| ZNF92 | M61872 | 0.2186316 | 0.0272 | 2.2469139 | -3.685245 | 0.31009942 |
| RBMX2 | NM_016024 | 0.2921387 | 0.0492 | 1.9932612 | -4.215179 | 0.31006788 |
| DIAPH2 | NM_006729 | 0.2070505 | 0.0241 | 2.2993688 | -3.585997 | 0.30979219 |
| GTF2E2 | NM_002095 | 0.2223515 | 0.0282 | 2.2351043 | -3.691574 | 0.30968906 |
| TIMP2 | AL110197 | 0.1738254 | 0.0167 | 2.4376841 | -3.323888 | 0.30965308 |
| ABI2 | AF085867 | 0.2642384 | 0.0405 | 2.0786979 | -4.054966 | 0.30939631 |
| TIAL1 | NM_003252 | 0.2778201 | 0.0445 | 2.0367037 | -4.160148 | 0.3093527 |
| ZNF146 | NM_007145 | 0.279356 | 0.0451 | 2.0311033 | -4.135838 | 0.30884841 |
| GAPDHS | NM_014364 | 0.1842801 | 0.0188 | 2.4083908 | -3.305262 | 0.30874161 |
| CYP2E1 | J02625 | 0.136162 | 0.0102 | 2.6270112 | -2.893693 | 0.30865612 |
| GMDS | NM_001500 | 0.1989551 | 0.0224 | 2.328892 | -3.513962 | 0.30861719 |
| PAX7 | NM_002584 | 0.2297508 | 0.0302 | 2.2015733 | -3.82873 | 0.30804954 |
| RPE | AJ224326 | 0.2304283 | 0.0304 | 2.2143592 | -3.663474 | 0.30802907 |
| NOP16 | NM_016391 | 0.2662583 | 0.0409 | 2.0729463 | -4.091774 | 0.30792086 |
| SUOX | NM_000456 | 0.2400269 | 0.033 | 2.1682345 | -3.862092 | 0.30768987 |
| ATP10B | AB018258 | 0.2218508 | 0.028 | 2.2392246 | -3.68132 | 0.30731318 |
| SAV1 | AK023071 | 0.1149191 | 0.00716 | 2.7543022 | -2.591934 | 0.30698333 |
| MRPL2 | NM_015950 | 0.1503974 | 0.0126 | 2.5504448 | -3.047725 | 0.30653953 |
| USP14 | NM_005151 | 0.160142 | 0.014 | 2.5080693 | -3.164725 | 0.30639717 |
| PTPN23 | AB025194 | 0.2100976 | 0.0248 | 2.2839324 | -3.634733 | 0.30635124 |
| PADI3 | NM_016233 | 0.1756086 | 0.017 | 2.4365491 | -3.286952 | 0.30631739 |
| SETD3 | AK026680 | 0.2244411 | 0.0288 | 2.2301128 | -3.619108 | 0.30620838 |
| NKAP | AK026279 | 0.2763419 | 0.044 | 2.0423068 | -4.124561 | 0.30620101 |
| SREK1 | AL049309 | 0.2763419 | 0.0441 | 2.0413679 | -4.139157 | 0.30603777 |
| FER1L4 | AK026266 | 0.1834162 | 0.0185 | 2.3996511 | -3.413953 | 0.30579503 |
| PIGA | D11466 | 0.1789884 | 0.0177 | 2.4181945 | -3.339897 | 0.30560894 |
| FAM206A | NM_017832 | 0.1298985 | 0.0091 | 2.6726633 | -2.757468 | 0.3054467 |
| TCEAL8 | AK026349 | 0.1274252 | 0.00865 | 2.6895402 | -2.737552 | 0.30505795 |
| NT5C | NM_014595 | 0.1502516 | 0.0125 | 2.5471488 | -3.078312 | 0.30440272 |
| DIAPH1 | NM_005219 | 0.2370211 | 0.0322 | 2.1737817 | -3.887922 | 0.3042991 |
| SCAMP1 | NM_004866 | 0.2181298 | 0.0269 | 2.2490766 | -3.731724 | 0.30413507 |
| NDUFA4 | NM_002489 | 0.1128359 | 0.00686 | 2.7685602 | -2.548004 | 0.30408197 |
| ANAPC15 | NM_014042 | 0.2763419 | 0.0441 | 2.0417889 | -4.137191 | 0.30389446 |
| FUT4 | NM_002033 | 0.2268822 | 0.0295 | 2.2222362 | -3.633919 | 0.30365812 |
| CCDC167 | AF161383 | 0.1554696 | 0.0134 | 2.5246607 | -3.127562 | 0.30327196 |
| PQLC2 | NM_017765 | 0.2694865 | 0.0418 | 2.0651737 | -4.090818 | 0.30321322 |
| COPB2 | NM_004766 | 0.2636178 | 0.0402 | 2.0837647 | -4.050457 | 0.30317516 |
| ZFR | NM_016107 | 0.2678224 | 0.0413 | 2.0698447 | -4.085527 | 0.30312708 |
| LRP2 | NM_004525 | 0.257798 | 0.0386 | 2.1051959 | -3.924053 | 0.30273731 |
| C5orf24 | AF085880 | 0.2828834 | 0.0464 | 2.0206664 | -4.158817 | 0.30233303 |
| ERCC1 | NM_001983 | 0.081779 | 0.00358 | 2.992954 | -2.000899 | 0.30227265 |
| AP1M2 | NM_005498 | 0.2856754 | 0.0474 | 2.0139801 | -4.079474 | 0.30218052 |
| CCR10 | NM_016602 | 0.1989551 | 0.0223 | 2.335034 | -3.47114 | 0.30211011 |
| CEBPG | NM_001806 | 0.1123742 | 0.00679 | 2.7693466 | -2.563884 | 0.30175483 |
| PAIP1 | NM_006451 | 0.2360748 | 0.032 | 2.1845591 | -3.72593 | 0.30125381 |
| USP39 | NM_006590 | 0.2787867 | 0.0449 | 2.033222 | -4.154374 | 0.300908 |
| MED9 | NM_018019 | 0.2748566 | 0.0436 | 2.0540115 | -3.966819 | 0.30049385 |
| SLC10A3 | NM_019848 | 0.1954377 | 0.0214 | 2.3414254 | -3.539859 | 0.30045341 |
| SEPHS1 | NM_012247 | 0.2321878 | 0.0309 | 2.1974867 | -3.790902 | 0.30016063 |
| OXCT1 | NM_000436 | 0.2491961 | 0.0359 | 2.1318102 | -3.895587 | 0.30003539 |
| TNNC1 | NM_003280 | 0.1762363 | 0.0172 | 2.4354638 | -3.275911 | 0.2999111 |
| BLOC1S4 | NM_018366 | 0.1697769 | 0.0158 | 2.4614138 | -3.257147 | 0.29924621 |
| PTGES2 | AK024100 | 0.257798 | 0.0386 | 2.0990411 | -4.004979 | 0.29854673 |
| IBTK | AF235049 | 0.1943126 | 0.0212 | 2.3457102 | -3.530791 | 0.29851298 |
| COMMD1 | D85433 | 0.2464837 | 0.035 | 2.1444563 | -3.883566 | 0.29812292 |
| PLIN1 | NM_002666 | 0.2742267 | 0.0433 | 2.057093 | -3.96124 | 0.29740225 |
| LOC338667 | AF086259 | 0.2464837 | 0.035 | 2.1542069 | -3.760377 | 0.29668359 |
| THOC1 | NM_005131 | 0.2186316 | 0.0271 | 2.2506993 | -3.686621 | 0.29625534 |
| C2orf47 | AK026208 | 0.1839197 | 0.0186 | 2.3977854 | -3.407708 | 0.29607527 |
| ZFYVE21 | AK001921 | 0.1934196 | 0.0209 | 2.3496416 | -3.535777 | 0.29547915 |
| GDI2 | NM_001494 | 0.2634252 | 0.0401 | 2.0815972 | -4.040049 | 0.29532094 |
| ADPRHL2 | NM_017825 | 0.1512579 | 0.0128 | 2.5384227 | -3.098795 | 0.29529222 |
| ATP6V1B1 | NM_001692 | 0.266287 | 0.041 | 2.0859203 | -3.885663 | 0.29303226 |
| CENPT | AK023173 | 0.2464837 | 0.035 | 2.1426713 | -3.888742 | 0.29284554 |
| EPAS1 | NM_001430 | 0.2929067 | 0.0495 | 1.9919822 | -4.186278 | 0.29277864 |
| FAM204A | AK023250 | 0.2858989 | 0.0475 | 2.0093604 | -4.183338 | 0.29241344 |
| SNAPC5 | NM_006049 | 0.2696514 | 0.0419 | 2.0654553 | -4.064617 | 0.29222446 |
| OXLD1 | AY007126 | 0.2091832 | 0.0246 | 2.2871763 | -3.640932 | 0.29220664 |
| NUDT2 | NM_001161 | 0.1745098 | 0.0168 | 2.4354443 | -3.347256 | 0.29212178 |
| EPB41L4A-AS1 | AB002437 | 0.2685123 | 0.0415 | 2.0702836 | -4.07597 | 0.29211842 |
| SNX4 | NM_003794 | 0.2124475 | 0.0253 | 2.2816197 | -3.594229 | 0.29146436 |
| SMPD1 | NM_000543 | 0.1253368 | 0.00841 | 2.6965998 | -2.71068 | 0.29143818 |
| VBP1 | NM_003372 | 0.2660696 | 0.0408 | 2.0736892 | -4.091542 | 0.29040108 |
| CFTR | NM_000492 | 0.1667447 | 0.0152 | 2.4774142 | -3.210639 | 0.29006929 |
| ZNF592 | NM_014630 | 0.1369265 | 0.0103 | 2.6186974 | -2.903738 | 0.29006707 |
| PTPRZ1 | NM_002851 | 0.2464837 | 0.0351 | 2.1400696 | -3.910468 | 0.28993328 |
| CCL27 | NM_006664 | 0.1842801 | 0.0188 | 2.39481 | -3.401421 | 0.2898823 |
| AMBN | NM_016519 | 0.1866425 | 0.0194 | 2.389753 | -3.358835 | 0.28983145 |
| PDE6G | NM_002602 | 0.1789884 | 0.0177 | 2.4207718 | -3.32086 | 0.28850612 |
| IGF2BP1 | NM_006546 | 0.1760107 | 0.0171 | 2.4318156 | -3.332183 | 0.28841888 |
| PCGF1 | AL049296 | 0.2523608 | 0.0371 | 2.1191934 | -3.969493 | 0.28837979 |
| MBNL3 | NM_018388 | 0.2010691 | 0.0228 | 2.3150649 | -3.608106 | 0.28781548 |
| SLC30A9 | NM_006345 | 0.2688753 | 0.0416 | 2.0696397 | -4.011427 | 0.28780131 |
| SLC7A1 | AL050021 | 0.2845257 | 0.0471 | 2.0149654 | -4.128567 | 0.28752946 |
| STXBP1 | NM_003165 | 0.1789884 | 0.0177 | 2.4259957 | -3.269605 | 0.28752708 |
| TNPO1 | NM_002270 | 0.2872229 | 0.0479 | 2.0045211 | -4.208734 | 0.28708122 |
| C1orf54 | AK026874 | 0.2244411 | 0.0289 | 2.2196512 | -3.793316 | 0.28704696 |
| ITFG1 | AF212247 | 0.0759704 | 0.00313 | 3.0388338 | -1.874228 | 0.28703515 |
| C11orf71 | NM_019021 | 0.2500105 | 0.0364 | 2.1314042 | -3.87287 | 0.28648685 |
| CLPP | NM_006012 | 0.2347748 | 0.0317 | 2.1808905 | -3.871939 | 0.28605473 |
| FAM234B | AB040900 | 0.2002609 | 0.0227 | 2.3203611 | -3.580714 | 0.28603844 |
| GALT | NM_000155 | 0.2486828 | 0.0357 | 2.1323048 | -3.926339 | 0.28600075 |
| CD1B | NM_001764 | 0.1967942 | 0.0219 | 2.3389009 | -3.491993 | 0.28598884 |
| SUPV3L1 | NM_003171 | 0.1298985 | 0.00909 | 2.6652275 | -2.81442 | 0.28595818 |
| ERCC1 | S94539 | 0.2917491 | 0.0491 | 1.994713 | -4.198839 | 0.28590794 |
| DYNC2LI1 | NM_016008 | 0.2048893 | 0.0236 | 2.3032065 | -3.595705 | 0.28575242 |
| EPHB6 | NM_004445 | 0.2241679 | 0.0287 | 2.2271124 | -3.692725 | 0.28553361 |
| GPC1 | NM_002081 | 0.203751 | 0.0234 | 2.316172 | -3.52277 | 0.28524635 |
| RPA2 | NM_002946 | 0.2117172 | 0.0251 | 2.2906714 | -3.544936 | 0.2851233 |
| PNKD | AL080092 | 0.2301949 | 0.0303 | 2.2013306 | -3.815229 | 0.28487203 |
| SERF1A | AF073519 | 0.270097 | 0.042 | 2.062788 | -4.086127 | 0.28447174 |
| ZNF154 | U20648 | 0.2493265 | 0.036 | 2.13878 | -3.840031 | 0.2842591 |
| ALDOC | NM_005165 | 0.254994 | 0.0377 | 2.1093529 | -3.983337 | 0.28408405 |
| HTR3B | NM_006028 | 0.2249229 | 0.029 | 2.223438 | -3.728037 | 0.28328725 |
| CEP104 | NM_014704 | 0.2123544 | 0.0253 | 2.2739678 | -3.680683 | 0.28321218 |
| POLR1D | AF131855 | 0.2214248 | 0.0278 | 2.2388809 | -3.67096 | 0.2831742 |
| RNASE1 | NM_002933 | 0.278402 | 0.0447 | 2.0373184 | -4.12799 | 0.28293948 |
| XRN2 | NM_012255 | 0.2929067 | 0.0495 | 1.9905268 | -4.220172 | 0.28238476 |
| TGDS | NM_014305 | 0.2186316 | 0.0272 | 2.2486425 | -3.692399 | 0.28237519 |
| DOCK6 | AK022412 | 0.1943126 | 0.0212 | 2.3521017 | -3.438608 | 0.28170236 |
| ZBTB5 | NM_014872 | 0.2397153 | 0.0329 | 2.1670204 | -3.870497 | 0.28164149 |
| MAPKAPK5 | NM_003668 | 0.2456812 | 0.0344 | 2.1477235 | -3.933291 | 0.28127859 |
| PAPD5 | AF089897 | 0.2619585 | 0.0397 | 2.0864547 | -4.041284 | 0.28110083 |
| GOSR1 | NM_004871 | 0.2746695 | 0.0435 | 2.0470733 | -4.115647 | 0.28073584 |
| MRPS16 | NM_016065 | 0.1626794 | 0.0144 | 2.4942977 | -3.206853 | 0.28049626 |
| GNAI3 | NM_006496 | 0.2342629 | 0.0315 | 2.1834467 | -3.866852 | 0.28022769 |
| UGDH | NM_003359 | 0.2236067 | 0.0286 | 2.2270637 | -3.723069 | 0.28022277 |
| ZNF330 | NM_014487 | 0.1863506 | 0.0193 | 2.3855935 | -3.440939 | 0.27980516 |
| REV1 | NM_016316 | 0.251617 | 0.0369 | 2.12104 | -3.900388 | 0.27955095 |
| RAB38 | AF235022 | 0.1603612 | 0.014 | 2.5066201 | -3.146703 | 0.27846265 |
| MYO5A | U90942 | 0.2558899 | 0.0381 | 2.1040931 | -4.021215 | 0.27838332 |
| FILIP1 | AB033101 | 0.1357279 | 0.01 | 2.6493461 | -2.773074 | 0.27835631 |
| MKKS | NM_018848 | 0.2512732 | 0.0368 | 2.1234953 | -3.908258 | 0.2782656 |
| DDT | NM_001355 | 0.2079212 | 0.0243 | 2.2899708 | -3.649306 | 0.27806623 |
| BCOR | NM_017745 | 0.216106 | 0.0264 | 2.2592127 | -3.683622 | 0.27788893 |
| ATRX | NM_000489 | 0.2688125 | 0.0416 | 2.0668589 | -4.066066 | 0.27744115 |
| PBDC1 | NM_016500 | 0.204763 | 0.0236 | 2.3070053 | -3.571836 | 0.27663972 |
| GFRA1 | NM_005264 | 0.1103882 | 0.0066 | 2.7952951 | -2.470726 | 0.27645032 |
| GLI3 | NM_000168 | 0.2380332 | 0.0324 | 2.1740024 | -3.890817 | 0.27588439 |
| CDO1 | NM_001801 | 0.2336615 | 0.0313 | 2.1921669 | -3.775686 | 0.27582278 |
| LIFR | NM_002310 | 0.282682 | 0.0463 | 2.0215 | -4.146418 | 0.27559203 |
| FXYD3 | NM_005971 | 0.2287411 | 0.03 | 2.2076019 | -3.775409 | 0.27553104 |
| SNW1 | NM_012245 | 0.1760683 | 0.0171 | 2.4264775 | -3.370394 | 0.27533186 |
| ZNF41 | X60155 | 0.2832417 | 0.0465 | 2.0307374 | -3.94319 | 0.2751207 |
| LOXL1 | NM_005576 | 0.2224184 | 0.0282 | 2.2391084 | -3.601664 | 0.27508677 |
| HGS | NM_004712 | 0.2200167 | 0.0275 | 2.2436679 | -3.688385 | 0.27501024 |
| TMEM181 | AK023059 | 0.2464837 | 0.0351 | 2.1543528 | -3.679637 | 0.27483 |
| HPN | NM_002151 | 0.2508461 | 0.0366 | 2.1218521 | -3.983645 | 0.27470858 |
| CYTH3 | NM_004227 | 0.2186316 | 0.0272 | 2.2523922 | -3.625282 | 0.27460205 |
| POLR2K | NM_005034 | 0.2098625 | 0.0247 | 2.2886613 | -3.596651 | 0.27359048 |
| RNH1 | NM_002939 | 0.2787867 | 0.0449 | 2.0370416 | -4.084972 | 0.27357332 |
| HAGH | NM_005326 | 0.2364436 | 0.0321 | 2.1758208 | -3.882012 | 0.27309886 |
| RALA | NM_005402 | 0.2262344 | 0.0293 | 2.2196451 | -3.73393 | 0.2730656 |
| SRSF4 | NM_005626 | 0.2842376 | 0.047 | 2.01367 | -4.177714 | 0.27297141 |
| AP1S1 | NM_001283 | 0.1762363 | 0.0172 | 2.4332166 | -3.254909 | 0.27254067 |
| DNAJC2 | X98260 | 0.2312419 | 0.0306 | 2.1981092 | -3.809077 | 0.27223847 |
| ATP12A | NM_001676 | 0.2264051 | 0.0293 | 2.2191613 | -3.707843 | 0.27139133 |
| CLN5 | NM_006493 | 0.2464837 | 0.0349 | 2.141745 | -3.933731 | 0.27135033 |
| HMGXB3 | D83778 | 0.2301949 | 0.0303 | 2.2033934 | -3.816391 | 0.27104141 |
| BLM | NM_000057 | 0.2895379 | 0.0485 | 2.0063303 | -4.0879 | 0.26996937 |
| ZNF358 | NM_018083 | 0.2464837 | 0.0349 | 2.1417205 | -3.908929 | 0.26909033 |
| ISCA1 | AF038186 | 0.1827372 | 0.0184 | 2.4059834 | -3.352472 | 0.26875805 |
| DOCK9 | AB028981 | 0.2512732 | 0.0368 | 2.1203691 | -3.947583 | 0.2673755 |
| PHC1 | U89277 | 0.1352168 | 0.00998 | 2.6356201 | -2.84281 | 0.26674026 |
| PPP5C | NM_006247 | 0.1123742 | 0.0068 | 2.7816299 | -2.501691 | 0.26630442 |
| USP1 | NM_003368 | 0.2880562 | 0.0481 | 2.0043093 | -4.190156 | 0.26588245 |
| NPRL2 | NM_006545 | 0.2147504 | 0.0259 | 2.2653697 | -3.686614 | 0.26516096 |
| BAP1 | NM_004656 | 0.2464837 | 0.0351 | 2.1422315 | -3.888735 | 0.26459919 |
| RPS6KA4 | NM_003942 | 0.2479036 | 0.0355 | 2.1335724 | -3.952553 | 0.26456173 |
| CUL2 | NM_003591 | 0.2139928 | 0.0257 | 2.2729407 | -3.651117 | 0.26448729 |
| PTBP2 | AF176085 | 0.2308335 | 0.0305 | 2.2031844 | -3.76732 | 0.26443996 |
| L1TD1 | NM_019079 | 0.2311159 | 0.0305 | 2.1967367 | -3.839376 | 0.26367032 |
| AP1S2 | NM_003916 | 0.2778201 | 0.0445 | 2.0368008 | -4.148849 | 0.26356353 |
| COMMD3 | NM_012071 | 0.2576088 | 0.0385 | 2.1009721 | -4.011439 | 0.26333718 |
| UNC13B | NM_006377 | 0.1646272 | 0.0149 | 2.4856341 | -3.203837 | 0.26318091 |
| ABCC10 | AK000002 | 0.2498502 | 0.0363 | 2.1278727 | -3.917208 | 0.26246824 |
| PUM3 | NM_014878 | 0.1667447 | 0.0152 | 2.4748008 | -3.249639 | 0.26228986 |
| PECR | NM_018441 | 0.2130801 | 0.0255 | 2.2730311 | -3.6444 | 0.26153065 |
| LRRC8D | NM_018103 | 0.282682 | 0.0463 | 2.0199273 | -4.166158 | 0.26122477 |
| TRPT1 | AK026364 | 0.216106 | 0.0263 | 2.2648727 | -3.630005 | 0.2607833 |
| UBAP2 | NM_018449 | 0.2278065 | 0.0297 | 2.2103835 | -3.79419 | 0.26074864 |
| FSTL1 | NM_007085 | 0.257921 | 0.0388 | 2.1007554 | -4.002124 | 0.26058962 |
| MOCOS | NM_017947 | 0.2342629 | 0.0315 | 2.1836454 | -3.864516 | 0.26053071 |
| TIMM10B | NM_012192 | 0.1838669 | 0.0186 | 2.3998414 | -3.390642 | 0.26017701 |
| MTFR1 | NM_014637 | 0.2438013 | 0.0341 | 2.1532823 | -3.883952 | 0.25939548 |
| PDE8B | AF079529 | 0.2579868 | 0.0389 | 2.1054617 | -3.833237 | 0.259109 |
| HAUS7 | NM_017518 | 0.1480421 | 0.0122 | 2.5645903 | -2.99336 | 0.25876215 |
| COL16A1 | M92642 | 0.188028 | 0.0197 | 2.3818909 | -3.400413 | 0.25844815 |
| FAM189B | NM_006589 | 0.2690814 | 0.0417 | 2.0665695 | -4.076803 | 0.25818055 |
| PATZ1 | NM_014323 | 0.2531597 | 0.0373 | 2.1142307 | -3.974925 | 0.25800597 |
| GARS | NM_002047 | 0.2737694 | 0.0432 | 2.0533819 | -4.094153 | 0.25749159 |
| NAXD | NM_018210 | 0.1352168 | 0.00998 | 2.6355151 | -2.853812 | 0.25700054 |
| CLDN10 | NM_006984 | 0.2317261 | 0.0307 | 2.1986141 | -3.777986 | 0.25684382 |
| RNF113A | NM_006978 | 0.1184467 | 0.00763 | 2.7299554 | -2.640972 | 0.25638808 |
| ARF6 | NM_001663 | 0.1957791 | 0.0215 | 2.3394546 | -3.534042 | 0.25604834 |
| POLR1A | NM_015425 | 0.2547875 | 0.0377 | 2.1158346 | -3.888898 | 0.25601699 |
| CENPB | X05299 | 0.2842376 | 0.047 | 2.0146754 | -4.146975 | 0.25594784 |
| RAD51D | NM_002878 | 0.1740706 | 0.0167 | 2.451107 | -3.202949 | 0.25518729 |
| NFKBIL1 | NM_005007 | 0.2101009 | 0.0248 | 2.2848523 | -3.60637 | 0.25445175 |
| HNRNPD | NM_002138 | 0.1543388 | 0.0132 | 2.5320848 | -3.099294 | 0.25415718 |
| NPY1R | NM_000909 | 0.2070505 | 0.024 | 2.3067042 | -3.539837 | 0.25407225 |
| FDFT1 | NM_004462 | 0.1626794 | 0.0145 | 2.5017926 | -3.120136 | 0.25367088 |
| DDX28 | NM_018380 | 0.203751 | 0.0234 | 2.3072402 | -3.588045 | 0.25322086 |
| SS18L2 | NM_016305 | 0.2837498 | 0.0467 | 2.0183555 | -4.123364 | 0.25320197 |
| TRAPPC13 | AK023673 | 0.2745031 | 0.0435 | 2.0545097 | -4.031344 | 0.2527778 |
| LYPD3 | NM_014400 | 0.287999 | 0.0481 | 2.0041136 | -4.192972 | 0.25142551 |
| DHX32 | NM_018180 | 0.216106 | 0.0263 | 2.2606662 | -3.703098 | 0.25097048 |
| H2AFY2 | NM_018649 | 0.282682 | 0.0461 | 2.0287933 | -4.031664 | 0.25047993 |
| KCTD20 | AL049282 | 0.1857561 | 0.0191 | 2.3862613 | -3.452422 | 0.25045713 |
| TCF12 | NM_003205 | 0.2664102 | 0.041 | 2.0722517 | -4.070315 | 0.25012293 |
| SAGE1 | NM_018666 | 0.2292518 | 0.0301 | 2.2080132 | -3.770107 | 0.25007454 |
| PPM1H | AB032983 | 0.2301148 | 0.0303 | 2.2165867 | -3.625261 | 0.24928562 |
| ZAK | NM_016653 | 0.2636624 | 0.0403 | 2.0832135 | -4.013244 | 0.24908165 |
| ERCC4 | NM_005236 | 0.2889643 | 0.0484 | 2.0061861 | -4.121681 | 0.24795095 |
| PRKRA | NM_003690 | 0.2258607 | 0.0292 | 2.2148418 | -3.766879 | 0.24792159 |
| FLNC | NM_001458 | 0.2787014 | 0.0448 | 2.0381835 | -4.052613 | 0.24781094 |
| CSPG4 | NM_001897 | 0.1967942 | 0.0218 | 2.336982 | -3.498856 | 0.24742551 |
| FARS2 | NM_006567 | 0.1845572 | 0.0189 | 2.3940553 | -3.365714 | 0.2461997 |
| TRIP10 | AJ000414 | 0.1715931 | 0.0162 | 2.4588356 | -3.200019 | 0.2456115 |
| IGFBP6 | M62402 | 0.2411514 | 0.0334 | 2.1616274 | -3.881072 | 0.24414674 |
| MMP1 | NM_002421 | 0.2710608 | 0.0424 | 2.0608748 | -4.043873 | 0.24386849 |
| BORA | AK026277 | 0.0719307 | 0.00282 | 3.0807059 | -1.757303 | 0.24371176 |
| DDB2 | NM_000107 | 0.2496392 | 0.0361 | 2.1280654 | -3.933665 | 0.24206048 |
| NPTX1 | NM_002522 | 0.216106 | 0.0264 | 2.2577424 | -3.677611 | 0.24138087 |
| PMP22 | NM_000304 | 0.1868836 | 0.0195 | 2.3786176 | -3.459152 | 0.24098018 |
| LSR | NM_015925 | 0.2234789 | 0.0286 | 2.227624 | -3.72195 | 0.2406586 |
| SPC25 | AF225416 | 0.2234789 | 0.0286 | 2.2271237 | -3.759607 | 0.24065044 |
| TCP11 | NM_018679 | 0.2222798 | 0.0281 | 2.2377078 | -3.669966 | 0.2399721 |
| ZNF853 | AL133055 | 0.2836274 | 0.0467 | 2.024614 | -4.068229 | 0.23922503 |
| NGEF | AK025225 | 0.2309291 | 0.0305 | 2.2013463 | -3.773388 | 0.2389211 |
| UTS2 | NM_006786 | 0.2732799 | 0.043 | 2.0530408 | -4.090013 | 0.23887153 |
| NIF3L1 | AF182416 | 0.249277 | 0.036 | 2.133559 | -3.873727 | 0.23837166 |
| RUSC1 | NM_014328 | 0.2464837 | 0.0349 | 2.1431219 | -3.937616 | 0.23771617 |
| DHRS11 | AK026196 | 0.2479036 | 0.0355 | 2.1486287 | -3.770254 | 0.23728368 |
| TESPA1 | NM_014796 | 0.2002609 | 0.0227 | 2.3213454 | -3.555819 | 0.23657669 |
| RNF115 | AL079314 | 0.2577555 | 0.0385 | 2.1034466 | -3.960994 | 0.23583515 |
| SOX9 | NM_000346 | 0.1903387 | 0.0204 | 2.3608809 | -3.487971 | 0.23552873 |
| RBM19 | NM_014852 | 0.2758969 | 0.0439 | 2.0444899 | -4.118241 | 0.2349935 |
| COMMD9 | NM_014186 | 0.2694432 | 0.0418 | 2.0683834 | -4.01287 | 0.23493514 |
| CENPA | NM_001809 | 0.2859898 | 0.0476 | 2.015951 | -4.052214 | 0.2343838 |
| KIF9 | AF311212 | 0.2218047 | 0.028 | 2.238038 | -3.711218 | 0.23434836 |
| SCAMP2 | NM_005697 | 0.2508248 | 0.0366 | 2.1243382 | -3.960833 | 0.23377331 |
| CDK6 | NM_001259 | 0.1719556 | 0.0162 | 2.4527343 | -3.252923 | 0.23311646 |
| MCTS1 | NM_014060 | 0.2229331 | 0.0285 | 2.2335751 | -3.69106 | 0.23298465 |
| ETHE1 | NM_014297 | 0.2567023 | 0.0382 | 2.105627 | -3.959667 | 0.23163909 |
| PRTG | AK023283 | 0.2747491 | 0.0436 | 2.0510769 | -4.069754 | 0.23158658 |
| FAM13A | NM_014883 | 0.2420344 | 0.0336 | 2.1575498 | -3.90283 | 0.23130252 |
| EXOSC9 | NM_005033 | 0.274267 | 0.0433 | 2.0490488 | -4.123588 | 0.23127044 |
| NXT1 | NM_013248 | 0.2487077 | 0.0358 | 2.1367469 | -3.894567 | 0.22959954 |
| ZHX1 | NM_007222 | 0.2056573 | 0.0238 | 2.3054016 | -3.548461 | 0.22508248 |
| RAB23 | NM_016277 | 0.256048 | 0.0381 | 2.1076946 | -3.978996 | 0.22459389 |
| UGT2B10 | NM_001075 | 0.2226485 | 0.0283 | 2.2336714 | -3.706857 | 0.22432875 |
| VPS45 | NM_007259 | 0.2636624 | 0.0403 | 2.0830419 | -4.002366 | 0.22228831 |
| TEFM | AK026382 | 0.2909544 | 0.0489 | 1.9990762 | -4.156434 | 0.22198895 |
| NDUFAF5 | AK025977 | 0.2679891 | 0.0413 | 2.0721769 | -4.036143 | 0.22185679 |
| POU3F1 | NM_002699 | 0.2342629 | 0.0316 | 2.1987269 | -3.676481 | 0.22180219 |
| PAXIP1 | U80735 | 0.282682 | 0.0461 | 2.0231536 | -4.130381 | 0.22175956 |
| CCAR1 | AK001701 | 0.2607169 | 0.0395 | 2.090438 | -4.041642 | 0.22091612 |
| PCK1 | NM_002591 | 0.2225464 | 0.0282 | 2.2354613 | -3.724655 | 0.22014492 |
| SLC12A2 | AK025062 | 0.1965399 | 0.0217 | 2.3500627 | -3.411841 | 0.21875709 |
| RPL32P3 | AL117606 | 0.1756985 | 0.017 | 2.4328318 | -3.320003 | 0.2183434 |
| ACR | NM_001097 | 0.2745031 | 0.0435 | 2.0499132 | -4.091462 | 0.21493317 |
| FANCF | AF181994 | 0.1737714 | 0.0166 | 2.4467096 | -3.275484 | 0.21486026 |
| CEP170 | NM_014812 | 0.2303532 | 0.0303 | 2.2032271 | -3.756077 | 0.21317102 |
| CDX2 | NM_001265 | 0.2732799 | 0.043 | 2.0581529 | -4.052715 | 0.21081571 |
| SRSF3 | NM_003017 | 0.2710608 | 0.0424 | 2.0638028 | -4.002878 | 0.21018844 |
| CRTC3 | AK025521 | 0.2438013 | 0.0341 | 2.1552654 | -3.86268 | 0.20532837 |
| RHBDD1 | AK026955 | 0.2620335 | 0.0398 | 2.0960394 | -3.869527 | 0.20214227 |
| PNPLA4 | U03886 | 0.2913871 | 0.049 | 2.0004565 | -4.146983 | 0.19954628 |
| GK2 | X78712 | 0.2529714 | 0.0372 | 2.1188393 | -3.930778 | 0.19899472 |
| GFER | NM_005262 | 0.1870423 | 0.0196 | 2.3751097 | -3.481261 | 0.1956898 |
| FAM174B | AL110139 | 0.2499157 | 0.0363 | 2.1260535 | -3.923184 | 0.18857073 |
| CACNA1C | AL359562 | 0.2569771 | 0.0383 | 2.1033954 | -3.992736 | 0.1814083 |
| PMS2P4 | D38438 | 0.2342629 | 0.0315 | 2.1888615 | -3.807874 | 0.1716034 |
| ARHGAP10 | AK024549 | 0.2904551 | 0.0487 | -2.0013798 | -4.119412 | -0.17249684 |
| CPXM1 | NM_019609 | 0.2842296 | 0.0469 | -2.017499 | -4.108413 | -0.17793117 |
| PDE5A | NM_001083 | 0.2139928 | 0.0257 | -2.2835057 | -3.56966 | -0.19106448 |
| SPTLC1 | AF116613 | 0.2850554 | 0.0473 | -2.0171816 | -4.086063 | -0.19151769 |
| LOC101928565 | AF085892 | 0.2459848 | 0.0345 | -2.1518213 | -3.806338 | -0.19396196 |
| MAS1 | NM_002377 | 0.2636624 | 0.0402 | -2.0919915 | -3.940293 | -0.19584641 |
| N4BP2 | AB037834 | 0.2759596 | 0.0439 | -2.0492935 | -4.028637 | -0.19670008 |
| COX11 | NM_004375 | 0.2833012 | 0.0466 | -2.0232419 | -4.104391 | -0.2041307 |
| IL36G | NM_019618 | 0.2538409 | 0.0375 | -2.1158523 | -3.936463 | -0.20512536 |
| FXYD5 | NM_014164 | 0.2620335 | 0.0398 | -2.0962594 | -3.887207 | -0.21141781 |
| NEUROG1 | NM_006161 | 0.2883803 | 0.0483 | -2.0032223 | -4.192147 | -0.21491522 |
| SNTG1 | NM_018967 | 0.2500105 | 0.0364 | -2.136186 | -3.828212 | -0.21536947 |
| EPB41L4B | NM_019114 | 0.212861 | 0.0254 | -2.2796508 | -3.598892 | -0.21647488 |
| LCT | NM_002299 | 0.2803568 | 0.0453 | -2.0439976 | -3.939096 | -0.22011496 |
| EFHC1 | NM_018100 | 0.2526582 | 0.0371 | -2.1225978 | -3.841921 | -0.22032219 |
| ALDH7A1 | NM_001182 | 0.1998041 | 0.0226 | -2.3270803 | -3.503443 | -0.22482612 |
| APH1A | NM_016022 | 0.1892138 | 0.02 | -2.3769426 | -3.397983 | -0.22538363 |
| SMURF1 | AB046845 | 0.2588543 | 0.0391 | -2.0972002 | -3.986827 | -0.22586698 |
| PTGER1 | NM_000955 | 0.2647036 | 0.0406 | -2.0885893 | -3.862732 | -0.22605392 |
| RC3H2 | NM_018835 | 0.2736918 | 0.0431 | -2.0603259 | -3.934797 | -0.22681211 |
| IFITM1 | NM_003641 | 0.282682 | 0.0462 | -2.0301338 | -4.041075 | -0.22862933 |
| PLXNB3 | AF149019 | 0.2432755 | 0.0339 | -2.1702898 | -3.745178 | -0.23018226 |
| NAT8B | NM_016347 | 0.282682 | 0.0461 | -2.0284648 | -4.032255 | -0.23186581 |
| NCAPD2 | NM_014865 | 0.2268907 | 0.0295 | -2.2223008 | -3.666726 | -0.23216401 |
| LAMB3 | NM_000228 | 0.2500105 | 0.0364 | -2.1311344 | -3.859349 | -0.23264819 |
| MYH4 | AF111783 | 0.2825576 | 0.046 | -2.0312904 | -4.006313 | -0.23408038 |
| CHUK | NM_001278 | 0.2575556 | 0.0384 | -2.1019744 | -4.018345 | -0.23757004 |
| MED13 | NM_005121 | 0.2840863 | 0.0468 | -2.0140502 | -4.193411 | -0.23818994 |
| ZMAT4 | AK023904 | 0.2198117 | 0.0275 | -2.2474467 | -3.650024 | -0.24067362 |
| TRIM17 | NM_016102 | 0.257798 | 0.0386 | -2.1050092 | -3.892778 | -0.24077183 |
| DUSP2 | NM_004418 | 0.2156903 | 0.0261 | -2.2683993 | -3.634781 | -0.24150005 |
| ZNF140 | NM_003440 | 0.2685123 | 0.0415 | -2.0740366 | -4.023167 | -0.24150571 |
| ZBTB39 | NM_014830 | 0.2606265 | 0.0394 | -2.0949506 | -3.975008 | -0.24292209 |
| CD40LG | NM_000074 | 0.2735839 | 0.0431 | -2.0654463 | -3.859909 | -0.24347943 |
| EFR3B | AF131834 | 0.2771369 | 0.0443 | -2.0470511 | -4.014424 | -0.24479479 |
| MLLT11 | NM_006818 | 0.1334078 | 0.00962 | -2.6693364 | -2.730718 | -0.24510063 |
| AMELY | NM_001143 | 0.1870423 | 0.0196 | -2.3888279 | -3.346855 | -0.24519822 |
| ARNT | NM_001668 | 0.278402 | 0.0447 | -2.0383155 | -4.099232 | -0.24693137 |
| CASP3 | NM_004346 | 0.2777453 | 0.0444 | -2.0457844 | -4.044935 | -0.24775964 |
| HAX1 | NM_006118 | 0.1817112 | 0.0181 | -2.4169951 | -3.259972 | -0.24786666 |
| LAG3 | NM_002286 | 0.1407324 | 0.011 | -2.6098634 | -2.881025 | -0.24803474 |
| SNCB | NM_003085 | 0.2410863 | 0.0333 | -2.1770753 | -3.716796 | -0.2491763 |
| ADRA1D | NM_000678 | 0.2706263 | 0.0422 | -2.0685425 | -4.003112 | -0.24967897 |
| FLRT2 | NM_013231 | 0.257921 | 0.0388 | -2.0997525 | -3.969897 | -0.25164763 |
| CCP110 | NM_014711 | 0.286151 | 0.0477 | -2.014404 | -4.038579 | -0.25212216 |
| CD248 | NM_020404 | 0.273867 | 0.0432 | -2.0563395 | -4.01474 | -0.25278698 |
| NPPB | NM_002521 | 0.282682 | 0.0463 | -2.0358007 | -3.835915 | -0.25298641 |
| POU6F2 | NM_007252 | 0.2662125 | 0.0409 | -2.0820342 | -3.897021 | -0.253606 |
| FAP | NM_004460 | 0.2777453 | 0.0445 | -2.0477113 | -3.936819 | -0.25387352 |
| GLP2R | NM_004246 | 0.271293 | 0.0425 | -2.0654002 | -3.963727 | -0.25399519 |
| ANKS1B | NM_020140 | 0.1612765 | 0.0141 | -2.5138345 | -3.081547 | -0.25437908 |
| TRPV2 | NM_016113 | 0.2464837 | 0.0348 | -2.1502437 | -3.823134 | -0.25649269 |
| LCE2B | NM_014357 | 0.2472731 | 0.0353 | -2.1504136 | -3.749159 | -0.25774364 |
| GHRHR | NM_000823 | 0.2808959 | 0.0455 | -2.0428478 | -3.940469 | -0.25797935 |
| RIT2 | NM_002930 | 0.1745098 | 0.0168 | -2.4550194 | -3.195138 | -0.258701 |
| SPACA9 | NM_018956 | 0.2066046 | 0.0239 | -2.3045814 | -3.490037 | -0.25891788 |
| CAPZA1 | NM_006135 | 0.1646272 | 0.0148 | -2.4997315 | -3.086786 | -0.25932963 |
| PLXNA4 | AB046770 | 0.1715555 | 0.0161 | -2.4567038 | -3.243361 | -0.26007581 |
| LOC149401 | AL137339 | 0.1756086 | 0.017 | -2.4477274 | -3.196736 | -0.26121304 |
| KMT2E | NM_018682 | 0.2842376 | 0.0469 | -2.0131865 | -4.196101 | -0.26139757 |
| RNF103 | NM_005667 | 0.2139928 | 0.0257 | -2.274917 | -3.609115 | -0.26148592 |
| BECN1 | AJ227885 | 0.2859152 | 0.0476 | -2.0144202 | -4.119123 | -0.26163471 |
| TRIM35 | AB029021 | 0.2828834 | 0.0464 | -2.0317869 | -3.94136 | -0.26189541 |
| ZNF614 | AK023547 | 0.2459695 | 0.0345 | -2.1604334 | -3.765604 | -0.2619718 |
| UHMK1 | AL137257 | 0.2642119 | 0.0405 | -2.0822765 | -4.000904 | -0.26438889 |
| CNPPD1 | NM_015680 | 0.2480109 | 0.0356 | -2.1361991 | -3.91432 | -0.26527781 |
| MYL4 | X52005 | 0.2328881 | 0.0311 | -2.2077479 | -3.641377 | -0.26563443 |
| AGO2 | AL050151 | 0.2017761 | 0.0229 | -2.3225497 | -3.534135 | -0.26570058 |
| RMND5A | AL050139 | 0.2856754 | 0.0474 | -2.0103659 | -4.155871 | -0.26651425 |
| STIM2 | AB040915 | 0.2867446 | 0.0478 | -2.0155918 | -4.032718 | -0.26701665 |
| KIAA0825 | AB020632 | 0.1866425 | 0.0194 | -2.4008827 | -3.265415 | -0.26730761 |
| PDE1C | NM_005020 | 0.1757233 | 0.0171 | -2.4438043 | -3.231557 | -0.26779479 |
| ASB4 | NM_016116 | 0.2464837 | 0.0351 | -2.1539482 | -3.777089 | -0.26786861 |
| TNFRSF10D | NM_003840 | 0.2317261 | 0.0307 | -2.2051626 | -3.683766 | -0.26838688 |
| NEU1 | NM_000434 | 0.2321878 | 0.0309 | -2.201289 | -3.752038 | -0.26911084 |
| SPPL3 | AL110147 | 0.1740706 | 0.0167 | -2.4440546 | -3.245291 | -0.26913594 |
| ST7 | NM_018412 | 0.285783 | 0.0475 | -2.0234898 | -3.993904 | -0.2692934 |
| CCDC85C | AK026211 | 0.1866856 | 0.0195 | -2.4028937 | -3.247571 | -0.2695687 |
| ROM1 | NM_000327 | 0.1897973 | 0.0203 | -2.3767202 | -3.328026 | -0.27032836 |
| SGCA | NM_000023 | 0.1989551 | 0.0224 | -2.3303196 | -3.483545 | -0.27129149 |
| PTPRQ | AF169351 | 0.2314812 | 0.0307 | -2.2029149 | -3.763236 | -0.27178096 |
| MBP | NM_002385 | 0.0899319 | 0.00442 | -2.9362918 | -2.109928 | -0.27221924 |
| PCDHGB8P | AF152529 | 0.2186316 | 0.0272 | -2.26396 | -3.517347 | -0.27260069 |
| LOC100508226///MAGI1-IT1///MAGI1 | AK022064 | 0.2558741 | 0.038 | -2.1335525 | -3.718693 | -0.27268608 |
| ABHD17B | NM_016014 | 0.2419447 | 0.0336 | -2.1664418 | -3.818576 | -0.27375315 |
| SH3BP5-AS1 | AL133111 | 0.2662125 | 0.0409 | -2.0875058 | -3.863377 | -0.27392513 |
| CABP2 | NM_016366 | 0.2714443 | 0.0426 | -2.0753797 | -3.841311 | -0.27509292 |
| ERVW-1 | NM_014590 | 0.2380332 | 0.0324 | -2.1787541 | -3.771031 | -0.27593116 |
| PARP11 | NM_020367 | 0.2464837 | 0.0351 | -2.1472056 | -3.857483 | -0.27630414 |
| CLEC5A | AF139768 | 0.2298892 | 0.0302 | -2.2141213 | -3.695347 | -0.27681645 |
| FNBP1 | AL049935 | 0.2464471 | 0.0347 | -2.1443519 | -3.928648 | -0.27692055 |
| SOS1 | L13857 | 0.2135865 | 0.0256 | -2.2727515 | -3.676222 | -0.27777762 |
| EPYC | NM_004950 | 0.1216492 | 0.008 | -2.7240231 | -2.647964 | -0.2791096 |
| RAB36 | NM_004914 | 0.257798 | 0.0387 | -2.0990741 | -4.000983 | -0.27930947 |
| KTI12///TXNDC12 | L31901 | 0.0791609 | 0.00338 | -3.0319149 | -1.866343 | -0.27938933 |
| RAB6A | AF052130 | 0.2850305 | 0.0472 | -2.0144894 | -4.164739 | -0.27983599 |
| AQP7 | NM_001170 | 0.2492848 | 0.036 | -2.1357332 | -3.865558 | -0.28115839 |
| SYT5 | NM_003180 | 0.2179811 | 0.0268 | -2.2636396 | -3.53554 | -0.28168706 |
| MCOLN1 | NM_020533 | 0.2191653 | 0.0274 | -2.244416 | -3.702473 | -0.28234356 |
| N4BP2L1 | U57962 | 0.2411486 | 0.0333 | -2.1622541 | -3.890536 | -0.2825074 |
| UPF1 | NM_002911 | 0.2420344 | 0.0336 | -2.1613144 | -3.874481 | -0.2827198 |
| CYFIP2 | AF132197 | 0.2488785 | 0.0358 | -2.1379673 | -3.846432 | -0.2834618 |
| EIF4A1 | U79273 | 0.2622837 | 0.0399 | -2.098035 | -3.863689 | -0.28371439 |
| PCDH18 | AB046782 | 0.0766741 | 0.00321 | -3.0322568 | -1.900699 | -0.28409849 |
| KPNA1 | NM_002264 | 0.2787867 | 0.0449 | -2.0354879 | -4.13011 | -0.28501361 |
| TNXB | Y17866 | 0.1625625 | 0.0144 | -2.5106557 | -3.111373 | -0.28516592 |
| TEX264 | NM_015926 | 0.2395561 | 0.0328 | -2.1774434 | -3.736725 | -0.28549899 |
| ARHGAP42 | AF143327 | 0.1737714 | 0.0166 | -2.4602469 | -3.157859 | -0.28572184 |
| ABCC5 | AL359600 | 0.1852395 | 0.019 | -2.3957666 | -3.371192 | -0.28632707 |
| MCM10 | NM_018518 | 0.2227307 | 0.0283 | -2.2280079 | -3.751679 | -0.28701144 |
| ANKMY1 | NM_016552 | 0.2840863 | 0.0468 | -2.0211237 | -4.093494 | -0.28712151 |
| SRSF5 | NM_006925 | 0.2488785 | 0.0359 | -2.1370091 | -3.877935 | -0.28778518 |
| DIS3L | AF147431 | 0.2483363 | 0.0357 | -2.1518283 | -3.706012 | -0.28784452 |
| LOC100287576///DGCR5 | U84528 | 0.2848445 | 0.0472 | -2.026982 | -3.947891 | -0.28853268 |
| MYOD1 | NM_002478 | 0.2407612 | 0.0332 | -2.1696047 | -3.770877 | -0.28902074 |
| CLPS | NM_001832 | 0.1502516 | 0.0125 | -2.5711538 | -2.924485 | -0.28905337 |
| FGF22 | AB021925 | 0.2625427 | 0.0399 | -2.1018161 | -3.835167 | -0.28991832 |
| SOBP | NM_018013 | 0.2234789 | 0.0286 | -2.240972 | -3.611859 | -0.29302846 |
| PRMT3 | AF059531 | 0.228124 | 0.0298 | -2.2078728 | -3.802155 | -0.29308063 |
| APC | S67789 | 0.1328676 | 0.00953 | -2.6690744 | -2.717393 | -0.2932826 |
| HOXC8 | X99680 | 0.238555 | 0.0326 | -2.1778232 | -3.785003 | -0.29357903 |
| ST3GAL1 | NM_003033 | 0.282682 | 0.0463 | -2.0236849 | -4.122711 | -0.29364425 |
| RAPGEF3 | U79275 | 0.2090025 | 0.0245 | -2.3052308 | -3.454875 | -0.29369045 |
| SUDS3 | AK024460 | 0.191213 | 0.0205 | -2.3682939 | -3.389881 | -0.29415369 |
| ADAMTS6 | NM_014273 | 0.2842707 | 0.047 | -2.0206685 | -4.076506 | -0.29524449 |
| CLDN5 | NM_003277 | 0.2205214 | 0.0276 | -2.2564519 | -3.549181 | -0.29534221 |
| ANGPTL7 | Y16132 | 0.1359438 | 0.0101 | -2.6389773 | -2.791175 | -0.29573212 |
| AKT1 | NM_005163 | 0.2577555 | 0.0385 | -2.1002791 | -4.000702 | -0.29579429 |
| GABRB2 | NM_000813 | 0.2752526 | 0.0437 | -2.0475831 | -4.082058 | -0.29617096 |
| KCNK7 | NM_005714 | 0.2463376 | 0.0347 | -2.174141 | -3.666073 | -0.29643921 |
| FAM76A | AL035307 | 0.1967942 | 0.0219 | -2.3482959 | -3.385595 | -0.29774209 |
| KIF3C | NM_002254 | 0.257921 | 0.0388 | -2.1064314 | -3.916862 | -0.2988985 |
| CCL2 | NM_002982 | 0.2875636 | 0.048 | -2.0090208 | -4.157561 | -0.30013378 |
| COL11A2 | J04974 | 0.1431328 | 0.0113 | -2.6045124 | -2.846345 | -0.30153012 |
| FMNL1 | AJ008121 | 0.1967942 | 0.0218 | -2.3448036 | -3.375324 | -0.30176261 |
| USP48 | NM_018391 | 0.1292596 | 0.00893 | -2.6804058 | -2.717857 | -0.30211283 |
| NGFR | NM_002507 | 0.2842376 | 0.047 | -2.0261842 | -3.951116 | -0.30228455 |
| KIAA1033 | AL137753 | 0.2385949 | 0.0326 | -2.1759616 | -3.841104 | -0.30233722 |
| MIATNB | AK026502 | 0.1695325 | 0.0157 | -2.4840716 | -3.12228 | -0.30243584 |
| AGTR2 | NM_000686 | 0.1936893 | 0.0209 | -2.351578 | -3.506313 | -0.30266957 |
| TRAF3IP1 | AF230877 | 0.2410863 | 0.0333 | -2.1700748 | -3.783861 | -0.30351108 |
| HOXC13 | NM_017410 | 0.2056573 | 0.0238 | -2.3023761 | -3.571048 | -0.30361536 |
| NRXN1 | AB035356 | 0.257921 | 0.0388 | -2.1159827 | -3.769956 | -0.3039547 |
| UBL3 | NM_007106 | 0.216106 | 0.0264 | -2.2586592 | -3.686421 | -0.30415341 |
| ATG7 | NM_006395 | 0.1915361 | 0.0206 | -2.3610668 | -3.459931 | -0.30434523 |
| PCDH8 | NM_002590 | 0.1967942 | 0.0218 | -2.3500352 | -3.436575 | -0.30446435 |
| CRK | NM_016823 | 0.1965399 | 0.0217 | -2.3390766 | -3.517295 | -0.30461748 |
| PKI55 | AL137534 | 0.2763419 | 0.044 | -2.0464942 | -4.066465 | -0.30467899 |
| FGF5 | NM_004464 | 0.2399886 | 0.033 | -2.1858801 | -3.644424 | -0.30483709 |
| CBR3 | AJ000096 | 0.2927986 | 0.0494 | -1.9974846 | -4.134926 | -0.3050457 |
| GFI1 | NM_005263 | 0.2500105 | 0.0364 | -2.1296959 | -3.893776 | -0.30523123 |
| EPHB4 | NM_004444 | 0.2631649 | 0.0401 | -2.0840044 | -4.04268 | -0.30602672 |
| FAM118A | NM_017911 | 0.1550215 | 0.0133 | -2.5320406 | -3.097791 | -0.30634355 |
| ZFYVE16 | NM_014733 | 0.2340457 | 0.0314 | -2.1925422 | -3.771763 | -0.30642558 |
| OAZ2 | NM_002537 | 0.2418742 | 0.0335 | -2.1563902 | -3.934385 | -0.30650235 |
| DYNLT1 | NM_006519 | 0.1820927 | 0.0183 | -2.4070347 | -3.373997 | -0.30695926 |
| CRKL | AK000311 | 0.278402 | 0.0447 | -2.042243 | -4.038491 | -0.30782336 |
| LOC100505534 | AL137606 | 0.1982537 | 0.0222 | -2.3490263 | -3.369414 | -0.30861006 |
| LINGO1 | AL109677 | 0.2803225 | 0.0453 | -2.0445183 | -3.973977 | -0.30908069 |
| PPFIA1 | NM_003626 | 0.2169415 | 0.0267 | -2.2582654 | -3.680889 | -0.30943586 |
| GAST | NM_000805 | 0.2686691 | 0.0415 | -2.0788115 | -3.949388 | -0.3106114 |
| TNFRSF10A | NM_003844 | 0.286151 | 0.0476 | -2.0164798 | -4.049449 | -0.31078102 |
| TTN | X98115 | 0.1842801 | 0.0187 | -2.4056801 | -3.324817 | -0.31204731 |
| POC1A | AL117629 | 0.1704108 | 0.016 | -2.4730546 | -3.143872 | -0.3120695 |
| GON4L | AK026693 | 0.2360748 | 0.032 | -2.1906617 | -3.74057 | -0.3121553 |
| PCBP3 | NM_020528 | 0.1016449 | 0.00566 | -2.8497483 | -2.330686 | -0.31225823 |
| YWHAZ | NM_003406 | 0.2908266 | 0.0488 | -1.9957951 | -4.227073 | -0.31243302 |
| OPCML | NM_002545 | 0.2938553 | 0.0499 | -2.0020798 | -3.990772 | -0.31281614 |
| RAB11FIP3 | NM_014700 | 0.2929026 | 0.0495 | -1.9951971 | -4.182657 | -0.31377048 |
| MGC70870 | D16888 | 0.1734265 | 0.0165 | -2.4519602 | -3.239946 | -0.31427326 |
| PHF1 | NM_002636 | 0.1359438 | 0.0101 | -2.624878 | -2.919151 | -0.31436551 |
| C21orf62 | NM_019596 | 0.2510017 | 0.0367 | -2.1348233 | -3.8282 | -0.31510358 |
| POLR2E | NM_002695 | 0.2636624 | 0.0403 | -2.0873175 | -3.924927 | -0.31515217 |
| MIR3142HG | L43345 | 0.2381674 | 0.0324 | -2.1928497 | -3.631609 | -0.31570715 |
| POM121L8P | AL117485 | 0.1944217 | 0.0212 | -2.3495661 | -3.458379 | -0.31622865 |
| SORBS1 | NM_015385 | 0.1979702 | 0.0221 | -2.3374683 | -3.406919 | -0.3162431 |
| CDK17 | AF086067 | 0.1964853 | 0.0217 | -2.3408972 | -3.523103 | -0.31634599 |
| CD7 | NM_006137 | 0.212861 | 0.0255 | -2.2868796 | -3.490455 | -0.31698515 |
| KLRB1 | NM_002258 | 0.2934012 | 0.0497 | -2.0021854 | -3.991658 | -0.31726821 |
| GPR157 | AK022194 | 0.0628051 | 0.00206 | -3.196595 | -1.440861 | -0.31872173 |
| TDP1 | NM_018319 | 0.1842801 | 0.0188 | -2.4017932 | -3.369352 | -0.31889824 |
| RHOB | NM_004040 | 0.2702815 | 0.0421 | -2.0612922 | -4.101705 | -0.31897496 |
| EXOSC6 | AK024276 | 0.2464837 | 0.0349 | -2.1699801 | -3.690994 | -0.31917403 |
| CDKN2A | NM_000077 | 0.2791097 | 0.045 | -2.0377136 | -4.050767 | -0.31939558 |
| GRK6 | NM_002082 | 0.2836274 | 0.0467 | -2.0174809 | -4.168381 | -0.31959756 |
| BCL2 | NM_000633 | 0.1789884 | 0.0177 | -2.4247767 | -3.310113 | -0.31972237 |
| SFTPB | NM_000542 | 0.1624289 | 0.0144 | -2.4990202 | -3.151503 | -0.32015352 |
| SERPINI1 | NM_005025 | 0.0667358 | 0.00238 | -3.1380315 | -1.600699 | -0.32065147 |
| TNXB | Y17867 | 0.1540171 | 0.0132 | -2.5450578 | -2.989096 | -0.32072009 |
| SULT1A1 | NM_001055 | 0.2498502 | 0.0362 | -2.1241696 | -3.996562 | -0.32113964 |
| ATG16L1 | NM_017974 | 0.1436022 | 0.0114 | -2.6034535 | -2.863695 | -0.32175666 |
| SIPA1L1 | NM_015556 | 0.1512579 | 0.0128 | -2.5410287 | -3.091089 | -0.32179549 |
| DKFZP434H168 | AL117580 | 0.234925 | 0.0317 | -2.19753 | -3.6423 | -0.32217597 |
| VIPR2 | NM_003382 | 0.1714522 | 0.0161 | -2.4752977 | -3.084294 | -0.32226332 |
| KISS1 | NM_002256 | 0.2522172 | 0.037 | -2.1478298 | -3.712573 | -0.32230111 |
| CREBBP | NM_004380 | 0.2098625 | 0.0247 | -2.2818987 | -3.679122 | -0.32237102 |
| MPZL2 | NM_005797 | 0.2688125 | 0.0416 | -2.0721849 | -4.001939 | -0.32262582 |
| PRO0471 | AF111846 | 0.1813691 | 0.0181 | -2.411683 | -3.340721 | -0.32283194 |
| DDX6 | AK021715 | 0.1184174 | 0.00759 | -2.7305793 | -2.658074 | -0.32288283 |
| PLXNA2 | AB007932 | 0.1836569 | 0.0185 | -2.4219301 | -3.193384 | -0.3232627 |
| CNBP | NM_003418 | 0.2432755 | 0.0339 | -2.1519779 | -3.931942 | -0.32378591 |
| PBX2 | NM_002586 | 0.2479036 | 0.0355 | -2.1419681 | -3.879059 | -0.32383071 |
| NR1H3 | NM_005693 | 0.1282245 | 0.00879 | -2.6926186 | -2.670632 | -0.3240226 |
| CPLX1 | NM_006651 | 0.175439 | 0.017 | -2.4442049 | -3.217466 | -0.32440641 |
| WDR60 | AK025192 | 0.1842801 | 0.0188 | -2.4083589 | -3.278442 | -0.32441384 |
| SPACA6 | AK024362 | 0.2538409 | 0.0375 | -2.1248682 | -3.831785 | -0.32464043 |
| TMPRSS11E | NM_014058 | 0.1678083 | 0.0155 | -2.4892033 | -3.110822 | -0.32465092 |
| PIF1 | AK026345 | 0.1662504 | 0.0151 | -2.4821965 | -3.199032 | -0.32484575 |
| CTAGE5 | NM_005930 | 0.1843805 | 0.0189 | -2.4095302 | -3.276263 | -0.32505614 |
| CARMIL1 | Z83935 | 0.2200238 | 0.0276 | -2.2526261 | -3.606073 | -0.32541382 |
| PCYT1B | AK022741 | 0.2832417 | 0.0465 | -2.0393125 | -3.831943 | -0.3259149 |
| CDH8 | NM_001796 | 0.1462109 | 0.0118 | -2.589499 | -2.89423 | -0.32593291 |
| TFAP2C | NM_003222 | 0.1646272 | 0.0148 | -2.4962594 | -3.131864 | -0.32617561 |
| CRYBA2 | NM_005209 | 0.2256142 | 0.0291 | -2.2497612 | -3.545311 | -0.32658974 |
| STAC | NM_003149 | 0.2049525 | 0.0237 | -2.3163392 | -3.464942 | -0.32792997 |
| NUP50 | AL050007 | 0.2803914 | 0.0454 | -2.0392195 | -4.006834 | -0.32847131 |
| LMTK2 | NM_014916 | 0.216106 | 0.0264 | -2.2671044 | -3.632952 | -0.32866194 |
| RNFT1 | NM_016125 | 0.2771369 | 0.0442 | -2.0466234 | -4.059014 | -0.32867118 |
| EVPL | NM_001988 | 0.1719792 | 0.0163 | -2.4514361 | -3.267468 | -0.32892077 |
| UPB1 | NM_016327 | 0.2763419 | 0.0441 | -2.0478539 | -3.979827 | -0.32908902 |
| PTPRN2 | U66702 | 0.1613774 | 0.0142 | -2.515591 | -3.10085 | -0.32919016 |
| ATP2C1 | NM_014382 | 0.2631649 | 0.04 | -2.090064 | -3.919853 | -0.32930605 |
| SSFA2 | NM_006751 | 0.2464471 | 0.0347 | -2.1473885 | -3.890737 | -0.33016682 |
| EPHA6 | AL133666 | 0.1678203 | 0.0155 | -2.4912255 | -3.094343 | -0.33092175 |
| GPR20 | NM_005293 | 0.2397153 | 0.0329 | -2.181728 | -3.70808 | -0.33144072 |
| SPAST | NM_014946 | 0.1910034 | 0.0205 | -2.3654993 | -3.424257 | -0.33161172 |
| TBX21 | NM_013351 | 0.2825576 | 0.046 | -2.0290354 | -4.031992 | -0.33219636 |
| RBSN | AY009133 | 0.1697769 | 0.0159 | -2.4619142 | -3.23265 | -0.33303389 |
| HSD17B14 | NM_016246 | 0.1939452 | 0.0209 | -2.3568641 | -3.428735 | -0.33304895 |
| PGBD2 | AK021482 | 0.1897973 | 0.0203 | -2.3728609 | -3.405968 | -0.33351153 |
| TOX4 | NM_014828 | 0.1350764 | 0.00989 | -2.6601497 | -2.729819 | -0.33399333 |
| MCF2L | AF086413 | 0.2186316 | 0.0272 | -2.2718202 | -3.450874 | -0.33436107 |
| ELOVL6 | AK027031 | 0.2441257 | 0.0341 | -2.1589675 | -3.858007 | -0.33502302 |
| RUNX2 | L40992 | 0.1382055 | 0.0106 | -2.6226156 | -2.862988 | -0.33512304 |
| GCNT3 | NM_004751 | 0.1352168 | 0.00993 | -2.6574256 | -2.734681 | -0.3352653 |
| CYP20A1 | AK021770 | 0.2738229 | 0.0432 | -2.0602113 | -3.952848 | -0.33538561 |
| LPP | NM_005578 | 0.2036611 | 0.0233 | -2.3148209 | -3.540343 | -0.33539405 |
| ABCG1 | NM_004915 | 0.2895559 | 0.0486 | -2.0015032 | -4.166756 | -0.33641472 |
| ARID1A | NM_006015 | 0.1819598 | 0.0182 | -2.4064758 | -3.365087 | -0.33672953 |
| NWD2 | AB033065 | 0.2828834 | 0.0464 | -2.0280887 | -3.992423 | -0.33723536 |
| ZAN | U83191 | 0.1643023 | 0.0147 | -2.5054156 | -3.075048 | -0.33727315 |
| ZNF444 | NM_018337 | 0.203751 | 0.0234 | -2.3121936 | -3.521107 | -0.33766501 |
| PAFAH1B1 | NM_000430 | 0.203751 | 0.0234 | -2.3203879 | -3.457037 | -0.33777912 |
| MEF2D | AK027180 | 0.2123544 | 0.0253 | -2.2875444 | -3.50622 | -0.3378598 |
| SYNJ1 | NM_003895 | 0.2186316 | 0.0272 | -2.2503752 | -3.685545 | -0.33799595 |
| GADD45G | NM_006705 | 0.1634577 | 0.0146 | -2.4997971 | -3.147538 | -0.33811011 |
| SPATA2L | AF070574 | 0.1532649 | 0.0131 | -2.5597296 | -2.96325 | -0.33812609 |
| HTR2C | NM_000868 | 0.1695325 | 0.0158 | -2.4930965 | -3.056238 | -0.33842538 |
| ACKR4 | NM_016557 | 0.2061381 | 0.0238 | -2.3156413 | -3.479765 | -0.33874929 |
| USP34 | AK024341 | 0.1185939 | 0.00766 | -2.7595477 | -2.483486 | -0.33919072 |
| MOB3B | AK022201 | 0.2922265 | 0.0493 | -2.0097012 | -3.930769 | -0.33937641 |
| IP6K1 | D87452 | 0.1943126 | 0.0212 | -2.3484219 | -3.519905 | -0.33977347 |
| C5orf15 | NM_020199 | 0.1555818 | 0.0134 | -2.5479963 | -2.975514 | -0.33996798 |
| BANP | NM_017869 | 0.1079621 | 0.00631 | -2.8061324 | -2.421761 | -0.34005414 |
| POU3F3 | NM_006236 | 0.228869 | 0.03 | -2.2074307 | -3.747784 | -0.34016436 |
| CXCL2 | NM_002089 | 0.2464837 | 0.035 | -2.1458691 | -3.876038 | -0.3401707 |
| DCTN2 | AK022171 | 0.2707221 | 0.0422 | -2.0708843 | -3.932789 | -0.34052622 |
| KANSL3 | AF117338 | 0.1860974 | 0.0192 | -2.3927186 | -3.339802 | -0.34123134 |
| C1GALT1 | AK023557 | 0.1892191 | 0.0201 | -2.3767303 | -3.372674 | -0.34151201 |
| BRSK2 | AF020089 | 0.1737714 | 0.0166 | -2.4404171 | -3.337065 | -0.34213132 |
| MORC3 | D50926 | 0.2869546 | 0.0478 | -2.0103857 | -4.116333 | -0.34217005 |
| CRHR2 | NM_001883 | 0.2876213 | 0.048 | -2.0105251 | -4.046162 | -0.34223812 |
| MAP4K4 | NM_017792 | 0.2464837 | 0.0349 | -2.1456668 | -3.880329 | -0.34245296 |
| RUNX2 | AF053952 | 0.1715931 | 0.0161 | -2.4597435 | -3.184126 | -0.3424904 |
| TESK2 | NM_007170 | 0.1866856 | 0.0195 | -2.3887946 | -3.373195 | -0.34264958 |
| HNRNPH3 | AF132362 | 0.1064568 | 0.00615 | -2.8018145 | -2.492397 | -0.34369418 |
| MAPK8IP3 | AB028989 | 0.2078456 | 0.0243 | -2.302991 | -3.507579 | -0.34395281 |
| SLC2A5 | NM_003039 | 0.0753405 | 0.00305 | -3.0637949 | -1.781589 | -0.3445369 |
| DRD2 | NM_000795 | 0.2532038 | 0.0373 | -2.1432947 | -3.682059 | -0.34458312 |
| NAPSA | NM_004851 | 0.1893475 | 0.0201 | -2.3967467 | -3.277082 | -0.34499297 |
| NTNG1 | NM_014917 | 0.216106 | 0.0263 | -2.2748782 | -3.476967 | -0.34503412 |
| TP53INP2 | AL137597 | 0.0678009 | 0.00248 | -3.1256651 | -1.655932 | -0.34539717 |
| LGALS8-AS1 | AK026321 | 0.2895559 | 0.0486 | -2.0199091 | -3.974648 | -0.34542122 |
| C11orf86 | AK026328 | 0.2181298 | 0.0269 | -2.2715136 | -3.467164 | -0.34578558 |
| PCLO | AB011131 | 0.2732799 | 0.043 | -2.0527319 | -4.103946 | -0.34581762 |
| USP19 | AB020698 | 0.1823016 | 0.0183 | -2.4089907 | -3.366454 | -0.34589873 |
| NECAP1 | NM_015509 | 0.1999595 | 0.0226 | -2.3276251 | -3.488211 | -0.34704221 |
| LINC00598 | AK025090 | 0.1788119 | 0.0176 | -2.4407537 | -3.184558 | -0.34752972 |
| RNF166 | AK026860 | 0.2837498 | 0.0467 | -2.0161321 | -4.184913 | -0.34764094 |
| DNAL4 | NM_005740 | 0.0988601 | 0.00526 | -2.8655485 | -2.302468 | -0.34800822 |
| FRY | U50526 | 0.216106 | 0.0264 | -2.2781822 | -3.523104 | -0.34810898 |
| XRCC5 | AK023296 | 0.1657326 | 0.015 | -2.4962968 | -3.080577 | -0.34812571 |
| ZKSCAN3 | U71601 | 0.1624289 | 0.0144 | -2.5189285 | -3.0356 | -0.34813081 |
| SLC9A2 | NM_003048 | 0.0924658 | 0.00465 | -2.9439398 | -2.091756 | -0.34818428 |
| HPX | J03048 | 0.1563324 | 0.0135 | -2.5296808 | -3.059135 | -0.34846068 |
| ABCF3 | NM_018358 | 0.1681304 | 0.0155 | -2.4667438 | -3.268077 | -0.34857228 |
| ZNF117 | AF075025 | 0.2554454 | 0.0378 | -2.1133909 | -3.936903 | -0.34860455 |
| JARID2 | NM_004973 | 0.0766741 | 0.00321 | -3.0325208 | -1.884807 | -0.34933357 |
| SMPDL3B | NM_014474 | 0.2152969 | 0.026 | -2.2718646 | -3.586065 | -0.35025901 |
| AGA | NM_000027 | 0.2488785 | 0.0358 | -2.1548116 | -3.680736 | -0.35181266 |
| GDI1 | NM_001493 | 0.1166352 | 0.00733 | -2.7408787 | -2.623812 | -0.35200564 |
| BOK | AF174487 | 0.1074067 | 0.00626 | -2.8214532 | -2.366832 | -0.35241889 |
| HABP2 | NM_004132 | 0.1119967 | 0.00675 | -2.7957974 | -2.448025 | -0.35244789 |
| TRIM62 | NM_018207 | 0.2184318 | 0.027 | -2.254535 | -3.606421 | -0.35290461 |
| APBB3 | NM_006051 | 0.176811 | 0.0173 | -2.4277965 | -3.319609 | -0.35323127 |
| TMEM55B | AL137727 | 0.1882468 | 0.0198 | -2.372947 | -3.460921 | -0.35358125 |
| DIP2C | AB023151 | 0.2381674 | 0.0324 | -2.1764226 | -3.807172 | -0.35424697 |
| SARAF | NM_016127 | 0.2432755 | 0.0339 | -2.1531265 | -3.91435 | -0.35427945 |
| ZNF445 | AK026891 | 0.2512732 | 0.0368 | -2.1431793 | -3.721313 | -0.35429183 |
| ESRRA | NM_004451 | 0.1697769 | 0.0158 | -2.4701093 | -3.162442 | -0.35457533 |
| CACUL1 | AK026985 | 0.2880562 | 0.0481 | -2.0076729 | -4.148079 | -0.35463805 |
| KIAA1614-AS1 | AK026359 | 0.1819598 | 0.0182 | -2.4249668 | -3.200809 | -0.35507027 |
| AVPR1B | NM_000707 | 0.2611378 | 0.0396 | -2.0917889 | -3.997066 | -0.35546443 |
| SNX2 | NM_003100 | 0.228869 | 0.03 | -2.2074557 | -3.773985 | -0.35577789 |
| SWT1 | NM_017673 | 0.2182562 | 0.027 | -2.2588256 | -3.648519 | -0.35583415 |
| ARFIP1 | NM_014447 | 0.2338154 | 0.0313 | -2.1886585 | -3.848083 | -0.35614552 |
| PIAS2 | NM_004671 | 0.1688731 | 0.0156 | -2.4721763 | -3.171364 | -0.35618867 |
| MYH14 | L29142 | 0.2397153 | 0.033 | -2.1857312 | -3.644604 | -0.35631335 |
| HIST2H4A | NM_003548 | 0.2182562 | 0.027 | -2.2595266 | -3.593906 | -0.35671464 |
| DMTN | NM_001978 | 0.2432755 | 0.0339 | -2.1662894 | -3.72027 | -0.35742995 |
| NPR3 | NM_000908 | 0.221853 | 0.028 | -2.2471821 | -3.548717 | -0.35753311 |
| FGF7 | NM_002009 | 0.2007113 | 0.0227 | -2.3393209 | -3.430892 | -0.35760251 |
| BMP2K | AK021620 | 0.1667447 | 0.0152 | -2.5034736 | -3.071212 | -0.35775717 |
| DNAJC24 | AL050199 | 0.2257739 | 0.0292 | -2.2290843 | -3.679517 | -0.35787222 |
| SFXN5 | AK025170 | 0.2716382 | 0.0426 | -2.0662629 | -3.974495 | -0.35799582 |
| GRB2 | AF302079 | 0.282682 | 0.0461 | -2.0204061 | -4.182709 | -0.35806505 |
| TNRC6B | AB029016 | 0.2707673 | 0.0422 | -2.0617924 | -4.082268 | -0.35837814 |
| ADAMTS7 | AL110226 | 0.1943126 | 0.0211 | -2.3493092 | -3.508026 | -0.35848367 |
| GUCA1B | NM_002098 | 0.2314641 | 0.0306 | -2.1983134 | -3.805921 | -0.35930856 |
| MKLN1 | AK025088 | 0.1699197 | 0.0159 | -2.45836 | -3.275915 | -0.35934846 |
| USP15 | AF106069 | 0.1844438 | 0.0189 | -2.3909956 | -3.41188 | -0.35963593 |
| MAPK8IP2 | NM_012324 | 0.1278324 | 0.00874 | -2.6936007 | -2.690174 | -0.36036895 |
| ZNF646 | NM_014699 | 0.1814882 | 0.0181 | -2.4209184 | -3.252262 | -0.36037922 |
| APLNR | NM_005161 | 0.216106 | 0.0263 | -2.2777071 | -3.508331 | -0.36088027 |
| NKX3-1 | NM_006167 | 0.0360761 | 0.000665 | -3.5519009 | -0.470193 | -0.36179382 |
| FLJ11710 | AK021772 | 0.2681917 | 0.0414 | -2.0870006 | -3.798963 | -0.36188872 |
| NAGLU | NM_000263 | 0.2380332 | 0.0324 | -2.1773009 | -3.775487 | -0.36216153 |
| SF3B1 | NM_012433 | 0.2700839 | 0.042 | -2.0608532 | -4.120391 | -0.362171 |
| PRELP | NM_002725 | 0.2716382 | 0.0426 | -2.0708933 | -3.871927 | -0.36218846 |
| ATP2B1 | NM_001682 | 0.1363392 | 0.0102 | -2.6239555 | -2.891215 | -0.36231967 |
| HOMER2 | NM_004839 | 0.2340873 | 0.0315 | -2.1941329 | -3.722236 | -0.36240297 |
| DHX16 | NM_003587 | 0.1236078 | 0.00823 | -2.7177015 | -2.625006 | -0.36302328 |
| LRWD1 | AL133057 | 0.2356937 | 0.0319 | -2.1789949 | -3.862435 | -0.36316601 |
| BCL2A1 | NM_004049 | 0.1586008 | 0.0139 | -2.5330393 | -3.041466 | -0.36395563 |
| MTMR14 | AK021608 | 0.2499157 | 0.0363 | -2.1279754 | -3.940161 | -0.36398129 |
| FAM120A | NM_014612 | 0.1643023 | 0.0147 | -2.4847348 | -3.241281 | -0.36473407 |
| NELL1 | NM_006157 | 0.2069058 | 0.024 | -2.3182993 | -3.37869 | -0.36507289 |
| KRT75 | NM_004693 | 0.0808448 | 0.00347 | -3.0622099 | -1.825426 | -0.36577165 |
| GALNT8 | NM_017417 | 0.2278609 | 0.0298 | -2.220672 | -3.695927 | -0.36596004 |
| CASZ1 | NM_017766 | 0.0310183 | 0.000479 | -3.6345669 | -0.200486 | -0.36597344 |
| PDLIM2 | AY007729 | 0.2662583 | 0.0409 | -2.0722877 | -4.096485 | -0.36637029 |
| WLS | AK026744 | 0.0918792 | 0.00457 | -2.9120086 | -2.210198 | -0.36650114 |
| TFE3 | NM_006521 | 0.2088863 | 0.0245 | -2.2933181 | -3.585765 | -0.3665237 |
| ITGA9 | D25303 | 0.1981559 | 0.0221 | -2.3491466 | -3.398432 | -0.36682484 |
| PNMAL2 | AB033009 | 0.2226237 | 0.0283 | -2.2293182 | -3.781161 | -0.36768944 |
| CMC1 | AF086095 | 0.1605346 | 0.0141 | -2.5091225 | -3.104174 | -0.36785 |
| C17orf75 | AF305686 | 0.1434156 | 0.0113 | -2.5860798 | -2.989034 | -0.36786256 |
| MKLN1 | AF086386 | 0.0660645 | 0.00231 | -3.1557861 | -1.565722 | -0.36786538 |
| MAML1 | NM_014757 | 0.2842376 | 0.047 | -2.0141149 | -4.187401 | -0.36802213 |
| SERHL2 | NM_014509 | 0.0231308 | 0.000282 | -3.829724 | 0.296171 | -0.36805066 |
| IL22 | NM_020525 | 0.183221 | 0.0185 | -2.4066216 | -3.296476 | -0.368796 |
| PNRC1 | NM_006813 | 0.1490667 | 0.0123 | -2.5581453 | -3.030391 | -0.36889685 |
| ACTN1 | NM_001102 | 0.1052729 | 0.00601 | -2.8101984 | -2.471633 | -0.3690948 |
| BBC3 | U82987 | 0.1346767 | 0.00985 | -2.6426904 | -2.815757 | -0.36934182 |
| KIAA0430 | AB007890 | 0.1314749 | 0.00933 | -2.6543363 | -2.849759 | -0.36980519 |
| ITGA11 | AL117454 | 0.1822663 | 0.0183 | -2.4025696 | -3.409087 | -0.37026466 |
| THSD1 | NM_018676 | 0.1962397 | 0.0216 | -2.358564 | -3.379704 | -0.37028353 |
| LOC103344931 | AL049387 | 0.1989582 | 0.0224 | -2.331822 | -3.465398 | -0.37053059 |
| ATG2A | AB007864 | 0.0732915 | 0.00292 | -3.0689052 | -1.802712 | -0.37194741 |
| CCL22 | NM_002990 | 0.1340255 | 0.00974 | -2.6746411 | -2.684646 | -0.37211027 |
| CEP68 | AF090099 | 0.2569046 | 0.0383 | -2.1168281 | -3.845101 | -0.37221435 |
| TAP2 | NM_000544 | 0.2526582 | 0.0371 | -2.1249777 | -3.867728 | -0.37242945 |
| NCOA6 | NM_014071 | 0.2332667 | 0.0312 | -2.1903831 | -3.823512 | -0.37257925 |
| GSTT2 | NM_000854 | 0.2493265 | 0.0361 | -2.1304867 | -3.912192 | -0.3728786 |
| TMEM168 | AK023638 | 0.0513886 | 0.00125 | -3.369413 | -0.995339 | -0.37312813 |
| IL17RD | AL133097 | 0.1842801 | 0.0188 | -2.4001101 | -3.324492 | -0.37318516 |
| SNU13 | NM_005008 | 0.0640443 | 0.00214 | -3.1842501 | -1.469341 | -0.37332539 |
| SPIB | AK025419 | 0.2314641 | 0.0307 | -2.2128935 | -3.594153 | -0.37345014 |
| ITCH | AF038564 | 0.0958373 | 0.005 | -2.8862043 | -2.253232 | -0.37354086 |
| MYLIP | NM_013262 | 0.2512732 | 0.0368 | -2.1186017 | -3.992143 | -0.3738932 |
| GNG7 | AK024465 | 0.2464837 | 0.035 | -2.1582244 | -3.714458 | -0.3745541 |
| F13B | NM_001994 | 0.216106 | 0.0263 | -2.2815743 | -3.484513 | -0.37458163 |
| TBC1D2B | AK000173 | 0.2619357 | 0.0397 | -2.0971209 | -3.885626 | -0.37469705 |
| DLL4 | NM_019074 | 0.0866583 | 0.00403 | -2.9576074 | -2.071321 | -0.37528514 |
| ITPK1 | NM_014216 | 0.1942796 | 0.021 | -2.3465126 | -3.52043 | -0.37531167 |
| CACNA2D2 | NM_006030 | 0.1898759 | 0.0203 | -2.368447 | -3.430361 | -0.37538835 |
| MAP3K14 | AJ008158 | 0.2327579 | 0.031 | -2.2012632 | -3.673882 | -0.37576635 |
| IBSP | NM_004967 | 0.1449966 | 0.0116 | -2.6102029 | -2.812576 | -0.37579745 |
| ODF2 | NM_002540 | 0.1788119 | 0.0175 | -2.423049 | -3.304219 | -0.37610248 |
| LOC647115///UGP2 | U00954 | 0.257798 | 0.0387 | -2.1119785 | -3.87051 | -0.37632847 |
| ZNF200 | NM_003454 | 0.2746695 | 0.0435 | -2.0484352 | -4.119725 | -0.37641755 |
| MID1IP1 | AJ272057 | 0.1017826 | 0.00568 | -2.8305461 | -2.403456 | -0.37664597 |
| DUSP6 | NM_001946 | 0.2327579 | 0.031 | -2.1884554 | -3.870619 | -0.37691199 |
| SLC22A31 | AL137382 | 0.1465527 | 0.0119 | -2.5759001 | -2.978978 | -0.37705421 |
| MTMR1 | AF057354 | 0.2579868 | 0.0389 | -2.1100405 | -3.840626 | -0.37709382 |
| PCDH1 | NM_002587 | 0.1956277 | 0.0214 | -2.3427366 | -3.52347 | -0.37714321 |
| SLC26A6 | AF161369 | 0.1467802 | 0.0119 | -2.5757217 | -2.968552 | -0.37728313 |
| SCARF1 | NM_003693 | 0.2736918 | 0.0432 | -2.0595993 | -3.988699 | -0.37741466 |
| NUFIP2 | AK001838 | 0.2709663 | 0.0423 | -2.0572248 | -4.127268 | -0.37750082 |
| RNF217 | AK023584 | 0.1216492 | 0.00799 | -2.7341983 | -2.558967 | -0.37800412 |
| ANAPC10 | NM_014885 | 0.2438013 | 0.0341 | -2.1655183 | -3.756065 | -0.37855299 |
| TBC1D27 | AK024458 | 0.1866856 | 0.0195 | -2.3862914 | -3.379813 | -0.3789971 |
| IHH | L38517 | 0.054419 | 0.00151 | -3.3095772 | -1.162612 | -0.37916431 |
| KIF21B | NM_017596 | 0.2511551 | 0.0367 | -2.1195195 | -3.991427 | -0.37940919 |
| FEM1C | AL365415 | 0.157838 | 0.0138 | -2.5230746 | -3.073596 | -0.37996753 |
| LRRC20 | NM_018239 | 0.1357279 | 0.0101 | -2.6417289 | -2.79758 | -0.38001601 |
| ATXN8///ATXN3 | U63332 | 0.1503974 | 0.0126 | -2.571117 | -2.912776 | -0.38014848 |
| STAT3 | AK024535 | 0.2356937 | 0.0319 | -2.1773219 | -3.892856 | -0.38122945 |
| CDK17 | NM_002595 | 0.1942796 | 0.021 | -2.3599829 | -3.419434 | -0.38134915 |
| CALCA | NM_001741 | 0.1502516 | 0.0125 | -2.5655531 | -2.919028 | -0.38176662 |
| PITPNA | NM_006224 | 0.1788119 | 0.0176 | -2.4165045 | -3.391548 | -0.38215271 |
| MIER1 | AB046830 | 0.191213 | 0.0205 | -2.3606067 | -3.462662 | -0.38218272 |
| SPOCK3 | NM_016950 | 0.203751 | 0.0234 | -2.329776 | -3.407584 | -0.3822225 |
| ARC | NM_015193 | 0.1274252 | 0.00868 | -2.7037958 | -2.627721 | -0.38266679 |
| BTG1 | NM_001731 | 0.1934196 | 0.0209 | -2.3497558 | -3.514175 | -0.38282194 |
| LY6D | X82693 | 0.2731509 | 0.0429 | -2.0587983 | -4.025678 | -0.38313661 |
| LCAL1 | AK023806 | 0.2148823 | 0.0259 | -2.2765831 | -3.559801 | -0.38351121 |
| PIGP | NM_016430 | 0.2882297 | 0.0482 | -2.0159587 | -3.988793 | -0.38427873 |
| STK4 | NM_006282 | 0.25313 | 0.0372 | -2.1127634 | -3.994735 | -0.38448207 |
| CTSB | L38712 | 0.1423017 | 0.0112 | -2.6069649 | -2.866167 | -0.38472208 |
| C20orf181 | U63828 | 0.1582016 | 0.0138 | -2.5172428 | -3.086323 | -0.38530627 |
| UBXN2B | U79282 | 0.0709068 | 0.00273 | -3.092592 | -1.727666 | -0.38531334 |
| SGK1 | NM_005627 | 0.1326671 | 0.00951 | -2.6480767 | -2.855122 | -0.38551642 |
| WBP1L | NM_017787 | 0.2494326 | 0.0361 | -2.1359468 | -3.876857 | -0.38604518 |
| CLTC | AF130062 | 0.0882579 | 0.00425 | -2.9517371 | -2.046191 | -0.3860603 |
| DCC | S81335 | 0.0819837 | 0.0036 | -3.0058581 | -1.917465 | -0.38618488 |
| DST | AL049215 | 0.1737714 | 0.0166 | -2.4642922 | -3.122456 | -0.38639644 |
| CNIH3 | AF070524 | 0.2820469 | 0.0458 | -2.038996 | -3.927944 | -0.38649246 |
| TNFSF8 | NM_001244 | 0.2531777 | 0.0373 | -2.1243737 | -3.881107 | -0.38692529 |
| WDR78 | AL133617 | 0.2542828 | 0.0376 | -2.1275575 | -3.727804 | -0.38700039 |
| POU1F1 | NM_000306 | 0.0640443 | 0.00215 | -3.1985824 | -1.474586 | -0.38719546 |
| PRKAG2 | NM_016203 | 0.2808959 | 0.0455 | -2.0361082 | -4.072552 | -0.38773415 |
| CALY | NM_015722 | 0.1373219 | 0.0104 | -2.6286773 | -2.802207 | -0.38776199 |
| PRICKLE1 | AK021728 | 0.2498502 | 0.0363 | -2.1645898 | -3.614673 | -0.38778846 |
| TTC21A | AK022483 | 0.238132 | 0.0324 | -2.1912319 | -3.634349 | -0.38780379 |
| MMP27 | AF195192 | 0.1497064 | 0.0124 | -2.5695505 | -2.949895 | -0.38788185 |
| ABO | U15197 | 0.1391336 | 0.0107 | -2.6187959 | -2.837106 | -0.38866501 |
| FGF19 | NM_005117 | 0.0963597 | 0.00503 | -2.9143636 | -2.150895 | -0.3887726 |
| LOC284561 | AK023809 | 0.0513927 | 0.00126 | -3.3643832 | -1.003693 | -0.38939871 |
| PPP1R12B | AB007972 | 0.0989871 | 0.00531 | -2.8750583 | -2.271306 | -0.38952515 |
| CBFA2T3 | NM_005187 | 0.0915925 | 0.00454 | -2.9203379 | -2.168904 | -0.38960977 |
| IGF2 | S51971 | 0.2258027 | 0.0292 | -2.2140319 | -3.807337 | -0.38962075 |
| NCOA2 | NM_006540 | 0.1659965 | 0.0151 | -2.4832362 | -3.226298 | -0.38978556 |
| ZNF407 | AK023901 | 0.2934012 | 0.0497 | -1.9959709 | -4.135052 | -0.38981913 |
| CWC25 | NM_017748 | 0.1226241 | 0.00811 | -2.7083921 | -2.719799 | -0.39003787 |
| PI16 | AL050088 | 0.1020863 | 0.00572 | -2.8453841 | -2.330872 | -0.39027979 |
| TMEM115 | NM_007024 | 0.2327579 | 0.031 | -2.1903644 | -3.852109 | -0.3904047 |
| PRR5L | AK024275 | 0.2153734 | 0.026 | -2.277671 | -3.508354 | -0.39068264 |
| FRMD4B | AB023230 | 0.2492848 | 0.036 | -2.1434745 | -3.779276 | -0.39078894 |
| CEP85L | AL133101 | 0.2336706 | 0.0313 | -2.1909872 | -3.828345 | -0.39080467 |
| JMJD1C | AB037801 | 0.2459695 | 0.0345 | -2.1442116 | -3.959374 | -0.39155149 |
| RET | X15262 | 0.0759704 | 0.00313 | -3.0533959 | -1.822061 | -0.39162948 |
| OPRK1 | AK023198 | 0.2883803 | 0.0483 | -2.0028591 | -4.194059 | -0.39178429 |
| RGS4 | U27768 | 0.1813691 | 0.0181 | -2.4204256 | -3.280912 | -0.39189981 |
| FBRS | AK022551 | 0.2837498 | 0.0467 | -2.0181372 | -4.150273 | -0.39231163 |
| KIAA0319 | NM_014809 | 0.2145555 | 0.0258 | -2.2855011 | -3.493298 | -0.39238014 |
| SLC16A4 | NM_004696 | 0.2752526 | 0.0437 | -2.0499684 | -4.029252 | -0.39335369 |
| TMOD2 | NM_014548 | 0.1326671 | 0.00951 | -2.6763882 | -2.695155 | -0.39362617 |
| PACSIN2 | NM_007229 | 0.1648951 | 0.0149 | -2.4819504 | -3.234361 | -0.39396653 |
| SNX27 | AK001395 | 0.1496119 | 0.0124 | -2.5569878 | -3.021826 | -0.39401587 |
| FAM134C | L38937 | 0.2938553 | 0.0499 | -1.9940841 | -4.125344 | -0.39432099 |
| S100A10 | NM_002966 | 0.1188131 | 0.00772 | -2.7351252 | -2.621262 | -0.39449631 |
| ZHX2 | AF075099 | 0.233627 | 0.0312 | -2.2107036 | -3.63466 | -0.39467432 |
| HERC6 | NM_017912 | 0.1981878 | 0.0221 | -2.3350077 | -3.486568 | -0.39474235 |
| CAPNS1 | NM_001749 | 0.1813691 | 0.0181 | -2.4086698 | -3.394466 | -0.39562426 |
| NOV | NM_002514 | 0.0868362 | 0.00409 | -2.9561698 | -2.094289 | -0.39565091 |
| PPP6R2 | NM_014678 | 0.1843199 | 0.0188 | -2.399693 | -3.363437 | -0.39667345 |
| PAK6 | NM_020168 | 0.1877333 | 0.0197 | -2.3945872 | -3.306911 | -0.39669879 |
| SNED1 | AL050143 | 0.2043777 | 0.0235 | -2.3181138 | -3.489963 | -0.39782454 |
| TIMM10 | NM_012456 | 0.0344313 | 0.000614 | -3.5925034 | -0.382781 | -0.3992014 |
| AIRE | NM_000383 | 0.106631 | 0.00617 | -2.8371431 | -2.342267 | -0.39924353 |
| TMX4 | AB032988 | 0.1568827 | 0.0136 | -2.5204088 | -3.114911 | -0.40024865 |
| LINC00869 | AB007962 | 0.1865645 | 0.0194 | -2.3874964 | -3.351252 | -0.40046358 |
| CRTAC1 | NM_018058 | 0.28081 | 0.0455 | -2.0426901 | -3.875424 | -0.40048603 |
| ABHD3 | AF007152 | 0.1095633 | 0.00647 | -2.7913156 | -2.510596 | -0.4007042 |
| FAM193A | NM_003704 | 0.1302889 | 0.00916 | -2.6888569 | -2.687852 | -0.4007875 |
| PIK3CA | AK021510 | 0.2914905 | 0.049 | -2.0037831 | -4.072024 | -0.40141271 |
| MFN2 | NM_014874 | 0.1099701 | 0.00652 | -2.7927952 | -2.472722 | -0.40156907 |
| CRYGD | NM_006891 | 0.1362626 | 0.0102 | -2.6481629 | -2.7443 | -0.40266367 |
| BAIAP3 | NM_003933 | 0.1398508 | 0.0108 | -2.607492 | -2.928337 | -0.40271197 |
| NDE1 | NM_017668 | 0.1409908 | 0.011 | -2.5986905 | -2.938761 | -0.40305906 |
| CD46 | M58050 | 0.1988542 | 0.0223 | -2.3226649 | -3.594571 | -0.40373631 |
| CD40 | NM_001250 | 0.1028461 | 0.00578 | -2.8475017 | -2.331242 | -0.4037701 |
| SNX29 | AK022425 | 0.108788 | 0.0064 | -2.8273108 | -2.342936 | -0.40497345 |
| KCNJ16 | NM_018658 | 0.1373219 | 0.0104 | -2.6230881 | -2.871959 | -0.40513223 |
| ZNF536 | NM_014717 | 0.2833692 | 0.0466 | -2.0394979 | -3.879754 | -0.40514646 |
| SOS2 | L20686 | 0.2880562 | 0.0482 | -2.0024003 | -4.200657 | -0.40542815 |
| TRIP12 | D28476 | 0.1893475 | 0.0201 | -2.3794578 | -3.410646 | -0.40574145 |
| GMDS-AS1 | AK023629 | 0.1468345 | 0.012 | -2.5795095 | -2.926288 | -0.40642341 |
| HIST1H4H | NM_003543 | 0.1170195 | 0.00742 | -2.7641416 | -2.533159 | -0.40653547 |
| PLAUR | NM_002659 | 0.0815565 | 0.00355 | -3.027235 | -1.888784 | -0.40661471 |
| TCF3 | AL117663 | 0.1291459 | 0.0089 | -2.7040764 | -2.615136 | -0.4070161 |
| PELO | NM_014100 | 0.2036611 | 0.0233 | -2.3243748 | -3.399879 | -0.40712856 |
| BPY2 | NM_004678 | 0.2685123 | 0.0414 | -2.0816245 | -3.892787 | -0.40714345 |
| ZMYM3 | NM_005096 | 0.1519057 | 0.0129 | -2.5389446 | -3.105443 | -0.407517 |
| POU2F2 | NM_002698 | 0.216106 | 0.0265 | -2.2680722 | -3.544617 | -0.40775099 |
| RRM2B | AB036063 | 0.1656899 | 0.015 | -2.4873651 | -3.163838 | -0.40783698 |
| GEN1 | AK025489 | 0.1676391 | 0.0154 | -2.4786681 | -3.170262 | -0.40875816 |
| SPATA31A3 | AL080148 | 0.143959 | 0.0114 | -2.6201812 | -2.808037 | -0.40921363 |
| HTR1A | NM_000524 | 0.2070505 | 0.0241 | -2.3143413 | -3.366742 | -0.40926078 |
| OPTC | NM_014359 | 0.054419 | 0.00145 | -3.3061415 | -1.129522 | -0.40953534 |
| NFAT5 | AK001067 | 0.1897973 | 0.0202 | -2.3672729 | -3.458719 | -0.40991752 |
| CYB5D1 | AK022811 | 0.0628051 | 0.00206 | -3.1948917 | -1.449358 | -0.4103765 |
| PDZRN4 | NM_013377 | 0.0756724 | 0.00309 | -3.0665123 | -1.757287 | -0.41101563 |
| SP140 | NM_007237 | 0.2463376 | 0.0346 | -2.1551519 | -3.823992 | -0.41110805 |
| HERPUD2 | AK025966 | 0.2086433 | 0.0244 | -2.2943465 | -3.583667 | -0.41148239 |
| OMP | NM_006189 | 0.188673 | 0.0199 | -2.3850592 | -3.311063 | -0.41189594 |
| GRIK4 | NM_014619 | 0.0699709 | 0.00265 | -3.1435506 | -1.611325 | -0.41192378 |
| SNUPN | NM_005701 | 0.282682 | 0.0462 | -2.0213204 | -4.164682 | -0.41229059 |
| CSNK1D | U31285 | 0.2155546 | 0.0261 | -2.278791 | -3.522842 | -0.41355067 |
| MTM1 | NM_000252 | 0.1369265 | 0.0103 | -2.6306701 | -2.844504 | -0.41424575 |
| VGLL1 | NM_016267 | 0.1101729 | 0.00656 | -2.7998588 | -2.432912 | -0.41453503 |
| TMEM268 | AF038174 | 0.1870423 | 0.0195 | -2.402762 | -3.202031 | -0.41462058 |
| FLI1 | S64709 | 0.2928173 | 0.0495 | -1.9922626 | -4.212166 | -0.41463631 |
| STXBP6 | NM_014178 | 0.1373219 | 0.0104 | -2.6285788 | -2.839112 | -0.41485029 |
| TMEM138 | NM_016464 | 0.088704 | 0.00432 | -2.9498436 | -2.074089 | -0.4151343 |
| EFCC1 | AK022119 | 0.0868362 | 0.0041 | -2.9934605 | -1.987406 | -0.41541746 |
| AMY2A | NM_000699 | 0.2686691 | 0.0415 | -2.0775146 | -3.903208 | -0.41549724 |
| SOBP | AK021973 | 0.1468345 | 0.012 | -2.6043843 | -2.821989 | -0.41573879 |
| HIST1H1T | NM_005323 | 0.1893475 | 0.0201 | -2.3850211 | -3.280435 | -0.41644192 |
| SAMD14 | AF035306 | 0.1747585 | 0.0169 | -2.4505326 | -3.176513 | -0.41702046 |
| ZBTB18 | NM_006352 | 0.2219001 | 0.028 | -2.2395051 | -3.691842 | -0.4171325 |
| SLC17A1 | Y10513 | 0.0886388 | 0.00431 | -2.9551395 | -2.066589 | -0.41716471 |
| RNF139 | NM_007218 | 0.0409853 | 0.000845 | -3.4815597 | -0.67375 | -0.41748531 |
| OR3A1 | NM_002550 | 0.098963 | 0.00528 | -2.9071532 | -2.182844 | -0.4174966 |
| OAZ1 | NM_004152 | 0.0973875 | 0.00511 | -2.8725967 | -2.309108 | -0.41776874 |
| ST6GALNAC2 | NM_006456 | 0.1555823 | 0.0134 | -2.5216108 | -3.146075 | -0.41828496 |
| MTMR4 | AF264717 | 0.1000418 | 0.00544 | -2.8662567 | -2.26464 | -0.41874216 |
| MAP4K4 | NM_004834 | 0.0639603 | 0.00212 | -3.1596481 | -1.558544 | -0.42050332 |
| PELI2 | AF302502 | 0.1895971 | 0.0202 | -2.3659415 | -3.475842 | -0.4210199 |
| MYO5B | AB032945 | 0.0602222 | 0.00185 | -3.23982 | -1.337345 | -0.42129775 |
| IL9R | NM_002186 | 0.1052729 | 0.00602 | -2.8385428 | -2.301351 | -0.42185638 |
| ATXN7 | NM_000333 | 0.1482563 | 0.0122 | -2.5595837 | -3.047899 | -0.42190931 |
| HESX1 | NM_003865 | 0.1434215 | 0.0113 | -2.60148 | -2.890021 | -0.42277435 |
| ELF1 | M82882 | 0.2858989 | 0.0475 | -2.0083991 | -4.188538 | -0.42297584 |
| FMN2 | AF218941 | 0.2777453 | 0.0445 | -2.0430798 | -4.081937 | -0.42307945 |
| CYLC1 | Z22780 | 0.1722851 | 0.0163 | -2.4697425 | -3.110519 | -0.42404561 |
| PARP8 | AK024961 | 0.257798 | 0.0387 | -2.0961599 | -4.050786 | -0.42424422 |
| RAB21 | NM_014999 | 0.1462109 | 0.0119 | -2.5711613 | -2.978393 | -0.42465202 |
| MICAL3 | AB037785 | 0.1897973 | 0.0203 | -2.3730639 | -3.350815 | -0.42479243 |
| EPS15L1 | AF110265 | 0.0775441 | 0.00328 | -3.0281285 | -1.891469 | -0.42722826 |
| LOC102724002 | AK025166 | 0.1667447 | 0.0153 | -2.4756603 | -3.214844 | -0.42729153 |
| IGKV1D-8 | AF035035 | 0.0842358 | 0.00382 | -3.0164208 | -1.929173 | -0.42821934 |
| NCK2 | NM_003581 | 0.0607189 | 0.00188 | -3.210977 | -1.414161 | -0.42897845 |
| MED13L | AL133033 | 0.150826 | 0.0127 | -2.5458147 | -3.047429 | -0.42900741 |
| MAPK1 | AL157438 | 0.1970083 | 0.0219 | -2.3311636 | -3.563095 | -0.42905728 |
| NUP50 | NM_007172 | 0.1496119 | 0.0124 | -2.5517298 | -3.067214 | -0.42920433 |
| TMEM216 | NM_016499 | 0.2340873 | 0.0315 | -2.1929151 | -3.769279 | -0.42973135 |
| LGR6 | AF190501 | 0.0812343 | 0.00352 | -3.0207654 | -1.890397 | -0.43032239 |
| CHST7 | NM_019886 | 0.0607189 | 0.00189 | -3.2398489 | -1.343431 | -0.43091715 |
| TINAGL1 | AB050716 | 0.1440794 | 0.0115 | -2.5867095 | -2.96543 | -0.43103015 |
| SLC6A12 | NM_003044 | 0.216408 | 0.0265 | -2.277377 | -3.474652 | -0.4316437 |
| COL9A2 | M95610 | 0.212861 | 0.0254 | -2.2750777 | -3.671455 | -0.43220896 |
| NODAL | NM_018055 | 0.065408 | 0.00223 | -3.1784979 | -1.496275 | -0.43221082 |
| DOCK4 | AF085922 | 0.0345677 | 0.000623 | -3.5761553 | -0.403757 | -0.43316828 |
| GTF2H5 | AK024874 | 0.2218508 | 0.028 | -2.2560086 | -3.532914 | -0.43369736 |
| FBXL20 | AL117623 | 0.0801519 | 0.00344 | -3.0367807 | -1.862611 | -0.4338395 |
| TCEB3B | NM_016427 | 0.1888657 | 0.0199 | -2.3673158 | -3.487702 | -0.43459944 |
| ST3GAL1 | AK024824 | 0.1717809 | 0.0162 | -2.4797655 | -3.094517 | -0.43522424 |
| CTC1 | AK025823 | 0.1440794 | 0.0115 | -2.5991229 | -2.884589 | -0.4352582 |
| JAG1 | NM_000214 | 0.0738979 | 0.00297 | -3.0756982 | -1.747905 | -0.43534717 |
| KCNJ15 | Y13895 | 0.2736918 | 0.0431 | -2.0548761 | -4.052024 | -0.4359893 |
| VPREB3 | NM_013378 | 0.1064568 | 0.00615 | -2.8263587 | -2.382869 | -0.43646322 |
| NLGN3 | NM_018977 | 0.1399213 | 0.0108 | -2.6245458 | -2.806974 | -0.43706869 |
| LTK | NM_002344 | 0.1166352 | 0.00734 | -2.7885387 | -2.428996 | -0.43783882 |
| OR52K3P | AF143328 | 0.1714522 | 0.0161 | -2.4678655 | -3.167772 | -0.43867344 |
| PTBP3 | NM_005156 | 0.1241757 | 0.00828 | -2.696181 | -2.750415 | -0.4395338 |
| TRANK1 | AB002340 | 0.2418742 | 0.0335 | -2.1576425 | -3.927737 | -0.44051362 |
| PTPRK | AK021778 | 0.2529565 | 0.0372 | -2.1345204 | -3.756779 | -0.44084087 |
| SUGT1P3 | AF086053 | 0.2257704 | 0.0291 | -2.2283481 | -3.65446 | -0.44116158 |
| SYNM | AF147375 | 0.1934196 | 0.0208 | -2.3643377 | -3.336283 | -0.4415934 |
| PSD4 | NM_012455 | 0.2396502 | 0.0329 | -2.166621 | -3.897225 | -0.44190024 |
| PTPN9 | AL360201 | 0.2218047 | 0.0279 | -2.2750598 | -3.422378 | -0.44200582 |
| ABAT | NM_000663 | 0.0063444 | 2.58E-05 | -4.4733101 | 2.411774 | -0.44207367 |
| RBBP6 | AK023612 | 0.1083291 | 0.00635 | -2.8208012 | -2.363336 | -0.44226172 |
| NEDD9 | NM_006403 | 0.0988601 | 0.00526 | -2.8628212 | -2.341349 | -0.44240235 |
| NEBL | NM_006393 | 0.1455237 | 0.0117 | -2.5872959 | -2.931996 | -0.44312222 |
| ARF1 | AK023803 | 0.2218047 | 0.0279 | -2.2513761 | -3.605722 | -0.44320487 |
| SPG11 | AK025092 | 0.1565062 | 0.0136 | -2.5223259 | -3.130909 | -0.44335755 |
| KATNBL1 | AK021466 | 0.2746695 | 0.0435 | -2.0551246 | -4.026561 | -0.44420285 |
| DLG4 | NM_001365 | 0.0790106 | 0.00337 | -3.0375146 | -1.850918 | -0.4442684 |
| TMCC1 | AB018322 | 0.0042569 | 9.51E-06 | -4.7148507 | 3.316223 | -0.44443444 |
| SPA17 | NM_017425 | 0.2493175 | 0.036 | -2.1371929 | -3.845586 | -0.4447028 |
| THBS4 | NM_003248 | 0.129688 | 0.00899 | -2.7051409 | -2.638832 | -0.44493854 |
| BIRC5 | AF086186 | 0.0868362 | 0.00408 | -2.9684402 | -2.017889 | -0.4460923 |
| PODNL1 | AK027100 | 0.0717808 | 0.0028 | -3.1106086 | -1.678519 | -0.44612719 |
| ANKRD13A | AF155103 | 0.1866425 | 0.0194 | -2.3867262 | -3.413847 | -0.44616782 |
| CD226 | NM_006566 | 0.1172969 | 0.00744 | -2.7897912 | -2.439414 | -0.44645287 |
| HSD17B1 | NM_000413 | 0.2278065 | 0.0297 | -2.2315499 | -3.485553 | -0.44649047 |
| KMT2C | AF264750 | 0.1314749 | 0.00936 | -2.6599556 | -2.806968 | -0.44727714 |
| GABPB2 | AF143872 | 0.054419 | 0.00149 | -3.2984337 | -1.170197 | -0.44749337 |
| S100G | NM_004057 | 0.2475225 | 0.0354 | -2.1527292 | -3.704388 | -0.44758974 |
| ABI1 | NM_005470 | 0.0607189 | 0.00189 | -3.2094265 | -1.411478 | -0.44759473 |
| GNG7 | NM_005145 | 0.1360448 | 0.0102 | -2.6501128 | -2.763452 | -0.44808179 |
| TMEM185B | AK024632 | 0.1064568 | 0.00615 | -2.8115931 | -2.444715 | -0.44814405 |
| FMO1 | NM_002021 | 0.2437347 | 0.034 | -2.1808175 | -3.63551 | -0.44887394 |
| LOC105375531 | AK000794 | 0.1158476 | 0.00726 | -2.7508507 | -2.581836 | -0.44936716 |
| SLC44A2 | NM_020428 | 0.1619244 | 0.0143 | -2.5016756 | -3.157401 | -0.45006848 |
| GNL3 | NM_014366 | 0.0996693 | 0.00539 | -2.8497259 | -2.373111 | -0.45035818 |
| ANKRD33B | AK023999 | 0.0879057 | 0.00421 | -2.9615321 | -2.049036 | -0.45044626 |
| ABHD5 | AF007132 | 0.2522172 | 0.037 | -2.1200141 | -3.96664 | -0.45073096 |
| ANO3 | X81896 | 0.2498502 | 0.0363 | -2.1461925 | -3.673751 | -0.45075067 |
| CNNM1 | NM_020348 | 0.0340707 | 0.000604 | -3.5582124 | -0.427868 | -0.45136858 |
| N4BP2L1 | U50528 | 0.0615754 | 0.00195 | -3.2315253 | -1.364586 | -0.45144376 |
| 8-Mar | AK023636 | 0.2088863 | 0.0245 | -2.2927676 | -3.599519 | -0.4517966 |
| LRP10 | AF131760 | 0.2777453 | 0.0445 | -2.0373556 | -4.147812 | -0.45235195 |
| USP25 | NM_013396 | 0.1681304 | 0.0155 | -2.4692909 | -3.250207 | -0.45236286 |
| MAP2K3 | NM_002756 | 0.1959255 | 0.0215 | -2.3397295 | -3.520786 | -0.45255871 |
| LINC00302 | AF005082 | 0.2611378 | 0.0396 | -2.0987515 | -3.914752 | -0.45304347 |
| DIP2B | AB040896 | 0.1540171 | 0.0132 | -2.5395243 | -3.025174 | -0.45438579 |
| SIGLEC1 | AK024462 | 0.2139928 | 0.0257 | -2.278188 | -3.598107 | -0.45446922 |
| SERINC3 | NM_006811 | 0.1419132 | 0.0111 | -2.5895434 | -3.001907 | -0.45482347 |
| ANKRD44 | AL133087 | 0.2707221 | 0.0422 | -2.0636698 | -4.062154 | -0.45496891 |
| TMCC3 | AB032971 | 0.0812343 | 0.00351 | -3.0051411 | -1.950766 | -0.4550817 |
| N4BP2L2 | NM_014887 | 0.1450954 | 0.0116 | -2.5718448 | -3.044048 | -0.45540575 |
| ERN1 | NM_001433 | 0.1519057 | 0.0129 | -2.5493597 | -3.015925 | -0.45549337 |
| GPR37 | NM_005302 | 0.0866784 | 0.00404 | -2.9841852 | -1.968388 | -0.45563496 |
| UIMC1 | NM_016290 | 0.2083515 | 0.0244 | -2.2916406 | -3.606285 | -0.45566034 |
| FMNL1 | AJ008117 | 0.2336615 | 0.0313 | -2.1937876 | -3.741345 | -0.45698898 |
| DDX17 | NM_006386 | 0.0513886 | 0.00125 | -3.3255912 | -1.093394 | -0.45742094 |
| LEMD3 | NM_014319 | 0.0405229 | 0.000832 | -3.5027284 | -0.640656 | -0.45759576 |
| B9D1 | AL137568 | 0.0457732 | 0.00104 | -3.4076218 | -0.871754 | -0.45772115 |
| LOC100508226///MAGI1-IT1///MAGI1 | AL050129 | 0.1988542 | 0.0223 | -2.3530863 | -3.284983 | -0.45788083 |
| PPP1R12A | NM_002480 | 0.06392 | 0.00211 | -3.1770239 | -1.480343 | -0.45813591 |
| PXN | NM_002859 | 0.2920206 | 0.0492 | -1.9932805 | -4.204549 | -0.45813693 |
| KLK12 | NM_019598 | 0.151167 | 0.0128 | -2.5418338 | -3.099383 | -0.45909011 |
| CHRNG | NM_005199 | 0.2340873 | 0.0314 | -2.2125629 | -3.539965 | -0.45915205 |
| FBXL20 | AK024690 | 0.1462109 | 0.0119 | -2.5761013 | -2.978686 | -0.45940175 |
| WNT10B | NM_003394 | 0.282682 | 0.0462 | -2.022352 | -4.155707 | -0.45973267 |
| NPTN-IT1 | AL360198 | 0.0812343 | 0.0035 | -3.0377421 | -1.85824 | -0.45974552 |
| SNN | NM_003498 | 0.097855 | 0.00516 | -2.8703898 | -2.298264 | -0.45979152 |
| KRT14 | NM_000526 | 0.0838311 | 0.00379 | -2.9951063 | -1.978094 | -0.46038621 |
| PKP4 | NM_003628 | 0.1697769 | 0.0158 | -2.4712149 | -3.185809 | -0.46121388 |
| PILRA | NM_013439 | 0.2473686 | 0.0353 | -2.1343488 | -3.96652 | -0.46232567 |
| ERGIC1 | AB033007 | 0.1965625 | 0.0218 | -2.3356223 | -3.528781 | -0.46239765 |
| GRAPL | AK000842 | 0.2567844 | 0.0383 | -2.1260847 | -3.751606 | -0.46247949 |
| FAM172A | AF070617 | 0.1013524 | 0.00561 | -2.8607309 | -2.274271 | -0.46384507 |
| UBE2D3 | NM_003340 | 0.1357279 | 0.0101 | -2.6246524 | -2.920935 | -0.4639262 |
| LINC00588 | AL080200 | 0.1314749 | 0.00938 | -2.6830454 | -2.670599 | -0.46424497 |
| YPEL5 | NM_016061 | 0.1700117 | 0.0159 | -2.4553277 | -3.295623 | -0.46555401 |
| MEGF6 | AB011539 | 0.0580471 | 0.0017 | -3.2397758 | -1.34179 | -0.46615628 |
| PPCDC | AF182419 | 0.11382 | 0.00701 | -2.7666936 | -2.562677 | -0.46629708 |
| SCLY | NM_016510 | 0.0439456 | 0.00097 | -3.4211742 | -0.818373 | -0.46686218 |
| DGCR12 | L77562 | 0.1212715 | 0.00794 | -2.7378612 | -2.573516 | -0.46803399 |
| CYHR1 | AB007965 | 0.0623547 | 0.00202 | -3.1782637 | -1.505076 | -0.46805718 |
| P2RY10 | NM_014499 | 0.0924658 | 0.00466 | -2.9398308 | -2.088463 | -0.46908848 |
| WASF2 | AB026542 | 0.1789884 | 0.0176 | -2.4155693 | -3.382842 | -0.4695692 |
| FAM126B | AK001843 | 0.2206434 | 0.0277 | -2.2359038 | -3.772862 | -0.47025236 |
| RASL12 | NM_016563 | 0.2777385 | 0.0444 | -2.0558091 | -3.875563 | -0.47034228 |
| IL17B | NM_014443 | 0.023401 | 0.000292 | -3.8223163 | 0.263376 | -0.47038158 |
| PGLYRP4 | NM_020393 | 0.0515912 | 0.00126 | -3.3787086 | -0.99819 | -0.47069976 |
| PLCG2 | NM_002661 | 0.0543013 | 0.00141 | -3.2970086 | -1.161293 | -0.47083841 |
| CHMP1B | NM_020412 | 0.0970567 | 0.00508 | -2.8682878 | -2.31749 | -0.47112204 |
| PGPEP1 | NM_017712 | 0.0677732 | 0.00246 | -3.1099496 | -1.685291 | -0.47156725 |
| PCNX1 | AB018348 | 0.2801049 | 0.0452 | -2.031045 | -4.103722 | -0.47209459 |
| ADCY4 | AF086230 | 0.1302889 | 0.00918 | -2.677374 | -2.738732 | -0.47314905 |
| PLXDC2 | AL080095 | 0.2642119 | 0.0405 | -2.0831868 | -4.009843 | -0.47327658 |
| NFATC2 | NM_012340 | 0.1084161 | 0.00636 | -2.7996547 | -2.455063 | -0.47351515 |
| PIK3CG | NM_002649 | 0.191213 | 0.0205 | -2.3689931 | -3.413455 | -0.47415316 |
| CEACAM3 | NM_001815 | 0.2278609 | 0.0298 | -2.2103846 | -3.803624 | -0.47435503 |
| RHCG | NM_016321 | 0.0869908 | 0.00413 | -2.9752891 | -2.015888 | -0.47508573 |
| HOXC12 | X99631 | 0.111606 | 0.00672 | -2.783094 | -2.523233 | -0.47508801 |
| ONECUT1 | U96173 | 0.0178752 | 0.000186 | -3.9175837 | 0.644095 | -0.47560173 |
| TM9SF3 | NM_020123 | 0.0946907 | 0.0049 | -2.8827726 | -2.281498 | -0.47647878 |
| PIK3R4 | AK025026 | 0.1842801 | 0.0188 | -2.4227323 | -3.224836 | -0.47682274 |
| SLC12A6 | NM_005135 | 0.2078456 | 0.0243 | -2.2904813 | -3.63721 | -0.47815546 |
| RAPGEF2 | NM_014247 | 0.1184467 | 0.00762 | -2.7394085 | -2.601684 | -0.47873173 |
| TRIM39 | AB046381 | 0.0275018 | 0.000395 | -3.7254164 | -0.000333 | -0.47933975 |
| LBR | NM_002296 | 0.2872229 | 0.0479 | -2.0036898 | -4.213649 | -0.47965008 |
| LINC00893 | U66044 | 0.1469313 | 0.012 | -2.5878292 | -2.876044 | -0.4799249 |
| ZZEF1 | AB007859 | 0.0861869 | 0.00399 | -2.9793217 | -2.003348 | -0.48041753 |
| PIK3CA | NM_006218 | 0.0585081 | 0.00177 | -3.2363212 | -1.326566 | -0.48046858 |
| PIK3R5 | NM_014308 | 0.2419447 | 0.0336 | -2.16589 | -3.833128 | -0.48054666 |
| HCN1 | AF064876 | 0.1745098 | 0.0168 | -2.4422185 | -3.273587 | -0.4806781 |
| ARNT | AL137290 | 0.1268613 | 0.00855 | -2.7014441 | -2.672047 | -0.48318057 |
| SRGAP3 | AL390174 | 0.0734207 | 0.00294 | -3.0853949 | -1.719579 | -0.48382689 |
| CD79B | M89957 | 0.0622293 | 0.00202 | -3.2395484 | -1.382057 | -0.48411177 |
| STRN | AF085351 | 0.1013184 | 0.00558 | -2.8874485 | -2.221334 | -0.48422928 |
| TMEM92-AS1 | AK026439 | 0.0461998 | 0.00106 | -3.418438 | -0.851932 | -0.48423786 |
| AOC1 | NM_001091 | 0.0861216 | 0.00397 | -2.9586436 | -2.090194 | -0.48463927 |
| TMEM55A | AL359591 | 0.1001873 | 0.00549 | -2.8610148 | -2.294133 | -0.48558808 |
| STX3 | NM_004177 | 0.2339569 | 0.0314 | -2.185149 | -3.863461 | -0.48584789 |
| MSN | NM_002444 | 0.1745098 | 0.0168 | -2.4356523 | -3.326777 | -0.48613013 |
| SETD1B | AB028999 | 0.150826 | 0.0127 | -2.5466344 | -3.056507 | -0.486229 |
| TMCC2 | AB007950 | 0.1346767 | 0.00985 | -2.6495243 | -2.791243 | -0.48638726 |
| VSIG2 | NM_014312 | 0.0697057 | 0.00263 | -3.129988 | -1.61611 | -0.48642092 |
| PLEKHG3 | AB011171 | 0.0845947 | 0.00385 | -2.9715192 | -2.04335 | -0.48736526 |
| RDH8 | NM_015725 | 0.1302889 | 0.00915 | -2.7185418 | -2.600147 | -0.48815523 |
| ATG16L2 | AK024423 | 0.2186316 | 0.0272 | -2.2477191 | -3.719007 | -0.48835804 |
| PRSS16 | NM_005865 | 0.1848127 | 0.019 | -2.4006179 | -3.322541 | -0.48876425 |
| PIGB | NM_004855 | 0.2186316 | 0.0271 | -2.2480439 | -3.682331 | -0.48893894 |
| CHRNA4 | X89744 | 0.1866856 | 0.0195 | -2.4160066 | -3.228315 | -0.48909159 |
| FEM1C | AK025265 | 0.0513886 | 0.00125 | -3.3508177 | -1.015763 | -0.49135751 |
| CLK1 | NM_004071 | 0.0461998 | 0.00106 | -3.3813649 | -0.936376 | -0.49147449 |
| RIPK1 | NM_003804 | 0.0640443 | 0.00215 | -3.1662557 | -1.526054 | -0.49157417 |
| LOR | NM_000427 | 0.1667366 | 0.0152 | -2.5053619 | -3.028854 | -0.49195141 |
| CNTF | NM_000614 | 0.1070351 | 0.00621 | -2.8288644 | -2.363782 | -0.492025 |
| RNF24 | NM_007219 | 0.2327579 | 0.031 | -2.1889691 | -3.845204 | -0.49265337 |
| NCKAP5L | AB046822 | 0.1802222 | 0.0179 | -2.4260228 | -3.281115 | -0.49289237 |
| SH2B2 | AB000520 | 0.1585197 | 0.0138 | -2.51766 | -3.129773 | -0.49373471 |
| MON2 | AB028963 | 0.156062 | 0.0135 | -2.5553613 | -2.909594 | -0.494378 |
| KLHDC8B | AK002164 | 0.065773 | 0.00228 | -3.1656084 | -1.527154 | -0.49475888 |
| C8B | NM_000066 | 0.1467802 | 0.0119 | -2.5912992 | -2.882074 | -0.49514677 |
| CMKLR1 | NM_004072 | 0.0899319 | 0.00443 | -2.9490464 | -2.053098 | -0.49614986 |
| ARHGAP26 | NM_015071 | 0.2237543 | 0.0287 | -2.2234932 | -3.772589 | -0.49628144 |
| LOC100131541 | AK022121 | 0.0541996 | 0.00139 | -3.333407 | -1.090659 | -0.49814673 |
| LOC401052 | AK022260 | 0.0890728 | 0.00434 | -2.9357108 | -2.121799 | -0.49871722 |
| SIRPB1 | NM_006065 | 0.2620335 | 0.0398 | -2.0879187 | -4.031705 | -0.49897945 |
| AKNA | AK024431 | 0.2222798 | 0.0281 | -2.2296586 | -3.763412 | -0.49957614 |
| ARHGEF12 | AL080067 | 0.0640443 | 0.00215 | -3.2127702 | -1.435838 | -0.49968714 |
| ELMO1 | NM_014800 | 0.0323271 | 0.00055 | -3.5999693 | -0.322494 | -0.49976819 |
| IRS2 | NM_003749 | 0.2153734 | 0.026 | -2.2613184 | -3.709323 | -0.50144241 |
| YTHDC1 | AL049340 | 0.1250326 | 0.00837 | -2.7149411 | -2.653414 | -0.5024802 |
| CREB1 | M34356 | 0.0273904 | 0.000392 | -3.7017532 | -0.010483 | -0.50307131 |
| NFE2L1 | U08853 | 0.0580471 | 0.00169 | -3.2476089 | -1.298253 | -0.50338114 |
| ELOVL5 | AF111849 | 0.0660673 | 0.00232 | -3.1278769 | -1.642458 | -0.50357032 |
| ATP1B4 | NM_012069 | 0.1373219 | 0.0104 | -2.6539352 | -2.715069 | -0.50374897 |
| NPBWR1 | NM_005285 | 0.1015888 | 0.00565 | -2.8413843 | -2.362715 | -0.50415018 |
| PACSIN1 | AB037800 | 0.0713541 | 0.00277 | -3.0857894 | -1.74257 | -0.50509158 |
| SIRPG | NM_018556 | 0.1626794 | 0.0145 | -2.4958873 | -3.191374 | -0.50529488 |
| PLEKHM1 | AB002354 | 0.2472731 | 0.0353 | -2.1432381 | -3.867003 | -0.5052989 |
| DAZL | NM_001351 | 0.2498502 | 0.0362 | -2.1460951 | -3.716291 | -0.50620292 |
| CRYGB | NM_005210 | 0.0586227 | 0.00179 | -3.2857447 | -1.27772 | -0.50622025 |
| MBNL1 | AK021883 | 0.1646272 | 0.0149 | -2.499169 | -3.113611 | -0.50680073 |
| ZRANB1 | AJ252060 | 0.0608758 | 0.00191 | -3.2078716 | -1.437097 | -0.50707612 |
| FFAR1 | NM_005303 | 0.0651194 | 0.00221 | -3.2203753 | -1.450287 | -0.50717081 |
| OPRD1 | NM_000911 | 0.0608758 | 0.00191 | -3.2646178 | -1.329764 | -0.50731131 |
| FBLIM1 | AL133035 | 0.0996693 | 0.00538 | -2.9188695 | -2.173299 | -0.50741396 |
| CYP39A1 | NM_016593 | 0.0332742 | 0.00058 | -3.6074983 | -0.337853 | -0.50771464 |
| MBNL1 | AJ227863 | 0.1848142 | 0.019 | -2.3861617 | -3.458875 | -0.50885024 |
| ZNF239 | NM_005674 | 0.1711364 | 0.0161 | -2.4709441 | -3.14831 | -0.5091822 |
| U2AF1 | AK022152 | 0.1188131 | 0.00771 | -2.7623704 | -2.520681 | -0.51007463 |
| MYD88 | NM_002468 | 0.1866856 | 0.0195 | -2.3759901 | -3.470389 | -0.51032652 |
| UNC5B | AL049370 | 0.1519057 | 0.0129 | -2.5493498 | -2.94924 | -0.51126266 |
| BMP2K | AK021725 | 0.0709068 | 0.00273 | -3.0951442 | -1.716724 | -0.5122932 |
| CFLAR | NM_003879 | 0.278402 | 0.0447 | -2.0350978 | -4.138006 | -0.51256396 |
| G3BP1 | X78262 | 0.1633771 | 0.0146 | -2.5330622 | -2.994839 | -0.51305999 |
| GPSM1 | AL117478 | 0.1892138 | 0.02 | -2.369836 | -3.454634 | -0.51349201 |
| TDP2 | NM_016614 | 0.2101009 | 0.0248 | -2.2800229 | -3.671561 | -0.51392131 |
| ALDH2 | NM_000690 | 0.1040257 | 0.00589 | -2.839205 | -2.320501 | -0.5142336 |
| RAD23A | NM_005053 | 0.1866425 | 0.0194 | -2.3988312 | -3.29819 | -0.51445954 |
| FCF1 | NM_015962 | 0.0974784 | 0.00512 | -2.9039878 | -2.169643 | -0.51489322 |
| GFRA3 | AF086292 | 0.0939476 | 0.0048 | -2.9439903 | -2.096215 | -0.51518593 |
| TGFBR2 | NM_003242 | 0.1482563 | 0.0123 | -2.5574352 | -3.053895 | -0.51528509 |
| PFN1 | NM_005022 | 0.0829795 | 0.00369 | -2.9770618 | -2.038529 | -0.51565775 |
| VPS50 | NM_017667 | 0.0156667 | 0.000152 | -4.016285 | 0.841232 | -0.51630955 |
| PHF23 | AK026537 | 0.0868362 | 0.00407 | -2.9748615 | -1.993945 | -0.51678077 |
| CAMK1D | AL137430 | 0.1910941 | 0.0205 | -2.3627746 | -3.487668 | -0.51707722 |
| FLOT2 | NM_004475 | 0.2709663 | 0.0423 | -2.0579059 | -4.112446 | -0.51723956 |
| NCKAP1L | NM_005337 | 0.1646272 | 0.0149 | -2.4964885 | -3.119215 | -0.51769516 |
| SPAG6 | NM_012443 | 0.216106 | 0.0263 | -2.2656493 | -3.613718 | -0.51818828 |
| C1QTNF5///MFRP | AL110261 | 0.0869908 | 0.00413 | -2.9700253 | -2.045471 | -0.51954858 |
| CAMK2D | AF071569 | 0.1298985 | 0.00908 | -2.6765587 | -2.738937 | -0.52002111 |
| ZC3HAV1 | NM_020119 | 0.0996693 | 0.0054 | -2.8674968 | -2.250147 | -0.52096175 |
| NECAB2 | AF070637 | 0.0894193 | 0.00437 | -2.9308407 | -2.163941 | -0.52245927 |
| UBE2D3 | AF116686 | 0.257798 | 0.0387 | -2.1074431 | -3.884062 | -0.52306758 |
| SLC7A4 | NM_004173 | 0.0629918 | 0.00207 | -3.1836523 | -1.472268 | -0.52343581 |
| HNRNPM | AK022050 | 0.131205 | 0.00928 | -2.6815238 | -2.680036 | -0.52523729 |
| IFI16 | NM_005531 | 0.1971363 | 0.0219 | -2.3289239 | -3.581339 | -0.52581389 |
| OSM | NM_020530 | 0.2825245 | 0.0459 | -2.0236181 | -4.181998 | -0.52619289 |
| ABHD2 | NM_007011 | 0.1502516 | 0.0125 | -2.5575709 | -3.030022 | -0.52624261 |
| PRKD2 | NM_016457 | 0.1296036 | 0.00897 | -2.6746075 | -2.772671 | -0.52637302 |
| CPQ | NM_006102 | 0.088015 | 0.00423 | -2.9351787 | -2.149328 | -0.5271584 |
| FRY | U50534 | 0.0373686 | 0.000713 | -3.5283711 | -0.537323 | -0.52758294 |
| GVINP1 | AK023435 | 0.2557852 | 0.0379 | -2.1215135 | -3.835693 | -0.52856182 |
| HIST1H4D | NM_003539 | 0.0883494 | 0.00427 | -2.9729482 | -2.02014 | -0.52872264 |
| TMEM140 | NM_018295 | 0.1646272 | 0.0148 | -2.4841429 | -3.208747 | -0.52901199 |
| STAT3 | NM_003150 | 0.1119967 | 0.00675 | -2.7736639 | -2.544692 | -0.52923918 |
| SH2B3 | NM_005475 | 0.1064568 | 0.00613 | -2.8291833 | -2.385906 | -0.52960003 |
| ALKBH5 | AK000315 | 0.2117172 | 0.0252 | -2.278794 | -3.64449 | -0.52962902 |
| NET1 | NM_005863 | 0.2510017 | 0.0367 | -2.1206147 | -3.987157 | -0.52973161 |
| CDKL5 | NM_003159 | 0.1149191 | 0.00715 | -2.7685575 | -2.516789 | -0.5312557 |
| NT5C2 | NM_012229 | 0.0979995 | 0.00519 | -2.8643497 | -2.319965 | -0.53192934 |
| MIR3142HG | AL389942 | 0.0921278 | 0.00459 | -2.9601051 | -2.065945 | -0.53291305 |
| MAB21L2 | NM_006439 | 0.118903 | 0.00773 | -2.7709574 | -2.469567 | -0.53302899 |
| MAGIX | AK025340 | 0.0852088 | 0.0039 | -2.9916277 | -1.971367 | -0.53323892 |
| IVNS1ABP | AK023363 | 0.1887147 | 0.0199 | -2.3930525 | -3.296456 | -0.53398276 |
| SLC34A1 | NM_003052 | 0.1830103 | 0.0184 | -2.4398878 | -3.133744 | -0.53479339 |
| NOTCH1 | NM_017617 | 0.2387091 | 0.0327 | -2.1716818 | -3.869848 | -0.53490051 |
| MARCKS | NM_002356 | 0.1018958 | 0.00569 | -2.8284447 | -2.426033 | -0.53521276 |
| FOXO3 | NM_001455 | 0.0133446 | 0.000102 | -4.076706 | 1.179808 | -0.53532626 |
| NINJ1 | NM_004148 | 0.1242184 | 0.0083 | -2.6977589 | -2.717675 | -0.53608106 |
| PRPH | NM_006262 | 0.1658084 | 0.015 | -2.488884 | -3.135412 | -0.53613574 |
| CRACR2A | AK021694 | 0.04338 | 0.000937 | -3.4783242 | -0.736625 | -0.53635956 |
| HHATL | AB042554 | 0.0989871 | 0.00531 | -2.8988136 | -2.176302 | -0.53658107 |
| SUPT20H | AK021457 | 0.108788 | 0.00641 | -2.8038519 | -2.4578 | -0.53667207 |
| RAPGEF1 | NM_005312 | 0.0612578 | 0.00193 | -3.2131646 | -1.382441 | -0.53804043 |
| RRAGA | NM_006570 | 0.0133459 | 0.000103 | -4.0590337 | 1.152714 | -0.53900798 |
| DNHD1 | AL137619 | 0.2432755 | 0.0339 | -2.1580106 | -3.84365 | -0.53910924 |
| KIAA1257 | AB033083 | 0.0879057 | 0.00422 | -2.9613201 | -2.05761 | -0.53922128 |
| PLCB2 | NM_004573 | 0.2579868 | 0.0389 | -2.0973804 | -4.003156 | -0.54068117 |
| PAK1 | NM_002576 | 0.1667366 | 0.0152 | -2.4869822 | -3.174445 | -0.54074344 |
| TET1///KMT2A | AF272373 | 0.0461998 | 0.00106 | -3.4041237 | -0.872723 | -0.54080664 |
| MKNK2 | Z25424 | 0.0531928 | 0.00135 | -3.3052234 | -1.157477 | -0.54107224 |
| SH3BP2 | L37198 | 0.1394995 | 0.0108 | -2.6104103 | -2.921249 | -0.54155697 |
| MAP3K14 | AJ008144 | 0.1000418 | 0.00545 | -2.8755144 | -2.244677 | -0.5441048 |
| PSMD6-AS2 | AK023371 | 0.1888292 | 0.0199 | -2.3821712 | -3.373107 | -0.54497572 |
| PPP4R1 | NM_005134 | 0.0901352 | 0.00445 | -2.9172146 | -2.194767 | -0.54595805 |
| PLEKHA2 | AF286164 | 0.0762261 | 0.00316 | -3.0399635 | -1.826195 | -0.54663229 |
| IL1RL2 | NM_003854 | 0.1253368 | 0.00841 | -2.7300309 | -2.546834 | -0.54682668 |
| FAM101B | AF085987 | 0.2554574 | 0.0379 | -2.1062847 | -4.016995 | -0.54697368 |
| R3HCC1 | AL050297 | 0.0143077 | 0.000118 | -4.031603 | 1.040325 | -0.54818337 |
| KLHL24 | AK024270 | 0.0111961 | 7.68E-05 | -4.1669104 | 1.437255 | -0.54929841 |
| SLC4A3 | U05597 | 0.0763139 | 0.00318 | -3.0623725 | -1.789348 | -0.54975254 |
| ELL | AF157562 | 0.0639603 | 0.00212 | -3.1684084 | -1.524409 | -0.55178524 |
| CHRNA4 | NM_000744 | 0.0660238 | 0.00229 | -3.1605944 | -1.55529 | -0.55242881 |
| RUFY2 | NM_017987 | 0.0552445 | 0.00156 | -3.2801003 | -1.220213 | -0.55252699 |
| CD300A | AF020314 | 0.2606622 | 0.0394 | -2.0877247 | -4.06912 | -0.55308322 |
| MAP3K3 | NM_002401 | 0.065408 | 0.00222 | -3.1430825 | -1.595418 | -0.55649897 |
| DHFR | NM_000791 | 0.2478424 | 0.0354 | -2.139052 | -3.905805 | -0.55721066 |
| DDX58 | NM_014314 | 0.2700169 | 0.0419 | -2.073985 | -3.973481 | -0.55751623 |
| ALPK1 | AK026323 | 0.2842376 | 0.047 | -2.0164302 | -4.124867 | -0.55836017 |
| DDX60 | NM_017631 | 0.2343631 | 0.0316 | -2.1891877 | -3.804089 | -0.5617424 |
| C7orf43 | NM_018275 | 0.088427 | 0.00427 | -2.9508637 | -2.068178 | -0.56294829 |
| DOCK5 | AK026663 | 0.172848 | 0.0164 | -2.4480413 | -3.296203 | -0.5638103 |
| SF3B4 | NM_005850 | 0.0378116 | 0.000729 | -3.4895692 | -0.608574 | -0.56395924 |
| SCN10A | NM_006514 | 0.0667358 | 0.00238 | -3.1891449 | -1.519696 | -0.56400103 |
| PRF1 | NM_005041 | 0.1188517 | 0.00772 | -2.7347096 | -2.592172 | -0.56435015 |
| ATG4B | AL050288 | 0.0827977 | 0.00367 | -2.9801936 | -2.030951 | -0.5653199 |
| LOC148696 | AL137491 | 0.2702815 | 0.042 | -2.0782297 | -3.858218 | -0.56587296 |
| STAT2 | NM_005419 | 0.1842801 | 0.0187 | -2.4067622 | -3.295863 | -0.56603561 |
| COMMD6 | AF086081 | 0.0708709 | 0.00272 | -3.1316632 | -1.633036 | -0.566454 |
| CPPED1 | NM_018340 | 0.2175913 | 0.0268 | -2.250029 | -3.743048 | -0.56762349 |
| TAGLN2 | NM_003564 | 0.0585081 | 0.00176 | -3.2586643 | -1.291754 | -0.56786311 |
| AFF1 | NM_005935 | 0.0756724 | 0.00308 | -3.0631826 | -1.795072 | -0.56841647 |
| PARP16 | NM_017851 | 0.077315 | 0.00326 | -3.0644946 | -1.784612 | -0.56870602 |
| CASP4 | NM_001225 | 0.2073018 | 0.0241 | -2.2903045 | -3.663294 | -0.57058736 |
| TSPOAP1-AS1 | AK000271 | 0.1319953 | 0.00943 | -2.6923738 | -2.652366 | -0.57151053 |
| CLEC7A | AY009090 | 0.2634252 | 0.0401 | -2.0808083 | -4.080228 | -0.57165204 |
| TNFRSF1A | NM_001065 | 0.1332223 | 0.00959 | -2.6445556 | -2.863626 | -0.57220311 |
| LRRFIP1 | AK023938 | 0.0063444 | 2.72E-05 | -4.4830678 | 2.365216 | -0.572416 |
| PRUNE2 | AB002365 | 0.113181 | 0.00693 | -2.7677918 | -2.541074 | -0.57343198 |
| RAX | NM_013435 | 0.0677732 | 0.00247 | -3.1632236 | -1.551616 | -0.57518813 |
| KRT1 | NM_006121 | 0.2091832 | 0.0246 | -2.3085587 | -3.448759 | -0.57606922 |
| AVPR2 | NM_000054 | 0.1394995 | 0.0108 | -2.6224714 | -2.843723 | -0.57621535 |
| KLK5 | NM_012427 | 0.1182861 | 0.00756 | -2.7773172 | -2.462419 | -0.57695451 |
| TADA2B | AK026299 | 0.1497064 | 0.0124 | -2.5628441 | -2.961701 | -0.57696995 |
| PTPRE | NM_006504 | 0.1839197 | 0.0186 | -2.3938136 | -3.441608 | -0.57742339 |
| SLC6A6 | NM_003043 | 0.216106 | 0.0264 | -2.2602412 | -3.691597 | -0.57941223 |
| IPCEF1 | AB007863 | 0.1894734 | 0.0201 | -2.3853646 | -3.35119 | -0.58020219 |
| SLC10A1 | NM_003049 | 0.216106 | 0.0264 | -2.2649059 | -3.599933 | -0.58222677 |
| DDX5 | AL133585 | 0.054419 | 0.00143 | -3.318283 | -1.134973 | -0.5830707 |
| RGS1 | NM_002922 | 0.0766741 | 0.00322 | -3.042053 | -1.871698 | -0.58408238 |
| EDEM1 | NM_014674 | 0.0245148 | 0.000332 | -3.769393 | 0.148199 | -0.58441523 |
| KCNN1 | NM_002248 | 0.1602764 | 0.014 | -2.5270499 | -2.990222 | -0.58496496 |
| GRAMD1C | NM_017577 | 0.0045092 | 1.14E-05 | -4.7001484 | 3.14143 | -0.58577479 |
| TCF7L1 | U15553 | 0.1434156 | 0.0113 | -2.586496 | -2.966868 | -0.58578118 |
| ROPN1L | AK026656 | 0.0815565 | 0.00356 | -3.0005515 | -1.962562 | -0.58617732 |
| PART1 | AF070547 | 0.0640443 | 0.00215 | -3.2196783 | -1.435617 | -0.58625245 |
| B3GALT2 | NM_003783 | 0.0391347 | 0.000788 | -3.5701027 | -0.639069 | -0.58829725 |
| ZNF208 | D70833 | 0.033029 | 0.00057 | -3.5621333 | -0.395209 | -0.58902354 |
| FMNL1 | AJ008129 | 0.1462109 | 0.0118 | -2.568819 | -3.028909 | -0.58905128 |
| TINAG | NM_014464 | 0.1013524 | 0.00561 | -2.879002 | -2.220623 | -0.59061536 |
| ACPP | NM_001099 | 0.0185023 | 0.000197 | -3.9846127 | 0.579513 | -0.59076363 |
| ZNF551 | X52354 | 0.0386078 | 0.000754 | -3.5284844 | -0.559429 | -0.59218504 |
| STEAP4 | AK026806 | 0.2934012 | 0.0497 | -1.9937225 | -4.15912 | -0.59283761 |
| ARHGAP15 | NM_018460 | 0.2196635 | 0.0275 | -2.240257 | -3.750623 | -0.59313656 |
| SLC31A2 | NM_001860 | 0.1989582 | 0.0224 | -2.3242916 | -3.563751 | -0.59374874 |
| KIAA0226L | AK025215 | 0.1071441 | 0.00624 | -2.8022766 | -2.474898 | -0.5982164 |
| LOC101928524 | AL137400 | 0.0237469 | 0.000304 | -3.8166589 | 0.234772 | -0.59907613 |
| GPAA1 | NM_003801 | 0.0657251 | 0.00226 | -3.1615979 | -1.527218 | -0.59999417 |
| IL6R | NM_000565 | 0.1646272 | 0.0149 | -2.480955 | -3.238745 | -0.60015993 |
| ASIC5 | AJ252011 | 0.2432755 | 0.0339 | -2.1910111 | -3.619766 | -0.60348977 |
| HECW2 | AB037722 | 0.0257458 | 0.000357 | -3.7236469 | 0.065712 | -0.60445184 |
| RABGAP1L | NM_014857 | 0.008699 | 4.57E-05 | -4.3652449 | 1.906827 | -0.60546089 |
| NR4A3 | S81242 | 0.2314641 | 0.0307 | -2.1945808 | -3.844631 | -0.60685006 |
| SMG6 | NM_017575 | 0.0143235 | 0.000126 | -4.0605704 | 1.004051 | -0.60767041 |
| ICAM2 | NM_000873 | 0.1276123 | 0.00872 | -2.682892 | -2.780394 | -0.60790518 |
| CBL | NM_005188 | 0.054419 | 0.00151 | -3.3201618 | -1.14861 | -0.60833021 |
| CHST11 | NM_018413 | 0.027086 | 0.000382 | -3.7018043 | -0.003216 | -0.60843235 |
| PLAGL1 | NM_006718 | 0.1967942 | 0.0218 | -2.3426003 | -3.443027 | -0.60855286 |
| GJB6 | NM_006783 | 0.0020184 | 2.60E-06 | -5.0824127 | 4.461136 | -0.60906792 |
| B2M | NM_004048 | 0.108788 | 0.00639 | -2.7922138 | -2.507995 | -0.60992654 |
| 7-Mar | AK023400 | 0.0157686 | 0.000154 | -3.9993042 | 0.818779 | -0.61082209 |
| PSEN1 | NM_007318 | 0.1000418 | 0.00546 | -2.8441203 | -2.378533 | -0.61130753 |
| GLIPR2 | AJ011129 | 0.1197763 | 0.0078 | -2.7181356 | -2.688292 | -0.61135787 |
| OVGP1 | NM_002557 | 0.1331129 | 0.00957 | -2.6592782 | -2.779345 | -0.61232408 |
| NLRP1 | NM_014922 | 0.054419 | 0.0015 | -3.2840123 | -1.232075 | -0.61271658 |
| NEK7 | AL080111 | 0.0412635 | 0.00086 | -3.4552922 | -0.737327 | -0.61296644 |
| LRCH4 | NM_002319 | 0.0696501 | 0.00262 | -3.0916317 | -1.728595 | -0.61408162 |
| PTH2R | NM_005048 | 0.0738979 | 0.00298 | -3.0809475 | -1.730664 | -0.61424938 |
| ZNF326 | AK000410 | 0.2306196 | 0.0304 | -2.197935 | -3.826472 | -0.61568923 |
| EGR3 | NM_004430 | 0.0045092 | 1.12E-05 | -4.7431318 | 3.115049 | -0.61603611 |
| EPB42 | NM_000119 | 0.0365333 | 0.000689 | -3.5430701 | -0.507738 | -0.61616727 |
| OGFRL1 | AK024732 | 0.0639603 | 0.00212 | -3.1597127 | -1.552209 | -0.61719165 |
| SBF2 | AK022478 | 0.0521155 | 0.0013 | -3.3735158 | -1.022581 | -0.61783332 |
| PLXNC1 | NM_005761 | 0.2103445 | 0.0249 | -2.2796448 | -3.66037 | -0.61796342 |
| SOD2 | D17152 | 0.271293 | 0.0425 | -2.056289 | -4.103412 | -0.61813253 |
| TFEC | AL110232 | 0.2833012 | 0.0466 | -2.0441447 | -3.848998 | -0.61815496 |
| THBD | NM_000361 | 0.1548656 | 0.0133 | -2.5312344 | -3.100818 | -0.61932312 |
| IGF2R | NM_000876 | 0.1576957 | 0.0137 | -2.511434 | -3.149184 | -0.61938674 |
| KRT37 | NM_003770 | 0.0545224 | 0.00152 | -3.3008263 | -1.169524 | -0.62023324 |
| NSDHL | NM_015922 | 0.0699709 | 0.00265 | -3.0887069 | -1.75258 | -0.62092439 |
| PBXIP1 | NM_020524 | 0.0321337 | 0.000513 | -3.5971316 | -0.296936 | -0.62236033 |
| VAMP2 | NM_014232 | 0.0143077 | 0.000124 | -4.0311357 | 1.00946 | -0.62276283 |
| FMNL1 | AJ008118 | 0.2191047 | 0.0273 | -2.2406527 | -3.765006 | -0.62392966 |
| AOX1 | NM_001159 | 0.0829795 | 0.00371 | -3.0055306 | -1.916842 | -0.62496595 |
| LCORL | AL133031 | 0.0833651 | 0.00376 | -2.9796918 | -2.030358 | -0.62531918 |
| RHBDD3 | AL050346 | 0.0906683 | 0.00449 | -2.9276427 | -2.153323 | -0.62554762 |
| EI24 | NM_004879 | 0.1269501 | 0.00858 | -2.6873058 | -2.770414 | -0.62640282 |
| LOC101927830///TMLHE-AS1 | L23867 | 0.0205228 | 0.000241 | -3.9498142 | 0.321801 | -0.62653853 |
| SEMA4D | NM_006378 | 0.1388609 | 0.0106 | -2.611361 | -2.919957 | -0.62742568 |
| CDH23 | AY010111 | 0.054321 | 0.00142 | -3.3465759 | -1.090223 | -0.62903483 |
| TLR4 | NM_003266 | 0.2248207 | 0.0289 | -2.218415 | -3.773239 | -0.63005405 |
| ZCCHC2 | AK000229 | 0.1819598 | 0.0183 | -2.4358591 | -3.185615 | -0.63127225 |
| ARNTL | NM_001178 | 0.0713714 | 0.00277 | -3.091493 | -1.742122 | -0.63197031 |
| PGAP1 | AL050078 | 0.0107374 | 6.56E-05 | -4.2048601 | 1.576267 | -0.63391529 |
| PIM2 | NM_006875 | 0.0186103 | 0.000204 | -3.8783079 | 0.547062 | -0.63588159 |
| CASS4 | NM_020356 | 0.0093078 | 5.19E-05 | -4.2777684 | 1.79053 | -0.6360011 |
| ZCCHC2 | NM_017742 | 0.1192669 | 0.00776 | -2.7447903 | -2.51272 | -0.63613313 |
| CHSY1 | NM_014918 | 0.0412635 | 0.000859 | -3.4511714 | -0.730411 | -0.63687693 |
| NPC2 | NM_006432 | 0.1863506 | 0.0193 | -2.3804318 | -3.458884 | -0.63695066 |
| CDC42SE1 | NM_020239 | 0.2034424 | 0.0232 | -2.3099857 | -3.55698 | -0.63883191 |
| ADAR | NM_001111 | 0.0143077 | 0.000125 | -4.0103503 | 0.988627 | -0.63963922 |
| GPR132 | NM_013345 | 0.097619 | 0.00514 | -2.8866488 | -2.225101 | -0.64006661 |
| BOD1L1 | AK025965 | 0.0244156 | 0.000319 | -3.7440135 | 0.141037 | -0.64291384 |
| LRMP | NM_006152 | 0.1297693 | 0.00904 | -2.6660387 | -2.82201 | -0.64446921 |
| DAZAP2 | NM_014764 | 0.0521155 | 0.0013 | -3.3144329 | -1.118547 | -0.64597333 |
| PRPH2 | NM_000322 | 0.067079 | 0.00242 | -3.1775149 | -1.533043 | -0.64967929 |
| LMBRD1 | NM_018368 | 0.0846969 | 0.00387 | -2.9755403 | -2.037548 | -0.64980693 |
| ADAM17 | NM_003183 | 0.054419 | 0.00145 | -3.3110266 | -1.145769 | -0.65021633 |
| XCR1 | NM_005283 | 0.101408 | 0.00562 | -2.8626543 | -2.273212 | -0.65026032 |
| SH3PXD2B | AB037716 | 0.0583001 | 0.00173 | -3.2244828 | -1.372673 | -0.65030245 |
| LYN | NM_002350 | 0.2636624 | 0.0402 | -2.0805952 | -4.041952 | -0.65123034 |
| MAP3K14 | AJ008141 | 0.0310763 | 0.000481 | -3.7038115 | -0.189459 | -0.65151577 |
| KCNJ15 | NM_002243 | 0.2230111 | 0.0285 | -2.2239103 | -3.788194 | -0.65177372 |
| IRX4 | NM_016358 | 0.0766741 | 0.00323 | -3.0261711 | -1.905646 | -0.6521891 |
| LOC101926913 | AK023515 | 0.1071441 | 0.00622 | -2.8092915 | -2.432824 | -0.65257121 |
| SLC10A2 | NM_000452 | 0.2037758 | 0.0234 | -2.3205852 | -3.440897 | -0.65648777 |
| IFRD1 | NM_001550 | 0.0660645 | 0.00231 | -3.1440274 | -1.594385 | -0.65708328 |
| RBMY3AP | U94386 | 0.0185338 | 0.000199 | -3.8724511 | 0.559263 | -0.65823949 |
| U2AF1 | AK023589 | 0.0111961 | 7.68E-05 | -4.1783748 | 1.437898 | -0.65853879 |
| FMNL1 | AJ008114 | 0.0332742 | 0.000581 | -3.5627063 | -0.400556 | -0.6636339 |
| TEKT2 | NM_014466 | 0.0063444 | 2.23E-05 | -4.6353888 | 2.334539 | -0.66438823 |
| SLC45A4 | AB032952 | 0.188673 | 0.0199 | -2.3895265 | -3.271439 | -0.66644922 |
| NABP1 | AK026486 | 0.2321878 | 0.0309 | -2.1976907 | -3.790499 | -0.66768609 |
| FGL2 | NM_006682 | 0.1842801 | 0.0188 | -2.3920888 | -3.441553 | -0.66976175 |
| IGF2R | S80797 | 0.0115729 | 8.11E-05 | -4.2521219 | 1.372859 | -0.67049747 |
| F2RL1 | NM_005242 | 0.0321337 | 0.000531 | -3.6322974 | -0.26025 | -0.67135844 |
| ITGA5 | NM_002205 | 0.0086874 | 4.51E-05 | -4.2812199 | 1.901897 | -0.67197481 |
| SETX | NM_015046 | 0.0276217 | 0.000403 | -3.6879057 | -0.05707 | -0.6720903 |
| IFIT3 | AF026943 | 0.2693906 | 0.0418 | -2.0687675 | -4.062583 | -0.67411391 |
| SEC14L1 | NM_003003 | 0.1137476 | 0.00699 | -2.7553744 | -2.606952 | -0.67433258 |
| PDE4B | NM_002600 | 0.0559296 | 0.00159 | -3.267777 | -1.256769 | -0.67507268 |
| WDR45 | NM_007075 | 0.0384291 | 0.000747 | -3.5602415 | -0.538449 | -0.67665476 |
| FGD3 | AK000004 | 0.1185939 | 0.00768 | -2.7248912 | -2.671891 | -0.67791539 |
| SLC9A8 | AB023156 | 0.0996693 | 0.0054 | -2.8870934 | -2.235891 | -0.67864851 |
| HCAR3 | NM_006018 | 0.2833012 | 0.0466 | -2.0179915 | -4.191099 | -0.67872671 |
| PPP1R12B | NM_002481 | 0.0457732 | 0.00104 | -3.4670974 | -0.818215 | -0.67958316 |
| RPGR | NM_000328 | 0.0218868 | 0.000265 | -3.8340037 | 0.345591 | -0.68017434 |
| HAL | NM_002108 | 0.1550215 | 0.0133 | -2.5267666 | -3.122389 | -0.68081073 |
| KLF6 | AL117595 | 0.156062 | 0.0135 | -2.5188751 | -3.142616 | -0.68092917 |
| VASP | NM_003370 | 0.1844438 | 0.0189 | -2.3878432 | -3.444836 | -0.68196275 |
| BBIP1 | AK025724 | 0.271293 | 0.0425 | -2.0806861 | -3.722451 | -0.682445 |
| CASP8 | NM_001228 | 0.0669542 | 0.0024 | -3.1198593 | -1.667793 | -0.68422372 |
| LINC01314 | AL109671 | 0.0111961 | 7.54E-05 | -4.2230654 | 1.39667 | -0.68496579 |
| LRRK2 | AK026776 | 0.2241679 | 0.0288 | -2.2209773 | -3.791547 | -0.68511607 |
| HES2 | NM_019089 | 0.053037 | 0.00134 | -3.3335971 | -1.089113 | -0.68513294 |
| C15orf39 | NM_015492 | 0.0833651 | 0.00375 | -2.9774792 | -2.041647 | -0.68547225 |
| SLC8B1 | AK025886 | 0.0581501 | 0.0017 | -3.2718626 | -1.261074 | -0.68859851 |
| KIAA1109 | AB037792 | 0.0036705 | 7.28E-06 | -4.8122868 | 3.555633 | -0.68874897 |
| LRRC46 | AK027206 | 0.1291422 | 0.00889 | -2.7145117 | -2.614011 | -0.68881301 |
| BAZ2B | NM_013450 | 0.0390066 | 0.000784 | -3.485357 | -0.637445 | -0.68984005 |
| RICTOR | AK024327 | 0.0237524 | 0.000305 | -3.7531842 | 0.176746 | -0.6911236 |
| ZBP1 | AJ300575 | 0.065408 | 0.00223 | -3.1542476 | -1.565525 | -0.69129921 |
| MX2 | M30818 | 0.1465527 | 0.0119 | -2.5664791 | -3.053343 | -0.69432875 |
| AKAP13 | NM_006738 | 0.0214045 | 0.000256 | -3.800476 | 0.33169 | -0.69478927 |
| PPP1R16B | AK026900 | 0.2016953 | 0.0229 | -2.3183809 | -3.536889 | -0.69495026 |
| IFI44 | NM_006417 | 0.2488785 | 0.0358 | -2.1317771 | -3.939179 | -0.69502091 |
| IDS | NM_000202 | 0.0388849 | 0.000769 | -3.4785495 | -0.650022 | -0.69550374 |
| FUS | U36561 | 0.1185939 | 0.00769 | -2.7296441 | -2.632339 | -0.69793362 |
| UBE2D1 | NM_003338 | 0.1791112 | 0.0177 | -2.4133123 | -3.377072 | -0.69987353 |
| RPS6KA5 | NM_004755 | 0.0111961 | 7.42E-05 | -4.1736781 | 1.466892 | -0.70030574 |
| H3F3A | NM_002107 | 0.0580471 | 0.00169 | -3.2348563 | -1.347946 | -0.7008081 |
| CACNA2D4 | AL137658 | 0.1297693 | 0.00904 | -2.6634713 | -2.82906 | -0.70156873 |
| CACNB4 | NM_000726 | 0.0738979 | 0.00298 | -3.0962744 | -1.718909 | -0.70205134 |
| NCF2 | NM_000433 | 0.2097765 | 0.0247 | -2.2822346 | -3.668528 | -0.70263752 |
| ZNF641 | AF086127 | 0.1149191 | 0.00716 | -2.7560631 | -2.587878 | -0.70868952 |
| SMAP2 | AL137764 | 0.2128625 | 0.0255 | -2.268712 | -3.708157 | -0.71337516 |
| LOC101928474 | AK000958 | 0.00518 | 1.42E-05 | -4.6549457 | 2.953449 | -0.71339801 |
| SLA | NM_006748 | 0.2015295 | 0.0229 | -2.3146322 | -3.586195 | -0.71371235 |
| BTNL8 | AK025111 | 0.0924658 | 0.00464 | -2.9117005 | -2.220201 | -0.71665973 |
| KRT23 | NM_015515 | 0.0939476 | 0.0048 | -2.9032957 | -2.213097 | -0.71693575 |
| KIAA1109 | AL137254 | 0.0175438 | 0.00018 | -3.9113676 | 0.659356 | -0.71697087 |
| HSPC102 | AF161365 | 0.0844345 | 0.00383 | -2.9687154 | -2.037649 | -0.71742329 |
| FAM63A | NM_018379 | 0.0143077 | 0.000125 | -4.0282524 | 1.001856 | -0.71851926 |
| PRSS22 | AF321182 | 0.0057662 | 1.76E-05 | -4.6361983 | 2.732015 | -0.71940433 |
| SSH2 | AF086010 | 0.0363239 | 0.000682 | -3.5365619 | -0.493316 | -0.72130616 |
| FPR2 | NM_001462 | 0.1646272 | 0.0148 | -2.483763 | -3.210323 | -0.72435611 |
| PROK2 | AF182069 | 0.1226812 | 0.00812 | -2.7034128 | -2.714109 | -0.72629404 |
| PPP1R15A | NM_014330 | 0.0894193 | 0.00438 | -2.9211098 | -2.19205 | -0.72664371 |
| LOC105373860 | AK026761 | 0.1083291 | 0.00635 | -2.9034324 | -2.284791 | -0.7275618 |
| SERPINA1 | NM_000295 | 0.1176582 | 0.00751 | -2.7312801 | -2.656523 | -0.72945724 |
| IL16 | AL109669 | 0.0345677 | 0.000624 | -3.5735229 | -0.40633 | -0.73049502 |
| FMNL1 | AJ008132 | 0.0539101 | 0.00138 | -3.3056403 | -1.152455 | -0.73194351 |
| DOCK4 | NM_014705 | 0.0273716 | 0.000389 | -3.7390072 | 0.016785 | -0.73211054 |
| PLEKHO1 | NM_016274 | 0.0051907 | 1.45E-05 | -4.593253 | 2.939993 | -0.73233145 |
| SETX | AK024331 | 0.0074031 | 3.53E-05 | -4.3541296 | 2.12819 | -0.73346088 |
| UBR2 | AB002347 | 0.054419 | 0.00148 | -3.3011408 | -1.157023 | -0.73464843 |
| NLRP3 | NM_004895 | 0.0109317 | 6.85E-05 | -4.1875953 | 1.535078 | -0.73828804 |
| SULF2 | AB033073 | 0.0042569 | 9.13E-06 | -4.7148622 | 3.357603 | -0.74774258 |
| FAM65B | NM_014722 | 0.1729136 | 0.0164 | -2.4415683 | -3.33783 | -0.74940801 |
| OSGIN2 | NM_004337 | 0.0163947 | 0.000163 | -3.9645862 | 0.766871 | -0.7498842 |
| PTPRA | NM_002836 | 0.0257458 | 0.000358 | -3.7089986 | 0.041245 | -0.75152595 |
| BTG2 | NM_006763 | 0.0719307 | 0.00283 | -3.0809364 | -1.758144 | -0.75660273 |
| CRTAM | NM_019604 | 0.1402432 | 0.0109 | -2.6340604 | -2.762418 | -0.75740719 |
| CD14 | NM_000591 | 0.1482563 | 0.0123 | -2.5525835 | -3.078154 | -0.76320108 |
| CDH12 | NM_004061 | 0.0665112 | 0.00236 | -3.1576645 | -1.559769 | -0.76337074 |
| TREML2 | AK023755 | 0.113181 | 0.00691 | -2.7648885 | -2.565917 | -0.76674632 |
| GPR25 | NM_005298 | 0.0694154 | 0.00258 | -3.1382735 | -1.60932 | -0.76839181 |
| NCOR2 | S83390 | 0.2018321 | 0.0229 | -2.3139224 | -3.574824 | -0.77004268 |
| GIMAP4 | NM_018326 | 0.0148125 | 0.000134 | -3.9860881 | 0.922912 | -0.77098434 |
| MCL1 | AF118124 | 0.0393494 | 0.000801 | -3.4600653 | -0.69969 | -0.77243865 |
| KALRN | NM_007064 | 0.1942796 | 0.021 | -2.3462334 | -3.543033 | -0.77366774 |
| PELI1 | AF302505 | 0.0503178 | 0.00122 | -3.3343885 | -1.061714 | -0.77424093 |
| EFHC2 | AK026496 | 0.1069101 | 0.00619 | -2.8535538 | -2.318098 | -0.77435018 |
| CYP1A1 | NM_000499 | 0.0947211 | 0.00491 | -2.8934403 | -2.235998 | -0.77456957 |
| FMNL1 | AJ008131 | 0.0323271 | 0.000539 | -3.6160319 | -0.282629 | -0.77782119 |
| FOXI1 | NM_012188 | 0.0015528 | 1.50E-06 | -5.2049842 | 4.980359 | -0.78393105 |
| SLC22A1 | NM_003057 | 0.0456285 | 0.00103 | -3.4473432 | -0.818768 | -0.785908 |
| DCHS1 | AK021852 | 0.0357683 | 0.000655 | -3.6059688 | -0.447351 | -0.78876013 |
| ECE1 | NM_001397 | 0.0110071 | 7.02E-05 | -4.1566025 | 1.497813 | -0.79302795 |
| ALOX5AP | NM_001629 | 0.2464837 | 0.035 | -2.1383801 | -3.957698 | -0.79335853 |
| ARGLU1 | NM_018011 | 0.2283515 | 0.0299 | -2.2077006 | -3.801544 | -0.79684543 |
| CHST15 | NM_014863 | 0.0323271 | 0.000548 | -3.5846227 | -0.338337 | -0.80115644 |
| FMNL1 | NM_005892 | 0.0620976 | 0.002 | -3.1866132 | -1.473622 | -0.80267672 |
| CYTH4 | NM_013385 | 0.1602764 | 0.014 | -2.5013934 | -3.204908 | -0.8056241 |
| HSPA6 | NM_002155 | 0.1236078 | 0.00822 | -2.6988064 | -2.73489 | -0.80859372 |
| GARNL3 | AK025650 | 0.0683784 | 0.00253 | -3.1706344 | -1.579197 | -0.81429088 |
| WDR83OS | NM_016145 | 0.0244857 | 0.000323 | -3.832997 | 0.162521 | -0.8206766 |
| CSRNP1 | AL117565 | 0.0245148 | 0.000333 | -3.7498847 | 0.130385 | -0.82084086 |
| TNFRSF1B | NM_001066 | 0.054321 | 0.00142 | -3.2896815 | -1.186475 | -0.82181291 |
| PREX1 | AB037836 | 0.1440794 | 0.0115 | -2.5780737 | -3.028456 | -0.82191084 |
| CXCL8 | M17017 | 0.0762261 | 0.00317 | -3.0301262 | -1.908452 | -0.82725448 |
| NFATC2 | AK025758 | 0.0193377 | 0.000221 | -3.861537 | 0.486027 | -0.83064044 |
| HES2 | AK023754 | 0.0586227 | 0.00179 | -3.2768497 | -1.284135 | -0.83250255 |
| FPR1 | NM_002029 | 0.2244411 | 0.0289 | -2.2179496 | -3.812126 | -0.83262834 |
| HCP5 | NM_006674 | 0.0583001 | 0.00172 | -3.2620717 | -1.307711 | -0.83280337 |
| PTPRC | NM_002838 | 0.2259034 | 0.0292 | -2.2126528 | -3.822853 | -0.83734694 |
| H3F3AP4///H3F3A | D17130 | 0.0724323 | 0.00286 | -3.0594233 | -1.827243 | -0.84015523 |
| PISD | AL050371 | 0.0321337 | 0.00052 | -3.5957362 | -0.290975 | -0.84029137 |
| ACTR2 | AK025051 | 0.0036705 | 7.22E-06 | -4.8924317 | 3.531858 | -0.85249544 |
| PPP1R10 | NM_002714 | 0.0115729 | 8.13E-05 | -4.1268124 | 1.370299 | -0.85690377 |
| TMEM123 | AL110202 | 0.1297693 | 0.00905 | -2.6641981 | -2.817528 | -0.86007269 |
| GPR65 | NM_003608 | 0.0379524 | 0.000734 | -3.4965266 | -0.598953 | -0.8625935 |
| NOTCH2 | U77493 | 0.0143077 | 0.00012 | -4.0670531 | 1.038456 | -0.86993619 |
| SLC25A37 | S94541 | 0.0291837 | 0.000436 | -3.6461728 | -0.140764 | -0.87234499 |
| G0S2 | NM_015714 | 0.0886015 | 0.00429 | -2.9262131 | -2.170003 | -0.87601115 |
| XPO6 | AB002368 | 0.0321337 | 0.000524 | -3.592075 | -0.309689 | -0.87961737 |
| MBOAT7 | S82470 | 0.0332742 | 0.000581 | -3.5578186 | -0.41057 | -0.88086065 |
| SMCHD1 | AB014550 | 0.0056045 | 1.65E-05 | -4.5446673 | 2.813879 | -0.88164014 |
| DIO3OS | AF305836 | 0.1475936 | 0.0121 | -2.5600064 | -3.0589 | -0.88962616 |
| RUNX2 | AL353944 | 0.0021755 | 3.03E-06 | -5.0536911 | 4.344618 | -0.89920593 |
| NAMPT | NM_005746 | 0.2173788 | 0.0267 | -2.2506034 | -3.708039 | -0.89922182 |
| FES | NM_002005 | 0.0664961 | 0.00235 | -3.1311373 | -1.619205 | -0.89933507 |
| CLIC1 | NM_001288 | 0.0086874 | 4.47E-05 | -4.2753443 | 1.906095 | -0.89944952 |
| ACSL1 | NM_001995 | 0.1359438 | 0.0101 | -2.6237708 | -2.912905 | -0.90077725 |
| IFIT2 | AF026944 | 0.0939476 | 0.00477 | -2.8992201 | -2.227316 | -0.90356241 |
| FMNL1 | AJ008123 | 0.0235571 | 0.000298 | -3.8263237 | 0.249569 | -0.90586489 |
| ICAM3 | NM_002162 | 0.0531309 | 0.00135 | -3.3029749 | -1.145734 | -0.91047376 |
| COQ4 | NM_016035 | 0.1177625 | 0.00752 | -2.7300585 | -2.668682 | -0.91822938 |
| RNF130 | NM_018434 | 0.0150557 | 0.00014 | -3.9667482 | 0.875323 | -0.92429036 |
| LAPTM5 | NM_006762 | 0.0863546 | 0.004 | -2.9476487 | -2.121757 | -0.92534731 |
| MAP3K14 | AJ008151 | 0.012562 | 9.29E-05 | -4.0883096 | 1.24889 | -0.94217758 |
| NADK | AK023114 | 0.0063444 | 2.67E-05 | -4.4221113 | 2.37762 | -0.94770004 |
| KLF2 | NM_016270 | 0.0521155 | 0.00129 | -3.3211586 | -1.107803 | -0.95279626 |
| CKAP4 | NM_006825 | 0.0244857 | 0.000327 | -3.7272257 | 0.109814 | -0.96776738 |
| CTSS | NM_004079 | 0.0607189 | 0.00189 | -3.1929851 | -1.46364 | -0.98228984 |
| CXCR4 | NM_003467 | 0.0100636 | 5.88E-05 | -4.2127719 | 1.662922 | -0.98575177 |
| IFIT1 | NM_001548 | 0.1217675 | 0.00801 | -2.7253642 | -2.626683 | -0.9972428 |
| STK17B | NM_004226 | 0.0281913 | 0.000417 | -3.6595883 | -0.099904 | -0.99913871 |
| CELF2 | NM_006561 | 0.0143077 | 0.000118 | -4.0203613 | 1.034168 | -1.0187063 |
| DUSP1 | AJ227912 | 0.033557 | 0.000592 | -3.5558215 | -0.407481 | -1.02136639 |
| DPP6 | AK001153 | 0.0620872 | 0.00198 | -3.2718636 | -1.642996 | -1.02378464 |
| FAM8A1 | NM_016255 | 0.0150118 | 0.000137 | -4.0669704 | 0.918806 | -1.03075832 |
| PTGS2 | NM_000963 | 0.0063444 | 2.05E-05 | -4.5151312 | 2.628566 | -1.03629162 |
| ZNF25 | X52350 | 0.0063444 | 2.03E-05 | -4.4811024 | 2.621811 | -1.05181062 |
| PTPN6 | NM_002831 | 0.0424582 | 0.000901 | -3.4290535 | -0.792551 | -1.06073474 |
| RAF1 | NM_002880 | 0.0010003 | 6.25E-07 | -5.3641271 | 5.800067 | -1.07964447 |
| DUSP1 | NM_004417 | 0.0418652 | 0.000877 | -3.4371526 | -0.762852 | -1.08662467 |
| QPCT | NM_012413 | 0.0143077 | 0.000123 | -4.0065237 | 0.991186 | -1.08797039 |
| BNIP3L | NM_004331 | 3.57E-05 | 1.91E-09 | -6.6503297 | 11.124616 | -1.0973528 |
| FBXL5 | AF157323 | 0.054206 | 0.0014 | -3.2939999 | -1.181108 | -1.10125667 |
| ARAP1 | AB018325 | 0.0036623 | 6.67E-06 | -4.7661046 | 3.638356 | -1.10351094 |
| ZFP36 | NM_003407 | 0.054419 | 0.00146 | -3.2754667 | -1.232879 | -1.11339405 |
| SELL | NM_000655 | 0.0861666 | 0.00398 | -2.9520972 | -2.094756 | -1.12680923 |
| KIAA1551 | NM_018169 | 0.0101373 | 5.97E-05 | -4.2083436 | 1.649577 | -1.20009996 |
| RGS2 | NM_002923 | 0.1040257 | 0.00589 | -2.8162714 | -2.447503 | -1.26564415 |
| MME | NM_007289 | 0.0067451 | 3.11E-05 | -4.3850305 | 2.241053 | -1.28043315 |
| MXD1 | NM_002357 | 0.0086874 | 4.32E-05 | -4.2952288 | 1.942686 | -1.28298957 |
| FUS | NM_004960 | 0.0115729 | 8.18E-05 | -4.1188538 | 1.360865 | -1.31715079 |
| TREM1 | NM_018643 | 0.0009647 | 3.59E-07 | -5.4729336 | 6.306923 | -1.33885304 |
| LITAF | NM_004862 | 0.012946 | 9.73E-05 | -4.0677467 | 1.201917 | -1.41882443 |
| CHI3L1 | NM_001276 | 0.000986 | 4.24E-07 | -5.4980186 | 6.169142 | -1.42596783 |
| EVI2B | NM_006495 | 0.003133 | 5.20E-06 | -4.830873 | 3.866006 | -1.68950204 |
